# Comparative developmental genomics of sex-biased gene expression in early embryogenesis across mammals

**DOI:** 10.1186/s13293-023-00520-z

**Published:** 2023-05-19

**Authors:** Victorya Richardson, Nora Engel, Rob J. Kulathinal

**Affiliations:** 1 Department of Biology, Temple University, 1900 N. 12th Street, Philadelphia, PA 19122 USA; 2 Department of Cancer Biology, Lewis Katz School of Medicine, Fels Cancer Institute for Personalized Medicine, Temple University, 3400 N. Broad Street, Philadelphia, PA 19140 USA; 3 Institute for Genomics and Evolutionary Medicine, Temple University, Philadelphia, PA 19122 USA

**Keywords:** Sex-biased gene expression, Evolutionary development, Genomics, Mammalian embryogenesis

## Abstract

**Background:**

Mammalian gonadal sex is determined by the presence or absence of a Y chromosome and the subsequent production of sex hormones contributes to secondary sexual differentiation. However, sex chromosome-linked genes encoding dosage-sensitive transcription and epigenetic factors are expressed well before gonad formation and have the potential to establish sex-biased expression that persists beyond the appearance of gonadal hormones. Here, we apply a comparative bioinformatics analysis on a pair of published single-cell datasets from mouse and human during very early embryogenesis—from two-cell to pre-implantation stages—to characterize sex-specific signals and to assess the degree of conservation among early acting sex-specific genes and pathways.

**Results:**

Clustering and regression analyses of gene expression across samples reveal that sex initially plays a significant role in overall gene expression patterns at the earliest stages of embryogenesis which potentially may be the byproduct of signals from male and female gametes during fertilization. Although these transcriptional sex effects rapidly diminish, sex-biased genes appear to form sex-specific protein–protein interaction networks across pre-implantation stages in both mammals providing evidence that sex-biased expression of epigenetic enzymes may establish sex-specific patterns that persist beyond pre-implantation. Non-negative matrix factorization (NMF) on male and female transcriptomes generated clusters of genes with similar expression patterns across sex and developmental stages, including post-fertilization, epigenetic, and pre-implantation ontologies conserved between mouse and human. While the fraction of sex-differentially expressed genes (sexDEGs) in early embryonic stages is similar and functional ontologies are conserved, the genes involved are generally different in mouse and human.

**Conclusions:**

This comparative study uncovers much earlier than expected sex-specific signals in mouse and human embryos that pre-date hormonal signaling from the gonads. These early signals are diverged with respect to orthologs yet conserved in terms of function with important implications in the use of genetic models for sex-specific disease.

**Supplementary Information:**

The online version contains supplementary material available at 10.1186/s13293-023-00520-z.

## Plain language summary

Sex differences are traditionally assumed to arise after the reproductive systems are set up in male and female embryos, and especially after these organs begin producing sex hormones. However, very early in embryo development, the sex chromosomes distinguish males (XY) and females (XX) and genes on these chromosomes are expressed differentially. In this study, we have analyzed gene expression data from mouse and human early embryos to determine whether there is sex-biased expression before implantation. Our results confirm that there is abundant sex-biased expression from the earliest stages of development, soon after fertilization. The comparison between human and mouse embryos shows comparable function of the sex-biased genes, although the specific genes involved differ between the two species.

**Supplementary Information:**

The online version contains supplementary material available at 10.1186/s13293-023-00520-z.

## Highlights

- Sex-stratification and computational analyses of previously published single-cell RNA-seq data from human and mouse pre-implantation embryos reveal substantial sex-biased gene expression.
- Transcriptional sex differences diminish as pre-implantation proceeds, but sex-biased genes form sex-specific protein–protein interaction networks which include epigenetic enzymes.
- These patterns have the potential to establish sex-specific epigenetic features that persist beyond implantation.

**Supplementary Information:**

The online version contains supplementary material available at 10.1186/s13293-023-00520-z.

## Background

The dichotomy between the sexes is distinct, pervasive, and often extreme across the eukaryotic tree of life [1–3]. Males and females harbor both conspicuous and cryptic differences in reproduction, physiology, morphology, and behavior despite sharing much of their genomic content. Sexual dimorphism is thought to have originated over a billion years ago, representing a defining hallmark of eukaryote diversity, particularly among animals. Indeed, the ubiquity of sexual dimorphism represents a conserved biological trait shared across the diversity of life. Evolutionary mechanisms such as sexual selection [1] and sexual conflict [4] have been hypothesized to maintain and promote the presence of sexually dimorphic traits, most of which are taxon-specific.

While sex-specific differences are readily observed in adults, these differences are initiated by early acting sex determining and compensatory mechanisms that occur early in embryogenesis. In mammals, the process of establishing sex differences has traditionally been divided into two phases: (i) an initial genetic stage of gonad formation, also known as “sex determination”, with SRY as the master regulator of male gonadogenesis and (ii) a later secondary stage of sexual differentiation regulated by gonadal hormones [5].

However, this view ignores the consequences of the inherent differences in sex chromosome composition (i.e., XX in females and XY in males) which affect the embryo beginning soon after fertilization as well as the effects of sex chromosome-linked genes on gonadal and non-gonadal tissues throughout the organism’s lifespan [6]. Sex-linked genes, some of which are transcription and epigenetic factors with downstream autosomal targets, are expressed soon after fertilization [7–11]. In addition, female embryos undergo X chromosome inactivation during implantation, a massive epigenetic event that has been hypothesized to alter the levels of epigenetic factors in female cells relative to male cells [12–17]. These early differences in regulatory factors have the potential to modify the transcriptional and epigenetic landscape in a sex-specific manner that can persist across an organism’s lifespan.

Recent developmental studies have reported transcriptional programs using single-cell RNA-sequencing experiments in early mammalian embryogenesis [18–20]. Yet sex-biased gene expression has rarely been surveyed during or preceding pre-implantation which encompasses the stages from zygote to blastocyst before the embryo interacts and connects with the uterus [21, 22]. The few studies that exist show that sex-biased gene expression from both sex chromosomes and autosomes is detectable in pre-implantation embryos and in embryonic stem cells in rodents, bovine, and primates, including humans [7, 8, 11]. These differences can propagate through regulatory networks, resulting in distinct male and female cell states well before gonadal development.

In this paper, we identify and compare male and female gene expression levels in pre-implantation embryogenesis across two mammalian species using published transcriptomic time series. Our goals are twofold: (i) to identify early sex-specific patterns of gene expression in the earliest stages of embryogenesis (i.e., in dividing cells soon after fertilization) in both mouse and human and (ii) to compare how conserved early acting sex-specific networks are across mammalian lineages. We identify very early acting sex-specific genes and networks in both mouse and human that, surprisingly, are not shared among orthologs. In contrast, however, functional ontologies appear to be similar between mouse and human across developmental stages. Our study provides support for a dynamic stage- and sex-specific landscape of gene expression that underlies conserved phenotypes of development and sexual identity during the earliest stages of an individual’s lifecycle.

## Materials and methods

### Sequence retrieval, pre-processing, and normalization

Single-cell transcriptomic data were downloaded from two independent studies surveying the earliest stages of embryonic development in mouse and human: 106 mouse samples representing 2-cell to early blastocyst development [23] (https://www.ncbi.nlm.nih.gov/geo/query/acc.cgi?acc=GSE80810) and 1529 human samples stemming from 8-cell to late blastocyst [18] (https://www.ebi.ac.uk/arrayexpress/experiments/E-MTAB-3929/). A summary of the temporal sample sources for the pair of datasets is found in Additional file 2: Table S1. Briefly, the mouse embryos analyzed were originally derived from natural matings between C57BL/6J (B6) females crossed with CAST/EiJ (castaneous) males or its reciprocal cross. The direction of the cross is not a source of variability with regard to the sex biases in gene expression, at least at these early stages. Human embryos were obtained by in vitro fertilization and frozen E2 human embryos were cultured to obtain the subsequent stages. Mouse genes with RIKEN annotations (*n* = 1860) were removed from the dataset. Lowly expressed genes and samples with poor coverage were filtered using R package “Seurat” (min.cells = 3, min.features = 350) [24–27]. Inter-cell normalization was performed using a deconvolution approach for single-cell transcriptomic data with many zero values [28] (Additional file 1: Fig. S1).

### Sexing single-cell data

Single cells were sexed in mouse samples by determining the expression ratios of two early expressed genes, *Xist* and *Eif2s3y*, located on, respectively, the X and Y chromosomes. *Xist* was primarily used as a technical control to filter cells with sufficient sequencing signal and to identify contaminated cells. *Xist* to *Eif2s3y* ratios were estimated individually on a cell-by-cell basis with samples harboring ratios below one labeled as male and samples that did not express Y-linked *EiF2s3Y* labeled as female. Care was taken not to map EiF2s3Y’s X-linked mouse paralog, *Eif2s3x*. Samples with *Xist/Eif2s3y* ratios greater than one were considered ambiguous and removed from the analysis. We note that the total numbers of male and female samples are relatively low (Additional file 2: Table S1). Human samples were previously sexed in the original dataset via the presence of Y-linked expression [18].

### Sex-differential expression analyses

Sex-differentially expressed genes between male and female cells (sexDEGs) were independently identified at each embryonic stage using “DESeq2” in R, with default parameters. To contrast expression levels between males and females at each stage, we used the design formula “ ~ sex_stage”, where “sex_stage” is a column of combined sex and stage data. Log_2_ fold change results were transformed using lfcShrink() in R with default parameters. Genes with |log_2_ fold change|≥ 0.58 and adjusted *p*-values < 0.05 were marked as differentially expressed. Results of the analyses are reported in Additional file 2: Tables S2 and S3 for, respectively, mouse and human.

### Non-negative matrix factorization (NMF)

To identify longitudinal sex-specific subnetworks of co-expression, mouse and human datasets were divided into male- and female-specific count tables (mouse samples: 71 female, 35 male; human samples: 821 female, 708 male). The resulting subsets were filtered for low expression (min.cells = 3, min.features = 350) and log-normalized using R package “Seurat” [25]. To identify the optimal matrix rank (or number of resulting clusters), NMF was run 10 times for each user-inputted rank across ranks 5 to 30, using R package “NMF” [29]. For each sex-specific count table, the rank used for identification of clusters (or metagenes) was chosen as the rank corresponding to the highest cophenetic correlation coefficient (Additional file 1: Figs. S2, S3; Additional file 2: Table S4). The coefficient, or *W*, matrix for the chosen rank was extracted for subsequent gene set enrichment analysis (Additional file 2: Tables S5–S8).

### Functional enrichment analysis

Gene collections (ontology gene sets, hallmark gene sets, and regulatory target gene sets) were accessed from the Molecular Signatures Database (MSigDB) [30, 31]. Pre-ranked gene set enrichment analysis was performed on each metagene using R package “fgsea” (minSize = 15, maxSize = 500, scoreType = ‘pos’), with genes ranked according to their entry in the coefficient matrix corresponding to the metagene column [32]. Significant gene pathways (adjusted *p*-value < 0.05) for each metagene were kept for comparison of functional enrichment between emergent sex-specific subnetworks from mouse and human (Additional file 2: Tables S9–S12).

## Results

### Transcriptomics of early development in mouse and human

**Fig. 1:**
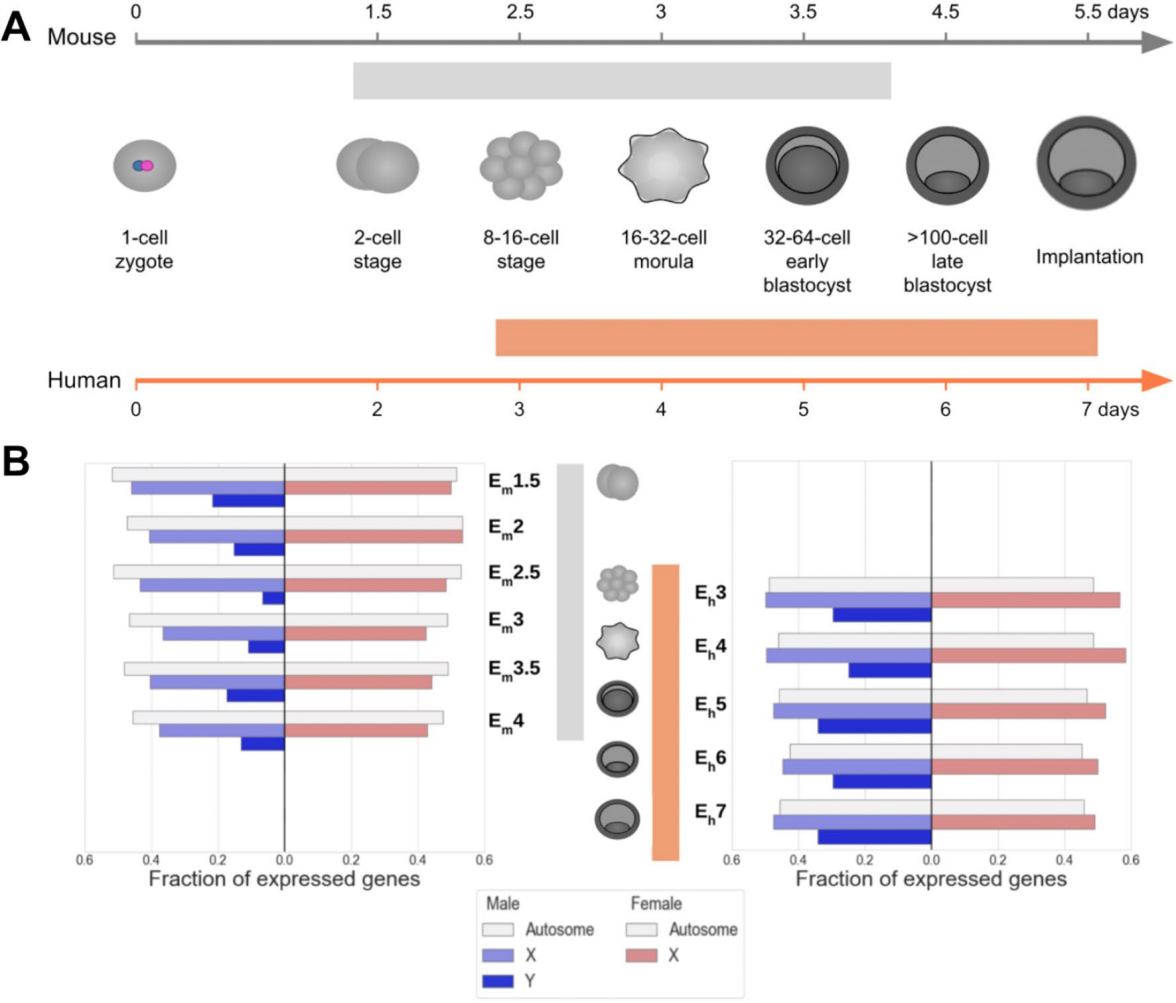
Developmental timeline and gene expression levels between mouse and human samples used in this study. **A** The relative timing of embryogenic stages from zygote to post-implantation differs across the two mammals, mouse and human. Samples taken from a pair of published datasets derived from mouse (gray) and human (orange) represent an overlapping series of early developmental stages [18, 23]. **B** The total fraction of annotated genes that are expressed in males (blue) and females (red) on the autosomes, X-, and Y-chromosomes, are displayed separately for mouse (left) and human (right) datasets

Nearly half of all autosomal genes are expressed in early embryogenesis, i.e., during the first week of development (Fig. 1A), in both sexes of mouse and human (Fig. 1B). While the number of expressed autosomal genes remains relatively constant from the two-cell to pre-implantation stages across sex and species, a larger variance in the fraction of sex-chromosomal genes expressed across mouse and human developmental stages is observed. The ratio of X chromosomal to autosomal gene expression is different between mammals with relatively lower fractions of X-linked genes being expressed in mouse. In addition, a larger proportion of Y-chromosome genes are expressed in male human compared to male mouse. However, the Y-linked expressed fraction is generally much lower than the expressed fraction of X-linked and autosomal genes in both mammals (Fig. 1B).

Of the 1000 most highly expressed genes in mouse pre-implantation stages, the number of genes that encode transcription factors (TF) and epigenetic enzymes (EE) peaked at the two-cell stage with steadily diminishing numbers as development proceeded (E_m_1.5: 51 TFs, 27 EEs; E_m_3: 24 TFs, 12 EEs). A similar trend was observed for human embryos in transcription factors (E_m_3: 49 TFs; E_m_7: 23 TFs). However, the number of expressed genes encoding EEs was almost constant between stages. Surprisingly, the orthologs of only 17 TFs and 6 EEs expressed in the mouse were detected in human embryos. Among the common regulatory factors between mouse and human, most were expressed at analogous stages of early embryonic development, such as *Atf4*, *Elf3*, *Sall4*, *Tfap2c*, *Hdac1*, *Kdm5b* and *Tet1*.

Several of the so-called “pluripotency factors” [33, 34], which include transcription factors, epigenetic enzymes, and signaling molecules, were detected in the two datasets with several uniquely expressed in one of the two species. For example, *Dnmt3b*, *Dnmt3l*, *Sall4*, and *Tead4* were present in both mouse and human while *Nanog*, *Esrrb*, *Gata4*, and *Pou5f1* (*Oct4*) were not detected in human embryos, and *Klf4, Myc and Dnmt3a* were not detected in mouse.

### Relative contribution of developmental stage versus sex-to-gene expression in early embryogenesis

**Fig. 2:**
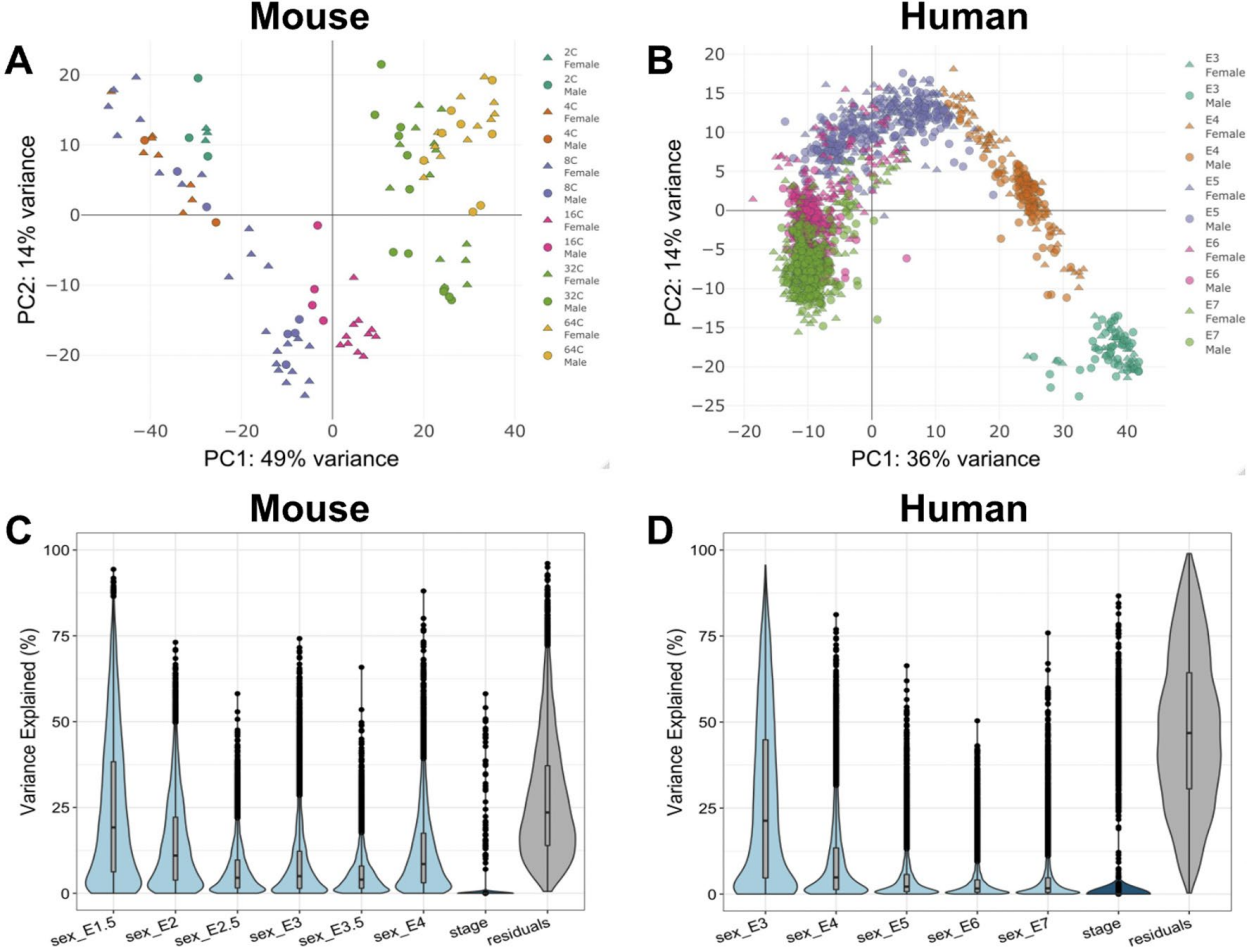
Contribution of stage and sex to total gene expression variation in mouse and human embryogenesis. Principal component analysis of **A** mouse embryonic samples (*n* = 106) and **B** human embryonic samples (*n* = 1529) reveal strong clustering across developmental stages. In both mouse and human, the majority of the total variance is explained by PC1 and PC2. A regression model was applied in **C** mouse and **D** human to estimate the distribution and density of the fraction in expression variance for each gene, as seen in the violin plots, that can be explained by embryonic stage and sex

Normalized genome-wide expression counts from each sample were found to primarily cluster according to developmental stage progression in both mouse and human (Fig. 2A, B). The first two principal components explained 63% and 50% of the total variance in gene expression in, respectively, mouse and human. While male and female samples from each developmental stage clustered in a time-dependent manner, it was difficult to visually differentiate among sexed samples via PCA. Male and female samples appear to weakly cluster together at very early stages and less during later stages of pre-implantation embryogenesis. To better understand the quantitative contribution of sex across development stages, we employed a linear regression model and found that sex explained nearly a quarter of the genetic variance in gene expression during the earliest stages of embryogenesis in both mouse and human (Fig. 2C, D) but that this contribution of sex rapidly decreased. This rapid diminution in the expression variation that is explained by sex reflects that sex’s relative role decreases rapidly and substantially across very early development. Genes with the highest and lowest principal component scores for the top 15 principal components of gene expression data in mouse and human are shown in Additional file 1: Figs. S6 and S7.

### Enrichment of sex-differentially expressed genes

Common enrichment patterns among sex-differentially expressed genes (sexDEGs) are observed in males and females in both mouse and human (Table 1). As expected, due to the different number of sex chromosomes in males and females, sexDEGs that are X- and Y-linked are enriched in females and males across both species, respectively (Table 1). In contrast, the number of sex-differentially expressed autosomal genes is statistically under-represented in both sexes in most early developmental stages in mouse and human. X-linked genes also have lower than expected number of sexDEGs in early stages (although with far lower sample sizes). The X-chromosome becomes over-enriched for sexDEGs during the blastocyst stage. In terms of functional annotations, while the majority of DEGs are protein-coding genes, these genes are under-enriched for DEGs in many developmental stages with a more noticeable effect in humans, likely due to increased sample size and statistical power (Table 1). On the other hand, a lack of power likely impedes our ability to detect enrichment in epigenetic and transcription factor sexDEGs in early embryogenesis.

**Table 1:**
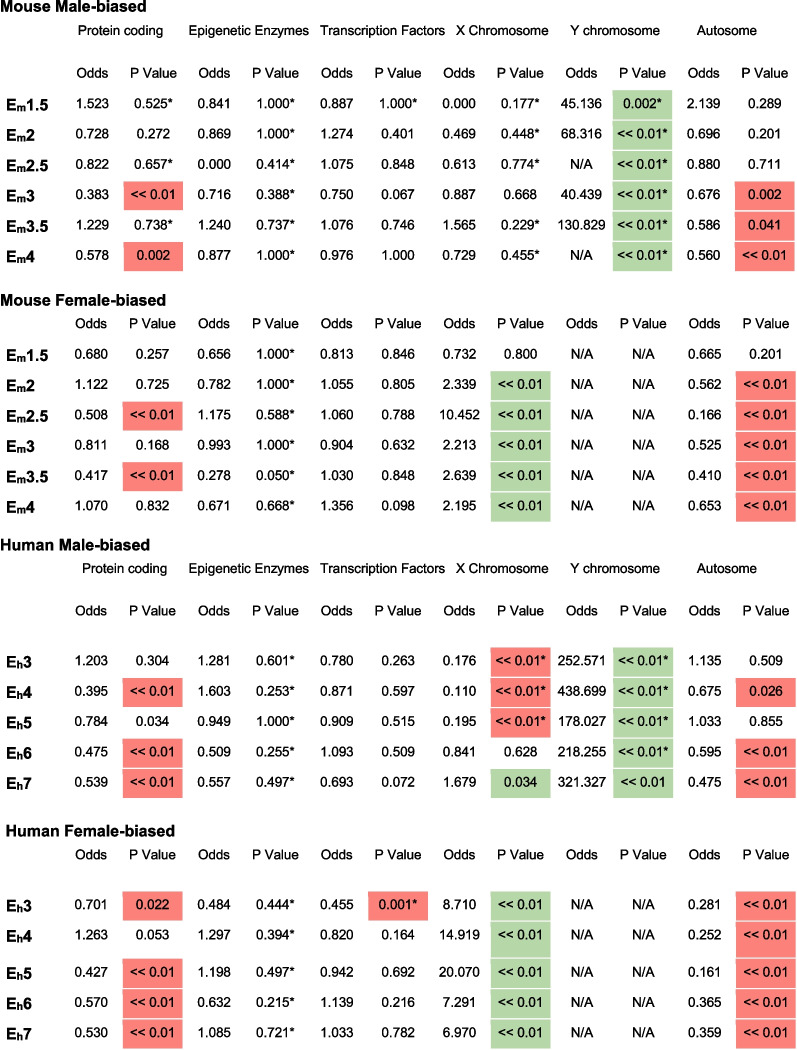
Enrichment of differentially expressed genes between males and females (sexDEGs) for function and genomic location

For each stage in (A) mouse and (B) human, we tested for enrichment (over and under) of sex-differentially expressed genes (sexDEGs) according to general functional classes (protein-coding, epigenetic factors, transcription factors) and chromosomal location. sexDEGs were separated into their male-biased and female-biased classes. *P*-values shaded in red represent an under-enrichment while *p*-values shaded in green represent an over-enrichment. *P*-values with asterisk are low-confidence due to low sample size in the given group

### Characterization of sex-specific differences in early mammalian embryogenesis

**Fig. 3:**
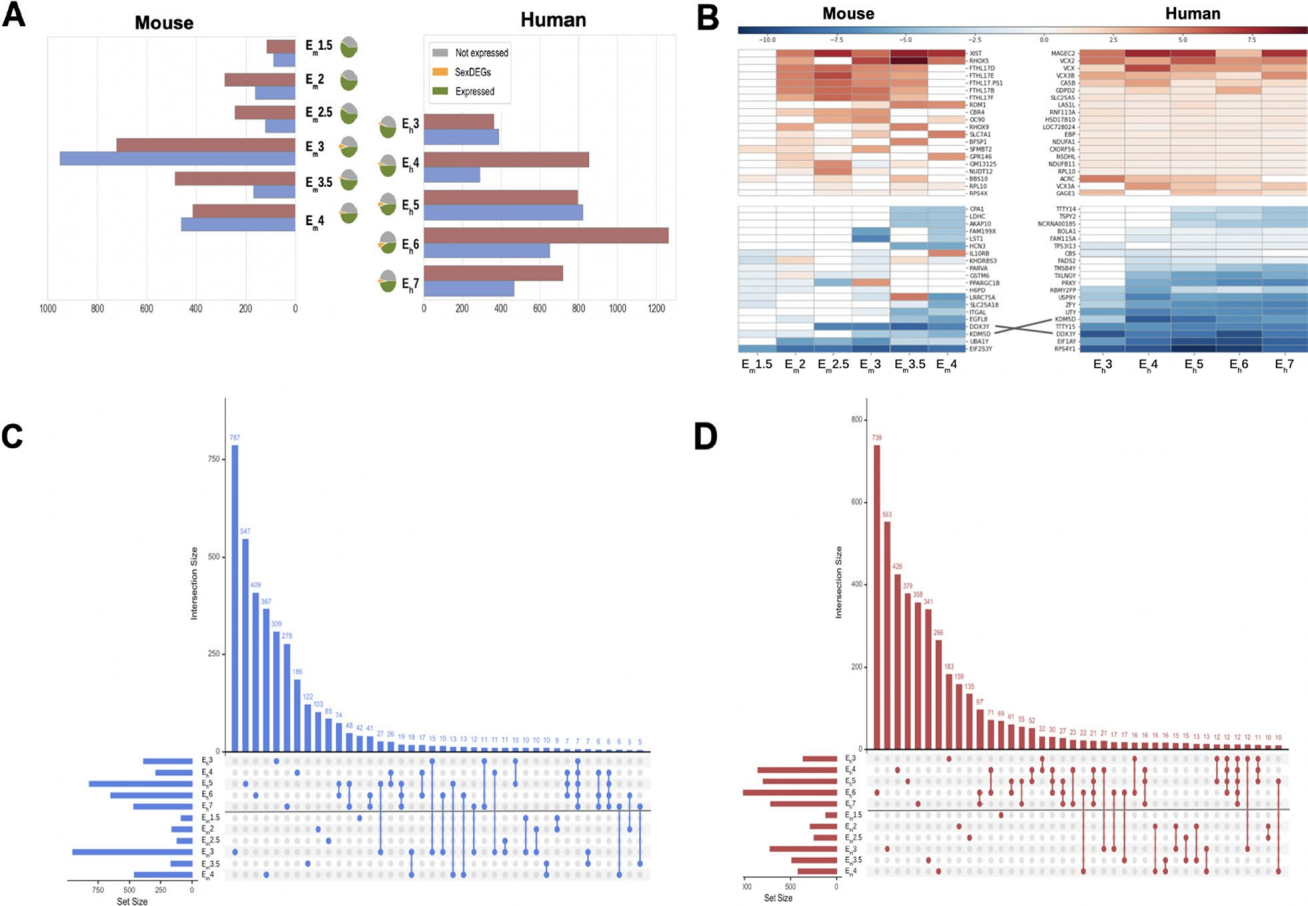
Sex-differentially expressed (sexDEGs) genes across early embryonic stages of mouse and human. **A** The number and direction (female-biased in red; male-biased in blue) of sex-differentially expressed genes (sexDEGs) are shown across sampled embryonic stages in mouse and human. The number of sex-biased genes (orange) is relatively small compared to the total number of expressed genes (green) or non-expressed genes (gray), as seen in the pie-charts. **B** Heatmap of the 20 most female-biased genes (top, red) and the 20 most male-biased genes (bottom, blue) for mouse (left) and human (right). Color intensity indicates log-fold change obtained from a Wald test for differential expression. X-, and Y-linked genes are indicated by, respectively, short red and purple lines. A pair of orthologs found among these highly sex-biased genes in mouse and human are indicated. Upset plots displaying the number of unique and shared (intersecting) sexDEGs across mouse and human developmental stages for **C** male-biased and **D** female-biased sexDEGs

During the early stages of embryogenesis in both mouse and human, more genes appear to be expressed than not expressed (Fig. 3A), however, this ratio quickly becomes approximately 1:1. The number of sex-differentially expressed genes in both mouse and human is small (Fig. 3A). In mouse, the total number of sexDEGs increases starting from the four-cell stage and peaks at the 16-cell stage (E_m_3). Although the number of male-biased genes is eight times higher than female-biased genes at the four-cell and eight-cell stages, this ratio changes at the 16-cell stage in which upregulated female-biased genes are about twice the number of upregulated male DEGs. Our functional enrichment analyses of DEGs found that transcription factors (TFs) were enriched at the four-cell, eight-cell, and 64-cell stages while genes on the sex chromosomes were enriched at the eight-cell and 64-cell stages. We also found a significant difference in the number of DEGs encoding transcription factors (TFs) over-expressed in females compared with TFs that were over-expressed in males across developmental stages. The number of female-biased TF DEGs follows the same pattern as total DEGs, while the number of male-biased TF DEGs remains constant across developmental stages. In human, a comparable magnitude of sexDEGs is seen across stages with a peak in the late blastocyst stage, where female DEGs were found to be twice as high as male DEGs (Fig. 3A).

Figure 3B presents heatmaps of the 20 genes showing the greatest differences in expression between males and females, i.e., female-biased (red, top half) and male-biased (blue, bottom half), across development stages for mouse (left heatmap) and human (right heatmap). Many of the male-biased genes in human and several male-biased genes in mouse are Y-linked and should, therefore, be considered as uniquely male-expressed since they are not present in females. Interestingly, there were only two orthologs between mouse and human that showed similar sex-biased patterns, *KDM5D* and *DDX3Y*, both Y-linked genes. *KDM5D* is a histone lysine demethylase with a repressive transcriptional role and *DDX3Y* is a DEAD-box RNA helicase. A higher rate of gene loss among Y-linked orthologs may also explain their lower than expected numbers between mouse and human. Figure 3B also highlights how sex-biased landscapes can dramatically differ between early embryonic stages and adult stages. For example, the four most female-biased genes (MAGEC2, VCX2, VCX, VCX3B) expressed in early human embryos are also known to be highly expressed in the adult testis (www.genecards.org). In fact, members of the VCX gene family are also ubiquitously expressed across multiple adult tissues, albeit at much lower levels, and are known to be involved in inborn disorders.

### Differences in sex-differentially expressed protein interactions between mouse and human

**Fig. 4:**
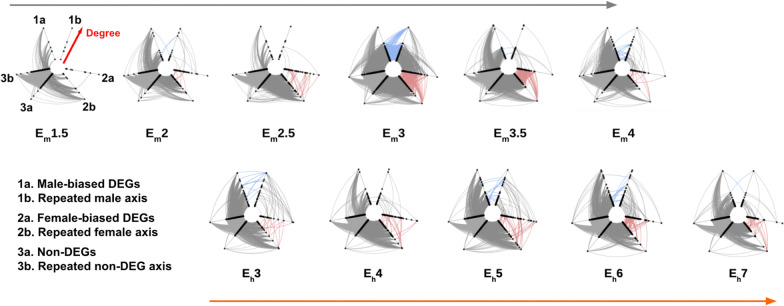
Hive plots of emerging sex-biased protein–protein interaction networks across development stages in mouse and human. Sex-differentially expressed genes between sexes (sexDEGs) are mapped onto full protein–protein interaction networks constructed from expressed genes for each embryonic stage. The three pairs of axes correspond to the differential expression classes: (i) male-biased DEGs (axes 1a and 1b), (ii) female-biased DEGs (axes 2a and 2b, and (iii) non-DEGs (axes 3a and 3b. The additional axis of each pair was added to show the density of interactions within each class (i.e., male–male (blue), female–female (red), and nonDEG–nonDEG)

In early mouse development, protein–protein interactions (PPi) are dominated by non-differentially expressed genes (Fig. 4A). In the 16-cell stage, we observe a burst of male-specific interactions and, in the 32-cell stage, we see a similar burst in female-specific interactions while the number of interactions between non-DEGs decreases accordingly. During these periods of increased PPi activity, interactions between male or female DEGs with non-DEGs also increases, but the same decrease is seen in both male and female in the blastocyst stage. A different pattern is observed in humans. Comparing the normalized fraction of interactions to total DEGs, we do not observe such a burst in male- or female-specific interactions as we do in mouse but there are distinct patterns. Non-DEGs interactions are higher in earlier stages, decrease in middle stages E5 and E6, and increase again in the latest sampled stage. We also observe an increase in female–female DEG interactions in human stage E6, but not like the sharp increase observed in mouse. It should be noted that the network sizes for human tend to be smaller due to fewer human genes mapping to the protein–protein interaction data.

### Lack of conservation signal in orthologous genes across very early mouse and human embryonic stages

**Fig. 5:**
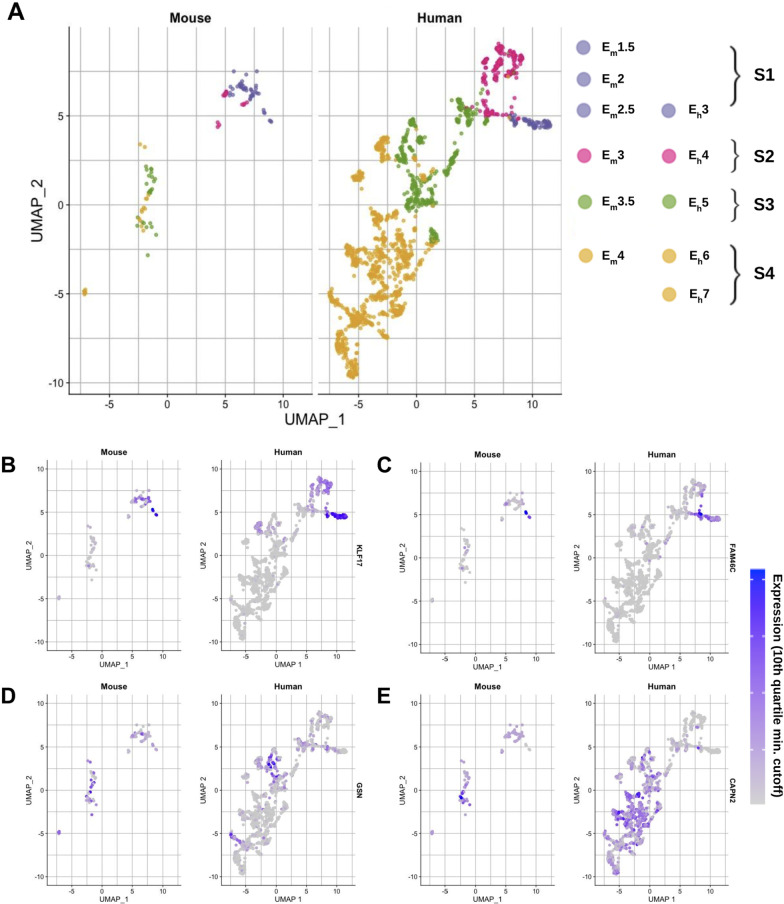
Identifying conserved signals among orthologous genes across very early mouse and human embryonic stages. **A** An integrated UMAP analysis of human and mouse samples across embryonic stages separates mouse (two left clusters) and human (large right cluster). The two datasets were integrated using Seurat to identify common cell types. Four developmental stage-adjacent groups that include both mouse and human samples are denoted as S1, S2, S3, and S4. **B**–**E** The most conserved orthologs between mouse and human with respect to expression levels across each of the four developmental groups reveals similar positioning of ortholog expression between mouse (left) and human (right) clusters. Expression values were filtered to be within the 10th quantile of the integrated expression data. The top homologously expressed genes from the four developmental stage-adjacent groups, S1, S2, S3, and S4 are, respectively, **B** KLF17, **C** FAM46C, **D** GSN, **E** CAPN2. Gene ontology function of the top ten similarly positioned genes from this integrated UMAP analysis for each of the four developmental groups is found in Additional file 2: Table S13

Clustering analysis (UMAP) of integrated gene expression data using all embryo samples of both mammals allow us to align the two datasets and compare the structure of gene expression clusters between mouse and human across timepoints. We observe similar stages in mouse and human in the same region of the graph although, again, it is difficult to directly compare due to the relatively reduced number of mouse samples (Fig. 5A). To mitigate the small sample sizes in mouse, cells were grouped based on “generalized stage of development” timepoints representing four developmental stage-adjacent groups (denoted as S1, S2, S3, and S4 and color-coded) and include both mouse and human embryos sampled at overlapping developmental stages (see key in Fig. 5A).

Categorizing the sampled cells from mouse and human into four generalized developmental stages/groups further allows us to identify genes that are transcriptionally conserved between these two mammals across embryonic development. (Again, to perform this analysis, it is important to have a sufficient number of cells represented at each timepoint from both species.) In Fig. 5B–E, the same UMAP as in Fig. 5A is superimposed with normalized gene expression levels for a representative highly conserved gene from each of the four generalized developmental stages/groups (see Additional file 2: Table S13 for an annotated list of the top conserved genes for each general timepoint S1, S2, S3, S4, ranked by *p*-value). Each gene (Fig. 5B: group S1, KLF17; Fig. 5C: group S2, FAM46C; Fig. 5D: group S3, GSN; Fig. 5E: group S4, CAPN2) represents the most transcriptionally conserved genes across species with respect to developmental timing, and their relative expression values are highlighted on a gradient in each plot. Only samples that have gene expression levels in the top 10th quartile in each species are colored. The rest are gray to highlight high gene expression across certain developmental timepoints vs. others.

### Conserved sex-biased genes and networks between mouse and human

**Fig. 6:**
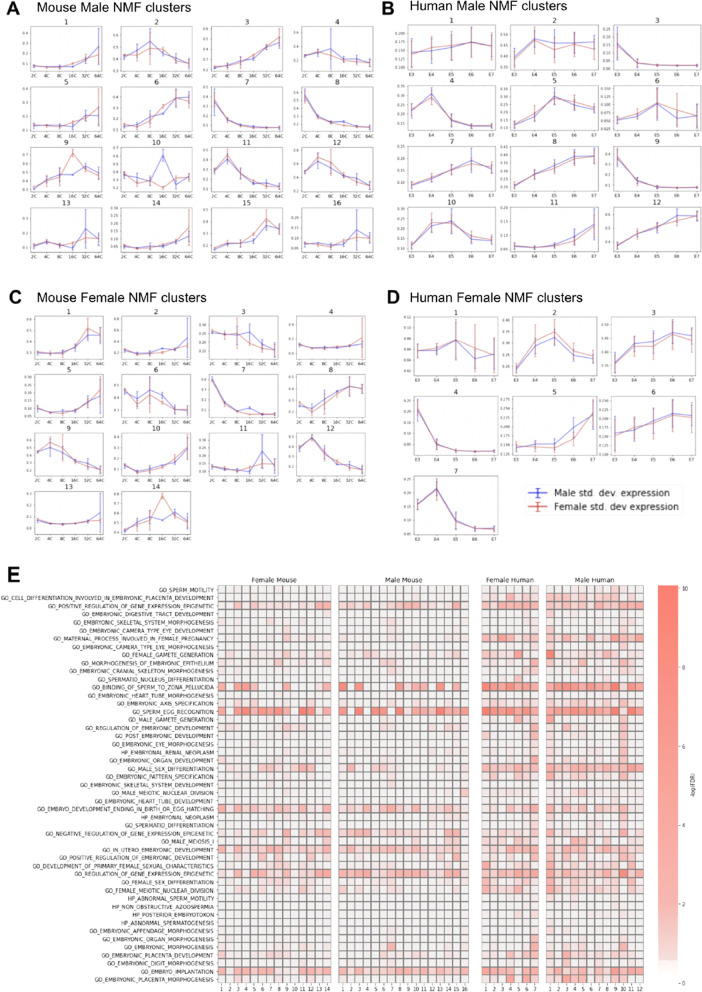
Co-expressed gene expression clusters and pathway enrichment over early developmental time-course in mouse and human. **A**–**D** Unsupervised clusters of gene expression across developmental timepoints resulting from the non-negative matrix factorization (NMF) of expression data. Each plot depicts mean expression across samples for each stage in **A** male mouse clusters, **B** male human clusters, **C** female mouse clusters, and **D** female human clusters. In each graph, the other sex’s expression is co-mapped. **E** Heatmap of enriched Gene Ontology pathways (via GSEA) across NMF clusters reveals both concordance and nuance across female mouse, male mouse, female human, and male human samples

We applied non-negative matrix factorization (NMF) to identify co-expressed genes across stages separately in (i) male mouse (Fig. 6A), (ii) male human (Fig. 6B), (iii) female mouse (Fig. 6C), and (iv) female human (Fig. 6D). NMF analysis of gene expression data provides unsupervised clusters of genes with similar expression patterns across timepoints (Fig. 6A–D). A heatmap describing the results of the gene set enrichment analysis (GSEA) of the NMF clusters shows concordance between enriched clusters in mouse and human that include signals from male and female gametes, possibly remnants of events during fertilization (Fig. 6E) (Additional file 1:Figs. S9–14).

## Discussion

In this study, we compare molecular signals of sex differences at the earliest stages in mammalian embryogenesis, during the first cell divisions immediately after fertilization. Our observations challenge the prevailing view that sexual differentiation occurs only after gonad formation and the subsequent circulation of sex-specific hormones. From our single-cell transcriptomics results in both mouse and human, we observe the establishment of rapidly changing female and male network landscapes that are enriched in X- and Y-linked protein-coding genes involved in sex-specific cellular development (Table 1). Significant differences, both temporal and unique, exist in gene ontological processes among male and female cells during early stages of development. In female cells, biological processes belonging to cell organization and cell compartment are delayed, while in male cells, RNA- and DNA-related processes are delayed. Overall, our results reveal the emergence of early acting sexual networks among males and females, thus, placing prominence on the role of sex chromosomes during early stages of mammalian development [35, 36].

Our work also reveals early developmental differences across two phylogenetically distant mammals. Early stages of embryogenesis are generally characterized by a high degree of morphological and developmental conservation across mammals which has been historically associated to recapitulation [37, 38]. There are several known differences in pre-implantation embryogenesis between mouse and human. Human early development is protracted compared to mouse, with zygotic genome activation at the 8-cell stage, whereas in mouse it occurs at the 2-cell stage. Maternal transcripts also exhibit differences, with human maternal programs extended until zygotic genome activation. Our results corroborate recent decades of molecular studies that have shown that the underlying expression programs between similar processes differ significantly between human and mouse in both content and timing [39, 40]. For example, some of the master regulators that determine the fates of the outer trophectoderm and the inner cell mass act through different pathways in mouse and human or are different altogether [21, 39]. With new technologies, we can now identify critical milestones such as the zygotic genome activation (ZGA) [41, 42] and the first and second lineage segregations that give rise to the future trophectoderm, epiblast, and primitive endoderm.

The availability of single-cell sequencing data and cell-lineage reconstruction provides the opportunity to interrogate sex-specific gene expression across the earliest stages of development and several recent genome-wide studies reveal novel lineage-specific factors in early pre-implantation embryogenesis. For example, Blakeley et al. [43] observed key components of the TGF-B signaling pathway that were enriched in human epiblasts but not mouse while key trophectoderm factors are expressed in mouse pre-implantation embryos but not in similar stages of humans. Applying lineage reconstruction approaches using single-cell RNA-seq data from 8-cell stages to B6 blastocysts, Meistermann et al. [44] found differences in the timing of distinct transcriptomic profiles between mouse and human that are necessary to induce or complete lineage specification. Such pseudotime analyses of sc-RNAseq data to reconstruct early development are powerful approaches to compare conserved and diverged processes and genes involved in cellular identify and fate.

X-chromosome inactivation in mouse and human female cells also exhibit different dynamics. In mouse, the paternal X chromosome is preferentially inactivated, and then reactivated during pre-implantation, with random X chromosome inactivation occurring upon implantation [45]. In humans, both maternal and paternal X chromosomes are initially active, despite biallelic expression of *Xist*, with random inactivation initiating and progressing throughout pre-implantation [17]. Our results reveal differences between mouse and human in X-chromosomal expression levels as well as the fraction of Y-linked expression (Fig. 1B), the number of sexDEGs (Fig. 3A, B), and patterns of sexDEG PPi networks (Fig. 4). One caveat to our cross-mammalian comparison is that the mouse transcription data were derived from natural matings while the human embryonic data originated from cultured IVF cells. While epigenetics does not generally play a role in such early embryonic stages, culturing embryos has been shown to alter some epigenetic marks.

Our results also reveal the existence of temporal shifts in gene expression between these two mammals. For example, several regulatory factors that exhibit sex-biased expression are expressed at different stages in male and female mouse embryos (Additional file 2: Tables S2, S3). For example, *Rfx4*, *Hnf1b*, *Glis3*, *Sox17* and *Smad9* are male-biased at early embryonic stages and female-biased at slightly later pre-implantation stages. Similarly, *Hey1*, *Hoxb13*, *Setd7*, *Irf5* and *Hif1a* are female-biased at earlier stages and male-biased later. These results suggest that, at least for processes regulated by these factors, pre-implantation development is offset between males and females. However, a substantial number of regulatory factors show sex-specific expression throughout this phase, the majority of which are encoded by autosomal genes. In another example from our survey of sex-biased gene expression, *Kdm5d*, a Y-linked histone lysine demethylase, is expressed throughout all stages in the males, whereas the X-linked *Kdm6a* is female-biased only at the 8- and 16-cell stages, either because the paternal X is undergoing reactivation or because it escapes paternal X inactivation, although there is some evidence that many genes from the paternal X are fully active at the earliest stages of mouse development [46].

Interestingly, both mouse and human embryos exhibit a diminishment of sex-biased expression as development progresses towards formation of the blastocyst. We also observe that Y-linked genes in male embryos are featured more prominently in expression patterns compared to X-linked genes in females which may reflect that some of the initial sex differences in expression could reflect that the reprogramming of the Y chromosome, possibly the replacement of protamines, could occur faster than that of the X chromosome provided by the sperm, due to their size differences. More comparative transcriptomic studies of early development will shed light on this dynamic pattern of sex-linked gene expression with both conserved and unique molecular features evolving across mammalian lineages.

### Perspectives and significance

Our study’s conclusions have direct implications on the study of sexual differences across later stages of development and on sex-specific disease [47]. The sex-biased expression of transcriptional and epigenetic factors reported here prompts questions on how sex-specific networks in pre-implantation affect sex-biased gene expression after implantation, as lineage determination and organogenesis proceed, including how hormones interact with pre-established sex differences by compensating for or enhancing them. We also note that because transcription factors are generally expressed at low levels, many sex-biased genes are likely under-represented in single-cell RNA-seq experiments. In addition, relative to our much larger set of human samples, the sample sizes for the earliest stages of mouse embryogenesis are small (Additional file 2: Table S1) and additional studies may increase the power to detect sex-biased expression. Furthermore, the availability of samples from earlier time points for human embryos would allow a more precise match of the developmental stages with the mouse. Thus, we anticipate that there are more differences in gene expression of regulatory factors than detected in our study. With the increasing availability of single-cell RNA-seq data from other species and new empirical and analytical approaches, future studies will allow comparisons of gene expression in pre-implantation stages and beyond across mammals to find common substructures, conserved functional modules, and sex chromosome-dependent regulatory networks.

## Supplementary Information

**Additional file 1: Figure S1.** Distributions of counts per gene for unfiltered and filtered expression data in mouse and human. **Figure S2.** Cophenetic correlation plots of NMF clusters across factorization rank, or the specified number of clusters inputted into NMF, in male mouseand female mouse. **Figure S3.** Cophenetic correlation plots of NMF clusters across factorization rank, or the specified number of clusters inputted into NMF, in male humanand female human. **Figure S4.** Average expression value of each gene from each NMF cluster metagene across samples in male and female mouse. **Figure S5.** Average expression value of each gene from each NMF cluster metagene across human samples of males and females. **Figure S6.** Genes with the highest and lowest principal component scores for the top 15 principal components of gene expression data in mouse. **Figure S7.** Genes with the highest and lowest principal component scores for the top 15 principal components of gene expression data in human. **Figure S8.** Bar plots of the number of interactions between each groupnormalized by the number of DEGs at each developmental stage. **Figure S9.** Median number of enriched NMF clusters per biological processGOSlim term, normalized by cluster size, between mouse male and female. **Figure S10.** Median number of enriched NMF clusters per biological processGOSlim term, normalized by cluster size, between human male and female. **Figure S11.** Median number of enriched NMF clusters per cellular componentGOSlim term, normalized by cluster size, between mouse male and female. **Figure S12.** Median number of enriched NMF clusters per cellular componentGOSlim term, normalized by cluster size, between human male and female. **Figure S13.** Median number of enriched NMF clusters per molecular functionGOSlim term, normalized by cluster size, between mouse male and female. **Figure S14.** Median number of enriched NMF clusters per molecular functionGOSlim term, normalized by cluster size, between human male and female. **Figure S15.** Median number of enriched NMF clusters per biological processGOSlim term, normalized by cluster size, between male mouse vs. humanand female mouse vs. human. Top five terms with the largest difference between mouse and human are labeled. **Figure S16.** Median number of enriched NMF clusters per cellular componentGOSlim term, normalized by cluster size, between male mouse vs. humanand female mouse vs. human. Top five terms with the largest difference between mouse and human are labeled. **Figure S17.** Median number of enriched NMF clusters per molecular functionGOSlim term, normalized by cluster size, between male mouse vs. humanand female mouse vs. human. Top five terms with the largest difference between mouse and human are labeled. **Figure S18.** Heatmap of the normalized number of NMF clusters enriched under each GOslim term for biological process. **Figure S19.** Heatmap of the normalized number of NMF clusters enriched under each GOslim term for cellular component. **Figure S20**. Heatmap of the normalized number of NMF clusters enriched under each GOslim term for molecular function.

**Additional file 2: Table S1.** Transcriptomic datasets used across developmental time and sex.. **Table S2.** Mouse sex-differential expression results. **Table S3.** Human sex-differential expression results. **Table S4.** NMF cluster size and number of genes per cluster. **Table S5.** Mouse male NMF coefficient matrix of gene expression data. **Table S6.** Mouse female NMF coefficient matrix of gene expression data. **Table S7.** Human male NMF coefficient matrix of gene expression data. **Table S8.** Human female NMF coefficient matrix of gene expression data. **Table S9.** Mouse male gene set enrichment analysis results of NMF clusters. **Table S10.** Mouse female gene set enrichment analysis results of NMF clusters. **Table S11.** Human male gene set enrichment analysis results of NMF clusters. **Table S12.** Human female gene set enrichment analysis results of NMF clusters. **Table S13.** Functional annotations of top 10 UMAP-positionally conserved genes between mouse and human from each developmental stage-adjacent group.

## Data Availability

The datasets analyzed in the current study are included in this article, are publicly available and referenced herein.
