# Supplementary material for: Comparative developmental genomics of sex-biased gene expression in early embryogenesis across mammals
Source: Biol Sex Differ. 2023 May 19;14:30. doi: 10.1186/s13293-023-00520-z (PMC10199522; doi:10.1186/s13293-023-00520-z)

SUPPLEMENTARY FIGURES

Supplementary Figure S1. Distributions of counts per gene for unfiltered and filtered expression data in mouse and human.

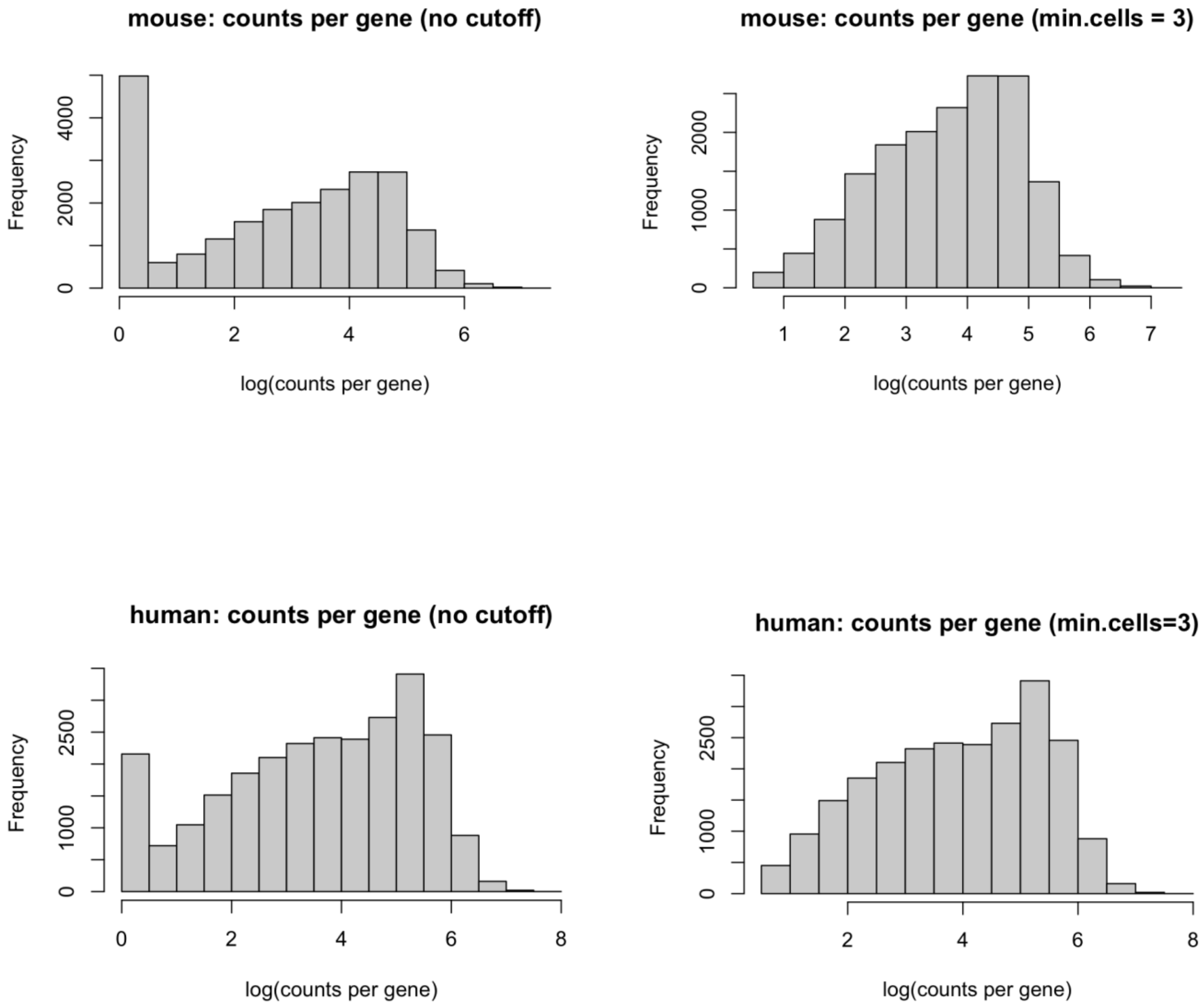

Supplementary Figure S2. Cophenetic correlation plots of NMF clusters across factorization rank, or the specified number of clusters inputted into NMF, in male mouse (top) and female mouse (bottom). Ranks of 16 and 14 were chosen for the male and female groups respectively based on a high cophenetic correlation coefficient, or the stability of the clusters obtained from NMF.

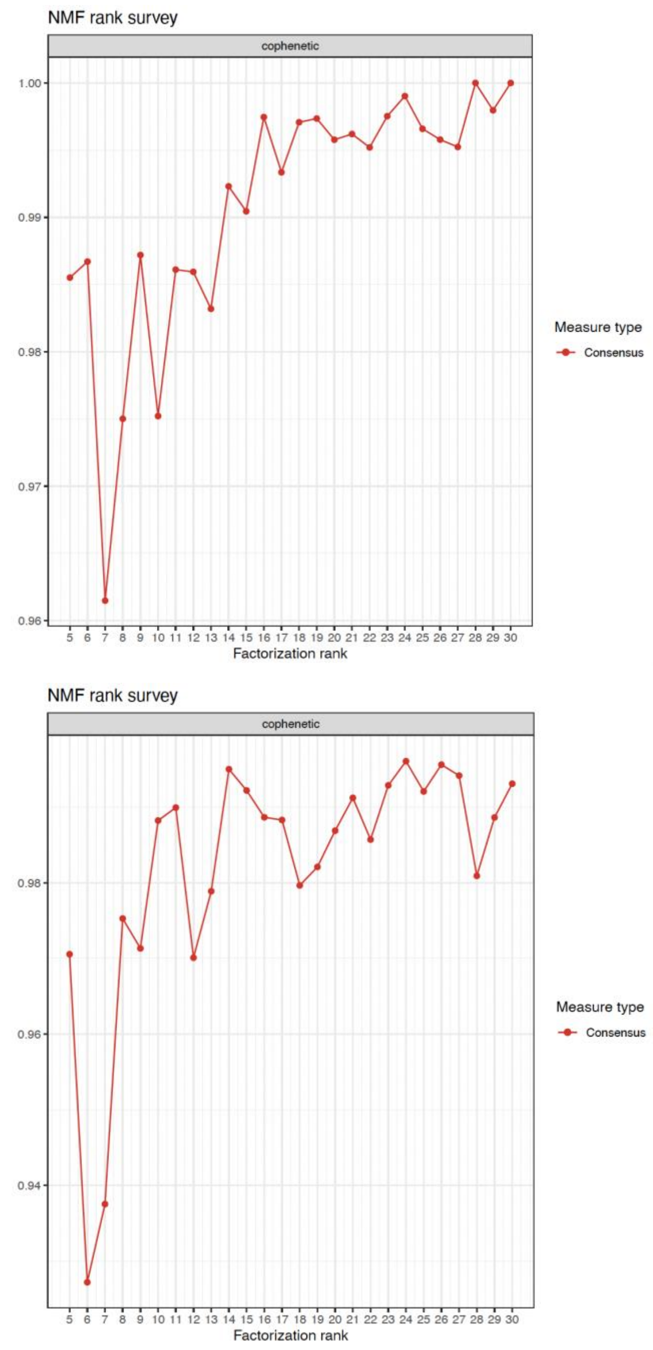

Supplementary Figure S3. Cophenetic correlation plots of NMF clusters across factorization rank, or the specified number of clusters inputted into NMF, in male human (top) and female human (bottom). Ranks of 12 and 7 were chosen for the male and female groups respectively based on a high cophenetic correlation coefficient, or the stability of the clusters obtained from NMF.

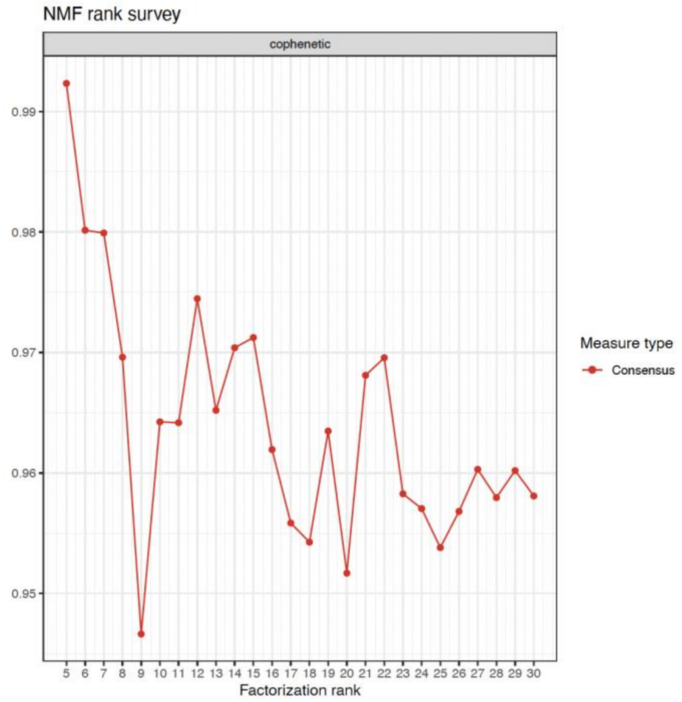

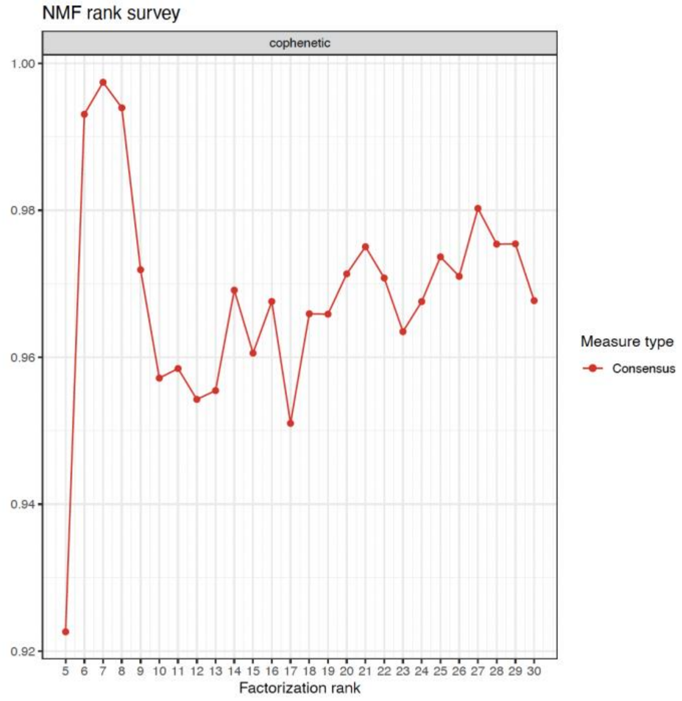

Supplementary Figure S4. Average expression value of each gene from each NMF cluster metagene across samples in male and female mouse. Each plot indicates an NMF cluster (metagene), and plot titles indicate cluster size (i.e., number of genes). Green lines demarcate embryonic stage which scale directly to the number of samples available from each developmental stage (see Figure 1 for stage descriptions).

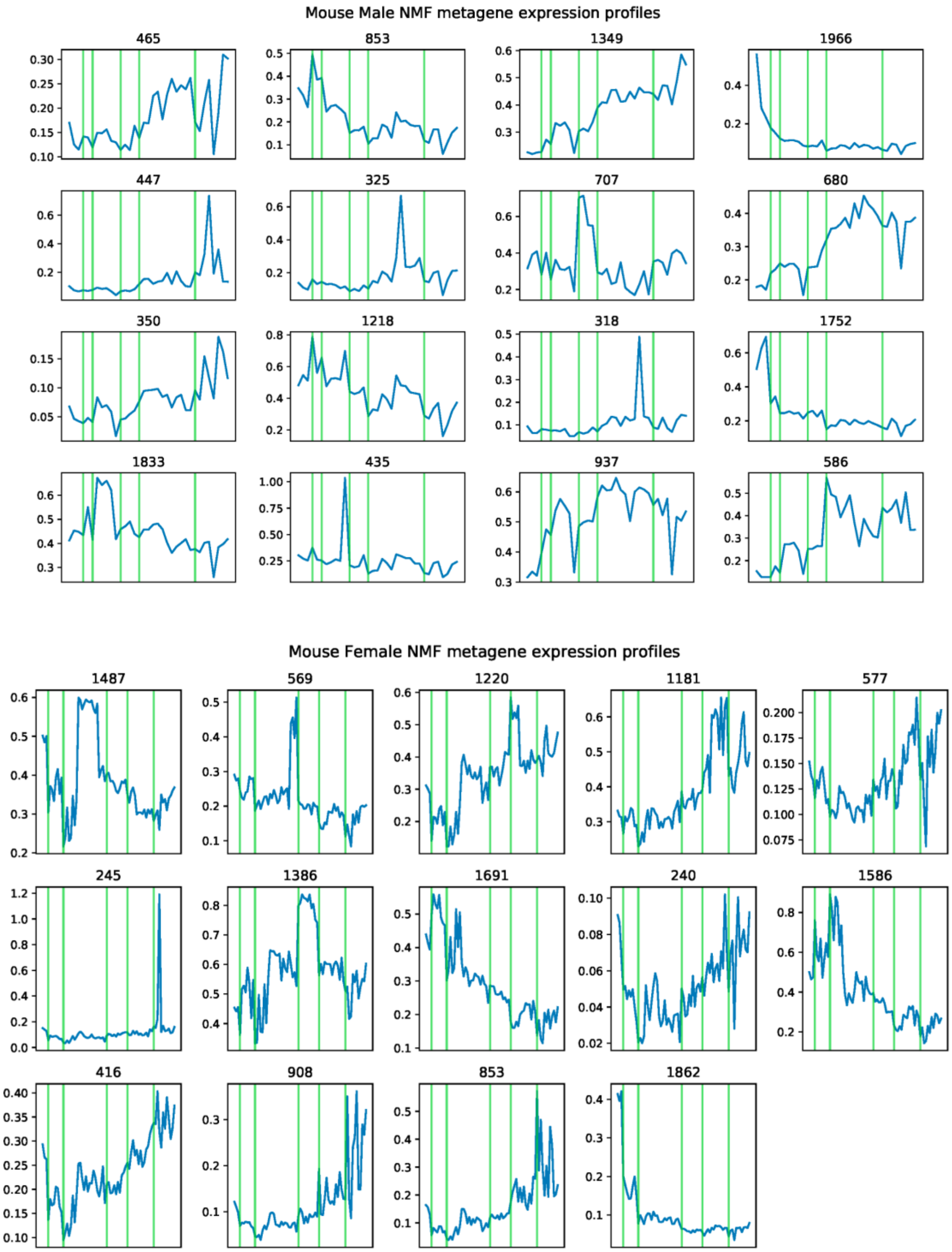

Supplementary Figure S5. Average expression value of each gene from each NMF cluster metagene across human samples of males and females. Each plot indicates an NMF cluster (metagene), and plot titles indicate cluster size (i.e., number of genes). Green lines demarcate embryonic stage which scale directly to the number of samples available from each developmental stage (see Figure 1 for stage descriptions).

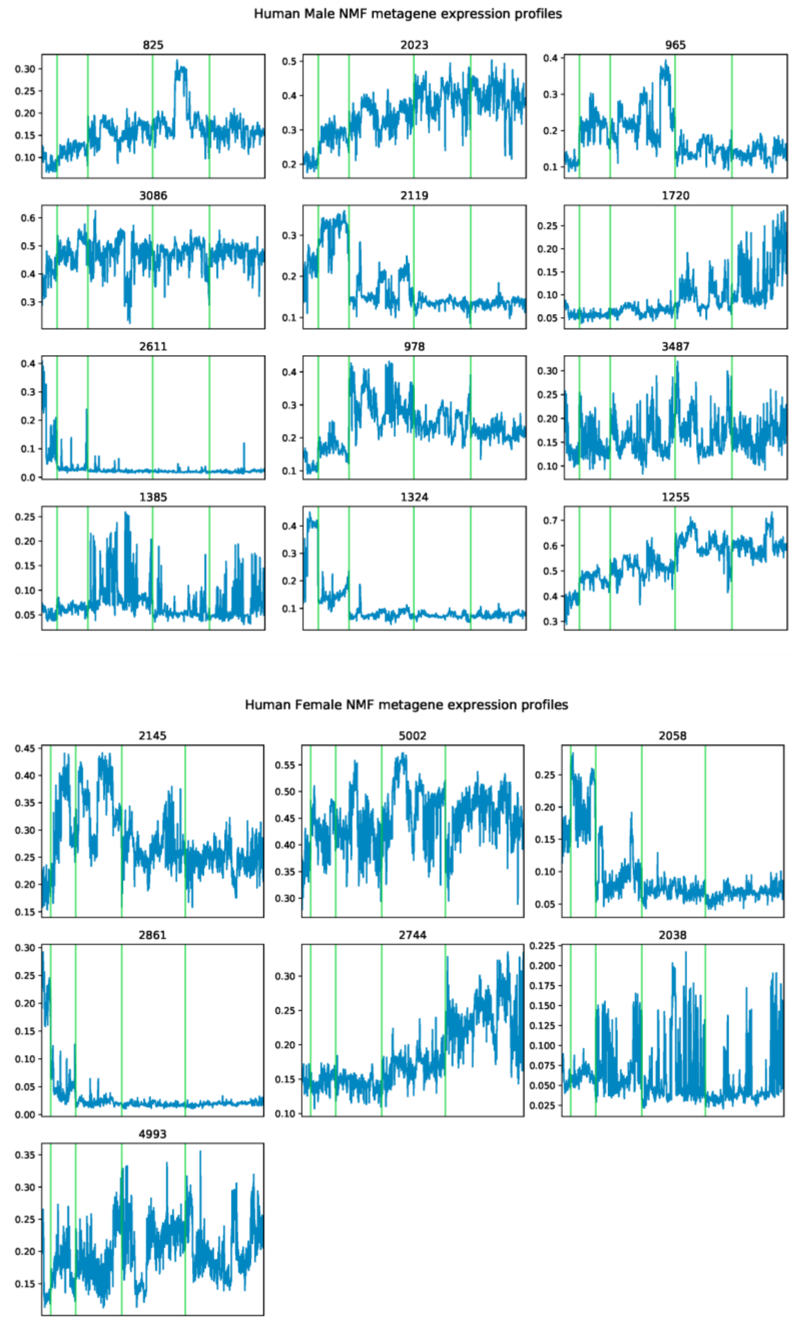

Supplementary Figure S6. Genes with the highest and lowest principal component scores for the top 15 principal components of gene expression data in mouse.

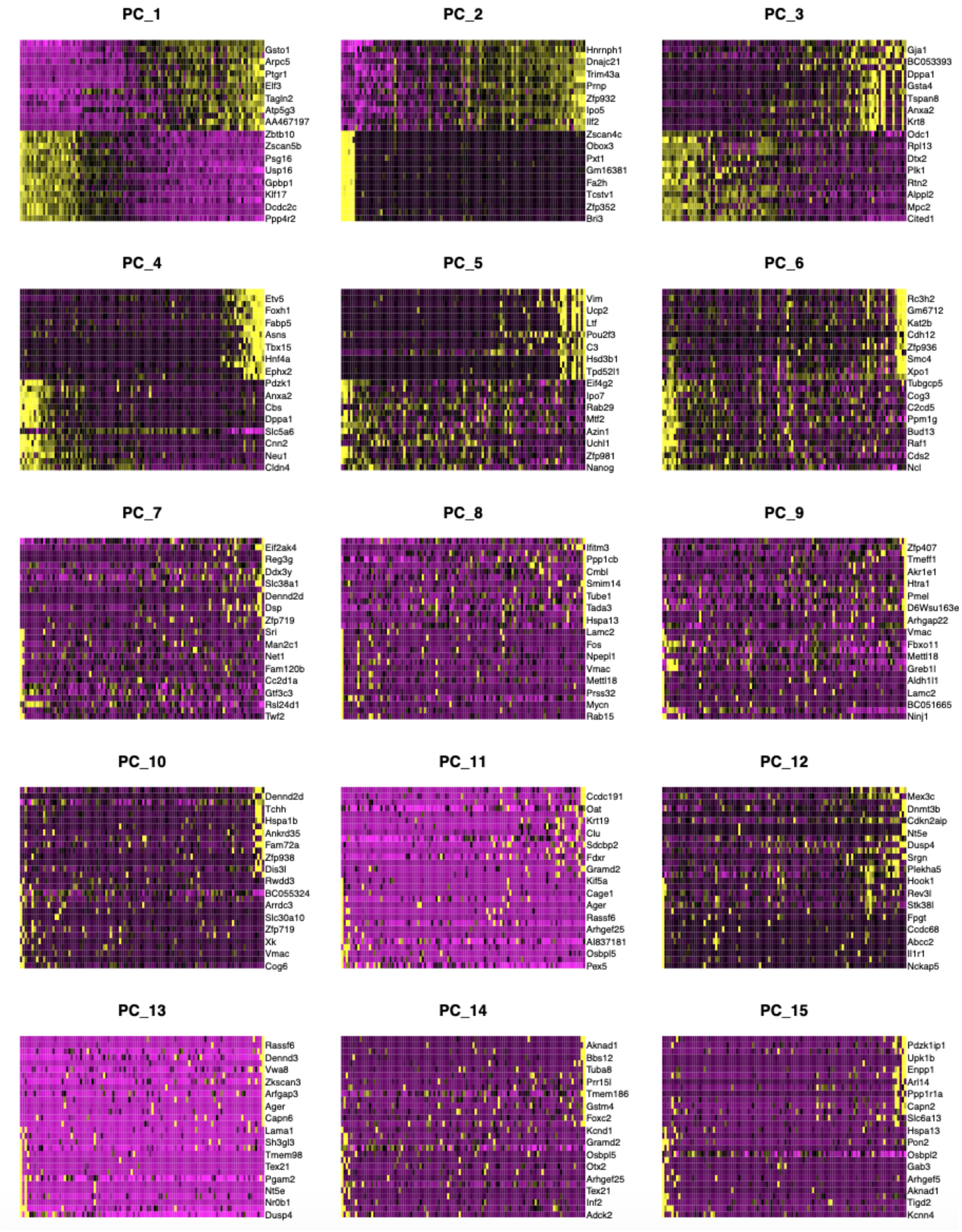

Supplementary Figure S7. Genes with the highest and lowest principal component scores for the top 15 principal components of gene expression data in human.

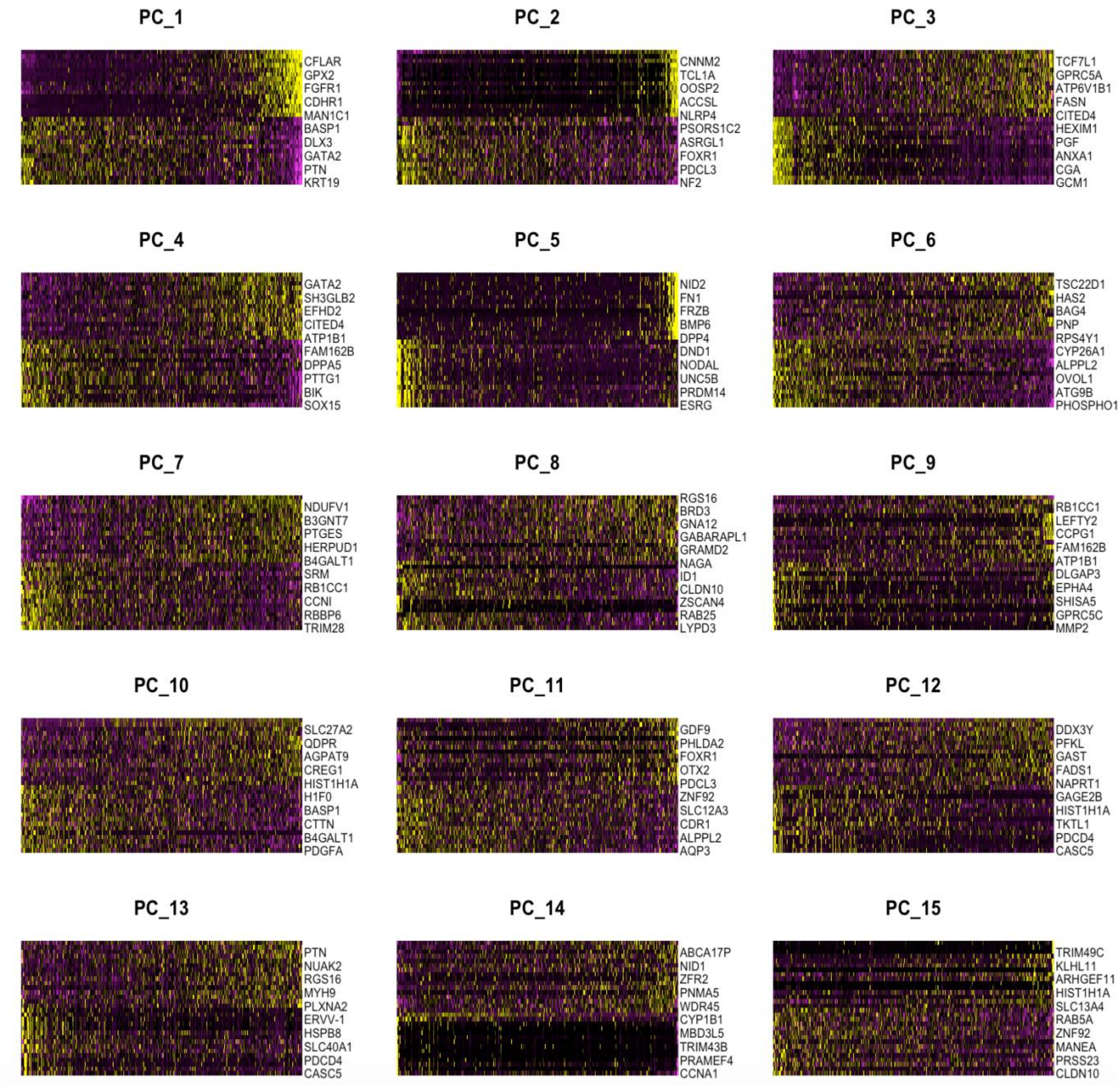

Supplementary Figure S8. Bar plots of the number of interactions between each group (male-female, female-female, etc.) normalized by the number of DEGs at each developmental stage.

Mouse:

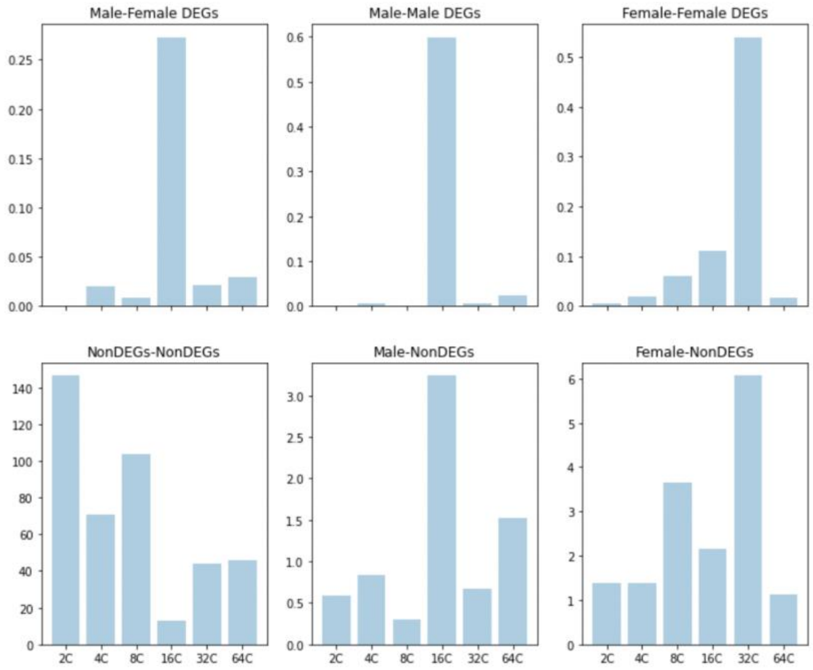

Human:

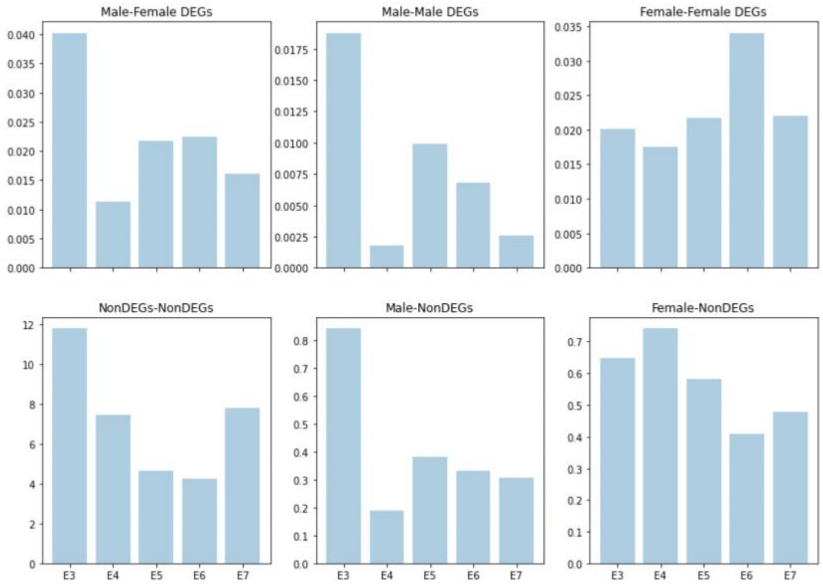

Supplementary Figure S9. Median number of enriched NMF clusters per biological process (BP) GOSlim term, normalized by cluster size, between mouse male and female.

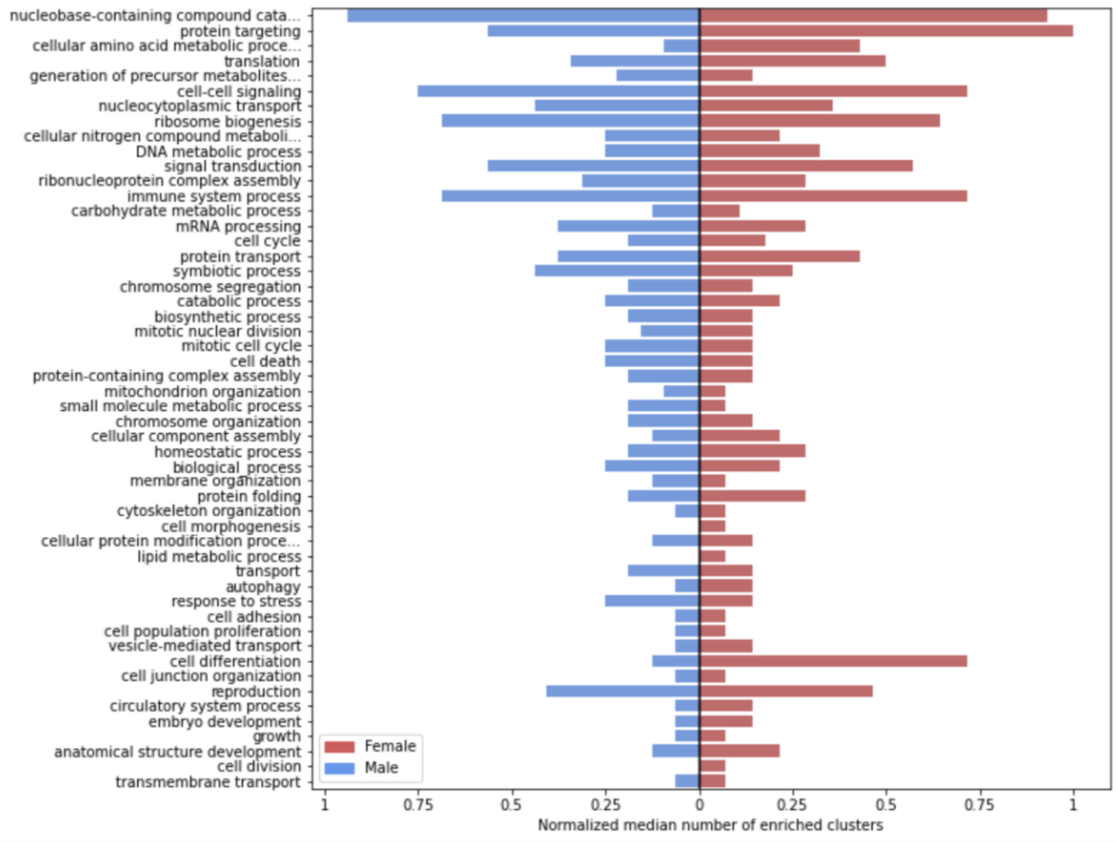

Supplementary Figure S10. Median number of enriched NMF clusters per biological process (BP) GOSlim term, normalized by cluster size, between human male and female.

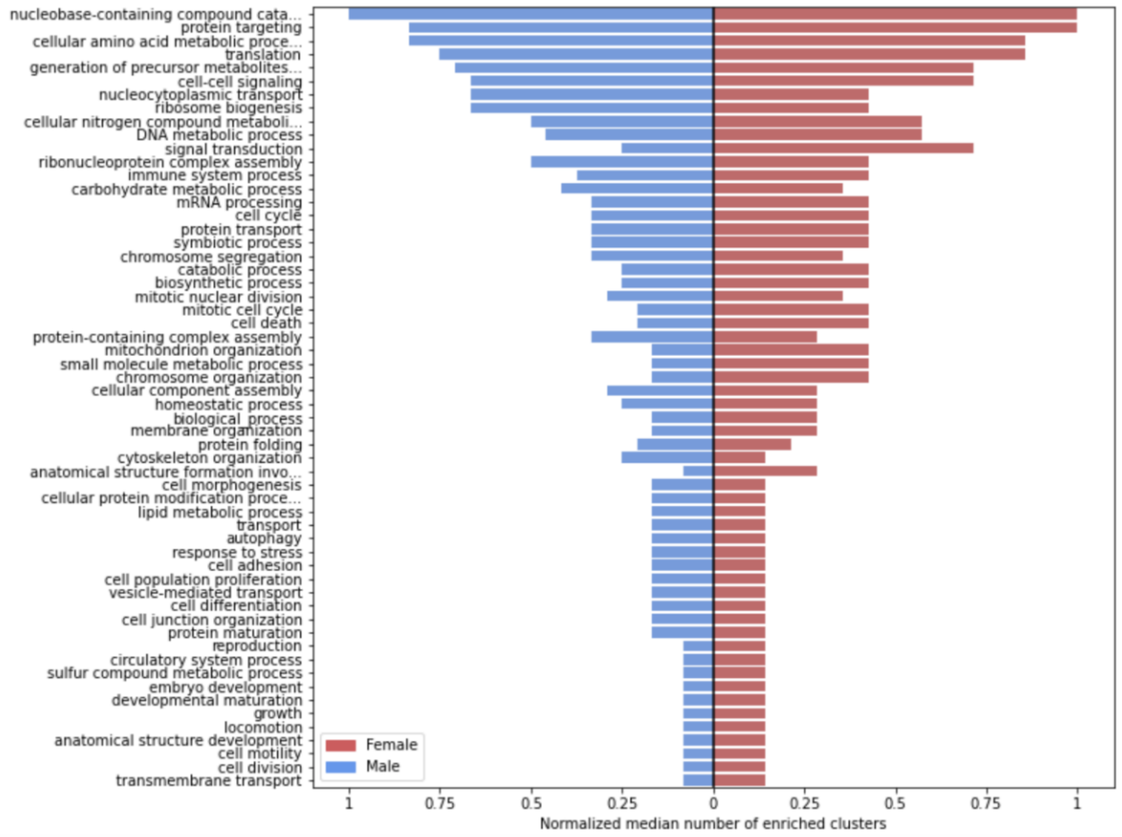

Supplementary Figure S11. Median number of enriched NMF clusters per cellular component (CC) GOSlim term, normalized by cluster size, between mouse male and female.

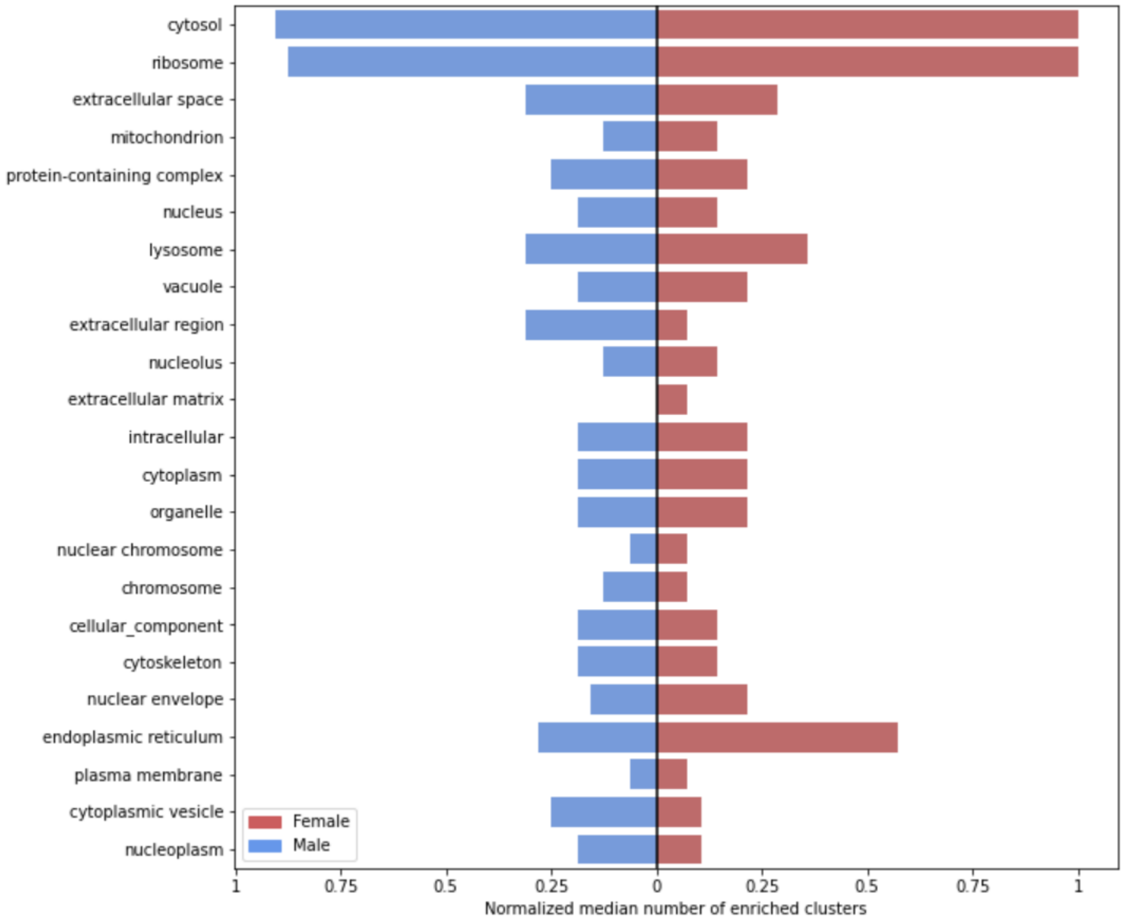

Supplementary Figure S12.
Median number of enriched NMF clusters per cellular component (CC) GOSlim term, normalized by cluster size, between human male and female.

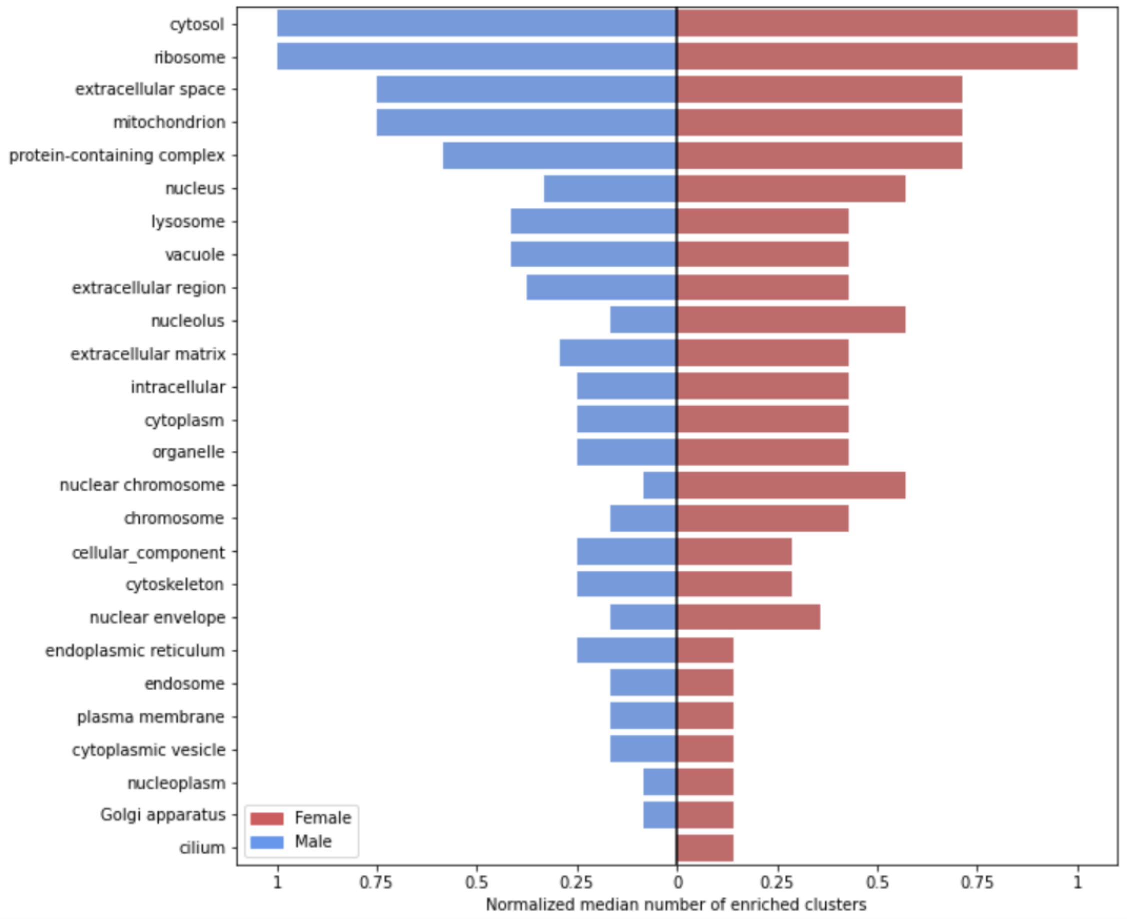

Supplementary Figure S13. Median number of enriched NMF clusters per molecular function (MF) GOSlim term, normalized by cluster size, between mouse male and female.

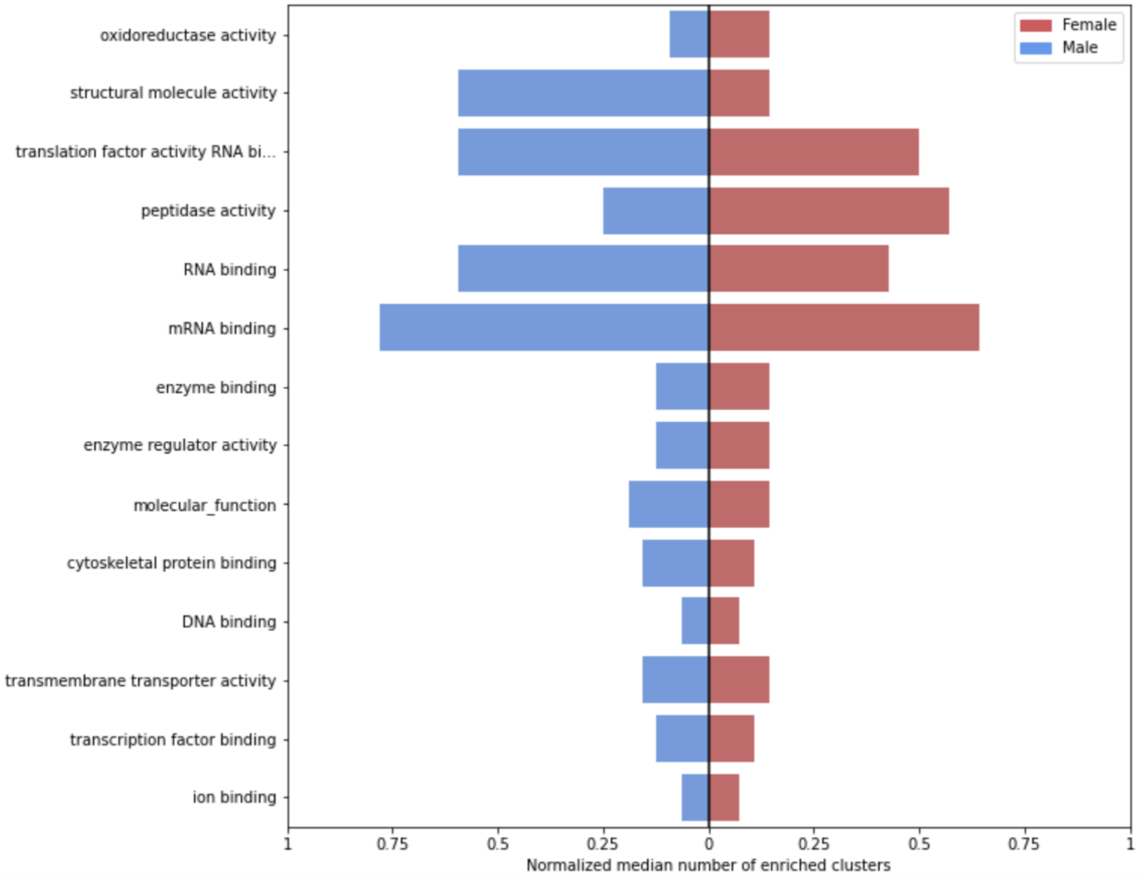

Supplementary Figure S14. Median number of enriched NMF clusters per molecular function (MF) GOSlim term, normalized by cluster size, between human male and female.

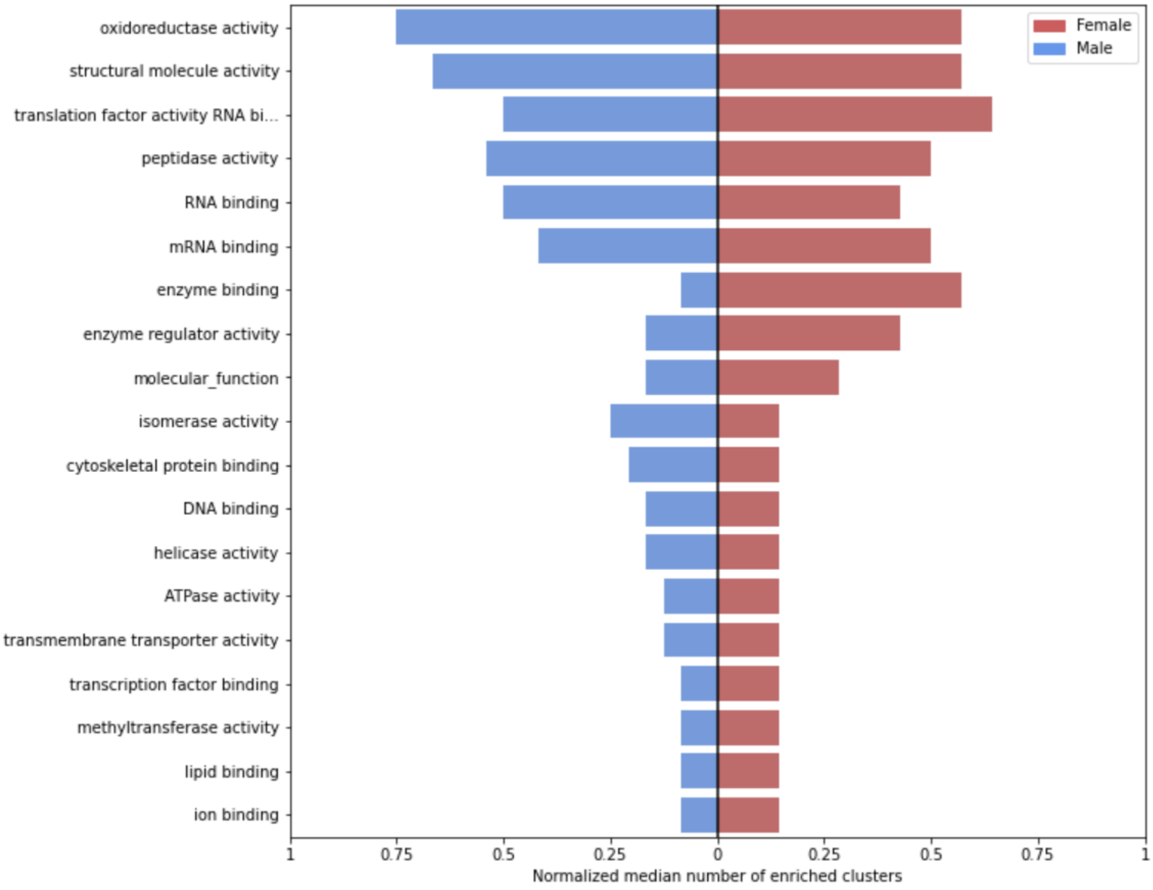

Supplementary Figure S15. Median number of enriched NMF clusters per biological process (BP) GOSlim term, normalized by cluster size, between male mouse vs. human (top) and female mouse vs. human (bottom) . Top five terms with the largest difference between mouse and human are labeled.

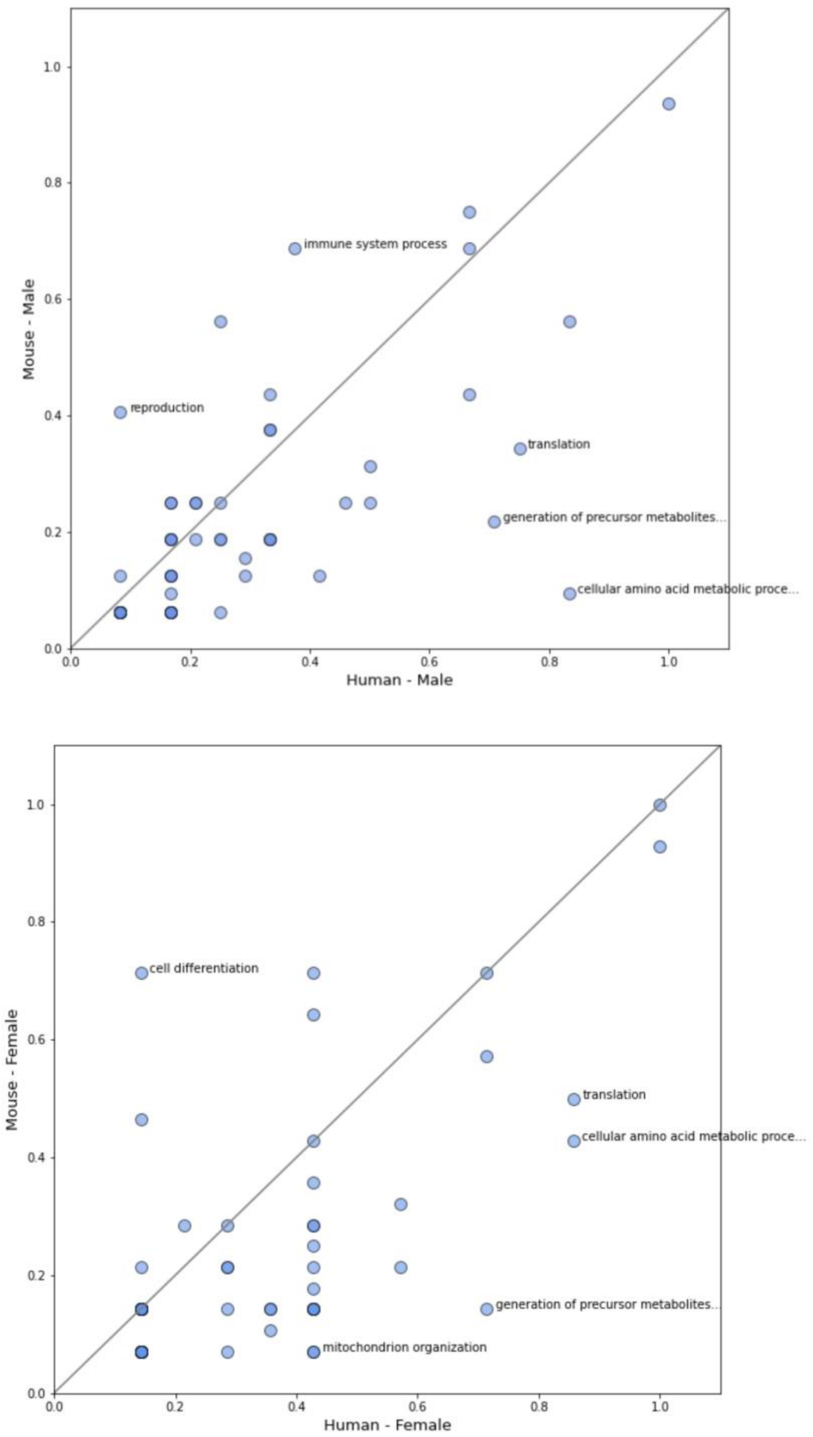

Supplementary Figure S16. Median number of enriched NMF clusters per cellular component (CC) GOSlim term, normalized by cluster size, between male mouse vs. human (top) and female mouse vs. human (bottom) . Top five terms with the largest difference between mouse and human are labeled.

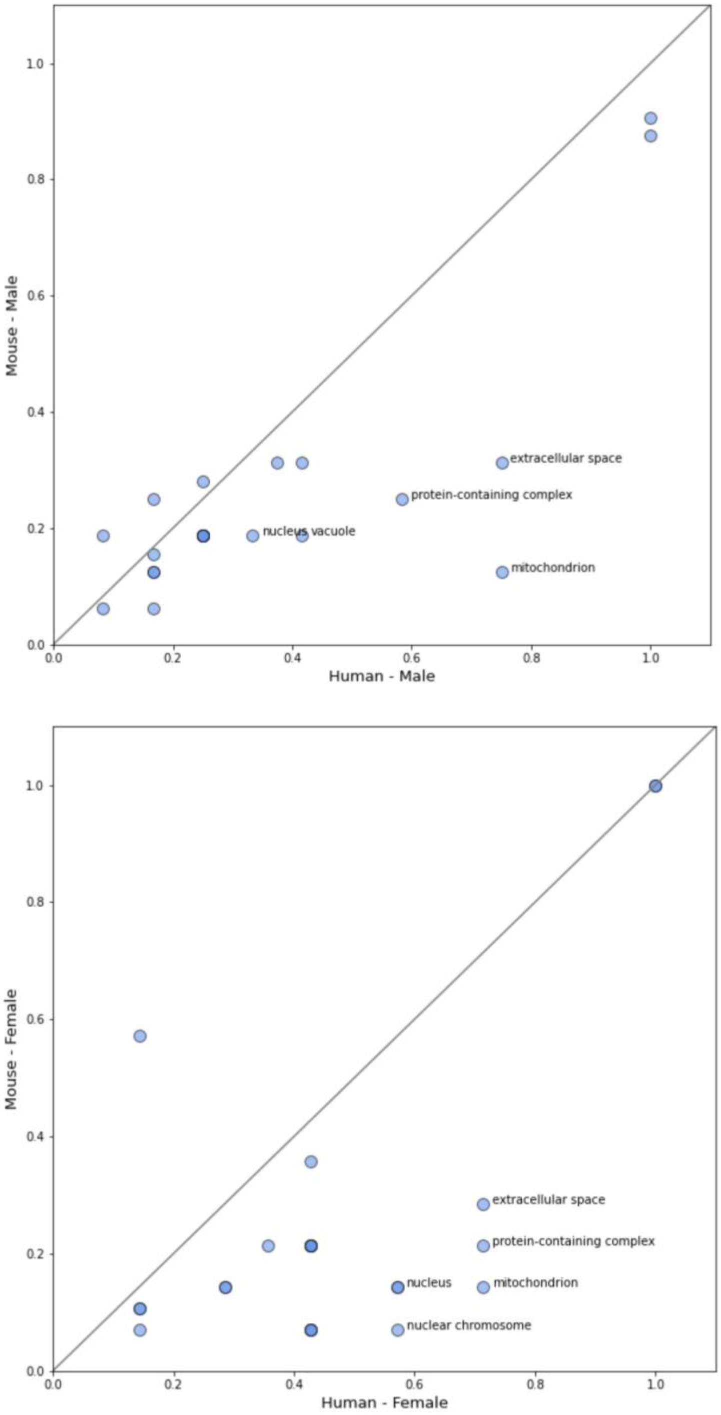

Supplementary Figure S17. Median number of enriched NMF clusters per molecular function (MF) GOSlim term, normalized by cluster size, between male mouse vs. human (top) and female mouse vs. human (bottom) . Top five terms with the largest difference between mouse and human are labeled.

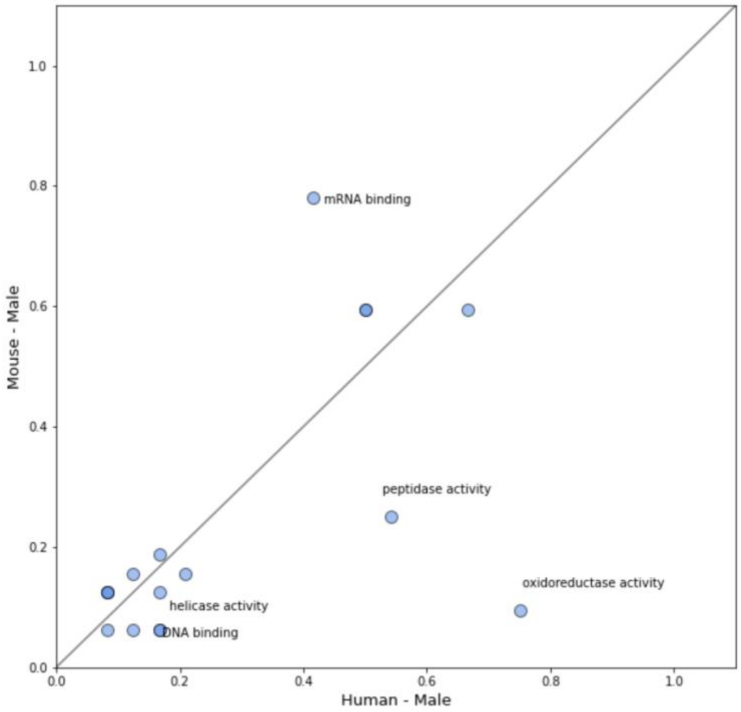

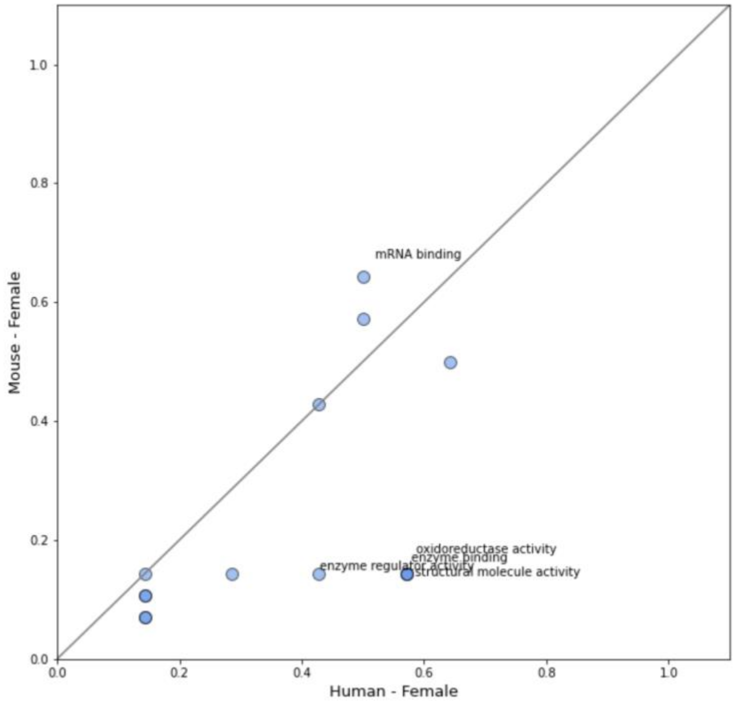

Supplementary Figure S18. Heatmap of the normalized number of NMF clusters enriched under each GOslim term for biological process.

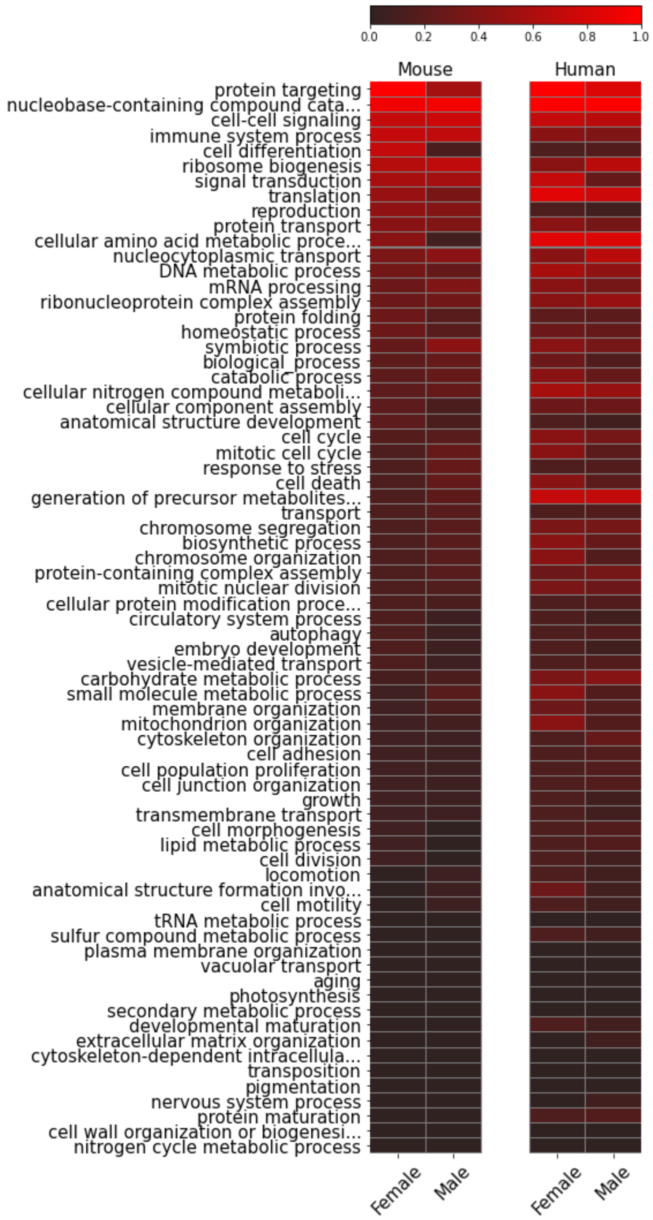

Supplementary Figure S19. Heatmap of the normalized number of NMF clusters enriched under each GOslim term for cellular component.

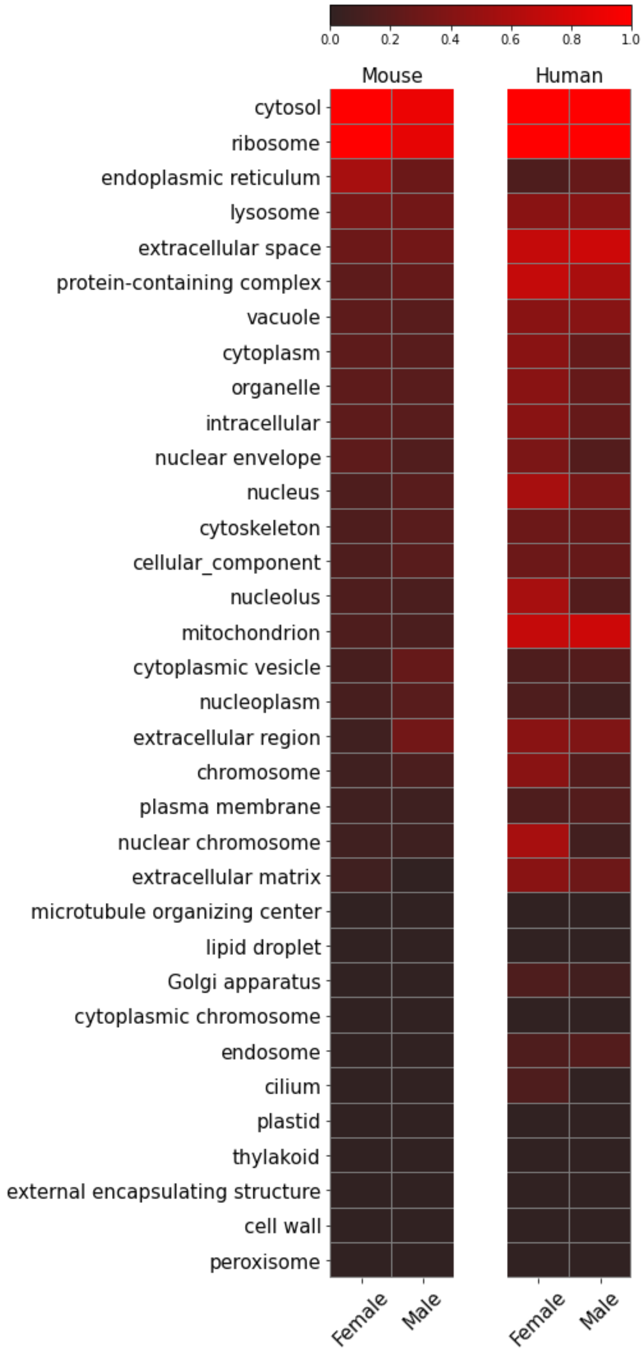

Supplementary Figure S20. Heatmap of the normalized number of NMF clusters enriched under each GOslim term for molecular function.

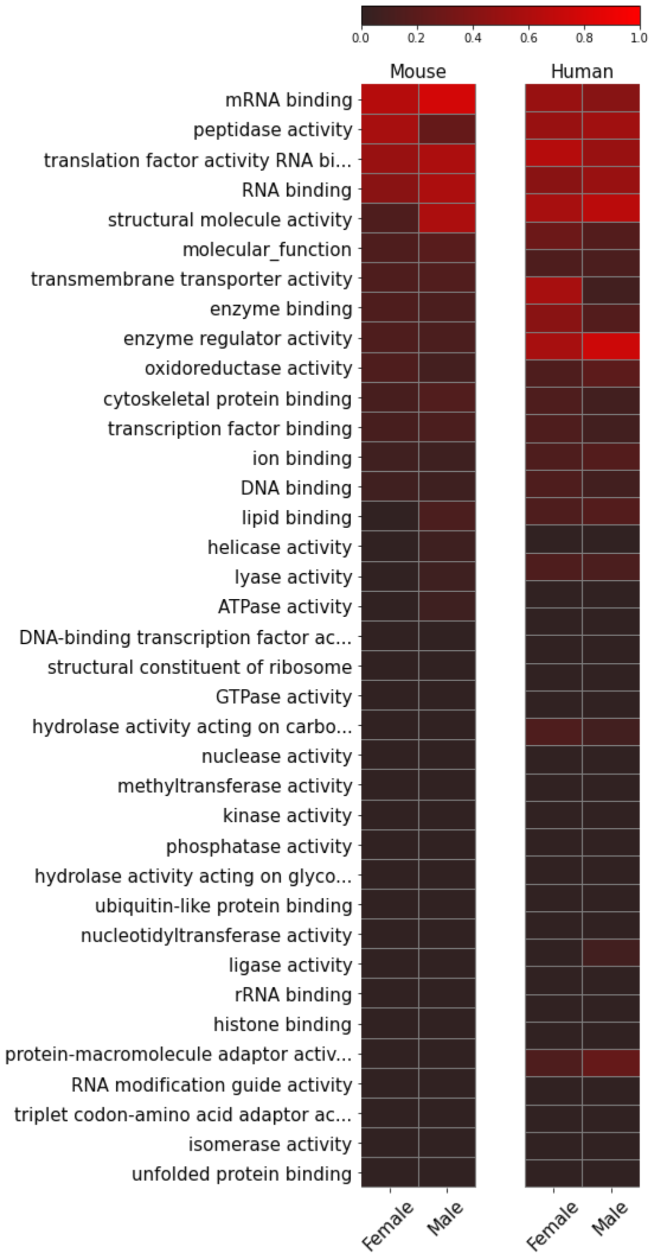

Supplement: Supplementary file 1 — Additional file 1: Figure S1. Distributions of counts per gene for unfiltered and filtered expression data in mouse and human. Figure S2. Cophenetic correlation plots of NMF clusters across factorization rank, or the specified number of clusters inputted into NMF, in male mouseand female mouse. Figure S3. Cophenetic correlation plots of NMF clusters across factorization rank, or the specified number of clusters inputted into NMF, in male humanand female human. Figure S4. Average expression value of each gene from each NMF cluster metagene across samples in male and female mouse. Figure S5. Average expression value of each gene from each NMF cluster metagene across human samples of males and females. Figure S6. Genes with the highest and lowest principal component scores for the top 15 principal components of gene expression data in mouse. Figure S7. Genes with the highest and lowest principal component scores for the top 15 principal components of gene expression data in human. Figure S8. Bar plots of the number of interactions between each groupnormalized by the number of DEGs at each developmental stage. Figure S9. Median number of enriched NMF clusters per biological processGOSlim term, normalized by cluster size, between mouse male and female. Figure S10. Median number of enriched NMF clusters per biological processGOSlim term, normalized by cluster size, between human male and female. Figure S11. Median number of enriched NMF clusters per cellular componentGOSlim term, normalized by cluster size, between mouse male and female. Figure S12. Median number of enriched NMF clusters per cellular componentGOSlim term, normalized by cluster size, between human male and female. Figure S13. Median number of enriched NMF clusters per molecular functionGOSlim term, normalized by cluster size, between mouse male and female. Figure S14. Median number of enriched NMF clusters per molecular functionGOSlim term, normalized by cluster size, between human male and female. Figure [file 13293_2023_520_MOESM1_ESM.pdf]
