# Supplementary material for: Comparative developmental genomics of sex-biased gene expression in early embryogenesis across mammals
Source: Biol Sex Differ. 2023 May 19;14:30. doi: 10.1186/s13293-023-00520-z (PMC10199522; doi:10.1186/s13293-023-00520-z)
Supplement: Supplementary file 2 — Additional file 2: Table S1. Transcriptomic datasets used across developmental time and sex.. Table S2. Mouse sex-differential expression results. Table S3. Human sex-differential expression results. Table S4. NMF cluster size and number of genes per cluster. Table S5. Mouse male NMF coefficient matrix of gene expression data. Table S6. Mouse female NMF coefficient matrix of gene expression data. Table S7. Human male NMF coefficient matrix of gene expression data. Table S8. Human female NMF coefficient matrix of gene expression data. Table S9. Mouse male gene set enrichment analysis results of NMF clusters. Table S10. Mouse female gene set enrichment analysis results of NMF clusters. Table S11. Human male gene set enrichment analysis results of NMF clusters. Table S12. Human female gene set enrichment analysis results of NMF clusters. Table S13. Functional annotations of top 10 UMAP-positionally conserved genes between mouse and human from each developmental stage-adjacent group. [file 13293_2023_520_MOESM2_ESM.pdf]

Supplementary Tables

Supplementary Table S1. Transcriptomic datasets used across developmental time and sex 2
Supplementary Table S2. Mouse sex-differential expression results 3
Supplementary Table S3. Human sex-differential expression results 52
Supplementary Table S4. NMF cluster size and number of genes per cluster 148
Supplementary Table S5. Mouse male NMF coefficient matrix 149
Supplementary Table S6. Mouse female NMF coefficient matrix 379
Supplementary Table S7. Human male NMF coefficient matrix 638
Supplementary Table S8. Human female NMF coefficient matrix 1092
Supplementary Table S9. Mouse male GSEA of NMF clusters 1717
Supplementary Table S10. Mouse female GSEA of NMF clusters 1852
Supplementary Table S11. Human male GSEA of NMF clusters 1912
Supplementary Table S12. Human female GSEA of NMF clusters 1995
Supplementary Table S13. Functional annotations of top 10 UMAP-positionally conserved genes between mouse and human from each developmental stage-adjacent group. 2126

Supplementary Table S1.
Transcriptomic datasets used across developmental time and sex.

Mouse	E1.5	E2	E2.5	E3	E3.5	E4
Male	3	2	6	4	12	8
Female	3	8	23	11	14	12

Human	E3	E4	E5	E6	E7
Male	48	98	206	180	176
Female	33	92	171	235	290

Mouse data derives from single-cell transcriptomes of 41 embryos from various developmental timepoints (Borensztein et al. 2017).
Human data derives from single-cell transcriptomes of 88 embryos from various developmental timepoints (Petropoulos et al. 2016).

Supplementary Table S2

Mouse: differential expression results

Gene	padj_E1.5	log2fc_E1.5	sex_E1.5	padj_E2	log2fc_E2	sex_E2	padj_E2.5	log2fc_E2.5	sex_E2.5	padj_E3	log2fc_E3	sex_E3	padj_E3.5	log2fc_E3.5	sex_E3.5	padj_E4	log2fc_E4	sex_E4
Fdxacb1																0.00514	-3.77291	Male
Plekhh3				0.00853	-1.83615	Male												
Gm4737										0.00493	2.27809	Female						
Ccdc15										0.00000	1.43222	Female						
Sec61g										0.00000	-5.41077	Male	0.00007	1.94508	Female			
Usp5										0.02467	1.69283	Female						
Mppe1										0.00000	5.60231	Female						
Rwdd4a										0.00841	-2.44859	Male						
Evi5l										0.02878	-1.29386	Male				0.02960	-3.95567	Male
Rpia										0.00000	-5.30180	Male	0.00344	2.17864	Female			
Sh2d5										0.00000	-5.19746	Male						
Xlr5a													0.00222	-3.50321	Male			
Snhg6										0.00008	-3.54764	Male						
Lhfp										0.00224	-1.91512	Male						
Mmrn2				0.00004	0.93124	Female												
Slc10a6																0.00001	-6.05510	Male
Rps19bp1										0.00541	-3.19644	Male						
Timm8b										0.00001	-3.82171	Male	0.00948	1.86593	Female			
Cdk14							0.00511	3.67960	Female									
Gfpt2													0.00311	5.33344	Female			
Efhb													0.01147	-3.44539	Male	0.00108	4.36913	Female
Cndp1										0.00285	-1.29659	Male				0.00000	-1.25494	Male
Eef2k																0.02610	3.59078	Female
Alox8										0.00386	-2.52890	Male						
D2Wsu81e										0.04462	2.22777	Female						
Pramel5																0.00332	-1.53824	Male
Rpl3										0.02882	0.93406	Female						
St3gal2													0.00000	6.17789	Female			
Sall1				0.00004	4.16055	Female												
Ccng1				0.02618	1.59409	Female												
Ube2e1										0.00000	5.56333	Female						
Slc38a9										0.00000	3.67057	Female						
Steap1													0.04457	1.88863	Female			
Tmem108							0.00000	1.01149	Female									
Atp6v0e2				0.01890	0.98399	Female	0.00000	-2.67621	Male									
Pfn1										0.00000	4.53822	Female						
Mbd6													0.04102	2.62668	Female			
Slc35b3										0.04958	2.59948	Female						
Rac1										0.02251	-1.30285	Male						
Galns													0.01488	4.34897	Female			
Khdrbs3	0.00000	-2.56800	Male	0.00000	1.94152	Female				0.00451	-1.42879	Male	0.00000	1.57259	Female	0.00000	-1.19161	Male
Dnajc6	0.00007	-2.23308	Male										0.00225	-5.39671	Male			
Vipas39										0.02105	2.69127	Female						
Nat8f1							0.00000	1.00208	Female	0.00013	-1.16987	Male						
Tagln																0.00293	-4.82329	Male
Trmt12																0.01925	-3.26180	Male
Gm12338										0.00000	-4.90393	Male	0.00106	1.71279	Female			
Kat2b																0.02986	3.01617	Female
Ifitm3				0.00000	0.88794	Female												
Btg3							0.00969	-3.97680	Male									
Lrrc2										0.04422	-2.40942	Male						
Plag1																0.03377	-4.49298	Male
Wdr45										0.04923	1.48385	Female						
Poldip3													0.01233	1.01073	Female			
Anxa5										0.00000	1.34952	Female						
Zfp644										0.01843	-3.94087	Male	0.00000	5.23736	Female			
Cep41										0.00450	3.55418	Female						
Efcab5																0.01058	4.00984	Female
Usp26										0.03259	-1.00850	Male						
Naa25										0.00152	2.45727	Female						
Ppp1r16b										0.00000	-4.26653	Male	0.00025	2.00724	Female			
Fbxw19													0.00007	4.89803	Female			
Mfsd6										0.00011	-2.12729	Male						
Ythdf2										0.00055	-2.36489	Male	0.00364	1.51426	Female			
Eif2s3y	0.00028	-6.14121	Male	0.00000	-8.34606	Male	0.00000	-8.11657	Male	0.00000	-9.35471	Male	0.00000	-9.08826	Male	0.00000	-8.22968	Male
Obox2										0.02092	-4.57991	Male						
Tnfsf13b																0.02309	-2.15461	Male
Icam4				0.00000	-2.61720	Male												

Plk1										0.02164	2.98153	Female						
Dnajb4										0.00000	-3.05312	Male						
Gm16157																0.02396	-4.83569	Male
Srgn																0.01420	3.96057	Female
Alg14				0.00168	3.94545	Female												
Pitx1	0.00003	0.74747	Female															
Wnt3a							0.00000	-5.08947	Male	0.00000	3.62858	Female						
Rpl38										0.00000	-5.52442	Male	0.00395	1.46699	Female			
Acta1										0.00000	6.16149	Female						
Gm10865																0.01632	-1.70022	Male
Firre							0.03472	3.49807	Female									
Cox7b							0.04109	1.46185	Female	0.00000	-4.06131	Male	0.00007	1.89555	Female			
Fgd4							0.00015	4.79156	Female							0.00115	-4.14248	Male
Krt7	0.00015	0.61925	Female	0.00072	-1.64017	Male												
Gcm1										0.00001	-0.91730	Male				0.00000	-1.68549	Male
Lrriq4													0.00003	1.01842	Female	0.00001	4.54247	Female
Prr19	0.00193	1.25707	Female															
Aarsd1										0.04098	2.34308	Female						
Khdc3										0.00002	-4.29517	Male						
Bbs10	0.00025	1.05675	Female				0.00000	1.78572	Female				0.03302	3.07102	Female			
Prss45													0.00000	3.25400	Female			
Gm4262										0.00866	-1.88331	Male	0.02393	1.28021	Female			
Rnf32	0.00000	-1.11682	Male							0.00002	-2.24375	Male						
Tenm3													0.00000	-2.72038	Male			
Ccdc177										0.02243	-1.24219	Male						
Rpa3										0.01408	-2.31230	Male						
Slc5a5																0.00919	4.14089	Female
C87436																0.00380	3.07794	Female
Gjb3				0.00211	2.96843	Female												
Mtl5										0.01679	2.87412	Female				0.00265	0.64390	Female
Pnp2																0.01368	-1.72884	Male
Hdac2							0.04953	1.50779	Female									
Phlda3				0.00000	-0.60011	Male												
Adamts10				0.00212	-1.47847	Male												
Atp8b1							0.00756	4.21216	Female									
Slc25a5							0.01222	1.15322	Female									
Ltf										0.00013	1.65296	Female						
Gm7325	0.00002	1.99378	Female				0.00000	-0.98662	Male									
Prtg				0.00000	-1.11132	Male	0.00000	2.44361	Female	0.00129	-1.58329	Male						
Mogat2				0.00886	-2.09344	Male										0.02170	4.78222	Female
Msc							0.01139	3.69329	Female									
Peli2							0.00000	-1.09913	Male	0.00303	-1.52452	Male						
Apol6										0.00133	-3.61898	Male						
Zfp458																0.00000	3.72621	Female
Rab17							0.04109	2.06799	Female									
Pih1d2				0.00000	2.04613	Female												
Nab1										0.00000	-4.46070	Male						
LOC102634812							0.00004	2.28771	Female	0.00000	-5.60601	Male	0.00000	2.52966	Female			
Baz2b				0.02884	3.54772	Female												
Glrx2										0.00001	-3.52869	Male						
Hal				0.00000	-2.60856	Male				0.00000	0.58535	Female						
Cdk16										0.00157	1.98727	Female						
Atp6v1e1										0.00816	1.49817	Female						
Dbi										0.00006	-3.83307	Male						
Snora69													0.02348	4.09287	Female			
Vmn2r.ps129										0.00000	-5.56052	Male	0.00001	2.46528	Female			
BC065397																0.03087	3.83885	Female
Hand1																0.00657	3.09911	Female
Shroom4													0.00085	4.98337	Female			
Herpud1				0.00316	-2.17867	Male				0.00000	3.21441	Female						
Smim24																0.00000	1.59549	Female
Csl										0.00341	1.73232	Female						
Src										0.00000	1.40145	Female						
Kbtbd13				0.00001	-0.67215	Male												
Ergic2										0.02456	1.71453	Female						
Flt3l																0.00053	-4.87323	Male
Foxa1																0.00000	4.63820	Female
Tmem173				0.00002	3.19806	Female				0.00685	-1.20603	Male						
Nmrk1										0.00011	-3.20294	Male						
Klf17																0.00003	-4.68710	Male

Pef1										0.01118	1.98500	Female						
Emx2os							0.01924	-3.60325	Male									
Dut										0.00000	-3.82686	Male						
Med9										0.01672	-2.75621	Male						
Retsat				0.02785	1.91303	Female												
Tfe3				0.00157	3.87909	Female												
Ano10										0.00005	-2.28747	Male						
Foxc1				0.01121	-2.06925	Male												
Mocs2							0.01325	4.05283	Female									
Prrt3													0.00000	-1.51487	Male			
Ero1lb																0.00401	-4.96907	Male
Cdh23																0.00000	-1.84145	Male
Kng2																0.00207	-3.50856	Male
Llgl2										0.00853	3.82171	Female						
Gm5148										0.00000	-3.67239	Male	0.00156	1.51817	Female			
Thnsl1										0.00000	-0.73443	Male	0.00000	5.31457	Female			
Eef1a1										0.00248	1.05993	Female						
Gm19434										0.00956	-2.23278	Male						
Midn										0.04425	-2.97189	Male						
Chrna10																0.02986	-4.01453	Male
Psmg1										0.02776	1.86284	Female						
Smtnl2										0.00000	-3.76921	Male	0.04102	1.34324	Female			
Ankrd24							0.00000	1.91613	Female									
Slc19a2				0.00000	2.12996	Female												
Cast																0.00076	-2.81264	Male
Cd300lg				0.00000	2.50401	Female										0.00000	0.94834	Female
C1rl							0.00834	-4.01326	Male									
Ninl																0.00000	5.53655	Female
Hacd1				0.03245	-1.18456	Male												
Atp12a							0.00000	-1.98485	Male									
AA619741										0.02291	-4.94562	Male						
Arid5b										0.00000	1.80734	Female						
Pdgfra										0.02682	3.28070	Female						
Miox													0.04033	-3.49798	Male			
Hk1																0.00958	3.02725	Female
Chil5				0.00851	0.79913	Female												
Plek										0.00090	-4.78016	Male						
Ctnna1										0.04871	1.09821	Female						
Nfatc1													0.00000	4.68039	Female			
Cbfa2t2																0.04412	1.64376	Female
Klhdc3										0.00000	4.81172	Female						
Nfatc2ip										0.00010	-2.60777	Male						
Capn6										0.00000	1.43578	Female						
Gsdmc4							0.02820	3.88482	Female							0.00054	5.09059	Female
Tmem206										0.00000	-4.37510	Male						
Zfp316										0.00000	-2.88733	Male						
Rogdi										0.02933	2.04793	Female						
Rad23b										0.00553	-1.39290	Male						
Ccdc58										0.01973	-1.43701	Male						
Jund										0.00059	-4.45970	Male						
Il34													0.00083	5.57799	Female			
Purg							0.00000	0.70268	Female									
Cntln													0.01651	4.26157	Female			
Mpdu1										0.01369	1.95603	Female						
Magee1													0.00000	1.87103	Female			
Gm10046										0.00932	-0.96461	Male						
Pip4k2c										0.02224	1.98694	Female						
Lta										0.01431	-1.16353	Male				0.00000	-0.74065	Male
Ppp1r14b										0.04535	-2.21667	Male						
Fsd1l							0.01448	3.38186	Female									
Uhmk1																0.00243	3.82479	Female
Frk													0.00000	6.34356	Female			
Cdc20										0.00570	1.85891	Female						
Maoa													0.00237	3.31403	Female			
Neu3										0.00000	-4.24098	Male						
Cpsf4										0.00059	3.52305	Female						
Slc10a1										0.02491	-3.97601	Male						
Zbtb37																0.00000	1.14491	Female
Lhfpl5	0.00000	-1.39584	Male															
Slc44a3													0.02446	4.00732	Female			

Epb41l3				0.00000	2.42946	Female										0.00000	3.59365	Female
Smim8				0.01178	1.86348	Female												
Omd							0.04281	1.80604	Female	0.00000	-6.39173	Male	0.00536	1.83930	Female			
Gm2837																0.02200	3.81967	Female
Dpm3										0.02644	-2.69261	Male						
Ecel1							0.00022	-0.63435	Male									
Gm20337							0.02585	2.12741	Female	0.00000	-4.27736	Male				0.01430	-2.13152	Male
Rpl32l										0.00000	-4.29586	Male	0.00025	1.51050	Female			
Nt5e							0.00001	1.63449	Female				0.00000	1.87843	Female			
Kpna6										0.00121	2.05206	Female						
Gm10548										0.00464	-2.71119	Male						
Gapt																0.00000	-0.98692	Male
Tcam1																0.00014	4.63207	Female
Tcstv3																0.00000	-6.51847	Male
Nek9				0.03790	2.94394	Female												
Cracr2a																0.00000	-2.23258	Male
Mysm1																0.01683	-1.52448	Male
Ebpl										0.02925	-3.98087	Male						
Brd4										0.02764	1.67126	Female						
Rad1													0.00206	1.23502	Female			
Ppl				0.00890	-1.87736	Male												
Inadl										0.04777	3.12091	Female						
Urb1																0.02592	2.07015	Female
Dnajc11										0.02045	1.69288	Female						
Tomm7										0.00000	-5.14391	Male	0.00195	1.45370	Female			
Shcbp1l				0.00000	1.03556	Female										0.00000	-4.75315	Male
Tmem30b																0.00182	-5.15733	Male
Gm17801										0.00000	-5.76689	Male						
Gm13078										0.00000	3.74294	Female				0.00927	-1.82513	Male
Manea																0.01310	3.53146	Female
Syt12										0.00003	-3.39901	Male						
Rabgap1l				0.00000	1.02126	Female				0.00002	-2.66663	Male				0.00000	2.17764	Female
Sepp1				0.00000	2.12798	Female										0.00000	3.74677	Female
Rasip1				0.01003	-1.53147	Male				0.00000	2.63768	Female						
Nr3c1																0.01925	4.56282	Female
Rpgrip1l																0.00898	3.83739	Female
Gzmm													0.00001	5.57221	Female			
Nsmce4a										0.00460	-2.14869	Male						
Apod										0.04425	-3.59747	Male						
Srek1																0.00814	2.12831	Female
Svbp													0.04821	3.48128	Female			
H2.T3										0.00460	-3.55126	Male				0.00010	-3.61662	Male
Gm13498										0.00000	3.96194	Female						
Asap2																0.01490	2.82365	Female
Grwd1										0.01567	1.83976	Female						
Rrm1										0.00556	1.27071	Female						
Cyp2b10				0.00166	0.92526	Female												
Fkbp2										0.00598	-2.54158	Male						
Uqcrh										0.00000	-4.46858	Male	0.00017	1.76873	Female			
Krt19										0.00001	-3.15208	Male						
Gm11961										0.00000	-3.43855	Male						
Zfp937																0.02476	2.00005	Female
Dppa3										0.01938	-0.91937	Male						
Vasn																0.00000	3.26973	Female
Cldn12																0.03018	-3.02657	Male
Ptpn9																0.00834	3.40619	Female
Gm5113							0.01406	2.44254	Female									
Mina										0.00027	3.90902	Female						
Serpinb6b													0.04410	2.53972	Female			
Acp7																0.00000	2.98426	Female
Myl6b	0.02308	4.64816	Female															
Ddx18										0.00402	1.61139	Female						
Aph1c													0.02267	3.53359	Female			
Ier5													0.00000	-0.58678	Male			
Dennd5b							0.00866	2.20824	Female	0.04371	-2.00823	Male						
Fmo5										0.00807	-4.98537	Male						
Pdap1										0.00000	-3.73668	Male						
Pik3cb										0.04422	-2.71645	Male						
Rps12										0.00000	-3.65519	Male	0.03290	1.26914	Female			
Ndufa13										0.00015	-3.17128	Male	0.02665	1.59217	Female			

Casd1				0.00545	-0.69772	Male	0.00000	2.00729	Female							0.04721	3.99700	Female
Mog													0.00000	2.67401	Female			
Ccdc7b										0.01661	-5.05499	Male						
Mrps5	0.00005	5.45015	Female															
Hapln4	0.00000	-0.81156	Male															
Tmem198				0.02027	-1.46130	Male												
Rel							0.00000	2.75289	Female	0.00003	1.01418	Female						
Pld2													0.04642	2.65417	Female			
Rbl2										0.00000	2.65393	Female						
Coq3																0.01639	-1.66709	Male
Zcchc17										0.00333	2.97078	Female						
Ryr3													0.03811	4.69088	Female			
Chac2				0.00284	1.48131	Female												
Lysmd3				0.00000	1.53543	Female										0.00426	-5.13692	Male
Cox4i2													0.01621	-3.35622	Male			
Fam129a				0.00903	1.14211	Female												
Gm7534																0.00000	-0.98676	Male
Zfp184										0.01186	3.20760	Female						
Trim7										0.00000	9.61748	Female						
Tmem45b										0.00051	4.57552	Female						
Drosha																0.03851	-1.71703	Male
Nudt16							0.02274	-2.42447	Male									
Bloc1s1	0.01889	3.80662	Female							0.00002	-4.20511	Male						
Gpr157										0.00341	-1.85650	Male						
Gm5										0.01588	-3.77921	Male						
Lrrc31										0.00000	-4.45198	Male						
Fam69b				0.00021	1.34843	Female												
Dhps							0.04338	1.85432	Female	0.02540	-2.14596	Male						
Gm5039										0.00525	2.51154	Female						
Romo1										0.00107	-3.25567	Male						
Tapbpl										0.00000	-5.69813	Male	0.00051	2.17742	Female			
Cnksr1				0.00000	0.85412	Female							0.01272	4.12640	Female			
Cat							0.01182	-3.36692	Male									
Insig2																0.01368	4.14387	Female
Npr3	0.02417	0.91612	Female													0.00000	2.39946	Female
AA792892																0.04222	-1.31952	Male
Slc18b1																0.00869	3.36203	Female
Slc17a8										0.00059	-3.24359	Male						
H2.T24										0.00000	-4.82018	Male	0.03762	1.56804	Female			
P3h3										0.00000	-6.14815	Male	0.01837	1.65264	Female			
Ptbp1										0.00668	1.36793	Female						
Anxa11										0.04498	-2.03979	Male						
Pag1																0.03241	3.50257	Female
Lbh				0.00000	1.34646	Female										0.00000	5.75167	Female
Usp29										0.02265	-3.18995	Male						
Prelid2																0.00078	-1.99170	Male
Trappc1				0.00075	3.98929	Female												
Zfp606				0.00022	-0.72603	Male				0.00000	2.16074	Female						
Acyp1										0.01183	-3.23495	Male				0.00372	3.05656	Female
Fcho2													0.00082	-2.81176	Male			
Ptpn4																0.01161	4.12950	Female
Mmp17	0.00267	1.32744	Female													0.00000	2.84252	Female
Pcbp2										0.00724	1.76590	Female						
Pla2g12b													0.00000	3.11887	Female			
Rsrc2																0.02694	-1.11011	Male
Psmc5										0.01976	1.14517	Female						
Slc2a8																0.04721	-1.95855	Male
Rn4.5s										0.00032	-4.36233	Male						
Zfp503										0.00022	-2.36617	Male						
Sdr39u1										0.00016	5.68025	Female						
Zfp341																0.02986	3.39606	Female
Mrgprb1										0.01135	-3.36892	Male						
Cobl				0.00000	3.43332	Female												
Wbscr25							0.00045	-2.15392	Male									
Stambpl1				0.00173	-0.62385	Male				0.04411	-0.99098	Male						
Cycs										0.04762	-1.00670	Male						
Ica1l													0.03392	-3.76336	Male	0.00000	4.70515	Female
Ubr5													0.01123	-1.25284	Male			
Vmp1																0.02484	-1.02125	Male
Cd9										0.00807	1.54067	Female						

Cox19	0.01977	-3.71392	Male															
Nudt12				0.00000	0.76686	Female	0.00000	4.86001	Female	0.00000	0.83194	Female						
Taco1										0.01584	3.73669	Female						
Ccl9																0.01180	-3.03112	Male
Lgals12				0.03842	-1.09920	Male												
Ppp1r26				0.00822	3.21524	Female												
Prkcg																0.01925	-2.42796	Male
Stk25										0.01785	2.31376	Female						
Ndrg2				0.03818	-1.50999	Male												
Gm6548										0.00000	1.61925	Female						
Mir682										0.00000	-5.33800	Male						
Pex26				0.02807	2.19265	Female	0.02064	3.28156	Female									
Fbxl21							0.00202	-0.95095	Male	0.03317	-0.58887	Male						
Emg1										0.00000	2.91767	Female						
Oscar	0.00000	-2.18917	Male															
Csrnp2																0.00542	-3.12642	Male
Epb41l1				0.00000	0.73440	Female												
Galnt7				0.00015	2.95637	Female				0.02482	-0.72283	Male						
Acta2										0.00002	5.35068	Female						
Tsacc										0.04690	-3.49793	Male						
Zfp940				0.00368	0.60130	Female				0.00002	0.79507	Female						
Agrp	0.00000	2.73842	Female															
Bbs12										0.01411	-1.19897	Male						
Zfp467													0.00000	-1.45844	Male	0.00000	-1.57971	Male
Crtam							0.00000	3.15926	Female									
Fstl3										0.00016	5.94318	Female						
Crxos										0.00000	-2.39659	Male						
Mageb16.ps1										0.00115	3.25692	Female						
Ddb2										0.00002	5.20697	Female						
C3													0.00000	-3.16136	Male			
Tada3																0.01977	-2.13687	Male
Gm561										0.00000	-5.21377	Male	0.00001	2.98591	Female			
Ctsk				0.00004	2.03092	Female												
Prkx							0.01016	3.31095	Female									
Luc7l										0.01788	1.56621	Female				0.00019	-1.79954	Male
Tex40	0.00001	1.37656	Female	0.00000	-0.76392	Male												
Pou4f1													0.00000	-2.75635	Male	0.00000	-4.76786	Male
Rn45s										0.00896	2.53498	Female						
Eif1										0.00000	-2.61084	Male						
Lsm5										0.00000	-3.74887	Male	0.01960	1.29019	Female			
Chchd3										0.00242	2.91667	Female						
Ifngr1										0.02033	-4.17162	Male	0.01646	3.86840	Female			
Crym										0.00000	0.87848	Female						
Lrrc23				0.00000	-3.18166	Male							0.04703	1.12625	Female	0.01130	4.31276	Female
Ifit1bl2													0.01508	2.53099	Female			
Filip1l																0.00753	-4.87449	Male
Myo1d										0.01053	3.60706	Female						
Irgm1							0.04497	3.10797	Female	0.00000	-5.72966	Male						
Acoxl	0.00007	1.61879	Female															
Gmds				0.00000	0.92976	Female												
Pcdh19										0.00025	-0.61378	Male	0.00000	3.60998	Female			
Myo3b										0.00000	-3.50508	Male						
Prom1										0.00000	1.12579	Female						
Pfdn1										0.04060	-1.58303	Male						
Bik										0.00056	-1.10189	Male						
Ddx60																0.00000	-0.60318	Male
Rpl41										0.00000	-4.55243	Male	0.04893	1.11076	Female			
Nsun3							0.00000	1.75009	Female	0.00204	-0.61155	Male						
Daxx										0.00840	2.89737	Female						
Crls1										0.03987	-2.00547	Male						
Plekhg4													0.00667	0.92392	Female	0.00559	2.35886	Female
Ostc										0.04776	1.20685	Female						
Serinc5																0.00939	3.92812	Female
Tom1l1																0.02374	-2.97980	Male
Camk4										0.02645	-2.13104	Male						
Cpxm1	0.00000	-0.67725	Male															
Eif2a										0.02953	1.31551	Female				0.01009	1.22215	Female
Alas2				0.00280	-1.30672	Male												
Mib2										0.00441	4.01289	Female						
Nedd8										0.00570	-1.80013	Male						

Gng5										0.00000	-3.97865	Male	0.00919	1.82156	Female			
Asb1	0.00000	0.85456	Female							0.00005	-1.92925	Male						
Phf8							0.03684	2.50551	Female									
Arpp19										0.00000	-3.53401	Male	0.00283	1.57634	Female			
Smim13																0.01937	-1.67468	Male
Slfn2													0.00079	-1.63657	Male	0.00000	3.48426	Female
Cox6a1										0.00005	-3.85348	Male						
Sumo1																0.01529	-0.99395	Male
Nek4										0.00005	-4.37706	Male						
BC024386										0.00000	-6.16394	Male						
Zfp943																0.00753	3.96407	Female
Dnah9				0.00000	3.11246	Female							0.00000	-1.87255	Male			
Smagp										0.00999	-3.96964	Male						
Dand5										0.00000	-3.95391	Male						
Nup54													0.00964	-1.52845	Male			
Adck2				0.00000	1.79559	Female												
Adam8													0.00000	1.61559	Female	0.00000	3.23140	Female
Ndufs6										0.00000	-3.90509	Male	0.01737	1.61582	Female			
Tbca										0.00000	-4.05557	Male						
AY761184										0.04952	-4.23206	Male						
Ormdl2										0.00002	3.05181	Female						
AI662270							0.00333	4.62375	Female									
Morf4l1.ps1										0.01785	1.47026	Female						
Clcnkb				0.00006	-1.64596	Male	0.00000	0.91394	Female									
Snx17										0.02328	2.51115	Female						
U2af2										0.00000	-5.24699	Male	0.00000	2.21427	Female			
Fbf1				0.04835	1.84845	Female												
Dalrd3										0.03457	2.35275	Female						
Itga11	0.00000	-2.61824	Male															
Nckap5										0.04429	-0.97433	Male						
Pop4										0.03145	1.38495	Female						
Gbp9																0.01438	-4.17097	Male
Gad2										0.00350	-2.22018	Male						
N4bp2																0.01339	3.47311	Female
Rpl27										0.00007	-2.64158	Male						
Fam151a										0.00000	7.56759	Female						
Gm14139							0.02606	2.92039	Female									
Bgn										0.00017	-1.93846	Male				0.00000	-5.28439	Male
Ccne1																0.02590	-1.54120	Male
Ctxn1													0.00018	5.18606	Female			
Ube2f	0.03154	3.32821	Female															
Kri1										0.00000	5.66460	Female						
Mir3091										0.02009	-4.28641	Male						
Nlrp5																0.00008	-5.05946	Male
B3galt6							0.00796	4.29375	Female							0.00007	-5.92067	Male
Spcs1										0.03334	-1.14858	Male						
Trpm6							0.00000	-3.17316	Male	0.00003	-2.17291	Male						
Fgfbp1							0.00000	1.57270	Female									
Il1rl1										0.00007	-4.84121	Male						
Kdelc1										0.00000	1.18602	Female						
Rhag										0.04735	-3.60648	Male						
Plp2										0.00226	2.11038	Female						
Mbd3l2													0.00005	-4.26991	Male			
Plxna2													0.00000	2.62955	Female			
Plcg1										0.00016	5.70096	Female						
Egr1																0.00000	7.56750	Female
Sp9																0.00069	3.25091	Female
Uggt2				0.00046	1.53046	Female												
Nudt16l1	0.02652	3.77261	Female															
Nfib													0.00000	2.10635	Female			
Nfil3													0.00222	4.45876	Female			
Cables1													0.04831	3.64524	Female			
Rpl37										0.00000	-5.44875	Male	0.01542	1.25609	Female			
Rom1										0.00000	1.63023	Female	0.00023	4.81442	Female	0.00181	4.45603	Female
Dennd4a																0.00000	4.11680	Female
Gm4832										0.00355	-3.05493	Male	0.04089	1.93737	Female			
Setdb2										0.04010	-4.54777	Male						
Obox5										0.03149	-3.83070	Male				0.00395	4.01718	Female
Dpysl2										0.00000	0.69507	Female						
Agpat4										0.00473	-4.84132	Male						

Dio1																0.00300	-4.77379	Male
Kif5a				0.02695	-0.91936	Male												
Sp100										0.01770	-2.46404	Male						
Smim4										0.00590	-3.62614	Male						
Poc1b																0.03714	3.35807	Female
S100a16	0.00000	-2.05502	Male															
Gtsf1																0.00050	4.50008	Female
Gm12504										0.00556	1.33103	Female						
Prkd3													0.00000	-3.80720	Male	0.01543	4.25520	Female
Ly6f				0.00000	-2.66461	Male	0.00000	-1.14648	Male									
Lcn2																0.00000	2.25304	Female
Setx																0.01103	1.35533	Female
Tmem14a							0.00000	-0.58195	Male									
Clta										0.00001	-3.24426	Male						
Lrrc46																0.00791	4.15817	Female
Pdlim3										0.03265	4.20419	Female	0.00078	5.12270	Female			
Rpl37rt										0.00000	-5.25391	Male	0.01256	1.30046	Female			
Pigx										0.00005	-3.94966	Male						
Tc2n										0.00000	-5.47817	Male						
Nutf2										0.00231	4.46561	Female						
Snx25				0.00000	1.30270	Female												
Cnr2										0.02566	-1.99460	Male						
Vps13c										0.00571	3.93656	Female						
Hmgb1										0.04837	1.13206	Female						
Appbp2										0.03063	2.13374	Female						
Snrpe										0.00000	-4.83987	Male	0.00581	1.25952	Female			
Fth1										0.00370	-2.75218	Male						
Arhgap18																0.00000	-4.16451	Male
Ncam1																0.04573	3.89964	Female
Laptm5	0.01083	1.42788	Female															
Gm6402										0.00000	-4.05335	Male	0.01524	1.12476	Female			
Adcy3													0.03116	-4.39448	Male			
H2.T23										0.00000	-4.95971	Male	0.00246	1.65370	Female			
Syt7																0.00000	3.11809	Female
B4galt7										0.00017	-2.92967	Male						
Fam98b										0.00000	-4.43895	Male						
Prpf3										0.03767	1.92675	Female						
Hif1a				0.01556	1.59140	Female	0.03112	-3.94087	Male									
Rtn1	0.00000	0.94029	Female										0.00000	2.21129	Female			
Mrps35				0.00004	4.53528	Female												
Tat	0.04473	-3.06229	Male															
Rnf165							0.04765	4.06354	Female									
Adrb3							0.00000	2.83908	Female									
Ptma										0.00942	1.24551	Female						
Asap3																0.00000	-4.22093	Male
Cd209c													0.01684	2.06353	Female			
Xk													0.01892	3.79770	Female			
Slc9b2							0.00513	-0.68794	Male				0.00061	1.07324	Female			
Setmar				0.00000	2.45953	Female							0.00000	5.71632	Female			
Aco2										0.01189	1.63238	Female						
Zfp692							0.02064	2.90142	Female									
Asf1b										0.03219	1.77723	Female						
Necab2													0.00014	4.99460	Female			
Hspa1b										0.04890	-3.39838	Male						
Zp3																0.00461	3.07838	Female
LOC100505025										0.00008	-4.97330	Male						
Arhgap5							0.02631	3.18232	Female									
Tspan9																0.00121	4.49346	Female
Vdac2										0.00740	1.35046	Female						
Zfp865																0.03020	3.43489	Female
Mgst2							0.00000	-2.99823	Male									
Tmem232							0.00000	1.91832	Female				0.00000	-0.98662	Male			
Serpinb1c				0.00000	2.53756	Female												
Pkd2l1							0.00004	-0.68053	Male									
Spcs3	0.00324	4.26602	Female	0.00739	3.35770	Female												
Zfp207										0.00245	1.68633	Female						
Kpnb1										0.00644	1.25004	Female						
Phactr4													0.00806	-3.42686	Male			
Chtf8										0.00410	2.41795	Female						
Cbx7										0.01294	2.18293	Female						

Nrip2													0.00000	-1.60863	Male			
Tpd52l2																0.01172	-1.18099	Male
Rab8a										0.04902	1.69224	Female						
Stard5										0.00000	-3.29843	Male						
Taf10										0.00000	-5.54993	Male						
Dpp10				0.01235	2.79976	Female												
Yod1							0.00000	2.87290	Female	0.01002	-1.33424	Male						
Ep300										0.00002	-5.90884	Male						
Pi4k2b																0.00225	-2.18208	Male
C1s2													0.03515	4.26202	Female			
Phpt1										0.03640	-2.37789	Male						
Armc9										0.03326	2.86824	Female						
Mfap2				0.00000	1.23841	Female				0.00001	-2.98367	Male						
Atg9a																0.00652	-1.73504	Male
Zfp646										0.00002	5.83143	Female						
Zfp612																0.02738	3.52187	Female
Fam214b																0.00001	-3.06670	Male
Ndufa11										0.01727	-2.10968	Male						
Ss18										0.04902	1.07618	Female						
Thra													0.00000	6.36616	Female			
Csf3r										0.03682	2.18995	Female						
Foxl1										0.00003	-4.05525	Male						
Efr3b										0.04389	-3.03019	Male						
Sema4a													0.00354	-4.75603	Male			
Slc16a13							0.04296	2.93240	Female									
Tmed6																0.00292	-4.49714	Male
Rpl27a										0.00643	-2.39215	Male						
Tfap2e													0.04343	-4.29755	Male			
Plxdc2										0.00036	-0.92276	Male						
Zfp799										0.00002	-2.66262	Male	0.03040	1.20272	Female			
Calr													0.01580	1.07054	Female			
Srek1ip1				0.01796	3.52203	Female				0.00015	4.82218	Female						
LOC106029237																0.00044	-5.86203	Male
Trim62										0.01561	-0.90936	Male						
P2ry4										0.00134	-3.92231	Male	0.00464	2.59848	Female			
Fxn										0.00780	-3.80592	Male						
Ppp2r2b													0.00206	4.31115	Female	0.00005	-6.15749	Male
Fut10																0.00000	1.22345	Female
Pctp													0.03851	3.00000	Female			
Ifrd2										0.03707	2.16512	Female						
Fthl17f				0.01451	3.83223	Female	0.00000	5.63787	Female	0.00026	5.12400	Female	0.01249	3.06270	Female			
Kit													0.01125	-4.04951	Male			
Utp6																0.00013	-2.61978	Male
Myoz3										0.00299	-3.67081	Male						
Rsl1d1										0.01061	1.02298	Female						
Dnah2										0.04733	3.43649	Female						
Nop56										0.00987	0.88698	Female						
Ogfod1																0.01859	2.20129	Female
Grhl1				0.00000	2.07708	Female												
Zfp202										0.00011	-4.62294	Male	0.00004	3.46806	Female			
Rnf113a1										0.02570	3.27924	Female						
Zfp106													0.04965	-0.84795	Male			
Trp73				0.00900	-1.60223	Male												
Nsf										0.02468	1.89884	Female						
Rnf185																0.04406	-1.60240	Male
Ndufa8	0.00707	4.11402	Female															
Ndufb4										0.00000	-3.77180	Male	0.00209	1.92846	Female			
Nxpe4										0.03708	-0.86158	Male						
Guf1																0.03813	-3.54832	Male
Uba1										0.00000	2.06399	Female						
Pou6f1													0.02856	2.46661	Female			
Magea5							0.02476	4.30822	Female	0.00000	-1.53256	Male						
D5Ertd579e				0.03905	2.75936	Female												
Tmem81										0.04120	2.99181	Female						
Ugt2b36										0.00406	-6.28266	Male						
Il1r1										0.01649	-1.15239	Male						
Alg3				0.00732	2.94335	Female												
Nr2f2													0.03627	3.32955	Female			
Exosc6										0.00013	-4.63509	Male						
Etaa1																0.00028	2.91708	Female

Eif4g1										0.00432	3.12923	Female						
Gngt1										0.00001	-4.47529	Male						
Fam65b										0.00022	-3.82409	Male	0.01027	2.20226	Female			
Cbx3										0.00002	-2.69131	Male						
Cox6b1										0.00000	-4.60905	Male	0.00106	1.52278	Female			
Lrrc1													0.00001	0.88862	Female			
Zfp82										0.04923	3.02539	Female						
Actr3										0.00100	1.62849	Female						
Ubb										0.00010	1.85566	Female						
Rab42										0.00111	0.73413	Female						
Acads										0.01187	2.81366	Female						
Cnot9										0.00033	1.93438	Female						
Ick										0.03109	3.02813	Female	0.03053	3.23934	Female			
Cd8b1										0.01658	-1.76117	Male						
Lix1										0.00010	-2.73939	Male	0.04831	1.27204	Female			
Ndufb6										0.00012	-3.10313	Male						
Hebp1													0.04102	4.11028	Female			
Actb										0.00381	-1.86233	Male	0.00000	1.97040	Female			
Abca7				0.00509	-1.42962	Male				0.00000	2.75148	Female						
Ggt5							0.00201	-1.32654	Male							0.00000	-4.40083	Male
Cdc25a																0.02971	-1.62168	Male
Zfp39										0.00000	1.06973	Female						
Itpka	0.00000	2.02428	Female															
Tm9sf1																0.00069	-2.90560	Male
Cfap53										0.00410	-1.46497	Male				0.00000	3.86357	Female
Csnk1e										0.02482	1.47020	Female						
Syce3										0.00909	-5.38123	Male						
Tufm										0.00086	1.93177	Female						
Camsap1																0.01375	-1.47438	Male
Pcnx3										0.00001	5.90875	Female						
Phf7							0.04604	-4.38249	Male							0.01375	4.10327	Female
Bysl										0.00126	2.15551	Female						
Capn15										0.00025	5.48475	Female						
Tor3a																0.00000	1.88766	Female
Thbs1													0.00000	2.14951	Female	0.00001	1.31556	Female
Xlr5b													0.00089	-3.58208	Male			
Maneal													0.00213	4.54662	Female			
Mmachc							0.02647	2.23532	Female									
Mt2										0.00000	-6.58727	Male						
Slc31a2										0.04814	-4.34949	Male						
Cox6c										0.00000	-5.25416	Male	0.00000	2.29033	Female			
T2										0.00001	-5.29979	Male						
Snx21																0.03255	4.69744	Female
Uqcr10										0.00000	-4.46817	Male	0.00000	2.38102	Female			
Fbxo46													0.00284	4.09081	Female			
Cdh11										0.04837	2.11740	Female				0.03018	-1.80093	Male
Spef1										0.00029	-2.01615	Male						
Plekhf1				0.00060	2.27351	Female												
Stim1										0.04688	2.90391	Female						
Rbm11										0.00000	-4.41398	Male						
Yif1b										0.00194	3.88304	Female						
Akap10													0.00550	-4.24871	Male	0.00384	-4.23796	Male
Ctf2										0.01638	2.04209	Female				0.00123	1.03566	Female
Rpl10a										0.04498	-1.43898	Male						
Imp3										0.00075	-3.50782	Male						
Nrg4				0.00001	1.65025	Female												
Kansl1l							0.02287	3.63318	Female									
Pgp										0.00709	-3.21443	Male						
Clu																0.00027	6.46424	Female
Ssr4	0.00315	2.43496	Female															
Htra2										0.00008	3.55405	Female				0.02200	-1.89051	Male
Cass4										0.00007	0.67570	Female						
Lif										0.00297	4.46161	Female						
Supt20										0.00088	3.25494	Female						
Rps13										0.00000	-3.51229	Male						
Tnnc2																0.04792	-3.98745	Male
Homez										0.03585	-1.77157	Male						
B3glct	0.04177	3.24623	Female															
Ccdc60	0.00166	-1.94581	Male	0.03318	-0.70926	Male												
Uck1				0.00493	3.38780	Female												

Tmem136										0.00000	-0.93329	Male						
Rps4x							0.00139	0.92507	Female	0.03633	0.78822	Female	0.00020	0.85620	Female			
Zscan22										0.00582	3.95260	Female	0.01464	4.06473	Female			
Tm4sf5				0.00000	1.17357	Female							0.02069	4.28880	Female			
Fthl17d				0.00181	4.93143	Female	0.00000	5.63447	Female	0.00211	4.63801	Female	0.00001	4.44212	Female			
Nptx1																0.00810	5.19007	Female
Prmt5										0.00454	1.55307	Female						
Ralgps1																0.01430	3.26828	Female
Pif1				0.01346	2.16705	Female												
Csrnp3							0.00013	4.54791	Female	0.00340	-4.12152	Male						
D17H6S53E										0.00000	-4.86311	Male						
Creb3l3													0.00000	-2.86202	Male	0.01483	3.31340	Female
Azin2										0.00038	1.63980	Female	0.00000	3.61945	Female			
Pcbd2										0.04660	-4.02392	Male						
Zfp422													0.00070	2.63610	Female			
Tmed7				0.00000	5.16549	Female												
Pla2g4e																0.00001	-5.68444	Male
Ccdc124										0.01219	3.11202	Female						
Nes										0.02257	-1.24834	Male						
Rbpms										0.00346	-1.92202	Male						
Hyal2							0.00634	-4.89772	Male									
Disp1																0.00000	1.10951	Female
Mpdz																0.00042	4.71556	Female
Tnfrsf11a																0.02614	-2.64309	Male
Fbxl6										0.00000	5.49555	Female						
Pld1													0.00000	-3.30411	Male			
Pla2g4c										0.03336	-4.43069	Male	0.00073	4.77047	Female			
Il17rb				0.00115	-0.59662	Male												
Adgra2																0.04062	-2.70386	Male
Dusp3																0.00034	-3.85993	Male
Itpripl1										0.00000	2.10512	Female						
D6Ertd527e													0.00000	-5.40200	Male			
Hyal3										0.01068	-2.40999	Male						
Fn1										0.03272	3.19702	Female						
Slco5a1													0.00000	0.98784	Female			
Skida1													0.02149	4.40289	Female			
Erh										0.00180	-1.29269	Male						
Wdfy2										0.04087	2.46063	Female						
Fus										0.00000	-6.16046	Male						
Fam210a																0.01673	1.84945	Female
Birc2																0.03615	2.52207	Female
Tbc1d13																0.00453	2.06258	Female
Daglb													0.03894	-3.70589	Male	0.00000	3.77486	Female
Gm8624										0.00000	-4.43874	Male	0.00043	1.53111	Female			
Pdha1							0.00201	1.16881	Female	0.00002	1.75351	Female						
Fbxw24																0.00012	-5.27778	Male
S1pr5							0.00000	4.21860	Female									
Rps21										0.00000	-5.44461	Male	0.00048	1.72274	Female			
Ttf1													0.00103	1.84875	Female			
Pik3r6				0.00000	-1.00483	Male				0.01337	4.40380	Female						
Otud5																0.02703	1.48341	Female
Slc6a9													0.00000	-3.27419	Male			
Selplg										0.00632	-1.97373	Male						
Gm9926										0.01062	-3.16195	Male						
Prex2										0.00435	-1.53342	Male						
Cnga3													0.00985	3.36609	Female			
Ptk6													0.02209	2.74664	Female	0.00683	3.41139	Female
Mrpl54										0.00002	-4.36985	Male						
Ndufa12										0.00014	-3.12484	Male						
Actg2				0.00035	1.15379	Female												
Trim43c																0.04915	-0.99092	Male
Shc1										0.01311	2.41574	Female						
Sft2d3													0.01127	5.24460	Female			
Tchh				0.00000	4.53483	Female												
Mospd4				0.00000	-0.74637	Male	0.00000	2.58552	Female									
Chga										0.00099	5.19588	Female						
Nanp										0.00000	1.75562	Female						
Tcl1b2																0.00130	-6.23177	Male
Saxo1				0.01080	2.50166	Female				0.04981	1.69201	Female						
Kank4										0.02452	-3.48482	Male						

Slc38a6																0.02122	-2.33147	Male
Trim43b																0.03865	-1.13370	Male
BC055324																0.00272	2.18347	Female
Kctd2										0.00658	3.44954	Female						
Klhl7										0.04552	-1.82585	Male						
Ttc39a				0.00005	0.81345	Female												
Teddm1b										0.00000	-5.44439	Male	0.00078	1.75281	Female			
Sgcb													0.00000	-4.31118	Male	0.00006	-0.63811	Male
Upk3b				0.01785	-1.90488	Male												
Dennd1c																0.04334	-3.75051	Male
Oasl2																0.00275	4.58033	Female
Sox2				0.00000	-1.18891	Male												
Gm11127										0.00703	-1.88877	Male	0.03851	1.19436	Female			
Exosc5										0.01611	3.09397	Female						
Slc4a1ap																0.00002	-2.93174	Male
Mycn							0.03588	-5.41718	Male									
Cyba	0.00000	1.79552	Female													0.00135	-4.71031	Male
Thbs3													0.00000	-3.43719	Male			
Myo5b													0.04617	2.95141	Female			
Tfpi				0.00000	2.38298	Female												
Klhl8													0.00311	3.79520	Female	0.00096	4.11199	Female
Cryba1	0.00458	-1.73712	Male															
Cd74																0.00000	-4.09045	Male
Lcn9	0.00981	1.05594	Female															
D1Pas1																0.00000	-1.07619	Male
Umodl1																0.04513	-4.25919	Male
Nars										0.00209	1.56485	Female						
Zfp974													0.00222	4.43554	Female			
Bhmt2																0.00542	-2.17363	Male
Gm805				0.00021	-0.86375	Male	0.00000	-0.64439	Male									
Mirt1										0.00018	-2.56939	Male						
Tbxa2r													0.00797	-4.27647	Male			
Bcorl1										0.04036	3.36114	Female						
Mia2										0.00134	-0.96927	Male						
Gpd1l																0.00031	-1.69959	Male
B3gnt6							0.00008	-1.82751	Male	0.00001	2.36807	Female						
Osbpl2																0.00422	-2.28890	Male
Rnd2										0.00510	3.62446	Female						
Hdac10				0.00001	0.58146	Female												
Hist1h1a				0.00000	1.11356	Female							0.00000	2.45704	Female			
Cpe				0.00009	-0.84862	Male							0.01816	3.90038	Female			
Hmgcll1										0.00001	-2.56787	Male						
Tmem2							0.00031	5.06938	Female							0.00002	-6.06453	Male
Spag6l																0.00000	1.82934	Female
Mrps34										0.01230	-3.23207	Male						
Inafm1																0.00747	-4.97469	Male
Insm1				0.03069	4.43684	Female												
Kdm4a										0.00001	-5.73509	Male						
Prr11										0.02209	-2.64123	Male						
Rps6ka6																0.00000	3.00531	Female
Zfp873																0.00017	4.80272	Female
Ovol2																0.00247	-3.38714	Male
Gpha2																0.01009	4.37518	Female
Zrsr2													0.00691	2.10437	Female			
Mrpl47																0.01478	-1.06292	Male
Trpm4							0.00749	-5.04123	Male									
BC021614																0.02440	3.58812	Female
Ccdc136										0.00760	-1.29877	Male				0.00000	-2.46636	Male
Lrrc36							0.02720	-2.53699	Male									
Dgcr8										0.03575	2.30427	Female						
Sil1																0.00465	-3.37091	Male
Gng7	0.00440	0.98677	Female															
Mapk8ip3				0.00903	1.55477	Female												
Lig3										0.00418	2.05335	Female						
Sepn1				0.00343	-1.47285	Male												
Abo										0.00032	0.96798	Female				0.00000	-2.90140	Male
Eif3i										0.00000	1.76084	Female						
Rpl18a										0.00023	-2.72421	Male						
Megf8																0.02747	3.53112	Female
Zfp991																0.02818	-1.91711	Male

Dpep1													0.00911	3.61932	Female	0.00000	3.74154	Female
Gsdmc2																0.00013	5.10245	Female
Mep1b																0.00021	4.12151	Female
Rdh9													0.03798	3.18664	Female			
Gch1										0.00808	-2.39898	Male						
Rps28										0.00000	-5.67313	Male	0.00000	2.28773	Female			
Klf6										0.00175	4.72018	Female						
Sp3os										0.00000	-4.88784	Male						
Morn1																0.02284	-4.23910	Male
Nrap				0.01026	2.64181	Female										0.03725	5.60874	Female
Mtus1																0.00311	-2.19127	Male
Dgkz										0.00056	3.13342	Female						
Oosp1													0.00515	-3.78362	Male	0.01135	-3.71347	Male
Hsd3b1	0.02611	-0.95814	Male										0.00000	1.66209	Female			
Trim66													0.00000	-5.30157	Male	0.00000	2.04365	Female
Krt18				0.01084	-6.09707	Male												
Asxl2																0.00014	-2.73610	Male
Fam174b													0.00003	1.08558	Female			
Snord100				0.00869	3.78730	Female												
AU015228													0.03040	-3.14294	Male			
Drd3										0.00000	1.48695	Female						
Htatsf1							0.02866	1.86099	Female									
Ube2n																0.04792	-0.92877	Male
Ooep										0.02224	-2.64419	Male						
Adprm				0.01817	1.87456	Female	0.01571	3.45862	Female									
Car8										0.00016	-4.42070	Male						
Arl6										0.02188	3.07370	Female						
Mtx2										0.02762	-3.05102	Male						
Ptcra										0.00000	-4.95646	Male	0.01731	1.65779	Female			
Dtx3l										0.00173	-3.78448	Male						
Arl13b																0.03731	-3.24980	Male
Nphp1				0.00143	1.32581	Female												
Wdr73																0.04930	1.46476	Female
Nxpe3										0.01893	-5.01212	Male						
Golga4										0.00194	3.15692	Female						
Slc39a11																0.00191	4.59391	Female
Rmdn2							0.00000	3.47719	Female									
Erich1																0.00606	2.90555	Female
Capn1										0.00383	-3.37766	Male						
Ccdc78							0.02297	-1.97729	Male	0.00396	-2.73528	Male	0.00318	2.10860	Female			
Aurka										0.01348	1.73466	Female						
Nectin2				0.00362	1.98298	Female												
Chpf2										0.02363	4.01443	Female						
Fgl1																0.00014	1.81939	Female
Mcrs1										0.01702	1.93660	Female						
Ccr8							0.00000	4.17801	Female									
Tfr2													0.00000	-2.90021	Male			
S100a14										0.00000	-4.05231	Male						
Arfgap3										0.00004	-2.25493	Male						
Rxrg																0.00821	3.27519	Female
Hnf1b				0.00001	-2.76609	Male				0.00000	3.11720	Female						
Tcea1													0.00141	-1.17921	Male			
Ube2l6							0.03914	-1.57943	Male							0.00040	2.10602	Female
Foxc2																0.00000	-5.58371	Male
Hmgcs1													0.00618	1.67314	Female			
Pigp										0.00000	-3.73791	Male	0.00112	1.35497	Female			
Fmn1										0.00000	0.89269	Female						
Ube2j2										0.00000	-2.63959	Male						
Krtcap2										0.00945	-2.46617	Male						
Psmd11										0.00227	1.39397	Female						
Pigh																0.00109	5.03119	Female
Terf2ip																0.00035	3.16757	Female
Adprhl1	0.02693	-0.64315	Male				0.00000	1.26209	Female									
Gstm1	0.00000	-0.94518	Male							0.00000	-1.55676	Male						
Amot							0.00029	3.21206	Female									
Tmem47																0.03020	3.43826	Female
Pigz				0.01419	-1.53306	Male												
Neb							0.01615	-5.04453	Male									
Scaper										0.03400	3.49928	Female						
Ppm1g										0.04196	1.30266	Female						

Parp8													0.01878	-1.50349	Male			
Strn4				0.04107	2.33245	Female												
C5ar2										0.00014	-4.19597	Male						
Wnt7b										0.02709	4.18252	Female						
Tmppe										0.03766	-1.72024	Male						
Vprbp													0.00052	-2.55745	Male			
Hdhd3	0.00045	1.28159	Female	0.00000	2.25557	Female	0.00000	-3.56407	Male	0.00445	-1.34922	Male						
Hibch										0.01795	2.49194	Female						
Rtn4r																0.00058	-5.22263	Male
Ahnak										0.00411	-4.43929	Male	0.00062	3.69439	Female			
Kpna4										0.01441	-1.92514	Male						
Ndst1													0.01487	2.87144	Female			
Rnasel																0.04546	3.11045	Female
Fam35a										0.03111	2.82144	Female						
Fam110c																0.00076	1.12260	Female
Htr5b																0.00000	3.04466	Female
Akna																0.00000	3.49230	Female
Dpys				0.00144	1.13995	Female												
Kcp	0.03593	-1.05664	Male				0.00002	-1.76994	Male									
Edn2										0.02465	2.44955	Female						
Tcf24				0.00222	-2.13248	Male												
Elmod2																0.01350	-3.08476	Male
Diexf										0.02682	2.26249	Female						
Arpin				0.00001	0.99013	Female							0.02822	3.93599	Female			
Med30										0.00027	-6.09736	Male						
Syt9										0.02385	3.72357	Female						
Tomm20										0.00000	-3.26891	Male						
Zfp523										0.00000	3.32838	Female	0.00000	-1.39169	Male			
Mpp1													0.00052	1.51894	Female			
Lnpep							0.04416	4.37514	Female									
Adtrp																0.00274	-2.49132	Male
Pggt1b	0.00000	1.49106	Female															
Catsper2																0.01368	-4.65355	Male
Impdh2													0.02406	-0.88626	Male			
Lzts3										0.03753	-3.62920	Male						
Ccnt1																0.04932	-2.20948	Male
Fhl3										0.00165	-5.28173	Male						
Slc25a26																0.04387	-2.39084	Male
Arpc4										0.00120	3.34100	Female						
Idi2										0.00401	4.55564	Female						
Pnisr										0.00247	2.86422	Female						
Lrrc3										0.00003	-3.13651	Male						
Cpne9																0.00946	3.58691	Female
Ccl25	0.00000	1.08097	Female															
Cfc1							0.00000	-2.64789	Male									
Shfm1										0.00000	-4.44805	Male	0.00000	2.37109	Female			
Aif1l										0.02814	-4.74745	Male						
Erdr1										0.01912	-2.67785	Male						
Ipo5										0.04960	0.81033	Female						
Elovl3	0.04770	1.56531	Female															
Timm10										0.00840	-1.89805	Male						
Zfp28	0.00000	-1.22371	Male															
Rpl4										0.03218	0.80285	Female						
Abhd6																0.00051	-3.28655	Male
Trps1										0.00022	-2.40182	Male	0.00000	0.88037	Female			
D17Ertd648e																0.00958	-1.47275	Male
Mfsd4a							0.04090	2.36846	Female	0.00274	-3.19045	Male						
Dpysl4				0.00071	-2.67254	Male												
Net1																0.04194	-2.10813	Male
Msrb1										0.00003	-3.85576	Male						
Cfap43							0.00000	5.59705	Female									
Prrc2b				0.01041	3.09745	Female												
Adora1										0.00001	-1.10645	Male						
Xist				0.00003	5.13387	Female	0.00000	7.59985	Female	0.00000	5.55145	Female	0.00000	8.04782	Female	0.00000	7.38646	Female
Actn2																0.03198	-2.07619	Male
Sptssa										0.01890	1.42715	Female						
Psg16										0.03207	1.53936	Female						
Lyrm9										0.01895	-3.20321	Male						
Dpy19l4																0.00358	-4.90775	Male
Xpot													0.00316	-1.21172	Male			

Brip1os										0.00084	-2.61769	Male						
Bex1										0.02272	4.20701	Female						
Txn1										0.00001	-3.11379	Male	0.01140	1.46842	Female			
Myoz1										0.01068	2.27977	Female						
Prss41													0.00000	4.02721	Female			
Igf1r																0.00000	2.82480	Female
Snai2							0.00977	-3.49010	Male									
Ak4										0.00033	2.06284	Female						
Kcnn4				0.00002	-2.34127	Male	0.01804	-4.80920	Male	0.00000	3.53432	Female						
Ccdc181																0.01178	-5.27784	Male
Apoe										0.01004	-3.76743	Male				0.00152	3.77981	Female
Scml4										0.04869	-3.04073	Male						
Prl3b1																0.00723	-4.25803	Male
Pomgnt2				0.00340	-0.80649	Male	0.00000	1.30704	Female									
Ap3m2							0.00000	3.57698	Female				0.00000	3.77661	Female			
S100pbp													0.00550	3.18554	Female			
Hspe1										0.00000	-3.01664	Male	0.00000	1.95775	Female			
Hpgds										0.00002	-4.13297	Male						
Thg1l				0.02915	3.02308	Female												
Rab4a				0.00000	-0.76277	Male												
Fam168a																0.00034	2.88112	Female
Fam199x										0.00262	-6.12352	Male				0.02453	-4.12826	Male
Fcgbp																0.00753	-5.13083	Male
Cblc																0.01410	4.84727	Female
Dcun1d5										0.02440	-1.82108	Male						
Abcc3																0.00015	5.09256	Female
Gm6525							0.00598	1.64061	Female	0.00000	-4.77553	Male	0.00020	1.66824	Female			
Rpl13										0.00000	-4.90932	Male						
Slirp										0.00000	-5.42636	Male	0.00034	1.84790	Female			
Dio3							0.00000	0.80920	Female	0.00020	-2.15744	Male				0.00000	2.75710	Female
Psmd1										0.00945	1.41184	Female						
Rbm20				0.00000	0.99918	Female												
Clptm1										0.01947	2.76364	Female						
Aox4	0.00438	0.80260	Female													0.00123	-0.58628	Male
Usf1										0.00006	4.78162	Female						
Cpb2							0.00000	-1.32918	Male									
Ubl5										0.00000	-5.16160	Male						
Rps14										0.00001	-2.78685	Male	0.00989	1.31536	Female			
Zbtb25													0.01565	4.51637	Female			
Rcan3																0.00251	3.77074	Female
Apoa1										0.00056	6.22215	Female						
Btbd3										0.00000	-3.04588	Male				0.00791	4.61751	Female
Elac2										0.03125	2.28392	Female						
Sys1										0.03143	-2.29664	Male						
Mob3b										0.00000	-5.21187	Male						
Prdx3										0.00000	3.50572	Female						
Snrpb2							0.00435	1.23801	Female									
Fmc1													0.01721	2.49675	Female			
Tnfrsf25				0.04154	-0.68147	Male				0.00005	2.58205	Female				0.00000	-4.09122	Male
Sox2ot										0.03539	-2.79234	Male						
Inpp4a																0.03731	-4.08173	Male
Cyld										0.00000	-3.71244	Male	0.01542	1.42300	Female			
Smyd1				0.00000	0.95189	Female												
Zdhhc16										0.02417	2.97399	Female						
AF067061																0.00036	-4.14294	Male
Tnfaip6										0.01624	-3.18043	Male						
Wtap																0.01293	-1.41479	Male
Cald1													0.00000	3.56662	Female			
Fam171a1										0.00000	1.20480	Female						
Impg2													0.01374	-4.73626	Male			
Rpl35a										0.00000	-4.55379	Male	0.00012	1.58643	Female			
Lrmp																0.00002	0.91979	Female
Rin3	0.00000	-0.97951	Male															
Ube2l3										0.00631	-1.17988	Male						
Otud3													0.03311	3.19569	Female			
Ggt6																0.00612	3.98016	Female
Mei4										0.00102	-3.89608	Male						
Ranbp3l										0.00220	-2.62125	Male	0.03851	1.44260	Female			
Fhl1				0.01587	-0.91472	Male							0.00000	-1.65848	Male	0.00000	1.66972	Female
Rgs11																0.04035	-2.60654	Male

Fcgr1				0.03492	1.87472	Female										0.00011	-0.75088	Male
Bccip										0.00000	5.36605	Female						
Fdx1										0.01940	-2.66533	Male						
Abcc9										0.00001	-3.16429	Male						
Pdia3										0.02060	-2.21762	Male						
Myh15							0.00723	1.06328	Female	0.03365	1.42980	Female						
Egf																0.02824	3.52891	Female
Tert										0.00000	3.41413	Female						
Aim2							0.03477	3.85219	Female									
Oat										0.00081	-5.50312	Male						
Saal1	0.03830	3.26349	Female															
Slc50a1										0.00144	-0.94723	Male						
Erich5										0.00000	-5.30243	Male						
Hcfc2										0.00030	-1.95403	Male						
Elk4																0.00217	4.05393	Female
Tnfaip8l2																0.02887	3.00388	Female
Akap5										0.00110	-4.99053	Male				0.02200	3.31185	Female
Ndufa3										0.00000	-4.47148	Male						
Pax6													0.01038	3.57854	Female			
Atp2a3				0.00801	-1.71477	Male	0.00000	-1.09525	Male									
Plscr1	0.00000	-1.82470	Male															
Slc30a3				0.00009	-0.68383	Male												
Dok1	0.00000	1.50949	Female				0.00064	4.03773	Female									
Eif3m										0.00176	1.13223	Female						
Inf2	0.00000	1.18635	Female															
Ripply3										0.00000	-5.00587	Male	0.02612	1.55219	Female			
Slc43a2										0.01589	-3.86036	Male						
Slc7a2										0.00006	-3.49606	Male						
Ciita													0.00000	-4.40902	Male			
Idi1							0.00513	3.21856	Female									
Ids				0.03951	3.15070	Female												
Chchd5																0.00016	-2.22975	Male
Trp53i11							0.01012	3.90832	Female									
Astl													0.00674	4.01295	Female			
Nupl2																0.00000	4.53890	Female
Sdf2l1																0.02818	3.71992	Female
Ccdc85b										0.00001	-5.33963	Male						
Tmem64																0.01716	-2.20738	Male
X3.Sep																0.00075	3.81940	Female
Mrpl52										0.03397	-2.60380	Male						
Ttll4										0.02732	2.90358	Female						
Mprip										0.04052	1.39745	Female						
Snhg15										0.02511	-2.52151	Male						
Gm6654										0.00000	-3.98004	Male	0.01711	1.38931	Female			
Calm2										0.00324	1.43769	Female						
Tbrg3										0.02012	-2.64703	Male						
Fanca										0.04981	3.04557	Female						
Actn1										0.00051	-3.83608	Male						
Gtf3a										0.02619	3.78463	Female						
Fam193a										0.00091	4.21681	Female						
Eftud2										0.01592	1.65832	Female						
Kcnj2										0.00185	-5.21077	Male						
Rwdd1										0.00152	-2.32830	Male						
Gm2560				0.03876	0.90465	Female							0.00000	0.74513	Female	0.00000	-3.48719	Male
Ppp1ca										0.01162	2.00843	Female						
Psg29										0.00173	-1.93422	Male	0.00985	1.29689	Female			
Hnrnpf										0.00281	1.28900	Female						
Piwil1																0.00491	-4.99219	Male
Apobr										0.00000	2.39186	Female						
Gm10012							0.00760	1.70295	Female	0.00000	-6.07376	Male	0.00000	2.17363	Female			
Etv1										0.00241	-1.58024	Male						
Naip5										0.00001	-3.33290	Male						
Wdr77										0.00473	1.21446	Female						
Wbscr17				0.00434	1.99249	Female												
Tm2d2										0.01554	3.06236	Female						
Ankrd11										0.00000	5.07561	Female						
Znrd1										0.00704	-1.80642	Male						
Dclre1c										0.00036	-2.84711	Male						
Zcrb1										0.00019	-3.25478	Male						
Myeov2										0.00000	-6.91333	Male						

Lrrc58										0.00690	-1.71312	Male						
Aard							0.00000	1.81349	Female	0.00001	-1.47158	Male						
Cdc16																0.01179	-1.58518	Male
Rpl24										0.00000	-2.50288	Male	0.01636	1.04549	Female			
Tmem88				0.02039	-1.07682	Male				0.00000	1.71506	Female						
Mrpl23										0.00000	-4.08856	Male						
Cox5a										0.00000	-5.74158	Male						
Nfkb1										0.01320	-1.13040	Male	0.00000	2.20132	Female			
Lap3										0.03633	1.30383	Female						
Rpl22l1										0.00000	-4.89446	Male	0.00550	1.47828	Female			
Usp48										0.03238	0.89636	Female						
Psd2													0.00000	1.02155	Female			
Calml4										0.00454	-5.06879	Male						
Gpld1	0.00000	0.76780	Female							0.01728	3.35685	Female						
Mylk				0.00000	1.27292	Female				0.00556	-1.17482	Male						
Slc25a21										0.00537	4.68219	Female				0.00004	-5.66013	Male
Ciao1																0.01159	-1.33908	Male
Umad1				0.00000	1.31565	Female												
Slc26a8										0.00006	-0.80059	Male						
Sdk1				0.00049	-1.40301	Male												
Hypk										0.00000	-4.19164	Male	0.00057	1.64101	Female			
Irf5	0.00116	0.81327	Female													0.00600	-6.32617	Male
Atpif1										0.00000	-4.50595	Male	0.00014	1.82449	Female			
Sap30l										0.00004	-3.97696	Male						
Gm9895													0.01361	4.69221	Female			
Rab27b										0.02110	-3.17663	Male						
Myom1				0.04200	2.39793	Female												
Mdh1										0.00087	3.11411	Female						
Il5ra										0.00268	-3.60730	Male						
Rpp38							0.01424	2.17352	Female									
Pcnx2										0.00016	2.80433	Female				0.00000	1.79226	Female
Upk1a													0.00107	4.79215	Female			
Bre				0.00434	2.55340	Female												
Rnaseh2b	0.03878	3.01960	Female															
Slc22a18							0.00114	-2.23616	Male									
Stub1										0.00000	6.94877	Female						
Creb3l2				0.00006	-1.41669	Male				0.00015	-1.26425	Male						
Prune2										0.00034	-2.21509	Male	0.00000	1.32451	Female			
Utp14a										0.00004	2.86149	Female						
Fam45a																0.02638	-1.70212	Male
Srsf4										0.01828	4.73947	Female						
Fam65c										0.04530	5.05708	Female						
Flii										0.00207	2.96892	Female				0.02410	-1.91390	Male
Aldoart1										0.00000	3.27953	Female						
Rhbdl2										0.02402	3.10504	Female						
Rbbp7							0.02207	1.15091	Female	0.00000	2.26104	Female						
Akap8l										0.01732	3.65937	Female						
Ccny										0.02569	3.30630	Female						
Stra13										0.00003	-3.21547	Male	0.00249	1.83844	Female			
Fos										0.00000	-2.77262	Male						
Ldhc													0.03455	-4.32241	Male	0.04387	-4.14120	Male
Tmc5										0.00030	-2.47775	Male						
Anapc13										0.00000	-4.76707	Male	0.00049	2.09099	Female			
Copg2										0.00252	-5.60735	Male						
Sf3b3										0.04380	1.23515	Female						
Mfsd4b1	0.00000	-1.04633	Male															
Ticam1				0.02985	1.53237	Female												
Loxl2										0.00000	-6.07562	Male	0.00106	1.95095	Female			
Itgb5										0.02642	2.03541	Female						
Nubpl				0.00000	1.29839	Female												
Hus1b							0.00000	-1.28869	Male									
Gtf2b										0.01407	1.98801	Female						
Patl2										0.00000	1.32961	Female						
Alox12e													0.00000	2.61267	Female			
Lrp4																0.00894	4.16413	Female
Accsl																0.00568	3.73426	Female
Upb1													0.02387	3.48192	Female			
Ncbp3										0.02882	-1.54367	Male						
Xlr5c													0.00243	-3.46474	Male			
Nol11										0.00152	2.23476	Female						

Rltpr							0.00000	3.52778	Female									
Lgr5							0.02677	2.70120	Female	0.00516	-3.40032	Male						
Spink2										0.00599	-3.51946	Male						
Slc6a4													0.00703	3.80916	Female			
Ptprn				0.00000	-1.23620	Male	0.00000	3.79962	Female									
Smu1										0.01169	1.24372	Female						
Enpp3																0.00224	3.95312	Female
Mroh2b																0.02148	1.70137	Female
Zfp345							0.00033	2.76658	Female									
Gm5607										0.00069	-2.43125	Male						
Dhrs3				0.00000	-1.27641	Male										0.00000	4.66698	Female
C87414													0.01737	-1.82203	Male			
Pfas																0.02168	1.84193	Female
Arrdc2																0.00778	3.38820	Female
Klhl41										0.00000	-5.10428	Male						
Trp53										0.01156	3.08746	Female						
Nlrp12																0.00754	3.75341	Female
Ddx6													0.00195	2.21374	Female			
Rbbp4										0.00239	-2.48755	Male						
Lats2																0.00225	-3.58674	Male
Emb				0.00000	0.77275	Female												
Noc2l										0.00000	3.39166	Female						
Dph3										0.00556	-2.20821	Male						
Stra8				0.00002	-0.83668	Male												
Fam160b1				0.01026	2.65635	Female												
Eed										0.03315	1.42811	Female						
Myom3				0.00028	1.65114	Female												
Tacstd2										0.04867	-4.12544	Male						
Chm							0.01289	3.99816	Female									
Ebf3										0.00044	-1.04980	Male						
Il33										0.00000	1.85050	Female						
Ttll9										0.00000	0.80599	Female				0.00000	1.10056	Female
Atp5l										0.00000	-5.16106	Male	0.00023	1.66496	Female			
Fgd3				0.00050	0.94843	Female										0.00000	3.03920	Female
Sash1				0.00000	1.97787	Female												
Spns3							0.00001	-1.98794	Male									
Lmx1a				0.00000	2.76526	Female												
Bnc1										0.00000	-3.34499	Male						
Serf2										0.00002	-2.80371	Male						
Mgme1										0.03807	2.07240	Female						
Exosc9										0.04058	1.61650	Female						
Camk2b				0.00000	-0.75275	Male												
Prcc										0.01624	3.59951	Female						
Map3k8							0.00005	-3.41451	Male									
Gstm7				0.01168	-1.44850	Male				0.00000	1.69988	Female						
Cdc23										0.01625	2.25856	Female						
Rps24										0.00000	-4.22623	Male	0.00207	1.24643	Female			
Noa1																0.00002	3.64080	Female
Dnajc9										0.00073	-3.68373	Male						
App	0.00000	1.13995	Female	0.00001	0.70822	Female												
Fgf13							0.00154	4.47044	Female									
Adam28										0.00000	-3.44536	Male						
Ccdc152				0.00258	0.90265	Female												
Rarb										0.00120	4.85845	Female						
Ech1				0.00676	2.54155	Female												
Rnf41																0.00581	-2.19675	Male
Ccdc88b										0.04886	-0.97587	Male						
Snrpb										0.04425	2.24764	Female						
Cdo1													0.00000	2.04340	Female			
Elovl5							0.00033	3.47474	Female									
Prkra				0.00600	-1.74882	Male										0.00287	-5.58293	Male
Gtf2h4										0.00000	6.93752	Female						
Elf3										0.02926	2.31737	Female						
Ier3ip1										0.02776	-2.24269	Male						
Scpep1os										0.00000	-3.46408	Male						
Carf							0.03425	2.35465	Female									
Arl10													0.00000	-3.06252	Male			
Rassf8	0.00000	-1.47378	Male															
Bnip3										0.02475	-2.28776	Male						
Flywch2													0.00691	4.30251	Female	0.00312	-5.05810	Male

Cse1l													0.04572	-0.98025	Male			
Fah				0.00001	2.77266	Female							0.00000	2.50506	Female			
Pi4kb							0.00575	4.44337	Female									
Ddx3x							0.01758	1.40820	Female									
Olfr1372.ps1													0.02069	2.27467	Female			
Klf7													0.00000	-2.62698	Male			
Zfp516	0.00000	-1.05797	Male							0.00046	-1.80781	Male	0.00513	5.15890	Female			
Sdhd							0.00999	2.45849	Female									
Hcfc1r1										0.04425	2.37409	Female						
Hdac9										0.02817	-0.58746	Male	0.00000	-2.12554	Male			
Syt15										0.00000	-4.71006	Male						
Sh3kbp1													0.04487	3.58396	Female			
Eml6	0.00243	-0.86405	Male	0.00001	2.13757	Female	0.00000	3.82153	Female							0.00000	-1.86099	Male
Nlrc4				0.00010	0.83686	Female										0.00000	2.46016	Female
Slc22a17				0.00005	-2.48306	Male												
Smad7							0.00426	3.65207	Female									
Abhd15							0.00092	-3.37153	Male	0.00079	-4.08974	Male						
Fbxl8				0.00434	-0.92634	Male				0.01811	-1.06081	Male						
Cbr4				0.02199	2.41873	Female	0.01772	3.81304	Female	0.00350	4.19971	Female						
Blnk							0.00000	-4.23994	Male									
Rdh11							0.04589	2.91823	Female									
Dkk1										0.00288	-4.62722	Male						
Ncapd2										0.00004	2.32593	Female						
Mak				0.00028	2.80838	Female				0.02142	-1.09383	Male						
Cenpv										0.01557	-4.88312	Male						
Ms4a6c																0.00175	4.47798	Female
Zfp647																0.00000	2.43758	Female
Osbpl5				0.00027	-0.58681	Male												
Mir3473f										0.00014	-4.59252	Male						
Krcc1																0.03415	-2.72971	Male
Psmg4										0.02171	-3.38279	Male						
Nyap1																0.03477	-4.32129	Male
Rmi1																0.00007	-3.82189	Male
Dnajc14										0.00063	2.93366	Female						
Bhlhb9							0.00022	5.42556	Female							0.00003	5.79291	Female
Ptpn2													0.04050	-1.17905	Male			
Btbd18																0.00000	-4.96604	Male
Cox7a2l										0.03093	1.60746	Female						
Nagk	0.00663	4.02191	Female															
Rpl11										0.00000	-2.57758	Male	0.01508	1.15840	Female			
Prob1										0.00190	-2.08352	Male						
Zap70	0.00000	2.21040	Female															
Adap1																0.04222	3.69659	Female
Lrrn4cl										0.00000	-4.06858	Male						
Foxo4													0.04100	-3.67280	Male			
Polr1c										0.00087	1.56057	Female						
Plk5																0.00976	-4.38070	Male
Adamts4										0.00000	-5.50132	Male	0.00261	2.10389	Female			
Clk3				0.01412	3.14417	Female												
Polr1b										0.04867	1.41180	Female						
Asb4													0.03988	3.90226	Female			
Gm14403																0.00606	-1.99832	Male
Rbm22										0.03336	-1.43140	Male						
Cldn9				0.00238	-2.05751	Male												
Prss36				0.02581	2.15915	Female												
Fam43a							0.00000	2.98778	Female									
Cox8a										0.00003	-4.27761	Male						
Gtf2h5										0.01289	-1.64569	Male						
Sox17										0.00001	-0.77633	Male				0.00123	6.30443	Female
Fbxo3				0.00000	1.04098	Female				0.00070	4.84341	Female						
Atp1a1										0.01874	1.80350	Female						
Abr				0.04808	2.65099	Female												
Snx11										0.03800	3.71845	Female				0.02519	3.27780	Female
Zfand5										0.01116	-2.06992	Male						
Galntl6										0.02112	-2.90402	Male						
Fam73b																0.02876	-3.56883	Male
Npm3										0.03895	-2.98422	Male						
Rbfa										0.00075	5.45660	Female						
Mrpl42										0.00000	-2.48619	Male						
Lrch1													0.00608	4.09059	Female			

Sfswap										0.00218	3.53294	Female						
Gm13648																0.04579	2.75627	Female
Oplah										0.02858	4.03370	Female						
Coro1c										0.04522	1.73376	Female						
Gm10636										0.00268	-3.46767	Male						
B9d2				0.00087	-0.65646	Male												
Exoc3l4				0.00686	-1.14549	Male												
Cebpzos										0.00003	-3.52758	Male						
Hspd1										0.01973	1.15400	Female						
Acaa1a				0.03360	0.90742	Female												
Doc2g										0.03588	3.36597	Female						
Serpinf1													0.00000	-2.12354	Male			
Gm16702										0.00000	-5.46626	Male	0.00000	1.87275	Female			
Rest										0.01406	3.46633	Female						
Kdsr																0.01134	-2.41351	Male
Brdt													0.00392	3.73711	Female			
Bud13							0.03732	2.02874	Female	0.00001	3.89750	Female						
Polr2i										0.03776	-2.22292	Male						
Apoc1										0.00000	-4.68959	Male	0.01919	1.73956	Female			
Cog8										0.00396	4.57200	Female						
Ift22				0.00001	-1.43509	Male												
Epb42										0.01209	-3.45434	Male						
Wfdc2	0.00000	2.49662	Female	0.00000	-1.13265	Male												
Gpr155										0.00099	-1.22510	Male	0.00000	3.11186	Female	0.00000	1.44541	Female
Tmem145																0.01178	-4.37832	Male
Zfp706										0.00180	-1.27362	Male						
Exoc6													0.00271	-2.66966	Male	0.00078	-3.26380	Male
P2rx5													0.00000	1.76241	Female	0.00000	-2.06668	Male
Chmp4b										0.00000	-5.29950	Male						
Cab39l																0.02284	1.60871	Female
Vim	0.00000	1.42790	Female															
Dnajb11										0.04916	1.55948	Female						
Ppp6r2										0.00002	-3.02614	Male						
Rpl39l										0.00000	-3.23751	Male	0.02446	1.36066	Female			
Ccdc22										0.00000	6.80855	Female						
Pds5a										0.00000	4.26416	Female						
Ap2a1										0.04064	3.14049	Female						
Mrps31	0.00147	4.01875	Female							0.04404	1.58073	Female						
Fpgt										0.01496	-4.71710	Male						
Gal3st3																0.00036	-3.27949	Male
Ffar2																0.01632	0.65065	Female
Rab4b							0.01887	-4.38853	Male									
Trim71							0.00000	3.06179	Female	0.00001	-1.61900	Male						
Iqgap2													0.00000	-5.14337	Male	0.00000	4.09358	Female
Zeb1													0.01947	3.51418	Female			
Znrf2													0.04274	-1.05973	Male			
Fthl17b				0.00110	4.77047	Female	0.00000	5.51200	Female	0.00011	5.39636	Female	0.00066	3.72784	Female			
Kdm7a																0.00003	-3.04969	Male
Gsk3a										0.00000	-5.43927	Male						
AW209491																0.02828	2.68050	Female
Ranbp1										0.02527	-1.71054	Male						
Trappc4																0.00171	-1.47207	Male
Siva1										0.03651	-2.21558	Male						
Naa15							0.04604	1.44646	Female									
Taf11										0.01068	1.77928	Female						
Gmfb																0.02238	-2.40214	Male
Vbp1							0.01314	1.69362	Female									
Mfn1										0.02245	1.33276	Female						
Rpl32										0.00000	-3.75954	Male	0.00039	1.49414	Female			
Xlr3b							0.01893	2.75915	Female									
Dgat2																0.00013	3.45975	Female
Uqcrb										0.00000	-4.99217	Male						
Itm2a																0.01005	-4.02127	Male
Itga5				0.00352	1.83469	Female												
Ccne2										0.01060	3.92612	Female				0.04697	-3.28215	Male
Spesp1				0.00005	1.38788	Female												
Tpm2	0.00000	-2.05728	Male	0.00121	-2.71596	Male												
Ly6g5b																0.00292	4.07829	Female
Dusp6				0.00000	2.83411	Female												
Nespas							0.00000	1.50585	Female	0.00017	1.19907	Female						

Mmel1							0.00000	-1.31354	Male									
Trmt13										0.00006	5.70418	Female						
Clmp																0.00000	-1.31708	Male
Pak7										0.00021	-1.83260	Male						
Nmrk2										0.00001	-3.58251	Male						
Psmc2										0.00000	1.79580	Female						
Rnf40																0.00737	2.26931	Female
Lrrc25										0.00025	2.47344	Female						
Cyp2e1										0.00000	-5.27752	Male	0.00013	2.16697	Female			
Platr14										0.02698	2.72804	Female						
Polr2d										0.00000	3.54290	Female						
Hmgn1										0.00003	-2.58129	Male						
Capn2				0.01061	-2.12202	Male				0.00000	2.03376	Female						
Cybrd1										0.00605	-2.95127	Male						
Gstm6	0.00031	-0.95387	Male	0.00000	1.01458	Female	0.00002	-1.50879	Male	0.00048	-1.29876	Male						
Gk5										0.00180	4.67155	Female						
Dsg1b										0.00003	-4.02825	Male						
Pard3b				0.02695	5.94732	Female												
Zar1										0.02965	-5.35694	Male						
Arhgap30																0.04580	-3.62807	Male
Ap1s2																0.00000	4.44631	Female
Adarb1										0.02569	-2.29676	Male						
Chpf							0.01865	3.41618	Female									
Cask																0.01375	3.72795	Female
Cdc37										0.03125	2.04513	Female						
Rfx2										0.02805	2.97883	Female						
Zfp36l2										0.00345	-3.39956	Male						
Casc4										0.00080	-2.47048	Male						
Timp3													0.01560	-4.70928	Male			
Plekhb2																0.00063	-3.37869	Male
BC051665				0.00018	0.88250	Female										0.03678	5.12562	Female
Dvl3										0.00584	4.09657	Female						
Nsun6				0.01815	2.77310	Female												
Plod2				0.00000	2.27615	Female												
Adcy7													0.00000	1.67924	Female			
Fam46b																0.00181	-4.43207	Male
Tigd3										0.00001	-3.84814	Male						
Flcn							0.02375	2.11969	Female							0.00013	-2.94851	Male
Btrc										0.00841	1.89534	Female						
Sec16b													0.00000	-2.18332	Male			
Slc7a6os																0.00005	2.92150	Female
Bola2										0.00000	-3.87800	Male						
Ccdc109b							0.00000	1.54829	Female							0.00001	0.58695	Female
Gm20939							0.03788	1.64436	Female									
Neat1							0.00618	3.21864	Female									
Atat1																0.04462	3.58271	Female
Surf2										0.00004	2.62395	Female						
Cyp17a1																0.00000	-4.02484	Male
Pus10				0.00124	3.85665	Female												
Trim47	0.00000	-0.63017	Male															
Jun				0.00000	-0.77022	Male												
Mfsd9																0.02064	-5.02187	Male
Snord72																0.03014	3.28193	Female
Zdhhc15																0.00001	1.36383	Female
Ankrd45							0.00977	3.90555	Female									
Cacna2d1																0.00000	-3.04037	Male
Wdr6				0.04612	3.16851	Female				0.01450	2.98382	Female						
Gm7102										0.00016	-2.07815	Male						
Rab34																0.02064	2.83459	Female
Mmp1b							0.00653	-1.93773	Male	0.02502	-0.69979	Male						
Tuba1c										0.00012	1.54219	Female	0.02418	0.75537	Female			
Psmd13										0.00000	2.05378	Female						
Oc90							0.00000	3.58260	Female	0.00000	4.37796	Female				0.00000	2.01926	Female
Ramp2										0.01521	-3.77552	Male				0.00002	5.21776	Female
Catsperd																0.00007	-6.05338	Male
Carm1										0.00000	7.37471	Female						
Vamp2	0.00000	1.49169	Female													0.00730	-4.74751	Male
Fasn										0.04886	2.11586	Female						
Aldh9a1																0.02105	2.06133	Female
Rps19										0.00000	-3.23812	Male	0.01442	1.28894	Female			

Nrf1										0.03746	1.95122	Female						
Lsm14b										0.03207	-3.06068	Male						
Hspb2							0.00000	-1.16493	Male									
Cyp4f16				0.00000	-0.97820	Male	0.00000	1.56061	Female									
Msantd3										0.00000	1.70986	Female	0.00254	4.13538	Female			
Zfp687										0.03065	3.32689	Female						
Esr2										0.00004	-4.93441	Male						
Nlrp9c													0.02387	4.62567	Female			
Dph1										0.00004	5.50976	Female						
Mvb12b													0.01543	-4.01521	Male	0.00542	4.24958	Female
Cyp4f13				0.00559	-1.47041	Male	0.00000	0.64895	Female	0.00145	-0.78801	Male						
Sesn2										0.01010	3.18760	Female						
Susd2										0.00000	2.45296	Female	0.02951	4.42462	Female			
Gm4425	0.04632	-3.93931	Male															
Zfp976							0.02397	1.76380	Female									
Gm11549										0.00000	-4.70250	Male						
Rps6kc1																0.01805	3.70449	Female
Dact3				0.00000	0.60355	Female				0.00000	-4.20862	Male						
Cryl1																0.00382	4.36913	Female
Gas2	0.00007	2.14258	Female															
BC002163										0.00000	-4.13769	Male	0.00049	2.01417	Female			
Orai2				0.00002	1.14004	Female												
Rpl36										0.00019	-3.43478	Male						
Chp1																0.01368	-1.39432	Male
Cstb										0.00000	-4.38263	Male	0.00210	1.72701	Female			
Slc22a5							0.00000	-7.22207	Male									
Ttc8																0.00003	1.22678	Female
Cybb										0.00373	0.85682	Female				0.00000	1.23652	Female
Ppara				0.00110	1.35563	Female										0.00000	1.87526	Female
Zdhhc24							0.00723	3.84857	Female									
Slc6a2										0.00001	-3.69141	Male	0.03361	1.73495	Female			
Map6													0.00000	-4.47637	Male	0.00000	2.11256	Female
Gpr68										0.03315	-2.97432	Male						
Cep131																0.00329	4.53398	Female
Eps8l1				0.04674	-1.38727	Male												
BC028528							0.00668	3.24095	Female									
Apobec3																0.00001	5.03062	Female
Pepd	0.00391	4.32732	Female															
Aifm3																0.00458	-4.76325	Male
Orc3	0.00554	-3.05594	Male															
Mplkip										0.04588	-2.31164	Male						
Trdmt1							0.02287	4.35685	Female									
Rcn3																0.02147	3.70540	Female
Cox17										0.00000	-4.57302	Male	0.01589	1.75293	Female			
Nav3										0.00000	2.59997	Female						
Lsg1										0.03421	2.17603	Female						
Myom2													0.00000	4.14248	Female	0.00000	0.81100	Female
Pbld1										0.00000	-5.73353	Male	0.01532	1.46762	Female			
Gcnt4				0.00001	1.46614	Female				0.00804	-1.30277	Male				0.00000	4.58966	Female
Gm8234										0.00254	1.57367	Female						
Ccdc150										0.00000	2.95913	Female						
Gm16287										0.00001	-4.03076	Male						
Cilp2													0.00000	-2.70926	Male			
Nop10										0.00000	-4.17800	Male	0.00000	2.08994	Female			
Abcd2										0.00000	-4.95965	Male						
Eci1										0.03959	-2.14054	Male						
Trpc5os				0.00081	1.42812	Female							0.00000	3.94133	Female	0.00000	-2.84661	Male
Rps19.ps3										0.00000	-3.02519	Male	0.01535	1.25689	Female			
Phyhd1													0.00000	3.58847	Female			
Kdm1b																0.02986	3.24689	Female
Fmr1				0.03048	2.91273	Female												
X6.Sep	0.00000	2.65460	Female	0.00000	0.65376	Female												
Slc39a1										0.00007	-3.45362	Male	0.00094	2.18578	Female			
Sycn										0.02955	-3.19228	Male						
Med21										0.00418	-1.79943	Male						
Slc7a8				0.00772	0.73369	Female	0.00000	-0.68946	Male							0.00000	2.16773	Female
Snx19				0.00000	2.33822	Female							0.00193	4.43978	Female	0.00739	-4.86206	Male
Pdzd4										0.03109	-4.24060	Male						
Acbd4				0.03638	2.29325	Female												
Oraov1																0.01199	-2.05442	Male

Gpr4				0.00000	-1.07747	Male												
Hmgb2										0.00350	1.89889	Female						
Ogn										0.00001	-4.05380	Male						
Pxmp2										0.03205	-2.43734	Male						
Fam96a										0.02468	1.29123	Female						
Cox7a1										0.00307	-5.04529	Male						
Mtss1l																0.00000	3.66415	Female
Serpinh1				0.00000	-0.62282	Male												
BC051019										0.00000	3.52681	Female						
Srsf1							0.00853	1.45260	Female									
Dppa5a										0.00000	-2.93566	Male	0.00380	1.30926	Female			
Nfe2l1										0.03082	1.78923	Female						
Ift27													0.03070	-3.97234	Male			
Zfp182										0.00000	0.69827	Female						
Zc3h12a										0.02947	-1.94215	Male						
Crx										0.00063	-2.95014	Male						
Prpf19										0.00000	-3.59247	Male	0.03051	1.30734	Female			
Btg4													0.00017	-3.30770	Male			
Btk													0.00000	-6.06134	Male			
Cep57l1																0.04873	-1.82340	Male
Gm15645										0.02411	-1.21430	Male						
Kng1																0.00230	-3.34041	Male
Gm5577													0.02526	-3.33084	Male			
Eogt																0.01341	3.64542	Female
Omt2a										0.00012	-4.51060	Male	0.00008	3.75226	Female			
Snhg1										0.00000	-3.38357	Male				0.00976	1.55949	Female
Chd1l				0.04154	2.41205	Female												
Pqlc1																0.00247	-2.66231	Male
Cda													0.00022	0.74217	Female			
Rps15a										0.00000	-4.57088	Male	0.01757	1.22559	Female			
Psmd7										0.00312	1.32658	Female						
Gm7616										0.00000	-5.38265	Male						
Jpx										0.00000	-4.45096	Male						
Lrrc15													0.00000	5.13661	Female			
B3gnt3	0.00019	2.25759	Female				0.00000	1.03961	Female									
Swi5										0.00604	-2.10187	Male						
Golt1a	0.00054	-0.81799	Male										0.00000	-4.79655	Male			
Creb3l4																0.00715	2.93280	Female
Yipf2										0.00669	3.91793	Female						
Thsd7b							0.00854	-0.63498	Male									
Anks4b				0.04151	1.31495	Female										0.00000	1.55855	Female
Hdgfrp3				0.02618	-1.03208	Male												
AU016765							0.00000	2.40704	Female									
Mdm1										0.00167	-5.30410	Male						
Serpinb1a										0.00009	-1.85028	Male						
Mtcl1				0.01893	1.74273	Female				0.00740	3.86994	Female						
Duoxa1																0.00396	2.53641	Female
Ddx23				0.00002	-0.61652	Male												
Gm11544													0.04289	-1.60683	Male			
Reg1													0.01508	-3.63956	Male	0.00002	-0.75079	Male
Clic1	0.00000	1.16519	Female	0.00000	1.01963	Female												
X6.Mar																0.00320	-1.70671	Male
Cflar										0.00000	-5.68884	Male	0.00004	1.87638	Female			
Khsrp										0.00112	-2.48816	Male						
Tekt2																0.00028	5.18841	Female
Dhx9										0.00000	-4.80366	Male	0.00526	1.52524	Female			
Rhox9				0.00012	3.74271	Female				0.00004	0.91563	Female	0.00000	4.78164	Female			
Rps8										0.02282	-1.37993	Male						
Nmnat3							0.00000	-2.41264	Male									
Cth																0.00001	-2.12577	Male
Arrdc1																0.00025	4.04988	Female
Ttc26										0.00015	-3.69087	Male				0.04362	1.92971	Female
Gc										0.00001	-7.62828	Male						
Huwe1							0.04289	1.22408	Female	0.02699	1.41454	Female						
Tcf15										0.00226	-4.87972	Male						
Furin													0.03054	2.00237	Female			
Tcp11													0.00000	2.84462	Female			
Ganc																0.00030	-6.13798	Male
B3gnt5										0.04408	-4.66464	Male						
Rps11										0.00000	-3.35375	Male	0.00022	1.35928	Female			

Eif4e1b																0.01490	3.80391	Female
Tcf7l1				0.00018	-1.76203	Male												
Chrnb4													0.00036	4.11256	Female			
Ppib										0.00000	4.46323	Female						
Ppia										0.04970	-1.02339	Male						
Tceal8				0.00000	3.24440	Female							0.00004	5.69719	Female			
Gsdmc3																0.00834	3.91723	Female
Muc13																0.04753	4.47517	Female
Gm13083										0.00001	3.21397	Female				0.00436	-1.86074	Male
Cd1d1													0.00000	2.23594	Female			
Cyb5b																0.01243	2.61720	Female
Mllt11																0.01368	3.71380	Female
Lsm6													0.03262	1.36209	Female			
Nono							0.00535	1.12623	Female									
Zfp395										0.03659	2.86014	Female						
H2afj																0.01139	-2.95222	Male
Spin2d							0.00095	5.02628	Female									
Rab20										0.00075	3.03824	Female						
Set										0.00006	1.36376	Female						
Gm12250							0.02263	5.00251	Female				0.00000	4.08463	Female			
Efcab3													0.00000	2.24199	Female	0.00000	-1.30991	Male
Cdx1													0.00000	2.76202	Female			
Amdhd2				0.00268	2.25994	Female												
Arl1										0.00082	2.61326	Female						
Rplp0										0.03751	2.24538	Female						
Grm4							0.00000	-1.85981	Male									
Mnt																0.00069	-3.34689	Male
AY074887										0.03763	-1.58400	Male						
Pdha2				0.00000	2.49929	Female	0.00511	-1.41396	Male							0.00001	0.99797	Female
Eif2s3x							0.03818	1.13157	Female	0.04496	1.20253	Female						
Fbxo7										0.00000	2.11393	Female						
Rpl39							0.00001	2.39503	Female	0.00000	-5.78278	Male	0.00000	2.99210	Female			
Ciart										0.01657	3.41814	Female						
Bcl3				0.01292	1.77823	Female												
Afap1										0.01467	-3.23463	Male	0.00060	3.22107	Female			
Gm6367										0.02476	-2.85390	Male						
Mroh8													0.02058	-3.88238	Male			
Cct5										0.00268	1.16914	Female						
Id1													0.03197	-3.09098	Male			
Snord47				0.03163	3.41663	Female												
Agbl5																0.00000	4.00610	Female
D5Ertd577e																0.00000	-4.80607	Male
Magoh										0.00000	-2.62486	Male						
Gm6083										0.00000	-3.35856	Male	0.00229	1.36126	Female			
Pawr										0.00350	4.00156	Female						
Pitpnc1							0.02728	2.77819	Female	0.00080	4.03039	Female						
Fam219a							0.00000	-1.26232	Male									
Gjc3										0.00007	-3.04577	Male						
Pygo1																0.00000	-0.60929	Male
S1pr4										0.00015	-4.31005	Male						
Neil1										0.00722	4.92694	Female						
Mark2										0.03588	2.06666	Female						
Kif2c																0.00002	-3.04681	Male
Lepr										0.00001	-3.18273	Male						
Myo7a	0.00002	-1.16555	Male	0.00000	1.22320	Female												
Dapp1										0.04183	-3.79902	Male						
Ppp2r5a										0.00194	4.74331	Female						
Slamf8										0.01183	-3.35271	Male						
Grn																0.00959	-1.59342	Male
Nxn										0.00806	4.29011	Female				0.00317	4.50242	Female
Seh1l													0.04617	-0.99013	Male			
Snhg9													0.01646	2.80881	Female			
Pttg1										0.00015	-2.33813	Male						
Ilf2										0.00229	2.31668	Female						
Mt1										0.00057	-4.01708	Male						
Cubn																0.00186	5.27981	Female
Npepl1				0.00000	-1.85050	Male												
Liph													0.00390	3.77526	Female			
Prcp	0.04461	-3.77236	Male															
Mrpl1																0.00272	-2.15782	Male

Rapgef4										0.00000	-4.60924	Male						
Rpl36a							0.00301	1.61928	Female	0.00000	-4.37088	Male	0.00008	1.65727	Female			
Cacna1d				0.02354	1.15200	Female				0.00519	0.80939	Female						
Ap2m1																0.00086	-1.31706	Male
Txndc5				0.00502	3.46929	Female												
Tmem63b							0.02728	3.57825	Female									
Gm6815										0.00910	2.37626	Female						
Fsd1										0.02690	4.74013	Female						
Arhgef2										0.00000	1.73072	Female						
Gadd45gip1										0.00001	-4.65462	Male						
Ebp													0.00109	1.82469	Female			
Kank1				0.02354	0.86710	Female												
Zfp972													0.00187	2.43023	Female	0.00000	-4.85152	Male
Parva	0.00003	-0.66695	Male	0.00000	-0.68898	Male				0.00000	-0.71108	Male						
Cdk2										0.00000	4.14766	Female						
Cdc42bpg										0.00002	-5.69479	Male						
Cep164										0.00185	3.89878	Female						
Arnt																0.02454	-2.28703	Male
Ddx26b																0.00000	4.96349	Female
Gpc4																0.00009	5.23292	Female
Xdh				0.00110	0.59609	Female							0.00000	-3.02347	Male			
Rad9a										0.04074	2.35790	Female						
Morf4l2										0.03848	1.39119	Female						
Kdm3b										0.00004	2.52148	Female						
Drc1																0.00188	-3.73702	Male
Hnrnph2							0.03555	1.82076	Female									
Jak3				0.00022	-1.35573	Male												
Fbxw13										0.03076	-5.15924	Male	0.00000	1.41077	Female			
Zbed3										0.00000	-6.84021	Male						
Cul2										0.01274	1.90022	Female						
Ndufab1										0.00011	-4.01008	Male						
Sbf1										0.00179	4.49661	Female						
Sat2																0.00315	3.05089	Female
Themis3										0.00001	2.97584	Female						
Wdr54							0.01056	-5.96215	Male									
Rps26										0.00000	-3.84468	Male	0.04266	1.25209	Female			
Chac1				0.00625	1.13567	Female												
Kcnv2				0.02831	1.85758	Female	0.00000	-2.12394	Male							0.00000	2.08342	Female
Dap				0.01728	0.68462	Female												
Fbxo10																0.03731	4.13439	Female
Ireb2																0.02986	1.86855	Female
Grid2ip										0.00084	2.39138	Female	0.00000	-2.95578	Male			
Uba1y				0.00002	-6.03973	Male	0.00000	-5.10728	Male	0.00000	-6.57654	Male	0.00035	-3.02280	Male	0.00669	-2.82586	Male
BC017158										0.00907	4.69578	Female						
Brd9																0.03117	-1.36244	Male
Slc12a7										0.04830	1.78644	Female						
Slc36a1os										0.00139	-1.90856	Male	0.01123	1.20510	Female			
Eno2																0.02614	4.58283	Female
Phf2				0.00783	-1.69543	Male				0.00000	2.08076	Female						
Gm1110													0.01142	-5.04329	Male			
Kin										0.00740	2.88092	Female						
Tor4a										0.00000	-4.39490	Male						
Slc6a8							0.01541	1.38320	Female	0.03055	1.44022	Female						
Irak1										0.00000	-5.47580	Male						
Mical2										0.00016	2.39200	Female				0.00000	1.38026	Female
Paqr7										0.04552	-3.43062	Male						
Ulk3										0.03767	3.49613	Female						
Klhl23										0.00026	-1.99765	Male				0.00898	-5.58448	Male
Pth1r																0.00297	4.61127	Female
Ddi2										0.00033	5.68881	Female						
Irx2										0.00102	-1.80556	Male						
Mtmr9				0.00000	1.19938	Female												
Fabp3										0.01890	-2.95793	Male						
Smtn										0.03488	3.59923	Female						
Idh3g										0.00000	1.94713	Female				0.00305	1.10341	Female
Ccno																0.04930	3.62696	Female
Umps										0.00907	1.29137	Female						
Ogt							0.02647	1.85252	Female									
Pkd2l2													0.01336	4.10796	Female			
Papss1																0.00062	-3.66739	Male

Sms							0.00064	1.35330	Female							0.01902	0.98188	Female
Scube1										0.00000	-5.99752	Male						
BC049715													0.01542	-2.82765	Male			
Lmo1													0.02748	-4.62928	Male			
Rtcb										0.02524	1.62459	Female						
Pdlim4	0.00001	2.20070	Female															
Esrp2	0.00985	3.81520	Female							0.01041	2.84833	Female						
Mtrf1				0.00000	1.18980	Female												
X7.Sep													0.01233	-2.00809	Male			
Clpb																0.02218	-2.22047	Male
Trmt2b							0.00643	4.65173	Female									
Ehd2	0.00011	0.90509	Female													0.00000	-1.32259	Male
Frem2										0.00000	1.78361	Female						
Uaca	0.00000	-0.64492	Male															
Gm19583	0.01977	0.99125	Female															
Ccdc114										0.00014	1.94206	Female				0.00000	-3.08886	Male
Rpl28										0.00000	-3.42045	Male	0.01698	1.40510	Female			
Flnb										0.00082	2.33888	Female						
Arg1																0.00226	-4.37323	Male
Svopl										0.01004	-4.10461	Male						
Mlycd	0.00004	-1.26264	Male															
Rab33b										0.00018	-3.08689	Male						
Fbxw26													0.00228	4.94501	Female			
Gbp4													0.01508	-4.87146	Male			
AU015836																0.00020	-5.62911	Male
Snhg12										0.02272	-2.27603	Male						
Armc6										0.02837	2.57192	Female				0.04216	-2.02227	Male
Dnm3							0.00002	2.60450	Female									
Rassf5																0.00000	1.49834	Female
Htatip2				0.02878	1.89126	Female												
Rps2										0.00000	-3.43213	Male						
Mpc2										0.00016	-4.22145	Male						
Ndufa1										0.00000	-4.16300	Male	0.00001	2.43343	Female			
Ppp2r1a										0.00002	2.13468	Female						
Chd3os													0.00054	5.71448	Female			
Rsrc1	0.00233	-4.42714	Male															
Pim2																0.00000	5.53264	Female
Glul																0.01746	-1.15648	Male
Scg3													0.00000	2.84901	Female			
Ubox5																0.01172	3.01933	Female
Intu										0.00153	-2.64659	Male						
BC039771										0.00005	-3.31207	Male						
Abcg1				0.00002	1.15920	Female										0.00999	2.23520	Female
Kctd10										0.00000	4.58765	Female						
Pus1										0.00609	2.15677	Female						
Ano9																0.00000	-5.35433	Male
Hoxb7																0.00000	2.07587	Female
Atg9b							0.00000	0.80915	Female	0.00000	2.07521	Female						
Zfp407																0.00045	-3.96158	Male
Fbxo43										0.02847	-3.35016	Male						
Ttll3																0.02273	-3.33458	Male
Tspo	0.00036	0.75263	Female							0.00001	-0.65132	Male						
Hspa4l										0.00005	-2.26269	Male						
Shkbp1										0.01481	3.78956	Female						
Rps15										0.00000	-4.55735	Male	0.03379	1.32596	Female			
Ccdc88c				0.00853	2.96714	Female				0.00000	6.91284	Female						
Frmpd1				0.00021	2.74456	Female				0.04243	2.97934	Female						
Omt2b										0.00109	-4.02775	Male	0.00024	3.46765	Female			
Irs3													0.00066	0.93316	Female	0.00354	1.50108	Female
Sapcd2																0.01279	-2.51738	Male
Tmod2																0.00013	4.79100	Female
Med28										0.03070	-2.19798	Male						
Smco1										0.00000	-4.12456	Male						
Tmem256										0.00335	-4.42212	Male						
Pced1b										0.00081	-3.92168	Male				0.01126	2.70805	Female
Prr14										0.03079	2.41042	Female						
Mcts1							0.00087	2.02786	Female									
Dmrt1	0.00061	0.91860	Female															
Kcnj14				0.00151	1.04538	Female							0.00000	4.42813	Female			
Fam213b				0.00005	-1.38680	Male	0.00000	0.85119	Female	0.00000	-4.11689	Male						

Ncln										0.00061	4.06760	Female						
Psmc3										0.03240	1.18936	Female						
Hax1																0.01210	-1.10356	Male
P3h1													0.02856	3.29271	Female			
Gstm2	0.00000	-0.84695	Male							0.00001	-1.96343	Male						
Gm9376										0.00000	-3.55465	Male						
Tst										0.00000	1.69062	Female						
Hspa13										0.00000	0.93371	Female						
Mex3a				0.00000	1.33509	Female	0.00712	3.67696	Female									
Fam8a1																0.00022	-6.55832	Male
Kdm6a							0.01124	2.55206	Female	0.00183	3.22370	Female						
Zfp729b																0.04728	-2.29112	Male
Thoc2							0.00885	2.65998	Female									
Parp14										0.00030	-2.27132	Male						
BB557941													0.00000	5.23475	Female			
Sgsm1																0.02280	2.82265	Female
Rnf150																0.00258	4.14651	Female
Zfp941										0.00006	-4.31486	Male						
Prrg4				0.02182	0.74182	Female												
Jmy																0.01746	-2.36826	Male
Ephx3	0.00066	3.24681	Female				0.04379	-4.98809	Male									
Tppp3													0.02859	3.62316	Female			
H2afv										0.00017	-3.29820	Male						
Pld4							0.00024	-3.34654	Male									
Sh3bp5				0.02307	-1.19466	Male												
Stk11										0.03037	2.57353	Female						
Zfp709				0.00314	1.16986	Female	0.00000	1.63343	Female									
Adgrf3	0.00000	1.88551	Female										0.00010	-5.28888	Male			
Swap70										0.04733	2.17834	Female						
Rps29										0.00000	-5.81626	Male	0.00001	2.19724	Female			
Cav2													0.00000	-0.67298	Male			
Syngr3																0.00349	-4.96864	Male
Snrpd1										0.00000	-2.93878	Male	0.02652	0.99348	Female			
Dis3													0.00143	-2.04936	Male			
Tmem63a																0.00919	3.83025	Female
Dmbx1													0.00004	-5.35156	Male			
Ptcd3										0.04255	1.22953	Female						
Fcer2a										0.00000	-3.92778	Male						
Map7																0.00051	-1.62249	Male
Trip4																0.01376	1.63667	Female
Gm15698																0.03936	-1.24187	Male
Psmd14										0.03055	1.08893	Female						
Rps15a.ps4										0.00000	-4.76692	Male						
Ccpg1																0.02150	-1.55115	Male
Adh1										0.00005	-2.01665	Male						
Phldb2	0.00000	-1.18628	Male															
Homer3																0.00000	-5.15409	Male
Tma16																0.04334	-1.55151	Male
Crip1										0.00098	-4.62323	Male						
Slc7a1				0.00000	1.26327	Female				0.00000	2.61798	Female				0.01043	4.92636	Female
Taf9b										0.00000	-2.59728	Male	0.00000	-2.58666	Male			
Vcl				0.01016	3.40260	Female												
Herc3							0.00019	-2.28488	Male									
Cog6																0.02448	3.45069	Female
Lpl							0.04770	-3.06465	Male									
Spag4																0.03082	3.39320	Female
Tubb3																0.00158	4.14308	Female
Med6																0.00132	-2.30939	Male
Sowahb							0.00037	-1.76509	Male	0.00033	1.93396	Female						
Srp9										0.00858	-2.04017	Male						
Zfand2a																0.00000	-2.37051	Male
Prr18										0.00000	-1.97094	Male						
Pianp				0.00011	-1.40417	Male				0.00000	4.49359	Female						
Zfp113							0.01406	2.12311	Female	0.00280	-2.73733	Male						
Pin4										0.00000	-4.23892	Male	0.00000	2.56559	Female			
Bach2										0.00311	-3.45274	Male						
Gm1600																0.01825	-4.09340	Male
Pip5k1a													0.01508	2.29800	Female			
Hsd17b2	0.00030	2.39164	Female										0.00000	2.38120	Female			
Npc1l1																0.03777	2.66140	Female

Rasal2																0.02042	3.18484	Female
Acrbp																0.02614	-3.59926	Male
Gm29443										0.00014	-4.12214	Male						
Mpnd							0.02728	3.44574	Female									
Rpl12										0.01241	-2.05412	Male						
Tbc1d14				0.00005	4.50728	Female												
Abhd4				0.00190	2.00919	Female												
Eef1e1																0.04546	-0.94482	Male
Mcm3										0.01374	1.73411	Female						
Mef2d				0.00034	3.99606	Female												
Popdc3																0.01818	4.38620	Female
Ddx39										0.02500	1.05268	Female						
Mdh2										0.00468	1.71566	Female						
Taf7										0.00000	5.40855	Female						
Gcat	0.02674	3.52782	Female															
Amn										0.03141	-4.62970	Male				0.01879	3.96676	Female
Smad2																0.01199	1.91192	Female
Sod2										0.00131	-1.65041	Male						
Bri3										0.00000	-5.17423	Male						
Mir684.1										0.00003	-1.84676	Male						
Slc27a1																0.00413	-2.81339	Male
Aldh1l1																0.02410	4.12008	Female
Tbx3																0.03520	1.51906	Female
Ndufa6										0.04923	-2.30260	Male						
Fthl17e				0.00131	4.90826	Female	0.00000	6.45251	Female	0.00204	4.38553	Female	0.00011	3.89300	Female			
Guca1b				0.00030	0.72247	Female												
Myod1				0.00062	0.95399	Female	0.00000	-0.65521	Male				0.00000	1.04838	Female			
Hspb8				0.04399	-1.85033	Male												
Mbd5										0.03530	2.87874	Female						
Arfgap2				0.04211	2.32202	Female												
Tuba8										0.00000	3.74089	Female	0.00364	5.07508	Female	0.00021	-6.61241	Male
Acmsd										0.01084	-2.59730	Male						
Tsnaxip1													0.00081	4.08242	Female			
As3mt	0.00786	-4.22613	Male	0.02226	2.09438	Female												
Pfdn5										0.00000	-3.31243	Male						
Abcb8							0.00013	4.17560	Female									
Nfkbia																0.00174	4.40127	Female
Ccdc94										0.04464	2.89430	Female				0.00813	-2.92762	Male
Zfp607a										0.00001	-3.30454	Male	0.00669	1.66669	Female			
Trnau1ap										0.02512	-2.70297	Male						
Oas1b							0.03414	-2.42593	Male									
Cd59b										0.01001	-4.25347	Male						
Tbx15				0.00006	-1.37748	Male				0.00013	-1.53157	Male						
Zfp599							0.03797	2.95801	Female									
Ndufv3										0.00001	-3.45375	Male	0.00026	2.16609	Female			
Nfe2																0.00542	4.33079	Female
Ap2s1										0.00168	4.30680	Female						
Hspb1										0.00000	-7.18998	Male						
Ftx																0.00159	4.17310	Female
Ddx49										0.00283	3.28897	Female						
Dad1										0.01004	-1.80261	Male						
Etv6										0.03076	3.34789	Female						
Il6ra										0.00161	3.87411	Female						
Rbm3							0.04338	0.93144	Female							0.03858	0.85502	Female
Tnk1													0.00000	1.48167	Female			
Arl6ip5	0.00000	-0.98798	Male							0.00055	-2.02744	Male						
Palld																0.00002	4.88139	Female
Hck										0.00000	1.82726	Female						
Fundc2													0.00002	-5.65334	Male			
Hint3										0.00033	-3.88702	Male						
Kdm5d	0.00075	-1.45859	Male	0.00000	-1.18539	Male				0.00004	-3.82511	Male	0.00000	-3.86405	Male	0.00000	-5.11971	Male
Tex19.1							0.03914	1.27864	Female	0.00158	1.91826	Female						
Irf1													0.00000	-0.81227	Male	0.02247	3.79377	Female
Ksr1	0.00174	-0.87426	Male													0.00000	-1.40205	Male
Hspb6										0.00000	0.87873	Female						
Fabp4										0.02125	-3.56619	Male						
Mlh3							0.00200	3.84158	Female				0.01752	2.67365	Female			
Hoxa10										0.00798	1.42068	Female	0.00001	1.44273	Female			
Pgap3										0.00121	1.05095	Female						
Cfap74													0.04458	0.96142	Female			

Gorasp1																0.04789	2.01253	Female
Dcdc2c										0.00003	-2.28513	Male						
Cops3										0.00000	2.63223	Female						
Stx1a																0.00018	-4.26593	Male
Tnfrsf10b										0.00553	-2.56848	Male						
Sgpp2										0.00464	-2.66286	Male						
Mtg2										0.00039	5.24057	Female						
Gm14005										0.00000	-4.53308	Male	0.00165	1.95621	Female			
Lgals1										0.00194	-4.99306	Male						
Fundc1										0.01281	2.68229	Female						
Obsl1	0.00000	-1.81988	Male	0.00000	-1.08215	Male												
Nt5c	0.00974	4.19316	Female															
Dnajb3																0.02808	-4.73899	Male
Fbxo36																0.00000	-5.46971	Male
Cdh4													0.00000	-3.78905	Male			
Rps15a.ps6										0.00000	-4.68437	Male	0.04089	1.14174	Female			
Spin2c																0.00113	-6.03546	Male
Stk35																0.00500	2.85029	Female
Nckap1l	0.00499	-1.42903	Male							0.01827	-0.91486	Male						
Cpa1													0.00229	-4.13230	Male	0.00335	-4.28484	Male
Atp5e										0.00000	-4.78423	Male	0.03851	1.55447	Female			
Pi4ka																0.00223	2.36373	Female
Txn2										0.00627	2.17368	Female						
Rimbp3				0.00168	1.97392	Female	0.00000	0.81763	Female							0.00000	-2.80560	Male
Kcnd1							0.00000	2.27503	Female									
Rnf13	0.00004	5.96659	Female															
Atp5k										0.00000	-5.23206	Male	0.00033	1.73469	Female			
Atp11b																0.00063	-3.54252	Male
Toe1										0.01241	1.85387	Female						
Tmem40																0.04485	-4.52642	Male
Spry2										0.00910	-3.32535	Male						
Sfi1																0.00217	-2.17832	Male
Mrpl44										0.00741	1.60410	Female						
Ell3																0.04222	-2.66922	Male
Aqp11													0.04811	3.02229	Female	0.00784	3.71252	Female
Pcolce2	0.00867	-1.19772	Male										0.00000	1.98116	Female			
Tmem218													0.00000	4.21276	Female			
Ccnd1																0.00004	-5.95355	Male
Faxc																0.00000	-1.21057	Male
Fbxo2				0.00000	-0.80163	Male												
Stk4																0.00783	-2.04865	Male
Klhl17	0.00004	1.47797	Female													0.01545	3.98165	Female
Lst1										0.00000	-7.25638	Male				0.00366	-3.72670	Male
Thyn1										0.00902	2.22540	Female						
Tmem258										0.00000	-5.38273	Male						
Pde4c										0.00050	-2.90285	Male						
Tgfb2													0.02209	-5.03503	Male			
Pced1a	0.00000	2.95797	Female															
Nedd4l										0.00051	3.83249	Female						
Ost4										0.00015	-2.97751	Male						
Asprv1							0.00561	1.92543	Female									
Smdt1										0.00704	-3.02588	Male						
Man1c1													0.00998	4.21165	Female			
Psmb11																0.00506	-2.49302	Male
Ndufa5	0.01390	3.28566	Female							0.00000	-5.73748	Male	0.00000	2.87191	Female			
Bin1										0.00000	1.73298	Female						
Gm3139																0.00622	-1.27933	Male
Marveld1	0.00004	1.84516	Female										0.00000	1.24417	Female			
Zfp423										0.02188	-1.08644	Male						
Ptch1							0.03472	-4.65974	Male									
Ptprh										0.04717	-4.59780	Male						
Plxna3				0.00854	1.64759	Female							0.00000	2.28939	Female			
Prtn3	0.00001	1.20994	Female													0.02149	-4.56276	Male
Nub1																0.00977	-1.16290	Male
Hif3a													0.02522	3.90372	Female			
Nbl1	0.00237	1.25013	Female	0.01135	-1.39659	Male												
Idh3b										0.01135	1.43807	Female						
Eln																0.00000	-0.59285	Male
Cs										0.00004	1.81581	Female						
Fam135a																0.00108	-4.91274	Male

Ilk																0.00009	-3.02986	Male
Fndc4	0.00014	1.28215	Female															
Tuba1a	0.03040	-1.66131	Male							0.00019	1.51307	Female	0.01508	0.80046	Female			
Iars																0.00167	1.63425	Female
Milr1							0.01685	3.77690	Female							0.01728	3.46265	Female
Mettl20	0.00000	1.47951	Female															
Dleu2				0.02896	-1.53664	Male												
Ddx3y							0.00000	-7.41047	Male	0.00000	-7.48924	Male	0.00000	-8.94889	Male	0.00000	-7.30054	Male
Ube2h										0.02775	1.68128	Female						
Il6st										0.00000	0.91302	Female	0.01919	3.80364	Female			
Cep126	0.00000	-0.79742	Male	0.00000	1.25789	Female												
Gm7977										0.00000	4.43525	Female						
Zfp938																0.00000	-6.07022	Male
Minos1										0.00000	-4.61146	Male	0.01374	1.42755	Female			
Uqcrfs1										0.00798	-2.07589	Male						
Abt1										0.00013	2.96801	Female						
Tspan31										0.02251	1.77835	Female						
Dync2h1				0.01709	2.89765	Female												
Sirt4										0.03205	3.90969	Female				0.02775	-4.19699	Male
Gm6498										0.00583	2.06804	Female						
Nme2										0.00317	-1.92133	Male						
H60b													0.03851	4.33461	Female			
Actn4										0.00000	3.93987	Female						
Npy1r										0.00051	-2.71495	Male						
Pnrc1													0.01140	-3.95297	Male			
Desi1																0.01945	2.29734	Female
Slc33a1				0.01479	3.26583	Female												
Acadl	0.00002	-3.39687	Male															
Gpr50																0.00000	-0.88266	Male
Elf1										0.04733	-2.83783	Male						
Gpr146				0.00043	2.32073	Female	0.00000	0.59287	Female							0.01859	4.26171	Female
Chrna1										0.00000	-6.51781	Male	0.00000	2.50949	Female			
Pipox				0.00011	1.67577	Female												
Ttyh3																0.00226	4.42663	Female
Mslnl													0.02766	4.10452	Female			
Cox7a2										0.00000	-5.36937	Male	0.00000	2.32044	Female			
Dynlt1b										0.00568	-1.77402	Male						
Taf4b													0.00793	-2.01429	Male			
Mtif2							0.00073	2.35657	Female	0.02661	-2.00166	Male						
Sfmbt2	0.00000	1.67045	Female	0.00000	1.72036	Female				0.00377	3.89139	Female						
Cgnl1										0.00055	3.59584	Female						
Uqcc2										0.00008	-4.05983	Male	0.02251	2.04768	Female			
Rps16										0.01912	-2.01628	Male						
Zc3h7b													0.00174	4.88141	Female			
Hibadh				0.00073	3.05853	Female												
Myl6										0.03133	-1.49696	Male						
Arl14epl	0.04499	3.72141	Female															
Obox7										0.01091	-4.31587	Male	0.00464	-3.82784	Male			
Pom121										0.03524	2.02379	Female						
Pdzk1ip1										0.00000	-0.87429	Male						
Slc35b1										0.03334	1.48727	Female						
Greb1										0.00000	-0.58277	Male						
Iqcb1																0.04717	5.42506	Female
Stk17b																0.01508	-2.92535	Male
Cdc5l										0.01436	1.30286	Female						
Magi3										0.03763	-1.45958	Male						
Gm6710																0.00141	-2.67145	Male
Dock2							0.00002	-3.06822	Male									
Vat1										0.01308	-2.83161	Male				0.03168	-2.25804	Male
Zfp287							0.02631	3.35135	Female	0.04414	-2.88947	Male						
Obox6																0.00100	-3.10345	Male
Sepw1										0.00856	-2.26848	Male						
Dync1h1										0.04890	1.96668	Female						
Fbxo32										0.00000	-4.95008	Male	0.02938	2.07233	Female			
Map4k3							0.00614	2.77177	Female				0.01721	-2.10785	Male			
Emilin2													0.02665	-5.23748	Male			
Lrrc59										0.00106	1.56969	Female						
Gfra3																0.02881	2.66334	Female
Zfp398							0.00000	0.83740	Female									
Med15				0.00099	3.64919	Female												

Scai																0.03770	2.99090	Female
Slc30a7	0.01335	-3.92928	Male															
Smo				0.00945	0.79654	Female				0.00054	1.38560	Female	0.00000	-4.39828	Male			
Snhg8										0.02352	-3.21683	Male						
Maml1										0.03272	3.01441	Female						
Cnot3										0.00771	3.11524	Female						
Clec2g													0.00034	4.00657	Female			
Copz1										0.04944	1.02880	Female						
Pdlim2																0.01824	-4.90558	Male
Nol7										0.00000	-3.90312	Male						
Gm1976										0.04391	-2.31555	Male						
Gm15421										0.00000	-5.12728	Male	0.00962	1.40849	Female			
Nphp3																0.03042	3.85826	Female
Gpr137b																0.00976	-1.39669	Male
Evx1							0.00001	2.32493	Female				0.00000	1.72037	Female			
Evpl				0.00734	-0.81822	Male												
Epb41										0.00226	3.02613	Female						
Mrps24										0.00000	-5.81371	Male						
Zcchc6										0.00001	5.03579	Female				0.01724	-2.59517	Male
Cmip										0.00226	3.77339	Female						
Tceb1										0.00002	-2.11553	Male				0.00731	-1.23414	Male
Cdnf																0.00013	-3.43680	Male
Itgb7																0.01406	3.13165	Female
Zp2																0.00000	4.85828	Female
Eif3e										0.01690	1.03764	Female						
Fhad1																0.00009	1.47545	Female
Snrnp27										0.00395	-1.93958	Male						
Stamos							0.00000	-1.86992	Male									
Siae	0.00031	-0.88599	Male	0.00008	0.71216	Female												
Rrp15										0.00869	3.59679	Female						
Panx1	0.00000	-1.59029	Male															
Fam19a5				0.02896	-1.56403	Male				0.00000	2.22829	Female						
Cisd1										0.02063	-2.36713	Male						
Gm16513										0.00525	-2.08362	Male						
Map1a										0.00171	-1.91367	Male				0.00000	1.36423	Female
Adamtsl1										0.00000	-3.38454	Male	0.00000	-2.13787	Male			
Ifngr2													0.00189	4.00724	Female			
Lrrn4													0.03370	3.70158	Female	0.00558	4.60653	Female
Trim14																0.04172	2.47966	Female
Lncenc1	0.00007	-0.93671	Male	0.00001	0.97310	Female												
Fkbp1b										0.04762	-3.95641	Male						
Fbxw21													0.00216	4.74281	Female			
Ift74																0.00495	4.01886	Female
Wdr93																0.00144	-4.50594	Male
Cox7c							0.02952	1.52394	Female	0.00000	-4.55599	Male	0.00019	1.80958	Female			
Gpx6							0.00038	0.98074	Female				0.00000	1.12607	Female			
Cntn2										0.00117	-3.63164	Male						
Ing5																0.00898	-1.89853	Male
Cd200																0.00000	0.95999	Female
Chil1										0.00011	1.72141	Female						
Rpl9										0.02754	-1.11213	Male						
Kdm4b							0.00122	4.01631	Female									
Crlf1																0.00022	-0.91327	Male
Fkbp10													0.02446	-3.19349	Male			
Cpne5																0.00000	2.99269	Female
Bfsp1				0.00006	2.02862	Female	0.00001	1.03037	Female				0.04674	4.62054	Female			
Abtb2							0.00627	2.41574	Female									
Mrps14										0.00667	-1.84090	Male						
Galnt16													0.04980	2.91373	Female			
Tceanc							0.00885	4.28255	Female									
Tmem242										0.02251	2.31091	Female						
Tpm3										0.00439	1.29503	Female						
Naip2										0.02212	-2.95176	Male						
Pptc7													0.02058	2.63319	Female			
Gm13125				0.00001	2.05101	Female	0.00000	4.53706	Female	0.02081	-0.84542	Male	0.00000	1.15704	Female			
Prmt6																0.00841	2.21999	Female
Smarce1										0.01765	1.57566	Female				0.00611	-1.44202	Male
Malsu1										0.00000	-6.08102	Male						
Mgl2													0.03135	2.04827	Female	0.00052	-3.21397	Male
Oacyl																0.00034	-0.63747	Male

Ntpcr	0.00037	-1.03056	Male	0.00210	-1.22548	Male				0.00000	1.18456	Female						
Colec12										0.04365	-1.59258	Male						
Parp12													0.00000	-6.27793	Male			
Ptpdc1													0.00012	-5.59945	Male	0.00000	-1.18430	Male
Eno1										0.02750	3.26389	Female						
Dcaf12l1							0.00215	3.85573	Female									
Cdkl1													0.00007	0.86137	Female	0.00132	-0.59352	Male
Uqcr11										0.00057	-3.19844	Male						
Rab19													0.00000	0.73503	Female	0.00000	0.58654	Female
Eomes				0.00493	2.67357	Female	0.00045	3.81038	Female									
Gpsm1																0.00000	5.25684	Female
Snrpg										0.00000	-5.62905	Male	0.00000	2.05797	Female			
Vcp										0.01022	1.76301	Female						
Chd5				0.00824	-1.53897	Male										0.00181	-0.58610	Male
Taf5l													0.00937	-1.78698	Male			
Dynlrb1										0.00000	-2.77953	Male						
Phkg2										0.00001	6.16665	Female						
Ramp1																0.00000	-1.07148	Male
Glod5																0.00960	4.61662	Female
Ric8b	0.00000	2.02973	Female															
Dpcr1							0.04338	-3.74022	Male									
Chsy1										0.01766	-4.69000	Male	0.00011	5.05200	Female			
Tmem29										0.02692	-2.82187	Male				0.00888	2.67076	Female
Megf6							0.01758	4.01972	Female									
Chml							0.00000	3.42248	Female	0.00014	-2.18994	Male						
Nphs1										0.01187	-2.67213	Male						
Slc29a4	0.00001	-2.44885	Male															
Bcar3				0.00000	2.14319	Female												
Apela				0.00014	-1.28366	Male												
Ppp4c										0.00000	6.17599	Female						
Wwc1										0.00016	5.69222	Female						
Atrnl1													0.03379	4.63977	Female			
Ndufb7										0.00000	-4.12652	Male						
Ube2s										0.00464	-2.33386	Male						
Rps17										0.00000	-4.37961	Male	0.00452	1.33334	Female			
Stxbp4										0.00027	-2.93139	Male						
Gm5860										0.00563	-1.70818	Male						
Gm5127													0.00000	2.66080	Female			
Csta1										0.03143	-3.64928	Male						
Taf2													0.00835	-1.69691	Male			
Mttp																0.00000	-5.57816	Male
Lemd2				0.00000	-0.97693	Male												
Fcgr2b										0.00000	7.21667	Female				0.01728	2.49932	Female
Amigo3																0.01432	-4.45209	Male
Tdpoz1																0.04385	2.49431	Female
Ulk1										0.03111	1.39247	Female						
Uqcc3										0.02272	-2.15902	Male						
Erg										0.00067	-1.69416	Male						
Vmn1r65																0.00573	2.98542	Female
Lpin2													0.02139	-3.72459	Male			
Acvr1b				0.00000	-1.50102	Male				0.00000	-2.66974	Male						
Crtc2							0.03552	3.18567	Female									
Trappc2				0.03142	2.62958	Female												
Pex6				0.03048	0.69806	Female												
Gm17644										0.00158	3.65493	Female						
Scand1										0.00041	-5.36301	Male						
Med31										0.00510	-2.45237	Male						
Snhg3										0.00057	-3.25418	Male						
Inhbb													0.00000	1.83752	Female			
Abhd8				0.01597	-1.55263	Male												
Atg4a				0.01815	2.28002	Female				0.02090	2.85154	Female						
Tdrd3				0.00560	3.56749	Female												
Nradd																0.00699	4.82835	Female
Magea8	0.00000	1.80301	Female							0.00000	-1.14576	Male						
Itga3				0.03229	0.81900	Female												
Mbnl2										0.00000	3.01679	Female						
Spon2										0.00192	1.42034	Female						
AI854703													0.00000	-5.37839	Male	0.00000	1.99130	Female
Gstt2										0.01066	-1.00901	Male						
Lsm2										0.01082	-2.23363	Male						

Nenf										0.00824	-6.18776	Male				0.03197	4.20276	Female
Slc6a15													0.00179	3.25254	Female			
Vwa5b2													0.00000	-3.24677	Male			
Aldh3b2													0.04667	4.76169	Female			
Sp3							0.04676	2.66246	Female									
Tulp1													0.00420	-3.96768	Male			
Sae1										0.00952	1.47040	Female						
Nostrin																0.01028	4.56571	Female
Cln6				0.00001	-1.44129	Male												
Map1lc3a										0.03395	-2.24575	Male						
Coq9																0.03669	1.70207	Female
Ppie										0.00004	3.51426	Female						
Usmg5										0.00000	-6.27587	Male	0.00000	2.43228	Female			
Triap1										0.00000	2.50252	Female				0.00146	-1.45516	Male
Pga5													0.00000	0.59535	Female			
Luzp2	0.00378	2.06272	Female															
Sbspon													0.00000	0.87875	Female	0.00001	1.68069	Female
Gm26705																0.04728	2.86158	Female
Mllt10																0.03813	-1.77792	Male
Rpl37a										0.00000	-4.94562	Male	0.00007	1.66772	Female			
Kars										0.02699	1.38695	Female						
Chek2													0.00002	-1.94784	Male			
Nlrp5.ps																0.00002	-5.57155	Male
Gas2l3										0.00004	-3.22218	Male						
Obox3										0.00000	-6.00544	Male						
Slc27a3	0.00000	-0.86850	Male															
Lman1	0.03235	3.92329	Female															
Cnga4							0.00000	2.47854	Female	0.02455	-1.02196	Male				0.00000	3.07516	Female
Pomp										0.00214	-1.43809	Male				0.03900	-0.89547	Male
Slc28a3										0.02482	-2.03608	Male						
Panct2													0.00029	3.83548	Female			
Zbed6										0.00007	-1.58719	Male	0.01404	3.61985	Female			
Sfr1										0.01455	1.43411	Female						
Ppt2										0.00000	-4.25813	Male						
Hsf5	0.02826	-1.60465	Male	0.00045	0.77093	Female	0.01661	-1.24229	Male							0.00051	0.93400	Female
Ovgp1				0.00006	-1.29050	Male							0.00000	2.22655	Female	0.00000	-0.61114	Male
Aqp7										0.04511	-2.40388	Male						
Ankrd33b										0.00000	2.48665	Female				0.00989	5.15915	Female
Lmntd2	0.03022	0.84705	Female	0.00369	-0.92357	Male	0.00000	1.64169	Female									
Acin1										0.00003	3.96252	Female						
Gm13830													0.03553	2.53230	Female			
Slc7a11										0.00022	1.90444	Female						
Snx24													0.04667	4.38218	Female			
Art5																0.00000	-1.42034	Male
Smim6	0.00107	1.53167	Female	0.00050	1.12777	Female												
Spice1	0.00000	-2.25756	Male															
Epha10																0.00243	4.46207	Female
Ubtfl1										0.03831	2.78878	Female						
Lrrc56																0.02362	-4.44316	Male
Gm15910										0.00026	-3.75086	Male						
Nol4l																0.00000	-3.72824	Male
Tefm																0.00413	-2.05636	Male
Dkc1										0.00116	1.82677	Female						
Pou2f3													0.00000	5.51908	Female			
Brip1										0.00194	-3.12945	Male						
Dcxr	0.00000	-1.30118	Male	0.00000	-2.73201	Male												
B4galt1				0.00890	2.87878	Female												
Ftsj1										0.01103	1.81039	Female						
Ddx58																0.00682	4.21205	Female
Lemd3																0.02505	-2.95232	Male
Scpep1																0.00128	-1.59267	Male
BC005537										0.00796	2.02902	Female						
Atxn7l1				0.00000	-1.88714	Male				0.00000	3.05708	Female						
Ndufv1	0.03885	3.10800	Female															
Anapc16										0.00521	-2.66136	Male						
Tuba1b										0.00718	1.15306	Female	0.03851	0.70588	Female			
Cdc42ep3				0.00000	1.15353	Female												
Cyp11a1													0.00000	2.32993	Female			
Slc4a2				0.00000	-3.03052	Male												
Tspyl2																0.03871	-3.77955	Male

Gm20219				0.00860	0.90709	Female												
Cnot1										0.00007	1.75477	Female						
Rac2										0.00160	-3.12261	Male						
Armcx4							0.00458	2.74309	Female	0.00011	-3.83083	Male						
X2.Mar													0.03509	2.20107	Female			
Spint5							0.00000	1.48257	Female									
Tomm5										0.00395	-1.75876	Male						
Hey1				0.00008	0.92956	Female										0.00000	-0.79213	Male
Dexi				0.00002	0.87904	Female												
Fam195a										0.01737	-3.19217	Male						
Wdr70										0.03079	2.54026	Female						
Fthl17.ps1				0.00102	4.82321	Female	0.00000	5.62317	Female	0.00160	4.62315	Female	0.00001	4.51635	Female			
Hes1										0.00022	-5.23411	Male						
Cfap126										0.00000	0.80390	Female				0.00000	2.24337	Female
Rpl22										0.00000	-4.64774	Male	0.00462	1.13036	Female			
Dynll1										0.02502	-1.79867	Male						
Tnpo1										0.00181	-2.64507	Male						
Camk1							0.00327	-4.22619	Male									
Igfbp2																0.02210	3.37869	Female
Vstm5	0.00189	0.90527	Female	0.00024	0.76882	Female	0.00000	-2.10228	Male	0.00000	2.10076	Female						
Nbas				0.02618	1.65774	Female												
Nudt7																0.03239	4.04304	Female
Pxdn										0.00130	-0.89101	Male				0.02737	5.19587	Female
Ccdc153	0.00000	2.81209	Female															
Pinlyp																0.00000	-6.44535	Male
Strip2				0.00000	2.33596	Female				0.00000	-3.49614	Male						
Dimt1							0.00501	2.15820	Female				0.02665	-1.54768	Male	0.02448	1.72733	Female
Cldn3				0.00038	-2.30537	Male												
Stx11							0.02003	-0.65348	Male									
Cav1										0.00350	0.80670	Female						
Ctrl																0.00000	-5.97194	Male
St7l																0.00052	-3.34762	Male
Atp6v1c2																0.00000	2.24698	Female
Tpbgl										0.00647	-1.60929	Male						
Mixl1													0.00000	0.71854	Female			
Deb1										0.00000	-3.66688	Male	0.00174	1.82477	Female			
Pla2g10os										0.04656	-4.09930	Male						
Gm21284										0.00000	-4.40855	Male	0.01964	1.80147	Female			
Zfp939																0.00156	-3.95123	Male
Asb6										0.01012	3.02904	Female						
Hmox2										0.01769	2.04395	Female						
Epn1										0.00000	-4.18862	Male				0.02365	-2.04506	Male
Emc2				0.00000	4.74220	Female												
Nup93										0.02500	1.80426	Female						
Cxcl13							0.00123	-1.92795	Male									
Lrrc75a	0.00000	-1.23038	Male							0.00004	-2.27981	Male	0.01752	5.17048	Female	0.00731	-6.23560	Male
Rac3																0.03691	-3.34011	Male
Wscd2							0.01058	-4.75219	Male									
Il11										0.00000	-4.71839	Male						
Kank3				0.01113	2.31563	Female												
Bmp5													0.04780	3.08347	Female			
Sult5a1																0.04407	2.90208	Female
Sppl3										0.01241	1.86557	Female						
Lama4										0.00000	0.62362	Female						
Setd1b										0.03395	-2.40724	Male						
Gm7104													0.00004	4.62011	Female			
Bmp8a										0.03336	-0.61872	Male	0.00000	2.18008	Female			
Allc																0.00000	5.00333	Female
Ly6k							0.00001	-1.32892	Male									
Snrpf										0.00036	-3.83804	Male						
Got1										0.01130	1.48160	Female						
LOC105242579										0.00000	-3.20214	Male						
Cilp																0.00000	-5.89358	Male
Dgka																0.00158	-2.47985	Male
Tmem69										0.00000	-4.63047	Male	0.00011	1.95354	Female			
Park2							0.00282	2.65597	Female	0.00000	-5.26255	Male	0.02954	1.81805	Female			
Gm6578				0.03915	-2.45061	Male												
Cdx2							0.01758	3.23658	Female									
Klf10				0.00002	3.89391	Female												
Mrps21										0.00000	-4.20969	Male	0.00083	1.62450	Female			

Cdc37l1																0.03787	2.51442	Female
Abcg2	0.00017	-0.81850	Male	0.00359	-1.68703	Male												
Crebrf																0.00833	-2.69753	Male
Srd5a1																0.03851	-4.44724	Male
Gapvd1										0.02284	1.96451	Female						
Fau										0.00685	-2.52960	Male						
Ppme1										0.02797	2.91149	Female						
Gm5532													0.01370	3.91090	Female			
Ist1										0.03101	1.40844	Female						
Gm6639													0.00107	4.00160	Female			
Dgkq							0.03409	3.49218	Female									
Oxa1l										0.00445	2.23844	Female						
Syt2																0.00000	0.78795	Female
C2cd2										0.03959	2.90565	Female						
Scn10a										0.00435	1.75406	Female						
Nr1d1																0.00108	-4.87950	Male
Mt4							0.00000	1.70650	Female									
Igbp1										0.00032	2.17804	Female						
Oosp3										0.00000	-6.07396	Male	0.00000	-1.71503	Male			
Aqp8										0.00000	1.15814	Female						
Chchd2										0.00245	-3.46851	Male						
Usp49													0.00004	4.52866	Female			
Foxo3	0.00000	1.72744	Female															
Lta4h	0.00000	1.43437	Female															
Fut4				0.00160	2.05294	Female												
Mlf1										0.00030	-1.63185	Male						
Piezo1										0.00013	5.69843	Female						
Becn1										0.00087	2.48755	Female						
Dstyk										0.03007	-4.36990	Male						
Rbp7													0.00000	2.18818	Female			
Ppargc1b	0.00000	-1.06446	Male	0.00000	-0.85478	Male	0.00410	-5.06668	Male	0.00336	4.23538	Female						
Ecd																0.02194	-1.49308	Male
Polr2j										0.00000	-2.79546	Male	0.00115	1.51065	Female			
Gsto1				0.04153	-3.69096	Male												
Rhox5				0.00000	3.88092	Female				0.00008	6.67684	Female	0.00000	9.49075	Female	0.00069	5.32766	Female
Ptpru	0.02483	0.61282	Female	0.04107	-0.59342	Male	0.00000	-3.89379	Male									
Gfra1										0.00000	1.19665	Female						
Ppef1				0.03192	1.29326	Female							0.00000	1.49385	Female			
Cdh16										0.02208	-3.10598	Male						
Anxa7										0.00579	1.64726	Female						
Atg10																0.03517	3.51722	Female
Tmed9													0.00687	2.01872	Female			
Zxda										0.01123	-2.80236	Male	0.04588	1.81303	Female			
Bend6										0.00012	-2.00250	Male						
Rps20										0.00001	-3.32314	Male						
Eif2ak1																0.00722	2.57756	Female
Irf2bp2										0.00001	-2.32918	Male	0.00513	4.33741	Female			
Mrps16										0.00000	-3.68362	Male						
Mrpl33										0.00000	-4.99440	Male	0.00150	1.66089	Female			
Npas2										0.02443	3.83714	Female						
Lama5				0.02045	1.08620	Female												
Lypla1													0.00063	-1.54425	Male			
Pik3c2b													0.01565	2.41987	Female			
Csf2rb							0.00923	-3.33735	Male	0.00714	-4.12416	Male						
Oas1c																0.00061	-4.69713	Male
Rpl35										0.00000	-4.42468	Male	0.00009	1.68941	Female			
Tppp	0.00864	1.75536	Female															
H2.DMb1	0.00704	0.61217	Female															
Thap7										0.04931	3.02119	Female						
Serpinb9b										0.00000	7.48603	Female						
Cebpz										0.04886	1.49580	Female						
Arxes2							0.00000	5.42911	Female									
Dnajc19										0.04616	-2.45927	Male						
Dppa2													0.01593	-1.42796	Male			
Aebp1													0.00282	5.26652	Female			
Cdc42ep2																0.00000	-6.16491	Male
Pigb				0.00000	1.32919	Female				0.00365	4.60522	Female						
Ramp3																0.00000	-1.98852	Male
Crebbp										0.00003	5.39565	Female				0.00009	4.42639	Female
Cldn5										0.00016	-3.14020	Male						

Epha2				0.00409	-1.74487	Male												
Slc25a35	0.00039	-1.00358	Male															
Gtpbp6										0.00148	5.27272	Female						
Apof										0.02286	-2.43199	Male						
Syvn1										0.00999	3.24490	Female						
Lpar6				0.02383	2.18160	Female										0.00000	2.21832	Female
Ndufc1										0.00000	-4.77690	Male						
Spon1																0.00025	-0.66432	Male
Man2a1				0.02312	1.28992	Female												
Mfsd7a	0.00000	1.79902	Female													0.00000	0.75701	Female
Rpl3l																0.00000	-5.11366	Male
Add3										0.00418	4.43034	Female						
Fam13b										0.01556	3.56285	Female	0.01058	-3.19476	Male			
Tenm4													0.00000	-1.10583	Male	0.00001	2.41244	Female
Kcnf1																0.00288	-4.64910	Male
Polr2c										0.04132	1.49523	Female						
Rcsd1				0.00051	2.24596	Female							0.00000	-4.14851	Male			
Klhl26				0.00005	-1.67352	Male												
Smc1a							0.00009	1.88399	Female	0.03182	1.40640	Female						
Cgref1																0.02599	3.19102	Female
BC037704				0.00036	-0.95804	Male										0.00511	-5.08356	Male
Csnk1g2										0.02822	3.09926	Female						
BC147527													0.00817	4.22890	Female	0.03928	-4.01978	Male
Psg23										0.00268	-1.83911	Male	0.00391	1.31526	Female			
Slx1b										0.00007	-2.42070	Male						
Gm4926													0.01532	2.31778	Female			
Pclo										0.00082	-3.48015	Male						
Dsg2							0.00138	-3.69443	Male	0.00082	4.58233	Female						
Ccnk										0.00274	-2.50534	Male						
Gm4951										0.00001	-4.21917	Male						
Olfr376							0.00076	1.75127	Female									
Gm10653										0.00000	-3.69544	Male						
Cir1													0.01345	-1.48186	Male			
Klrb1a																0.00000	-1.96999	Male
Trpc5							0.02189	2.98681	Female	0.00256	-4.05305	Male				0.00869	-2.97799	Male
Gm12185										0.01838	-2.13212	Male						
Drg1																0.03082	0.92650	Female
Ccdc125										0.00226	-3.86045	Male						
Snrpd3										0.00001	-3.25298	Male						
Ildr1				0.00000	0.88255	Female				0.00000	-1.10746	Male						
Sac3d1										0.01279	-3.08078	Male						
BC026585										0.00577	3.36528	Female						
Slc22a15										0.00028	-3.43668	Male						
Gm38426													0.04089	4.98432	Female			
Ssrp1										0.02443	1.24185	Female						
Plce1										0.00038	-1.97574	Male				0.00000	0.97439	Female
Sema6d																0.01683	3.42067	Female
Pfn2				0.00000	1.17581	Female												
Zfp780b										0.02471	-2.57980	Male						
Rangap1										0.00000	1.98203	Female						
Alg9				0.00158	2.23354	Female												
Txnrd3							0.00000	2.38410	Female							0.00000	-2.76793	Male
Ubtf										0.00907	1.84734	Female						
Pemt																0.00001	-2.52029	Male
Cpn1										0.03079	2.57477	Female						
Paip1										0.04082	-3.18019	Male	0.02339	2.66658	Female			
Rhbdl3																0.00953	4.27003	Female
Gm20767																0.00201	-1.80713	Male
Gtf2h3										0.04547	2.18400	Female						
Ttc38										0.02171	-2.66422	Male						
Lsm7										0.00000	-4.35280	Male	0.02460	1.59792	Female			
Grb14				0.01450	1.03099	Female												
Crlf2										0.00239	-5.75280	Male						
Becn2																0.00042	-2.38116	Male
Akr1c21				0.00000	1.89946	Female												
Stat6				0.02801	1.00477	Female												
Zfp369										0.03697	3.18255	Female						
Hdac3										0.00006	5.49180	Female						
Atf7																0.01338	3.18792	Female
Ddr2																0.01859	3.12543	Female

Rps7										0.01270	-1.48589	Male						
Abhd11os										0.01450	-3.70864	Male						
Map7d2																0.04915	-2.66123	Male
Snw1							0.00690	2.48745	Female									
Nfatc4				0.00067	-2.12179	Male												
Ndst2	0.00648	0.60452	Female															
Trpa1	0.03782	-1.20857	Male															
Frrs1l				0.00000	-1.54698	Male	0.00000	4.15232	Female									
Aamp										0.00066	2.12928	Female						
Setdb1																0.00657	-1.82374	Male
Dip2a				0.00000	0.70064	Female				0.00000	-3.46933	Male						
Qars										0.01170	1.22773	Female						
Krt27																0.00000	-5.19515	Male
Pcbp4													0.01696	4.29701	Female			
Pelp1										0.00435	-2.77741	Male						
Trim54				0.00020	-0.87459	Male												
Fam83a										0.02417	-2.98575	Male						
Pgam5										0.03024	1.82241	Female						
Zfp558																0.03584	-3.59100	Male
Tpgs2										0.00541	-3.95932	Male						
Mien1	0.00183	4.23268	Female															
Atp5j										0.00000	-4.45589	Male	0.00003	1.87595	Female			
Rrn3																0.00624	1.21864	Female
Hprt							0.00758	1.04152	Female							0.00867	-0.95857	Male
Pdcd5										0.00000	-4.83402	Male	0.04163	1.68206	Female			
Tcerg1l							0.00000	-4.40469	Male	0.04469	-0.71447	Male						
Arsk										0.00000	-5.16665	Male						
Hoxb13	0.00000	0.80072	Female				0.00000	-2.75247	Male	0.00006	-2.20853	Male						
Nr1h4																0.00726	1.72170	Female
Exd2																0.00939	2.68469	Female
Vamp8										0.02724	-1.72446	Male				0.04915	-1.34550	Male
Maged1										0.00025	3.92877	Female						
Plekhh2																0.00319	4.02414	Female
Sugct							0.00310	-0.81189	Male							0.00000	1.29983	Female
Cfap45							0.00000	-1.55985	Male	0.00002	-2.45608	Male						
Erf										0.00149	-4.08838	Male						
Snora41																0.02218	-3.50912	Male
Cxcr2										0.00000	-5.89698	Male	0.00047	1.80264	Female			
Aptx																0.00495	-2.11171	Male
Luc7l2										0.01320	-1.85144	Male						
Vsig10													0.00210	3.47654	Female			
Mmgt2																0.01375	-3.81120	Male
Cited4										0.00000	-2.87001	Male						
Lars																0.00879	1.65669	Female
Meg3																0.03944	3.89913	Female
Shb																0.00158	-5.23087	Male
Bloc1s3										0.00098	-2.57108	Male						
Ccnb1ip1																0.00128	-2.65474	Male
Gm715										0.03219	-1.67585	Male						
Anxa8				0.00001	1.02642	Female										0.00500	-5.47841	Male
Nudt4										0.00469	-1.36078	Male						
Rcn1										0.00000	-0.68456	Male						
Lgals8																0.04753	-1.35893	Male
Cdkl3													0.01462	-4.03064	Male			
Sap130																0.00061	-2.51142	Male
Cggbp1							0.00660	1.57221	Female									
Sbds										0.01956	1.73401	Female						
Ephx1										0.00180	4.80061	Female						
Ndufa4										0.00002	-3.04015	Male	0.00016	1.96678	Female			
Ndufs4										0.00406	-2.84793	Male						
Prkaca										0.02669	2.99614	Female						
Dnajc5g																0.00000	4.41147	Female
Fscn1										0.00031	-4.40478	Male						
Snord99																0.00008	5.16779	Female
Pank1							0.00536	4.21528	Female	0.00006	-5.93855	Male						
Tmem8b													0.00049	4.35212	Female	0.00493	4.27606	Female
Ccdc166																0.01602	3.57115	Female
Trappc9																0.00467	-1.91552	Male
Cd300ld													0.00000	3.92333	Female	0.00000	2.20261	Female
Suclg1										0.03181	1.63126	Female						

Pramef6										0.00000	5.40959	Female				0.00392	-2.12604	Male
Ccar1																0.01328	-1.45481	Male
Taf1c										0.00004	4.63672	Female						
Pygb				0.00708	2.26033	Female												
Spta1							0.00205	-3.42268	Male									
Cand2										0.01891	-3.09655	Male						
Zfp36l1				0.00000	-1.44469	Male				0.00000	-0.98902	Male						
Tnrc6b													0.00246	3.31116	Female			
Mageb16							0.02682	2.14330	Female									
Sugp2													0.01524	3.54623	Female	0.00450	-4.36846	Male
Gm6787																0.02167	-4.40426	Male
Osr2				0.00075	-1.75695	Male												
Kank2										0.03913	-2.79648	Male						
Nptn																0.01255	3.03769	Female
Gm1995													0.00146	2.97419	Female			
Drg2										0.00779	1.59836	Female						
Egflam	0.01459	-1.58323	Male															
Dlgap4										0.03336	2.76088	Female						
Yars										0.04371	1.33569	Female						
Tmcc3													0.03361	-3.39911	Male			
Col1a1				0.00204	1.23360	Female												
Dynlt1a										0.01578	-1.44092	Male						
Acsl4																0.00000	4.18666	Female
Ddx47										0.01187	1.81961	Female						
Ikbkb				0.00000	-0.81737	Male												
Pde4d										0.00000	-3.38018	Male	0.00000	-2.16944	Male			
Plekhg1							0.03241	-4.98447	Male									
Gm14461										0.00959	-1.66893	Male						
Cept1				0.00038	3.65240	Female	0.00055	3.56324	Female									
Ppil3	0.01009	4.02737	Female															
Suox										0.03049	3.47198	Female						
Lca5				0.00629	0.68271	Female												
Nup98							0.00977	1.82830	Female	0.00097	-2.48206	Male						
Atp2b1										0.00057	3.81175	Female						
Fam168b										0.00932	-2.01187	Male						
Sema3e										0.00000	-4.32210	Male						
Tnpo2							0.00859	2.65683	Female	0.00223	-3.25722	Male						
Gstm4	0.00002	1.28398	Female				0.00000	2.46600	Female									
Nkiras2				0.00157	3.22173	Female				0.00193	4.23435	Female						
Tmem241										0.00001	1.45185	Female						
Pet2																0.00000	1.01367	Female
Lrrc8e													0.03894	2.29135	Female			
Gje1										0.00000	-4.15108	Male	0.00233	1.97293	Female			
Glis3										0.00000	-3.22576	Male				0.02740	4.05061	Female
Ctrc				0.00393	1.18719	Female												
Pdcd11										0.00003	4.01528	Female						
Zfp626										0.00374	-2.73787	Male						
Gpcpd1													0.02016	-1.47116	Male			
Tpt1										0.00000	-3.82438	Male						
Slc28a1										0.00858	-0.66626	Male				0.00000	2.98817	Female
Rps18										0.00000	-3.04460	Male	0.01374	1.37220	Female			
Hnrnpl										0.01272	-3.11222	Male						
Gm16381							0.03060	3.97280	Female									
Alms1										0.03395	3.27240	Female						
Adamts2										0.00516	-1.70951	Male						
Rhobtb1																0.00000	-1.38595	Male
Sdcbp2																0.00013	5.84344	Female
Cnih4										0.00000	-2.97270	Male						
Slc35e2										0.00002	-3.09854	Male						
Gm15401										0.00000	-4.34968	Male						
Pde6g																0.04266	4.18954	Female
Kcnip2				0.00488	1.68210	Female												
Ccdc69										0.01349	-4.67735	Male						
Fgd1										0.00000	2.79776	Female						
Sub1										0.00000	-3.07392	Male						
Hdlbp										0.00987	1.45183	Female						
Efcc1							0.02250	2.92162	Female	0.00000	-6.14459	Male	0.04445	2.28480	Female			
Trim63							0.00001	1.02027	Female									
Ndufa2										0.00000	-4.33894	Male						
Spats2										0.00009	5.17020	Female						

AF067063																0.00000	-6.62289	Male
Lilrb4a							0.00030	-3.76468	Male									
Car2													0.00017	-0.62322	Male	0.00000	2.73369	Female
Spib										0.03679	-3.63404	Male						
Rnf168										0.00001	-3.13258	Male						
Rpl21										0.00000	-3.25276	Male	0.01508	1.15197	Female			
Grin3b	0.03154	-0.80980	Male	0.00004	0.73906	Female												
Gjb4				0.00001	-0.71737	Male										0.00976	5.43551	Female
Fastkd1							0.02336	3.25792	Female									
Xrcc3				0.01744	1.39852	Female												
Tex13	0.03830	2.88427	Female															
Gsdma3																0.00627	-4.73217	Male
Glrx										0.00221	3.86654	Female						
Mrps18a	0.00010	5.24422	Female															
Cct6a										0.00196	1.03032	Female						
Morf4l1										0.02858	1.36930	Female						
Xlr3c							0.00776	3.06706	Female									
Gm13985										0.00000	-3.45842	Male						
Snrpd2										0.00000	-3.80067	Male	0.04168	1.34645	Female			
Slc25a18	0.00003	-2.05964	Male										0.00000	-1.36917	Male	0.00000	-5.49529	Male
Slfn5										0.00000	-5.96726	Male	0.00243	1.77773	Female			
Smox				0.00003	-1.00315	Male												
Entpd8							0.00058	-3.76872	Male									
Cyct										0.02640	-4.67464	Male						
Aatf										0.00009	3.03963	Female						
Gm5069										0.03057	1.77885	Female						
Meis1																0.04462	4.30125	Female
Rps23										0.00000	-3.76844	Male	0.00111	1.51454	Female			
Gpr137b.ps																0.00001	-1.93522	Male
Slc22a13										0.00004	5.02624	Female						
Rgl1				0.00007	0.85339	Female												
Pramel4																0.02730	-1.25203	Male
Dusp23							0.00000	3.30782	Female				0.00000	2.75765	Female			
Cap1				0.00168	3.98405	Female												
Cct7										0.00045	1.78712	Female						
Gm5803										0.00168	2.83809	Female						
Lcn10										0.00000	2.27022	Female						
Ppm1m										0.00000	2.52568	Female						
Pdia4										0.00100	4.15872	Female						
Gm595													0.00000	-2.77472	Male			
Slain1													0.00000	-6.20980	Male			
Pld6										0.03171	-4.31244	Male				0.00000	-2.07344	Male
Psmb10										0.01716	-2.92613	Male						
Calm3										0.04126	-2.17355	Male						
Alms1.ps2										0.03124	3.15169	Female						
Wfdc3				0.00019	2.06799	Female							0.01012	-5.17504	Male			
Akr1b10				0.03438	3.51429	Female	0.00055	-4.47978	Male	0.01819	3.87859	Female						
Rnf223																0.01632	-4.85776	Male
Dync1i1	0.03296	-0.89756	Male							0.01499	-0.78828	Male						
Dmgdh																0.00991	-1.68089	Male
Krba1							0.04109	3.46580	Female									
Mael																0.00000	1.14887	Female
Sh3pxd2a																0.00320	4.18602	Female
AI427809							0.00000	-0.76242	Male									
Adamtsl4																0.02904	3.43995	Female
Nolc1										0.00027	1.69937	Female						
Gm20187																0.01627	3.96498	Female
Gm4285							0.00931	3.01540	Female									
H2.D1																0.02241	2.21061	Female
Gm3286																0.00000	-2.45340	Male
Bnip2																0.00576	-3.60210	Male
Oxld1																0.02122	4.06204	Female
Gm2381							0.04247	1.91750	Female									
Nobox													0.00000	-3.82514	Male			
Slc38a2																0.00044	5.62594	Female
Ssb										0.00100	2.03541	Female						
Eif5a2										0.00000	-4.36485	Male						
Lysmd2				0.00065	-1.65282	Male												
Agl										0.00667	-2.61247	Male						
Gm12253										0.00205	-3.80249	Male						

Prox2										0.00011	-5.39193	Male						
Zfp979				0.02602	3.01449	Female												
Rps27l										0.00000	-4.53986	Male	0.00135	1.64140	Female			
Tma7										0.00000	-3.48247	Male	0.00074	1.34941	Female			
Emc3																0.02614	-1.06430	Male
Abca3										0.00180	4.13020	Female						
Slc11a1													0.00232	2.80825	Female			
Nifk										0.02199	1.13750	Female						
B3gnt8				0.00000	-1.93018	Male												
Tpd52l1				0.00004	1.53937	Female												
Pilra										0.00000	-4.82687	Male	0.00641	2.45493	Female			
Rpl23a										0.00000	-3.15994	Male	0.03135	1.11879	Female			
Lyrm1																0.01484	-3.07913	Male
Lca5l																0.00008	0.79494	Female
Rpa2										0.00318	1.77353	Female						
Erap1													0.00000	3.29262	Female			
Ngfrap1										0.00000	3.67500	Female				0.00236	5.14965	Female
Trim23										0.00295	4.50757	Female						
Samd10							0.00000	-2.01505	Male				0.00000	-4.16175	Male			
Atp1a2										0.01704	-3.65428	Male						
Traf5										0.00147	3.19967	Female						
Majin	0.00000	0.81113	Female															
Gm14124																0.00108	-3.03951	Male
Letm2																0.00000	-1.59762	Male
Tpp1										0.03400	2.82323	Female						
Acsm3										0.04256	2.89184	Female						
Mrpl18										0.01835	1.32276	Female						
Zhx3													0.00004	5.30071	Female			
Ufsp1				0.00021	-1.35551	Male												
Ipo13										0.03336	2.73876	Female						
Prkar2a										0.02908	2.28472	Female						
Mcm5										0.01515	1.58608	Female						
Tbc1d16	0.00000	-1.12391	Male							0.00000	-3.51596	Male						
Fgf1																0.00000	-0.90159	Male
Cecr2													0.04089	-1.06656	Male			
Fancb				0.04674	2.20331	Female										0.00010	5.18589	Female
Maged2										0.00000	3.24126	Female	0.01721	-4.17004	Male			
Cep162																0.00283	4.65000	Female
Usp20																0.03669	-3.51135	Male
Csrp1				0.00379	2.76581	Female												
Fam49a										0.00004	-2.27914	Male	0.00000	4.65364	Female	0.00000	-1.50144	Male
Tbl1x							0.04071	2.27074	Female									
Kif20a										0.00000	6.22460	Female						
Glg1										0.00000	6.59347	Female						
Abcd3	0.01299	4.37850	Female	0.00071	3.74975	Female												
Clec2f	0.00027	-0.82119	Male	0.02226	-1.58701	Male												
Ppm1k										0.00000	-4.65320	Male						
Rab11a										0.00052	-2.41626	Male						
Tcf19										0.01562	3.25453	Female						
Dguok				0.00686	1.69184	Female												
Gm6086													0.03361	2.63050	Female			
Spp1	0.00000	-2.25428	Male							0.00000	1.52746	Female						
Zfp575										0.01538	-1.82720	Male						
Asz1				0.02045	3.35625	Female												
Hnrnpul1										0.00001	5.73036	Female						
Slc15a1										0.04082	-2.17573	Male						
Trim61										0.00917	-4.45364	Male	0.00005	5.30588	Female			
Timd2																0.00942	-1.62738	Male
Mmp2										0.01300	-0.76343	Male	0.00000	1.70071	Female			
Nuak1				0.00005	-1.31242	Male				0.00000	2.74564	Female				0.03170	4.33680	Female
Gm10324							0.00923	1.83467	Female									
Gnpda1													0.01508	-1.38985	Male			
Ifitm6				0.00316	1.70528	Female				0.01673	-1.11959	Male						
Card6																0.03851	2.94473	Female
Zfp454	0.00003	0.74002	Female													0.00000	-0.59083	Male
Gm4925										0.02224	-2.43293	Male						
Klhdc1										0.00000	2.42782	Female				0.00898	-4.95814	Male
Nxf7													0.00000	2.69711	Female			
Psmb2										0.00000	2.51841	Female						
Mrps18c										0.00000	-4.94894	Male						

Xcr1													0.00624	2.90197	Female			
Rps27a										0.00000	-3.06589	Male						
Eif3b										0.01412	1.88446	Female						
Fez1													0.00000	6.08447	Female			
Ccdc173																0.00002	-5.77860	Male
Dhx32										0.00747	2.06286	Female						
Brms1l																0.01362	-2.20357	Male
Traf4										0.03852	2.22521	Female						
Exd1																0.00052	-3.74828	Male
Tktl1																0.00010	3.59931	Female
Dyx1c1	0.00373	-0.97927	Male				0.00000	4.38638	Female							0.00000	-1.91906	Male
Atp5g1										0.00115	-2.27051	Male	0.02626	1.28185	Female			
Cela2a				0.00837	-1.85800	Male				0.00000	2.72644	Female						
Cul7										0.00311	3.75284	Female						
Srrm1										0.01141	1.39936	Female						
Sat1							0.00075	2.23345	Female	0.01679	2.00360	Female						
Tcstv1																0.00052	-5.32606	Male
Ankrd44																0.00000	-5.90767	Male
Gjb5										0.00001	6.03737	Female						
Snord16a										0.00481	3.36172	Female						
Arpc3													0.02291	-1.67526	Male			
Reln													0.00621	-4.84052	Male			
Bex4										0.04637	4.47237	Female	0.02010	4.42274	Female			
Gmeb1										0.00445	-2.14209	Male						
Dennd6a										0.04087	-2.07263	Male						
Atox1										0.00000	-4.69788	Male	0.00006	2.15364	Female			
Cnbd2																0.02239	-1.36025	Male
Arfgef3							0.00005	-2.16362	Male									
Gm9776							0.01253	2.02120	Female	0.00000	-5.51205	Male	0.00128	1.99915	Female			
Rhou													0.02251	3.55660	Female			
Usp44																0.00002	1.88065	Female
Tmsb10										0.00005	-4.06328	Male						
Ctdp1										0.00897	3.28898	Female						
Adk										0.02978	2.77251	Female						
Ddx24										0.02985	1.38881	Female						
Icam2										0.00164	-1.42854	Male						
Snx5													0.01628	-0.94129	Male			
Dcbld1																0.03311	-1.80527	Male
Ccdc43				0.00006	4.22222	Female	0.02595	1.96501	Female									
Nt5dc3										0.00000	-5.27847	Male						
Camkmt				0.00107	-2.00837	Male												
Sft2d2	0.00003	0.74962	Female	0.00000	-1.63808	Male	0.02243	3.76835	Female	0.00001	-1.32607	Male						
Apom																0.00076	4.40154	Female
Fam210b				0.00000	1.46729	Female				0.00000	1.50598	Female						
Sod1													0.01443	1.42666	Female			
Israa										0.00041	-1.94928	Male						
Hcn3													0.00001	-5.60213	Male	0.00009	-5.64545	Male
Rhoh										0.01354	-3.49833	Male						
Gcn1l1										0.00460	3.62354	Female						
Ints3										0.01608	1.84966	Female						
Nlrp1a										0.02251	-1.44681	Male						
Pisd.ps2										0.00341	3.47092	Female						
Rmi2										0.00000	-5.05188	Male	0.00170	1.46204	Female			
Pet100										0.00000	-5.90432	Male						
Abhd11										0.01470	2.47652	Female						
Gm15800										0.04276	2.46456	Female						
Ccdc39										0.01062	1.03579	Female	0.00000	-2.10066	Male	0.00000	-1.37862	Male
Setd7	0.00001	2.25463	Female	0.00050	-0.76672	Male												
Pyy				0.00002	-1.05680	Male												
Dnajc2																0.00500	-1.26703	Male
BC025920																0.00002	-0.82343	Male
Fam3b	0.00000	0.84432	Female							0.00000	-4.08393	Male				0.00000	3.22624	Female
Cdk5rap2				0.00594	3.18650	Female												
Maea										0.04414	1.95566	Female						
Wdr81																0.00106	-2.97805	Male
Wnt10b													0.02604	4.27035	Female			
Sh3gl3										0.01134	-1.20849	Male	0.00000	3.13272	Female			
Reps2																0.00009	-4.53765	Male
Ptpn7										0.00001	-3.55859	Male						
Etohd2				0.00006	0.90426	Female				0.00000	1.42641	Female				0.00000	-5.07336	Male

Vnn1																0.04842	-1.81991	Male
Dbhos													0.00557	1.98203	Female			
Slc6a14				0.00000	0.89453	Female												
Pla2g10				0.04789	2.13078	Female												
Kif2a										0.03165	1.63729	Female						
Bzw2										0.01138	1.48701	Female						
Dqx1																0.00284	-2.52732	Male
Dync2li1													0.00000	-1.68844	Male			
Ppp3ca										0.03336	3.45019	Female						
Ube2e2				0.00161	1.13146	Female												
H2.Q4										0.00581	3.11406	Female						
Srsf2										0.04947	-1.05991	Male						
Wnt4										0.00011	-3.18384	Male						
Arhgap20os							0.03559	2.96821	Female	0.04422	-3.03894	Male						
Rpl30										0.01027	-2.27568	Male						
Ptov1										0.01272	3.45343	Female						
Syna													0.00000	2.92116	Female	0.00000	-2.90164	Male
Gm8267										0.00000	2.26521	Female						
Prom2																0.00556	-4.79518	Male
Mmp23				0.03735	-1.12237	Male												
Nr0b1							0.00054	-0.74640	Male									
Derl3																0.00656	4.21465	Female
Suz12													0.02031	-1.06911	Male			
Unc45b													0.00000	-5.86486	Male			
Zkscan6							0.03513	3.40623	Female									
Nob1										0.01272	1.61532	Female						
Kat2a										0.03679	1.68708	Female						
Cercam										0.04203	-2.55875	Male						
Polr2k										0.00000	-6.52237	Male	0.02954	1.44749	Female			
Ntf5																0.00212	4.26235	Female
Ptpn22																0.04004	-3.23470	Male
Snord58b																0.00622	-4.32357	Male
Stard10										0.00030	4.08282	Female						
Eapp																0.00288	-1.42930	Male
Stab1				0.02363	1.54505	Female							0.00000	-0.67503	Male			
Chad							0.00003	-2.49841	Male									
Mir17hg	0.00000	-1.82324	Male															
Kcnk6										0.01515	-2.31145	Male						
Qrich1										0.00121	3.20264	Female						
Slco3a1													0.02999	4.54186	Female			
Smad9										0.02415	-3.53911	Male	0.00094	3.69481	Female			
Adgrg1																0.02908	-3.23430	Male
Wdr35										0.00001	-2.62012	Male						
Shbg													0.01819	-3.36196	Male			
Lrig3													0.00000	-2.85424	Male	0.00000	1.21828	Female
Khdc1c										0.00086	-3.54969	Male						
Ogdh										0.01744	-1.24162	Male						
Snord96a										0.04388	3.01886	Female						
Mex3c							0.02709	2.14658	Female									
Hnrnpk										0.03815	1.04135	Female						
Rpl31										0.00000	-4.60588	Male	0.00004	1.66310	Female			
Arl5b																0.00829	4.26555	Female
Ankrd23																0.03018	2.55817	Female
Nrp2																0.01992	3.92677	Female
Myh11				0.00002	2.69886	Female							0.00000	-4.78778	Male			
Oog3													0.03377	-3.25092	Male	0.00226	-4.54538	Male
Iqcf1																0.00000	-3.09290	Male
Arid3b										0.03209	2.64150	Female						
Cops5	0.02389	1.91064	Female															
Gdi1				0.00695	3.59819	Female												
Slc14a1													0.00004	-1.13475	Male			
Plxnb3										0.04058	-0.89757	Male						
Uba3													0.01299	-1.99141	Male			
Tnfsf10										0.00000	-4.40707	Male	0.03509	1.32469	Female			
Eif3d										0.00509	1.41048	Female						
Gadd45g				0.00026	-2.42934	Male												
Nek7										0.02049	-2.08214	Male						
Trim40													0.00000	3.18120	Female			
St8sia1																0.02096	-3.32782	Male
Adamtsl5				0.00006	-1.39929	Male												

Mcm6																0.01138	1.12298	Female
Il2rg																0.00284	4.10068	Female
Phlpp1				0.00158	-1.41952	Male												
Mmp12							0.00000	-0.74192	Male	0.00000	1.93204	Female						
Bag1																0.02505	-1.55804	Male
Chchd1										0.00052	-2.54436	Male						
Polg										0.01380	2.56893	Female						
Psma5										0.01409	1.33851	Female						
Ctcfl										0.00000	-4.00540	Male	0.04793	1.36232	Female			
Psen2																0.00391	5.24762	Female
Ak3													0.00000	1.47143	Female			
Dmkn				0.00783	-1.72971	Male												
Brcc3							0.00923	3.28827	Female									
Stip1										0.00102	1.69009	Female				0.01641	-1.09663	Male
Hist3h2a				0.00019	-1.98842	Male				0.03379	4.13176	Female						
Irf7													0.03886	4.47386	Female			
Zkscan7				0.02033	1.87371	Female	0.00000	-1.18880	Male	0.00045	0.60829	Female						
Glmp																0.00538	-1.49244	Male
Igip	0.00001	1.46287	Female							0.00000	1.82153	Female						
Zar1l																0.02374	-4.63327	Male
Gm13102																0.03227	-1.21545	Male
C1qbp										0.00003	-2.25761	Male						
Fbxo44													0.00237	4.82520	Female			
Gigyf2																0.03731	1.85452	Female
Map2																0.00000	1.14212	Female
Cks2										0.00000	-3.18967	Male						
Mrps33										0.02272	-1.83883	Male						
Cdc42bpb										0.00027	4.48326	Female						
Tceb2										0.00000	-4.20991	Male	0.00336	1.93327	Female			
Xrcc6bp1										0.03987	-3.81136	Male						
Mfap3l							0.00000	-1.33514	Male									
Gin1				0.00991	3.93150	Female												
Havcr2							0.01411	-4.23472	Male									
Vdr										0.00003	2.65214	Female				0.00000	-3.68265	Male
Iscu										0.00454	-2.79321	Male						
Def6				0.00001	-0.74564	Male												
Sh3bp2																0.00183	-4.99613	Male
Rab3b																0.00633	4.35163	Female
Mb21d1										0.01557	-2.80457	Male	0.02209	2.03104	Female			
Adarb2										0.03149	-1.43907	Male						
Ctrcos										0.00013	-4.20084	Male						
Arhgdib													0.01722	4.21439	Female			
Cd47				0.00000	2.87802	Female				0.00001	-2.40641	Male				0.02592	4.61323	Female
Ifi35																0.04462	2.45250	Female
Dr1																0.00688	-2.14765	Male
Kif3c																0.00958	-3.47806	Male
Mov10l1																0.02170	3.22429	Female
Ccdc112				0.00005	1.58545	Female	0.00000	1.45238	Female									
Abca1	0.00057	0.96321	Female															
Oas1e																0.00012	-5.70665	Male
Rbx1										0.00000	-3.33912	Male	0.01921	0.94558	Female			
Dnajc5													0.01436	1.42851	Female			
Eepd1							0.00000	1.86210	Female									
Hist1h2bc				0.00001	-1.23866	Male												
S100a10				0.00974	-5.79702	Male												
Thop1										0.01000	3.93179	Female						
Ddx31																0.02986	2.26850	Female
P4htm	0.00000	3.60077	Female															
Gpr3										0.04612	-3.44534	Male	0.00452	-3.77916	Male			
Invs										0.00235	1.41843	Female						
Kat6a										0.00027	-3.97711	Male						
Tsr2										0.00000	3.30587	Female						
Ccdc13	0.00039	-0.86631	Male	0.00040	-1.06658	Male												
Glrx3										0.02822	-1.31556	Male						
Mylip							0.03368	-4.64797	Male									
Zfp385c										0.00000	-4.03071	Male						
Akr1c14																0.00000	-2.10979	Male
Gja4																0.00000	3.58707	Female
Nudcd3				0.00095	3.50818	Female												
Hk2										0.00571	3.72535	Female						

Agpat5													0.01841	-1.33957	Male			
Ifnar1										0.00000	-3.06188	Male				0.04682	-0.94531	Male
Higd1a										0.00000	-3.98451	Male	0.02066	1.62645	Female			
Lcor				0.00000	0.75528	Female												
Tspan3										0.00015	5.22592	Female						
Arglu1													0.00082	1.51890	Female			
Iars2													0.02961	-2.09164	Male			
Mfsd11										0.00056	-3.08063	Male						
Gata6	0.00275	-3.66339	Male															
Efhc2							0.00000	2.12247	Female	0.00140	0.88358	Female						
Txnip																0.00985	4.42884	Female
Zfp811										0.00001	-0.78609	Male						
Olfr1055										0.01625	-1.81632	Male						
Rps25										0.00000	-4.87860	Male	0.00024	1.49045	Female			
Trpc1													0.04050	2.01060	Female			
LOC101056073										0.01624	2.26273	Female						
Ncan													0.00036	3.73096	Female			
Pcgf5																0.00753	-2.45065	Male
H6pd	0.00997	-1.22959	Male	0.00078	-1.03715	Male				0.03208	-1.13141	Male						
Hint1										0.00548	-2.75429	Male						
Dpy30										0.03509	-1.56887	Male						
Id3																0.03547	2.80959	Female
Lat2																0.00186	-4.46558	Male
Zfp708																0.03197	-3.54174	Male
Mcc										0.00002	-3.57782	Male	0.01954	1.65307	Female			
Xkrx							0.01527	3.75375	Female									
Chd4										0.02251	2.40810	Female						
Spry4																0.00071	4.60284	Female
Ptgfrn				0.00000	1.27481	Female				0.00000	4.93986	Female						
Pard6a				0.01192	1.68638	Female												
Blcap																0.01775	-2.39619	Male
Cct3										0.00001	1.32523	Female						
Traf2										0.00171	3.30811	Female						
Gucy2e																0.00000	-7.17421	Male
P2rx4																0.00054	-3.43979	Male
Dancr										0.00113	-4.32362	Male						
Fam83b	0.04038	-1.54187	Male							0.00002	2.73556	Female	0.00000	-4.93788	Male			
Pars2				0.00000	1.96708	Female												
Tomm6										0.00000	-3.68340	Male	0.01139	1.44257	Female			
Gm14325																0.00422	-1.66483	Male
Ankrd13d				0.01037	-0.70707	Male												
Prps1				0.01459	1.69731	Female	0.01436	1.03678	Female									
Pfkfb4																0.04434	-3.44480	Male
Sergef																0.03813	4.08947	Female
Atp5j2										0.00000	-4.78137	Male	0.00004	1.81533	Female			
Mylpf										0.00017	-2.87654	Male						
Apcdd1				0.00000	1.16853	Female												
Sspn										0.00008	-2.04368	Male						
Msh5										0.00000	1.21040	Female						
Rpl31.ps12										0.00000	-5.44792	Male	0.00002	1.73436	Female			
Slc7a14										0.00000	-4.90548	Male	0.00155	1.57255	Female			
Bex6																0.03731	-2.88197	Male
Tbc1d24										0.00930	-2.81502	Male						
Vangl2													0.03610	3.86527	Female			
Pgap1				0.00001	2.21872	Female												
AV320801	0.02369	1.26054	Female	0.00002	3.35756	Female	0.00001	0.81469	Female	0.03820	-0.77111	Male				0.00000	-4.66968	Male
Tmem88b										0.00033	-4.40472	Male						
Sf3b2										0.04779	1.21119	Female						
Uxt										0.00102	-2.52630	Male						
Zfyve19										0.01441	3.12743	Female						
Dync1li1										0.03867	1.94430	Female						
Mib1													0.00893	-3.53429	Male			
Gm6607										0.00717	-4.38655	Male						
Rpl17										0.00001	-2.19022	Male						
Nubp2										0.01533	1.60505	Female						
Smap1										0.00000	6.05170	Female						
Zbtb20													0.00000	3.11167	Female	0.00012	1.02435	Female
Ldlrad2										0.00000	-3.81941	Male						
Fgf3													0.00008	-4.93099	Male			
AI661453										0.00299	-3.49174	Male	0.00983	2.41490	Female			

Mrps15										0.00080	-3.57996	Male						
Ccdc64b										0.00000	3.23201	Female						
Eif2ak4							0.00114	-4.36553	Male									
Il1f9													0.00173	2.83813	Female	0.00032	-3.63694	Male
Klf5										0.00000	5.62447	Female						
Mettl1										0.04779	2.20582	Female						
Mfap1b										0.00001	3.58696	Female						
Rspo2										0.00000	-3.84181	Male				0.00000	-1.42865	Male
Srpr																0.04485	-1.21002	Male
Pdgfa							0.00000	5.35796	Female									
Ddx19b																0.00002	-2.64823	Male
Jakmip3							0.00013	-3.20477	Male									
Per2																0.00002	-0.68163	Male
Gm13752																0.00000	-3.58289	Male
Slc29a3																0.00500	-3.26699	Male
Ppm1j										0.00000	2.67258	Female						
Col7a1				0.00006	-0.89479	Male												
Arf5										0.00631	4.33166	Female						
Rph3a							0.01483	-3.32532	Male									
Cd68							0.00332	4.64384	Female							0.00102	4.85894	Female
Cyb5rl										0.00000	1.57475	Female						
Sec1																0.00450	3.79439	Female
Duoxa2							0.01182	-1.61896	Male	0.00219	0.73871	Female						
Dusp26																0.00898	-4.43959	Male
Churc1										0.00000	-4.38725	Male						
Fam214a																0.00000	-5.32480	Male
Flrt3																0.01239	5.99879	Female
Mbtps2										0.01283	3.08970	Female						
Mospd2							0.00000	6.39885	Female									
Thap2																0.02241	-3.20003	Male
Ccser1							0.00000	-1.46649	Male	0.00044	-0.94798	Male						
Igsf23																0.00001	-2.47133	Male
Ccdc42													0.02406	5.21441	Female			
Gpr143							0.00000	3.24329	Female									
Slc12a9							0.04294	3.25771	Female									
A4galt										0.02165	-2.36061	Male						
Skp1a										0.04300	-1.23642	Male						
Sox15																0.00443	-2.77751	Male
Fsbp																0.01450	-4.46533	Male
Rasa2										0.00741	-4.44420	Male						
Kdelc2										0.00000	-4.84028	Male						
Rpl23										0.00000	-3.38527	Male	0.00095	1.20203	Female			
Dock1				0.00000	-0.93773	Male												
Ctps2																0.00117	3.75980	Female
Nop16										0.01535	1.63619	Female						
Slc1a7										0.00076	-2.92262	Male	0.03191	1.63568	Female			
Sirt5																0.00167	-4.74592	Male
Fsd2				0.00043	0.90387	Female				0.00000	1.58447	Female						
Wdr19																0.02988	4.54690	Female
Akr1d1										0.01468	-3.93154	Male						
Crygc							0.00000	-2.61036	Male									
Hint2										0.00935	-4.66299	Male						
Padi3							0.00076	-1.74728	Male							0.00000	3.45493	Female
Mtx1										0.02048	2.84638	Female						
Gmfg										0.00025	-6.80773	Male						
Pi16													0.00000	3.82566	Female	0.00000	1.36971	Female
Dopey2																0.04890	3.36979	Female
Mdp1																0.02238	-1.96556	Male
Mpv17l										0.00000	-5.06489	Male	0.00377	1.70889	Female			
Fbxo27	0.00003	1.14463	Female															
Spryd4				0.04422	2.97750	Female												
Ahsa1										0.00940	1.49131	Female						
Cxxc1																0.02986	-1.88796	Male
Rgs10																0.00000	-4.86897	Male
Cgrrf1	0.00000	-0.96662	Male															
Zfp605							0.00756	2.75043	Female	0.01766	-2.81267	Male				0.02122	-2.36126	Male
Eny2										0.00000	-4.26139	Male	0.00098	1.71096	Female			
Mrap										0.00000	1.71085	Female				0.00000	1.56800	Female
Smim11										0.00006	-5.09898	Male	0.02196	2.49037	Female			
Dnajc1																0.00015	3.83595	Female

Xaf1										0.00000	-5.07574	Male	0.00007	2.39185	Female			
Arhgap26				0.00000	2.02340	Female							0.00158	4.25039	Female			
H2.Eb1				0.00006	-1.93870	Male												
Cox5b										0.00000	-4.70022	Male						
Sema4b										0.00000	-4.84313	Male						
Prokr2																0.00834	-4.75217	Male
Fsip1										0.00003	2.15036	Female	0.00000	1.95085	Female			
Clca3a1										0.01865	-1.97450	Male						
Rps6ka5																0.00525	3.72044	Female
Gabarapl2													0.01695	0.66074	Female			
Rfx4	0.00054	-1.29235	Male							0.00000	1.12481	Female						
Epb41l4aos										0.00001	-5.17735	Male						
Ccr4							0.02109	4.31974	Female				0.00627	3.95618	Female			
Clint1										0.01953	2.38588	Female						
Lypd8							0.00029	-1.67612	Male									
Naf1										0.01118	-3.25658	Male						
Rpp21										0.00003	-3.17321	Male						
AU023762										0.00044	-3.29211	Male						
Egfl8				0.00034	-1.76545	Male							0.01123	-4.37317	Male	0.00028	-5.94410	Male
Rsph9	0.00116	1.45757	Female															
Mvd																0.01450	2.89136	Female
H1foo																0.00006	-5.75362	Male
Gm5779										0.00800	2.58437	Female						
Smim14																0.03311	-3.38504	Male
Nrros																0.00000	2.50222	Female
Prickle1																0.04932	2.24698	Female
Tmem260	0.00106	-1.13333	Male							0.01004	-1.43437	Male						
Tmem243										0.00001	0.96656	Female						
Ucp2																0.00001	5.97585	Female
Rfesd										0.01395	3.33125	Female						
Gas5										0.00002	-2.35116	Male						
Gon4l										0.01281	1.84491	Female						
Prkag2os1													0.00000	1.33094	Female			
Cel							0.00222	-3.17481	Male									
B3galt4																0.00000	1.99758	Female
Calm5																0.00000	-1.71437	Male
Carkd	0.00329	4.71602	Female															
BC029214				0.00156	-2.07721	Male												
Proc										0.00488	-1.22402	Male	0.00000	1.63057	Female			
Il10rb	0.00020	-1.98505	Male	0.00006	-0.98613	Male				0.00280	-1.21508	Male				0.00000	4.74496	Female
Cdc42ep1							0.00041	4.15617	Female									
Crabp2													0.00000	-1.25469	Male			
Dpep3																0.03105	-2.63596	Male
Brk1										0.04182	1.08452	Female						
Ly6e				0.00000	0.86688	Female												
Gspt2																0.01240	3.77694	Female
Sec24b																0.00587	-2.25680	Male
Enpep										0.00086	4.92655	Female						
Gab2										0.00648	3.51106	Female						
Ptprt							0.03477	-4.63761	Male									
Fcf1										0.00315	-1.83219	Male						
Qtrt1										0.04890	2.16870	Female						
Snx7																0.04153	-5.00382	Male
Clk2																0.03377	-2.04750	Male
Cfi				0.01416	1.07407	Female							0.00000	-1.45765	Male			
Lpin1	0.00000	-1.07371	Male															
B4galt2										0.00000	2.99034	Female				0.00202	4.49081	Female
Jph1							0.00712	5.36017	Female									
Slc23a1										0.00000	-5.52324	Male	0.01750	1.48150	Female			
Polr2e										0.04508	1.45973	Female						
Cpa2										0.00000	-3.19641	Male						
Slco2a1										0.01493	3.68050	Female						
Mbl2																0.00000	2.63123	Female
Ak8													0.00156	5.31058	Female			
Ldhb																0.00000	-5.45576	Male
Pomk										0.00000	2.79596	Female						
Pla2g7				0.00016	-0.95318	Male												
Map9	0.04829	-0.85903	Male							0.04902	-3.87005	Male						
Myl9				0.00024	-1.58884	Male												
Cyp2d9										0.00275	6.38591	Female						

Polr2l										0.00000	-4.67031	Male	0.01232	1.43822	Female			
Snhg20										0.01455	-4.01441	Male						
Trpc6										0.00049	-2.33927	Male						
Scn2b										0.00004	-4.07402	Male						
Uba52										0.00000	-3.93836	Male	0.00069	1.56338	Female			
Tigar										0.04733	1.26356	Female				0.00660	-1.30389	Male
Naa38										0.00790	-2.66985	Male						
Muc1	0.00000	-1.52713	Male	0.00321	-2.39382	Male				0.00000	3.74846	Female						
Tomm40l				0.00000	-1.29306	Male				0.00000	-1.96199	Male						
Nucks1							0.03552	1.08312	Female									
Lypd1										0.00000	-5.20912	Male	0.00446	1.64122	Female			
Mirc35hg										0.00000	-4.30334	Male						
Tcp1										0.00000	1.88232	Female						
Galnt9													0.00550	5.36034	Female			
Zbtb33													0.01638	4.79754	Female			
Zfp667				0.00000	1.48893	Female	0.00000	-1.87104	Male									
D3Ertd254e							0.00211	2.77456	Female							0.00452	-2.53787	Male
Fam213a							0.00018	-4.66688	Male	0.01912	3.24334	Female						
Ing1													0.01263	1.27100	Female			
Fam212b																0.01254	-4.63077	Male
Dhx37										0.01034	3.19237	Female						
Itgal										0.00001	-0.83940	Male	0.00000	-5.20786	Male	0.00000	-3.63552	Male
Upp1										0.04885	1.64697	Female						
Minpp1										0.03334	2.66849	Female						
Pfkm	0.00057	0.74551	Female															
Duox1	0.00015	-0.97103	Male															
Ppp4r1l.ps																0.00781	-4.97268	Male
Ahsg							0.00000	-1.77586	Male									
Cd93										0.00000	-4.29083	Male						
Prr15l																0.00449	-5.05855	Male
Arf1										0.00308	2.13313	Female						
Rin1										0.00182	-3.37415	Male						
Gimap9										0.00891	-1.00100	Male						
Dusp22													0.04329	2.06187	Female			
Eif2b1										0.00024	2.13559	Female						
Rab2b																0.02280	-3.05753	Male
Gja1							0.00489	2.50286	Female									
Bcl2l10										0.00269	-5.56647	Male						
Scaf4										0.04789	2.42451	Female						
Scamp3										0.00000	6.86772	Female						
Coq10b				0.00703	3.02400	Female												
Ndufb2										0.00002	-5.22281	Male						
Kdm5b																0.04574	1.66741	Female
Ube3a							0.01128	2.90563	Female									
Gm1965													0.02612	-4.53895	Male			
Agpat9							0.03513	1.84010	Female									
Tcf7l2				0.00001	-0.61156	Male												
Wdsub1										0.03694	3.46568	Female						
Tpcn1																0.01694	4.33558	Female
Gpbp1l1																0.03033	2.14106	Female
Nol10																0.00137	2.06384	Female
Slc2a1							0.00380	-3.74658	Male									
Rab26os										0.01698	-3.99777	Male						
Il17rc	0.00241	1.35501	Female							0.00541	-0.78649	Male						
Sapcd1	0.00396	1.69022	Female															
Sptb	0.00000	1.76626	Female															
Rhbdd3							0.02016	3.11628	Female									
Ndufa7										0.00000	-4.00912	Male	0.00002	1.94062	Female			
Bmp15													0.02856	3.93043	Female			
Itpr2													0.00071	4.31398	Female	0.00000	-0.63322	Male
Nxf2										0.00000	3.01091	Female						
Mgst3	0.03830	-4.54815	Male															
Rnf128										0.04690	1.82432	Female						
Pbdc1										0.00027	2.07810	Female						
Lcmt1				0.00199	3.38003	Female												
Ndufb3										0.00000	-3.34490	Male	0.00228	1.46736	Female			
Gm11517										0.00942	-1.92964	Male						
Ociad2										0.00004	-3.63371	Male						
D6Wsu163e																0.01368	-2.73153	Male
Cdca3										0.04141	1.37036	Female						

Fbln7													0.00000	4.36891	Female			
Zfp961																0.02986	1.83459	Female
Gramd1a	0.00000	-0.85796	Male															
Aloxe3																0.00265	4.07118	Female
Dll3										0.00074	-5.76939	Male						
Oxct2a																0.00305	2.66676	Female
Psma7										0.00368	1.53973	Female						
Hmgn3				0.00359	-0.72766	Male												
Tyr										0.00000	-4.88792	Male	0.01542	1.39370	Female			
Phb										0.01091	2.82138	Female						
Mrpl45										0.02816	1.67526	Female						
Ndc80																0.00007	-2.31335	Male
Tfcp2l1				0.02436	2.25613	Female	0.00000	3.12162	Female									
Syncrip							0.02243	1.07765	Female									
Fam46a	0.00027	1.27984	Female							0.00009	-0.71023	Male						
Chchd10										0.00129	-3.96770	Male						
Sap18										0.00054	-2.97728	Male						
Plp1													0.01743	-1.33325	Male			
Ndufb11													0.00738	1.53053	Female			
Zfp951																0.00001	-3.44937	Male
Vps72										0.03176	2.77541	Female						
Dnah17													0.00042	-4.86646	Male			
Cd84										0.00000	2.92339	Female						
Eef1g										0.00002	1.46590	Female						
Agmat																0.01257	-2.95200	Male
Gm10190										0.00814	-3.25472	Male						
Pdk1													0.00093	4.63461	Female			
Fndc10							0.00000	-4.88522	Male									
Rpl10							0.00222	1.02045	Female				0.00010	1.00695	Female	0.00929	0.86087	Female
Irf8										0.03149	-0.92345	Male						
Pja1				0.00000	1.42735	Female												
Ttc9c																0.02464	2.44609	Female
Acly										0.03586	1.37743	Female						
Mir703										0.00000	-1.64591	Male						
Tmc2																0.00005	-3.59487	Male
Poli										0.00062	-5.37255	Male						
Perp				0.00264	2.17134	Female												
Jmjd4							0.00095	3.70937	Female									
Kctd14							0.04812	-2.38603	Male	0.00870	-3.39202	Male						
Ppm1e										0.00000	1.25153	Female						
Immp1l				0.00797	2.70436	Female												
Ptafr										0.00001	-3.86809	Male						
Sox12																0.00001	5.02657	Female
Ltv1																0.00226	-1.00410	Male
Acp6				0.02436	1.11650	Female												
Hist1h2be				0.00001	-1.14551	Male				0.00001	-0.93746	Male						
Lrrc14b										0.00333	-2.96496	Male						
Ptrh2																0.03188	-1.41900	Male
Arsa	0.00031	0.84510	Female															
Cbln3										0.00000	-3.54297	Male	0.02954	1.43190	Female			
Ints6																0.00306	-3.23619	Male
Arrdc3										0.00000	2.16005	Female				0.00970	5.04206	Female
Zfp783	0.00663	2.41923	Female	0.00000	2.32510	Female												
Wdr36													0.03383	-1.09785	Male			
Orc6										0.00628	1.91084	Female				0.00944	-1.53261	Male
Fgr													0.00000	-5.52209	Male			
Tnfsf12																0.01199	-3.36224	Male
Topaz1				0.03318	1.00788	Female												
Atp5h										0.00000	-3.24627	Male	0.00917	1.09110	Female			
Gmppb	0.01788	3.65186	Female															
Mrps30										0.01188	-2.34221	Male						
Phkg1																0.01273	3.29507	Female
Nicn1										0.00724	3.15098	Female						
Phlda2													0.00032	5.09566	Female			
Anxa3				0.00036	-3.36235	Male												
Vgll4				0.00000	-2.25446	Male				0.00000	3.15926	Female						
Cpsf4l																0.00000	-0.83870	Male
Pop5										0.00016	-3.44114	Male						
Copg1										0.00307	1.63946	Female						
Prps2							0.03693	-4.76215	Male									

Rims3																0.02284	3.24997	Female
Ssr2										0.02008	1.29703	Female						
Sh2d4b	0.00796	-1.57053	Male				0.00000	-2.74912	Male	0.00000	2.79001	Female						
Rpl36al										0.00000	-3.81740	Male	0.00016	1.55227	Female			

Supplementary Table S3

Human: differential expression results

Gene	padj_E3	log2fc_E3	sex_E3	padj_E4	log2fc_E4	sex_E4	padj_E5	log2fc_E5	sex_E5	padj_E6	log2fc_E6	sex_E6	padj_E7	log2fc_E7	sex_E7
ZNF791										0.00203	0.58666	Female			
INSR										0.00000	1.03876	Female			
NCAM1							0.03580	0.74937	Female				0.02441	-0.75766	Male
ZNF280B										0.00000	1.89189	Female			
AGK										0.01101	0.66244	Female			
PIH1D1	0.01276	0.90882	Female												
ZNF275	0.00081	4.74426	Female				0.00266	2.38854	Female						
MEF2D													0.03502	0.64670	Female
MKRN2													0.00014	0.65549	Female
MVB12B													0.00539	1.92401	Female
TXNRD2	0.00000	3.81244	Female	0.01406	1.34763	Female									
BAX				0.00290	0.75400	Female				0.00003	-0.65178	Male			
CCL15							0.00101	-1.99953	Male						
COL9A3							0.01072	-0.85559	Male						
TMEM256							0.00000	-0.76163	Male						
ZNF672							0.00050	-0.95802	Male	0.01492	-0.67451	Male			
METAP1	0.00211	-0.91666	Male										0.00000	-0.59825	Male
MIR4435-1HG				0.00236	1.28959	Female	0.00000	1.04438	Female						
PSMB1	0.00008	-0.64094	Male												
SLC7A11				0.00001	2.04664	Female							0.02382	-0.71622	Male
RARRES1							0.00001	-2.23215	Male	0.01863	-1.19473	Male			
SNHG10							0.01293	0.58438	Female						
PRDM1	0.03623	2.90588	Female												
SNORA75													0.00879	1.15678	Female
MINOS1	0.00000	1.02018	Female	0.00000	0.58473	Female									
ALDH18A1				0.01216	0.98415	Female									
CNNM4				0.03773	0.79253	Female									
LSR	0.01497	1.10240	Female												
PDLIM1	0.04221	-0.79606	Male												
MAGI1				0.00253	0.88093	Female									
NSUN3							0.04408	-0.97718	Male						
ATP6V0E1	0.00038	0.68717	Female												
KCNK6	0.00005	3.46718	Female												
HSDL1							0.02291	-1.04942	Male						
RNASE1							0.04010	0.88301	Female				0.04853	-0.79742	Male
TEX14										0.04957	-1.29640	Male			
RIPK2													0.00452	-0.60710	Male
LINC00624				0.04676	-1.05204	Male									
ANO1	0.00002	4.29809	Female	0.00152	2.21785	Female									
ZNF37BP	0.04403	1.70746	Female				0.00784	0.88819	Female						
POLI							0.01095	-0.86898	Male				0.01932	-0.76098	Male
SLC3A1										0.01325	1.77448	Female			
P2RX5				0.00104	3.46192	Female	0.00000	3.65361	Female						
MBOAT1							0.00001	1.16507	Female	0.00949	0.73049	Female	0.00001	1.13808	Female
C14orf159										0.02744	0.89376	Female			
SLC39A7										0.00000	-0.85315	Male			
NPIPB5													0.00009	0.62310	Female
TFCP2L1	0.00000	-1.52471	Male	0.00236	0.61010	Female									
ZNF286A				0.03246	-0.59062	Male									

CFTR										0.00073	-3.49012	Male			
BCL3				0.00255	2.42034	Female				0.00048	1.85951	Female			
HAS2										0.00000	2.79413	Female	0.01368	-1.49878	Male
PRKCH										0.03123	-1.12677	Male			
GAST										0.00927	-1.48057	Male			
GLTPD2										0.01326	1.31632	Female			
MPO				0.01420	-2.92706	Male	0.02445	2.13603	Female						
CCDC66				0.03537	-0.88393	Male									
HNF4A										0.00000	1.20521	Female	0.02052	-0.63075	Male
PBDC1				0.00000	1.06665	Female									
ABCC6P1							0.03999	1.42136	Female				0.00269	-1.89930	Male
PNMA5	0.00076	3.90801	Female							0.00000	3.62065	Female			
COPRS				0.01672	0.79571	Female									
MAT2A							0.00000	-0.59575	Male						
THUMPD2										0.00001	-1.02470	Male			
CD274	0.01042	-2.28716	Male	0.00388	1.77977	Female				0.00580	-1.04423	Male			
RTKN				0.00000	1.57683	Female									
SLC16A4							0.02344	-1.24733	Male						
RPL10L										0.00000	-2.63195	Male	0.03236	0.80432	Female
TXLNGY				0.00000	-4.78953	Male	0.00000	-5.99741	Male	0.00000	-6.37012	Male	0.00000	-5.59213	Male
C9orf37							0.02258	0.66160	Female				0.01610	0.64772	Female
COX7B	0.00022	0.61136	Female	0.00000	0.77757	Female	0.00000	0.66975	Female	0.00000	0.58748	Female			
CRYBB2P1							0.00001	0.99795	Female						
ANKRD18B										0.03917	2.06242	Female			
TMEM229B										0.00000	2.61210	Female			
DENND2D													0.00200	1.26810	Female
PJA2							0.00000	-1.06734	Male						
C5orf34							0.03814	-0.66435	Male						
NELL2										0.02678	1.50653	Female			
ENOSF1				0.01799	0.58392	Female									
RAB8B							0.00018	-1.00284	Male						
DST				0.01816	0.85750	Female				0.00007	0.85817	Female			
UNC119B													0.00241	-0.96224	Male
NDUFS2	0.01969	-0.64880	Male												
ERVFRD-1							0.00013	2.17375	Female				0.00054	1.90182	Female
PPP1R16B										0.02062	2.51241	Female			
PTGES				0.00000	1.80697	Female							0.00028	0.72578	Female
TMEM67				0.00656	-1.81806	Male									
OTX2													0.02745	-1.16516	Male
TMEM185A				0.03451	1.21180	Female							0.02711	-0.81823	Male
NANOGNB										0.00000	1.90535	Female	0.00000	2.17604	Female
MAL										0.02716	2.43234	Female			
GNAI1							0.02442	-1.07653	Male						
DECR1				0.00000	-3.01803	Male	0.00547	-1.16240	Male						
BNC1										0.00395	-2.53877	Male			
FANCL							0.00050	-0.68722	Male						
NPIPA1										0.01364	0.77016	Female	0.00104	0.94288	Female
TPT1-AS1	0.04232	1.21869	Female				0.00012	-0.72479	Male						
PRKACB				0.00179	2.72591	Female									
NRG1										0.01098	1.72999	Female			
TTTY15	0.00000	-6.74729	Male	0.00000	-6.63303	Male	0.00000	-7.37300	Male	0.00000	-7.22747	Male	0.00000	-7.39977	Male

FAM115A				0.00006	-1.83872	Male	0.04080	-0.72659	Male	0.02549	-0.70431	Male	0.02002	-0.69947	Male
SETBP1				0.02120	-1.54782	Male									
SLC8A3													0.04509	-2.10976	Male
BAG6				0.00235	0.69048	Female									
SBNO1	0.00149	-0.65986	Male												
CD52							0.00019	2.16791	Female	0.01132	1.50627	Female	0.00000	3.36998	Female
LRRC47							0.00000	0.71443	Female						
KCNQ1OT1				0.00962	-1.16366	Male									
NIM1K				0.00050	-2.37445	Male	0.01232	-1.20211	Male	0.04375	-0.93821	Male			
LOC101927380				0.04491	-1.62601	Male									
SMNDC1	0.04252	-0.61759	Male												
LOC101928254													0.04712	-1.34187	Male
EYS													0.00606	-1.47950	Male
FAM220A							0.03627	-0.74631	Male						
ZSWIM7				0.00253	0.58636	Female									
MYEF2							0.01537	-1.76398	Male						
GABRQ				0.04306	2.90530	Female									
FCGBP							0.03678	1.30915	Female						
SCRN3							0.03438	-0.82361	Male						
TRMT61A							0.00054	0.60453	Female	0.00024	-0.60755	Male			
DLGAP1-AS2										0.00894	1.04178	Female			
ID4													0.00472	-1.27504	Male
FGF13				0.02440	1.29487	Female									
LOC284379							0.00555	-1.73501	Male	0.02104	-1.40637	Male			
SFT2D3										0.02956	-0.94984	Male			
FBXO44										0.00016	2.37390	Female	0.00248	1.93983	Female
HDDC3	0.01196	-1.63230	Male												
DLX4				0.00000	2.52118	Female									
HPS3				0.02848	1.14888	Female									
DSTN							0.00000	-0.64117	Male						
HMGCL										0.03660	0.85133	Female			
RAB40B										0.00011	1.76139	Female			
SETD1A										0.00610	0.63681	Female	0.00108	0.72700	Female
FCER1G										0.03191	1.53530	Female			
NIT2				0.00000	0.88947	Female									
BACH2													0.00204	-1.23456	Male
COPS8				0.00293	0.64570	Female									
PKMYT1							0.00002	-0.87981	Male						
PRDX2							0.00000	0.87532	Female						
NOVA1				0.03859	-1.11784	Male				0.03562	-0.73112	Male			
FAM122C	0.00280	2.43047	Female							0.00000	1.77959	Female			
CCDC22				0.00008	2.28898	Female	0.00960	1.11700	Female	0.01950	0.99026	Female			
PI4K2B													0.03917	-0.87841	Male
MAN2B2	0.00010	3.11095	Female												
SNAR-G1	0.00000	-6.24438	Male										0.00331	0.93880	Female
PINK1-AS													0.01605	0.59918	Female
ARHGEF2	0.00031	3.08875	Female	0.00013	-1.96965	Male	0.01204	-0.93650	Male						
LOC100506675							0.00447	0.64873	Female	0.00002	-0.89955	Male	0.00000	-1.21733	Male
SLC35F6							0.00000	0.77898	Female	0.00002	0.67010	Female			
NR2F2										0.03610	-0.61933	Male			
CUTC	0.00074	-1.31676	Male												

ATAD3C				0.00765	1.51258	Female									
LINC00842										0.01102	-0.66787	Male			
CASC2													0.01008	1.93492	Female
KCND1	0.01337	3.40651	Female												
PUS1	0.00001	-1.53493	Male												
PTBP3				0.00002	0.91103	Female									
FYTTD1										0.01390	0.58467	Female			
TMEM191B							0.00004	-1.14993	Male						
SORBS1										0.00023	0.76318	Female			
SPATS2L										0.01235	0.93334	Female			
KIF14							0.01369	-0.58344	Male						
TRIM77													0.00022	-3.03583	Male
LINC00116				0.00160	0.76814	Female									
LOC399715										0.00048	1.05940	Female	0.04509	0.65858	Female
EXOSC10	0.00266	-0.77153	Male												
ART5										0.01349	1.97489	Female			
PDLIM3													0.00753	-2.57999	Male
EIF3M	0.00011	-0.64643	Male												
LRRC8A							0.00001	0.67193	Female						
CASKIN1										0.00351	1.88443	Female			
MCM9							0.00545	-0.91076	Male						
RAB18	0.00000	-1.67025	Male												
C1D				0.00216	-0.61788	Male							0.00000	-0.82067	Male
TRMU							0.00000	0.78359	Female						
S100P	0.00009	2.89802	Female							0.00092	0.88570	Female	0.00347	0.79428	Female
CCT7	0.00029	-0.76001	Male												
SLC25A16							0.00020	-0.60378	Male	0.00000	0.73142	Female			
CYB561A3										0.01817	-0.70757	Male			
PLOD2													0.00088	-0.94578	Male
ZFP37							0.00154	-1.57323	Male						
PER2							0.00066	0.73294	Female						
TMEM91										0.03537	-1.37904	Male			
MINA							0.03301	-0.68709	Male						
MT1E													0.00193	1.70150	Female
TRPV1													0.03592	0.76319	Female
LOC101928559													0.00001	-5.61557	Male
ALG6							0.00132	-0.65956	Male						
HMGN2							0.00000	0.91059	Female						
NRGN										0.00000	3.87322	Female			
TRPS1	0.00710	3.52169	Female												
VTI1B							0.00000	0.60048	Female						
NTN4										0.01635	1.70785	Female			
ZNF480										0.00001	0.60356	Female			
DNAJB6	0.01091	-0.66507	Male												
ANKRD19P	0.00014	0.87745	Female												
GLRX				0.00054	1.93161	Female									
PDCL3P4										0.00019	0.75494	Female			
LEFTY2							0.00018	0.74562	Female	0.04129	0.73288	Female			
TBC1D25										0.04756	0.65870	Female	0.04860	0.64806	Female
FNBP1							0.00052	0.63287	Female	0.00091	0.58541	Female			
DYRK1A	0.00147	-1.55470	Male												

TPTE2P6										0.00225	1.61576	Female			
HSP90AB4P										0.00000	-1.50791	Male			
EGR1													0.00018	1.67233	Female
CLDN3				0.00000	2.81681	Female	0.00003	-1.34708	Male	0.00117	-1.01062	Male			
DDX43										0.00355	0.69981	Female			
LOC100996324										0.04046	1.47660	Female			
ADAMTS20										0.00832	-1.73507	Male			
ACTA1										0.00000	-2.20342	Male			
FBXO48										0.01316	1.01817	Female			
KLF17										0.00000	2.75074	Female			
NSUN5P1													0.00313	0.60767	Female
PTCD1							0.00062	-0.89241	Male				0.00674	0.69617	Female
FBN2										0.01363	-1.83664	Male			
SNORA78													0.00964	0.88780	Female
CYB5R1										0.01446	-0.60097	Male			
LDHAL6A										0.01321	-1.41164	Male			
AURKA	0.00939	-0.77924	Male												
ZNF75D				0.00001	2.08534	Female	0.01094	0.93814	Female	0.01989	0.83570	Female			
PGC							0.01342	1.46898	Female				0.03157	1.27032	Female
LYAR	0.00338	-0.67760	Male												
OGFR	0.04955	-1.76441	Male												
RAB15										0.00000	1.56130	Female			
TGFBR2										0.03489	-1.14414	Male			
USP25													0.00007	-0.86445	Male
FOXE1				0.03388	1.88374	Female									
TPST2										0.00046	0.71152	Female			
PCDH1										0.00735	0.72445	Female			
CCDC102A													0.03242	1.21785	Female
SERHL2													0.00000	0.96046	Female
C15orf41							0.01991	-0.59804	Male						
AUH							0.00315	-0.74865	Male				0.00737	-0.64470	Male
MCAT							0.00126	-0.67413	Male	0.00177	-0.62491	Male			
TMEM106C							0.00000	-0.85231	Male	0.00000	-1.03371	Male			
ROGDI				0.00000	1.70101	Female									
KCNC3													0.00053	0.94575	Female
FKBP9	0.04437	1.94102	Female												
NFAT5				0.04367	0.80122	Female									
GEM													0.00398	1.88854	Female
SKAP2				0.03670	-0.67002	Male									
PDLIM7							0.00761	0.58084	Female	0.00003	0.81915	Female			
OSBPL1A							0.00084	-1.24549	Male				0.01835	-0.87287	Male
RPL34-AS1							0.01304	-1.28586	Male						
TENC1							0.03527	-2.50078	Male						
CBFA2T2										0.00000	1.08430	Female			
ERF										0.00000	0.92054	Female			
PDE4A										0.00175	1.50997	Female			
ITGB3													0.02542	-2.41590	Male
DCAF10				0.00183	1.79663	Female									
FZD5							0.00000	0.95966	Female						
SNTB1													0.00000	-3.36521	Male
DIS3L	0.01582	-1.21070	Male												

LINC00987										0.00003	-0.61459	Male			
MKL1							0.00559	-0.62675	Male						
RNF139							0.00198	-0.67701	Male						
AP2A1							0.00011	-0.71310	Male	0.00067	0.61258	Female			
KDM4B													0.01777	0.79591	Female
BPGM										0.00119	-0.82973	Male			
DDX60L													0.00798	2.37705	Female
POMT2							0.01010	0.65529	Female						
ARHGEF1							0.02425	-0.66499	Male						
CFL1P1										0.01132	-1.36996	Male			
GRK5										0.00005	1.92005	Female			
ACADM				0.01218	-0.76063	Male									
MAFG	0.03750	-1.06886	Male												
LAMA4										0.00038	2.12061	Female			
LINC00668										0.00000	-0.90123	Male			
NMRK2										0.00029	1.11174	Female	0.00047	1.06381	Female
IFNGR2													0.00003	-1.56165	Male
IL6							0.03266	0.92102	Female	0.00112	-1.24519	Male			
TMEM65													0.00472	-0.86960	Male
PTPRZ1										0.00080	2.19468	Female			
DPF1				0.04433	1.58873	Female				0.00000	2.44583	Female			
SCD5										0.02560	-1.11310	Male			
MIR6847							0.00000	1.17564	Female						
CIPC				0.00234	-1.09489	Male									
ARL4C				0.00161	1.73339	Female									
PGRMC1	0.00904	1.13676	Female							0.00009	0.63203	Female			
APP				0.01431	0.90292	Female				0.00000	-1.00791	Male			
TNFSF13B				0.00045	-1.43621	Male				0.00001	-1.19088	Male			
ARL8A										0.01037	0.83003	Female			
CLCN6				0.00083	-1.41136	Male									
HIST1H2AC							0.00060	1.27224	Female						
OAF				0.00664	-2.12335	Male									
SLC23A2										0.00022	0.75815	Female	0.00012	0.77144	Female
TPM4				0.00005	1.02255	Female									
VASH1										0.00024	1.89216	Female			
ACTR6							0.00016	-0.58397	Male						
FKBPL										0.00407	-0.58714	Male			
PIEZO1				0.00049	1.27878	Female									
PTN							0.00009	-0.89238	Male	0.00027	-0.79523	Male			
AGTRAP				0.02798	0.99722	Female									
EPSTI1				0.00677	1.43504	Female									
CCDC181										0.01944	-1.41295	Male			
CNNM3	0.00002	1.83651	Female												
LINC00479				0.01120	2.03454	Female									
NPTX1				0.00013	2.67907	Female				0.00497	1.44971	Female	0.00000	2.23873	Female
CTDP1										0.01113	0.64104	Female			
PTS										0.00000	-0.74990	Male			
CITED4	0.00000	3.22419	Female	0.00006	1.51599	Female							0.00002	1.07075	Female
GRK6	0.00000	3.31965	Female												
JAK1				0.00420	1.03271	Female									
CDKN1B				0.04806	-1.35059	Male				0.02888	-0.91955	Male			

SRSF12				0.04563	1.35614	Female									
MFN1							0.00000	-0.71993	Male						
SKIL										0.00000	0.89026	Female			
ATP5L2													0.00320	0.87538	Female
DNAH12							0.02752	-1.37478	Male						
S100A14	0.00064	2.51005	Female	0.00000	2.12827	Female									
SLC35D1				0.03101	1.05204	Female									
CBX2										0.00306	-0.86584	Male			
DLG3				0.04476	0.73180	Female	0.00000	1.07878	Female	0.00000	1.05966	Female			
RNF14	0.02490	-1.07682	Male												
KIAA0020	0.00008	-0.75943	Male												
SLC22A5													0.02351	0.80850	Female
AP1G2				0.01641	0.97694	Female									
SNORD25													0.01816	0.69624	Female
CHI3L1							0.02473	2.09446	Female						
LMOD2							0.01950	1.12736	Female				0.02849	1.01459	Female
RCN2							0.00009	-0.72677	Male				0.00000	-0.87782	Male
GNB4							0.00766	-0.81101	Male	0.00005	-1.09880	Male	0.00253	-0.84017	Male
RPS6KA5							0.01322	0.63050	Female						
PROCR	0.00074	1.46372	Female												
ABCB6	0.00013	1.83011	Female				0.00000	-0.90676	Male	0.00003	-0.79368	Male			
CRYGB										0.00464	2.87308	Female			
BICD1				0.00831	0.95793	Female				0.00000	1.01272	Female			
PLXNA2				0.01428	1.27939	Female									
ZP4										0.00000	-2.73189	Male			
KAZN	0.02760	2.32670	Female							0.00036	1.18602	Female			
STOM										0.00019	0.76693	Female			
INPPL1	0.03750	-1.34360	Male	0.02449	0.84908	Female									
NTMT1							0.00000	0.77884	Female						
LGALS16										0.00047	-1.80054	Male			
IGFBP3													0.00000	-3.93043	Male
TCTA													0.01111	0.74937	Female
CD81				0.00000	1.57861	Female				0.00056	0.58435	Female			
MYO10										0.00676	-0.63813	Male			
HPCAL1	0.00012	2.67574	Female	0.00001	1.78896	Female									
OR51B5							0.02169	-1.13465	Male						
DYSF				0.01279	-0.94230	Male									
ARPC4	0.01091	-0.59554	Male				0.00000	0.68538	Female						
KIAA0355										0.00019	-0.95460	Male			
HEXB							0.00000	-0.77814	Male						
LLPH	0.01497	-0.58302	Male												
UBE2E2							0.00005	-0.80997	Male						
PRSS3										0.00000	4.08549	Female	0.01670	-1.50691	Male
NPTX2							0.00000	2.91338	Female						
SLC38A3	0.03308	-1.83046	Male										0.01552	1.73132	Female
PPCS	0.02265	-0.89691	Male												
C21orf33				0.00000	0.92607	Female									
IFITM3										0.00000	1.09687	Female	0.00000	1.14456	Female
PREB							0.00001	-0.65440	Male						
FAM127B	0.02218	1.35944	Female	0.00021	1.22658	Female	0.00005	0.92729	Female						
FGF23				0.02600	-0.95004	Male				0.00746	-0.76667	Male	0.00248	-0.83098	Male

OTUD5				0.00010	1.05212	Female	0.00029	0.69068	Female						
HIST1H2BE										0.00215	1.63868	Female			
HINT3	0.04081	1.25206	Female												
ZYG11A										0.00557	0.64298	Female			
PHF2	0.01101	-2.49582	Male												
FADS1				0.03258	-0.99029	Male				0.00000	-1.29066	Male			
TSPYL4							0.00269	-1.15191	Male						
RGS5													0.00000	-1.86928	Male
GPR176										0.00026	1.73133	Female			
CBFB													0.00010	-0.58733	Male
KLRG2				0.00035	1.15040	Female				0.00060	0.74038	Female			
SLC30A1				0.00263	1.65528	Female									
MAPRE2							0.00581	1.03931	Female						
FAM9B				0.00509	3.22259	Female									
RHBDD1							0.00159	-1.15155	Male						
OXA1L	0.01471	-0.58269	Male												
FAM120C				0.00062	1.40673	Female	0.00038	1.01799	Female				0.01265	0.70448	Female
SURF1										0.01017	-0.60909	Male			
LGALS3BP													0.01641	1.57708	Female
EGFR							0.00023	-2.56109	Male						
CENPM										0.00529	0.63570	Female			
DXO													0.01340	0.71586	Female
PPP1R35							0.00107	-0.97617	Male						
CXorf38										0.02200	0.60170	Female			
SLC44A3							0.00145	-2.21958	Male						
CD70										0.00225	2.80962	Female			
SPAG5-AS1							0.00381	0.80078	Female	0.01735	-0.64935	Male	0.03310	0.58713	Female
GPR39													0.00015	-3.48925	Male
CD151	0.02522	-1.09646	Male	0.00000	1.34585	Female									
CELF6										0.02542	-1.54951	Male			
KLK7	0.00273	3.84075	Female												
EFNA4										0.00953	0.58283	Female			
SUSD5										0.00514	2.67760	Female			
C8orf31							0.01238	1.84152	Female				0.00001	2.85295	Female
IDO1							0.02534	2.24577	Female						
SNHG9							0.00000	0.93412	Female				0.00000	0.75218	Female
HAUS1	0.00178	1.13018	Female												
CGB										0.00000	-2.91123	Male			
S100A16	0.02761	-0.89333	Male	0.00000	1.00986	Female									
ASRGL1										0.00000	0.77797	Female			
CTBP1-AS													0.01340	-1.12171	Male
THSD7A													0.00004	-3.71429	Male
CCDC142				0.00696	-1.07916	Male				0.02497	-0.60061	Male			
MTRNR2L8										0.00000	1.32022	Female			
IFT43				0.01047	1.29270	Female									
CDA										0.00000	3.40254	Female			
ZNF160										0.03814	0.60402	Female			
DNMT3L	0.00477	-0.87300	Male	0.00000	1.26444	Female				0.00000	0.73879	Female			
TUBB2B				0.00252	1.63057	Female				0.00000	1.47386	Female			
SH3TC1				0.00000	2.65476	Female									
CAPZA2	0.00019	1.28785	Female												

C9orf135										0.00338	1.75596	Female	0.03003	-1.32456	Male
ZNF350-AS1							0.00003	2.33626	Female						
NOA1													0.00001	-0.60873	Male
SPNS3							0.04443	1.84254	Female	0.00780	2.25262	Female			
DENND6A				0.02089	1.35321	Female									
LOC100294362													0.02250	1.30804	Female
C11orf83							0.00000	0.72759	Female	0.00000	0.91117	Female			
MDP1										0.00072	1.11759	Female			
RND3										0.03357	-1.99547	Male			
ZBED6							0.00000	-2.07381	Male						
SH3D19				0.00702	1.38278	Female									
ABCB10	0.00011	-2.26911	Male												
DNAJC19	0.00015	-0.90402	Male												
ERBB3	0.02274	1.18308	Female												
STAG2				0.00008	0.70975	Female	0.00000	0.78938	Female						
DALRD3							0.01655	-0.65457	Male						
HMGN3				0.00107	1.17779	Female							0.00084	-0.72985	Male
FBXL3													0.00505	-0.77708	Male
MID1IP1				0.00000	2.40293	Female				0.02153	0.58964	Female			
CDS2	0.01112	-1.20864	Male							0.00053	-0.63194	Male			
EIF1AD							0.00000	0.58930	Female						
EGLN3				0.00000	1.27556	Female				0.00012	0.73298	Female			
NDUFAF6	0.03913	1.18124	Female	0.02402	0.68907	Female									
MIR6764							0.00244	0.76216	Female						
ST6GALNAC3				0.00033	3.19448	Female				0.00074	2.33309	Female			
GOLPH3													0.00000	-0.59498	Male
LOC100130093							0.00159	0.78964	Female						
MIR663A										0.00000	3.93251	Female			
C10orf32										0.00008	-0.77097	Male			
TOP1MT	0.02366	-0.97836	Male												
RSU1	0.00180	-1.10921	Male												
FLNA				0.00000	1.91172	Female									
DSC2	0.00001	1.91749	Female	0.00058	0.91522	Female									
MIR17HG				0.00027	-1.05873	Male									
H2AFX				0.00392	0.69629	Female									
EMC2							0.00001	-0.69562	Male						
EMX1							0.03734	1.25958	Female						
RBM38							0.00000	0.66499	Female						
STARD4							0.03911	-0.75220	Male						
PITPNM2										0.04707	1.22785	Female			
IQCH-AS1				0.00528	1.28401	Female									
IGSF9													0.04656	0.87687	Female
LYPD3				0.03947	-1.27989	Male				0.00000	1.86345	Female			
CLIC4	0.00105	-0.86126	Male												
S100A9													0.01663	2.29450	Female
SPARC										0.00000	1.42447	Female			
SREBF1							0.00993	-0.71912	Male						
SUZ12P1										0.01448	0.65387	Female			
KLF8	0.00162	2.41552	Female	0.01200	1.18328	Female	0.02594	0.72744	Female	0.00005	1.16369	Female			
PDZD11				0.00000	1.01931	Female	0.00000	0.83731	Female	0.00000	0.74159	Female			
LMO7													0.00393	-1.24493	Male

PCBP1	0.00006	-0.85928	Male												
FZD7										0.00000	4.08769	Female			
PJA1													0.00003	-1.63796	Male
ATRX				0.00001	0.84558	Female				0.00000	0.68471	Female			
SNHG21										0.01326	0.61066	Female			
NDUFS8	0.03061	0.58521	Female												
MREG	0.01700	2.17592	Female				0.04823	-0.74603	Male						
C12orf57				0.00010	-0.77847	Male									
NHSL1				0.00020	0.88830	Female									
APEX2				0.02610	0.62059	Female	0.00000	0.77040	Female						
ZNF138							0.00007	-0.74190	Male						
CCDC57	0.01174	-1.93576	Male	0.03859	1.24533	Female							0.01902	0.88357	Female
E2F5										0.04930	-1.13272	Male			
POLR2G	0.00021	0.80558	Female												
CRYBA2				0.02519	1.73437	Female	0.01639	1.25790	Female				0.00043	1.65049	Female
TNIP2				0.00171	0.88725	Female									
NOS2				0.00584	-2.23894	Male									
ELOVL6							0.00000	-0.83808	Male						
PDE1A							0.00549	-2.68535	Male						
TM4SF5										0.00020	1.58299	Female	0.00096	1.43368	Female
GATA3				0.00018	1.05485	Female									
RASSF8													0.01984	-0.58158	Male
AIF1L	0.00057	-1.06164	Male	0.00120	0.62365	Female									
MAP1B										0.00058	1.45750	Female			
CD19										0.04871	1.38189	Female			
KPNA5	0.01280	-2.04727	Male												
PRAMEF19.uid-865													0.00002	0.83415	Female
MOB1B										0.00006	-0.73165	Male	0.00001	-0.77202	Male
FBXW5	0.00009	-1.51511	Male												
FBXW4				0.04634	1.21367	Female									
ESD				0.00277	0.95283	Female									
MIR4668							0.00222	-0.69209	Male	0.01019	0.62088	Female			
HIST1H2BF				0.00139	-2.35547	Male									
C19orf48	0.00497	0.67630	Female												
HMHA1				0.01686	0.86458	Female							0.00265	-0.65656	Male
FAM174B										0.00498	1.88095	Female			
SUN1										0.00006	0.64877	Female			
PPFIBP2													0.00006	0.71810	Female
NCRNA00185							0.00073	-3.00643	Male	0.00000	-3.98578	Male	0.00000	-4.28839	Male
ARHGAP17				0.01663	0.74860	Female									
SGSH							0.00313	-1.16947	Male						
EIF4EBP2	0.00002	-1.34657	Male												
SMIM1				0.03651	-1.57785	Male									
CDCA7	0.00002	-1.97614	Male												
C1orf64							0.02550	2.41118	Female	0.01399	2.33370	Female	0.00000	-4.40564	Male
ZDHHC2							0.04902	-1.01410	Male						
GTF2IRD1										0.00018	1.90494	Female			
PSME2				0.01663	-0.58700	Male									
GTPBP4	0.00032	-0.76468	Male												
WNT7A				0.00000	1.03309	Female									
LOC100506071										0.00000	-1.29662	Male	0.00922	-0.73123	Male

NMT2										0.00000	1.39918	Female			
LINC00643				0.00000	0.94508	Female							0.01820	-1.21481	Male
FOXN4													0.01904	1.83369	Female
HOXB7													0.01253	-0.89585	Male
LOC101927069													0.02836	1.42445	Female
FOSB							0.00000	-3.09954	Male	0.00001	-2.37949	Male			
EIF5A2				0.01500	0.85518	Female	0.00527	0.65561	Female						
ID3							0.00263	-0.99972	Male	0.00664	0.87816	Female			
LOC728024	0.01676	0.99187	Female	0.01372	0.64453	Female	0.00000	1.10005	Female	0.00000	0.98910	Female	0.00000	0.94540	Female
APOOP5				0.00004	1.11819	Female									
NUDT10							0.00004	2.15311	Female	0.01401	0.99384	Female	0.00119	2.05808	Female
C8orf37-AS1										0.03032	-1.08458	Male			
FAM214B										0.00610	1.22775	Female			
HRAS										0.00020	-0.90440	Male			
HOXD1										0.00149	2.61843	Female			
HIST1H1E				0.00032	-1.83682	Male				0.00397	1.02653	Female			
LOC653513										0.00190	1.38804	Female			
COBLL1							0.01012	-0.69602	Male						
KRT8				0.00120	0.68320	Female									
MUC4							0.03733	1.64197	Female						
SLC43A2							0.00011	0.98883	Female	0.00151	0.80150	Female			
B3GNT7				0.00000	1.88400	Female	0.00000	1.23933	Female	0.02898	0.58901	Female	0.00013	0.92577	Female
AJAP1													0.00323	2.93403	Female
ZYG11B							0.00720	-0.78916	Male						
BTN2A1							0.03503	-0.96997	Male						
CAPN5										0.04505	0.95608	Female			
RPS28													0.00000	-0.71416	Male
LOC101928371							0.00012	2.19669	Female						
ZP2													0.00003	-2.16639	Male
DNAH3							0.00006	1.61788	Female				0.00768	1.08738	Female
PSMD7	0.00000	-0.85967	Male												
PYCARD										0.00191	2.16374	Female			
GLA	0.00016	1.32140	Female	0.00010	0.85378	Female									
MAP3K9										0.04101	0.66730	Female			
THOC2				0.00000	0.94456	Female	0.00000	1.02230	Female	0.00000	0.80635	Female	0.00000	0.78369	Female
RMRP										0.00002	0.88433	Female			
TRIM46	0.00052	4.09968	Female												
LOC641367										0.00533	1.30163	Female			
IPP							0.03602	-0.58957	Male						
TMEM107							0.01176	-0.95471	Male						
KMT2B													0.00076	0.64935	Female
FGFBP2													0.00858	1.82739	Female
GAB2							0.00469	-0.71643	Male				0.00582	0.66417	Female
OVOL1				0.00000	1.77437	Female							0.02288	0.62603	Female
ANKRD36										0.00053	0.95998	Female			
SLC15A4				0.00562	1.07548	Female									
DUSP4							0.03698	0.99053	Female						
PTGDS							0.03742	1.47291	Female						
DEFB122										0.00000	2.42756	Female			
PRUNE2										0.00345	1.49264	Female			
BCL2L12	0.03391	0.65170	Female												

MSMB										0.00000	2.18016	Female	0.00113	1.55975	Female
SH2D4A										0.00304	-0.68004	Male			
MIPOL1							0.03999	-1.29220	Male						
MOCOS							0.00694	-1.77417	Male	0.00135	-1.98832	Male			
EPHB2				0.00001	3.23612	Female				0.03914	1.20177	Female			
COMMD10	0.00979	-1.28945	Male												
CITED2							0.00087	0.84607	Female	0.01997	-0.59293	Male			
LRAT													0.00200	-2.43670	Male
PRKAR1B							0.00116	0.85356	Female						
ZC3HAV1										0.02297	0.73527	Female	0.04456	-0.65327	Male
FA2H							0.00734	-0.97414	Male				0.00000	-1.74918	Male
GLRX3	0.00000	-0.91739	Male												
LOC100507291										0.04103	0.61949	Female			
RSAD1										0.04762	0.60464	Female			
CHPT1							0.00001	-0.75516	Male						
MRPL36	0.00100	0.79734	Female				0.00000	0.65832	Female						
ATAT1	0.04446	1.42855	Female												
HAP1										0.00152	-1.42202	Male			
SEC61A2										0.01754	0.60244	Female	0.01187	0.61893	Female
UCHL1										0.00023	-1.23993	Male			
LOC100131564	0.04780	-2.34327	Male												
MOSPD2										0.00185	0.80704	Female			
SLITRK1										0.03659	-1.56220	Male			
PIGU							0.00927	-0.69377	Male						
RPS4X				0.00000	0.59722	Female				0.00000	0.73626	Female	0.00000	0.90349	Female
HMGN4										0.00000	-0.60727	Male			
CMTM7	0.00477	-1.69356	Male	0.00000	1.70383	Female									
FAM13A										0.00116	-1.71129	Male			
BCKDHA													0.00019	0.76174	Female
PPP2R2A	0.03273	-0.72610	Male												
MKRN3													0.02428	0.68793	Female
ACADVL				0.02039	0.75555	Female									
RAPGEF1				0.00004	1.37793	Female									
PRCP	0.00052	2.43290	Female												
DDAH2							0.00005	-1.16973	Male						
AHNAK							0.00021	-0.67059	Male						
UTF1										0.00017	2.47101	Female			
CEACAM6										0.00014	2.55502	Female			
NTNG1										0.00293	2.03108	Female			
TOM1L2													0.02018	1.21745	Female
TXNIP	0.04780	1.62035	Female							0.03548	0.60242	Female			
RAI14				0.00021	1.02235	Female									
BCLAF1	0.00155	-0.66089	Male												
G3BP1	0.00105	-0.73635	Male												
CEBPD							0.00053	-1.36605	Male						
SNORD58C										0.03948	0.67817	Female			
BEX2	0.00012	2.78865	Female							0.00000	-1.52764	Male			
WNK3				0.02326	1.29857	Female	0.00003	1.48407	Female	0.00000	1.67813	Female	0.00064	1.17916	Female
GDAP1										0.00583	-1.28932	Male			
ANKRD55										0.00034	1.65701	Female			
KLHL3										0.01654	2.03392	Female			

STIM1							0.01749	-0.77950	Male						
STEAP3				0.00188	-1.64672	Male									
DAZL										0.00129	-2.21201	Male			
LINC00707													0.04084	1.23488	Female
UBASH3B										0.00003	1.04963	Female			
POU6F1										0.02518	1.55632	Female			
LOC339166							0.00027	3.35654	Female						
PDPN							0.01058	-0.92146	Male	0.00498	0.95217	Female			
ARFGAP3										0.00030	0.90183	Female			
MED12L										0.00000	-0.88297	Male			
CPT2							0.00081	-1.07588	Male						
REST										0.00000	0.76459	Female			
LILRB3	0.03671	2.57484	Female												
SCAMP3	0.00000	1.65787	Female	0.00085	0.73189	Female									
POLM							0.02188	-0.74528	Male						
TGFB1										0.02801	1.23623	Female			
TMBIM1										0.00000	1.30368	Female			
HBA1							0.00256	1.02232	Female						
MAEL										0.00000	1.83231	Female	0.00051	0.99264	Female
UBE3B													0.00060	-0.62859	Male
TNNT1										0.00049	1.60463	Female			
SMAGP				0.00000	1.96964	Female									
SHROOM2				0.00000	2.35228	Female									
FAM133A							0.00000	3.06431	Female						
MICB	0.00211	-2.92662	Male												
KNG1							0.00000	-1.47920	Male						
1.00000	0.00001	-1.04603	Male												
TARDBP	0.00124	0.62340	Female												
PLOD1							0.00050	-0.84867	Male						
ITPRIPL2										0.02268	-0.97147	Male			
HIST1H2BG				0.00694	-1.84447	Male	0.00000	2.23570	Female	0.02373	1.07397	Female			
CYP19A1	0.04291	-1.70028	Male				0.02240	1.14577	Female						
DOK1							0.02234	-1.53867	Male						
LPCAT1				0.01441	0.91931	Female									
NLRP8							0.02568	2.61490	Female				0.01227	4.31542	Female
PSKH1							0.01671	-1.18803	Male						
UCHL5				0.00059	0.71791	Female									
PLD6							0.02187	-1.28815	Male						
UPK2							0.00183	-1.94915	Male						
ZNF385A							0.00283	0.97624	Female	0.00890	0.83470	Female	0.02204	0.74350	Female
SH3GLB2				0.00000	2.16776	Female				0.00003	1.01099	Female			
NACC2				0.02343	1.61010	Female									
EPHA7										0.03492	1.74487	Female			
CD47							0.00000	-1.34441	Male						
TTC38										0.03480	-0.95409	Male			
GNL1	0.01314	0.86397	Female												
LIMK1							0.01204	-0.93838	Male						
EEFSEC										0.02116	0.72499	Female			
LOC101928378				0.03835	1.18366	Female							0.04059	0.73119	Female
SNORD32A							0.00121	0.74896	Female	0.01112	0.59517	Female			
MYOF				0.00000	1.21739	Female									

CREG1							0.00000	-1.35186	Male						
KIFC2										0.04064	1.07769	Female			
WDR47										0.00305	0.63059	Female			
KRT17													0.00000	4.60549	Female
RAB13							0.00624	-0.81174	Male						
BRD8													0.00000	0.70641	Female
ANXA2P2	0.00217	0.99573	Female	0.00014	-0.69763	Male									
JAG1							0.01772	1.04914	Female	0.00013	1.49374	Female			
EPHX1										0.00001	1.27137	Female			
TTC9													0.04989	0.88041	Female
CCDC71							0.03429	-1.46684	Male	0.00000	-3.07586	Male			
ANKS4B													0.00652	-2.33969	Male
ABCD4										0.00077	0.90502	Female	0.01383	0.68573	Female
MYO1E										0.02740	-0.58990	Male			
GALNT8										0.03918	1.20421	Female			
TCF7L1				0.00000	1.92499	Female							0.01843	0.60538	Female
RASGRP2	0.00279	-1.44274	Male												
KRT18				0.00017	0.79724	Female									
MAFA							0.03094	1.02490	Female						
FAM149B1													0.00245	-0.65323	Male
LZTR1	0.00003	-3.17266	Male												
TRABD2A							0.00001	2.85949	Female						
EIF5AL1													0.00000	-1.02198	Male
LOC101927258										0.00326	1.69742	Female			
MAP1S							0.00436	-0.59018	Male						
DAZAP1	0.00222	-1.13786	Male	0.00032	0.75981	Female									
CES2				0.00049	1.19401	Female				0.00302	-0.69013	Male	0.00440	0.66215	Female
CRYM										0.00215	2.34948	Female			
MUC21										0.03808	1.78631	Female			
SRSF8				0.01231	1.22038	Female									
PMEL				0.01284	-1.48393	Male	0.03586	-0.88336	Male						
C15orf65										0.00731	1.36068	Female			
ZNF782	0.02853	-2.67295	Male												
GSE1				0.00000	1.02404	Female									
ODAM										0.00000	-1.33628	Male	0.03051	-0.64975	Male
SCAND2P							0.01396	-0.86805	Male						
HLTF										0.00568	0.74915	Female			
NPM2													0.01919	-1.30003	Male
KCMF1				0.00000	1.35766	Female									
MAP7				0.01467	0.71677	Female									
THY1							0.00049	2.90034	Female	0.00197	2.51496	Female			
HOMEZ				0.00387	-2.09670	Male									
SOCS5							0.02081	0.86509	Female						
S100A6				0.03442	0.81458	Female									
POLR3G				0.03507	-0.69038	Male	0.00533	-0.58430	Male				0.00027	-0.68941	Male
YIPF6				0.00000	1.05689	Female	0.00000	0.74186	Female	0.00000	0.61585	Female	0.00000	0.61467	Female
TOR3A	0.00477	-1.32719	Male										0.00075	-0.61710	Male
TNRC6C							0.02038	0.83536	Female						
CRIM1										0.00008	-0.81173	Male			
COBL				0.00714	0.82799	Female									
LUZP6+MTPN										0.00000	-0.62038	Male			

DPH3P1	0.03830	-0.76511	Male												
TLE4										0.00012	-0.88449	Male			
FAM91A1													0.00004	-0.62001	Male
HBA2							0.00002	1.93275	Female						
PIBF1													0.00000	-1.02207	Male
ALDH3A2										0.00179	0.60340	Female			
RDH10				0.01307	-0.76108	Male									
SPTBN2										0.00267	-1.33625	Male			
SEPT14													0.00522	-2.27579	Male
SAMD4A													0.00899	-2.05990	Male
TPCN2	0.03519	-1.65438	Male				0.00805	-0.82000	Male						
MIR492							0.00402	0.60705	Female						
H19							0.04069	-0.77141	Male						
DYNC1LI1	0.00241	-0.87494	Male												
ANTXR2				0.03023	2.20420	Female									
NEB										0.00392	0.89399	Female			
SEPT12										0.01138	1.24299	Female	0.00020	1.67913	Female
CCDC87													0.04541	1.57738	Female
ZSCAN22													0.01248	0.98880	Female
SOX2-OT										0.01913	1.48075	Female			
ATXN1										0.02940	1.47581	Female			
DHX33							0.00000	0.73186	Female						
ALDH1B1				0.00000	3.02346	Female									
SMS				0.01378	0.58053	Female									
GPATCH3				0.00028	-0.86919	Male									
C11orf70										0.00142	-1.59629	Male			
ZDHHC11	0.01714	1.39007	Female				0.00362	0.91635	Female						
FAM96B				0.00000	0.67486	Female									
FAM120B							0.03444	-0.93126	Male						
GRIN2A										0.04305	1.56284	Female			
SLCO4C1													0.00012	-1.00636	Male
MRS2	0.00000	-1.81590	Male												
WBP5	0.00108	1.24363	Female	0.00000	1.09907	Female				0.00000	-0.93489	Male	0.00000	-1.05267	Male
NLRP7				0.00000	0.75240	Female									
PHF7										0.04424	-0.80685	Male			
RGS3				0.03817	1.42913	Female									
KLF16							0.00447	0.60404	Female						
FRZB							0.00106	-4.11027	Male						
GADD45A							0.00208	0.77376	Female				0.00839	0.64020	Female
SLC35A5							0.00003	-0.82213	Male						
NFYC	0.00014	-1.00735	Male												
SLC8B1	0.00002	2.47399	Female	0.03859	0.77012	Female				0.00001	0.94310	Female	0.00868	0.59383	Female
PARD3B							0.00000	-2.89010	Male						
LRRC32										0.00033	2.46855	Female			
CELSR3							0.04075	-1.00304	Male						
BTG1										0.00003	-0.64252	Male			
LOC101929397													0.02119	1.39068	Female
MTURN							0.04261	-1.61434	Male						
CCDC120				0.00135	1.56293	Female	0.02953	0.78641	Female						
DERL3										0.01584	1.12145	Female	0.01321	1.12445	Female
LEUTX										0.00168	6.17071	Female			

TOB1							0.00008	1.27151	Female				0.01143	0.82282	Female
SLC47A1										0.00000	2.99780	Female			
SLC25A30													0.02799	1.00233	Female
NCMAP										0.00173	-1.03656	Male	0.00000	1.47679	Female
SLC12A3				0.00001	1.40430	Female				0.00200	0.69437	Female			
BRD3							0.00000	0.83357	Female						
LOC101929718							0.00746	0.68172	Female						
TEAD3										0.00204	-0.81462	Male			
TP53RK													0.00000	-0.64006	Male
EEF1A2													0.00000	2.97390	Female
TRMT6	0.00418	-0.70372	Male												
BAAT							0.02818	-2.27453	Male						
CXADRP3				0.00754	0.71369	Female									
MGAT2							0.02320	-0.61754	Male						
ZDHHC20							0.00013	-0.64250	Male						
ADORA3										0.03352	1.90203	Female			
PLCB4										0.00007	2.26740	Female			
ZNF280A										0.00000	2.60259	Female	0.00000	-1.68276	Male
ECM1	0.00077	4.10138	Female	0.02674	-1.98531	Male				0.00015	2.06517	Female	0.00094	1.82616	Female
ATP6V1B1				0.00000	2.88888	Female									
SLC7A4				0.00000	3.45268	Female							0.00259	0.76905	Female
SEPHS1	0.00651	-0.72344	Male												
H1F0				0.04536	-0.58970	Male	0.00000	1.44727	Female				0.00000	0.97052	Female
PDE4C										0.00093	3.21629	Female			
PRKAB2										0.00036	0.96703	Female			
TESK2										0.01223	0.86991	Female			
CCND2													0.00191	-2.03534	Male
KLHL5													0.01494	-1.08713	Male
RNF11													0.00027	-0.63964	Male
SUMO3	0.00013	-1.02789	Male												
SLC27A4				0.00067	0.82522	Female									
TMEM42										0.01537	-1.37102	Male			
SH3BP5-AS1							0.00002	-1.80367	Male						
WDTC1										0.00859	0.66624	Female			
NAPRT1							0.00110	-0.94296	Male						
CKMT1A							0.00188	1.27187	Female				0.00023	1.33167	Female
RAD9A													0.00452	0.66316	Female
MIR3661							0.00017	-0.79535	Male						
MOB3B				0.00000	1.27566	Female									
RAB17				0.00001	3.50244	Female				0.00004	-2.13889	Male			
ZNF773													0.01794	0.66953	Female
LINC00341										0.01325	-1.73696	Male			
HENMT1							0.00062	-0.67398	Male						
SLC44A1				0.00000	1.57729	Female									
GLS										0.00149	-0.63609	Male			
SCOC							0.00039	-0.63293	Male				0.00002	-0.69526	Male
AP3B2										0.03245	-1.80978	Male	0.03127	1.77017	Female
ARPC1A	0.02391	-0.61170	Male												
LOC729218										0.04581	0.70736	Female			
EPS8													0.04337	-0.84743	Male
N4BP2										0.00000	0.70042	Female			

NBPF10							0.00071	1.07950	Female						
FAM151A							0.02363	0.74353	Female	0.00000	2.77893	Female			
STRIP2													0.02190	0.65721	Female
POLD4										0.00518	1.06016	Female			
HDAC1				0.00002	-0.64686	Male									
TYSND1							0.01998	-0.80176	Male						
ASPH													0.00262	-0.66156	Male
RIC3													0.04523	1.01936	Female
NXT2										0.00746	0.71659	Female			
HCAR2										0.00614	1.70726	Female			
SLC7A1	0.01011	-3.14678	Male	0.00147	-2.71952	Male	0.00415	-1.75833	Male						
SPG20				0.01181	-0.79524	Male	0.00001	-0.85488	Male	0.00036	-0.68423	Male			
RBM10				0.00004	0.77545	Female									
IRAK4	0.00064	3.01683	Female				0.01447	-0.98360	Male						
PAQR5							0.01049	-1.52679	Male						
B2M										0.04926	0.90340	Female			
CD55							0.00000	-1.17545	Male						
FGFR4				0.04036	-0.69421	Male	0.00001	-0.87134	Male						
SLC30A9													0.00060	-0.60532	Male
TRIM4							0.00748	-1.14748	Male						
DPPA3							0.00000	-0.58725	Male						
DYNC1H1	0.00027	1.15653	Female												
GNAI2										0.00000	1.03890	Female			
ZP3										0.00000	-1.77672	Male	0.00098	1.22059	Female
DOCK3										0.00138	1.12752	Female			
ALOX12										0.01608	-1.03586	Male			
LPHN1										0.00003	1.10496	Female	0.00053	0.92981	Female
CCRN4L							0.00000	1.35609	Female						
MINOS1P1										0.01140	-1.09686	Male			
OTUD1										0.00036	1.24886	Female			
ALPK3													0.02009	1.24507	Female
C6orf132				0.00067	0.83040	Female									
TMEM133				0.00089	-2.49959	Male									
THAP1										0.00018	-0.60746	Male			
SNORA57				0.00007	-1.18117	Male									
AIFM1				0.00000	0.93080	Female	0.00000	0.83614	Female						
ARHGEF4							0.00687	-2.09456	Male						
KATNB1										0.01182	0.69079	Female			
LOC646762										0.03426	-0.76185	Male			
SUV420H2				0.00016	2.03697	Female									
DPP4										0.00220	1.48231	Female			
MRPL55							0.00000	0.59650	Female						
HOMER2										0.00000	2.06388	Female			
ARID1B				0.00024	1.00701	Female									
BVES							0.04699	2.05757	Female						
P2RX4				0.00803	1.49891	Female									
SENP3													0.00047	-1.48627	Male
NDST1							0.00168	-0.67650	Male						
RNF207				0.00536	1.78201	Female									
KLF11										0.00016	1.19464	Female	0.00014	1.17832	Female
MMP9										0.02373	1.79783	Female			

TREML2				0.00112	-1.83727	Male									
LRRC61				0.02144	0.92920	Female				0.00342	-0.72435	Male			
GBAS							0.00559	-0.71522	Male						
IRX4							0.02937	-1.11054	Male						
TPTE2P1										0.00007	1.96732	Female			
NEFL										0.00964	1.36910	Female			
LAS1L	0.00023	1.03320	Female	0.00000	0.92379	Female	0.00000	1.70413	Female	0.00000	0.82325	Female	0.00000	1.06904	Female
SNHG17										0.00000	0.63402	Female			
PKD1L2				0.03743	1.42338	Female									
VPS18							0.00143	-0.70288	Male						
MED10										0.00000	-0.62422	Male			
CYP27B1	0.00930	-2.60524	Male												
TMEM8A										0.00042	1.01525	Female			
LYPLA1													0.00000	-0.59215	Male
STAT3							0.00000	0.91717	Female						
LOC100128164										0.04744	-1.17714	Male			
PAPOLG							0.00166	-0.73886	Male						
TMED1							0.00010	0.65401	Female						
CGA				0.00000	-4.63811	Male	0.02006	-1.19357	Male	0.00000	-3.16567	Male	0.00883	1.39356	Female
ALOXE3							0.00178	4.58746	Female						
PUS10													0.02952	-0.75801	Male
C5orf24										0.00017	-0.72536	Male			
LAMB2							0.04835	0.96745	Female						
CBWD5.uid-11734				0.00083	0.71166	Female	0.00000	0.79295	Female						
LARP4B							0.00000	0.65316	Female						
RAB34				0.03092	-1.27361	Male	0.00000	-2.16844	Male	0.00004	-1.32565	Male			
MICU2							0.00000	-0.93676	Male				0.00000	-0.66309	Male
PFKFB4				0.04508	0.79550	Female									
TRMT10B				0.03870	1.08167	Female									
NOS3							0.00005	1.32173	Female	0.00475	0.98486	Female	0.00001	1.37778	Female
PAX8-AS1.uid-3717							0.00478	0.73375	Female						
SWSAP1							0.04516	0.60312	Female						
MAPKAP1				0.00234	0.78425	Female									
IFT172										0.00354	0.98736	Female			
PEMT	0.00170	1.64370	Female	0.00000	1.64122	Female				0.00000	1.09077	Female			
LOC101929694							0.02612	2.26663	Female						
RYR3							0.01538	-1.19553	Male						
EDIL3										0.04142	-1.66071	Male			
QTRT1							0.00102	0.58431	Female						
UBXN11													0.02135	0.65061	Female
SNORD49A				0.01973	-0.77689	Male									
IL1R1							0.04912	-1.24741	Male						
NDUFB2				0.00000	0.79115	Female									
TDH										0.00106	-2.10192	Male			
GRSF1							0.00000	-0.58481	Male						
LOC100289230													0.00707	1.58282	Female
PPP1R13L	0.00290	1.99979	Female												
MROH6							0.00019	1.34657	Female						
HUS1										0.00000	0.74581	Female			
SLC35E2										0.00544	-0.83538	Male			
AKT1S1	0.00733	1.02409	Female												

WWOX							0.01914	0.70248	Female						
ARL8B	0.03076	-0.59245	Male												
RBMY2FP	0.04045	-3.61808	Male	0.00000	-5.70147	Male	0.00049	-2.90832	Male	0.00977	-2.23021	Male	0.00001	-3.55604	Male
LRIG1										0.00068	1.41836	Female			
ZNF48										0.00886	1.04855	Female	0.00855	1.03539	Female
SPHK1							0.01271	1.00449	Female	0.00001	1.57861	Female			
EMP2							0.00001	-0.91992	Male	0.00000	-1.00402	Male			
FAM9C				0.02120	1.62968	Female									
LEMD3							0.03608	-0.64090	Male						
AQP6													0.02988	0.61426	Female
TRERF1							0.01369	-0.63439	Male						
MAGEA4				0.02641	1.37498	Female	0.00012	1.47855	Female				0.00000	2.22244	Female
PRAMEF4										0.00001	6.62271	Female			
C19orf67													0.00488	-2.75999	Male
GLYATL1													0.00121	-6.16333	Male
ACACB										0.01895	1.64463	Female			
OTUB2							0.02074	1.79359	Female						
SNORD16				0.01189	-1.17813	Male									
VMA21				0.00001	0.71230	Female	0.00000	0.58693	Female						
LOXL2													0.01369	1.25439	Female
CTDSPL										0.00037	-0.93438	Male			
RPS6KL1													0.00005	1.56353	Female
SCN2A	0.01175	2.81435	Female												
C19orf44										0.00566	-1.10035	Male			
DDX21	0.00002	-0.74541	Male												
TRAM1L1													0.00189	-0.77051	Male
CYHR1													0.00147	0.78052	Female
TMSB4X													0.00000	0.58113	Female
BMPR1A				0.02101	0.65670	Female	0.00002	-0.73871	Male				0.00000	-0.79898	Male
TUBA4B										0.04762	-0.86885	Male	0.00997	-1.04943	Male
C14orf37													0.02975	1.32696	Female
KIAA0825				0.02725	-1.03848	Male				0.00138	-0.93512	Male	0.00139	-0.88537	Male
FAM69B				0.01188	1.12664	Female	0.00680	0.79685	Female	0.00037	0.96572	Female			
SLC38A8							0.00585	2.14436	Female						
ITPR2										0.04356	1.02210	Female			
PCNA	0.00023	-0.75403	Male												
MSX1							0.00501	1.68513	Female				0.00004	2.20671	Female
HSPA6										0.00002	-3.98997	Male			
FLNB				0.00006	0.83643	Female									
BAIAP2L1							0.00001	-0.60110	Male						
WSB2							0.00025	-0.73393	Male						
APOC1P1										0.00001	1.01959	Female			
BAG3										0.00359	-0.66955	Male			
COL27A1										0.00006	1.93680	Female			
FAM83E										0.01177	-1.55708	Male			
LIN54										0.01523	-0.58158	Male			
CSPG5													0.02803	1.51002	Female
ANXA2	0.03460	0.66770	Female												
HBZ													0.01713	1.61601	Female
LGALS13										0.00267	-2.13361	Male			
OGT				0.00015	0.70275	Female				0.00000	0.95275	Female	0.00000	0.66294	Female

CXCL14													0.01806	-1.70949	Male
TCF7L2										0.00034	-0.62362	Male			
MCM3AP-AS1							0.02680	-0.78027	Male						
EPB41L2										0.00007	0.91523	Female			
UST													0.01254	-1.05932	Male
ZIC2													0.01900	-1.68934	Male
LOC101928099										0.01034	-1.06154	Male			
CD44	0.00000	-1.42113	Male												
HP1BP3	0.02265	-0.75804	Male												
EPB41L5										0.00000	0.79799	Female			
PMPCA							0.00000	0.67595	Female						
LAD1				0.00199	1.15537	Female									
C11orf95							0.00006	1.24913	Female	0.00656	0.86516	Female			
RIMS2							0.00862	2.02336	Female						
PNCK							0.00995	2.18043	Female						
EIF2S3	0.00054	0.70312	Female	0.00000	0.64675	Female	0.00000	1.07476	Female						
GABBR2										0.01230	0.99137	Female			
PTGR2													0.04643	0.75970	Female
NOL4							0.03051	2.97679	Female						
CA2	0.00009	-1.45726	Male	0.01433	0.62153	Female									
SCPEP1										0.00000	1.13300	Female			
ATAD3A				0.00000	1.05149	Female									
TCERG1	0.01539	-0.59171	Male												
RHOF							0.03784	-0.86958	Male						
STRADB							0.00165	-0.76723	Male						
YY1							0.00000	0.63404	Female						
SMPDL3A	0.01868	1.02817	Female												
LOC100506730							0.01432	-1.58616	Male						
EML3							0.00040	-0.90793	Male						
KLHL11										0.00000	1.74394	Female			
ZNF823							0.00006	-0.92833	Male						
RSPH10B+RSPH10B2.uid-9484							0.04394	-0.91502	Male						
SCO2.uid-2610	0.04168	1.15205	Female												
SCARB2										0.00447	0.63742	Female			
ENTPD5													0.00969	1.46164	Female
GSTM3													0.00262	1.13186	Female
CMTM3				0.02562	1.84065	Female									
UPP1							0.00000	0.87873	Female	0.00000	1.04249	Female	0.00000	0.76541	Female
ZHX1										0.01070	0.76399	Female			
LBH				0.02865	1.64104	Female				0.00273	1.39011	Female			
NANOG							0.03429	0.86979	Female	0.00000	2.22803	Female	0.00000	1.84032	Female
NABP2										0.00000	-1.15588	Male			
MX1										0.00040	0.97803	Female			
PEX5													0.00056	0.90316	Female
TPD52L1				0.04806	1.09286	Female	0.00310	-0.91407	Male	0.00027	-1.01940	Male	0.02005	-0.68294	Male
KLK13										0.00140	2.40690	Female			
LOC100507420.uid-10773										0.01597	1.86434	Female	0.04963	-1.56743	Male
METTL12				0.00008	-0.86587	Male									
CPPED1										0.00486	0.61858	Female			
SCAMP1										0.00000	0.83331	Female			
PARP12				0.02171	2.38023	Female				0.00025	2.36411	Female	0.00329	1.91987	Female

CTAG2	0.03393	2.66150	Female				0.02571	1.19646	Female				0.00015	1.76054	Female
ZNF841							0.04451	0.73095	Female						
HMGCR				0.00283	-0.66383	Male									
JPH3										0.00020	1.68900	Female			
SPOCK1													0.00145	-2.58235	Male
IQGAP3				0.00000	1.31083	Female				0.00001	0.73731	Female			
ATG4C							0.00675	-0.61435	Male						
AQPEP										0.00056	1.80278	Female			
REEP1	0.00200	-1.47254	Male	0.00000	1.52920	Female	0.00001	-0.76294	Male						
BRSK1							0.00004	1.13254	Female	0.00000	-1.57841	Male			
ZNF142										0.00002	0.96404	Female			
MCTP1													0.00125	-1.54587	Male
TSPY2							0.00000	-3.57098	Male	0.00000	-2.71574	Male	0.00000	-4.11341	Male
UBE2E4P	0.00012	-1.62506	Male										0.00003	-0.58999	Male
MIR205HG							0.00003	1.42232	Female						
PATL2										0.02471	-1.69106	Male			
MOCS1				0.02543	1.48569	Female									
ALPL										0.00000	1.25925	Female	0.00262	0.62729	Female
RFX1													0.00665	0.60621	Female
LAMTOR4	0.00116	0.80126	Female												
NUDT21	0.00497	-0.60365	Male												
GDF15							0.02291	1.03605	Female	0.04166	-0.90783	Male			
GTF2B							0.00000	-0.72573	Male						
MRPS31P5										0.00004	1.04104	Female			
SLC25A42													0.04219	1.20786	Female
SNORD102				0.00316	-1.09589	Male				0.00255	0.71566	Female			
MBD3L2										0.00000	7.05651	Female			
CDKN1A							0.02618	1.07485	Female						
ZFP42	0.00008	-0.87707	Male												
SRD5A3							0.01463	-0.68866	Male	0.01284	-0.65976	Male	0.00138	-0.81306	Male
CASP8										0.00005	1.56327	Female			
RARRES2										0.00032	1.78895	Female			
EZH2	0.00082	-0.97667	Male												
NRCAM										0.01341	1.68955	Female			
DUSP9							0.02811	1.12540	Female				0.00203	-1.46881	Male
AMOTL1							0.00099	-1.50792	Male						
TDRP				0.00000	2.22765	Female				0.00092	-0.82461	Male	0.00113	-0.79598	Male
FYB							0.00807	-1.02958	Male	0.04179	-0.74179	Male			
FAM83F				0.03552	1.51184	Female	0.01512	1.10531	Female						
SNHG20							0.04892	0.92142	Female						
MAGEA10													0.00645	1.67071	Female
TSIX							0.00000	3.04874	Female	0.04595	0.89237	Female	0.00000	2.82697	Female
WEE1	0.00000	-2.62183	Male												
HGS				0.03372	-0.58800	Male	0.00007	-0.66559	Male						
FZD4										0.00684	0.98773	Female			
LOC441601										0.00357	-0.64181	Male			
KDM5C				0.00001	1.29500	Female	0.00082	0.71006	Female	0.00000	1.05931	Female	0.00000	1.06142	Female
FAM102A							0.01761	-0.64913	Male	0.02581	0.58660	Female			
CSNK2A2							0.00117	0.74432	Female						
ACRC	0.00000	4.99105	Female	0.00010	2.94109	Female	0.00000	2.81186	Female	0.00005	2.09331	Female			
ZNF613							0.01810	0.99806	Female						

ABCC13										0.00000	2.91687	Female			
ARPC5	0.00268	-0.77083	Male												
POU5F1B	0.00999	-1.59507	Male							0.00000	1.77920	Female			
TAX1BP3				0.00000	1.32084	Female									
VASH2										0.03544	1.35819	Female			
MYLK-AS1							0.00302	-1.55044	Male						
CNNM2							0.01722	0.83208	Female	0.01449	-0.81218	Male			
PWARSN										0.00003	1.04306	Female			
ANKRD1										0.02090	1.10763	Female			
NDUFS6	0.00003	0.79610	Female												
LEPROTL1													0.00000	-0.58268	Male
FLJ37453							0.00354	1.21567	Female						
LINC00618													0.03770	1.78911	Female
COMMD9	0.04397	0.75660	Female												
SPPL2B							0.00001	-1.26855	Male						
HIST2H2BE										0.00056	1.66811	Female			
CEP192							0.00601	0.61869	Female						
ITGB4										0.00142	-1.45278	Male	0.01768	1.16041	Female
PRC1							0.00000	-0.65172	Male						
LOC391003.uid-852							0.00001	1.26755	Female	0.00000	1.53196	Female			
CXXC5	0.03671	2.67896	Female												
CDC25C				0.04007	-0.88468	Male	0.02972	-0.62593	Male						
GAD1													0.03920	-1.57975	Male
GCSHP3	0.03076	1.58363	Female												
APOO				0.00124	1.01531	Female	0.00721	0.60379	Female						
CXCR6							0.03207	-1.71642	Male						
AOC3										0.03537	-0.86547	Male			
SLC44A2				0.00332	0.60507	Female									
ARRDC2										0.01062	1.02977	Female			
SEPT10							0.00001	-1.07143	Male						
PSMG3	0.00337	0.78415	Female				0.00000	0.79969	Female						
USP28				0.02470	0.60989	Female									
E2F6							0.00313	-0.67759	Male						
EPAS1													0.00772	0.90454	Female
NEK7							0.03184	-0.61035	Male				0.01581	-0.62718	Male
RPL13AP20				0.00195	-0.66507	Male									
PARP16										0.00191	1.41113	Female			
AAMDC	0.02218	1.32848	Female				0.01419	0.59309	Female	0.00000	0.98690	Female			
SHPK										0.00000	1.09311	Female			
ARHGAP32				0.00927	1.18754	Female									
ZNF708				0.01056	-1.21059	Male	0.03703	-0.70461	Male						
TM7SF3							0.00163	-0.87369	Male						
PNPO										0.03920	0.74517	Female	0.01221	0.86200	Female
BRCA2													0.00049	-0.60380	Male
ZSCAN5B													0.00579	1.59029	Female
C1orf56				0.00627	-1.32081	Male									
KCNV1	0.01821	3.33147	Female												
FBXL21				0.00492	-2.13178	Male	0.00706	-1.43040	Male						
ETNPPL										0.00000	3.20840	Female			
IRX3													0.00001	1.99136	Female
SLC52A2				0.01479	0.61511	Female									

TIMP3													0.02712	-1.61370	Male
LOC643387										0.00000	-0.59558	Male			
ANKRD20A19P	0.01592	3.23334	Female	0.04999	1.86891	Female									
ZFP36L1	0.00000	2.41357	Female	0.04495	-0.59667	Male									
RMND5A							0.00000	0.68222	Female						
CTNND1							0.00000	-1.17668	Male	0.00004	-0.69175	Male			
HID1				0.03815	-2.28634	Male									
GDPD5										0.00918	0.96722	Female			
SEMA4G										0.00573	1.25847	Female			
CHIC1													0.00014	-2.74972	Male
RNF122													0.03370	-0.71898	Male
PKP3	0.02262	-1.71508	Male												
FSTL1				0.00004	2.17462	Female									
UBAC2	0.00751	-1.05892	Male												
LINC00673	0.03638	-2.32022	Male							0.00001	2.88577	Female			
RNF212							0.00348	-0.83144	Male				0.00001	-1.11993	Male
SUV39H1							0.00532	0.79437	Female						
RABAC1										0.00000	0.62514	Female			
SLC16A10										0.04024	0.85716	Female			
HPRT1				0.00005	0.93186	Female							0.00000	-0.93433	Male
LASP1	0.01145	0.77782	Female												
LZIC	0.02292	-0.78594	Male												
CELA1							0.00594	1.29248	Female				0.02737	1.01340	Female
GLT1D1				0.00005	2.70478	Female				0.03801	1.00602	Female			
ITM2B							0.00000	-0.78905	Male				0.00000	-0.81358	Male
A4GALT				0.00005	0.82022	Female									
ST6GAL1	0.00000	-1.62190	Male												
EPB41L4A-AS1				0.00654	-0.60370	Male									
GPM6A				0.00000	-2.32608	Male	0.00000	1.52798	Female	0.01928	-0.88724	Male			
BATF3	0.03913	-1.57718	Male							0.03074	0.65769	Female			
RSRP1							0.00017	0.66077	Female						
ANXA8L1										0.00012	-1.03259	Male	0.00929	0.72373	Female
BECN1P1										0.00000	4.12122	Female	0.00000	1.08415	Female
MMP14										0.01634	-0.98663	Male			
EIF2AK2							0.00447	-0.93460	Male						
ABLIM3										0.01840	-1.63833	Male			
ACAA2	0.02987	-0.77139	Male												
TFG	0.00151	-0.75998	Male												
PNMAL1										0.00317	1.56331	Female			
DYRK4										0.00373	1.16805	Female			
ROCK1P1										0.00579	-1.42279	Male	0.02787	-1.17179	Male
EBP	0.00017	0.87855	Female	0.00000	0.87029	Female	0.00000	1.21493	Female	0.00000	1.03733	Female	0.00000	0.60136	Female
ADAD1										0.00235	1.98117	Female			
C5orf66-AS1										0.04712	1.55994	Female			
CCDC122							0.02081	-1.23165	Male						
FAM65B													0.04135	1.04473	Female
PCAT4										0.00031	2.93679	Female			
ZFP62										0.00001	-1.15971	Male			
PAQR8							0.00241	-1.32859	Male						
DIP2B							0.00061	-0.65521	Male	0.00060	-0.62264	Male			
SEPT6							0.03742	1.62315	Female	0.00016	2.58010	Female			

ZNF398										0.00028	0.96603	Female			
EIF1AX	0.00008	0.71965	Female	0.00000	0.86250	Female	0.00000	0.86788	Female	0.00000	0.58826	Female			
STYK1										0.00170	2.23771	Female			
STK10	0.04221	-1.11612	Male										0.00268	0.59711	Female
P2RY6	0.00057	2.65289	Female	0.00002	-1.77736	Male				0.03594	-0.60404	Male			
COL4A1				0.00000	0.83107	Female	0.00000	-3.00033	Male	0.00092	2.26047	Female			
PTDSS2				0.00004	1.14670	Female									
ACE2										0.03734	0.85007	Female			
PSMB10	0.01224	1.32953	Female												
ARHGAP44				0.04921	1.38226	Female									
CAPN6										0.00993	-1.83462	Male			
HIST1H2AH										0.00000	2.36239	Female			
NUPR1													0.00000	2.89257	Female
IGBP1				0.00041	0.61578	Female									
SNORA55													0.01104	1.45211	Female
ZNF519													0.01741	-0.77841	Male
C16orf74							0.00155	-1.12691	Male						
G6PC3				0.00038	1.21099	Female									
MYO1G	0.00000	1.65785	Female							0.00077	2.57661	Female			
MDK										0.00000	1.14141	Female			
ITM2C							0.00168	-0.62621	Male	0.00000	1.49304	Female			
SEC23A				0.01792	-0.88200	Male									
JUNB							0.00355	0.80506	Female						
CSF3R				0.00000	3.35037	Female									
CSRNP1										0.01725	0.75578	Female	0.00681	0.82634	Female
PARM1										0.00115	2.14903	Female			
LOC286437							0.01425	1.80694	Female				0.03348	1.55613	Female
HOXC10													0.00000	3.21491	Female
CHODL										0.02039	2.04239	Female			
PDSS1							0.00062	-0.80659	Male						
TMEM31										0.01155	1.26523	Female			
PQLC1										0.00000	1.88726	Female			
ITPR3													0.04075	-0.58653	Male
AZIN2										0.00610	1.59055	Female			
ZRSR2							0.00313	0.70832	Female						
CYP1B1	0.00065	-2.03450	Male							0.00242	1.18292	Female	0.00899	1.03569	Female
MMP2										0.00000	5.44101	Female			
CASK				0.00027	1.62933	Female									
FOXRED2													0.00218	0.79712	Female
SNN													0.00392	1.41695	Female
STON2				0.00628	0.81502	Female									
REM1										0.00019	2.47928	Female			
GATA4							0.00646	-2.08075	Male						
GSTM4	0.02554	2.73012	Female	0.00003	2.53909	Female									
SF3A1	0.04441	-0.60309	Male												
WDR73				0.01654	-0.62619	Male									
OSTM1							0.00757	-0.60080	Male						
TSPAN18				0.03453	1.41975	Female				0.00000	1.96966	Female			
SIGMAR1	0.03152	1.40276	Female												
NNT-AS1	0.00000	-3.20431	Male												
PSTPIP2													0.00309	1.67071	Female

MAPT										0.02001	-1.38991	Male			
POU5F1P4	0.02391	-1.31544	Male							0.00000	1.31947	Female			
FMR1NB				0.00002	1.47030	Female	0.00003	1.02981	Female	0.00000	1.77483	Female	0.00680	0.67025	Female
ABHD17C	0.00603	-1.47703	Male										0.00066	-0.66829	Male
ALKBH7	0.01011	0.78668	Female												
COPS2	0.02338	-0.68624	Male												
CCBL2							0.00110	-0.78431	Male						
NME2.uid-2204	0.00796	-0.61755	Male												
TMEM106B							0.00125	-0.88023	Male						
TXNDC12				0.00019	0.60601	Female									
ZDHHC14				0.00000	3.01134	Female									
LOC100506790							0.00002	2.03742	Female	0.00000	4.13009	Female			
GPM6B							0.00006	1.51374	Female						
FAM162A	0.00010	1.06359	Female												
ZNF677							0.01683	-0.60810	Male						
RNASEK.uid-21	0.00279	2.31777	Female												
NFKBIA										0.00000	0.67770	Female			
TMEM92										0.01899	1.02069	Female			
MLK7-AS1							0.04599	1.05784	Female						
ZNF165							0.01712	0.60613	Female						
FAM124A										0.00000	2.67323	Female			
CNN3	0.03671	-0.69383	Male												
PRR13										0.00000	0.92895	Female			
RAB3D										0.00016	0.91866	Female	0.01523	0.62096	Female
MUC5B										0.00890	2.02696	Female	0.01265	1.91079	Female
FRMD4B													0.00014	0.64815	Female
LOC100129138							0.04306	0.69672	Female				0.00079	-0.95313	Male
CXCL12										0.01425	1.95673	Female			
GNB3													0.02135	1.02538	Female
PPP1CC													0.00000	-0.62360	Male
DCTD							0.00291	-0.68718	Male	0.00154	-0.68764	Male			
C2CD5										0.00627	0.86963	Female			
SLC20A2										0.00376	-0.79778	Male			
EVC2													0.03892	1.87096	Female
EGFLAM										0.00000	1.80899	Female			
DDX56	0.00794	-0.64434	Male												
HSD17B3										0.00411	2.53651	Female			
RXRA										0.03448	-0.66825	Male			
EPM2AIP1													0.02415	0.63666	Female
UFSP1				0.01441	2.11938	Female				0.03819	-1.23539	Male			
ADCY3				0.00739	-0.88754	Male									
C22orf29													0.00000	1.06492	Female
ID1										0.00000	1.89915	Female	0.00339	0.78277	Female
CTNNA2													0.00056	-6.58023	Male
RFC3	0.04026	-0.67263	Male												
SDCBP	0.00419	1.10173	Female	0.01400	0.60231	Female									
DKC1				0.00000	0.67080	Female	0.00000	0.76250	Female				0.00000	0.68908	Female
FUT1							0.04170	-1.03975	Male						
CDX2										0.02387	0.66398	Female			
ALG13				0.00001	0.81977	Female	0.00000	0.66479	Female				0.00000	0.65955	Female
SLK	0.03947	-0.90803	Male	0.01271	0.62621	Female									

NQO2				0.02534	0.73934	Female				0.00000	1.26018	Female			
CD38										0.00000	3.19014	Female			
PBX1										0.00009	1.88351	Female			
HTT										0.00187	0.65995	Female			
ACKR2				0.00475	-1.48309	Male	0.00000	-1.95086	Male						
RELL1	0.01948	-0.79587	Male												
TAF12	0.02218	-0.75649	Male												
SNAR-F	0.00000	-4.58776	Male	0.02370	1.31771	Female	0.00000	2.58889	Female	0.01098	0.94388	Female			
CGB7				0.04848	-1.85775	Male				0.00001	-2.40895	Male			
ERCC6L	0.01036	1.25611	Female				0.00000	0.87573	Female	0.00000	0.98921	Female	0.00200	0.58886	Female
ARAP1							0.01913	-0.60473	Male						
FGF4										0.00851	2.44255	Female			
HORMAD1													0.02456	-0.66879	Male
HECA							0.00161	-0.98986	Male						
TPM1				0.00040	0.76786	Female	0.00000	-0.95130	Male	0.00000	-0.86314	Male			
MARK1	0.03268	1.85006	Female												
CBS	0.04717	-1.46406	Male	0.00000	-2.22041	Male	0.00001	-1.10737	Male	0.00783	-0.68342	Male			
MAGI2-AS3							0.04019	1.96284	Female						
FRMD8							0.00000	-1.05877	Male						
IL23R													0.03770	-1.67375	Male
CPSF2	0.00603	-0.94414	Male												
PITPNA-AS1													0.00203	0.64913	Female
FADS2				0.00000	-2.45613	Male	0.00000	-1.73408	Male	0.00059	-1.03194	Male	0.01554	-0.74745	Male
FRRS1							0.04918	-0.96632	Male						
MAZ	0.00578	-0.77476	Male												
LINC00909										0.00664	-0.97610	Male			
SVOPL										0.03753	1.16636	Female			
GAGE2A.uid-12821				0.00033	2.79296	Female	0.00000	7.36993	Female						
KANTR										0.00000	-1.62648	Male			
LETMD1							0.00761	-0.63571	Male						
FOXRED1	0.00000	1.79149	Female												
TMEM14A													0.00000	-0.87730	Male
SMEK3P							0.00922	1.82912	Female						
TLE6													0.00012	2.23114	Female
NEK8				0.00045	1.94006	Female									
PCDH9				0.03624	-2.06493	Male									
BOLA2+BOLA2B+LOC388242+LOC613038+SLX1A+SLX1A-SULT1A3+SLX1B+							0.00000	0.89701	Female						
VOPP1							0.00264	-0.84827	Male						
ACTA2										0.00000	-2.11332	Male			
IGFBP2										0.00378	1.03595	Female			
MS4A15				0.00000	2.97443	Female									
AARS2										0.00011	0.82203	Female			
TRMT2B				0.00027	1.58037	Female	0.02610	0.72442	Female						
LOC728323							0.00384	-0.76605	Male						
ZNF589	0.00796	1.12691	Female												
GSTZ1	0.02218	1.25541	Female												
CD83										0.00213	1.89575	Female			
MARC2							0.00006	-2.54394	Male	0.00319	-1.86629	Male			
TULP2										0.00082	1.31106	Female			
DOCK9				0.01139	1.06340	Female				0.00893	-0.63623	Male			
SEC24B							0.00128	-0.62146	Male						

IPO7										0.00000	-0.67413	Male			
CACUL1							0.00000	0.80436	Female						
PCBP3										0.03012	1.96651	Female	0.00306	2.44783	Female
RRP15	0.00392	-0.70666	Male												
HMBS	0.00009	1.94241	Female												
ME1										0.00885	-1.75934	Male			
CRKL							0.00000	0.66403	Female						
ATAD3B							0.00000	0.58970	Female						
NKX3-1				0.02960	1.02341	Female	0.00000	1.91552	Female	0.01091	-0.77504	Male			
SPATA17										0.01049	1.16353	Female			
IFITM2				0.00000	3.63427	Female	0.00024	0.92238	Female	0.00000	-1.07632	Male	0.00969	0.65856	Female
DAB2IP				0.00864	1.49501	Female									
CHST2	0.01813	1.78555	Female							0.00000	1.32955	Female			
SP7	0.03013	3.07047	Female												
DGKE										0.04229	1.46493	Female			
ZSWIM4										0.01818	-0.94631	Male			
PRAP1				0.00000	1.86957	Female				0.00009	0.75350	Female			
RAB5A	0.00089	-0.93769	Male												
SYNJ2BP				0.03202	0.59358	Female	0.00000	0.95827	Female						
ANKRD37							0.00163	1.27826	Female						
PRAMEF11							0.04579	2.94680	Female	0.00082	2.74669	Female			
PVRL3										0.03885	-0.84299	Male			
HOXB4										0.03366	-0.75394	Male			
PDPR							0.02187	-0.71488	Male						
FAM184A							0.00000	-1.60649	Male				0.02603	-0.72555	Male
C1orf198										0.00381	-1.11398	Male			
KDELC2							0.00606	-1.08544	Male						
CDH3													0.01718	0.65681	Female
MSL3				0.04404	0.59955	Female	0.00102	0.60220	Female						
LOC729739				0.03507	-1.36309	Male							0.02440	0.92346	Female
TIFA													0.00143	-4.25897	Male
L1CAM				0.00059	3.14298	Female				0.01972	1.62021	Female			
RET													0.00000	2.91567	Female
LOC100130744										0.04292	-1.24223	Male			
TRNP1										0.04305	1.19552	Female			
HCCS				0.00000	0.92967	Female	0.00000	1.16878	Female						
TCEAL8				0.00002	1.34481	Female							0.00001	-0.92308	Male
BTBD2							0.02386	-0.72698	Male						
ZNF680							0.00002	-0.83075	Male						
YWHAG										0.00000	-0.65631	Male			
MPP7													0.00144	-0.87510	Male
SLC16A2	0.00000	2.94265	Female							0.00001	2.45905	Female			
ZNF596							0.02126	-1.34000	Male	0.02334	1.25958	Female			
BTG2				0.02983	1.19384	Female				0.03709	-0.70519	Male	0.03617	0.71513	Female
CHCHD3				0.00000	0.74234	Female									
PCYT2				0.04112	0.74839	Female									
NCK2							0.00599	0.58937	Female						
KLC4													0.00481	0.85463	Female
BRCC3	0.00000	2.85989	Female	0.00000	1.03153	Female									
DUSP5							0.00010	1.02977	Female						
EDA										0.01231	1.42037	Female			

HOXC13													0.02793	0.99135	Female
ECD	0.00732	-1.19985	Male												
SLC36A2							0.02321	1.21974	Female				0.03543	1.07490	Female
ENSA	0.00001	0.76718	Female												
MZT1							0.00000	-0.73652	Male						
ABCB7							0.01537	0.58125	Female						
SLC26A11				0.03454	-1.08464	Male									
BMPR2							0.01450	-0.67577	Male						
FOXK2							0.00000	0.84406	Female						
DNAJC3				0.00002	0.75140	Female									
PDCL2							0.00009	-1.25230	Male	0.01267	0.82731	Female			
APOD										0.00026	1.92723	Female	0.00007	2.03616	Female
NCR3LG1										0.04279	-2.09377	Male			
ZNF516										0.00872	1.18536	Female			
C20orf194													0.00950	0.89786	Female
KIF1A										0.02612	1.40568	Female			
HIST1H1B										0.00031	1.21980	Female			
MTRNR2L10										0.00028	0.72664	Female			
ZNF331													0.00029	0.67747	Female
NES										0.00951	2.06513	Female			
WDR11-AS1										0.00000	2.82338	Female			
SH2B2							0.00062	-1.96021	Male						
CLN5													0.00001	-0.81241	Male
TTC28				0.00903	1.44403	Female									
SLC17A5							0.00241	-0.64798	Male						
DYNLT1	0.04719	-0.63198	Male												
ARHGAP9							0.02446	1.88816	Female						
COA3	0.00266	0.94370	Female				0.00000	0.72682	Female						
FAM3A				0.03207	1.11174	Female							0.03807	0.69059	Female
ZBTB33				0.03386	0.88374	Female									
C9orf142							0.00012	-0.60224	Male						
BHMT										0.00846	1.64018	Female	0.00008	-2.31244	Male
DACH1										0.03814	1.73923	Female			
MATN2										0.00294	2.03783	Female	0.02064	1.40330	Female
ZNF664										0.00000	-0.88273	Male			
IQSEC2										0.00497	2.19265	Female			
RPL41	0.00020	0.65821	Female												
PLK2							0.03454	1.03717	Female						
GPSM3										0.00354	1.09650	Female			
STARD9													0.02626	1.18785	Female
LPCAT4				0.00577	0.81713	Female				0.00015	0.69645	Female			
FAM120AOS							0.00235	-0.62791	Male						
BCL7C				0.03815	1.09534	Female									
WNT3							0.00065	1.85423	Female	0.00000	2.59368	Female			
STK32B													0.03206	1.44447	Female
TSPAN5										0.00057	-1.74436	Male			
SNAR-H							0.00808	0.79572	Female						
RCC2	0.00259	-0.72944	Male												
GFER				0.03019	0.65268	Female									
DUSP7							0.00002	1.12414	Female	0.02716	0.62015	Female	0.03211	0.59468	Female
FREM2										0.01866	-1.30977	Male			

PAGE1	0.00683	2.59844	Female	0.00002	2.41648	Female	0.00000	2.06920	Female	0.00006	1.53339	Female			
LINC00936				0.03998	0.62734	Female									
PMEPA1										0.00416	2.57561	Female			
ARL5B													0.00010	-0.74850	Male
FAM193B				0.00024	-1.24330	Male									
SNORA19							0.00816	-0.93775	Male						
FBXL17				0.02039	2.12230	Female									
SLC6A6	0.03076	-1.39331	Male												
RAB11FIP4				0.00019	1.01101	Female									
GABRB3							0.00036	-1.08059	Male						
IDH1				0.00010	0.71458	Female				0.00000	0.58207	Female			
SFT2D2				0.04354	0.67789	Female									
LINC00630							0.02447	0.98964	Female						
SMTN				0.00255	1.29910	Female									
IFT57							0.01733	0.79868	Female						
GNPTAB										0.00140	0.59583	Female			
MIR4461							0.01810	-0.67527	Male	0.00939	0.73839	Female			
RASSF1										0.00316	-0.64555	Male			
CPOX	0.00972	-1.70678	Male												
LOC100129520				0.02564	2.38613	Female							0.02945	1.57321	Female
RTBDN										0.00232	1.49896	Female			
DGKK										0.04038	3.02899	Female			
C2CD4C													0.02332	1.18292	Female
GSN										0.00000	1.88546	Female			
TMEM243							0.00201	-1.21957	Male	0.00147	-1.17104	Male	0.00027	-1.27428	Male
CROT							0.04686	1.36960	Female	0.01983	-1.48918	Male	0.02091	-1.43676	Male
HDGFL1							0.00042	-1.61969	Male						
ATP1B2										0.01173	1.36877	Female			
ESRG							0.00178	-0.73892	Male	0.00000	1.10770	Female			
SH3GL3				0.00001	1.73790	Female	0.00001	-1.09666	Male						
TUBB4A	0.01330	1.23133	Female							0.00000	0.80307	Female			
LTBR							0.03455	-0.95007	Male						
VEGFA				0.01488	-1.42279	Male	0.04080	-0.75084	Male						
EPHA2										0.00451	-1.75362	Male	0.01648	1.50409	Female
JADE3				0.04628	0.83375	Female									
STAT6							0.03539	1.59580	Female						
CD2AP													0.00001	-0.59037	Male
LY6E				0.01933	0.77715	Female				0.00004	0.80318	Female	0.00001	0.86095	Female
LGALS2										0.00003	2.56310	Female			
CCZ1B													0.00162	-0.61880	Male
SAT1				0.01188	1.21616	Female				0.00000	2.18227	Female			
VCX3A				0.00000	4.19069	Female	0.00000	2.61251	Female	0.00002	1.65742	Female	0.00000	2.57034	Female
CLEC6A										0.00000	2.45809	Female	0.00000	2.71919	Female
ZNF204P	0.01032	3.66417	Female												
ING1							0.00340	0.65711	Female						
UNC45A										0.00285	-0.70582	Male			
PTCH1							0.01145	-1.37938	Male						
YAE1D1													0.00156	-0.64449	Male
ACVR2B										0.00019	-0.67990	Male			
TLE1				0.00000	1.64331	Female									
RCN1				0.00000	-1.32744	Male									

PRR9				0.00000	-1.53259	Male	0.00000	-1.09060	Male						
EBNA1BP2							0.00000	0.58573	Female						
IMP3	0.00009	0.74245	Female												
ECHS1				0.00000	0.77171	Female									
DOCK5										0.00858	1.47470	Female			
FAM104B				0.00959	0.72163	Female									
DSCR8				0.02674	0.74536	Female									
PITX2										0.02824	1.22217	Female			
RBM41				0.00000	2.20301	Female	0.00041	0.91811	Female	0.00000	1.09221	Female			
TRAPPC2				0.04957	0.70373	Female	0.00005	0.86097	Female	0.00157	0.65833	Female	0.00023	0.73465	Female
LOC202181										0.03113	1.30621	Female			
VCX2	0.00000	5.19443	Female	0.00000	3.99797	Female	0.00000	6.06016	Female	0.00000	4.38935	Female	0.00000	4.83440	Female
GADD45G				0.00000	3.04060	Female									
H1FOO							0.04918	1.54691	Female	0.04059	-1.59679	Male	0.00045	-2.44088	Male
SKP2	0.00139	-1.06278	Male												
EPCAM				0.00000	1.14356	Female									
CMC1	0.04271	-0.91817	Male												
PDS5B	0.00201	-1.03332	Male												
MAN1C1							0.04894	1.11969	Female	0.00000	3.00890	Female			
TOR2A										0.00091	0.96880	Female	0.00064	0.97659	Female
PTPRB										0.00611	0.86071	Female	0.02424	0.71817	Female
SLC7A3							0.01960	0.89997	Female	0.00000	1.75110	Female	0.00621	0.97857	Female
SPIN2B							0.00447	1.78290	Female						
HNF1B				0.00000	2.01882	Female									
ATP6V0C				0.00007	0.72798	Female									
LINC01030							0.01176	1.37888	Female	0.03339	1.15125	Female	0.01457	1.26573	Female
TAGAP										0.01919	-1.09511	Male			
HS6ST2										0.00339	-1.38117	Male	0.00001	-1.91228	Male
NDUFA5							0.00000	-0.61785	Male						
PRMT7				0.04090	0.58045	Female									
GLTSCR1L				0.00419	1.53107	Female									
CYB561D2							0.00022	0.59222	Female						
PARD6A				0.01604	2.48667	Female									
SLC4A8										0.00028	1.35678	Female			
DPF3							0.00089	-1.02007	Male				0.01739	0.72699	Female
ITGAV							0.01267	-1.22693	Male						
MAP4K4				0.00918	0.73229	Female									
LINC01176													0.01417	1.74983	Female
SMPD1										0.04101	0.59884	Female			
PXN-AS1				0.01772	-0.77579	Male									
MORF4L2	0.00000	1.26249	Female	0.00027	0.59818	Female	0.00000	0.59570	Female						
TRPV2							0.00004	2.13130	Female						
HDGF										0.00001	-0.61353	Male			
NUDT7													0.01476	-1.51390	Male
HIST1H4C	0.00000	1.77180	Female				0.00000	0.70689	Female						
FAM46C										0.04219	1.06613	Female			
UXT				0.00000	0.88883	Female	0.00000	0.75976	Female	0.00000	0.83842	Female	0.00000	0.84482	Female
CALCOCO1										0.00762	-0.83326	Male	0.00016	1.09677	Female
MIR8072				0.02991	-0.61047	Male									
FAM117B													0.00145	-0.71187	Male
MEIS2				0.00416	-2.54047	Male									

TBL1XR1	0.00292	-0.81580	Male												
SGPL1							0.00000	0.80693	Female				0.00001	0.58322	Female
GAL3ST4													0.02204	1.39313	Female
LOC650368							0.01103	1.58126	Female	0.03704	1.28576	Female	0.01426	1.46470	Female
KCNE3							0.00606	1.60194	Female						
ITGB7													0.00991	1.54340	Female
IGSF1										0.00852	2.10393	Female	0.00091	2.46820	Female
EIF4E1B							0.00364	2.25691	Female						
LOC102724814				0.00001	1.93581	Female									
SYNGR4							0.00117	2.26386	Female				0.01823	1.59910	Female
BRPF1							0.00045	0.75393	Female						
ARHGEF15							0.03269	-2.32872	Male						
PIN4				0.00000	1.09381	Female	0.00000	1.22479	Female	0.00000	0.88378	Female	0.00000	0.84722	Female
PKD1										0.00005	1.41296	Female			
BCAP29							0.00000	-0.98934	Male						
SP6				0.00000	-2.82669	Male	0.02474	-0.72401	Male	0.02952	-0.65802	Male			
BLOC1S3							0.00000	1.04542	Female				0.00008	0.75879	Female
MZF1							0.00425	1.08428	Female						
STT3B							0.00000	-0.64517	Male				0.00001	-0.59038	Male
GRB14													0.02070	-1.39604	Male
HNRNPCL2										0.00000	2.83108	Female			
BATF				0.00248	2.40378	Female									
POT1							0.04594	-0.64912	Male						
ZNF74	0.00457	-2.43743	Male												
ATG10	0.00817	-1.31759	Male												
C7orf13										0.00001	-2.64762	Male			
AGBL5	0.00452	2.06775	Female							0.04305	-0.64348	Male			
NUDT16L1							0.00024	0.58100	Female						
DGKD										0.02136	-0.62514	Male			
PRSS41										0.00006	1.77793	Female			
SLC36A4										0.00001	-1.69514	Male	0.01793	-0.93643	Male
ZNF704				0.00003	2.10511	Female									
OSMR										0.04985	1.43479	Female			
POLE4										0.00003	-0.78342	Male			
CD97				0.00138	1.36964	Female									
UBTD1										0.00001	1.56887	Female			
SNORD88B							0.00249	0.69240	Female						
ONECUT2													0.03039	2.65753	Female
CEP170B				0.00000	2.53342	Female							0.03660	0.77901	Female
CDH23							0.00003	2.06591	Female						
GDF3										0.00014	1.82201	Female			
FOSL1	0.00216	3.54401	Female							0.00016	-1.87358	Male			
NDUFB11	0.00000	1.14935	Female	0.00000	0.76340	Female	0.00000	0.69058	Female	0.00000	0.67624	Female	0.00000	0.63380	Female
IRS2							0.00154	2.06749	Female						
EAF2										0.01592	-0.79077	Male			
P2RY14										0.00004	-1.63919	Male			
PHLDA1										0.00000	2.06279	Female			
SLC9A3										0.00052	1.83405	Female			
ELK3										0.00259	1.19286	Female			
DMC1										0.01022	1.31143	Female	0.00979	1.29775	Female
HEG1							0.00052	-1.50156	Male				0.02091	1.01836	Female

CHAC2				0.02900	1.20472	Female									
DPY19L1										0.00090	1.17402	Female			
LARP6										0.00000	2.16409	Female			
COMMD5							0.00278	0.63788	Female						
PITX1										0.02433	0.66723	Female			
LINC00839													0.00665	-2.31897	Male
MACF1	0.01767	-1.09962	Male												
CRNDE													0.00867	-1.20037	Male
TMEM50B										0.00001	0.77716	Female			
UHRF1BP1L										0.00070	0.85379	Female			
SLC6A19										0.00000	1.16966	Female			
RBMX	0.00000	1.16621	Female												
PRKAR2B										0.01456	1.17065	Female			
LOC100420587													0.03210	1.66428	Female
HDDC2	0.00000	-1.08263	Male												
HYKK										0.04142	1.16602	Female			
CNGB3													0.01357	-5.11164	Male
LMOD3							0.00000	1.89111	Female	0.02713	-0.84195	Male			
TARS2										0.00110	0.78262	Female	0.00974	0.63423	Female
HTATSF1							0.00002	0.59960	Female						
C16orf91							0.00001	0.62732	Female						
C1orf27	0.00516	1.41439	Female												
RTFDC1	0.00064	-0.79218	Male												
PPIL1							0.00000	-0.70310	Male						
C10orf35										0.00000	-1.67283	Male			
TPM3P9													0.00005	0.76934	Female
PAPSS2										0.01326	1.69543	Female			
DONSON							0.00150	-0.68128	Male						
HNRNPU	0.00047	-0.65885	Male												
SSTR5-AS1							0.01671	-1.31253	Male	0.00027	1.79317	Female	0.00800	-1.39319	Male
C6orf201										0.00054	0.99483	Female			
UBE2D1							0.00763	-0.58314	Male						
ZDBF2							0.00538	-0.74194	Male						
SCARNA2													0.00407	1.89460	Female
ZC4H2				0.03101	1.41853	Female	0.04364	0.91327	Female						
FECH							0.00002	-1.26723	Male						
TTYH1				0.01569	2.37921	Female									
SLC4A7							0.01543	-0.81532	Male				0.04963	-0.64597	Male
ENO2										0.00000	2.06329	Female			
MGST2										0.00012	0.83852	Female	0.00077	-0.74021	Male
MT1F										0.04605	1.22458	Female			
WWC3													0.00530	0.89646	Female
RTKN2							0.00226	-0.68513	Male						
GRAMD1A										0.03745	0.94697	Female			
PRIM2													0.00010	-0.66346	Male
TMEM45B							0.02648	-0.75715	Male						
SH3D21				0.00797	1.58511	Female									
ATP10D										0.02757	-1.56050	Male			
HAGH				0.00973	1.18510	Female									
RASGEF1A										0.00000	1.73709	Female	0.01187	0.81779	Female
KLC1							0.00008	-0.83888	Male						

PLBD1							0.00074	-0.61456	Male						
FAM127C													0.00002	-1.99924	Male
CLPSL1													0.03153	1.22738	Female
GAS7													0.00625	0.63383	Female
MAD2L2										0.00000	0.91485	Female			
MRPL28							0.00000	0.76346	Female						
BIVM				0.00285	2.49506	Female									
LOC728392							0.04738	2.11402	Female						
ACSF2										0.04762	1.40308	Female			
GMDS				0.01123	1.11074	Female									
ATP2B4				0.01874	0.96534	Female							0.00021	0.88435	Female
HK1							0.00004	0.65018	Female						
APOA4										0.00291	2.17975	Female			
ABHD15	0.00034	-3.05495	Male												
NFE2				0.01139	2.14753	Female	0.00000	3.11750	Female						
COMMD1				0.00021	0.99729	Female									
ZNF593	0.00200	-0.66610	Male												
EPHB3							0.00278	2.08926	Female						
N6AMT2										0.00746	-0.89235	Male			
HEATR3							0.00663	-0.79688	Male						
SERINC5							0.00113	0.62939	Female	0.00013	0.68979	Female			
UROS							0.04373	-0.71098	Male						
HIST1H4J													0.02371	-0.73154	Male
USE1				0.00497	-0.99926	Male									
MIR372							0.00000	1.25276	Female						
CC2D1A							0.00044	0.94584	Female						
TDRD3	0.02915	-1.42458	Male												
HIST1H3G							0.00343	1.16672	Female						
MIEF1				0.00009	-0.61353	Male									
PNPLA4				0.00000	1.49364	Female	0.00000	0.98398	Female	0.00013	0.76311	Female	0.00272	0.61186	Female
ZNF98							0.03139	0.75116	Female						
NGEF										0.00000	2.40980	Female			
RPL23AP7										0.00475	-0.65951	Male			
GRHL2				0.00000	2.04254	Female									
MAPKAPK3							0.00000	-1.78538	Male						
SFXN2	0.00039	1.43956	Female												
TRIM53AP.uid-15951										0.00092	1.52771	Female			
NCF2													0.02812	1.48404	Female
OLFML1													0.01099	0.85045	Female
ESAM										0.02281	1.80326	Female			
SMO							0.03660	-2.44894	Male						
MAGEB16													0.02925	2.67994	Female
CALML4	0.04719	-1.30998	Male												
SLC1A5				0.04271	-0.67530	Male	0.00015	-0.75813	Male						
UGP2	0.00074	-0.84530	Male												
PLXNB2				0.01410	1.06542	Female				0.00344	0.80434	Female	0.02284	0.64412	Female
C4orf48				0.00880	0.78542	Female									
SRSF6	0.00619	-0.65613	Male												
ZC3H7B				0.00888	1.36867	Female									
CEP55							0.02409	-0.62095	Male	0.00238	-0.75474	Male			
MAGEB1							0.03641	1.05182	Female						

LYPD6B	0.02263	-2.23979	Male							0.00305	1.43964	Female			
SCGB3A2										0.00000	2.04432	Female			
NFKBIB				0.02277	-0.62497	Male									
UPRT													0.00664	-1.16550	Male
LINC01021							0.00601	1.13316	Female						
FAM89A							0.00427	-1.25115	Male	0.00052	-1.38270	Male			
IL36RN										0.00068	-2.43418	Male			
ACP5										0.03636	0.59825	Female			
EPHA1-AS1							0.04275	1.29922	Female						
DHCR7							0.00194	-0.67015	Male						
HSD17B10	0.00000	1.21690	Female	0.00000	0.92630	Female	0.00000	0.87437	Female	0.00000	0.74702	Female	0.00000	1.24506	Female
RPIA										0.00000	-0.93906	Male			
SLC9A2													0.00424	-2.81156	Male
WDR44							0.00385	0.79244	Female	0.00003	1.02963	Female			
MPP1				0.00612	1.03350	Female									
MAGEB2							0.00000	1.34729	Female				0.00063	0.77539	Female
FIRRE										0.00540	0.72280	Female	0.00000	-1.41878	Male
DLG5										0.00000	-0.76748	Male			
SPRYD3				0.00387	-1.45206	Male									
TMEM99							0.00309	-0.73858	Male	0.00397	-0.69461	Male	0.00914	-0.62965	Male
ROBO3							0.00258	-2.28182	Male						
PALM										0.02898	-1.55706	Male			
TWIST1										0.03803	1.12975	Female			
LOC440416													0.01251	0.73022	Female
GNAL							0.03241	0.77998	Female						
PROSER2-AS1										0.00014	0.85251	Female			
RILPL2							0.00469	-1.33004	Male	0.00030	-1.52569	Male			
AGAP3				0.00143	1.18286	Female									
ALKBH6	0.00450	2.11891	Female												
BMS1							0.00000	0.59143	Female						
PRNCR1	0.00748	-0.66495	Male												
TOMM20				0.00000	-0.59053	Male									
SCN1A													0.00331	-3.31540	Male
TMEM180							0.00733	0.60281	Female						
COLGALT1							0.00000	0.72272	Female						
OSGEPL1													0.02769	-0.59301	Male
PANK4				0.00991	1.26591	Female	0.00882	0.86955	Female						
EGFL7	0.00086	2.27823	Female							0.01463	0.64648	Female	0.01237	0.65221	Female
GPR75							0.00010	-1.98341	Male						
MAGOHB	0.00003	-1.07942	Male												
TRIM61	0.00749	-0.99341	Male							0.00000	0.83900	Female			
ANO6				0.00045	0.95496	Female									
NEK6				0.00001	1.48989	Female									
DCAF11	0.02070	1.52310	Female												
SHMT2				0.00001	-0.78898	Male									
MGC12916							0.04611	1.71791	Female						
MST4										0.01623	-0.82391	Male	0.00000	-1.73566	Male
CPTP							0.03924	0.73602	Female						
CCNI2													0.00365	1.25227	Female
FBRSL1													0.01108	0.75930	Female
ZBTB20													0.00004	-0.73359	Male

DOCK8										0.01090	-1.48529	Male			
RPL13AP3										0.00000	-1.30295	Male			
HIST1H2BM													0.01075	-1.38727	Male
MRPS26				0.00000	1.12526	Female									
DDIT4L													0.00220	-0.87663	Male
IL13RA1										0.00429	1.73639	Female			
SOD2	0.00008	-1.22171	Male	0.00058	0.67681	Female	0.00000	0.76818	Female				0.00000	0.62322	Female
MFSD2A							0.02360	-1.38889	Male	0.00000	3.55932	Female			
FASN							0.00291	-0.62572	Male						
ACTN4	0.00925	0.84451	Female												
HIST1H4H							0.02674	1.26197	Female	0.01107	1.34292	Female			
HSDL2							0.00113	-0.73213	Male						
HIST1H2BD										0.00020	1.59095	Female			
NEFH										0.00011	0.83378	Female			
ZMYM6NB										0.00001	1.50492	Female			
ZNF729										0.00132	0.79224	Female			
JPX				0.00000	1.05019	Female				0.00000	1.20135	Female			
ZNF280D							0.03249	-1.30679	Male						
FAM49A										0.01632	-1.19062	Male			
SPOP							0.00136	0.66940	Female						
WFDC2													0.00000	2.15822	Female
ANKRD6				0.00050	-1.57327	Male									
BDH1				0.01930	2.41239	Female									
KDM4E										0.00000	2.87887	Female			
ZNF57										0.00147	0.61040	Female			
EXOSC6				0.00000	1.13737	Female	0.00000	0.94659	Female						
POC1B				0.00182	1.35509	Female									
MFAP2							0.00637	1.38927	Female	0.00001	1.99722	Female			
LYN	0.03485	1.47477	Female	0.00000	1.51511	Female									
PDP1							0.00641	0.84387	Female						
SULT2B1							0.00035	2.29841	Female	0.00708	1.73342	Female			
MT1M													0.01634	1.68353	Female
CTSD				0.01201	0.64776	Female	0.00001	-0.71666	Male						
CASZ1													0.02561	1.78025	Female
CTSE	0.00000	1.19979	Female	0.00000	0.94255	Female									
SEPT7	0.00224	-0.67111	Male												
HSH2D				0.00045	-1.08816	Male									
SLC25A53							0.00123	1.04580	Female	0.00346	0.93181	Female	0.00004	1.16862	Female
CCDC109B				0.00005	1.25995	Female	0.00900	-0.60774	Male						
PDE6G										0.00001	2.44979	Female			
FAM45A							0.02617	0.64338	Female	0.02894	0.69156	Female			
FPGS							0.00455	-0.60953	Male						
MMAB													0.00857	0.61834	Female
FOXK1							0.00107	0.58624	Female				0.00000	0.78637	Female
TMEM14C							0.00000	-0.69900	Male						
IQCG													0.01051	0.86390	Female
ADAMTSL4				0.00036	-1.87877	Male									
RHOB													0.00064	0.66087	Female
TFAP2A										0.00009	-0.79189	Male			
FAM227B				0.01748	-1.82267	Male									
DCLRE1B							0.00691	-0.59703	Male						

HLA-C										0.04800	-0.83116	Male			
CENPI				0.02385	1.17886	Female				0.02109	0.77846	Female			
ZFP14							0.02111	-1.36155	Male						
FMO2							0.00001	-2.64114	Male						
LIAS	0.03081	-0.99733	Male												
NCOR2										0.00000	1.22070	Female			
LIG4													0.02750	-0.76717	Male
EPB41L3				0.00085	-1.49282	Male									
C1orf106				0.00000	1.41179	Female									
PAGE5				0.00298	1.52542	Female	0.00000	2.13505	Female	0.00000	2.17903	Female			
GLRX5										0.00000	-0.66853	Male			
TUSC1										0.03369	-0.74356	Male			
FAM53C				0.01827	-0.63845	Male									
GATA2				0.00000	2.53942	Female									
RGS14										0.01332	1.20080	Female			
HLA-G				0.03197	-2.27195	Male									
FLJ32255										0.04561	-2.30523	Male			
ZNF586													0.00212	0.61522	Female
FGFR3										0.00037	0.73830	Female	0.00432	0.60615	Female
MRPS6							0.00000	-0.62705	Male						
USP9Y	0.00000	-4.07012	Male	0.00000	-7.32548	Male	0.00000	-5.83612	Male	0.00000	-6.08672	Male	0.00000	-6.49154	Male
TIMP2							0.00025	-1.87394	Male						
C4orf19							0.00384	-0.84382	Male	0.03750	-0.60626	Male			
DNAJB4							0.00140	-0.80849	Male						
SEMA4B										0.00544	0.74816	Female			
HSD17B1	0.00460	1.85061	Female												
ABCC5	0.01314	0.99128	Female												
VCAN										0.01946	1.23510	Female			
SLFN13													0.03842	1.79405	Female
FGFR1										0.00000	1.59192	Female			
COTL1							0.00087	1.12471	Female	0.00520	0.91938	Female	0.01818	0.79539	Female
NFYB							0.00000	-0.66067	Male						
FAM162B										0.02256	1.10109	Female			
TRIM56										0.00422	1.35101	Female			
MATK													0.03636	-1.95610	Male
APOBEC3F										0.00314	1.73195	Female			
MEIS3													0.00181	2.11559	Female
C16orf62										0.01165	0.62096	Female	0.00046	0.80746	Female
CDK2	0.01101	1.12088	Female	0.01139	-0.69597	Male									
ZNF544										0.00138	-0.74887	Male			
DNMT3B				0.00000	2.08602	Female									
STX4							0.01862	0.67554	Female						
PHKA1	0.01630	1.51064	Female	0.00000	1.82137	Female	0.00000	1.03884	Female	0.00610	0.60012	Female			
PITRM1-AS1										0.02894	-0.67993	Male			
CORO2A				0.00014	1.19127	Female									
NONO	0.00033	0.65083	Female	0.00000	0.76484	Female	0.00000	0.72548	Female						
MTRNR2L1				0.01834	-1.29948	Male				0.00001	-1.47781	Male			
NUDT4							0.01975	0.68105	Female						
BMP2K										0.03461	0.61513	Female			
HRH1				0.02089	-2.06782	Male									
SH2D1B										0.00438	2.43757	Female			

TUSC3							0.00349	-0.83806	Male						
ACCSL							0.03858	1.82255	Female				0.00000	-4.19578	Male
LAGE3				0.00000	1.25951	Female				0.00034	0.61159	Female			
BRIX1	0.00052	-0.81178	Male												
FBLIM1				0.01898	1.82028	Female									
NUP93				0.00000	0.77769	Female									
GSTA3							0.03767	1.05039	Female						
VAT1							0.00000	-0.61606	Male						
ZAR1L							0.03305	1.79115	Female				0.00346	-2.40498	Male
GPRC5C	0.00037	-2.92719	Male							0.00000	1.87684	Female			
LOC100287792										0.00000	-1.03239	Male	0.00330	-0.63680	Male
MLXIP										0.00001	0.86401	Female			
FAM98B													0.00661	-0.60666	Male
PRPF39				0.00010	0.92106	Female									
SOCS6				0.01403	0.92649	Female									
KIAA1671										0.00068	1.07849	Female			
FCHO1							0.00000	2.02267	Female						
CDR1							0.00144	-0.86623	Male				0.00583	0.72528	Female
PIM1													0.00059	0.59967	Female
FUZ	0.01750	-1.84067	Male							0.01472	-0.76354	Male			
UGCG							0.00131	-0.65448	Male						
ST8SIA1										0.00637	-2.47972	Male			
PSMD5													0.01652	-0.60173	Male
CREBL2										0.00013	1.13349	Female			
ZSCAN16							0.04835	-0.99785	Male						
ADA				0.00000	2.26907	Female									
CSNK2A3	0.01160	-0.76956	Male												
GAPDHS													0.00131	1.85690	Female
HIST1H1C				0.00113	-1.31244	Male									
NBN													0.00083	-0.58869	Male
B4GALT1				0.00000	1.27957	Female	0.00000	0.92796	Female						
CMTM8							0.02185	-0.98692	Male						
ENTPD6	0.00000	-2.50537	Male	0.00000	1.53813	Female									
SERTAD4	0.02380	-2.24661	Male							0.00049	-1.52745	Male			
HES4							0.00138	-2.33218	Male						
TMEM170B							0.01610	-2.47956	Male						
WWC2	0.00834	-1.15135	Male												
NREP				0.01284	-1.94449	Male									
CLCC1							0.00297	-0.78093	Male						
MASP2							0.00000	1.56058	Female				0.02863	0.69227	Female
APOBEC3D										0.00064	2.21779	Female			
PDHA1				0.00000	0.94346	Female	0.00000	0.96411	Female	0.00000	0.67905	Female	0.00000	0.64429	Female
FMR1				0.00006	1.17155	Female							0.00313	0.59649	Female
LINC00273							0.00006	-1.07862	Male						
COQ5										0.00008	0.90781	Female			
SH3PXD2A										0.00000	2.00006	Female			
STS				0.00000	1.87705	Female							0.00009	0.90126	Female
NPNT				0.00008	-2.87258	Male									
DNAJC30	0.02203	1.37066	Female												
SOCS3	0.03152	-1.98441	Male				0.00000	1.76829	Female						
SMAD5-AS1													0.02330	1.10712	Female

CDH11	0.00000	1.11363	Female	0.03769	-0.68201	Male									
SBSPON							0.00818	-1.25113	Male						
SAP30													0.00001	-1.42698	Male
SMLR1										0.03462	1.79096	Female	0.01243	-2.23889	Male
CLDN19	0.00000	2.36421	Female							0.00000	2.21277	Female			
TLE3				0.00000	1.42824	Female									
RMI2													0.04647	-0.74675	Male
SLC5A3							0.02269	-0.58421	Male						
RNF187				0.00000	1.12238	Female									
SMPD4							0.00000	-0.81345	Male						
TMTC4							0.00051	-1.18289	Male				0.04164	-0.70789	Male
OCA2										0.00218	1.82452	Female			
PTPLB										0.00649	-0.73565	Male	0.01253	-0.66819	Male
ACOT8										0.00039	0.79852	Female			
RNF113A	0.00007	1.43071	Female	0.00001	0.99373	Female	0.00000	1.23424	Female	0.00000	0.86450	Female	0.00000	0.89060	Female
CASP10										0.00156	1.71841	Female			
SGCE							0.00016	1.80652	Female						
SOGA1													0.02803	1.09571	Female
DYNC2LI1										0.04239	0.72702	Female			
PINK1										0.00000	1.61398	Female			
KDM5D	0.00000	-3.65515	Male	0.00000	-9.29016	Male	0.00000	-8.54804	Male	0.00000	-6.81853	Male	0.00000	-6.13763	Male
LMBR1				0.03260	0.81860	Female	0.00266	-0.72491	Male						
SIX5	0.04258	-1.84949	Male	0.01799	1.32070	Female									
GPR137C				0.01514	2.93608	Female									
GRIPAP1							0.00002	0.87595	Female						
PITPNC1				0.00000	1.49191	Female									
GPR157							0.00174	-2.07970	Male						
EEA1										0.00000	0.63294	Female			
BOK-AS1				0.01069	-2.18306	Male	0.03484	1.29955	Female						
DCBLD1							0.03958	-0.88021	Male						
PCNT										0.00000	0.64294	Female			
RGL4													0.01839	1.44048	Female
ETHE1				0.02758	0.77736	Female				0.00000	1.21760	Female			
PITHD1							0.00000	-0.61941	Male						
DNAH11													0.00652	-1.91477	Male
LOC374443													0.01316	-0.65043	Male
MRAS										0.01323	1.49501	Female	0.02951	1.32359	Female
MYCT1							0.04526	-0.64241	Male						
HARS							0.00000	0.62918	Female						
HAND1				0.00000	-2.13463	Male	0.00992	-0.95309	Male				0.00007	1.30134	Female
XAGE3				0.00000	2.46433	Female	0.00000	2.03920	Female						
AMPD2							0.01585	-0.75570	Male						
NRXN2										0.00723	0.92486	Female			
TMSB4Y				0.00000	-2.98467	Male	0.00000	-2.68284	Male	0.00000	-4.29379	Male	0.00000	-5.03917	Male
TRIM38										0.00000	2.44214	Female			
LOC101927873										0.00813	1.82856	Female			
SOX2										0.00008	1.58045	Female			
OPLAH							0.00027	-2.63837	Male						
SATB1							0.00000	-3.45919	Male	0.00000	-1.80558	Male			
SNORD83B							0.00996	-0.93330	Male						
C3orf56										0.00032	-2.70965	Male			

NKAPP1										0.01295	1.32081	Female			
GAGE12J	0.01217	2.44617	Female	0.00001	2.23773	Female	0.00000	4.26257	Female				0.00016	-1.32364	Male
HSD17B8							0.00008	-2.15343	Male						
DPPA5										0.00000	1.91754	Female			
FGFR1OP2				0.00098	-0.76053	Male									
TMEM209							0.00000	-0.83899	Male						
ETV5										0.03845	0.74955	Female			
PTGR1				0.00001	1.33524	Female									
MYLK3										0.01647	-1.61182	Male			
RPS6KA2										0.00020	2.41150	Female			
MOSPD3							0.00003	-1.34726	Male						
MAGEC2	0.00003	5.25505	Female	0.00000	7.78050	Female	0.00000	7.25890	Female	0.00000	2.90823	Female	0.00000	7.28963	Female
ARHGEF11										0.00265	0.87295	Female			
MKRN1										0.00000	0.61839	Female			
GDPD2	0.00000	0.76925	Female	0.00014	2.11075	Female	0.01023	1.05797	Female	0.00000	3.62059	Female	0.01102	1.00792	Female
SNORD65				0.03678	-0.87671	Male	0.00000	-1.22508	Male						
LOC646471	0.03979	1.81657	Female												
TMEM125				0.03466	-2.13931	Male									
NT5DC2				0.00000	-2.86361	Male				0.00096	-1.12955	Male			
UBA1	0.00001	0.94694	Female	0.00000	0.94345	Female	0.00000	0.84940	Female				0.00000	0.66089	Female
PTK2	0.00751	-1.07256	Male												
CLEC11A													0.00005	1.47012	Female
BTBD18							0.04948	1.70783	Female						
POU2F3				0.04722	2.10540	Female									
ARSD				0.03353	2.31774	Female									
CHMP4B				0.03004	-2.63156	Male				0.00000	4.70545	Female			
AP4E1										0.00300	-0.63231	Male			
RND2										0.00000	1.42275	Female			
NEURL1										0.01473	-1.11568	Male			
LAMA1	0.00105	1.13328	Female	0.00130	0.66534	Female									
MAP7D1										0.04027	-0.75519	Male			
ST3GAL6	0.02380	-1.79263	Male												
AVIL							0.01463	1.61529	Female						
TST				0.00725	1.58431	Female									
CCAR1	0.00331	-0.61859	Male												
DEFB124				0.03199	2.03027	Female				0.00479	1.70395	Female			
CCNG2													0.01270	-0.95536	Male
ADORA2B	0.02826	-2.05814	Male												
SNORA66				0.03763	-1.19911	Male							0.00981	0.89798	Female
SNORD17							0.00000	0.76072	Female						
PLEKHM2				0.03114	0.71261	Female									
LRP5L				0.00367	-2.74284	Male									
ALDH7A1				0.00103	-0.84527	Male									
MIR6817							0.00022	1.01993	Female	0.01204	-0.75359	Male	0.00887	0.71880	Female
FAM20B	0.00011	1.95847	Female												
CAPG										0.00000	1.63520	Female			
NAV2													0.01409	0.74767	Female
HNRNPA1P33				0.01676	1.17623	Female				0.01230	0.74490	Female			
MEX3A										0.00000	-1.58806	Male			
LINC00260													0.03592	1.54019	Female
OSGIN1										0.01980	-2.11610	Male			

MAP3K1													0.04737	-0.63082	Male
SMC1A				0.00000	0.97815	Female	0.00000	0.83991	Female	0.00000	0.90950	Female	0.00000	0.96211	Female
APOBR							0.00099	2.23150	Female	0.00000	3.07456	Female	0.01917	1.59173	Female
MRPL23				0.00943	0.83965	Female									
PRAF2							0.00198	1.23674	Female				0.03661	0.83581	Female
NDUFAF3										0.00076	0.68384	Female			
PRR19										0.02203	-0.69963	Male	0.02699	0.67067	Female
SNHG15										0.00000	0.71458	Female			
GPR1-AS	0.00822	2.26289	Female												
RAB2A													0.00000	-0.69501	Male
CCPG1										0.00006	0.86832	Female			
MTRNR2L2										0.00000	0.90820	Female			
PTGES2										0.00000	0.96987	Female			
OAZ2										0.00500	-0.64951	Male			
PHACTR3							0.01919	1.69092	Female						
SHROOM3				0.00146	0.99262	Female									
MMACHC													0.00204	0.73444	Female
PHKA2							0.00000	1.49703	Female	0.01454	0.82449	Female			
SYNE3							0.03018	-1.97862	Male						
MPST										0.00001	-0.85513	Male			
SNORA76C													0.01094	0.83518	Female
KIF18B							0.00393	-0.66858	Male						
ELAVL1	0.00258	-1.07741	Male												
MIIP	0.03789	-0.60417	Male												
SNX10							0.00016	-1.32165	Male						
VPS51							0.00669	-0.68534	Male						
SSX3										0.00000	2.89104	Female			
SCNN1A										0.00156	1.67242	Female			
C7orf50	0.03493	-0.73998	Male												
GSC										0.00000	3.20498	Female			
MDH1B													0.00139	1.94338	Female
SLC31A1	0.00802	0.92088	Female												
SNORA70							0.00000	1.07480	Female	0.00000	1.26609	Female	0.00000	1.02506	Female
PRAMEF2							0.00000	3.77385	Female	0.00000	5.95855	Female	0.00028	1.54098	Female
TNFRSF25							0.02413	1.68495	Female	0.00000	2.97508	Female			
WDR87							0.02374	0.65566	Female						
TRIM71							0.00072	0.58300	Female						
RAB3B				0.00001	-1.70407	Male	0.00000	-1.50159	Male						
LOC101929549										0.00013	1.87640	Female			
SLC16A9										0.00000	2.36036	Female			
TMED7							0.00000	-0.74001	Male						
STXBP1													0.03236	1.03152	Female
XIAP				0.00045	0.83645	Female	0.00000	0.82104	Female						
C1orf85							0.00184	-0.80217	Male						
ANKMY1	0.04350	2.77950	Female												
KIT										0.00236	1.41549	Female			
APBA3	0.00216	2.30209	Female				0.01470	-0.74450	Male						
TUBA3C										0.00000	2.97349	Female	0.01700	1.24072	Female
SRRM5							0.03426	1.30300	Female						
MLYCD				0.04187	1.76546	Female									
CREBRF													0.00525	1.02410	Female

UBLCP1				0.01056	-0.78369	Male	0.00001	-0.86833	Male				0.00024	-0.67363	Male
TARS	0.00749	-0.70396	Male												
ATF5							0.00000	0.85442	Female						
EBF2							0.04577	2.19163	Female						
ATG3										0.00000	0.66812	Female			
NSMAF				0.02987	0.65555	Female									
PHLDA3				0.00024	1.54433	Female	0.00579	0.84144	Female						
KMT2E-AS1							0.01470	0.60892	Female				0.01289	0.58025	Female
CAMSAP3				0.00005	2.00998	Female									
ZNF703				0.00149	1.19434	Female				0.00000	1.07526	Female	0.00000	1.24225	Female
ABHD14A	0.00132	-3.21363	Male												
MAML1				0.01400	-0.79387	Male	0.00048	-0.72056	Male						
POTEF							0.01554	1.03359	Female	0.00000	-2.58486	Male	0.00166	1.18985	Female
SMAD9										0.00363	0.80992	Female			
UNC119										0.02263	0.83950	Female			
VTCN1													0.04923	1.10157	Female
SNX33							0.01245	-1.27375	Male						
ARSE										0.00355	1.58062	Female			
MIR6790				0.02091	-0.63549	Male									
TMEM74B										0.00222	1.92485	Female			
GUCA1A										0.00028	2.14468	Female			
MZT2B										0.00036	-0.62842	Male			
RAD54B													0.00762	-0.59330	Male
IRF2BPL				0.02960	0.59534	Female									
DENND2A										0.00000	2.71107	Female			
RNPEPL1				0.00023	1.93296	Female									
ZZEF1							0.00242	0.73668	Female				0.00080	0.75610	Female
CDC26				0.00179	-0.59935	Male									
ABCC6							0.00905	1.80109	Female						
PXDN										0.00094	2.02317	Female			
BMP2										0.00002	2.53132	Female			
SP8										0.00228	1.41972	Female			
RAP2B	0.03391	-0.63500	Male				0.00000	0.59302	Female						
LYPD2										0.03710	-2.20761	Male			
AP4S1										0.01740	-0.84652	Male			
SNAP29				0.02029	0.70658	Female									
RITA1	0.01749	1.18994	Female												
IQUB													0.04141	-1.58716	Male
HLA-E							0.00147	0.62617	Female						
TVP23C.uid-20973							0.01094	-0.73558	Male	0.03050	0.64116	Female			
SEMA3F													0.00234	1.30900	Female
IFNLR1										0.03288	1.50388	Female			
LY6G6C							0.00606	-1.53302	Male						
BOLA1				0.01834	-0.89785	Male	0.00614	-0.68933	Male	0.00000	-1.35994	Male	0.00154	-0.73112	Male
FBXO30										0.00864	-0.69167	Male			
PHGDH				0.00000	-2.03432	Male	0.00044	-1.08084	Male	0.04863	-0.63043	Male			
RPS7P5										0.00000	-3.85426	Male			
NHLRC2										0.00348	0.66901	Female			
NF2	0.00045	-1.02999	Male												
WDR66													0.00076	-0.60371	Male
FILIP1							0.01789	-0.60432	Male						

CCHCR1	0.01125	-1.76082	Male												
SDHAP2										0.00293	0.66791	Female	0.00198	0.66759	Female
DENND3													0.02451	1.67172	Female
TRAPPC5	0.00012	1.09720	Female				0.00000	0.66296	Female						
NUDT2	0.00914	1.47097	Female												
NCBP2-AS2							0.00000	0.74189	Female						
PRTG							0.00297	-0.89691	Male						
PDCD7							0.00000	0.87454	Female						
DUSP19													0.00085	1.74105	Female
ZSCAN16-AS1	0.00014	3.15673	Female										0.02001	0.88180	Female
TTC39B										0.00003	1.73056	Female			
FGD4										0.00093	0.88765	Female			
LOC101927284										0.00001	-1.19800	Male	0.00004	-1.05764	Male
SLC35A1				0.00038	1.18253	Female				0.00484	0.64440	Female			
LOC158960							0.00049	1.32419	Female	0.00195	1.15897	Female			
SEZ6L2										0.00037	0.84448	Female			
S1PR3	0.00718	3.21334	Female												
IRAK2							0.01599	-1.51632	Male						
FAM195B							0.00003	-1.05490	Male						
GLIPR1										0.00038	-0.97917	Male	0.00000	1.31533	Female
NDRG2										0.01718	1.18636	Female			
LINC00657				0.00641	0.66051	Female	0.00002	0.65486	Female						
HIST1H2BB							0.01371	1.79784	Female						
LRG1	0.01072	3.64235	Female							0.00128	1.98613	Female			
SGSM1													0.00060	2.48824	Female
NEDD1				0.01398	-0.71905	Male									
ZNF655							0.00083	-0.76546	Male	0.00000	-1.03551	Male			
WBP1L							0.00016	0.68007	Female				0.00018	0.63485	Female
ARFGEF1										0.00004	0.59433	Female			
CLCN4										0.00436	-2.30550	Male	0.00005	-3.07360	Male
KIAA1958				0.03998	1.04049	Female									
FAM216A										0.00042	-1.06551	Male			
KHDC1L										0.00000	1.19393	Female			
DHRS3	0.00000	4.22044	Female							0.00195	1.04621	Female			
ATL3										0.00010	0.78304	Female			
CACNA2D1										0.00031	1.92498	Female			
KSR1													0.02803	0.84290	Female
ZFPM2													0.00096	-2.24967	Male
MARCH9	0.01568	2.76225	Female												
ACOT9							0.00558	0.97591	Female						
RNFT2	0.02115	2.15343	Female												
MGST1							0.00044	-0.92054	Male				0.00151	-0.78758	Male
MTRNR2L9										0.00000	1.08303	Female			
ZMAT2										0.00000	-0.61181	Male			
FAR2													0.00769	2.02414	Female
ATP13A2				0.00127	0.94910	Female				0.00002	0.78856	Female			
LOC100506844							0.00497	2.05233	Female						
ADARB1													0.00033	1.20607	Female
SPPL2A													0.00000	-0.62738	Male
ECEL1P2				0.00330	1.89021	Female	0.00000	-2.74113	Male	0.00000	-2.25895	Male			
ZNF524							0.03191	-1.06412	Male						

THOC7							0.00000	-0.65559	Male						
HSPB1										0.00416	0.73874	Female			
AIFM2	0.02819	-2.05874	Male	0.00068	1.91455	Female									
SLC39A10										0.00004	0.76024	Female			
LINC00605										0.00000	3.15241	Female			
COA6	0.00241	-0.68110	Male												
SNX12				0.01646	0.90002	Female	0.02000	0.59454	Female				0.00001	0.94103	Female
GNAQ	0.04481	-1.14746	Male												
TBC1D4							0.02125	1.39800	Female						
MICU3				0.03288	-1.81220	Male				0.02952	-1.16692	Male	0.03418	-1.11367	Male
EPPK1				0.00000	1.67623	Female	0.00000	1.12066	Female	0.00001	1.03864	Female			
CCNY				0.00027	0.99966	Female									
TAAR5				0.00679	-1.20999	Male	0.00622	0.85300	Female	0.00000	-1.47562	Male	0.00000	-3.42031	Male
TMEM144													0.01552	-1.12697	Male
CRELD1										0.00444	0.77187	Female			
DNHD1													0.03892	0.71197	Female
AIG1							0.03300	-1.52724	Male						
SLC25A13				0.00025	0.97831	Female									
BAMBI										0.00000	2.62920	Female			
LRRC37BP1										0.02325	0.64867	Female	0.01020	0.70454	Female
CYP26A1													0.00144	1.57427	Female
LINC00200													0.02509	1.56643	Female
IRAK1							0.00014	1.15070	Female	0.00456	-0.85368	Male			
EDNRB	0.00010	2.61617	Female												
PLEC				0.00000	1.64802	Female				0.00006	0.78265	Female			
RHOV	0.00000	0.84452	Female	0.02277	1.16289	Female									
BAIAP2L2										0.00000	1.91818	Female	0.00047	1.44980	Female
FABP5				0.00007	0.82310	Female									
MFI2										0.00500	2.37263	Female			
TMTC1							0.01933	-1.89142	Male						
DGKB							0.04174	-1.96088	Male						
EDEM1										0.00595	0.63610	Female			
SYCE1										0.00486	1.28021	Female	0.00007	1.67737	Female
MTHFS.uid-196	0.04306	1.14213	Female							0.00036	0.61083	Female			
PPAPDC1B	0.00349	2.62083	Female												
IPW										0.01610	0.75943	Female			
TRIO							0.00278	0.89041	Female						
DAGLA										0.00005	2.53503	Female			
BCL6							0.00093	-1.43167	Male	0.00011	-1.53772	Male			
SGMS1							0.00000	-1.27759	Male				0.00248	-0.64957	Male
SYAP1	0.00233	0.77579	Female	0.00002	0.62832	Female	0.00000	1.06123	Female	0.00000	0.65838	Female			
MMP15										0.00002	1.82553	Female			
HOMER3										0.00364	1.88699	Female			
PP14571							0.03742	-1.19849	Male						
CYP51A1-AS1													0.04287	0.81373	Female
JADE2				0.00000	2.99838	Female				0.00000	1.91281	Female	0.01525	0.98341	Female
MBTD1										0.01467	-0.64700	Male			
LONRF2													0.04807	2.35242	Female
PRR12				0.04834	-1.07565	Male									
SCAMP5													0.01232	1.37447	Female
ZNF608										0.03462	1.24255	Female			

HRASLS5										0.00404	0.96172	Female	0.00068	1.09139	Female
ZMIZ1	0.00516	1.40192	Female	0.00001	1.09544	Female									
GABARAP	0.00000	1.49995	Female	0.00006	-0.65006	Male									
TMEM184B							0.00003	-0.82795	Male						
TUBB2A										0.00000	1.38263	Female			
TOP3A							0.01862	0.59565	Female						
BCAR4										0.00885	-1.04300	Male			
LOC401242										0.00000	-3.48675	Male			
MXRA7	0.04508	1.57892	Female	0.01507	1.03043	Female	0.01275	-0.70779	Male						
FBXO11				0.00316	0.80448	Female									
IGDCC3				0.04563	1.41853	Female									
RBPMS2				0.00133	0.68203	Female				0.00000	0.83535	Female			
SFR1							0.01754	-0.64320	Male				0.00505	-0.70113	Male
XBP1	0.04126	-1.01377	Male												
COL26A1										0.04821	1.47293	Female			
MED18										0.00216	-0.60447	Male			
TPTEP1										0.00000	0.63641	Female			
ZFYVE1													0.00012	0.87075	Female
HOXC13-AS				0.03291	-2.41919	Male									
TTN							0.00246	-0.66194	Male						
C1orf159	0.00200	-2.15517	Male												
NMI				0.01664	-1.36695	Male	0.02045	0.75482	Female						
ZCCHC11				0.02046	-0.91275	Male									
DNALI1							0.00002	-1.80268	Male	0.00528	-1.16765	Male			
TIGD5	0.04955	2.54259	Female	0.00204	2.11875	Female									
NSUN7							0.03013	-0.81019	Male	0.04690	-0.72325	Male			
PHF21B							0.01824	1.26854	Female				0.00130	1.56456	Female
PIGM							0.04399	-0.78633	Male						
HIST1H3I										0.00317	-1.14891	Male			
KRT80										0.00000	-2.86760	Male			
COMMD8													0.00004	-0.66172	Male
ARAP2													0.02724	-1.72930	Male
HCLS1				0.02925	-1.31325	Male									
SORBS3										0.03150	0.94626	Female			
RAB11FIP3										0.00388	-0.81567	Male			
TBX19				0.00032	2.84414	Female									
RHNO1							0.00137	-0.72058	Male						
SH2B1										0.00913	0.70030	Female	0.03304	0.58234	Female
CARS2				0.03930	1.00988	Female									
FUOM				0.00000	1.62133	Female									
HEY2										0.00071	1.70687	Female	0.02990	1.14674	Female
TUBA3D							0.00028	1.13056	Female				0.00000	1.52102	Female
RB1										0.03543	-0.63652	Male			
ANKRD9				0.02425	1.30949	Female									
ZNF311										0.02116	1.50068	Female			
KITLG													0.01442	-1.09152	Male
DUXA										0.00000	2.65777	Female			
TMEM63B										0.00395	-0.76873	Male			
WDR18	0.00388	-0.88695	Male				0.00000	-0.58449	Male						
15.00000	0.03996	-1.12846	Male										0.00000	-0.85573	Male
CLEC2D							0.00034	-0.75818	Male				0.00004	-0.75631	Male

DBNDD2										0.00486	-0.73337	Male			
CACNA2D2										0.00001	1.74183	Female			
ADAMTSL3							0.04130	-1.34027	Male						
SYVN1							0.00015	-0.82644	Male						
BTBD3				0.02109	-0.89067	Male									
LCP1							0.00187	-0.92010	Male						
OGDH	0.02522	0.91448	Female												
ALG1L										0.04214	1.09695	Female			
JUND				0.00595	0.83831	Female							0.00001	0.78663	Female
CCDC171										0.02045	1.41830	Female			
PRAME										0.00004	1.97099	Female	0.00000	3.26499	Female
MXRA8													0.02990	-0.78772	Male
TRG-AS1													0.04217	1.76225	Female
PURA										0.00007	-0.94826	Male			
MTRF1													0.00140	-0.62852	Male
NRN1L				0.01967	1.58731	Female	0.00649	1.19885	Female						
HAUS6	0.00594	-0.90979	Male												
TSPAN6	0.00053	1.29458	Female	0.00113	0.73138	Female									
ZFY	0.00006	-3.42423	Male	0.00000	-5.94546	Male	0.00000	-6.65757	Male	0.00000	-7.08029	Male	0.00000	-7.27079	Male
MEX3D										0.01725	-0.60493	Male			
CREB3L1				0.00252	2.57730	Female				0.00001	2.44555	Female			
TPI1	0.00043	0.67091	Female	0.00000	0.89481	Female									
CDK4	0.02160	0.60816	Female												
FN1										0.04766	0.84140	Female	0.00310	-1.15887	Male
PGPEP1													0.00489	0.95387	Female
SPINK1										0.02485	1.34236	Female	0.00495	-1.61269	Male
KLF7										0.00651	1.86659	Female			
BTLA										0.01359	1.46545	Female			
IFFO2										0.03250	-1.67147	Male			
OCIAD2				0.01307	-2.53576	Male									
PKIA				0.00184	-2.25623	Male				0.00018	-1.70595	Male	0.01629	-1.11241	Male
KIF21A				0.00001	1.61332	Female									
DENND1A				0.04772	0.94170	Female									
ATF4				0.00000	-0.90521	Male									
UTP20	0.02822	-0.74291	Male												
MRPS35	0.02000	-0.60555	Male												
ABHD13							0.01645	-0.62727	Male				0.01777	-0.58117	Male
MRPS10	0.00007	-1.04179	Male												
DROSHA				0.00001	1.05271	Female									
FOXD1							0.00965	1.97265	Female	0.02835	-1.65832	Male			
HSPB8	0.00000	1.24730	Female	0.00000	-1.35724	Male									
DUSP6	0.00147	-2.31497	Male												
PSAT1				0.01640	-0.85876	Male	0.00000	-1.10664	Male						
APTR							0.00074	-0.81728	Male						
LOC100505817							0.01271	-0.85119	Male				0.02715	-0.72677	Male
SSBP3							0.00000	0.78636	Female						
NPAS1										0.01244	1.20613	Female			
CAB39				0.00000	1.38890	Female									
ANPEP	0.00000	2.07824	Female	0.00000	1.54957	Female				0.00000	1.04063	Female			
GPR37L1										0.00213	1.53183	Female			
CNN2										0.00528	-0.62680	Male			

CNOT6L				0.00135	1.12989	Female				0.00070	-0.78396	Male			
SLC30A2				0.00017	2.72795	Female				0.02391	1.20114	Female			
PPL										0.03935	-0.99192	Male			
PLEKHG1													0.00966	1.89213	Female
EN1													0.00000	-1.64045	Male
XYLT2										0.02164	0.68064	Female			
LOC729603				0.04331	1.45633	Female									
RARS	0.00412	-0.72964	Male												
SRC							0.00001	-1.65045	Male	0.01758	-0.88880	Male			
TEX11										0.00000	1.96961	Female	0.00000	1.68319	Female
TFF1										0.03733	1.51526	Female			
FAM127A				0.00022	1.07230	Female	0.00000	1.10141	Female						
ECHDC2										0.00251	-0.63312	Male			
MEI1													0.01054	1.03364	Female
OXR1													0.01289	-0.71839	Male
FAM60A	0.01022	-0.61663	Male												
CASP3	0.02822	-0.84213	Male												
SLC30A6							0.00001	-0.75557	Male						
CAPN13										0.01456	1.65129	Female			
CD200										0.00984	1.85631	Female			
FARP1				0.00002	1.64183	Female									
NUDT19							0.02183	1.11850	Female						
BAGE										0.00735	-1.16773	Male			
C10orf95							0.00099	0.75763	Female	0.00198	0.73022	Female	0.00162	0.69436	Female
GPR160										0.00000	1.16512	Female			
FUCA1										0.00001	0.84970	Female			
FGF12													0.02325	-1.17115	Male
RAB20				0.00000	2.03580	Female									
LETM1	0.00828	-0.78405	Male				0.00000	0.64895	Female						
CSTA							0.00316	-1.96537	Male	0.01955	1.56029	Female			
PHLDA2	0.00089	-1.49988	Male				0.00000	1.05439	Female	0.00000	0.84501	Female			
ESYT3							0.03824	-1.84773	Male						
GGCX				0.00000	1.54653	Female									
ANKRD35							0.00000	-4.07778	Male						
COLEC12							0.00054	-0.88020	Male						
C6orf99				0.00339	1.26098	Female							0.00232	0.81717	Female
ZMYM4				0.02983	0.71394	Female									
ALX1													0.04137	-1.18607	Male
GOLGA8A	0.04258	1.32417	Female				0.02186	-0.59416	Male						
PIN4P1				0.00000	1.14841	Female	0.00000	1.48207	Female	0.00000	0.89177	Female	0.00000	0.78510	Female
GADD45B													0.02338	0.89669	Female
NID2							0.00017	-1.98402	Male						
SLC25A37										0.00306	-0.62818	Male			
TAF1							0.00094	0.86570	Female	0.01230	0.65630	Female	0.00500	0.71224	Female
B4GALT2							0.02381	-0.92262	Male	0.03854	-0.81222	Male			
KLK8				0.01816	-1.01310	Male	0.00932	0.74270	Female	0.00000	1.48466	Female			
SLC38A1							0.00278	-0.87063	Male	0.00076	-0.90442	Male			
RUFY2							0.01551	-0.63820	Male	0.00174	0.75783	Female			
MTMR11										0.03923	1.79559	Female			
TEX40										0.01590	1.02157	Female			
ZSCAN26										0.01892	1.45708	Female			

SEC14L1P1													0.00654	-0.90835	Male
NEDD4L										0.03891	0.65667	Female			
CDK5RAP3										0.00494	0.62474	Female			
MANF										0.00000	-0.78100	Male			
MAGEA12							0.04610	2.23485	Female				0.00489	1.99260	Female
MBTPS2				0.03716	1.31942	Female	0.00278	1.16190	Female						
ATP1A1-AS1	0.02475	-1.03704	Male												
HSD17B7				0.00000	-1.97216	Male									
COCH				0.00016	1.81060	Female	0.00000	-2.06396	Male	0.00003	-1.26227	Male	0.01598	-0.77068	Male
WEE2							0.04534	1.51814	Female	0.00494	-1.95250	Male	0.00018	-2.46507	Male
DENND2C							0.03120	-1.50885	Male						
GAGE2B							0.00000	3.60793	Female						
MFSD3							0.00290	-1.33290	Male						
C6orf57													0.00789	-1.03429	Male
TNFAIP2							0.00405	1.79639	Female						
MANBA	0.02282	1.88786	Female												
RALGDS				0.00437	1.84977	Female									
RPL13A							0.00000	-0.61038	Male						
CCDC80							0.01964	1.30450	Female	0.00000	2.34262	Female			
MAN2B1							0.01518	-0.76946	Male						
CHD7	0.04384	-0.72110	Male												
ZNF34										0.04414	1.01950	Female			
PODXL										0.00654	0.84077	Female			
DIAPH1	0.04441	-0.82131	Male												
POLB							0.00003	-0.69559	Male						
APOC3										0.00563	1.24704	Female	0.00002	1.77724	Female
SLC1A3	0.00712	-1.68389	Male												
NAALAD2							0.00032	0.94521	Female						
HBQ1										0.03020	-1.81345	Male			
SYTL2													0.00511	-1.89138	Male
EPHA4										0.00156	2.12743	Female			
HAUS7				0.00001	1.10838	Female	0.00000	1.45036	Female				0.00000	0.79962	Female
NME1	0.00088	-0.59767	Male												
HPSE										0.00160	1.82086	Female			
GMPR2	0.00264	1.34736	Female												
SLC22A11				0.00061	2.58237	Female									
ERICH1-AS1							0.00046	-2.34046	Male						
KCNK12										0.00994	2.19892	Female			
TOMM40L				0.03537	1.23004	Female									
ZNF440				0.00004	2.05034	Female	0.02111	-0.83544	Male						
DBN1							0.00732	-0.61594	Male	0.00764	0.58705	Female			
DEPTOR										0.00000	1.80300	Female			
SLC7A11-AS1										0.01249	1.56295	Female	0.00370	-1.75109	Male
TYK2	0.04423	-1.38371	Male												
ASPHD1													0.00978	1.41654	Female
BCAP31				0.00000	0.95324	Female	0.00000	0.96767	Female						
GNG7										0.01741	1.63857	Female			
IMPAD1													0.00001	-0.65135	Male
SPRY4										0.00000	1.88233	Female	0.04068	0.86478	Female
MORN2							0.00107	-0.67465	Male						
MYL12A	0.00000	-0.94118	Male												

ATP5G2							0.00000	-0.61314	Male						
DGUOK-AS1	0.03121	-1.29776	Male												
BCOR							0.00340	0.80108	Female	0.00000	1.61311	Female			
MND1							0.00001	-0.76556	Male						
ASGR2										0.03158	2.01909	Female			
AASS							0.01160	-0.76130	Male	0.00251	-0.83467	Male			
TRIM54													0.00196	2.03536	Female
IRF6							0.01338	-0.93305	Male						
ACD													0.00006	0.62669	Female
MIAT							0.00777	1.69421	Female	0.00004	2.24994	Female			
DDIT3													0.00001	0.95776	Female
HLA-F				0.01549	1.21879	Female				0.00000	1.96216	Female	0.00140	0.98772	Female
PRKY				0.00000	-5.45828	Male	0.00000	-6.00501	Male	0.00000	-7.01340	Male	0.00000	-7.13151	Male
HN1L										0.00000	-0.65321	Male			
RASIP1							0.01776	-1.54653	Male						
SCRN2				0.04090	-1.45130	Male									
RHOC				0.00000	0.94790	Female									
ZNF503							0.00032	2.53333	Female						
MED31				0.00001	-1.05998	Male									
ZBTB6										0.04968	-0.71868	Male	0.04710	-0.71392	Male
DOT1L										0.00000	0.77329	Female			
SMYD3													0.00574	0.72791	Female
TPPP3													0.03059	-2.13382	Male
TMEM129	0.02488	-2.12059	Male												
MTRNR2L3				0.00056	-1.27798	Male				0.00894	0.72449	Female			
SEH1L	0.00828	0.59762	Female												
PRR14				0.01275	-0.79347	Male									
SLC35E4										0.00000	1.53152	Female			
LRRC37A11P													0.04953	0.98312	Female
HIST1H2AI										0.00000	-2.73474	Male			
TUBD1	0.01855	-1.23821	Male												
CABIN1	0.02109	1.24810	Female				0.00484	0.60988	Female						
PRKX				0.00000	1.47020	Female	0.00000	1.42473	Female	0.00004	0.82006	Female	0.00000	1.05850	Female
GRIN2D							0.03670	1.01936	Female				0.03266	0.97713	Female
IFNAR2							0.00283	-0.99219	Male						
BPNT1							0.00021	-0.91211	Male						
REEP4							0.00066	-0.58164	Male						
OCRL										0.00026	1.28437	Female			
PROS1										0.02894	1.21613	Female			
FBXO36										0.00191	0.83560	Female			
WDR72							0.00704	-1.09146	Male				0.00001	-1.56390	Male
APOA2										0.04410	1.04247	Female	0.01519	1.20440	Female
ST14				0.00000	1.69884	Female									
NUCB1				0.00000	0.87447	Female				0.00000	0.59470	Female			
MSH6										0.00000	0.63795	Female			
PHF5A				0.00000	-0.97125	Male									
PTPRD										0.00088	2.40184	Female			
PHACTR1										0.03845	1.10940	Female			
CD276	0.00000	-2.14791	Male												
CHMP4C				0.00007	-2.19807	Male									
ICOSLG										0.00239	0.88687	Female	0.00047	0.98256	Female

PVR				0.00033	1.16908	Female									
LOC101927151							0.00386	0.81477	Female	0.02231	-0.64252	Male			
FOS													0.00000	2.00518	Female
RPS6KA4	0.01192	2.10199	Female	0.00000	1.99562	Female							0.02536	0.60524	Female
BAG1				0.00000	1.06976	Female									
HRH2							0.00039	2.71369	Female						
SLC37A4							0.00010	-0.71265	Male						
SSR4				0.00000	1.14034	Female	0.00000	0.91369	Female	0.00000	0.64060	Female			
FASTKD2	0.00915	-0.87378	Male												
LEP							0.02817	1.18989	Female	0.00491	1.38775	Female	0.03279	1.10773	Female
NDUFS5	0.00000	1.11903	Female												
TREX1				0.04657	1.61892	Female									
GGPS1							0.00002	-0.77017	Male	0.00015	-0.66842	Male			
LOC388553							0.00777	1.61114	Female				0.04229	1.22402	Female
SLC12A9				0.00564	1.22181	Female	0.00918	-0.78578	Male						
DBP										0.00005	1.64686	Female			
WDR34							0.00000	-0.81255	Male						
PAGE2				0.00000	2.44745	Female	0.00000	2.80843	Female	0.00002	1.08795	Female	0.01844	0.64185	Female
SAYSD1							0.00113	0.87093	Female						
BCL11A				0.01569	1.30781	Female	0.01114	0.93055	Female	0.00060	1.16365	Female			
HMGN5										0.00377	0.79634	Female			
SNX25										0.01955	1.14743	Female			
SLC6A9													0.02989	0.90683	Female
SEPSECS													0.02240	-0.78431	Male
TEX35													0.02553	1.91467	Female
PLA2G10										0.00000	1.79873	Female			
FAM173A	0.01700	1.64983	Female	0.00233	1.02742	Female									
KIF19							0.01268	-5.41309	Male						
CCDC84	0.00269	1.31404	Female												
PNRC1							0.00486	0.71112	Female						
DRAP1				0.00202	0.63830	Female				0.00000	0.67928	Female			
CRTAP				0.00486	0.90956	Female									
FUCA2				0.00008	1.35023	Female									
ACTG1P4							0.02704	-0.67122	Male	0.00310	-0.84027	Male			
SRPK2	0.01507	-0.65517	Male												
CGB5				0.00005	-3.03851	Male				0.00000	-3.01220	Male			
OLR1							0.01953	-0.66374	Male						
YPEL5										0.00000	-0.67431	Male			
PAX5										0.00432	2.03111	Female			
VMO1													0.00421	1.39782	Female
PLCB3										0.00003	0.77382	Female			
GRINA													0.00005	0.84784	Female
XAB2							0.00001	-0.63371	Male				0.00000	0.88002	Female
FST				0.01943	1.86995	Female	0.00639	-1.41947	Male						
LRP1										0.00131	0.77476	Female	0.00516	0.68103	Female
DYRK1B													0.02715	0.96468	Female
U2AF1L4							0.00000	1.05493	Female	0.00517	0.59889	Female			
PRELP										0.01277	1.98931	Female	0.02750	0.86337	Female
FBXL19							0.03519	-0.95420	Male						
SEMA6D				0.01999	1.25495	Female							0.00393	-0.92590	Male
TENM1													0.00007	3.37879	Female

DPEP1													0.00530	1.82939	Female
CRIP1	0.00003	2.07415	Female	0.00000	1.74658	Female							0.00000	0.72321	Female
IQSEC1				0.00001	1.38626	Female									
RNF149							0.00830	-0.61680	Male						
ACOXL				0.00075	1.37234	Female									
ACBD7													0.02460	1.54881	Female
NLGN3							0.01022	2.16612	Female						
JOSD2							0.00100	-0.94404	Male	0.01167	-0.71452	Male			
ARGFXP2										0.00040	1.20483	Female	0.00030	1.20901	Female
MBNL3	0.01211	1.71008	Female							0.00002	-1.02729	Male			
ABCA1				0.00001	2.01531	Female				0.00000	1.56885	Female			
LOC100130899				0.01367	1.85372	Female	0.00380	1.44143	Female						
HDAC10													0.03423	1.06118	Female
PSD3													0.03252	-0.90518	Male
HESX1										0.00455	1.74914	Female			
SUN2							0.01501	-0.73469	Male						
CDC42EP4										0.00000	1.18698	Female			
RPS26P11							0.00000	0.93173	Female						
SSBP3-AS1										0.01507	1.18778	Female			
LOC646214	0.00275	0.95435	Female												
ADPRH										0.01272	-2.84054	Male			
ETS1										0.00057	2.26028	Female			
ZNF518A	0.02265	-0.82319	Male												
FTSJ1				0.00005	0.96030	Female	0.00001	0.70834	Female	0.00000	0.78038	Female	0.00010	0.60740	Female
GALNT14							0.00791	1.84071	Female						
GUSBP5										0.00578	1.44682	Female			
ZCCHC3							0.00521	0.75051	Female						
ST5				0.01569	1.26225	Female									
FRMD8P1							0.00000	-1.98648	Male						
RNF7	0.00508	0.71623	Female												
BBS5													0.02328	0.97637	Female
ZNF689													0.00994	0.63084	Female
PADI6							0.00305	2.16765	Female	0.00536	-1.90773	Male	0.00002	2.63057	Female
LOC101926892													0.02455	1.86423	Female
ABHD12				0.00027	0.93121	Female									
CSGALNACT1				0.01380	1.56915	Female									
FAM101B				0.00000	1.98187	Female									
LOC100506548				0.00596	-0.84631	Male	0.00315	-0.61857	Male						
FLRT3							0.02895	-2.56620	Male						
LOC100289019							0.03089	0.85380	Female						
LINC00501							0.03334	1.31869	Female				0.00612	-1.57134	Male
COQ3										0.02580	0.69917	Female			
KIAA0368				0.00278	0.70725	Female									
SLC2A1	0.00000	1.71285	Female	0.01009	0.64856	Female									
SMYD2				0.04701	0.60115	Female	0.00001	-0.78973	Male						
VPS13C										0.02397	0.88204	Female			
VAMP2										0.00044	-0.76841	Male			
DYM				0.03202	0.73027	Female									
CRYBG3							0.00675	-2.09331	Male						
PPFIA3				0.01201	1.46486	Female									
GATA6-AS1	0.00006	-2.59968	Male												

LXN	0.04089	-2.63969	Male												
TCN2										0.00614	1.62384	Female			
ADAMTS5							0.00180	2.02940	Female						
NBAS										0.00029	0.74579	Female			
ANGPT1													0.01943	-0.64101	Male
CDCA7L										0.03587	1.36680	Female			
OFD1				0.00001	0.68196	Female	0.00000	0.85297	Female	0.00000	0.68821	Female			
TIPRL	0.02237	-0.67618	Male												
TYMSOS										0.00336	0.97931	Female			
PI4KAP2							0.00000	0.82476	Female				0.00000	0.76687	Female
RRP36	0.04178	-0.86954	Male												
EXTL2													0.00009	-0.88353	Male
HIST1H1D	0.03963	-1.70650	Male	0.00006	-1.79849	Male				0.00007	1.20378	Female			
FAM208B				0.00153	0.84123	Female									
C3orf33													0.01563	-1.29455	Male
DPP6							0.00000	-2.39717	Male						
HIST2H2BA							0.03091	1.43777	Female						
NLRC4							0.00099	1.73664	Female						
NSL1	0.04136	-0.81348	Male												
DDB1										0.00000	-0.58304	Male			
UBXN8	0.03706	-1.06400	Male				0.00050	-0.67041	Male				0.00001	-0.78934	Male
CDK16				0.00000	1.09000	Female									
PCDH17				0.01407	2.17297	Female									
ZNF787				0.00397	1.26084	Female									
PARP14										0.00724	2.00399	Female			
ALDH6A1	0.02942	1.21271	Female												
CDKN2AIPNL										0.00043	0.58694	Female			
ATF3				0.02695	-0.87683	Male							0.00018	0.86404	Female
MIR4458HG	0.02523	-2.01206	Male												
NSDHL	0.02218	0.77575	Female	0.00000	0.96774	Female	0.00000	0.98937	Female	0.00000	0.94191	Female	0.00000	0.59874	Female
ITGA6				0.00244	1.02468	Female				0.00000	1.07578	Female			
LEF1							0.02663	2.96568	Female				0.02252	-4.86570	Male
RAC2										0.00315	1.74156	Female			
PHF1							0.01446	-0.96732	Male						
MTM1				0.04513	1.13440	Female	0.02610	0.82996	Female	0.01869	0.82908	Female			
SETD8							0.00000	0.73927	Female						
SYNPO2L										0.00668	-1.98250	Male			
OBSCN							0.00221	2.89051	Female						
SLC35D2							0.01520	-0.94405	Male						
GFRA3										0.01634	1.97826	Female			
THEM6										0.00003	-2.50896	Male			
MPHOSPH6	0.02621	-0.65303	Male												
EEPD1										0.00315	-2.03323	Male			
LGALS1										0.00000	1.33339	Female			
VGLL1				0.00000	-0.67923	Male				0.00118	-0.86564	Male			
FLJ42627							0.01667	1.15859	Female						
PLLP										0.00004	1.54523	Female			
KIAA0930				0.00002	1.48818	Female									
PWWP2B				0.00125	1.51671	Female				0.02260	-0.64674	Male			
ANKRD40							0.00000	0.75238	Female						
DPRX										0.01506	1.15694	Female	0.00166	1.40050	Female

CERS5	0.00376	-0.85169	Male												
ACTG2				0.00106	1.80997	Female									
L3HYPDH										0.00404	-0.75066	Male			
MARC1							0.04921	-0.98791	Male				0.04928	-0.89645	Male
MTOR				0.00048	0.74020	Female									
SPP1	0.00000	2.24261	Female							0.00033	2.51087	Female			
FMN1													0.03984	-0.94830	Male
PIM2	0.04148	1.37524	Female				0.00000	1.10452	Female	0.00000	2.00017	Female			
ANXA6				0.00097	-1.06673	Male									
DOCK7				0.01976	0.98790	Female									
UBE2E3	0.00396	-0.99529	Male										0.00000	-0.63396	Male
PAQR4				0.01188	0.99606	Female									
SMEK2				0.00148	-0.59254	Male									
LINC00261							0.00000	-4.64942	Male						
EFNB1							0.04109	1.51195	Female						
ACER3										0.01306	-0.58668	Male			
PQBP1	0.00619	0.69379	Female	0.00000	0.88048	Female	0.00000	0.87685	Female	0.00000	0.60670	Female			
FOXN3				0.00000	1.25706	Female									
NDRG1				0.01654	1.46069	Female									
ELK1										0.01905	0.63518	Female	0.00406	0.74061	Female
PRSS16							0.00004	-1.72161	Male						
KLF3	0.00010	-1.79419	Male										0.00068	-0.66277	Male
EBPL							0.00000	-1.04589	Male				0.00004	-0.71230	Male
MSI2										0.04707	-0.80779	Male			
PRKCQ-AS1							0.03218	-0.94804	Male						
RORA							0.03142	0.63956	Female	0.00000	-1.22444	Male	0.00623	-0.74197	Male
ZNF24	0.00828	-0.93887	Male												
SOAT2										0.00036	1.17478	Female			
ATP1A3													0.00009	2.36482	Female
CLIC1	0.00000	-0.72617	Male												
TRIM2	0.00036	2.76841	Female										0.00010	-1.03784	Male
AOX1										0.00162	1.83134	Female			
RENBP										0.00741	-2.25259	Male			
SDF2				0.00010	-0.86247	Male									
GPC4				0.00213	2.99074	Female									
MAGOH2	0.00963	-0.58603	Male												
TDGF1P3										0.00002	1.69590	Female	0.00099	1.34359	Female
UROD	0.00231	0.96013	Female												
SOX9										0.00476	2.09199	Female			
TNFSF9							0.00002	1.66737	Female	0.00001	1.70075	Female	0.00000	2.47222	Female
CYP2F1										0.00107	1.82407	Female	0.01212	1.43783	Female
NAB1													0.00464	-0.90450	Male
SC5D							0.00963	-0.67150	Male				0.00969	-0.62488	Male
IL17RC										0.00497	1.89461	Female			
SELM										0.04808	0.68657	Female			
ACN9	0.03519	0.69564	Female												
CENPH							0.00002	-0.77049	Male						
RPL36A.uid-13034							0.01063	0.80716	Female				0.00781	0.78517	Female
MTMR1				0.01829	1.13932	Female									
CHST12										0.00000	1.09040	Female			
C17orf75										0.00711	0.74004	Female			

DHCR24	0.00822	0.98926	Female												
KRTCAP3										0.01880	0.60478	Female			
MEAF6	0.03758	-0.66720	Male												
FIBCD1				0.00000	3.91663	Female									
SUSD3				0.00724	2.85349	Female				0.00708	1.96956	Female			
PLAC8	0.00166	2.07203	Female	0.01078	0.94996	Female									
CEBPA				0.00000	1.67493	Female	0.00997	0.63796	Female						
C6orf147										0.00407	-0.74896	Male			
REG1A										0.03859	2.36803	Female			
CHCHD10				0.00083	0.90469	Female									
NCOA1										0.00027	0.85855	Female			
FAM222A							0.00537	1.21488	Female						
SLC25A44										0.00000	0.60245	Female			
RFX2							0.02350	-1.11224	Male						
PSD4										0.04666	-1.23322	Male			
FXYD5	0.00113	2.70634	Female				0.00045	1.10926	Female						
KIAA0226L	0.01604	-3.37964	Male												
PRPSAP2	0.00861	-0.99575	Male												
TMEM159										0.00000	2.07951	Female			
FDXR													0.00001	0.86940	Female
DNASE1L3										0.01989	2.34632	Female			
TRIP6							0.00144	-0.99580	Male	0.00097	-0.95299	Male			
ACTL8										0.00555	-1.81141	Male	0.00046	-2.21113	Male
ATXN7L2													0.02853	0.76409	Female
MIR1292													0.00001	0.59070	Female
RNF24										0.00027	1.06963	Female			
PPAP2A										0.00000	2.26898	Female	0.00596	0.82186	Female
LTBP4										0.00237	0.82829	Female			
EPS8L2				0.01320	1.15339	Female	0.02984	-0.66942	Male						
C1orf61				0.00019	-1.43237	Male									
GLI3				0.00401	3.21107	Female									
SIAE				0.00923	1.27517	Female				0.04994	0.65834	Female			
GREM2	0.00065	3.20853	Female				0.00021	-1.41277	Male						
PPP1R14D													0.00495	1.48494	Female
PFN2							0.00000	-0.84827	Male	0.00000	-1.07662	Male			
SULT2A1							0.01295	1.76207	Female						
TP53										0.00002	-0.83030	Male			
NOC4L				0.00001	0.98619	Female	0.00000	1.26750	Female						
PDCD11							0.00000	0.63892	Female						
DCUN1D1										0.00008	-0.58761	Male			
PRPSAP1										0.00000	0.86595	Female			
DUSP1													0.04984	0.74449	Female
MIR6834										0.00003	-1.06738	Male	0.00025	0.93879	Female
FOXR1							0.00189	0.69435	Female	0.00000	1.04389	Female			
HERC2P2										0.00668	0.92750	Female			
IDH2										0.00094	-1.06583	Male			
DHRS11													0.01844	0.91946	Female
UHMK1							0.00000	0.86077	Female						
EI24				0.00110	0.59585	Female									
ATP6AP1				0.00000	1.18104	Female	0.00000	0.97668	Female	0.00000	0.64424	Female	0.00000	0.61299	Female
PDP2										0.00000	-1.22373	Male			

TRIM53AP.uid-15946										0.00784	1.46138	Female			
MYLK							0.03826	-1.05245	Male						
LOC643355										0.00001	-1.41518	Male	0.00304	-0.96111	Male
LGR5													0.04929	-2.47594	Male
CDH2													0.00193	-1.63105	Male
NENF							0.00000	-1.17842	Male	0.00914	-0.61451	Male			
PIDD1							0.00389	-0.96968	Male						
EDA2R	0.00003	4.03111	Female				0.04159	0.95864	Female						
PI4KA	0.00085	1.36158	Female												
FAM199X				0.00001	1.35239	Female				0.00001	0.89025	Female			
FOXD3-AS1										0.02236	1.12313	Female			
PARK2										0.00000	2.22313	Female			
PRR14L				0.00304	-0.63811	Male									
IVNS1ABP										0.00181	-0.66266	Male			
NGFRAP1	0.00000	2.26138	Female							0.00000	-1.40130	Male	0.00099	-0.63128	Male
LINC00907													0.00825	-1.71084	Male
PGF										0.00003	-1.14361	Male			
ARHGEF16										0.04240	0.71054	Female			
FBXO6										0.00000	2.51053	Female			
XIST				0.02960	0.95411	Female	0.00000	2.12944	Female	0.00000	1.97173	Female	0.00000	3.34972	Female
SLC34A2				0.02051	-0.96851	Male				0.00256	-0.78026	Male			
NAT1													0.04548	-0.83230	Male
TLN1													0.01552	0.58412	Female
YTHDF1	0.00330	-0.81458	Male												
TUBA8										0.01176	-0.98552	Male	0.00203	1.16451	Female
RASAL2							0.01845	0.91359	Female						
NLGN2							0.00398	-1.38154	Male						
GBX2										0.00224	1.37607	Female			
GIN1							0.00120	-1.17959	Male						
B3GNT2							0.00000	-1.02302	Male	0.00190	-0.58315	Male			
CCDC106							0.00926	-1.11569	Male						
LAPTM4B										0.00000	0.81473	Female			
SLC52A1													0.01089	0.83235	Female
PPA1	0.00044	-0.71158	Male												
IL10RB							0.00546	-0.84261	Male						
TMEM183A													0.00024	-1.62682	Male
SYNPO							0.00579	-1.22772	Male	0.02442	-0.94054	Male	0.00511	1.15430	Female
CLDN6				0.00176	0.68238	Female									
NEURL2										0.04214	0.92947	Female			
AP1S1				0.02208	0.64029	Female									
INMT													0.03433	2.12665	Female
LDLRAP1										0.00002	1.27936	Female			
NUDT22	0.01328	-1.58816	Male												
CA5B	0.01100	2.53602	Female	0.00000	4.01878	Female	0.00948	1.16324	Female	0.00000	2.17857	Female	0.00001	1.69674	Female
NUAK2	0.01314	1.88134	Female										0.00056	0.97842	Female
CLTB										0.00160	0.61811	Female			
HNRNPKP3										0.00000	-1.03734	Male			
SALL3										0.00111	1.65008	Female			
CARD8													0.01119	0.94315	Female
AGRN										0.04508	0.66340	Female	0.04920	0.64791	Female
CKMT2-AS1													0.00454	0.88997	Female

AKAP9	0.00122	1.16710	Female												
SNORA67							0.00004	0.75834	Female				0.00032	0.63317	Female
MGC72080										0.03300	-0.61972	Male			
ARMCX6													0.02873	-2.08223	Male
DUS4L							0.04477	-0.59051	Male						
WTAP	0.00015	-0.82709	Male												
ING2	0.00306	-1.37209	Male												
PRDM14										0.00009	1.78776	Female			
DTX2										0.00143	-0.72440	Male			
HPS4				0.04080	-0.81434	Male									
ZDHHC9				0.00017	1.07192	Female	0.00261	0.61913	Female	0.00006	0.75618	Female			
PDE5A										0.00030	1.17205	Female			
PPARD										0.00011	-0.69982	Male			
KIAA1551										0.00000	0.69608	Female			
HUNK				0.00127	1.45472	Female									
PACSIN1							0.01700	-1.83722	Male						
OR5AU1							0.00000	2.48193	Female	0.01531	-1.34116	Male			
CYP1A1							0.00155	-1.56938	Male				0.00969	1.27731	Female
SLC29A2							0.00064	-0.79707	Male						
PTPRU				0.00000	1.79396	Female									
RSG1	0.04508	-2.81566	Male												
TNNC2				0.01796	1.60961	Female				0.00082	1.40598	Female	0.00452	1.21127	Female
PQLC3										0.00000	1.03464	Female			
RTCB	0.00124	-0.81449	Male												
ZNF750				0.00181	-1.68986	Male									
MSI1													0.01554	-0.72284	Male
WNT3A										0.04367	2.01604	Female			
CEBPG	0.02338	-0.72702	Male												
YBX2										0.00008	1.54468	Female			
ZNF112	0.01358	3.32058	Female												
NEAT1							0.00239	0.86518	Female						
RNF103							0.04328	0.82019	Female						
CNTN6										0.03989	0.92865	Female	0.00004	-1.67750	Male
ELOVL2										0.03146	-1.72099	Male			
NCAM2							0.00424	-2.81145	Male						
LINC01420				0.01534	1.78122	Female									
IGSF3				0.00446	1.14805	Female									
MIR3064							0.01160	0.65023	Female						
VAV2	0.04441	-1.19940	Male	0.00010	1.17049	Female									
ADNP2							0.00303	0.62251	Female						
NOS1							0.00001	1.77415	Female	0.00000	-1.52667	Male			
LRRC58	0.00053	-2.12798	Male							0.00020	-0.77273	Male			
REXO4							0.00000	0.91607	Female						
LIME1							0.00459	1.18024	Female						
ZER1							0.02059	0.69703	Female						
ZNF316										0.03461	-0.91098	Male			
EMG1	0.00019	-0.78659	Male												
IL17RB	0.00198	3.17429	Female										0.02479	-0.93582	Male
RSPO3				0.00000	1.02220	Female	0.00034	-2.45246	Male	0.03300	1.61985	Female			
ENG							0.02543	1.59746	Female						
ABCC4													0.00000	-1.22886	Male

TTC3P1				0.00405	1.29047	Female									
HEXIM1				0.00455	-0.70201	Male									
TYMS	0.01125	-1.09379	Male				0.00000	-0.81472	Male						
ZNF780B				0.04563	-1.56237	Male									
DDX1	0.00000	-0.94020	Male												
CTBP1				0.00651	0.60509	Female	0.00001	0.61851	Female						
TCEB2	0.00040	-0.72157	Male				0.00000	0.63279	Female						
WLS							0.00000	-1.80110	Male	0.00008	-1.14411	Male	0.00684	-0.81581	Male
ZNF730							0.00895	-0.74964	Male						
L1TD1										0.00001	0.59140	Female			
FUNDC2P2							0.00000	0.73815	Female						
NAV1										0.03843	0.80376	Female			
AJUBA							0.00017	-1.05957	Male	0.00281	-0.82629	Male			
OXLD1										0.01419	-0.70841	Male			
SLC39A11													0.00080	-0.99990	Male
CSTF2							0.00000	1.36127	Female	0.00000	0.73659	Female	0.00000	0.88028	Female
RRAD				0.00000	3.93504	Female	0.00027	1.62257	Female	0.00068	1.48182	Female			
CHRNB1										0.04150	-0.68368	Male	0.02382	0.72725	Female
SLC4A11													0.00000	0.91533	Female
MIR614							0.00157	1.01242	Female				0.00572	0.89340	Female
CASP6	0.00008	-1.60469	Male												
ARIH1				0.00454	0.69839	Female									
SLC6A8				0.00000	1.34749	Female	0.00000	2.03541	Female	0.00000	1.29215	Female			
ALPPL2										0.00000	1.02489	Female	0.00000	0.87681	Female
ANXA2P1				0.00003	-0.74485	Male									
TTLL3							0.00075	-1.26583	Male						
PELO										0.00003	-0.75433	Male			
DENND4B													0.02643	0.79638	Female
VWA5A										0.00000	3.19480	Female			
BRI3				0.03101	0.73192	Female									
WNT6							0.00008	-1.44781	Male						
PKIB	0.02822	-1.37703	Male				0.00002	-1.00569	Male						
FSCN1				0.00069	0.79306	Female									
FAM64A										0.00000	2.70617	Female	0.02208	1.43861	Female
TPP1							0.00085	-1.03800	Male						
METTL2B	0.03283	0.76495	Female												
WWC1													0.00169	0.64387	Female
PIGQ				0.01578	1.37478	Female									
TNFRSF1A										0.00095	1.23730	Female			
DYNLT3													0.00000	-1.87321	Male
SCML1													0.00133	-1.35504	Male
NFE2L3				0.03113	0.64715	Female	0.00000	-0.82416	Male						
MGAT4A							0.00022	-1.34210	Male						
LYG2							0.04542	1.16073	Female						
ZNF92							0.00003	-0.72991	Male						
SAPCD2	0.00000	-3.59182	Male	0.00000	4.48750	Female									
APOL2							0.00000	2.90471	Female	0.03442	1.27682	Female			
KBTBD4										0.00006	-1.11299	Male			
PLXNA3													0.01489	0.98353	Female
SNORD48													0.00305	0.81342	Female
TP53I13	0.01969	-1.88716	Male				0.00030	-1.15715	Male	0.01260	-0.80412	Male	0.00601	-0.86186	Male

RHOT1				0.02458	1.08099	Female	0.01112	-0.80323	Male						
ASNS				0.00000	-2.55705	Male	0.00001	-1.15898	Male						
C1orf86							0.00278	-0.79613	Male	0.02216	-0.59681	Male			
FRAT2							0.00013	0.62749	Female						
PIGO							0.01050	-0.73353	Male						
DDN										0.00458	-2.26651	Male			
LMBR1L				0.01937	-0.68266	Male									
MLLT11										0.00251	-1.20733	Male			
PBX3										0.00187	1.08579	Female			
GSTP1				0.00000	1.51136	Female				0.00000	0.91471	Female			
POLR2I	0.00495	0.64197	Female												
SHISA2										0.02546	1.65916	Female			
TTTY14							0.00085	-2.47344	Male	0.00000	-3.13467	Male	0.00000	-4.15901	Male
ARGFX										0.00000	1.69603	Female			
MED12				0.00231	0.95817	Female	0.00268	0.65160	Female	0.00149	0.65563	Female	0.00007	0.78053	Female
SLC38A9	0.00006	-1.94675	Male												
LOC728613				0.00007	1.45546	Female									
AGPAT9							0.03709	-0.78490	Male	0.02999	-0.77570	Male	0.04610	-0.73342	Male
PRNP										0.03025	0.63139	Female			
20.00000	0.00340	3.17853	Female							0.00351	1.28526	Female			
ICE1	0.04852	-0.91080	Male												
FAM168B	0.02349	-0.80872	Male												
ATG16L1							0.00011	0.59565	Female						
BCORL1				0.00102	1.18575	Female									
PDHA2	0.00043	-1.16231	Male	0.00000	0.88294	Female	0.00000	0.83101	Female				0.00000	0.65613	Female
RPS4Y2							0.00000	-3.64839	Male	0.00012	-3.35831	Male			
MIR3648										0.00000	2.84103	Female			
CTB-113P19.1										0.00054	0.95426	Female			
LOC400043										0.03659	1.22694	Female			
C20orf96							0.00138	-1.29939	Male						
DEDD2							0.00471	-0.63751	Male	0.00007	-0.80847	Male			
LOC641746													0.02418	-0.83088	Male
SYCP1													0.00149	-2.33862	Male
CGREF1							0.00391	1.33796	Female	0.04309	0.95512	Female			
TMX3							0.00053	-0.71577	Male				0.00033	-0.68777	Male
MOGS							0.01052	-0.58776	Male						
ZNF124	0.00492	-1.61073	Male												
GPRC5B										0.00024	1.80598	Female			
ZBTB34							0.02887	0.76754	Female						
CNBP	0.00013	-0.64531	Male												
TMEM205	0.00164	-1.38999	Male												
PDGFA				0.00056	0.90829	Female	0.00009	0.69318	Female				0.00036	0.60897	Female
COX7B2							0.00000	-0.77283	Male						
PIH1D2										0.01495	-1.44844	Male			
SOX17										0.00007	2.97450	Female			
LMO1													0.00405	2.05647	Female
SLC7A6OS							0.00325	0.61895	Female						
RPL39	0.00102	0.83588	Female	0.00000	0.69687	Female	0.00000	0.62035	Female						
MYBBP1A										0.00009	-0.62934	Male			
SLC25A12										0.02116	1.00870	Female			
PPP1R14A	0.01011	-1.08578	Male	0.00000	1.45749	Female	0.00000	0.84348	Female						

TM7SF2							0.00000	-2.45304	Male						
CKMT2				0.03142	1.66862	Female				0.03870	1.06556	Female			
MOSPD1				0.00120	0.87327	Female				0.00069	0.60429	Female			
SUPT20HL2							0.01274	2.58057	Female						
ETV1										0.00854	1.82801	Female			
GPC3										0.02263	1.30187	Female			
PURB										0.04717	-0.63547	Male			
CASP7													0.00021	1.94673	Female
SNORD87													0.00612	-0.62794	Male
NTRK3										0.03529	1.67656	Female			
VENTX	0.00015	-2.13145	Male							0.00002	2.35907	Female			
ZNF217				0.03343	-0.62649	Male									
CLDN4				0.00000	3.83957	Female									
KLHL23										0.00076	-1.23230	Male			
UACA				0.00008	1.27827	Female				0.00000	1.58171	Female			
CPSF6	0.00005	-0.87863	Male												
TPM2							0.00198	0.63532	Female						
FABP7				0.00816	1.60472	Female				0.00856	0.92945	Female			
VWA1				0.00906	1.07943	Female									
CLDN12							0.00104	-0.75898	Male						
GAL										0.02852	0.71678	Female			
LIPG							0.04110	1.16700	Female						
NLRP12										0.01082	0.79051	Female			
BTBD1													0.00129	-0.75962	Male
CUEDC2	0.00027	2.69153	Female												
IKBKB							0.00152	0.80116	Female						
IL17RE							0.02125	-1.46436	Male	0.02846	-1.30186	Male			
SRGAP2D.uid-421										0.00033	0.68632	Female			
SNORA84							0.03239	0.83715	Female						
UNC93A										0.00191	2.23673	Female			
LOC100506178				0.00169	1.76223	Female	0.00214	1.17513	Female						
TOB2	0.01330	0.83514	Female	0.00206	-0.61476	Male									
PIGY+PYURF	0.02581	0.78755	Female												
JPH2													0.02277	2.80061	Female
RTP1													0.00874	-1.21333	Male
SPATA13										0.02008	-0.87079	Male			
EXOSC4							0.00000	0.64557	Female						
CCDC50				0.00454	1.07026	Female									
KIAA1804										0.00380	1.53060	Female			
PILRB							0.00000	1.96068	Female						
RAD50										0.00014	-0.66743	Male			
PRDX1	0.00000	0.77490	Female												
CDKN1C				0.00010	-2.10275	Male	0.04005	0.85862	Female				0.01828	0.90478	Female
CCDC183-AS1							0.00125	2.05597	Female						
RGCC										0.00024	1.02066	Female			
TPRX1										0.00000	6.03232	Female			
SHISA4				0.00634	-2.04887	Male									
APOBEC3G							0.02164	-2.35228	Male						
NMB										0.00495	-1.29439	Male			
DDX3X	0.00000	0.78957	Female				0.00000	0.95360	Female	0.00000	0.61970	Female			
PEG10							0.00049	1.21567	Female				0.02328	-0.77397	Male

GGA2				0.00611	0.63786	Female									
SUN3				0.00050	0.85190	Female	0.00007	-0.64318	Male						
NT5C3B				0.00002	1.25052	Female									
ZNF484							0.03230	-1.09564	Male						
ZNF557							0.00003	0.78491	Female						
AKAP2.uid-11393							0.00838	-0.95068	Male						
C5orf30										0.00047	-1.52743	Male			
FSD2										0.01827	0.77385	Female			
SH3BGRL							0.04199	-2.03567	Male						
TPRXL										0.00140	-1.65979	Male			
NMNAT1													0.00803	0.73577	Female
PRDX4	0.00025	1.47728	Female	0.00000	1.10733	Female									
CCNK							0.00000	-0.64602	Male						
HIST1H2AM				0.00416	-1.33898	Male									
TGFBI							0.02617	-1.69938	Male				0.00424	1.93665	Female
CCDC88B							0.01396	1.84598	Female						
OLAH										0.00694	1.59907	Female			
FBLN7													0.00190	-4.34915	Male
FNTA				0.00122	0.75502	Female									
TMEM109				0.01568	0.62114	Female									
FAM83H				0.00265	1.06538	Female									
CTSH										0.03339	0.68597	Female	0.04223	0.65433	Female
PLP1										0.01693	2.37549	Female			
TBC1D24										0.00383	-0.94668	Male			
TESK1							0.00494	0.95317	Female						
TUBA3E							0.02006	1.15977	Female	0.00008	1.75160	Female	0.02405	1.06151	Female
CAPN2				0.03870	0.77355	Female									
FAM207A				0.00576	0.77142	Female									
GAS6				0.00120	1.04738	Female							0.00198	-0.65300	Male
ERGIC1										0.00472	-0.99867	Male			
PLCD1										0.01284	0.97932	Female			
CHGA							0.00091	1.66255	Female	0.00000	4.48667	Female			
PRAMEF1										0.00000	7.19203	Female			
IRS1										0.00166	-1.26912	Male			
GGT1										0.00064	1.73178	Female			
EFCAB2							0.00694	-0.74366	Male						
AK8										0.01332	1.36028	Female	0.01396	-1.35374	Male
CCDC58													0.00000	-0.71785	Male
ZNF462										0.00320	1.84069	Female			
ZDHHC7	0.01875	-1.14622	Male												
EIF4E													0.00000	-0.67128	Male
SNORD52										0.00000	0.98005	Female			
LOC100505666													0.03581	1.68759	Female
TTC4										0.00019	0.70742	Female			
PALLD				0.04229	0.64207	Female									
RPS10							0.00000	0.63793	Female						
TMEM86A										0.00001	2.35583	Female			
ATG9B							0.00004	1.08130	Female	0.00000	2.20551	Female	0.00000	1.60549	Female
NDFIP2													0.00000	-0.62210	Male
GNB1L	0.00092	1.74337	Female												
PPP2R4				0.00906	0.65392	Female									

ISG15										0.00494	1.79328	Female			
SESN1										0.00555	0.69095	Female			
ZNF529	0.01644	1.45954	Female												
SLC9A6							0.00184	0.90637	Female						
PPP1R8										0.00000	-1.30246	Male			
MALT1							0.00436	-1.02219	Male						
RELT				0.00157	-1.93182	Male									
ARPC3	0.00000	-0.88441	Male												
HBP1													0.00215	0.63827	Female
1.00000							0.00419	-0.59394	Male						
PLEKHF2	0.00788	-1.57337	Male	0.04973	0.78917	Female									
YES1	0.02948	-0.79072	Male				0.00000	-0.62226	Male						
MEG3										0.00168	0.94744	Female			
PLEKHB1										0.00961	1.58987	Female	0.01509	1.47637	Female
LGALS3							0.00001	-1.44756	Male	0.00000	-1.88678	Male			
SMPDL3B										0.00000	-3.48437	Male			
ELMOD2													0.00144	-0.71130	Male
IGSF10							0.04522	-1.59154	Male						
OTUD3							0.00030	0.99571	Female						
RGS19				0.00073	-1.91106	Male									
MAPK3	0.00166	-1.50862	Male	0.00039	1.00189	Female							0.00012	0.70298	Female
TNNC1				0.03322	1.26247	Female									
FAM219A							0.04757	0.75755	Female						
NMT1							0.00000	0.78536	Female						
GPR114										0.00161	-1.63209	Male			
SERTAD4-AS1										0.02486	-1.88060	Male			
CTSF										0.00000	2.16345	Female			
MAGT1				0.02120	0.68275	Female									
CPA1										0.01936	-2.21941	Male	0.00939	2.33186	Female
GNPDA2										0.00886	-0.84873	Male			
ALDH4A1				0.00002	1.62950	Female	0.04614	-0.59962	Male						
ANXA1	0.00015	1.92643	Female	0.00000	-2.19766	Male									
HPGD				0.00010	1.15638	Female									
KAT7				0.01795	-0.64421	Male									
TMPRSS2							0.00095	-1.17736	Male						
COL6A1										0.00003	1.92893	Female	0.00008	1.79146	Female
TP53INP1										0.00869	-0.72058	Male			
ZNF69							0.00006	-1.72477	Male	0.02127	-1.00276	Male			
ZNF300													0.02585	0.80680	Female
MIR6878										0.04751	0.64863	Female	0.00680	0.83685	Female
NPFFR2							0.02413	-1.14999	Male						
VAMP8				0.00002	0.73245	Female									
PIP5K1P1										0.00196	-0.66621	Male			
C5orf58													0.00140	-1.28553	Male
TMEM128													0.00001	-0.82188	Male
MRPS36	0.00471	0.79723	Female												
SWT1										0.03484	-0.58704	Male			
CNST										0.00016	-0.70633	Male			
HNRNPA1L2										0.00003	0.88221	Female	0.01045	-0.58107	Male
AVL9							0.00844	-0.69708	Male						
SMDT1										0.00018	-0.93331	Male			

LDOC1L							0.00109	-2.35323	Male						
WWP1													0.00196	-0.58422	Male
SDC4				0.00003	-0.66480	Male									
RECK													0.00593	1.42615	Female
CCDC144A							0.00419	1.59932	Female						
NYNRIN				0.03920	-1.04391	Male									
CFLAR										0.00000	2.09167	Female			
SIGLEC10							0.03568	-1.79420	Male						
GCNT2				0.02868	1.07176	Female									
MIR6857							0.00000	1.20518	Female	0.00000	1.08565	Female	0.00000	0.90911	Female
ELF4				0.03251	1.09910	Female									
MPRIP				0.00016	0.90116	Female									
NFKB2				0.00715	0.91908	Female				0.00000	1.21241	Female			
AKIRIN1							0.00000	-0.66906	Male						
HDHD1										0.00003	0.79193	Female	0.00004	0.76748	Female
CBWD1	0.04221	-0.69254	Male												
SNHG4.uid-7434				0.00828	-1.08148	Male									
CEACAM1										0.00000	2.34641	Female	0.01599	1.25084	Female
TALDO1				0.00000	0.87859	Female									
CDC42EP2										0.00225	-0.95522	Male	0.01389	0.79079	Female
RASSF2	0.00417	2.55770	Female												
SAMHD1										0.00162	0.74896	Female	0.01270	0.60633	Female
TFE3				0.03705	0.85979	Female	0.00928	0.67693	Female						
ASTN2										0.00809	0.92337	Female			
IFI6	0.02590	2.38199	Female	0.00005	2.39063	Female	0.02984	0.99567	Female				0.00264	1.18660	Female
LOC643623										0.00000	3.16991	Female			
VSIG10	0.01211	-1.06972	Male							0.00000	0.97058	Female			
C5orf63							0.00075	-1.06372	Male						
ZRANB2	0.00094	-0.77171	Male												
AFG3L1P										0.00070	0.91662	Female			
DLST										0.00000	-1.40176	Male			
PLEKHA8				0.00110	1.18457	Female									
MGST3	0.00421	0.82510	Female												
GPR89B	0.00007	2.14326	Female												
RP2				0.01377	0.83757	Female	0.00001	0.93862	Female						
BCAM							0.00010	-1.60446	Male						
CHST8										0.00009	1.84387	Female	0.02354	1.14378	Female
AP5B1				0.03381	-1.17874	Male									
NME1-NME2										0.00917	-0.62727	Male			
OMA1	0.00550	-1.56572	Male				0.01757	-0.58111	Male						
PDLIM2										0.02997	-1.04545	Male			
ZNF668										0.01859	-0.60469	Male			
HIP1				0.01664	0.86780	Female									
PTPN18				0.03500	1.54757	Female									
CCDC138							0.00768	-0.65693	Male						
CETN2	0.00080	0.99575	Female	0.00002	0.75838	Female	0.00000	0.75882	Female	0.00000	0.64227	Female			
IRX2	0.02684	-1.76139	Male				0.00921	0.86293	Female	0.00197	-0.92951	Male			
UBE2D3	0.00000	-0.69400	Male												
HNRNPH2				0.01062	0.60485	Female	0.00000	0.77935	Female	0.00000	0.67437	Female	0.00001	0.60655	Female
CCIN	0.00000	-1.27162	Male												
ACKR3	0.00000	1.17307	Female	0.00189	1.38531	Female									

TCF15							0.02287	1.40170	Female						
BCRP3										0.01989	1.89772	Female			
KCNJ14										0.00170	-2.00623	Male			
HSPA1A													0.00001	1.18948	Female
CLCN5				0.00419	1.17585	Female				0.01387	0.67818	Female			
VPS53							0.00000	0.72496	Female						
CA11													0.00795	1.96285	Female
NOL12							0.00000	0.72534	Female						
SNHG6				0.00000	-0.71717	Male									
PPP3R1										0.00458	-0.60878	Male			
KIAA1462							0.03153	-0.75497	Male						
MTAP							0.01337	-0.73020	Male	0.00832	-0.72563	Male			
ATP11A	0.00004	-1.45043	Male												
ELF3	0.00052	-1.47918	Male							0.00001	0.66036	Female	0.00000	0.76542	Female
HOXB3							0.00070	-1.92293	Male						
NRIP3							0.00005	0.99394	Female	0.00000	1.57531	Female			
OSBPL7													0.00488	2.60276	Female
ZEB1													0.01519	1.94956	Female
LOC100132352							0.00000	-0.90126	Male						
CRMP1							0.01824	2.21332	Female						
CASC8										0.01483	1.78587	Female			
ACSS2							0.00000	-1.36329	Male	0.00356	0.71096	Female			
ACTC1										0.00000	-2.50845	Male	0.00023	1.71789	Female
PFDN6										0.00000	-1.24077	Male	0.00002	0.69213	Female
HTATIP2													0.00103	-0.68142	Male
GLIS2							0.03942	0.97385	Female	0.00035	1.48450	Female			
HLA-F-AS1										0.00082	1.79608	Female			
DDHD2							0.01830	-0.88791	Male						
ABHD2	0.01855	-0.67716	Male												
FGD1				0.03537	1.21894	Female	0.00001	1.57718	Female	0.04394	0.77830	Female			
KIF26A				0.01189	1.29865	Female							0.00012	1.20481	Female
BIN1	0.00598	1.90844	Female	0.00000	2.08089	Female									
CHAMP1							0.01622	-0.63434	Male				0.00602	-0.66192	Male
NT5C				0.00923	0.60009	Female									
DOCK6										0.00004	1.08958	Female			
HLA-DRB5										0.00154	-2.23502	Male			
CHML													0.00488	-0.71075	Male
GATA6	0.04947	-0.85421	Male												
PRR5				0.02090	0.70388	Female									
MEF2BNB.uid-23522							0.00320	1.19100	Female						
ZNF622							0.00000	0.67083	Female						
ORMDL3							0.03228	0.61871	Female						
HAVCR2	0.00452	3.26731	Female	0.00935	-1.86119	Male									
TCEA1	0.03428	-0.61949	Male												
CCDC112	0.00021	-3.20488	Male												
GALNT12										0.00717	2.05614	Female			
NRIP1													0.04728	-1.10427	Male
HEY1							0.02932	-1.65951	Male						
GRTP1										0.00034	0.75123	Female	0.00217	-0.64813	Male
GSR										0.00129	-0.74495	Male			
WDR45	0.04664	0.78503	Female	0.00111	0.69576	Female	0.00000	1.58305	Female				0.00000	0.87534	Female

RASSF8-AS1										0.00815	1.17351	Female			
BNIP3L										0.00070	-0.73817	Male			
FOXI3							0.04019	0.86577	Female				0.04928	0.79538	Female
MARCKS				0.00043	1.01123	Female									
CCDC64B							0.00487	-1.64226	Male						
LRRC8D							0.00052	-0.87985	Male						
NPEPPS	0.00451	-0.62543	Male												
PELI2										0.00117	1.93225	Female			
MT1HL1													0.00004	2.04353	Female
ZBTB11-AS1													0.03254	0.60065	Female
ARHGEF38										0.00101	0.96323	Female			
FNDC4				0.03260	-2.06019	Male	0.01795	-2.02906	Male	0.00001	3.15846	Female			
LINC00884							0.04689	1.64039	Female				0.00044	2.43830	Female
TMEM247							0.00215	1.64165	Female						
OCIAD1	0.01811	-0.59274	Male												
UTP14A							0.00000	0.73585	Female						
CCNJ										0.00080	-0.70341	Male			
CSAG1				0.03693	2.01625	Female									
SMG1P3										0.00181	1.06034	Female			
RPSAP58	0.00005	0.89086	Female												
PYROXD1	0.00028	-1.76205	Male												
UAP1L1				0.00005	1.70867	Female				0.01218	0.76088	Female			
KIF4B							0.00002	0.68296	Female						
TAS1R1													0.00342	1.71984	Female
CNIH4	0.04579	0.61244	Female												
C6orf211	0.02743	-0.70987	Male										0.00000	-0.58678	Male
MED14				0.00080	1.01489	Female	0.00015	0.78474	Female						
TMEM164				0.00001	1.57603	Female	0.00001	1.07327	Female	0.01074	0.65400	Female			
PTPRS							0.02348	0.71822	Female	0.00001	1.23234	Female			
CENPT	0.01091	0.92107	Female												
AFAP1-AS1										0.01105	1.44542	Female	0.00404	1.55666	Female
DNAJC22										0.03097	1.99095	Female			
DLEU2L										0.00002	0.89732	Female			
RERE				0.00073	0.78806	Female									
PHOSPHO1							0.00000	1.19052	Female	0.00000	2.68424	Female			
PPIL4	0.03963	-0.78445	Male												
SLC35G3	0.03835	3.45057	Female												
PI16										0.00000	3.58505	Female			
TPK1										0.00000	-1.45569	Male			
TMEM160	0.00388	1.46949	Female							0.00000	0.89354	Female			
LHX4													0.01358	1.59004	Female
ANKRD45										0.02428	1.77538	Female			
DUS1L				0.03787	0.61563	Female									
MTA2				0.00002	0.76983	Female									
TMEM52							0.00018	-1.41554	Male						
LAMC3				0.03653	1.78522	Female									
ADI1	0.00002	-1.23768	Male												
TIMM17B				0.00000	0.81518	Female	0.00000	0.64759	Female	0.00000	0.62960	Female	0.00000	0.63661	Female
TRMT10A													0.00000	-0.73842	Male
PRPS1L1				0.00277	0.78159	Female	0.00000	1.24322	Female						
LINC01356							0.00000	-1.33429	Male	0.01069	0.68368	Female			

PCBD1							0.00113	-0.88385	Male						
SNAPC2										0.01234	0.70200	Female			
LOC101927746				0.01645	0.78246	Female									
AMBN							0.00036	-1.26847	Male						
HIST1H2BN							0.00996	1.66774	Female						
CLU	0.03215	-2.63384	Male				0.00036	2.08930	Female						
STKLD1				0.01747	2.19666	Female									
TUBB8							0.03006	1.48713	Female	0.00003	-2.53188	Male	0.01463	-1.59244	Male
CCDC39													0.00264	-1.56765	Male
NXN				0.04891	1.29075	Female									
ORC3							0.00008	-0.61772	Male						
ZNF439										0.04934	-1.28601	Male			
CCDC8				0.00943	-2.29508	Male									
EPHB1										0.00060	1.95040	Female			
MSL3P1							0.00184	-0.88158	Male	0.02204	-0.65965	Male	0.00257	-0.81202	Male
ERVMER34-1										0.00759	-0.90005	Male			
ZNF205										0.01853	1.09408	Female			
ACOT1							0.00550	-1.15246	Male	0.00029	-1.37475	Male			
HDGFRP3										0.01002	1.02967	Female			
CCND1				0.00001	2.28150	Female	0.00000	1.65086	Female				0.00000	1.89435	Female
MSX2										0.00071	-0.63105	Male			
M1AP							0.02023	-1.12176	Male						
SLC5A10				0.00000	2.22944	Female									
CCR8										0.00000	3.45671	Female			
ALDH1A1	0.00000	-2.18180	Male							0.00000	3.17188	Female			
SLC22A18										0.00211	1.86365	Female			
SH3BGRL2							0.01938	-0.58599	Male	0.00004	-0.88812	Male			
HSPG2							0.00096	-1.95392	Male						
RELL2							0.02829	-1.48851	Male						
RPL23AP32										0.00062	-0.90785	Male	0.02283	-0.62980	Male
PLAU										0.00003	1.62566	Female			
CACNB3										0.00020	1.34739	Female			
TREM1										0.00233	-1.87300	Male			
KANK4				0.00131	1.32277	Female									
SPTAN1				0.00834	0.58995	Female									
GOLGA8B										0.00239	-0.86290	Male			
LHFP							0.00183	-1.64733	Male						
PAIP1							0.00000	-0.61111	Male						
SULT1A1										0.00324	1.47370	Female			
LOC100507002	0.03493	2.82094	Female							0.00237	1.65365	Female			
JAZF1										0.01990	0.94090	Female			
HIST3H2A				0.00235	-1.82588	Male				0.00000	-2.13758	Male			
SNX7	0.00053	2.92745	Female												
SLC15A3										0.02525	1.85710	Female			
CCNA1										0.00000	1.99648	Female	0.01454	1.06499	Female
WNT9A													0.00000	2.88801	Female
VWF							0.03907	2.33401	Female						
PHACTR2	0.00000	3.02121	Female	0.02698	0.77868	Female	0.00000	-1.03557	Male						
OLFML3	0.00216	3.22726	Female	0.00000	1.31822	Female							0.00021	2.44913	Female
HNRNPD	0.00012	-0.92347	Male												
ZHX2							0.00400	-2.02162	Male	0.01288	-1.75727	Male			

SPIN4	0.00000	5.55047	Female	0.01783	0.87204	Female	0.00217	0.72447	Female						
DCAF4L2													0.04744	-0.65737	Male
TCF12				0.01834	0.88923	Female	0.00288	-0.72157	Male						
SNORA27				0.01012	-0.97690	Male									
H1FX							0.00000	1.34104	Female						
NHEJ1	0.02329	1.49216	Female												
MIR5047							0.02363	0.60415	Female	0.00000	1.46237	Female	0.00838	0.64433	Female
KAT2B										0.03843	-0.74678	Male			
DCXR				0.00053	0.83321	Female									
SPIC										0.00000	3.09262	Female			
HIST1H4F										0.00859	2.24655	Female			
IFT140							0.03930	0.94540	Female						
MAPKAPK2							0.00181	0.83692	Female						
TMLHE							0.02809	1.04895	Female						
LPGAT1										0.01074	0.66808	Female			
PLCG1										0.00122	-0.89587	Male			
RAD23B										0.00000	-1.16871	Male			
GPR133													0.00766	1.56534	Female
PP7080										0.02011	0.62491	Female			
SAC3D1										0.01983	-0.69163	Male			
ZFX				0.00071	0.92923	Female	0.00000	0.84142	Female	0.00142	0.58534	Female			
CATSPER2													0.02954	1.60311	Female
PIP5KL1													0.00580	2.70568	Female
SUMO4										0.00004	-1.67711	Male			
POMC										0.00586	0.71425	Female	0.00004	0.98365	Female
LIX1													0.00519	2.58371	Female
CYP2S1										0.00000	0.83800	Female			
MACC1				0.00060	-2.60351	Male				0.00182	-1.72540	Male			
HLA-DRB6										0.00000	-1.83240	Male			
HIST1H3C				0.00044	-1.82519	Male				0.04891	0.76982	Female			
NPC1				0.00308	-0.89021	Male									
RMDN2							0.00963	-1.41931	Male				0.03585	-1.14470	Male
DPH5	0.00382	1.37789	Female												
FABP3	0.00000	-2.71120	Male	0.00018	1.43954	Female	0.00337	0.78460	Female						
LOC728739							0.01738	-0.60020	Male						
ARRDC3										0.00528	0.63822	Female			
C9orf84										0.02263	1.30832	Female			
FAM71E1													0.00107	0.78571	Female
CACNA1H										0.00593	2.27757	Female			
8.00000							0.00000	-0.63866	Male						
FAM76B							0.00127	-0.66891	Male						
S100Z										0.01094	1.81823	Female			
SCARNA22				0.01921	0.94627	Female				0.00124	0.84472	Female	0.00685	0.69751	Female
MRVI1													0.01888	1.62604	Female
SEMA6A				0.00000	1.55524	Female									
NELFCD	0.00074	-0.92316	Male												
ZNF667													0.03770	0.90487	Female
MIER1	0.00855	-0.86237	Male												
GRAMD1B										0.00003	-2.54099	Male			
SOX11										0.00012	-1.88650	Male			
MYO5C													0.02595	-0.83645	Male

HMGB3				0.00000	1.00794	Female	0.00000	0.97645	Female	0.00000	1.33932	Female	0.00000	0.66574	Female
RNF39													0.04844	1.02627	Female
NFKBIL1							0.00028	-1.14906	Male						
HERPUD1				0.01372	-0.80249	Male									
DCAF8L1										0.03250	-1.15252	Male	0.01196	1.30976	Female
CADM1				0.00104	1.44478	Female							0.02282	0.69984	Female
CYGB							0.00184	-1.58040	Male				0.01811	1.20529	Female
LOC100288778										0.01735	0.90950	Female	0.00113	-1.14383	Male
NCEH1										0.00009	0.90984	Female			
MRPL32	0.00000	-0.93057	Male												
PAMR1										0.00045	2.32062	Female	0.00002	2.80848	Female
PLAUR										0.00070	1.43045	Female			
SIPA1L2										0.00192	1.35244	Female			
CLCN7													0.00413	-0.92437	Male
NPPB													0.00000	2.34801	Female
LPHN3													0.01750	-1.08589	Male
YWHAZ	0.00004	-0.69597	Male												
ABHD6							0.03051	-0.69744	Male						
B3GNT5										0.00725	-0.97553	Male			
IGF1							0.01170	-1.67926	Male						
NDN										0.00004	-1.46472	Male			
STYXL1										0.00351	0.83876	Female			
RMDN1				0.00924	0.66595	Female									
LOC100131691										0.00570	-1.23502	Male			
NT5E				0.01769	2.81559	Female									
CCT4	0.00151	-0.60730	Male												
CBLC				0.00048	-2.63452	Male									
ZBTB39													0.03300	0.89715	Female
HSPA1B										0.00001	-0.92203	Male			
HOTAIRM1							0.01470	1.22885	Female						
C19orf60				0.00002	1.05782	Female				0.00007	0.66292	Female			
ZNF496				0.02394	1.22819	Female				0.00542	0.94653	Female	0.02860	0.76372	Female
NPIPA5							0.04195	0.68994	Female				0.00632	0.84039	Female
PLEK2							0.00526	1.18785	Female				0.00772	1.06342	Female
CRHR2													0.01289	2.09631	Female
FANCA										0.00952	0.65552	Female			
LIX1L										0.00194	2.26766	Female			
LOC100506469				0.01188	-1.61711	Male	0.02723	0.99272	Female				0.03370	0.90735	Female
R3HCC1										0.00029	1.07718	Female			
PMS2P4										0.00000	2.00900	Female			
LARP1B	0.01628	-0.77837	Male												
IGSF8	0.00000	-3.61432	Male												
LOC100129518													0.00495	1.33712	Female
PAWR							0.00984	-0.59472	Male						
PAM													0.02825	-1.30297	Male
SRGAP1										0.00027	1.42006	Female			
ANKRD36BP1													0.02215	0.85772	Female
IL17RD										0.03772	1.43799	Female			
BHLHE40							0.01855	0.93221	Female						
EXPH5	0.00067	2.15430	Female	0.01251	0.99851	Female	0.00124	-0.83439	Male						
CUBN							0.00663	-2.76033	Male						

MAP3K8							0.00005	-1.02432	Male	0.01754	-0.61692	Male			
XPR1							0.00004	-1.03848	Male						
YPEL2							0.00000	0.87755	Female	0.00000	0.87258	Female	0.00012	0.62000	Female
APOE	0.03283	-1.50467	Male	0.00000	1.93423	Female				0.00000	1.71841	Female			
COMMD4	0.02133	0.85841	Female												
PAK1										0.00002	1.03582	Female			
PLEKHA6				0.00000	-3.24069	Male									
UBA2	0.04335	-0.63731	Male										0.00000	-0.63587	Male
FAM117A										0.03153	0.95171	Female			
C17orf96													0.02194	1.00455	Female
ZBTB17				0.02448	-0.94782	Male									
NFATC1										0.03610	1.35974	Female			
PAK2				0.00006	0.64546	Female									
STK19.uid-8457													0.00521	0.71934	Female
TMEM165							0.00000	-1.05593	Male				0.00000	-0.88014	Male
MTCL1										0.00889	1.33144	Female			
AIMP2	0.00061	-1.05900	Male												
SLC25A5-AS1										0.00498	1.80486	Female			
SLC35A2										0.00058	0.61568	Female	0.00005	0.69637	Female
LECT2										0.02955	-1.65492	Male			
INSIG2							0.01964	-0.64781	Male						
BLVRB													0.00354	0.62758	Female
PPARG	0.00707	-1.91640	Male				0.00008	-1.12537	Male						
GNG10				0.00000	1.01720	Female									
AFAP1L1	0.03862	2.70844	Female												
SLC16A3				0.00001	2.61462	Female									
ANP32C							0.00000	-0.66555	Male						
LOC100507547										0.03829	1.32500	Female			
SCAPER													0.04042	-0.58872	Male
RNASEH2B							0.00000	-0.91136	Male				0.00123	-0.62585	Male
NCCRP1	0.00001	4.97866	Female												
KBTBD8													0.03236	-0.72005	Male
CCDC107				0.01780	-1.07935	Male									
PTPRJ										0.04752	0.87218	Female			
CTSA	0.02113	0.91799	Female							0.00001	0.61570	Female			
PACSIN2	0.01283	-0.79409	Male												
SNX8										0.00001	0.58720	Female			
FAM83G				0.00000	2.80144	Female									
FGF11										0.03421	-1.40975	Male			
TET1				0.00050	0.71044	Female				0.00000	0.60007	Female			
SUSD2										0.00000	1.18878	Female	0.00706	0.69031	Female
FHDC1				0.00530	-1.82401	Male				0.02298	-0.98345	Male			
NKRF				0.00000	1.39988	Female	0.00002	0.84009	Female	0.00017	0.71371	Female			
CEP72	0.00733	-2.09812	Male												
SIX1										0.01311	2.48425	Female	0.03851	2.09868	Female
TAB3										0.00950	0.71579	Female			
TGFB3										0.00006	2.04273	Female			
ATP11C				0.00000	1.56747	Female	0.00000	0.81027	Female	0.00000	1.30391	Female			
CIDEC													0.00103	1.14007	Female
KRT23				0.00048	-1.00041	Male									
NFIL3										0.00001	-1.22328	Male			

NRF1				0.02386	-0.74095	Male									
ARL13B							0.00007	1.14087	Female						
TIMM8A	0.00008	0.91094	Female	0.00000	1.11093	Female	0.00000	1.11624	Female	0.00000	0.74008	Female			
GCNT1				0.01288	-1.44136	Male	0.00225	1.16601	Female	0.00547	1.03020	Female			
NRDE2				0.02561	-0.65788	Male									
DLG2							0.00549	1.01363	Female						
CASP9													0.03087	0.78936	Female
HDAC8				0.00000	1.52203	Female	0.00000	1.23410	Female	0.00000	1.28746	Female	0.00002	0.92106	Female
SP140L				0.01394	1.94448	Female							0.00926	1.33983	Female
ISOC2				0.01400	1.01055	Female	0.00707	0.73254	Female	0.00000	1.32746	Female	0.02314	0.60466	Female
VEGFB							0.00266	-1.41034	Male	0.03328	-0.99451	Male			
ACSL4										0.00558	0.61447	Female			
REC114													0.00036	-2.67818	Male
SH3PXD2B										0.01437	1.21577	Female			
GPANK1				0.00000	-0.77691	Male									
TIMM8B	0.00275	0.62447	Female												
SAP30L-AS1	0.00816	3.05252	Female												
TGIF1	0.01700	-0.83631	Male												
LGALS4										0.00001	1.88088	Female			
CLPTM1L							0.00000	0.74493	Female						
LPAR5				0.02209	2.87641	Female									
CGNL1										0.00377	1.51625	Female			
PLSCR1													0.02860	-0.59135	Male
CRHBP										0.04584	1.60837	Female	0.00124	-2.43508	Male
SLC35B4				0.03165	0.61773	Female									
LOC391322							0.02703	-1.03221	Male						
ANKRD20A5P										0.00314	1.69239	Female	0.00857	1.51832	Female
SNHG5				0.00000	-1.00010	Male							0.00000	-0.90935	Male
AUTS2										0.00233	1.07915	Female			
CBR3										0.00000	2.31937	Female			
CA5BP1				0.00130	0.87572	Female	0.00000	1.65283	Female	0.00002	0.73714	Female	0.00005	0.69007	Female
GCDH							0.00000	0.83088	Female						
INA							0.02348	0.75231	Female						
DHRS4-AS1										0.03646	-1.51519	Male			
HMGN2P46										0.00935	-1.48382	Male			
ARL4D							0.00001	0.67867	Female						
CYFIP2													0.00870	1.44164	Female
MT1G													0.03748	1.03729	Female
ZNF415													0.00001	-1.36120	Male
RAB9A				0.00034	0.76388	Female									
PMAIP1										0.00006	1.40022	Female			
MIR22HG	0.00613	1.51235	Female	0.01188	-0.89488	Male									
SNORD12C				0.00728	-1.08386	Male									
H3F3C							0.00000	1.16492	Female						
LSS							0.00000	-1.12253	Male						
CRAT							0.00444	-1.82470	Male						
PFKFB2							0.04074	-0.68384	Male						
BMF				0.00023	-2.42615	Male									
FASTK							0.00996	-0.58929	Male						
DARS	0.01806	0.58448	Female												
LPIN3							0.00000	1.37343	Female				0.00648	0.68188	Female

PTRF	0.01307	2.20629	Female	0.01306	1.40495	Female							0.01174	0.91649	Female
SNAR-I				0.00002	1.51114	Female	0.00001	1.08758	Female	0.00003	1.01715	Female			
DLGAP3										0.00000	2.38659	Female			
CYP51A1							0.00004	-0.61969	Male						
TRIM43										0.01955	6.25041	Female			
C14orf80	0.01101	-2.04335	Male										0.00500	0.93459	Female
TMCO6	0.01750	2.25656	Female				0.00512	-1.09636	Male						
MAK16	0.01581	-0.72119	Male												
WDR13							0.00153	0.90218	Female				0.03414	0.61220	Female
NDUFA1	0.00000	1.13300	Female	0.00000	0.99716	Female	0.00000	0.80168	Female	0.00000	0.69878	Female	0.00000	0.87029	Female
KCTD13				0.04036	0.96152	Female									
TRAF3IP1							0.00215	1.03222	Female				0.00289	0.94503	Female
IKBKAP	0.00074	1.44003	Female												
CCDC170										0.00153	-2.58332	Male	0.00495	-2.36789	Male
APOOL				0.02725	0.58083	Female	0.00000	1.06849	Female						
MAP7D3				0.03053	0.77457	Female	0.00136	0.72170	Female						
SESN3				0.01069	1.44396	Female				0.00000	1.57717	Female			
GYG2										0.03341	1.13747	Female			
PLS3				0.00000	1.19344	Female									
CDX1							0.00000	1.66336	Female						
LINC01447							0.00426	2.38671	Female						
BUB1										0.00000	1.58157	Female			
OTUD6A							0.04441	2.23510	Female						
EFR3B							0.00249	0.81398	Female				0.00014	0.93746	Female
FAM107B										0.01067	0.59092	Female			
FCHSD2	0.00685	-2.44344	Male												
RPS4Y1	0.00000	-9.31666	Male	0.00000	-9.45687	Male	0.00000	-11.05569	Male	0.00000	-10.47092	Male	0.00000	-8.98075	Male
PPAP2B				0.00319	0.99723	Female									
POLR2J4							0.02699	0.60996	Female						
DOHH							0.00728	-0.83806	Male						
ISYNA1	0.00013	-2.09996	Male	0.00000	2.66713	Female									
RPAP1							0.00000	-0.91070	Male						
ALDH2										0.00183	0.74232	Female			
AGGF1							0.00001	-0.74215	Male						
PGAP2							0.00000	-0.95936	Male						
PGM1							0.00189	-0.91422	Male	0.02030	0.68707	Female	0.00933	-0.74068	Male
HTRA2	0.00829	0.91723	Female												
MDFI	0.00000	2.38708	Female	0.00000	2.75262	Female	0.03269	1.20167	Female						
GAGE1	0.00043	3.18219	Female				0.00000	2.90962	Female	0.00000	2.48396	Female	0.00004	1.50252	Female
AKT1				0.01558	0.98531	Female									
CERS6							0.03824	-1.46930	Male						
SLC25A25							0.04745	0.61674	Female						
USP2							0.04118	0.77374	Female						
PPDPF										0.00000	1.18919	Female	0.00153	0.68931	Female
GPKOW				0.00542	0.93171	Female	0.00003	0.90230	Female	0.00138	0.68825	Female			
SNORA53													0.01504	1.50848	Female
SYT2										0.00002	1.38235	Female			
STARD10							0.00242	-0.72088	Male						
FDXACB1							0.04669	-0.83473	Male						
RWDD2B							0.03082	-0.99060	Male						
ALYREF										0.00000	-0.63373	Male			

ZNF679										0.00000	-3.37744	Male			
TMEM200A				0.01324	2.86442	Female				0.00076	-2.62803	Male			
HS3ST3A1							0.02761	0.95207	Female						
RPAP2							0.01317	-0.58402	Male						
TOP2B				0.00011	1.00669	Female									
FAM83B	0.01636	1.99258	Female				0.00004	-1.29238	Male				0.04514	-0.65867	Male
UBL4A	0.00000	3.25343	Female	0.00430	0.77639	Female									
FTX	0.01592	2.14135	Female							0.01368	0.87195	Female			
LINC01278				0.00183	1.34640	Female									
TIGD2							0.00512	-1.52765	Male						
LAMA2										0.02791	1.26846	Female			
FOXH1				0.01035	1.14120	Female				0.00138	0.89462	Female			
NAA10				0.00000	1.15899	Female	0.00000	0.75203	Female						
ZNF618										0.00007	2.38323	Female	0.00102	-2.00727	Male
FAM8A1													0.04071	-0.60677	Male
NCBP2	0.00006	-0.78032	Male												
SLBP							0.00000	-0.67248	Male				0.00000	-0.65595	Male
LRP4										0.00098	1.37332	Female			
TRPM1										0.02400	-1.94775	Male			
ZG16							0.00402	0.71091	Female						
AXL				0.00110	-1.23194	Male				0.00000	-1.09037	Male	0.00096	-0.81318	Male
IMPA1							0.00103	-0.71036	Male						
TLCD1							0.00074	-0.78976	Male						
ETV4										0.00000	2.02998	Female			
RAP1GAP2										0.00328	1.34358	Female	0.02542	1.05102	Female
EFCAB10													0.01319	0.88748	Female
RRAGB							0.01217	1.07962	Female	0.00250	1.20666	Female			
MYT1L										0.00560	-1.93502	Male			
ABAT										0.00003	2.41277	Female			
KLHL2													0.01830	-0.76044	Male
MGC45800										0.00458	-1.92672	Male			
FAM217B	0.00860	1.92369	Female												
SEPHS2				0.00148	0.83759	Female									
FARSA							0.00000	0.67909	Female						
POP1							0.00003	0.68774	Female						
HIVEP3													0.01885	0.85414	Female
COLQ							0.02542	1.79676	Female						
SFN										0.00262	-0.70832	Male	0.00001	0.96607	Female
WRB							0.00080	-0.74350	Male						
PLD1							0.00343	-1.64093	Male						
TMEM18				0.00098	0.79522	Female									
SLC2A14	0.03414	1.39060	Female										0.00869	0.65800	Female
ACE							0.02360	0.97070	Female	0.00000	2.96502	Female			
KIAA1549L										0.01588	1.89326	Female			
MAP3K15							0.02025	1.48614	Female						
PCSK7				0.00691	0.89243	Female									
MYLPF										0.00000	-1.15531	Male			
TP63				0.01890	1.60036	Female	0.00535	1.29853	Female						
CCDC7				0.00214	-0.65693	Male									
POMK										0.00394	-0.88844	Male			
ZNRF2													0.02138	-0.91056	Male

MAGEC1				0.00827	2.74167	Female	0.01777	1.80980	Female				0.00045	0.74968	Female
F2RL1				0.00147	-0.74835	Male									
MRPL44	0.00124	-0.74270	Male												
FAM212B										0.01038	1.60052	Female			
WDR6										0.00818	-0.58166	Male			
LACTB2							0.01713	-0.74104	Male						
HAPLN1										0.00110	-0.96541	Male	0.00111	-0.94234	Male
TINAGL1										0.02579	-0.77574	Male			
PCLO										0.00228	1.66058	Female			
WNK2													0.04594	0.90481	Female
MED29													0.00000	0.62605	Female
KIF4A							0.00000	0.70709	Female						
QRICH2							0.00683	1.29493	Female						
GINS3							0.00000	-0.82914	Male				0.00008	-0.61642	Male
DEPDC7										0.00076	1.53323	Female			
GTF2I										0.00064	0.87093	Female			
PLEKHH1				0.00005	-2.49317	Male									
TRIM24				0.03778	0.79157	Female									
KATNBL1				0.02452	-0.77969	Male									
AICDA							0.01418	2.31250	Female	0.00000	3.19631	Female			
SYNGR3							0.04796	0.63803	Female						
HES1				0.00040	-1.57283	Male									
PLA2G4F										0.02504	1.08386	Female	0.01692	1.11602	Female
FAM214A													0.00000	-1.50901	Male
DPPA2										0.00002	1.04334	Female			
VAMP3	0.01042	-0.83426	Male												
SPHK2							0.01824	-0.61836	Male	0.00000	-1.33341	Male			
PRPS1				0.00000	1.14281	Female	0.00000	1.32780	Female						
ANKRD11				0.01005	0.60409	Female	0.00002	0.61891	Female						
UGDH							0.01493	-0.58505	Male						
FHL1										0.00088	-2.82594	Male			
UBE2Q2P1										0.04349	1.68983	Female			
METTL2A	0.00207	0.97265	Female												
HORMAD2-AS1										0.02265	0.73020	Female			
VCX3B	0.00001	3.81261	Female	0.00000	3.41708	Female	0.00000	2.98189	Female	0.00076	1.36547	Female	0.00000	4.53617	Female
PSMD10				0.00010	0.73816	Female	0.00000	0.64899	Female	0.00000	0.59307	Female			
SCMH1							0.02774	-1.32200	Male						
NME4										0.01142	-0.72612	Male	0.01986	-0.66501	Male
CALB2				0.02510	-2.34201	Male							0.00000	3.59048	Female
MFSD9	0.01779	-1.71856	Male												
TPRG1L							0.00679	0.69986	Female						
MANEA							0.00001	-1.13491	Male				0.00717	-0.68438	Male
LOC645513										0.00010	1.02572	Female			
MT2A													0.00008	1.41807	Female
ARHGAP24				0.04102	1.26103	Female	0.00003	-1.56766	Male	0.02234	-0.88000	Male			
JAG2				0.01402	1.31488	Female				0.00076	1.12392	Female			
LRRC8B							0.00000	-1.80421	Male	0.00008	-1.27816	Male	0.00601	-0.92285	Male
CDC42										0.00000	-0.59064	Male			
LINC01234										0.04860	1.07896	Female	0.00008	1.88477	Female
PMP22										0.00000	1.93195	Female	0.00379	1.02363	Female
BEX1										0.00555	-1.59459	Male	0.00505	-1.51852	Male

SLC30A5							0.00040	-0.60438	Male						
PPME1										0.00000	-0.87609	Male			
MYO9A				0.03064	0.75606	Female				0.00164	0.66309	Female			
WASH1										0.01096	-0.67879	Male	0.00047	0.88007	Female
ACP6										0.01954	-0.98018	Male			
RNF6													0.00070	-0.59902	Male
FAM50A	0.00174	-0.97019	Male	0.00000	1.11586	Female	0.00000	0.72210	Female				0.00000	0.72195	Female
MAGED2	0.00006	3.74677	Female										0.00041	-1.39739	Male
GLE1	0.04947	-1.06273	Male												
NAA11													0.00181	-0.66750	Male
C1orf233													0.02284	1.11320	Female
HUWE1				0.00000	1.18110	Female				0.00000	0.91328	Female	0.00000	0.79153	Female
SKA1				0.00531	-0.62796	Male									
ZNF490										0.01487	0.68043	Female			
PPP2R5B										0.02256	1.05624	Female			
MBD3L3				0.02309	-2.68636	Male				0.01712	4.53425	Female			
LRP2	0.00110	-1.88583	Male												
SLC38A2				0.00002	-1.19606	Male	0.00001	-0.85985	Male						
PTMS													0.00013	0.80034	Female
SUMO1P1													0.00833	-2.27223	Male
CCDC163P										0.00152	0.84558	Female			
STMN2										0.00049	-4.64513	Male			
RASL11A													0.01001	-1.12933	Male
SFI1				0.03476	1.28688	Female									
DNPEP							0.00527	0.80585	Female				0.03103	0.61303	Female
SNORD63													0.00494	0.66069	Female
ZNF354A													0.00639	0.87925	Female
MOGAT3										0.00000	3.23219	Female			
ZNF277							0.00053	-0.73457	Male	0.00100	-0.66864	Male			
TCL1B										0.00317	1.17337	Female			
SNHG7													0.00001	0.70242	Female
LY6G5C							0.02875	-2.20925	Male						
PGLS	0.00105	1.63866	Female												
MYO15B													0.00007	2.30291	Female
AXIN1				0.01245	0.89406	Female							0.00270	0.66221	Female
SGPP2							0.01486	-1.42594	Male						
BTN1A1										0.00089	-1.98550	Male			
PPP1R32							0.03438	-1.80736	Male						
ENOX2							0.00001	1.11169	Female						
ALPP							0.00000	-1.06652	Male						
MORN1													0.04006	1.02450	Female
CIC	0.01007	-1.73504	Male												
DND1							0.01503	0.72209	Female	0.00021	0.98166	Female			
AK9				0.00503	-2.42559	Male									
KCNJ3										0.00000	-1.41488	Male	0.00000	-1.73897	Male
SEPW1										0.00000	0.62127	Female			
FAM129B							0.00054	1.36512	Female						
TSPAN3							0.00085	-0.62940	Male						
SSX6							0.00001	2.37482	Female	0.01104	1.42995	Female			
TGDS													0.00121	-0.62000	Male
TGM2	0.00000	2.49664	Female										0.00021	1.29681	Female

WNT7B				0.00241	1.87760	Female	0.01610	-0.94412	Male						
CLIC3										0.00028	-1.22574	Male			
LHFPL3-AS2										0.00045	0.83444	Female			
GLIPR2				0.01937	0.58729	Female									
ARL4A										0.02479	0.59452	Female			
NOL9	0.01648	-0.84873	Male												
CCDC124	0.01011	-0.59982	Male												
TNS3				0.00000	1.61782	Female									
GYPC										0.00000	3.75179	Female			
SNX29				0.01288	1.05878	Female									
NAGA							0.04985	0.63290	Female	0.00005	1.11230	Female			
BLOC1S2	0.03710	0.67660	Female												
SNORD104													0.00001	0.88360	Female
SNORD10	0.00594	1.83060	Female							0.00726	-0.70118	Male	0.00001	1.06633	Female
NSUN6							0.04263	-0.59710	Male						
TFPT				0.00003	-0.94511	Male									
CGB8										0.00000	-2.50946	Male			
ACVR1B													0.00969	0.79549	Female
LOC102724153							0.00267	1.73695	Female	0.03952	1.22001	Female			
GPR126				0.03039	1.45050	Female							0.01405	-0.97758	Male
RANBP17										0.02163	0.78672	Female			
SHB										0.00184	0.91369	Female	0.00681	0.79923	Female
TMCO4										0.03336	1.19370	Female			
RCBTB1							0.01394	-0.63878	Male	0.00000	1.19022	Female			
RPP25L							0.01125	0.59029	Female						
NT5DC1							0.00200	-0.75611	Male						
ACO1										0.00027	0.76213	Female			
CPZ													0.00056	1.75379	Female
AIRE				0.00009	2.86872	Female				0.00000	3.23400	Female			
LAMA5				0.00009	1.18945	Female									
MIDN				0.00000	1.01489	Female	0.00003	0.64542	Female						
TBCD										0.00000	1.36586	Female			
WASF1							0.00000	-1.16694	Male	0.00873	-0.64018	Male			
TMPPE													0.04921	0.85065	Female
NKAP	0.01074	1.00068	Female	0.00017	0.82254	Female	0.00000	1.35248	Female	0.00000	1.14389	Female			
CLEC4GP1							0.02694	-1.34003	Male						
MORC4				0.00312	1.20845	Female				0.00326	0.78612	Female			
SIRT2							0.04395	0.62804	Female						
SHD										0.00076	1.80654	Female			
RHOU	0.01191	1.91174	Female												
HEBP1							0.00232	-1.05379	Male						
KIAA0895										0.02342	-1.48262	Male			
TIGIT										0.00582	-5.33296	Male			
MED17							0.00101	-0.61433	Male						
LINC01108										0.00848	1.37517	Female			
PPP1R26										0.00022	2.13703	Female			
LBR							0.00000	-0.58143	Male						
G6PD				0.01449	0.67563	Female									
CNOT3				0.02983	0.75037	Female									
IZUMO4							0.04647	-1.99548	Male						
FAM200A							0.00953	-0.98359	Male						

ZNF619													0.00203	1.94628	Female
EIF1AY	0.00000	-6.27476	Male	0.00000	-8.53148	Male	0.00000	-9.75581	Male	0.00000	-9.81738	Male	0.00000	-9.01656	Male
APEH	0.04026	0.86475	Female												
HIST1H3H				0.00792	-2.05326	Male									
MIR1973										0.00030	0.96313	Female			
CT55							0.00024	2.01156	Female						
TMEM37							0.01165	0.81475	Female	0.00080	0.99833	Female			
ZNF132										0.02116	-1.55161	Male			
C16orf59				0.00889	-1.25555	Male	0.00278	-0.95970	Male						
HIST1H4I							0.00002	-1.89327	Male	0.03110	-0.98829	Male	0.03553	-0.95233	Male
NARS	0.00003	-0.76750	Male												
DMXL2							0.00246	-1.19499	Male						
HIBADH							0.00490	-0.74529	Male						
PRAMEF19.uid-875							0.01808	0.85461	Female						
SNAR-E							0.00000	1.06178	Female	0.00000	0.98568	Female			
ACADS										0.00000	1.54492	Female	0.01649	-0.83694	Male
ARHGAP42										0.01311	0.77220	Female			
RFPL4B										0.02777	-0.70651	Male			
ADCK4							0.00975	-0.95546	Male						
LINC00152				0.00004	1.69556	Female	0.00001	1.00544	Female				0.00948	0.59224	Female
SELO	0.02275	-1.91694	Male	0.00637	1.39370	Female									
SAA1										0.03508	-1.33796	Male			
CNKSR1										0.01693	-0.86887	Male			
PDE6C				0.00009	3.94779	Female									
HPD													0.04235	1.40027	Female
SNORD50A										0.00000	-0.93233	Male			
HSPA12B													0.02415	1.94244	Female
UGGT2													0.00068	-0.81210	Male
PRMT5-AS1							0.00046	0.81138	Female				0.00000	1.00838	Female
TOPORS-AS1							0.00416	0.71600	Female						
LOC101928012										0.01096	1.16896	Female			
PAX8-AS1.uid-3716							0.01296	0.84730	Female						
SDHAP1	0.00322	1.04590	Female				0.00000	0.77674	Female						
PYGB				0.00651	0.78147	Female				0.00000	0.94626	Female			
TSR2				0.00002	0.85910	Female	0.00000	1.27730	Female	0.00000	0.80409	Female			
EMB													0.00422	-2.60952	Male
EFHD2				0.00000	1.41174	Female									
RPL10	0.00065	0.62077	Female	0.00000	0.65224	Female	0.00000	0.64325	Female	0.00000	0.77890	Female	0.00000	0.76620	Female
NIPA2							0.00000	-0.68519	Male				0.00000	-0.74635	Male
PDCL3							0.00000	0.59220	Female						
WASH3P							0.00073	1.06168	Female						
APOBEC3C										0.00084	2.48201	Female			
PRSS22													0.00709	-1.30769	Male
XRRA1													0.00898	0.79066	Female
SRXN1				0.04772	0.72345	Female									
SLC40A1	0.00311	-2.43806	Male												
C4orf27	0.03287	-0.80620	Male										0.00000	-0.65566	Male
GPRC5A				0.00000	2.07147	Female				0.01654	0.73625	Female			
SLC15A2							0.01315	-1.12652	Male						
PSORS1C2										0.00000	2.67471	Female			
ARID3B				0.00215	-0.71241	Male									

LOC100506655	0.00099	3.00384	Female												
TMEM62							0.00504	-0.69504	Male						
SLC33A1										0.00433	0.59949	Female			
SEPT9				0.00411	1.10444	Female	0.00125	0.82795	Female						
THOP1	0.00529	-1.39245	Male												
LEFTY1	0.00000	0.89122	Female							0.00000	3.13977	Female			
YJEFN3							0.00000	1.50948	Female	0.02675	-0.64289	Male	0.00678	0.74295	Female
CCDC178													0.00133	1.87294	Female
BCL2L10													0.01104	-2.59770	Male
ELANE							0.00046	-2.67627	Male						
ABHD12B										0.00141	0.94391	Female			
PLXNB3							0.00362	2.19508	Female	0.00005	2.77206	Female			
IL11RA	0.00574	2.89394	Female	0.02825	-1.49666	Male									
TENM3				0.00888	-1.26460	Male				0.00785	-0.79746	Male			
KCNH2										0.00723	2.16217	Female			
APELA										0.02052	0.92683	Female			
BRF1													0.03254	0.67424	Female
EIF4EBP1				0.00000	1.11508	Female									
NR5A2										0.00103	1.05344	Female			
FAM45B.uid-12498										0.00000	0.79355	Female			
PKN3				0.00069	-1.79003	Male									
IKBKG							0.00449	0.74122	Female	0.02803	0.58245	Female	0.00042	0.83330	Female
VBP1				0.00021	0.59692	Female				0.00000	0.59506	Female			
FBP1										0.00405	1.83261	Female			
ARL17A.uid-21953							0.03584	1.67450	Female						
SEMA7A							0.00012	-1.72160	Male						
ATP9B										0.01542	0.61365	Female			
HIPK2										0.00020	1.70920	Female	0.01606	-1.19933	Male
HIST1H2BJ							0.00573	1.29555	Female						
DHFR										0.00000	-0.72905	Male			
CAPN12										0.00000	2.04383	Female	0.01696	1.10403	Female
SH3BP5L										0.01429	-0.79761	Male			
ISL2													0.01108	2.01724	Female
SNRPD1	0.00000	-0.69480	Male												
ODC1	0.03307	-0.69619	Male												
TXLNG	0.04258	0.71360	Female	0.00000	1.15153	Female	0.00000	0.59777	Female	0.00000	0.65231	Female			
FAM178B							0.02356	1.02193	Female						
SLC23A1													0.01572	1.49103	Female
SPSB1													0.01794	0.81656	Female
SF3A2	0.02218	-1.07467	Male												
IBTK													0.00175	-0.61303	Male
TMPRSS4													0.01809	-1.37136	Male
CHRAC1							0.00000	-0.76337	Male	0.00004	-0.64850	Male			
KRT18P55	0.00000	-3.75021	Male				0.00481	0.62835	Female						
PSMG3-AS1				0.00584	-1.68575	Male									
CCKBR	0.00000	2.89552	Female				0.00000	-1.22161	Male	0.00000	-1.48136	Male			
ACADSB				0.00045	1.25897	Female									
KIAA0101							0.01108	-0.63900	Male				0.00543	-0.63554	Male
GTF2H3							0.00000	-0.94624	Male						
ECHDC3				0.00000	3.53977	Female	0.03866	-1.26460	Male						
DCK							0.00623	-0.69189	Male						

COL18A1										0.02488	1.67951	Female			
CXorf57													0.03065	-1.55725	Male
GATAD2B													0.00542	0.63330	Female
KCNC4										0.01097	1.21982	Female			
DEPDC1B							0.00066	-0.74670	Male						
SHANK2							0.00579	1.38552	Female	0.00003	1.90425	Female			
CDH8				0.02716	2.01304	Female				0.02092	1.39149	Female			
BCL9							0.01834	-0.68119	Male						
LIM2							0.03880	1.70603	Female	0.00019	2.65064	Female			
RABEP2										0.02893	-0.76928	Male			
RPSAP9										0.02525	-0.85641	Male			
FCF1	0.00355	-0.75506	Male												
DDHD1				0.01229	0.67123	Female									
GRAMD1C										0.04715	1.35921	Female			
SYCP3													0.00000	0.62090	Female
NIPSNAP1													0.04235	0.60545	Female
HIST1H3B				0.00067	-2.34961	Male				0.01512	1.20872	Female			
ABCA17P							0.04682	0.99826	Female						
CDC42BPG	0.02404	1.95255	Female												
DLX5							0.03060	-0.90479	Male						
CAB39L							0.02155	-0.64707	Male	0.00077	-0.84258	Male	0.00001	-1.05109	Male
IL6R										0.00000	1.92466	Female			
LIMCH1										0.00000	3.24333	Female			
ZNF610													0.00316	1.46097	Female
ENDOG				0.00006	1.60275	Female									
SMARCD1							0.00163	-0.58541	Male						
SKIDA1				0.02543	0.97269	Female	0.02718	0.63761	Female						
KLF6				0.03652	0.81942	Female	0.00016	0.87454	Female				0.00000	1.03628	Female
NRBP2										0.00000	1.91582	Female	0.00982	1.02678	Female
C15orf40	0.02835	1.13961	Female												
LAT							0.04376	1.10715	Female						
SERPINB6										0.00000	1.50878	Female			
EMC1	0.00733	1.21204	Female												
URAD							0.00072	-1.81978	Male						
SNORA4										0.00348	0.80874	Female			
PTPN5							0.00015	-2.24273	Male	0.00191	1.81736	Female			
CELA3A										0.02739	1.10636	Female			
JAKMIP1										0.00477	-0.86528	Male			
CECR1										0.00000	3.08528	Female			
NT5C3A				0.00759	0.66112	Female									
GCLM										0.00026	-0.69593	Male			
DOK2							0.03861	1.68698	Female						
DUSP10										0.00817	1.91575	Female			
GMFB										0.00000	1.23403	Female			
SYNE4										0.01461	-1.57618	Male			
ITLN2										0.00498	1.23723	Female	0.00026	1.51276	Female
CTHRC1							0.00891	-1.74575	Male						
ASS1	0.00171	2.17773	Female												
ATP10A										0.00779	2.25438	Female			
MMP24							0.04062	1.05195	Female	0.00500	1.33370	Female			
ABRACL	0.04324	-1.01423	Male	0.00114	0.82661	Female	0.00061	-0.59585	Male				0.00007	-0.63586	Male

YEATS4							0.00000	-0.60338	Male						
HSPA2										0.00031	-0.99790	Male			
ANKRD46							0.01840	-0.93599	Male						
TMEM219							0.00036	-0.69708	Male						
TMEM261	0.00261	0.85839	Female	0.00000	0.86175	Female									
CCR7				0.00000	-1.86286	Male									
LRCH4										0.01326	0.73492	Female			
HDAC7										0.00124	1.58170	Female			
PEX12							0.01697	-0.93692	Male						
PRELID2							0.02102	-1.40178	Male						
ZFR2										0.00373	1.74350	Female			
UBXN6							0.00579	0.67757	Female						
SLC25A5	0.00000	1.39078	Female	0.00000	1.10371	Female	0.00000	1.15505	Female	0.00000	1.01317	Female	0.00000	0.89787	Female
MID1				0.04950	0.85729	Female				0.00243	-0.77985	Male			
HIST1H3D				0.03369	-1.55680	Male	0.01283	1.21386	Female						
1.00000				0.00000	1.15878	Female	0.00000	0.92934	Female						
ARL5A										0.00000	-0.62838	Male			
SLC27A2							0.00000	-1.94373	Male	0.00000	-1.48211	Male	0.00000	-1.54956	Male
SPDYC										0.04516	0.91502	Female			
BLACE													0.02135	-1.20913	Male
CCNT2-AS1										0.00196	-1.55424	Male			
PER3										0.00145	-1.07439	Male			
LOC100129534				0.00447	0.98662	Female	0.00100	0.78634	Female						
KIAA0141				0.03385	-0.97026	Male	0.00120	-0.91937	Male						
C5orf28							0.03056	-0.70784	Male						
TSPYL6										0.00936	-1.25071	Male			
TSPAN15				0.00211	-1.31685	Male									
MAP2K4P1							0.01339	-0.87453	Male						
MVP							0.01755	0.82241	Female						
TMC6							0.02473	-1.38626	Male						
TUBGCP5													0.00400	-0.81831	Male
FANCB				0.02277	0.76049	Female	0.00000	0.95097	Female						
S100A10	0.00000	-1.90955	Male												
HIST1H2BH	0.03092	1.99379	Female				0.01180	1.03245	Female	0.03150	0.85952	Female			
ZNF107							0.00281	-0.68489	Male						
ANKRD20A9P										0.00030	-1.31292	Male			
ZNF667-AS1													0.00042	0.90488	Female
METTL9				0.02100	0.58749	Female							0.00007	-0.58333	Male
RHOBTB1										0.00056	-1.19137	Male			
ABHD4	0.00002	3.21021	Female	0.00000	1.53935	Female	0.00077	-1.14713	Male	0.01570	-0.81768	Male			
NLRP5													0.00000	-4.02136	Male
LDLR				0.00000	1.75844	Female	0.00692	-0.71453	Male				0.02460	0.58261	Female
CBR4													0.00000	-0.78844	Male
RBBP7				0.00009	0.69749	Female							0.00000	-0.75616	Male
NODAL										0.01230	1.60894	Female			
MKRN9P													0.01080	-0.58921	Male
GTF2H2							0.00185	-0.61947	Male						
MMP24-AS1										0.00091	1.26312	Female			
CAMK2D	0.00108	2.88268	Female				0.00724	-0.96645	Male						
EXT1										0.03414	0.65586	Female			
C1orf168				0.03528	1.98662	Female									

KLF4							0.01729	0.63049	Female	0.00000	1.79376	Female			
SPATA22							0.02164	0.79135	Female						
C1QTNF9										0.01366	2.19304	Female	0.00022	-3.03984	Male
SFXN1	0.00074	-1.08873	Male												
HBM				0.02674	-2.55662	Male									
MYLIP										0.00700	0.74423	Female	0.01814	0.65838	Female
DGAT2										0.00993	1.91466	Female	0.03823	1.55834	Female
RMI1							0.00006	-0.79883	Male						
AK6	0.01593	-0.72122	Male												
ZNF880										0.00404	-0.73526	Male			
EPS15L1				0.00209	0.78056	Female									
DVL1				0.03693	0.89435	Female									
AMHR2													0.04522	1.39264	Female
ZNF771										0.01182	-0.93288	Male			
KIAA1161				0.00000	2.57557	Female									
TPD52							0.00001	-0.93293	Male						
FZD6							0.02641	0.98601	Female						
GNG12										0.01058	-0.84861	Male			
RAP2A													0.00270	-0.65296	Male
ATP6AP2				0.00000	0.91135	Female	0.00000	0.64940	Female	0.00000	0.62400	Female			
TUBA4A							0.00130	1.57636	Female	0.00000	-3.27196	Male	0.00082	-1.51885	Male
EMP3										0.00225	1.79368	Female			
CACNA1A										0.00001	-1.27479	Male	0.00166	-0.92651	Male
RBM46										0.00000	-1.96041	Male	0.00574	-1.19977	Male
APOC1	0.02275	-0.63959	Male							0.00000	0.96251	Female			
HSD3B1							0.03213	-1.13326	Male						
TACC3	0.04397	-0.64103	Male												
TXN	0.00001	-0.81623	Male												
MAFG-AS1							0.02025	1.62601	Female						
B3GAT3										0.00975	-0.71753	Male			
C11orf96							0.00232	2.57012	Female	0.01610	2.08300	Female			
TACSTD2				0.03563	-0.70162	Male	0.01450	-0.72861	Male	0.00373	-0.79076	Male			
NOG							0.00155	1.01953	Female				0.00000	-2.25544	Male
RALGPS1							0.00714	-1.15670	Male						
C6orf223							0.00000	-1.87979	Male						
GUSBP1							0.00264	0.61291	Female						
DLEU1							0.00011	-0.64875	Male						
FKBP8										0.00532	0.62519	Female			
RPS6KA3							0.03550	0.63478	Female						
MRPL38	0.00074	-0.77699	Male												
TMX2	0.00068	-0.67350	Male												
KBTBD3							0.02704	-1.35480	Male						
FAR2P1													0.03321	1.35181	Female
GSTM1													0.00347	1.68110	Female
TRIM8				0.01250	1.22178	Female	0.01501	0.80442	Female	0.00000	1.33519	Female			
FER							0.02433	-0.60361	Male						
WWC2-AS2	0.03033	3.21808	Female												
ARHGAP30							0.00595	1.98426	Female						
PRAMEF12										0.00885	2.14979	Female			
TDRD6													0.01362	-1.29652	Male
LOC100133077							0.03032	2.04027	Female						

TOX2													0.00067	1.84616	Female
CEPT1	0.00996	-1.00874	Male												
IL13RA2										0.01664	2.17348	Female			
ATF6B										0.02453	-0.68476	Male	0.03262	0.65017	Female
HEATR6	0.00052	2.43862	Female												
COQ2							0.00000	-1.24617	Male				0.00124	-0.78823	Male
FAT2							0.00043	2.16047	Female						
KCNN1										0.00000	2.86682	Female	0.00822	1.58898	Female
PIF1										0.02116	0.65527	Female			
TJP2				0.00000	1.99200	Female									
LINC01118													0.00007	-5.25767	Male
GPR153										0.00001	2.61639	Female	0.01426	1.54923	Female
RAB31				0.00001	1.78339	Female	0.00000	-1.30753	Male	0.00007	-1.02160	Male			
SERPINB1				0.00000	2.84665	Female									
STK36										0.00456	1.13506	Female			
TMEM27				0.02395	2.30323	Female									
ZNF140										0.04812	0.62605	Female			
GOLGA3							0.00000	0.76638	Female						
ARAF							0.00000	0.89193	Female	0.00095	0.59956	Female	0.00004	0.70758	Female
ZNF276							0.04267	0.66021	Female						
FAM122B	0.03152	1.44222	Female	0.00059	1.29161	Female	0.00001	1.08797	Female	0.00317	0.74795	Female			
ZFAND2A							0.00000	0.81123	Female	0.00000	-1.04958	Male			
UBALD2													0.00674	0.67988	Female
NUP62							0.00000	0.61150	Female						
DCAF8L2													0.04739	0.97369	Female
MAPKBP1							0.00074	-1.12777	Male						
IDH3G				0.00005	0.87320	Female	0.00000	1.21235	Female	0.00000	0.83905	Female	0.00000	0.70751	Female
EFNB3										0.00052	1.65512	Female			
ANXA2P3				0.00000	-0.82025	Male									
MSMO1				0.00043	-0.85212	Male	0.00044	-0.58883	Male						
BBC3							0.00098	1.32567	Female						
TTLL1							0.03125	-1.69939	Male						
ZMYND11													0.00264	-0.73160	Male
RIBC1													0.00912	1.98299	Female
ZNF789							0.00002	-0.94133	Male						
VPS9D1-AS1										0.00292	0.85897	Female			
TMEM52B	0.00000	3.53591	Female												
DPM3							0.00000	0.90943	Female				0.00025	0.68573	Female
TMPRSS13							0.03249	-0.74655	Male	0.01304	-0.78347	Male			
HAS3										0.00009	-1.94036	Male			
RIMKLB										0.00000	1.22855	Female	0.00004	0.75437	Female
CS	0.00158	-0.62182	Male												
LOC100862671							0.03384	0.70709	Female						
ITPK1				0.04090	0.65219	Female									
CHMP7										0.00001	-0.80533	Male			
ELOVL5							0.00000	-0.88007	Male						
ZC3H12B	0.00289	2.53801	Female				0.00101	1.19296	Female				0.00983	0.92311	Female
PRSS12				0.04165	1.36271	Female									
STC2				0.00000	-3.47917	Male				0.00355	0.76525	Female			
ITGA2										0.00867	1.64557	Female			
HOXB9										0.00288	2.56012	Female			

SETD9	0.00411	2.42976	Female				0.00384	-0.97633	Male						
SHISA5				0.00021	0.71502	Female									
ANXA4										0.00058	1.73998	Female			
HIST2H2AC				0.04067	-1.16483	Male				0.00110	1.11638	Female			
MAGED1	0.00032	2.14771	Female							0.00056	-0.86042	Male	0.00005	-0.96153	Male
MPZL1				0.00000	0.81241	Female									
POLA1				0.00066	0.99326	Female	0.00002	0.82817	Female						
UBE2A				0.00000	1.40334	Female	0.00000	0.89346	Female	0.00000	0.74638	Female	0.00000	0.85734	Female
P4HA1							0.00000	-1.44474	Male				0.04573	-0.58930	Male
PLBD2							0.01470	-0.82658	Male						
ZNF675							0.00246	-0.69228	Male						
SLPI													0.02518	1.15608	Female
THNSL1							0.01678	-1.60349	Male						
MSMP							0.00279	-1.62213	Male						
MYADML										0.00035	-1.51132	Male			
SLITRK2				0.00201	1.88331	Female									
COL7A1							0.00048	1.37071	Female						
USP9X				0.00000	1.21680	Female	0.00000	0.90991	Female	0.00000	0.81426	Female			
SMOX				0.00263	0.68067	Female									
RPS15AP10										0.01704	-1.16359	Male			
MUC3A													0.01928	1.00769	Female
LINC00997										0.00536	-1.25886	Male			
SPTBN1				0.00228	0.63294	Female									
SMIM14							0.01130	-1.25079	Male						
GPR125				0.00006	2.00039	Female									
CAMKV	0.00000	-0.60420	Male												
CXorf40B	0.02057	0.92177	Female				0.00000	1.03277	Female	0.00000	0.75536	Female			
SSX5							0.03649	1.91254	Female						
MRGPRX1							0.00652	-1.12480	Male						
SNORA48				0.02847	-1.14104	Male	0.03142	0.77303	Female						
MYC				0.00244	-0.78038	Male									
RHBDL2							0.00966	2.19219	Female						
TRMT2A							0.00050	-0.65699	Male						
LOC100287225													0.00018	-1.78606	Male
NDUFB4	0.00000	0.82165	Female												
PM20D2										0.00017	-1.27349	Male			
GYLTL1B	0.00000	1.17156	Female	0.00000	1.90924	Female									
DTWD1							0.00356	-0.62105	Male				0.00012	-0.72766	Male
FAM171A1										0.03158	1.13577	Female			
TET2							0.00045	0.60429	Female						
MYL9										0.00000	-1.99597	Male			
MCTS1	0.00231	0.71671	Female	0.00000	1.04909	Female				0.00000	0.82320	Female			
MUTYH													0.00389	0.81603	Female
SLCO2A1							0.00120	-1.56223	Male						
BIK										0.03440	-0.61107	Male			
PIK3R3										0.00223	0.79075	Female			
SNORA16A	0.00322	2.43059	Female				0.04359	-0.72853	Male						
ATP6V0D2							0.02413	-0.89768	Male	0.01107	0.94242	Female			
TSNARE1													0.01345	1.37899	Female
GNL3L	0.00667	0.89624	Female	0.00000	0.87342	Female	0.00000	1.01263	Female				0.00000	0.69863	Female
CNIH3				0.00552	2.23959	Female				0.00348	2.45466	Female			

RUNX1T1										0.00000	1.91501	Female			
RGS16							0.00355	0.81159	Female						
DDX3Y	0.00000	-9.41916	Male	0.00000	-7.70717	Male	0.00000	-8.05749	Male	0.00000	-9.67669	Male	0.00000	-7.93532	Male
WBSCR16				0.00009	1.29805	Female									
GRIP1							0.00001	-2.15350	Male						
FAM151B							0.00784	-1.29876	Male						
POU5F1										0.00000	1.73161	Female			
RPL13P5										0.00191	1.05233	Female			
AQP3													0.00026	0.70527	Female
LINC00458													0.00000	-2.39711	Male
IGBP1P1	0.04402	0.75835	Female	0.00093	0.66369	Female	0.00000	0.67523	Female						
STX2										0.01291	1.02103	Female			
GABRE	0.04804	3.45485	Female												
EEF1E1.uid-8163													0.00000	-0.59710	Male
NRARP				0.00833	2.72243	Female									
AGPAT2				0.00000	1.71098	Female				0.00000	1.30572	Female			
CKMT1B										0.04816	0.99578	Female			
PQLC2										0.00422	-0.69924	Male			
SLC8A2							0.00852	1.99921	Female						
MTMR10										0.00209	-0.78072	Male			
ANXA8.uid-14184										0.00909	-0.90628	Male	0.00000	1.58382	Female
KIAA1143										0.00000	-0.66393	Male			
GIGYF1													0.00026	0.60827	Female
FAM58A							0.00000	1.37234	Female	0.00213	0.71980	Female	0.00304	0.68663	Female
RAB11FIP2							0.00602	0.58316	Female						
ST6GALNAC1	0.00110	2.59941	Female												
TTPAL										0.01334	-0.63070	Male			
MYO1D	0.01207	2.75591	Female												
TRIB3							0.03811	-0.73604	Male						
CBLB										0.00195	-0.80440	Male			
GCNT3										0.00000	2.65862	Female			
ZBTB16										0.02601	-0.92989	Male	0.01104	3.78587	Female
POGLUT1				0.00004	2.51098	Female									
ENTPD1										0.00133	-0.58811	Male			
GJC1							0.00042	-0.80830	Male	0.00275	-0.66442	Male			
RFFL							0.00025	-1.70582	Male						
ASAP2				0.02120	0.91998	Female									
CLEC10A							0.02105	2.96350	Female						
FCER2							0.02703	1.13505	Female						
LINC00669							0.02543	-1.01936	Male						
ABCE1													0.00000	-0.58356	Male
NMU													0.00003	-1.38216	Male
PDXP													0.01283	0.68849	Female
ALS2CR11										0.04521	1.21952	Female			
POLQ										0.00919	0.74110	Female			
CASC5										0.00000	5.89009	Female			
APOA1	0.00000	-1.07822	Male				0.00000	-4.35021	Male						
RAB25				0.00045	1.13477	Female	0.00000	-1.09731	Male						
PRSS23							0.00557	-0.61204	Male	0.00000	1.02456	Female			
TMEM132B							0.03429	1.65476	Female						
CD59													0.00000	0.71580	Female

EIF2S2	0.00004	-0.69450	Male												
RND1													0.00109	-2.01253	Male
NBR2	0.02158	1.67283	Female												
KRT10							0.00000	-0.80141	Male	0.00000	-0.80406	Male			
CD37										0.04505	0.98294	Female			
CCM2							0.03698	0.60113	Female						
OPN3										0.04145	-1.04140	Male	0.03320	-1.01566	Male
SPC25	0.00012	1.51398	Female												
CTNS	0.01439	1.30318	Female												
CRIP2							0.02330	-1.56949	Male						
DPH6							0.00532	-0.72038	Male				0.01392	-0.61541	Male
CCDC117	0.00930	-0.87110	Male				0.00000	-0.59198	Male						
MAN1A1							0.01337	-1.13159	Male				0.00970	-1.09737	Male
C2orf74							0.03093	-0.67466	Male						
YWHAE	0.00000	-0.73397	Male												
SNORD69										0.00003	1.19686	Female			
EDAR													0.04172	-3.28575	Male
LINC01012				0.00013	-2.51466	Male									
CRIPAK	0.02831	3.24271	Female												
PDXK				0.01747	0.65561	Female									
SP100				0.00542	-1.31214	Male	0.00614	1.66309	Female						
ZNF428							0.00514	0.76530	Female	0.01110	0.67840	Female			
STEAP1	0.00019	3.32622	Female				0.00137	-1.26819	Male						
ZNF800							0.00399	-0.67326	Male						
CA4										0.01719	2.44553	Female			
CLEC4D										0.00000	2.84470	Female			
NLRP13										0.00001	-2.94440	Male	0.00000	-3.79474	Male
ESYT1													0.00169	0.69585	Female
MCMDC2							0.00754	-1.72778	Male						
SVIL													0.01732	0.65938	Female
C4BPB							0.00198	-1.14304	Male				0.01345	-0.87563	Male
COMT				0.04834	-1.01311	Male									
TRIM52				0.02391	-1.43338	Male									
ZSCAN18										0.03120	-1.06700	Male	0.03002	1.02509	Female
ABCA3							0.03305	-1.56635	Male						
RIC8B										0.02805	-0.77918	Male			
SLC27A1													0.01080	1.49740	Female
MFGE8													0.00143	1.36949	Female
GRB7										0.01745	0.68536	Female			
LOC101928372													0.00650	1.06612	Female
FBLN1				0.00034	-1.36260	Male							0.02793	0.58527	Female
HECTD3				0.02638	0.62225	Female									
ACPP	0.00000	2.51003	Female												
GSPT2	0.00001	1.99087	Female				0.00278	0.64050	Female				0.00003	-0.78157	Male
FAM154A							0.02885	1.89795	Female				0.00997	-2.17117	Male
MT1H										0.00622	1.52061	Female	0.00003	2.22852	Female
ORAI1										0.02580	0.60556	Female			
DDX51							0.00033	0.74267	Female						
ITFG1													0.00003	-0.76491	Male
RNF219													0.00003	-0.61416	Male
USP22							0.00000	0.77216	Female						

MSX2P1							0.00000	-1.33319	Male	0.00001	-1.18162	Male			
TBC1D9													0.01630	-1.28567	Male
SCARNA9										0.00583	1.33594	Female			
RNF115							0.00015	-0.61877	Male						
CBR1	0.04508	1.35415	Female	0.00909	1.01781	Female	0.01712	0.64898	Female						
DNPH1				0.00878	0.85519	Female									
THG1L										0.02940	0.59414	Female			
DSTNP2										0.00001	0.72603	Female			
KIAA1211										0.01132	-1.22493	Male	0.04124	-1.00808	Male
FOXP4				0.01933	0.96805	Female									
SMIM20													0.00014	-0.66028	Male
HIVEP2													0.01121	1.53392	Female
PSORS1C1										0.00359	1.83447	Female			
AGPS				0.02448	0.60500	Female				0.00000	-0.70401	Male			
UBE2E1							0.00194	-0.58532	Male	0.00000	-1.31741	Male			
WNT10B	0.04860	-0.87678	Male												
TSHZ3							0.04040	2.13385	Female	0.04542	2.04725	Female			
TMPRSS11E										0.00733	1.05040	Female			
SNORD38A				0.01058	-1.47581	Male									
ATP8B2										0.01964	1.24276	Female			
VTRNA1-3													0.00000	-1.85142	Male
ERVV-2										0.00587	-1.18354	Male			
METTL21A							0.00009	-0.90561	Male						
MRPL11	0.00064	0.65380	Female												
TPPP							0.02359	1.31942	Female	0.01607	1.33931	Female	0.03272	1.17795	Female
LGALS12										0.00014	2.41142	Female			
PERP	0.00105	-1.18405	Male												
ENPEP				0.00002	-1.80293	Male	0.00628	-0.73454	Male				0.01504	-0.61647	Male
STAP2										0.00259	1.77896	Female	0.00087	-1.95852	Male
BRWD3				0.00001	1.22776	Female	0.00000	1.39046	Female	0.00000	1.30171	Female			
SLC25A35													0.00203	0.59488	Female
SDHAP3							0.01172	0.71695	Female	0.00001	1.13004	Female			
GHITM	0.00001	-0.78390	Male												
CXorf65	0.03186	2.11383	Female	0.00122	1.46443	Female	0.04577	0.68363	Female						
ADAMTS2				0.01255	2.75564	Female	0.04453	1.61190	Female	0.00956	1.91270	Female			
ECI1							0.00014	-0.67794	Male						
SBF2-AS1													0.00102	0.95735	Female
FAM49B							0.00000	-0.59935	Male						
ZNF749										0.01708	0.62775	Female			
SCN8A													0.02708	0.98844	Female
FBN1							0.03709	1.87020	Female						
NLRP9										0.00000	2.58257	Female			
ADAMTS1							0.00074	-1.48653	Male	0.00741	-1.15899	Male	0.02860	-0.95244	Male
CTXN1							0.04040	1.10483	Female				0.00650	1.34222	Female
GDF9							0.00001	0.87135	Female						
RTN3							0.00000	-0.62287	Male						
BOLL													0.00540	-2.02353	Male
AIM1L							0.02720	-0.65170	Male						
MT1X										0.00000	1.80154	Female	0.01243	0.80538	Female
COMMD7				0.04635	0.59093	Female									
ZNF449							0.02246	1.06422	Female						

AHRR							0.00436	1.90502	Female						
NPB													0.00544	-1.80527	Male
NEK4							0.04835	-0.59266	Male						
ZBTB44				0.04441	0.59226	Female									
HDAC9										0.01507	1.79734	Female			
MARCH1													0.00461	-1.33910	Male
CLUHP3				0.00427	-2.23911	Male									
ETFB							0.00034	0.61286	Female						
TMEM150A				0.00002	1.60099	Female									
SIGIRR				0.01188	0.81353	Female									
SNORA24				0.00946	-1.14344	Male									
ATP6V0A4							0.04130	-0.59087	Male						
CACNA1B							0.00001	2.44579	Female				0.00000	2.43227	Female
KLF9										0.00116	-1.17052	Male			
TMEM123							0.00000	-1.71510	Male				0.00000	-1.02885	Male
TERC										0.00137	1.35256	Female	0.01272	1.06247	Female
AIDA													0.00011	-0.68692	Male
ALOX15													0.00665	2.06184	Female
HAUS5													0.00297	0.66585	Female
LGR4													0.00307	-0.61224	Male
IFI16				0.03436	1.74731	Female				0.00010	1.93137	Female			
SLC22A20										0.01183	1.28972	Female			
CCDC144CP							0.00103	0.96403	Female						
HADH				0.04198	0.70887	Female									
KCNN4										0.01284	-0.89998	Male			
OOSP2													0.00000	-3.36799	Male
PDCD4				0.00492	-3.08086	Male				0.00000	4.63040	Female			
EMD				0.00000	0.92597	Female	0.00000	0.79043	Female						
MAP7D2										0.00091	1.15355	Female	0.00016	1.25760	Female
TSPAN4										0.03154	-0.66109	Male			
PLCD3				0.02043	0.84698	Female									
TPST1							0.01335	-0.84040	Male						
IRF3	0.00005	-1.39851	Male												
PRKCD				0.00153	1.33420	Female									
CCDC152													0.00320	-1.42826	Male
HSPB3				0.02752	-2.42787	Male									
HSD17B11	0.00955	-1.68211	Male												
LURAP1L										0.02885	-1.57400	Male			
PROX2													0.02418	-1.39978	Male
UBR3													0.00829	-0.58161	Male
RGMA				0.00159	1.63415	Female									
PLXDC1										0.01275	-0.88785	Male			
ZIC5													0.02495	-1.20136	Male
NPPA							0.00003	-1.90112	Male						
METTL4				0.02672	-0.65303	Male									
TMEM141				0.00058	1.16588	Female									
ADCY5							0.00427	1.81063	Female						
ZNF286B							0.00706	0.62968	Female	0.00160	-0.64736	Male			
RASSF7	0.01725	1.72501	Female				0.00004	-0.90708	Male						
MVD							0.00161	-0.62940	Male						
CHRNA5										0.00013	-1.27573	Male			

MRPL57	0.00129	0.62372	Female												
ACTBL2										0.00000	-2.75950	Male			
INSL4							0.04179	1.54466	Female						
LZTS1										0.02969	1.82529	Female			
ZMAT5										0.00011	-0.63558	Male			
RNF5P1.uid-10791										0.00021	-0.66744	Male			
MOV10													0.00022	0.77407	Female
MIR6869							0.00000	0.64559	Female						
DLX3				0.00010	-1.70653	Male									
NBEAL2				0.00549	1.49462	Female	0.00211	-1.10983	Male						
FOXO4													0.04485	0.72191	Female
MCPH1				0.02385	-0.59210	Male									
PDE4D							0.03187	0.88163	Female						
SNHG1	0.02562	0.60237	Female												
LINC00888				0.03628	1.13145	Female									
HIST1H2BC							0.00187	1.21502	Female	0.00000	2.23780	Female			
SERPINH1	0.00000	-2.18232	Male							0.00000	2.05446	Female	0.02319	-1.06067	Male
VCX	0.03059	2.09321	Female	0.00000	6.54785	Female	0.00000	4.13820	Female	0.00000	4.13908	Female	0.00000	3.35299	Female
NUMA1							0.00415	-0.59042	Male						
SLC25A24	0.00000	-3.67632	Male				0.00000	-1.68033	Male	0.02624	-0.82219	Male			
RAB39A										0.00029	-1.39612	Male			
FUT3													0.00014	2.19456	Female
ATPAF1				0.03652	0.89806	Female				0.01042	0.68047	Female			
FOLR1				0.00000	2.19677	Female									
HLA-A							0.02060	-1.57269	Male						
B3GALT6										0.01138	-0.90883	Male			
RBP7										0.00001	1.73755	Female			
PABPN1L							0.00090	2.43310	Female						
COX20				0.00000	1.28040	Female									
SNORD56													0.03485	0.60770	Female
TLE2										0.02191	1.68555	Female			
POLG2	0.00416	1.60097	Female												
ZIM3							0.00925	-1.64532	Male						
MAL2							0.00068	-1.63535	Male						
ULK2										0.04932	0.79362	Female			
SNORD34				0.01898	-1.11930	Male									
TAF5													0.01787	-0.58655	Male
PRDM16				0.01618	3.64570	Female	0.00000	4.63525	Female						
SERPINE1				0.00213	1.98240	Female	0.00000	2.03960	Female	0.03402	0.97904	Female			
MAP2K2							0.00008	0.63178	Female						
CLPS													0.01055	1.52868	Female
KIAA1430										0.01017	0.95584	Female	0.00377	-1.05652	Male
LOC401127							0.00000	-0.59034	Male						
CD3EAP										0.00000	0.62110	Female			
PUS7L										0.00527	-0.64252	Male	0.00193	-0.68897	Male
SEPT3													0.00765	2.00382	Female
BSCL2.uid-15607							0.00060	0.69916	Female						
PKP2	0.00022	-1.83658	Male	0.03947	0.70432	Female									
AQP11										0.00468	1.14543	Female			
RAB43										0.00015	1.31784	Female			
SLC25A40							0.00038	-1.26322	Male						

SVIP							0.00000	-0.98810	Male						
ICA1				0.00771	2.32574	Female	0.00001	-2.54569	Male				0.00664	-1.56906	Male
ERMN							0.02654	-2.00140	Male	0.03036	-1.88293	Male	0.00343	2.50158	Female
TRIM14										0.01480	1.12808	Female			
SH2D3A	0.04355	1.67424	Female												
MCTS2P	0.00902	0.94603	Female												
SREK1IP1				0.00093	-0.62974	Male									
TMEM220							0.01241	-1.32870	Male						
FAM46B				0.01069	1.06716	Female	0.03875	0.60764	Female	0.00000	1.52383	Female	0.00530	0.73444	Female
LDOC1				0.00113	1.23700	Female	0.00185	0.82362	Female				0.00004	0.97442	Female
ERCC1													0.00096	0.66435	Female
LOC100335030													0.01562	-0.72741	Male
GDI1				0.00002	1.03869	Female							0.00002	0.68573	Female
ANXA3							0.00000	-0.87708	Male						
DLX2										0.02050	1.93028	Female			
GCM1										0.00870	-0.86482	Male			
KIF16B										0.01234	-0.64430	Male			
FYN										0.00544	1.31016	Female			
PTGFRN										0.00032	1.15776	Female	0.00000	1.52909	Female
POU5F1P3										0.00000	1.69331	Female			
LOC643441										0.00627	-1.27942	Male			
PAGE4										0.00568	-1.70745	Male	0.00070	-1.96380	Male
MFAP5										0.01547	1.06999	Female			
TMEM88	0.00074	-3.25554	Male				0.00235	1.39915	Female						
ATP7B	0.00251	-2.08883	Male												
DIAPH2				0.00000	2.47273	Female	0.00063	1.33664	Female	0.00003	1.51499	Female			
POU4F1				0.04436	1.56666	Female	0.01071	1.30161	Female						
WNT11	0.00006	-2.74840	Male				0.00626	0.88410	Female				0.00003	1.20507	Female
UPF3B	0.00003	1.18744	Female	0.00002	0.76420	Female	0.00000	0.74975	Female						
DSP	0.01392	-0.91382	Male												
ALG5													0.00000	-0.83661	Male
TAF1A							0.00282	-0.77413	Male						
ZNF780A	0.01374	2.12443	Female												
SLC4A5										0.00300	-0.68825	Male			
TDGF1				0.00001	1.56705	Female				0.00000	1.93119	Female			
PPM1N							0.00004	0.84017	Female						
ZNF22										0.00776	0.68200	Female			
SLC46A1	0.04026	2.22457	Female												
FBXL20										0.00002	0.76628	Female			
INE1							0.00070	2.23918	Female						
AFF2										0.03273	2.16776	Female			
MZT2A										0.00003	-0.75375	Male			
LOC100129148													0.00722	0.62526	Female
PAGE2B	0.00832	1.99583	Female				0.00000	2.16843	Female	0.00000	1.90778	Female			
BEX4	0.02948	1.33672	Female												
HSPH1				0.00703	0.64885	Female									
ACSBG1													0.02592	2.01490	Female
NRSN2				0.00577	1.41262	Female									
RUNX1				0.01377	1.13233	Female									
MRFAP1	0.00001	0.69778	Female												
ZNF367				0.00409	1.98663	Female									

RHOBTB2										0.01208	-0.84940	Male			
APH1B							0.00155	-2.12709	Male						
PNLDC1										0.01074	1.33418	Female			
CSMD3				0.04765	-0.95080	Male	0.04609	-0.70075	Male	0.00023	-1.10031	Male			
MAP1LC3C				0.01457	2.98828	Female				0.00031	2.83106	Female			
GNRH2							0.00002	3.75390	Female						
NBEAP1							0.02479	-1.59415	Male						
JUN													0.00002	2.69327	Female
MRM1													0.01733	0.67166	Female
ANKHD1.uid-6876							0.00131	1.02350	Female						
NID1	0.02531	-1.26529	Male												
CUL4B				0.00874	0.71192	Female									
MBNL2							0.00004	-2.17745	Male						
LOC729083							0.02868	-1.77076	Male						
NR1D1							0.01245	1.32376	Female						
SLC28A3							0.01290	-0.58025	Male				0.00079	0.70319	Female
TMEM135							0.00080	-0.80424	Male						
PPAPDC3				0.02046	2.88491	Female									
HN1	0.00005	-0.69888	Male												
MAOA				0.00204	2.63472	Female	0.00697	1.67659	Female						
CLDN11				0.00000	-1.09934	Male									
COPS7A				0.00465	-0.75657	Male									
TFEB				0.01201	1.25532	Female				0.04275	-0.68309	Male			
TMEM87B										0.02829	0.62217	Female			
PAIP2B										0.00208	-0.91243	Male	0.00179	0.91245	Female
LINC00086										0.00449	2.45825	Female	0.04996	1.78792	Female
PRSS8	0.00002	2.71387	Female	0.00013	1.28702	Female									
F3	0.04324	-1.43313	Male										0.00001	1.05239	Female
BACE1							0.02618	0.81527	Female						
SNORD19													0.01254	1.21405	Female
TFAP4				0.00013	1.88162	Female	0.00000	1.68181	Female						
PHKA2-AS1							0.00036	1.70973	Female	0.00000	2.73932	Female			
CELA3B										0.02162	1.14105	Female			
HNRNPM							0.00000	0.59862	Female						
FCHO2										0.02344	-0.66698	Male			
PPP2R2D				0.01332	0.79414	Female									
LPPR3										0.00701	-1.59155	Male			
ZNF713										0.03841	-1.57694	Male			
ZNF587B							0.00004	0.82161	Female						
ERO1L							0.00154	-0.83506	Male				0.00106	-0.79622	Male
SYT6													0.02055	2.75920	Female
BMP15													0.00197	5.02944	Female
BHMT2										0.00242	-2.13845	Male			
CD9										0.00000	1.13415	Female			
ESRP2	0.00397	-1.47791	Male												
GPR78										0.03955	-1.29066	Male			
ARSI										0.03056	1.86127	Female			
HOPX										0.04515	-1.25026	Male			
ZNF318										0.00608	0.63707	Female			
MYH13				0.00161	-2.89807	Male	0.01779	-1.66764	Male						
SNORD89													0.04949	0.90213	Female

GPHA2										0.01635	1.76648	Female	0.00017	-2.64868	Male
RYR1													0.00158	1.01093	Female
NCOA7				0.01413	0.95838	Female									
TERT													0.00614	-1.08634	Male
CALD1	0.00071	-1.89680	Male				0.00033	-0.81593	Male	0.00540	-0.62300	Male			
GSTO1	0.00120	-1.79330	Male										0.00000	-1.09551	Male
SLC35A3	0.00984	-1.59104	Male												
KIAA1217	0.04136	-1.55067	Male												
CGN				0.01188	1.04569	Female									
AKT3							0.04076	-0.58222	Male						
DDX55							0.00000	0.62045	Female						
1.00000							0.00000	0.74002	Female						
ACOT11										0.00100	1.47001	Female			
BAZ2B.uid-2643				0.03859	-2.16731	Male				0.02642	-1.52430	Male			
LOC441666													0.00000	-2.26516	Male
FOXP1							0.02622	-0.75906	Male	0.00509	0.88093	Female			
MGC32805										0.00108	1.35976	Female			
LAMP2				0.00008	0.79571	Female	0.00000	0.97197	Female	0.00000	0.78427	Female			
DCAF4													0.00000	0.70831	Female
COL21A1				0.00291	-1.80413	Male									
WNT1													0.00034	2.95530	Female
KLF15										0.00010	-0.86744	Male			
GALNT18										0.01712	1.83713	Female			
MEI4													0.04185	-2.07625	Male
LARP7							0.00000	0.82024	Female	0.00000	0.72614	Female			
RIN2				0.00664	1.21261	Female	0.03187	0.68138	Female						
RGL2							0.00000	-1.16530	Male	0.00900	-0.64884	Male			
NEDD4							0.00767	-1.22287	Male	0.02898	-0.99025	Male			
SPA17							0.00074	-0.80128	Male				0.01202	-0.58275	Male
SYBU				0.00007	-2.29729	Male									
PGBD1										0.00237	1.26197	Female			
QSOX1													0.00041	0.92402	Female
SPATA6L													0.02390	-1.60969	Male
KIF18A							0.00487	-0.67417	Male						
SERPINB9				0.00001	1.66928	Female									
ASXL3										0.04595	-1.88611	Male			
FOXO3										0.02612	0.63906	Female			
OGFRL1										0.00957	1.20249	Female			
HMG20B							0.00143	-0.58365	Male						
TATDN1							0.00008	-0.88969	Male						
CLMP										0.01745	-1.33927	Male			
GSTA2										0.03012	2.38477	Female			
MSN				0.00298	1.60620	Female	0.00013	1.36740	Female	0.03486	0.80633	Female			
GK				0.01663	1.60287	Female	0.00003	1.73644	Female	0.00000	2.12479	Female			
DPRXP4										0.00746	-1.29412	Male			
GSTT1										0.01972	-0.79217	Male	0.00126	1.05788	Female
MAN2C1	0.02275	1.97726	Female												
CDH4													0.00079	2.08044	Female
SOHLH2													0.00008	-1.30601	Male
EFNB2				0.01757	1.40254	Female	0.03524	0.85824	Female	0.00195	1.12863	Female			
DDIT4				0.00000	-2.18939	Male							0.01911	0.75352	Female

TUBB3	0.01821	-1.46033	Male				0.00001	1.03678	Female	0.00000	1.27454	Female			
ANKRD30BL										0.02978	0.63616	Female			
HSPA1L							0.03162	-1.89568	Male	0.00000	-3.95501	Male			
CDO1										0.03529	-0.94230	Male			
SLC5A5										0.00000	1.27139	Female			
FAM132A										0.00228	1.91297	Female			
LONRF1							0.03570	-0.88256	Male						
JTB							0.00000	-0.63258	Male						
SUPT4H1				0.00000	-0.58052	Male									
NME7										0.00493	-0.91376	Male			
SLC25A43										0.00286	0.92146	Female			
CTCFL													0.03067	-1.60132	Male
NDUFB2-AS1													0.01580	0.67297	Female
SNORD76	0.02524	1.54619	Female												
RFPL4A										0.01603	4.55433	Female			
LUCAT1													0.00043	1.64696	Female
DDX53													0.00622	2.98975	Female
TNFSF12				0.03507	1.59490	Female									
PI4K2A							0.01283	0.67544	Female						
MGC27345	0.00184	-2.03623	Male												
WDSUB1	0.00329	-1.68392	Male												
TBX3				0.00160	0.86623	Female									
RAB3IP							0.00001	-0.74617	Male						
WDR24							0.03226	-0.68726	Male						
EMC3-AS1										0.00161	-0.61413	Male			
ATP2A1	0.04868	2.57303	Female												
COL4A2				0.01361	1.91611	Female	0.01213	-1.46947	Male	0.01365	1.37010	Female			
C6orf52													0.03069	0.85504	Female
RAB29							0.04030	0.74737	Female						
GRAMD2	0.00297	-2.07813	Male	0.00001	1.87122	Female	0.04275	-0.65962	Male	0.00037	-0.99055	Male			
NFATC2										0.02577	-1.35093	Male			
PCDH15							0.00127	-1.89377	Male	0.01623	1.42001	Female			
LOC389906				0.01676	1.10692	Female	0.00000	1.49478	Female	0.00555	0.81448	Female	0.00000	1.42997	Female
IFITM1				0.01387	1.24478	Female				0.00000	2.27292	Female			
NET1										0.00009	-0.64108	Male			
ADM	0.04208	-1.20100	Male												
S1PR2				0.02066	-1.27923	Male				0.00199	-1.03710	Male			
LTBP3							0.00098	-1.09783	Male						
SLC9A1										0.00023	0.60259	Female			
TRPT1							0.00009	-0.95060	Male						
FN3K										0.00000	2.35470	Female			
PCSK5													0.01033	0.95862	Female
ZNF578							0.01757	0.69854	Female						
NUTM1	0.01814	3.00937	Female												
MEST							0.00000	-0.75163	Male				0.00003	-0.58628	Male
PNP							0.00037	0.76366	Female						
RFNG													0.01843	0.62823	Female
TBC1D14							0.00532	-0.83734	Male						
ZNF625							0.02583	-0.98194	Male						
C15orf61				0.00000	1.08799	Female									
CORO1B							0.00144	-0.59980	Male						

KIFC1										0.00001	-0.86985	Male			
GPX2										0.00000	2.37714	Female			
PCOLCE2							0.04436	-1.47104	Male						
LINC01160	0.00120	-2.44050	Male	0.01500	1.23750	Female				0.00110	1.04040	Female	0.00096	-1.02838	Male
GNA12										0.01570	-0.65098	Male			
ISCA2	0.00037	1.14243	Female												
PSME1							0.03598	-0.62932	Male						
GAS2L1										0.04773	0.70487	Female			
FAM110B	0.00000	4.35376	Female												
SRSF2							0.00000	-0.67800	Male						
NSA2	0.00506	-0.63286	Male												
PLOD3							0.00360	-0.70111	Male						
B3GNT1				0.03810	-0.90061	Male									
SCCPDH							0.02078	-1.02418	Male	0.00085	-1.31674	Male			
APRT				0.00006	0.59275	Female									
ACTN1				0.00037	0.79336	Female									
RNPEP				0.00421	1.02643	Female									
TMEM191C							0.00000	-1.20079	Male	0.00510	-0.66145	Male			
VPS9D1							0.00380	1.04301	Female	0.03370	0.79114	Female			
ETV3										0.00000	1.60720	Female			
TULP1										0.03237	-1.42660	Male	0.03592	1.38616	Female
UCP2													0.00383	0.79347	Female
DZANK1										0.01806	1.04033	Female			
IL4R										0.01677	1.42762	Female			
PROSER1				0.01748	0.58450	Female	0.00004	-0.61969	Male						
HS6ST1				0.01933	0.83096	Female	0.00287	0.68136	Female	0.00000	1.06546	Female	0.00390	0.63880	Female
PANK1							0.00127	-1.28610	Male						
ARHGAP11A							0.00001	-0.64279	Male				0.00002	-0.58067	Male
PTPLA							0.00203	-1.43392	Male						
TCL1A													0.00000	-2.90740	Male
CCDC90B							0.00010	-0.63889	Male						
PTPMT1	0.00007	1.54948	Female												
BAIAP2							0.00017	-0.62892	Male						
RBM3	0.00000	0.89402	Female	0.00000	0.82676	Female	0.00000	0.59626	Female	0.00000	0.69020	Female			
PHPT1				0.00000	0.74698	Female	0.00000	0.75461	Female				0.00000	0.60628	Female
CSNK1E													0.00014	0.58424	Female
ZFP57	0.00037	-2.18484	Male												
C19orf70	0.01750	0.69301	Female												
OXT										0.00000	1.76863	Female	0.00011	0.93218	Female
MIR373							0.00008	0.96442	Female						
PYY2										0.00239	1.06742	Female			
PEBP1	0.04026	-0.64328	Male												
ZNF717										0.02001	-0.83231	Male			
PVRL1				0.01058	0.92916	Female	0.00254	0.71421	Female				0.00000	1.08780	Female
JUP				0.00000	1.24520	Female	0.00017	-0.65362	Male						
LANCL2				0.00194	1.09467	Female									
IL2RB							0.01783	1.56693	Female						
SENP5	0.02323	0.67664	Female												
YWHAEP1							0.00520	-0.88994	Male						
WIPI1							0.02363	-1.13657	Male						
ABCA13										0.02937	1.02246	Female	0.02987	-1.01557	Male

PIGP							0.00120	-0.92345	Male				0.02292	-0.64774	Male
TCEAL4										0.02721	0.94404	Female	0.00403	-1.15906	Male
NETO2													0.01809	-0.74221	Male
SUMF2							0.00074	-0.67940	Male						
TFDP3	0.00052	4.62559	Female	0.00000	4.71916	Female									
CD320				0.00000	1.51032	Female	0.00000	0.79204	Female						
PFKL				0.00000	1.01774	Female	0.00000	-0.66429	Male						
FAM175A										0.00612	-0.59634	Male	0.00000	-0.94223	Male
ZSCAN4										0.00000	5.62373	Female			
C11orf1	0.01969	2.23518	Female												
TBL1X				0.00531	1.17205	Female	0.00027	1.00750	Female				0.00408	0.77612	Female
PRKXP1							0.04084	-0.89059	Male						
RBM15	0.00713	-1.19243	Male	0.00671	-0.74368	Male									
DCAF4L1				0.03451	-0.87209	Male									
PECAM1							0.02934	1.65411	Female						
RRP7B							0.00000	0.78508	Female						
FAM86EP										0.00066	1.37485	Female			
NSMCE4A							0.00000	-0.77024	Male						
MRI1										0.00469	-0.70670	Male			
SLC6A3										0.00003	2.36658	Female	0.00017	2.16628	Female
KLF12													0.02527	-1.57523	Male
TKTL1				0.01490	1.50066	Female	0.00000	1.86782	Female	0.00000	3.48605	Female	0.00268	1.14649	Female
MTERFD1													0.00009	-0.64427	Male
SNORA9													0.03788	0.59396	Female
ZNRD1-AS1													0.00895	-1.66630	Male
CHKA										0.00721	-0.88649	Male			
SLC25A10							0.02470	-0.70859	Male						
SGK494							0.03429	0.64192	Female						
TRIML1				0.04525	-1.11616	Male									
KDM6A							0.00275	0.64423	Female	0.00000	1.08773	Female	0.00000	1.16022	Female
SSX1				0.00392	2.86658	Female									
PML							0.01610	-0.86170	Male						
CAPNS1				0.04397	0.58529	Female				0.00019	0.62212	Female			
TEAD2							0.00007	1.06041	Female						
LOC101927501	0.04208	2.88881	Female	0.00000	3.59877	Female	0.00000	3.14790	Female						
UNC5B	0.00004	2.91642	Female							0.00002	2.23962	Female			
NACAP1	0.00183	-0.70836	Male												
PTK7							0.02096	-0.80156	Male						
GNE										0.00000	-2.40283	Male			
YAP1										0.00000	-0.76251	Male			
CDIP1							0.04688	0.82182	Female						
HDC													0.00541	-2.59301	Male
GSK3B										0.00029	-0.64743	Male			
LMO2										0.00001	-2.70688	Male			
TBL2							0.00365	-0.73614	Male						
MUC15										0.00727	-1.12218	Male			
CTTN							0.00000	0.82427	Female						
HSPE1				0.00000	0.95875	Female									
TSPO										0.00005	-1.73411	Male			
IMPACT							0.00051	-0.76825	Male				0.00001	-0.88830	Male
C1GALT1							0.00007	-0.74266	Male						

TMX4				0.00217	1.82621	Female									
ZFYVE20	0.01885	-1.23303	Male												
PPP1R9B							0.00797	1.08518	Female						
RASD1										0.00000	2.28732	Female			
ABHD14B							0.00051	-1.03875	Male						
FUNDC2	0.02338	0.64937	Female	0.00001	0.66787	Female	0.00000	0.80060	Female						
DLX6										0.00909	-0.84754	Male	0.00625	-0.86354	Male
FOXA2										0.03158	1.18161	Female			
SLC43A3													0.00106	-1.83242	Male
ERCC2							0.00040	-0.99755	Male						
SPTB													0.00000	1.59070	Female
SLCO3A1							0.02038	1.89885	Female						
ALG14	0.00001	2.45127	Female												
SLC9A7							0.03573	1.21818	Female	0.00860	-1.40622	Male			
LOC100130075													0.01648	0.73843	Female
PLRG1	0.02742	-0.62760	Male												
MAPK12										0.00909	1.96370	Female			
1.00000										0.02995	1.25112	Female			
TANK							0.00201	-0.64923	Male						
CPNE8	0.02023	-2.39407	Male												
UTY	0.00000	-6.03357	Male	0.00000	-7.39858	Male	0.00000	-6.61215	Male	0.00000	-6.81694	Male	0.00000	-7.02608	Male
KATNBL1P6				0.01401	-0.87444	Male	0.00207	-0.71205	Male						
ZNF649				0.04487	1.04919	Female									
GREB1							0.00156	-2.08740	Male						
LRGUK							0.03496	-2.16723	Male				0.03461	2.06515	Female
LINC00665							0.00281	0.76122	Female	0.00066	-0.79634	Male			
COMTD1	0.00181	2.07670	Female												
MECP2				0.00001	1.22860	Female	0.00000	1.27923	Female						
HSPBAP1							0.00001	-1.16714	Male						
HOOK1							0.00852	-0.88609	Male						
CHST4													0.00867	-2.20504	Male
FBXO41													0.00019	1.55737	Female
AGPAT5										0.00055	-0.64643	Male	0.00016	-0.68619	Male
PAQR3	0.00093	2.03938	Female										0.00325	-0.67381	Male
ERICH1	0.01793	0.80432	Female												
HDAC6	0.01118	1.81244	Female												
CNBD2							0.02429	-1.75708	Male						
AGER													0.00085	1.09801	Female
EIF2B5				0.03585	0.76065	Female									
H2AFY2										0.00083	1.54408	Female			
DHX32													0.01959	-0.66214	Male
BCKDK				0.00006	1.38532	Female									
ANKRD33B				0.00000	1.62935	Female							0.00616	0.68889	Female
ENGASE				0.02431	1.97659	Female									
FAM3C													0.00001	-0.67638	Male
LOC101927027	0.01426	-2.37809	Male												
CDR2L				0.01863	0.98070	Female	0.00527	0.75547	Female				0.00432	0.73039	Female
PLXNB1							0.00000	-1.99598	Male	0.02525	-0.75063	Male			
NEK3										0.01062	0.91625	Female			
CACNG8							0.00004	3.28541	Female						
PDGFRA										0.00000	2.26425	Female			

C16orf13				0.00110	0.68738	Female									
CXorf56	0.00231	0.92038	Female	0.00002	0.75220	Female	0.00000	1.03998	Female	0.00000	0.79820	Female	0.00000	0.81735	Female
TEC										0.00233	1.24546	Female			
SNAR-G2							0.00081	0.88937	Female	0.00174	0.82654	Female			
GRM4										0.00285	2.01632	Female	0.02443	1.55866	Female
POTEE							0.02703	1.05219	Female				0.00776	1.12956	Female
ARID3A										0.00000	-0.87383	Male			
KHDC3L										0.00008	1.84105	Female			
C1orf131	0.00264	-0.85617	Male												
SFXN5										0.01628	0.68758	Female	0.00732	0.74301	Female
REPS1	0.03312	-0.96759	Male												
PTENP1							0.00000	-1.07651	Male						
TSPAN1				0.00004	3.01803	Female							0.00544	1.44046	Female
ITGAM										0.00176	1.76084	Female			
DSCC1							0.00012	-0.62571	Male						
MIXL1										0.00075	1.54964	Female	0.00000	-2.00224	Male
LAMC2				0.00099	2.22496	Female									
RPPH1							0.00000	1.51070	Female						
CDC14A	0.03849	2.33813	Female												
ZC2HC1C													0.01320	1.34108	Female
DDAH1				0.02311	2.34875	Female									
CHI3L2	0.00169	-1.80209	Male							0.00008	0.91998	Female	0.01246	0.62002	Female
GHDC							0.01203	-1.56293	Male						
ERRFI1				0.03431	-0.67849	Male									
HLA-DRB1										0.00000	-2.92991	Male			
RNF10	0.02831	-0.65012	Male												
SNTB2										0.00027	-0.69750	Male			
FOXO3B										0.00065	-1.17103	Male			
PHTF1							0.00235	-0.86002	Male	0.00906	-0.71359	Male			
UBALD1				0.00792	0.96238	Female									
LYPLAL1													0.00219	-0.58322	Male
FGFR2				0.00006	1.52132	Female									
SORT1				0.00000	1.80142	Female									
BAG2										0.00000	-1.74154	Male	0.00000	-1.71801	Male
BZRAP1-AS1										0.03012	-1.04593	Male	0.00016	1.60836	Female
PPP1R14B	0.00094	-1.22374	Male												
PHF8										0.00055	0.68521	Female			
C5orf51							0.00000	-0.82055	Male	0.00000	-0.76684	Male			
UBQLN2							0.00166	0.76689	Female						
PLEKHA5										0.00046	0.71463	Female			
SYDE2				0.00000	3.41512	Female									
SLC25A22				0.00000	2.24070	Female									
EPN1				0.00846	0.88208	Female									
LLGL2	0.02453	-0.93804	Male										0.00000	0.72078	Female
SLC2A11				0.04673	-1.05316	Male							0.02371	0.73888	Female
LOC101928259							0.03586	1.31679	Female						
LRBA				0.04505	-0.79895	Male									
CASC4							0.00039	-1.08461	Male						
CD24				0.00045	1.35062	Female	0.00009	-0.97824	Male	0.00001	-1.01100	Male			
PPIC							0.00050	-0.67756	Male						
PPP1R15A													0.00002	0.73525	Female

AFMID				0.01514	-0.67770	Male									
ANKLE1										0.00912	-1.10977	Male			
CRYZ													0.00014	-0.83613	Male
ADCK1	0.00056	-2.42060	Male												
FAM131A							0.03965	-1.23628	Male						
STAG3													0.00000	1.14501	Female
SERAC1				0.00831	0.99880	Female									
CAV1	0.00330	1.94240	Female	0.00394	-2.40086	Male	0.00764	-1.70848	Male				0.00089	-1.89598	Male
CHAC1				0.02448	-1.41494	Male	0.00443	1.18156	Female	0.00282	1.17288	Female			
MRPL15	0.00652	0.60234	Female												
LINC00926										0.03710	-2.09112	Male			
MIB1													0.02638	-0.60951	Male
ST3GAL2							0.00032	0.80012	Female						
LEPR										0.01721	-1.22863	Male			
IGF2BP1	0.00094	-0.62781	Male												
GLDC				0.00840	0.84666	Female									
NR2F6				0.01279	0.73836	Female									
METTL23							0.00239	-0.87009	Male						
NRP1										0.00021	-1.71493	Male			
MECOM										0.00000	2.16827	Female			
DHODH										0.00984	0.62084	Female			
GLS2													0.02127	1.71438	Female
ABHD5							0.00045	-0.59153	Male						
PI4KAP1							0.00015	0.75262	Female						
THRSP													0.01449	1.66047	Female
TSR3	0.00678	-1.62186	Male												
EXOC6							0.00126	-0.91727	Male						
RAB30-AS1													0.00228	0.59813	Female
CSK							0.00196	-0.59370	Male						
MTSS1L										0.00124	-1.68521	Male			
TEX37				0.04848	-1.30848	Male	0.00170	1.31131	Female						
ANGPT4													0.03770	2.84185	Female
PLEKHA2													0.00004	2.59894	Female
TCERG1L				0.01840	1.36093	Female				0.00382	1.01939	Female			
KLHL22										0.01110	-1.21360	Male			
CYB5R2							0.02229	-0.89780	Male				0.02410	-0.82284	Male
KRT19	0.00051	-1.93054	Male												
LINC01091							0.02250	-1.16557	Male						
LOC113230													0.00489	2.34202	Female
SNORD79	0.04434	1.08099	Female												
MORC2-AS1							0.00074	0.79461	Female						
GLCE							0.00403	-0.89733	Male						
ZBTB25	0.02841	2.47100	Female												
TBC1D16										0.04890	1.06358	Female			
ZNF93							0.00499	-0.58945	Male						
BTF3P11										0.00018	-1.44780	Male			
ANKS6													0.02715	1.07614	Female
ENTPD1-AS1													0.03756	-1.07820	Male
NKD2				0.00649	1.01437	Female									
LCMT2										0.02417	-0.77984	Male			
MYCL										0.00000	1.48776	Female			

AMMECR1	0.00395	1.83305	Female	0.01654	0.91538	Female									
LMNB1				0.00405	-0.68403	Male				0.00000	-0.81594	Male			
PRRT2										0.00175	-0.64519	Male			
AKNA										0.04349	1.15980	Female			
LRRC45							0.00480	-1.06690	Male						
IPMK	0.02921	-1.40996	Male												
CLDN15										0.02098	-0.94688	Male			
LOC100129935										0.00003	-2.10395	Male	0.04418	1.18359	Female
KPNA7							0.00037	2.93562	Female						
LZTS2							0.00083	-1.28074	Male						
LINC00974				0.01150	-2.46589	Male	0.04823	1.57947	Female						
UBE2J1							0.02035	-0.69689	Male	0.00059	-0.88874	Male			
ZNF214							0.04380	-1.23650	Male						
BUB1B				0.00224	-1.01749	Male	0.00030	-0.80636	Male	0.00000	2.21089	Female			
ZNF814							0.00000	0.65144	Female						
TLN2										0.01245	0.65350	Female			
DGAT1													0.04555	0.67426	Female
CPB2-AS1													0.00581	2.09276	Female
MRPL3	0.00003	-0.79278	Male												
SNORD116-26										0.01645	1.03805	Female			
ABCG2				0.00001	1.75523	Female									
PRR7				0.03870	2.17174	Female									
FUNDC1	0.00042	1.14693	Female	0.00010	0.77021	Female	0.00001	0.58960	Female	0.00000	1.13317	Female			
PORCN				0.00866	1.77000	Female				0.00000	2.04665	Female			
RABL6				0.00033	1.19023	Female									
MRPS11	0.00000	-1.14426	Male												
GNA11							0.00000	0.95753	Female						
LRP8							0.00034	-1.56818	Male						
TBC1D23										0.00002	0.59000	Female			
HPDL	0.00000	2.74396	Female												
GUCA1B													0.02905	1.43431	Female
DPH1	0.01713	1.94778	Female				0.01919	-0.77147	Male						
CREBBP							0.00020	0.60773	Female	0.00011	0.60099	Female			
TRIM43B										0.01857	4.27432	Female			
RAB33B				0.00146	-1.09897	Male									
LINC00847										0.04821	-1.98458	Male			
FAHD1				0.00609	1.01515	Female							0.00336	0.68741	Female
PPP2CA							0.00000	-0.68237	Male						
ZNF826P													0.00142	0.65851	Female
SMG1P7										0.00010	1.20441	Female			
ARSB							0.02375	-0.97462	Male						
EBLN1							0.03268	3.05477	Female						
SLC19A2							0.01438	-0.67386	Male	0.00064	-0.84098	Male	0.00262	-0.74634	Male
CORO1A										0.00000	1.77142	Female			
LINC00339										0.01838	0.91620	Female			
LYZ										0.00000	2.99910	Female			
ATP1B1										0.00000	1.49558	Female			
NOTCH1													0.00650	1.59701	Female
ECHDC1							0.00083	-0.73346	Male	0.00000	-1.46478	Male	0.00001	-0.85410	Male
ZNF669							0.04573	-0.81170	Male						
SLC35A4										0.00000	-0.72113	Male			

KIF12										0.00008	2.31752	Female			
LRSAM1										0.04106	-0.65659	Male			
H3F3B							0.00000	0.63400	Female						
ANKRD36B							0.00006	1.27830	Female	0.00527	0.89069	Female			
EEF1A1							0.00000	-0.68029	Male						
ZNF687							0.00000	-1.74665	Male						
WDR81										0.03568	-1.06330	Male			
TPI1P2							0.00000	0.59399	Female						
FBXO28	0.01002	-0.92994	Male												
SMARCA1							0.02112	-1.84922	Male						
VWA8										0.02163	0.68157	Female			
TCIRG1	0.04508	-1.68826	Male												
SMCO4										0.02094	0.96039	Female			
RBMX2				0.00002	0.75087	Female	0.00000	0.73784	Female						
SNORD45A													0.02252	-0.81773	Male
ATG4A										0.01438	0.64788	Female			
CIDECP													0.00270	0.67195	Female

Supplementary Table S4. NMF cluster size and number of genes per cluster

	Cluster size			
	Mouse		Human	
Cluster label	Male	Female	Male	Female
1	447	1181	3487	2038
2	1833	416	3086	2145
3	1349	569	2611	5002
4	435	245	2119	2861
5	465	908	978	2744
6	586	1487	1385	4993
7	1966	1862	825	2058
8	1752	1220	2023	
9	937	1586	1324	
10	707	853	965	
11	853	577	1720	
12	1218	1691	1255	
13	325	240		
14	350	1386		
15	680			
16	318			

Supplementary Table S5

Mouse male NMF coefficient matrix

Gene	Cluster 1	Cluster 2	Cluster 3	Cluster 4	Cluster 5	Cluster 6	Cluster 7	Cluster 8	Cluster 9	Cluster 10	Cluster 11	Cluster 12	Cluster 13	Cluster 14	Cluster 15	Cluster 16
A4galt	0.05379	0.01966	0.05667	0.21749	0.01045	0.00610	0.02970	0.06273	0.19832	0.04475	0.24274	0.03775	0.03734	0.05182	0.40484	0.02199
A630020A06	0.09850	0.01843	0.06304	2.74110	0.00763	0.07640	0.01772	0.08494	2.75599	0.26921	0.21385	0.21966	0.05599	0.43859	0.65147	0.00644
AA388235	0.16935	0.00778	0.14572	0.63476	0.00940	0.04807	0.12110	0.07019	2.05481	0.44345	0.66872	0.12241	0.03931	1.90506	0.40670	0.02843
AA414768	0.00000	3.00398	2.02521	2.02529	2.52654	0.00640	1.39723	0.77336	7.87031	1.68702	0.00000	0.00000	1.20441	1.48466	1.06174	0.10957
AA415398	0.01279	0.00702	0.08796	8.97458	0.00866	0.00363	1.43255	0.01035	12.44484	0.00416	0.00000	0.00000	0.00971	0.00006	0.55378	0.00472
AA465934	0.35790	0.00739	0.00596	0.17364	0.00724	0.00703	0.00732	0.01209	0.38163	0.01138	0.01032	0.00728	0.01233	0.00965	0.04522	0.00694
AA467197	24.27979	6.89574	17.26449	0.62084	22.23862	12.00025	5.53252	5.51120	0.31648	1.97989	5.75353	0.08175	11.93972	1.40480	0.00000	12.93772
AA536875	0.03902	0.00749	0.03265	0.18494	0.01842	0.01263	0.01746	0.02341	0.16376	0.05633	0.08698	0.02556	0.05706	0.12085	0.48176	0.00722
AA543186	0.04349	0.04297	0.03398	0.37634	0.01177	0.01245	0.02897	0.02096	0.29558	0.03661	0.05397	0.00720	0.02313	0.00595	0.41162	0.01498
AA543401	0.01762	0.00777	0.01151	2.27027	0.00903	0.00626	0.00738	0.01297	1.63480	0.01274	0.00000	0.00417	0.01314	0.00184	0.20515	0.00691
AA545190	0.01377	0.00802	0.01225	5.62067	0.00931	0.00612	0.00732	0.01312	0.00000	0.01100	0.04191	0.00750	0.01195	0.00110	0.04445	0.00643
AA619741	0.01287	0.00793	0.00826	4.95695	0.00892	0.00565	0.00721	0.00480	4.66247	0.49858	0.15285	0.00510	0.00968	0.00000	0.84013	0.00595
AA792892	0.10272	0.14143	0.08863	33.70022	0.07213	0.04674	0.13755	0.72089	32.05632	5.19036	2.11182	0.85954	0.62888	5.26636	10.54997	0.09265
AA986860	0.02363	0.00870	0.01226	0.01056	0.01052	0.00722	0.00784	0.05205	0.01234	0.01217	0.01417	0.00800	0.71073	0.01312	0.00965	0.00732
Aaas	11.76583	12.55330	11.36294	3.05769	1.25785	2.47491	9.99631	17.23082	2.37060	9.10665	10.55734	0.84675	8.14643	0.52815	3.88029	7.64411
Aacs	6.78200	2.48043	4.10657	0.08208	7.54879	5.69845	6.15111	20.38400	1.14080	19.52037	7.58921	1.18823	1.28134	1.45080	3.97512	1.43665
Aaed1	0.08127	0.00562	0.00742	0.49467	5.43853	0.00688	0.00765	0.00977	0.66148	0.34423	0.31265	0.00000	0.00880	0.00767	0.00000	0.00757
Aagab	0.79780	2.97048	4.47571	0.48469	0.06134	0.00567	3.14100	2.20499	0.00576	1.78431	0.71943	0.02373	3.25270	0.37879	2.79471	0.43115
Aak1	2.35932	2.80278	3.03106	3.39595	3.05530	0.47794	2.04898	4.68108	3.58525	2.10228	1.63367	3.29214	2.55738	4.17277	2.36056	1.76208
Aamdc	2.65090	0.32913	2.52480	2.28818	0.00697	0.01048	0.00367	0.83080	4.57962	0.49129	0.80285	0.26925	2.52857	0.04970	1.42038	0.00156
Aamp	17.78961	15.10377	19.39963	4.05193	14.59720	6.72508	12.20625	24.65539	6.37309	16.31169	9.86326	2.32375	17.29956	0.09991	5.31644	9.17935
Aar2	7.54515	3.27510	3.46123	0.51357	0.00000	5.67356	6.70124	8.42407	2.19597	6.47176	3.88263	5.37948	4.99933	1.89875	4.11474	1.81145
Aard	4.75909	0.01552	0.00294	0.68796	0.01209	0.00402	0.00400	0.00475	0.00000	0.07097	0.01144	0.00395	0.06756	0.00720	0.31329	0.09917
Aars	16.85861	12.01500	10.51237	9.61801	6.26564	6.74257	9.08956	21.83323	15.92448	13.11352	9.57695	1.31746	14.75469	0.20276	6.03418	10.42344
Aars2	1.69849	0.02671	0.00000	0.72110	0.27235	1.32455	0.00344	3.00012	0.53252	2.55572	1.96070	0.01043	3.24990	0.00172	0.00000	2.11660
Aarsd1	13.83233	6.67650	10.53162	0.58731	1.00303	5.08862	4.84262	15.50291	0.46286	6.06169	3.12563	0.86286	7.67817	0.00918	0.00000	5.16685
Aasdh	1.82797	4.77932	2.09110	3.53311	0.00800	0.00232	4.20168	2.40773	2.82273	0.41280	1.36369	4.73253	1.12935	0.06387	2.95462	3.60403
Aasdhppt	8.52806	6.26742	6.09947	3.71320	5.43677	0.01994	5.60822	2.88501	5.47491	4.83916	1.97978	1.73996	4.41444	1.32782	2.29850	0.57347
Aass	0.01370	0.00766	0.01152	0.45620	0.00920	0.00662	0.00739	0.01117	0.00461	0.19992	0.02438	0.00690	0.01225	0.01327	0.31816	0.00737
Aatf	12.51278	8.78759	6.01413	0.68977	0.28300	0.00000	5.22246	7.50887	0.48425	11.90783	2.10263	2.79250	6.70833	3.74575	10.29375	4.33178
AB124611	0.01367	0.00786	0.01236	0.09180	0.00933	0.00658	0.00750	0.01343	0.09793	0.01041	0.01216	0.00736	0.01260	0.00680	0.11504	0.01465
Abat	0.00203	0.00774	0.00000	0.20365	0.01880	3.71898	0.00710	1.74303	0.99180	2.23765	0.33123	0.10587	0.00000	1.47995	3.21259	0.00592
Abca1	0.02935	0.00720	0.52370	0.53377	2.17429	0.00552	0.86679	0.05114	0.00000	0.00000	0.00000	0.00000	0.00698	1.86891	0.95838	0.00667
Abca13	0.62158	0.00764	0.19793	2.62396	0.00620	0.00618	0.00731	0.05361	0.42529	0.04139	0.00581	0.00522	0.00920	0.31260	0.45304	0.00639
Abca16	0.02904	0.00792	0.58317	0.01088	0.00991	0.00564	0.00729	0.01206	0.01225	0.01469	0.01220	0.00758	0.00895	0.01476	0.00189	0.00717
Abca17	0.00386	0.06354	0.41441	0.93422	0.00797	0.36153	0.09312	0.99829	1.76316	1.09344	0.67599	0.03149	0.06908	0.15143	2.70846	0.23578
Abca2	0.71283	0.00620	0.08618	0.41316	0.00673	1.22457	0.69354	0.00000	0.16756	0.00000	0.70765	0.00143	1.22543	0.00385	0.08994	0.00534
Abca3	0.00000	1.70167	3.74872	0.00000	0.00000	7.01104	5.21856	5.13043	0.05828	0.99563	0.03972	0.00516	5.63500	0.02622	0.17155	6.15010
Abca4	0.00947	0.00781	0.01074	0.24013	0.00906	0.00622	0.00676	0.01013	1.09597	0.00000	0.00024	0.03347	0.95911	0.03848	0.21304	0.00659
Abca5	0.01662	0.00768	0.41177	0.00636	0.00851	0.00560	0.00721	0.01061	0.29281	0.00797	0.00974	0.03722	0.00939	0.03089	0.18058	0.00708
Abca7	2.34306	0.00469	0.24847	0.01052	0.00307	0.00647	0.45180	0.25002	0.01218	0.00753	0.01103	0.00492	0.11739	0.01127	0.01529	0.00463
Abca8b	0.01293	0.00812	0.02402	0.00470	0.00928	0.00767	0.00763	0.01727	0.32745	0.01109	0.00972	0.00767	0.01231	0.03743	0.02549	0.00741
Abcb10	3.61893	2.69055	2.22684	5.32652	0.00000	0.00265	2.42845	3.89925	1.11853	1.63052	2.94043	0.20253	0.10224	10.03091	2.88713	2.06213
Abcb11	0.01371	0.00782	0.01167	0.00765	0.00933	0.00666	0.00750	0.01262	0.01140	0.01819	0.01082	0.01757	0.01318	0.00571	0.52934	0.00740
Abcb1a	0.00637	0.00700	0.90984	0.11068	0.02052	0.00875	0.13797	3.63829	0.00718	0.89608	1.49844	0.95075	0.10361	0.00508	0.47729	0.02096
Abcb1b	0.00000	0.03069	0.52915	1.75300	0.00928	0.00581	1.34435	1.24392	0.00853	0.63106	1.31171	0.26485	0.62646	0.10313	0.42464	0.00642
Abcb4	0.00945	0.00768	0.03810	0.39621	0.00964	0.00782	0.06898	0.14920	0.00445	0.03542	0.01166	0.04854	0.02487	0.01743	0.15516	0.00723
Abcb5	0.00000	0.00780	0.43314	1.24771	0.01033	0.00580	0.00764	2.85791	0.36551	0.77154	0.07233	1.10666	0.52438	0.67386	3.45180	0.00708
Abcb6	3.51618	0.00676	12.54355	3.86648	1.12141	7.08725	7.65621	9.77976	0.59032	3.80803	6.23420	0.46704	7.52883	0.39110	1.88005	2.11744
Abcb7	2.80324	0.12922	2.87794	4.22401	3.37377	4.47316	1.95645	2.63551	2.74610	1.75661	3.72795	9.30909	3.50418	12.15987	4.27443	3.52941
Abcb8	6.20223	0.36403	3.68949	0.61015	0.04478	1.68214	1.81934	3.97901	0.00529	0.86397	0.28952	0.03147	6.88312	0.04845	0.00000	0.30864
Abcb9	0.01087	0.00778	0.00996	0.22250	0.00957	0.00663	0.00720	0.12894	0.64156	0.01259	0.00906	0.01636	0.00817	0.01232	0.01096	0.00734
Abcc1	3.65445	0.01285	1.56079	0.64115	0.00000	0.00239	2.66529	1.47876	0.08681	1.92949	0.46545	0.51784	1.32952	2.71836	0.51898	1.29174
Abcc10	1.34197	0.00309	0.20173	1.30268	3.76922	0.00459	0.00415	5.03366	0.76991	2.87048	2.25927	0.00000	1.99279	0.01572	0.00000	3.50741
Abcc2	0.01305	0.00791	0.01021	0.00000	0.00915	0.00626	0.00728	0.00777	0.00903	0.74406	0.08569	0.00227	0.01037	0.00536	1.60417	0.00722
Abcc3	2.78799	0.01619	0.00000	0.00720	0.00488	0.00460	0.00476	2.96982	0.11567	1.90807	1.23358	0.02800	0.00000	0.02009	1.41648	0.00442
Abcc4	0.04975	0.00803	0.37540	0.38068	0.00630	0.01839	0.22617	1.04343	0.00518	0.00000	0.00000	0.00000	4.74710	0.35052	0.86567	3.33150
Abcc5	4.77536	2.32178	4.04151	3.96447	3.20764	1.04418	2.78748	9.33453	5.98771	3.36969	2.95366	1.39315	2.36901	2.39210	4.98224	3.75669
Abcc6	0.01375	0.00799	0.01215	0.08936	0.00931	0.00664	0.00753	0.01377	0.09132	0.01458	0.01314	0.00794	0.01271	0.01371	0.01224	0.00740
Abcc8	0.95252	0.00661	1.41406	0.01099	0.00368	0.00461	0.00689	0.01000	0.01218	0.01359	0.01086	0.00728	0.00732	0.01597	0.00000	0.00579

**Abcc9**	0.14395	0.06049	0.21484	0.28044	0.12783	0.07237	0.04707	0.05237	0.26297	0.15427	1.15962	0.19970	0.56184	1.38977	0.29441	0.08577
**Abcd1**	2.73913	0.10766	2.73242	0.98420	3.69084	5.26637	0.01468	2.52182	3.13601	4.67325	0.83998	0.13054	0.00000	0.00000	1.06758	0.54477
**Abcd2**	0.11060	0.00815	0.10373	0.13679	0.02490	0.00658	0.01781	0.00487	0.15947	0.03274	0.77675	0.02395	0.07005	0.24224	0.08509	0.02176
**Abcd3**	10.29224	6.93527	3.48906	0.10844	0.13639	0.22679	7.00518	5.28403	0.87765	1.97464	4.09938	0.52882	3.38746	0.99773	1.18195	1.60686
**Abcd4**	3.68131	1.77862	10.38975	1.15358	0.18748	3.68381	4.78824	0.06568	1.40133	0.33111	2.25432	0.11570	3.24520	0.64524	0.15932	0.94718
**Abce1**	14.19414	14.24075	8.48476	4.37079	9.14636	6.46513	9.77576	14.40251	9.98061	12.39502	19.06948	14.03494	10.09232	28.44896	17.97990	2.49388
**Abcf1**	18.98990	9.59222	12.73694	17.96873	6.40865	6.60795	7.02967	18.14976	16.18189	25.30111	15.67232	2.89579	13.86934	1.84617	14.08086	8.86204
**Abcf2**	14.05251	12.86943	16.38724	19.96035	11.48750	12.26575	9.29846	26.98740	18.20800	22.99329	13.54681	7.73914	20.81167	0.47874	10.28797	8.83152
**Abcf3**	4.58444	10.07599	10.54552	4.12976	5.19891	0.94890	5.93834	13.05497	8.52380	8.79637	9.18933	1.10572	7.47535	2.24686	9.71152	5.19647
**Abcg1**	1.72390	0.00654	0.00000	0.08315	0.01265	0.01540	0.00686	0.23738	0.00887	0.00554	0.07127	0.02054	0.02087	0.00913	0.08815	0.00546
**Abcg2**	0.36471	0.00507	1.02916	0.20147	1.08212	0.00562	2.42354	0.59620	0.00421	0.00000	0.00767	0.00310	0.00000	0.01472	0.00000	3.63988
**Abcg4**	3.08049	0.00239	1.20219	0.01021	1.06602	0.00408	0.00306	2.43530	0.82905	1.16647	2.94343	2.27296	0.30702	0.00332	0.39465	0.00265
**Abhd1**	0.01335	0.00805	0.08266	0.01062	0.00918	0.00581	0.08033	0.01300	0.01235	0.01464	0.01284	0.00825	0.01774	0.01270	0.00920	0.00733
**Abhd10**	0.31194	0.15284	0.00000	0.78655	3.26423	1.87838	1.95472	0.22358	0.34066	1.62132	1.23823	0.32885	2.75099	0.88769	1.85441	2.64770
**Abhd11**	10.51729	5.74286	8.58478	1.59822	7.29705	6.25384	8.42481	13.98126	0.26799	6.15674	3.47870	0.58597	6.58230	0.15780	0.00000	6.01974
**Abhd11os**	0.64979	0.02829	0.66408	0.01575	0.04222	0.00513	0.01530	0.42577	0.01029	0.86844	5.13220	0.05838	0.00004	0.00417	0.00381	0.39953
**Abhd12**	3.02487	5.18357	5.63876	3.68759	0.04248	0.36716	5.84995	0.06699	3.30966	3.93455	0.03174	0.11791	3.35749	0.00000	0.11626	3.83052
**Abhd12b**	0.01215	0.00770	0.12212	0.01327	0.00849	0.00578	0.01736	0.00000	0.01042	2.31500	0.77347	0.05804	0.01063	0.00985	0.00000	0.00711
**Abhd13**	0.87909	0.00391	1.32793	6.69412	0.00553	4.37517	0.00244	2.00132	3.15128	1.56432	1.11440	9.28052	4.18910	4.90098	2.58512	0.44850
**Abhd14a**	2.37790	0.00475	0.04491	0.14403	0.02977	0.00657	0.00416	0.46432	0.00860	0.57966	0.00685	0.02112	2.28870	0.00478	0.81323	0.01861
**Abhd14b**	0.00806	0.00762	0.30471	1.05610	0.00679	0.00921	0.00719	0.00993	0.94241	2.19744	0.00408	0.01771	0.01832	0.03842	0.17481	3.05997
**Abhd15**	0.03463	0.12057	0.07508	0.28700	0.01942	0.01222	0.01723	0.02093	0.18987	0.05703	0.41997	0.00502	0.04175	0.03290	0.07825	0.01432
**Abhd16a**	0.00000	0.00564	3.43846	1.99056	0.07393	1.45317	2.76047	3.45885	0.32417	1.23896	1.08628	0.35564	1.41069	0.03051	0.01013	2.52902
**Abhd17a**	7.39541	0.25852	0.33491	0.01775	0.26568	0.15403	1.23602	2.52228	0.42728	15.65815	0.42593	1.02925	4.14842	0.02852	0.00000	2.36613
**Abhd17b**	0.02917	4.10771	0.60070	8.22382	0.55235	0.26805	0.00471	1.17283	5.22151	0.44559	0.78233	1.31096	0.76584	1.36334	1.45683	0.35751
**Abhd17c**	1.29629	1.45844	0.79270	4.61791	0.03926	4.08555	4.66146	3.52421	6.94023	1.78335	0.84772	0.06568	6.27380	0.68884	6.92689	0.21507
**Abhd18**	0.00000	0.94538	0.04892	1.45709	0.02558	0.15214	0.03751	0.02189	0.50227	0.09228	0.00000	1.53782	2.79302	1.03071	1.29416	0.19898
**Abhd2**	0.00821	0.00829	1.03561	1.57327	0.02673	0.00551	0.00719	0.31116	0.64389	0.00000	0.20881	0.02587	1.24515	0.00342	1.07754	0.00596
**Abhd3**	0.00176	0.00790	0.16163	0.18871	0.01139	0.00906	0.00690	1.64378	0.09277	0.25748	0.43728	0.00000	0.00292	2.71842	1.66807	0.00759
**Abhd4**	3.20950	3.99980	2.08437	4.27565	5.10279	6.74597	0.72511	0.66384	1.76665	2.00207	0.85596	0.08392	1.47325	1.44105	0.29629	3.16187
**Abhd5**	0.64757	0.00264	0.37340	2.56080	0.02119	1.71143	4.59892	1.66134	0.62218	1.21852	1.21609	6.92595	8.27162	0.01508	0.31347	1.99153
**Abhd6**	0.00000	0.02421	3.58160	15.85188	0.05310	0.00430	0.00000	16.09474	12.95614	12.51691	14.20173	2.06288	8.55208	14.21399	16.23278	0.28396
**Abhd8**	0.00875	0.00715	0.01734	0.07112	1.10596	0.00660	0.00705	0.02671	0.01030	0.67401	0.05074	0.02768	0.66858	0.01110	0.00704	0.98859
**Abi1**	6.36742	4.05519	2.41462	7.48935	3.20304	0.60812	8.62025	6.60977	8.11455	8.03490	4.21393	17.55593	4.27343	11.16391	5.48160	5.16880
**Abi2**	2.56009	0.00000	0.72775	2.14390	0.02869	0.01392	4.33107	0.09825	0.16522	1.11336	0.56004	8.57753	1.41254	1.68092	1.21931	0.03858
**Abi3bp**	0.01376	0.00806	0.01200	1.27519	0.00933	0.00652	0.00744	0.01255	0.00000	0.01112	0.02484	0.00789	0.01257	0.00439	0.27074	0.00717
**Abl1**	5.17716	1.35106	3.73165	6.91470	0.01028	0.31015	0.62255	5.89797	5.03746	6.23585	3.48110	0.85477	4.87671	0.51354	0.40089	6.52259
**Abl2**	5.47135	0.01215	1.09543	19.03354	0.28312	0.34186	0.08743	1.80097	10.00410	4.66220	1.56506	2.07717	1.63077	4.81713	14.83999	0.00376
**Ablim1**	0.00000	4.39643	1.33761	0.86000	0.01386	5.38401	2.34375	0.33189	0.79067	1.51827	0.04792	0.26176	1.67108	0.29364	1.73509	0.00210
**Ablim2**	0.01686	0.81590	0.00000	3.56438	0.00865	3.83870	0.00666	0.00508	0.00000	0.00000	0.19766	0.00680	0.00611	0.00027	0.05887	0.01221
**Ablim3**	0.01374	0.00816	0.01203	0.35105	0.00932	0.00663	0.00751	0.01368	0.17833	0.01408	0.01198	0.01856	0.01276	0.01149	0.00452	0.00735
**Abo**	0.01360	0.00808	0.01075	0.01555	0.00934	0.00652	0.00736	0.01139	0.01183	0.00772	0.01089	0.00713	0.01908	0.14463	0.36438	0.00731
**Abr**	10.41752	3.50748	1.90544	1.39925	0.73883	0.69748	4.84848	3.98849	0.41576	6.00049	1.73368	1.82144	1.81788	0.02504	1.50354	4.85986
**Abracl**	2.82712	0.00284	1.27401	0.00000	3.42311	0.00466	0.40411	0.00000	0.30471	2.11015	0.00000	0.13376	0.92008	1.95557	0.78143	0.40171
**Abt1**	3.27956	3.41761	1.17345	5.13274	3.86219	2.35604	3.01488	9.62470	8.96023	4.03020	1.07100	0.35154	0.05987	11.27288	21.36803	1.42260
**Abtb1**	5.32437	1.18399	0.72920	1.23498	1.88097	4.33749	3.83650	0.21348	1.73134	1.47118	0.00000	0.00000	1.51075	0.00265	0.00000	1.32943
**Abtb2**	0.41429	0.00909	0.00000	6.71543	0.01551	0.00585	0.00670	0.97131	5.64956	0.70568	0.43096	0.00000	1.23315	0.01927	0.01136	5.13529
**Acaa1a**	3.91819	0.70277	2.71420	0.56959	1.75979	3.80721	1.34985	0.00000	0.00000	1.05571	1.35901	0.00790	2.23226	0.00232	0.00000	3.40363
**Acaa1b**	1.70438	0.13879	0.96567	0.28639	0.72349	1.46615	0.73562	0.00667	0.00453	0.44650	0.21617	0.00174	0.73557	0.00631	0.01706	1.37864
**Acaa2**	13.05254	12.04217	14.35765	0.16507	9.34989	10.48408	4.63847	1.85655	0.00000	0.00000	2.43911	0.02704	4.97099	0.00000	0.01725	11.16812
**Acaca**	1.43167	4.49965	6.55104	2.08759	4.98835	0.01133	2.15375	6.65069	1.70152	3.28601	1.35973	0.09127	2.00621	0.00000	1.97532	1.65318
**Acacb**	4.13072	3.52472	0.66466	0.88618	0.00000	0.00162	0.81689	8.02166	0.93244	7.32850	3.43054	0.61507	1.80046	0.05636	0.27475	2.46023
**Acad10**	0.94883	1.14025	0.16153	0.02276	0.53321	0.80571	0.00551	1.01729	1.25747	0.04461	1.31965	0.02463	0.03645	0.00275	0.02657	2.20971
**Acad11**	6.41141	2.60610	3.91101	1.75384	9.76204	0.00362	0.10926	2.37226	1.15747	2.05994	0.54234	1.88753	1.48614	0.38503	4.31156	3.20971
**Acad12**	0.98790	3.37679	0.49066	0.00000	3.64326	0.61493	0.00477	0.81518	0.00933	0.38936	0.12436	0.00778	0.71767	0.00685	0.00381	1.49565
**Acad8**	8.19882	3.22073	12.20491	1.56417	3.23040	3.38022	5.63383	11.53581	7.10714	5.10392	6.46021	0.55666	8.84182	9.07870	8.57274	2.25271
**Acad9**	2.82177	4.97320	7.91444	0.32687	1.37630	0.05781	1.32021	7.70508	2.62461	3.45173	1.60872	1.12853	2.99964	3.98328	4.65168	0.28119
**Acadl**	18.12070	10.66908	19.74851	0.00000	12.82105	9.51675	11.06862	18.55855	0.84205	12.92547	21.27062	14.08147	16.02776	9.97651	6.20992	9.06393
**Acadm**	12.73706	13.61120	16.67383	3.46093	3.94790	16.10776	11.85104	23.58359	1.16021	23.29116	18.11234	13.95442	13.92948	25.55708	17.32557	5.37389
**Acads**	2.94318	0.16654	1.54399	0.12451	0.03086	1.65008	1.19446	4.38592	0.31932	8.48464	0.31127	0.23813	1.61519	0.00527	0.29034	0.88010
**Acadsb**	1.16034	0.00641	1.06766	2.90966	0.18254	0.05272	0.00659	0.53638	0.61976	1.30949	0.01400	0.02036	0.00000	0.10908	1.19328	0.50975
**Acadvl**	17.23505	7.01328	8.74358	0.66343	7.96942	10.09002	5.63640	8.44453	0.00000	0.79612	3.28983	0.26967	9.29839	0.00000	0.19348	9.57185

**Acan**	0.00790	0.00759	0.03334	0.01083	0.01906	0.00816	0.00714	0.35613	0.01194	0.00890	0.01062	0.02618	0.00603	0.01313	0.00658	0.00739
**Acap1**	0.01360	0.00804	0.01042	0.35609	0.00921	0.00662	0.00763	0.01340	0.00623	0.00000	0.21246	0.00674	0.00956	0.00572	0.16067	0.00721
**Acap2**	1.34247	0.86024	1.72025	1.83325	0.95713	0.00378	0.97751	0.06782	0.02264	2.07383	0.89834	5.20597	1.80761	0.04738	0.60636	0.00324
**Acap3**	1.38897	0.00508	0.19261	2.32442	0.01328	4.01345	2.79737	0.15549	3.52958	1.14937	0.99467	0.23923	4.31807	0.00000	0.00000	1.91338
**Acat1**	2.02588	0.32520	3.69943	3.13811	3.88983	0.20547	4.95201	4.25638	0.92313	3.13231	2.10979	0.35501	3.24914	1.89264	5.31609	0.55618
**Acat2**	7.66166	3.12442	12.28131	4.38357	6.51775	2.79742	2.01783	5.22762	4.66964	6.38368	3.80938	1.25622	2.17362	1.36134	4.52640	3.42968
**Acat3**	0.74503	0.63548	2.42552	0.42786	0.85330	0.01248	0.00643	0.82307	0.39327	0.42510	0.01848	0.02043	0.31299	0.03179	1.24667	0.00554
**Acbd3**	2.56543	1.80879	1.01643	6.33289	0.02834	2.87734	2.11284	5.16400	3.78241	8.02808	3.89562	1.73110	0.58840	1.03736	10.28725	0.05941
**Acbd4**	0.53906	0.01745	0.14732	0.00000	0.00632	5.20691	0.01516	1.90267	0.44472	3.06847	2.98871	0.11459	2.42916	0.00080	0.48756	0.01775
**Acbd5**	1.72229	0.17770	2.24122	8.77269	3.61549	1.49928	2.98794	4.43928	9.80182	8.01829	3.10146	3.00783	2.02594	15.22088	14.32168	0.02906
**Acbd6**	6.25578	0.08351	0.06753	3.43962	2.07499	0.81720	0.22381	2.80417	1.44052	9.24962	5.48694	0.12599	5.27422	0.48727	0.67348	2.25078
**Acbd7**	0.01316	0.00811	0.01563	1.01567	0.00937	0.00624	0.12981	0.01176	0.41900	0.05002	0.01008	0.00713	0.01207	0.00532	0.10487	0.00719
**Accs**	0.00000	0.07827	0.91666	0.00459	0.00850	0.00948	2.88417	0.01120	0.02248	0.00000	0.13023	0.01248	1.29367	0.00624	0.13613	0.00649
**Accsl**	0.01219	0.00774	0.00000	25.41079	0.00961	0.00295	0.00595	0.25497	7.68069	2.44960	0.69540	0.14271	0.00367	0.24825	12.10058	0.00262
**Acd**	13.68880	1.96958	4.77459	5.48663	0.16526	6.20317	2.53966	10.97383	3.22484	4.89918	6.51929	1.10221	3.98193	0.00000	0.70649	1.82688
**Ace**	0.17686	0.00827	0.00770	0.11684	0.01503	0.00698	0.00718	0.01045	0.01025	0.50728	0.01416	0.00610	0.06546	0.01078	0.00779	0.00782
**Ace2**	0.01375	0.00811	0.01229	0.28201	0.00931	0.00662	0.00752	0.01393	0.00745	0.01543	0.01405	0.00820	0.01266	0.01130	0.00676	0.00737
**Acer1**	0.05275	3.00959	0.08301	0.25224	0.04745	6.23823	0.01453	0.00000	2.10745	0.00000	1.04748	0.03281	1.38227	0.19498	1.05760	4.11863
**Acer2**	9.39850	3.78400	5.57902	3.12869	4.13858	2.68483	4.75762	10.20299	2.75592	12.02462	9.78349	2.89082	4.52151	9.75979	6.75990	0.53283
**Acer3**	0.21902	0.00752	0.89080	0.35256	0.00594	1.05668	0.70566	0.27983	0.69561	0.82649	0.17375	0.29429	2.08947	4.33803	0.00000	0.00549
**Acin1**	8.41513	2.31468	2.11447	0.00000	3.01614	2.47253	1.34700	4.39955	0.15377	9.56375	0.07091	0.24044	2.73358	0.83186	2.69526	2.87193
**Ackr4**	0.01064	0.02827	0.69253	0.00652	0.00890	0.00944	0.00736	0.01181	0.01143	0.32853	0.01199	0.00444	0.16005	0.02003	0.51143	0.00754
**Acly**	12.85243	12.77281	8.06673	7.02804	10.11504	5.33233	14.64602	18.55121	9.21698	7.03627	7.32478	8.10509	6.61147	6.49863	9.42878	6.78071
**Acmsd**	0.04614	0.01881	0.10454	0.49506	0.04455	0.00758	0.00707	0.03473	0.55045	0.01688	0.38635	0.12578	0.03983	0.44106	0.26397	0.02159
**Acnat1**	0.01623	0.03959	0.07851	0.36881	0.00850	0.01383	0.11923	0.07626	0.38343	0.18235	0.02670	0.00000	0.02911	1.14817	2.20604	0.04974
**Aco1**	13.00357	12.58974	3.17746	0.68180	10.73485	4.32400	10.36703	4.79942	1.24431	2.35950	0.55488	0.16121	2.20031	0.27602	1.36398	3.83790
**Aco2**	19.55584	9.03164	18.33211	2.66854	10.23969	10.37295	12.78846	22.13698	2.14329	9.56300	9.06777	1.85714	16.09629	0.63342	2.89459	9.42121
**Acot1**	0.00726	0.00734	0.00304	0.12242	1.82984	0.00849	0.00817	0.22174	0.95398	0.86381	0.13075	0.00217	0.00684	0.01005	0.02903	0.00751
**Acot10**	0.82336	1.13490	1.27662	0.03274	0.84966	0.76800	1.21174	1.05032	0.54336	0.37593	1.55388	1.96028	0.68217	1.17914	0.60060	0.19980
**Acot11**	0.68045	0.00654	0.39282	0.71830	0.36187	0.79551	1.97040	1.80506	0.52742	0.49867	0.74047	0.25489	0.31298	0.94508	1.01351	0.00594
**Acot13**	3.22943	2.36146	3.16952	5.67286	0.00000	0.11277	1.94955	1.43127	1.17227	3.38185	6.91681	0.49125	1.94705	1.28499	0.75468	0.03436
**Acot2**	0.04119	0.04170	0.10511	0.20480	0.15802	0.15558	0.00756	0.06074	0.09741	0.11362	0.01174	0.00751	0.54770	0.04646	0.02069	0.00719
**Acot3**	0.01428	0.00822	0.00773	1.65374	0.00938	0.16008	0.00741	0.01310	0.00000	0.04299	0.01297	0.00813	0.01051	0.00969	0.00296	0.00709
**Acot5**	0.01444	0.00797	0.00693	0.43422	0.00931	1.66550	0.00719	0.01088	0.00330	0.20417	0.00978	0.00732	0.00465	0.01015	0.00624	0.00674
**Acot7**	3.93266	4.35985	1.31841	5.08870	0.01341	3.06350	0.91574	7.51199	5.08447	9.52414	7.44016	0.38290	8.72182	0.15415	1.12515	3.67637
**Acot8**	6.64406	2.58172	6.27422	1.41020	2.38969	1.80528	4.38281	2.95285	3.52133	3.45701	2.12901	0.66744	4.55632	0.00000	0.00000	5.09406
**Acot9**	6.72412	7.90791	8.50102	0.48531	6.83382	5.00788	7.42853	9.05838	3.55045	3.96687	5.94936	10.58016	6.34147	7.22778	4.52813	1.79413
**Acox1**	5.34435	4.60158	5.90728	8.66759	5.02147	0.33769	12.06354	5.07102	9.34466	4.04632	6.36989	0.15494	2.18109	0.03442	3.70253	2.53618
**Acox3**	0.01928	0.00817	0.05869	1.78200	0.01033	0.01171	0.00719	0.00948	0.41455	0.35417	0.00000	0.03099	0.01183	1.38845	1.71813	0.00696
**Acoxl**	0.05211	0.00863	0.00911	0.47664	0.01405	0.00670	0.00659	0.01392	0.00519	1.95942	0.05004	0.02361	1.37064	0.01048	0.00016	0.00679
**Acp1**	20.43530	15.69776	15.59543	0.00000	10.72578	9.31945	12.23898	11.72499	0.64893	11.62783	5.87790	2.04280	15.19464	5.67862	6.32253	9.15730
**Acp2**	0.15028	0.07607	1.52826	0.57770	0.00500	0.06193	2.89587	0.83772	1.21632	0.07682	0.79652	0.02088	0.48481	0.07805	0.50372	1.54289
**Acp5**	9.50448	1.81392	9.46454	0.13130	1.86934	3.30782	2.85878	7.47135	0.01240	3.72549	2.87371	0.07041	11.24637	0.05411	0.08088	3.83691
**Acp6**	9.97060	0.00000	0.00000	1.14160	0.19227	4.97394	0.95903	3.34667	0.01401	0.79817	0.07742	0.07454	0.00000	0.01085	0.11429	3.40926
**Acp7**	0.82963	0.01700	0.00436	0.44348	0.00615	0.00684	0.00643	0.19018	0.03450	0.20813	0.14894	0.00615	0.00615	0.01030	0.04358	0.00610
**Acpp**	0.11868	0.00481	1.90490	1.30241	1.17226	0.00541	2.56102	4.85356	2.85978	5.14601	1.91208	0.72955	0.86073	6.36860	7.35437	1.61984
**Acrbp**	0.06759	0.00697	2.95955	1.10335	0.07346	0.00683	1.52532	0.00000	0.00000	2.07704	0.73159	0.11313	0.05774	0.23940	0.55333	0.00602
**Acsbg1**	9.15865	2.88082	3.13899	0.24529	2.19770	1.72361	3.17818	3.30657	0.45276	0.00037	0.22310	0.18156	0.64687	0.01885	0.22963	0.52002
**Acsbg2**	0.00000	0.00717	0.00000	0.38251	0.01841	0.00611	0.00630	2.97681	0.76598	2.23076	3.19452	0.92207	0.00000	2.41561	1.93158	0.00646
**Acsf2**	0.91655	0.22171	2.05804	0.00230	0.00272	1.45630	1.03696	0.83656	0.04596	1.86392	1.81840	0.08777	1.14150	0.75125	0.15604	2.44486
**Acsf3**	0.87881	0.23566	0.13805	0.00427	0.00720	0.00494	0.00533	1.81787	0.00929	0.00000	0.85762	0.01022	1.97142	0.00837	0.00432	0.00528
**Acsl1**	1.79289	0.01553	0.15005	4.17363	0.00482	1.51318	3.20790	0.37890	3.83836	2.79062	0.04220	0.03074	0.00006	0.00000	2.52607	0.00416
**Acsl3**	2.75512	7.67519	4.79860	5.66482	6.42566	2.28616	8.61721	6.06456	14.72616	7.75768	5.83036	0.21383	0.60770	17.63918	11.45287	2.82777
**Acsl4**	2.55454	1.16497	6.00740	10.01118	6.90066	0.00259	5.70368	12.67482	20.52038	17.30293	4.43378	19.04475	5.20533	35.16666	20.35349	0.02969
**Acsl5**	0.21302	1.79428	0.00000	0.00792	5.11363	0.00685	0.00631	0.00347	0.01021	0.00216	0.01245	0.00221	0.00782	0.01317	0.02742	0.95044
**Acsl6**	0.04623	0.00796	0.00000	0.00178	0.00845	0.16333	0.00624	0.00000	0.00911	0.20950	1.51031	0.05181	0.88530	0.00381	0.96119	0.01388
**Acsm2**	0.02049	0.00809	0.02781	0.11933	0.00861	0.03667	0.00763	0.01796	0.01383	0.04373	0.04702	0.02976	0.02507	0.06593	0.06997	0.00728
**Acsm3**	0.00000	0.01709	0.21091	0.00957	0.95540	2.16956	0.00563	0.72468	0.01113	0.00000	0.00360	0.03321	3.03317	0.04531	0.00000	0.07455
**Acss1**	3.07121	2.08450	1.58973	0.23982	0.00987	0.03219	2.07626	2.02364	0.00426	0.00000	0.59422	0.03749	2.17519	0.46850	0.99862	0.34674
**Acss2**	3.40523	1.82755	0.16288	0.30904	1.76162	2.80315	0.00325	2.94210	1.92032	0.80597	0.78343	0.02422	0.75270	0.00000	2.06873	0.00915
**Acta1**	1.10638	0.34434	1.18109	0.00195	0.99041	1.25664	0.72354	1.43707	0.07946	0.22506	0.01093	0.06020	1.60998	0.01171	0.01504	0.81948
**Acta2**	0.91338	0.57514	1.40292	0.00000	1.10456	1.12198	0.70766	1.09247	0.00850	4.18179	0.00000	0.86807	1.53157	0.05863	0.00000	0.67151

**Actb**	29.40271	7.66673	36.29374	1.10668	19.03271	13.84283	10.57997	13.93549	4.53701	14.21363	30.95542	2.93441	26.20889	21.09907	6.83150	20.77288
**Actg1**	55.14451	31.13930	54.74761	6.43094	42.60748	32.05160	32.58648	55.13984	3.19502	33.16637	41.11180	22.16416	59.27234	14.80574	9.65471	31.98801
**Actg2**	0.01289	0.00770	0.00824	0.00501	0.00878	0.00663	0.00711	0.00951	0.01049	1.81441	0.00763	0.33212	0.01051	0.00777	0.26495	0.00727
**Actl6a**	10.42297	7.05199	8.73270	5.51080	12.55033	5.18371	6.36289	14.06001	8.51772	9.28190	7.47482	2.57165	11.52346	14.91024	11.36707	8.07743
**Actn1**	3.77084	0.22784	0.51497	4.11852	3.91360	0.01606	0.98016	0.71492	1.25501	0.04802	6.02694	0.45182	0.94261	0.29918	0.00000	0.53756
**Actn2**	6.41131	4.97357	11.50719	2.32738	0.02924	4.62344	0.29137	22.67674	4.86927	16.72450	24.47068	2.59780	11.89708	1.56761	4.57195	0.66463
**Actn4**	11.40651	11.14681	15.63472	1.29215	1.82438	16.63877	14.16468	34.00691	1.11931	17.44595	4.07854	3.10455	23.84683	0.00000	0.57147	5.95391
**Actr10**	5.19431	10.81763	8.12804	5.28910	9.20403	0.73719	3.74245	11.66160	9.65011	9.17008	12.78999	1.32607	11.24258	5.20839	6.75801	4.91742
**Actr1a**	9.06135	0.26645	5.65015	10.96668	3.15325	7.36652	4.03006	12.34063	5.27443	12.25194	6.41793	2.65094	5.49826	1.03933	4.00005	5.07036
**Actr1b**	5.50093	4.49868	3.86001	2.23390	10.51397	4.14858	5.13555	5.69990	1.65375	2.29991	3.61186	0.59181	7.19009	0.34973	2.93647	3.94864
**Actr2**	8.62336	4.26273	7.43394	4.85487	6.15951	2.49240	6.20278	5.63684	5.04890	4.65894	7.52782	9.35408	6.37538	11.24485	8.52608	1.98426
**Actr3**	17.25851	16.05499	18.93555	6.90331	16.95021	13.33937	13.59513	11.10887	6.88391	11.05317	6.57631	11.61955	19.99518	15.89234	11.82042	9.51403
**Actr3b**	0.58250	0.00686	0.44226	0.56009	0.00614	0.00738	0.00662	0.17746	0.00290	1.39673	1.15690	0.00688	0.25715	0.00294	0.08562	1.27373
**Actr5**	0.78136	0.01613	0.00625	3.94776	0.97832	0.00604	0.01472	7.23277	9.83515	13.04764	3.65836	0.35710	2.08038	0.12857	0.14997	0.71270
**Actr6**	3.50352	1.44060	2.31143	2.03340	0.00998	1.12354	2.38420	3.37341	8.01935	0.00000	0.55224	0.95525	1.17470	15.75952	8.78104	0.01414
**Actr8**	6.66089	4.07716	5.27574	0.36266	0.03214	0.00000	1.52713	10.07059	0.98309	7.60582	4.35914	2.30680	2.59420	2.00773	5.69731	4.38041
**Acvr1**	0.25838	1.95153	1.11048	0.16560	0.01551	0.00562	0.02711	0.90400	0.42047	0.61751	1.00105	1.09738	0.00000	0.47035	0.29331	1.26264
**Acvr1b**	0.00723	0.10224	0.01056	0.14373	0.00830	0.01292	0.02772	0.01100	0.00692	0.00539	0.01236	0.01816	0.37207	0.01153	0.00591	0.50156
**Acvr2a**	0.17943	0.01649	0.45454	1.75799	0.01054	1.34999	2.33593	1.65530	1.19516	2.88119	1.17135	3.65561	2.05793	5.78340	1.02627	0.00488
**Acvr2b**	1.58794	0.00548	1.02359	0.29601	0.01066	3.99727	0.05414	0.07072	0.89766	0.66633	0.81132	0.01524	4.28512	0.18868	0.60192	1.77307
**Acvrl1**	0.01360	0.00793	0.01203	0.70271	0.00922	0.00676	0.00754	0.01322	0.00000	0.18662	0.01135	0.00785	0.01295	0.00450	0.26968	0.00730
**Acy1**	0.71335	3.35182	0.48891	0.20262	0.00619	0.11750	0.00532	2.18072	0.00730	0.07532	1.75227	0.03347	0.00000	0.03672	1.48532	0.34235
**Acy3**	0.97450	0.00634	0.69864	5.69407	0.00462	0.00709	0.00386	0.74755	6.70445	0.00000	1.13278	0.00000	3.23885	0.00000	0.06416	0.00299
**Acyp1**	1.10169	0.04701	0.36816	2.26453	3.22491	0.00423	0.01599	0.27053	0.47485	0.91896	2.14857	0.29345	0.00000	0.03487	1.01607	1.11705
**Acyp2**	0.08716	0.00642	0.18961	0.00000	2.10084	0.00785	0.25362	0.04684	0.28782	0.02365	0.33621	0.04117	0.04493	0.13038	0.45549	0.01443
**Ada**	0.02903	0.00748	0.96355	1.32061	0.01096	0.00640	0.00678	1.86791	0.26749	0.00000	0.54190	0.00855	0.00000	0.01726	0.00000	0.01480
**Adad2**	0.00000	0.00800	0.39501	0.00725	0.07935	0.00972	0.05112	0.77618	0.02097	0.37443	0.09080	0.00552	2.14228	0.01544	0.00000	0.54037
**Adal**	0.00000	1.43093	1.12990	0.19223	0.03289	0.00675	3.01167	0.82762	0.08359	1.87507	1.58861	0.19118	0.79915	2.17398	3.63177	0.02704
**Adam10**	2.51346	1.42486	3.13518	6.41708	0.73049	0.02343	4.58893	1.78701	4.60000	5.01741	1.83382	1.29254	3.82303	17.33883	6.83237	1.35715
**Adam12**	0.01353	0.00824	0.01165	0.58989	0.00927	0.00653	0.00751	0.01365	1.54355	0.00855	0.00000	0.01698	0.01316	0.01190	0.00268	0.00722
**Adam15**	0.44830	0.00572	0.27495	0.02096	0.05593	0.07757	1.29321	1.13413	0.37377	1.03627	1.83212	0.01728	3.32469	0.01296	0.00000	3.70616
**Adam17**	2.20678	3.35148	5.91309	8.71884	0.10464	2.81164	1.20600	8.03448	13.83238	11.46796	10.19640	8.13353	0.18428	3.38788	11.97989	2.84385
**Adam19**	6.84421	0.00000	2.39632	0.52511	6.38379	4.04164	4.73161	0.00000	0.94780	0.19059	0.03129	0.13383	2.53567	0.00954	1.38349	0.17716
**Adam1a**	0.00980	0.00779	0.01069	0.79350	0.00964	0.00625	0.00749	0.22414	1.08919	0.00000	0.01873	0.00390	0.00662	0.03118	0.64808	0.00703
**Adam2**	0.10127	0.00718	0.00538	0.29397	0.00764	0.00720	0.35539	0.00987	0.11280	0.00816	0.01121	0.00733	0.01042	0.01694	0.02052	0.26803
**Adam22**	0.01382	0.00780	0.01159	0.23391	0.00935	0.00666	0.00748	0.01295	0.03450	0.01218	0.01182	0.02798	0.01281	0.01838	0.04517	0.00738
**Adam28**	0.16099	0.01863	0.27483	0.30963	0.12105	0.06436	0.14322	0.02610	0.46126	0.15023	1.09208	0.03168	0.16724	0.44805	0.16632	0.07859
**Adam33**	0.01346	0.00823	0.01201	0.38626	0.00907	0.00642	0.00742	0.01119	0.87853	0.41874	0.00653	0.00550	0.01119	0.01029	0.00393	0.00701
**Adam4**	0.01369	0.00787	0.00985	0.07196	0.00914	0.00667	0.00742	0.00826	0.61827	0.00368	0.16442	0.00606	0.01683	0.09282	0.00000	0.00727
**Adam8**	1.58376	0.00596	0.20924	1.02454	0.00447	0.00719	0.00606	0.00968	0.63505	0.00591	0.02400	0.00501	0.00998	0.00475	0.13046	0.01945
**Adam9**	0.67147	0.01663	0.28873	2.40319	1.00065	1.25791	0.21611	0.48872	0.20960	0.00000	1.07242	1.47876	0.27584	2.94188	0.68098	0.99406
**Adamts10**	0.01477	0.00830	0.01717	0.00583	0.01981	0.00723	0.00752	0.01439	0.01131	1.97214	0.00971	0.04611	0.01039	0.01005	0.02595	0.00736
**Adamts12**	0.01371	0.00766	0.01053	0.00929	0.00913	0.00675	0.00727	0.01069	0.01203	0.33040	0.01212	0.09047	0.01253	0.03214	0.01050	0.00780
**Adamts13**	1.52108	0.23724	3.73532	3.65527	0.53416	0.50062	2.35937	2.29269	4.06539	6.06664	1.59572	7.67429	0.86354	2.15054	2.26354	0.26839
**Adamts14**	0.01385	0.00817	0.00940	0.01472	0.00918	0.00688	0.00757	0.00679	0.01083	0.00637	0.36838	0.01743	0.00924	0.01055	0.00471	0.00723
**Adamts15**	0.01375	0.00811	0.01225	0.09136	0.00930	0.00664	0.00753	0.01389	0.02116	0.01510	0.01373	0.00823	0.01268	0.01155	0.01292	0.00740
**Adamts17**	0.02352	0.00775	0.02041	0.31564	0.02376	0.02162	0.00715	0.01373	0.01219	0.91478	0.02847	0.00504	0.01663	0.00856	0.17009	0.00712
**Adamts2**	0.14807	0.18735	0.14134	22.79298	0.05054	0.15543	0.07603	0.18656	17.51317	3.61782	0.53852	0.98757	0.34233	4.15279	9.31668	0.08026
**Adamts4**	0.16081	0.01792	0.23799	2.12313	0.04395	0.05051	0.04674	0.00000	1.51017	0.34331	2.17430	0.10692	0.05075	0.78161	0.15293	0.05982
**Adamts5**	0.01377	0.00806	0.01152	0.01057	0.00928	0.00684	0.00756	0.01376	0.01224	0.03339	0.01234	0.04176	0.01310	0.01191	0.01085	0.00742
**Adamts7**	0.00815	0.00841	0.00433	0.01056	0.00952	0.70606	0.00759	0.55142	0.01181	0.00000	0.00891	0.00758	1.09352	0.02006	0.00069	0.02084
**Adamtsl1**	0.01524	0.11264	0.00995	2.59379	0.01000	0.00626	0.00720	0.01021	0.63191	0.00217	0.02544	0.01610	0.01210	0.00380	0.54493	0.00681
**Adamtsl4**	0.64857	0.00703	0.78486	0.92455	0.01092	0.00844	0.00646	1.01734	0.12230	2.83015	0.09533	0.06101	0.00190	0.00327	1.12106	0.00642
**Adamtsl5**	0.00000	0.00694	0.15570	0.00450	0.00734	0.00953	0.00520	0.72891	0.27485	0.20511	3.39908	0.10602	1.15849	0.00662	0.07361	2.87885
**Adap1**	1.28808	0.01786	0.71248	0.74750	0.00400	0.00811	0.00672	0.62157	0.20087	0.71280	0.00539	0.05500	0.19217	0.00988	0.00000	0.01334
**Adap2**	0.00829	0.00803	0.14430	0.00458	0.00889	0.46553	0.00689	0.37911	0.01023	0.00000	0.20153	0.00454	1.04947	0.09625	0.45602	0.02143
**Adar**	5.85039	6.80454	3.50501	2.38475	0.00000	0.00000	0.01955	5.40856	1.16917	0.98883	1.67418	1.51722	0.84135	0.37761	2.68151	5.96548
**Adarb1**	0.31233	0.10548	0.71155	2.73231	0.04787	0.05995	0.07909	0.04957	3.14749	0.22620	2.52544	0.21354	0.98864	0.79347	0.58998	2.21202
**Adarb2**	5.66069	1.33356	7.52648	9.30109	1.66798	0.91592	5.54742	1.61778	7.20450	2.96539	3.55325	1.48185	3.02178	1.70088	0.95811	4.85169
**Adat1**	0.20277	4.13337	1.03409	0.19387	0.00649	0.00592	0.00599	0.01338	0.01274	1.35573	0.31049	0.11130	0.22284	0.00422	1.19137	0.14893
**Adat2**	2.75505	0.00455	0.84719	0.00390	0.02040	0.01969	0.09489	0.83091	0.02309	0.97979	2.32017	0.00000	0.57825	0.82505	0.00000	0.03972
**Adat3**	0.17465	0.01869	0.00000	0.61174	0.12767	0.23161	0.00502	0.02349	2.71819	2.19972	1.29035	0.10093	3.20557	0.02182	0.00000	1.62831

**Adck1**	1.21201	0.03105	3.49709	0.54394	6.25396	2.59623	6.97347	4.56953	1.23280	3.68900	1.88958	1.06061	0.17196	0.03122	1.47588	0.03286
**Adck2**	0.11146	0.00912	0.00000	0.35158	0.01278	2.51600	0.00783	0.24918	0.26724	0.00000	0.00433	0.04639	0.00000	0.00850	0.97812	2.06831
**Adck5**	7.05549	0.38741	5.13410	1.25747	2.50219	2.85718	0.87811	1.23134	1.16202	0.19677	0.98870	0.02977	2.86578	0.00074	0.22267	4.21343
**Adcy1**	0.01294	0.00803	0.01216	0.01667	0.01878	0.00666	0.00754	0.01394	0.07458	0.01491	0.01217	0.00804	0.01265	0.01250	0.00976	0.00740
**Adcy10**	0.01713	0.00862	0.00784	0.07814	0.01077	0.00690	0.00791	0.03459	0.00780	0.15403	0.00753	0.02791	0.01163	0.55217	0.21244	0.00747
**Adcy3**	0.01270	0.00796	0.01343	0.13116	0.01005	1.30457	0.00742	0.57273	0.00942	0.18508	0.00821	0.02551	0.00000	0.01014	0.00893	0.00697
**Adcy5**	0.01257	0.00783	0.13185	0.04864	0.00901	0.00569	0.00753	0.01178	1.15449	0.12597	0.00000	0.02536	0.01151	0.01403	0.14521	0.00763
**Adcy6**	0.00813	0.00753	0.00000	0.63313	0.00946	0.01348	0.00671	1.31424	0.02096	0.53165	1.26521	0.04373	0.00000	1.56176	0.81339	0.00711
**Adcy7**	0.01088	0.00805	0.35733	0.83757	0.00846	0.00886	0.00718	0.04051	0.27330	0.47925	0.00892	0.00592	0.00782	0.00576	0.07621	0.00683
**Adcy9**	0.43479	0.00422	8.22132	0.70972	2.58858	0.01277	0.36097	1.61462	0.08809	4.86558	0.76628	0.17633	0.00000	2.06185	1.88648	2.61160
**Adcyap1r1**	0.01977	0.00791	0.01963	0.19590	0.00984	0.01994	0.00725	0.01227	0.43784	0.00918	0.01865	0.00713	0.38124	0.01662	0.16324	0.01424
**Add1**	8.50754	6.00369	5.18976	7.84985	4.83782	0.02010	9.20430	2.44737	2.78909	9.68568	1.14644	5.07736	7.47499	0.01133	0.00000	1.52983
**Add2**	0.01763	0.00792	0.01207	0.16528	0.01166	0.00704	0.00758	0.01339	0.33609	0.00000	0.01122	0.05587	0.15068	0.01325	0.40598	0.00762
**Add3**	3.57461	4.98975	2.83494	0.98448	4.08380	0.00188	2.01937	2.82898	0.62431	1.05637	0.00000	0.02123	0.00000	0.00234	0.29359	1.45891
**Adgra2**	0.03748	0.00753	0.00891	1.75872	0.00846	0.71196	0.00728	0.05644	5.37344	0.18900	0.16984	0.09256	0.15087	0.05600	0.26212	0.01421
**Adgra3**	0.07769	0.00776	0.36144	4.23082	0.02644	0.35317	0.00679	0.00745	0.07845	1.99540	0.03739	0.07231	0.00626	0.00140	1.63476	1.18672
**Adgre5**	6.46742	0.52929	8.13933	0.00000	5.58724	6.19307	5.11654	9.66040	0.00060	0.00000	3.47378	0.00615	3.28642	0.00010	0.00979	1.24036
**Adgrf3**	0.00724	0.00803	0.00124	0.07920	0.00910	0.00826	0.00739	0.67314	0.01056	1.44200	1.62330	0.01705	0.00000	0.00789	0.48244	0.00709
**Adgrg1**	0.00514	0.00837	0.00285	0.56212	0.00904	0.00832	0.04120	0.28902	0.18734	0.42180	1.31260	0.02808	1.56851	0.00598	0.00000	0.01371
**Adgrg2**	0.01366	0.00818	0.01206	0.12242	0.00922	0.00686	0.00760	0.01412	0.07916	0.11391	0.01253	0.00886	0.01304	0.01122	0.00920	0.00740
**Adgrg3**	0.11215	0.00797	0.00668	0.00459	0.00774	0.00717	0.00753	0.01564	0.31283	0.00507	0.17056	0.06014	0.01152	0.01117	0.00775	0.00715
**Adgrg6**	4.18686	0.00231	0.00339	0.00915	1.87808	0.00498	0.00436	0.00553	0.01131	0.07128	0.01122	0.00436	0.01731	0.01197	0.01272	0.00292
**Adgrl1**	2.48590	0.00000	1.81852	1.66278	3.17694	0.01529	3.22604	0.10470	0.58194	0.10108	0.00532	0.02971	1.54904	0.00871	0.00000	0.12444
**Adgrl2**	0.00000	1.88953	0.00684	0.07436	0.00754	0.00637	1.72172	0.00908	0.00817	0.03134	0.01125	0.01645	0.46323	0.00714	0.00000	0.85270
**Adh1**	0.00501	0.01848	0.03024	0.00666	0.06423	5.34487	0.00661	0.00971	0.01300	0.19678	0.00774	0.00589	0.02061	0.01136	0.00983	14.55291
**Adh5**	15.97264	13.65097	14.66812	11.39314	13.96681	2.56032	11.94716	11.89144	1.05724	7.07170	4.75525	5.54461	11.41109	1.80174	8.37908	8.93751
**Adhfe1**	0.00731	0.20927	0.00869	0.77066	0.00849	0.00683	0.00813	0.25543	1.09220	0.46705	0.00000	0.01710	0.00704	0.01152	0.00000	0.00736
**Adi1**	16.82428	1.36027	6.65999	8.84244	3.16081	0.13317	3.69523	7.44051	5.90980	7.36984	5.10489	1.29098	10.37103	4.89600	2.99290	5.46980
**Adipor1**	9.58693	8.13582	9.60544	6.87897	8.61462	8.86649	14.56342	9.55316	6.44648	16.37717	8.21123	6.87214	6.35794	6.43455	9.50503	6.74537
**Adipor2**	10.25136	3.97568	6.38237	2.59432	7.25138	8.24816	3.93228	2.01153	4.18107	6.02122	0.77957	1.07912	8.09853	1.86803	4.42785	3.55922
**Adk**	2.42507	9.37565	3.23076	2.36219	3.16046	1.12342	2.43090	0.00000	4.28307	1.83015	0.00000	0.09049	1.15680	0.00000	3.83257	5.05199
**Adm**	0.01560	0.02837	0.17160	0.16118	0.00882	0.00581	0.00725	0.01119	0.35152	0.10424	0.00985	0.02508	0.01108	0.01150	0.00985	0.00759
**Adm2**	0.01377	0.00795	0.01216	0.73820	0.00925	0.00662	0.00742	0.01274	0.00666	0.08551	0.01311	0.00747	0.01299	0.01186	0.00935	0.00764
**Adnp2**	7.76115	2.96805	0.07253	2.06702	0.02439	0.00000	0.01738	0.06608	2.87045	0.97301	0.76852	5.33669	2.92696	0.01274	5.10273	1.28846
**Ado**	3.19999	3.02609	1.32423	0.46547	0.01212	0.01379	0.00255	1.39399	2.40248	1.04656	0.62763	0.05047	1.65479	2.30814	0.81161	0.00810
**Adora1**	1.43483	0.04357	0.89812	0.00925	2.09921	0.00752	1.48646	0.00000	0.01116	0.00000	0.01182	0.00173	1.12253	0.01781	0.00038	6.99420
**Adpgk**	1.69063	1.88348	4.55756	2.56685	2.19334	2.81681	3.23480	4.00754	4.87193	0.73612	1.97840	5.39115	5.51719	0.37897	4.63001	3.25359
**Adprh**	13.01595	3.90044	4.02139	2.62514	9.18799	6.29701	7.99111	16.64145	1.04366	7.29500	8.32006	1.05207	9.81608	0.00000	0.00000	6.93222
**Adprhl1**	0.00895	0.00741	0.01518	0.03763	0.00928	0.00624	0.01640	0.71023	0.17387	0.00000	0.09002	0.00488	0.83445	0.01354	0.00000	0.00678
**Adprhl2**	6.16370	3.76275	5.89579	0.06392	0.03535	0.04723	0.57846	2.28932	0.30256	6.62397	0.00000	0.44255	3.28342	0.00256	0.58625	3.81689
**Adprm**	4.85683	0.00016	3.31919	1.87259	1.17884	0.00008	0.01292	0.00000	0.44190	1.72135	0.29775	0.01206	0.66796	0.00523	0.17438	1.79794
**Adrb3**	0.19514	0.00603	0.08254	0.00457	0.00655	0.13217	2.14134	0.78260	0.01091	0.00000	0.19113	0.00267	0.45187	0.00576	0.09838	3.07653
**Adrbk1**	4.64348	0.52508	2.32703	0.90372	5.05356	0.19485	1.25978	6.55343	1.35328	14.63334	2.80642	0.84472	3.24973	0.00000	0.35170	0.40778
**Adrm1**	23.61496	1.50133	2.55990	21.61121	16.01623	2.91676	3.24716	13.55168	22.73638	37.97671	29.35349	2.76199	34.78437	0.21067	0.00000	12.92497
**Adsl**	7.04947	7.34639	9.31100	11.97093	9.23486	2.55037	2.80554	13.96897	15.26257	13.36837	9.58622	2.76792	11.06278	1.59960	7.57842	6.28271
**Adss**	12.23394	3.87291	11.13967	6.93226	10.06829	5.19096	8.18784	7.82439	5.10241	9.33280	8.49641	7.56091	10.76344	4.33698	10.71739	6.03583
**Adssl1**	0.01338	0.00711	0.00437	0.00469	0.00813	0.00769	1.04349	0.00000	0.01112	0.13015	0.41126	0.01683	0.00806	0.01011	0.00490	0.58600
**Adtrp**	0.80253	0.03903	8.08654	4.55389	0.03325	3.60307	1.24145	15.19331	3.48315	13.18242	10.35220	14.30324	6.08370	18.15070	7.24795	0.01752
**Aebp1**	0.12995	0.00784	0.40937	0.00796	0.01489	0.00925	0.00801	0.24956	0.01137	0.74711	0.00727	0.25420	0.00495	0.01536	0.00000	0.00730
**Aebp2**	12.95750	6.45855	13.17467	10.33235	0.38042	5.00351	5.41599	12.45856	10.09630	13.88441	19.02565	8.96152	4.87799	20.60444	16.70619	1.61626
**Aen**	7.83241	5.25434	7.65370	8.39700	9.94862	0.12305	7.02711	9.56577	6.38471	6.28700	6.70629	1.12701	8.46784	2.40732	2.96246	2.93871
**Aes**	0.88319	0.00583	0.06270	6.08496	0.00543	0.06564	0.16148	2.32750	7.11113	5.91167	4.62726	0.00000	0.98507	0.00000	0.00000	2.29874
**AF067061**	0.01105	0.00860	0.01815	7.07057	0.00953	0.04984	0.00788	0.07965	12.34182	0.25134	0.14975	0.02432	0.08537	0.01234	0.51311	0.00585
**AF067063**	0.00000	0.01607	0.00000	20.90326	0.02450	1.84138	0.00007	0.87349	29.54844	10.88681	5.32011	4.37930	4.69302	8.98281	10.03821	0.00516
**AF357399**	0.01360	0.62170	0.10535	0.00545	0.00777	0.00578	0.00775	0.03589	0.07965	0.00636	0.01114	0.00953	0.21807	0.01211	0.06926	0.02815
**AF366264**	0.08944	0.01726	0.00598	0.00576	0.00843	0.00787	0.00741	0.23029	0.10290	0.25100	0.04514	0.05672	0.08485	0.03644	0.04534	0.00734
**Afap1**	0.02371	0.00779	0.03475	0.22706	0.03835	0.05319	0.00728	0.02610	0.76737	0.23252	0.26918	0.03821	0.03293	0.13739	0.09423	0.01450
**Afap1l1**	0.03501	0.00681	0.02510	0.00226	0.00893	0.00662	2.04023	0.65222	0.44409	0.00000	0.49587	0.00115	0.18836	0.00886	0.39909	0.01463
**Afap1l2**	0.01319	0.00785	0.00972	2.59533	0.00918	0.00609	0.00727	0.01345	0.53350	0.21823	0.05979	0.02298	0.01062	0.00177	1.34030	0.01370
**Aff1**	1.35961	0.06737	0.54646	13.63140	1.44074	0.03274	2.35453	3.77916	16.92354	7.64912	7.09286	1.20303	0.00000	3.07648	6.63874	0.00837
**Aff3**	0.00859	0.00836	0.02374	2.17392	0.01009	0.00809	0.00811	0.21060	0.02367	0.26973	0.01087	0.04623	0.27219	0.01926	0.43624	0.00765
**Aff4**	6.47693	2.60035	4.14776	10.41900	0.41102	8.11711	1.87371	7.55238	9.55360	7.46647	2.19360	0.83182	4.76502	11.42104	13.62450	4.01780

**Afg3l1**	4.71699	4.16826	7.70936	1.58562	4.04929	0.00674	6.97616	6.73191	0.82071	8.35315	3.91491	0.58037	8.34101	0.04158	1.30907	4.37394
**Afg3l2**	5.86464	1.28243	2.30491	7.56192	7.96792	3.71867	3.33012	1.49848	5.05598	2.44607	1.57453	1.79359	3.81914	0.00000	0.00000	4.97685
**Afmid**	0.88971	4.18257	10.01134	2.75270	3.85329	2.57462	7.55296	8.97856	3.16738	10.02823	8.40181	1.69776	5.66408	5.34227	7.89548	2.90436
**Aftph**	1.48398	3.68889	1.18723	8.05581	0.30347	1.70792	1.79186	3.32481	4.83409	4.92992	2.05623	6.19968	1.54289	6.62287	9.24296	0.04458
**Aga**	0.01349	0.00841	0.01179	0.61783	0.00928	0.00683	0.00774	0.01882	0.00000	0.05799	0.01252	0.02146	0.01281	0.01077	0.00510	0.00732
**Agap1**	0.95588	0.11066	1.54919	0.44075	2.84222	0.27947	0.00648	1.78202	1.21514	0.81241	0.37792	0.00000	0.01249	0.24888	0.24923	0.02812
**Agap3**	2.84885	0.06158	2.00351	3.89390	4.55120	4.79809	2.43948	2.56259	0.49325	2.14404	1.13965	0.00000	8.43674	0.02879	0.00000	1.51854
**Agbl2**	0.02042	0.00803	0.00219	0.03840	0.00967	0.00609	0.00715	0.01197	1.57438	1.38710	0.03295	0.09998	0.01044	1.32573	2.94407	0.00708
**Agbl3**	0.01374	0.00817	0.01235	0.92137	0.00934	0.00657	0.00751	0.01399	0.01466	0.01502	0.01295	0.00819	0.01267	0.01024	0.00621	0.00726
**Agbl5**	0.00000	0.00781	1.00425	0.34758	0.00541	0.00592	0.00752	0.08446	1.24208	0.14261	0.11073	0.02927	0.00709	0.01424	0.02837	3.08129
**Ager**	0.01463	0.00786	0.00673	0.00911	0.00917	2.79421	0.00716	0.01069	0.01279	0.00610	0.00963	0.00806	0.00968	0.01303	0.01019	0.00623
**Agfg1**	5.64015	8.25754	5.53340	14.53775	7.89595	8.19791	5.70988	5.30379	15.42388	3.56570	5.46342	7.69525	3.52333	6.53862	4.81936	0.32994
**Agfg2**	0.73673	0.01850	0.57423	1.96467	0.01439	0.00608	0.87536	3.36856	5.50804	0.15411	0.74726	0.08311	0.85505	2.84441	1.82705	0.23639
**Aggf1**	6.25615	4.45723	6.73813	10.02242	4.17338	2.01952	6.06882	11.20437	12.52281	15.53982	6.83146	6.35138	7.84200	15.04357	19.12822	3.46899
**Agk**	0.97990	0.00606	0.06295	0.25443	0.00585	0.00667	0.06552	2.40931	0.00523	0.26461	0.78841	0.18738	0.00000	0.26660	0.57884	0.07231
**Agl**	0.88303	0.08808	2.07935	5.61307	0.57457	0.12133	0.12246	2.06401	8.70650	0.59857	9.53702	0.39428	0.20281	3.33672	0.29276	0.63766
**Agmat**	0.11391	0.11772	1.92994	1.81242	0.00454	0.00540	0.17841	1.30043	1.47848	2.94188	2.40837	0.11839	0.90512	0.90380	2.07415	0.00528
**Ago1**	3.77638	0.00314	0.06069	0.97229	0.01937	0.00217	0.00262	2.24291	0.01652	4.30543	4.12379	0.28727	0.56684	3.58704	3.03927	0.04608
**Ago2**	2.49928	5.78003	10.45851	8.43244	1.57820	0.01235	7.10208	17.25121	13.44344	12.89306	6.88366	9.24890	2.83316	3.05359	8.61821	1.02418
**Ago3**	0.00000	0.00787	0.01746	4.19538	0.00795	0.00620	0.00749	0.72358	1.08215	1.68273	0.33916	0.68534	0.01847	3.84038	3.51724	1.74807
**Ago4**	0.00000	0.00603	0.00000	0.09837	0.01585	0.00465	0.00534	0.00000	1.21601	5.08371	1.75174	6.30905	0.55914	12.24866	5.42379	1.99434
**Agpat1**	1.48334	0.00639	0.04934	3.41712	0.00741	0.00643	0.00609	0.00521	0.62801	0.00000	0.17322	0.01374	0.09621	0.00001	0.20062	0.00512
**Agpat2**	20.19735	11.17312	10.40094	3.85649	5.54308	12.58886	11.21462	17.30147	5.46037	18.15447	13.71959	10.58763	20.95628	0.12828	4.11597	11.41305
**Agpat3**	1.40941	0.00546	3.22340	0.89279	0.00838	0.00551	0.00483	0.05149	0.02135	0.06361	2.62200	0.26895	0.21340	0.00064	0.39444	0.14533
**Agpat4**	0.05106	0.04596	2.89469	0.00000	0.01048	3.85766	4.23911	0.00000	0.10283	0.69126	0.63267	0.00000	0.25354	0.05448	0.00000	2.24040
**Agpat5**	4.30899	3.37203	1.65690	12.42507	2.42251	2.09026	1.26639	11.11745	19.98847	13.46046	15.71295	9.51087	5.68070	12.08539	14.23665	2.99301
**Agpat9**	1.64451	2.15072	3.50574	2.43332	1.10783	4.22079	1.98109	12.35815	4.25557	2.81191	6.17804	2.62045	2.41537	24.85866	12.42832	1.94208
**Agps**	6.03501	6.59141	6.12578	10.25807	10.71051	7.12513	5.37700	1.31977	7.62567	8.36505	7.77483	7.83701	4.16154	10.84796	10.91927	2.96029
**Agrn**	0.89455	0.00436	3.98070	0.67220	4.45083	2.04339	0.00457	1.13709	0.98525	0.00000	1.49352	0.07929	2.06661	0.45036	0.00000	4.52281
**Agrp**	0.01259	0.00851	1.16249	0.36918	0.02094	3.03692	0.00606	0.00000	0.33915	0.86516	2.84452	0.23160	1.26622	0.01181	0.08081	0.00468
**Agtpbp1**	0.12185	0.00833	0.57372	7.25150	0.00682	0.00476	0.00737	0.09285	1.81596	1.04077	0.69233	0.08786	0.26801	1.40189	6.76636	0.00541
**Agtr1a**	0.01221	0.00807	0.20696	0.33876	0.01826	0.00562	0.00742	0.07964	0.57774	0.00899	0.03614	0.00706	0.00780	0.01152	0.00000	0.00718
**Agtrap**	13.32140	1.89441	10.19213	1.90076	6.69688	5.05949	8.17717	9.25234	5.16958	3.69510	5.99253	0.93321	2.74798	0.24133	0.30491	7.14179
**Ahctf1**	3.62860	11.37889	5.28674	22.37861	1.77156	5.64036	1.45651	12.66446	24.70692	18.19189	8.59903	11.85161	2.80041	7.01512	10.54389	2.44041
**Ahcy**	21.78438	9.67101	16.64257	0.00000	14.65852	5.68474	11.95384	14.30357	0.57943	5.42600	5.14912	0.61905	13.85117	0.01797	1.77461	7.61544
**Ahcyl1**	5.58360	11.53538	5.62549	5.52967	8.59114	3.94699	5.49428	5.56182	3.58790	7.39934	4.30023	17.61945	2.35586	7.21710	5.62512	3.73958
**Ahcyl2**	3.95712	0.01469	1.09230	3.07619	0.01102	4.33301	0.01332	0.00000	3.14969	2.38880	1.24457	0.11365	1.68112	6.40753	1.81995	0.00691
**Ahdc1**	0.00000	0.00748	0.01382	0.49667	0.00700	0.00710	0.00713	0.35799	1.54305	0.00460	0.00000	0.01468	0.00000	0.02485	0.12251	2.55973
**Ahi1**	0.01375	0.00814	0.01230	0.37643	0.00932	0.00664	0.00751	0.01377	0.00425	0.02255	0.01293	0.00806	0.01285	0.01093	0.00615	0.00747
**Ahnak**	0.00000	0.00718	1.62332	1.14955	0.16006	0.44402	0.02604	0.00000	1.89152	0.12738	0.41174	0.29393	0.73494	3.55710	0.00000	2.42760
**Ahr**	0.01357	0.00753	0.00645	1.00238	0.00924	0.00682	0.00712	0.00207	0.00000	0.00000	0.26624	0.00449	0.00941	0.50279	0.11597	0.00705
**Ahrr**	0.03101	0.00809	0.04342	0.01035	0.01326	0.00587	0.00729	0.74798	0.02229	0.00531	0.00874	0.00660	0.01283	0.01652	0.00000	0.00747
**Ahsa1**	29.45009	15.31452	26.12790	13.93448	13.45689	14.07434	17.05972	23.99904	12.45772	11.61274	8.15263	2.66678	23.34442	0.40876	7.18103	15.56806
**Ahsa2**	2.97658	0.00422	1.83479	0.59261	0.00000	2.47622	0.00408	0.30856	0.47160	1.83226	0.26711	0.01264	1.05617	0.59756	0.16907	0.57957
**Ahsg**	0.28589	0.00724	0.00691	0.00572	0.00661	0.00821	0.00710	0.56764	0.01077	0.00000	0.36356	0.01426	0.07292	0.01155	0.00210	2.37465
**AI314180**	3.55091	5.02873	8.71790	6.88524	10.61664	0.47134	8.06518	8.67224	2.83598	8.31428	4.73520	1.52682	3.94706	13.46380	7.69431	2.27704
**AI314278**	0.00836	0.00768	0.02641	0.30059	0.00942	0.00781	0.01743	0.11773	0.36098	0.61406	0.34310	0.31180	0.04287	0.26968	0.61698	0.00723
**AI413582**	2.45277	0.20253	1.62630	3.43845	0.01079	0.05939	0.17367	1.16885	0.28476	3.53260	0.95473	0.10330	1.33792	0.20154	0.05444	0.01094
**AI427809**	0.01316	0.00808	0.01184	0.43030	0.00915	0.00644	0.00738	0.01361	0.03472	0.01155	0.01181	0.00817	0.12673	0.01226	0.00675	0.00715
**AI450353**	0.67789	0.00679	0.00484	0.01583	0.00657	0.00691	0.00688	0.02473	0.02797	0.01047	0.01271	0.00736	0.01103	0.01179	0.09799	0.02825
**AI464131**	0.49245	0.04433	4.13164	8.40581	0.06736	5.37412	3.01013	2.01310	8.92389	5.95394	7.75312	2.54827	2.82111	8.37657	13.93676	4.44979
**AI467606**	0.00992	0.00740	0.00760	0.01101	0.00957	0.00690	0.00707	0.97859	0.01137	0.00000	0.00761	0.00559	0.03215	0.03363	0.00000	0.01472
**AI506816**	0.09292	0.08635	0.25474	0.74983	0.07643	0.10796	0.06596	0.01606	0.68610	1.99766	0.30292	0.36489	0.47290	0.72990	3.19699	0.01339
**AI507597**	0.00441	0.22834	0.02431	0.01072	0.01762	0.00607	0.28497	0.37137	0.01198	0.00828	0.01091	0.00701	0.00601	0.01356	0.00129	0.00726
**AI597479**	3.55741	1.99814	4.55797	3.10626	0.00000	0.66700	2.07958	2.58716	0.24027	6.39618	2.32597	3.64194	1.47246	2.47563	8.02538	0.02826
**AI661453**	0.02251	0.00707	1.08685	6.63691	0.00644	0.29448	0.03654	0.22965	10.54074	2.05566	6.44122	0.21436	0.18592	1.31703	2.37555	0.04076
**AI662270**	8.61171	8.34267	2.97648	0.01824	1.07330	0.00000	2.43961	0.00000	0.00835	0.00000	0.00000	0.04101	1.12546	2.57948	1.89872	3.21091
**AI837181**	0.86563	0.02933	0.18951	0.68026	0.01010	7.95704	0.01432	3.76332	0.07414	1.71068	0.90985	0.01916	3.86521	0.00118	0.00000	3.56197
**AI846148**	1.78096	0.24317	0.84216	4.83711	0.55610	4.31281	2.44949	2.74475	3.83393	2.01095	0.83040	0.12602	0.90471	0.69565	5.48513	0.16033
**AI854703**	0.00297	0.00812	0.00371	2.66700	0.01688	0.00681	0.00785	0.19126	0.74616	0.00000	0.56446	0.02473	0.00000	0.00049	0.62688	0.81506
**AI987944**	0.00399	0.03874	1.47299	3.53950	0.01733	0.01627	0.02715	0.49612	0.07632	0.37075	0.06042	0.06455	0.23505	0.08316	2.99035	0.01393
**Aicda**	0.01374	0.00786	0.01187	0.00460	0.00932	0.00654	0.00750	0.01245	0.18187	0.03060	0.00953	0.01661	0.01236	0.00678	0.28890	0.00723

**Aida**	4.59588	0.03328	0.25485	4.18768	1.86975	0.05054	0.02251	0.44693	3.08492	0.00000	1.06488	0.05877	0.14093	3.14396	5.06254	1.61381
**Aif1**	0.01272	0.00839	0.01122	0.00692	0.00932	0.00674	0.00799	0.03421	1.08410	0.01709	0.00443	0.00718	0.01186	0.01676	0.01193	0.00735
**Aif1l**	0.50518	0.00738	0.00079	0.53036	0.83018	0.00714	0.01572	0.00000	1.35501	0.73371	1.59148	0.09369	1.05965	0.00140	0.86910	0.00635
**Aifm1**	10.05675	7.72034	4.19322	4.95484	5.11602	7.64523	2.23057	3.21606	7.46399	11.18367	4.82631	3.83554	4.88935	11.51014	14.09467	7.49236
**Aifm2**	0.29663	0.00614	2.25002	0.02813	0.00269	0.00816	1.62335	0.00000	0.17046	0.38156	1.53799	0.00371	0.00000	0.39511	0.00000	1.61126
**Aifm3**	0.00671	0.00807	0.44193	0.00301	0.00821	0.00580	0.00699	0.37215	0.00923	3.07418	0.94881	0.05929	0.39901	0.01681	0.90320	0.00712
**Aig1**	0.01331	0.00770	0.01226	0.60132	0.00898	0.00648	0.00730	0.01057	0.00386	0.82367	0.01207	0.00497	0.01130	0.00470	0.15983	0.00738
**Aim1**	1.60697	0.00423	1.23162	1.90800	0.00216	4.61620	2.93596	2.63624	0.43525	0.00000	0.00000	0.21380	1.72147	5.00760	0.96414	1.20586
**Aim1l**	0.62680	1.97801	4.46073	1.40256	1.28640	5.13086	3.98666	3.44774	0.58239	0.00000	1.26020	0.00000	2.36836	0.00000	0.02335	0.05075
**Aim2**	0.01512	0.00773	0.02420	0.03225	0.00902	0.05859	0.02751	0.03557	0.01155	0.40600	0.01109	0.03438	0.03105	0.14356	0.03447	0.00738
**Aimp1**	23.79788	14.33499	19.78887	0.63288	9.93513	12.43812	8.79406	20.04987	0.34616	12.39675	14.44688	5.45499	14.47275	9.53209	3.82364	9.85943
**Aimp2**	11.79572	8.38891	14.29079	9.64582	10.86500	12.86277	6.19842	13.23192	13.32678	17.43189	8.92418	9.14097	11.48221	0.26700	7.87234	5.91078
**Aip**	6.48313	4.92391	5.16153	1.25716	2.24171	4.71153	5.01160	7.66123	2.81300	9.00044	1.40083	0.63011	7.79981	0.01602	0.00000	2.07809
**Aire**	0.01044	0.00776	0.00885	1.03236	0.00838	0.00655	0.00722	0.00777	0.58496	0.12249	0.38715	0.01415	0.00708	0.00551	0.00000	0.51328
**Airn**	0.01135	0.00756	0.01542	0.14495	0.00945	0.00734	0.00723	0.20147	0.02116	0.00887	0.01071	0.00706	0.00985	0.01926	0.05636	0.00734
**Ajuba**	1.56900	0.03263	1.86051	8.53559	4.01224	5.99976	0.00978	7.02561	2.64030	10.91174	9.95300	4.28309	2.90760	1.20860	10.94561	0.13513
**AK010878**	2.79217	1.57233	3.47234	7.51158	4.65200	0.91661	1.46638	2.40278	11.71877	4.40585	4.30270	4.69138	2.26305	1.92598	1.85586	0.17938
**Ak1**	0.93024	0.00647	0.03013	0.00991	0.00597	0.00772	0.03707	0.01224	0.01226	0.15992	0.01229	0.03482	0.01129	0.01137	0.00974	0.00658
**Ak2**	17.30649	10.18920	11.41613	2.17526	7.55410	11.13474	6.07011	7.47308	1.32272	8.21833	10.19078	2.32705	11.83593	0.97105	1.08513	6.20748
**Ak3**	0.01315	0.00794	0.01214	0.00704	0.00891	0.00660	0.00735	0.01053	0.01172	1.14241	0.01100	0.00518	0.01098	0.01183	0.00853	0.00733
**Ak4**	34.36381	19.13015	27.10886	1.10362	17.72652	12.69632	19.39597	21.07427	0.76166	19.80500	5.40630	11.33985	20.77982	16.01426	4.80863	17.41858
**Ak6**	11.04315	2.15514	7.31598	10.58198	9.75263	4.55432	1.61452	8.14727	17.65060	11.92907	6.45876	7.97167	7.89918	11.09271	8.53715	7.04332
**Ak7**	2.94228	0.01131	2.58257	1.07852	0.06640	2.21697	0.27809	13.24035	1.59216	4.78118	4.68823	1.20421	1.80986	4.30731	3.28536	0.03643
**Ak8**	0.00974	0.00803	0.31429	0.28997	0.00844	0.00905	0.00784	0.76288	0.03099	0.84932	0.01795	0.01322	0.00385	0.88717	0.15647	0.00695
**Akap1**	11.69212	2.40031	11.51638	5.38307	9.26207	9.92324	7.41484	20.87342	3.94077	19.25076	5.92566	1.89352	6.02657	1.40993	15.61940	5.22637
**Akap10**	0.01381	2.77737	0.00321	2.69020	0.00793	0.00601	0.00610	0.04719	1.37956	0.70848	0.03057	0.04715	0.00939	0.03173	1.05923	0.00610
**Akap11**	0.00632	0.00689	0.00000	3.61631	1.25371	0.01829	0.01692	0.00729	0.58196	0.82358	0.00063	0.07139	0.01053	0.01021	2.92758	0.00677
**Akap12**	13.31215	9.10803	5.96687	3.36749	13.84873	7.51116	7.90513	5.74060	3.90982	4.71176	5.13783	0.57451	7.67635	7.20352	7.11271	4.59654
**Akap13**	1.40195	0.01500	1.60521	2.66715	3.24627	0.00718	0.00475	0.08309	3.20324	2.59266	0.32701	0.14281	1.52634	1.40315	1.12479	0.00411
**Akap14**	0.27523	0.00787	0.00536	0.06271	0.00720	0.00713	0.00741	0.03395	0.00857	0.76230	0.01225	0.02998	0.00970	0.05552	0.00000	0.00673
**Akap17b**	0.00849	0.12708	0.00000	11.00908	0.91942	0.00511	0.00691	0.00798	2.97982	2.93993	0.09928	0.04921	0.08813	2.79827	9.49329	0.02802
**Akap5**	0.02128	0.02885	0.05378	0.27014	0.00854	0.00680	0.01734	0.00790	1.90053	0.00013	0.30854	0.00194	0.03691	0.07958	0.32266	0.00701
**Akap7**	1.03707	2.03770	0.25246	2.40779	3.56615	0.00523	0.10416	0.00000	1.86145	1.25958	0.00000	0.04855	0.16587	1.22809	4.66446	0.00445
**Akap8**	11.34763	10.98585	9.93421	22.70785	5.89386	5.46969	13.02338	18.01646	32.17397	30.73040	16.73928	21.30204	9.77885	30.84965	24.08147	5.43926
**Akap8l**	6.15963	0.01196	0.00000	0.05128	0.08463	0.54255	0.09341	2.00225	0.10685	5.13128	0.05005	0.23423	3.73869	0.00808	0.13279	2.35730
**Akap9**	0.52073	1.25985	0.00000	3.48307	0.00742	2.41360	0.00663	0.04388	0.19839	0.39020	0.44978	0.00880	0.27926	2.52191	3.51481	0.00508
**Akip1**	4.22307	0.45248	3.46720	2.66008	6.85693	3.57026	3.40148	3.13383	2.51852	2.11088	2.21767	0.14478	7.38448	0.97169	0.00000	0.26393
**Akirin1**	2.36062	6.78240	4.72353	7.79702	2.54630	3.33157	4.47962	9.67559	10.74792	14.43989	11.63554	11.27256	6.85619	5.66631	9.63033	2.22566
**Akirin2**	5.80698	5.23162	2.66439	19.51855	10.09617	0.51350	0.90998	5.55923	19.54512	6.99778	9.33406	0.99471	3.61094	6.76998	6.54370	1.52430
**Akna**	0.72829	0.00798	0.01128	0.14923	0.01715	0.00750	0.00687	0.00907	0.09735	0.00607	0.31084	0.01639	0.00936	0.00876	0.00416	0.02898
**Akr1a1**	24.83529	14.69078	21.39972	12.46128	12.90531	13.99443	12.64822	22.29602	12.24932	25.93541	18.48381	16.17792	20.97973	6.07106	10.33011	17.78109
**Akr1b10**	0.03626	0.38633	0.14478	0.00637	0.03523	0.95531	0.37973	0.06145	0.01150	0.04675	0.00886	0.00751	0.91008	0.00593	0.45087	0.16488
**Akr1b7**	0.01290	0.03024	0.00776	0.50748	0.00908	0.02515	0.00746	0.01659	1.33161	0.00000	0.14518	0.00495	0.01672	0.00488	0.24115	0.00703
**Akr1b8**	10.50533	18.18115	21.81494	1.38891	11.06022	22.59958	14.08789	13.88330	0.28417	0.00000	6.74652	0.03319	30.81651	0.35032	0.00000	9.99279
**Akr1c13**	0.09447	0.00742	0.04517	0.01051	0.00785	0.01200	0.00724	0.19490	0.01198	0.06995	0.01131	0.00796	0.00730	0.01199	0.00865	0.00722
**Akr1c14**	0.01091	0.00800	0.02615	0.00871	0.00957	0.00796	0.00737	0.01396	0.01203	0.44281	0.01404	0.00605	0.40479	0.01150	0.00969	0.00790
**Akr1c21**	4.69028	2.48390	2.18980	0.01210	0.03640	0.00000	0.00000	2.62978	0.00784	0.45390	0.00000	14.71638	0.62287	0.00673	0.00000	0.02240
**Akr1cl**	0.16837	0.00770	0.00554	0.57818	0.01126	0.00661	0.00721	0.00920	0.62286	5.09318	0.00426	0.00000	0.01064	0.00643	0.90932	0.01482
**Akr1d1**	0.01331	0.00795	0.02302	0.16462	0.00911	0.00764	0.00744	0.00888	0.43899	0.00700	0.17844	0.00651	0.01007	0.03517	0.01400	0.00724
**Akr1e1**	2.61516	1.15524	0.84310	0.20689	0.86033	0.24998	0.00450	0.00000	0.00460	0.27321	0.29658	0.01194	0.22360	0.00536	0.00000	0.29346
**Akr7a5**	3.54064	0.00376	0.00023	1.40682	0.02269	0.01332	0.03439	1.73204	0.08490	0.71891	0.21669	0.00000	0.00740	0.00386	0.02212	0.57529
**Akt1**	7.98510	2.88324	1.92204	6.83981	6.16964	7.94013	3.63402	8.85838	6.11756	9.31186	4.98066	0.60570	5.95976	0.00000	0.00000	1.55312
**Akt1s1**	9.61346	0.05467	0.00000	1.42619	8.32229	2.98461	0.49621	13.63903	1.27153	22.28649	5.05634	0.49934	9.25339	0.00000	0.00000	6.47185
**Akt2**	15.54251	2.94647	8.14256	9.61771	6.24668	7.09231	7.79832	6.81729	9.06565	8.55669	6.03449	0.89068	7.69874	0.04325	2.46272	3.30262
**Akt3**	0.00000	4.24940	4.14503	6.97738	0.87023	0.46481	0.02601	1.00762	1.96332	0.31012	0.03148	0.29431	2.63681	0.36440	4.23498	0.04486
**Aktip**	0.00000	1.43801	1.43281	0.92427	0.00610	5.44208	0.37475	0.90539	1.06011	1.57914	0.30821	0.03511	0.22159	0.77050	3.00818	0.00428
**Alad**	11.25845	6.73563	13.76315	1.60581	2.53441	8.82148	7.00631	17.49282	2.94560	7.93125	7.46685	0.22909	9.82324	0.04487	0.00000	8.95826
**Alas1**	2.08743	1.48535	2.12520	6.72581	0.00000	0.38706	1.08066	4.68308	6.09732	0.24953	2.64973	5.68986	0.50325	0.18912	0.80835	1.27472
**Alas2**	0.00000	2.49402	4.91813	0.00000	0.00000	0.00533	2.66047	0.00550	0.67017	17.22812	0.00000	0.00000	0.00410	0.01351	0.00000	0.00448
**Aldh16a1**	8.02510	0.11221	2.00844	1.30023	3.28712	6.41241	4.19334	7.67040	0.98820	4.57509	2.43063	0.15514	9.15940	0.00000	0.00000	1.11856
**Aldh18a1**	6.59511	3.94837	0.00000	2.99953	10.91305	4.84095	1.28814	0.00000	1.99081	1.74589	0.21443	0.06029	1.22226	0.00000	0.57101	13.70880
**Aldh1a1**	0.01254	0.00778	0.01206	0.00549	0.00874	0.00657	0.00726	0.00962	0.02456	2.32100	0.02708	0.00000	0.01085	0.01225	0.00669	0.00760

**Aldh1b1**	0.96813	1.88265	0.70036	0.00000	0.01374	0.02035	3.05453	0.00356	0.08387	1.67101	0.02251	0.00000	0.59890	0.01605	0.00000	0.00487
**Aldh1l1**	0.35566	0.58765	3.17936	0.00526	0.08451	0.58776	0.00690	0.21303	0.01161	0.69717	0.02306	0.04922	0.00000	0.00701	0.06592	2.94466
**Aldh1l2**	0.01128	0.00728	0.00629	1.01544	0.00988	0.00654	0.00716	0.00875	2.04097	0.00000	0.00360	0.73269	0.02924	1.88330	0.00000	0.00713
**Aldh2**	3.94491	0.76512	3.54676	1.98971	0.00834	0.77541	0.00267	0.39684	1.67459	2.28255	0.30726	0.08395	1.05746	0.00000	0.94883	0.79792
**Aldh3a2**	1.11292	0.04994	0.80131	1.26562	0.01417	0.00442	0.02849	0.67449	0.01117	1.14527	0.39271	0.03216	0.00543	0.00299	0.11086	0.00571
**Aldh3b1**	0.01374	0.00783	0.00746	0.00870	0.00919	0.27635	0.00732	0.01107	0.01228	0.39421	0.01213	0.00660	0.00914	0.01166	0.00855	0.05839
**Aldh3b2**	0.00836	0.00770	0.00959	0.00867	0.00775	4.08129	0.00694	0.01047	0.01287	0.00000	0.00853	0.03862	0.00000	0.01431	0.00922	1.84448
**Aldh3b3**	0.01724	0.00811	0.00627	0.20405	0.01003	0.74888	0.00735	0.01199	0.00572	0.02094	0.01087	0.01821	0.00644	0.01121	0.00682	0.18668
**Aldh4a1**	1.02484	0.29747	7.01761	0.43224	0.67595	3.88321	0.85115	1.95216	1.20323	0.33649	3.69019	0.07507	2.39551	0.02576	0.71571	3.97581
**Aldh5a1**	0.01179	0.00790	0.98800	1.40605	0.01508	0.00552	0.00728	0.01078	0.00000	0.00000	0.01070	0.02991	0.00848	0.00619	1.02943	1.00481
**Aldh6a1**	2.15666	2.47638	1.82102	0.95136	0.05039	2.59484	3.64348	2.96600	0.37246	4.99862	1.52544	0.07863	1.69920	0.00205	1.40163	0.63289
**Aldh7a1**	0.34004	2.94519	0.14989	1.71924	1.43075	2.29791	5.28565	1.76609	0.92988	3.26502	0.23275	0.14329	0.42392	0.00000	1.18263	0.01250
**Aldh9a1**	17.33031	10.71590	5.03311	9.68447	17.98915	3.54634	7.27925	6.16645	5.60845	2.11288	1.19622	0.49142	5.79638	0.41827	5.80033	8.99584
**Aldoa**	36.55638	22.76950	32.27388	4.44893	26.75400	16.58035	20.08537	22.40173	13.64268	13.18828	15.11441	4.28042	29.29968	0.21555	1.56213	21.51718
**Aldoart1**	2.09335	1.58223	1.93075	0.10914	1.81051	0.84808	1.44490	0.68075	0.04810	0.25218	0.08607	0.03852	1.52369	0.00606	0.00000	1.61827
**Aldoart2**	0.08733	0.06274	0.04469	0.26872	0.01852	0.02699	0.03883	0.02042	0.73098	0.01744	0.00842	0.01842	0.07852	0.01130	0.00302	0.02904
**Aldoc**	0.67084	0.00593	0.00535	1.28111	0.42915	0.00672	1.59146	0.01261	0.00000	5.63448	0.39449	0.01541	0.15403	0.01828	0.75409	0.00657
**Alg1**	0.06134	0.00595	1.28666	0.00176	0.00534	0.00742	2.01676	2.69455	0.33125	0.44345	0.19392	0.82786	3.00202	0.00380	0.37071	1.16945
**Alg10b**	0.55885	0.00639	0.05649	1.78856	0.07606	0.07930	2.40198	1.02531	0.85338	1.27955	0.32709	0.15417	0.00638	0.00747	0.99316	0.33465
**Alg11**	0.18426	0.00731	2.47019	0.20825	0.00472	0.07874	0.00596	1.90477	0.23513	0.00001	1.99517	0.05359	0.46656	1.74031	0.53740	0.02004
**Alg12**	0.00000	2.21735	2.45283	0.62844	0.02327	0.45513	1.93470	2.00195	1.35149	1.18986	0.92977	0.94768	2.02075	0.05567	0.06464	0.50759
**Alg13**	12.96999	5.49728	9.64333	18.97415	8.29566	1.34209	0.44765	7.28840	20.36549	6.17654	15.54323	12.27369	2.55790	14.65831	5.58618	0.20538
**Alg14**	7.00137	3.95655	8.53449	0.03120	4.67849	0.17199	4.57212	4.60840	0.32800	3.44746	0.04389	1.20462	4.16852	0.56315	1.37875	2.40547
**Alg2**	0.45767	0.00416	0.70336	0.51579	0.01418	0.00378	0.09747	2.25368	0.88144	2.00576	0.40875	8.89812	0.72485	0.02472	3.30835	0.09875
**Alg3**	9.49585	0.02332	6.20837	2.57100	0.17399	5.45814	6.97172	7.69009	0.71033	1.63112	2.94205	4.24856	2.07454	0.00000	0.24743	5.43532
**Alg5**	6.38485	7.14014	4.23004	1.27412	2.11290	4.96418	2.30621	4.65956	0.18074	0.54788	2.44119	0.16557	7.25856	1.71066	0.00004	6.98950
**Alg6**	0.07899	0.03278	0.92952	0.05721	0.02980	0.04322	0.00723	0.12416	0.09028	0.48312	0.78441	0.34304	0.29703	1.63524	0.00105	0.05179
**Alg8**	0.94576	0.11488	1.58658	1.06587	0.03666	0.00635	3.72995	2.41626	0.01661	1.97423	1.99658	0.01043	3.20442	0.44526	0.09736	0.00383
**Alg9**	5.00379	0.00000	4.16447	2.08590	0.01293	0.03443	3.76667	0.82505	0.57024	1.25083	0.33891	0.00000	2.41643	0.26579	0.00000	3.94875
**Alkbh1**	5.67767	2.09534	2.98039	0.10801	6.44815	4.01517	0.68952	3.14097	3.16295	5.05037	1.47291	0.20935	1.95434	2.47946	5.55197	1.41654
**Alkbh2**	3.27790	1.30137	0.52090	3.99573	2.56486	0.01162	0.01360	1.33474	2.98592	5.12669	2.12700	0.15243	0.82836	0.00000	0.36667	0.61461
**Alkbh3**	8.32238	7.88645	4.85860	2.09040	8.38612	4.51297	6.17053	5.99099	1.25337	5.00384	2.90445	4.75961	8.58854	0.02057	2.99826	1.76115
**Alkbh3os1**	0.01110	0.00811	0.00370	0.00930	0.01008	0.69681	0.43644	0.05475	0.01233	0.00407	0.03099	0.00793	0.24122	0.01379	0.00889	0.00713
**Alkbh4**	1.94950	2.40156	1.09476	4.01229	0.09034	0.00461	2.44727	2.35752	3.99533	2.83765	3.06572	0.34390	3.78378	0.02952	0.41699	2.18580
**Alkbh5**	4.10745	0.26315	4.05689	10.97269	0.86007	1.15586	1.85664	4.23989	11.91177	5.19486	2.50248	0.37016	3.03313	2.37438	5.72068	1.13991
**Alkbh6**	4.79699	0.00201	0.02796	0.00330	1.51822	0.01899	0.00022	0.30990	0.01011	1.93485	0.06844	0.01805	4.15951	0.01560	0.00348	3.83836
**Alkbh7**	4.94093	0.01132	1.61305	1.46303	1.57875	0.57127	0.04181	1.19709	2.03120	2.05570	1.53058	0.09045	4.14002	0.00000	0.07921	3.86478
**Alkbh8**	0.01341	0.25507	0.38309	0.48957	0.09024	0.00757	0.00734	1.47190	0.93841	0.00000	0.00000	0.01455	0.32256	1.56064	1.18356	0.01385
**Allc**	1.73315	0.00591	1.10662	0.12932	0.00282	0.00913	0.02753	0.33434	0.02433	1.39916	0.36178	0.60635	0.44914	7.34289	0.52080	0.00437
**Alms1**	0.78683	0.05666	1.43259	2.52724	0.00407	0.00675	0.93385	1.18967	1.39365	0.70170	0.00000	0.01873	0.04176	2.88042	3.81611	0.00567
**Alms1.ps2**	0.57863	0.00740	0.10641	0.17099	0.00655	0.00648	0.00770	0.24318	0.17153	0.49841	0.00000	0.04346	0.00647	0.87608	1.71448	0.00682
**Alox12**	3.12654	0.85386	3.24390	8.81283	0.02731	0.05231	0.13338	3.28487	11.65848	3.36265	3.85807	0.30866	2.30124	4.30412	2.49891	0.03507
**Alox12e**	0.01286	0.00794	0.01194	0.02044	0.00880	0.00656	0.00733	0.01021	0.03278	1.67490	0.01021	0.01411	0.01057	0.01196	0.00689	0.00724
**Alox15**	0.01238	0.00799	0.01202	0.06840	0.00882	0.00666	0.00732	0.01556	0.00818	2.01868	0.01028	0.00339	0.01091	0.00821	0.05710	0.00745
**Alox5ap**	0.01328	0.00781	0.01199	0.00874	0.00903	0.00661	0.00725	0.01027	0.01206	0.44696	0.01265	0.00635	0.07377	0.01141	0.00992	0.00770
**Alox8**	0.08548	0.05021	0.07748	0.50120	0.02896	0.03157	0.06874	0.00734	0.69422	0.02686	0.55113	0.09010	0.04239	0.62117	0.18362	0.02148
**Aloxe3**	0.14745	0.00745	0.00000	1.22351	1.22165	0.00601	0.00711	0.96917	7.54320	2.99605	0.10951	0.19052	0.00501	0.01681	1.81074	0.00664
**Alpi**	0.00739	0.00780	0.07507	0.00906	0.00988	0.02504	0.16248	0.50158	0.01159	0.20573	0.03571	0.00612	0.26997	0.01302	0.01789	0.00739
**Alpk3**	1.64485	0.00681	0.12576	0.27973	0.00957	0.00733	0.00619	0.00897	0.90045	0.01137	0.00581	0.00570	0.01066	0.01251	0.00208	0.00565
**Alpl**	18.68821	0.00000	2.67381	2.65820	2.06482	0.29850	6.65910	0.33290	0.15453	4.44198	0.95995	0.38026	2.42355	0.01984	0.00000	9.10475
**Alppl2**	2.63109	1.89129	28.38337	19.01476	0.90368	10.02491	24.66177	63.99246	13.78556	49.02122	42.58926	9.18784	47.20259	10.99196	10.22072	4.36612
**Als2**	6.85279	9.32501	3.68603	9.33389	2.52245	0.12584	2.84893	6.44695	15.04033	12.36563	10.40427	9.26626	4.91446	14.37320	18.21664	0.98269
**Als2cl**	0.00877	0.00745	0.01106	0.35127	0.00946	0.01524	0.00704	0.99036	0.12802	0.23616	0.13051	0.01321	0.00516	0.02876	0.02677	0.01533
**Als2cr11**	0.01299	0.00808	0.00793	0.00785	0.00943	0.00689	0.00737	0.01096	0.01098	0.04209	0.00686	0.00606	0.01374	0.69653	0.44361	0.00744
**Alx4**	0.00117	0.00713	0.04408	0.90158	0.01861	0.00639	0.04678	0.48866	0.78706	2.14768	1.46584	1.07912	0.34962	1.23125	1.74220	0.01388
**Alyref**	2.63917	3.05285	2.12524	0.53886	0.59457	0.04666	2.26825	1.55002	3.37446	9.38551	2.11589	0.03403	3.52850	3.95798	4.54049	1.65625
**Alyref2**	0.12971	0.04113	0.07614	1.46704	0.02206	0.01205	0.04982	0.02781	1.95929	0.19459	0.27270	0.00199	0.12152	0.16333	0.15643	0.06570
**Amacr**	1.30689	0.00694	0.01338	0.52898	1.50048	0.01150	0.00710	0.18936	0.38451	0.01653	0.00682	0.03496	0.35596	0.00860	0.25722	0.01317
**Ambra1**	2.62461	0.17162	1.27706	6.08600	0.00287	0.01706	0.00197	2.53331	9.66765	2.28157	4.17233	0.08370	1.95567	1.29869	3.12280	1.72855
**Amdhd2**	4.09779	6.11089	9.46122	1.22374	2.60868	4.72971	1.09250	9.63281	0.87846	2.70275	5.92251	0.05800	11.99862	0.00000	0.00000	6.65719
**Amer1**	0.01380	0.00761	0.01118	1.40626	0.00932	0.00640	0.00733	0.01140	0.00000	0.00613	0.02795	0.01688	0.01887	0.01648	0.32630	0.00715
**Amfr**	3.73523	0.61025	3.46440	2.39287	0.11031	2.58750	3.47352	0.06585	0.02925	0.83961	0.60704	0.15726	0.14617	0.00208	3.87798	0.02924

**Amhr2**	4.41805	0.85392	0.83685	9.41755	1.32030	1.90990	5.79914	5.81089	12.28857	18.36495	8.51297	0.51299	2.13730	0.00000	0.00000	0.62191
**Amigo2**	0.01073	0.00705	0.00750	0.00858	0.00937	0.00688	1.53272	0.00969	0.01216	0.31524	0.01178	0.00610	0.02366	0.01136	0.00971	0.01432
**Amigo3**	0.00774	0.00721	0.00429	2.72146	0.00923	0.00736	0.91387	0.46064	0.16068	1.24434	0.51027	0.08709	0.00399	0.00003	0.15620	0.00681
**Ammecr1l**	2.78871	1.54526	3.76386	19.15192	0.05240	3.05972	4.56847	6.55338	18.89190	19.79140	9.27743	4.57335	5.36783	30.94389	27.50192	1.08098
**Amn**	0.00000	0.00701	0.11276	0.37395	0.00642	0.00892	0.00510	0.00000	0.41840	2.38422	3.05367	0.00003	1.69032	0.00142	0.00000	3.59342
**Amn1**	1.67973	3.21225	8.07447	1.18885	0.00000	0.17828	1.22930	0.75721	1.16388	1.75108	3.82081	0.24626	0.10528	1.28112	1.51390	0.08398
**Amot**	7.17296	0.07864	6.12403	1.95164	9.72849	3.97729	7.43324	5.05067	0.95335	2.96999	5.84099	0.04722	5.17006	14.40427	0.00000	6.89630
**Amotl1**	0.01324	0.00812	0.01064	0.43499	0.00909	0.00681	0.00751	0.01244	0.04025	0.32750	0.03614	0.05210	0.06258	0.05093	0.02970	0.00720
**Amotl2**	0.00000	0.00510	3.74759	0.00000	0.04628	0.00913	0.00000	10.31040	0.04171	8.90146	6.39717	1.20092	8.65162	3.01486	3.54394	6.33380
**Ampd1**	0.01336	0.00800	0.00847	0.72506	0.00910	0.00633	0.00720	0.00015	3.89023	0.29291	0.31678	0.00000	0.00929	0.02020	0.12521	0.00702
**Ampd2**	1.41205	0.66569	0.00000	0.26687	1.01954	2.09211	0.02834	1.00393	0.34821	0.78467	0.03656	0.08841	2.55037	0.00638	0.00000	0.33465
**Ampd3**	0.01289	0.01841	0.00468	9.19530	0.00904	0.00545	0.00684	0.00021	3.69523	0.00000	0.25925	0.04411	0.00965	0.12814	1.73790	0.00539
**Amph**	3.04173	0.00506	0.00272	0.00349	0.17770	0.00549	0.00504	0.00000	0.29212	0.00000	0.78389	0.00203	0.00533	0.00556	0.20105	0.00392
**Amt**	0.04789	0.00762	0.04384	0.05572	0.01676	0.00834	0.00713	0.01027	0.00866	0.00462	0.00726	0.00389	0.01241	1.03756	0.11096	0.00731
**Amy1**	0.01255	0.00773	0.00782	0.03787	0.00928	0.00667	0.00726	0.00972	0.01233	0.32965	0.00786	0.00348	0.01159	1.02316	0.00000	0.00717
**Amz1**	0.01406	0.00773	0.00900	0.38860	0.00913	0.00659	0.00715	0.17032	1.64244	0.01721	0.00000	0.00405	0.00772	0.00654	0.15028	0.00739
**Amz2**	6.08141	6.00749	6.64276	0.67327	0.00000	3.51568	4.49395	3.97677	0.58254	6.47447	4.50715	0.21200	2.13102	0.59281	3.22599	0.00759
**Anapc1**	5.80996	9.46247	8.86872	8.05357	3.80937	2.60546	3.51767	4.56469	2.27401	8.46253	6.43413	6.11736	4.84946	4.60360	9.68955	0.40894
**Anapc10**	8.66645	7.95891	9.91790	5.19271	4.91474	5.19358	5.55709	10.47804	4.94280	16.03225	15.61184	6.32920	7.21851	18.94716	10.64623	4.42442
**Anapc11**	4.22129	0.48440	2.38441	6.44668	3.46165	2.16394	2.74074	6.22718	5.96399	9.71492	6.29312	1.22417	4.44484	0.56952	1.21851	2.46934
**Anapc13**	2.81029	0.08867	1.62950	1.84485	1.53381	0.80395	0.03309	0.05567	1.00334	0.45789	6.49716	0.12263	1.47195	0.02993	0.21230	0.99912
**Anapc15**	11.03105	3.59956	16.07862	13.12464	12.30551	5.18221	5.10868	11.28872	15.55266	15.59390	10.01686	2.02947	10.73822	3.54870	11.75568	1.70355
**Anapc16**	0.38108	0.93508	0.33760	12.35435	1.49970	0.00409	0.20530	0.59234	4.56506	1.01963	6.20847	0.45529	1.48298	0.47803	2.48609	0.00235
**Anapc2**	8.60036	8.81546	10.25646	2.17188	7.43070	1.44415	2.41650	12.33344	3.53878	9.85278	6.66450	0.48586	7.35244	3.82232	4.84878	8.39382
**Anapc4**	6.12509	3.02683	6.07123	1.37214	4.19456	4.48011	4.89110	8.79861	4.94358	8.29297	3.67520	7.32492	3.22677	12.88825	9.71912	1.25651
**Anapc5**	19.10361	9.75580	11.53681	12.43914	9.94559	11.56431	10.78173	22.45003	12.51562	26.42438	21.63343	10.03178	20.91777	11.56101	16.71008	11.84155
**Anapc7**	7.95882	6.47811	7.05723	7.70585	0.74964	3.53803	3.16906	11.54093	14.67052	19.08355	13.91794	1.63287	4.78979	0.42886	10.56976	1.81708
**Angel1**	0.03411	0.00752	1.25814	3.33185	0.01474	0.00888	0.02666	1.18538	0.98703	1.00467	0.27496	0.02544	1.47369	0.00000	0.41109	0.00544
**Angel2**	2.77703	3.27433	0.06779	19.50700	5.82730	3.20757	0.47303	0.20701	3.44586	4.18563	2.91310	0.28210	2.36461	7.38466	11.33995	1.16070
**Angpt2**	0.04377	0.03671	0.02758	0.00467	0.00808	0.00594	0.00689	0.07039	0.62882	0.22085	0.35593	0.00212	0.23750	0.51084	0.00000	0.00720
**Angptl1**	0.00888	0.00770	0.00860	0.00970	0.00946	0.00702	0.02744	0.16361	0.01211	0.19956	0.00903	0.00674	0.26279	0.32496	0.00000	0.00728
**Angptl2**	0.01626	0.00789	0.12045	7.35186	0.00901	0.00501	0.00701	0.01029	0.72531	0.01584	0.02217	0.11896	0.01068	0.00000	0.19512	0.00587
**Angptl4**	3.82644	0.06242	1.37711	0.67934	5.92904	0.00193	0.32859	3.12950	0.00000	3.28508	2.64397	0.26354	2.59536	3.08000	1.90164	0.02230
**Angptl6**	0.01610	0.00783	0.07007	0.16987	0.01126	0.00628	0.00745	1.11029	0.00837	0.63035	0.19239	0.00278	0.04439	0.00879	0.00000	0.00778
**Ank**	0.00000	3.61806	1.00400	1.45576	0.02429	11.92674	0.03161	2.11722	1.70217	0.48259	2.29317	0.05542	7.32188	0.00000	1.07863	3.93855
**Ank2**	0.00321	0.00478	0.63068	0.03937	3.08713	0.00554	1.84625	0.02575	0.02424	0.00588	0.02477	0.01288	0.00703	0.04416	0.01791	0.00689
**Ank3**	0.01717	0.00810	0.01720	0.01587	0.00907	0.00635	0.00769	0.01855	0.07878	0.02993	0.01183	0.00768	0.01235	0.00758	0.06933	0.00741
**Ankar**	0.01322	0.01761	0.01141	0.64820	0.00907	0.00635	0.00726	0.01034	0.26129	0.41001	0.01040	0.00559	0.01114	0.01599	0.47504	0.01403
**Ankdd1b**	0.02162	0.00767	0.91014	1.89172	0.31449	0.00899	0.53427	0.08630	0.25189	0.07570	0.66670	0.07498	0.10148	0.00874	0.54175	0.00719
**Ankfy1**	2.85652	7.98178	5.45657	5.12668	0.61939	4.46693	3.92344	4.75346	5.84599	7.85976	7.01378	7.46376	2.65154	4.72286	3.92855	4.49299
**Ankhd1**	2.00919	0.02290	2.76024	1.30333	0.01569	0.04486	1.86092	1.01449	1.43497	3.58391	0.33561	7.69689	2.94974	9.28361	5.25637	1.63754
**Ankib1**	2.20967	2.33071	5.94591	0.62758	6.27324	0.01614	1.18736	2.88822	1.93551	3.68724	1.72234	13.90517	1.70422	2.55038	2.69062	0.18870
**Ankle1**	1.07115	0.24382	1.25089	0.92975	0.02096	0.01752	0.00576	1.08010	0.98721	0.16614	0.96616	0.06166	0.50911	0.02975	0.35753	0.07081
**Ankle2**	2.90155	0.04948	5.64939	10.50101	2.72943	0.01922	1.31116	2.28366	12.03543	3.33500	3.63841	19.61701	2.10411	8.66114	11.80177	2.72895
**Ankmy2**	3.76367	5.81318	3.62902	0.81322	5.06062	0.00291	8.21732	8.50921	2.11312	6.79854	10.65798	1.28607	2.73642	3.67541	7.76796	1.70024
**Ankra2**	0.76776	0.04916	0.32679	4.30750	0.00753	0.00752	0.01644	0.00792	3.02716	0.40801	0.05397	0.00000	0.01061	0.68326	0.22818	0.00560
**Ankrd1**	0.00916	0.12256	0.01237	0.00953	0.00920	0.00700	0.00719	0.01149	0.01222	0.19894	0.01451	0.00653	0.56003	0.01040	0.00901	0.00777
**Ankrd10**	7.22560	2.50112	0.80771	21.28967	10.23219	1.79038	10.65385	15.63137	26.95759	42.27846	19.98957	22.32606	8.58094	13.69210	21.60613	0.07106
**Ankrd11**	5.96493	2.54970	2.40149	0.37549	0.02997	1.83107	6.26849	3.82604	0.01090	1.54313	0.07170	0.17296	0.32876	0.26745	1.57649	1.29154
**Ankrd12**	0.22286	0.00550	0.86570	1.00101	0.01413	0.00588	2.06531	0.71410	1.30169	2.51859	2.63555	0.83855	0.48008	10.72964	3.82198	1.03590
**Ankrd13a**	6.52414	0.99302	6.54573	7.20510	5.40565	5.61985	6.79477	14.38457	2.07755	9.17412	5.59880	1.00984	9.40550	0.08424	4.42075	4.50874
**Ankrd13b**	0.35781	0.00735	0.39163	0.05233	0.01615	0.00587	1.99945	0.04596	0.12133	0.00000	0.24253	0.00493	0.24201	0.01203	0.00000	0.00712
**Ankrd13c**	2.19258	1.72381	0.68982	16.20115	7.06651	2.76172	1.77772	3.76776	14.82621	12.70314	7.90087	14.15418	2.43026	14.48472	11.43630	0.08973
**Ankrd13d**	0.01028	0.00804	0.01232	0.08651	0.02712	0.00672	0.00732	0.00002	0.01095	0.06307	0.59551	0.03065	0.44480	0.00755	0.03102	0.00704
**Ankrd16**	1.94003	0.17452	0.00000	2.33610	0.00421	2.42170	3.09294	2.96283	0.62135	1.06404	1.03853	3.23875	0.22393	0.00000	0.92013	0.09741
**Ankrd17**	11.45629	9.16526	11.51920	16.09548	3.94510	5.42653	5.92776	8.81078	17.32295	18.61772	12.10707	16.81943	4.56121	23.49497	18.57937	5.08432
**Ankrd22**	0.01371	0.00830	0.01222	2.84663	0.00930	0.00637	0.00742	0.01387	0.32195	0.01240	0.01185	0.00788	0.01270	0.00959	0.00267	0.00692
**Ankrd23**	0.74077	0.00638	0.18948	2.17043	0.00599	0.00719	0.00629	1.16506	3.09542	0.81032	0.40655	0.01281	0.00379	0.00000	0.53095	0.00603
**Ankrd24**	1.11907	0.00561	0.29631	0.00489	0.00522	0.00639	1.01734	0.10862	0.28622	0.00803	0.02204	0.00541	0.00650	0.01486	0.00246	0.00600
**Ankrd26**	0.01291	0.00765	0.00511	0.51472	0.00911	0.00687	0.00716	0.00177	0.01770	0.69131	0.28062	0.02219	0.00969	0.44939	0.77882	0.00725
**Ankrd27**	5.76836	2.62860	1.67866	1.58805	0.00651	0.28510	0.42822	1.06429	0.53449	2.45508	0.91476	0.43600	2.73374	0.00170	1.36132	0.02642
**Ankrd28**	1.92465	1.17981	0.53794	3.68383	0.02977	1.30096	2.10394	0.10452	10.88980	1.85616	4.58800	0.20971	1.84232	5.50618	4.51675	0.04565

**Ankrd33b**	0.00477	0.00675	1.43787	0.56171	1.37759	0.00897	0.00706	0.01386	0.00000	0.40914	0.01063	0.03164	0.00582	0.00931	0.00000	1.56981
**Ankrd35**	0.01044	0.00770	0.00353	5.36643	0.00707	2.54932	0.00662	0.00316	8.03333	1.15212	0.07976	0.03048	0.21442	0.00001	0.28494	3.04868
**Ankrd36**	0.01373	0.00819	1.09656	0.75329	0.00742	0.00553	0.00738	0.01181	0.01834	0.01111	0.02880	0.03075	0.00764	0.01041	0.00850	0.00681
**Ankrd37**	0.00954	0.16724	0.11756	3.50124	0.00905	0.00585	0.00678	0.00000	2.26011	0.30471	1.07391	0.06056	0.34922	0.62966	0.00000	0.00648
**Ankrd39**	1.40236	0.01538	1.04640	0.50664	0.00349	0.01219	0.38046	1.98918	1.60884	0.29702	0.11656	0.04032	3.78303	0.00685	0.00000	0.00395
**Ankrd40**	4.44851	3.16959	3.01027	10.92263	7.05911	4.10703	1.90494	3.27752	12.91080	5.62713	5.00018	3.27197	2.60492	3.22666	5.72216	0.87406
**Ankrd42**	2.00809	0.00552	0.12557	2.64171	0.00408	0.00306	0.00488	0.00000	3.45699	4.83574	2.22352	0.26214	0.00000	6.30298	3.94834	0.00391
**Ankrd44**	0.00753	0.00785	3.33942	14.10311	0.00431	0.00994	0.15475	0.03867	6.74730	0.97382	2.65331	0.34247	0.03331	0.27194	12.51210	0.00322
**Ankrd45**	2.10390	0.35083	0.00151	0.11585	1.13698	0.00672	0.00607	0.28193	1.05786	0.00000	0.41483	0.01241	0.08807	0.05011	0.02293	0.01157
**Ankrd46**	3.34216	7.99616	5.68950	6.63938	1.32219	1.28936	2.69567	4.21530	1.36957	1.01136	3.82757	0.19647	2.15168	1.19382	1.99052	1.44535
**Ankrd49**	2.60927	2.10562	2.77473	0.33420	0.00000	0.00285	0.00449	2.06753	0.00000	1.69230	0.00000	0.08065	0.29146	1.19424	7.14941	1.94479
**Ankrd50**	0.01033	0.00713	0.67340	5.27323	0.23314	0.01489	0.00636	0.07139	2.85366	0.18250	1.05474	0.58818	0.58257	0.03138	0.77666	0.00588
**Ankrd52**	0.02058	0.00837	0.05547	0.56238	0.01496	0.00624	0.00762	1.45281	0.72912	0.67648	0.01391	0.18551	0.12552	0.00371	0.59559	0.00757
**Ankrd54**	7.39214	0.08413	3.83671	2.83439	2.03542	3.72364	0.06013	1.46352	6.38784	2.56833	1.38732	0.25684	1.29556	0.00637	0.99001	3.06291
**Ankrd6**	0.30260	0.00720	2.01269	12.80324	0.01435	0.00702	0.00555	0.74005	7.40745	2.42150	0.99899	0.73291	0.47445	0.10403	7.09842	0.00357
**Ankrd9**	0.01435	0.00760	0.03924	0.42587	0.01076	0.84376	0.00679	0.20723	0.65601	1.94917	0.64213	0.02548	0.14013	0.02727	0.03711	0.16705
**Anks1**	4.98271	1.40564	3.29680	0.74904	2.16804	1.03688	2.55273	3.17574	5.63400	0.52375	0.86446	4.12948	2.06989	0.00000	0.00000	0.00580
**Anks1b**	0.00845	0.00737	0.01082	0.00740	0.68053	0.00666	0.00766	0.01934	0.22111	0.78484	0.00998	0.01467	0.01052	0.01510	0.05235	0.00762
**Anks3**	0.00000	0.14706	1.94895	5.32305	5.10637	4.91989	0.00675	1.34763	3.09191	2.43540	0.40247	0.10817	0.18630	0.01040	0.20489	1.61440
**Anks4b**	0.00713	0.00756	0.01139	0.01287	0.00750	0.00634	0.00734	0.01227	0.05593	0.00000	0.01148	0.00737	0.00731	0.02438	0.20123	1.61445
**Ankzf1**	0.78075	0.00596	0.76887	0.68525	0.06589	2.60868	1.30905	1.15841	1.42635	1.23567	0.16502	0.05093	1.10742	0.09377	0.83890	0.00492
**Anln**	1.76751	6.11316	0.83283	10.09683	1.86319	2.46555	0.70285	8.48500	14.53313	2.16645	7.10778	14.38283	3.49106	20.53701	14.45188	0.44289
**Ano1**	0.01367	0.00786	0.01217	0.00928	0.00915	0.00665	0.00735	0.01139	0.01215	0.29872	0.01317	0.00672	0.01252	0.01131	0.01042	0.00781
**Ano10**	0.00913	0.37212	0.03253	0.15579	0.00975	0.00836	0.02842	0.01196	0.26318	0.00139	0.03120	0.03685	0.19339	0.00713	0.14825	0.00743
**Ano2**	0.01178	0.00796	0.01422	0.00475	0.00991	0.00715	0.00747	0.01247	0.22887	0.70254	0.01267	0.00485	0.28945	0.00956	0.01915	0.00810
**Ano6**	0.00000	0.00539	2.82066	1.58901	0.00622	0.27280	3.22293	1.51189	1.12728	2.22240	5.02977	0.17858	2.18939	0.05227	0.38284	0.00438
**Ano7**	0.00783	0.00836	0.03965	0.00962	0.00944	0.00849	0.00822	0.75421	0.01116	0.36545	0.00691	0.00613	0.00565	0.01838	0.03105	0.00776
**Ano9**	0.00000	0.00631	0.00000	0.76843	0.00935	3.93451	0.00278	7.31406	1.93306	1.85885	5.45199	0.00000	3.71966	1.78654	2.05505	0.00324
**Anp32a**	3.84483	0.00190	0.78635	0.00000	2.91168	2.64319	0.00426	0.95399	0.00806	1.87802	0.61525	0.24368	0.34279	0.01864	0.04306	0.13962
**Anp32b**	25.71570	12.64028	18.09366	18.95230	17.82715	2.34827	10.75050	18.49849	18.47327	26.21090	21.70939	9.45821	21.45191	18.62889	15.50097	10.78548
**Anp32e**	12.17589	10.38225	15.89116	12.28633	15.97310	5.56663	6.25403	9.55308	17.72067	11.41229	14.57804	5.08502	7.35913	14.89926	15.63083	4.72130
**Anpep**	0.22108	0.00724	0.09029	0.01229	0.00719	0.00546	0.39752	1.04130	0.12345	0.50946	0.44244	0.36981	0.00319	0.39023	0.57526	0.00689
**Antxr1**	0.00836	0.00766	0.00916	6.14389	0.00894	0.00563	0.00627	0.00760	1.92326	0.27482	0.01808	0.10150	1.25139	0.00022	0.47858	0.00540
**Antxr2**	0.01335	0.00791	0.01095	0.73275	0.00918	0.00632	0.00731	0.00999	0.00001	0.55417	0.02411	0.01494	0.01103	0.00610	0.88693	0.00709
**Anxa1**	0.01246	0.00783	0.01092	0.00570	0.00868	0.00672	0.00732	0.01051	0.01113	2.54516	0.00917	0.06502	0.01085	0.01288	0.00470	0.00741
**Anxa11**	0.18773	0.37786	0.47999	1.68534	0.14678	0.01612	1.01527	1.52549	2.57725	3.17416	7.47250	0.31982	1.17574	1.20340	0.00000	0.67329
**Anxa2**	0.00000	6.60215	3.80862	0.30578	0.11267	16.22802	2.53336	1.38334	0.23869	4.48896	0.21958	0.08368	8.21899	1.52861	0.89782	4.84536
**Anxa3**	0.00429	0.00625	0.41958	0.15138	0.04171	0.02642	0.00466	0.33784	0.00600	0.63503	4.16800	1.55894	1.23379	0.00088	0.00000	1.12423
**Anxa4**	0.21006	5.42279	3.85628	0.76397	1.52074	0.00583	0.12157	0.00000	0.00000	3.12884	1.00550	5.27856	0.74758	0.01994	0.39798	0.01713
**Anxa5**	1.86695	1.62117	0.00000	0.46730	0.00364	0.01148	0.00508	0.01411	0.00384	8.27452	0.00736	0.01340	0.00694	0.00391	0.23585	1.91327
**Anxa6**	9.40871	6.52767	0.00000	0.50318	0.44081	11.75653	9.35126	1.77268	1.31823	0.57328	2.36746	0.01927	4.73595	0.00000	0.33339	6.38132
**Anxa7**	18.68194	14.60303	17.72155	19.63886	11.69290	9.99448	11.20535	9.07560	5.12399	19.20186	7.21969	2.37007	15.92976	9.52075	16.11277	7.41813
**Anxa8**	0.01338	0.00773	0.01016	0.00399	0.01031	0.00633	0.00675	0.00976	0.01011	0.63870	0.00841	0.03283	0.97853	0.00513	1.05066	0.00699
**Anxa9**	3.58898	0.00338	2.82197	0.00000	0.00686	4.25297	0.00416	2.72011	0.97987	2.58457	0.54709	0.00000	0.07870	0.90347	0.63490	0.01589
**Aoc1**	0.01332	0.00778	0.01206	0.00781	0.00899	0.00657	0.00725	0.01049	0.01188	0.75932	0.01247	0.00549	0.02509	0.01155	0.00912	0.00774
**Aoc2**	0.21761	0.00756	2.99218	0.64234	0.17852	0.16654	0.07549	0.31585	0.55463	0.63024	1.53322	0.10470	0.82909	0.26151	0.39556	0.01252
**Aoc3**	0.01383	0.00772	0.00591	1.15758	0.00897	0.00702	0.00709	0.00000	0.97138	0.00000	0.72499	0.14467	0.00588	0.00271	0.02067	0.00679
**Aox2**	0.01263	0.00794	0.32025	0.00521	0.00876	0.01209	0.00757	0.01306	0.17988	0.00971	0.01018	0.00769	0.00973	0.01563	0.07970	0.00721
**Aox4**	0.00833	0.00788	0.30254	0.00000	0.00931	0.00558	0.00738	0.75452	0.86314	0.00145	0.00000	0.05915	0.00418	0.00619	2.16806	0.00708
**Ap1ar**	3.40136	2.88697	3.13980	5.29639	2.17350	4.70567	0.69627	2.37729	2.47232	3.34197	1.95214	3.99549	0.59952	7.58150	13.53295	3.54689
**Ap1b1**	4.30298	6.68344	8.75555	7.73297	2.29038	0.01553	0.65610	9.77631	5.26503	8.61762	4.63977	0.32983	7.43567	0.55059	4.37320	5.95826
**Ap1g1**	5.70331	9.62268	7.57847	7.63656	6.46047	9.87362	7.58582	13.22792	12.65988	10.53261	7.28681	4.40737	7.67798	13.66938	12.66601	1.69019
**Ap1g2**	0.01052	0.00789	0.12614	0.00557	0.00904	0.00595	0.00669	0.00193	0.01139	0.00289	0.41778	0.00521	1.28794	0.01128	0.00246	0.00651
**Ap1m1**	7.76045	3.90571	1.97684	7.42906	3.33292	9.28314	2.67489	2.33581	5.25813	12.44285	4.66756	4.50930	1.57881	0.00000	0.87693	2.16917
**Ap1m2**	4.33892	8.16497	10.36071	5.72559	6.96227	2.20053	5.15376	12.84831	3.93618	9.32026	6.26151	1.53299	9.10368	2.70960	5.49705	6.56448
**Ap1s1**	12.42116	3.08740	4.81328	4.27597	0.14766	14.67197	6.30391	19.02017	3.17180	15.35311	11.31600	1.40436	17.93236	0.02587	2.03699	8.48371
**Ap1s2**	1.33894	0.13199	0.00000	2.43562	0.00548	0.00513	0.00678	0.11223	0.93682	0.45361	0.00000	0.00000	0.01617	3.37610	0.00000	0.01258
**Ap1s3**	0.11844	0.00684	2.21625	0.38873	0.01276	0.00721	0.66762	1.92309	0.19632	4.31846	0.71247	0.25927	0.05365	11.89373	3.61362	0.01969
**Ap2a1**	2.56527	0.04632	0.00000	0.03895	0.81716	0.85681	0.59325	3.01461	0.00891	3.41284	0.00000	0.08200	3.69027	0.01205	0.00000	3.09372
**Ap2a2**	6.12033	4.21554	3.50244	8.09739	0.26122	15.90476	1.72484	19.28838	4.81711	10.63713	14.19048	2.70588	19.96672	1.61147	0.76468	6.28256
**Ap2b1**	4.45866	2.76461	2.23873	3.12058	6.40503	8.64382	0.03775	5.85507	0.35182	2.41320	6.83522	5.89410	6.62867	6.49121	4.23846	3.43822
**Ap2m1**	18.43964	18.58833	22.32865	8.12098	14.42270	17.91425	15.14460	24.21870	6.93658	18.03523	19.50348	7.78880	26.42286	12.01491	12.06899	11.89769

**Ap2s1**	11.37575	0.61700	0.00000	0.04004	9.42754	7.18757	0.62591	13.83972	0.00895	14.65529	0.50023	0.14068	13.33036	0.00245	0.00000	10.42847
**Ap3b1**	13.52232	7.77865	8.56169	3.30234	0.96564	7.32678	1.43794	8.25691	1.15319	7.80716	5.91828	7.51508	8.66592	7.78837	2.47139	5.11482
**Ap3b2**	0.50498	0.00797	0.49900	0.01690	0.00663	0.27576	0.00660	0.10751	0.01134	2.66949	3.09249	0.00000	0.28357	0.00193	0.69357	0.00623
**Ap3d1**	14.58658	9.65088	9.40026	6.63922	2.83544	10.86467	7.36523	12.89171	5.16817	14.25984	9.26746	10.39395	9.58576	9.98186	13.41721	4.63123
**Ap3m1**	5.76035	7.74948	4.56992	7.22580	0.27104	0.07298	6.67381	6.69829	1.72391	7.43266	3.00546	8.15466	2.88535	10.56751	4.77810	1.78960
**Ap3m2**	0.01583	0.00806	0.91616	0.83663	0.00877	0.20703	0.00739	0.00915	2.02778	0.59787	0.00000	0.00000	0.00688	0.10117	0.27939	0.00645
**Ap3s1**	0.90987	4.27279	0.44432	2.08207	3.36610	0.03293	0.75920	1.55528	2.13770	4.10347	1.34489	9.08018	0.79170	2.05677	0.52529	0.25546
**Ap3s2**	8.47314	6.96046	6.14664	2.92442	3.14181	2.41505	2.48992	10.08167	3.45697	10.49179	4.35887	0.26928	7.75221	1.79162	2.81044	3.96760
**Ap4b1**	4.97710	10.64718	3.13713	10.37033	2.28703	7.16015	3.07927	4.44603	9.80247	13.41851	5.97454	8.43016	2.86262	8.93116	15.22705	2.53068
**Ap4e1**	3.03283	1.76833	1.40101	3.20119	0.00000	0.02516	0.12670	0.62040	6.97903	1.48865	1.06293	1.76718	0.32832	2.28862	2.67239	0.86348
**Ap4m1**	0.25236	0.00736	0.54707	1.05765	0.01313	0.97274	1.69371	1.34534	0.69836	0.37170	1.04553	0.04809	1.86746	0.25013	3.02086	0.12989
**Ap4s1**	4.37540	0.01670	4.54062	5.45047	1.06991	1.38801	0.87367	8.17376	9.60298	13.32489	16.84482	10.82145	3.25363	8.98289	8.38123	0.00836
**Ap5b1**	0.01533	0.00765	0.01689	1.19494	0.01066	0.01212	0.00715	1.30306	0.38937	0.00000	0.01712	0.02459	0.00504	0.01256	0.00000	0.01442
**Ap5m1**	0.85929	0.41934	0.74688	1.60258	0.01498	2.30974	0.28969	1.25131	1.15875	1.75581	0.64289	3.93784	0.36087	3.83686	1.97108	1.40154
**Ap5s1**	4.00589	0.00000	6.78964	1.83702	0.00000	0.49793	1.59943	2.07554	1.60642	1.01504	0.45385	0.03448	3.16301	0.04688	0.97418	0.00624
**Ap5z1**	0.98086	0.00455	3.54570	0.67987	0.03404	3.70644	1.76971	1.90099	1.31674	2.91067	3.11292	0.31354	7.64393	0.62151	0.35959	2.89489
**Apaf1**	2.37028	0.00000	1.80501	8.53120	2.03822	2.55468	6.88944	1.86282	10.84114	13.29057	4.08963	8.52508	1.24435	3.00017	14.03470	3.14857
**Apba1**	0.01374	0.00791	0.01219	0.89241	0.00949	0.00669	0.00753	0.01380	0.01850	0.01336	0.01290	0.01806	0.01588	0.01656	0.02326	0.00734
**Apba2**	0.02089	0.00779	0.03204	0.41638	0.01776	0.00588	0.00734	0.01562	0.00416	0.36972	0.01062	0.00644	0.02868	0.11129	0.02827	0.01395
**Apba3**	2.29982	0.01474	0.00000	0.00536	0.94795	0.18867	0.02514	1.98145	0.24036	0.54020	0.71402	0.03723	1.90305	0.01631	0.01386	0.70495
**Apbb1**	1.65744	4.16174	0.00000	4.06123	4.53539	4.87495	2.51617	2.78904	0.00000	0.55879	2.16308	0.04143	1.61616	0.00742	0.14280	0.01520
**Apbb1ip**	0.04344	0.01885	0.11076	0.01064	0.00853	0.00616	0.00779	0.01470	0.01233	0.00880	0.01398	0.05095	0.18875	0.01305	0.00732	0.00744
**Apbb2**	0.09205	0.82594	1.57948	2.23186	0.06478	0.00513	3.72874	0.34780	2.35860	1.26301	2.41828	0.25003	0.00000	0.85725	2.86828	1.28595
**Apbb3**	0.85444	0.00675	0.07180	0.22075	0.00683	0.56831	0.32097	0.01920	0.51721	0.00083	0.01104	0.00569	1.39428	0.01460	0.00257	0.00580
**Apc**	2.31986	2.11932	1.53818	16.26738	5.10123	5.68456	2.83094	0.03199	8.93122	10.11359	2.89649	5.43831	0.36464	9.26069	16.25883	0.18104
**Apc2**	0.01376	0.00802	0.01228	3.59995	0.00934	0.00624	0.00734	0.01298	0.09948	0.00892	0.01305	0.00765	0.01262	0.00087	0.18135	0.00675
**Apcdd1**	0.54967	0.17571	0.86777	0.00916	0.09712	0.00498	0.20967	0.00727	0.01218	0.32895	0.01023	0.01627	0.00785	0.01299	0.00000	0.01314
**Apeh**	8.01205	0.80631	3.07610	0.48496	0.83431	0.01679	2.35357	2.98240	0.00000	2.93149	0.64707	0.02047	4.05142	0.00456	0.08384	4.00166
**Apela**	3.08018	1.54598	2.32141	0.00955	0.00000	0.00226	0.00378	0.00000	0.01240	0.00000	0.01105	0.00434	0.54471	0.01380	0.00000	0.00223
**Apex1**	26.69441	17.18034	21.33549	6.22799	15.14792	8.89455	14.09616	20.79215	20.26601	26.16683	20.70048	16.07404	21.51346	12.32858	21.15336	12.37516
**Apex2**	1.85380	0.02562	1.01169	2.66012	3.62037	0.23783	1.26063	2.07735	3.88393	2.78822	0.75632	0.35690	3.54545	2.81499	3.79583	2.76312
**Aph1a**	2.26086	0.19164	1.87293	1.69015	0.01381	0.01773	0.09870	3.42598	0.53416	2.23063	0.80509	0.06185	0.93564	1.80850	0.06078	0.57789
**Aph1b**	0.01220	0.00825	0.01331	0.38514	0.01025	0.00700	0.00756	0.01414	0.63117	0.03517	0.01049	0.00592	0.24260	0.09186	0.00000	0.00764
**Aph1c**	0.01704	0.04063	0.02080	0.22984	0.00951	0.00664	0.02871	0.01773	0.78386	0.00938	0.11258	0.00563	0.05994	0.04718	0.00159	0.03038
**Api5**	13.56267	9.20416	10.41388	3.83589	13.72083	8.26035	8.66310	14.70812	5.45768	18.24245	12.73855	11.46962	10.33833	16.35082	9.40992	2.44498
**Apip**	4.88976	4.40287	4.55343	2.62431	0.01025	0.00004	2.06357	3.22517	2.34601	0.89462	1.91217	0.27006	0.00000	0.78526	0.78398	2.79311
**Apitd1**	8.59855	0.05916	3.07771	12.87312	3.93148	0.00000	3.16730	5.55436	17.39534	7.65415	2.02299	0.61795	3.75689	0.09831	1.75998	0.11581
**Aplf**	5.17272	0.00000	0.33080	2.89414	3.66703	0.22064	0.08481	0.36374	1.77332	0.63873	2.38514	0.74887	2.46214	2.63063	1.45633	0.41384
**Aplp1**	0.01622	0.00778	0.01094	0.00459	0.00901	0.00657	0.00739	0.03814	0.43059	0.17911	0.00768	0.00625	0.01031	0.01494	0.08487	0.00711
**Aplp2**	9.87165	8.89898	12.21656	11.92264	8.58539	5.82650	7.74801	3.06729	14.89964	5.07388	4.31680	8.18573	6.72890	2.74419	7.58576	4.88320
**Apmap**	1.51820	3.03016	5.02813	4.54605	0.89239	0.10487	0.10809	1.83986	3.30683	2.39322	4.27041	0.73119	5.33823	1.63520	2.16390	2.04105
**Apoa1**	0.00000	0.00463	0.00000	0.00190	1.60705	4.25965	2.01499	7.04461	0.00668	0.00000	0.12779	0.00000	2.40418	0.01371	0.00000	0.00441
**Apoa1bp**	4.13395	0.06856	2.67820	4.99790	0.03360	5.12602	0.04078	1.85635	2.40155	2.70137	4.00843	0.02474	3.70438	0.18772	0.40160	1.49752
**Apoa4**	0.00545	0.00765	0.42780	0.00848	0.01809	0.00560	0.00677	0.69260	0.04042	0.00000	0.00800	0.00619	1.04844	0.01853	0.00000	0.00656
**Apoa5**	0.01375	0.00796	0.00799	1.67072	0.00897	0.00686	0.00732	0.00046	1.08447	0.26841	0.40371	0.03729	0.00906	0.00398	0.25920	0.00695
**Apobec1**	0.01347	0.00802	0.00686	0.63284	0.00893	0.00705	0.00690	0.00000	0.00000	0.63671	0.93729	0.00178	0.00518	0.02707	0.04813	0.00733
**Apobec2**	0.00687	0.00860	0.00816	0.02554	0.04499	0.00694	0.00833	0.46967	0.01006	0.68642	0.00735	0.00661	0.00558	0.01458	0.00206	0.00708
**Apobec3**	3.98014	0.00284	1.08991	0.56527	0.00000	0.00115	0.00287	6.85961	1.70858	3.94863	3.15203	0.05907	0.14938	6.70135	5.23654	0.03042
**Apobr**	0.76762	0.00658	0.82246	0.00649	0.02454	0.00562	0.00648	0.17481	0.10778	0.06182	0.01190	0.01437	0.75398	0.01439	0.00000	0.17409
**Apoc1**	5.24165	0.19292	3.67476	0.00000	7.25852	0.82008	0.80748	0.06672	1.04868	0.45658	8.38979	0.05230	2.19520	0.32379	0.00000	4.85904
**Apoc2**	0.01264	0.00784	0.02676	0.04998	0.00945	0.00604	0.00732	0.11552	0.00902	0.04816	0.01146	0.00743	0.00840	0.01086	0.07866	0.00742
**Apod**	2.80159	0.01568	0.01885	0.55330	0.00294	0.00482	0.00512	0.01916	0.02319	0.00257	0.22100	0.02421	0.01838	0.10605	0.06601	1.65845
**Apoe**	4.04749	0.02276	0.34932	3.27791	0.00553	0.00823	0.04191	0.00000	2.40086	5.45871	5.40221	0.17130	1.14742	0.01100	0.00000	13.39332
**Apof**	0.02601	0.00800	0.02723	0.53192	0.01768	0.01314	0.01804	0.04845	0.98239	0.05566	0.08191	0.04929	0.05140	0.07167	0.04458	0.01437
**Apoh**	0.01376	0.00800	0.01115	0.00444	0.00933	0.00669	0.00747	0.01276	0.52102	0.01086	0.00854	0.05885	0.01300	0.01376	0.00519	0.00741
**Apol10a**	0.01282	0.00779	0.01213	0.00737	0.00906	0.00654	0.00722	0.00984	0.01184	0.96978	0.01163	0.00524	0.09169	0.01200	0.00764	0.00739
**Apol6**	0.02600	0.01857	0.07353	0.56311	0.00845	0.02904	0.02931	0.01083	0.24234	0.06900	0.20876	0.01698	0.01072	0.16204	0.07819	0.03589
**Apol7a**	0.01254	0.00844	0.02453	0.01014	0.00996	0.00666	0.00807	0.02745	0.01231	0.03221	0.01471	0.00868	0.04344	0.01075	0.04036	0.00758
**Apol7b**	0.00000	0.00837	1.27240	3.94417	0.00752	0.00519	0.04332	0.38652	4.77120	2.10224	0.44243	0.11339	0.90495	0.31730	2.12352	0.01297
**Apol7c**	0.01247	0.00841	0.01133	0.01039	0.00941	0.00677	0.00802	0.04468	0.01227	0.01736	0.01220	0.02014	0.02626	0.01025	0.04727	0.00740
**Apol7e**	0.00431	0.00826	0.86159	3.60521	0.00794	0.00515	0.12264	0.17081	5.23317	1.55238	0.38058	0.12627	0.66369	0.25283	1.36329	0.02045
**Apom**	0.46912	0.00777	0.04409	5.88526	0.00668	0.00352	0.00695	0.21097	12.70721	0.66393	2.09430	0.37915	0.00000	1.95420	0.93397	0.39738

**Apon**	0.01371	0.00741	0.00638	0.05366	0.01028	0.00662	0.00714	0.87730	1.66936	0.00000	0.00000	0.00189	0.01131	0.02227	0.44874	0.14811
**Apoo**	1.63037	2.05315	2.10504	1.02844	0.00587	0.84735	1.17825	1.05723	0.96581	1.97813	1.84611	2.95546	1.14808	0.63021	0.77000	0.68383
**Apool**	5.56133	3.12536	4.07151	0.01057	0.00000	2.31039	2.46393	0.73886	0.07833	1.63153	2.17148	0.07186	4.43505	2.62350	0.20942	4.15156
**Apopt1**	0.80845	0.10485	1.41777	1.75080	0.44217	1.93286	0.04681	0.61091	3.29216	0.79316	1.33237	0.24686	1.25910	1.69121	0.90210	0.05458
**App**	0.02297	1.58319	1.06551	0.58348	0.01416	0.00956	0.00737	0.63316	0.00361	1.54521	0.02039	0.10536	0.00865	0.00461	0.00000	0.00626
**Appbp2**	9.07431	5.28638	7.15163	2.03878	3.67749	0.56889	8.22792	7.14289	2.98084	6.57799	2.43701	3.88192	0.71233	12.87248	11.04250	2.08402
**Appbp2os**	0.00666	0.00706	1.09991	0.04186	0.55415	0.21574	0.54063	0.00984	0.01132	0.00904	0.01258	0.00648	0.01374	0.01438	0.00000	0.00693
**Appl1**	6.14017	2.71874	6.22180	7.22980	1.82665	0.42990	5.31722	3.30590	1.28904	6.69363	5.12191	3.40534	2.79877	8.61084	6.75755	2.15120
**Appl2**	2.01045	1.14559	1.25238	0.00000	0.43409	2.84125	1.76717	0.07329	0.50481	0.21381	0.00000	0.09816	0.00088	0.01431	0.01274	2.38694
**Aprt**	21.04087	12.03691	27.37216	15.67373	5.76422	12.08313	13.20986	36.19870	19.05272	36.55109	32.23144	11.36597	30.25587	0.55748	7.18593	10.13140
**Aptx**	5.25470	2.01365	7.00342	2.15052	0.22801	0.04739	2.49303	9.88327	1.58812	6.22201	4.77998	7.11630	6.16544	2.62430	5.05229	0.01614
**Aqp11**	0.13888	0.00754	0.00000	3.91777	0.00720	0.00635	0.01756	2.51659	1.03266	5.65978	0.68536	0.02133	0.00249	0.00000	2.17226	0.00642
**Aqp3**	2.42309	2.88486	0.98803	0.03435	1.58909	0.10026	1.47717	2.27321	0.00703	0.00000	7.81278	0.03905	1.08778	2.04855	0.00000	0.16098
**Aqp7**	0.05320	0.01885	0.08177	0.54153	0.02254	0.01332	0.01737	0.00000	0.79766	0.06877	0.47026	0.03231	0.11531	0.45788	0.00990	0.02200
**Aqp8**	12.39872	4.92242	0.00000	0.00665	14.39985	0.00000	0.00530	0.00000	0.00854	0.00000	0.00922	0.06905	0.02646	0.00635	0.01397	8.05361
**Aqp9**	2.31151	2.89540	7.10037	9.17260	0.02480	5.64611	5.66089	4.87717	9.31960	13.76619	11.78427	14.26270	5.99828	18.18264	16.75592	0.02775
**Aqr**	7.65237	3.78402	5.42281	3.50048	3.84153	5.02318	1.56295	6.74788	6.42693	6.55953	4.52369	5.39143	4.42313	0.41601	6.30586	0.01189
**Araf**	6.97372	6.82536	10.05239	3.76069	4.23329	7.77346	3.92566	6.94049	5.08958	6.86195	6.25067	1.01495	7.22962	5.65347	7.20989	3.16968
**Arap1**	0.31307	0.30344	1.25826	0.05572	0.00757	0.00893	0.00507	1.85909	0.47318	1.17711	3.70207	0.09267	1.16332	0.32539	2.19161	0.01307
**Arap2**	0.00760	2.94228	0.17326	11.06089	0.43919	0.00494	0.00547	0.37526	3.44041	1.06791	0.72524	1.67136	0.03291	0.20264	13.79198	0.00401
**Arcn1**	5.59546	3.41975	4.88570	8.16651	12.96804	6.60258	6.26623	6.00450	14.97395	6.83188	5.85369	6.49180	3.50846	10.06287	9.34161	4.02037
**Arel1**	2.48874	0.20240	1.98259	6.75704	0.02784	3.15677	1.90825	1.91687	3.88462	2.75130	0.37941	10.80652	2.61158	4.54489	5.42829	0.02904
**Arf1**	29.91912	17.80612	23.53075	13.54422	22.83018	18.47370	15.98339	30.38102	11.23569	29.03773	13.08122	8.00017	30.50475	0.57879	3.48949	16.97730
**Arf2**	1.72719	3.30351	7.17511	6.58479	0.02260	7.49148	8.48741	6.54043	12.86798	7.03029	4.04643	1.10361	1.14059	0.44449	4.78673	3.94099
**Arf3**	16.08508	10.22551	11.38994	6.19854	3.61893	4.08550	6.20649	10.41542	5.42347	11.29717	3.39542	1.10453	5.56353	6.42241	5.75217	7.12223
**Arf4**	12.57783	9.25494	16.43065	6.28566	14.83063	8.39534	7.50556	12.23198	5.38330	11.35636	9.18803	5.05162	15.25056	7.55340	4.61033	11.68959
**Arf5**	3.95688	0.04435	0.00000	0.10553	0.02274	1.21233	0.80920	2.61128	0.00732	0.88263	0.02313	0.00000	1.42286	0.01694	0.00942	5.76465
**Arf6**	7.40795	4.52214	4.35406	13.75039	2.93196	1.99890	6.08961	4.19908	17.63738	9.45521	10.42892	0.44811	4.83773	1.40758	4.62074	3.19152
**Arfgap1**	6.76629	2.64168	7.52541	2.78338	3.42075	4.27492	8.76229	10.45566	3.03194	6.27395	3.87107	0.45742	8.91302	0.44898	3.30560	8.14177
**Arfgap2**	0.14551	0.75624	0.55699	0.00168	0.63025	0.68770	0.03886	1.71017	0.01922	0.04532	0.30252	0.00236	1.33220	0.01168	0.05535	0.61768
**Arfgap3**	0.88936	2.69338	0.00000	1.56582	0.00640	4.85328	0.00518	0.00496	0.96722	0.90634	0.00000	0.02856	0.01513	0.07099	0.00000	1.11198
**Arfgef1**	6.76443	0.06449	1.31056	16.30171	0.49557	1.77552	2.55062	0.00000	10.20681	5.21117	0.71391	0.78494	1.69124	9.65867	17.86545	1.36384
**Arfgef2**	6.03952	2.25831	4.49918	7.20263	2.14899	3.88211	5.21415	4.55529	7.66589	7.61900	2.80633	0.77597	2.88999	0.38019	2.75200	0.00000
**Arfgef3**	0.01399	0.00771	0.00953	3.13301	0.00915	0.00633	0.00722	0.00343	0.00000	0.00000	0.31378	0.00509	0.00901	0.00052	0.45288	0.00676
**Arfip1**	3.62524	0.06859	4.51478	13.52542	1.64143	1.30346	2.20487	0.62526	1.92613	1.49423	1.91673	2.51628	1.98498	2.11976	15.09612	1.48764
**Arfip2**	2.53659	2.91392	2.07912	1.15679	5.04289	6.03263	0.64624	3.90298	0.00000	1.29970	1.78543	0.11059	3.24954	0.00000	0.53265	5.55615
**Arfrp1**	4.02152	3.37554	7.82450	10.35429	0.03923	3.65520	0.51254	6.77820	13.26731	8.91912	9.33513	4.43677	8.28609	1.42937	7.12316	1.03825
**Arg1**	0.00000	0.00745	0.64376	3.22117	0.00768	0.00476	0.00568	0.00000	2.41717	2.87078	4.03732	2.77081	0.69942	1.79476	7.37241	0.00534
**Arg2**	0.23311	1.31768	1.46033	2.85394	0.03158	0.00853	0.00691	0.14230	2.52211	0.32057	0.32576	0.06433	0.00000	2.51146	0.92393	0.00571
**Arglu1**	8.10296	0.51875	7.30807	12.71636	5.97251	2.17775	2.74395	4.53272	20.37296	7.74370	7.14820	0.87833	4.38885	9.42696	6.36560	7.00670
**Arhgap1**	10.81961	7.32369	12.29497	8.37609	1.83464	12.19384	6.63543	12.51960	6.31519	14.01153	14.85994	3.49659	12.44499	6.26666	15.13807	1.03211
**Arhgap10**	0.52622	0.01775	0.48952	0.57948	0.02060	0.00514	0.31848	1.08270	0.72121	1.41683	1.34023	0.06428	0.06069	1.36914	0.79145	0.55537
**Arhgap11a**	3.83291	2.88735	1.10290	18.59968	2.82455	4.58175	1.32503	5.62692	24.62558	9.96253	4.69862	3.59029	5.95011	7.69938	19.14130	2.51376
**Arhgap12**	0.17320	0.21215	2.42921	11.54981	3.57694	0.23868	4.79500	1.69157	7.70896	2.93051	1.73499	1.35910	0.06564	3.02315	10.71984	0.01024
**Arhgap15**	0.05774	0.00736	0.00802	0.06668	0.00810	0.00633	0.00732	0.00965	0.59383	0.68739	0.00425	0.00156	0.01132	0.00684	1.32547	0.00718
**Arhgap17**	4.65487	0.43796	0.00000	3.19103	4.37303	2.66605	2.69300	3.52761	0.83579	0.81503	1.38216	0.15560	2.35210	0.01393	0.00000	5.08917
**Arhgap18**	0.00000	0.00428	0.41030	0.68870	0.00768	3.09104	0.00000	6.94552	0.66429	7.58534	12.57469	4.60890	3.99166	13.90983	3.32106	0.00148
**Arhgap19**	3.77036	4.81640	1.44166	1.58873	2.11410	3.99028	1.67373	3.70752	1.38858	0.38348	3.02882	3.07177	0.34440	7.35285	8.05134	0.00542
**Arhgap20**	0.01311	0.00860	0.01157	0.32889	0.00929	0.00685	0.00792	0.02694	0.00540	0.07199	0.01166	0.00988	0.01234	0.00695	0.17237	0.00736
**Arhgap20os**	0.06109	0.01903	0.01957	8.48662	0.04490	0.11412	0.00687	0.04077	1.58694	0.70344	0.27972	0.02002	0.04977	0.00750	0.71010	0.00551
**Arhgap21**	2.85009	0.15458	2.49592	18.10151	0.56626	4.13509	6.86081	12.94140	8.36769	19.22172	9.53539	19.68359	2.67535	7.87120	3.98134	0.25603
**Arhgap22**	0.73827	0.00767	0.08777	0.43210	0.03597	0.00786	0.00675	0.01013	0.33424	0.01726	0.01005	0.00611	0.01596	0.00659	0.06733	0.74023
**Arhgap23**	0.26492	0.00809	0.00327	0.27038	0.00724	0.00783	0.00787	0.19891	2.65313	0.26789	0.00000	0.12924	0.00745	0.01131	0.00000	0.00686
**Arhgap26**	1.00084	0.00792	0.54149	0.20822	0.01385	0.00871	0.00704	0.03153	0.16227	0.43886	0.18277	0.07981	0.01386	0.00512	0.75160	0.00625
**Arhgap27**	0.00814	0.00840	0.09202	0.09875	0.00964	0.00887	0.00817	0.50869	0.04529	0.08576	0.00901	0.00730	0.00554	0.01807	0.00873	0.00741
**Arhgap28**	0.01265	0.00784	0.00342	3.56182	0.00883	0.00641	0.00690	0.00000	0.80773	0.94294	0.80583	0.02970	0.01268	0.00000	1.61527	0.01364
**Arhgap29**	0.70773	0.00248	1.93012	10.00344	0.48769	0.44247	4.85466	1.64735	13.93968	5.58298	4.81345	3.32290	0.09568	7.48225	13.93394	1.03948
**Arhgap30**	0.01511	0.01848	0.00697	0.02353	0.00890	0.00637	0.02696	0.01946	0.01039	0.46821	0.00000	0.01869	0.03728	1.24505	1.06840	0.01500
**Arhgap31**	0.08243	0.00864	0.00017	0.16345	0.01513	3.30812	0.00725	0.01094	0.00882	0.02242	0.00871	0.03267	0.02303	0.01134	0.00829	0.00620
**Arhgap32**	0.00000	0.87214	1.88799	0.94112	0.00609	0.00934	1.68804	3.09315	0.40047	2.88885	1.48208	0.07873	0.00000	1.94352	0.41498	0.00603
**Arhgap33**	0.01187	0.02792	0.01626	0.89539	0.00867	0.00716	0.07698	0.00688	0.94593	1.62339	0.09327	0.04763	0.01043	0.00340	0.12107	0.00725
**Arhgap35**	3.52747	5.14774	4.26336	8.07476	5.25834	3.05227	6.13546	5.98162	6.58861	5.31602	4.97597	0.08885	4.87558	6.14294	4.56274	0.97025

**Arhgap39**	0.18784	0.00794	0.32355	4.24951	0.11325	0.02360	0.00710	0.00857	1.50349	0.31946	0.01866	0.01317	0.01712	0.00000	0.38139	0.00639
**Arhgap4**	0.35907	0.00787	0.00271	0.35492	0.01273	0.00831	0.00700	0.31730	0.54415	0.42010	0.00633	0.00592	0.00682	0.00902	0.08791	0.00707
**Arhgap42**	0.23687	0.01757	0.88860	4.12839	1.20441	0.00472	0.00713	2.75395	3.22634	4.87746	1.02919	1.36757	0.16167	1.28311	4.09108	0.01295
**Arhgap44**	0.07104	0.00751	0.14128	0.67462	0.00654	0.00655	0.00698	0.01013	0.77725	1.20915	0.00613	0.01094	0.01459	0.00523	0.07890	4.36772
**Arhgap5**	0.87613	4.10219	1.89132	1.31825	0.09399	2.42596	0.47201	1.23973	1.23057	0.00000	0.74745	0.00000	0.36282	14.33224	0.65684	0.08781
**Arhgap8**	5.51267	0.00954	0.97328	1.64067	0.86336	6.83800	1.25677	5.29593	2.74827	1.59188	2.26892	0.00893	5.64885	0.00000	0.00000	0.00960
**Arhgap9**	1.51311	0.01583	0.24242	0.05812	0.01621	0.14206	1.65718	1.29868	0.00989	0.57145	0.22524	0.02918	1.24225	0.01396	0.00208	0.01178
**Arhgdia**	21.96092	3.75664	8.55276	9.98699	16.63810	10.04189	6.77298	23.31804	3.70997	17.31270	9.16806	1.12631	22.52911	0.01847	0.18195	13.39042
**Arhgdib**	0.03661	0.00836	0.00869	0.00625	0.01806	1.37724	0.00772	0.02881	0.01205	0.77939	0.00986	0.01709	0.01843	0.01250	0.00855	0.03750
**Arhgdig**	0.29351	0.01619	2.17723	4.91922	0.02055	2.80363	0.10504	5.12658	2.11394	5.01347	6.84542	0.28990	0.33083	0.01738	0.56202	0.00303
**Arhgef1**	3.98840	0.80196	0.29102	1.09469	0.56047	4.24948	1.66708	6.29892	0.04304	1.42077	2.78104	0.38693	0.48181	0.01861	0.00000	0.51146
**Arhgef10**	0.02430	0.00775	0.16010	0.01760	0.00825	0.00885	0.00726	0.01253	0.01258	0.08618	0.01257	0.02627	0.01086	0.01205	0.00644	0.00733
**Arhgef10l**	0.01829	0.00771	0.00899	0.34052	0.00883	0.00677	0.00721	0.06251	0.46195	0.00022	0.19120	0.00585	0.00823	0.00946	0.02558	0.00721
**Arhgef11**	0.21255	0.00827	0.00531	0.58847	0.01698	0.00679	0.00707	0.00025	0.02657	0.20836	0.33556	0.00404	0.00886	0.00678	0.54918	0.00727
**Arhgef12**	5.11861	1.74631	1.10240	13.45385	6.92816	0.31098	3.66830	4.81465	13.58721	10.52763	2.54889	2.21138	1.72791	7.33333	12.97341	2.98383
**Arhgef15**	0.18485	0.00794	0.00690	0.00457	0.00746	0.00719	0.00743	0.01520	0.37812	0.05109	0.03819	0.00821	0.01911	0.01208	0.01018	0.00708
**Arhgef16**	0.02169	2.28532	0.74084	1.74848	0.03296	2.70959	1.28367	20.47257	1.56078	13.35839	5.76471	2.63102	3.67013	0.07100	0.00000	0.00969
**Arhgef17**	1.08164	0.00595	0.00288	0.00852	0.00587	0.00718	0.48821	0.00594	0.01215	0.45440	0.01189	0.05124	0.01104	0.01174	0.00703	0.00624
**Arhgef18**	4.13172	0.60133	2.25637	3.31961	0.00000	0.16662	0.00231	1.61957	1.29842	1.01117	0.95066	0.01883	2.42555	0.00436	1.98351	0.02920
**Arhgef19**	2.08686	0.00430	0.20522	0.96211	0.13878	0.00697	0.08462	4.65665	0.00000	2.39431	3.14276	0.05870	0.02021	0.00000	0.00000	0.00446
**Arhgef2**	0.25952	0.00676	2.54138	1.51109	0.00298	0.00714	1.81068	0.26084	1.88568	0.86699	0.00000	0.05332	0.07715	0.00812	0.00000	0.02007
**Arhgef26**	0.00439	0.00717	0.00000	3.24058	0.00910	5.54154	0.00567	1.76236	1.34788	1.36266	1.88952	0.46338	1.19518	0.00000	1.65801	0.00425
**Arhgef28**	0.01362	0.00826	0.01201	0.61518	0.00930	0.00656	0.00752	0.01391	0.82822	0.01104	0.00760	0.00705	0.01289	0.01154	0.00332	0.00727
**Arhgef3**	2.77289	2.58498	2.42371	2.32125	0.05251	0.41782	3.85980	6.45786	1.43753	14.92582	10.01905	17.00257	1.95291	12.43970	12.21543	0.01258
**Arhgef39**	2.20184	0.00000	4.87256	11.60709	3.29801	0.01886	2.12483	10.53037	8.17343	2.72031	4.79935	0.00000	5.15978	0.02475	0.45767	0.01150
**Arhgef4**	0.01459	0.00749	0.40586	0.67466	0.00826	0.00556	0.00720	0.81488	1.79979	0.67872	0.45826	0.00000	0.00479	0.00187	0.14192	0.02297
**Arhgef40**	0.01373	0.00802	0.01190	0.07675	0.00922	0.00659	0.00750	0.01264	0.27414	0.10034	0.01096	0.01726	0.01218	0.01281	0.01154	0.00730
**Arhgef5**	0.08590	0.00548	0.69328	0.00049	0.02478	1.21702	3.96744	0.00000	0.01097	0.00000	0.50686	0.02170	0.00000	0.01217	0.00000	3.09052
**Arhgef7**	0.48489	0.80181	1.77560	2.06929	0.14537	5.75537	0.01443	1.99713	4.76436	0.93475	1.03490	5.80446	1.70690	0.00000	1.66582	0.89214
**Arid1a**	12.15821	5.43943	13.00482	15.34420	5.79557	12.13387	7.12446	19.23040	12.95213	28.33534	15.89032	14.93082	7.84205	16.38919	19.83707	9.77308
**Arid1b**	1.00105	4.08139	1.19748	3.20738	2.35673	0.00589	0.01369	6.59464	2.94510	2.55606	2.80906	0.60141	0.99206	0.31807	1.79844	0.17325
**Arid2**	4.78211	0.42852	4.10121	11.00958	4.33302	0.31469	2.91362	5.20002	4.71157	6.19226	5.57230	1.21205	2.35369	2.59740	7.25711	5.34811
**Arid3a**	1.39662	0.00690	0.11003	1.01442	0.00664	0.00421	0.00674	0.00651	0.00000	0.00600	0.30862	0.00664	0.00761	0.00881	0.00000	0.23095
**Arid3b**	8.52441	3.01118	3.40502	1.04154	1.47086	0.83879	2.92998	3.16883	0.36689	5.08094	0.61933	0.61011	5.09676	0.00278	1.59775	0.01494
**Arid3c**	0.01097	0.00821	0.19777	1.97138	0.00886	0.00543	0.00785	0.07008	0.29407	0.43450	0.00807	0.17208	0.24043	0.00873	0.17906	0.00657
**Arid4a**	0.37762	0.00574	0.04922	1.90099	2.49146	0.02767	0.37599	0.00000	3.63727	2.11512	0.23657	0.13343	0.00645	0.00000	1.09491	1.32739
**Arid4b**	1.62919	2.33772	0.14577	2.59810	0.66015	0.18285	0.01473	1.80840	7.68757	0.39387	0.11075	0.27576	1.05304	6.01576	5.64114	0.66561
**Arid5a**	0.02479	0.00778	0.01743	6.31246	0.00915	0.00504	0.05877	1.34296	12.79023	0.00000	0.00000	0.02886	0.36408	0.05333	0.01153	0.00553
**Arid5b**	3.13770	5.70556	1.84754	0.86336	1.18465	0.00338	1.41085	0.51134	0.00000	0.05215	0.00000	0.02839	0.00000	1.29990	0.58736	0.00000
**Arih1**	5.86296	10.38118	6.03336	14.15967	5.98061	0.00000	4.86590	6.33563	20.37144	8.01515	4.76642	12.12944	2.99101	4.28500	14.62941	2.36466
**Arih2**	15.19605	11.16569	19.80546	8.48890	11.72425	10.50467	5.19117	34.37384	13.98759	39.25248	26.47051	20.62329	18.72700	9.03588	19.80880	6.46623
**Arl1**	10.20531	2.71427	4.73906	2.43935	6.69607	6.90859	1.07832	7.48796	1.58383	4.44462	1.57973	0.96493	9.34296	3.88710	0.00000	4.75439
**Arl10**	0.02580	0.00786	0.01070	0.03304	0.00846	0.00702	0.00756	0.02318	0.00997	0.18615	0.07002	0.00805	0.01259	0.00828	0.03602	0.11645
**Arl13b**	0.53238	0.00654	1.04098	0.28038	0.01570	2.27356	0.00621	0.51697	0.59649	0.17367	0.17501	2.08199	0.91846	0.00457	0.39204	0.01251
**Arl14ep**	1.17870	3.01897	3.91461	10.37579	0.01588	0.00203	0.01246	2.36818	15.65335	4.53271	5.04659	0.49997	0.60455	0.14949	3.82923	0.11333
**Arl14epl**	6.72387	4.80485	5.22929	0.57878	0.20650	0.46341	3.27087	0.00000	0.03785	3.87433	4.48939	0.09769	4.01859	0.79979	0.85619	0.01569
**Arl15**	0.25051	0.02821	1.06057	3.71562	0.03208	0.15056	0.00575	2.02311	6.85465	0.00000	2.31722	0.07268	0.08358	0.15094	1.29437	0.26879
**Arl16**	0.01536	0.36857	0.84776	1.72887	2.43894	0.00554	0.15027	1.94485	3.49535	0.04063	0.40867	0.00604	0.06618	1.90992	0.09252	0.00609
**Arl2**	10.60564	3.77287	5.80376	0.55403	4.83402	3.77809	2.62946	2.94225	0.02391	1.13995	0.15484	0.00000	3.21100	0.00108	0.00000	2.89900
**Arl2bp**	0.04014	0.00729	0.05671	7.45423	0.00813	0.01624	0.01637	0.00000	4.58512	0.51833	0.79942	0.00000	0.00580	0.11882	0.65295	0.01344
**Arl3**	1.90001	0.01655	2.93628	8.97727	0.01199	1.09213	0.00467	1.14165	2.80733	1.91516	1.25415	0.09738	0.18578	0.00000	0.85123	0.06689
**Arl4a**	5.95336	0.00000	1.01959	12.83035	1.45499	2.06730	0.00000	0.91206	6.35491	0.65697	3.05869	0.12625	0.41306	1.22503	8.32641	0.01827
**Arl4c**	0.03628	0.00844	0.01070	0.17268	0.01291	0.00666	0.00735	0.01086	0.00863	1.13055	0.01209	0.00521	0.01100	0.01096	0.00580	0.00750
**Arl4d**	2.66645	0.03711	0.10327	0.16422	0.02865	0.00291	0.72366	0.00585	0.19860	1.90552	0.00971	0.00000	0.01713	0.00549	0.20730	0.00469
**Arl5a**	8.90701	3.86868	2.41799	6.80293	7.95634	1.83851	0.63967	2.53063	3.55879	4.59817	1.62000	7.10761	4.75716	0.62605	1.26440	0.00532
**Arl5b**	0.89629	0.00729	0.10174	1.54098	0.00706	0.00534	0.01855	0.35280	0.56587	0.11836	0.00697	0.03504	0.13664	0.00243	0.44943	0.01406
**Arl5c**	0.01322	0.00800	0.01267	0.11115	0.00932	0.00669	0.00749	0.01090	0.06354	0.82733	0.02898	0.00553	0.01679	0.03110	0.00728	0.00725
**Arl6**	4.12330	0.00286	0.00000	1.63425	1.81047	0.00921	0.00342	0.23116	0.33964	0.81330	0.00756	0.09486	0.83865	1.39137	0.78012	0.00922
**Arl6ip1**	19.20366	8.66462	19.93326	5.77703	10.23441	4.18262	11.90050	20.84228	2.20116	15.64605	14.99114	6.99004	12.43591	18.49188	19.58523	7.88827
**Arl6ip4**	1.79196	3.31794	2.76318	2.56269	15.08971	1.08818	0.93515	6.06349	5.11903	10.77338	5.00570	0.56592	9.03962	0.00588	1.46672	4.60930
**Arl6ip5**	0.00000	1.51731	0.01876	0.00153	0.00756	0.45384	0.00668	0.00914	0.13193	0.00763	0.01095	0.02627	0.00659	0.01153	0.00000	1.09602
**Arl6ip6**	1.73820	0.25936	0.39422	3.48140	0.04487	0.70951	0.00420	1.47920	0.00000	2.19713	0.66764	6.52968	0.86586	1.07924	4.75270	1.24680

**Arl8a**	0.97580	3.32496	0.03286	1.41676	2.06623	0.43589	1.66220	0.00496	0.73908	0.36155	0.00548	0.03560	0.93675	0.00147	0.78355	1.57649
**Arl8b**	9.15318	11.42095	12.93118	8.81792	10.94013	7.26485	9.21930	14.39923	9.68201	18.76874	15.93012	18.93512	10.67080	20.35150	15.10454	5.08910
**Armc1**	7.88952	3.74505	7.94392	7.78782	7.31675	2.83483	1.39380	6.22440	5.17887	8.63539	3.04338	8.71412	3.49578	2.93048	5.92733	1.77133
**Armc10**	4.43351	0.09578	1.15157	2.06220	0.10953	2.53995	0.15444	4.43321	1.02682	2.53931	2.65583	0.12056	3.23226	0.05358	1.35215	2.61920
**Armc12**	0.01379	0.00802	0.00865	0.89380	0.00907	0.00671	0.00750	0.00461	0.59550	0.00000	0.48666	0.00455	0.00756	0.00402	0.10744	0.00696
**Armc2**	0.01213	0.02946	0.01044	0.98288	0.00917	0.00643	0.14069	0.01341	1.69770	0.66122	0.00000	0.00237	0.01304	0.01525	0.19198	0.00733
**Armc5**	1.97954	0.00505	0.00000	0.55472	0.00417	0.83972	0.01565	3.49448	1.42094	1.91548	0.77924	0.05397	1.56585	0.02153	0.00000	0.00418
**Armc6**	2.56903	0.39893	3.13166	2.22548	0.84939	3.45654	2.16259	13.33920	0.22589	6.24896	1.57272	0.25592	3.51191	0.01613	0.11290	0.02190
**Armc7**	0.00000	0.00660	0.21215	6.01356	0.02464	0.00617	0.91364	1.60086	13.34659	5.05305	1.88576	0.66125	1.48298	2.65345	3.64745	1.16059
**Armc8**	8.34482	3.92273	2.07875	0.95900	6.71732	6.43574	2.87398	4.00934	1.00254	2.39917	3.73670	4.09928	2.52102	2.41293	6.03809	0.03605
**Armc9**	1.88814	0.23684	0.97819	0.38587	0.00758	0.00788	0.04676	0.32299	0.95844	0.33839	0.00000	0.03034	0.63096	0.00422	1.01659	0.00540
**Armcx4**	0.15858	0.08198	0.22025	0.48134	0.05688	0.07231	0.06786	0.08661	0.48576	0.14583	1.51539	0.01478	0.15688	0.71273	0.67537	0.07092
**Armcx5**	0.00000	3.14586	0.00000	12.19018	3.49759	0.76410	0.00486	0.91151	6.42683	1.32564	0.90337	0.42331	0.56164	4.73444	7.43616	0.44439
**Armt1**	0.81365	0.00209	6.32642	0.52455	5.47262	0.01335	2.53917	0.00000	0.86056	0.19077	0.22323	0.06436	2.75324	0.00432	0.65044	0.02556
**Arnt**	0.45799	0.12183	0.72009	3.62873	0.57007	0.06419	0.42936	1.64620	2.42745	6.92005	3.53439	6.99370	2.58665	6.28991	1.80142	2.43122
**Arntl**	4.18778	0.00000	0.17622	8.09259	7.27630	0.00000	0.00327	1.07399	6.18125	0.79414	0.82261	0.89080	0.06724	0.47914	7.00002	5.12378
**Arntl2**	0.04378	0.00766	0.05725	0.73755	0.00829	0.00581	0.00728	0.01901	0.00000	0.01354	0.01328	0.01722	0.01147	0.00933	0.02957	0.00714
**Arpc1a**	16.07424	6.04123	8.84228	11.44561	10.20199	9.03682	8.95978	13.02334	11.65156	17.11764	8.52995	2.40883	11.36490	1.23489	3.42918	12.29208
**Arpc1b**	21.71109	7.96067	9.36658	4.47398	16.47361	10.88274	15.88181	14.06904	2.37242	17.79881	12.81087	0.89477	18.27930	0.13696	0.00000	17.96748
**Arpc2**	18.70668	11.15630	22.46402	7.29124	16.46279	12.85269	7.59028	17.14630	12.18501	16.59533	7.92638	9.87234	19.27531	9.73729	7.99760	9.46104
**Arpc3**	2.93997	12.99784	3.01691	1.68703	5.27021	3.08810	2.76562	1.84564	2.31605	3.43102	6.53443	0.18638	3.31012	4.14913	4.93314	1.08363
**Arpc4**	17.87280	3.00647	5.90193	0.00000	4.86391	9.90798	3.26602	20.51409	0.12963	13.43186	1.31101	0.51330	19.02602	0.16895	0.01442	8.73388
**Arpc5**	14.16004	13.59276	12.72254	3.98406	15.46520	18.35739	13.09207	14.16985	6.50360	11.12484	8.78646	11.78859	19.03328	5.66298	0.75274	12.51391
**Arpc5l**	6.95554	0.31125	5.11738	13.21188	7.62495	7.87581	1.44114	4.51440	13.94243	15.42277	5.53519	3.41264	8.96678	1.12240	2.53719	6.72565
**Arpin**	1.75161	0.00564	0.00307	0.52544	0.00463	0.00636	0.04525	0.00000	1.43521	0.59439	0.49563	0.00896	0.00782	0.00584	0.10429	0.01246
**Arpp19**	9.45805	1.53474	6.60412	4.21707	5.56489	2.13904	2.41858	0.20746	5.97866	4.82784	11.50958	2.90089	1.64631	7.26160	1.93729	1.02306
**Arrb1**	1.18680	0.00408	4.92297	0.36627	5.00628	3.92828	0.00471	0.44260	0.23242	1.29242	1.61161	0.01836	1.42754	0.33555	0.36541	0.38291
**Arrb2**	0.58349	0.02700	0.62322	5.82000	0.00709	0.00431	0.27652	3.23865	2.93006	3.13492	2.13412	0.05537	0.09590	0.00000	0.14260	0.28348
**Arrdc1**	0.97435	0.22511	0.00000	8.32008	0.00546	0.00963	2.82760	6.11484	7.69032	12.87468	3.60410	0.47064	1.72842	0.03880	4.18770	0.01002
**Arrdc2**	0.40428	0.00597	0.45950	8.13305	0.00613	0.00671	0.00499	1.57333	11.03674	2.88142	3.13782	2.16031	0.00000	0.00000	1.00010	0.12224
**Arrdc3**	0.06608	0.00791	0.00840	0.66792	0.00897	0.00663	0.00751	0.01430	0.12931	0.00362	0.00889	0.00334	0.03927	0.00655	2.14968	0.02209
**Arrdc4**	0.01409	0.00797	0.09882	0.27385	0.00898	0.00567	0.00741	0.01156	0.54999	0.20046	0.01062	0.00691	0.01062	0.01631	0.00341	0.02133
**Arrdc5**	0.29934	0.00783	0.00507	0.79917	0.00719	0.00722	0.00716	0.02718	0.65550	0.28723	0.36373	0.00415	0.00832	0.00551	0.03431	0.00712
**Arsa**	2.50647	0.00562	0.00000	0.21678	0.00318	2.16977	0.00607	0.45582	0.00840	0.44520	0.11446	0.01601	0.00000	0.01072	0.00000	0.00361
**Arsb**	0.00472	0.02758	0.04921	1.14101	0.01944	0.00767	0.00702	0.37340	0.37092	0.00770	0.01415	0.46954	0.01596	1.70888	0.00000	0.00712
**Arsg**	0.01302	0.00796	0.01213	1.01393	0.00885	0.00647	0.00726	0.01027	0.04971	1.26485	0.01315	0.00571	0.01068	0.01374	0.00258	0.01409
**Arsk**	0.02439	0.00821	1.07658	8.59469	0.00759	0.00447	0.00716	0.00266	5.48326	0.16551	0.21853	0.00787	0.00730	0.00000	0.84681	0.00513
**Art2a.ps**	0.01371	0.00786	0.01183	0.33179	0.00927	0.00675	0.00755	0.01331	0.00625	0.04906	0.01165	0.00796	0.01324	0.00745	0.30777	0.00736
**Art3**	0.00000	0.00759	0.29338	0.31800	0.00861	0.00571	0.58503	0.00000	0.00506	0.91769	0.46958	0.07689	2.71365	2.66905	1.23754	0.01289
**Art5**	0.01284	0.00787	0.01348	0.79438	0.00994	0.00694	0.00752	0.01152	0.00000	0.49630	0.05428	0.00471	0.09778	0.00506	0.47187	0.00785
**Artn**	0.01383	0.00825	0.01085	0.01679	0.00920	0.00666	0.00749	0.00969	0.01232	0.00467	0.16523	0.00774	0.01027	0.00767	0.08781	0.00730
**Arv1**	0.04743	0.91795	0.95820	1.87343	0.00608	0.00544	0.68101	2.94876	2.49369	0.47614	2.69321	0.35799	2.35867	0.41965	0.24192	0.03327
**Arvcf**	5.49610	0.67062	0.54695	0.82509	5.05243	6.58524	3.03306	14.63341	1.55003	4.01447	5.14820	0.14000	8.51004	0.29544	0.23724	4.02633
**Arx**	0.01346	0.00786	0.01141	0.42898	0.00918	0.00645	0.00738	0.01100	1.94049	0.12670	0.01731	0.00287	0.01292	0.00921	0.17269	0.00759
**Arxes1**	0.01214	0.00810	0.02576	0.32464	0.01029	0.00662	0.00767	0.02050	0.00545	0.01504	0.03396	0.00697	0.16887	0.00550	0.30748	0.00766
**Arxes2**	0.01089	0.00791	0.02458	0.09047	0.02068	0.00650	0.00758	0.02449	0.00778	0.30576	0.03146	0.00279	0.10339	0.00511	1.77774	0.00776
**As3mt**	12.54003	4.14837	9.03842	0.10692	7.29485	3.87311	3.17076	7.05318	0.67911	0.25458	3.75118	0.03683	7.04040	0.00000	0.15448	1.35514
**Asah1**	6.89913	2.56157	6.83167	1.00109	3.98995	11.89921	8.07365	7.80003	0.89679	4.68139	11.52944	4.03600	6.39577	16.59432	8.70753	5.03144
**Asah2**	0.01370	0.00783	0.01117	0.06384	0.00931	0.00679	0.00754	0.01265	0.03491	0.05936	0.00930	0.01881	0.01340	0.01036	0.53566	0.00741
**Asap1**	2.32929	0.00284	0.14793	7.75750	3.07935	0.00215	0.28346	0.22456	2.22063	1.97555	0.28517	0.67022	0.22568	1.57873	1.27014	3.44008
**Asap2**	1.08263	0.03557	0.75503	0.00000	2.10420	0.23350	0.00509	2.27755	0.00584	1.69114	2.16898	2.64168	0.26812	0.05504	0.21436	0.04797
**Asap3**	0.03316	0.01753	0.83583	0.00664	0.02939	0.00869	0.05687	3.58715	0.10272	0.00000	1.71172	0.02863	2.31118	0.34649	0.00000	0.02023
**Asb1**	1.82831	0.02621	0.00000	0.16245	0.05316	3.31369	0.00548	0.00806	0.04023	0.00000	0.00946	0.02748	0.00016	0.01266	0.07805	2.53904
**Asb10**	0.01373	0.00820	0.01154	0.42464	0.00924	0.00655	0.00748	0.01205	0.42288	0.00619	0.06371	0.00755	0.01165	0.00748	0.07992	0.00727
**Asb13**	4.77513	0.36663	0.78856	6.40202	3.95875	8.38817	0.03296	3.16230	7.15864	3.16737	2.30305	0.40919	4.22091	1.68261	4.76754	1.67916
**Asb14**	0.01783	0.00786	0.01195	0.01042	0.00900	0.00664	0.00751	0.01389	0.01230	0.01260	0.01281	0.00772	0.01268	0.00999	0.06580	0.00737
**Asb16**	0.00689	0.00778	0.00897	0.01053	0.01022	0.00691	0.61543	0.18017	0.01212	0.00428	0.01262	0.00734	0.36237	0.01451	0.00706	0.00722
**Asb17**	0.01056	0.00763	0.38421	0.01030	0.00917	0.00933	0.27757	0.03563	0.01245	0.01188	0.01319	0.00690	0.01721	0.00934	0.02942	0.00742
**Asb17os**	0.05949	0.00735	0.33140	0.01007	0.00805	0.00835	0.43675	0.21957	0.01221	0.02649	0.01317	0.00601	0.08020	0.00811	0.05444	0.01510
**Asb3**	1.73611	0.00471	0.19861	0.00000	3.60538	0.00449	0.01856	0.17988	0.32270	0.27692	0.00637	0.04139	0.06592	0.00620	0.66374	0.02052
**Asb4**	2.05060	0.00555	0.25275	0.52871	0.00512	0.00344	0.00537	0.00000	0.00000	0.50369	0.01251	0.03078	0.01030	3.07714	0.82129	0.00506
**Asb6**	0.97844	2.18459	0.34293	1.18766	1.65616	0.01077	0.53733	6.95963	3.88735	0.96558	0.90971	0.23843	0.01834	0.01537	0.02690	0.24607

**Asb7**	0.69149	0.00582	0.22890	1.27551	1.89442	0.00481	0.00638	0.32843	0.11030	0.03364	0.74754	0.04242	0.46197	0.01078	0.03368	0.52891
**Asb8**	5.65029	0.00000	1.30188	4.16131	1.35903	0.02131	1.54033	2.74421	1.00510	1.97561	2.01630	0.10736	3.09527	0.54295	3.95632	1.26905
**Ascc1**	4.43470	0.01185	1.72728	1.07554	5.31413	4.54963	1.14264	0.38811	0.92818	2.86843	1.00517	0.22185	4.19810	0.47657	1.42967	1.17498
**Ascc2**	3.10219	4.39336	4.24978	5.55265	2.42888	4.40343	4.18706	8.67068	8.27415	7.91368	7.17639	1.51515	6.19954	0.04519	3.02691	1.42409
**Ascc3**	2.88590	8.04582	0.41518	6.34432	0.71228	0.00169	0.00000	2.64542	4.23358	3.49186	1.52353	2.54333	1.70276	9.27434	9.33780	4.39485
**Asf1a**	4.87587	2.48038	3.10448	4.10690	6.68813	0.02339	1.33349	0.10781	4.59020	3.08435	5.11937	6.69153	0.05009	1.73142	0.27862	0.01269
**Asf1b**	7.70696	8.30221	14.23428	10.77459	5.01753	5.20342	8.09421	8.28670	10.27217	7.50688	1.63598	1.37822	4.09973	3.14323	7.30140	3.59558
**Asgr2**	0.01327	0.00780	0.01010	0.09133	0.00914	0.00671	0.10792	0.00824	0.58523	0.41481	0.08599	0.00532	0.01120	0.04864	0.03643	0.00745
**Ash1l**	0.08486	0.00580	0.37674	2.54479	0.00725	0.00558	0.01531	0.27936	5.08943	0.74923	2.18952	4.91420	0.00000	0.62983	3.94023	0.02005
**Ash2l**	20.61074	15.08734	16.02320	10.54862	10.76727	15.79701	15.32180	21.70393	11.22056	11.78504	15.49039	1.41683	19.14505	12.11920	9.27580	8.56389
**Asic1**	0.14860	0.00779	0.00282	0.13382	0.01603	0.01505	0.00716	0.00000	0.04462	0.25260	0.47920	0.08550	0.01173	0.08500	0.08599	0.00700
**Asic3**	0.00241	0.56208	2.13753	0.00820	0.00493	5.97436	0.00662	0.53851	0.01082	0.00000	0.18493	0.00397	0.00000	0.01088	0.00000	0.00436
**Asl**	1.37731	3.67038	1.34915	2.40947	2.65830	1.56918	0.00352	0.00000	1.56710	1.93289	2.95713	0.09905	0.00000	0.01769	1.08537	0.12769
**Asna1**	11.15425	10.07769	11.33122	3.18054	11.38777	8.58934	11.05529	12.72597	2.27417	15.25418	8.00679	1.88130	13.03687	0.38084	4.42136	9.92257
**Asns**	21.86554	12.22561	0.00000	0.57938	26.22617	0.02046	9.28253	9.03296	0.46945	0.15097	0.51245	0.94590	0.51863	0.00828	1.68020	13.03135
**Asnsd1**	8.76693	8.43110	7.66362	3.87119	13.75853	5.31363	4.09756	7.60010	3.19907	7.71709	7.08241	6.26621	5.12967	5.24304	7.63059	5.20350
**Aspa**	0.03875	0.00387	1.10311	0.00817	7.17939	0.00543	0.00724	0.01123	0.00951	0.01133	0.01080	0.00035	0.00695	0.01612	0.00000	0.00681
**Aspg**	0.00384	0.00475	0.00000	7.62941	0.02453	0.01195	0.00388	4.60827	5.15976	7.75326	7.32196	5.04196	0.00000	0.24684	2.26960	0.00411
**Asph**	0.00000	0.04490	1.73946	0.56067	1.26514	0.00503	3.37611	1.16184	3.12405	0.16552	0.73315	3.18221	0.35289	0.72943	3.41754	0.32354
**Asphd1**	0.75014	0.15905	0.00352	0.00555	0.01740	0.00740	0.00677	0.00172	0.01154	0.00541	0.30715	0.00619	0.00975	0.01008	0.00520	0.00667
**Asphd2**	0.01373	0.00812	0.01104	0.09383	0.00925	0.00665	0.00751	0.01095	0.41449	0.01543	0.12724	0.00687	0.01084	0.01142	0.00252	0.00734
**Aspm**	1.36396	0.02600	2.69620	6.21808	0.04251	0.97740	0.20950	0.45141	10.79519	0.82675	0.38004	2.54016	0.50198	5.33971	8.87169	0.00272
**Aspn**	0.01371	0.00791	0.00564	0.00617	0.00930	0.11065	0.00736	0.02247	0.02213	0.00000	0.16819	0.00699	0.00860	0.00789	0.15289	0.00726
**Aspscr1**	5.29595	3.62521	3.46424	2.36875	8.34507	4.86697	0.70532	9.19235	3.05463	7.11754	2.74564	0.36536	5.98751	0.00000	0.00000	4.49842
**Asrgl1**	0.73333	0.00796	0.85431	0.38800	0.02333	0.00477	0.00623	0.00620	0.00594	0.81755	0.00991	0.07163	1.07621	0.00886	0.02209	0.00562
**Ass1**	0.01268	0.00834	0.01175	0.00781	0.00967	0.00717	0.04068	0.03002	0.02613	0.04504	0.06347	0.00866	0.04961	0.01181	0.01031	0.00755
**Aste1**	1.41840	0.00480	0.68022	5.46056	0.00404	0.29342	1.04310	3.81354	6.45023	5.63833	3.20124	0.07136	0.98683	7.72691	3.00261	0.02440
**Astl**	0.00012	0.00806	0.75045	3.08051	0.00855	0.00502	0.00721	0.93480	3.11953	2.10135	0.00000	0.08518	0.00301	1.92274	0.00000	0.00626
**Asun**	7.54930	1.64090	4.80693	9.69045	6.62912	0.91177	2.30558	6.65023	3.31542	10.09165	2.74978	9.23828	2.64384	10.27379	14.52025	0.02091
**Asxl1**	3.64637	2.90257	1.88169	11.79410	2.77614	0.00000	0.00000	4.07586	13.82301	6.53792	3.79647	3.22685	1.71901	2.65438	7.70557	4.29632
**Asxl2**	0.86399	0.06873	0.50961	6.29723	0.45306	0.66958	0.41268	3.85275	11.30089	3.69635	1.22288	4.42374	1.09933	6.48103	6.08944	0.07569
**Asz1**	1.75987	0.00411	4.96642	1.74473	0.90364	0.00328	1.77188	0.48450	0.62464	1.04672	1.47091	1.10502	3.19573	4.77441	2.79577	0.00148
**Atad1**	1.57283	3.95735	5.40633	9.50884	0.00000	0.00799	5.51104	4.51371	7.67421	2.69944	2.58049	6.17803	0.15785	11.00613	9.73989	0.97386
**Atad2**	4.95484	1.91866	4.00007	11.35930	5.35542	0.09678	1.59376	2.24612	15.52806	5.50521	6.53080	2.84562	3.04555	12.25544	9.10813	1.68434
**Atad2b**	3.71134	2.56003	2.66045	8.32947	2.55820	4.17769	7.29540	7.35304	8.21757	10.67440	4.39114	10.14360	2.99844	9.87767	14.80110	0.83809
**Atad3a**	19.70720	8.40862	12.97615	6.63837	9.29042	6.92311	11.06517	15.11971	3.25156	10.33939	3.65222	0.85640	11.52607	2.17633	4.29194	4.37850
**Atad5**	2.45843	0.00000	0.00000	4.03703	0.00329	0.01124	2.01901	1.74586	4.20065	2.13514	0.36767	7.74379	0.13808	7.59861	5.07686	0.19682
**Atat1**	2.75097	0.00434	0.00000	4.69652	0.00208	0.00562	0.00383	0.53517	3.27856	1.27440	1.08903	0.01970	1.11301	0.18249	0.00000	2.08448
**Atcay**	0.01376	0.00765	0.00877	0.76227	0.00924	0.00671	0.00722	0.01065	0.87361	0.00348	0.00149	0.37355	0.01353	0.00985	0.00000	0.01478
**Ate1**	1.49348	1.76235	5.16756	2.35301	1.38019	0.00400	3.44097	2.29912	3.70344	2.13902	1.72967	1.64963	2.86247	0.70906	0.71446	1.47590
**Atf1**	6.59834	3.91193	4.71564	16.93756	2.50723	6.87626	4.52319	11.09608	18.44383	14.68202	8.40645	13.42093	7.70457	22.66789	20.18505	1.79106
**Atf2**	1.88224	1.35949	1.03419	7.11732	5.14300	0.31388	4.99168	1.26693	10.40928	6.94471	6.30418	1.68025	0.53860	15.51695	13.67719	0.04362
**Atf4**	19.93654	10.75299	18.41742	19.74084	13.96331	12.00241	10.38008	26.68518	25.82456	30.05025	16.31268	7.35041	17.25655	3.91694	8.86634	23.13815
**Atf5**	0.00530	0.03723	0.50079	0.16061	0.24520	0.00879	0.00698	0.55772	0.44395	0.97745	0.24732	0.06995	0.01353	0.00516	0.45218	0.01438
**Atf6**	0.00000	0.16262	2.69962	0.53382	1.22275	0.97191	3.01530	5.64476	0.01642	3.76982	1.21962	0.25776	1.34081	2.05926	1.94346	2.33100
**Atf6b**	2.44947	0.89191	2.12486	2.53742	0.42485	0.01130	0.60761	2.20295	2.50954	2.44574	3.63533	0.25632	6.51706	0.01679	0.06801	3.33034
**Atf7**	3.19917	0.00265	0.54660	1.13877	0.00886	0.12544	2.33567	0.31623	0.76060	1.42953	0.21496	0.13823	2.11947	0.65181	0.81361	1.58145
**Atf7ip**	0.81798	0.21649	3.87284	8.24073	6.62827	0.01378	1.43804	4.69270	15.63533	4.02634	1.61842	0.09203	4.45908	0.02751	0.76996	0.93897
**Atf7ip2**	1.33550	0.00179	0.13818	10.81320	3.36216	0.00124	0.00316	0.00000	14.48328	4.16239	4.18341	9.05623	0.47744	6.03534	6.41625	0.19157
**Atg10**	1.92507	0.00602	0.00000	0.46955	0.00780	0.00738	0.00594	0.00000	0.46695	1.64115	0.24286	0.00567	0.18893	1.13015	0.16311	0.01399
**Atg101**	9.54647	1.54504	6.50377	10.12366	8.72620	0.88562	2.41050	11.54991	12.01226	8.09901	8.40046	0.80653	7.32174	0.33484	0.47327	1.00127
**Atg12**	6.06869	7.09677	9.24561	5.26158	5.19432	5.29987	1.11718	6.02016	8.59035	16.72714	8.39632	13.15071	7.34698	6.68196	10.48030	0.00769
**Atg13**	18.20444	8.52425	8.01545	6.70776	6.77379	4.27881	11.29135	11.64704	9.57409	16.27245	8.55676	1.54206	11.96329	3.28296	13.04109	7.72245
**Atg14**	5.16267	5.43287	13.91625	12.71919	3.42154	2.70412	8.08948	18.49224	14.05183	19.29549	18.40795	4.31542	14.55142	14.78568	18.51132	0.61296
**Atg16l1**	4.72570	0.53168	5.97208	3.98416	0.09319	1.18980	0.00000	3.77818	9.31723	11.21693	1.81117	6.36823	3.29693	2.09482	14.62345	1.31096
**Atg16l2**	5.93166	1.48567	5.98171	0.06424	0.04878	5.93427	5.72738	12.10129	0.03773	5.33210	5.33454	0.04819	16.96866	0.52676	3.08945	2.46774
**Atg2a**	9.47683	6.60406	14.60088	6.40286	14.65569	10.65537	10.60865	21.26897	9.40536	20.27295	7.20182	3.47317	20.23429	0.00000	1.76918	10.16290
**Atg2b**	5.66443	5.84362	5.08886	2.13335	4.09347	4.71274	6.62117	9.44393	6.22726	10.53560	4.17048	2.95365	2.91615	7.33286	9.57746	0.04362
**Atg3**	1.70757	2.33854	1.47640	11.22955	2.31959	2.79751	1.68653	0.58972	11.63868	21.20199	9.92487	2.26864	2.67408	12.56303	11.24393	0.07730
**Atg4a**	0.49717	2.02471	2.77186	0.34993	3.52641	1.93042	0.00586	0.61670	0.00000	0.69399	0.00000	0.29102	0.38056	0.99382	0.78598	0.02588
**Atg4b**	13.77077	15.23212	32.05116	6.47359	11.25912	1.06665	20.14624	29.32952	14.43793	22.08555	25.34513	2.60867	20.31932	2.64919	14.23693	5.44248
**Atg4c**	0.36795	0.54152	0.03925	0.03256	0.00740	0.00691	0.00676	0.57112	0.02057	0.12082	0.03498	0.16073	0.17227	0.19795	0.12133	0.04346

**Atg4d**	0.05577	0.14735	0.44803	0.74337	0.04456	4.29789	0.51966	7.53073	0.02064	5.31411	1.83492	0.08878	5.67746	0.12802	0.38405	0.01020
**Atg5**	2.94402	0.89930	3.93264	6.34012	7.40623	4.67515	4.02714	10.45979	3.29832	4.52393	2.65820	0.46327	5.57609	3.32505	2.66559	4.19708
**Atg7**	7.62355	4.56177	7.48482	1.10200	1.23784	1.77003	3.14104	16.50660	2.18202	12.89858	10.55595	1.04712	14.03361	0.45997	1.78862	0.02172
**Atg9a**	1.50047	2.57056	9.43277	11.45139	1.81859	0.13614	7.31089	24.21921	13.88586	25.70113	19.79649	6.75220	13.66341	4.85393	9.04407	1.38542
**Atg9b**	0.00804	0.00728	0.92553	0.01125	0.00809	0.00916	0.00685	1.00369	0.01120	0.00001	0.00646	0.00502	0.21031	0.01785	0.00000	0.00687
**Atic**	2.62293	2.83308	3.63356	0.42044	1.59395	0.70031	2.07411	5.88761	0.67655	0.42074	1.76210	0.17340	3.43105	0.60820	3.66101	2.03531
**Atl1**	0.00909	0.00793	0.41574	0.68805	0.00772	0.00926	0.00695	0.00000	0.00000	0.75401	0.45080	0.00980	0.01927	0.69867	0.95772	0.31015
**Atl2**	3.62981	3.91143	2.50991	4.86941	1.27627	4.10171	0.15078	5.42363	6.85021	0.66278	4.50706	7.16575	1.30301	7.29617	5.75819	0.97627
**Atl3**	4.12615	0.63991	0.46053	13.13794	3.06576	0.00656	1.98105	2.24321	11.65689	1.61965	1.66660	0.09197	0.13195	11.20418	6.53038	0.02935
**Atm**	6.14715	0.00000	5.80330	7.55959	3.47645	0.72758	4.32402	6.56625	5.07990	9.33517	6.24267	6.72591	3.23240	12.99556	10.31391	0.63926
**Atmin**	9.27831	7.49739	7.98420	10.99935	1.87463	5.88793	8.25816	7.20609	11.25264	13.10603	7.34981	0.57110	4.63729	8.37995	12.16178	0.16449
**Atn1**	3.59016	0.55173	1.34403	3.04376	2.31289	1.52469	2.18096	8.32770	4.09421	3.95564	11.53184	0.40752	2.50996	1.38063	0.95371	2.11225
**Atox1**	9.68385	0.67840	6.79771	5.35718	10.14370	2.75794	0.74534	1.17120	2.78194	6.23263	16.05613	0.57087	3.75474	0.05443	2.18952	5.99392
**Atp10a**	0.01810	0.00786	0.00963	0.51329	0.00898	0.00676	0.00732	0.01188	0.00474	0.01184	0.01266	0.17305	0.01299	0.01078	0.00000	0.00734
**Atp10b**	1.11181	0.01563	0.01393	1.23891	0.00575	0.00697	0.00635	0.01015	1.04421	0.00000	0.02911	0.00270	0.02992	0.04946	0.85747	0.00580
**Atp10d**	0.00558	0.00755	0.24108	0.00367	0.01915	0.00605	0.00692	0.76754	0.00908	0.15688	1.40289	0.75948	0.42622	0.06152	0.24182	0.00670
**Atp11a**	0.00343	0.00544	0.00000	11.11133	0.01863	0.00366	0.00493	0.58590	3.04319	2.11914	1.01179	6.66190	1.00951	0.01398	6.15577	0.01149
**Atp11b**	0.01361	3.86580	0.09808	3.07353	0.01098	1.25843	0.00334	1.56328	1.49711	0.46287	3.81572	5.54925	0.25260	9.03778	4.01089	0.00355
**Atp11c**	0.14555	3.21706	1.09093	5.99719	0.00531	0.50787	1.27204	0.34233	1.07920	0.65240	0.20565	9.57284	2.25093	9.29655	4.30079	0.63800
**Atp12a**	0.00000	0.00793	0.01105	0.01060	0.00672	0.01807	0.00686	0.01233	0.01241	0.00469	0.01250	0.00725	0.84220	0.01344	0.01020	3.85302
**Atp13a1**	3.47253	3.31549	7.07396	1.24480	0.08204	3.31695	6.49876	10.54434	4.77546	7.60146	2.33281	0.23048	9.68053	0.27642	0.61447	2.73397
**Atp13a2**	0.10056	0.01817	0.70853	2.82516	0.00794	0.25886	0.18424	1.32217	5.21230	1.87375	1.83817	0.71777	2.45989	0.81895	0.35838	0.24742
**Atp13a3**	13.32450	9.91282	8.67014	20.69028	4.42026	6.78736	10.40536	13.89150	19.43662	15.35230	18.52390	23.02707	7.61001	34.63383	23.43644	1.96599
**Atp13a5**	0.01328	0.00768	0.01237	0.13737	0.00907	0.00642	0.00730	0.01052	0.00974	0.86040	0.01182	0.00480	0.01100	0.00559	0.14362	0.00727
**Atp1a1**	11.69490	4.64581	11.82143	3.64852	9.79827	8.65794	14.16813	16.69183	3.46989	18.85826	8.71238	0.35289	17.85894	3.86826	4.84031	9.08412
**Atp1a2**	0.71469	0.01799	0.02017	0.05877	0.04752	0.00729	0.01726	0.00630	0.23875	0.02196	0.25159	0.00535	0.03440	0.22532	0.03563	0.00641
**Atp1a3**	0.01451	0.00707	0.25772	0.42337	0.00887	0.00561	0.74339	0.00952	0.22801	0.00945	0.02279	0.00595	0.04759	0.00574	0.27337	0.00704
**Atp1b1**	4.74023	3.89765	3.53245	4.41140	0.41281	7.14493	6.50973	2.58148	8.97761	0.42438	1.74827	0.14897	7.91461	2.74349	1.18550	5.93320
**Atp1b2**	0.89689	0.01767	0.03519	0.48269	0.00617	0.00483	0.00601	1.03771	1.35447	0.75522	2.98825	0.09479	0.00000	0.33728	1.90660	0.01317
**Atp1b3**	5.43537	7.25219	7.16186	14.48853	5.82241	4.49418	3.72908	8.62147	12.23507	19.41653	2.57695	13.40360	3.87078	13.71030	18.38016	3.72885
**Atp2a1**	0.01130	0.00765	0.01168	0.00475	0.05418	0.00652	0.00745	0.01284	0.14565	0.00755	0.01105	0.00698	0.01865	0.00698	0.22328	0.00728
**Atp2a2**	9.64467	10.03604	15.98573	18.56873	2.97616	7.65988	8.12517	11.72240	8.75001	12.08832	6.95468	4.48516	10.93499	13.58717	17.27290	5.85840
**Atp2a3**	0.05127	0.00749	0.51224	0.01053	0.00700	0.01121	0.00725	1.23686	0.02141	0.00271	0.00485	0.00549	0.00335	0.01694	0.00000	0.00701
**Atp2b1**	1.98870	0.08854	2.48732	0.23252	0.15504	0.81411	0.00406	1.54310	1.94396	1.11182	0.06844	6.17384	1.45983	8.50329	1.09305	2.93607
**Atp2b2**	0.00894	0.00750	0.00795	0.04944	0.00955	0.00699	0.00709	0.72576	0.00870	0.34450	0.00782	0.00491	0.00598	0.01282	0.00000	0.00782
**Atp2b3**	0.01015	0.00833	0.00830	0.07079	0.00971	0.00679	0.00810	0.08889	0.50739	0.00345	0.00451	0.00541	0.00937	0.64443	0.00000	0.00743
**Atp2b4**	0.00130	2.28459	0.00000	0.16607	0.13640	0.00608	0.28618	0.00000	0.65529	0.00000	2.75655	0.02499	1.31837	0.00000	2.05880	0.01202
**Atp2c1**	3.31163	1.67117	1.14073	6.08337	1.72714	0.78515	3.14428	1.92856	2.73455	5.04899	0.36094	0.28435	0.00000	0.05190	12.25704	3.32846
**Atp2c2**	0.35914	0.00679	0.14929	0.00764	1.15793	0.00589	0.00711	0.01066	0.01151	0.43769	0.01468	0.02289	0.21640	0.01188	0.00653	0.00744
**Atp4b**	0.00002	0.00734	0.00351	4.20286	0.00881	2.81754	0.00497	2.52104	3.08695	0.31491	0.00000	0.00000	3.73194	0.00000	0.15509	0.00366
**Atp5a1**	39.94049	22.64359	35.43673	13.27873	29.09977	21.25819	20.84879	35.77459	18.96589	32.44879	29.30140	13.77420	39.21827	16.88149	22.91967	24.53801
**Atp5b**	43.59215	19.94262	31.56107	24.46706	30.99949	19.73754	17.86213	29.83226	34.83509	40.15873	32.68041	18.21364	46.18478	23.84076	20.90557	29.18489
**Atp5c1**	32.51642	18.04962	26.53968	11.52830	22.98187	16.19415	14.84691	23.76596	10.35451	30.76655	17.91705	11.75727	27.99901	9.03843	7.90770	19.03515
**Atp5d**	15.71790	0.78954	8.28609	1.24068	12.99633	5.39536	1.43596	9.29160	0.86807	20.49096	11.77234	0.37216	27.82791	0.06325	0.00000	12.00390
**Atp5e**	7.69065	0.30949	4.85731	2.48078	3.42009	1.46436	1.03864	0.24928	1.47418	2.68094	14.22795	0.98298	3.01377	0.04900	0.00000	1.09191
**Atp5f1**	35.86409	15.93082	27.54766	15.76130	21.69596	18.64827	18.82506	33.73679	19.68186	36.70857	24.99538	20.91166	32.66624	17.02123	8.28422	20.13998
**Atp5g1**	26.15814	5.07589	26.95509	18.50578	19.66463	11.60384	10.69986	21.19457	27.46868	24.78314	32.75256	5.07741	24.33955	1.83169	13.05134	13.29943
**Atp5g2**	22.60682	10.68824	14.87677	1.74290	16.30200	5.05706	9.82367	13.05548	2.55766	12.01620	13.72811	1.36285	17.31443	0.62389	4.46641	13.40339
**Atp5g3**	34.83053	3.86051	16.14442	6.05647	21.50519	13.08515	7.40624	12.47189	9.84299	18.66615	18.20177	1.25472	22.33303	3.50598	1.32385	15.29324
**Atp5h**	21.91345	6.19177	17.69689	12.01050	14.68757	8.58044	5.25554	6.63995	10.74301	13.90725	24.93097	2.08699	19.49931	10.10090	8.32642	9.39431
**Atp5j**	15.58724	1.68270	16.08665	7.96234	16.30704	4.26433	2.33896	2.88178	15.90304	13.48382	25.29838	2.31650	8.01678	7.02266	4.05865	5.43224
**Atp5j2**	7.72653	0.32340	6.36446	8.46205	4.57516	1.33613	0.72010	0.47779	11.87727	5.19644	17.66066	0.60899	4.05367	3.69934	2.73521	4.37258
**Atp5k**	6.51687	0.35928	5.02650	4.37438	7.15748	1.59974	0.49989	1.29909	4.68492	1.43125	22.11217	0.97247	2.46348	1.44793	1.57897	3.93900
**Atp5l**	13.75705	1.33550	12.19778	5.82070	12.27814	4.58184	1.99149	1.09907	1.17673	7.49680	22.71401	1.70032	9.43716	5.54797	3.33603	6.14531
**Atp5o**	28.77810	16.71654	20.45717	6.43809	15.09946	15.49354	14.47947	16.29555	6.85378	18.97518	17.91145	6.42758	23.54011	4.47326	6.26578	17.62344
**Atp5s**	1.66487	0.78279	1.00930	2.69316	0.00549	1.81949	0.29219	2.13351	3.47941	1.39164	2.65072	0.28777	1.83159	1.80731	0.67619	1.85237
**Atp5sl**	6.67103	0.43761	3.68471	1.77368	3.44235	0.00000	4.31877	7.72957	3.14712	6.41675	3.32800	0.78353	1.42365	0.04134	0.00000	2.00868
**Atp6ap1**	1.70789	4.71410	8.29241	5.24974	5.69645	4.48631	1.20736	8.01846	3.66081	6.98631	8.38252	0.30558	10.71437	13.42256	15.18411	2.65017
**Atp6ap1l**	0.00000	0.00557	2.02696	0.21015	0.00694	1.31873	3.77716	1.65297	1.36497	0.73438	1.65028	0.06700	1.32599	2.11111	4.96819	0.00502
**Atp6ap2**	3.81491	3.67147	18.33336	8.06803	8.12384	5.44447	10.31340	16.09456	5.48722	9.96921	9.84118	11.82993	11.49781	22.90784	11.99345	6.47108
**Atp6v0a1**	5.79659	0.00000	2.50271	0.17056	0.47694	0.05413	6.61146	4.71635	0.02658	6.30096	4.33688	0.13526	2.16379	0.00295	0.44359	0.22556
**Atp6v0a2**	0.78430	6.97098	2.46071	6.30132	0.00174	0.43639	0.00221	1.87654	1.98418	3.10386	0.83479	0.36789	2.71463	0.23547	2.75646	1.46710

**Atp6v0a4**	0.00000	1.81007	0.46986	0.48139	0.02431	2.15412	0.00468	0.72423	1.18664	0.81081	4.39603	0.02204	0.18141	0.61206	1.40580	0.25527
**Atp6v0b**	18.66576	7.11039	15.84351	7.89261	8.83478	13.73025	8.69080	15.43260	0.85633	14.12254	10.53211	11.93065	23.92628	0.05980	0.64360	8.01021
**Atp6v0d1**	9.54693	10.44846	12.60649	8.12330	12.49215	7.82615	7.21806	9.86831	7.94087	16.33814	14.67751	8.17090	13.09268	6.58077	5.09555	6.21065
**Atp6v0e**	15.54191	11.41860	25.61817	6.00647	4.61438	8.95022	7.33451	19.11347	12.56681	25.28835	16.10266	1.97994	19.15718	9.70256	11.59571	8.62904
**Atp6v0e2**	0.01227	0.00786	0.01214	0.33261	0.00946	0.00700	0.00750	0.01269	0.00612	0.25687	0.01155	0.00758	0.26264	0.01216	0.31563	0.00739
**Atp6v1a**	6.96691	9.14334	9.47505	4.25879	6.12041	4.82757	7.41393	5.33014	6.90901	5.84100	3.98125	15.63210	3.82031	11.66361	14.59901	3.27798
**Atp6v1b2**	13.33772	5.57585	6.95981	6.67731	6.50635	11.12685	9.00757	12.23448	1.14182	12.43455	8.40622	1.42672	8.67860	7.15223	6.77823	6.07534
**Atp6v1c1**	14.78226	13.17623	13.00047	3.44233	11.23725	11.55024	10.48498	15.52505	6.05208	8.74418	8.78838	11.16263	12.10569	0.20985	6.83112	6.79946
**Atp6v1c2**	0.41749	0.00764	0.00000	1.61959	0.00698	0.00628	0.00704	0.09950	0.00000	2.50241	1.36456	0.52951	0.00000	0.90264	1.87892	0.00623
**Atp6v1d**	12.85560	10.72154	11.20265	3.74541	3.86670	8.46219	3.29594	14.38186	8.15711	24.55921	9.39435	7.18390	11.27676	5.67200	13.05174	5.70845
**Atp6v1e1**	21.94367	16.97032	22.97370	7.47588	15.56366	11.53600	15.40734	22.14649	10.04857	23.31329	11.76104	13.81438	21.83168	2.15159	12.62763	13.46255
**Atp6v1f**	7.72579	1.89552	5.53055	1.18394	8.88496	2.51914	1.50715	3.69388	0.14217	4.51035	1.51627	0.37403	4.76288	0.00141	0.36610	6.60690
**Atp6v1g1**	20.24880	10.08092	14.78547	3.96843	11.90477	11.43285	9.65402	10.89818	1.17411	12.60077	9.09285	0.32541	13.13420	0.28614	0.59908	10.51702
**Atp6v1g2**	0.01377	0.00776	0.01045	0.21872	0.00907	0.00670	0.00725	0.00670	0.00828	0.36262	0.14997	0.03426	0.01077	0.01067	0.00512	0.00765
**Atp6v1h**	8.15747	9.33236	3.00644	7.02411	10.35666	2.81273	0.38251	2.38497	0.00000	8.24048	4.99788	4.11469	3.83328	0.94325	3.01130	0.00000
**Atp7a**	1.04310	0.00373	0.05909	4.75209	0.00619	0.00474	0.37321	0.00000	3.48760	0.51369	1.48380	8.94665	0.21864	4.85351	8.38960	0.00414
**Atp8a1**	1.35163	0.00521	0.76525	0.72134	0.01266	0.00413	1.46719	0.71323	1.20312	0.42885	1.77702	0.00000	0.68823	0.88945	0.00000	0.01277
**Atp8b1**	1.71051	0.00527	0.00000	0.51056	0.03923	1.17882	0.46925	0.00000	0.18401	0.26161	0.19082	0.00000	0.01603	1.54511	0.54166	0.63843
**Atp8b2**	0.44118	0.00767	0.00000	0.00004	0.03588	3.62388	0.00683	0.40407	0.14253	0.53827	0.00493	0.05016	0.00000	0.07733	0.03408	0.00655
**Atp8b4**	0.00000	0.00819	0.22100	0.01126	0.01119	0.00609	0.09157	0.01613	0.01085	1.14866	0.00000	0.20711	0.48430	6.31216	0.00000	0.00730
**Atp8b5**	0.01376	0.00775	0.01220	0.00899	0.00936	0.00655	0.00745	0.01258	0.01197	0.00932	0.01238	0.00705	0.01275	0.00670	0.28176	0.00741
**Atp9a**	2.21390	0.00281	1.59449	1.09842	0.82829	0.01369	1.12286	7.54975	1.62508	5.97271	2.24566	0.16820	3.61352	0.00000	0.42428	1.82215
**Atp9b**	4.30116	6.31941	1.69795	0.35980	0.01359	0.00759	0.04691	2.17922	0.71022	1.01897	1.46130	0.02024	0.39708	0.00000	0.19477	5.40885
**Atpaf1**	4.24183	2.80174	2.65593	0.91318	8.26165	0.06306	1.85882	0.31761	1.47602	7.30746	5.94041	5.10307	3.93798	2.48043	1.81400	1.73665
**Atpaf2**	4.62978	0.51973	1.31078	1.83209	3.74230	6.37154	4.29086	8.26175	4.73321	3.77121	7.68537	0.92916	4.76378	0.76696	3.80498	0.03215
**Atpif1**	11.36912	0.71818	6.93109	8.74808	7.82612	3.37056	0.88267	0.38883	14.04916	5.29100	13.40380	0.56241	5.04263	0.36007	1.29396	7.05870
**Atr**	1.63015	4.56343	2.12553	1.24648	2.64239	3.01330	5.79379	1.00288	0.70875	0.00000	0.47380	0.23346	0.44720	6.63991	1.39698	0.70275
**Atraid**	4.10696	2.24550	3.53261	7.38030	1.08903	0.25615	0.87166	5.06343	11.83879	9.91477	5.53523	9.11282	2.19452	1.14428	5.36788	5.31647
**Atrip**	1.86053	0.10215	1.48712	1.60092	0.02324	2.86856	0.07884	3.54774	0.73608	0.44499	1.06450	0.10824	2.34107	0.41560	0.83547	1.32276
**Atrn**	1.34085	0.01315	4.35295	1.92638	0.01122	0.00610	5.17457	1.38995	0.80821	3.91278	2.05311	0.16280	0.03181	1.18787	2.07140	0.01123
**Atrnl1**	0.01328	0.00774	0.00891	6.59205	0.00945	0.00587	0.00703	0.01016	0.68403	0.00000	0.00270	0.01277	0.01318	0.31890	0.99620	0.00616
**Atrx**	2.35375	0.89480	4.45040	10.43347	2.44612	3.74441	4.62335	4.12922	5.98375	7.60832	5.28318	9.56530	2.13094	23.15149	11.76296	0.60398
**Atxn1**	0.00000	2.46634	0.53952	1.83181	0.01605	0.00576	0.00663	0.35346	0.79548	0.41893	0.53264	0.01860	0.01425	1.21469	0.00000	0.00572
**Atxn10**	7.60053	1.07335	2.95783	3.80246	10.52487	6.60188	0.06527	5.39653	1.38176	10.56170	2.27918	1.93111	11.00599	1.28386	3.16522	11.49140
**Atxn1l**	1.59818	0.02805	0.00000	10.03381	0.33116	2.24849	0.13201	0.00000	7.33473	1.61596	3.39848	0.35091	0.54356	8.29486	2.61740	1.16647
**Atxn2**	2.42022	1.76828	1.88174	5.80441	2.43895	0.36187	3.50228	5.60811	1.92889	5.63982	4.31315	3.62159	0.77415	2.11963	7.66941	0.94231
**Atxn2l**	1.00766	0.88782	1.04681	9.38693	0.04714	0.49383	0.46462	1.21766	6.72362	3.00926	2.39590	0.22750	0.26301	0.15462	1.31182	0.14125
**Atxn3**	4.82355	0.03019	2.25783	8.64076	2.56397	1.05622	1.25416	6.62889	7.37953	6.35945	7.29876	0.93260	1.04660	9.41283	7.74907	0.11777
**Atxn7**	1.12605	0.65708	0.96898	5.05262	0.01320	0.62809	1.96895	1.63048	8.06028	1.05189	2.61911	5.92493	0.21880	10.87680	7.94042	0.00267
**Atxn7l1**	1.17553	5.67990	6.01726	0.35049	0.22770	0.31993	3.59805	0.77850	0.00594	0.15888	0.00113	0.00726	1.64987	0.00294	0.00000	4.34397
**Atxn7l2**	0.35799	0.00854	0.17037	0.00437	0.01806	0.00533	0.00735	0.01164	0.66858	0.07438	0.00882	0.00756	0.01932	0.02160	0.00181	0.00696
**Atxn7l3**	1.64167	0.66081	2.42069	1.82176	1.08012	0.14103	0.06635	5.83709	2.92161	12.37236	0.90728	0.31690	0.45337	0.01315	3.10385	0.54856
**Atxn7l3b**	2.88354	1.24160	5.63975	1.04369	0.40915	3.02816	3.35307	0.90087	4.08651	2.76164	1.40383	0.12274	3.19248	8.07452	3.27288	9.29939
**AU015228**	0.00000	0.15404	0.00739	15.57765	0.01739	0.02130	0.23951	0.10722	16.62205	0.00000	0.00000	6.91986	0.00854	0.05256	2.72303	0.00293
**AU015836**	0.00384	0.00830	1.95363	5.30101	0.00679	1.18253	0.00788	0.37924	5.13881	1.84386	0.00000	0.15921	0.09565	0.00000	0.23678	0.00524
**AU016765**	0.01403	0.00771	0.00846	7.24102	0.00913	0.00597	0.00697	0.00031	0.00000	0.00000	0.38240	0.00434	0.00829	0.03782	0.61177	0.00589
**AU018091**	6.87187	5.66347	8.28574	2.99115	2.01346	11.72429	3.19469	14.54682	11.33508	16.22434	21.52907	14.81392	16.35705	36.72990	15.03595	6.37021
**AU019823**	5.83292	0.11431	0.63214	5.54156	0.00000	0.00322	2.26245	0.00000	8.12154	2.44322	2.72477	11.66414	0.82582	8.72643	2.89327	1.32037
**AU019990**	0.01129	0.00793	0.05885	3.91591	0.00900	0.00798	0.00742	0.02027	0.39361	0.35104	0.00867	0.03783	0.17418	0.00758	0.17763	0.00635
**AU021092**	0.00000	0.00680	2.12888	0.00000	0.02368	7.36302	0.02295	2.36600	0.00898	2.40426	0.69483	0.01556	6.14751	0.01030	0.00000	0.00000
**AU022252**	9.26196	0.22694	1.99355	4.11156	4.74814	1.30864	2.39448	1.99481	3.27092	2.67290	2.34725	0.08472	6.39502	0.01172	0.80628	1.45020
**AU022751**	1.17559	0.45343	0.91900	3.56185	0.01548	0.01062	0.02627	1.05916	1.74692	0.58054	1.01521	0.00000	0.14957	0.00000	1.00699	0.00486
**AU022754**	0.07063	0.13234	0.03665	0.74624	0.00808	0.03062	0.00722	0.03291	0.73137	0.03631	0.02102	0.01568	0.08725	0.17149	0.18109	0.07245
**AU023762**	0.17577	0.00741	0.10229	1.43101	0.02592	0.02154	0.04749	0.03363	0.84057	0.05560	0.52251	0.00162	0.01321	0.52204	0.10773	0.02835
**AU040320**	3.18405	0.00000	3.47790	5.05287	0.05508	0.09223	3.84681	2.51553	0.82735	6.25766	1.39247	0.02853	0.07889	0.49373	2.31288	0.00001
**AU041133**	0.00000	0.00718	0.13722	0.39426	0.02092	0.38668	0.76753	0.92899	0.03144	0.63446	0.40025	0.02788	0.00000	4.87731	0.15207	0.00673
**Auh**	0.74090	0.00487	0.05422	4.94018	4.46654	0.00745	0.02774	0.01502	4.20880	1.64668	0.32452	0.04078	0.04732	0.05032	0.65318	0.02043
**Aunip**	0.39208	0.01615	2.04799	4.99695	0.00364	3.28272	0.00340	0.00000	8.87655	1.26501	5.11455	0.09548	1.42091	0.21392	2.24936	0.03776
**Aup1**	12.08507	3.41049	8.18947	0.60580	6.75787	1.73901	4.64565	6.91622	0.00000	9.30705	3.36798	0.09991	12.73951	0.01952	0.00000	6.05912
**Aurka**	10.33939	17.28262	20.70674	6.34807	9.29755	8.77867	5.64325	32.59828	17.25405	27.32942	15.76646	5.80462	16.73528	17.75369	26.42748	4.86095
**Aurkaip1**	14.72537	1.55508	8.58697	6.83177	8.16671	7.15133	6.12767	14.60913	6.87050	11.91536	10.94857	1.63799	12.48324	1.55430	1.11670	11.23599
**Aurkb**	7.08755	0.45310	8.27023	3.56979	4.55625	0.93470	1.14873	6.29139	6.04756	13.31073	3.57877	1.50743	3.72891	0.16482	6.70290	1.08881

**Aurkc**	7.38096	2.96949	0.76950	5.97197	0.03462	0.00000	3.40327	6.00831	6.84806	9.74470	11.80388	1.71707	3.92570	4.67388	11.85741	0.05630
**Auts2**	1.31811	1.73357	2.04869	1.44437	2.00569	0.00598	0.05485	0.67126	1.22859	2.48842	1.04005	0.38203	0.39008	2.62919	3.64877	1.17153
**AV051173**	0.04143	0.00799	0.00794	0.32936	0.00800	0.00672	0.04774	0.01089	0.54816	0.04071	0.00775	0.01563	0.08143	0.21517	0.01765	0.11173
**AV320801**	0.01342	0.00761	0.01078	0.08247	0.00936	0.00631	0.00729	0.01535	0.13857	0.00393	0.00962	0.00504	0.01871	0.01037	1.22460	0.00727
**Aven**	4.05415	4.11946	7.54798	1.10355	0.41709	0.01004	0.09267	3.97448	3.16315	6.75790	5.58211	0.26293	4.49972	6.95457	1.93121	0.23644
**Avl9**	0.00000	1.91396	1.27368	8.83395	4.24643	0.41356	0.11929	2.68268	12.09864	14.12192	7.32257	17.81432	0.00000	21.12914	10.63674	0.00811
**Avpi1**	13.29513	0.10677	0.71360	4.54132	2.43123	6.92808	7.06439	4.75481	2.38987	4.92572	3.48633	0.15664	6.75194	0.11606	0.00000	6.65567
**AW011738**	0.01338	0.00818	0.01144	1.70020	0.00915	0.00662	0.00754	0.01330	1.96889	0.16883	0.00000	0.00530	0.01328	0.01125	0.00832	0.00702
**AW146154**	0.10371	0.27533	0.43126	0.50992	0.03603	0.16116	0.16331	0.11708	0.06119	0.75347	0.12936	0.43614	0.67335	1.50671	1.05658	0.04854
**AW209491**	0.44769	0.00606	2.45016	4.34077	0.01084	0.00474	0.00454	3.03229	1.60561	4.26866	2.53831	3.59071	1.37541	1.65898	9.56802	0.15786
**AW495222**	0.01190	0.00746	0.00819	0.01130	0.00943	0.00682	0.00716	0.30057	0.01183	0.00866	0.00978	0.00636	0.00642	0.15393	0.02389	0.00737
**AW549542**	0.01338	0.00806	0.01170	0.26882	0.00919	0.00683	0.00750	0.01769	0.00559	0.23147	0.02731	0.00859	0.01207	0.02964	0.00456	0.00733
**AW549877**	2.18329	0.00000	2.70932	1.62628	1.68431	1.37714	2.56410	0.29360	0.00000	1.28472	0.52520	11.18514	2.18602	1.53757	2.51430	2.34396
**AW554918**	0.62746	0.00755	0.05856	6.27018	0.00719	0.00572	0.01902	0.12973	0.96052	0.46234	0.00779	0.03814	0.00749	0.01511	1.20436	0.00540
**AW822252**	0.01223	0.00801	0.20302	0.31976	0.00877	0.00700	0.00708	0.01098	0.00654	0.00633	0.01326	0.00796	0.41762	0.01016	0.03565	0.00692
**Awat2**	0.15410	0.00785	0.00612	0.01854	0.31038	0.00716	0.00728	0.02270	0.01258	0.01854	0.01275	0.00758	0.01064	0.01195	0.01055	0.00719
**Axin1**	3.12733	4.21884	6.84362	9.97834	0.00000	2.58096	6.38543	6.94766	10.75396	8.49666	3.76533	0.76953	2.33988	3.51120	10.28106	5.98145
**Axin2**	1.41131	0.02694	0.03377	4.14635	0.05310	1.60207	0.00591	0.00408	2.86331	0.11964	0.01707	0.00000	0.07978	0.38156	0.33275	2.11504
**Axl**	0.66462	0.00806	0.00199	0.00855	0.01729	0.00799	0.00683	0.01374	0.01187	0.35691	0.00867	0.13723	0.01877	0.33737	0.07363	0.00692
**AY074887**	0.19774	0.14487	0.27242	0.03598	0.20723	0.08765	0.13971	0.10349	0.10649	0.09678	0.81875	0.02185	0.21023	0.21095	0.19621	0.17960
**AY358078**	0.33195	0.00748	0.11024	0.13019	0.01012	0.00595	0.03745	0.15559	0.75617	0.21522	0.09372	0.03355	0.01438	0.06054	0.14583	0.09511
**AY512915**	0.01296	0.00816	0.00872	5.48525	0.00951	0.00587	0.00730	0.01246	0.00000	0.00530	0.01061	0.00000	0.01347	0.11189	3.55912	0.00649
**AY512931**	0.16285	0.17615	0.28707	4.57715	0.06190	0.79688	0.13198	0.24468	2.85509	0.35877	0.58925	0.14995	0.13832	0.73692	0.92225	0.26420
**AY761184**	0.02009	0.00788	0.00915	1.68596	0.00879	0.00647	0.00736	0.00874	2.41500	0.03063	0.12060	0.05533	0.05612	0.00526	0.06457	0.00691
**AY761185**	0.01376	0.00815	0.01226	0.50695	0.00932	0.00659	0.00750	0.01372	0.00427	0.01364	0.01361	0.00830	0.01266	0.00940	0.03879	0.00732
**Azi2**	5.63311	3.89420	1.00261	1.94028	0.00000	0.14557	3.43609	3.08472	0.16968	1.68039	2.52353	7.68349	1.80511	4.09452	2.34444	1.19149
**Azin1**	6.69806	13.51238	16.21469	18.27377	9.33602	5.55012	14.53476	17.19907	20.67123	21.89511	25.37335	22.39122	7.43621	37.85627	23.12286	4.65436
**Azin2**	0.05037	0.00735	0.37838	0.00946	0.26111	1.58540	0.00702	0.01031	0.01257	0.00560	0.00994	0.04518	0.00433	0.01052	0.02059	0.00643
**B2m**	0.00000	6.41106	5.30251	8.54169	3.75534	4.27109	2.23496	1.76362	9.59414	4.95166	2.14372	2.12971	3.59991	4.11483	3.78018	1.97936
**B3galnt1**	0.54163	0.00809	0.05474	0.24378	0.05582	0.01135	0.02663	0.00576	0.00420	0.00000	0.00398	0.00000	0.63781	2.17603	0.13238	0.00645
**B3galnt2**	0.37091	0.00334	1.18571	4.14724	4.56842	0.31855	3.43595	0.01226	1.62418	0.60486	0.00000	0.30671	0.37930	2.48005	2.70844	0.01938
**B3galt4**	0.41307	0.00688	0.01189	0.05919	0.00683	0.00593	0.00600	0.80801	0.99096	0.07160	0.91776	0.00000	1.10514	0.00361	0.18209	0.00652
**B3galt5**	0.01139	0.00769	0.00478	0.35030	0.00976	0.00644	0.00706	0.00812	0.06322	0.00805	0.00484	0.02181	0.01394	3.80550	0.00000	0.00748
**B3galt6**	0.01838	0.00792	1.69763	1.78337	0.00593	0.01073	0.00761	0.54880	3.17536	0.59088	0.38816	0.02632	0.22044	0.00000	1.77184	0.00598
**B3gat2**	0.01548	0.00868	0.30591	0.00802	0.01042	0.00564	0.00776	0.01898	0.04104	0.00476	0.00735	0.00521	0.05344	0.98882	0.00000	0.00726
**B3gat3**	4.18504	0.01134	3.39029	1.40600	0.51862	0.00140	1.86555	1.67376	1.54005	6.21009	1.28936	0.10752	2.62150	0.15299	0.57545	2.11741
**B3glct**	4.71868	2.04934	2.26973	0.76356	5.94666	0.01170	5.11207	0.17335	0.32640	0.53594	0.74361	1.88988	0.07614	0.01546	0.08934	0.47649
**B3gnt2**	2.15062	0.00335	0.59733	7.67219	1.78284	0.02308	1.37410	0.71478	0.71973	0.79573	0.31451	0.00871	0.24627	0.38836	3.84084	0.01052
**B3gnt3**	0.30859	0.00780	0.00414	0.66698	0.01649	0.01511	0.00722	0.07074	0.48279	0.00725	0.01256	0.00690	0.14552	0.01061	0.01231	0.01448
**B3gnt4**	0.05929	0.00856	0.00985	0.01059	0.01493	0.00680	0.00751	0.01324	0.01238	0.01934	0.01285	0.00812	0.01250	0.01214	0.01072	0.00748
**B3gnt5**	0.09451	0.00584	0.84355	0.00805	0.10150	0.00905	3.26782	0.00553	0.01107	0.00000	0.02614	0.00339	0.62743	0.02276	0.28466	2.07581
**B3gnt6**	0.01202	0.00848	0.00701	0.01129	0.02198	0.00708	0.00819	1.19300	0.01110	0.00337	0.00535	0.00684	0.00567	0.01883	0.00000	0.00739
**B3gnt7**	4.07725	1.48401	0.19601	0.11711	2.44584	0.00112	1.36437	0.00000	1.27672	1.82383	0.06251	0.04648	0.03150	0.99833	0.00000	4.43199
**B3gnt8**	0.04703	0.00834	0.00400	0.00880	0.01431	0.00750	0.00673	0.56981	0.01139	0.15808	0.05808	0.09048	1.37016	0.01420	0.00000	0.00689
**B3gntl1**	0.01306	0.00768	0.00388	1.77664	0.00915	0.00648	0.03692	0.00000	0.00000	0.06758	0.89803	0.00985	0.00509	0.00116	1.49719	0.04340
**B4galnt1**	0.03207	0.01771	0.30741	0.09303	0.00799	0.15329	0.00539	4.70801	0.11864	1.79909	2.34876	0.28478	2.33459	0.03038	0.68374	0.00559
**B4galnt2**	1.11510	0.01798	0.44556	5.86489	0.45467	0.09071	0.09714	0.29046	7.84318	0.83251	1.48646	0.57834	0.25882	2.02346	1.03528	0.10037
**B4galnt3**	0.00685	0.23211	0.00803	0.15623	0.00857	0.00673	0.00703	0.68127	0.13681	0.21667	0.00685	0.00505	0.00558	0.00855	0.00000	0.03649
**B4galnt4**	0.23804	0.00762	0.00665	0.06583	0.00735	0.00706	0.00717	0.01180	0.01134	0.82197	0.01208	0.01652	0.01165	0.00968	0.02006	0.00707
**B4galt1**	9.62892	2.05583	4.18045	0.00000	1.89779	3.20900	2.42342	3.32763	0.88343	1.34638	0.39136	0.17336	1.67219	0.00667	0.00000	4.69906
**B4galt2**	0.00525	0.00761	0.01080	2.36438	0.00691	0.00601	0.00711	0.01150	0.32560	0.00309	0.00940	0.03566	0.02456	0.00575	0.44556	2.93125
**B4galt3**	5.52590	2.54540	5.70699	10.48515	0.04538	6.55719	3.28923	11.36495	13.10988	14.89435	6.70317	1.08860	9.78920	0.16513	1.72437	7.24453
**B4galt4**	0.01263	0.00763	0.01232	2.23848	0.00878	0.00624	0.00718	0.00981	0.22852	2.02006	0.00865	0.00000	0.01063	0.00152	0.16189	0.00702
**B4galt5**	4.07352	1.09751	4.74955	3.13114	0.05817	3.38466	0.05121	0.02295	3.57311	0.22572	1.03603	0.10511	3.21267	0.00000	0.23749	0.44167
**B4galt6**	5.09259	1.83486	0.57319	14.13274	8.37927	2.20725	2.74423	0.25606	3.34797	2.31078	1.35339	0.16868	1.41959	0.25274	3.54834	2.55119
**B4galt7**	0.76080	0.02664	1.30790	3.02692	0.61771	0.04424	0.28113	0.00000	4.86234	1.57238	5.01072	0.33479	1.06432	2.19651	0.65317	0.32832
**B4gat1**	0.12891	0.00712	0.46978	0.44813	0.03389	0.23041	0.00638	0.92725	0.16639	0.75832	0.68259	1.59822	0.60796	0.00860	0.00000	0.01370
**B9d1**	2.82076	0.24711	1.21858	4.29831	3.54851	0.00223	0.05016	0.14441	2.32605	0.00000	0.08906	0.04194	0.51866	1.17106	1.87362	1.85548
**B9d2**	3.17239	0.00433	0.00443	0.00436	0.13038	0.00578	0.00506	0.00066	0.35031	0.72811	0.02388	0.01171	0.15818	0.01037	0.01333	0.02021
**Baat**	0.01872	0.00787	0.00639	0.35603	0.01027	0.00708	0.00717	0.00084	0.00546	0.00000	0.34781	0.24531	0.00876	0.00739	0.00000	0.00719
**Babam1**	8.94342	0.46203	3.14579	1.68628	5.51744	3.99619	0.04414	3.79467	0.28240	3.50085	3.83851	5.51157	9.38126	0.00022	0.26339	3.80287
**Bace1**	1.21648	0.00000	4.89630	4.44613	4.51308	2.65717	2.94607	5.42525	7.81029	6.57423	4.93063	0.20883	0.45646	4.00048	9.68849	1.19147

**Bace2**	0.01308	0.00755	0.01180	0.00701	0.00890	0.00659	0.00729	0.01054	0.01129	1.23533	0.01116	0.00394	0.01143	0.01425	0.10234	0.00778
**Bach1**	6.56684	5.48163	3.08864	6.89381	2.15099	4.52430	0.92860	8.45888	7.25756	8.13059	14.22640	4.10014	3.44945	27.97057	8.77469	1.23952
**Bach2**	0.99736	0.01940	0.07910	0.10531	0.07391	0.02166	0.02713	0.00675	0.08694	0.22886	0.28142	0.02376	0.13822	0.11851	0.45634	0.00644
**Bad**	10.18517	5.05304	10.51828	0.91114	4.59783	5.71706	5.69049	7.26775	2.09683	6.94837	4.11424	0.43771	7.11311	0.00000	0.19699	3.12409
**Bag1**	10.92273	3.07916	12.13745	10.98866	7.55166	7.61290	7.51104	4.89145	25.23020	17.14654	18.44011	6.16578	9.10953	8.09738	4.44625	1.64822
**Bag2**	0.08696	0.09688	0.14866	0.25869	0.00761	0.03550	0.00662	0.00788	0.00491	0.84310	0.01062	0.02419	0.89359	0.01098	0.01271	0.01453
**Bag3**	4.77907	4.30025	9.34533	3.93725	5.55711	7.03460	3.72283	10.22096	22.29699	4.32112	15.04101	0.25741	12.27973	5.81699	7.19836	3.24542
**Bag4**	8.75950	8.59732	2.46404	4.88161	4.31686	4.87231	6.17185	4.23207	4.05960	4.16817	1.86531	7.07088	6.84218	8.18101	2.86075	1.45576
**Bag5**	4.22049	3.26431	6.51702	19.16861	0.22680	4.43364	6.77529	6.86556	29.25787	9.87517	5.62562	0.94020	4.19888	2.38063	11.53686	5.86482
**Bag6**	12.28398	0.95648	3.08829	6.47654	4.73258	5.52069	3.18692	19.54768	9.16286	19.25586	13.45222	1.78284	14.67198	0.63144	3.07169	12.07146
**Bahcc1**	0.01367	0.00799	0.01237	0.41363	0.00929	0.00659	0.00752	0.01393	0.05322	0.01399	0.01312	0.00778	0.01260	0.00882	0.02659	0.01464
**Bahd1**	2.68170	0.00479	0.31100	5.23939	0.00417	0.05288	0.01370	0.03684	2.74911	0.53133	2.17929	0.06634	1.78705	0.35374	0.76785	0.72530
**Baiap2**	10.27282	0.00000	6.46352	2.07725	8.22923	0.00699	7.89868	15.53575	3.20579	4.07974	5.65806	0.17177	0.71172	0.00172	0.00000	3.61060
**Baiap2l1**	1.23473	0.55324	1.00606	2.47930	0.00345	0.00763	0.49355	4.33677	0.80550	1.54388	2.22263	0.25755	1.47446	0.04304	1.31668	0.01829
**Baiap2l2**	0.10686	0.00881	0.00782	1.04117	0.01534	0.00691	0.00745	0.01257	1.00648	0.02271	0.02202	0.01853	0.01269	0.01041	0.00215	0.00728
**Baiap3**	0.54228	0.00659	0.00972	0.00878	0.00670	0.00784	0.00662	0.31547	0.22164	0.02797	0.30713	0.00450	0.00653	0.01246	0.00017	0.00673
**Bak1**	0.06321	0.00772	0.01424	0.02023	0.00796	0.00638	0.00749	0.01712	0.04500	0.01300	0.03898	0.01837	0.02426	0.01215	0.00910	0.00725
**Bambi**	1.00805	0.00343	0.00115	7.00541	6.73992	0.00645	0.02563	0.98183	5.84576	0.16136	0.00000	0.11059	0.01663	0.00697	0.12499	0.98303
**Bambi.ps1**	0.01275	0.00812	0.02877	0.71389	0.00911	0.00709	0.01799	0.01396	0.04603	0.24665	0.01045	0.00724	0.01722	0.08108	0.00629	0.00700
**Banf1**	23.39191	6.90456	16.60330	7.16399	10.64153	7.72434	10.56059	16.87151	6.50040	22.24187	19.13185	3.25238	14.73089	2.23250	5.29947	9.92697
**Banp**	0.26013	0.03752	3.50558	2.97472	5.44926	0.07648	2.04748	2.36085	7.07019	7.21742	1.91487	0.13615	6.90604	0.20041	0.49716	1.45648
**Bap1**	7.74874	1.00454	4.88183	9.90165	6.22140	1.75785	2.64318	8.76292	5.76231	9.16459	4.63786	4.24655	5.49586	0.13258	3.11618	2.91259
**Bard1**	2.16659	1.41606	1.19655	4.38850	0.00326	0.07369	1.23790	0.82705	12.01448	1.90011	3.65093	5.80658	1.84278	0.12552	1.72325	1.19309
**Barx2**	0.01387	0.01878	0.04061	0.00508	0.00918	0.00616	0.04978	0.00920	0.01106	0.01993	0.66676	0.01662	0.00861	0.01053	0.00204	0.00730
**Basp1**	1.98070	0.11049	0.08527	0.23375	0.92519	1.44595	0.00500	0.01891	0.43836	0.12659	0.29598	0.05671	1.27646	0.00598	0.02589	1.75657
**Batf**	0.02617	0.00780	0.12663	0.00918	0.00852	0.00576	0.00735	0.01293	0.02694	0.01842	0.01239	0.02776	0.01090	0.01250	0.00796	0.00722
**Batf3**	0.01372	0.00814	0.01203	0.15097	0.00927	0.00663	0.00753	0.01354	0.24438	0.01327	0.02577	0.00781	0.01242	0.01158	0.00894	0.00737
**Bax**	1.42752	0.01162	3.69041	3.01347	8.68689	2.39623	0.01972	1.50662	1.57547	4.49645	5.63283	0.06419	7.00353	0.34800	0.36373	3.00261
**Baz1a**	4.02957	9.55149	8.44603	13.57274	4.55346	7.37058	7.19316	11.46991	14.24445	30.26625	15.65079	23.96122	11.20823	35.68636	26.69645	2.85868
**Baz1b**	8.11302	2.02601	7.48146	12.82597	0.12922	1.38335	5.34499	11.18417	9.85115	10.43546	3.93183	0.76406	4.74666	1.37097	8.85034	3.07361
**Baz2a**	11.82556	8.01211	6.73575	13.05664	7.33386	2.81203	12.48799	16.25188	14.42292	21.14999	11.65124	8.14718	6.65849	10.66875	20.18759	3.59719
**Baz2b**	6.88227	5.77538	0.08924	3.41191	2.20924	0.00000	1.70904	0.50092	3.62374	1.99773	0.32709	0.19717	0.46307	0.25659	5.75350	0.00000
**BB283400**	0.04979	0.00757	0.05987	0.23272	0.00806	0.00577	0.01787	0.00404	0.09305	0.04714	0.48616	0.03651	0.11055	0.03824	0.07515	0.02149
**BB287469**	0.01829	0.01877	0.16248	10.58503	0.03841	0.00403	0.02736	0.05776	9.04846	0.00000	0.00000	0.00000	0.03887	2.20913	1.09070	0.01987
**BB557941**	0.10064	0.00789	0.02783	3.78995	0.01477	0.00549	0.00701	0.00897	1.45333	0.09887	0.00000	0.07458	0.01296	0.01942	1.85040	0.00711
**Bbc3**	1.05826	0.00624	0.34757	0.01254	0.82455	0.00515	0.31085	0.03634	0.23214	0.88096	0.00793	0.02965	0.39237	0.01496	0.03211	0.00599
**Bbip1**	5.70203	1.15334	4.60569	3.60024	3.21167	1.25809	0.82107	2.55110	3.57877	1.16450	2.04033	3.30503	4.07904	1.17128	1.63813	0.07728
**Bbof1**	0.00388	0.00550	0.00000	0.26834	1.62194	0.00670	2.64155	0.00501	2.93774	0.44747	0.00000	0.01174	0.01002	1.57172	0.09885	0.03576
**Bbs1**	0.03761	1.73088	0.08100	3.28880	0.08275	0.02209	0.01657	0.00402	5.23934	0.95900	0.08228	0.00000	0.02684	0.02557	1.61527	0.13573
**Bbs10**	2.87629	0.01427	0.00411	0.50925	0.00271	0.00438	0.00506	0.00287	0.00482	0.00658	0.01169	0.00456	0.01109	0.00734	0.54342	0.00414
**Bbs12**	0.05768	0.00837	0.05418	0.10699	0.01309	0.01168	0.00735	0.01229	0.31264	0.01151	0.01182	0.01826	0.01171	0.00976	0.03785	0.01480
**Bbs2**	0.54982	0.00749	1.28767	10.92669	0.01004	0.01150	0.00525	0.00000	9.70623	6.26066	0.76021	1.41785	1.71170	1.13894	4.42972	0.00310
**Bbs4**	1.15925	5.18986	1.00448	1.96532	0.01258	0.00715	1.58301	0.37714	1.58488	0.52809	0.46509	0.00000	0.14052	6.48743	5.28675	1.75305
**Bbs5**	1.46089	4.19656	2.36746	1.84585	0.01073	2.88588	2.35876	1.98675	2.03969	1.49574	2.92586	1.22813	3.56655	5.20553	2.70656	0.00695
**Bbs7**	0.01374	0.00772	0.01183	0.23256	0.00945	0.00648	0.00739	0.01152	0.08462	0.01465	0.02134	0.00628	0.01266	0.00648	0.49201	0.00738
**Bbs9**	0.03102	0.00890	0.00578	3.28081	0.01270	0.00773	0.00785	0.11945	0.12053	0.09446	1.07385	0.00234	0.06290	0.00810	0.17206	0.00683
**Bbx**	0.00000	2.64813	0.00000	0.00000	0.03404	0.09613	0.01537	0.20132	1.02377	1.47617	0.58366	0.45810	3.10882	18.53137	5.54102	0.03353
**BC002163**	4.43766	0.24079	3.57172	0.80034	3.36278	1.15876	0.37511	0.58424	0.23911	2.32908	8.04445	0.20516	0.92004	0.00950	0.74303	0.93573
**BC003331**	2.65896	0.00003	1.18073	1.90209	0.81870	3.52631	2.69661	4.37426	1.46438	1.97355	7.89251	0.45464	3.35914	8.26375	4.55963	2.23997
**BC003965**	6.53396	0.06715	2.59692	6.71459	4.92306	5.10331	2.27579	8.35688	9.12475	10.68347	5.81546	0.66701	6.11165	3.62590	2.56850	5.01027
**BC004004**	6.18567	3.69486	2.23838	2.10851	4.53728	3.80916	2.46720	3.07112	0.49491	1.57129	1.58950	4.39524	4.13026	0.89966	1.38060	0.07378
**BC005537**	9.32221	9.82798	7.69174	6.02355	6.90777	3.35518	5.59089	10.11501	6.75892	6.13736	1.90632	2.59393	1.27792	8.82255	6.26511	0.97636
**BC005561**	0.41754	0.00686	0.50331	1.35972	0.00612	0.00518	0.00665	0.23213	0.00000	1.13840	0.96879	0.00000	0.00000	1.79033	2.39183	0.01375
**BC005624**	9.21560	4.32207	6.44884	1.25282	6.27213	0.01511	6.69547	5.31543	3.29861	5.96483	2.66808	1.34391	5.46503	3.27170	5.51247	0.48166
**BC006965**	0.01896	0.00765	0.01474	0.02538	0.00874	0.00719	0.04743	0.00981	0.02949	0.01658	0.02442	0.02525	0.02465	0.28355	0.38260	0.05050
**BC017158**	3.96022	0.00320	0.24808	0.49721	0.26889	0.52092	0.19648	0.43183	0.36853	0.00000	0.00758	0.00301	0.99335	0.01431	0.00000	2.32371
**BC017643**	1.51737	0.00513	2.16229	0.58781	0.01683	5.71920	0.00484	0.62309	10.22527	0.08143	0.64480	2.44236	0.00000	1.78253	6.23856	0.02411
**BC018473**	0.04167	0.00680	0.03957	0.29656	0.00890	0.00614	1.18039	0.02333	0.42105	0.02719	0.12643	0.06986	0.02223	0.27710	0.06509	0.00723
**BC020402**	0.01078	0.07754	0.00599	0.00638	0.00904	0.00668	0.29758	0.05967	0.14176	0.01223	0.03374	0.00012	0.00963	1.57376	0.00656	0.00751
**BC021614**	1.94569	0.02544	0.05749	1.68670	0.00423	0.00656	0.00473	0.00000	1.44496	1.25648	0.86812	0.02124	0.87376	0.08779	0.76015	0.00439
**BC021767**	1.92992	0.00454	0.61990	2.36694	0.09816	2.75154	1.50264	2.21494	1.62657	1.35966	1.15605	0.10792	0.32508	0.85728	0.69064	0.18615
**BC021891**	0.01368	0.00778	0.01197	0.58196	0.00938	0.00650	0.00736	0.01144	0.06193	0.18795	0.02650	0.00686	0.01211	0.03290	0.01124	0.00734

**BC022687**	0.02040	0.00712	0.04333	0.37778	0.01708	0.01149	0.17189	1.06086	1.24636	5.16138	0.33607	0.08115	0.10738	0.00486	0.88193	0.00700
**BC024386**	0.29968	0.00726	0.23924	1.29098	0.05484	0.01791	0.02686	0.00000	1.75325	0.02785	1.74548	0.05128	0.07686	0.63494	0.04022	0.03573
**BC024978**	0.19862	0.07250	0.12944	0.48669	0.02861	0.02285	0.00695	0.00422	1.08252	0.31442	1.02415	0.20979	0.03417	0.22285	0.75864	0.63952
**BC025920**	0.01437	0.00782	0.00665	1.28318	0.00891	0.00621	0.00693	0.00783	2.82249	0.19113	0.00000	0.00000	0.38902	0.73344	0.76124	0.00681
**BC026585**	1.92558	1.40253	1.59712	0.69081	0.11295	0.12518	0.00512	2.07537	0.46670	2.03511	0.00000	0.02280	0.24592	0.00687	0.00000	0.55754
**BC028528**	0.32658	0.00693	0.26861	0.25533	0.37952	0.00684	0.20184	0.26430	0.04075	0.01510	0.21000	0.03063	0.28645	1.15025	0.23693	0.00678
**BC029214**	0.00807	0.00746	0.22950	0.01097	0.01870	0.00802	0.00707	0.49376	0.01175	0.02937	0.00977	0.01540	0.00486	0.01398	0.00132	0.02241
**BC029722**	0.01318	0.00802	0.01138	0.00913	0.00903	0.00680	0.00742	0.01182	0.01202	0.35996	0.01136	0.03102	0.09284	0.01167	0.01060	0.00731
**BC030336**	0.95412	0.00709	0.00000	1.00343	0.01599	0.00502	0.00579	0.21785	0.61342	1.01937	0.59249	0.27833	0.37769	4.32765	2.14250	0.00591
**BC030867**	4.25677	0.03932	10.20074	11.11127	3.62968	8.10702	2.56449	16.90345	14.60150	17.92691	8.83046	1.65299	3.34571	0.89116	11.16934	6.73563
**BC031181**	13.62988	6.72391	19.82377	9.03860	9.92003	6.25822	10.70337	17.46169	5.96151	19.11907	8.16949	10.93922	13.00649	0.74743	5.41375	8.20157
**BC031361**	0.04903	0.00827	0.00419	0.83502	0.00794	0.00703	0.00818	0.46292	2.12082	0.00266	0.00000	0.00429	0.00577	0.00908	0.43735	0.00683
**BC037034**	1.75793	0.00547	0.05913	0.93690	0.01115	0.00426	0.24790	2.55866	1.02535	0.86053	0.72459	0.00001	0.74665	0.00156	0.10397	0.00501
**BC037704**	0.01253	0.00809	0.01459	0.80733	0.00869	4.67704	0.00690	0.00270	5.60529	0.92719	0.13319	0.00000	0.00000	0.02399	0.01516	0.00492
**BC039771**	0.21990	0.03845	0.37014	0.18633	0.05910	0.01761	0.08794	0.04818	0.08621	0.10468	0.87293	0.07078	0.41298	0.49827	0.21029	0.05005
**BC046251**	0.01633	0.00776	0.03078	0.03144	0.01840	0.00671	0.00738	0.02782	0.11042	0.03367	0.03838	0.00715	0.01339	0.07228	0.02792	0.01509
**BC048403**	0.09356	0.19607	0.02240	0.02972	0.01224	0.00629	0.00731	0.07342	0.19940	0.54084	0.03938	0.00551	0.01689	0.01686	0.04737	0.00747
**BC048507**	0.48790	0.03825	0.32399	0.23990	0.42721	0.09663	0.17755	0.13741	0.63721	0.36801	0.17594	0.04362	0.13587	0.04597	0.00000	0.32182
**BC048546**	0.01201	0.00726	0.68245	0.00443	0.00735	0.01128	0.44442	0.00812	0.01062	6.22243	0.00826	0.00000	0.00891	0.03353	0.00000	0.01478
**BC048679**	0.91134	3.14537	1.88789	0.00149	0.38812	0.65998	3.45383	0.99801	0.00949	1.14660	1.90395	0.00761	6.63847	0.00506	0.00491	1.20228
**BC049715**	0.02621	0.97270	0.00526	0.03457	0.00854	0.00704	0.01722	0.06237	0.11274	0.07797	0.03758	0.01639	0.06895	0.07907	0.05252	0.00700
**BC051019**	0.00000	0.00569	0.98845	0.87647	3.96734	0.01721	0.24126	0.35604	0.35887	3.14548	0.00516	0.01752	0.87207	0.01100	0.05708	2.95363
**BC051142**	0.00227	0.03802	0.10937	0.00557	0.01047	0.00850	1.32971	1.75738	0.02930	0.00000	0.20509	0.02090	0.00372	0.01313	0.00000	0.00714
**BC051226**	0.02036	0.00812	0.01024	0.24295	0.00836	0.00652	0.00724	0.03056	0.42009	1.54492	0.00822	0.02565	0.40547	0.01222	0.00396	0.04267
**BC051537**	0.01154	0.00805	0.08786	0.02516	0.00903	0.00884	0.00725	0.02801	0.05097	0.01941	0.01138	0.00834	0.12006	0.00835	0.07657	0.00726
**BC051665**	0.01103	0.00767	0.01064	0.00797	0.02460	0.00638	0.00716	0.00981	0.01209	0.65904	0.01193	0.03368	0.01776	0.03251	0.00445	2.08510
**BC052040**	4.87324	6.33335	5.43854	0.00000	0.03009	0.25327	0.03966	6.47557	0.13945	5.61642	7.53387	1.09938	1.67927	0.66641	0.42412	0.03999
**BC052688**	8.82523	14.01165	19.58819	10.94618	9.07587	9.14270	13.41704	12.75338	5.46016	11.13814	11.03207	13.09076	16.01783	22.19627	10.97181	6.81691
**BC053393**	0.00000	10.86533	6.86946	0.21422	0.13257	11.85931	8.67123	0.00788	0.42839	0.00000	0.00000	0.05463	4.93951	0.01786	0.05613	4.57321
**BC055324**	3.45632	8.28994	3.60294	1.06170	4.65726	0.00226	0.95548	9.17037	0.49705	7.53881	2.73583	5.54285	3.21842	5.44633	10.50222	3.03202
**BC064078**	0.01247	0.00807	0.08807	0.22890	0.00895	0.00906	0.00739	0.01220	0.19022	0.06224	0.01025	0.00808	0.04681	0.00602	0.22280	0.00730
**BC065397**	0.00000	3.02998	0.87417	0.00483	0.04872	0.00789	2.66495	0.35375	0.01238	0.00000	0.21766	0.00399	0.00333	0.00566	0.01492	0.00605
**BC068281**	0.01500	0.01783	0.53886	0.54201	0.02636	0.01140	0.18085	0.10115	0.63900	0.18823	0.00000	0.41578	0.03033	0.03172	0.29355	0.00703
**BC080695**	0.01260	0.01828	0.00912	2.86663	0.00910	0.00604	0.00722	0.01332	5.66646	0.00000	0.00000	0.15397	0.01302	0.05755	0.08836	0.00647
**BC100530**	0.07783	0.00797	0.03232	0.10952	0.00852	0.04030	0.00759	0.01609	0.41124	0.02270	0.03880	0.00741	0.03388	0.01311	0.00405	0.00723
**BC147527**	0.00697	0.00823	0.00812	8.72124	0.00916	0.00495	0.02926	0.51913	11.31265	0.39888	0.00008	0.00000	0.00619	0.00000	0.45923	0.00504
**Bcam**	0.02122	0.00791	0.01295	0.06343	0.00932	0.00715	0.00766	0.01571	0.01136	0.05586	0.01531	0.00735	0.07642	0.01475	0.03173	0.01584
**Bcan**	0.01325	0.00731	0.00437	0.16127	0.00899	0.00701	0.00696	0.00000	0.60344	0.21554	0.30573	0.40986	0.01616	0.42564	0.18834	0.00760
**Bcap29**	5.24945	4.19483	3.50008	1.63407	0.00000	0.05703	4.47944	1.03377	3.44615	5.02085	4.40196	0.64080	2.29788	8.49867	2.87439	0.01057
**Bcap31**	17.69664	12.20695	26.32940	8.53183	9.19631	3.53018	11.96515	19.95572	9.13326	14.28190	15.08752	14.45006	17.34470	23.13972	19.61936	15.42335
**Bcar1**	1.12373	0.00370	0.09998	5.50635	5.22497	0.00512	0.00582	2.64278	1.29221	0.72374	0.48583	0.07284	0.00000	0.00000	0.00000	0.01920
**Bcar3**	0.01095	0.00772	0.00645	1.78585	0.00926	0.00629	0.00706	0.00940	0.00236	0.35146	0.00000	0.03566	0.01178	1.32298	0.78345	0.13859
**Bcas1**	0.01757	0.00791	0.01130	0.53322	0.00881	0.00648	0.00729	0.01094	1.18268	0.40881	0.00000	0.00385	0.01253	0.00827	0.24749	0.00760
**Bcas2**	17.85537	16.05001	16.68721	10.40821	13.03536	5.23912	16.12178	21.16512	7.00569	17.80592	17.75798	13.73976	21.25929	8.43122	1.67330	14.16605
**Bcas3**	10.04854	3.73146	9.21791	3.93431	3.78194	4.87142	1.27185	17.00423	6.36116	12.53705	15.68404	2.10287	8.53728	7.96867	9.33192	6.29722
**Bcas3os1**	0.04781	0.00807	0.03535	0.16658	0.00975	0.00818	0.01821	0.11915	0.23866	0.09643	0.27345	0.00605	0.02197	0.15974	0.11977	0.00734
**Bcat1**	17.31323	6.58585	20.65379	3.91909	19.20495	9.41488	11.23623	30.28744	4.10218	28.83802	29.26491	6.50635	20.86586	22.23090	18.29923	3.81744
**Bcat2**	5.67593	0.19115	9.36878	0.99814	2.49885	13.92884	2.51065	6.76811	1.80357	5.19401	4.72236	0.46911	9.83411	0.43264	1.56431	2.78484
**Bccip**	5.55445	8.77170	4.49986	0.00000	9.29807	5.74492	0.33435	5.82954	0.08021	1.56578	0.00000	0.09626	4.10860	8.78253	5.02906	2.81624
**Bcdin3d**	2.37437	2.22014	4.65519	1.12021	0.00059	0.00282	1.30053	2.53988	1.52809	1.23593	0.00000	0.02448	0.70643	0.00445	0.20735	1.11103
**Bckdha**	6.01729	4.01750	2.79597	0.91081	0.09762	0.08233	4.21870	9.94952	0.03253	11.35314	2.89826	0.47145	5.06942	0.08881	0.00000	1.25384
**Bckdhb**	1.17359	2.20770	3.53843	0.77677	0.37692	1.49326	3.07142	7.01934	1.69284	7.54492	0.44650	4.65976	2.39680	6.02964	3.56610	0.47071
**Bckdk**	12.32543	0.50629	5.37820	1.24515	0.38918	2.23674	5.85450	10.76024	1.80500	8.60679	3.59723	0.47554	7.04483	0.39009	2.18396	9.77835
**Bcl10**	6.04049	0.40040	1.20338	1.65264	2.11519	5.04028	3.33895	4.49435	0.49127	2.03590	0.38767	0.94912	1.45337	2.17724	2.14049	1.78776
**Bcl11b**	0.44806	0.23356	0.00356	0.02090	0.01670	0.00723	0.00676	0.00000	0.05940	0.20121	0.31883	0.06326	0.00845	0.10653	0.00805	0.00671
**Bcl2**	0.01344	0.00824	0.01156	0.04660	0.00916	0.00687	0.00770	0.01703	0.01170	0.17228	0.02457	0.02085	0.01912	0.01039	0.01881	0.00737
**Bcl2l1**	2.35289	2.08898	4.04548	2.50169	3.02715	6.96130	1.60096	6.06395	1.77458	6.87674	2.00660	0.24140	2.80799	0.00482	0.00000	6.35178
**Bcl2l10**	0.01937	0.00811	0.00326	24.71215	0.00808	0.00537	0.01726	0.04421	6.02371	0.37878	1.70741	0.01816	0.00000	0.08790	0.24785	0.24016
**Bcl2l11**	1.64801	0.00489	0.80452	7.03593	0.04028	0.08521	0.02591	1.31108	9.63805	3.58679	4.08471	3.16811	1.71298	1.94785	0.80170	2.10185
**Bcl2l12**	0.01658	0.00725	0.28895	0.00338	0.00736	1.90428	0.06526	0.10717	0.11795	1.73258	0.17001	0.03106	1.00908	0.01686	0.00020	0.97912
**Bcl2l13**	6.58805	0.16670	1.78106	13.43023	0.44777	1.87420	1.48967	5.49400	15.84763	13.94567	8.78790	9.24330	4.86119	10.64746	14.84287	3.14816
**Bcl2l14**	6.66739	5.84145	1.06624	1.81810	0.10663	0.00887	1.33888	9.01283	6.51093	17.98461	10.02535	3.95309	3.41443	5.87110	7.61808	0.01518

**Bcl2l15**	0.00968	0.00792	0.01029	0.04425	0.00929	0.00681	0.00747	0.35380	0.45080	0.75172	0.00600	0.00501	0.00657	0.03055	0.00000	0.00757
**Bcl2l2**	0.67226	0.00699	0.00000	0.95156	0.22835	0.77237	0.00638	0.42145	0.02273	1.08054	0.07159	0.10046	0.75462	1.96922	4.32558	0.04110
**Bcl3**	5.67138	0.13569	0.00000	0.27049	5.39444	2.39343	1.90613	1.33435	1.31914	1.05101	0.90158	0.03487	0.78426	0.00335	0.00000	0.24380
**Bcl7a**	2.10726	0.00531	1.56841	0.08143	0.00415	2.24309	0.00458	1.21241	0.78795	6.30086	2.58495	0.22323	0.00000	0.35990	0.79850	1.25806
**Bcl7b**	4.79461	2.42293	2.83162	1.33260	0.02439	0.00182	0.02445	2.78618	1.04500	4.90200	1.58634	0.28798	1.43861	0.00810	0.22196	1.28492
**Bcl7c**	0.97796	0.06590	0.63763	13.76559	0.04841	0.02208	0.00597	0.22404	3.80405	2.00705	1.03387	0.10314	0.35445	0.01709	0.61953	0.00352
**Bcl9**	0.01270	0.00783	0.04230	2.08346	0.00917	0.01157	0.00744	0.01175	0.83526	0.28921	0.00684	0.00527	0.01157	0.00220	0.30532	0.01414
**Bcl9l**	1.44086	0.02948	0.07142	0.00971	0.01711	0.00425	0.00652	0.00759	0.01241	0.18111	0.01150	0.00698	0.01568	0.03233	0.00721	0.48740
**Bclaf1**	13.08742	13.09920	11.38077	19.14134	9.39946	7.48764	10.45169	17.81240	25.39272	19.44078	17.89277	17.04972	9.08616	32.32967	26.41405	3.90181
**Bco1**	0.00335	0.01731	0.06098	0.11623	0.02884	0.01318	0.03642	0.34019	0.02570	0.81031	0.00004	0.00000	0.02361	2.29147	0.00000	0.01464
**Bco2**	0.01247	0.00791	0.01164	0.00876	0.00922	0.00647	0.00713	0.00990	0.01233	0.39999	0.01230	0.00606	0.21092	0.07109	0.00383	0.00734
**Bcor**	4.42271	0.00000	0.28691	4.75723	4.07971	0.10105	0.86288	3.92035	3.34140	2.11160	0.38987	0.71828	1.61667	0.00000	3.91706	2.41451
**Bcorl1**	0.77481	0.00654	0.88000	7.95822	0.27435	0.00377	0.00664	1.34493	4.39831	0.03237	0.00000	0.01483	0.00229	0.00498	0.76945	0.00482
**Bcr**	4.48431	2.18439	0.00000	3.43794	4.06409	2.09819	0.01303	0.40350	2.44212	1.04526	0.00000	0.76540	0.77273	0.00000	0.45476	0.44962
**Bcs1l**	7.84920	6.36659	3.68581	1.02311	3.54962	2.07239	3.89483	1.59375	0.01605	2.84643	2.20895	1.11930	4.38000	0.08733	2.25275	1.53732
**Bdh1**	1.97738	1.05790	2.73608	0.82565	2.44362	1.61025	3.38852	6.14229	3.47927	2.80045	1.80192	6.27622	7.06484	0.24683	0.24104	3.10525
**Bdh2**	0.01362	0.00779	0.01216	0.10629	0.00938	0.00647	0.00740	0.01175	0.56878	0.15818	0.02482	0.00573	0.01228	0.00692	0.04951	0.00742
**Bdp1**	1.64459	0.00247	2.21760	4.59522	0.00000	1.69878	0.47281	4.06086	6.88596	6.28947	3.75681	7.03041	0.54163	12.21865	10.08853	0.56190
**Becn1**	6.22462	4.08813	1.98129	4.66857	5.83303	0.98553	2.69818	6.71314	1.94062	9.28779	0.57601	6.40818	7.14515	7.43241	5.74088	7.01847
**Becn2**	0.32532	5.53145	17.72167	8.75904	2.33104	3.15003	4.14739	17.55006	14.85996	13.94275	12.70382	2.09819	11.15769	14.98585	15.14524	0.02394
**Bend3**	1.44489	0.65923	0.22634	9.07877	0.95776	1.89587	1.00621	3.10674	13.28079	2.68982	6.57110	7.21931	1.15004	14.40405	9.29916	0.02998
**Bend4**	3.43949	0.01458	0.00000	0.61273	0.00000	7.33976	2.07335	0.32612	0.26001	9.07143	1.52632	0.31486	2.49856	0.04598	2.36965	0.05737
**Bend5**	0.04326	0.00849	0.00946	0.23066	0.01352	0.00658	0.00737	0.02212	0.14875	0.00982	0.01265	0.00762	0.00847	0.01107	0.00978	1.57261
**Bend6**	0.00959	0.00786	0.00563	0.50966	0.00883	0.00637	0.00705	0.00847	0.00347	0.00000	0.01053	0.00868	0.00968	2.35871	0.19333	0.93848
**Bend7**	0.70456	0.00722	0.03598	0.00487	0.01395	0.00505	0.03519	0.36671	0.25167	0.00000	0.02153	0.00534	1.13046	0.01675	0.00185	0.00602
**Best2**	0.01240	0.00744	0.00611	4.62635	0.00856	0.00675	0.00655	0.00000	0.78715	2.25869	1.25402	0.00000	0.01322	0.00000	0.04568	0.00633
**Bet1**	2.90338	0.06169	1.80252	3.36069	7.18800	0.92787	0.17146	0.86758	0.52395	1.68473	4.34684	3.92247	1.22646	0.07954	1.93226	0.36308
**Bet1l**	0.45236	3.85485	3.41502	3.31582	6.11410	2.31821	0.00472	4.53384	0.38701	4.00981	0.83795	0.27627	0.93816	0.11287	6.33140	2.75447
**Bex1**	0.02991	0.00788	0.00627	2.58970	0.01390	0.00728	0.00703	1.34397	0.69174	0.29426	0.00000	0.04738	0.26045	0.00853	0.00000	0.00736
**Bex2**	0.23034	0.00775	0.00618	0.38956	0.01652	0.00773	0.00729	0.10158	0.00615	0.08059	0.01502	0.00709	0.04565	0.01021	0.00473	0.00748
**Bex4**	0.24283	0.00808	0.00426	0.00727	0.07016	0.00747	0.00652	0.31349	0.01139	0.98749	0.00932	0.02338	0.85749	0.01184	0.00739	0.00722
**Bex6**	0.00000	0.00551	0.24860	8.39958	0.01578	0.00396	0.00180	6.04053	7.88226	15.33588	11.73588	1.09470	0.87464	11.44466	15.83707	0.00982
**Bfar**	2.57743	4.30771	6.08747	8.28568	1.06914	0.28945	4.65472	4.58801	8.27512	5.31387	1.91451	0.56871	0.34690	12.84844	7.33282	2.44209
**Bfsp1**	0.01357	0.00773	0.79358	0.01195	0.00778	0.00668	0.05829	0.01066	3.29284	0.00666	0.00000	0.00000	0.00896	0.01625	0.00000	0.00682
**Bfsp2**	2.39249	0.00597	0.05778	0.23386	0.00958	0.00325	0.00575	0.00678	0.00811	0.05127	0.01287	0.02776	0.02382	0.01357	0.01039	0.00499
**Bglap**	0.04911	0.00824	0.11246	0.00645	0.01292	0.00644	0.00737	0.01261	0.10086	0.01474	0.01187	0.00773	0.01112	0.01293	0.00807	0.00742
**Bglap2**	0.08315	0.00812	0.15035	0.00540	0.01195	0.00597	0.00733	0.01243	0.21040	0.01411	0.01091	0.00752	0.01080	0.01340	0.00668	0.00733
**Bglap3**	0.53521	0.00786	0.23239	0.05323	0.04735	0.22377	0.00695	0.00934	0.23063	0.01861	0.04045	0.00690	0.02268	0.01264	0.00335	0.00672
**Bgn**	0.01222	0.00743	0.01398	0.00593	0.00872	0.00754	0.00713	0.00982	0.01090	1.89412	0.00545	0.57669	0.01120	0.01212	0.00000	0.00752
**Bhlha15**	0.01922	0.00761	0.02051	0.00635	0.00850	0.02200	0.00723	0.01437	0.02499	1.26694	0.01216	0.00519	0.01690	0.02045	0.01592	0.00713
**Bhlhb9**	0.32368	0.00398	1.09915	1.49414	6.13303	0.00507	0.00650	0.85868	0.00000	0.83129	0.99300	0.06680	0.00000	1.03156	8.33767	0.00603
**Bhlhe40**	0.01729	2.56593	0.23478	0.80518	0.00714	0.04402	0.03722	0.00000	0.83413	0.00000	4.52578	0.01599	0.00000	0.00000	0.20287	0.00504
**Bhlhe41**	0.01323	0.00764	0.01095	0.68331	0.00914	0.00626	0.22737	0.00024	1.17358	0.00000	0.34007	0.01237	0.00847	0.00303	0.43950	0.00691
**Bhmt**	54.48173	33.08203	50.26210	0.58862	34.20086	23.63597	33.85645	55.41694	6.99901	55.29181	52.29159	22.13828	51.58990	24.95384	22.64116	10.89185
**Bhmt2**	4.67360	1.90388	4.28007	0.60438	0.83844	4.99996	1.74552	19.35663	0.30658	9.83826	8.34052	5.67548	8.18777	3.51396	2.60692	0.02944
**Bicc1**	0.00914	0.29227	0.01628	0.29262	0.00841	0.00728	0.00725	0.01064	0.52809	0.01014	0.02079	0.01681	0.01193	0.00866	0.00000	0.00718
**Bicd2**	1.67196	0.00508	0.00000	6.06614	0.00736	0.94583	1.35639	0.71610	3.51825	0.40847	0.00000	1.47203	0.41878	0.00000	4.60359	0.00410
**Bid**	2.56463	0.79804	3.82749	4.04647	0.00411	3.12386	4.08872	13.30588	1.71219	7.94650	6.14746	0.71463	5.22345	2.50347	7.07980	0.00000
**Bik**	0.02524	0.00798	0.22503	1.50722	0.00738	0.00891	0.00719	0.01215	1.70830	0.16240	0.00000	0.00379	0.01115	0.00836	0.00847	0.77801
**Bin1**	1.61553	0.00585	0.00000	0.01037	0.00398	0.00640	0.01580	0.00569	0.01243	0.05130	0.01252	0.03460	1.08728	0.01171	0.00949	3.02104
**Bin2**	6.87165	0.03624	0.23501	2.12805	7.30789	9.92323	2.34394	7.14980	0.02357	7.87021	2.63146	0.65699	1.39008	0.03139	0.22503	4.45041
**Bin3**	3.29638	2.24406	4.17870	2.50866	0.08645	0.00249	0.00219	0.94575	2.13986	2.50612	0.00000	0.01454	1.95577	0.00000	0.68744	0.00000
**Birc2**	2.80874	3.03127	3.20982	1.52559	4.18056	0.02184	1.09823	1.42428	5.01931	0.47943	0.61630	0.21460	0.00000	0.26891	4.23780	1.07551
**Birc3**	0.00000	0.02589	1.93300	12.09147	3.21354	0.00578	0.00533	1.77249	8.26714	4.07365	2.37613	1.92432	0.00000	10.34604	7.85499	0.45585
**Birc5**	25.97633	13.85529	22.86673	15.24819	15.77858	14.58937	12.58517	22.47294	19.14921	22.59412	13.43334	6.92227	20.36219	4.48041	11.00891	10.63143
**Birc6**	0.84358	0.67459	1.25712	0.76196	0.01255	0.00592	0.60258	1.43213	3.17383	1.54205	5.40072	0.18374	1.53814	5.27145	5.18316	0.82130
**Bivm**	0.01636	0.07186	0.01125	0.29619	0.00876	0.00664	0.00743	0.01284	0.08257	0.01356	0.03072	0.00782	0.01219	0.00988	0.00839	0.00729
**Blcap**	2.01769	1.53690	1.79422	5.58922	2.20016	3.56442	2.30851	2.12550	1.25784	2.60902	0.71310	0.15615	0.52672	5.06221	3.66020	3.34375
**Blm**	2.59459	1.43321	4.36005	6.46406	3.94032	1.63397	4.64091	6.62085	5.93061	4.13319	5.82685	10.85846	2.89526	11.17421	8.65619	0.01134
**Blmh**	12.96800	9.09875	4.64207	7.89829	10.13963	4.43121	5.19688	3.14943	2.53944	8.25397	3.15848	0.22681	3.51638	0.26088	4.62590	5.31097
**Blnk**	0.07146	0.00713	0.29535	0.03548	0.03009	0.31823	2.33316	0.00901	0.62175	0.00452	0.00755	0.00567	0.03285	0.01577	0.00105	0.01441
**Bloc1s1**	4.02194	0.46462	3.88978	0.00000	2.94941	0.86230	1.05417	0.65134	0.46272	3.02605	9.18836	0.31797	2.34857	0.00000	0.69757	1.20909

**Bloc1s2**	10.39701	3.64818	4.83507	4.52443	7.88106	5.26239	5.11277	5.42965	6.82740	7.66222	5.80831	3.04498	7.31340	2.25206	0.24048	7.10269
**Bloc1s3**	0.27612	0.01804	0.27285	1.23137	0.26683	0.08498	0.04855	0.07578	1.80885	0.29989	1.64995	0.05989	0.45009	0.91479	0.73626	0.07245
**Bloc1s4**	0.17130	0.00766	0.33667	2.90093	0.01290	0.00521	0.01537	0.09416	6.81345	2.66210	0.92789	0.09983	2.21200	0.00000	0.68726	1.41166
**Bloc1s5**	3.15896	8.50839	3.11100	0.00000	7.98129	2.48131	4.49860	2.75538	0.07562	3.95377	2.54204	0.71765	2.78915	0.05017	0.00000	2.24778
**Bloc1s6**	5.52520	5.34320	5.45022	0.67512	2.60306	4.62606	6.85175	2.45366	1.11271	2.35736	4.52815	7.45686	3.45398	0.34441	2.46162	0.05213
**Blvra**	0.64540	0.03629	1.81157	0.21482	0.01073	0.43956	0.00546	1.67170	1.52102	0.69761	1.33879	0.10202	0.96909	0.00273	0.45582	2.64361
**Blvrb**	3.15669	0.61088	1.83863	0.09835	2.88238	2.48701	0.31105	1.25735	1.27331	1.86746	1.33340	0.02637	0.18846	0.00374	0.00000	1.24660
**Blzf1**	3.69114	2.12290	4.95013	0.92931	0.00000	2.63435	2.10529	3.55933	2.31492	0.61405	2.42516	4.84306	0.70646	8.47019	4.78703	0.22085
**Bmf**	1.96398	0.00563	0.52601	1.45512	0.00402	0.00404	0.00584	0.55953	0.00000	0.00000	0.05883	0.07807	0.14931	0.57378	0.58121	0.00481
**Bmi1**	0.00000	7.05777	2.32994	31.35280	6.32212	0.02336	2.52730	0.07000	17.80280	4.72525	1.92752	5.97592	0.83640	1.70760	31.99216	0.58975
**Bmp1**	0.62191	0.00779	0.00216	0.07166	0.00649	0.00756	0.01837	0.01199	0.01081	0.00382	0.78326	0.03896	0.01008	0.32159	0.10691	0.00631
**Bmp15**	0.01735	0.00732	0.00146	10.87646	0.00871	0.00512	0.00659	0.00000	6.00159	0.00000	0.25178	0.50199	0.00942	0.00000	1.90046	0.00485
**Bmp2k**	0.01402	0.00749	0.26871	1.48790	0.00853	0.00556	0.00710	0.00939	0.63168	0.01221	0.00001	0.03885	0.00946	0.00374	2.87419	0.14601
**Bmp4**	11.90797	0.00000	0.00475	0.09075	2.31625	0.00000	0.00000	0.00000	0.00840	0.44628	0.01200	0.07769	0.00950	0.01578	0.01333	0.07753
**Bmp5**	0.01214	0.00842	0.00961	8.31586	0.00951	0.00580	0.00766	0.04288	0.66813	0.00696	0.06138	0.00517	0.02625	0.04765	0.82358	0.01331
**Bmp6**	2.74579	0.00442	0.00000	10.14169	0.00818	0.00175	0.00408	0.43092	5.36386	1.12519	0.54232	1.79604	0.00000	0.00000	3.14477	2.49338
**Bmp7**	0.01340	0.00781	0.01749	0.00497	0.00926	0.00729	0.00746	0.01221	0.40153	0.00907	0.03266	0.02716	0.01223	0.01811	0.03733	0.00738
**Bmp8a**	0.01104	0.00791	0.01802	0.00981	0.00868	0.00718	0.00741	0.01844	0.02362	0.01108	0.01268	0.00798	0.01746	0.01259	0.01023	0.26929
**Bmp8b**	0.02550	0.00769	0.01009	0.00990	0.00861	0.00670	0.00738	0.01797	0.02283	0.00980	0.01120	0.00712	0.01226	0.11158	0.12324	0.02902
**Bmper**	0.01327	0.00806	0.01039	0.00665	0.00903	0.00673	0.00752	0.00933	0.01173	0.00805	0.19921	0.01780	0.00967	0.01121	0.00686	0.07187
**Bmpr1a**	4.03517	0.18030	1.41346	4.31595	0.31539	6.27619	3.06446	1.97437	5.25276	2.50792	3.15509	10.84069	1.78842	10.64033	3.24933	0.20881
**Bmpr1b**	0.01326	0.00796	0.00984	5.16683	0.00932	0.00595	0.00726	0.01058	1.20500	0.01917	0.01636	0.00198	0.01338	0.00000	2.19243	0.00644
**Bmpr2**	0.03757	0.00609	0.83730	6.05419	0.12413	0.92685	0.00567	0.13175	3.05638	0.98727	1.48293	6.21366	0.19272	4.79105	2.60724	1.53165
**Bms1**	2.82721	6.12815	5.36159	5.88890	11.11312	4.99691	6.45519	11.32270	12.62619	8.79527	7.96569	9.37631	6.13253	13.84206	12.28520	3.24152
**Bmt2**	0.00000	2.19159	2.99499	2.11079	1.86365	0.00779	0.00605	0.00205	0.83958	0.70549	0.12017	0.14120	0.13918	0.01043	0.56195	1.11856
**Bmyc**	0.00000	0.01726	0.70396	0.00342	0.03530	0.70621	0.13871	0.38280	0.01040	1.74485	1.24356	0.00000	16.59585	0.01008	0.00000	4.13825
**Bnc1**	0.01282	0.00763	0.00673	3.88300	0.00911	0.00587	0.00723	0.00869	3.09439	0.39782	0.02744	0.08196	0.01249	0.00000	2.85415	0.00660
**Bnc2**	0.07890	0.00867	0.00721	2.53557	0.01469	0.00672	0.00747	0.01308	0.50601	0.13729	0.02175	0.01925	0.01281	0.00167	0.76605	0.00706
**Bnip1**	5.18267	6.99249	4.89196	0.62633	3.00662	0.60669	2.01268	2.02402	0.10655	3.39525	0.04475	0.06364	3.07224	0.02180	0.77875	0.57573
**Bnip2**	0.12449	0.02589	0.23690	1.39002	0.00752	1.74887	0.21386	0.94830	3.64798	3.25806	1.16739	3.97309	0.18838	2.01348	3.06572	0.00548
**Bnip3**	1.53753	1.41151	1.38458	13.50469	2.93075	1.83176	2.08875	8.74120	12.57412	2.84301	23.19908	18.24889	2.18663	19.50365	14.17837	0.04534
**Bnip3l**	11.60232	12.60018	18.82767	16.30105	15.18248	7.09971	13.64970	30.02320	13.04792	32.92982	22.78898	22.65281	21.91633	26.89250	15.20699	6.05649
**Bnipl**	0.62802	0.00700	0.35243	0.02474	0.00594	0.00645	0.00673	0.00651	0.66168	0.57721	0.03240	0.00252	0.01005	0.28951	0.21420	0.00684
**Boc**	1.20434	0.00813	0.03901	0.00906	0.01601	0.00788	0.05156	0.38782	0.02662	0.06545	0.00853	0.07362	1.41607	0.01422	0.00433	0.00587
**Bod1**	8.19692	3.04142	5.04729	21.55492	1.16905	3.48052	5.28186	7.73020	9.62582	15.75980	8.30188	1.38225	3.88181	11.40048	13.69122	4.05080
**Bod1l**	3.44204	0.01336	3.88526	5.89190	0.00000	0.00000	0.69257	1.63023	9.58113	4.56183	2.79257	2.58551	0.38253	27.05918	19.32144	0.32670
**Bok**	9.33958	0.00000	2.62281	0.95809	0.05726	4.42756	1.76260	7.87220	0.00000	9.07970	11.04811	0.11509	6.00682	0.29416	0.00000	4.62979
**Bola1**	1.79765	0.01322	0.04336	3.51179	4.37609	0.90008	0.38362	2.88245	7.33163	1.72343	1.30767	0.04094	6.34468	0.00610	0.00000	0.74179
**Bola2**	4.19342	0.32436	2.52586	7.48497	3.29952	1.11382	0.38625	0.53517	13.92173	4.13901	8.58669	0.70553	2.37555	0.00000	0.00000	4.27814
**Bola3**	1.65457	0.00427	0.08815	0.44608	1.41324	1.17183	0.80213	0.02811	0.65323	0.32498	2.20573	0.02999	0.18904	0.00144	0.00000	0.01231
**Bop1**	6.97988	0.09039	0.89714	7.67314	6.00390	4.72947	1.17525	3.13021	10.49804	13.96513	10.03647	0.72223	7.58488	0.25455	2.49296	3.01519
**Bora**	3.08113	4.40502	4.89551	3.06450	0.04189	0.96786	5.18607	6.64202	1.82773	5.85779	4.44828	0.58105	4.03575	3.24989	8.43640	1.82852
**Borcs5**	1.08362	0.00743	0.76226	3.01884	0.01154	0.03928	0.36637	1.01140	0.78559	2.75300	0.01478	0.49818	1.51397	0.00008	0.33620	2.67839
**Borcs6**	1.36250	0.00612	0.00000	4.12619	0.00835	0.68201	0.00525	0.69227	5.18537	5.00466	1.46220	0.17546	0.00000	0.45175	0.56527	0.00452
**Borcs7**	2.86006	0.01447	1.13052	0.28669	0.00000	1.44280	0.12862	1.32399	1.06205	1.99229	4.20736	0.07800	0.00000	0.17566	0.05886	1.30812
**Borcs8**	0.00000	2.39315	2.70591	1.15565	4.23468	0.01717	0.35683	2.27330	1.55667	3.46249	1.23474	0.22303	2.51033	0.00506	1.78991	3.50150
**Bpgm**	0.00000	0.00768	0.86652	18.35104	0.00588	0.00530	0.00601	0.00000	1.43137	7.15115	1.07570	0.10398	0.00000	0.61865	4.07650	1.66764
**Bphl**	0.00690	0.00669	1.93347	0.74278	0.88892	0.00884	0.01670	0.00673	0.50278	1.15681	0.00628	0.00000	0.00691	0.00896	0.00000	0.00648
**Bpifb9a**	0.01345	0.00774	0.01014	0.01050	0.00940	0.00673	0.00734	0.01099	0.01265	0.01041	0.01116	0.00637	0.01310	0.36251	0.00360	0.00746
**Bpifb9b**	0.01355	0.00781	0.01069	0.01050	0.00938	0.00672	0.00738	0.01140	0.01263	0.01118	0.01168	0.00678	0.01305	0.25821	0.00397	0.00746
**Bpifc**	0.01362	0.00760	0.01119	0.02055	0.00934	0.00646	0.00742	0.01167	1.28380	0.00000	0.00000	0.00484	0.01306	0.02706	0.34707	0.00725
**Bpnt1**	4.24046	3.10181	3.02228	0.74713	0.29115	3.92005	0.08561	2.51824	1.69824	2.12034	3.06548	0.31923	3.86710	1.18200	3.78918	2.30614
**Bptf**	3.44806	4.64741	4.61832	15.06092	4.41049	1.55328	4.95661	3.61274	7.50510	19.77730	6.48727	8.41969	4.00348	16.84570	14.96029	0.54513
**Braf**	2.12733	2.84787	2.80596	1.00042	3.37428	0.43115	3.61799	0.46237	1.92974	3.66571	2.75570	9.31866	1.18212	10.84276	2.03476	0.00000
**Brap**	1.53006	0.10340	0.41720	11.10597	3.33662	1.99694	0.34179	4.13222	8.18589	11.75962	2.72431	0.76599	1.91050	0.00000	0.47774	2.53796
**Brat1**	0.97140	0.04330	1.15570	4.21671	0.00732	0.00421	0.54175	0.07886	1.79076	1.74644	0.37824	0.00000	4.23338	0.01996	0.90650	2.57008
**Brca1**	3.47084	7.77915	9.86316	3.93742	0.01313	6.32979	6.52096	2.20838	2.09770	7.93867	1.40606	6.81294	2.67374	1.64681	3.39573	4.39406
**Brca2**	2.81639	0.61137	2.33209	22.12604	6.45722	4.13889	2.67565	2.37530	15.20894	19.72616	7.30919	1.31265	3.55792	25.23167	17.99595	0.98534
**Brcc3**	1.43459	2.95331	4.59442	0.04593	2.59674	0.00392	3.31738	0.68042	0.28182	1.74287	3.26985	0.02410	2.96924	1.86127	0.21939	0.07821
**Brd1**	4.78407	3.11657	6.32340	19.26375	3.49956	0.57323	3.30718	12.78446	19.08558	8.80959	10.10688	10.04194	8.11191	0.62487	10.88762	5.72693
**Brd2**	8.30026	7.31967	9.62329	7.01755	0.86938	6.06512	5.08479	19.39575	10.53486	17.95072	9.76944	7.73865	12.07349	9.74521	16.39506	7.47568
**Brd3**	9.56160	0.33008	6.34933	2.55414	5.64212	3.58025	4.03278	17.32034	2.41867	18.54871	9.64150	1.22843	4.85510	1.28944	10.50179	1.29867

**Brd4**	11.04092	1.08789	3.77536	4.52654	0.24289	2.10486	4.18980	12.54327	6.64303	16.94447	5.38471	0.85017	6.60330	0.48993	8.89147	4.73048
**Brd7**	12.74599	7.09599	6.56236	12.56677	6.72314	5.26141	4.85794	6.10816	9.44914	13.59332	8.25189	3.48111	7.59855	4.26504	13.51985	4.47358
**Brd8**	6.59122	0.11106	4.87439	13.10642	5.41952	3.29064	7.92689	9.24985	20.25100	8.45430	13.21219	4.56346	4.74354	9.27665	14.89591	3.02563
**Brd9**	6.66523	0.65036	8.56667	5.11122	0.02976	10.69707	0.01866	16.19938	14.74496	19.41172	15.32708	17.22431	8.40625	15.20310	16.81098	4.23229
**Brdt**	0.09846	0.00625	0.00000	16.15831	0.01334	0.00205	0.00544	0.00000	14.15744	1.31069	1.02444	5.64896	0.00875	0.11827	7.12813	0.00296
**Bre**	8.36557	0.00000	5.63671	0.00000	6.70093	5.28024	4.01330	4.93559	0.00097	2.64601	1.24468	0.29851	2.96860	0.02252	0.03681	3.64540
**Brf1**	2.32617	0.00306	3.10767	7.80113	3.27653	0.00514	0.00405	2.14330	6.18883	3.45328	1.96849	0.09833	1.25925	0.00000	0.14046	3.70587
**Brf2**	1.28287	0.20904	0.52458	1.68916	0.46873	0.01699	1.59910	1.49619	9.74278	4.37509	0.56355	0.13370	1.62785	0.00000	1.02475	0.03323
**Bri3**	1.94517	0.15229	0.47733	33.19449	2.30284	1.05589	0.43212	0.12641	21.16356	2.25490	9.88907	0.16212	0.76151	0.04505	0.07197	1.06264
**Bri3bp**	8.04175	0.02001	2.39377	7.12393	4.08526	4.62633	4.68325	15.97681	13.55550	24.45548	15.86825	7.70683	2.88856	3.43947	5.46479	4.42274
**Brip1**	1.43502	4.19376	4.54018	6.73437	0.18730	0.40888	4.55232	0.07685	7.37514	1.96792	3.73492	0.42898	0.10007	6.68758	3.06114	0.46174
**Brip1os**	3.55390	1.82717	4.62431	6.79599	1.17183	3.81674	2.22960	0.81330	10.06745	1.07328	7.33974	0.88765	2.08778	5.94882	2.00666	0.41659
**Brix1**	13.87780	13.21137	17.96657	18.91112	12.47811	10.79384	10.18766	20.99542	29.38979	29.91285	23.98736	15.07200	14.13648	19.28464	14.92067	10.11241
**Brk1**	17.25352	9.29254	19.67874	11.76962	13.27225	12.44270	11.92563	16.93823	5.24798	16.62137	8.75291	10.85035	17.49827	10.82409	10.44195	12.37569
**Brms1**	10.06998	3.78003	9.72230	10.22915	3.85908	1.64584	3.15477	12.45662	8.04007	8.89614	5.63253	1.44219	9.88345	0.08168	3.79299	5.73288
**Brms1l**	0.18293	1.67303	0.27849	6.13294	0.02176	2.34349	0.84954	1.57896	12.22430	1.18122	3.43053	6.38718	0.34402	3.32690	8.00340	0.10328
**Brox**	1.33724	4.64126	1.23880	5.46188	0.00208	3.09225	3.26450	2.47482	2.32328	3.51494	2.01720	3.55085	1.71011	1.37803	4.46257	4.33218
**Brpf1**	3.78962	0.09702	1.45338	2.72437	0.72892	3.84751	1.57716	4.85584	11.34110	6.47722	3.96608	0.28981	3.45093	1.35033	3.89731	0.52114
**Brpf3**	5.58704	1.08908	0.55907	9.96111	0.02479	0.07293	0.00000	1.49705	12.92318	2.16651	5.82640	2.78124	1.29864	0.01213	2.45338	3.58583
**Brsk1**	0.01181	0.00797	0.00963	0.09490	0.00943	0.00658	0.00721	0.06681	0.68254	0.00000	0.09083	0.01612	0.00878	0.01243	0.22031	0.00724
**Brsk2**	0.00777	0.01806	0.41750	3.83673	0.00931	0.00546	1.09349	0.11192	0.70357	0.00000	0.55832	0.00203	0.57177	0.00000	0.19279	0.00613
**Brwd1**	2.11847	7.02621	7.82492	5.88378	3.09560	3.25402	6.04181	9.30794	4.86179	9.87077	6.74450	6.69789	0.68089	9.21806	13.30950	1.14202
**Brwd3**	2.70617	0.01281	0.29144	7.54095	0.00584	1.81593	0.65563	0.32200	13.77016	5.71804	0.31766	8.88977	0.02116	14.73432	8.50907	0.74527
**Bscl2**	3.04822	4.41909	0.00000	9.41370	0.01057	0.00428	1.97542	0.00000	2.14306	1.26016	0.97974	0.03932	0.74641	0.00000	1.12945	0.00703
**Bsdc1**	4.21113	0.53996	1.49532	0.23378	1.36934	2.74204	2.51220	7.53109	3.61507	7.87830	2.41480	0.73469	2.27449	1.78218	6.64477	0.39732
**Bsg**	11.91202	5.48971	14.35138	4.73047	3.29334	6.35482	6.35127	11.68563	0.26548	9.81025	10.73399	1.15145	21.96559	0.19963	0.00000	15.16916
**Bsn**	0.01359	0.00824	0.01231	0.00580	0.00938	0.00695	0.00765	0.01426	0.07907	0.12235	0.01130	0.00879	0.01609	0.00690	0.14103	0.00748
**Bspry**	0.07218	0.00611	0.84369	16.33726	0.00598	3.56092	0.90274	6.18585	22.68398	8.65708	4.97960	0.39947	2.42238	0.08397	5.49919	0.00000
**Bst2**	0.87212	0.00608	0.60937	0.86327	0.00526	0.00824	0.01605	0.90368	0.00002	1.02731	1.87424	0.00000	0.00968	0.01882	0.00225	0.00595
**Btaf1**	3.79341	7.36569	1.98372	12.81973	0.09991	2.24417	3.55061	4.54072	13.32127	6.92408	4.03897	7.87145	1.79078	14.84724	11.92020	0.09718
**Btbd1**	2.20282	6.00439	7.01778	0.96803	5.32761	1.00428	4.99207	1.93908	7.10127	4.69038	7.11270	6.89653	1.82911	3.35640	3.24644	3.37198
**Btbd10**	8.01377	3.75832	2.71457	9.80502	4.31402	0.07958	3.66880	4.14151	4.61873	5.60226	4.19303	6.52795	2.45195	7.56703	10.53592	2.06977
**Btbd16**	0.06869	0.00778	0.11714	1.52226	0.00838	0.05150	0.03807	0.02935	2.42930	0.20798	0.06006	0.02008	0.01933	0.35874	0.45899	0.01429
**Btbd18**	0.00000	0.00531	0.51450	22.09754	0.00754	4.21295	0.00879	5.63391	22.57879	19.36291	10.11797	2.44581	6.27121	22.75683	26.43760	0.00254
**Btbd19**	0.97253	0.08537	1.98014	0.93420	0.02731	0.03532	0.09031	0.00000	1.43505	2.86140	3.33167	0.05402	0.15117	0.44184	0.87871	0.03384
**Btbd2**	1.01608	0.00678	0.00000	7.20015	0.00471	0.01223	0.00488	0.00213	1.58677	1.44642	0.13298	0.04056	2.25068	0.00000	0.19317	3.18730
**Btbd3**	0.01120	0.00507	0.00000	0.00000	4.94811	0.01414	0.00724	0.00437	0.49185	0.03722	0.00471	0.00000	0.02025	0.04819	1.20956	0.00738
**Btbd6**	0.36298	0.00459	0.05093	0.11083	3.27826	0.02772	2.56647	1.57857	0.53947	1.46625	0.28869	0.01971	0.14068	0.02402	1.18939	0.03115
**Btbd7**	0.16505	0.00648	0.05677	3.17362	0.00773	0.00883	1.96032	0.61159	3.00205	0.61126	0.40125	0.14017	0.44250	0.05584	2.11577	0.06800
**Btbd8**	0.01254	0.00781	0.01201	0.01000	0.00896	0.00643	0.00740	0.01342	0.01246	0.00873	0.01261	0.00761	0.08474	0.00752	0.08988	0.09963
**Btbd9**	0.92092	0.01745	1.04611	0.66472	0.01259	0.00618	0.00652	0.56197	1.32968	3.27870	0.42644	0.00000	0.00588	0.68624	0.49905	0.01254
**Btd**	0.00323	0.00667	2.22061	0.00265	0.00569	0.00520	0.00637	0.95889	0.02283	0.88631	0.17858	3.12391	0.47537	0.00518	0.44601	0.00609
**Btf3**	42.97931	23.39455	33.66318	15.20402	25.36398	18.04243	21.16883	35.35632	16.77413	37.08790	30.86620	23.99731	37.46710	17.71876	19.17507	22.09827
**Btf3l4**	15.49440	7.87385	10.32957	7.27243	8.52470	8.88988	5.26030	9.69414	8.11473	13.20814	13.29077	12.95869	11.87723	17.77704	9.60610	4.34204
**Btg1**	8.23177	7.48954	10.75209	5.36504	6.75409	2.68003	9.41641	14.38586	17.12751	21.09363	14.74302	14.74474	14.65871	25.41034	6.40831	1.56671
**Btg2**	6.31017	3.13146	1.24162	1.67739	0.01788	3.74113	0.04186	0.00000	1.27221	1.42851	0.41097	0.13416	0.02172	0.00000	0.32293	0.00000
**Btg3**	2.09509	0.00164	0.08535	4.74371	2.58047	0.00166	0.07414	0.32707	3.94451	0.43291	0.42356	6.87731	0.31211	0.02762	0.10273	0.04849
**Btg4**	0.00424	0.00849	0.00000	16.20489	0.00887	0.00446	0.00735	0.35892	4.54495	2.59952	0.74489	0.22824	0.00299	0.55909	4.79592	0.00421
**Btk**	0.00577	1.28884	0.00841	2.09513	0.00873	0.00673	0.00699	0.01109	0.78135	0.07910	0.00693	0.00399	0.10887	0.17052	0.62700	0.00683
**Btn2a2**	0.01660	0.00775	0.66487	0.42533	0.00805	0.00558	0.00729	0.01222	0.31469	0.01286	0.01031	0.00696	0.00874	0.01240	0.00000	0.00699
**Btnl1**	0.01656	0.00790	0.23424	0.01033	0.00875	0.00567	0.00737	0.01204	0.01239	0.09228	0.01175	0.00746	0.00974	0.01211	0.00683	0.00704
**Btrc**	16.49387	14.57751	16.39989	1.84493	14.57984	5.83036	15.13602	25.92243	6.32967	19.94304	14.20116	7.47132	14.98851	2.17960	5.51869	7.58866
**Bub1**	4.19371	9.47444	4.20832	9.19331	6.51803	4.21086	4.10099	2.93074	15.80371	13.61649	7.29821	11.06577	3.17811	12.09147	12.32335	0.84304
**Bub1b**	10.06179	4.35761	6.55608	0.66914	5.75418	2.22321	2.81116	7.19085	2.42459	4.65414	3.51522	7.83029	3.55006	7.05343	7.04274	1.19472
**Bub3**	18.18566	15.08087	17.27397	2.03322	9.96404	8.51760	12.05188	17.67320	11.46053	20.13060	11.53859	8.09396	13.64488	0.51060	9.43941	13.04350
**Bud13**	2.28908	0.44588	2.60202	0.04703	0.02314	0.04325	2.58677	5.21435	0.04416	1.50322	0.30583	0.20809	1.04524	2.10896	9.69832	0.73092
**Bud31**	17.82020	7.21916	10.74397	4.63633	3.56504	6.60614	6.08175	10.62317	10.07482	14.49093	5.85193	7.45893	12.01050	9.94654	7.83990	3.94824
**Bves**	1.34086	0.00637	0.00362	0.00927	0.00537	0.00657	0.16373	0.01179	0.01184	0.00920	0.01236	0.00745	0.01101	0.00792	0.18096	0.00589
**Bvht**	0.05083	0.04113	0.33808	0.07729	0.00744	0.00564	0.00754	0.01461	0.03541	0.01409	0.01144	0.00847	0.02282	0.01314	0.00203	0.00707
**Bysl**	10.99302	6.98380	11.14864	9.63459	8.61413	5.87885	8.23531	24.28898	10.13177	22.09954	7.61854	5.78271	15.54920	2.87142	4.58371	8.81958
**Bzw1**	17.14466	23.04586	20.75548	21.69991	17.91339	12.92034	19.83424	26.16985	27.27718	26.01238	13.53031	14.24540	20.04943	39.58483	32.26844	6.85732
**Bzw2**	12.16814	9.46840	12.55789	2.75547	13.45170	7.22385	12.11987	11.91565	5.65476	18.78831	6.37906	7.12486	9.64210	8.94176	8.72179	8.32967

**C1d**	8.72647	12.17262	12.57959	6.18570	9.75364	9.94860	10.36676	8.43265	6.72247	18.32403	7.53628	11.06266	16.33139	16.63159	17.10928	3.32833
**C1galt1**	0.02092	0.00796	0.01974	0.56218	0.00864	0.00612	0.00748	0.01394	0.90131	0.00000	0.23463	0.00529	0.01775	0.00662	0.04985	0.03579
**C1galt1c1**	0.41242	1.88377	1.78692	0.00000	0.00384	6.14411	0.00462	0.85984	0.20163	1.48624	1.38110	0.00000	1.60382	4.96943	1.48342	0.00217
**C1qa**	0.16294	0.00782	0.00693	0.00815	0.00809	0.00781	0.00742	0.09464	0.01192	0.01990	0.09701	0.00706	0.10733	0.01129	0.00804	0.01498
**C1qb**	0.01104	0.00786	0.01199	0.48233	0.00904	0.00640	0.00695	0.00939	0.00387	0.35840	0.01415	0.00740	0.54881	0.01641	0.00332	0.00680
**C1qbp**	23.63984	12.17105	21.85277	19.73873	13.98744	3.63880	10.10024	19.47592	27.22680	30.39267	36.51250	14.97663	18.82659	14.44258	13.09281	4.45830
**C1ql1**	0.00777	0.00744	0.00433	6.88477	0.00916	0.00688	0.00679	0.80994	0.41641	1.51005	0.41042	0.12244	0.00392	0.00000	0.25985	0.00612
**C1ql4**	1.74643	0.44524	0.00000	0.01028	0.03031	0.05411	0.00634	0.05956	0.01241	0.00145	0.01213	0.00791	0.78859	0.01465	0.00417	0.96251
**C1qtnf2**	0.11721	0.00768	0.44990	0.00803	0.01662	0.01100	1.11125	0.00887	0.01237	0.43621	0.01191	0.00541	0.00921	0.00879	0.02634	0.00730
**C1qtnf4**	0.01000	0.00792	0.01385	0.01006	0.00887	0.00692	0.00752	0.01211	0.02678	2.31791	0.01250	0.00000	0.03765	0.01247	0.00640	0.43301
**C1ra**	0.01424	0.00780	0.01961	0.02378	0.00912	0.00613	0.00735	0.01231	0.01301	0.00801	0.05195	0.00734	0.10834	0.00767	0.06403	0.00720
**C1rb**	0.01375	0.00782	0.01236	0.00952	0.00935	0.00657	0.00748	0.01317	0.01231	0.00971	0.01276	0.00737	0.01266	0.00658	0.16724	0.00738
**C1rl**	0.01765	0.00760	0.02195	0.05562	0.01724	0.00688	0.00726	0.01788	0.09438	0.52056	0.06021	0.00570	0.01035	0.03136	0.01189	0.18897
**C1s1**	0.01200	0.00765	0.09123	0.28385	0.00892	0.00567	0.00736	0.01059	0.56400	0.92690	0.00011	0.30777	0.01113	0.00455	0.29852	0.00747
**C1s2**	0.01202	0.00798	0.53929	0.81737	0.00828	0.00548	0.00752	0.01141	1.75158	0.17993	0.00000	0.11949	0.01649	0.04826	0.00000	0.00697
**C2**	1.03920	0.00390	4.75300	0.02729	0.00000	0.01440	1.90339	6.80137	1.84686	7.40779	4.60347	0.33259	6.51869	9.43448	2.48863	0.00000
**C2cd2**	1.26308	0.00561	0.30952	1.68468	0.02142	3.23954	0.74763	0.00368	1.01088	1.02425	0.03238	0.12029	0.00166	0.55459	0.03845	0.00425
**C2cd2l**	0.00000	0.01585	0.34557	3.71494	0.01471	2.56383	0.00265	11.45370	13.46588	10.80064	9.40748	0.68362	1.10014	4.30418	6.30096	2.28362
**C2cd3**	0.51569	0.15259	1.49085	9.44106	2.44317	3.94806	4.75642	0.00000	7.74217	1.23379	1.72948	4.74896	0.00000	0.00000	4.75653	0.00908
**C2cd4a**	0.01257	0.00810	0.40834	0.00814	0.00855	0.00653	0.00754	0.01344	0.04856	0.01462	0.01169	0.00818	0.00946	0.01441	0.00381	0.00721
**C2cd4b**	0.01358	0.00825	0.01192	0.47071	0.00929	0.00656	0.00753	0.01388	1.08987	0.01019	0.00608	0.00660	0.01295	0.01184	0.00343	0.00727
**C2cd5**	1.24326	0.00533	0.77688	9.83701	1.31099	0.00587	0.01542	3.20945	3.02892	0.33277	0.32198	0.14413	1.34724	0.77866	8.57928	0.01818
**C3**	0.01152	0.00756	0.01183	0.00505	0.00840	0.00651	0.00720	0.00794	0.01069	5.58291	0.04865	0.00000	0.01003	0.01583	0.01628	0.00733
**C4a**	0.01385	0.00759	0.09066	0.24429	0.00911	0.00619	0.00706	0.34325	0.07948	0.01092	0.01047	0.02574	0.05375	0.01173	0.00000	0.02177
**C4b**	0.01593	0.00769	0.03386	0.04945	0.00912	0.00642	0.00717	0.06939	0.00968	0.02113	0.01225	0.02655	0.03117	0.01173	0.00748	0.00735
**C5ar2**	0.02667	0.00794	0.03096	0.12675	0.00941	0.02027	0.01730	0.01282	0.19576	0.04788	0.30846	0.00546	0.03534	0.13920	0.02627	0.02173
**C6**	0.01231	0.03980	0.08894	0.05297	0.00920	0.03593	0.12989	0.01112	0.00936	0.03866	0.17548	0.00592	0.01266	0.20448	0.04820	0.00757
**C7**	0.01375	0.00818	0.01225	0.13911	0.00931	0.00663	0.00752	0.01377	0.00926	0.01424	0.01384	0.00840	0.01266	0.01023	0.02994	0.00738
**C77080**	8.35845	2.71330	5.68377	0.67197	0.16626	8.52274	1.41852	9.24399	5.77674	9.00712	7.05240	0.37025	5.01451	0.75050	6.19648	2.06692
**C77370**	6.06225	0.00717	1.34611	0.30085	9.74171	0.00000	0.94036	0.28404	0.00000	0.78379	0.84627	0.04751	0.10868	1.23412	1.18423	1.89164
**C86187**	0.00843	0.00755	0.00000	20.35356	0.00916	0.00382	0.00586	0.00000	7.56018	0.00000	1.10861	0.03040	0.00001	2.66995	2.01611	0.00303
**C87198**	0.02214	0.00780	0.00694	2.06332	0.00881	0.04284	0.03806	0.01168	1.60056	0.04675	0.01285	0.01371	0.02780	0.03553	0.28491	0.00683
**C87414**	0.05443	0.03917	0.03694	10.28380	0.03845	0.01183	0.06882	0.12121	4.28863	0.42338	0.35566	0.43111	0.11986	0.77167	3.96364	0.00508
**C87436**	0.54060	0.15896	1.71459	8.42478	0.00411	0.00515	0.62175	1.01205	4.96842	2.00075	0.22676	0.49895	0.12653	1.45753	4.28913	1.02310
**C87499**	0.00508	0.00828	0.00000	8.74751	0.00955	0.00469	0.00751	0.09356	7.49227	0.32927	0.51386	0.00000	0.00587	1.44078	5.14497	0.00570
**C87977**	0.00000	0.00731	1.15214	5.78034	0.01061	0.00715	0.00472	0.78618	4.65217	0.00000	4.98380	0.03002	0.00000	9.63041	4.11506	0.00488
**C8b**	0.02233	0.00802	0.47052	0.61749	0.00962	0.00566	0.00738	0.01287	0.74579	0.01110	0.00727	0.00638	0.01201	0.01103	0.00000	0.00713
**Caap1**	0.14435	0.01871	0.06435	3.29246	0.01084	0.04538	0.00608	0.00000	1.57585	0.00000	0.86809	1.17345	0.70846	3.79477	4.45034	0.02017
**Cab39**	8.20821	6.85942	11.57151	15.56590	7.70076	3.07314	6.82038	17.06206	28.03282	21.96163	16.36835	13.87728	12.41010	31.25944	21.98484	3.98274
**Cab39l**	9.66100	10.70059	6.52658	3.91342	4.61474	3.05096	4.26279	3.03357	3.47326	8.42395	4.21303	8.96791	3.22527	12.75118	6.04160	3.32849
**Cabin1**	6.34670	0.06468	3.63430	12.00917	1.64087	0.02500	1.13141	4.93175	7.32496	11.22047	2.57219	0.42783	2.72989	0.24382	3.41724	0.48187
**Cables1**	0.24055	0.00739	0.00327	3.38669	0.00766	0.00627	0.00705	0.01148	0.25288	0.25357	0.00677	0.02234	0.01254	0.00000	1.73793	0.00654
**Cables2**	1.81120	0.06549	5.29620	5.35760	5.47623	0.01104	0.50741	3.97781	1.93017	1.90129	0.37266	4.12229	0.00000	1.03777	3.21688	1.85172
**Cabp4**	0.31316	0.00840	0.00593	0.22981	0.01741	0.00713	0.00731	0.00928	0.00809	0.01677	0.15676	0.00753	0.01052	0.00720	0.04746	0.00721
**Cabp5**	0.01833	0.00818	0.01157	0.01055	0.01038	0.00665	0.00745	0.01266	0.01231	0.02339	0.01277	0.00812	0.05967	0.01188	0.01232	0.00748
**Cabp7**	0.01362	0.00768	0.00912	0.01820	0.00923	0.00678	0.00718	0.20250	0.01234	0.01157	0.01233	0.00724	0.00710	0.01272	0.00713	0.00737
**Cabyr**	0.23031	0.00722	0.00583	0.21146	0.00738	0.00678	0.00725	0.00502	0.02030	0.00000	0.22482	0.00553	0.01556	0.00525	0.39173	0.01398
**Cacfd1**	2.79994	0.92441	0.15949	2.36179	0.00145	0.00409	0.00413	1.24480	1.24369	1.46455	0.97879	0.01046	0.21951	0.00000	1.56304	1.76188
**Cachd1**	1.60421	0.00549	0.19310	0.62590	0.00433	0.00369	1.50859	0.30229	3.15031	0.14807	0.14972	0.06991	0.00000	0.00188	0.55409	3.18965
**Cacna1a**	1.06064	0.00610	0.06560	0.00313	0.78044	0.00767	0.08896	0.00000	0.15415	1.22399	1.25894	0.02847	0.00000	0.00660	0.04152	0.00568
**Cacna1b**	0.01143	0.00787	0.11770	1.96027	0.00852	0.00806	0.00705	0.00781	4.03188	1.10019	0.00000	0.00842	0.02247	0.04641	0.01543	0.00647
**Cacna1d**	0.01144	0.00748	0.00981	0.00865	0.00952	0.00656	0.00721	0.09630	0.01137	0.00547	0.01088	0.01597	0.01638	0.00884	0.42489	0.00733
**Cacna1h**	0.01782	0.00771	0.01139	1.38104	0.00906	0.00639	0.00734	0.01205	0.13241	0.00576	0.01080	0.01636	0.01276	0.00473	0.49819	0.00708
**Cacna2d1**	0.01311	0.00777	0.01152	0.37653	0.00897	0.00631	0.00723	0.00988	0.37820	0.94658	0.00894	0.00407	0.01071	0.00726	0.60582	0.00711
**Cacna2d3**	0.01380	0.00766	0.01173	0.70385	0.00940	0.00651	0.00741	0.01214	0.00607	0.00672	0.01131	0.00716	0.01281	0.01773	0.29750	0.00727
**Cacnb1**	1.46827	0.00425	0.04364	0.05730	3.75810	0.01043	0.01604	1.53007	0.25992	2.59191	1.00224	0.07938	0.00000	0.00672	0.00000	5.04053
**Cacnb2**	0.01369	0.00798	0.01189	0.11563	0.00922	0.00688	0.00760	0.01384	0.00986	0.11732	0.01246	0.00877	0.01307	0.01432	0.03203	0.00740
**Cacnb3**	2.18588	0.00351	2.11495	0.73000	0.04572	8.97449	1.59404	2.90878	0.35999	3.60691	4.74900	0.04478	7.49641	0.00000	0.40733	3.54497
**Cactin**	7.10438	0.11445	3.12787	6.70390	3.14125	0.66333	3.03430	6.98489	12.84162	7.98780	2.33944	0.54444	5.70277	0.14814	0.00000	0.25940
**Cacul1**	9.26316	6.77506	10.53993	3.67666	7.57182	5.27544	11.10853	10.49980	0.17407	4.66110	8.15141	6.57608	7.03436	10.83763	2.20377	0.02753
**Cacybp**	22.50195	8.98698	17.21562	6.67991	19.27834	7.96790	9.35211	6.03255	11.46417	15.90842	7.02983	18.37814	15.36770	7.82564	4.66387	9.23895
**Cad**	13.78664	6.89499	11.08281	11.77610	12.05681	4.62441	11.10283	25.12422	9.91917	22.95142	15.77443	2.44466	16.06044	0.20121	2.40224	8.92579

**Cadm1**	1.14368	0.16975	1.51710	3.06260	0.01096	0.05372	0.13152	3.35868	4.98623	0.00000	3.78625	0.01842	5.31348	1.91220	0.13018	0.03154
**Cadm4**	0.12740	0.00788	0.16288	0.63605	0.01560	0.32852	0.05690	0.19730	2.35724	0.03122	0.37156	0.01981	0.39167	0.22869	0.32093	0.00632
**Cadps2**	0.01360	0.00785	0.01133	0.96990	0.00942	0.00627	0.00730	0.01096	0.38377	0.00761	0.00866	0.00495	0.01294	0.00495	1.09576	0.00718
**Cage1**	0.00686	0.00711	0.20424	0.56025	1.95435	2.46282	0.00736	0.00829	0.01785	0.09407	0.00845	0.00638	0.00980	0.01115	0.00257	0.00596
**Calb1**	0.01130	0.00737	0.01132	0.32868	0.00840	0.00638	0.00719	0.00902	0.74648	5.71315	0.00006	0.00000	0.01068	0.01149	0.43043	0.00747
**Calb2**	0.15423	0.00747	0.00773	0.24055	0.00768	0.00674	0.00720	0.01874	0.00797	0.22232	0.01301	0.00629	0.01193	0.00559	0.12932	0.00719
**Calcoco1**	0.04754	0.00782	1.71259	0.18645	0.06824	0.01222	0.00706	0.24648	0.05677	1.43267	1.61114	0.19841	0.33039	0.00625	0.20182	0.01366
**Calcoco2**	57.07913	39.35981	62.37520	10.68133	43.30833	26.66967	33.36790	60.57298	26.93752	38.73126	48.51803	10.67922	58.85709	45.62771	24.64245	23.84294
**Calcrl**	0.02122	0.00773	0.02557	0.01101	0.00881	0.00678	0.00744	0.01243	0.01208	0.01232	0.01245	0.00760	0.01249	0.04871	0.03510	0.00739
**Cald1**	0.01145	0.00744	1.86446	0.00858	0.00586	0.00912	0.00700	0.00886	0.01175	0.54405	0.00658	0.28036	0.00665	0.01225	0.00000	0.00666
**Calm1**	29.35702	10.34392	24.08384	7.87335	25.58543	17.40173	11.27644	22.33201	13.90166	21.23027	13.10025	5.73731	24.37905	5.90002	2.80270	19.24451
**Calm2**	32.00314	17.74302	22.79167	11.75423	21.55219	14.13869	17.81969	20.13795	14.77334	16.73081	9.54707	11.83034	20.15033	20.76052	15.64512	13.97548
**Calm3**	5.78395	1.13279	2.99779	2.68273	9.85086	2.35764	0.34460	1.67867	5.82381	5.95483	6.23295	0.25023	6.36494	0.02225	0.85382	5.54560
**Calm4**	0.01325	0.00822	0.00972	0.00734	0.00884	0.00666	0.01888	0.05335	0.01163	0.46635	0.07830	0.01971	0.35956	0.02428	0.04299	0.00692
**Calm5**	0.00703	0.00819	0.00843	0.00721	0.00901	0.00673	0.04160	0.22252	0.01119	1.00866	0.00959	0.07934	0.92989	0.03465	0.08607	0.00670
**Calml4**	0.01078	0.00745	0.25811	4.07250	0.00856	0.00531	0.00644	0.00000	0.99838	1.67668	0.26726	0.09803	0.92149	0.00000	1.56915	0.00612
**Caln1**	0.01372	0.00792	0.01182	0.25423	0.00935	0.00666	0.00749	0.01304	0.15685	0.01261	0.01125	0.00762	0.01289	0.05667	0.00658	0.00739
**Calr**	13.63763	5.66839	22.87743	11.37691	3.74509	9.74497	6.45267	12.52718	14.50446	9.42497	10.31568	2.24347	24.79909	2.86165	6.38163	10.87955
**Calr3**	0.01108	0.00762	0.00633	0.01534	0.00934	0.00668	0.00745	0.74283	0.02318	0.21582	0.00536	0.02697	0.00532	0.04254	0.75738	0.00710
**Calr4**	1.53745	0.00416	2.10011	1.81762	0.01034	0.00524	2.18894	1.03377	0.12474	0.49437	0.45778	0.13822	0.00000	1.42548	0.83375	0.00461
**Calu**	6.31496	7.59350	8.48898	5.87199	7.25927	9.61943	9.95407	8.54635	4.72264	7.46328	6.82302	9.87179	8.91663	18.42585	14.22939	7.64650
**Camk1**	1.15506	0.05623	0.14983	0.00000	0.00551	5.77756	0.00022	1.00430	0.06878	0.48616	1.97539	0.03187	7.37561	0.00844	0.03973	0.00000
**Camk1d**	0.00941	0.00753	0.00654	1.29177	0.00863	0.00632	0.00706	0.00931	0.00000	0.00000	0.00005	0.00199	0.01042	1.32051	0.71091	0.44247
**Camk2a**	0.01552	0.01840	0.05521	0.03985	0.00911	0.02243	0.00743	0.02342	0.02730	0.03182	0.01207	0.00697	0.03739	0.13700	0.08129	0.00752
**Camk2b**	0.00942	0.00725	0.00335	0.00992	0.00928	0.00826	0.00666	0.50058	0.58875	0.95543	1.49594	0.00000	0.00000	0.00885	0.00000	0.01509
**Camk2d**	1.21996	1.68472	0.49133	0.00000	4.40993	3.04343	2.21562	0.81507	0.00706	0.11313	0.65998	0.03486	1.00974	0.00356	0.21025	2.47821
**Camk2g**	4.58632	0.63679	4.17724	8.33751	0.00405	2.07915	3.61626	3.34107	6.92426	6.33852	7.31253	0.45217	3.63810	1.94093	4.04747	1.56976
**Camk2n2**	0.01289	0.00794	0.00817	0.92708	0.00943	0.00693	0.04824	0.02187	0.40879	0.00000	0.37753	0.06347	0.01128	0.01760	0.05752	0.00715
**Camk4**	0.55393	0.01926	0.15220	0.29660	0.04968	0.02029	0.06906	0.06578	0.41098	0.19389	0.37786	0.11464	0.09074	0.39973	0.24362	0.05850
**Camkk2**	0.01366	0.00795	0.01212	2.13119	0.00922	0.00633	0.00745	0.01258	0.55222	0.11566	0.00812	0.00626	0.01196	0.00398	0.11074	0.00680
**Camkmt**	1.65782	0.00686	0.76404	0.10467	0.00634	0.00441	0.00690	0.40150	0.06691	0.29940	0.44202	0.02543	0.02437	0.00922	0.00000	1.99170
**Caml**	11.10667	0.04366	6.28044	6.91113	0.03410	2.01049	2.51333	5.64886	13.60302	8.98065	5.34481	7.16207	6.56327	0.63884	5.87508	1.34732
**Camp**	0.01364	0.00770	0.01070	0.53259	0.00930	0.00657	0.00734	0.01135	0.00470	0.25334	0.01108	0.06587	0.01255	0.00526	0.44887	0.00736
**Camsap1**	2.87628	3.93452	6.14499	13.97953	3.41720	4.45209	3.79359	7.80048	16.82628	7.76122	7.34214	1.74975	5.86483	4.88451	10.55867	3.71478
**Camsap2**	0.93635	4.98351	1.71826	12.54343	0.30824	0.56403	0.39617	0.23625	4.94318	0.20358	0.40658	12.24954	1.06086	7.22628	11.79609	1.55408
**Camsap3**	1.08081	0.00613	0.87930	0.12930	0.04798	2.16131	1.27691	0.38646	0.00680	0.57603	0.33452	0.03907	2.15068	0.00809	0.00000	0.27275
**Camta1**	0.09730	0.00862	0.75897	0.76964	0.01582	0.00907	0.02806	0.00888	0.26161	0.23875	0.01666	0.00930	0.03866	2.07453	0.26070	0.38092
**Camta2**	0.72853	0.00671	0.00000	0.00214	0.01468	4.61458	0.01715	0.85045	0.01156	1.18366	0.00011	0.02166	0.00000	0.01453	0.00012	2.57351
**Cand1**	23.13256	18.86165	23.01524	21.97063	13.15598	9.76494	13.00009	29.05168	23.81968	36.17407	26.20615	26.45270	16.12621	37.46319	32.94314	1.75266
**Cand2**	2.00336	0.00435	0.04506	0.08294	2.25241	0.02504	0.06558	0.01785	2.16931	0.11603	0.45018	0.03315	0.08314	0.16515	1.17289	0.01185
**Cant1**	0.75255	0.70270	3.45630	3.31519	0.58088	0.24673	0.65208	2.56084	1.64481	2.77959	0.05661	0.06553	1.64061	0.02170	0.60006	3.91614
**Canx**	11.82744	7.51816	24.76500	9.81317	7.95669	9.57581	12.32581	10.34517	8.09819	12.75186	11.69705	17.60188	16.35641	31.45549	15.80637	8.18259
**Cap1**	2.71184	1.94896	0.00000	0.56862	0.00330	0.00992	2.45252	0.52504	0.00073	0.73467	0.01767	0.08747	0.00648	0.32720	1.66491	0.01092
**Capg**	4.85416	2.51211	0.00000	0.00000	2.48909	8.68045	0.02130	0.00000	0.02187	0.40329	0.24867	0.03725	1.26669	0.00747	0.00232	7.37922
**Capn1**	1.88696	0.00533	0.11516	1.95303	0.10570	0.04109	0.02361	0.10585	1.63776	4.13183	3.93312	0.11463	1.36027	0.10357	0.48736	0.73431
**Capn10**	4.77227	0.34442	1.09041	1.68209	0.04944	0.00773	0.85503	3.73615	4.87288	2.51594	1.94174	0.07662	4.13078	0.49984	0.04988	2.95137
**Capn11**	0.32458	0.13648	0.00139	0.40073	0.00725	0.00828	0.00666	0.76156	0.91066	2.12000	1.14321	0.04941	0.05990	0.00465	0.24344	0.00669
**Capn15**	0.00000	0.63734	0.24654	0.00143	0.33452	1.97665	1.12873	0.90374	0.01090	1.47420	0.01936	0.02993	1.10655	0.00882	0.00426	1.52638
**Capn2**	0.01311	0.00787	0.14488	0.00743	0.01095	8.26427	0.00623	0.00742	0.01323	0.00000	0.00809	0.03627	0.99143	0.01344	0.01143	0.29025
**Capn5**	4.27690	0.00309	1.60583	0.23735	0.01732	3.17965	0.00231	1.04372	0.00741	0.33647	0.00000	0.00000	2.20875	0.01132	0.00000	0.00765
**Capn6**	0.00938	0.00767	2.08673	0.00887	0.00656	4.00384	0.00685	0.00873	0.01261	0.00000	0.00962	0.00655	0.47889	0.01777	0.00000	0.01250
**Capn7**	1.20882	6.04909	2.53555	5.98090	0.00470	0.38594	1.31012	1.94996	0.00000	0.00000	0.19128	0.85928	0.46980	1.96253	4.52211	0.04771
**Capns1**	23.59603	7.61958	17.49780	1.80543	15.73635	13.56255	8.98031	5.37546	0.60539	7.36765	1.73940	0.12481	16.01496	0.00000	0.33377	12.62119
**Caprin1**	14.51413	15.66901	16.79183	12.19619	21.23633	9.26703	10.59598	18.15402	12.85488	22.41276	12.41415	23.27942	13.64198	27.22315	19.12004	7.72910
**Caprin2**	7.09916	0.01010	5.14402	10.00082	0.48982	2.41045	0.00979	0.70166	7.32354	3.43684	1.90823	0.39101	0.78650	4.59000	6.39524	0.01708
**Capsl**	0.01235	0.00804	0.03731	0.25187	0.00882	0.00601	0.00739	0.01052	0.00765	1.42966	0.01264	0.00460	0.01122	0.01088	0.00477	0.00761
**Capza1**	9.72461	12.51825	10.51343	4.77952	8.47935	11.81028	6.89758	8.59029	6.90177	9.02460	8.25489	11.24749	8.93215	23.03218	9.33763	4.80604
**Capza2**	10.20133	10.01726	9.67428	4.00585	2.25582	10.15957	11.12281	14.85410	1.61187	16.05820	8.01419	11.95344	14.99958	26.56922	13.07362	7.05775
**Capzb**	19.71400	8.06721	16.07160	1.11870	16.73158	14.96671	9.60806	16.68772	0.00000	9.80740	10.30286	2.63971	17.62654	0.15763	1.01786	11.07505
**Car2**	0.00748	0.00768	0.01208	0.00503	0.00753	0.00648	0.00717	0.00952	0.01079	4.69402	0.00916	0.00000	0.00834	0.01246	0.00641	1.33810
**Car4**	0.00000	0.01914	1.90618	0.00095	0.03544	0.00685	0.00608	0.07957	0.29246	1.75799	1.06339	0.08291	2.07176	0.09604	1.07280	5.91498
**Car5a**	0.00838	0.44576	0.59970	0.09533	0.00725	0.01277	0.01731	0.00988	0.11968	0.08116	0.25024	0.00616	0.01470	0.03312	0.04998	0.02835

**Car5b**	1.93316	0.61939	0.96140	0.51095	0.09916	0.05461	0.79520	0.47676	0.59852	0.43330	1.36575	0.74868	0.81588	1.61629	0.46052	0.05561
**Car6**	0.01266	0.00798	0.07150	0.00437	0.00920	0.00841	0.00726	0.01155	0.17995	0.09730	0.01028	0.00673	0.01832	0.00623	0.28179	0.00742
**Car8**	0.09949	0.00786	0.12106	0.39877	0.03003	0.01236	0.00714	0.00994	0.14874	0.54480	0.35304	0.03161	0.11828	0.15201	0.07416	0.03016
**Car9**	1.68251	0.00616	0.40697	0.60649	0.06143	0.00523	0.00643	0.21524	0.89391	0.75545	0.01618	0.01317	0.54572	0.08814	0.37958	0.00502
**Card10**	3.51393	1.47872	0.15484	0.26572	0.01812	0.16842	0.00352	0.81856	0.40116	2.12352	0.44489	0.00519	0.47733	0.00742	0.00000	0.00241
**Card11**	0.01356	0.00823	0.01943	0.01118	0.00917	0.00622	0.00761	0.04969	0.09787	0.00000	1.19169	0.00209	0.00000	0.00441	0.16998	0.00694
**Card14**	0.00842	0.00786	0.00775	0.00744	0.00932	0.00700	0.00740	1.06149	0.01068	0.95504	0.00454	0.03714	0.00582	0.01254	0.01099	0.00726
**Card19**	0.01176	0.00778	0.00324	0.00932	0.00952	1.94394	0.00718	0.20068	0.01226	0.06950	0.00840	0.01886	0.00000	0.01185	0.00894	0.00671
**Card6**	0.06622	0.00784	0.03090	1.60618	0.01635	0.00495	0.00707	0.15124	6.21733	3.81471	0.00000	0.07680	0.01533	7.26968	4.90824	0.01337
**Card9**	0.01374	0.00788	0.01220	0.05501	0.00920	0.00653	0.00739	0.01195	0.01144	0.16220	0.01523	0.00812	0.01163	0.01798	0.00834	0.00716
**Carf**	0.49466	0.01701	0.17597	1.23994	0.06198	0.01262	0.05718	1.05058	0.40144	0.12608	0.28585	0.05751	0.15159	0.44734	1.58684	0.04213
**Carhsp1**	6.24992	4.61533	5.29818	12.23152	2.20551	6.36464	4.18051	4.37432	10.60357	6.66213	3.53471	0.85034	5.10888	0.40721	3.44450	4.05377
**Carkd**	6.73822	1.48155	7.73347	0.55109	0.04856	4.08831	6.14425	3.08515	0.81594	3.42692	2.30968	2.35439	7.25357	0.02276	0.27850	3.29048
**Carm1**	2.80000	0.16207	0.75459	0.00000	0.89382	2.72749	1.85368	2.67834	0.05806	2.82051	0.00000	0.04925	2.04001	0.01042	0.00463	1.88571
**Carnmt1**	4.55798	4.40908	5.24940	7.39202	2.67828	2.10055	3.26057	4.26838	12.88207	2.83489	6.43908	8.70822	5.20540	1.82621	0.73718	2.87791
**Carns1**	0.06962	0.00745	0.49052	1.35465	0.01530	0.00856	0.00526	0.00000	4.22059	0.00000	2.66986	0.00000	1.48848	0.00000	0.90433	0.00539
**Cars**	8.19142	8.00398	4.43397	1.90281	6.08625	0.00000	4.07622	11.92071	5.46368	5.42066	3.28904	0.44340	7.08840	0.03077	0.59626	5.82822
**Cars2**	7.01268	1.33259	3.03274	2.65806	4.65298	0.00551	0.09163	2.93604	4.37737	8.53516	5.48202	1.30167	1.59556	0.04358	1.72856	0.00145
**Casc1**	0.01636	0.00760	0.13050	0.69717	0.00904	0.00568	0.00730	0.01221	0.00000	0.00933	0.01355	0.00706	0.01086	0.00524	0.17372	0.00715
**Casc3**	4.65710	5.71692	3.20928	7.88362	3.49925	0.63314	4.75123	5.51573	9.07195	13.68746	7.21904	9.63191	4.54128	7.90738	6.37466	1.89661
**Casc4**	3.84740	4.11968	5.74218	16.63204	2.66585	7.68892	4.46160	4.95035	21.70115	0.88279	22.32688	4.88143	4.48090	14.77814	5.97393	6.81053
**Casc5**	0.30464	0.00655	0.10334	2.14439	0.01695	0.00765	0.38414	0.36633	2.39064	0.00000	0.62839	3.84329	0.73309	9.67913	4.69014	0.00556
**Casd1**	1.13154	0.00622	0.00292	0.01095	0.00607	0.00791	0.00655	0.58257	0.01169	0.00320	0.01180	0.00576	0.02555	0.01560	0.00270	0.26309
**Cask**	0.31495	0.00558	0.00000	1.63179	2.37100	0.00560	0.16268	1.03969	2.54142	0.57513	0.32877	0.08031	0.00410	1.85794	2.92952	0.00685
**Caskin1**	3.44351	0.78702	2.19065	3.94233	0.06252	2.31467	0.68365	6.80334	1.85064	7.52440	5.28653	0.63698	3.32463	0.02705	0.18443	0.92059
**Caskin2**	1.47598	0.00591	0.23997	1.00059	0.03637	4.14189	0.49048	0.96493	1.72843	3.50106	0.31630	0.10927	1.95293	0.01782	0.23932	2.60023
**Casp2**	2.39999	0.01481	0.66375	0.37138	0.02288	0.31826	0.00440	0.59526	0.28461	2.04786	0.89148	0.03937	0.82210	0.00647	2.47857	0.53232
**Casp3**	1.81371	5.95164	6.16691	7.05742	5.48197	0.00262	4.58816	6.44573	8.94459	10.11388	5.00838	0.75230	1.71534	12.40362	7.52448	1.75676
**Casp6**	12.49265	13.52787	15.79194	1.52419	17.47981	6.74589	8.98792	12.65498	3.86949	15.25074	15.22877	1.65134	6.53617	1.87985	7.05211	2.39381
**Casp7**	3.35359	0.30406	0.77215	0.00000	3.14017	3.11940	1.38443	2.32843	0.01885	1.29608	1.57632	0.00000	2.54345	0.00205	0.47784	1.28329
**Casp8ap2**	1.86866	3.99511	1.41684	1.83224	1.58849	0.02033	4.53967	5.80629	4.81835	5.62110	1.21677	6.17867	0.94064	15.99186	18.53642	0.46808
**Casp9**	2.73206	0.01187	4.89626	0.88217	4.44263	0.00310	2.68845	2.56143	0.00000	1.87459	0.00000	0.05234	1.61184	0.13514	1.13713	1.30193
**Casq1**	0.01366	0.00800	0.01216	0.00882	0.00915	0.00664	0.00735	0.01137	0.01197	0.29748	0.01317	0.00698	0.01253	0.00832	0.05404	0.00780
**Casq2**	1.48516	0.00602	0.00354	0.10019	0.01332	0.00682	0.00633	0.00768	0.00761	0.01836	0.01156	0.05720	0.01145	0.01167	0.00778	0.02029
**Cass4**	0.01223	0.00802	1.20766	0.45471	0.00725	0.00547	0.00733	0.01015	0.00510	0.08888	0.01215	0.01952	0.00719	0.00838	0.53585	0.00659
**Cast**	0.32441	0.01848	3.48022	0.79140	0.36822	2.04051	0.21009	3.06154	1.03343	1.27168	1.83899	0.11081	3.28143	4.82276	0.59238	0.21899
**Casz1**	0.20091	0.00848	0.21875	0.00844	0.01667	0.04731	0.00690	0.00894	0.02370	0.24232	0.01119	0.02709	0.67583	0.00785	0.05035	0.02169
**Cat**	7.42567	5.94180	2.85455	0.00000	9.66949	7.10760	6.83774	6.07438	0.07020	2.23357	0.84451	0.02829	10.27236	0.00498	0.00194	1.88247
**Catip**	0.23843	0.00748	0.00675	0.00655	0.00732	0.00694	0.00703	0.00851	0.01159	0.99572	0.07571	0.00450	0.01100	0.01154	0.00680	0.00733
**Catsper2**	0.07292	0.35713	0.69719	0.00175	0.00700	3.17102	0.00796	0.15408	0.79815	0.21440	0.00000	0.01848	0.00000	0.07334	0.08275	0.00595
**Catsperd**	0.00824	0.00868	0.00000	2.18647	0.00971	0.00663	0.00801	0.25925	0.50782	3.15579	0.42575	0.09105	0.08303	0.87397	4.24972	0.00726
**Catsperg1**	0.00974	0.00769	0.08874	1.62676	0.00943	0.00529	0.00724	0.57860	2.37349	0.00000	0.67380	0.12081	0.00612	1.10245	0.29526	0.00653
**Catsperg2**	0.01152	0.00771	0.01631	0.45172	0.00947	0.00613	0.00739	0.21676	0.62452	0.00000	0.08207	0.04246	0.00642	0.38820	0.12581	0.00716
**Cav1**	0.00970	0.00873	0.00802	0.00660	0.00938	0.00691	0.00818	0.24708	0.01069	0.43375	0.00798	0.10487	0.00709	0.00663	0.42590	0.00768
**Cav2**	0.01210	0.00780	0.00248	0.00541	0.00949	0.00677	0.00700	0.00232	0.01128	0.00205	0.21664	0.09646	0.01021	2.38749	0.00000	0.00733
**Cbarp**	1.27114	1.13912	0.11220	0.00174	0.00559	0.00784	0.00562	1.81548	0.00940	0.00000	1.21984	0.00639	0.04304	0.00495	0.00578	0.02020
**Cbfa2t2**	6.97785	5.30926	2.96432	8.26398	5.19459	0.30225	1.66030	2.43562	15.31575	5.42317	2.19708	0.47381	1.29537	7.00250	4.47696	3.51441
**Cbfa2t3**	0.01747	0.00789	1.58481	0.47582	0.00937	0.11384	0.00687	0.31258	0.33472	0.17779	0.03035	0.00551	0.33932	0.00921	0.00000	0.00662
**Cbfb**	6.99494	6.77761	3.16228	4.08629	3.75502	1.37966	6.10865	3.18851	7.41725	6.97748	2.59459	6.23963	1.36952	11.91662	5.37052	0.63254
**Cbl**	0.45921	0.00376	2.87499	7.68396	0.02426	0.00653	3.03276	3.36533	5.10342	3.73819	0.77487	6.35541	0.79101	9.14315	11.49541	1.60835
**Cblb**	0.00000	1.49184	0.00000	0.00000	1.61343	0.00728	1.26838	0.00000	0.01027	0.00000	0.53869	0.01060	0.56262	0.01010	0.00000	2.18904
**Cblc**	3.82548	2.28620	0.01109	0.00000	5.49518	0.00594	0.01383	0.00000	0.00862	0.77557	0.65892	0.00000	0.21490	0.00789	0.00243	0.00289
**Cbll1**	4.77868	5.92210	2.53485	14.07435	0.02258	0.02078	1.33564	3.02713	18.50685	6.14439	5.67585	7.61707	3.33441	9.73326	11.68621	3.37233
**Cbln1**	0.03932	0.00683	0.00002	2.45452	3.18763	0.00781	0.04235	0.25238	1.68348	0.57656	0.00000	0.00000	0.00708	0.00300	0.83095	0.00649
**Cbln3**	0.27057	0.04897	1.06889	0.26114	0.32075	0.06106	0.09524	0.00000	0.52486	0.32534	5.15567	0.38837	1.05348	1.23985	0.27670	0.20535
**Cbr1**	18.44685	0.09536	3.47985	2.25478	7.43099	0.01633	5.34951	18.39430	3.58433	12.19395	11.55360	0.43802	8.83149	1.20150	0.15763	10.34129
**Cbr2**	0.07624	0.00000	7.76563	0.00000	4.60482	0.99182	4.66470	5.93398	0.46321	9.74724	7.37600	0.05195	9.74544	0.06195	0.99903	0.00000
**Cbr3**	0.06124	0.01817	0.23907	0.00810	0.00815	0.00569	0.02773	0.05042	0.03400	0.33159	0.01226	0.00632	0.02930	0.01234	0.00595	0.01515
**Cbr4**	2.31676	3.80802	3.25821	0.00345	1.80264	0.00319	2.26992	1.05266	0.01045	0.45533	0.00000	0.09892	1.03548	0.00439	0.05139	0.01629
**Cbs**	0.00000	0.02737	2.29284	0.71347	0.01257	7.30245	0.63474	2.98591	0.00000	2.52641	0.56425	0.03854	0.22105	0.03730	0.00000	3.31454
**Cbwd1**	0.94019	0.22363	1.21066	1.21231	2.07493	1.35115	0.82902	2.25076	5.65479	0.79386	2.71613	5.00063	0.32937	8.20668	6.81322	0.01133
**Cbx1**	4.89382	0.03382	0.41732	0.93922	0.02988	0.27811	0.17242	2.89820	0.98120	0.73469	0.98738	0.19781	3.43030	0.53797	0.51094	2.59680

**Cbx2**	0.62454	0.00581	0.00000	7.85594	1.59481	0.00488	0.00669	1.04877	4.82262	1.12525	0.14366	0.02701	0.03827	0.00000	6.23354	0.35209
**Cbx3**	8.69809	6.35853	6.70681	11.52667	11.43758	4.68193	3.81373	5.75294	17.18585	10.08600	15.21041	4.25380	2.58334	19.37240	13.30845	2.12207
**Cbx4**	0.03677	0.00783	0.01030	0.33641	0.00833	0.00671	0.00739	0.02823	0.00589	0.01409	0.03044	0.00801	0.01137	0.01089	0.00700	0.00728
**Cbx5**	15.01701	4.43582	9.56552	6.13942	10.44253	10.03151	2.57053	6.92875	13.53616	16.74515	6.32090	14.66156	5.86127	25.15774	14.48633	3.00279
**Cbx6**	0.01693	0.00834	0.02938	0.00646	0.09877	0.00618	0.00754	0.01298	0.01130	1.03486	0.01055	0.04350	0.01791	0.00739	0.11263	0.95679
**Cbx7**	7.41423	4.69183	4.97660	2.33637	6.07096	0.00490	7.45889	5.20175	3.65078	4.91430	2.34938	0.88252	3.98979	2.74424	5.66957	2.13593
**Cbx8**	0.00583	0.00612	0.17324	0.44195	2.20786	0.00872	0.00699	0.86975	0.16521	0.02963	0.00493	0.00247	0.13620	0.03290	0.20691	0.00719
**Cby1**	1.63453	0.02566	2.31605	3.79314	0.00976	0.01283	0.00548	3.79325	0.88372	1.73826	0.66224	0.00000	0.06259	0.70024	1.31636	0.00429
**Cc2d1a**	3.66019	0.00187	0.05741	0.37162	1.79416	0.01853	0.48264	3.00237	0.57519	2.35834	0.03926	0.05713	8.51173	0.01186	0.01516	0.01469
**Cc2d1b**	6.33547	2.39178	3.23561	1.17792	0.51279	0.02431	5.98344	7.44964	1.32766	0.00000	0.63718	1.13355	6.66470	0.00000	0.46859	2.18682
**Ccar1**	7.45435	4.00880	6.74977	16.32083	1.36611	4.81899	5.47839	9.38121	17.13433	17.07157	10.08069	12.07648	8.50955	29.26608	23.56868	5.12804
**Ccar2**	1.14777	0.12250	0.00000	0.08085	0.00642	0.00709	1.41367	0.77390	0.02706	3.14765	0.00400	0.01657	1.10508	0.00820	0.59813	0.00565
**Ccbe1**	0.01355	0.00781	0.01132	0.02099	0.00909	0.00667	0.00728	0.01053	0.24922	0.51633	0.01032	0.04365	0.01219	0.01180	0.00662	0.00778
**Ccdc106**	0.68433	0.00621	0.00184	0.00520	0.00639	0.00799	1.24896	0.00821	0.07944	0.34431	0.01190	0.00501	0.01103	0.01131	0.00981	0.00688
**Ccdc107**	0.87105	0.00568	0.00000	3.52958	2.23510	1.58530	0.00252	0.20099	3.54230	1.37124	2.76764	0.03572	5.25731	0.06335	0.00000	0.01785
**Ccdc109b**	0.09284	0.00915	0.00402	1.51084	0.01586	0.00768	0.00805	0.54517	0.44370	0.00551	0.01836	0.00693	0.00572	0.00927	0.01756	0.00715
**Ccdc112**	1.66029	0.00404	0.23774	0.02329	0.85596	0.00587	1.45368	0.00150	0.02701	0.00939	0.01115	0.00379	0.02190	0.01768	0.24896	0.01246
**Ccdc114**	0.01360	0.00807	0.01198	0.77405	0.00916	0.00682	0.00753	0.01305	0.00000	0.28679	0.01224	0.00856	0.01268	0.01228	0.01284	0.00728
**Ccdc115**	4.20595	2.80443	8.19887	12.37020	6.35394	5.88514	5.10391	11.97653	16.42558	14.40905	7.67660	4.31264	11.76622	4.45133	6.19007	0.09605
**Ccdc116**	0.01561	0.00788	0.55292	1.16220	0.00808	0.00554	0.00732	0.08393	0.34554	0.00850	0.02605	0.03907	0.00627	0.01518	0.00000	0.00693
**Ccdc117**	1.02069	5.16507	12.31049	17.10938	0.43604	7.17388	2.15096	14.07063	18.71203	27.60522	13.75365	19.33006	3.21355	28.53786	27.17032	0.69033
**Ccdc12**	6.97416	3.95312	5.59019	9.34781	3.58681	6.03564	3.16289	7.83414	7.77699	5.99692	10.54937	1.74941	9.49187	0.22773	0.94525	9.42843
**Ccdc120**	0.19874	0.00822	0.00000	3.71323	0.01545	0.00678	0.00696	0.94626	0.00000	0.15030	0.01881	0.01399	0.00579	0.00583	1.90118	0.00672
**Ccdc124**	12.66939	0.07352	0.00000	4.32336	10.42172	11.44109	6.06661	30.21755	9.19448	25.70998	6.76999	2.43162	28.10654	0.00000	0.00000	7.25640
**Ccdc125**	0.05233	0.00747	0.07102	0.57590	0.04613	0.00619	0.02724	0.01342	3.96085	1.08019	0.10191	0.00000	0.03605	0.96971	2.46341	0.00692
**Ccdc126**	2.82616	2.85592	2.09554	7.01893	1.58596	3.43766	0.71454	0.90735	9.69855	1.04574	0.43411	0.40148	1.01596	1.11320	2.18599	0.32628
**Ccdc127**	5.44665	7.90206	5.81500	1.66583	0.67716	5.12457	4.39916	1.52131	0.07958	4.71701	2.78137	1.00592	4.76564	4.46365	3.82957	0.35214
**Ccdc13**	0.00619	0.00644	1.12911	0.06260	0.01192	0.00905	2.07831	1.39174	0.00775	0.00000	0.00001	0.00231	1.28067	0.01696	0.00000	0.12115
**Ccdc130**	1.45258	4.85491	6.55847	1.43474	0.03403	0.01891	7.91934	3.37590	2.71599	0.98262	0.43770	0.23859	2.17901	0.21736	0.00000	0.90941
**Ccdc134**	0.36343	0.48686	0.35360	4.44884	0.06749	0.12579	0.02542	2.25499	4.70339	0.00000	2.20788	0.08707	1.75368	0.00000	1.84572	0.15362
**Ccdc136**	0.01294	0.00774	0.01136	0.08754	0.00893	0.00662	0.00740	0.01048	1.19078	1.09819	0.01138	0.00254	0.01167	0.00515	0.32940	0.00737
**Ccdc137**	1.66272	4.79159	8.44994	12.04374	0.02647	5.96871	5.28280	2.90067	19.96711	17.89703	8.33349	11.46025	6.24503	14.43695	16.03301	2.63702
**Ccdc138**	1.02789	0.00670	0.19703	0.31608	0.02567	0.00788	0.78685	0.00209	0.00418	1.67704	0.28382	0.32685	0.36588	1.54593	0.74290	0.00596
**Ccdc14**	1.09693	1.12814	2.77640	1.05077	0.01043	0.00737	3.80587	0.11420	0.60665	0.93031	0.09091	0.03647	0.48126	5.60714	0.42468	0.42077
**Ccdc141**	0.01785	0.00791	0.01179	0.18481	0.00902	0.00667	0.00751	0.01395	0.00589	0.01426	0.01202	0.00800	0.01275	0.01622	0.02233	0.00736
**Ccdc142**	0.71905	0.00637	0.01018	0.14659	1.44768	0.00580	0.00706	0.00836	0.00819	0.00710	0.01268	0.03952	0.00868	0.01233	0.00747	0.59735
**Ccdc146**	0.01299	0.00793	0.01213	0.00662	0.00885	0.00660	0.00734	0.01037	0.01159	1.41980	0.01074	0.00449	0.01087	0.01194	0.00818	0.00734
**Ccdc148**	0.01353	0.00813	0.01110	0.16060	0.00931	0.00660	0.00756	0.01420	0.21568	0.02212	0.05279	0.00740	0.01915	0.04913	0.17612	0.00737
**Ccdc15**	0.01210	0.00810	0.15138	0.42372	0.00888	0.00565	0.08177	0.40516	0.09876	1.03356	0.01029	0.00575	0.00503	0.01610	0.05707	0.00694
**Ccdc150**	0.00962	0.00796	0.00744	0.00949	0.00961	0.00679	0.00785	0.65723	0.01131	0.00000	0.00547	0.23111	0.00630	0.00682	0.24412	0.00730
**Ccdc152**	0.30496	0.00626	0.00277	0.00274	2.40444	0.00787	0.00701	0.00300	0.01080	0.35090	0.16641	0.02210	0.01445	0.05580	0.06154	0.00667
**Ccdc153**	0.01667	0.00765	0.01507	0.32052	0.00837	0.00738	0.00708	0.00000	0.02717	2.74662	0.46323	0.00000	0.00793	0.02756	0.19795	0.00722
**Ccdc154**	0.00855	0.00778	0.00372	0.28459	0.00957	0.00775	0.00705	0.37633	0.75309	0.49305	0.73156	0.01210	0.00426	0.00469	0.31306	0.00714
**Ccdc155**	0.99036	0.00672	2.29173	1.27438	0.01093	0.00590	0.00594	0.00325	0.13673	1.13747	0.20218	0.06340	0.00522	0.00584	0.00000	0.00565
**Ccdc157**	0.01360	0.01835	0.65485	2.77132	0.00981	0.00489	0.01745	0.32240	4.76332	0.05549	0.00000	0.00611	0.00607	0.00000	0.49145	0.00647
**Ccdc158**	0.01378	0.00766	0.01161	0.28506	0.00941	0.00655	0.00743	0.01203	0.10996	0.00624	0.01034	0.00694	0.01289	0.01940	0.33870	0.00733
**Ccdc160**	0.15481	0.00766	0.01290	0.46993	0.00759	0.00619	0.01730	0.01053	0.70974	0.03715	0.05794	0.00538	0.01139	0.00558	0.47225	0.13137
**Ccdc163**	1.95643	0.01555	4.97818	1.23529	0.00000	0.01084	0.88178	4.96753	1.07942	6.91893	6.81046	1.21885	2.19177	6.36087	6.96793	0.00853
**Ccdc166**	0.00903	0.05801	0.02733	3.63875	0.00971	0.00715	0.00657	0.07522	2.44954	1.51775	0.00000	0.00532	0.81327	0.00000	0.61827	0.04323
**Ccdc167**	0.63993	0.59420	0.65335	0.00219	0.03888	0.00847	0.77166	0.07526	0.07769	0.93545	0.12759	0.00000	0.10129	0.47926	0.25272	0.09035
**Ccdc169**	0.02363	0.00745	0.00737	0.00631	0.00881	0.00667	0.00725	0.01394	0.20344	0.00467	0.00610	0.00262	0.01308	1.41623	0.00000	0.00740
**Ccdc17**	0.01550	0.00765	1.19913	0.00453	0.01621	0.00564	0.00656	0.00597	0.05148	0.02260	0.08358	0.01475	0.81622	0.00752	0.52826	0.00681
**Ccdc170**	0.01388	0.00770	0.53381	1.67298	0.01029	0.00537	0.46415	0.07683	0.57261	0.00347	0.02483	0.00587	0.01133	0.00404	0.19610	0.01401
**Ccdc171**	0.02011	0.00731	0.26604	0.15311	0.00822	0.00553	0.00688	0.00000	0.46343	2.27529	0.40697	0.00452	0.00709	0.20459	1.30185	0.00695
**Ccdc172**	0.01200	0.00772	0.00724	0.01220	0.00937	0.00658	0.00711	0.00919	0.01233	0.75135	0.00682	0.01874	0.01167	1.20555	0.10593	0.00743
**Ccdc173**	0.00974	0.00779	0.00355	0.84610	0.01992	1.94192	0.00719	0.03817	0.29660	0.72206	0.00000	0.01458	0.03110	1.12871	2.80352	0.00688
**Ccdc174**	1.82807	6.55421	7.44146	2.05287	5.34762	1.20915	4.34005	3.22126	1.36198	6.51537	4.20526	0.30693	2.39702	1.15602	6.03830	2.85504
**Ccdc177**	0.01231	0.00775	0.00648	0.34040	0.00966	0.00652	0.00716	0.00942	0.40926	0.00000	0.00000	0.00000	0.01391	1.40458	0.66504	0.00744
**Ccdc18**	0.00000	0.00761	0.45233	0.27831	0.07133	0.00555	0.00724	0.29329	0.50597	0.00000	0.00000	0.01414	0.00436	3.06048	1.09607	0.00687
**Ccdc181**	0.02010	0.00758	0.01040	0.78045	0.00856	0.00669	0.00726	0.00997	0.27969	1.14468	0.00944	0.05395	0.01158	0.01362	0.01798	0.00747
**Ccdc183**	0.01332	0.00817	0.02225	0.00564	0.00931	0.00625	0.00762	0.01389	0.16846	0.01377	0.02362	0.00805	0.01223	0.01285	0.00871	0.00739
**Ccdc184**	0.43308	0.00804	0.29761	0.01095	0.01692	0.00875	0.00702	0.01093	0.01221	0.01350	0.01203	0.00746	0.00973	0.01550	0.01193	0.06658

**Ccdc186**	0.00620	0.00772	0.00467	1.59245	0.00948	0.00616	0.00663	0.64256	1.13598	0.14851	0.01504	0.02757	1.06133	0.46565	3.01096	0.00658
**Ccdc189**	0.05898	0.00762	1.34738	0.96964	0.02279	0.00834	0.00602	0.01545	0.00000	1.74580	0.01243	0.06736	1.88500	0.24202	2.14298	1.14874
**Ccdc191**	0.37572	1.99969	1.15627	0.00000	0.00528	0.00930	3.99167	0.00002	0.01094	0.10551	0.24556	0.00000	0.58422	1.65347	0.00000	0.00537
**Ccdc22**	2.69723	0.03763	5.29630	0.00464	0.02926	0.01085	1.06235	2.50309	0.00926	1.69163	0.00000	0.03496	5.23950	0.00536	0.10561	1.49725
**Ccdc24**	0.01374	0.00814	0.01227	1.09684	0.00941	0.00648	0.00743	0.01313	0.09724	0.02378	0.01215	0.00781	0.01281	0.00542	0.12089	0.00726
**Ccdc25**	4.23139	9.59382	5.89238	10.91681	8.53215	6.26615	3.56461	4.77596	14.79646	12.17226	6.11308	6.34982	1.97346	25.35501	12.68638	0.15080
**Ccdc28a**	6.70575	0.00000	3.96093	1.90363	0.01677	2.81562	2.47604	2.49764	4.78523	4.93937	1.03763	2.09131	0.79551	0.01790	1.69876	2.60892
**Ccdc28b**	3.75552	0.00380	1.50856	2.19431	0.00000	2.40010	0.00390	0.54698	1.94444	0.00000	0.28975	0.01947	0.21004	0.00355	0.01084	0.01530
**Ccdc30**	0.01734	0.00665	0.19715	0.00455	0.01779	0.00568	1.58503	0.00939	0.71945	1.16913	0.00585	0.02213	0.01013	0.01240	0.02511	0.00720
**Ccdc32**	1.95890	3.31786	4.87430	1.93888	5.45630	2.58528	5.73689	0.32096	3.90551	3.35976	1.20761	0.16354	1.44497	0.74507	2.51921	4.43774
**Ccdc34**	3.68401	3.81748	0.00000	2.67027	7.38018	2.67118	5.39671	0.28264	0.00000	3.62753	0.01156	0.02820	0.29938	2.42821	1.05790	2.82126
**Ccdc36**	0.07100	0.05114	0.11516	5.35929	0.01156	0.01230	0.05709	0.01389	3.78819	2.92918	1.08671	0.04938	0.05050	0.58299	2.22718	0.04271
**Ccdc38**	0.00982	0.00745	0.00976	0.00447	0.17935	0.00658	0.00739	0.01139	0.11659	0.00459	0.01036	0.00662	0.01167	0.01268	0.45857	0.00725
**Ccdc39**	0.00602	0.40791	0.00663	0.00423	0.00832	0.00627	0.00709	0.09943	0.11649	0.01145	0.00000	2.39922	0.00858	0.00747	0.00000	0.00701
**Ccdc40**	0.01369	0.00783	2.61277	2.01829	0.00403	0.00699	0.20719	1.01652	0.62068	0.89462	0.36296	0.12209	1.16163	0.00134	0.46971	0.00532
**Ccdc42**	0.01009	0.00795	0.11897	0.20877	0.01747	0.02646	0.00736	0.01260	0.00646	0.01042	0.01233	0.00795	0.07094	0.01201	0.00515	0.23377
**Ccdc43**	2.01255	2.71938	5.13090	0.00000	3.11002	7.58106	1.69746	12.77359	0.03541	7.32214	5.14236	1.72078	6.82694	13.01703	0.00000	0.38346
**Ccdc47**	3.78685	1.80476	9.95067	6.39298	3.31783	1.32431	6.12884	8.37417	4.21666	0.85873	4.80247	3.13508	4.57906	1.72097	8.23922	2.35471
**Ccdc50**	2.36474	2.91300	3.29341	5.43972	0.03032	2.60072	2.14335	0.27348	3.18503	3.39934	0.22072	0.10180	0.16452	2.02231	2.41181	3.02238
**Ccdc51**	4.40754	3.84131	1.91788	0.37710	0.00000	2.26558	3.59793	2.37409	0.00427	2.28670	2.03423	0.22667	0.00000	0.00000	0.97585	0.24952
**Ccdc53**	12.63946	4.19235	6.08283	0.00000	3.93767	5.46408	3.41200	5.29517	0.15394	4.60364	2.54932	0.89371	3.78933	1.02828	0.97823	0.69446
**Ccdc57**	0.89592	0.00647	0.00841	0.74916	0.00601	0.01128	0.00556	2.57995	0.34218	5.09611	0.52451	0.16279	1.67654	0.00498	2.23240	0.00548
**Ccdc58**	11.15769	6.16192	9.87707	4.22996	11.07530	3.73602	3.14090	5.72493	2.06151	7.32723	9.68013	1.75588	10.69003	10.16008	4.54720	1.71712
**Ccdc59**	10.46329	7.84660	5.20709	10.98061	0.10175	0.02448	6.91022	10.45770	13.39346	15.09951	13.15549	2.55315	9.20575	2.20723	2.11610	6.24708
**Ccdc6**	6.58630	2.48847	1.66556	24.53555	0.43702	1.01964	0.02981	1.54565	18.51281	10.21179	4.97680	2.49516	0.66495	15.30755	31.25139	3.65334
**Ccdc60**	0.00614	0.00861	0.00832	0.97480	0.00930	0.00664	0.00724	0.35057	0.01232	0.02411	0.00938	0.00701	1.61515	0.03732	0.00000	0.00651
**Ccdc61**	2.99490	0.42409	0.00000	0.86480	0.06986	1.45778	0.02374	0.04538	0.00000	2.79502	4.55381	0.02260	0.15463	0.00000	0.00001	0.19732
**Ccdc62**	0.08676	0.00846	0.00939	0.49287	0.01346	0.00685	0.00745	0.01343	0.00689	0.04781	0.01316	0.00811	0.01526	0.01314	0.00562	0.00739
**Ccdc63**	0.03141	0.00712	0.00195	0.92664	0.18089	0.00672	0.00687	0.00000	5.06838	0.61845	0.21644	1.59633	0.00915	0.00562	0.15728	0.00639
**Ccdc64**	0.00439	0.72487	0.19441	0.00750	0.00775	0.00602	0.10752	0.30722	0.11200	0.10656	0.00936	0.00665	0.40100	0.01021	0.00184	0.00684
**Ccdc64b**	0.01214	0.00803	0.01817	1.05251	0.00904	0.00726	0.00730	0.01580	0.87558	1.50615	0.00612	0.04358	0.01549	0.00847	0.01516	0.00740
**Ccdc65**	0.00442	0.00777	0.00712	0.10769	0.02023	0.00660	0.00690	0.36251	0.00958	0.26006	0.00000	0.01967	0.52592	0.94924	0.72636	0.00729
**Ccdc66**	0.39031	2.43439	0.82712	1.53945	5.00497	0.89205	0.00458	2.10008	0.78172	0.13675	1.18773	3.81474	0.70016	2.61828	5.73629	1.07369
**Ccdc68**	1.30983	0.00709	0.00275	0.00890	0.01646	0.00669	0.00638	0.00778	0.01207	0.01262	0.01139	0.00624	0.01037	0.00616	0.28089	0.24523
**Ccdc69**	0.01322	0.00741	0.00670	14.60153	0.00891	0.00514	0.00655	0.00000	1.20874	0.90561	0.31104	0.06026	0.00855	0.00000	1.12594	0.00454
**Ccdc71**	1.73555	0.00332	3.17120	1.68745	0.00786	0.00483	3.54484	1.33557	0.00000	3.00913	0.54765	0.00000	3.58369	0.90193	1.73393	0.00940
**Ccdc71l**	0.00743	0.78339	0.04357	3.00464	0.00811	0.00467	0.01669	0.00550	8.63610	0.00000	0.00000	0.19277	0.01095	0.00000	0.37846	0.00576
**Ccdc73**	1.29420	0.00644	0.00979	1.12605	0.00543	0.00530	0.00679	0.01343	0.20025	0.84397	0.01022	0.02849	0.01059	0.01316	0.25639	0.00562
**Ccdc74a**	0.00991	0.00810	0.08153	0.94828	0.00907	0.00555	0.00699	0.75186	0.58963	0.17361	0.37668	0.00314	0.67494	0.00477	0.04066	0.00655
**Ccdc77**	2.18570	5.10471	6.51059	5.52330	7.32288	3.47508	1.58946	5.42215	6.08926	2.03669	1.25187	0.16482	4.49845	10.36229	6.69650	2.01099
**Ccdc78**	0.04118	0.00810	0.19072	0.14691	0.03280	0.02119	0.04899	0.05498	0.21675	0.83945	0.57931	0.01332	0.11726	0.11124	0.21986	0.01457
**Ccdc7b**	0.04986	0.00786	0.09310	0.13716	0.00796	0.00577	0.00765	0.00748	0.09073	0.00916	0.68573	0.00521	0.00592	0.04635	0.03380	0.00701
**Ccdc80**	0.01361	0.00787	0.00936	0.00928	0.00917	0.00672	0.00744	0.01335	0.01211	0.32246	0.01044	0.23436	0.01145	0.01314	0.00474	0.00720
**Ccdc81**	0.27998	0.00719	1.35919	0.01079	0.00500	0.00832	0.00689	0.31095	0.01195	0.07958	0.00885	0.00565	0.00271	0.01387	0.00000	0.00638
**Ccdc82**	1.72030	0.62307	2.59968	44.47688	0.28563	2.92036	2.59037	2.45927	39.14170	37.01031	7.16566	7.98649	4.87316	43.73344	52.40767	0.74025
**Ccdc84**	1.39481	0.00604	0.00000	0.63937	0.00542	2.47975	0.00520	0.63590	0.22771	2.49777	1.56973	1.40901	1.61988	0.60735	0.41375	0.01121
**Ccdc85a**	0.01325	0.00756	0.01746	0.45222	0.00926	0.00714	0.00721	0.01058	0.02297	0.00865	0.01020	0.45342	0.01304	0.02593	0.00000	0.00742
**Ccdc85b**	0.02204	0.00827	0.08626	2.07253	0.03564	0.03056	0.01736	0.01703	1.37871	0.09949	0.86808	0.08007	0.04454	0.23952	0.34855	0.05754
**Ccdc86**	9.46090	0.81236	0.00000	14.25994	8.23570	5.80321	1.25180	20.15412	18.77238	18.18560	6.25844	3.16689	14.61927	2.79111	5.16640	2.10969
**Ccdc87**	0.00952	0.00791	0.02011	0.20328	0.00905	0.00601	0.00667	0.01084	0.00867	0.00547	0.01351	0.00736	1.25725	0.01223	0.00712	0.00653
**Ccdc88a**	0.01182	0.00768	0.00619	0.51632	0.00957	0.00572	0.05746	0.01392	1.67894	0.07540	0.01195	0.03349	0.01223	0.03926	8.33733	0.00715
**Ccdc88b**	0.01340	0.00810	0.01166	0.01342	0.00906	0.00672	0.00741	0.01137	0.70040	0.48320	0.00778	0.00586	0.01236	0.03411	0.00454	0.00754
**Ccdc88c**	1.28563	0.66571	0.98753	0.00000	0.00527	0.00559	0.32113	4.09716	0.21461	0.59082	0.00000	0.05550	1.01825	0.00302	0.62414	0.01307
**Ccdc89**	0.01265	0.00820	1.10782	0.07575	0.00740	0.00564	0.00764	0.00825	0.01081	0.00435	0.32673	0.00680	0.00552	0.01049	0.00000	0.00681
**Ccdc9**	7.35004	2.73879	1.60609	5.70565	4.44809	0.53214	0.33517	5.13990	8.12827	10.43172	6.94300	0.61849	2.78711	0.05148	2.47387	5.73301
**Ccdc90b**	6.38570	6.67161	3.64946	2.64150	9.53067	3.43740	3.47479	2.15872	1.14740	4.02519	5.99661	1.26343	3.01499	0.26107	1.98958	1.03265
**Ccdc91**	3.43160	2.84108	0.00000	2.04103	3.93757	6.77876	2.39990	2.07225	0.70925	0.37500	0.78012	0.37045	1.06659	0.00000	1.91371	1.53836
**Ccdc92**	0.63993	0.04592	1.01494	4.22889	2.70908	0.00479	3.99731	1.05635	0.90630	0.00000	1.66896	0.05955	2.53929	0.00000	0.24192	2.88792
**Ccdc92b**	3.26931	0.01231	0.11795	0.00000	3.31835	0.00258	0.00252	5.41318	4.89765	5.13849	5.92271	0.67638	0.00000	1.84447	1.30284	0.02435
**Ccdc93**	6.19161	7.00786	10.00801	0.29470	1.14201	0.00000	3.46551	8.62418	1.50640	7.25798	5.04184	9.23223	2.04488	10.36705	5.83637	6.23431
**Ccdc94**	2.43101	1.70507	1.98924	0.44553	0.01149	0.01814	5.47144	7.51086	1.27297	7.69690	1.02417	0.18687	2.60816	0.00827	0.13185	0.00909
**Ccdc96**	0.01627	0.00736	0.33711	0.75718	0.00831	0.00547	0.00694	0.00025	0.61704	1.09552	0.28993	0.00237	0.00746	0.01111	0.00000	0.01370

**Ccdc97**	2.38656	0.01448	1.43458	2.28839	0.11516	0.37620	0.64277	6.60378	1.20045	2.68607	1.78757	1.44058	4.13947	0.59249	0.38746	1.55284
**Cchcr1**	0.00000	0.00687	0.00643	1.49933	0.02458	0.00865	0.18040	1.14088	1.66771	2.96678	3.72815	0.10224	1.00234	0.00000	0.00000	1.70526
**Cck**	0.01020	0.12165	0.20887	0.01063	0.00869	0.00647	0.00711	0.01593	0.01235	0.00580	0.01336	0.00743	0.46615	0.01313	0.00505	0.01427
**Ccl22**	0.01289	0.00764	0.01053	0.00512	0.00889	0.00659	0.00724	0.01135	0.01102	1.25996	0.12750	0.02551	0.00964	0.00754	0.23012	0.00714
**Ccl25**	0.00481	2.94972	1.01830	0.16503	0.00595	0.00564	0.00631	0.91132	0.00738	1.44376	0.90007	0.00021	0.00337	1.13817	1.07979	0.00566
**Ccl27a**	0.01056	0.00779	0.01119	0.01051	0.00909	0.00661	0.00729	0.06183	0.01233	0.04118	0.01265	0.00741	0.00922	0.01157	0.00982	0.15939
**Ccl28**	0.01225	0.00809	0.02383	0.05337	0.00924	0.00761	0.00776	0.03391	0.06035	0.09589	0.01273	0.00837	0.01060	0.01968	0.02660	0.00720
**Ccl9**	0.01345	0.02014	0.56616	0.11754	0.00870	0.48900	0.04162	0.85687	0.09303	1.00953	0.26070	0.05432	1.09828	0.27586	0.09308	0.02130
**Ccm2**	2.96579	0.02386	5.13620	0.33350	0.03619	3.49186	3.67156	2.51578	0.07181	3.04740	0.29246	0.11589	2.68244	0.00319	0.21152	0.77410
**Ccm2l**	0.01429	0.00863	0.00865	0.00612	0.00896	0.00688	0.00807	0.07185	0.01155	0.26854	0.23462	0.01831	0.00708	0.01186	0.00453	0.00706
**Ccna2**	9.81100	8.87894	12.12091	28.09333	7.46092	3.65006	3.51594	14.17998	38.64917	33.82016	23.49623	23.76735	9.01051	23.87061	27.00080	2.62167
**Ccnb1**	25.69767	15.32735	21.53973	22.40585	7.92291	13.00819	14.16666	35.65476	33.66982	46.33487	33.78595	31.49652	23.11402	45.29196	37.96821	8.23698
**Ccnb1ip1**	2.39755	3.85959	1.43522	2.94143	3.88723	2.29450	3.79970	3.91227	0.66915	6.81167	2.43376	6.46387	3.43996	2.19079	7.87880	1.02774
**Ccnb2**	12.81744	9.80727	12.81888	4.02228	13.93042	10.08116	6.47348	7.30080	7.02379	16.51465	14.46824	7.38934	12.65592	3.90246	5.24451	8.86988
**Ccnb3**	0.01083	0.00769	0.00435	0.02651	0.00973	0.00634	0.00707	0.00799	0.01142	0.25481	0.00123	0.00000	0.01280	5.04877	0.00461	0.00729
**Ccnc**	9.29991	4.77106	4.54464	4.74929	2.79719	0.33638	0.00000	3.55127	4.52878	6.45863	3.69469	0.89936	2.68748	6.29579	6.46734	2.20035
**Ccnd1**	0.00000	0.00762	0.00000	0.04512	0.01801	0.00654	0.00296	0.36520	0.66728	2.90607	2.26072	0.05209	7.26222	0.01819	2.33158	0.02615
**Ccnd2**	0.00898	0.00709	0.11246	0.00760	0.00908	0.00942	1.14926	0.01062	0.01171	0.55375	0.00686	0.15520	0.01074	0.63869	0.00000	0.00727
**Ccnd3**	15.79686	6.58334	13.30623	0.96157	10.55890	4.93593	11.22422	20.95168	1.31970	9.53143	4.50474	0.68050	13.91820	0.35591	0.69955	6.34018
**Ccndbp1**	6.32094	0.38580	6.01223	19.92686	3.55811	6.61509	2.79948	7.39433	12.52010	7.49555	2.76403	4.94595	5.07941	0.78313	10.48424	7.61436
**Ccne1**	2.60726	6.26818	13.42710	19.98333	4.54006	12.06227	10.75695	22.23347	32.29112	27.38221	23.50871	14.39734	13.78640	10.03816	22.04140	0.04171
**Ccne2**	0.00871	0.00692	0.02518	0.60939	0.00933	2.40074	0.84820	0.74620	0.13111	0.21230	0.00000	2.05823	0.00000	2.77084	1.59317	0.00615
**Ccnf**	4.98280	4.98111	7.28437	8.32379	1.90462	2.77656	3.27561	10.81364	12.04024	11.20199	8.39015	5.01413	3.26575	0.00000	7.94871	0.51170
**Ccng1**	8.24789	8.48334	4.11141	1.34493	11.89121	3.71742	10.30970	3.52662	0.00000	0.00000	4.83825	0.66306	4.24196	0.69523	0.00000	4.04408
**Ccng2**	0.44153	0.00780	0.52502	3.66547	0.01630	0.00789	0.00669	0.01028	3.47112	0.35596	0.00000	0.02224	0.00863	0.00000	2.89878	0.00582
**Ccnh**	12.67184	6.76443	2.55599	0.46995	8.01146	4.50514	1.76168	4.97194	5.94425	8.18939	7.30012	7.89907	7.04585	4.57831	2.79681	4.34654
**Ccni**	4.62130	4.72123	6.75793	1.85430	4.34632	5.25679	1.88740	1.78683	3.45184	2.90642	1.24488	7.88971	1.03972	1.30643	3.06881	1.32447
**Ccnj**	0.01050	0.00829	0.00785	3.17490	0.00941	0.00625	0.00802	0.14023	1.44372	0.11955	0.00024	0.04511	0.00838	0.02959	1.15412	0.00713
**Ccnjl**	0.01346	0.00822	0.01165	0.49958	0.00929	0.00654	0.00753	0.01346	1.78544	0.00592	0.00000	0.00491	0.01326	0.03362	0.00642	0.00725
**Ccnk**	3.23798	0.66795	1.70786	9.16505	3.97818	0.00927	1.58009	2.40599	13.21983	6.29698	7.94981	0.72355	2.43887	1.83630	0.84132	1.98237
**Ccnl1**	3.46940	8.14263	1.06894	4.41572	5.13487	0.82215	6.87359	4.79484	6.62891	10.50833	3.69527	10.13676	6.52447	9.76614	12.60024	0.23755
**Ccnl2**	8.27088	4.66446	8.72332	17.03305	2.96132	5.47037	4.42692	7.55679	20.55049	13.23488	10.25599	10.79605	11.36369	2.39935	11.37752	6.06991
**Ccno**	1.05182	0.00631	0.00376	6.41711	0.00581	0.00589	0.00614	0.00000	6.66440	2.00635	0.00000	0.00935	0.01065	0.04603	0.01154	0.00460
**Ccnt1**	0.67630	1.20305	3.32604	2.49925	0.00000	0.00424	0.31160	1.59993	3.40332	4.89710	0.83525	3.30627	1.63113	5.08834	3.67639	0.01766
**Ccnt2**	1.20362	5.41045	0.35452	3.11069	0.02156	0.10069	0.22507	2.77473	12.88825	1.07257	4.82206	0.38339	1.07314	0.64628	5.20546	0.05946
**Ccny**	1.54033	0.84325	1.55879	3.79349	0.97729	0.00707	1.25037	2.23880	1.94714	2.48226	0.00000	0.10273	1.38961	0.00197	1.09720	2.47110
**Ccnyl1**	5.17410	0.88056	2.65476	5.92846	1.23453	0.13386	2.32406	0.17169	9.70003	1.23983	0.16828	10.19575	2.16496	3.45876	2.27012	0.04670
**Ccp110**	4.81376	0.03972	3.59580	7.72774	4.58127	0.00783	2.20776	0.47843	4.85592	3.15446	3.27000	4.49095	0.07461	5.49256	7.51559	1.21566
**Ccpg1**	2.07485	2.86518	7.79438	5.03850	0.00603	6.07976	5.74272	17.81428	13.44755	12.66975	10.99489	10.47031	7.11970	14.49144	19.19374	1.29675
**Ccpg1os**	3.27320	0.81230	1.21036	1.44878	0.00000	0.00257	1.96705	0.26693	2.13743	0.95753	1.26454	0.63674	1.90403	2.31341	0.65437	0.02393
**Ccr4**	0.00047	0.01893	0.80234	0.40607	0.00985	0.00549	0.01728	0.29911	0.25707	1.02148	0.04647	0.13883	0.00793	3.29665	0.11914	0.00704
**Ccr8**	0.01337	0.00804	0.01192	0.00608	0.00907	0.00642	0.00739	0.01082	0.01117	0.60815	0.00978	0.00556	0.01085	0.00563	0.45672	0.00711
**Ccr9**	0.01247	0.00806	0.01195	0.15396	0.00895	0.00659	0.00750	0.01359	0.17695	0.01163	0.01212	0.01849	0.01159	0.01212	0.00600	0.15819
**Ccs**	7.46731	0.96252	6.31355	3.02272	0.47677	2.61956	3.81366	9.69933	3.01676	4.06396	3.95092	0.07342	4.46413	1.04772	0.54752	3.62099
**Ccsap**	1.36060	0.00586	0.00000	0.43772	0.00529	4.41856	0.00594	0.00424	0.04421	0.00000	0.02376	0.04467	0.01466	0.04061	0.16924	0.00413
**Ccser1**	0.23763	0.00816	0.01366	0.89250	0.01816	0.00735	0.00726	0.01142	1.04851	0.01120	0.00629	0.00521	0.01489	0.00477	0.24356	0.00712
**Ccser2**	3.23527	3.27612	0.05634	8.40930	0.47363	0.00372	0.00276	0.25755	4.15863	1.40087	1.20538	0.25834	0.00000	8.25365	8.01428	0.00000
**Cct2**	36.54917	21.33922	29.89120	9.92236	28.25722	15.45122	20.77832	30.68576	15.50143	34.26656	28.51208	25.70817	28.57787	36.93065	21.44836	15.96385
**Cct3**	37.06612	23.55858	32.84795	15.77797	23.89065	16.18280	20.48986	35.63079	27.08150	31.56616	25.34089	17.87351	32.73718	30.93398	25.13037	19.48556
**Cct4**	32.85969	17.87184	22.11387	10.43455	21.86891	11.93803	12.00001	24.98758	23.30603	37.36288	27.28110	20.34394	31.81080	30.43067	21.05643	18.52951
**Cct5**	40.09100	24.84740	29.81953	6.00707	26.84368	18.43765	19.89086	37.13548	6.59624	31.24437	26.65991	17.50709	36.43051	33.24418	21.31400	21.68394
**Cct6a**	32.60440	21.55863	26.68263	9.05466	25.11043	16.28701	18.31558	32.52247	14.16776	29.58034	22.52056	12.63511	26.56841	23.22102	23.58866	16.90434
**Cct6b**	1.40609	0.00602	0.00000	1.15180	0.00514	1.60598	0.00519	0.00938	1.12501	0.00000	0.13076	0.07281	1.49124	0.74956	2.11174	0.01861
**Cct7**	34.49362	22.08667	36.43739	4.87783	24.38279	13.42112	22.97650	41.21971	12.58890	37.37511	24.81902	14.92011	31.59159	5.92445	21.61436	18.53667
**Cct8**	35.27338	18.16446	25.42367	15.21417	24.65114	13.79082	15.87358	30.98852	25.09666	31.11061	26.35285	17.98046	31.73452	28.06545	15.75326	17.55912
**Cct8l1**	0.01189	0.00767	0.00630	2.34811	0.00966	0.00620	0.00706	0.00828	1.93913	0.01805	0.00000	0.06820	0.01395	1.68844	0.28834	0.00697
**Ccz1**	9.16057	5.34631	7.54991	5.67210	10.24391	7.44112	4.38728	7.72668	6.63166	16.05805	8.79830	4.29990	5.08179	5.70719	14.15384	3.48082
**Cd109**	1.53999	0.00461	0.00000	0.00728	0.21938	0.00805	2.12154	0.00442	0.01195	0.51314	0.01182	0.03074	0.01044	0.01178	0.00700	0.00564
**Cd14**	0.01356	0.00795	0.01212	0.00646	0.00913	0.00676	0.00744	0.01187	0.06374	0.39867	0.01173	0.00709	0.01241	0.01063	0.01510	0.00759
**Cd151**	12.90223	4.88883	10.59794	2.36906	11.10772	7.66517	3.88383	9.89339	0.09197	5.09891	7.86904	0.27157	7.59554	0.38802	3.65965	3.31200
**Cd160**	0.02953	0.00778	0.00215	1.26181	0.01210	2.73549	0.00701	0.07166	0.00000	0.00352	0.00852	0.00748	0.00000	0.02174	0.09097	0.00615
**Cd163**	0.01374	0.00823	0.02847	0.29941	0.00943	0.03893	0.00758	0.01484	0.15282	0.01509	0.01209	0.01798	0.02530	0.14744	0.02079	0.00749

**Cd164**	8.74877	10.24419	9.71768	10.89358	7.29234	7.35569	10.18112	14.77670	11.96855	9.15901	12.85869	20.74823	9.98816	16.14374	13.72011	3.60114
**Cd164l2**	0.00469	0.00710	0.07289	6.30143	0.00861	0.00495	1.19696	0.28635	3.28930	4.54159	0.27166	0.13441	0.59509	0.06612	0.03140	0.00559
**Cd177**	0.01127	0.00783	0.01338	0.37837	0.00985	0.00712	0.00736	0.01207	0.13390	0.77783	0.01362	0.05022	0.39384	0.00541	0.12048	0.00800
**Cd19**	0.01351	0.00777	0.01158	0.12185	0.00910	0.00653	0.00739	0.01107	0.00986	0.46868	0.01095	0.02544	0.01142	0.00598	0.32074	0.00725
**Cd1d1**	0.01363	0.00785	0.00572	0.00965	0.00929	0.81233	0.00727	0.03823	0.01245	0.00985	0.02330	0.00779	0.00435	0.01232	0.00979	0.00701
**Cd1d2**	0.01436	0.00802	0.00720	0.01045	0.00924	0.26823	0.00748	0.01325	0.01241	0.01325	0.01247	0.00818	0.01612	0.01245	0.01058	0.00723
**Cd2**	1.76614	0.03663	0.00346	0.01060	0.00459	0.00664	0.00617	0.00788	0.01237	0.01532	0.01255	0.00691	0.01127	0.01220	0.01053	0.01270
**Cd200**	0.07383	0.00772	0.00897	0.00946	0.00797	0.00673	0.00727	0.01249	0.01230	0.26747	0.01258	0.02593	0.01184	0.01160	0.00840	0.07990
**Cd209c**	0.59849	0.36530	2.96603	9.26179	0.03644	0.00806	0.44049	15.24744	17.64895	12.86803	8.06773	6.02050	4.27372	12.56125	6.86154	0.74419
**Cd209e**	0.01599	0.00771	0.23373	0.01073	0.00884	0.00571	0.00725	0.02557	0.01230	0.01471	0.01268	0.00758	0.00965	0.01298	0.00652	0.00726
**Cd209g**	0.01372	0.00796	0.00973	0.82508	0.00919	0.00659	0.00748	0.00727	0.80701	0.00477	0.30874	0.00472	0.00908	0.00449	0.06688	0.00713
**Cd22**	0.17477	1.99198	1.67696	0.61664	2.13406	0.02197	0.01590	0.20451	0.89170	0.55528	0.24627	0.22880	0.05985	1.22299	0.17827	0.01292
**Cd24a**	4.39740	0.00000	1.66102	4.48767	5.51552	2.75770	1.22694	0.88683	2.03280	11.47587	0.76625	0.29091	0.51265	0.05501	0.67527	3.11581
**Cd274**	0.02153	0.01873	0.02428	0.07038	0.07878	0.01482	0.04885	0.03187	0.02481	0.03020	0.01069	0.01728	0.02053	0.23437	0.09432	0.01474
**Cd276**	0.01384	0.00794	0.01120	0.07880	0.00930	0.00670	0.00745	0.01270	0.01133	0.01371	0.01338	0.06839	0.01281	0.01173	0.00787	0.00741
**Cd28**	0.01104	0.00834	0.50149	0.01109	0.00884	0.00571	0.00812	0.48151	0.01175	0.00788	0.00908	0.00777	0.00361	0.01769	0.00000	0.00719
**Cd2ap**	16.21057	10.94668	10.13805	6.65283	10.79632	8.96949	3.84508	8.02670	5.16473	9.00627	8.56961	17.53231	5.68674	21.23022	12.20604	6.81426
**Cd2bp2**	5.53204	4.07342	8.68604	10.66996	3.44709	0.01070	4.00423	15.20853	14.62294	12.51385	11.10157	0.80989	11.23826	2.22009	6.87343	5.12248
**Cd300a**	0.27571	0.05534	0.24352	2.33691	0.15001	0.02640	0.18045	0.07117	4.41157	0.08575	3.49682	0.15506	0.29457	2.85305	0.81710	0.13179
**Cd300c2**	0.01185	0.00870	0.01162	0.01026	0.00976	0.00692	0.00817	0.06809	0.01207	0.01226	0.01302	0.00903	0.01651	0.01123	0.05205	0.00753
**Cd300ld**	0.90293	0.00792	0.40438	0.23607	0.01171	0.00467	0.00684	0.00994	0.00594	0.01298	0.01206	0.00831	0.00931	0.00815	0.19643	0.00627
**Cd300lf**	0.09339	0.00855	0.05889	0.38489	0.01230	0.01811	0.00747	0.01189	0.00967	0.03009	0.02369	0.00810	0.01119	0.10256	0.01476	0.00721
**Cd300lg**	0.06019	0.00797	0.00000	0.01046	0.01215	0.00594	0.00664	0.00000	0.01900	0.00000	0.24727	0.00000	0.07297	9.75375	0.37250	0.00695
**Cd320**	6.87667	3.86349	7.26316	15.79064	4.57781	1.45281	0.72856	16.07095	21.41955	26.98730	19.42403	16.48816	14.60354	7.02524	8.49251	2.85946
**Cd33**	0.03832	0.01826	0.01859	0.01097	0.03626	0.02276	0.01751	0.02531	0.05019	0.03522	0.07819	0.00726	0.06842	0.06866	0.04992	0.01455
**Cd36**	0.01370	0.00770	0.01216	0.83135	0.00923	0.00653	0.00734	0.01162	0.00000	0.15968	0.01249	0.00625	0.01287	0.00398	0.29388	0.00765
**Cd38**	2.18727	2.61780	0.24951	7.38286	0.10733	0.17496	0.33968	0.35499	11.88715	1.21631	0.64693	0.27173	0.44739	2.17266	3.81842	0.07579
**Cd3d**	0.02284	0.01815	0.06715	0.09231	0.00961	0.03337	0.01728	0.14153	0.16602	0.11819	0.17460	0.16887	0.01944	0.18625	0.15994	0.01476
**Cd3eap**	3.57534	0.34171	1.88331	3.73659	0.00733	0.01880	4.76960	5.58802	18.56431	5.06351	1.93323	0.14240	3.76787	4.73371	2.35800	5.41410
**Cd40**	0.01336	0.00792	0.02212	0.01106	0.00934	0.00625	0.00759	0.01367	0.01204	0.01302	0.01249	0.00809	0.01248	0.01864	0.04432	0.00740
**Cd44**	0.01323	0.00794	0.01081	0.00717	0.00892	0.00668	0.00736	0.01063	0.01173	1.08160	0.00999	0.07866	0.01085	0.01217	0.00608	0.00718
**Cd47**	0.01298	0.00758	0.00873	2.61852	0.00894	0.00597	0.00719	0.00865	3.85387	0.63832	0.00000	0.14456	0.01138	0.00000	0.62313	0.00660
**Cd52**	0.01171	0.00800	0.20761	0.00324	0.00874	0.00893	0.00687	0.00000	0.97302	0.47105	0.62762	0.07757	0.00670	0.56453	0.29156	0.00721
**Cd55**	0.01484	0.00772	0.34886	5.85454	0.02007	0.00839	0.00671	0.00000	0.73972	0.28304	0.62771	0.00000	0.00587	0.10960	2.13109	0.00596
**Cd55b**	0.01694	0.00777	0.03588	0.10666	0.00888	0.00795	0.00738	0.01240	0.00933	0.06827	0.01170	0.00696	0.01187	0.00785	0.21021	0.00727
**Cd59a**	2.17221	3.05254	6.03324	0.40510	3.89073	0.01022	3.39335	0.00000	0.62604	0.96554	0.79289	0.09916	0.63747	5.54373	0.33005	0.15704
**Cd59b**	0.09256	0.09524	0.56266	0.54571	0.10220	0.00567	0.20406	0.00484	1.97522	0.13586	0.30106	0.00000	0.03005	0.97971	0.00000	0.00711
**Cd6**	0.01405	0.00780	0.00848	0.23363	0.00908	0.00670	0.00735	0.00113	0.00786	0.00000	0.45189	0.02439	0.00782	0.00458	0.30131	0.00709
**Cd63**	26.83300	21.79151	45.89889	23.11357	13.37103	19.65402	21.71616	41.47992	19.87798	35.73023	41.03056	16.13047	42.50235	25.89546	24.17384	21.54912
**Cd68**	0.01525	0.00800	2.81922	0.24200	0.00585	0.00674	0.16318	0.37317	0.16112	1.40088	0.48908	0.02204	0.09025	0.06754	0.87793	0.01324
**Cd7**	0.01719	0.93509	0.00000	0.46088	0.00754	0.00751	0.19474	2.04519	0.43399	0.87108	1.28244	0.00389	0.90727	0.00284	0.33251	0.03546
**Cd74**	0.00779	0.00741	0.00645	0.11517	0.00839	0.00713	0.00707	0.13178	0.02145	5.72367	0.26073	0.24784	0.03347	0.00908	0.00000	0.00731
**Cd79a**	0.02199	0.00773	0.01256	0.51143	0.00860	0.00747	0.00702	0.00000	0.17149	0.00000	0.79904	0.06183	0.00665	0.00556	0.47549	0.00696
**Cd79b**	1.10619	0.00480	0.04740	0.00642	3.03007	0.00769	0.00635	0.00329	0.01083	0.39338	0.01209	0.00262	0.01030	0.01144	0.01007	0.00645
**Cd81**	0.01258	0.00784	0.01171	0.00542	0.00874	0.00662	0.00729	0.01004	0.03775	2.25858	0.00976	0.02304	0.01075	0.01267	0.00622	0.00737
**Cd82**	0.00251	0.00828	0.02478	0.00500	0.01939	0.01215	0.01826	2.14168	0.00917	2.56270	0.32053	0.00710	2.78558	0.01097	0.00431	0.01390
**Cd84**	30.19659	17.37856	32.42982	1.57827	21.64834	8.24899	23.10147	34.74057	1.81853	33.37169	3.81178	25.69132	19.61644	39.69761	21.65593	10.54927
**Cd8a**	0.80350	0.00753	0.06161	0.01448	0.00669	0.00541	0.09947	0.19521	0.14750	0.05482	0.70924	0.10165	1.73081	0.00841	0.14071	0.00540
**Cd8b1**	0.05244	0.01890	0.11920	0.08621	0.05821	0.03611	0.07139	0.09356	0.17640	0.10543	0.21760	0.03733	0.07840	0.07712	0.12349	0.01449
**Cd9**	23.95910	15.20882	36.61933	1.63012	16.04508	16.19507	19.67927	36.59286	2.21246	27.40437	18.00699	8.87426	31.70558	28.10040	15.65474	15.02015
**Cd93**	0.05869	0.00748	0.15391	0.14856	0.01655	0.01942	0.03741	0.01111	0.12983	0.06744	0.37426	0.03355	0.08561	0.31304	0.12688	0.02139
**Cd99l2**	0.23130	0.00847	0.41129	0.40310	0.00646	0.00681	0.00627	0.17463	0.40532	1.90155	0.36056	0.09958	2.50731	0.07424	0.05963	0.99582
**Cda**	0.01366	0.00807	0.01242	1.45220	0.00936	0.00640	0.00731	0.01182	0.00000	0.24435	0.01388	0.00721	0.01227	0.00943	0.00282	0.00723
**Cdadc1**	0.03924	3.47534	2.53390	0.43472	0.00856	0.00697	0.00457	2.48361	2.50375	3.17810	2.33674	5.99951	0.07179	0.15801	1.60726	0.03527
**Cdan1**	2.62611	1.78672	8.82236	8.99539	0.00000	1.63014	2.21564	12.39907	17.34110	12.73245	6.02720	9.39747	5.60701	3.59570	9.17385	0.01541
**Cdc123**	9.08224	8.08368	5.94525	4.33103	3.97907	6.09859	6.75558	1.89275	7.14247	0.85887	1.94473	0.18733	2.96822	1.40329	1.78444	5.92758
**Cdc14a**	0.01154	0.00840	0.00737	0.51938	0.00928	0.00670	0.00812	0.06520	0.13353	0.26655	0.17426	0.00502	0.00837	0.00826	1.32915	0.00716
**Cdc14b**	5.06786	6.82935	5.81604	18.89721	0.03917	2.11262	9.32946	7.11061	19.01121	13.62070	15.88652	5.22375	4.07129	22.83510	18.66019	0.31906
**Cdc16**	5.69744	8.54817	8.94692	4.37105	0.27074	4.61419	4.69105	8.56339	2.75939	8.84997	9.69528	6.92202	6.19555	7.45359	8.74909	2.99639
**Cdc20**	12.19751	3.79239	11.01409	1.33932	16.46502	7.98639	3.53883	25.78361	11.87468	32.85257	11.27138	10.01734	24.26486	14.87877	23.25335	8.60039
**Cdc23**	3.42380	5.58563	2.48753	0.15115	3.93675	0.10947	5.24507	6.19372	0.99707	3.74959	0.50434	0.43621	1.21070	0.47109	5.22644	1.71025
**Cdc25a**	2.67760	5.70212	12.10099	2.09375	6.75196	1.87139	5.43190	11.43399	13.55341	12.53369	13.35602	2.64443	5.98535	13.65569	11.61838	1.75966

**Cdc25b**	0.00000	0.00594	2.88623	2.20791	0.01163	0.01822	0.00406	4.38384	2.11698	12.51699	6.77894	1.11323	0.45318	0.65851	9.62997	0.00443
**Cdc25c**	9.83107	2.41985	4.02493	12.06590	0.00000	1.93584	2.30303	5.04826	17.46913	10.90336	3.12106	10.66777	3.80590	0.10522	5.28203	2.70420
**Cdc26**	6.75506	0.13076	5.11169	0.65686	3.36807	4.07451	1.80759	2.20694	0.58070	2.53463	0.35187	0.08827	0.48573	1.57370	0.24271	2.15914
**Cdc27**	1.74037	7.84550	5.83724	12.58024	5.56968	4.90072	6.86087	4.78038	18.10272	7.97115	11.47816	4.60205	3.46820	32.94487	17.56207	0.04157
**Cdc34**	18.42564	4.03184	13.39574	17.55529	14.02018	14.70179	12.28904	27.17311	17.76418	25.12742	12.86042	1.52910	22.95095	0.00000	0.00000	13.82416
**Cdc37**	21.40245	9.49730	15.14932	1.30575	13.00163	8.09290	11.44708	29.57224	2.75187	22.93435	8.78957	1.22023	23.36280	0.54235	1.74829	12.73086
**Cdc37l1**	2.35480	2.60105	0.13841	6.89137	0.19327	0.00788	1.87880	0.00000	5.02716	0.64517	1.38100	3.59984	0.83467	4.06095	8.14379	0.33803
**Cdc40**	5.11605	6.39232	1.60353	7.60365	0.03363	0.00000	1.71411	5.37836	9.53244	10.05880	0.95758	7.24966	1.89547	13.45970	12.75265	1.16855
**Cdc42**	24.42664	13.85167	22.81235	15.38400	20.33387	15.47137	15.69320	21.31092	20.20415	25.85483	19.00799	11.57754	27.23420	25.10149	16.71274	15.85278
**Cdc42bpa**	0.02972	0.84650	0.00284	0.46378	0.01274	0.00678	1.09211	0.03111	0.44942	1.18048	0.00752	0.06374	0.00575	0.00554	0.00000	2.01942
**Cdc42bpb**	6.17422	0.01880	0.00000	1.85926	2.03865	2.45284	1.06309	9.20993	1.35987	7.89957	0.23982	0.22491	5.42726	0.00000	0.26445	2.07473
**Cdc42bpg**	0.12884	0.00668	0.00000	2.02467	0.01728	0.00667	0.09416	0.02088	2.88089	0.00000	4.55712	0.01160	0.15820	1.19108	0.71985	0.01282
**Cdc42ep1**	1.40971	0.01477	1.87030	0.28659	0.02019	4.29826	1.48633	4.37232	0.00902	0.56411	1.86825	0.05257	1.49264	0.01687	0.39433	0.02504
**Cdc42ep2**	0.00000	0.00672	2.31866	2.71211	0.00472	3.35247	0.00456	0.96669	5.54244	2.90933	2.42129	0.30192	1.96824	0.29508	1.33436	0.00330
**Cdc42ep3**	0.29261	4.35270	0.97341	0.46526	0.98330	0.09553	0.07744	9.76862	0.00315	0.24074	0.00000	0.03761	1.51713	0.00000	0.00000	2.21718
**Cdc42ep4**	2.72848	0.01318	2.92913	3.73154	0.89057	8.66513	0.00175	8.67352	1.51333	9.61982	5.23986	0.30825	0.27400	0.69153	2.16638	0.00000
**Cdc42ep5**	2.40485	0.00471	0.00000	0.07900	0.04423	5.90929	0.00956	1.75110	0.27762	4.46663	1.09129	0.03823	10.56435	0.01018	0.00000	0.03452
**Cdc42se1**	3.15212	1.01643	2.07966	7.30287	2.55921	4.06950	1.65277	5.83802	1.64199	4.92742	2.75326	7.05609	4.57413	0.33337	3.78842	2.45186
**Cdc42se2**	5.19349	2.93171	3.64498	17.60386	6.61358	0.46614	3.72030	3.09687	11.61123	6.58186	1.06138	7.42662	1.64153	4.76842	28.90717	1.88791
**Cdc45**	10.49352	7.53145	16.95164	10.93687	5.37825	5.84854	5.50520	12.85320	20.16674	20.38458	9.88426	11.15151	7.46138	2.19572	18.92865	1.98309
**Cdc5l**	20.20943	21.43187	18.78039	12.72399	15.81482	10.03331	14.26039	28.29529	20.65380	20.83467	18.64697	17.59139	17.61790	28.15331	13.81771	7.55688
**Cdc6**	6.04092	3.54030	15.30984	3.59861	4.81895	6.58479	3.37722	15.87012	14.45425	18.85792	10.13596	3.53622	5.64189	21.68725	17.73259	5.13399
**Cdc7**	7.55861	0.00601	0.54159	2.77996	0.77844	5.09860	5.69743	2.94864	2.59027	2.94194	3.16611	4.14058	3.83145	8.18136	8.31449	2.93692
**Cdc73**	9.03913	8.61762	4.32686	4.97622	5.27314	3.97290	2.06055	3.95251	1.66922	6.53434	3.22607	7.61661	2.11306	18.05888	10.93305	1.80892
**Cdca2**	4.11434	1.68881	4.73009	2.08194	0.16684	0.01035	0.82605	7.26564	9.48687	2.35059	1.49725	0.16769	3.92697	0.79853	2.92359	2.31396
**Cdca3**	21.22799	9.31859	18.47051	10.54266	12.65300	14.29718	9.65078	24.85248	10.88357	18.25003	13.05944	9.65485	16.81711	5.02249	7.17428	13.83009
**Cdca4**	2.13892	1.54801	5.34924	16.20839	5.36113	4.09803	1.99632	7.68894	10.05166	11.46272	2.41895	1.61824	4.34518	2.71031	7.38774	1.16875
**Cdca5**	9.65414	4.61593	9.13174	23.47157	10.16554	2.20071	7.81173	16.19266	28.81732	17.55480	8.52313	3.12563	10.92802	13.17060	14.05404	3.25991
**Cdca7**	4.37106	5.74242	3.79219	0.45213	0.56965	5.07008	4.65415	5.95229	3.21706	8.50001	6.84778	7.78968	2.89807	5.13894	5.98483	1.77865
**Cdca7l**	0.01328	0.00790	0.01183	0.42124	0.00905	0.00646	0.00723	0.01033	0.21504	0.74369	0.01020	0.00495	0.01848	0.00542	0.27467	0.00764
**Cdca8**	19.11486	6.37036	9.51730	18.61163	9.79775	12.59377	3.27555	20.82792	28.67508	26.78589	13.84032	11.55885	16.19395	0.73301	15.37790	9.34754
**Cdcp1**	0.01052	0.00836	0.10787	0.58337	0.00953	0.00576	0.00757	0.01322	0.00018	0.00450	0.00490	0.00286	0.01158	1.42093	0.44931	0.00731
**Cdh1**	6.71611	4.91843	15.77737	3.54378	0.47009	12.05094	9.52373	17.62968	7.45072	7.51263	14.14476	6.75649	15.46704	18.42371	3.41687	12.17780
**Cdh11**	0.59963	0.17380	1.34458	0.20826	0.04960	0.33436	0.22287	0.71846	0.18104	0.16467	0.16096	0.09573	0.42871	0.32773	0.21956	0.22199
**Cdh15**	0.01787	0.00808	0.01132	0.17921	0.00900	0.00669	0.00748	0.01357	0.20279	0.01331	0.01085	0.03911	0.01284	0.01192	0.00478	0.00736
**Cdh16**	0.09721	0.00856	0.03287	0.33841	0.08020	0.00626	0.02933	0.02023	0.26364	0.02443	0.10182	0.02946	0.05586	0.08148	0.02465	0.05075
**Cdh17**	0.01383	0.00836	0.00932	0.19732	0.00912	0.00681	0.00758	0.00670	0.00578	0.01162	0.41184	0.00674	0.00798	0.00848	0.00395	0.00712
**Cdh2**	0.62376	2.03923	0.00000	0.03663	0.01327	0.00630	0.00592	0.00707	0.04190	0.00022	0.01217	0.03549	0.01488	0.00653	0.16073	0.96047
**Cdh23**	0.01197	0.00817	0.01390	0.01793	0.01018	0.00720	0.00765	0.02769	0.01249	0.01317	0.01516	0.00794	0.24758	0.01224	0.01027	0.00764
**Cdh26**	0.01368	0.00809	0.01106	0.23950	0.00926	0.00662	0.00746	0.01269	1.03748	0.00859	0.00664	0.05977	0.01307	0.01246	0.00086	0.00733
**Cdh3**	0.47267	0.00770	0.00665	0.21882	0.00750	0.00753	0.00736	0.01754	0.00548	0.01209	0.01388	0.00772	0.12404	0.01153	0.00723	0.00706
**Cdh4**	0.01022	0.00786	0.01073	2.10276	0.01000	0.00681	0.00739	0.02924	0.00000	0.94588	0.04619	0.04035	0.42810	0.02265	2.06714	0.00724
**Cdip1**	4.04917	1.38958	3.98017	13.56033	4.41925	0.02785	2.35173	6.20531	14.10018	4.73520	4.87967	0.42849	3.46745	4.14117	4.61340	5.09740
**Cdipt**	13.13204	10.87937	21.37330	7.92030	11.90418	12.66094	12.52684	28.28540	15.88632	24.99960	20.77998	3.89781	27.44595	2.33755	11.90993	12.29953
**Cdk1**	22.12456	10.29319	14.24963	20.55986	8.16873	5.59275	9.63758	27.42857	28.01535	18.77231	24.71903	15.18093	13.65881	15.69632	21.16749	6.07757
**Cdk10**	7.56156	2.15800	0.71411	1.18035	3.17237	3.08218	3.04127	6.33375	0.95464	3.64504	1.24032	0.08191	8.34918	0.00466	0.32192	4.46712
**Cdk11b**	10.58673	7.35650	15.34091	5.21805	6.29825	10.69205	8.51247	15.46337	7.98784	15.47227	6.11979	2.86831	14.45425	0.01644	1.79739	7.45760
**Cdk12**	5.21645	0.41595	0.19480	12.71828	0.30502	0.00000	3.06040	1.07522	12.88470	1.66667	0.90770	1.11111	0.41618	11.56374	9.54763	0.08443
**Cdk13**	0.21580	0.73523	1.60990	3.05762	1.66554	1.40258	0.53756	0.79140	6.57629	3.41174	0.16062	3.23968	0.23982	3.26801	2.65201	1.21697
**Cdk14**	0.62280	1.81995	2.18752	0.26279	0.00314	0.65788	0.00589	0.17570	0.14125	0.00000	0.00675	0.00475	0.01464	0.04374	0.39542	0.06204
**Cdk16**	9.29301	3.06949	13.69748	8.76431	7.05319	6.41174	4.50873	15.08884	5.65502	14.87996	4.92020	2.00167	6.78247	1.66583	9.68609	4.27875
**Cdk17**	2.47095	0.02479	0.37328	1.01934	0.02714	0.01255	2.92072	0.75796	0.49251	3.22119	0.94144	0.07369	0.30321	0.57686	0.86320	0.87683
**Cdk18**	0.22767	4.98642	3.55901	0.02390	5.14278	0.01328	3.73248	4.82181	0.03976	0.54563	2.73289	3.14074	4.57259	0.00000	0.06615	0.00915
**Cdk19**	1.93459	0.63399	0.30283	1.35147	1.31396	0.00880	2.04257	2.51678	8.02739	2.99475	2.04135	1.84162	1.17307	6.38132	2.35208	0.43797
**Cdk2**	6.28027	1.98323	3.18362	0.76913	1.63587	2.39547	2.72987	3.09407	2.50441	9.08768	0.07749	0.32684	2.40667	13.39686	8.62234	5.59423
**Cdk20**	2.05208	0.02589	0.14078	2.46316	0.63303	0.01600	2.39299	0.00000	4.85098	0.94829	0.20923	0.03641	0.01457	0.00000	0.81616	0.00486
**Cdk2ap1**	10.94663	2.63838	12.24267	21.90024	5.15287	6.80980	9.79539	37.80992	24.87624	42.61369	36.75864	12.43130	21.92262	29.87227	33.82279	1.53358
**Cdk2ap2**	4.74134	0.00000	3.70204	17.76429	2.52068	2.49888	0.81668	2.30409	16.45262	11.29008	3.83869	0.81913	7.98467	0.24689	0.00423	0.02108
**Cdk3.ps**	0.01029	0.00807	0.84637	0.01084	0.00813	0.00577	0.00751	0.06720	0.01205	0.05564	0.01069	0.02082	0.00627	0.01507	0.00035	0.01435
**Cdk4**	26.36711	15.29430	22.24255	4.05271	19.24078	13.22720	13.19966	23.47251	4.03710	25.41578	18.44351	4.56287	26.37965	1.19223	8.93492	17.64301
**Cdk5**	7.62300	0.10101	3.54278	0.59353	4.33912	7.28021	1.64424	4.31193	0.00000	1.08871	0.00000	0.00101	4.87983	0.00687	0.00000	7.05147
**Cdk5r1**	0.00740	0.00764	0.26696	0.73089	0.00879	0.00679	0.00714	0.42777	0.36281	0.92957	0.00000	0.07588	0.28039	0.00647	0.72915	0.00709

**Cdk5r2**	0.01082	0.00833	0.01061	0.35436	0.00957	0.00676	0.00767	0.10600	0.02195	0.01002	0.01258	0.00831	0.13538	0.01214	0.00411	0.00728
**Cdk5rap1**	3.51840	2.70697	2.96368	2.24429	3.42033	0.06290	0.00144	1.69353	6.74412	2.13986	4.94738	0.45182	0.11228	1.21734	2.63662	0.04456
**Cdk5rap2**	2.70352	0.04036	0.09212	0.53828	0.03092	1.98985	0.01312	3.30478	0.23225	1.14856	0.43865	0.09426	3.60912	1.20900	1.01600	0.19573
**Cdk5rap3**	8.91078	3.79505	12.39804	4.11213	4.50049	4.30473	6.69261	13.54214	7.45672	1.66850	5.37854	0.18297	9.38021	0.03490	1.57172	4.26320
**Cdk6**	3.66579	0.12227	1.70960	0.41483	0.01461	0.01400	0.04502	0.92985	0.52917	0.01498	0.35765	0.05743	0.75755	0.38518	0.07038	0.02460
**Cdk7**	9.70910	7.29611	8.60338	16.24304	7.09577	3.67314	10.03888	14.13507	21.20216	14.69314	7.68374	10.62922	3.24757	7.90981	18.39484	6.96219
**Cdk8**	2.32398	6.04018	2.54569	12.16926	4.73653	2.90065	4.72383	7.40519	10.81884	19.23537	7.16069	12.82017	5.44348	0.80583	12.19353	2.21081
**Cdk9**	10.20823	9.10144	11.79238	14.98019	7.29319	2.11423	6.86523	11.87374	16.81828	15.79057	5.38718	1.04384	9.30632	2.50963	11.53705	12.07274
**Cdkal1**	0.71477	0.05050	2.13030	2.32968	0.37559	0.12844	1.11385	0.15276	4.71290	0.46265	2.99514	0.13828	0.04837	5.29717	0.00000	1.31181
**Cdkl2**	2.60334	2.62807	0.67405	5.20596	0.00497	0.00258	0.00262	0.41207	2.32025	2.41786	1.97033	5.05136	0.00000	10.66227	7.27161	0.80699
**Cdkl3**	0.01331	0.00783	0.01009	0.02472	0.00921	0.00667	0.00749	0.01157	0.71524	0.24845	0.00388	0.00482	0.01310	0.06611	1.06603	0.00737
**Cdkl4**	0.00539	0.00771	0.02945	2.01644	0.00982	0.00554	0.00714	0.20297	1.51260	0.61350	0.11424	0.08515	0.00588	1.08551	1.20430	0.00665
**Cdkl5**	0.01382	0.00816	0.01135	1.06611	0.00930	0.00660	0.00742	0.01283	0.10037	0.01326	0.01321	0.06077	0.01280	0.01093	0.00000	0.00724
**Cdkn1a**	2.63061	0.03651	3.89089	2.43388	1.29297	0.01353	2.84057	8.76148	2.94745	8.41727	10.92903	0.55251	0.00000	3.21273	1.81470	0.00798
**Cdkn1b**	0.07790	0.00832	0.02435	0.07977	0.01066	0.00802	0.00758	0.01573	0.00983	0.01699	0.01394	0.00789	0.02812	0.01173	0.00886	0.17053
**Cdkn1c**	0.00597	0.00767	0.00973	8.08089	0.00885	0.00547	0.00655	0.00901	6.92668	3.11901	0.00000	0.09467	1.38289	0.03122	0.22181	0.01987
**Cdkn2aip**	0.00000	1.09139	1.90651	2.40553	0.18111	0.03405	6.01194	1.70946	4.81261	6.04233	1.68880	0.13709	0.00000	4.68351	4.15115	2.20989
**Cdkn2aipnl**	5.43873	2.63471	5.70044	4.08270	3.72358	4.26213	3.06509	6.77464	1.91872	2.76255	5.84804	0.18090	8.69093	0.00000	2.41539	2.75309
**Cdkn2b**	0.02292	0.00835	0.01103	0.00469	0.01134	0.00694	0.00754	0.01252	0.28675	0.33185	0.00933	0.01965	0.01266	0.01112	0.01361	0.00747
**Cdkn2c**	0.16794	0.00453	0.05692	1.11319	0.58566	0.00512	2.28286	0.65254	0.12491	0.06034	0.00000	4.87865	0.29569	0.02883	0.00000	0.00622
**Cdkn2d**	2.62799	0.00000	0.00000	6.80020	2.68195	1.58920	3.20781	1.06766	9.88632	2.71498	0.29971	0.64092	1.39553	0.00000	1.82408	4.06712
**Cdkn3**	1.33880	0.33667	1.47124	1.85166	0.00292	2.92585	0.07621	0.00000	5.46515	1.53455	0.23038	0.09917	0.00000	1.07603	0.13760	0.62807
**Cdnf**	0.04945	0.12050	0.04688	0.38152	0.03601	0.03151	0.03688	0.03629	0.12068	0.03058	0.08657	0.08314	0.03824	0.24685	0.15683	0.02908
**Cdo1**	0.01247	0.00809	0.00299	5.47222	0.00788	0.17623	0.00759	0.03332	1.38088	0.50460	0.00765	0.46105	0.00707	0.01283	1.13572	1.03717
**Cdon**	1.23955	0.00480	0.00000	0.02813	0.00565	1.18467	2.42852	0.00000	0.01170	0.39538	0.18949	0.01562	0.09111	0.01313	0.03605	0.01285
**Cdpf1**	4.54731	5.63433	6.71900	0.95904	4.83514	6.51799	6.86464	6.39516	0.19913	3.32024	1.52588	0.50595	9.51311	0.22052	0.35853	3.08629
**Cdr2**	0.35642	0.00712	1.51489	18.40916	0.00479	0.24643	0.00589	0.00000	15.72274	4.11765	0.92682	0.91220	0.39190	1.26132	5.69906	0.00934
**Cdr2l**	1.28070	0.37062	0.53683	0.00622	2.65578	0.00404	1.50483	0.02381	2.18249	0.00008	0.00000	0.02430	0.00754	0.03319	0.00443	0.00544
**Cds1**	1.12934	0.14419	5.83004	1.04753	0.02704	0.00419	4.91231	6.04758	0.75001	2.31822	5.68027	4.05001	0.55727	0.69841	0.89184	0.02436
**Cds2**	13.71315	5.95813	13.10353	1.91127	4.76483	8.16790	12.75332	14.88548	1.63070	17.60264	17.23174	8.84128	18.16707	1.81599	12.85358	3.46529
**Cdsn**	0.01398	0.00821	0.05079	2.37579	0.00895	0.00558	0.00776	0.43783	1.15842	0.98573	0.01665	0.06020	0.00881	0.00120	0.56242	0.00687
**Cdt1**	12.47385	3.84033	4.14503	14.11369	7.98640	3.59798	3.43098	21.76382	14.77890	32.09927	20.20895	3.75662	16.66556	0.87665	2.30682	11.46607
**Cdv3**	28.65357	16.31331	29.32372	25.42277	11.37320	14.63532	17.39382	19.78602	30.66797	28.55036	22.20995	18.71583	23.81923	28.02720	24.63893	11.18776
**Cdx1**	0.00914	0.00784	0.01688	0.01054	0.00781	0.18505	0.00739	0.01316	0.01241	0.00632	0.01246	0.00802	0.00701	0.01314	0.01020	1.22298
**Cdx2**	0.00000	0.00663	1.59737	0.00000	0.00594	4.37155	0.27554	3.53976	0.00815	0.00000	2.35485	0.00238	6.21914	0.01263	0.00000	1.70210
**Cdyl**	2.84800	0.00508	1.49905	4.04427	0.00934	0.15790	0.01611	0.31141	5.09071	1.60403	1.19250	0.05868	0.78258	0.72712	4.95063	0.74307
**Cdyl2**	5.91765	2.94929	7.90660	0.24475	5.27620	0.78318	5.05602	4.11512	0.37756	3.38845	1.13962	4.29050	2.90022	3.80662	0.00000	0.00000
**Ceacam1**	0.85356	0.00583	1.42724	0.61102	0.00401	0.00473	1.54085	0.00601	0.53458	0.00778	0.00750	0.00466	0.02638	0.07149	0.02321	0.00563
**Ceacam10**	15.00143	0.00000	10.91189	0.33270	8.02521	3.17444	5.80146	0.00000	0.00000	2.63109	3.63291	0.00000	4.08705	0.91621	0.00000	1.86178
**Ceacam18**	0.11656	0.00795	0.00835	0.23369	0.00768	0.00691	0.00735	0.01426	0.00761	0.06237	0.01395	0.00783	0.01285	0.00896	0.03471	0.01496
**Ceacam19**	0.22301	0.07040	0.10868	0.00656	0.01773	0.18100	0.06689	0.11302	0.01183	0.13843	0.11971	0.01556	0.16269	0.01687	0.07091	0.04992
**Ceacam2**	0.32977	0.01818	0.25075	0.42988	0.00749	0.00525	0.44434	0.00918	0.30719	0.02801	0.01043	0.00724	0.00965	0.00989	0.00984	0.00667
**Ceacam20**	0.30846	0.00759	0.02214	2.30913	0.00727	0.00727	0.00579	0.00000	0.08143	0.00000	0.95725	0.02228	1.88167	0.04518	0.04924	0.00555
**Ceacam5**	0.01363	0.00811	0.01193	0.00432	0.00932	0.00664	0.00755	0.01383	0.81716	0.01134	0.00697	0.00688	0.01295	0.01399	0.00648	0.00737
**Cebpa**	2.57312	0.00423	0.00000	0.70245	0.01037	2.02398	0.01394	0.73753	0.34560	7.19688	0.98157	0.14563	0.79206	0.76826	1.83835	3.54849
**Cebpb**	0.07972	0.00837	0.00940	0.00998	0.01607	0.00674	0.00740	0.01216	0.01255	0.09817	0.01288	0.00721	0.01247	0.00894	0.02813	0.00757
**Cebpd**	0.01382	0.00806	0.00816	0.60337	0.00898	0.00700	0.00745	0.00225	0.93125	0.15602	0.41305	0.04052	0.00879	0.00632	0.00000	0.00711
**Cebpg**	2.82849	0.08056	3.68830	10.26928	0.04715	0.30010	1.30541	2.91535	1.35264	7.35463	6.17976	6.26473	0.46657	11.48667	6.86406	0.01418
**Cebpz**	6.85791	10.36603	5.31828	4.74022	7.81300	4.59060	3.27140	8.98005	6.54767	13.55372	5.73132	10.72455	5.73768	20.28626	19.81234	3.11578
**Cebpzos**	1.97291	0.13526	1.67780	0.09220	1.51191	0.56172	0.85454	0.55733	0.00585	1.66626	4.36609	0.02542	2.29834	0.28844	1.07116	1.54560
**Cecr2**	9.90816	6.96621	2.01096	20.49587	7.61255	5.34571	5.39458	9.72957	20.86104	20.05044	13.23906	15.10207	1.90950	28.16105	20.64276	5.66477
**Cecr5**	0.01210	0.00826	0.01211	0.23928	0.00895	0.00661	0.00769	0.01870	0.00523	0.01275	0.01215	0.00852	0.01119	0.01187	0.00695	0.17214
**Cela1**	1.37639	0.00611	1.03443	1.47809	0.01441	0.00465	0.00606	0.81391	0.72936	0.75617	0.95717	0.03404	0.06034	0.04704	0.00000	0.00584
**Cela2a**	3.36052	0.00255	0.96021	0.01034	0.33425	0.00285	1.44437	0.40061	0.01153	0.00000	0.01014	0.00248	0.63655	0.01793	0.00000	0.00302
**Cela3b**	0.01305	0.00761	0.01198	0.00770	0.00903	0.00647	0.00724	0.01034	0.01161	0.91634	0.01339	0.00587	0.05171	0.03213	0.01181	0.00709
**Celf1**	19.75939	8.13636	10.98498	7.21022	3.35841	10.05171	7.49811	12.75189	9.51535	13.67320	8.85212	8.84198	14.33169	9.29663	17.00047	5.27233
**Celf2**	0.00554	3.57313	2.35665	7.85634	0.02826	0.07166	0.12280	0.02196	0.90746	2.12065	0.88861	0.01084	0.99616	0.00000	1.16913	0.00320
**Celf3**	0.01816	0.00746	0.01489	0.00636	0.00868	0.00740	0.00708	0.41501	0.01112	0.00000	0.25397	0.00561	0.00475	0.01250	0.00131	0.00722
**Celf5**	0.01155	0.00795	0.02976	0.45485	0.02735	0.00799	0.01777	0.01312	0.01372	0.03586	0.01261	0.00840	0.01895	0.06476	0.00908	0.01452
**Celsr1**	0.05353	0.45089	1.49466	0.26038	0.00535	0.00684	0.01589	0.56765	0.41843	0.44102	0.04139	0.06710	1.03750	0.01163	0.00097	0.00605
**Celsr3**	0.00952	0.00882	0.00834	1.27350	0.00963	0.00682	0.00815	0.44935	0.00000	0.00817	0.01065	0.02108	0.00594	0.01033	0.02565	0.00716
**Cend1**	0.01268	0.00844	0.70232	0.00445	0.00809	0.00568	0.00763	0.00907	0.21440	0.00000	0.22725	0.00749	0.00712	0.00777	0.06485	0.00699

**Cenpa**	18.99564	0.24565	11.16035	13.67440	17.34506	8.25255	5.50816	17.97429	20.89101	19.62478	18.63857	2.79350	17.51848	3.28930	2.58038	8.44692
**Cenpb**	0.19245	0.01698	0.00000	0.29784	3.29468	1.13732	0.02609	0.02430	0.02005	1.45398	0.00870	0.02845	1.45433	0.00517	0.16649	1.06131
**Cenpc1**	0.54674	0.00682	1.12316	0.38991	0.00654	1.04954	0.04642	1.05837	0.95806	1.14877	0.58382	0.06908	0.55183	0.62283	3.20429	0.03417
**Cenpe**	5.52524	5.37727	4.97214	11.62425	4.31818	8.49377	0.55413	12.67347	14.90696	21.39110	10.72374	19.65464	4.13847	29.02793	20.36912	1.52379
**Cenpf**	4.05702	1.26938	6.21787	6.82131	0.29747	3.15454	1.36206	2.20634	13.69367	0.00000	5.21019	1.18941	0.00004	2.06293	3.26513	2.19446
**Cenph**	5.95386	4.65386	4.69716	4.62747	5.79087	3.05199	2.37858	7.78247	6.18020	8.61435	4.32361	8.74308	6.95020	0.16153	4.64165	2.53446
**Cenpi**	3.41041	0.60442	2.18759	3.56524	1.66283	0.00246	0.00203	3.37939	0.63946	5.41942	1.76529	4.49054	1.17206	12.03442	7.29665	0.00780
**Cenpj**	0.00000	0.13878	0.02690	5.87422	4.92741	1.67885	2.79285	0.62950	1.60116	0.00001	0.48206	6.98893	0.05315	1.01132	3.54486	0.00504
**Cenpk**	1.94867	2.78332	0.75422	1.76752	0.01158	1.84363	4.87569	0.99109	2.98927	2.80017	4.79048	14.18467	0.00000	4.75150	0.00000	0.01541
**Cenpl**	0.01825	0.09276	5.35489	2.79923	4.24226	0.01442	0.16644	3.09429	10.75262	4.06517	1.87979	1.57772	3.57556	0.01546	6.34690	0.01008
**Cenpm**	5.32765	1.52847	8.22854	0.30682	2.34320	0.03659	5.01269	7.41773	2.44628	3.51753	3.40382	0.33383	5.64505	0.00000	0.00000	0.06877
**Cenpn**	7.25012	3.38010	5.79422	18.64288	3.10386	5.28943	8.46452	6.49431	6.92621	8.31656	4.57792	1.59008	6.54392	5.95461	11.41857	4.45704
**Cenpo**	1.00345	8.32494	4.52531	2.29102	0.00741	0.01267	2.03370	2.16755	0.13023	0.83576	0.78634	0.06524	0.75089	0.00000	1.83246	0.00000
**Cenpp**	3.10523	1.90960	0.00000	3.20058	0.66287	3.61861	0.45386	3.26087	4.27381	3.73075	1.15138	0.47406	0.83221	1.40609	1.39936	2.13056
**Cenpq**	0.00000	2.86427	0.44940	3.81849	2.68897	0.00941	0.00407	2.76368	3.16353	4.91718	0.93734	8.59803	0.96424	4.72889	4.85884	1.50852
**Cenpt**	10.71518	3.08119	1.60363	9.05300	0.08005	5.82224	7.40029	10.06936	11.58687	7.63823	7.81639	3.99279	4.91334	0.23523	4.97271	6.80452
**Cenpu**	3.25731	1.92938	3.12208	0.00000	0.00000	0.76001	0.00354	2.22560	0.45397	2.39827	0.09170	2.30207	0.56090	0.23792	0.52504	1.75646
**Cenpv**	1.17426	0.27768	0.17166	4.57587	0.00516	0.00740	0.00460	0.00175	2.31329	0.22162	0.66621	0.00557	1.93525	0.00839	0.00000	0.18272
**Cenpw**	4.37367	0.00883	0.90924	7.04013	6.35649	1.03791	1.51954	1.89119	13.33632	4.57072	3.90212	0.49556	0.35821	0.10952	0.14188	1.59355
**Cep104**	0.94096	0.51575	2.46317	2.02787	0.03540	0.01113	0.70809	2.96448	1.31129	3.66782	0.89536	0.32074	0.57543	0.19693	1.57858	2.49638
**Cep120**	3.04255	4.44261	3.81406	11.09655	0.05255	2.07875	0.12567	0.58947	2.12210	7.41381	2.59620	0.57782	1.97844	5.47497	12.46668	3.08188
**Cep126**	0.92102	0.63455	0.26809	0.15474	0.89993	0.00975	0.00586	0.00000	0.00469	0.11073	0.16749	3.49378	0.05602	0.03220	0.03843	0.00601
**Cep128**	0.08632	0.00843	0.00276	0.38508	0.00894	0.01652	0.00785	0.97515	1.41803	0.13103	0.00000	0.00474	0.06368	0.00726	0.04509	0.01504
**Cep131**	3.12699	0.00423	0.03850	0.01215	0.00035	0.01506	0.00410	2.00148	0.03108	0.93437	0.17132	0.01843	1.90769	0.02244	0.00329	3.88675
**Cep135**	1.24001	0.14629	0.00000	3.30732	0.05097	0.00516	0.66709	0.00000	0.78818	0.30433	0.59321	0.08252	0.06149	0.00000	3.35961	0.00560
**Cep152**	1.77734	0.00402	1.09610	0.41689	0.02613	0.00693	2.68654	0.87816	3.08615	1.75995	0.00000	0.38626	1.06840	0.04160	0.04985	2.34768
**Cep162**	0.00000	0.00678	0.41324	1.31094	0.46924	0.00915	2.03375	0.58476	1.14114	1.05360	0.50026	0.12209	1.53509	0.00786	1.48359	0.02101
**Cep164**	8.57235	0.03207	3.87317	1.20624	7.93481	3.89699	6.29813	0.12484	0.00000	1.82656	0.00259	0.21338	3.77681	0.01011	1.76947	0.00000
**Cep170**	2.23605	2.71847	1.00428	2.50905	0.07804	0.02684	1.81338	0.74957	5.97628	0.00000	0.64219	5.28379	0.00000	7.49380	3.51964	0.00190
**Cep170b**	0.00000	0.00699	0.87156	5.49514	0.00644	0.15247	0.19354	1.25503	5.89931	0.99540	0.55847	0.08252	2.64307	0.00000	0.14878	1.85543
**Cep19**	4.08583	3.21638	0.67015	1.85972	0.57811	2.13367	0.19924	0.94930	1.14593	7.43579	0.87688	0.20869	3.24678	3.50126	0.34422	0.00000
**Cep192**	2.64874	2.06672	2.01595	7.10693	3.74378	3.26537	3.90088	3.47353	12.23024	8.05048	0.83155	8.53317	0.00000	3.51096	11.72071	1.38493
**Cep250**	3.02214	1.11519	1.32209	2.08546	0.02388	0.40220	2.23499	2.48353	3.23770	3.49146	0.75336	2.13344	0.00000	0.45503	0.14062	2.33982
**Cep290**	0.62718	0.28521	1.76587	0.13614	0.01194	0.00656	0.00568	0.95112	0.00367	0.00000	0.65222	0.00240	1.29983	2.86548	3.19363	0.28094
**Cep295**	2.88172	5.08158	1.01542	4.79941	0.02997	0.00410	3.08810	3.71795	1.70186	2.42333	1.48478	0.19827	1.42593	3.01920	2.50828	1.88515
**Cep350**	0.12158	0.00759	1.84215	1.64743	0.01252	0.94656	0.23891	0.01516	0.00000	0.00000	0.09472	0.51244	0.50607	2.75683	0.57119	0.65639
**Cep41**	1.68799	1.79357	0.00000	1.34914	0.00900	0.11810	3.18877	0.03584	0.70764	1.08740	0.00000	0.03480	0.03567	2.06039	0.00000	0.01223
**Cep44**	0.07942	0.18746	0.13590	0.00281	0.00759	0.00601	0.00683	0.77245	0.03854	0.03271	1.00014	0.00001	0.07651	0.00849	0.80329	0.06553
**Cep55**	3.83882	0.57306	0.32156	0.35240	0.00000	0.02295	0.71064	0.16116	0.22213	0.05712	0.17167	0.31509	0.72465	2.97862	2.13642	0.19998
**Cep57**	5.07065	3.74710	3.48011	3.54754	3.88796	0.80858	7.82595	4.13164	5.10778	0.97885	0.80961	0.49854	4.06901	1.61408	3.73856	4.21120
**Cep57l1**	5.32676	2.48667	3.73443	1.53857	1.65463	4.49093	8.32620	4.19101	0.00000	2.50924	3.25245	6.74900	4.53108	8.84721	3.10347	0.08789
**Cep63**	2.79063	1.10778	3.19197	5.11940	3.15814	0.01060	1.53767	3.55578	2.38622	11.16623	1.71023	5.67744	6.11883	12.67294	8.82635	0.02012
**Cep68**	1.36075	0.02685	2.55735	0.66441	0.00607	2.46468	1.00083	0.00000	2.27016	0.84168	0.64257	0.04927	0.53388	5.87649	0.31316	0.00378
**Cep70**	1.50560	2.43362	1.01949	7.53774	3.49547	5.05927	0.64376	0.39361	1.64762	0.00000	4.72037	1.06930	0.42989	2.58913	3.52107	0.00000
**Cep72**	0.01501	0.02881	1.29042	0.00295	0.01026	0.00890	0.00605	0.11962	0.00929	0.00000	1.56257	0.05888	0.51754	0.77829	0.65905	0.00628
**Cep76**	1.55176	4.23071	2.98167	2.88077	6.97992	0.93518	4.77068	3.27176	3.80717	2.42682	1.83318	0.69957	2.32456	10.47405	5.01196	0.83715
**Cep78**	1.35071	0.00394	1.24313	0.16043	2.73314	0.01435	1.63055	0.01062	0.84121	1.39738	1.01776	0.20195	0.95636	3.55941	2.19059	0.02618
**Cep83**	4.39745	3.13314	4.16817	2.00082	3.34796	1.37034	5.36835	1.61015	1.14733	2.08514	0.98924	3.77555	0.04537	1.81597	1.41243	0.86608
**Cep83os**	0.00980	0.11000	0.14678	0.30212	0.00863	0.00555	0.00732	0.11279	0.70676	0.00000	0.00322	0.18218	0.00740	0.00477	0.42273	0.00706
**Cep85**	2.10420	3.77280	0.00000	4.74202	0.04209	4.87829	0.69518	3.32163	3.95971	4.48225	4.18948	0.15348	0.00000	0.32898	6.34530	0.45112
**Cep85l**	0.01236	0.00787	0.00634	0.07345	0.00970	0.00648	0.00717	0.00945	0.36866	0.00000	0.00000	0.01100	0.01366	1.28802	0.92829	0.00736
**Cep89**	2.09287	0.09270	1.59544	1.81996	1.33808	0.00963	1.46104	2.49457	5.18487	1.69299	2.90559	0.22405	0.02198	0.09332	4.91580	1.03693
**Cep95**	3.19844	2.03956	0.53798	1.83104	0.09410	0.00145	3.24609	2.07104	0.10230	0.15442	0.49534	5.32805	0.66641	0.06535	2.89557	0.33006
**Cep97**	4.11322	0.00272	1.64703	4.34194	0.52411	0.00926	0.04284	0.08533	5.87415	0.00000	2.23076	0.08609	0.03978	1.45230	0.91382	0.02933
**Cept1**	1.67779	0.73998	5.75791	6.05788	0.67725	3.88411	2.43502	6.57902	2.19345	0.00000	2.71939	0.53413	1.52696	2.74231	1.25602	0.02788
**Cercam**	0.06347	0.01824	0.11975	1.21101	0.02668	0.01193	0.07842	0.01619	0.59913	0.09906	0.43733	0.04374	0.05138	0.15670	0.00000	0.01439
**Cerk**	0.92110	0.00560	0.13276	0.28325	0.56655	0.00682	0.82469	0.30367	0.00695	0.68253	0.11531	0.01061	0.32036	0.00543	0.08355	2.38731
**Cers2**	4.06687	10.48140	5.08576	4.31870	5.42397	7.91438	7.23529	18.92379	7.24997	10.43011	11.78445	9.13974	4.86837	17.42869	19.26283	4.48488
**Cers4**	0.01505	0.00785	0.00731	0.00542	0.00933	0.00679	0.00706	0.00000	0.01939	0.00000	0.49745	0.03229	0.45481	0.00694	1.15855	0.00705
**Cers5**	0.92404	0.09731	7.51670	7.67421	4.93286	1.24882	4.95789	2.07330	9.52087	13.49032	6.25720	7.49868	11.10206	11.40733	8.55516	3.81010
**Cers6**	2.39498	0.00472	1.46573	0.96521	0.01026	0.00735	0.24312	0.00000	0.45230	0.16386	1.26542	0.00757	0.28408	0.13754	0.87307	0.01181
**Ces1a**	0.01372	0.00819	0.01080	0.00537	0.00926	0.00670	0.00747	0.01018	0.01037	0.02304	0.08076	0.00760	0.01196	0.00623	0.69374	0.00737

**Ces1d**	0.01277	0.00789	0.01231	0.11323	0.00890	0.00646	0.00725	0.00990	0.00720	1.84839	0.01044	0.00302	0.01063	0.01138	0.00477	0.00729
**Ces1f**	0.01239	0.00770	0.01223	0.00559	0.00868	0.00652	0.00719	0.00943	0.01117	2.73248	0.01060	0.00000	0.01089	0.01224	0.00672	0.00767
**Cetn2**	7.87502	3.94246	3.70633	0.55486	1.09470	3.99351	3.55626	0.13017	0.86566	1.67370	1.65464	0.03155	3.62666	0.00000	2.19068	0.00000
**Cetn3**	13.52497	3.85162	8.65222	1.58666	9.46722	5.04130	0.90789	3.69735	0.22421	0.87998	6.06122	1.04676	4.96176	7.05016	1.05032	1.16682
**Cetn4**	0.00862	0.00820	0.48812	0.30788	0.00737	0.00577	0.00761	0.01322	0.00623	0.26333	0.01171	0.00867	0.00850	0.01138	0.00000	0.63051
**Cfap100**	0.01374	0.00796	0.01233	0.01045	0.00941	0.00659	0.00748	0.01336	0.01240	0.04804	0.01316	0.00765	0.01275	0.01175	0.00995	0.00747
**Cfap126**	0.25789	0.00799	0.00647	1.50645	0.01724	0.00661	0.00695	0.00823	1.12140	1.74197	0.00020	0.00000	0.01042	0.00759	0.05284	0.00683
**Cfap157**	0.01011	0.00762	0.01311	0.09774	0.00917	0.00796	0.00674	0.00000	1.68526	1.20987	1.31532	0.03866	0.22432	0.25284	2.11089	0.00707
**Cfap161**	0.01015	0.00767	0.00742	0.00639	0.00946	0.00676	0.00709	0.93707	0.53499	0.00371	0.00000	0.00521	0.00532	0.00766	0.21748	0.00721
**Cfap20**	5.85928	2.33594	1.42379	7.83648	0.60300	2.67735	0.71899	3.44485	13.44313	3.17474	4.97323	5.15137	2.17315	3.99569	0.69542	2.95555
**Cfap206**	0.00643	0.00729	0.00504	0.28722	0.00915	0.00723	0.00637	0.81100	0.00758	0.70714	0.32459	0.00142	1.06133	0.00608	0.01494	0.00704
**Cfap221**	0.01372	0.00788	0.01163	1.29582	0.00933	0.00654	0.00744	0.01277	0.33365	0.00905	0.00996	0.00708	0.01293	0.05317	0.03538	0.00720
**Cfap36**	2.89773	0.00227	0.74365	1.53473	3.31599	0.00502	1.01426	0.41748	0.00000	0.20872	0.04533	0.66025	0.15513	0.92978	0.68492	0.81324
**Cfap43**	0.16301	4.39689	0.96311	0.43858	0.01317	0.01067	0.00497	1.23864	0.81029	0.00000	1.42388	0.00000	0.31657	1.10262	0.61583	0.01162
**Cfap45**	0.28456	0.00764	0.00415	0.74557	0.00734	0.01358	0.00714	0.00945	0.20706	0.00000	0.14139	0.00653	0.01037	0.00535	0.31287	0.00676
**Cfap46**	0.01340	0.00752	0.02045	0.01066	0.00926	0.00617	0.00716	0.24898	0.01210	0.02877	0.01134	0.00664	0.01672	0.06770	0.01924	0.00755
**Cfap53**	0.01292	0.00784	0.01164	1.05425	0.01871	0.00665	0.00751	0.01262	0.00000	0.04038	0.02707	0.00764	0.01274	0.00506	0.37878	0.00720
**Cfap57**	0.17466	0.00690	2.87995	0.23136	0.00274	0.00724	0.00657	0.14262	0.69524	0.00000	0.51110	0.00907	0.00000	0.45838	0.39374	0.00584
**Cfap61**	0.00682	0.33866	0.01421	0.21829	0.00825	0.00713	0.02700	0.04561	0.00804	0.31156	0.02370	0.00575	0.20645	0.15598	0.35735	0.00690
**Cfap70**	0.01142	0.00763	0.00534	0.00000	0.00936	0.00637	0.00710	0.00838	1.04514	0.50537	0.00000	0.02344	0.01259	1.72529	1.29272	0.00772
**Cfap74**	1.33576	0.26280	1.98451	6.35881	0.02152	0.27469	1.04859	2.86181	7.80411	4.78501	3.68042	2.33759	1.19322	1.27050	2.16495	0.21048
**Cfap77**	0.08199	0.89885	0.05645	0.00903	0.00752	0.00574	0.19996	0.01201	0.01262	0.02147	0.01243	0.00801	0.41560	0.00671	0.06121	0.00653
**Cfap97**	1.76701	0.00357	1.34721	8.51224	0.00188	0.00244	1.79696	0.40887	3.23667	1.69782	1.11931	2.01884	2.39968	0.71779	4.76085	1.85458
**Cfb**	0.82049	0.00660	0.31454	0.41407	0.00579	0.00490	0.00665	1.31213	0.24449	1.63760	0.20377	0.02845	0.04828	0.00825	0.22062	0.01351
**Cfc1**	0.01403	0.00798	0.01004	0.01038	0.00920	0.37968	0.00743	0.01275	0.01243	0.01284	0.01223	0.00807	0.00841	0.01243	0.01052	0.00720
**Cfd**	0.01337	0.00765	0.01230	0.17062	0.00921	0.00641	0.00727	0.01058	0.49551	0.63057	0.01000	0.00454	0.01167	0.00681	0.04698	0.00740
**Cfdp1**	24.34300	14.68915	20.63719	10.93216	15.85179	6.82020	11.86225	24.19528	20.53845	32.71622	21.00828	16.47039	19.74097	25.78138	22.81769	9.46668
**Cfh**	0.01346	0.00829	0.01194	0.02040	0.00925	0.00688	0.00777	0.01889	0.04357	0.07855	0.01224	0.00916	0.01280	0.01198	0.01130	0.00742
**Cfi**	0.01288	0.00770	0.01204	1.02667	0.00885	0.00657	0.00733	0.01078	0.00000	1.45351	0.01074	0.00379	0.01142	0.00366	0.28033	0.00733
**Cfl1**	38.74078	18.30677	29.34116	14.94786	24.98006	17.82518	19.64535	32.91853	19.47783	34.51141	26.24258	3.94269	34.66928	7.16159	4.44718	19.98775
**Cfl2**	8.04688	1.10036	7.62254	11.35741	0.37664	6.93593	6.84905	8.47154	11.09224	15.72357	11.62619	4.49173	5.29550	11.04004	12.92327	0.96867
**Cflar**	4.44369	0.35495	4.47189	24.18466	3.26488	0.57630	0.67165	0.00000	28.84864	2.34823	21.44060	2.97906	2.46364	12.52879	9.06579	1.37102
**Cfp**	0.93886	0.00602	0.00216	0.18514	0.00591	0.00720	0.00613	0.34981	0.50414	0.00000	0.79686	0.36741	0.00019	0.00764	0.24566	0.00603
**Cftr**	0.01358	0.00782	0.01098	1.21186	0.00939	0.00639	0.00737	0.01136	0.00000	0.01180	0.00964	0.00551	0.01325	0.00579	1.42014	0.00717
**Cga**	0.06623	0.00784	0.00864	0.78389	0.00800	0.00669	0.00726	0.00862	0.00000	0.12812	0.18944	0.01652	0.01025	0.00809	0.00000	0.00714
**Cggbp1**	8.10558	4.06617	7.48716	21.09936	4.04316	2.66775	5.96449	8.96859	34.43994	7.43282	7.49200	5.02065	3.71450	21.19763	3.81948	5.38289
**Cgn**	1.03548	0.00390	0.78623	4.21985	1.92073	6.37916	1.16507	7.45422	3.89919	0.46797	4.76945	0.65614	2.34091	2.81104	1.23874	1.32667
**Cgnl1**	1.00001	0.00579	0.65540	1.86273	0.07457	10.40752	3.01160	2.33666	1.17789	0.51867	0.00000	0.02679	3.20349	0.02557	0.12150	5.01241
**Cgref1**	0.36572	1.03436	3.31950	0.00864	0.00145	0.00670	0.00601	0.46401	0.40107	1.44703	0.83861	0.06734	0.64176	2.23841	1.00232	0.00478
**Cgrrf1**	0.44778	0.01819	0.73239	0.07044	0.00578	1.02549	0.00711	0.01185	0.00840	0.01000	0.00959	0.02983	0.01481	0.01376	0.00000	0.01331
**Chac1**	0.01686	0.00756	0.01803	0.54527	0.00859	0.00697	0.00718	0.01004	0.22573	1.21301	0.01257	0.00427	0.01039	0.00942	0.06868	0.01399
**Chac2**	0.43151	0.00818	0.18728	0.00992	0.02029	0.00594	0.00728	0.01064	0.01225	0.10861	0.01081	0.02541	0.25863	0.70919	0.00000	0.00767
**Chadl**	0.02600	0.00782	0.00982	0.31365	0.00840	0.00658	0.00715	0.00420	0.26776	0.57847	0.24688	0.00443	0.04128	0.00831	0.00000	0.00732
**Chaf1a**	7.60015	5.24704	5.56959	9.27825	5.55145	10.87203	2.57385	8.16348	8.50581	9.72537	2.74815	1.07828	6.38054	1.99710	10.49936	6.88568
**Chaf1b**	5.72707	0.31939	4.67623	4.62563	1.65645	5.69648	5.84001	9.70993	5.61337	11.42566	9.73811	1.11701	4.47458	1.22790	1.49231	2.83076
**Champ1**	3.27056	0.04435	3.95035	7.09393	0.08458	0.27156	0.25168	11.01433	10.61936	14.73003	7.99977	8.18892	5.05422	13.31560	14.22894	1.23183
**Chchd1**	8.84263	1.11311	2.99523	2.82461	7.74109	1.32799	2.15107	3.11473	3.63154	4.61450	8.66636	0.74466	5.63199	1.44819	2.40436	5.39674
**Chchd10**	1.92651	0.09430	0.03136	1.70635	0.05475	0.57667	0.16237	0.07521	6.27982	3.94471	5.75980	0.11886	0.85951	0.00000	0.00000	0.39973
**Chchd2**	22.47995	0.91416	9.03793	21.11546	20.30303	3.76473	1.59194	3.95607	38.92982	41.52628	32.37118	3.02783	29.07430	3.69584	0.56624	14.20186
**Chchd3**	9.93267	1.53495	6.49415	0.98654	6.21286	4.00191	5.43360	6.05973	2.16152	5.49299	0.49698	0.34990	7.71843	0.30349	0.83887	9.73724
**Chchd4**	15.63456	8.37323	14.17327	0.94180	16.85249	6.55320	7.52017	8.23904	1.54076	4.51410	1.75453	7.45013	16.12554	1.66146	1.93503	6.61231
**Chchd5**	12.05364	5.80494	13.94172	0.37711	3.32196	2.39195	7.63239	5.31569	3.22441	7.26170	7.74439	1.27167	9.45101	0.70257	2.27351	3.83458
**Chchd6**	4.77712	0.01188	2.14212	0.64962	0.88772	0.00643	2.02161	2.26329	0.34692	0.87213	0.52578	0.10552	4.07133	0.05797	0.00000	0.00698
**Chchd7**	1.47683	0.00625	0.37547	0.04939	0.03707	0.45620	0.22873	0.00579	1.08061	0.56034	0.00000	0.05924	2.85929	0.01698	0.06850	3.87603
**Chd1**	3.16417	8.17072	4.94854	11.08981	8.02377	4.32624	5.26889	8.67852	13.41893	8.97099	2.00360	7.79384	0.60441	16.19579	18.79179	2.27691
**Chd1l**	0.00000	0.00454	2.43751	0.00000	3.74425	0.27528	1.65798	1.85360	0.31088	2.33190	1.28248	0.10799	1.31033	0.05710	1.08435	0.01237
**Chd2**	0.06204	3.01644	0.05641	0.20646	0.01262	0.00562	1.34515	1.15905	0.27324	0.46546	0.01199	0.13998	1.88565	0.01323	2.57770	1.10727
**Chd3**	0.65598	0.00607	0.86918	0.69441	0.67283	0.10897	0.01647	0.01715	1.17085	1.02873	0.04952	0.04675	0.01260	0.00342	0.20173	2.11885
**Chd3os**	0.92227	0.00600	0.00000	0.66037	0.00592	0.04835	0.00570	0.78797	0.56682	1.90334	0.95458	0.03982	0.77717	0.00293	0.54164	0.00571
**Chd4**	15.00128	0.26597	1.84412	2.16272	7.71417	3.22366	7.60230	17.60518	5.46000	21.87101	5.01587	5.64646	13.07335	1.72672	0.00000	11.17090
**Chd5**	0.01282	0.03784	0.00690	0.00488	0.00894	0.00699	0.00689	0.00000	0.01103	0.68477	0.90881	0.00126	0.00514	0.01072	0.00000	0.00725
**Chd6**	2.24774	0.23378	1.37409	2.21111	3.08680	3.96612	1.93943	3.91240	3.12033	3.22135	1.74321	2.78911	0.52967	0.00614	1.34631	2.14521

**Chd7**	2.43335	0.01325	0.18855	6.23302	0.09248	0.01168	4.56765	0.44553	3.50689	14.34718	2.70773	0.46017	0.45481	4.10257	9.27226	1.35406
**Chd8**	1.05338	0.58235	1.43438	9.32582	0.12968	1.14829	1.37055	4.43574	12.58411	4.95017	2.22814	0.24179	1.96332	6.92897	5.86657	0.80119
**Chd9**	1.59454	0.00526	0.05301	3.34029	0.01461	0.00428	1.20968	0.00378	0.81437	0.00000	0.00000	0.09278	0.23628	4.93391	1.78521	0.01244
**Chdh**	0.00000	0.00744	0.58223	0.37812	0.01619	0.00563	0.00723	0.39475	0.17405	0.10178	0.00728	0.00523	0.00000	0.20284	0.00000	1.65564
**Chek1**	7.09093	5.79590	4.04142	6.48808	9.86708	1.30021	3.26975	4.81981	4.55571	2.30369	1.39801	3.20828	1.95338	8.33016	8.35424	0.19866
**Chek2**	4.19200	1.71462	0.32109	9.66217	4.59389	1.65991	5.58687	12.73654	10.88883	10.21257	11.71123	7.58026	1.27465	18.55379	18.86839	0.02463
**Cherp**	11.47321	3.12265	5.51589	12.95362	4.85088	1.13639	4.66960	19.20427	10.37878	23.23528	9.34656	8.09223	11.38392	6.27560	9.48074	7.16050
**Chfr**	2.43939	3.94609	4.93892	2.16399	4.82868	1.83083	5.91336	3.70182	3.86824	3.70427	1.01500	4.34184	1.62937	0.79851	4.20001	1.03882
**Chga**	2.07211	0.23510	0.00803	0.29586	4.53173	0.01634	1.69335	0.49897	0.01325	0.03546	0.00653	0.06028	3.91591	0.00993	0.00000	0.01041
**Chgb**	0.01610	0.00785	0.07083	0.29337	0.00910	0.00579	0.00734	0.01251	0.00687	0.04768	0.01511	0.00817	0.01112	0.01476	0.00420	0.00713
**Chic1**	0.25898	0.01732	0.00000	3.11764	0.00763	0.01964	0.12011	0.00000	1.57452	4.13227	3.64616	0.90482	1.48966	13.33127	5.53110	0.22284
**Chic2**	3.76303	0.00000	0.12197	3.61318	3.09452	0.00125	0.49820	0.00000	0.00000	5.85454	4.30294	1.93367	1.43009	3.15344	0.12863	0.00000
**Chid1**	5.68290	4.17247	7.50703	0.26136	0.45014	8.22795	5.66797	2.93410	0.04023	0.46719	1.74677	0.15250	6.47218	2.53744	0.24768	0.15688
**Chil1**	0.00757	0.00511	1.17744	0.00877	4.67832	0.00552	0.00731	0.00874	0.01019	0.01171	0.01093	0.00280	0.00728	0.01606	0.00000	0.00675
**Chil5**	0.01348	0.00808	0.01035	1.44156	0.00937	0.00642	0.00734	0.01102	0.32739	0.01362	0.00776	0.00523	0.01270	0.21678	0.44639	0.00701
**Chit1**	0.00000	1.98419	0.91913	1.61734	0.02473	2.17913	1.12623	0.77513	2.27102	0.00406	0.00000	0.00000	0.00000	0.00165	0.25475	0.04709
**Chka**	0.00000	0.23397	9.29511	14.97095	4.55734	5.19103	8.41087	27.99796	19.67518	44.94642	21.10205	21.48357	9.14961	6.40681	19.51492	8.22437
**Chkb**	0.31067	0.07072	0.12642	0.22314	0.00720	0.03695	0.17494	0.04765	0.49554	0.09666	0.55056	0.00423	0.08114	0.01095	0.00438	0.00667
**Chm**	0.19112	0.01915	3.03934	0.50168	0.00732	0.00516	0.00637	1.32322	0.00002	0.03480	0.72918	0.67245	0.06511	4.17998	0.24041	0.30711
**Chml**	0.00603	0.00564	0.00198	1.11950	3.56765	0.00769	0.00722	0.00481	1.11233	0.01847	0.05756	0.00136	0.00907	0.01740	0.43505	0.00675
**Chmp1a**	8.00235	2.23199	6.47343	2.68852	9.42457	3.62837	0.15337	7.28576	1.94272	11.54380	2.84729	0.45391	9.10404	0.02071	3.79446	6.74748
**Chmp1b**	0.39184	0.06039	2.09998	4.29745	1.82430	0.10754	1.31399	7.73138	4.21969	1.58252	3.04511	0.48868	2.04625	3.30800	3.72508	0.56744
**Chmp2a**	12.83939	9.67516	17.27627	7.05803	4.58915	0.04342	12.31122	13.62786	9.49890	13.58780	8.31421	2.62658	8.47456	1.19980	10.02023	6.75000
**Chmp2b**	2.56363	1.65525	1.19816	1.22124	10.48596	0.01586	2.05572	0.32903	0.72015	1.26468	0.00000	0.00516	4.95100	0.00295	0.00000	3.54814
**Chmp3**	3.59080	6.91893	7.34574	1.50442	4.76558	7.73827	1.59753	7.06099	0.21076	5.13222	2.22981	7.08863	7.68758	6.57748	3.57442	4.43284
**Chmp4b**	3.31219	0.21646	1.84994	0.55720	1.19471	0.87734	0.11526	0.00000	4.36245	1.20925	6.30179	0.20882	0.11604	2.75218	0.25243	0.12721
**Chmp4c**	7.87570	0.50827	2.00240	0.00886	0.03999	0.00000	1.99013	5.62911	0.18519	1.57895	4.72699	0.05715	0.00000	0.58914	0.00000	0.08405
**Chmp5**	6.15172	6.02562	3.87156	5.93538	10.84472	2.26172	2.37529	3.65017	4.64942	2.33686	4.72968	0.05917	0.18238	9.72265	6.72113	4.07466
**Chmp6**	8.20621	0.00276	3.93397	0.09136	5.49896	4.55833	3.91758	2.85182	1.03185	1.62211	0.28797	0.08938	6.63755	0.17577	0.00000	5.12027
**Chmp7**	9.10654	2.28686	4.47180	4.93211	9.67268	4.28176	5.55874	11.51699	5.07075	11.43018	4.29269	2.10808	5.57663	0.09249	2.25373	4.17966
**Chn1**	1.88606	0.72239	0.13294	2.72057	0.79108	0.00916	0.00548	0.00000	2.26013	0.02649	0.32431	0.12974	0.08071	0.02250	1.55029	1.75134
**Chn2**	0.01561	0.00822	0.00778	0.42733	0.01192	0.00737	0.00739	0.01443	0.00489	0.33395	0.13503	0.00278	0.24872	0.91796	0.00000	0.00783
**Chordc1**	5.03912	8.00469	9.06894	1.26755	4.46752	3.00635	3.55397	5.33683	2.91782	4.36745	3.36908	8.81059	4.54658	11.89874	6.62626	0.92183
**Chp1**	1.73683	4.27201	12.51233	9.87020	8.65580	14.12560	6.25223	11.93085	11.35922	16.37140	13.14960	5.38327	9.97657	20.61259	16.54279	0.45032
**Chpf**	6.79710	0.00000	0.00000	0.28145	3.53041	0.06423	1.40612	3.44676	0.05278	0.00000	0.00000	0.02127	2.02251	0.01629	0.00000	4.02272
**Chpf2**	2.02173	0.39515	0.68642	0.28708	2.13217	0.00396	1.94552	2.29468	1.77195	0.09027	0.00067	0.02226	0.00159	0.00480	0.00000	0.00470
**Chpt1**	4.16843	4.70851	2.71178	1.67503	0.04262	1.61923	3.51096	1.96460	5.12434	8.42627	6.61988	5.38007	1.58014	15.98437	6.78476	0.00000
**Chrac1**	11.56685	4.91216	4.67893	2.70085	6.92482	0.02635	3.15302	6.85296	6.30913	3.73900	3.22590	0.23434	3.72927	0.31685	1.73331	2.11139
**Chrna1**	1.35573	0.05754	1.22018	7.76896	0.73351	0.18150	0.15329	0.00000	11.60309	0.00000	9.75886	0.74239	0.39966	3.66831	0.38677	0.28572
**Chrna10**	0.01108	0.00708	0.00183	0.00411	0.00909	0.00765	0.87799	0.00000	0.32353	0.77629	0.72342	0.03029	0.00647	0.00507	0.70333	0.00720
**Chrna5**	0.01206	0.00763	0.61402	0.79509	0.00819	0.00894	0.00709	0.00968	0.00000	0.51678	0.01113	0.03279	0.00993	0.00931	0.00000	0.01448
**Chrna9**	0.09990	0.00753	0.00000	0.18888	0.00759	0.00658	0.00686	0.00000	1.14327	0.69963	0.64237	0.00000	0.01139	2.32464	0.42662	0.00669
**Chrnb1**	0.01962	0.03874	1.25422	0.26464	0.00710	0.00573	0.00680	0.00000	0.45691	0.00000	0.68139	0.05549	0.01169	1.16489	0.53308	0.00661
**Chrnb4**	0.16519	0.03848	0.56881	0.03171	0.00694	0.00849	0.00722	0.01188	0.12886	0.05874	0.20970	0.03655	0.03436	0.05991	0.05548	0.00682
**Chrnd**	0.01393	0.00800	0.00850	0.48103	0.00904	0.00677	0.01739	0.00000	0.00274	0.18619	0.47980	0.00474	0.00830	0.00502	0.28022	0.00758
**Chrne**	0.01136	0.00834	0.97412	1.72811	0.00824	0.00572	0.01869	0.01016	2.24789	0.66288	0.06710	0.01205	0.05512	0.00705	0.00000	0.00669
**Chst10**	0.01321	0.00792	0.01226	0.47457	0.00902	0.00652	0.00723	0.01014	0.00286	1.06172	0.01172	0.00492	0.01143	0.01024	0.00416	0.00757
**Chst11**	0.01352	0.00788	0.01153	0.00484	0.00932	0.00657	0.00754	0.01337	1.69771	0.00990	0.00000	0.00492	0.01323	0.01931	0.02221	0.00733
**Chst12**	3.61174	0.02431	1.29046	2.50733	0.00669	2.16300	0.57180	1.47120	1.06889	2.20543	0.74129	0.10841	2.39909	0.02553	0.00000	3.13663
**Chst14**	0.07640	0.00851	0.00818	0.03438	0.01423	0.00716	0.00751	0.01290	0.00994	0.04280	0.01160	0.05387	0.01312	0.01170	0.01022	0.00752
**Chst2**	0.00811	0.00756	0.00611	0.19398	0.47640	0.01397	0.00748	0.02407	0.02588	0.04494	0.01113	0.01909	0.01704	0.01015	0.02697	0.00723
**Chst3**	0.03685	0.00631	1.20465	0.00994	0.01375	0.01856	1.91251	0.00000	0.31944	0.65306	1.68878	0.02546	0.34479	0.14614	0.39522	1.15050
**Chst5**	0.01321	0.00817	0.01113	0.39224	0.00917	0.00630	0.00756	0.01249	3.76019	0.04081	0.00000	0.01107	0.01259	0.00945	0.01032	0.00694
**Chst7**	0.01345	0.00810	0.01207	1.51692	0.00932	0.00630	0.00729	0.01137	1.20664	0.32185	0.00088	0.00484	0.01236	0.00925	0.00301	0.00715
**Chst8**	0.01362	0.00821	0.01197	0.36882	0.00927	0.00657	0.00753	0.01387	0.88089	0.01111	0.00823	0.00691	0.01289	0.01193	0.00362	0.00730
**Chsy1**	0.80397	0.00660	0.69162	7.01171	0.00506	0.00402	0.00649	0.00519	1.23278	1.87494	0.03568	0.03794	0.00822	0.00000	1.03882	0.33602
**Chtf18**	1.28318	3.14273	4.10770	3.81912	0.24627	1.38003	2.32043	5.17577	5.43363	5.29012	4.20659	0.20331	5.76192	0.01302	0.00000	3.95137
**Chtf8**	4.47807	1.71698	1.46042	1.42098	1.45778	0.79710	0.23837	2.79089	2.15077	4.73838	0.35594	0.63523	3.34300	0.38776	1.44875	1.32664
**Chtop**	11.22726	8.60583	10.51565	11.26657	7.79266	4.72033	2.47689	8.14849	15.68314	16.07351	4.60498	7.14677	9.99041	5.59820	10.80520	5.72661
**Chuk**	2.91238	1.64324	0.00000	4.02465	0.01337	1.72314	0.01351	0.78830	2.11330	0.41052	1.45854	1.52786	1.65124	8.07757	6.81625	0.36797
**Churc1**	3.76178	0.68238	1.25426	8.56777	1.25644	0.68206	1.13801	1.29237	13.25872	2.14907	15.47537	1.63474	2.21449	8.34711	3.41120	1.20923
**Ciao1**	3.48983	9.95622	9.13603	4.15358	6.07338	1.72818	3.29674	9.34562	4.95848	4.77707	7.07917	5.63431	7.60093	7.29603	3.44374	1.05565

**Ciapin1**	8.15528	2.15763	11.09682	3.73044	0.44730	0.39588	0.25623	7.46701	0.58558	3.88917	3.17652	3.34080	11.21739	1.36254	0.66197	8.79683
**Ciart**	2.41008	1.23710	0.71292	0.77482	0.00216	0.00287	2.32108	1.55384	1.92056	2.87313	0.00000	0.00166	0.46990	0.00271	2.90344	0.00325
**Cib1**	7.10434	1.35006	5.99460	0.24746	12.07041	0.04214	2.11151	10.10077	0.18205	11.15004	1.24588	0.61101	6.38834	0.00000	0.00000	0.44276
**Cib2**	0.01130	0.06983	0.03596	0.00372	0.00884	1.16464	0.00716	0.01112	0.13200	0.06021	0.00966	0.00714	0.02812	0.01145	0.00766	0.04345
**Cic**	4.03500	0.28897	1.49119	7.52490	0.36646	0.00904	0.45436	1.98823	5.32062	3.86126	2.19369	0.14034	4.73394	2.06285	2.33722	5.47081
**Cidea**	0.00000	1.72255	2.33812	0.18626	0.00399	3.29390	0.98477	0.37165	0.11197	0.30100	0.83775	0.02310	0.00000	1.15422	0.46011	0.02025
**Cideb**	0.00839	0.00757	0.27312	0.00963	0.08499	0.00825	0.00729	0.00876	0.05590	0.09751	0.08149	0.00608	0.07500	0.50550	0.00000	0.00713
**Cidec**	0.01789	0.00771	0.00801	0.01768	0.00880	0.00716	0.00732	0.25763	0.01076	0.15695	0.02130	0.00733	0.00709	0.00640	0.12244	0.00734
**Ciita**	0.01096	0.00753	0.00602	1.11149	0.00944	0.00678	0.00723	1.01154	0.38969	0.06741	0.01002	0.00312	0.00602	0.00230	1.08173	0.00718
**Cilp**	0.00696	0.00769	0.99917	0.00000	0.01996	0.01923	0.00722	0.08747	0.42974	3.10341	0.47931	1.21220	0.40347	0.00552	1.20956	0.01470
**Cilp2**	0.19211	0.00766	0.00511	0.00717	0.00738	0.00724	0.00714	0.01608	0.01176	0.86540	0.03832	0.12602	0.01630	0.01199	0.00446	0.00685
**Cinp**	3.72430	0.31361	4.91624	0.81039	0.12785	0.78147	1.53297	4.12636	3.59685	3.80606	0.97950	1.63193	3.25219	1.02686	3.04644	2.11266
**Cipc**	1.24511	0.00571	0.65430	0.56878	0.02613	0.00471	0.00619	0.98843	0.50521	0.56953	0.00000	0.04406	0.24690	0.01143	0.63457	1.76872
**Cir1**	6.25669	7.35061	3.05988	6.11716	5.15269	1.87415	3.60006	6.41468	12.24973	5.13921	4.58379	1.39913	2.79878	8.77765	4.79026	0.48839
**Cirbp**	2.19442	0.01540	1.73284	0.72204	0.00000	0.03830	0.00374	5.21037	1.10912	2.31185	1.38449	0.16910	2.38567	0.00233	0.24396	2.89471
**Cirh1a**	15.90808	11.69034	10.45515	7.48107	9.88505	5.65007	5.35873	21.56047	16.10462	26.21582	16.27102	18.80105	7.02049	12.81164	20.82486	5.90318
**Cisd1**	8.29985	0.15495	4.38381	3.38962	8.90622	3.40156	1.77729	1.15053	0.00000	8.28573	6.46657	0.15575	10.18429	1.27846	1.22738	3.18523
**Cisd2**	1.72769	0.27681	0.08876	3.49059	4.16582	2.63231	0.53431	1.86483	1.61224	6.22587	1.95740	1.81577	1.73323	2.81139	1.60464	1.18220
**Cisd3**	0.32444	0.00739	1.21045	0.14872	0.00754	0.03556	0.00678	0.12408	0.00661	0.65798	0.19443	0.05464	0.25476	0.01241	0.00562	0.00663
**Cish**	0.41920	0.00471	0.38024	0.25861	1.30553	5.64922	0.00347	7.13458	0.00371	15.15217	5.43955	0.62956	2.02204	0.53573	3.15118	1.57754
**Cit**	0.51892	0.00664	0.22578	0.11440	0.02249	0.00824	0.00647	0.16926	0.00859	1.19091	0.62389	1.03568	0.01436	0.05573	0.94772	0.01396
**Cited1**	14.79199	5.84559	14.84098	3.38543	6.15013	0.98133	10.55531	36.86908	9.20176	47.27966	27.34264	12.62055	22.46079	1.28478	7.26232	2.73378
**Cited2**	1.41833	0.00714	0.18946	0.45504	0.01034	0.00746	0.00691	0.40673	2.05179	0.79233	0.00000	0.13009	0.00882	0.41354	0.00000	1.30895
**Cited4**	0.01920	0.00840	0.00580	1.03190	0.01049	0.95908	0.00738	0.01127	0.57519	0.18735	0.00743	0.00671	0.01335	0.04729	0.00000	0.00683
**Ciz1**	1.16169	1.68987	0.72376	2.47569	0.02159	0.02203	0.04913	0.82379	4.19006	1.10429	0.37698	0.12609	2.25389	0.00000	0.03585	3.85407
**Ckap2**	4.94037	3.02349	4.36549	7.54262	3.26831	3.17508	3.92597	6.05343	13.45497	5.89335	5.46967	3.38240	3.96723	1.26547	10.04402	2.05951
**Ckap2l**	3.90918	8.85163	3.58279	2.25696	5.42644	3.29697	2.32754	1.57020	4.00181	3.28879	4.64555	0.61880	1.84003	2.83728	5.14852	0.58719
**Ckap4**	2.00991	0.00259	0.42389	0.00000	6.54876	0.00350	0.57205	1.13429	0.00596	1.77316	0.01612	0.12606	0.71145	0.00660	0.22891	9.09644
**Ckap5**	4.29005	10.00011	5.11618	5.63193	3.31870	3.55874	2.29802	6.48278	11.70115	7.24249	2.68196	2.71437	1.85734	5.15206	12.88190	2.41131
**Ckb**	41.84355	6.81109	11.84601	7.57630	27.80023	12.00151	8.81979	28.91076	6.62231	33.46555	23.34044	12.53443	37.60882	0.00000	0.00000	16.77175
**Cklf**	0.07510	0.01749	0.27907	0.95712	0.00675	0.01265	0.00597	1.04476	3.16056	0.00000	0.88844	0.02107	1.58483	0.47783	0.00000	1.10505
**Ckm**	0.63632	0.00752	0.00000	0.01030	0.01771	0.33451	0.11440	0.29170	0.01208	0.05474	0.01076	0.00583	0.47792	0.01206	0.00636	0.00689
**Ckmt1**	16.44452	7.14395	21.13911	0.00000	7.36495	1.12807	10.66214	14.94947	0.00873	4.92023	12.49304	1.04287	19.90845	5.77347	0.00000	12.63969
**Cks1b**	15.83905	5.42702	15.37518	6.67419	8.58640	10.54457	8.14243	11.86930	7.02723	13.06430	8.80102	2.84120	10.30104	3.10048	6.83454	3.91455
**Cks1brt**	1.10072	0.36928	1.20033	0.84679	0.61139	0.87354	0.63324	0.56057	0.73497	0.68740	1.00795	0.11043	0.59784	0.16827	0.36030	0.26629
**Cks2**	18.28303	6.11436	15.95053	24.43607	18.14089	5.53856	2.73569	19.66073	34.12815	40.05129	39.92893	12.02893	13.79710	15.65604	21.80093	3.99250
**Clasp1**	2.45802	2.96625	0.99580	1.87072	2.31931	0.03253	0.00402	1.78116	0.86215	2.25066	0.05700	0.06862	0.52246	0.00849	0.35766	3.72015
**Clasp2**	0.00292	1.73601	0.35903	3.98666	0.07612	0.00619	0.10843	0.75531	2.56414	2.55724	1.28049	0.03153	0.21794	0.39221	0.77676	0.02754
**Clasrp**	2.87373	1.08986	0.75881	1.77262	0.00915	0.00349	0.01292	1.21310	3.24427	0.26692	2.95819	0.05147	1.35895	0.00000	0.00000	1.58913
**Clca3a1**	0.09447	0.05064	0.29233	0.52829	0.07943	0.10062	0.06935	0.06708	0.56933	0.02577	0.53671	0.03242	0.08113	0.52158	0.12116	0.10970
**Clca3a2**	0.02141	0.01829	0.04938	0.22974	0.01993	0.00656	0.01741	0.02302	0.20653	0.00585	0.29783	0.00567	0.01781	0.05805	0.13326	0.02909
**Clca4c.ps**	0.05053	0.00765	0.02044	0.12119	0.00854	0.01531	0.00729	0.03510	0.09213	0.02413	0.02521	0.00732	0.01079	0.05914	0.01517	0.00731
**Clcc1**	2.20771	0.79855	4.40265	2.63659	4.31072	4.03298	0.00434	3.50319	0.81431	3.41408	0.54247	0.14373	0.00000	0.03104	1.88292	2.05809
**Clcn2**	1.71583	0.00451	0.47442	1.90306	0.08675	0.01827	1.14302	4.16174	2.27266	0.21699	2.16988	0.07515	1.44957	0.04811	1.30014	0.85805
**Clcn3**	3.02882	1.37032	4.52569	1.19695	1.55021	0.03016	1.10319	2.92928	0.47686	1.66824	3.17422	3.57495	0.43739	2.37894	2.21065	0.45616
**Clcn4**	0.33020	0.00240	2.90024	0.01751	1.65264	0.00483	0.53789	0.65322	0.04356	5.74203	2.65418	11.25497	4.53972	2.63933	2.01573	0.01732
**Clcn5**	0.00982	0.00785	0.01728	1.72789	0.00974	0.00655	0.00707	0.05433	0.15530	1.24675	0.02260	0.00000	0.91386	0.04781	1.35361	0.00686
**Clcn6**	0.45688	1.43068	0.49337	3.01205	0.08797	0.48785	1.93356	1.13501	2.82188	1.63922	3.42511	0.48076	1.79670	0.21117	2.12096	0.01119
**Clcn7**	5.15696	0.70308	3.17493	3.88580	0.56833	0.66577	4.42711	6.11556	4.98672	1.89947	2.68400	0.40482	4.97415	0.26922	2.17472	3.85722
**Clcnka**	0.00000	0.00665	0.70320	0.38729	0.02605	0.00573	0.00529	6.56976	0.00428	3.40004	3.12699	0.00000	1.84338	0.00000	0.03126	0.00590
**Clcnkb**	0.82016	0.00638	0.20394	0.00611	0.00582	0.00511	0.00631	0.66409	0.01064	0.00000	0.32084	0.00412	0.51176	0.01565	0.00536	0.01292
**Cldn10**	0.01310	0.00755	0.01121	0.12553	0.00894	0.00657	0.00730	0.01026	0.27772	0.96076	0.00896	0.01362	0.01167	0.00890	0.44219	0.01512
**Cldn12**	1.76225	2.26845	1.14995	0.52448	0.03280	1.01265	1.26043	1.02323	0.43040	0.11723	2.55502	0.19661	1.85710	1.41476	2.30087	0.22972
**Cldn15**	0.01224	0.00762	0.00543	0.92969	0.00937	0.00678	0.00701	0.27784	0.00000	0.00000	0.23755	0.00440	0.00520	0.00360	0.69937	0.00700
**Cldn19**	0.01104	0.07918	0.02132	0.00908	0.00897	0.00682	0.12827	0.01624	0.01221	0.00822	0.01243	0.00702	0.01783	0.00629	0.22016	0.00728
**Cldn22**	0.01542	0.00764	0.54334	0.00975	0.00836	0.00559	0.00722	0.01054	0.01222	0.25352	0.01207	0.00649	0.00923	0.01378	0.00147	0.01452
**Cldn3**	0.73422	0.00772	0.01107	0.27423	0.05648	0.00723	0.00647	0.00522	0.24768	5.29722	0.04132	0.00000	0.08567	0.01045	0.00212	0.25476
**Cldn34b1**	0.01351	0.00825	0.01106	0.11716	0.00924	0.00694	0.00753	0.01265	0.00768	0.16072	0.01123	0.00816	0.01309	0.15189	0.00435	0.00740
**Cldn34b2**	0.01271	0.00810	0.00805	0.81627	0.00924	0.00697	0.00736	0.01055	0.00000	0.36263	0.02122	0.00542	0.01258	0.77224	0.01517	0.00726
**Cldn34c1**	0.77522	0.00762	0.00000	0.78814	0.03766	0.09557	0.01730	0.01159	0.00595	0.12554	0.01020	0.00577	0.01206	0.08789	0.48152	0.04461
**Cldn34d**	0.01004	0.00841	0.00847	0.47732	0.00958	0.00665	0.00816	0.25945	0.28938	0.00423	0.00864	0.01870	0.00654	0.00611	0.42517	0.00718
**Cldn4**	0.00050	0.10381	6.97553	0.00000	0.53731	17.80297	2.56043	23.45781	0.53138	9.97935	12.89922	0.01702	25.73508	4.13357	0.68542	12.76557

Cldn5	0.01318	0.00772	0.00902	0.00467	0.00875	0.00684	0.00716	0.00000	0.01099	1.48451	0.49232	0.00000	0.00766	0.00600	0.11533	0.00723
Cldn6	0.00000	7.10709	6.01159	0.63681	0.10489	12.86469	0.00000	5.52615	0.00000	5.50385	1.01719	0.14726	11.00933	1.45041	2.39804	1.68983
Cldn7	3.85230	5.08215	13.01790	0.88356	0.08659	12.23946	4.51560	19.61171	1.87190	8.33644	10.95029	0.70621	19.90824	0.07848	0.00838	9.63030
Cldn9	0.00000	0.00730	0.44454	1.10415	0.00841	0.00890	0.00554	0.93122	1.82269	0.00000	0.01811	0.25162	2.84351	0.00887	0.00000	0.00560
Cldnd1	2.17048	0.12315	1.40516	2.67152	7.75273	3.72819	4.50166	1.75082	6.58025	2.28128	2.18459	6.03258	0.22024	4.63922	2.20249	0.74060
Clec10a	0.00305	0.00770	0.05894	0.25686	0.00848	0.00596	0.00358	1.76937	1.44113	0.91027	0.00000	0.02106	7.26441	0.56700	0.42327	0.00433
Clec11a	0.01571	0.00830	0.00868	0.21097	0.00991	0.28969	0.00761	0.01604	0.00648	0.00724	0.01461	0.00795	0.40897	0.01195	0.00703	0.00734
Clec16a	3.26205	0.00549	2.17408	0.44093	0.00431	2.21975	0.06103	0.02606	0.46896	1.00993	0.16408	0.03636	0.93034	0.00471	0.90695	0.00190
Clec2e	0.01365	0.00818	0.01199	0.36454	0.00924	0.00655	0.00750	0.01393	0.46263	0.01255	0.01065	0.00760	0.01914	0.01158	0.00572	0.00729
Clec2f	2.29163	0.00578	0.04072	0.21849	0.00547	0.00392	0.00578	0.01297	0.00545	0.33948	0.01307	0.01528	0.02839	0.01068	0.00601	0.00558
Clec2g	0.07582	0.00766	0.03109	0.83766	0.00809	0.08116	0.00704	0.02263	0.73943	0.03509	0.05938	0.04066	0.01179	3.43303	0.00000	0.01454
Clec2h	0.07304	0.00805	0.01682	0.67241	0.00795	0.02159	0.02832	0.03906	0.14908	0.29718	0.09417	0.00719	0.02236	0.04879	0.25179	0.00720
Clec2i	0.00935	0.00789	0.03364	0.03865	0.00981	0.00599	0.00735	0.88617	0.12477	0.01109	0.00000	0.00409	0.00610	0.66295	0.20276	0.00742
Clec2l	0.01377	0.00781	0.01124	0.17642	0.00934	0.00662	0.00744	0.01253	0.43100	0.00753	0.00892	0.06537	0.01290	0.00664	0.09326	0.00735
Clec3b	0.01330	0.00780	0.01138	0.00785	0.00934	0.00668	0.00744	0.01711	0.03391	0.01343	0.07457	0.01713	0.02001	0.01228	0.04743	0.02936
Clec4a1	0.16042	0.00805	0.08347	0.32602	0.04040	0.00573	0.00770	0.04090	0.79691	0.49377	0.00599	0.00717	0.01665	0.01501	0.02206	0.00697
Clgn	1.19938	0.00698	0.00254	0.00392	0.03355	0.00675	0.00641	0.00747	0.34689	0.00529	0.00880	0.00538	0.01148	0.00719	0.42845	0.00625
Clhc1	0.02885	0.00766	0.00098	0.01259	0.00836	0.02485	0.00705	0.01323	2.26419	0.60116	0.00000	0.02316	0.14491	2.95457	0.49381	0.00709
Clic1	3.97352	2.12545	0.00000	0.07903	0.03272	0.01038	2.12589	0.00000	0.12373	1.66024	0.75844	0.06018	0.92273	0.00405	0.46339	7.18305
Clic3	0.00000	0.26215	2.56483	0.00170	0.02064	0.07640	2.20201	0.79226	0.00990	0.00000	2.57102	0.05758	3.51105	0.00506	0.00961	0.45693
Clic4	7.20165	13.42566	14.90958	4.99758	8.06232	16.90092	4.73224	8.51512	14.36160	3.29149	4.29321	4.64865	11.28962	4.90122	1.28868	5.10217
Clint1	6.23081	8.03057	1.56170	1.79431	4.40769	3.29985	5.48257	6.10441	2.46742	4.00121	1.29660	1.96546	1.56015	7.70649	6.28286	5.69638
Clip1	0.83718	5.13145	1.04855	5.38811	0.00436	0.68779	0.37736	0.48225	4.87366	2.42131	4.75582	0.78390	0.31148	2.92239	4.51825	1.13319
Clip2	0.00878	0.00803	0.00997	0.45538	0.00890	0.00640	0.00669	0.00865	0.00532	0.54103	0.03859	0.10539	1.05455	0.03238	0.00000	0.02738
Clip3	0.01378	0.00789	0.01108	0.26558	0.00932	0.00655	0.00733	0.00882	0.04906	0.18414	0.11822	0.02596	0.01094	0.00923	0.00529	0.00739
Clip4	0.01379	0.00766	0.00967	1.32189	0.00924	0.00630	0.00724	0.00478	0.00000	0.08861	0.20889	0.00446	0.00979	0.00437	0.91616	0.00701
Clk1	7.54861	10.39900	1.24553	9.80388	10.37963	10.20844	10.43465	6.76855	18.00432	11.85835	6.89271	18.77604	5.30575	20.47603	11.85069	6.77242
Clk2	2.24157	1.81900	0.36388	5.76910	2.53677	3.53796	2.52789	2.14398	2.23212	3.00012	5.41014	0.32264	2.38398	0.71709	2.01231	2.15127
Clk3	3.85231	7.65322	5.69913	6.37521	7.17394	6.37189	5.43945	9.85492	10.35608	5.23090	2.41101	0.60199	5.59241	0.01847	1.04510	0.92224
Clk4	2.00029	2.32352	0.52821	2.29419	1.81690	5.04198	1.63962	0.95454	7.37465	2.62460	2.74383	0.83112	1.03191	2.85239	5.25858	0.73504
Clmn	1.64421	0.00508	1.33311	1.32484	0.01874	0.04623	0.04204	0.69957	1.87889	2.36182	1.56816	0.14655	3.25196	0.99857	0.10802	1.01580
Clmp	0.00443	0.00780	0.32402	2.49398	0.00829	0.00815	0.00603	0.00000	2.87681	1.47037	1.05002	0.07503	1.48068	0.34578	0.98294	0.00608
Cln3	1.97832	1.89684	11.04574	1.92497	0.02036	7.25828	0.13325	13.38749	1.67334	11.75076	5.98418	0.59344	4.28281	3.86159	3.74900	1.89349
Cln5	6.17054	6.46679	0.59275	0.00000	0.22444	3.51018	5.11640	0.55605	0.00689	1.71181	1.83195	0.03833	0.00000	0.00270	3.16466	1.58411
Cln6	0.01943	0.02863	0.00000	0.72592	0.02221	1.26478	0.00683	0.00000	0.28361	0.63593	0.78478	0.07496	0.03954	0.17299	0.00000	0.00690
Cln8	2.34086	2.80742	5.84197	1.05212	0.06758	6.13578	5.26611	15.27977	1.28296	5.55635	6.09915	0.60817	10.89074	7.82099	5.66288	4.07320
Clns1a	17.60093	1.75040	8.31253	10.39131	15.34496	9.85526	6.81172	21.30102	15.61269	32.37813	15.20313	15.62479	16.35961	15.58792	7.19777	8.13719
Clock	1.18899	0.00592	1.52246	5.76250	0.00730	0.04206	1.29914	0.30688	1.80545	0.98957	0.00000	0.07876	0.02395	6.74986	6.63551	0.00458
Clp1	3.70190	6.87486	8.09113	9.59797	4.42721	4.69026	5.51148	12.44655	8.46770	6.86608	3.52570	7.64984	1.82862	9.33588	12.48899	1.66765
Clpb	3.72289	0.04303	5.50171	0.55065	2.39174	4.33777	0.01739	9.38441	0.39804	3.63600	3.03132	1.03617	11.22954	0.36552	0.88402	1.29147
Clpp	19.77703	6.39841	19.14880	7.16401	14.35152	7.01419	10.93589	20.91507	13.79246	18.33329	14.54317	5.61648	16.16996	0.00000	0.35572	8.89095
Clps	0.01264	0.00829	0.26177	0.00568	0.00874	0.00569	0.00767	0.00733	0.63641	0.00000	0.34861	0.00636	0.05229	0.00609	0.14671	0.00711
Clptm1	11.15292	0.06215	0.00000	4.94473	4.98922	6.48941	0.76348	17.70557	4.82169	11.64578	4.40530	0.51618	15.49561	0.00000	0.00000	4.87074
Clptm1l	12.18365	6.51323	8.01540	6.44578	5.12826	1.23697	7.02645	11.47202	2.12974	11.88292	7.04531	9.23141	8.29486	6.33836	6.58847	0.45033
Clpx	8.59667	5.90836	8.27195	16.03461	13.22398	7.55461	6.31628	22.15835	26.62006	38.42058	26.92534	19.78279	5.82143	30.46639	28.95019	2.56204
Clrn1	0.04931	0.00737	0.04560	0.02286	0.00792	0.00857	0.00727	0.01453	0.04755	0.07713	0.02408	0.27640	0.01183	0.04916	0.01340	0.01476
Clspn	4.08759	4.99707	5.75854	7.69838	0.00000	0.03124	1.09537	3.68566	15.08094	4.49604	2.34171	1.30205	5.54129	4.70934	11.41621	0.37955
Clstn1	7.25124	0.02807	7.88795	2.38119	0.02195	6.05630	2.43640	0.00000	3.02860	2.44315	1.88054	0.02048	4.46686	0.66176	0.50285	7.33856
Clstn3	0.03976	0.00725	0.76746	0.51023	0.02227	0.00871	0.00648	2.35545	0.77028	0.00000	0.32059	0.01680	0.58412	0.17242	0.26678	1.73283
Clta	19.46823	5.77952	10.19150	13.75802	13.60397	7.67500	4.35936	5.02857	12.74053	28.78490	23.43958	3.04054	23.14157	5.47049	5.48707	14.40958
Cltb	0.56861	0.00645	0.21171	8.00270	0.00652	1.63416	0.01649	2.44888	5.74374	4.91346	2.49706	0.43538	0.18850	1.54375	1.03161	0.19346
Cltc	16.40578	15.33054	9.23245	11.57511	16.40579	10.18408	9.98271	9.89903	12.73748	11.97648	7.83548	9.94619	12.40505	23.55912	9.37773	7.69169
Clu	0.21601	0.00788	0.00736	0.33388	0.03427	0.00673	0.00705	0.02118	0.27142	6.07131	0.00611	0.03094	0.01003	0.01317	0.12601	0.00736
Cluap1	1.00198	4.83675	2.07698	0.29468	2.70367	0.00583	2.21926	3.63214	0.06544	2.86003	3.61055	0.55044	2.41116	0.04203	2.20040	0.11329
Cluh	16.69352	10.05549	14.87855	4.67261	7.49430	10.19158	7.42202	17.63468	4.18338	13.69187	12.32647	1.37368	13.18426	3.36313	3.17622	5.47001
Clvs2	0.01337	0.00769	0.00631	1.82950	0.00905	0.00638	0.00703	0.00000	0.12971	0.27760	0.48611	0.01039	0.00828	0.00170	1.77778	0.01488
Clybl	4.66594	0.00000	0.00000	4.27434	0.37510	4.50592	0.00000	0.54930	2.98358	3.83840	2.71632	5.01680	1.00118	4.15990	3.00187	0.00000
Cmah	7.40838	6.38292	8.91693	17.61257	1.77835	1.51409	8.09528	3.42327	16.13330	5.89698	2.96929	12.01178	2.96814	16.28606	8.37550	5.29299
Cmas	10.18409	2.55850	2.98546	6.64468	1.80933	0.00000	5.07189	1.12245	4.47603	2.42430	1.26575	0.99831	2.98485	0.00000	0.21018	1.24475
Cmbl	1.96843	3.02921	6.70078	0.00000	0.01038	0.16240	0.17472	3.73616	0.00771	0.78759	0.78983	0.00000	2.55144	0.01741	0.69855	4.16851
Cmc1	2.96059	0.13282	0.30418	0.15503	0.61165	0.02032	0.10298	0.34656	0.83225	0.64724	0.33909	0.00595	0.27443	0.25193	0.83337	0.49785
Cmc2	8.28928	1.77280	5.89829	1.29377	3.22026	2.06533	1.89506	5.00165	4.03023	3.53262	5.43354	0.65433	7.50013	0.33965	1.31564	2.18792

**Cmc4**	0.00652	0.00701	0.24643	0.97338	1.58457	0.05111	0.00716	0.14780	0.00000	0.23952	0.34034	0.01294	0.00000	0.00437	0.04233	1.01261
**Cmip**	3.61411	1.22302	2.02189	0.12592	6.11995	6.08258	0.00000	9.79775	0.00000	2.31302	0.80123	0.71357	6.50578	0.43587	0.45519	0.94325
**Cmklr1**	0.05993	0.00798	0.05736	1.54978	0.00829	0.00794	0.13940	0.02066	0.00000	0.01012	0.01238	0.00757	0.02593	0.01763	0.17568	0.00694
**Cmpk1**	4.26963	4.14860	2.26186	5.63762	5.70224	4.09303	1.23602	2.56841	4.14317	5.63076	3.35286	3.77615	3.82667	5.94705	5.88978	3.33239
**Cmpk2**	0.01361	0.00777	0.01118	3.33101	0.00944	0.00606	0.00722	0.01080	0.00000	0.00644	0.01009	0.00500	0.01280	0.00175	1.43071	0.00675
**Cmss1**	5.59463	2.35305	4.76203	4.22678	0.01811	4.14474	0.00000	3.12479	4.70229	7.11287	2.70447	7.86336	6.53089	2.99227	0.44904	2.36507
**Cmtm3**	0.01244	0.00785	0.01156	0.00633	0.00877	0.00651	0.06750	0.01014	0.01156	1.62261	0.01056	0.00355	0.02278	0.01194	0.00760	0.03600
**Cmtm4**	3.50449	0.01978	6.71869	14.72895	2.29574	3.69473	2.64100	8.16335	16.25745	11.45962	7.40589	1.73529	3.66993	2.99313	5.04733	0.02971
**Cmtm6**	3.62330	5.18530	7.81293	3.40951	1.03300	5.62665	10.48861	10.43020	9.43919	6.61132	6.18637	4.58544	7.30259	1.42736	3.72182	4.78816
**Cmtm7**	11.02215	1.86285	6.47797	0.00000	9.74632	8.95646	4.92089	6.99440	0.75239	11.73151	4.10148	3.58782	12.87620	0.48347	0.00000	8.33829
**Cmtm8**	0.00696	0.00841	0.01124	0.74904	0.00808	0.00653	0.00801	0.05423	0.02782	1.35059	0.01067	0.00512	0.00719	0.01247	0.00993	0.66406
**Cmtr1**	0.01125	0.00775	0.03278	0.01065	0.00969	0.00715	0.04751	0.09676	0.01225	0.01316	0.01314	0.00740	0.01135	0.01240	0.00872	0.00747
**Cmtr2**	0.00634	0.00709	0.70441	2.22402	0.00936	0.00541	2.35916	0.43275	0.41844	0.00000	0.03096	0.02758	0.07587	0.00355	0.31174	0.00644
**Cmya5**	0.01377	0.00790	0.01238	5.80639	0.00936	0.00604	0.00725	0.01278	0.00000	0.00845	0.01264	0.00709	0.01264	0.00188	0.24266	0.00638
**Cnbd2**	1.16220	1.75223	4.47323	9.03729	1.41251	2.67126	4.38608	2.73731	6.09977	13.71490	5.24977	5.52673	3.04475	3.97774	6.43663	0.43454
**Cnbp**	33.24468	25.35375	34.61772	22.69148	26.07511	15.67076	24.92979	42.46917	28.97080	46.53930	39.76008	19.17530	35.77401	31.85373	29.31387	20.74959
**Cndp1**	0.01179	0.00778	1.69832	1.35424	0.00631	0.00800	0.00720	0.01033	0.49312	0.29353	0.00691	0.00580	0.00734	0.00913	0.00000	0.00651
**Cndp2**	20.01179	20.16913	23.65534	3.29803	12.28457	19.02927	12.04791	25.14806	8.34641	17.36784	18.85104	12.49051	25.81700	11.32827	8.92969	13.23375
**Cnep1r1**	0.55094	2.17386	1.14620	7.73677	0.91022	0.90204	0.20712	1.65896	8.96434	0.88954	1.14362	4.66289	0.41938	7.15748	4.74040	0.22423
**Cnga1**	0.01361	0.00780	0.01147	0.88629	0.00923	0.00668	0.00747	0.01220	0.00000	0.22526	0.01070	0.00736	0.01286	0.00967	0.44910	0.00728
**Cnga3**	0.65573	0.01698	0.23731	0.03152	0.07937	0.01538	0.00668	0.00957	0.12272	0.02311	0.14591	0.00578	0.02228	0.18287	0.02847	0.00645
**Cnga4**	0.02808	0.00786	0.00865	0.51777	0.20918	0.00671	0.00738	0.01100	0.00348	0.00559	0.02422	0.00709	0.01140	0.01756	0.24071	0.00735
**Cngb1**	0.01305	0.00786	0.01032	2.89602	0.00910	0.00626	0.00743	0.01110	4.11206	0.20797	0.00000	0.01068	0.01333	0.00000	0.46460	0.00668
**Cnih1**	12.22330	8.52422	12.46237	11.09746	11.15299	9.71964	5.26686	7.50882	11.02187	11.07568	3.93332	7.42399	9.35466	5.31778	2.88627	2.61102
**Cnih2**	0.01375	0.00805	0.01225	0.01061	0.00932	0.00666	0.00754	0.01390	0.01235	0.01531	0.01310	0.00811	0.01270	0.01234	0.01062	0.00741
**Cnih4**	7.29439	1.98873	7.69119	3.23632	2.12679	4.81527	2.63518	2.57848	4.28752	5.63401	9.87283	0.59044	4.77499	2.98077	3.99321	2.27906
**Cnksr1**	0.01745	0.00752	0.47546	0.01011	0.01969	0.00636	0.00622	0.00000	0.36535	0.29937	2.75677	0.02119	0.13628	0.00224	0.18155	0.00645
**Cnksr3**	0.36272	1.70899	0.13428	6.37344	3.90218	0.00762	0.70724	0.73062	3.87354	0.42400	0.59535	1.40933	1.27089	0.06022	0.48435	2.03823
**Cnn1**	0.01393	0.00807	0.00958	4.04971	0.00912	0.00653	0.00730	0.00686	0.00000	0.03681	0.26007	0.03740	0.00982	0.00378	0.00000	0.00684
**Cnn2**	0.29547	0.01942	1.15661	0.48447	0.08657	13.97512	0.04395	4.37667	0.33773	5.45284	1.27476	0.04931	5.77655	0.04135	0.20059	9.19361
**Cnn3**	12.27156	10.23804	14.81739	10.40674	9.93605	13.17043	10.57731	15.99314	17.60297	4.54640	6.89034	7.52372	22.12210	5.74875	2.10180	7.45228
**Cnnm1**	0.01347	0.00772	0.00661	0.00456	0.00911	0.00699	0.00698	0.00000	0.34941	0.51380	0.64279	0.04717	0.00686	0.11029	0.00000	0.00729
**Cnnm2**	5.40366	1.52416	4.00099	5.38356	0.00813	0.01492	2.27426	5.86327	11.18160	5.42590	2.17464	0.14956	6.06561	0.00000	5.24342	0.91398
**Cnnm3**	0.01224	0.00629	3.47656	7.15799	1.03926	0.02006	0.01613	2.26822	3.82876	1.67064	0.75130	0.30163	1.66896	0.06680	2.94636	0.01111
**Cnnm4**	0.02505	1.85106	4.46974	3.31077	0.01620	1.68157	0.68389	10.59950	2.61452	5.65860	10.43897	5.99109	5.57226	3.48949	5.97210	0.49378
**Cnot1**	16.51356	15.74564	11.77436	12.46431	12.52160	7.87461	11.72378	26.51271	10.50446	26.93715	15.12870	18.55935	11.13656	29.33825	25.42861	8.09071
**Cnot10**	7.04741	8.26511	6.98225	3.37857	10.51250	9.33264	6.74403	7.45069	7.18038	10.43185	7.21311	1.40268	3.11419	0.68404	5.61608	3.29151
**Cnot11**	1.25684	2.32053	0.71567	4.96761	2.08544	2.69232	0.12824	3.05057	1.77490	1.20505	7.51294	0.90743	0.00000	4.23786	1.75879	0.02398
**Cnot2**	9.52792	11.57932	12.85779	9.01444	9.17298	2.98646	2.58440	14.25072	5.10251	15.02440	7.69266	6.05872	1.27734	21.94619	13.53437	7.14068
**Cnot3**	1.98602	1.33504	0.43889	0.16484	2.15861	0.26085	1.66130	4.45799	0.23307	1.93451	0.00000	0.07913	1.51530	0.00451	0.00944	1.70802
**Cnot4**	1.43753	2.11068	3.67178	2.79113	0.21517	1.67430	0.32187	3.95767	1.96872	4.52846	2.30951	1.90267	1.07402	4.48317	4.42805	0.79202
**Cnot6**	2.05173	2.24580	3.76963	5.59130	2.71442	0.00855	6.72373	1.81738	2.71527	5.74439	4.24627	8.06227	0.82289	9.59987	8.46598	0.36171
**Cnot6l**	1.96887	2.88288	1.41512	9.00221	0.01004	0.58051	1.11303	0.00000	2.29157	1.95407	0.44656	0.54833	2.92051	5.22655	3.40564	0.41244
**Cnot7**	1.67425	3.32836	1.60849	18.18728	1.32279	0.17381	4.05578	2.15012	9.58676	7.67345	5.83670	13.88191	1.34231	16.75395	8.10309	0.01140
**Cnot8**	12.64838	2.67410	7.33075	17.87529	7.45666	10.84906	3.65282	12.78811	18.59807	19.86117	16.17631	9.55398	10.21893	6.26976	20.18820	1.60932
**Cnot9**	9.21379	2.49023	12.45283	3.05854	5.52984	6.77653	9.28456	6.98877	2.34776	13.69096	1.10579	3.20253	6.99112	11.01606	6.26261	3.26184
**Cnp**	1.16873	0.00619	0.00000	0.15264	0.00539	0.16477	1.12686	1.99678	0.71619	4.83672	1.60545	0.07267	0.28043	0.01674	0.02394	0.01258
**Cnppd1**	5.71653	0.82930	7.89214	21.87473	0.01461	3.07415	0.45287	12.20131	25.28324	18.44940	6.92612	1.35762	5.83566	1.92885	5.05136	0.00983
**Cnpy1**	0.03461	0.00726	0.00000	1.12445	0.00730	0.00661	0.11484	0.00000	0.28560	0.00000	0.56447	0.48868	0.00375	0.00352	1.50511	1.47043
**Cnpy2**	14.95463	8.37061	11.06678	0.26929	11.31152	3.01386	3.02241	3.54932	0.00000	1.49584	4.16755	0.10300	3.70643	0.04286	0.63812	7.29649
**Cnpy3**	2.38597	3.78488	6.25313	1.51122	0.00000	0.00991	1.63580	2.87748	4.02269	3.72850	2.23135	0.08899	1.92847	0.01239	0.77025	2.73661
**Cnpy4**	0.70534	2.89543	0.00000	6.78869	0.37845	2.12634	0.06268	0.63455	3.97173	0.40793	1.08022	0.24466	0.49769	4.26631	4.88939	0.19932
**Cnr2**	0.09003	0.01817	0.14807	0.30043	0.02564	0.09561	0.03916	0.02866	0.32505	0.11653	0.40403	0.20412	0.04623	0.67667	0.47167	0.04358
**Cnrip1**	0.01374	0.00763	0.00940	0.29656	0.00924	0.00656	0.00730	0.01088	0.00712	0.22460	0.00946	0.18059	0.01198	0.00469	0.43961	0.00721
**Cnst**	3.75826	2.98808	2.25215	10.10213	0.07801	4.49091	0.60977	1.06212	3.05624	4.64113	0.35671	0.24614	1.72355	3.18302	8.59189	0.67287
**Cntd1**	0.44814	0.00739	0.00663	0.01056	0.00747	0.00747	0.00750	0.01418	0.01234	0.01408	0.01453	0.00775	0.05306	0.01241	0.01065	0.00717
**Cntf**	0.01197	0.00777	0.01180	0.01102	0.03681	0.00667	0.00752	0.01350	0.01207	0.01368	0.01261	0.00790	0.01251	0.01742	0.03252	0.00738
**Cntln**	0.01401	0.00623	0.00276	4.38357	0.00878	0.00490	0.00640	0.00745	1.97496	1.02555	0.00000	4.24332	0.01178	2.05295	2.70167	0.00617
**Cntn1**	0.01374	0.00782	0.01163	1.13120	0.00942	0.00636	0.00733	0.01137	0.00000	0.00583	0.01142	0.00663	0.01279	0.00683	0.71118	0.00716
**Cntn2**	0.04979	0.01852	0.14167	0.50716	0.05263	0.01928	0.04773	0.00680	0.13918	0.08786	0.31422	0.02354	0.10624	0.14984	0.14418	0.04363
**Cntn4**	0.01372	0.00766	0.01168	0.61664	0.00936	0.00644	0.00740	0.01189	0.48363	0.00435	0.00838	0.00609	0.01279	0.01007	0.40266	0.00720
**Cntn6**	0.01375	0.00779	0.01185	1.01196	0.00937	0.00655	0.00747	0.01310	0.28101	0.00935	0.00988	0.00725	0.01283	0.01922	0.07502	0.00722

**Cntnap1**	0.01375	0.00785	0.01123	5.48844	0.00925	0.00604	0.00720	0.01128	0.70120	0.03278	0.02671	0.02842	0.01199	0.00337	0.22532	0.00625
**Cntrl**	0.02400	0.00844	0.00000	2.68560	0.00839	0.04452	0.00767	0.22679	0.27797	0.58164	0.29103	0.02644	0.35868	0.02764	0.23568	0.00700
**Cntrob**	0.51753	2.00320	2.39022	1.90205	1.55940	1.31488	2.83530	1.45546	0.36087	0.00893	0.00236	0.03729	2.70722	0.00616	0.04179	0.01030
**Coa3**	10.49524	7.94164	9.00219	0.30225	5.17067	1.69628	4.65446	5.52023	0.19262	4.40550	3.98835	0.29086	9.20290	0.00767	0.33661	3.01743
**Coa4**	6.83360	5.31381	7.81417	0.00000	0.90293	5.70597	4.36557	3.30721	0.73421	2.55630	1.64271	2.00042	1.78555	0.15426	0.00000	3.05144
**Coa5**	7.08898	4.60458	13.13353	0.81909	4.20919	6.92944	9.42099	8.04711	0.51968	7.50843	3.99793	1.52435	7.38863	5.53011	5.15704	2.26749
**Coa6**	3.26242	0.00057	0.00000	3.60690	3.99683	0.80837	0.14718	3.49855	3.61501	3.66720	1.65710	0.16742	1.50857	0.00827	0.06353	1.41550
**Coa7**	8.49834	4.64793	6.85156	3.03249	4.93330	0.00896	3.73214	5.84369	1.06598	10.53337	5.16767	0.33751	6.73673	0.81789	8.11176	2.24877
**Coasy**	11.59501	8.61166	6.06892	0.50214	6.15241	4.51246	1.45096	5.60122	0.02507	3.49573	2.52208	0.08624	6.49371	0.20465	0.00000	3.06917
**Cobl**	0.01737	0.00776	0.01015	2.56659	0.00898	0.00638	0.00740	0.01195	0.28312	0.12691	0.00633	0.00459	0.01337	0.00446	1.62363	0.00694
**Cobll1**	0.01041	0.00842	0.00233	1.37027	0.00956	0.00698	0.00788	0.12778	0.07698	0.00000	0.60801	0.09248	0.00560	0.00339	1.29590	0.00687
**Coch**	0.01849	0.00837	0.01186	0.56030	0.01054	0.00671	0.00755	0.01413	0.04085	0.02454	0.01360	0.00864	0.01289	0.01082	0.00532	0.00737
**Cog1**	0.66483	3.11752	0.62725	0.10955	0.91371	0.02444	0.31280	4.67255	0.01857	0.72729	2.13443	0.06193	2.74430	0.03472	1.93813	2.15507
**Cog2**	4.00810	5.92188	4.14059	1.26473	9.52212	3.39777	1.77660	3.78875	2.72784	5.12342	3.61535	0.28128	1.24372	0.50196	2.10837	1.47493
**Cog3**	0.00000	0.00701	0.29345	2.78183	0.69358	0.00562	0.20395	1.58738	0.63722	0.78803	0.51129	0.21766	0.20521	0.30727	4.63353	2.96400
**Cog4**	9.70462	9.14774	8.57893	20.34117	0.61599	4.79658	7.25069	7.31467	20.13174	11.52001	9.20927	1.79710	7.12154	6.58599	15.61993	4.93534
**Cog5**	3.32462	2.64565	0.80688	0.40735	0.01141	4.08938	0.05223	0.77471	0.29756	0.96111	2.87919	0.04581	0.00000	0.92687	1.83840	0.00000
**Cog6**	1.72805	0.64315	0.00000	0.50146	0.00780	0.00367	3.69036	0.00000	0.00581	2.39535	1.64294	5.95606	2.72182	0.03534	0.30268	0.89082
**Cog7**	6.42390	6.02572	2.87050	2.34459	0.75211	2.87516	3.73671	3.81417	1.75696	3.22214	3.55657	0.01792	1.55434	0.05540	0.09395	2.02736
**Cog8**	0.00000	0.00688	0.04180	0.39301	1.39470	1.26325	0.26984	1.39295	0.27620	2.36043	0.01666	0.04295	1.31281	0.00965	0.00000	5.67205
**Coil**	5.15725	7.80943	13.40967	4.24961	5.79028	6.66224	7.62173	3.29219	7.69595	4.64241	3.64564	3.16431	13.68597	0.00000	0.00000	5.27627
**Col10a1**	0.05574	0.00824	0.20016	0.00350	0.01337	0.44371	0.00732	0.16948	0.51883	0.00000	0.14957	0.23064	0.00626	0.63012	0.00000	0.00722
**Col11a2**	0.02000	0.03976	0.01143	0.00611	0.00877	0.00670	0.00752	0.01317	0.09328	0.01463	0.01218	0.00795	0.01267	0.01239	0.00930	0.00737
**Col12a1**	0.01377	0.00761	0.00775	0.01060	0.00921	0.00682	0.00726	0.01115	0.01201	0.03313	0.00754	0.73668	0.01265	0.01078	0.00544	0.00722
**Col13a1**	0.15182	0.00877	0.00786	0.76831	0.01709	0.00689	0.00740	0.01298	0.03745	0.01479	0.01289	0.00832	0.01237	0.01091	0.00426	0.02237
**Col16a1**	0.01038	0.00764	0.00955	0.00895	0.00940	0.00681	0.00715	0.16231	0.01193	0.31076	0.02485	0.00605	0.00775	0.01140	0.00794	0.00781
**Col18a1**	0.01123	0.00759	0.00824	0.00721	0.00943	0.00690	0.00712	0.42016	0.07648	0.03408	0.00985	0.01572	0.00616	0.01287	0.00444	0.00758
**Col19a1**	0.01372	0.00824	0.01236	0.29755	0.00924	0.00655	0.00752	0.01293	0.00564	0.12559	0.01204	0.00784	0.01177	0.01043	0.00643	0.00713
**Col1a1**	0.01248	0.00696	0.00640	0.01744	0.00872	0.00660	0.00689	0.00962	0.01157	1.54639	0.00000	1.86197	0.01142	0.01059	0.00000	0.00755
**Col1a2**	0.01303	0.00730	0.00717	0.00650	0.00893	0.00669	0.00702	0.00947	0.01100	1.22595	0.00535	0.90225	0.01101	0.03422	0.00000	0.00730
**Col20a1**	0.00776	0.00782	0.01154	0.84550	0.00910	0.00696	0.00717	0.03494	0.00000	0.17020	0.01418	0.09382	0.50438	0.01161	0.00433	0.35252
**Col22a1**	0.01360	0.00829	0.01199	1.67520	0.00929	0.00645	0.00748	0.01385	0.98475	0.00965	0.00712	0.00677	0.01288	0.01062	0.00288	0.00707
**Col23a1**	0.01346	0.00827	0.01195	0.03485	0.00922	0.00689	0.00775	0.01789	0.01237	0.14692	0.01255	0.00910	0.01271	0.01171	0.01133	0.00741
**Col24a1**	0.01376	0.00798	0.01213	0.45052	0.00932	0.00662	0.00752	0.01375	0.05395	0.01444	0.01263	0.00801	0.01272	0.01442	0.01293	0.00734
**Col26a1**	0.74452	0.23446	0.00000	0.18278	0.54866	1.84379	0.00641	0.00537	0.00803	0.04724	0.02341	0.04021	0.03830	0.54325	0.04952	1.71933
**Col2a1**	0.01383	0.00797	0.01117	0.11621	0.00930	0.00669	0.00745	0.01268	0.12190	0.01304	0.01274	0.06866	0.01284	0.01187	0.00561	0.00740
**Col3a1**	0.01357	0.00775	0.00998	0.00879	0.00925	0.00670	0.00734	0.01191	0.01207	0.45622	0.01110	0.15876	0.01197	0.01202	0.00504	0.00747
**Col4a1**	9.96599	0.01158	4.67291	0.21457	7.96170	4.04079	1.94310	0.36988	0.27862	0.00000	1.53356	0.45037	3.52000	0.00353	0.00000	10.85144
**Col4a2**	12.22565	1.72598	5.54294	0.04029	5.48241	5.57596	4.58600	2.55419	0.15412	0.00000	1.35348	0.34566	10.12848	0.57402	0.00000	11.88478
**Col4a3bp**	1.74572	0.53094	2.75177	13.68021	3.79480	2.18832	1.85701	2.45966	10.71010	0.30752	3.39366	12.46011	1.27590	6.21643	7.54314	0.54832
**Col5a1**	0.44707	0.00767	0.00262	0.01072	0.01624	0.00787	0.00682	0.00855	0.01212	0.00859	0.00953	0.48319	0.01265	0.01113	0.00631	0.00725
**Col5a2**	0.00888	0.00824	0.00747	0.01103	0.00960	0.00689	0.00813	0.53217	0.01144	0.00315	0.00521	0.57335	0.00595	0.01370	0.00000	0.00749
**Col5a3**	0.01414	0.00727	0.54156	1.54836	0.02178	0.02314	0.00368	0.01043	0.72596	0.54757	7.48398	0.09788	1.68872	0.01257	0.25782	0.00512
**Col6a1**	0.01382	0.00781	0.01086	0.01000	0.00921	0.00675	0.00734	0.01151	0.01220	0.14088	0.01265	0.08296	0.01293	0.01134	0.00923	0.00776
**Col6a2**	0.01370	0.00803	0.01170	0.00950	0.00920	0.00658	0.00744	0.01175	0.01232	0.25800	0.01161	0.03726	0.01157	0.01143	0.00909	0.00720
**Col6a3**	0.01366	0.00762	0.00858	1.41557	0.00926	0.00649	0.00719	0.01003	1.90712	0.00000	0.00000	0.36397	0.01329	0.01650	0.12872	0.00697
**Col6a4**	0.00786	0.00785	0.00000	0.01144	0.01117	0.00766	0.00745	2.27866	0.32778	5.26550	0.85177	0.00000	0.00502	0.81439	0.70445	0.00727
**Col6a5**	0.00748	0.00852	1.21649	0.37500	0.00860	0.00581	0.00843	0.41860	0.06038	0.00000	0.00713	0.00736	0.00452	0.03350	0.31857	0.00696
**Col6a6**	0.01379	0.00827	0.01004	0.03526	0.00911	0.00690	0.00750	0.00730	0.09566	0.09531	0.21985	0.00793	0.01041	0.00616	0.26737	0.00733
**Col7a1**	2.18020	0.00472	0.22670	0.00190	1.46537	0.00641	0.00528	0.00429	0.85571	0.00000	0.00621	0.03477	0.95060	0.01796	0.00127	0.01157
**Col8a2**	0.01359	0.00775	0.00833	0.18787	0.00915	0.00660	0.00726	0.00636	0.59054	0.12413	0.13934	0.04329	0.01125	0.06464	0.95822	0.00755
**Col9a2**	0.27912	0.00722	0.11772	0.00316	0.04763	0.00559	0.48363	2.08341	0.00856	0.10695	5.79438	0.00000	2.07855	0.46357	0.18744	0.00521
**Col9a3**	0.01376	0.00820	0.01212	0.79972	0.00930	0.00657	0.00750	0.01350	0.00000	0.01448	0.03101	0.00832	0.01226	0.01064	0.00361	0.00727
**Colec12**	0.62271	0.21288	1.33667	24.91075	0.06710	0.18635	0.66787	0.30730	16.55414	4.86957	1.00010	1.25404	0.53806	10.24194	15.12690	0.21860
**Colgalt1**	5.10321	4.48566	3.43048	5.51970	0.40090	0.01518	1.22678	1.77637	3.00958	6.15910	3.41840	0.24044	2.99870	3.09672	6.28338	6.71445
**Colgalt2**	0.01317	0.00797	0.00985	2.97698	0.00928	0.00621	0.00722	0.00945	2.62133	0.00000	0.01713	0.00000	0.01272	0.28483	0.10532	0.00664
**Colq**	0.01322	0.00816	0.01039	0.23314	0.00904	0.01321	0.00756	0.01277	1.03050	0.53604	0.02125	0.00549	0.01203	0.00480	0.47022	0.00729
**Commd1**	9.72703	2.68974	2.77725	0.56393	8.03143	3.25858	2.19020	2.79102	0.40289	2.86134	1.98330	0.16377	2.21351	1.07582	0.14712	6.25798
**Commd10**	11.97475	2.25057	7.42835	0.00000	7.64223	3.24785	0.96628	3.20484	0.58052	4.97277	2.25102	0.75076	6.90504	3.39738	2.88386	2.77404
**Commd2**	11.96751	6.42275	7.17010	8.76967	8.27815	7.13892	4.94830	8.97678	9.58385	13.59431	6.91108	1.54185	11.54824	2.31657	3.22802	7.93682
**Commd3**	11.76436	5.69222	6.76107	3.65554	4.96265	7.59496	3.01947	1.55397	0.00000	3.32055	2.03966	0.31454	8.91951	0.00000	0.00000	3.66160
**Commd4**	16.72419	7.04833	5.29125	2.22833	2.75422	3.31761	7.59433	3.85885	0.34607	4.97348	4.90491	0.31090	4.53194	0.02843	0.36843	7.02084

**Commd5**	10.99913	0.42196	1.79556	1.35772	4.51082	1.17956	5.07481	19.64299	11.53198	17.78997	8.58891	0.77312	14.46063	0.47164	1.79636	6.77553
**Commd6**	5.32081	0.57793	3.16561	3.67215	2.69703	2.05138	0.18278	2.49573	0.61612	3.92599	1.89913	0.33166	2.10055	1.23664	2.66398	3.55384
**Commd7**	2.81884	1.03186	0.91531	9.77155	0.42249	2.49512	0.30615	2.73418	10.94159	9.79453	8.85189	5.60591	3.24246	1.66911	4.43682	2.57836
**Commd8**	6.80824	10.87614	8.43549	0.00000	6.67098	5.92316	7.04838	6.19540	1.13102	4.55258	2.01810	0.31886	5.66609	2.65685	1.48164	5.07277
**Commd9**	5.05444	3.78122	10.20090	0.28589	3.86512	0.35106	7.35354	4.37188	0.70503	6.07875	2.36107	0.31935	4.72492	0.07104	0.34840	0.00000
**Comp**	0.01140	0.01974	0.46374	0.02554	0.01167	0.00602	0.00760	1.66850	0.38704	0.03281	0.00000	0.20626	0.01498	0.00790	0.06109	0.00757
**Comt**	5.03179	0.62379	1.92503	7.76087	2.63828	2.67722	0.47479	2.89245	12.23368	1.87511	10.63665	1.22574	2.72343	4.94091	3.60293	0.66462
**Comtd1**	5.62805	0.01112	0.02302	0.01229	0.00785	0.01038	0.00046	0.21207	0.84701	0.03366	1.27288	0.00000	1.22537	0.00906	0.00000	1.55410
**Copa**	7.97783	3.06908	9.75525	7.51228	7.74769	10.22134	2.51819	5.08488	6.01023	12.33765	8.76985	13.54706	8.54002	11.02188	7.90516	9.48208
**Copb1**	2.23522	8.27560	3.44481	1.55965	4.72802	1.56539	0.06474	2.39634	1.94013	2.00879	0.52946	3.04117	1.10902	1.61695	0.91570	0.21633
**Copb2**	12.76603	12.58202	13.31452	6.84797	17.68732	5.55028	10.74035	12.63356	5.47346	14.70021	6.78657	4.80369	8.79543	12.27209	10.88810	6.99523
**Cope**	16.63204	10.16972	16.11112	2.47601	12.96441	3.97669	9.04344	15.92356	3.14677	18.51455	9.10587	1.04071	18.98867	0.08889	0.00000	7.62390
**Copg1**	13.54399	12.43043	11.07654	2.62839	11.57531	8.87039	7.67719	10.01902	0.81178	8.27843	5.04472	3.06521	9.80546	4.98474	4.36346	6.21262
**Copg2**	3.56388	0.19217	1.61139	2.70492	0.00000	0.26462	0.00418	0.00000	0.26039	0.00000	0.67578	0.01042	0.03380	0.00656	0.06918	0.00957
**Coprs**	3.01585	0.65750	0.90595	1.72957	0.01498	0.00296	0.93917	1.86885	1.94515	6.73071	6.53409	0.60114	5.32603	0.34105	0.00000	4.68445
**Cops2**	12.54927	9.72510	13.82709	5.51502	12.96621	0.35634	6.08406	10.12780	9.53725	14.65601	10.25869	11.90905	11.00294	23.34994	13.80964	4.47744
**Cops3**	12.69641	11.41997	13.36310	1.07803	5.12059	10.49761	7.23497	13.65686	1.67205	10.14295	2.36370	10.50368	9.47201	4.09472	9.54496	8.32503
**Cops4**	22.98032	16.08729	15.61882	7.31890	17.68646	6.22682	9.12139	16.54825	11.20120	25.19038	16.72286	18.01830	22.11781	5.52389	16.90904	10.20455
**Cops5**	24.83277	15.58041	22.21875	0.93892	17.00248	13.34884	14.64121	19.47619	4.17835	14.67865	13.93791	10.60372	17.76105	15.76980	6.78065	14.63444
**Cops6**	17.63522	8.27594	12.07431	6.32957	11.96746	4.07516	6.07749	9.41109	19.53711	14.49221	16.68382	9.78208	12.05947	6.47285	7.97927	6.13788
**Cops7a**	18.88998	8.77985	11.23845	12.78502	19.19116	12.73443	8.13256	26.81982	16.76358	12.66867	15.78605	2.94448	13.71729	6.87765	12.40598	9.33094
**Cops7b**	0.69324	0.02658	0.11707	0.56344	3.05697	0.02277	2.60025	6.51109	0.46954	8.09838	1.56957	0.17400	4.76967	0.55226	1.26475	0.64220
**Cops8**	13.92547	7.63348	11.82866	7.85647	2.29695	6.94021	9.18723	13.12489	6.16511	12.10107	8.72388	11.23543	12.00908	2.90595	11.02691	6.03725
**Copz1**	20.16456	15.47970	25.03487	3.89080	9.10442	8.36157	12.31750	19.82528	4.94999	17.23888	12.70079	8.02956	18.82694	17.97955	9.57118	10.76073
**Copz2**	0.01132	0.00779	0.09540	0.62810	0.00928	0.00667	0.00728	0.04613	0.11863	0.13364	0.01111	0.04582	0.01730	0.00877	0.06200	0.00735
**Coq10a**	0.12935	0.00853	0.00788	1.64886	0.01586	0.00693	0.00725	0.00631	0.05678	0.07113	0.19671	0.03973	0.39535	0.24413	1.02556	0.00720
**Coq10b**	1.82274	0.00000	0.91262	3.54649	3.13490	0.01560	3.62387	5.62385	2.17213	4.25570	1.79147	2.46568	2.71101	0.00000	2.00339	2.07689
**Coq2**	2.60455	2.10632	1.83843	2.74171	0.01096	4.55976	0.43296	1.76714	0.64533	0.53279	0.64144	1.61148	2.81319	0.00000	1.20918	1.95251
**Coq3**	3.91803	5.00984	7.10201	4.57060	7.36061	1.27712	8.68645	3.71578	2.63214	7.62945	7.38876	0.18961	4.57763	4.94807	5.41375	4.14638
**Coq4**	5.21450	0.06090	2.17012	8.73058	4.95024	2.70085	0.17219	3.86054	15.02867	12.27922	4.28821	0.07662	4.54212	0.00000	0.54041	3.95257
**Coq5**	3.19521	1.56250	3.54742	1.11447	5.05438	6.66447	5.06862	2.21045	1.24222	5.74992	4.96348	0.49510	5.53967	0.70864	1.35563	2.18891
**Coq6**	9.51334	7.60679	6.70860	2.67583	3.41346	7.94936	3.62738	13.17692	6.57753	7.52842	4.83873	0.65943	9.40941	0.07881	1.49397	2.89313
**Coq7**	11.56072	7.35499	9.85718	1.94105	1.70804	7.85199	2.45775	6.62984	1.19704	8.15145	7.83183	0.43290	10.03307	0.76553	0.46017	8.19705
**Coq8a**	0.02337	0.00736	0.00318	0.70470	0.00918	0.00679	0.74447	0.02597	0.00000	0.00000	0.19600	0.00394	1.65403	0.00568	0.78879	0.01403
**Coq8b**	2.07602	0.00450	0.10675	1.44614	0.00303	2.91007	0.07677	4.09014	0.15749	2.45106	1.50612	0.07166	1.25903	0.00000	0.52349	2.40645
**Coq9**	5.80866	6.80962	4.57416	1.95338	4.46428	1.61454	0.68555	5.75488	0.30462	3.86344	1.99367	0.20791	6.24996	3.03338	4.01586	5.14402
**Coro1a**	0.01368	0.00746	0.02393	0.22704	0.00860	0.09055	0.00705	0.00309	0.00800	3.30551	0.22554	0.18846	0.02852	0.00791	0.02138	0.00735
**Coro1b**	11.91347	5.11613	8.69580	2.29810	4.48925	6.03620	8.79053	4.93721	2.97936	5.46136	2.72424	0.13475	7.00539	0.01267	0.00000	7.29927
**Coro1c**	16.34384	7.96528	19.94434	3.29651	10.34304	4.41856	8.11714	21.18275	4.83582	12.22491	8.78686	1.23991	15.44932	0.18420	0.22117	11.94528
**Coro2a**	1.28108	0.00657	0.67005	0.00275	0.03416	0.60906	1.06025	1.60375	0.04375	0.20546	1.07337	0.30275	0.87896	0.10232	0.57206	0.00550
**Coro2b**	1.42093	0.00448	0.65118	0.96071	3.39836	0.00749	0.00598	0.02975	0.27141	0.02030	0.00884	0.02207	0.00699	0.00976	0.00000	0.00525
**Coro7**	2.78275	0.44136	0.65044	0.33981	0.00000	0.91374	0.00341	2.38001	0.00524	0.00000	0.26760	0.19402	2.47586	0.00460	0.07558	6.11011
**Cort**	0.01320	0.00787	0.00901	0.20057	0.01831	0.00680	0.00736	0.00288	0.37802	0.12965	0.39195	0.01483	0.00884	0.00795	0.00000	0.00752
**Cotl1**	4.90087	0.03575	0.54316	3.13486	0.02106	0.00000	0.00213	1.75614	1.82957	1.29763	2.79186	0.14660	1.68987	0.00000	0.00000	0.63507
**Cox10**	1.14945	0.09641	5.86354	6.96750	0.14172	2.68792	3.56867	6.04035	2.00286	5.26349	2.84964	0.22019	3.43011	0.51149	1.34658	3.03040
**Cox11**	1.49080	0.03931	0.00000	2.40092	0.31960	0.00457	0.56446	0.19515	2.84744	2.65544	2.12422	1.15864	1.85744	1.46046	1.11835	0.01906
**Cox14**	10.21008	0.08917	1.45749	6.74409	7.43822	3.09000	0.24492	2.70398	7.72962	5.76319	5.56947	0.24693	6.99622	0.02520	0.15099	2.05888
**Cox15**	0.85260	2.47413	1.83487	0.90212	0.02863	0.37171	0.02514	2.56853	0.37934	1.01354	1.54503	2.94099	1.75292	3.58755	3.36429	2.49379
**Cox16**	1.14888	0.01602	1.03433	0.08690	0.65588	0.31806	0.20774	0.38928	0.02726	1.13385	0.63679	0.07705	0.26500	0.00800	0.00000	1.19749
**Cox17**	8.02627	0.18859	2.66996	0.30582	4.92831	2.22708	0.42799	0.32050	1.89887	1.53498	10.22684	0.37013	2.90046	0.00000	0.10446	2.75845
**Cox18**	2.75894	4.29821	6.46857	4.52204	0.93549	1.25323	1.70715	2.32915	1.22047	6.45044	5.17885	0.81946	3.30014	0.04271	0.78989	3.34159
**Cox19**	5.71297	3.95061	2.52522	0.00000	0.22659	0.85878	1.57738	2.30613	0.33794	5.15317	1.62787	0.83620	1.99866	0.04466	0.43801	0.01209
**Cox20**	2.98629	0.08522	2.17572	1.14383	2.61750	0.31569	0.04341	0.40009	0.26516	0.72407	2.20733	3.46262	1.80412	0.87741	0.22136	1.47136
**Cox4i1**	31.59342	11.40002	24.66997	14.79043	20.82856	12.85572	13.99030	21.50438	18.84510	23.55651	23.96660	4.48825	30.72279	2.02257	3.08023	20.52233
**Cox4i2**	0.00754	0.00766	0.00164	2.29373	0.00931	0.00738	0.00693	0.19324	2.35786	2.24498	0.91710	0.22941	0.00934	0.03826	0.88803	0.00698
**Cox5a**	12.14909	3.44435	11.77826	17.38536	11.93703	4.68203	4.40928	0.20376	27.87095	23.25399	23.87168	1.12063	12.64024	6.33096	1.64330	6.73288
**Cox5b**	6.44982	0.09941	3.53836	7.91980	7.61471	2.18758	0.10355	0.00000	11.60768	7.42763	10.93243	0.07321	10.27912	0.00000	0.00000	6.86516
**Cox6a1**	14.63895	0.27823	6.97045	13.78761	13.44454	6.10528	0.53177	2.63852	18.76172	16.53885	23.05064	0.82491	13.53466	0.93405	0.00000	11.45074
**Cox6b1**	15.28463	1.43437	11.13156	6.79580	5.96561	4.77225	1.85431	0.84726	10.99133	10.27655	16.64879	0.86515	10.68495	5.44067	4.85100	9.56581
**Cox6b2**	1.68789	0.00563	0.13980	16.33374	0.00523	0.00226	0.00498	0.00000	3.25152	0.50776	1.51525	0.11547	0.00000	0.31480	2.76842	0.00219
**Cox6c**	12.91490	0.66912	13.28857	13.48316	13.10580	4.21906	1.42344	1.37875	13.18566	6.28338	27.79697	1.97644	6.12935	3.89299	1.89511	6.68972
**Cox7a1**	1.01555	0.00669	0.05909	0.76456	0.00648	0.22040	0.00620	0.00000	0.67635	0.00000	1.35572	0.00964	0.23460	0.02134	0.07357	0.54006

**Cox7a2**	10.27101	0.65700	9.74543	13.06445	7.26521	1.51687	1.27140	0.24754	14.33622	5.95595	18.31562	1.20070	4.56832	5.06970	2.91785	4.24068
**Cox7a2l**	24.83908	13.48869	23.53745	3.84680	7.85859	9.20758	14.37920	24.11580	3.09742	24.67049	12.23197	4.68269	23.42887	0.96807	7.93274	10.74579
**Cox7b**	11.57372	0.94247	10.75756	10.95299	8.09967	1.87824	0.77542	0.72568	6.92595	3.41070	7.81517	0.35725	5.03368	4.13697	2.25537	3.24940
**Cox7b2**	0.01216	0.00755	0.01074	0.01079	0.00951	0.00664	0.00728	0.06099	0.01174	0.00898	0.01205	0.00726	0.01006	0.01905	0.12379	0.00739
**Cox7c**	4.73596	0.21581	3.57142	0.46160	2.97479	0.87945	0.78197	0.50764	5.42497	1.27948	11.95976	0.48706	2.56863	0.47693	0.65359	3.60649
**Cox8a**	11.99679	0.03695	3.71897	13.62593	12.16462	2.10991	0.16307	0.33055	17.02065	18.03093	19.17695	0.37803	11.03536	0.46464	0.00000	7.55891
**Cox8c**	0.01348	0.00773	0.01199	0.00913	0.00924	0.00657	0.00742	0.01272	0.01193	0.45198	0.01217	0.00629	0.01193	0.01573	0.03150	0.00757
**Cp**	0.01213	0.00797	0.08201	0.00973	0.00901	0.00919	0.00752	0.01998	0.01183	0.18486	0.01077	0.00797	0.01149	0.08299	0.08302	0.00739
**Cpa1**	0.00333	0.00793	0.00000	15.72877	0.00905	0.00965	0.23313	1.65527	7.18243	1.31351	2.01884	0.24859	0.00000	0.00000	11.41724	0.00371
**Cpa2**	0.05070	0.00784	0.00626	0.35531	0.00791	0.00692	0.00723	0.00000	0.34567	0.00000	0.61692	0.04425	0.00663	0.00423	0.22708	0.00687
**Cpa4**	0.01358	0.00806	0.01080	0.05708	0.00935	0.00670	0.00739	0.01148	0.29478	0.00953	0.01095	0.00652	0.01311	0.20560	0.00000	0.00744
**Cpb2**	0.01383	0.00823	0.00918	1.54772	0.00911	0.00671	0.00754	0.00693	0.00000	0.00609	0.42343	0.00641	0.00791	0.00548	0.00000	0.00691
**Cpd**	3.24492	0.00414	0.23689	8.25702	0.01153	0.19499	0.04638	0.00000	6.06904	1.54560	0.22569	0.02858	1.25408	0.00000	4.85632	0.09142
**Cpe**	3.02959	0.00455	1.15205	0.32767	0.23674	0.00243	0.00464	0.00000	0.01068	1.79737	0.89799	0.00746	0.00868	0.00521	0.00000	0.01880
**Cpeb1**	0.00534	0.00808	0.37939	4.86571	0.00927	0.00535	0.00708	0.11506	1.56275	1.00774	0.64232	0.49018	0.01055	0.85191	2.34510	0.00659
**Cpeb2**	2.11266	0.00000	1.33325	2.47443	0.33334	3.31516	1.55471	1.60970	1.57412	0.05074	1.56757	13.44886	0.44618	6.10219	3.98299	0.37617
**Cpeb3**	1.29146	0.00559	0.22586	2.33377	0.01346	0.19641	0.00451	1.36887	2.63712	3.69181	2.62183	1.45287	1.02512	6.33783	3.94164	0.01114
**Cpeb4**	0.11343	0.10153	0.69388	8.62833	0.00696	0.00418	0.35383	0.12946	7.93208	0.00000	1.80068	1.71104	0.12133	4.27197	1.62372	0.00456
**Cplx1**	0.00704	2.15672	0.00326	0.39943	0.00809	0.89438	0.00637	0.00912	0.00767	0.32309	0.01081	0.00573	0.00622	0.00380	0.17721	0.00614
**Cplx2**	0.01806	0.02827	0.05253	0.18615	0.00879	0.02322	0.07820	0.02470	0.23375	0.08116	0.02151	0.02443	0.03016	0.31029	0.10005	0.00726
**Cpm**	0.00557	0.00785	0.00000	0.91015	0.02998	0.30785	0.00579	0.37436	3.15663	0.59406	1.34684	0.13672	1.52628	0.50652	0.54873	0.00640
**Cpn1**	7.73299	8.15698	6.60933	1.66455	8.35402	0.03441	5.45226	7.06083	5.64881	2.41702	0.37974	0.37034	10.80739	0.00000	0.84525	10.34929
**Cpne1**	1.30384	1.19422	1.06221	7.31088	1.08571	0.02393	1.98133	1.84352	8.33061	0.77325	2.35834	12.47587	0.26794	9.96618	7.53117	0.69831
**Cpne2**	0.01382	0.00813	0.01029	0.08054	0.00914	0.00671	0.00754	0.00901	0.01082	0.00852	0.24651	0.00735	0.00945	0.01046	0.00353	0.00725
**Cpne3**	2.39169	0.00257	4.07499	0.17020	5.38698	1.89464	0.08536	1.03968	0.45556	1.14424	0.02087	0.06691	0.27564	0.01322	0.00000	0.00256
**Cpne5**	1.19194	0.00716	0.74805	2.35288	0.00473	0.00471	0.00718	0.31223	0.33071	0.57960	0.00628	0.03245	0.01450	0.00155	0.31055	0.00557
**Cpne7**	1.35426	0.00686	0.00225	2.17956	0.01600	0.00714	0.00592	0.88811	1.46221	2.76016	0.02284	0.11900	0.00544	0.00575	0.01921	0.01334
**Cpne8**	3.43156	5.43260	3.20702	5.48773	2.67865	0.34455	5.89103	2.61316	10.57944	6.89224	1.98392	5.82026	1.65644	4.30927	2.95674	3.02845
**Cpne9**	1.87116	0.04365	0.01319	2.60250	0.03704	0.00586	0.01418	0.38567	1.74440	0.00000	1.53283	4.25568	0.00000	0.49134	0.29585	0.03201
**Cpox**	1.96065	2.53072	1.00018	0.46624	0.05415	0.00386	0.67904	0.05061	0.37165	0.79756	1.38298	0.09397	1.84891	0.40727	0.49839	0.01056
**Cpped1**	0.29090	0.00652	0.00000	0.02515	2.08650	2.47134	0.00648	1.63607	0.00968	0.00770	0.00000	0.02452	1.41486	0.00623	0.16861	0.00554
**Cpsf1**	9.61995	8.54481	9.35109	3.32960	5.44923	4.32384	4.96514	11.99400	1.37440	6.27011	8.07940	0.22568	5.57599	0.01694	2.01994	2.37077
**Cpsf2**	6.53171	2.88133	3.35670	4.55698	2.34823	2.41156	3.82354	8.87357	2.13014	5.46740	4.32162	0.72929	2.04645	7.34675	10.43276	2.22842
**Cpsf3**	13.73107	15.36305	16.51571	1.70280	8.67616	5.88464	12.52072	17.14824	5.80237	13.42418	16.53075	4.02894	14.39446	14.65299	12.27548	8.65566
**Cpsf3l**	8.36972	6.85157	15.49474	5.48496	7.25069	7.39073	8.13978	13.77241	7.92285	11.23176	8.34703	4.11612	12.29073	1.40856	9.85179	5.04071
**Cpsf4**	6.41066	0.79962	4.91267	2.62832	4.73355	0.62525	4.86398	6.25637	4.98421	4.73017	0.29741	0.28962	2.72253	0.00000	0.12237	2.86636
**Cpsf4l**	0.00819	0.00892	0.16434	2.39821	0.00915	0.00544	0.00825	0.18915	0.56387	1.00330	0.01507	0.03054	0.00931	0.00223	0.51323	0.01418
**Cpsf6**	5.35808	7.35918	3.05209	14.34815	5.96417	2.21779	9.01323	13.27860	21.86871	9.10086	7.85777	10.20940	10.07714	12.95506	4.02494	2.63567
**Cpsf7**	4.16036	0.03127	6.71852	1.15741	2.68666	3.68846	0.27783	2.40234	0.63046	1.22548	1.94938	7.36042	1.79889	3.89495	1.97221	2.81319
**Cpt1a**	9.50871	1.48514	2.96301	1.09198	4.19424	2.70740	1.56900	9.84597	6.31377	7.82739	7.55540	4.26882	2.48211	7.90201	8.34301	2.34707
**Cpt1c**	0.00554	0.00746	0.00734	1.15285	0.00947	0.00649	0.00631	0.71745	0.88027	0.73752	0.01843	0.03644	1.51905	0.10203	0.49515	0.00665
**Cpt2**	4.06529	0.21751	2.20148	5.78240	0.02909	2.63851	0.01398	0.01030	4.22250	0.13317	0.00000	0.01863	0.03279	0.00000	0.62290	1.18470
**Cptp**	2.64266	1.75245	3.40802	9.95597	0.00000	7.37661	2.98329	2.48757	11.67890	2.78016	0.53065	0.13911	2.95876	0.00000	2.47734	0.00000
**Cpxm1**	4.56095	0.96720	0.00000	0.01242	0.00550	0.00265	5.27499	1.33015	0.00936	0.00434	0.00000	0.00750	2.95507	0.00856	0.42439	7.04213
**Cpxm2**	0.13670	0.00841	0.00765	0.00944	0.01697	0.00699	0.00741	0.01246	0.02667	0.01401	0.01246	0.01829	0.01253	0.01573	0.02079	0.01501
**Cr1l**	3.62210	2.42774	4.51325	0.00000	2.99390	0.29238	0.68244	1.04159	0.01936	2.42828	1.48049	2.05591	1.05995	0.41284	0.34122	1.69893
**Crabp2**	0.01769	0.00800	0.00936	1.10696	0.00883	0.00658	0.00742	0.00822	1.63326	0.06897	0.13853	0.00439	0.01129	0.00446	0.51252	0.00706
**Cracr2a**	0.00844	0.00800	0.01196	0.25296	0.00905	0.00640	0.00668	0.00986	0.04735	0.00514	0.01313	0.00743	1.33127	0.01230	0.00643	0.00654
**Cracr2b**	0.12066	0.01738	0.10470	0.20029	0.73085	0.02784	0.08723	0.24983	0.71129	2.37650	2.90055	0.05417	0.16655	0.52808	0.06010	0.02763
**Cradd**	1.56548	0.00724	0.05454	0.00553	0.01209	0.00439	0.00679	0.34560	0.01090	0.20047	0.35213	0.00544	0.01129	0.03171	0.00000	0.00595
**Cramp1l**	3.00020	0.00713	5.27143	3.50966	6.00299	1.21023	4.60116	2.32238	4.22224	4.94079	1.54318	5.16355	0.26011	0.11135	7.33524	0.03632
**Crat**	3.77339	0.00000	0.37075	1.56436	5.83133	4.43407	1.97889	2.00494	0.00000	0.65695	0.91682	0.06357	0.00000	0.00008	0.12869	0.00000
**Crb3**	0.00000	0.11694	0.00000	17.55227	0.04107	7.70376	0.01544	20.06048	15.84611	25.85000	12.82208	1.50809	11.16083	0.02067	0.00000	0.00000
**Crbn**	2.35686	6.60314	3.38880	2.31027	6.16140	4.15455	0.81784	4.92090	1.71596	4.91624	3.75164	4.25614	1.36675	9.52996	3.98215	2.15520
**Crcp**	3.56348	4.91924	6.01545	4.43525	5.67590	0.22586	2.89142	6.86415	7.04837	6.20288	4.84613	2.28510	4.44994	2.19602	7.63354	0.00996
**Creb1**	3.99380	0.08613	2.16175	0.87229	3.10499	1.61635	3.91422	0.24870	7.31557	2.27474	0.77289	1.44142	0.95318	0.17373	2.45181	0.44551
**Creb3**	0.14689	3.42942	7.69407	0.61120	0.00000	3.56166	3.23694	5.64689	10.36343	3.16383	1.33639	0.46328	3.34626	4.33217	6.00157	3.75623
**Creb3l1**	0.01320	0.00794	0.00840	0.95873	0.00984	0.00747	0.00743	0.00000	0.78513	0.11538	0.88106	0.01972	0.10240	0.00214	0.12950	0.00778
**Creb3l2**	1.42990	0.00584	0.00000	0.18460	0.00316	0.00563	0.38146	0.00407	0.03713	0.36994	0.01000	0.05354	0.00714	0.00994	0.00957	5.67502
**Creb3l3**	0.01162	0.00772	0.00409	1.59065	0.01809	0.00676	0.01730	0.00000	2.99509	0.00000	0.94906	0.00689	0.14982	0.03106	0.66569	0.00656
**Creb3l4**	0.00805	0.00769	0.41488	6.15406	0.00680	0.01032	0.00677	0.00000	3.15894	0.92253	0.64579	0.00403	0.00000	0.00000	0.79984	1.61581
**Crebbp**	0.41935	0.01823	1.21446	3.34275	1.35965	0.01415	0.00592	1.29820	3.29611	1.93031	0.00000	0.01994	1.41629	7.32359	4.49113	0.00517

**Crebl2**	0.79235	0.00895	0.00297	0.93369	0.01694	0.00575	0.00760	0.28504	3.35397	0.00000	0.00000	0.02002	0.00807	0.71297	0.78308	0.00617
**Crebrf**	0.00000	0.00000	2.52171	6.96248	0.00346	0.00229	0.74464	1.16420	9.78368	10.91546	8.47733	20.06248	0.00000	12.53186	16.07585	0.00130
**Crebzf**	0.75677	0.61678	0.85727	6.02494	1.64037	0.37022	0.02619	1.85225	1.65425	1.52092	0.18672	2.39115	0.25800	2.11989	2.34568	0.27804
**Creg1**	8.63119	0.59921	6.75660	1.48833	2.88902	0.17661	2.19652	4.92187	0.06509	9.45879	6.26755	1.23488	3.59527	3.22103	3.05341	0.00000
**Creld1**	0.01164	0.00798	0.18680	1.64510	0.01000	0.00759	0.00630	0.00116	1.39353	0.06503	0.25673	0.00284	1.42093	0.00319	0.21070	0.00634
**Creld2**	3.14420	3.00970	5.90503	12.32324	0.03471	0.04763	4.81851	5.90735	4.49914	1.20460	6.28670	3.13961	11.24334	1.35473	3.04908	3.40458
**Crem**	1.65638	0.00533	3.43101	2.25548	0.00000	0.00486	0.01633	0.33195	1.47714	0.00000	0.41350	0.03469	0.00000	0.00285	0.89721	0.17492
**Crim1**	0.02649	0.00828	0.17706	0.19943	0.01199	0.00897	0.00733	0.01224	0.02755	0.01460	0.01311	0.02810	0.01058	0.01181	0.00337	0.00739
**Crip1**	1.63123	0.03447	0.10731	0.41338	0.01342	3.58911	0.02300	0.01071	0.00468	1.58020	3.79615	0.00000	1.87166	0.00000	0.00000	3.01860
**Crip2**	16.45450	4.86120	12.47531	0.69371	15.17254	14.70591	6.48500	38.36844	0.38504	18.09841	17.85370	0.64010	33.72514	0.07697	0.00000	9.77308
**Cript**	10.41101	10.46111	10.19523	17.89536	10.57021	8.40886	3.50689	7.23804	18.99114	14.57354	10.56779	12.75101	10.79465	11.21833	10.90392	3.52638
**Crispld2**	0.49790	0.00768	0.10263	1.18948	0.00672	0.00513	0.09056	0.01151	0.80732	0.00727	0.03667	0.03173	0.01085	0.00436	0.31543	0.00642
**Crk**	5.91386	6.30485	6.53411	12.68184	8.41209	5.21225	6.21740	1.69781	4.81641	11.84043	0.38182	9.24489	1.61145	17.10084	14.94355	0.90312
**Crkl**	6.56886	1.36878	2.34025	10.95475	4.08456	5.66714	2.12219	2.15952	3.42594	2.52531	2.43136	3.50284	2.75046	3.58202	4.14781	2.64689
**Crlf1**	0.01328	0.00800	0.00859	3.26886	0.00904	0.00649	0.00722	0.00453	0.53365	0.00000	0.28932	0.06751	0.12892	0.00408	0.00000	0.00655
**Crlf2**	0.05562	0.01793	0.00704	0.88624	0.00799	0.00711	0.00669	0.00000	0.41651	0.36010	1.58268	0.00744	0.01306	0.00802	0.00000	0.01479
**Crlf3**	5.47533	0.00682	3.86407	2.70706	2.40386	4.96049	3.66694	7.50598	5.13768	10.84426	4.84095	3.06558	4.24140	4.61509	9.20763	2.03879
**Crls1**	2.06107	1.95183	0.30492	1.13751	0.06035	0.07440	1.34787	1.97141	1.63442	3.51448	9.51100	1.51124	1.27705	11.07469	1.46035	0.21960
**Crmp1**	0.00620	0.00788	0.01120	0.23913	0.01813	0.00635	0.00633	0.00946	0.00769	0.02836	0.01206	0.03721	1.93015	0.01188	0.00869	0.00623
**Crnkl1**	2.23686	8.14650	7.64421	6.49211	5.09746	0.00000	2.60124	22.96210	18.86095	33.08924	12.16841	14.15018	4.09074	25.82274	31.30187	1.86368
**Crocc**	1.13103	0.02970	0.47301	0.92302	0.03290	0.00579	0.38398	0.00000	0.00000	3.36745	1.12544	0.03253	0.03216	0.00379	0.00000	3.48043
**Crot**	9.30880	5.17582	5.60109	1.57825	2.58218	5.97456	6.63687	2.21135	0.00000	4.66957	0.84633	0.08067	1.31486	1.48561	2.78301	0.00000
**Crtac1**	0.01368	0.00850	0.01096	0.00509	0.00975	0.00712	0.00768	0.00436	0.01119	0.18549	0.41325	0.00610	0.03225	0.00695	0.05776	0.00735
**Crtam**	0.01017	0.00828	0.02386	0.00934	0.01056	0.00660	0.00755	0.01160	0.02420	0.00687	0.00876	0.00180	0.11684	1.76043	0.00297	0.00773
**Crtap**	5.43309	5.11515	5.82884	2.64090	5.61911	0.10117	3.00726	8.66988	2.65766	4.75479	5.63393	8.33395	6.13648	1.77927	2.58958	2.64212
**Crtc1**	0.00462	0.00795	0.15556	0.12315	0.30631	0.01251	0.00764	0.23117	0.00654	0.44019	0.13127	0.01637	0.83678	0.01453	0.00352	0.00716
**Crtc2**	0.08678	0.11535	0.00000	0.34554	2.22669	0.07995	0.00701	0.34269	0.43320	0.03220	0.48192	0.02023	0.02556	0.01356	0.65794	0.02111
**Crtc3**	1.86326	0.01661	0.36731	1.06369	0.03075	0.25859	0.00585	0.00000	2.38300	0.63524	0.04744	0.01928	0.01611	0.07762	0.01548	0.83131
**Crx**	0.03685	0.01776	0.23872	6.59849	0.01653	0.02372	0.02666	0.02818	3.41457	3.13426	0.61656	0.19385	0.03538	1.88962	3.63247	0.07085
**Crxos**	27.54326	14.54120	31.40792	14.36798	22.14591	13.08025	15.14107	22.89954	33.45477	28.92922	41.93466	16.11519	28.64284	23.43484	20.85308	11.16334
**Cry1**	2.79588	1.93291	3.45442	16.43797	0.00566	0.00000	2.70580	6.71882	12.67027	11.44352	2.74367	9.99122	3.20275	1.68955	14.97965	1.70514
**Cry2**	1.69694	0.00531	0.68905	3.44518	0.01937	0.09429	0.01416	3.04652	3.41562	0.58329	1.38824	0.02865	2.43989	0.02809	2.09146	0.01005
**Cryab**	0.00000	0.03157	4.96970	0.00802	0.03671	0.15856	0.00744	0.15349	0.02194	1.86649	0.00323	0.29092	0.22899	0.03193	0.00000	2.84661
**Cryba1**	0.01292	0.00812	0.00631	0.00470	0.00924	0.00711	0.00772	0.02059	0.01053	0.00248	0.71254	0.02667	0.00584	0.01554	0.23075	0.00723
**Crybg3**	0.11247	0.00605	0.30314	1.25801	2.28116	0.00477	0.69502	0.30927	5.10711	0.90968	0.39324	0.07245	0.92672	0.00465	2.58310	0.00571
**Cryga**	0.05382	0.00796	0.05522	0.24393	0.00994	0.00589	0.00737	0.01248	0.00757	0.01753	0.01349	0.03893	0.01192	0.01129	0.00528	0.00732
**Crygc**	1.07692	0.00770	0.00379	0.01047	0.01624	0.00732	0.00673	0.01055	0.01222	0.05216	0.01205	0.00817	0.01171	0.01180	0.01219	0.08882
**Crygd**	2.09001	0.00595	0.02399	0.01025	0.00926	0.00452	0.00574	0.01223	0.01249	0.00980	0.01248	0.00628	0.01547	0.00858	0.05226	1.23025
**Cryge**	1.20733	0.00666	0.06271	0.00904	0.12325	0.00469	0.00645	0.00848	0.01206	0.08286	0.01145	0.01699	0.01072	0.00600	0.18461	0.21674
**Crygf**	0.31303	0.00782	0.01623	0.01029	0.02330	0.01227	0.00722	0.01260	0.01249	0.01935	0.01283	0.00755	0.01145	0.00880	0.03901	0.11677
**Crygs**	1.47640	0.33114	0.00000	0.07308	0.00495	0.25298	0.00422	0.26632	0.76420	1.07731	4.44234	0.14570	0.47243	0.48911	1.32583	0.00464
**Cryl1**	0.00227	0.00373	0.00000	1.63099	4.49838	0.00591	0.00655	0.02328	0.64654	0.94381	0.00000	4.34502	0.00933	0.00610	0.78982	0.00663
**Crym**	1.88278	0.02618	0.23850	0.01072	0.00278	0.00663	0.00561	0.00482	0.01238	0.00401	0.01196	0.01652	0.56512	0.01454	0.00562	3.40893
**Cryz**	7.11817	2.98013	3.86521	0.40485	5.68439	0.01869	0.00000	0.83874	0.11542	0.15480	1.36131	0.00830	1.38194	0.00199	0.00000	1.35145
**Cryzl1**	3.33675	2.84001	6.94933	0.00000	3.42678	5.18756	4.62631	0.15778	0.56575	1.87976	0.77518	0.02214	0.13065	0.00369	0.74529	1.93617
**Cs**	16.74591	12.94762	16.05676	13.61127	16.43898	9.18026	16.77362	21.56382	17.43514	10.56671	12.11671	7.12835	15.98202	11.64155	10.11410	10.98065
**Csad**	0.13837	0.08656	0.79971	1.98120	3.29641	0.00928	0.01667	1.74056	0.15708	0.09765	2.11163	0.40827	0.13205	0.20008	1.27591	0.00622
**Csdc2**	1.13615	0.00612	0.32470	0.93471	0.01302	0.00433	0.19414	3.42998	0.56643	4.23863	0.30278	0.13515	0.02756	0.39648	0.41497	0.00559
**Csde1**	14.59809	15.19969	12.41322	16.20095	12.59236	9.23465	10.19570	20.10001	21.96779	30.28366	20.36888	14.82686	11.15809	44.90874	32.15552	6.27281
**Cse1l**	10.00882	17.04987	6.92278	12.43854	11.17633	4.05373	6.28091	12.34530	8.99718	21.61723	14.77392	19.15170	7.16457	26.75321	21.27407	6.30929
**Csf1**	0.72534	0.00772	0.00340	2.49705	0.01778	0.00722	0.00659	0.00762	0.14423	0.01355	0.02554	0.23467	0.01194	0.00907	0.00001	0.00650
**Csf2ra**	0.00867	0.02977	0.19106	1.18421	1.81507	0.00590	0.50325	3.44984	0.63564	2.14100	2.10774	0.06671	0.36511	0.00455	0.03138	3.20199
**Csf2rb**	0.01761	0.00806	0.03419	0.13997	0.00996	0.01312	0.01784	0.01829	0.13478	1.23222	0.23067	0.01607	0.00930	0.13132	0.01455	0.00731
**Csf2rb2**	0.01655	0.00738	0.13120	0.47122	0.00954	0.01719	0.04703	0.21153	0.90768	1.04828	0.80829	0.85520	0.10500	0.49492	0.91958	0.00693
**Csf3r**	1.45087	1.77802	7.07055	0.00000	9.56935	10.00940	9.59647	5.54076	0.48554	1.57980	1.61577	0.16555	12.37945	0.57800	1.42210	0.50567
**Csgalnact2**	0.00000	0.00800	0.16694	0.34747	0.00999	1.68028	0.02888	2.41244	0.32810	0.29189	0.62254	0.02015	0.00000	3.61177	1.06879	0.22944
**Csk**	18.00624	2.51906	5.75227	3.34258	6.26348	6.42079	3.83763	14.10243	3.85752	10.54908	3.99865	0.91338	7.78242	0.00000	0.00000	3.55276
**Csl**	0.77934	0.76210	1.17272	0.65183	1.43825	0.65679	1.44691	1.33403	0.80627	0.56488	0.66303	0.08800	1.17163	0.35044	0.35532	0.58873
**Csnk1a1**	4.80065	9.81500	5.84464	14.74727	6.01679	0.14400	6.20431	6.82462	18.66655	8.70143	6.50718	0.67796	1.80678	6.14128	13.69416	2.62186
**Csnk1d**	7.73651	4.48442	1.44714	5.21043	0.04954	0.76575	4.46615	7.03072	3.37002	6.37839	2.97118	0.51143	4.46969	1.33380	4.32085	3.10708
**Csnk1e**	7.89482	3.43331	1.51945	13.39135	6.51584	5.45454	7.31325	22.67129	21.56211	26.44657	10.97685	9.47218	7.97019	8.71767	14.53004	2.14747
**Csnk1g1**	3.15702	0.00000	6.42081	2.71914	1.99230	0.00319	4.28256	1.94171	3.81292	7.09665	2.16155	1.19274	0.00000	15.08009	7.08351	2.20382

**Csnk1g2**	10.19098	0.03535	0.00000	1.96946	4.14093	7.26383	0.38510	6.79622	0.40574	7.43062	0.87682	0.19051	6.60933	0.00000	0.00000	8.89146
**Csnk1g3**	1.52924	0.20279	0.00000	8.13379	3.48820	1.79008	4.45120	3.99895	12.52876	10.36758	2.26052	10.16205	1.43067	16.88481	11.51753	0.02194
**Csnk2a1**	6.59881	6.05613	8.10949	4.79435	3.35931	3.97130	1.77179	6.69070	11.42874	9.60931	8.30992	2.02990	4.80081	15.53482	10.59543	4.03076
**Csnk2a2**	1.63503	8.09933	1.23298	6.54415	1.45987	1.03559	2.33647	5.17264	10.01421	3.67545	6.54645	5.09581	0.00000	4.05423	6.28265	0.31594
**Csnk2b**	18.66107	13.18982	19.87279	23.16663	17.75373	12.80448	13.01373	21.79026	21.03401	21.18367	22.25903	6.09309	20.86623	4.79254	8.50398	14.80820
**Cspg5**	0.00574	0.00726	1.66809	0.00413	0.01687	0.00915	0.00693	0.74098	0.63002	0.00000	0.00000	0.01381	0.09196	0.02005	0.17962	0.00660
**Cspp1**	0.81856	2.07121	1.47518	0.78019	0.01224	3.68524	2.71765	1.94191	1.40462	0.27616	1.50648	7.72102	1.73137	2.18569	0.70886	0.00922
**Csrnp1**	6.39361	0.16583	7.67860	3.92535	0.02111	5.16937	0.00013	6.96407	6.98925	13.00357	10.54803	4.62630	5.89222	0.14767	9.97928	3.13067
**Csrnp2**	0.83052	0.01439	0.87495	6.61676	0.04710	1.08652	0.00257	1.18796	10.69900	1.02526	5.09494	7.93644	1.18821	11.51973	6.62389	0.00905
**Csrnp3**	0.04268	0.01922	0.04694	2.28006	0.00937	0.01207	0.01698	0.00000	0.88167	0.70500	0.49703	0.00000	0.07279	2.75892	1.86927	0.02047
**Csrp1**	0.53869	0.98819	2.45031	0.07869	0.22625	8.50500	3.22124	5.70803	0.05714	7.76881	1.68434	0.32958	6.29949	0.00619	0.00000	1.80113
**Csrp2**	1.53635	1.23954	2.38215	0.03103	0.16545	4.45784	5.80302	2.23061	0.18154	2.00042	1.48354	0.19725	6.24602	0.05487	0.00000	1.80047
**Csrp2bp**	0.23337	3.44878	4.78449	3.67585	2.03495	1.94382	2.80899	10.71018	7.39017	9.21636	3.08027	1.64459	6.98889	0.97552	9.38827	3.79061
**Cst13**	0.01383	0.00820	0.00872	0.00537	0.00909	0.00687	0.00760	0.00592	0.01128	0.00593	0.49707	0.00637	0.00743	0.01119	0.00285	0.00715
**Cst3**	10.20738	0.01974	2.15867	0.00000	8.83476	5.15877	2.92660	6.80350	0.12708	8.91049	4.18317	0.22495	9.57796	0.00083	0.20905	8.51396
**Cst6**	0.01023	0.00885	0.04895	0.48106	0.01905	0.00613	0.00831	0.06443	0.00413	0.05632	0.01196	0.02384	0.01160	0.01221	0.00906	0.00735
**Cst7**	1.33528	0.00331	1.39554	8.30758	0.00291	2.05411	0.00342	2.66218	8.83326	4.02344	2.71835	6.75613	1.02425	3.94976	2.32205	0.00856
**Cst8**	0.01333	0.00793	0.01222	0.00768	0.00906	0.00653	0.00735	0.01069	0.01195	0.82330	0.01139	0.00579	0.01105	0.01176	0.00832	0.00726
**Csta1**	0.78330	0.00771	1.55719	4.60899	0.05193	1.69013	0.01561	0.01560	6.00169	0.15070	0.71971	0.00000	1.52021	0.25130	0.07277	0.52861
**Cstb**	7.58200	1.54041	10.07369	6.77707	9.14761	9.96434	2.85665	3.33656	9.99955	3.99505	25.91259	1.82744	11.07664	0.61270	0.88448	5.34821
**Cstf1**	6.08372	1.82732	10.90680	11.16009	5.42370	2.67639	6.45717	9.28059	16.97939	7.71049	6.34802	0.59542	7.71533	0.00730	2.58955	3.88450
**Cstf2**	7.02765	1.81588	3.50357	3.99868	8.18504	4.42554	5.17331	9.83596	3.08408	7.24055	3.45946	1.31661	3.07287	6.38904	3.46211	0.28985
**Cstf2t**	3.31396	2.22285	3.25203	7.95813	3.49675	0.11902	0.13257	1.50682	12.72427	3.66888	1.85677	12.81126	1.02270	16.50987	14.08598	1.40670
**Cstf3**	5.08829	2.24083	2.09794	4.94909	9.06199	1.17780	3.55761	4.12500	8.80657	6.68303	1.65580	6.81380	6.28518	7.03882	0.88744	1.32524
**Ctbp1**	5.21213	1.02940	2.19124	0.06341	4.56631	4.99135	1.71451	2.40370	0.00995	5.64298	0.12286	0.95867	7.21013	0.00626	0.00000	1.96642
**Ctbp2**	9.02826	5.37465	8.51768	2.43827	3.72376	6.20671	7.98289	3.22208	4.00420	4.23045	4.12242	1.36531	4.87289	0.01835	0.99673	8.92544
**Ctbs**	0.00448	0.21071	0.13160	0.28248	2.71348	2.63110	0.35732	1.05454	0.11237	1.49387	0.00446	0.00711	0.00000	0.00652	0.58604	0.01337
**Ctc1**	6.23126	3.55649	4.65785	10.02059	2.64631	5.10438	5.76079	4.24301	6.37537	8.09824	4.09739	0.41622	2.74674	2.15494	5.25250	3.10791
**Ctcf**	8.02087	5.21618	6.65136	19.65717	7.77438	0.41937	2.34598	11.17336	13.55962	19.91990	5.44968	9.26927	9.25309	8.14275	12.14338	3.02728
**Ctcfl**	0.10799	0.00758	0.27624	0.04758	0.04900	0.04137	0.06842	0.01553	0.19027	0.05264	0.58925	0.04354	0.27503	0.16180	0.15987	0.02113
**Ctdnep1**	7.75915	4.77340	3.77785	4.51176	0.07361	5.97279	5.01970	4.00240	8.59555	6.53270	2.02699	0.21313	4.26852	0.37455	3.98070	5.71380
**Ctdp1**	5.45574	0.00000	0.78595	1.40234	1.83295	1.41025	3.09047	8.40283	2.51402	9.79646	0.39816	5.86761	8.03273	0.02760	0.00000	1.98626
**Ctdsp1**	1.44984	0.90341	0.27423	3.88670	2.19155	0.76165	0.00539	0.06276	1.09523	0.75712	0.00000	0.02551	0.34538	0.00001	1.12438	3.09159
**Ctdsp2**	7.94023	0.19075	1.42342	2.06118	2.06245	7.63224	1.10222	2.24668	5.90138	8.26758	2.90292	0.59342	5.90687	2.11410	3.81973	6.57011
**Ctdspl**	0.00000	0.00731	0.07598	2.55141	0.01563	0.88678	0.00569	0.00315	0.17854	0.75745	0.34215	0.02092	1.97958	0.00302	0.47079	1.85702
**Ctdspl2**	2.28965	1.66079	2.71159	7.86595	1.60739	0.08294	2.73286	5.52284	8.02901	4.96064	3.07703	2.68964	0.41359	18.32799	8.22487	0.53112
**Ctf1**	0.01287	0.00775	0.01180	0.24638	0.00938	0.00658	0.00736	0.03559	0.04932	0.01120	0.01239	0.00730	0.01124	0.00675	0.08855	0.00734
**Ctf2**	0.01100	0.00756	0.00799	0.67340	0.00933	0.00672	0.00708	0.58641	1.07584	0.00483	0.00000	0.00455	0.00567	0.01155	0.00000	0.00715
**Ctgf**	0.01829	0.00743	0.00701	0.00485	0.01045	0.00695	0.00713	0.01845	0.17904	0.04147	0.00632	0.57443	0.01298	0.01199	0.13849	0.00776
**Cth**	5.80252	9.76747	11.80778	6.95979	0.15383	7.88937	6.95653	25.82305	13.56359	27.85546	14.74876	11.71481	15.08398	29.13679	20.45892	3.48510
**Cthrc1**	0.00000	0.00468	0.87824	0.00146	0.02280	0.00854	0.09619	7.49508	0.00523	6.60728	8.44032	3.05256	4.43953	2.57016	0.00000	0.00343
**Ctif**	0.01826	0.00803	0.15917	0.49227	0.07940	0.00604	0.28120	0.01130	0.20294	0.02089	0.01120	0.06291	0.01056	0.01129	0.00043	0.00722
**Ctla2a**	0.04815	0.03855	0.04823	0.10292	0.01645	0.00805	0.05806	0.07346	0.24036	0.07006	0.02299	0.02486	0.05850	0.47563	0.08249	0.02907
**Ctnna1**	16.54503	19.23922	15.40836	11.57622	10.71458	13.28192	13.62406	23.78814	17.64146	13.50467	15.26606	9.01159	16.33533	8.01776	5.53796	9.10307
**Ctnnal1**	0.83821	3.14354	3.84650	7.15305	0.00744	4.32098	2.25274	6.23214	12.17616	8.18777	3.19666	12.08628	2.11734	15.31318	6.21467	0.52244
**Ctnnb1**	16.06403	14.24440	14.04912	11.96139	15.58458	8.45851	12.78929	12.09867	18.53514	13.32874	9.14789	15.54413	10.55351	6.28024	16.00299	10.14475
**Ctnnbip1**	2.61870	0.00321	1.06471	5.80984	0.60497	3.65078	0.00267	4.51663	6.74720	2.04275	4.18493	0.24676	0.39922	0.17801	0.99768	1.75945
**Ctnnbl1**	4.16637	3.86220	6.69594	10.55040	11.48299	4.45787	7.13566	10.49862	13.44671	11.42839	6.78716	5.09830	8.94971	4.82817	9.91902	5.40373
**Ctnnd1**	13.59918	5.17290	9.00985	2.99112	7.30719	5.95706	2.44046	9.88413	4.59032	8.52108	7.76608	2.59049	7.34308	13.26366	13.61261	5.90872
**Ctns**	0.61009	3.64707	1.30474	0.19750	0.00670	0.00707	0.60232	1.69080	0.00597	0.00000	0.00000	0.05023	0.69368	1.86949	1.69474	1.09503
**Ctps**	8.78975	8.63851	9.32784	15.29804	8.37267	6.79288	9.75496	14.15775	10.91776	18.18069	11.14385	4.08716	8.11908	8.64394	18.96049	1.21391
**Ctps2**	4.21318	0.00051	1.05582	5.06220	2.33648	0.00176	0.00333	1.51635	4.57340	2.36085	0.69944	0.02148	0.00000	8.57216	2.12320	0.02311
**Ctr9**	8.26560	6.90225	6.42412	4.60866	6.89998	1.90502	3.12745	5.70909	6.73288	6.80238	1.64609	0.27263	0.89051	2.04668	6.15891	0.00559
**Ctrb1**	0.01211	0.00825	0.01371	0.26775	0.01000	0.00729	0.00779	0.01677	1.24004	0.14886	0.00003	0.00604	0.23463	0.00678	0.04875	0.00758
**Ctrc**	0.00659	1.56082	0.00702	0.10019	0.00796	0.00655	0.00669	0.00934	0.00871	0.00906	0.01303	0.00722	0.01084	0.00637	0.04476	0.00670
**Ctrcos**	0.02346	0.00807	0.04454	0.71470	0.00858	0.00702	0.01807	0.00801	0.65342	0.00717	0.23079	0.04689	0.05242	0.28927	0.06307	0.01428
**Ctrl**	0.00739	0.00784	0.00167	0.00529	0.00860	0.00839	0.00571	0.00000	0.69682	2.91960	4.59179	0.04058	0.94274	0.01089	0.23842	0.00625
**Cts8**	0.05226	0.00795	0.07838	0.03043	0.02661	0.01585	0.04978	0.02438	0.11391	0.05598	0.04848	0.01689	0.07367	0.17075	0.08646	0.02874
**Ctsa**	16.53916	11.35369	26.99828	12.24106	7.70233	6.41862	14.34682	34.73893	10.86347	27.71796	30.17443	6.83734	31.34335	4.05063	23.33190	15.53239
**Ctsb**	22.37089	7.38908	22.84284	19.59105	12.50455	9.30843	14.90363	14.93867	29.33806	25.54874	21.12230	18.46898	9.63368	20.73225	26.38693	11.11636
**Ctsc**	5.79595	4.60091	15.14216	9.99141	7.18335	7.67192	12.65820	25.95496	21.07014	30.43570	21.83597	17.79046	10.80122	36.83653	27.02730	2.39884
**Ctsd**	19.87404	10.27040	33.66962	1.23986	9.05422	9.33363	15.90117	31.15209	0.56973	21.49859	15.90744	1.32504	28.02635	10.87752	6.99194	8.11341

**Ctsf**	0.00855	0.43942	0.00939	0.00864	0.00821	0.00692	0.00731	0.01168	0.01231	0.25005	0.01179	0.00801	0.01885	0.00680	0.04380	0.00717
**Ctsh**	0.00000	0.07676	0.73923	9.97068	0.02428	0.03421	3.10494	4.67351	6.37720	9.02471	9.57301	8.43268	2.13550	3.79222	4.47688	0.00160
**Ctsk**	0.01314	0.00778	0.01060	0.14813	0.00909	0.00687	0.13032	0.01155	0.49570	0.48866	0.00771	0.00656	0.01215	0.01337	0.06105	0.00737
**Ctsl**	24.25766	14.03602	34.23804	27.17517	13.81881	12.26668	21.33944	37.69822	38.81977	42.82503	38.04343	30.23881	27.35634	53.31677	45.86616	19.72853
**Ctss**	0.01342	0.00806	0.01204	0.38771	0.00907	0.00668	0.00737	0.01114	0.31028	0.60412	0.00971	0.00621	0.01212	0.01050	0.00486	0.00755
**Ctsz**	2.85627	0.01830	4.59582	4.01062	0.09948	0.01172	1.72903	12.16379	5.40561	18.75349	17.53562	8.72261	15.75618	1.50686	6.34559	8.75885
**Cttn**	13.05502	7.25229	13.51576	7.11660	7.76043	13.73651	9.63138	21.41295	8.49876	20.76672	15.71656	2.38677	19.04760	1.19658	11.06700	10.96671
**Cttnbp2nl**	1.64307	0.00428	0.40288	13.89688	0.00688	0.16541	3.12527	0.23686	8.35880	2.45048	0.00000	0.11427	0.54577	0.17087	5.89443	0.18205
**Ctu1**	1.22334	0.44692	1.23869	1.68382	0.01274	0.02072	0.27539	0.67189	4.07782	6.17922	2.50029	0.20946	2.46381	2.68601	2.10531	0.03314
**Ctu2**	5.95199	0.94908	0.26633	1.07704	3.04986	2.35131	2.13017	6.81292	2.65695	6.19014	4.21556	0.14485	5.29669	0.00000	0.00000	3.22206
**Ctxn1**	0.09281	0.04867	0.67176	0.77305	0.01411	0.00623	0.00649	0.01890	1.22700	1.83364	0.00000	0.50068	1.21535	0.02946	0.14730	2.18226
**Cubn**	2.20869	0.00286	0.00217	0.13807	4.36992	0.00692	0.01583	0.00000	0.73684	0.52247	0.39776	0.05160	0.04354	0.00738	0.15164	0.09288
**Cuedc1**	0.01376	0.00794	0.01189	0.01039	0.00937	0.00666	0.00746	0.01297	0.01235	0.06154	0.01290	0.02766	0.01286	0.01166	0.00981	0.00750
**Cuedc2**	7.55359	4.73578	3.50994	1.70042	4.70628	6.13385	1.44213	3.90080	1.64371	0.85239	3.93616	0.25639	4.44931	0.00000	0.75281	4.22601
**Cul1**	6.17479	9.99482	5.52005	31.07637	8.28141	2.36264	3.78054	14.17083	16.93205	17.72787	9.22618	23.88669	5.59839	19.38545	23.07395	2.28710
**Cul2**	7.68793	10.73480	9.53151	0.72292	9.02482	2.70682	6.24743	12.07403	3.24689	9.95047	3.43111	7.39221	4.80842	10.86861	9.61838	0.62433
**Cul3**	9.93971	11.20227	9.38115	25.25543	13.27163	6.65095	7.81429	8.45716	31.04835	14.68836	9.12745	12.21258	5.74466	21.80185	21.65154	2.20293
**Cul4a**	2.58914	2.95307	5.39335	1.50965	5.11197	5.45680	0.00000	1.21389	1.00294	6.14566	3.66476	4.08065	2.68230	0.23942	3.22654	0.25288
**Cul4b**	7.34129	18.12663	11.49788	21.85994	10.33319	3.62675	11.13854	7.90578	19.98572	6.37720	4.30173	8.66620	5.98858	18.29670	16.30723	5.19482
**Cul5**	6.56448	5.52809	7.80611	2.36720	6.99645	1.44588	7.94431	19.12945	7.04995	20.60520	14.55160	16.21587	8.48724	34.07608	12.88213	0.87254
**Cul7**	7.03129	0.00000	0.00000	0.34608	3.63499	2.03946	1.57966	1.60270	0.00420	1.23734	0.00000	0.01999	0.00000	0.00562	0.05820	2.50872
**Cul9**	0.01213	0.00813	0.01107	0.76109	0.00916	0.00652	0.00730	0.04573	0.00000	0.52257	0.02330	0.02687	0.00929	0.00975	0.00195	0.00699
**Cuta**	11.26940	2.36200	8.52996	2.66333	6.23191	5.38591	2.89758	7.29257	0.83992	11.24362	5.28985	0.33654	13.36689	0.00000	0.53946	3.48240
**Cutc**	2.70707	2.99110	1.00369	0.40295	4.85128	0.01363	0.01554	1.44091	0.81274	1.21850	0.43960	2.67741	0.02204	1.30588	1.87424	2.60308
**Cux1**	0.58537	0.52616	2.41974	0.27701	3.51150	0.01315	3.15053	1.32200	0.00000	2.97183	1.62109	0.24553	1.35459	1.22595	0.38313	0.07084
**Cux2**	0.00000	0.26875	1.10656	2.73447	0.00578	0.00615	0.00456	7.84514	1.79088	4.33626	5.37993	0.26292	1.17603	0.55869	1.30875	0.00482
**Cuzd1**	0.01370	0.00814	0.01214	0.08056	0.00934	0.00665	0.00754	0.01391	0.33001	0.01358	0.01026	0.00770	0.01281	0.01263	0.00649	0.00739
**Cwc15**	17.08240	11.30289	11.45166	13.49402	5.56166	10.76444	7.35586	14.22390	22.39698	21.62742	20.42583	12.70871	13.57726	15.80191	17.42529	8.13249
**Cwc22**	8.83962	7.23721	9.87182	20.79755	1.48016	4.19330	3.42576	8.80547	15.96720	12.83938	8.07167	5.58399	8.06694	14.33931	14.84748	3.78547
**Cwc25**	0.48457	0.42900	1.48327	4.03839	1.40318	1.20075	0.07676	3.69931	8.22936	9.81698	2.60981	0.92380	4.20681	0.62179	5.48628	1.48360
**Cwc27**	7.21902	5.57868	1.89885	0.30383	10.68064	7.76135	4.12984	2.49984	0.00000	2.52950	1.51837	9.63263	7.43809	6.51838	2.48021	1.04703
**Cwf19l1**	6.99788	3.05123	5.62859	0.00807	0.00000	1.14075	6.31480	6.23376	3.21977	4.84368	1.23733	5.07297	4.04971	3.58257	2.75424	0.00863
**Cwf19l2**	0.00000	4.23098	0.58638	0.07861	1.49973	0.00758	2.05793	1.62989	1.08029	1.53281	0.62761	0.00000	1.59170	5.55925	1.03367	0.01146
**Cwh43**	0.01456	0.00670	0.00302	0.00375	0.00865	0.00799	1.45598	0.00000	0.01071	1.31511	0.78144	0.00000	0.00560	0.01394	0.00000	0.00702
**Cx3cl1**	0.01295	0.00778	0.01196	1.13859	0.00891	0.00634	0.00717	0.01441	0.00000	1.09512	0.01457	0.00577	0.01618	0.01878	0.00260	0.00691
**Cx3cr1**	0.01491	0.04995	0.04911	0.10610	0.02716	0.00750	0.00736	0.01154	0.17184	0.03657	0.01078	0.00648	0.04285	0.28963	0.05358	0.00736
**Cxadr**	1.76932	0.20682	1.31548	5.32248	0.67845	7.05513	1.72699	3.06306	15.08282	4.17343	12.19441	4.87578	3.18570	22.24447	9.06526	1.00125
**Cxcl10**	0.01299	0.00773	0.01186	0.52046	0.01881	0.00659	0.00748	0.01311	0.00444	0.01140	0.01304	0.00772	0.01265	0.01874	0.07606	0.00731
**Cxcl11**	0.01352	0.00807	0.01113	1.41804	0.00906	0.00632	0.00739	0.00977	1.26835	0.24496	0.06863	0.00473	0.01676	0.01061	0.01507	0.00679
**Cxcl12**	0.01338	0.00801	0.01211	0.00767	0.00904	0.00670	0.00736	0.01104	0.01177	0.72276	0.01204	0.00650	0.01190	0.00953	0.03069	0.00758
**Cxcl16**	0.00610	0.00782	1.54237	8.90660	0.00714	0.00842	0.00700	0.30156	5.60192	1.15683	0.00000	0.00000	0.01471	0.00839	0.10252	0.00531
**Cxcl9**	0.01737	0.00787	0.01576	0.19525	0.00906	0.00627	0.00738	0.01181	0.00822	0.00928	0.01180	0.03743	0.01271	0.02673	0.54981	0.02197
**Cxcr2**	1.11119	0.12238	1.22364	5.82578	0.67735	0.22588	0.18499	0.00000	10.67512	0.00000	9.35408	0.62105	0.51696	3.63373	1.04792	0.14034
**Cxx1a**	0.04561	0.00769	0.00990	0.06867	0.00823	0.00675	0.00742	0.01118	0.09659	0.14026	0.02740	0.02685	0.01239	0.01144	0.00691	0.00751
**Cxx1b**	0.02660	0.00769	0.01028	0.11412	0.00860	0.00673	0.00730	0.03164	0.11999	0.13761	0.01155	0.03550	0.01165	0.01105	0.00595	0.00762
**Cxx1c**	0.04567	0.00769	0.00948	0.03731	0.00824	0.00680	0.00744	0.01178	0.29752	0.07771	0.01040	0.04741	0.01281	0.01297	0.00564	0.00744
**Cxxc1**	6.87682	1.53290	7.02816	1.74820	4.57259	8.92525	3.50819	9.02553	4.20423	4.85821	2.82807	0.80514	10.90505	0.02165	1.07958	2.89560
**Cxxc5**	0.27251	2.31286	0.45166	0.13178	0.02532	2.82436	0.00576	0.78991	0.87889	0.77561	0.34263	0.00915	0.10795	0.00368	0.00000	0.01996
**Cyb561**	0.14079	0.00745	0.70658	0.07499	0.01542	0.00719	0.16598	0.00876	0.23740	1.47211	0.00978	0.02122	1.88234	0.03791	0.00000	0.00647
**Cyb561a3**	2.75240	1.26188	3.83860	4.84793	0.37792	1.64215	2.68592	7.04962	2.85888	2.40808	1.75289	0.90477	2.35086	1.36077	3.75816	0.04383
**Cyb561d1**	0.01594	0.00808	0.01621	0.00817	0.01029	0.00644	0.00730	0.01214	0.01182	0.26689	0.01061	0.01916	0.40504	0.00591	0.22963	0.00721
**Cyb561d2**	5.54430	2.12886	1.97351	0.34724	0.03151	0.16241	4.96371	2.09288	1.17532	0.59216	1.51450	3.41346	2.56384	0.04212	0.00000	0.05451
**Cyb5a**	19.76599	4.15569	8.35099	0.00000	14.42997	3.10551	0.00000	2.13796	0.79539	3.47935	2.06694	1.68497	4.99869	0.97138	0.80094	4.30594
**Cyb5b**	3.43164	4.31691	7.29002	1.14098	3.02206	0.01651	5.66790	0.00000	3.03708	14.05273	0.84893	0.43750	1.23694	7.59905	7.36564	6.00999
**Cyb5d1**	0.01749	0.00788	0.00574	0.11488	0.00901	0.00668	0.02701	0.00711	0.22625	0.00000	0.08673	0.00303	0.01145	0.93532	0.39018	0.00724
**Cyb5d2**	1.70434	0.16784	0.71443	1.37173	0.02944	0.58176	0.05400	0.14217	0.65279	0.44381	0.94571	0.05839	1.50150	1.40352	0.79971	2.24159
**Cyb5r1**	11.95660	5.16337	4.68469	0.00000	5.64379	7.89297	8.07311	3.73815	0.01056	0.00000	1.67521	0.14590	5.97353	0.52846	0.12753	8.85214
**Cyb5r2**	0.63307	0.05008	0.23862	0.52850	0.01047	0.00487	4.35161	0.01104	0.18599	0.27650	0.01053	0.02269	0.01531	0.00874	0.21178	0.00642
**Cyb5r3**	4.79528	4.73030	5.63567	1.33792	4.88099	14.23466	5.35395	1.64431	1.35575	6.39938	1.42878	0.17149	4.05758	0.00000	0.21442	3.90626
**Cyb5r4**	3.67466	0.25792	1.27449	6.53466	3.68454	4.02564	2.19451	5.59167	3.29415	10.35396	6.33001	6.67043	6.14865	12.09872	7.22444	1.55055
**Cyb5rl**	0.12738	0.00790	2.77534	0.09179	0.00986	0.00553	0.00712	0.00985	0.01015	0.04539	0.00934	0.02943	0.00605	0.00995	0.12951	0.01373
**Cyba**	0.00538	0.00709	0.12689	0.06028	0.00942	0.01870	0.00446	1.97264	0.01934	2.06875	3.97073	0.00000	2.41374	0.00146	0.09557	0.00583

**Cybb**	0.35348	0.00743	0.00081	0.00545	0.00718	0.00752	0.00692	0.01046	0.01010	0.14000	0.00796	0.21145	0.01292	0.00582	0.89914	0.00733
**Cybrd1**	0.02307	0.01886	0.14322	0.25424	0.03640	0.00680	0.06923	0.00752	0.14230	0.04543	0.36878	0.09181	0.10197	0.09493	0.05430	0.01477
**Cyc1**	19.12319	6.12586	9.48627	8.87916	16.71515	12.63904	6.88359	6.85958	0.15434	14.32385	9.45382	2.64599	16.29643	0.10062	2.66964	12.67696
**Cycs**	24.12400	10.03802	20.29714	9.13388	12.65995	10.05806	11.71109	15.27953	17.57166	18.58573	26.20862	17.06183	19.86145	14.13300	12.29358	8.77848
**Cyct**	2.00640	0.55007	0.44174	0.00246	0.00658	0.00954	0.00476	0.00000	0.01059	0.99326	1.84203	0.00000	0.00000	0.02217	0.18015	0.00481
**Cyfip1**	0.40386	2.70808	4.92368	5.05917	10.22950	5.70834	6.72406	4.41625	1.53311	6.15039	4.95607	11.32744	1.82153	3.87425	5.63274	3.59684
**Cyfip2**	1.91998	0.00569	0.00000	0.64003	0.00427	0.00576	0.00565	0.00001	2.27090	0.24623	0.00000	0.01531	0.01116	1.60252	0.00000	0.01171
**Cyhr1**	4.26358	2.44057	3.40852	3.23566	1.49255	3.67362	3.61088	0.61444	3.29406	2.14062	2.67785	0.02965	3.47524	0.06721	0.00000	1.84432
**Cyld**	0.28961	0.20342	0.69606	3.87959	0.23640	0.05531	0.22239	0.17297	4.06805	0.54590	3.84302	0.52796	0.23047	2.30792	1.99793	0.09978
**Cyp11a1**	0.01298	0.00757	0.25459	0.00472	0.00790	0.00570	0.00715	0.00913	0.01032	9.48099	0.00749	0.00000	0.00966	0.01610	0.00029	0.00724
**Cyp17a1**	0.00422	0.00820	1.95752	0.00512	0.00629	0.00586	0.00666	0.08855	0.18891	0.27852	0.00709	0.00645	1.47460	0.00619	0.46975	0.00613
**Cyp1a2**	0.01073	0.00792	0.01420	0.10450	0.00990	0.00715	0.00732	0.01200	0.00994	0.70947	0.01581	0.00516	0.53114	0.01212	0.01143	0.00803
**Cyp1b1**	0.01368	0.00766	0.01198	0.35775	0.00918	0.00662	0.00734	0.01125	0.00658	0.27893	0.01174	0.00636	0.01261	0.00918	0.14061	0.00774
**Cyp20a1**	3.63065	0.07659	2.59311	1.89562	0.23630	1.14790	0.65288	5.76104	0.75248	3.13551	5.55361	0.41111	2.17288	1.90710	2.30197	0.30576
**Cyp26a1**	4.86620	0.00263	0.00367	0.00838	0.00000	0.00433	0.00373	0.00351	0.02631	0.23778	0.01098	0.00532	0.01139	0.01176	0.01227	0.00231
**Cyp26b1**	0.01364	0.00777	0.01007	0.11062	0.00921	0.00671	0.00723	0.10817	0.00998	0.01310	0.01300	0.01739	0.00856	0.01173	0.00652	0.00736
**Cyp2ab1**	0.01402	0.00791	0.00799	0.35738	0.00905	0.00676	0.00741	0.00031	0.13020	0.00000	0.60057	0.01409	0.00670	0.00418	0.22877	0.00700
**Cyp2b10**	0.01848	0.00749	0.01013	0.09974	0.00861	0.00643	0.00705	0.00985	1.21727	0.00000	0.00000	0.02557	0.54416	0.02717	0.10761	0.00684
**Cyp2b13**	0.05155	0.00767	0.00989	0.01074	0.00803	0.00672	0.00748	0.01228	0.01232	0.01314	0.01258	0.00801	0.05845	0.01298	0.02111	0.00724
**Cyp2b23**	1.54224	0.00596	0.00361	0.02666	0.00499	0.00675	0.00625	0.01301	0.01061	0.01872	0.01202	0.01674	0.01821	0.01106	0.01524	0.02018
**Cyp2c44**	0.57462	2.25323	0.00000	0.94432	0.03616	0.01154	0.00560	0.05947	0.79622	0.50842	0.25925	0.03833	0.19954	0.86655	2.79799	0.00616
**Cyp2c55**	0.01391	0.00775	0.00952	0.00903	0.00931	0.00679	0.00732	0.01123	0.26826	0.00913	0.00969	0.21789	0.01316	0.01288	0.00324	0.00745
**Cyp2d10**	0.01951	0.00822	0.01065	0.01894	0.00881	0.00687	0.00784	0.05333	0.01109	0.03081	0.01196	0.00882	0.01074	0.01247	0.01051	0.00742
**Cyp2d12**	0.01047	0.00818	0.00948	0.03914	0.00957	0.00707	0.00767	0.17095	0.03577	0.06100	0.02292	0.02022	0.01719	0.01214	0.00784	0.00742
**Cyp2d22**	0.01329	0.00778	0.01119	0.00631	0.00891	0.00672	0.00725	0.00757	0.01146	1.05681	0.09717	0.01445	0.01091	0.01134	0.00679	0.00766
**Cyp2d40**	0.01305	0.00826	0.01128	0.09752	0.00930	0.00670	0.00770	0.02787	0.49456	0.02240	0.02679	0.01853	0.01173	0.01245	0.00480	0.00746
**Cyp2d9**	0.00803	0.00782	0.00735	0.07200	0.00941	0.00722	0.00772	2.21698	0.06423	0.73472	0.00000	0.01427	0.00571	0.01274	0.00000	0.00740
**Cyp2e1**	0.39454	0.02848	0.47283	3.25907	0.29624	0.06508	0.07731	0.00000	4.90283	0.00000	3.92583	0.28541	0.11049	1.29955	0.21132	0.16236
**Cyp2f2**	0.00789	0.00769	0.04543	0.17769	0.00896	0.00588	0.00746	0.39315	0.15174	2.09760	0.00436	0.04896	0.00607	0.00491	0.81943	0.00753
**Cyp2j13**	0.01909	0.00794	0.09400	0.12040	0.00858	0.00618	0.06031	0.04484	0.12504	0.08507	0.03913	0.29381	0.00937	0.13309	0.18694	0.05096
**Cyp2j6**	0.48153	0.00746	0.00542	0.01062	0.00688	0.00711	0.00712	0.01438	0.01235	0.01534	0.01280	0.00778	0.01215	0.01229	0.01080	0.00680
**Cyp2s1**	0.30099	9.62306	17.52741	2.13716	2.59319	12.86903	8.54753	25.74402	4.97392	7.58433	19.66168	5.21529	27.71331	2.57340	1.47803	8.00525
**Cyp2t4**	0.15213	0.00689	1.13106	0.00892	0.00593	0.01873	0.00665	2.32389	0.09928	0.00000	0.84019	0.00533	0.20578	0.00260	0.28932	0.00691
**Cyp39a1**	0.01317	0.00765	0.01007	4.47119	0.00912	0.00585	0.00709	0.00926	1.74977	0.39540	0.01053	0.00169	0.01129	0.06861	0.95613	0.00626
**Cyp46a1**	0.01126	0.00838	0.01778	0.93828	0.00966	0.00641	0.01808	0.01350	0.78776	0.00129	0.04085	0.00689	0.42359	0.01167	0.00154	0.00705
**Cyp4f13**	0.00662	0.00800	0.01257	0.01018	0.00906	0.10882	0.00638	0.00897	0.01247	0.07433	0.01159	0.00659	1.95809	0.01133	0.01076	0.00630
**Cyp4f16**	2.99189	0.00395	0.00301	0.02818	0.00363	0.00510	1.37352	0.00000	0.12346	0.46228	0.01050	0.00342	0.01644	0.01262	0.01519	0.00389
**Cyp4f17**	0.01678	0.01795	0.00836	0.01036	0.00889	0.00683	0.60325	0.01027	0.01234	0.01571	0.01281	0.01719	0.01797	0.01173	0.01125	0.01449
**Cyp4f37**	0.07395	0.00789	0.00830	0.01040	0.00788	0.00688	0.10987	0.01329	0.01243	0.05209	0.01218	0.00775	0.01171	0.01145	0.01077	0.00707
**Cyp4f39**	2.32940	0.00532	1.46984	1.20710	0.03325	2.93229	0.00303	0.00000	0.31178	0.00000	0.07921	0.01278	3.55774	0.02525	0.04755	0.01520
**Cyp4f41.ps**	0.01297	0.00810	0.08584	0.01058	0.00989	0.01629	0.01834	0.01563	0.01232	0.01291	0.01472	0.00773	0.08108	0.01257	0.00910	0.00767
**Cyp4v3**	0.01268	0.00765	0.00728	0.41108	0.00946	0.00672	0.00730	0.01045	0.00510	0.22410	0.01706	0.00354	0.01272	0.82994	0.55758	0.00737
**Cyp51**	3.48902	4.62058	5.36164	2.59686	0.07513	0.00900	6.11258	2.54603	1.44664	0.38092	1.20389	0.11565	3.08019	2.78458	0.00000	2.45049
**Cypt3**	0.01369	0.00805	0.01132	0.50437	0.00933	0.00652	0.00740	0.01215	0.45840	0.00500	0.00958	0.03964	0.01275	0.00694	0.22513	0.01452
**Cyr61**	0.99420	0.00511	0.10970	0.18186	0.00564	0.00807	1.58550	2.12954	0.31557	0.03529	0.52955	0.12040	0.98985	0.39906	1.14076	0.11105
**Cys1**	0.01373	0.00782	0.01196	0.00963	0.00919	0.00654	0.00737	0.01156	0.01213	0.21270	0.01435	0.01866	0.01151	0.01884	0.00740	0.00715
**Cysrt1**	0.00961	0.00804	0.00729	0.01030	0.00912	0.37278	0.00689	0.01178	0.01230	0.02866	0.01148	0.00836	1.12905	0.01198	0.01197	0.00668
**Cystm1**	7.23400	2.89962	6.05793	1.00584	1.74983	7.02509	4.42643	7.08802	0.00030	7.72017	3.27713	1.13078	7.42721	0.01793	0.08304	3.66842
**Cyth1**	0.00935	0.00794	0.05646	0.00554	0.00871	0.00749	0.00730	0.01199	0.35775	0.06278	0.00929	0.00735	0.35385	0.01227	0.02114	0.20891
**Cyth2**	7.94995	1.08754	9.42143	5.43711	5.83780	4.62306	4.02075	14.13113	4.43791	13.99324	12.76005	1.72237	5.75554	4.54433	9.21006	2.68515
**Cyth3**	2.17129	0.00420	0.43106	3.79291	0.00741	0.00419	0.00439	2.13218	0.00000	1.39675	0.62377	3.77048	0.52389	1.78906	2.56044	0.00416
**D10Jhu81e**	4.00526	0.00277	1.61658	0.28878	0.20954	4.51718	0.17054	0.85513	0.30701	1.01136	0.56515	0.00000	0.00000	0.00492	0.00000	2.12096
**D10Wsu102e**	6.88131	7.71714	3.02926	14.59020	8.71904	4.35617	4.74777	10.40243	13.28943	11.01004	4.45757	4.57092	6.08945	5.23422	14.65361	6.53787
**D11Wsu47e**	2.89621	0.05037	5.02759	3.73615	4.39488	0.06585	0.00175	0.00000	4.56698	3.13344	2.56786	0.36896	2.56164	2.50249	2.85947	0.43889
**D16Ertd472e**	6.83052	0.00000	3.97094	13.08563	0.03320	0.00315	0.84914	2.12757	7.67345	5.29105	0.00000	0.30320	1.70226	0.24322	8.66604	0.96784
**D16Ertd519e**	0.01375	0.00831	0.01234	2.84991	0.00932	0.00640	0.00743	0.01392	0.00000	0.01445	0.01353	0.00845	0.01263	0.00947	0.00269	0.00693
**D17Ertd648e**	0.14352	0.16195	0.44872	43.74434	0.03635	0.16401	0.32430	2.98255	44.52124	12.14586	4.98029	3.23232	1.81080	11.12885	19.09024	0.13194
**D17H6S53E**	0.29330	0.04825	0.30617	1.10106	0.07082	0.03713	0.10794	0.00000	1.19814	1.13195	3.72308	0.07985	0.44699	0.70976	0.33173	0.11363
**D17H6S56E.5**	1.92132	0.87733	5.53989	6.21742	1.35921	1.98551	4.28205	2.94172	4.98239	6.06878	4.32942	1.77397	1.57512	2.57902	7.07143	0.70754
**D17Wsu92e**	5.78831	3.15117	6.33534	14.25972	2.40075	4.82375	4.76889	9.34657	14.93627	9.14917	5.25171	0.44508	10.45192	0.56016	5.79319	3.87348
**D1Ertd622e**	1.44552	0.00358	0.86270	17.20015	0.86121	0.01228	0.00485	0.00000	5.21400	1.34001	0.00000	6.07853	0.01556	0.01591	6.09825	0.00185
**D1Pas1**	0.00745	0.00577	0.53472	7.45242	0.00784	0.00394	0.00616	0.00817	1.29545	0.00000	0.00000	7.06371	0.02331	1.06020	1.85749	0.01255

**D2hgdh**	3.01636	2.67091	1.93248	0.53329	0.66126	0.01627	2.87575	1.49474	2.30258	0.00000	1.70879	0.05484	1.77918	0.00000	2.00782	1.40736
**D2Wsu81e**	6.73154	6.02661	8.53523	3.00036	4.75427	4.39555	1.54490	6.05905	3.45696	5.31866	0.87137	0.39779	1.89236	0.00000	0.69835	6.22938
**D3Ertd254e**	0.41687	0.98122	1.02546	2.49339	0.19090	1.29138	0.05727	1.96981	4.64629	0.40771	4.16708	0.35558	0.07372	5.05752	5.94876	0.03370
**D3Ertd751e**	0.01253	0.00786	0.01250	0.28160	0.01023	0.00710	0.05028	0.01374	0.00749	0.00528	0.01203	0.04676	0.20527	0.01562	0.31253	0.00753
**D4Ertd617e**	0.01376	0.00812	0.01205	0.34268	0.00931	0.00658	0.00745	0.01294	0.00559	0.02296	0.01400	0.00855	0.01243	0.03312	0.04962	0.00726
**D5Ertd577e**	0.42563	0.33202	0.57261	4.72961	0.15176	0.10578	0.35729	1.43231	11.36017	4.19790	4.86464	7.32662	1.84238	8.01913	4.67476	0.00289
**D5Ertd579e**	1.48002	1.41246	0.29245	0.41828	0.05351	0.13061	0.09280	1.20315	0.69993	1.40225	0.15703	0.07854	2.44440	0.00438	0.28055	3.44443
**D5Ertd605e**	0.02060	0.03790	1.56276	0.75475	0.00577	3.14678	0.22834	0.93767	1.81228	2.54278	1.66442	0.10103	0.00000	0.08682	1.72777	0.71929
**D5Ertd615e**	0.08825	0.06901	0.16821	0.42297	0.01595	0.03055	0.11896	0.24991	0.61050	0.35185	0.12971	0.10112	0.08102	0.73886	0.38898	0.15070
**D6Ertd474e**	0.01053	0.00751	0.00312	11.47199	0.00902	0.00483	0.00665	0.00563	1.81219	1.65990	0.07257	0.09845	0.00997	0.88932	4.90679	0.00499
**D6Ertd527e**	0.01257	0.00802	0.01090	2.22501	0.00977	0.00700	0.00758	0.00910	3.02156	0.49524	0.12909	0.06529	0.09563	0.00388	0.06211	0.00714
**D6Wsu163e**	2.20954	3.10680	2.81327	0.00000	0.02095	0.00358	0.02186	4.96967	0.51946	6.97058	5.82705	8.81389	0.23393	3.79597	8.75587	0.84353
**D7Ertd143e**	1.85888	0.20271	2.46366	1.07770	0.01244	0.00376	0.18585	2.48473	3.47269	0.55499	1.89647	0.03430	0.16740	0.01122	0.00000	2.15755
**D7Ertd443e**	0.00358	0.00763	0.00429	0.17286	0.00920	0.00564	0.00690	0.01417	0.81440	3.45364	0.00000	0.06007	0.01022	7.98934	14.18706	0.00692
**D8Ertd738e**	16.65863	0.83977	5.89743	13.68134	4.23883	5.06671	3.39836	11.98944	27.21024	23.37469	17.20754	2.56554	14.11756	0.24577	0.00000	6.19487
**Daam1**	2.81849	0.03294	2.34052	5.19894	3.59581	0.74691	0.92334	1.60415	3.03246	1.37112	0.95643	0.12231	0.33515	0.01240	0.49357	3.67257
**Dab1**	0.01246	0.00801	0.31073	0.28737	0.00873	0.00553	0.00760	0.01330	1.18194	0.00632	0.00010	0.00589	0.00975	0.00618	0.13470	0.00705
**Dab2**	0.76076	6.21336	11.20358	5.46799	2.73615	20.93419	7.97230	12.92800	2.14252	8.84365	4.37752	4.74108	23.01347	4.46185	0.00000	14.09656
**Dab2ip**	1.02454	0.20553	0.84800	0.00000	0.82991	4.65665	3.17677	2.46870	0.00868	0.00000	0.26027	0.00746	1.86732	0.01860	0.03723	1.49539
**Dact1**	0.01357	0.00764	0.01181	0.95381	0.00920	0.00645	0.00729	0.01090	0.00000	0.27145	0.01093	0.00515	0.01266	0.00342	0.64263	0.00764
**Dact3**	0.00368	0.00646	0.00732	0.15351	0.00948	0.00649	2.01382	0.00731	1.44086	1.03560	0.01163	0.00860	0.75447	0.00594	0.26641	0.00685
**Dad1**	7.72040	3.66655	11.26886	3.33680	2.89289	4.97324	4.43925	5.44899	4.76559	9.28476	9.80676	2.29109	10.67419	0.64318	2.37023	7.93270
**Dag1**	3.36320	0.00000	7.04317	4.79283	0.07134	8.49163	7.02098	4.60090	3.60300	5.98544	2.16097	0.46827	9.72176	0.49199	1.26990	4.74521
**Dagla**	0.00836	0.00814	0.01788	2.11897	0.00790	0.00686	0.00735	0.01376	0.00000	0.00933	0.01293	0.00838	0.00854	0.01034	0.00261	0.94348
**Daglb**	0.39240	0.00699	0.00253	0.57965	0.00771	0.00781	0.42468	0.00000	2.00807	0.00000	0.70335	0.01642	0.12479	0.00196	0.53395	0.00671
**Dalrd3**	7.04980	1.01514	3.90197	0.19331	2.98325	4.74036	1.56260	8.49728	0.10613	7.54285	1.19241	0.55671	9.63167	0.18968	0.69063	2.75051
**Dancr**	1.39070	2.19425	1.10493	6.39092	1.20525	0.04723	0.00196	0.00000	11.50286	4.27336	4.37532	0.29526	2.73053	0.12211	0.27120	0.01560
**Dand5**	0.06765	0.00789	0.00833	1.71484	0.02603	0.00664	0.00744	0.01245	1.88640	0.01249	0.00000	0.01604	0.02639	0.01169	0.00406	0.00695
**Dap**	10.72314	0.67819	6.42222	0.15676	13.02075	15.84468	6.45023	13.60426	0.00000	1.03428	8.69624	0.20049	14.50099	0.08611	0.00000	4.57863
**Dap3**	16.87388	6.98262	15.35341	3.41243	7.70803	8.65787	5.94878	9.93261	6.26829	9.49033	5.72961	7.20419	12.59502	0.34350	4.01028	8.37549
**Dapk1**	0.00871	0.00726	0.13349	0.00264	0.00970	1.27675	1.52999	0.01579	0.01129	2.30431	0.00601	0.04200	1.09937	0.17587	0.00000	0.00639
**Dapk2**	0.49920	0.00749	0.00537	0.10014	0.00687	0.00712	0.00710	0.01439	0.00760	0.01538	0.01213	0.00783	0.01214	0.01181	0.00902	0.00677
**Dapk3**	0.03104	0.01945	0.01136	0.00621	0.01467	0.00724	0.00754	0.05571	0.42426	0.01545	0.01135	0.00684	0.16888	0.01772	0.02765	0.00769
**Dapp1**	0.02765	0.00846	0.00791	0.31959	0.01240	0.00721	0.00747	0.00599	0.00439	0.01145	0.60706	0.00531	0.01047	0.00740	0.21639	0.02226
**Dars**	17.35898	11.32103	12.28751	4.57070	9.88929	7.98795	11.08525	15.82302	4.23720	10.58650	11.03977	12.83871	11.60180	8.95634	15.10284	4.68761
**Dars2**	3.44299	0.07376	5.87264	1.34208	5.68849	0.38423	3.93307	5.97760	2.53693	3.85444	4.19764	6.51857	0.94172	3.26415	5.43865	4.23389
**Daw1**	0.01352	0.00767	0.01142	1.15230	0.00928	0.00627	0.00738	0.01164	1.85507	0.00400	0.00000	0.00358	0.01289	0.00566	0.40957	0.00697
**Daxx**	1.55132	0.19446	6.15207	1.21449	4.68666	0.38881	3.40973	3.95441	2.83853	7.99721	0.29789	3.02017	3.34245	0.08121	0.89433	3.71247
**Dazap1**	5.58183	0.00000	0.00000	1.92968	0.57108	0.40103	0.03055	1.37619	2.88997	2.65348	2.55701	0.06161	2.68677	0.00000	0.00000	3.45374
**Dazap2**	10.46800	6.74107	13.73750	13.14970	7.39977	5.21511	7.20003	15.42236	11.27516	18.79587	8.71039	8.61788	12.24933	13.34683	11.68230	5.50391
**Dazl**	0.00000	0.01401	0.00000	3.27234	0.00800	0.00325	0.00406	0.00000	0.65793	2.69960	3.18678	15.02005	0.26693	2.06899	1.19538	0.06143
**Dbf4**	4.10957	5.71788	1.61941	20.07050	6.69343	2.73182	3.17019	7.97282	9.44300	12.08690	7.84321	5.26080	3.22051	16.99764	26.27574	0.57486
**Dbhos**	0.22487	0.09191	1.00745	6.69646	0.00677	0.28643	0.11808	3.55080	11.12779	3.77978	2.93956	1.75075	0.75821	2.63351	1.58569	0.13964
**Dbi**	6.76364	0.23002	2.86855	9.46911	7.60828	1.10039	1.13975	0.66671	4.60589	1.20207	2.61373	0.38466	1.05663	0.13626	2.78301	3.59275
**Dbil5**	0.05002	0.01920	0.00832	0.77633	0.00799	0.00674	0.00739	0.00748	0.00000	0.00000	0.31435	0.00715	0.00885	0.00617	0.10112	0.00697
**Dbn1**	0.00838	0.00747	0.00854	0.00629	0.00934	0.00678	0.00711	0.46822	0.02488	1.43997	0.00923	0.00308	0.00593	0.01799	0.00000	0.00729
**Dbndd1**	0.01323	0.00798	0.01169	2.25995	0.00945	0.00645	0.00748	0.01112	1.76630	0.43540	0.01626	0.03331	0.02042	0.00499	0.17582	0.00681
**Dbndd2**	0.00000	0.00726	0.14101	3.20091	0.21549	0.02817	0.00646	0.03153	4.04664	1.79118	0.45582	0.00000	1.16762	0.00000	0.66362	0.52520
**Dbnl**	9.77777	2.82533	7.25154	2.91096	7.66356	6.88386	7.16037	15.99727	7.62116	13.04522	9.48299	3.72457	10.35150	3.47408	3.23285	6.53106
**Dbp**	0.61946	0.00732	0.00497	0.00961	0.00780	0.00704	0.00693	0.01025	0.01226	0.22458	0.01254	0.00688	0.01153	0.01228	0.01012	0.00675
**Dbr1**	4.81447	2.02760	0.60512	4.09788	2.24514	4.02648	1.14514	0.25515	2.40833	1.95037	2.76080	0.04926	3.04639	1.55974	4.77339	1.78060
**Dbt**	1.37029	4.94282	0.67403	2.89637	1.58953	1.64512	0.93400	2.40092	5.42397	1.02252	4.94984	3.50064	1.86978	14.11866	9.60821	0.13194
**Dcaf10**	6.80564	0.07008	3.84500	7.56848	0.07133	2.25343	4.36448	1.00014	13.82421	4.63064	5.22235	8.05234	1.65301	14.33913	8.03916	0.78112
**Dcaf11**	1.61418	9.20552	6.38645	4.47366	6.03182	6.92018	8.93821	6.40599	7.97359	10.22086	3.76146	2.32869	4.07698	5.03271	7.15269	1.17307
**Dcaf12**	10.40354	5.45269	7.23862	11.04336	5.57235	7.60674	4.97519	8.80430	17.56789	8.58953	8.02198	7.13162	4.41450	4.60577	11.96911	7.27932
**Dcaf12l1**	14.32307	0.00001	1.68908	0.00000	12.31357	0.00000	0.00510	1.79070	1.85125	0.84472	0.00000	0.00000	1.21805	0.51317	0.25742	0.00000
**Dcaf13**	8.31604	12.02302	16.31693	13.95350	12.59954	3.01628	10.25063	19.98132	16.34550	24.92348	17.98626	17.27528	10.77086	27.55864	19.95457	9.15690
**Dcaf15**	4.87243	1.28925	1.14748	0.09182	1.59059	1.58066	1.31732	8.25949	0.24256	7.09794	1.04593	0.76557	7.24267	0.00446	0.00000	3.06930
**Dcaf17**	2.09965	0.03837	2.61586	4.69778	0.95166	0.06639	0.11072	1.58311	6.98757	2.51869	6.28013	0.39329	1.76927	2.77106	4.88938	2.89332
**Dcaf4**	9.40015	5.47953	12.55688	3.36240	7.23822	5.52537	7.86172	21.94536	2.90311	27.27243	14.49316	1.62496	7.33634	0.02115	2.55260	5.59317
**Dcaf5**	1.19530	0.01275	0.58337	7.14824	4.39432	0.07143	2.67161	3.36231	16.40348	6.29291	2.67333	0.54280	0.22621	2.40607	5.70865	1.77956
**Dcaf6**	0.01209	0.00832	0.00733	0.67680	0.00964	0.00629	0.00777	0.03045	1.31512	0.00000	0.00000	0.02037	0.01534	0.22172	3.23400	0.00720

**Dcaf7**	10.32057	6.77109	6.44486	7.14595	4.10115	2.48231	5.44116	4.10588	12.58577	9.35129	6.70552	1.44888	5.38452	5.28655	7.83644	0.23561
**Dcaf8**	8.63099	6.29939	3.15580	11.00764	3.61715	1.27747	3.90559	4.45397	11.71869	4.94246	4.34236	0.52173	3.40336	3.23762	5.13383	3.54303
**Dcakd**	3.50646	0.48629	2.15752	5.45393	1.97644	0.01312	4.52283	2.35619	6.77941	1.70976	1.88168	0.20528	3.11688	0.01209	0.00000	3.34998
**Dcbld1**	5.48117	4.91018	2.94099	4.13521	6.02220	3.66280	5.74230	10.95788	4.30229	4.87993	5.99467	1.85788	4.36910	2.64591	2.88845	5.37206
**Dcbld2**	0.58198	0.07307	0.00000	4.27971	0.00660	0.00597	4.56607	2.02724	1.98428	0.00000	3.43875	0.05578	0.83791	2.96565	1.71759	0.00494
**Dcc**	0.01587	0.00792	0.01174	3.68089	0.00891	0.01321	0.00699	0.00932	2.15035	0.00000	0.00000	0.00000	0.12838	0.38511	1.26212	0.00636
**Dcdc2a**	0.02574	0.00770	0.01845	2.09175	0.01937	0.00743	0.01711	0.01900	0.27447	0.00000	0.03329	0.02148	0.01780	0.71550	0.51344	0.02156
**Dcdc2b**	0.62304	0.19942	0.66422	1.09228	0.01594	0.55644	0.18755	0.38212	1.36172	0.49997	0.72296	0.04624	0.38577	0.08524	0.16894	0.17300
**Dcdc2c**	0.68322	1.42497	2.18007	67.96443	1.03069	0.98758	1.17714	6.13381	65.23173	45.50991	15.23888	18.82159	2.45764	40.49353	49.25253	0.29428
**Dck**	0.80825	2.27413	2.11906	4.16221	1.05057	3.14865	0.56560	3.86926	7.20904	3.10319	0.90692	5.57463	2.24012	7.95697	6.59299	0.02281
**Dclk2**	0.65970	0.00660	0.43454	10.92122	0.00567	0.00343	0.00553	0.00000	5.38601	1.15437	1.16554	0.09038	0.20466	0.00000	0.10801	0.84427
**Dclk3**	0.01778	0.00761	0.01136	1.64300	0.00908	0.00632	0.00730	0.01164	0.01049	0.00592	0.01122	0.00611	0.01278	0.00579	0.72497	0.00705
**Dclre1a**	0.96415	0.00653	0.71415	1.41618	0.40415	0.00706	0.00643	0.30456	0.03221	0.64199	0.42902	0.02002	0.67337	1.25195	0.76414	0.00554
**Dclre1b**	0.26099	3.22941	0.61541	8.08987	2.87296	0.73302	0.35416	1.70331	7.93873	1.69217	5.57089	0.38114	1.16917	4.77849	7.68241	1.85664
**Dclre1c**	3.46123	0.10065	3.75611	9.85596	3.95761	0.17865	1.48167	1.39502	16.43417	2.89523	16.29635	6.73677	0.89985	10.56202	1.28498	0.52891
**Dcn**	0.01168	0.00775	0.01212	0.00503	0.00844	0.00650	0.00723	0.00940	0.01080	4.82481	0.00896	0.00565	0.01023	0.01421	0.00502	0.00729
**Dcp1a**	0.00000	5.75385	4.41710	9.79235	0.58935	0.30412	5.28720	2.21559	3.81013	2.86063	2.45482	0.25523	3.49088	0.40139	3.55954	3.58855
**Dcp1b**	0.01366	0.00823	0.01174	0.15721	0.00928	0.00671	0.00757	0.01353	0.52644	0.01881	0.03578	0.00768	0.01243	0.01188	0.00455	0.00735
**Dcp2**	2.40916	2.57224	6.65938	8.37524	1.83934	2.87438	2.89892	1.37243	3.62511	3.70492	2.51474	5.62695	0.68842	1.61751	9.05369	4.77683
**Dcps**	12.11859	3.68036	2.74701	1.48997	0.00000	0.00000	0.00000	0.28336	2.58204	1.08972	1.47600	0.09893	3.67420	0.10663	0.91245	2.50284
**Dcst1**	0.01373	0.00790	0.01239	0.03507	0.00941	0.00656	0.00745	0.01305	0.01276	0.07588	0.01372	0.00738	0.01268	0.01048	0.01404	0.00747
**Dctd**	9.71688	0.02437	12.17745	5.39802	11.27555	14.13009	6.57956	8.10886	3.36757	23.84396	6.25333	12.51220	12.80600	13.61043	16.45874	3.02272
**Dctn1**	6.23670	2.62294	2.73509	0.62827	5.17880	4.21872	0.33490	4.83558	0.33018	3.74133	2.14973	0.11427	7.37235	0.00015	0.00000	2.57809
**Dctn2**	16.75911	4.47808	6.30702	1.22265	6.50571	9.91433	3.49719	9.18257	0.19902	6.55392	8.20719	4.88213	15.35743	0.82319	1.07557	10.16159
**Dctn3**	8.55301	1.91272	9.86405	2.64430	9.57550	3.69785	2.78588	3.12704	1.52041	9.39709	4.58706	0.21295	5.67495	0.01254	0.09257	7.64553
**Dctn4**	1.46902	3.48362	3.25133	3.81050	1.61388	0.00422	0.16358	3.47258	1.81904	3.50077	4.46698	1.43700	1.35949	10.81241	2.42272	0.03900
**Dctn5**	8.84487	2.19793	7.07733	0.71928	3.85352	3.01444	0.00464	7.18574	1.49058	8.30418	4.35154	1.06320	4.56354	8.01378	7.13635	2.71382
**Dctn6**	9.23940	8.09134	5.27959	0.80576	3.19906	2.99503	2.64427	5.62262	0.59887	8.82243	4.11620	4.29358	1.88663	0.54606	1.10584	2.84291
**Dctpp1**	12.38967	0.61666	4.24477	3.03694	7.11495	2.96843	1.22095	5.96859	2.21773	5.87118	9.51850	0.66201	9.15232	0.00451	0.38014	8.07895
**Dcun1d1**	2.01724	7.60581	4.31353	11.05096	2.65105	0.06168	0.42407	2.37986	1.66056	0.00000	0.73897	0.03761	3.59315	4.44359	10.51574	1.43540
**Dcun1d2**	0.14444	0.00720	2.13475	8.56976	0.01268	1.25657	0.00645	2.69618	3.30506	3.05132	0.17458	0.43013	0.13639	0.07270	4.24622	0.81942
**Dcun1d3**	1.49777	3.08497	1.82850	5.29228	0.02666	0.02394	0.00183	0.12584	6.56158	5.77280	3.40495	5.51523	0.85777	13.50085	12.00226	0.01598
**Dcun1d4**	1.82674	0.00567	0.00000	0.72039	0.00627	0.00537	0.00575	0.15914	0.00000	0.41411	0.00000	0.21402	0.01959	2.42681	0.21686	1.32581
**Dcun1d5**	6.81900	4.57623	5.62642	21.49592	6.76805	5.25710	5.79556	4.24515	18.38724	9.58655	10.24301	6.87242	5.49770	7.48557	2.33978	2.23533
**Dcx**	0.02998	0.01800	0.02063	0.03633	0.02613	0.02625	0.01770	0.04234	0.05832	0.08118	0.06801	0.04792	0.03040	0.00955	0.06139	0.01433
**Dcxr**	0.26539	1.04926	0.30659	0.22918	2.37908	0.00733	1.16042	0.93392	0.00206	4.13721	1.86085	0.00000	1.05887	0.00980	0.00000	3.07364
**Dda1**	3.95130	0.84290	0.95935	11.53958	8.16040	1.39941	3.93458	5.73486	9.07930	8.62614	1.02505	4.81583	8.55121	0.46768	1.60507	6.30013
**Ddah1**	9.54872	6.37094	5.81273	0.00000	3.38862	8.29773	5.78926	5.14593	0.01157	5.17927	5.10486	0.61565	9.86025	13.91019	1.67762	8.20931
**Ddah2**	2.79139	0.00101	1.86024	0.00000	3.09563	0.00914	1.79462	2.32564	0.00587	4.87818	3.63504	0.08172	2.91711	0.00384	0.00265	3.39266
**Ddb1**	22.85531	15.07117	14.85991	15.49082	19.26063	8.35645	16.17068	15.27051	11.19777	19.01745	21.03875	4.31457	22.67748	25.36959	17.89828	7.63779
**Ddb2**	0.00000	0.00744	4.04961	0.00000	0.00469	0.33427	0.26727	2.05921	0.00558	2.57966	0.00000	0.41841	2.72059	2.68270	3.19725	2.27627
**Ddc**	0.65981	0.00801	0.19833	0.01273	0.01357	0.00499	0.02988	0.08474	0.81230	0.00499	0.00000	0.15380	0.00785	0.02273	0.10412	0.00667
**Ddhd1**	4.86337	0.30712	4.34103	5.88645	1.05805	0.00000	3.00002	1.25030	3.73864	1.32982	0.28216	0.22970	0.16290	0.00000	1.65444	0.04153
**Ddhd2**	4.45927	3.84256	3.44495	7.53040	0.02846	0.05030	4.50832	4.93760	3.78156	7.38519	3.83117	0.58298	3.69815	4.74163	7.19824	2.22245
**Ddi2**	0.02308	0.43516	0.01462	0.00106	0.02580	0.67330	0.71995	1.40944	0.74915	0.28142	0.00000	0.02239	0.04673	0.00535	0.33702	0.00689
**Ddias**	3.77945	4.74648	1.84716	11.83893	0.16698	0.45737	7.17639	7.28475	21.98094	15.33995	10.04222	13.50275	0.65335	24.78846	24.75799	2.91187
**Ddit3**	0.02819	0.02625	0.35019	0.30108	0.00770	1.80337	2.87221	1.44668	3.00163	2.41196	0.87765	0.87267	1.71139	0.00204	0.00000	1.36715
**Ddit4**	0.30171	0.00603	0.21963	2.18581	0.00698	0.00471	0.10475	2.07358	4.79505	4.22655	5.86979	0.17356	0.01782	1.12945	1.43911	0.01997
**Ddit4l**	1.01005	0.00711	0.48225	10.98536	0.01669	0.00224	0.00492	1.48480	13.72009	0.41740	1.74348	0.48729	1.22252	0.11850	0.00000	0.00266
**Ddo**	0.11265	0.00835	0.03443	0.36874	0.01224	0.01774	0.00748	0.02510	0.08910	0.08591	0.08730	0.00757	0.04209	0.00870	0.01589	0.00728
**Ddost**	9.67740	0.62307	15.83349	1.10880	5.74789	7.79508	4.88152	9.89764	0.25199	6.43002	3.66337	0.54325	7.76484	3.96388	1.68540	8.86185
**Ddr1**	0.58896	0.00511	0.02789	2.36046	0.01642	0.00503	3.35418	5.04614	1.20559	4.10923	5.43676	0.12911	0.49161	0.51886	1.13755	0.01268
**Ddr2**	2.64870	0.00381	0.00000	8.82837	2.53326	0.00780	0.00382	0.30099	3.74229	4.87814	2.76258	0.06178	0.19016	12.83294	2.19431	3.92305
**Ddrgk1**	8.46105	2.51948	9.01917	0.53740	10.25886	0.25913	4.01404	4.55122	0.35297	4.77475	3.76677	0.15713	10.50822	0.05424	0.00000	4.68430
**Ddt**	9.42108	2.77308	2.66267	2.88345	3.06333	0.87433	1.43979	1.80052	6.07432	3.42488	1.42072	0.10709	2.36279	0.00000	0.36961	4.90592
**Ddx1**	13.39767	13.29372	7.48901	2.33968	8.79024	8.48823	7.02793	12.46275	1.43068	6.06036	7.73721	5.56817	6.65296	7.42108	10.22108	10.40551
**Ddx10**	5.52396	8.07757	5.08731	3.57338	9.54087	0.00000	4.49514	6.18811	6.22712	6.78369	3.72086	4.94344	2.77253	19.14330	13.73408	0.60149
**Ddx11**	0.55230	0.00708	0.14029	0.77611	0.00755	0.02140	0.18158	0.02114	0.19256	0.00000	0.25766	0.01911	3.00583	0.27917	1.00653	1.76966
**Ddx17**	23.90172	15.02901	17.80968	9.83853	21.05149	7.71412	8.65937	14.97691	8.48182	24.44621	17.08235	15.35671	14.68065	26.26050	15.43551	5.61680
**Ddx18**	20.91376	9.47097	14.02594	11.60244	12.92150	10.88750	10.51324	24.98444	16.93287	20.02144	12.11314	14.61966	14.51343	24.79810	22.67027	9.61580
**Ddx19a**	0.32696	0.01945	0.41003	4.90874	0.38028	0.01438	0.64859	1.14036	6.91953	2.09166	1.93861	0.29126	0.52774	0.53350	0.57873	0.37247
**Ddx19b**	2.85863	3.08794	2.25882	3.52259	0.02247	3.39046	5.33511	7.99553	5.29506	7.49045	5.59016	0.98746	3.36524	11.27939	4.18677	2.01963

**Ddx20**	3.37436	6.49825	4.27969	9.64328	9.89743	5.90163	3.79611	9.49918	17.52693	7.88103	7.54335	2.15870	5.75876	16.44994	14.81102	2.64082
**Ddx21**	14.50108	14.64603	10.91887	12.37821	10.01910	7.73689	8.48534	26.50668	20.68102	28.43753	18.50040	11.32919	11.69569	25.07572	21.02314	4.46267
**Ddx23**	2.25823	0.00602	0.00000	0.00712	0.00867	0.01387	0.00556	0.02707	0.01139	1.22347	0.00997	0.02397	0.98988	0.01793	0.06421	0.62459
**Ddx24**	8.04464	6.08314	7.22902	7.57127	7.84435	4.18781	10.51017	6.33002	19.50064	16.08549	4.60734	9.71595	8.03741	2.45732	15.40338	2.67915
**Ddx25**	0.01319	0.00813	0.01047	0.00443	0.00900	0.00674	0.00743	0.01140	0.07177	0.84911	0.00838	0.05523	0.01175	0.00605	0.55486	0.00733
**Ddx26b**	2.84864	3.15150	0.00000	4.76873	4.15204	0.00181	0.69637	0.00000	6.45283	2.18605	0.00000	0.00000	0.00966	3.26858	2.34574	0.00173
**Ddx27**	16.55596	9.82471	8.04844	13.03462	6.65273	5.49398	10.43580	14.73346	13.82354	13.67734	6.91834	10.39805	10.72573	5.12253	8.72892	4.35338
**Ddx28**	1.59331	0.00193	4.89910	1.93885	2.68523	0.02828	1.39490	5.82888	3.99846	6.30274	0.61157	2.97490	5.96872	4.00235	4.97562	1.70647
**Ddx31**	1.28907	6.06158	2.37069	4.18130	3.04924	0.00452	5.29310	6.69562	3.64322	10.87644	1.70958	0.40598	1.53896	3.71574	6.57642	2.80650
**Ddx39**	24.64444	19.79745	24.58308	18.74776	14.09966	16.08380	17.66378	34.27838	29.67936	23.43879	23.58067	8.94280	25.63440	9.32001	14.65007	9.61416
**Ddx39b**	24.11633	18.22448	15.52924	4.91298	16.76090	6.58356	7.41579	21.29435	8.39960	17.14617	16.14466	8.11790	16.53633	2.47134	13.82264	11.63393
**Ddx3x**	12.10246	13.10925	25.01921	27.82790	14.24190	8.34138	14.46320	26.08647	25.10806	24.23187	24.56497	22.96769	16.91578	46.64340	26.70864	5.69707
**Ddx3y**	0.00000	5.97461	0.00000	0.00000	0.00767	1.72471	2.28822	0.00000	0.00712	0.18951	2.51013	7.80195	0.73672	0.01735	0.00000	0.00320
**Ddx4**	0.00831	0.00710	3.34554	4.33204	0.00358	0.00628	0.00611	0.00000	0.50696	1.14841	0.48561	0.01314	0.61497	0.00009	2.52672	0.00480
**Ddx41**	9.00333	3.50081	8.55588	5.26969	10.11778	4.90821	6.98137	15.71408	9.26991	13.07840	7.99283	3.03987	11.60834	0.15802	5.80302	4.86281
**Ddx42**	6.73495	8.61723	7.43807	4.25039	7.24039	2.31323	2.29903	7.14881	6.24272	3.61534	2.69820	0.52048	1.42669	10.30474	8.16913	1.94783
**Ddx43**	0.21236	0.00594	0.00000	0.82850	0.01554	0.00557	1.12460	0.80639	1.63043	0.61531	1.72097	2.30316	0.00078	4.99532	0.00000	0.00622
**Ddx46**	11.06864	9.54813	2.84376	17.65908	7.16269	8.07127	5.64627	12.55755	19.39516	16.57024	10.31644	13.01216	4.75508	15.93891	19.08854	5.52398
**Ddx47**	9.07932	14.34467	13.01990	7.54705	8.66178	6.02033	10.32823	17.86720	10.25931	8.93245	5.65190	1.18404	10.40797	13.70816	19.62800	4.80463
**Ddx49**	4.31929	3.76987	3.58132	0.16128	1.56036	0.04266	3.88644	13.31313	0.32658	12.79381	0.59214	0.42413	6.56767	0.01604	1.75799	3.76369
**Ddx5**	28.78631	23.09906	21.12622	22.77645	21.45022	15.25643	18.44768	35.29257	31.22029	41.78961	29.94317	27.64491	25.84413	39.37996	30.30426	16.29427
**Ddx50**	6.08475	5.77519	5.14741	3.04610	5.92391	0.00602	4.26211	3.07431	2.01290	4.89219	1.59768	10.68808	2.52014	0.55616	4.97567	2.50012
**Ddx51**	6.03119	2.04793	3.02397	7.11864	3.29064	0.00000	4.24361	6.54858	16.48267	5.98305	2.59778	1.16213	6.94039	3.27211	4.91212	0.00000
**Ddx52**	1.93105	8.24002	11.21529	11.07512	6.85236	1.33601	9.10092	7.64308	21.39152	13.41386	9.43995	8.84890	5.63494	19.41719	18.76339	2.57899
**Ddx54**	13.21919	0.11382	0.00000	17.38428	7.16863	5.65180	2.04268	20.70454	20.40066	21.58799	9.23072	0.88719	11.48664	0.02191	0.48909	6.67639
**Ddx55**	8.37013	0.01777	0.54169	5.07363	1.07518	0.67196	0.73693	8.23503	8.69955	3.63691	3.65997	1.69112	4.07449	3.46418	5.65492	3.35074
**Ddx56**	15.66965	9.31785	15.11162	7.87803	10.33347	7.68843	9.26653	20.82071	10.50564	11.29175	12.44768	6.70362	16.64625	5.41103	11.75086	15.63530
**Ddx58**	1.23384	0.00649	0.03315	0.87711	0.01344	0.00756	0.01612	0.00608	0.02974	1.13321	0.40104	0.05560	0.04895	0.30422	0.37377	0.00563
**Ddx59**	1.99910	0.01578	0.98255	4.05147	0.00570	0.43044	0.12725	0.71420	0.60703	1.22972	1.31842	0.03990	0.57365	0.00000	0.40714	0.00373
**Ddx6**	4.92519	0.32837	7.64725	3.41836	0.23969	3.23023	0.14561	1.42052	3.08015	5.08922	1.82063	0.07235	4.56914	2.38521	3.22800	0.03845
**Ddx60**	0.01475	0.00639	0.00315	1.43163	0.00899	0.00542	0.00656	0.00774	1.13005	0.55065	0.00000	3.79475	0.01428	2.91010	1.03065	0.00680
**Deaf1**	8.26331	0.35226	2.46092	3.61650	0.02096	3.59631	0.03827	6.89460	4.43885	5.16968	5.53153	6.06140	1.11462	0.00695	1.11361	0.05292
**Deb1**	4.50086	0.04402	1.66517	7.28965	0.03041	0.41354	0.69438	0.55820	6.87065	1.87569	5.86298	0.22597	2.30761	3.25114	2.20460	1.54369
**Decr1**	4.80287	5.06675	0.00000	0.57042	4.76343	0.00893	5.23995	0.00000	0.00000	1.05653	2.13426	2.85268	1.03628	0.75724	0.21513	0.00658
**Decr2**	0.01752	0.01732	0.53698	4.56535	1.28921	0.03045	1.80626	2.56034	5.62435	1.07987	4.89409	0.07187	0.86445	3.03513	2.40878	0.09258
**Dedd**	2.26828	1.80910	2.06001	2.99668	0.00000	4.18532	0.54405	1.08057	6.41609	4.81866	3.99520	0.41741	0.90803	9.38039	3.17001	2.19066
**Dedd2**	4.17629	1.92255	0.88926	8.61151	0.01957	0.21684	0.29552	2.40132	8.77701	8.14764	6.82338	0.17695	1.72771	0.06344	1.05306	0.03530
**Def6**	2.78179	0.00497	0.65283	1.01572	0.00007	0.00276	0.00565	0.10737	0.65754	0.42989	0.74511	0.00236	0.00600	0.00426	0.00000	0.00383
**Def8**	5.71548	0.16436	8.48892	6.16413	0.08882	0.01355	7.96817	15.30285	8.01188	12.67379	14.32551	1.74724	10.91234	12.61589	13.03231	3.10574
**Defa29**	0.01311	0.00801	0.00967	1.83786	0.00911	0.00628	0.00731	0.00908	2.59083	0.02109	0.12986	0.02372	0.07401	0.00373	0.08132	0.00684
**Defb23**	0.01227	0.00831	0.39090	1.31713	0.00854	0.00536	0.00761	0.01366	2.02197	0.00731	0.00000	0.00511	0.00956	0.01100	0.00000	0.00684
**Defb30**	0.01255	0.00759	0.00671	0.65115	0.00958	0.00659	0.00714	0.00949	0.00000	0.00823	0.00595	0.12664	0.01321	1.24444	0.31223	0.00722
**Defb42**	0.01406	0.00758	0.01730	2.30190	0.00888	0.00574	0.00677	0.00000	3.27614	0.63003	1.05277	0.09041	0.00419	0.29975	0.97206	0.00653
**Degs1**	7.78520	2.44080	0.00000	17.52134	2.22701	8.14710	2.55354	8.26630	8.61654	1.04625	4.85827	1.02575	6.36132	1.17123	2.67060	5.95383
**Degs2**	0.01241	0.00830	0.01621	0.01793	0.00929	0.00646	0.00798	0.03714	0.01118	0.14427	0.01204	0.01781	0.01138	0.00716	0.08518	0.00777
**Dek**	4.85033	3.80495	3.37777	2.29879	4.72005	2.72809	2.51921	5.13843	1.01862	12.43744	1.34405	1.13290	1.71196	12.17027	17.43439	1.73420
**Dennd1a**	0.18976	0.07264	0.68538	3.27311	0.01568	0.91735	0.01768	1.07144	0.96444	0.47373	0.75715	0.00000	0.00000	0.01498	0.33820	2.74510
**Dennd1b**	0.00810	0.00620	0.00000	0.20805	0.00841	3.66369	0.00523	0.53495	0.75667	1.29757	0.39930	4.60571	2.33354	4.17342	0.61418	0.01211
**Dennd1c**	0.01311	0.00741	0.06955	1.08611	0.00834	0.00865	0.00637	0.76474	0.27656	1.84224	1.84464	0.57227	0.32951	0.09861	0.20715	0.00660
**Dennd2a**	0.60636	0.00645	0.85284	5.55577	0.01300	0.00431	0.00561	0.66103	9.11177	4.48492	1.88442	0.03423	0.35055	0.16364	1.68326	1.49710
**Dennd2c**	1.54723	0.03422	1.79587	1.75569	0.20534	0.00365	3.36007	0.12911	1.07802	0.26928	0.24158	0.04764	0.02580	0.00127	0.15508	0.02485
**Dennd3**	0.00675	0.00804	0.00000	2.30878	0.01053	5.19282	0.00649	0.57392	1.59635	2.71351	0.60825	0.00735	0.00000	1.34047	0.73771	0.00476
**Dennd4a**	0.92148	0.00616	0.00000	3.40500	0.00597	0.00476	1.12587	0.00000	1.12257	0.00000	0.63824	0.03060	0.00615	0.60696	4.78986	0.00528
**Dennd4b**	1.61426	0.00569	0.00234	1.25713	0.00496	0.00555	0.00603	0.00457	1.02070	0.54528	0.00000	0.06360	0.01172	0.00280	1.08869	0.00541
**Dennd4c**	1.07295	1.61602	1.04386	8.88380	0.98900	0.00554	8.17069	2.45202	4.22732	2.07469	3.56921	0.44627	0.11686	6.71593	8.15151	0.04007
**Dennd5a**	5.72922	3.43279	2.35749	6.03308	9.52313	0.63271	6.28058	5.23714	6.22275	14.01844	1.27261	3.99196	0.24576	1.93944	0.52377	4.25836
**Dennd5b**	4.40343	4.70793	1.31627	8.31406	0.98404	0.45388	0.05691	2.57463	12.38907	1.25403	11.24789	1.58709	2.94662	16.65241	1.27030	1.75738
**Dennd6a**	0.44339	0.10648	1.51994	3.39297	2.11525	1.05065	1.25618	0.05685	4.01454	0.68543	2.44211	0.24149	0.78641	1.03907	3.83597	0.16488
**Dennd6b**	2.43259	0.00227	3.03234	0.16844	1.21961	0.00300	2.63782	1.26298	0.00716	0.99822	0.45165	0.01997	0.98690	0.00482	0.21071	3.74224
**Denr**	19.02335	3.94642	5.71432	13.59474	12.07060	5.45227	4.79111	12.38449	14.02576	18.10297	10.75000	2.34098	13.78080	9.18485	2.80919	8.16639
**Depdc1a**	0.06782	0.00760	0.00181	0.84062	1.08068	0.00753	0.33655	0.00893	0.98722	0.00000	0.09162	0.00339	0.01580	0.00428	0.55721	0.00705
**Depdc1b**	1.12044	0.11413	0.54496	0.00422	0.00488	0.00740	0.00600	0.40884	0.18920	0.73251	0.19832	0.00000	0.15389	0.51838	0.00000	0.00592

**Depdc5**	3.29819	0.21010	2.26888	3.56644	0.07580	1.07156	2.59956	0.69447	4.60420	0.61778	0.25370	0.40274	0.07604	0.18284	2.93615	0.94743
**Depdc7**	6.91351	0.62154	2.43270	11.40209	1.27250	5.29810	2.16699	8.27931	19.48298	20.69208	9.25741	14.12608	7.07105	15.16425	13.80325	1.80434
**Deptor**	4.00846	1.65245	3.43260	4.64056	7.57038	0.02894	2.40559	4.14265	5.27500	5.33200	2.37018	6.18817	1.21274	15.99487	4.02511	0.08547
**Dera**	4.90988	0.14428	1.26709	0.07896	8.66443	4.39136	0.29602	1.54175	0.49003	2.34617	3.02188	0.36165	0.41160	0.00000	0.80235	4.40696
**Derl1**	7.29748	5.85777	8.04254	1.95112	5.33657	7.69978	0.67000	4.21126	4.22316	6.25207	5.48699	1.10357	7.50052	3.74308	0.99437	4.88967
**Derl2**	10.47666	6.75076	12.55903	1.56868	3.68536	2.41255	7.35012	5.10285	0.03929	4.39055	3.04984	0.28212	6.55111	0.13938	2.30957	1.78029
**Derl3**	0.03291	0.00759	3.09692	3.54632	0.01671	0.00556	0.00686	0.00726	2.81941	1.03483	0.00000	0.00000	0.00842	0.02199	0.00000	0.00523
**Des**	0.01304	0.00789	0.01042	0.00870	0.00913	0.00678	0.21944	0.01113	0.01209	0.43942	0.01161	0.00714	0.01128	0.01159	0.01015	0.00724
**Desi1**	5.57308	3.62989	2.29082	4.98229	3.07130	1.21660	3.36623	2.88535	2.17537	7.30790	2.55454	0.23607	2.77982	4.46818	0.47249	3.95221
**Desi2**	2.74187	1.68637	2.17207	6.03146	0.45707	0.00947	0.01468	0.35442	5.68075	0.85172	0.14537	0.09929	1.52256	1.86938	4.43147	0.23523
**Det1**	0.71882	0.09590	4.41539	0.03294	0.67262	0.01221	2.26001	2.82400	2.06030	3.86003	0.00000	0.15997	1.77109	0.00475	2.03598	0.01877
**Dexi**	0.00460	0.00748	0.32344	1.76548	0.01549	0.00553	0.00707	0.04283	0.75445	0.96822	0.01853	0.00291	0.00484	0.00361	0.05665	2.17244
**Dffa**	1.64944	1.08581	3.45020	5.53633	0.52632	2.78390	3.36941	2.77167	8.44967	3.73760	2.70496	0.81648	3.43802	1.11448	2.29366	0.76798
**Dffb**	3.95464	2.43385	2.15106	0.23638	0.00823	0.00902	4.55772	8.00673	0.00459	6.74448	1.37784	1.17804	1.58909	0.24114	1.70613	2.01069
**Dfna5**	0.01835	0.00831	0.01173	0.01827	0.01064	0.00665	0.00745	0.01858	0.01099	0.01237	0.01289	0.00835	0.01231	0.00895	0.06682	0.00744
**Dgat1**	3.43020	0.00000	4.94528	2.88176	0.18826	0.03119	5.58957	1.97754	2.53996	0.39580	0.46807	0.14568	2.60020	0.08231	1.26386	2.56728
**Dgat2**	4.60516	2.61158	4.17296	3.12711	1.64845	0.00566	3.23221	6.58830	2.09220	16.47361	15.02569	2.02114	0.14727	0.34449	6.56200	2.80980
**Dgcr14**	3.69104	0.07600	2.88317	8.58110	1.86396	0.01089	2.77297	9.00986	10.01949	7.25915	4.94332	2.08538	3.28064	0.07015	3.36603	5.29408
**Dgcr2**	6.87056	3.14019	2.53215	6.81405	3.76969	3.33011	6.12881	4.28319	4.40709	1.67968	2.23060	0.27634	3.51140	1.01965	5.37876	5.81140
**Dgcr6**	5.36689	0.52326	4.20794	0.22262	3.20303	0.00941	0.44076	0.79919	0.29060	2.00387	3.08978	0.16405	4.98078	0.00281	0.01169	0.01202
**Dgcr8**	3.53604	0.00000	1.85543	3.17412	2.62050	1.91311	3.04124	4.55233	3.68919	3.11865	0.00000	4.21547	1.25864	4.18484	9.19061	3.49634
**Dgka**	3.52112	0.18771	7.76299	0.69307	1.20505	2.51999	7.56877	5.86284	0.76215	5.06543	4.64189	0.47509	1.52856	3.72957	2.14027	0.57554
**Dgkd**	0.98046	0.91030	2.38539	7.28395	5.47695	0.03208	2.55934	3.54581	2.99487	2.76024	1.41962	2.10917	2.71892	1.09950	5.01300	1.12396
**Dgke**	0.20780	0.10282	0.22011	15.13429	0.05541	0.01895	0.52470	2.18549	9.59304	4.51915	1.93091	8.30440	1.12604	10.63360	15.78096	0.05350
**Dgkeos**	0.07773	0.00757	0.00131	0.29829	0.00782	0.00706	0.00711	0.01447	1.13230	0.62284	0.00000	0.00000	0.01178	0.82463	0.80842	0.00733
**Dgkh**	0.01758	0.00811	0.00605	0.03541	0.01091	0.00658	0.00742	0.01271	0.94198	0.00000	0.00000	0.00000	0.01341	1.18292	0.57180	0.00740
**Dgkk**	0.42147	0.00739	0.00361	0.18996	0.00951	0.00754	0.00698	0.00929	0.00865	0.00976	0.01189	0.21337	0.01889	0.01209	0.02184	0.01442
**Dgkq**	0.42616	0.00673	0.06658	0.00703	0.00705	0.00537	0.69644	0.71532	0.01101	0.64255	0.00702	0.01217	1.59690	0.00713	0.19874	0.00623
**Dgkz**	3.31555	1.68992	4.30108	0.05747	5.68695	4.06381	1.63297	6.05991	0.71246	6.75952	0.69304	0.20938	2.05639	0.17352	1.27373	1.28507
**Dguok**	2.10670	0.02198	2.86287	0.57177	2.93197	2.88918	0.07483	1.72476	0.00000	0.88216	2.32371	3.39964	1.83491	0.00379	0.14695	1.03084
**Dhcr24**	1.44423	0.06603	5.10213	0.45416	1.18957	0.01006	6.52762	0.85263	0.06912	3.14572	1.11828	0.13335	6.07335	1.85467	4.31078	1.90511
**Dhcr7**	1.85690	0.83135	1.79883	2.96063	0.97002	1.26027	3.03838	0.73887	1.63734	2.66032	2.00975	1.38211	2.16718	0.11745	0.00000	1.98534
**Dhdds**	1.68740	2.66917	7.26198	3.11300	1.48186	2.82848	5.04916	6.61998	8.10944	6.14326	5.86760	1.62042	1.65417	3.22919	3.73798	0.79525
**Dhdh**	0.01355	0.00783	0.00986	0.67767	0.00916	0.01272	0.00737	0.01084	0.12477	0.34534	0.00827	0.03524	0.01131	0.08224	0.48748	0.00702
**Dhfr**	8.20934	6.59313	5.80273	2.30690	4.45410	6.09710	3.68722	3.61200	8.76471	5.46522	6.93268	4.25578	4.58094	3.58605	4.99435	0.18009
**Dhh**	0.27232	0.00781	0.00475	0.01266	0.01721	0.00723	0.00667	0.21860	0.01263	0.00000	0.77272	0.03045	0.00267	0.00479	0.06904	0.00698
**Dhodh**	7.79029	5.16758	6.22554	2.36354	5.72015	7.01854	5.29117	7.35616	1.47356	5.63629	1.90742	0.77091	3.33162	0.99864	0.00000	3.36039
**Dhps**	3.45995	0.69883	4.03193	2.51803	3.27807	5.13343	1.79398	0.72594	5.32772	5.88381	6.82608	0.14128	8.09259	3.91510	1.10478	4.39041
**Dhrs1**	4.11667	3.12643	1.06572	4.11006	0.00000	3.12578	0.32740	3.46250	1.01657	2.05820	2.42848	0.11966	1.24119	1.24793	0.00000	0.02138
**Dhrs11**	0.99215	0.01642	0.93187	0.57906	0.55442	5.73790	2.30672	0.06233	0.56485	0.19677	0.00000	0.00818	0.86547	0.71160	0.52406	0.82280
**Dhrs13**	1.92151	1.34871	0.10414	1.55251	0.01288	2.99927	0.22235	0.54392	1.65849	3.72309	1.22906	0.37845	2.53733	0.00000	0.91736	0.04488
**Dhrs2**	0.01364	0.00776	0.01150	0.28026	0.00942	0.00637	0.00735	0.01118	0.40087	0.00415	0.00791	0.00536	0.01279	0.00620	0.87821	0.00724
**Dhrs3**	4.00908	0.42097	0.00001	0.08666	1.00234	0.00594	0.00354	0.00000	0.00987	1.37403	0.57762	0.01588	0.00496	0.00693	0.00000	1.03069
**Dhrs4**	0.04381	0.00842	0.00999	0.01061	0.01394	0.00680	0.02819	0.01274	0.01235	0.01493	0.01300	0.01841	0.01260	0.01228	0.01041	0.00753
**Dhrs7**	2.63595	4.89359	7.18751	0.00000	0.05830	11.51170	2.39776	1.19263	0.58004	1.42441	3.05008	0.11335	3.77374	0.42609	0.54563	0.00000
**Dhrs7b**	11.22135	5.31506	12.81320	1.21839	5.16613	3.00042	1.20746	6.97975	0.76368	10.19246	4.95658	1.86622	8.82787	4.47575	9.27975	0.45003
**Dhtkd1**	0.00504	0.00817	2.27011	2.21023	0.01068	0.00586	0.00721	1.06790	0.00000	1.37809	0.71183	0.07454	0.09338	2.01089	2.14578	0.00655
**Dhx15**	11.40828	12.64640	11.33750	10.02082	16.51946	9.00833	10.64319	15.81513	10.56712	21.53958	9.90329	26.49174	9.85346	35.14568	19.03337	7.34624
**Dhx16**	7.27502	11.46074	10.93019	5.58245	16.59696	5.57561	16.24104	14.46373	4.33774	13.49093	12.25727	9.29485	6.53949	2.04926	5.15598	6.62114
**Dhx29**	2.63549	6.68288	4.10726	4.30348	5.31236	5.66627	0.12163	4.44248	4.34211	7.24045	2.63368	0.87965	3.41240	2.07996	7.55161	2.05596
**Dhx30**	6.77004	8.05403	7.01423	10.76639	0.65888	2.05184	2.89606	9.60698	10.11227	17.42591	9.65050	3.53644	5.76714	0.39130	11.31900	3.49564
**Dhx32**	12.12467	5.92370	6.80329	6.02150	3.84604	5.81326	3.49682	12.83179	2.33348	6.05970	2.38194	0.92202	2.81080	1.13822	9.62578	6.75198
**Dhx33**	4.40422	2.86755	7.69106	7.06558	5.28909	4.19810	5.84420	8.48762	12.04783	11.99963	5.16103	9.31248	5.30088	18.65005	11.87976	1.46726
**Dhx34**	2.35278	0.78272	0.42442	0.33110	1.70402	1.18598	0.72207	1.75923	0.03779	0.06249	1.15146	0.04395	2.54897	0.00338	0.00000	0.91603
**Dhx35**	3.16798	0.00391	0.67673	0.90257	0.00000	0.01145	0.07745	1.84567	0.06533	4.42428	0.63870	0.07072	1.75255	0.00412	0.09247	1.90955
**Dhx36**	3.86539	2.14579	1.34930	3.90729	2.49913	0.00000	4.92753	2.58982	10.01675	3.86807	3.49135	4.30740	1.02096	12.85829	8.76841	0.74540
**Dhx37**	5.33197	1.67074	0.00000	0.00000	0.00638	0.50941	0.09079	6.16538	2.99744	4.02793	0.72479	0.32700	1.59152	0.92065	2.63293	0.01467
**Dhx38**	6.32451	0.35796	3.41664	2.70466	6.41120	1.21634	0.09258	7.21011	7.42373	2.75131	1.81103	0.08680	3.98907	0.00650	0.28410	3.09459
**Dhx40**	5.88470	5.08321	3.83766	4.26949	9.02778	0.00288	7.03010	7.48858	11.29683	12.67050	5.72434	11.58954	3.33540	12.63151	12.08791	0.69528
**Dhx57**	0.06773	2.23189	0.41253	2.70418	0.02529	0.56729	1.75168	3.92429	2.73830	8.70718	1.38843	0.12091	2.26167	0.28790	0.66739	2.84128
**Dhx58**	0.01179	0.00774	0.03935	0.14699	0.00908	0.00561	0.00629	0.00000	0.98950	0.00000	2.51438	0.32657	0.00000	0.84179	0.55648	0.00653
**Dhx8**	0.79710	8.05335	5.25198	6.27156	4.21045	2.39935	4.03793	11.93097	12.04934	13.29444	3.49568	1.15770	3.29281	3.33116	12.35054	1.26405

**Dhx9**	3.04647	0.64323	2.68114	12.49633	2.00995	1.19400	0.47343	0.44017	21.03511	2.24477	13.99977	0.39658	1.89762	6.92881	4.47902	2.18091
**Diablo**	11.47648	9.03275	11.79981	3.42384	12.95909	6.25196	7.96758	16.18432	10.35735	13.78589	5.69674	1.45922	13.07241	2.34916	8.57836	6.00668
**Diaph1**	9.45298	1.51902	4.31844	5.74440	0.76658	10.15856	4.84634	3.04602	1.99068	3.16097	3.50796	0.43090	0.93912	0.06305	1.77355	2.94312
**Diaph2**	0.17647	4.77532	1.39107	0.19166	0.08239	0.04283	0.04770	0.03699	0.33304	0.40637	0.59718	0.09248	0.19244	2.89089	0.00000	1.34957
**Diaph3**	0.00762	0.04031	1.04186	5.50409	0.27102	3.23585	0.00585	0.00000	6.68027	3.70278	2.58222	0.28254	0.00000	7.58362	1.75604	0.38410
**Dicer1**	2.87336	2.25369	4.48814	11.45340	0.04712	4.43982	3.16594	2.92522	11.96988	8.25721	6.29027	7.07835	6.46962	6.03466	8.44218	4.26091
**Dido1**	9.76206	3.59013	3.27979	9.13989	3.22660	5.36869	3.93131	3.36218	10.57594	8.54932	2.74222	2.98743	1.62088	7.07406	10.67072	3.20187
**Diexf**	2.93492	2.51377	4.78259	2.34086	0.00001	2.85158	3.61478	4.21968	0.71086	5.77942	0.88446	0.23407	0.89646	1.55779	5.97623	0.15614
**Dimt1**	2.58154	0.97970	0.12413	8.05990	2.91920	0.00796	0.98463	8.76468	14.25093	2.82336	8.00525	0.87018	2.19926	8.69155	4.89020	0.43588
**Dio1**	0.00000	0.00721	0.00000	0.58209	0.00929	0.00661	0.00348	1.54734	2.78637	2.25477	3.96123	0.06925	4.00150	1.50774	0.16827	0.00472
**Dio3**	1.90452	0.00542	0.00288	3.08580	0.00425	0.00634	0.61876	0.00000	0.35835	0.00285	0.31329	0.00378	0.02197	0.00360	0.00002	0.01160
**Dio3os**	0.07393	0.00771	0.00924	0.34325	0.00788	0.00683	0.00733	0.01320	0.00555	0.14371	0.01330	0.00710	0.01285	0.01315	0.01771	0.00758
**Dip2a**	0.01265	0.12596	0.12190	1.15014	0.01029	2.31150	0.00721	0.00000	0.68697	0.88300	0.37099	0.00990	0.11040	0.00314	0.74171	0.00632
**Dip2b**	4.48201	2.94775	0.75738	3.22869	6.28057	0.56421	4.10241	2.66487	7.47045	4.43978	4.34225	0.09305	1.06764	1.75903	3.48380	2.54533
**Dip2c**	0.21756	4.71611	2.06714	2.50926	0.01680	2.26449	0.25013	0.00599	0.00000	0.00505	0.00969	0.01443	0.00595	0.00229	0.21093	1.63551
**Diras2**	0.01376	0.00805	0.00737	1.49032	0.00897	0.00698	0.00742	0.00006	0.82024	0.09801	0.71830	0.00303	0.00664	0.02084	0.00000	0.01464
**Dirc2**	9.02031	1.17222	12.95770	8.78334	0.00000	4.95968	6.18649	15.61708	5.29995	20.78791	15.79701	16.08977	6.67026	11.53675	19.23539	1.89067
**Dis3**	3.50071	4.69493	0.42693	5.95674	0.09912	3.19457	4.66906	1.26609	7.95507	7.56766	3.30757	4.99251	1.98891	13.60829	13.56253	0.01915
**Dis3l**	0.00000	0.35506	1.76735	9.14964	0.00474	0.00266	0.00622	0.07507	20.78098	3.80483	0.87564	0.03886	0.04853	5.04064	4.27048	2.09766
**Dis3l2**	0.38033	0.00780	0.00000	0.03926	0.00757	1.66824	0.00768	0.01842	0.00957	0.36586	0.01001	0.00861	0.04182	0.01174	0.01072	0.00667
**Disp1**	0.01066	0.04623	0.00571	1.30065	0.00877	0.00612	0.00655	0.00864	0.14578	0.00000	0.00000	2.73912	0.26480	0.00960	0.00000	0.00675
**Dixdc1**	0.01543	0.00737	0.04406	0.46917	0.00902	0.00592	0.00716	0.00980	0.00379	0.62273	0.01097	0.01373	0.01174	0.01129	0.42535	0.00768
**Dkc1**	19.79672	19.07185	16.28253	3.28404	14.60202	5.74763	8.82062	17.78796	4.63920	26.05577	6.20469	14.64275	17.17549	34.10845	25.73857	7.83403
**Dkk1**	0.68317	0.00682	0.01360	0.03186	0.04401	0.01530	0.01695	0.01358	0.02395	0.01242	0.02515	0.00636	0.01270	0.01408	0.02034	8.80517
**Dkk3**	0.01324	0.00778	0.01118	0.00620	0.00897	0.00671	0.00729	0.01044	0.03902	1.04691	0.01091	0.05474	0.01158	0.01105	0.01094	0.00759
**Dkkl1**	1.59608	1.87377	1.46977	2.91097	0.01853	8.29367	2.08300	1.70993	0.21943	1.18413	0.38598	0.24888	4.31193	0.00000	0.10776	2.09257
**Dlat**	8.26973	5.76794	5.86178	1.39164	11.49340	3.90764	4.12308	5.20235	0.33094	3.22059	3.88579	0.05305	2.87389	0.29069	3.29525	3.98959
**Dlc1**	5.55300	3.68939	1.17891	2.22109	1.15607	0.07346	2.71927	3.72789	0.39149	0.00000	4.07594	0.01713	1.99509	0.00000	0.00000	3.03246
**Dld**	7.97285	3.38900	10.76054	4.04422	11.24935	9.14811	7.24689	8.52628	4.49807	6.88903	2.84161	12.74047	6.77902	9.80090	9.50762	4.22285
**Dlec1**	0.01377	0.00792	0.01223	0.08669	0.00924	0.00655	0.00744	0.01264	0.01053	0.08464	0.01516	0.00828	0.01190	0.01682	0.00718	0.00719
**Dleu2**	0.63180	0.00735	0.00000	0.47798	0.00683	0.53408	0.00696	0.01615	0.40733	0.52468	0.00891	0.01580	0.00742	0.01139	0.00385	0.00638
**Dlg1**	0.99473	0.07264	1.27092	4.75519	3.68581	0.01598	0.33612	0.22245	0.89818	0.98179	2.91852	0.59027	0.27538	0.59735	2.23191	1.33102
**Dlg3**	0.13852	0.00693	0.27253	1.28905	0.01745	5.86523	0.38370	0.67521	1.48794	5.76832	4.12520	0.59954	2.30086	0.51033	2.51068	0.01031
**Dlg5**	2.15807	4.21526	0.00000	5.30684	0.16450	0.00197	0.12634	0.00000	1.99621	8.27524	1.89220	5.75354	0.18350	5.55714	10.23312	2.36038
**Dlgap1**	0.63016	0.01738	0.00506	1.38292	0.00657	0.00597	0.00679	0.00811	2.21211	1.22577	0.00990	0.09731	0.02596	0.16584	0.81082	0.00628
**Dlgap4**	8.39921	0.25999	2.54538	2.21824	0.03230	3.19077	2.06415	9.09326	2.27513	10.75411	1.58524	0.35381	7.48943	0.00000	0.00000	2.44551
**Dlgap5**	8.27868	6.77213	8.41974	8.22360	6.97101	5.72302	2.25349	7.29136	7.43463	4.46565	8.20665	5.28191	4.86666	6.79876	7.52452	4.44320
**Dlk2**	0.01313	0.00822	0.01024	1.61154	0.00907	0.00678	0.00740	0.00702	0.00000	0.09325	0.25667	0.00769	0.13815	0.00696	0.00000	0.00700
**Dll3**	0.55997	0.01715	0.30982	1.27845	0.00665	0.00522	0.00562	0.00000	2.30942	0.74410	4.32439	0.12651	0.10093	0.00000	0.10200	0.00575
**Dlst**	7.73155	5.50142	6.68881	1.26570	7.60630	10.68910	4.40500	2.56171	4.31595	2.83950	2.90497	0.09970	4.32275	0.30474	2.02178	8.66580
**Dlx2**	0.01377	0.00813	0.01207	0.73096	0.00940	0.00648	0.00741	0.01240	0.00000	0.00757	0.01241	0.00794	0.01270	0.00599	0.31138	0.00725
**Dlx3**	0.01361	0.00853	0.00365	0.01837	0.00893	0.00804	0.00699	0.00000	0.02917	0.02358	2.90922	0.00736	0.00000	0.00286	0.06339	0.00665
**Dmap1**	7.62737	0.06557	0.00000	0.05855	8.49779	2.70204	0.78733	11.18034	0.91930	16.08063	2.86059	0.13265	10.20796	0.00387	0.03152	6.09129
**Dmbx1**	0.00000	0.61597	0.00000	0.24113	0.00792	0.00638	0.00630	0.69234	0.00312	0.34847	1.58610	0.21256	0.16001	2.11708	2.41295	0.01378
**Dmc1**	3.26587	3.22107	1.97318	0.28117	0.01221	4.15114	6.21647	3.23482	0.39455	0.27568	1.26966	1.45688	3.74275	1.46367	0.32821	3.93180
**Dmd**	0.30356	6.32392	0.00000	0.51280	0.51669	0.00582	2.24843	0.69774	0.58172	1.01271	0.00000	0.27719	0.01706	3.15565	0.99151	1.32720
**Dmgdh**	3.13387	7.88385	6.32441	2.48351	2.34346	8.17102	5.66359	10.80257	0.44707	6.41619	16.86183	3.31699	9.66714	4.09722	5.61834	3.57072
**Dmkn**	1.20831	0.00586	0.80084	0.00813	0.00423	2.44519	0.00530	0.00278	0.01257	0.23859	0.00882	0.00455	1.26214	0.01439	0.00001	0.00447
**Dmpk**	0.51936	0.00695	0.00370	0.41121	0.00666	0.00739	0.02663	0.00000	1.82817	0.31845	0.62410	0.05702	0.03509	0.00602	0.00000	0.00672
**Dmrt1**	1.74052	0.00542	0.08458	0.29388	0.01469	0.00379	0.00549	0.58590	0.00695	0.62881	0.24255	0.00194	0.20599	0.00783	0.00000	0.00526
**Dmrta2**	0.26944	0.00809	0.00000	0.05832	0.00777	0.05390	0.00773	0.02466	0.06280	0.01796	0.01425	0.00818	0.03737	0.01216	0.00903	0.00720
**Dmrtb1**	0.00570	0.00706	0.00000	8.07245	0.00810	0.00458	0.00532	0.00000	13.22123	5.54379	4.09895	0.95075	0.39645	0.16558	2.14532	0.01157
**Dmtf1**	5.16833	0.41839	2.54247	4.91883	0.03789	3.22740	0.39027	7.57588	7.27758	9.76064	4.36874	9.98657	3.42602	19.03650	10.49667	1.21408
**Dmtn**	3.91093	0.09926	1.89148	3.06220	0.00990	0.01630	0.77196	2.29265	1.05300	1.77828	2.95737	0.58555	2.12190	0.02859	0.97341	0.05716
**Dmwd**	6.58684	0.01915	5.08152	11.59239	0.19936	5.22989	2.55850	5.69894	8.06597	7.61043	3.18605	0.27223	3.90212	0.00000	0.11801	1.14042
**Dmxl1**	1.68148	0.00536	0.00000	4.38534	0.00545	0.00340	0.27429	0.43703	3.38787	0.00000	0.46764	1.12776	0.01265	4.76502	2.77089	0.00428
**Dmxl2**	1.23750	0.00227	2.00139	5.49033	0.00929	0.02469	0.00337	4.04174	1.37684	1.42445	1.24421	11.09415	1.24894	5.72227	7.46356	3.75550
**Dna2**	2.82701	3.29867	1.91732	7.90281	0.08187	0.00000	2.29740	2.27551	9.20465	3.92962	4.49096	2.23545	0.59505	3.86177	2.96411	2.66800
**Dnaaf1**	0.00000	0.00513	2.94701	1.61195	0.00549	0.00604	2.56127	5.31947	0.38220	2.78043	0.78485	1.86134	2.38806	0.00612	0.16916	0.45830
**Dnaaf2**	4.80800	1.04432	1.05646	4.24720	8.86059	3.03566	2.52060	9.68658	9.73324	10.60264	5.55792	2.48163	2.51163	0.84363	6.10410	2.75195
**Dnaaf3**	0.01317	0.00809	0.02500	0.17303	0.00939	0.00771	0.00752	0.01395	0.08527	0.01461	0.01299	0.01819	0.01529	0.01170	0.00637	0.00745
**Dnaaf5**	0.39884	1.33794	1.56771	4.04946	5.21374	0.31931	0.09923	5.38663	4.75668	1.80469	1.90387	0.30714	0.81091	0.07531	1.95800	0.00405

**Dnah1**	0.02970	0.00809	0.01089	0.01028	0.00836	0.00664	0.00741	0.01358	0.01222	0.01584	0.01290	0.00829	0.05237	0.01025	0.03405	0.00732
**Dnah10**	0.01325	0.00797	0.00968	0.63784	0.00904	0.00659	0.03737	0.00383	0.08084	0.33800	0.21221	0.00503	0.08392	0.00447	0.43786	0.00745
**Dnah11**	0.01057	0.00753	0.00690	0.10190	0.00961	0.00651	0.00712	0.17902	0.00858	0.00000	0.09242	0.00392	0.00666	0.01728	1.18940	0.00724
**Dnah17**	0.00737	0.00793	0.00197	0.27778	0.00929	0.00808	0.00711	0.66294	0.54070	0.94403	1.41160	0.00000	0.08180	0.00647	0.30977	0.00720
**Dnah2**	1.39477	0.65261	0.22614	1.57620	0.40096	0.00566	0.00619	0.45187	1.14533	0.60519	0.00000	0.05447	1.35665	0.00415	0.82602	0.04193
**Dnah5**	0.01366	0.00798	0.01217	0.00903	0.00916	0.00666	0.00736	0.01146	0.01205	0.29853	0.01317	0.00699	0.01252	0.00963	0.02789	0.00778
**Dnah6**	0.24478	0.00767	0.00764	0.16589	0.00795	0.00747	0.00747	0.01758	0.51750	0.00495	0.01165	0.00664	0.22060	0.02042	0.03602	0.00721
**Dnah7a**	0.09895	0.00733	0.00746	0.24375	0.00769	0.00657	0.00716	0.00832	0.14652	0.95617	0.02158	0.01522	0.07277	0.01068	0.51544	0.00690
**Dnah7b**	0.11428	0.00726	0.00653	0.80384	0.00764	0.00651	0.00710	0.00612	0.21968	0.16093	0.10311	0.00491	0.16061	0.00902	0.80325	0.00691
**Dnah7c**	0.16858	0.00733	0.00563	0.57773	0.00754	0.00645	0.00714	0.00885	0.14655	0.35981	0.00896	0.00426	0.06446	0.00598	1.16869	0.00708
**Dnah8**	0.01887	0.00832	0.06102	0.53596	0.00855	0.00573	0.00770	0.00081	0.00138	0.40315	0.87353	0.00163	0.00457	0.26335	0.66880	0.00684
**Dnah9**	0.01346	0.00758	0.01019	1.37847	0.00916	0.00631	0.00723	0.00845	1.20534	0.22601	0.05726	0.01136	0.01210	0.00275	0.89876	0.00748
**Dnaic1**	0.00981	0.00746	0.00362	0.00458	0.01037	0.00873	0.02729	0.59471	0.01016	0.00000	1.94399	0.00000	0.02579	0.01411	0.00000	0.00733
**Dnaic2**	0.01345	0.00800	0.01156	2.18829	0.00921	0.00650	0.00750	0.01342	1.36668	0.08286	0.00000	0.02708	0.01305	0.00222	0.08637	0.01417
**Dnaja1**	39.21310	23.24173	43.52372	13.76758	29.45377	16.73918	24.07083	51.07403	19.76967	44.62897	45.16602	32.97282	46.98273	41.80200	23.49896	12.79608
**Dnaja2**	17.76526	12.90803	17.54845	20.43238	14.60748	6.65804	10.04016	19.71606	26.63928	28.25130	17.31499	19.01503	16.98114	27.96413	22.66865	8.38124
**Dnaja3**	9.20797	2.38389	6.30533	5.54157	4.69061	1.40307	7.70522	7.62298	5.22481	4.83627	6.23173	0.30931	7.47030	7.17888	6.13092	5.00771
**Dnaja4**	0.00305	0.00865	0.05636	5.79861	0.00877	0.00530	0.00700	0.51075	1.44184	0.48616	0.01881	0.08112	2.44574	0.00000	1.18382	0.01233
**Dnajb1**	10.76570	10.61269	11.43359	9.96687	2.84243	7.39772	7.44791	7.54836	21.79561	15.49721	8.48630	3.36865	12.43578	0.09521	6.03937	3.02626
**Dnajb11**	5.89775	5.44173	10.66450	3.47515	6.26749	7.59212	4.15366	7.63146	5.65070	8.90222	4.87220	6.61346	13.86920	12.60040	10.51473	10.89491
**Dnajb12**	0.05049	0.00756	0.00000	0.10492	0.00807	0.03246	0.00731	0.21984	0.90999	0.00000	0.15419	0.16455	0.03031	0.01430	0.69319	0.02125
**Dnajb13**	0.01343	0.00803	0.01186	0.40238	0.00908	0.00677	0.00752	0.01253	0.58851	0.39932	0.00821	0.00694	0.01255	0.00602	0.10309	0.00730
**Dnajb14**	0.00000	0.06705	0.32065	9.45547	0.00763	0.00285	0.00572	1.17450	15.41572	9.24167	1.77154	2.30938	0.00000	2.81164	11.54481	0.00402
**Dnajb2**	2.90172	3.72523	6.51042	1.10378	0.02826	3.60346	4.21427	2.95229	2.11432	5.67293	3.47692	0.13071	4.02930	0.49547	1.31098	0.00416
**Dnajb3**	0.00971	0.00754	0.01923	0.24457	0.00917	0.00607	0.00666	0.01877	0.49136	0.00000	0.00878	0.05445	1.11914	0.05023	0.02228	0.00662
**Dnajb4**	0.01897	0.00777	0.00435	0.65536	0.01027	2.35680	0.00710	0.00881	0.61296	0.59346	0.03294	0.05141	0.00513	0.00583	0.51893	0.00687
**Dnajb6**	21.47813	10.79921	20.49983	23.17589	16.83788	9.74669	10.48804	26.05094	25.67941	43.40434	31.71771	22.07013	28.54371	31.78883	23.70541	13.81058
**Dnajb9**	1.64304	2.91259	5.74282	22.37411	0.43633	4.42611	0.00000	7.51210	23.56476	19.39536	11.56704	30.10492	6.81380	27.40490	17.31943	0.29523
**Dnajc1**	0.77771	4.54130	4.01710	1.89774	0.11546	0.00525	0.18061	1.42056	5.23042	2.80430	2.72892	0.62251	0.00000	0.55862	1.51187	2.43964
**Dnajc10**	2.96294	0.00224	4.26452	1.10019	0.07646	3.51895	2.43374	4.10782	1.77115	1.06671	1.63490	0.08809	5.18987	0.34379	1.25954	7.70304
**Dnajc11**	12.19672	12.43040	11.56322	3.31680	4.45521	10.81842	9.65331	18.22879	3.03240	9.48303	6.76103	0.52180	18.12180	1.62167	5.59041	7.01958
**Dnajc12**	1.33162	0.00569	0.85142	5.27731	0.01320	0.00311	2.98816	0.00000	5.78981	1.84161	0.84094	0.35181	1.18977	2.09243	6.77149	0.01079
**Dnajc13**	10.06880	8.42340	6.09682	6.94058	6.74875	3.87750	7.29788	7.38085	3.95743	11.90347	3.16811	8.07943	5.57208	11.23601	8.05736	1.80793
**Dnajc14**	2.52435	1.76532	0.42449	1.09828	0.01040	7.50504	1.74591	5.26440	1.18462	4.09759	0.66438	2.90650	4.07643	0.06731	4.70022	1.49463
**Dnajc15**	11.67974	1.73205	7.11557	3.03509	2.41188	0.54672	5.77299	7.25733	12.80080	15.16268	16.94176	5.05302	10.00328	1.27747	0.31554	4.19226
**Dnajc16**	2.37818	0.00484	2.16860	0.90074	0.00000	0.00294	0.00544	2.15655	0.01732	1.52604	0.00000	0.09450	0.41972	0.00333	0.26692	0.00345
**Dnajc17**	3.58861	3.05979	4.33474	0.94394	0.85379	4.01238	0.16812	6.25538	0.20400	6.69923	1.42848	0.11903	0.85708	0.03763	0.05476	2.13048
**Dnajc18**	2.25629	0.01549	0.58979	1.36219	0.00291	0.00303	0.04396	0.00000	0.38250	0.18628	0.00000	0.00000	0.00908	2.84949	0.73584	0.00412
**Dnajc19**	11.62507	0.00000	0.83277	0.40392	7.51181	2.04612	0.84464	2.28147	0.00000	2.58476	9.60635	0.30291	6.67241	0.08435	0.34107	5.30567
**Dnajc2**	11.84923	3.72664	10.17205	4.91013	12.22066	7.59939	4.94139	11.98350	3.20449	25.14888	22.33372	19.05998	13.17383	25.34408	15.13990	3.83708
**Dnajc21**	10.25154	4.96198	7.23107	0.90537	6.12878	9.17804	6.74955	21.61875	1.15347	30.21220	14.69193	6.18614	8.46974	33.13436	31.37337	5.98235
**Dnajc22**	4.47396	0.16950	0.00000	0.00236	0.71027	0.00342	0.36580	0.00000	0.68523	0.63476	0.00515	0.01010	0.00635	0.04431	0.00000	3.46602
**Dnajc24**	3.21038	0.00000	0.71651	0.56693	8.70951	2.49359	0.06833	0.31528	0.03693	7.15291	6.80695	0.32265	1.51278	0.40560	0.00000	0.80204
**Dnajc25**	0.05249	0.02862	0.46164	2.99221	0.01659	0.32494	0.00572	2.12850	0.38252	1.42253	1.80308	0.55185	2.30683	0.00535	0.86661	0.01199
**Dnajc27**	0.60181	0.00616	0.00000	1.86376	0.01028	0.00547	2.15361	3.31599	1.96380	1.07531	0.33020	0.10045	3.19642	0.00038	2.15325	0.00485
**Dnajc28**	0.10066	0.00882	0.00954	0.44027	0.01761	0.00714	0.00761	0.01466	0.00575	0.01798	0.01511	0.00834	0.02639	0.01097	0.00582	0.00764
**Dnajc3**	1.03221	4.36980	6.43961	4.84231	0.00000	0.00396	0.72759	1.18376	4.44865	12.24435	2.49414	2.31632	0.29795	16.94540	15.10575	0.08685
**Dnajc30**	0.71779	1.22733	0.00000	2.92775	3.50857	2.14217	0.03497	1.95865	5.63561	0.51335	0.45481	0.23138	1.89152	0.00000	0.13882	0.03347
**Dnajc4**	3.23981	0.41149	1.06810	9.82433	0.00000	2.50222	0.76019	1.00275	9.74383	1.00949	1.70390	0.09325	1.05889	0.00000	0.24724	2.14453
**Dnajc5**	7.48084	0.20911	8.22283	7.37393	2.71991	6.50719	0.59578	4.99375	7.49300	9.54736	4.67361	9.80843	2.94339	6.47238	5.29464	4.38062
**Dnajc5g**	1.31860	0.00478	0.00000	1.54848	0.00545	0.00408	0.00492	0.21980	2.89361	2.91945	1.74622	3.91722	0.00000	4.08705	0.87700	0.00518
**Dnajc6**	0.56429	0.00830	0.00280	0.00422	0.03089	0.00821	0.00755	0.30792	0.99298	0.00000	0.28140	0.15165	0.00959	0.01267	0.00000	0.00688
**Dnajc7**	2.81204	5.57381	5.40025	1.92806	4.42017	3.90803	3.61013	11.30433	8.38921	5.56518	13.00182	6.86040	2.56738	12.54681	9.80683	2.09243
**Dnajc8**	17.18863	18.85386	18.37146	4.32687	9.66695	3.76391	11.71198	17.44054	9.65814	14.66502	10.52388	6.43611	16.66737	10.36068	14.03995	6.27077
**Dnajc9**	0.48074	2.84010	3.57318	4.50929	0.00008	0.35111	0.04560	0.12121	4.56668	3.02886	5.55400	0.18472	0.00000	2.26618	2.14616	0.00948
**Dnal1**	0.09943	0.00761	0.11936	0.49105	0.00777	0.00826	0.71341	0.03067	0.03092	0.04668	1.13944	0.00631	0.23166	1.84570	0.51650	0.02176
**Dnal4**	1.48689	1.75857	1.85807	11.39681	0.29495	0.65602	0.35523	3.95505	2.45179	5.88970	0.31176	0.08916	2.61755	0.00000	0.00000	0.08796
**Dnase1**	0.04848	0.00765	0.00596	0.13074	0.00796	0.00743	0.00725	0.27005	0.21055	0.23188	0.00938	0.00725	0.01652	0.01428	0.01416	0.00718
**Dnase1l1**	0.01330	0.00823	0.00563	0.03189	0.00907	0.00693	0.00757	0.00933	0.25123	0.00000	0.87896	0.01406	0.00464	0.00641	0.48999	0.00699
**Dnase1l2**	0.09351	0.00714	1.24824	0.00539	0.00564	0.03893	0.43044	0.00000	0.01078	0.60134	0.55747	0.00244	0.00583	0.01016	0.23998	0.00657
**Dnase1l3**	0.01343	0.00752	0.02595	1.11638	0.00924	0.00620	0.00724	0.01448	3.44193	0.20692	0.00000	0.01847	0.01217	0.00540	0.14178	0.00706
**Dnase2a**	1.51809	0.02557	0.99478	0.07287	0.02887	0.01531	2.21106	0.15425	0.27495	0.51434	1.24213	0.03369	0.18803	0.33895	0.55121	1.11849

**Dnd1**	0.04620	0.00884	0.39585	0.87698	0.01187	2.98185	0.00673	0.52156	1.11776	0.32928	0.14440	0.00279	1.82503	0.03678	0.00924	0.18741
**Dner**	0.01362	0.00816	0.01245	1.42694	0.00917	0.00642	0.00745	0.01238	0.00000	0.20993	0.01226	0.00736	0.01147	0.00784	0.01207	0.01398
**Dnlz**	0.76510	0.00691	0.51060	1.02289	0.28373	0.01069	0.05766	0.48282	0.47082	1.01428	0.97662	0.03908	1.97885	0.00533	0.13866	0.00541
**Dnm1**	0.01294	0.00809	0.03255	0.65525	0.00908	0.00601	0.00738	0.01104	0.00000	0.53038	0.01351	0.00653	0.01186	0.00990	0.00578	0.00769
**Dnm1l**	3.04724	8.98818	5.05944	8.32686	4.75470	2.51100	3.99882	4.91607	2.87425	5.05458	3.81201	1.36680	4.97605	0.04917	8.35477	5.63717
**Dnm2**	13.27191	0.12946	0.79896	2.02468	2.63486	7.29741	2.92475	11.38831	3.62757	8.41236	2.53749	0.35393	2.40444	0.00000	0.34637	3.78117
**Dnm3**	0.01263	0.00785	0.00843	0.03156	0.00917	0.00664	0.00728	0.00985	0.01286	0.52553	0.00832	0.00403	0.01108	0.77506	0.00000	0.00710
**Dnmbp**	1.64555	1.14997	1.48453	1.63063	0.01097	3.34177	0.01340	0.00000	1.81897	0.00000	1.37732	0.02443	2.06350	0.00262	0.03036	0.00181
**Dnmt1**	9.23716	1.19778	1.03515	6.74210	8.06483	2.09916	3.38232	7.96916	6.43919	10.41833	5.61169	0.58107	5.14933	0.03759	0.39066	11.76896
**Dnmt3a**	2.70510	4.61068	9.73648	6.79238	2.14314	0.00278	7.45781	7.07847	7.58413	5.64991	6.02491	1.34802	3.32639	3.88405	3.98353	4.51419
**Dnmt3b**	8.01421	3.84482	5.72926	1.92090	0.15658	11.54509	6.41934	13.59505	0.25811	9.04526	5.51497	1.67224	7.44427	3.73239	6.03627	20.52531
**Dnmt3l**	9.89699	3.01136	6.95181	3.62080	9.48183	16.92009	1.95018	4.90299	1.21829	3.08336	5.99656	5.20407	14.40535	1.35165	1.06776	22.57146
**Dnpep**	13.55052	3.10534	13.44132	7.47155	11.02133	12.34647	11.18210	16.47539	9.21415	8.86305	6.86470	4.60019	13.44120	0.26435	1.76028	8.33134
**Dnph1**	3.35729	0.01226	1.49487	0.91451	1.71841	0.00842	0.26757	5.15069	0.35500	3.09213	5.23998	0.73897	3.57979	0.07028	0.41591	0.50323
**Dnttip1**	4.23813	2.93603	4.80389	2.13969	1.96997	2.91932	0.02169	3.45208	3.93800	5.05654	2.06773	4.43532	0.00000	0.00000	0.46743	0.02000
**Dnttip2**	16.20605	13.60431	12.50150	11.76276	12.67207	4.32853	8.41680	17.14617	20.08184	23.17511	17.16191	9.36324	12.71057	28.58979	21.63548	6.39719
**Doc2a**	3.92014	2.93582	3.49813	2.60157	4.32427	3.13738	3.91603	5.65995	5.89112	7.29212	1.99517	0.13228	3.99090	0.00000	0.00000	2.25113
**Doc2b**	0.01080	0.00752	0.01021	0.15077	0.00944	0.00658	0.00717	0.12643	0.00964	0.25459	0.01479	0.00687	0.00753	0.01124	0.05356	0.00711
**Doc2g**	0.01016	0.00734	0.00388	0.01128	0.00963	0.05452	0.00707	1.11499	0.01119	0.00335	0.00648	0.00538	0.00468	0.01651	0.00000	0.00734
**Dock1**	1.43124	0.01536	2.61211	0.02159	0.37970	0.00772	0.00532	1.04617	0.04989	0.86097	0.04202	0.04343	0.73646	0.76555	0.02816	0.14600
**Dock10**	0.00726	0.00766	0.00827	0.01716	0.38690	0.00706	0.00736	0.01168	0.01220	0.03274	0.02431	0.00821	0.35136	0.01182	0.01147	0.00712
**Dock11**	0.00000	0.00777	0.29851	1.06827	0.00770	0.00880	1.02842	0.17911	1.18907	0.41746	0.50750	0.00000	0.00002	0.00229	0.23867	0.64868
**Dock3**	0.01064	0.00839	0.00837	1.35549	0.00923	0.00663	0.00788	0.08736	1.45424	0.13688	0.16881	0.02434	0.08696	0.03563	0.09816	0.00732
**Dock4**	0.15214	0.01812	1.33646	0.01752	0.00867	0.00576	0.00701	0.33434	0.61814	0.00000	0.06666	0.04543	0.00000	0.01628	0.40127	0.00660
**Dock5**	4.92113	1.09372	2.52539	5.04238	0.00747	0.00157	2.21403	3.23240	0.63988	3.27976	4.30740	0.03378	2.66868	2.12320	2.03026	0.29819
**Dock6**	3.34208	0.20996	1.30341	0.80164	0.00905	0.00549	0.00363	3.75678	0.90969	2.48261	1.76014	0.01790	0.16429	0.00000	0.49793	1.17094
**Dock7**	0.10250	3.79591	0.10663	10.54006	0.00747	0.32765	0.00622	0.13394	0.00000	0.29066	0.12863	0.04761	0.00222	0.71269	2.31526	0.00386
**Dock8**	0.29529	0.85220	0.98928	0.02605	0.00557	0.00550	0.00664	0.01867	0.01129	0.00313	0.01157	0.01793	0.24175	0.01195	0.00000	0.01316
**Dock9**	3.34993	0.01300	0.68921	6.89013	0.22956	0.50599	0.26621	5.65565	1.93102	2.08437	5.15545	0.19888	0.89477	2.60277	4.55718	2.55459
**Dohh**	8.84918	0.35648	0.06236	0.42857	4.77356	0.60833	0.04595	2.11992	2.05742	12.08860	2.23922	0.06036	10.66883	0.00000	0.00000	4.81161
**Dok1**	1.13373	0.00591	1.14718	0.18066	0.14480	0.90422	0.00594	2.94564	0.00770	0.96130	0.35291	0.00504	0.22239	0.00603	0.00000	0.01275
**Dok2**	0.00000	1.18725	2.95026	0.00000	0.00000	4.47977	2.43288	3.30445	0.00866	0.00000	0.40568	0.02150	5.96405	0.01600	0.00000	8.39075
**Dok3**	0.01364	0.00818	0.01195	0.24648	0.00924	0.00683	0.00761	0.01433	0.34783	0.07356	0.00998	0.00844	0.01318	0.01223	0.00891	0.00737
**Dok4**	0.97049	0.00666	0.00000	1.33283	0.03206	2.62264	1.37655	0.05206	1.51726	0.12321	0.00000	0.04135	0.89705	0.00244	0.32823	0.24959
**Dok5**	0.01181	0.00809	0.04278	0.01365	0.00986	0.01565	0.00757	0.02502	0.01253	0.57288	0.01390	0.00599	0.12002	0.04323	0.03710	0.03853
**Dolk**	2.31339	0.43550	2.49770	4.02719	2.73077	0.00351	3.69741	3.13848	8.38161	3.56949	2.38463	0.39331	1.51783	2.04201	3.95411	1.41846
**Dolpp1**	4.09461	1.80770	0.99865	0.17323	0.23996	2.61275	1.11047	5.69837	0.38157	0.50205	2.34181	0.21514	6.36526	0.00028	0.55261	4.31436
**Donson**	3.36889	2.44944	0.00000	11.90523	0.00588	0.02274	0.80695	1.84987	8.90003	3.32013	2.77020	0.77299	0.51232	2.86993	7.35468	1.17618
**Dopey1**	0.00000	0.00708	0.11526	1.06248	0.01121	0.10115	2.12360	1.38709	0.73940	0.79991	0.05776	0.08037	0.11153	1.25324	1.88170	0.00711
**Dopey2**	2.72728	0.00430	0.01133	1.56381	0.00491	0.00438	0.00410	0.60358	0.67901	0.60615	0.65624	0.03921	0.95543	0.00054	0.85318	0.00405
**Dot1l**	0.60751	0.02527	1.19406	0.54849	2.92895	0.01753	0.54568	3.17314	1.13996	2.31812	0.22945	0.10377	2.13562	0.12112	0.91082	1.10705
**Dpagt1**	1.51281	5.44093	4.08582	2.83813	2.45388	7.49155	0.79833	2.63161	3.94598	2.88313	1.51219	0.67549	3.26437	2.22273	2.98595	6.61602
**Dpcd**	5.19823	8.07482	3.04073	0.36452	5.80006	0.00094	1.37156	0.62732	0.35756	4.40493	2.38398	0.29187	3.40178	0.10809	1.55205	0.37381
**Dpcr1**	0.01510	0.00822	0.01741	0.03355	0.03093	0.00725	0.00764	0.01663	0.60702	0.02909	0.04501	0.00699	0.01139	0.01534	0.01457	0.00733
**Dpep1**	0.37307	0.00748	0.83667	0.00993	0.00558	0.00508	0.00698	0.45801	2.16098	0.66404	0.00000	0.06267	0.00382	0.00882	0.02183	0.00683
**Dpep2**	0.00000	0.00768	0.26145	3.93505	0.00860	0.00792	0.00694	0.24942	8.31119	2.13351	0.00000	0.04739	0.33100	1.90532	5.70445	0.00583
**Dpep3**	0.00000	0.01799	2.48412	1.14879	0.01968	1.25969	0.84644	0.03591	1.33702	2.32340	4.19372	0.14269	3.45515	0.15905	0.89613	0.01146
**Dpf1**	0.05755	0.00838	0.02590	0.56924	0.01411	0.00604	0.00752	0.01288	0.00000	0.01236	0.01176	0.01780	0.01216	0.00548	0.16232	0.00734
**Dpf2**	8.11637	10.71617	13.25513	10.43923	8.03927	9.24409	6.36928	17.85200	6.31057	22.83337	14.76872	2.83822	13.36899	3.97686	14.47712	3.16738
**Dpf3**	2.62707	0.06816	2.03705	1.06459	0.00000	0.00358	2.40893	6.75603	4.16152	3.29956	5.78424	0.03824	1.07305	0.39330	0.38102	0.00885
**Dph1**	1.66050	0.01605	0.00000	0.00188	0.12079	1.13197	1.06233	7.62324	0.00687	6.64922	0.03276	0.14217	4.81579	0.01218	0.00000	0.21958
**Dph2**	2.18653	2.48015	5.46853	5.54408	3.90352	0.00839	0.00000	4.49125	11.75185	6.32792	3.92351	3.89448	2.64761	0.02831	4.34062	1.04783
**Dph3**	7.11869	3.53352	8.47504	12.32710	8.78390	0.26303	6.93493	7.19509	11.84290	18.52784	15.36148	1.90275	13.06143	3.98890	2.91002	0.03555
**Dph5**	11.19041	5.20390	5.70556	0.52669	1.20629	0.09063	6.76259	5.00999	0.46489	3.64786	1.84510	3.56349	5.08457	8.09837	0.47072	0.24644
**Dph6**	3.04284	2.76425	3.46141	0.65315	2.05108	0.03829	2.60939	3.26727	0.30827	0.27569	1.81340	0.28294	1.76025	2.93093	2.84127	0.37719
**Dph7**	3.13993	0.60259	0.25031	1.42479	0.01244	0.00234	0.52527	0.32289	2.74164	0.77569	0.72766	0.10962	0.00000	0.28255	7.40964	1.48510
**Dpm1**	0.53376	0.40871	0.49676	2.17243	0.33275	0.02726	0.15206	0.26692	2.84387	0.40846	0.87120	0.24417	0.35439	0.11967	0.14247	0.44743
**Dpm2**	5.85471	5.53884	12.76369	3.68259	9.02955	0.89321	3.66437	7.28732	1.29748	9.24824	3.94368	1.85375	7.60967	2.48124	2.82278	5.53324
**Dpm3**	3.70619	0.06326	1.61718	0.13264	1.92108	0.37639	0.17436	0.12695	0.20839	0.60750	0.69847	0.04526	1.44727	0.00937	0.00000	0.46868
**Dpp10**	0.01370	0.00777	0.01136	2.16276	0.00943	0.00623	0.00727	0.01074	0.00000	0.00484	0.02035	0.00583	0.01258	0.00453	0.97799	0.00698
**Dpp3**	11.67048	1.07056	1.47629	0.74242	1.94396	2.23919	1.70048	7.38057	0.31070	4.81402	3.79766	0.06495	11.29474	0.00000	0.03956	6.79340
**Dpp4**	2.13742	0.00233	0.00363	0.00442	2.34314	0.00492	4.39035	0.00000	0.00954	0.12740	0.00865	0.20140	0.01207	0.00616	0.41155	0.00509

**Dpp7**	6.70282	3.33518	13.07246	0.00000	0.11730	6.13378	9.55003	7.94690	0.81258	0.37483	8.62411	0.09589	16.67109	0.10500	1.59221	2.19005
**Dpp8**	2.27741	0.00553	0.00000	0.66187	0.00595	0.00467	0.00534	1.56765	0.20307	0.23391	1.07696	0.02206	0.11325	2.00154	1.25139	0.00486
**Dpp9**	1.75061	2.46408	0.87746	5.28406	4.76473	0.45723	0.01348	2.69000	6.47441	3.20467	0.41371	0.63098	2.12302	0.00000	0.64719	1.97497
**Dppa1**	0.00000	0.01781	0.01970	0.00000	0.28252	12.28919	0.00556	0.00447	1.03395	0.00000	0.00000	0.00486	1.51524	0.01827	0.00441	2.19480
**Dppa2**	24.77857	20.06955	16.44406	14.28150	22.59207	7.83483	14.64659	29.50402	23.85140	17.88023	27.84472	14.90004	14.66049	43.82085	31.46315	4.56420
**Dppa3**	43.43137	30.19215	49.17332	31.96539	32.10870	20.67482	27.56540	45.66849	42.10666	55.38266	50.55017	22.55218	46.02930	43.41861	37.84792	16.25833
**Dppa4**	19.29090	9.62832	19.57113	17.38752	16.77631	8.48787	8.56788	20.21633	26.72493	28.41034	21.70930	13.57555	18.83032	22.63153	21.75564	8.09088
**Dppa5a**	44.26855	15.27019	34.41819	16.46749	27.14855	15.43145	13.43581	21.89725	29.06733	22.87828	38.36327	8.75707	28.28143	17.81818	17.01832	22.58817
**Dpy19l1**	2.48217	3.76332	0.15814	0.46996	0.43634	0.00598	1.36324	0.02823	0.00381	0.00000	0.02088	0.01061	1.14843	0.76865	0.27347	2.46690
**Dpy19l2**	0.00953	0.00856	0.00836	0.01095	0.00965	0.00695	0.00822	0.44588	0.01187	0.00897	0.00980	0.00829	0.00596	0.01625	0.00344	0.00739
**Dpy19l3**	0.01702	0.01768	0.00669	0.90953	0.00886	0.00691	0.05815	0.01125	0.49033	0.46081	0.00082	1.66978	0.01718	0.00949	0.00000	0.00721
**Dpy19l4**	0.10670	0.00756	0.36980	3.83076	0.00790	0.03339	0.00738	0.25690	0.82810	2.89490	0.02276	0.01755	0.05096	0.41065	0.51042	0.00675
**Dpy30**	18.42754	4.28077	9.51449	11.99667	9.04378	4.09577	5.45495	8.62784	11.18906	11.12084	11.95588	2.42264	12.15249	2.77396	5.44281	6.09159
**Dpys**	0.05036	0.00800	0.00751	0.45426	0.00808	0.00728	0.00743	0.01376	0.00384	0.11632	0.01089	0.15740	0.01323	0.01064	0.00299	0.00723
**Dpysl2**	0.00120	0.05067	0.01808	0.01632	2.25587	0.00861	0.00812	0.08822	0.01101	0.13578	0.01385	0.01846	0.02453	0.02051	0.00545	0.02142
**Dpysl3**	0.01349	0.00763	0.00845	2.66991	0.00926	0.00608	0.00725	0.00937	1.75373	0.00000	0.05192	0.06940	0.01180	0.00490	1.31081	0.00669
**Dpysl4**	1.55114	0.90201	0.01313	0.00291	1.02399	0.00443	0.12449	3.52893	0.00816	0.35946	0.45426	0.00306	2.43948	0.01617	0.00000	1.95576
**Dpysl5**	0.02258	0.00776	0.00736	0.38506	0.00966	0.00671	0.71501	0.00982	0.09428	0.40071	0.01005	0.00628	0.15544	0.00678	0.07577	0.00691
**Dqx1**	0.12423	0.02927	0.36175	0.35003	0.05215	0.04741	0.04838	0.01804	0.77004	0.06905	0.77598	0.10875	0.22814	0.54810	0.07304	0.05066
**Dr1**	1.99444	0.06343	0.82808	18.91862	2.61884	1.52234	3.04747	4.96811	16.71251	2.34536	0.36242	10.32400	2.35826	9.77435	10.58260	0.43680
**Dram1**	3.96494	0.01350	1.28385	0.00000	1.52395	0.00140	1.60436	0.03475	0.03943	0.52173	0.00000	1.54069	0.38938	0.19589	1.46502	0.00284
**Dram2**	0.11538	0.09984	1.29004	1.09104	4.92548	7.15156	5.88180	5.80361	0.11016	1.29175	2.39541	1.78348	1.91647	5.47535	0.00000	0.71049
**Drap1**	4.13588	0.04170	2.13672	0.89747	0.77953	1.05388	1.40783	1.26573	1.00629	1.43040	4.12216	0.35207	1.28022	0.03450	0.00000	1.35041
**Draxin**	0.01310	0.00788	0.01167	0.57126	0.00912	0.00643	0.03714	0.00956	0.10043	0.99740	0.02910	0.01474	0.01108	0.00899	0.01523	0.01468
**Drc1**	0.00000	0.00601	0.29619	0.44461	0.00911	0.00555	2.92154	1.76648	0.03429	2.74447	1.11569	0.25683	0.00000	2.35254	6.88076	0.00682
**Drc7**	0.01330	0.00767	0.00883	0.24677	0.00913	0.00658	0.00726	0.01472	1.37544	0.00000	0.21820	0.02279	0.00978	0.01286	0.31028	0.00738
**Drd3**	0.00604	0.00779	0.47966	0.00594	0.00863	0.00520	0.00775	0.16576	0.00937	0.00000	0.00000	2.59433	0.10674	0.00587	0.80293	0.00682
**Drg1**	19.77251	10.78171	18.53039	8.31851	16.28106	6.15408	9.07605	22.39937	11.94168	22.80105	16.98329	9.42860	17.79623	14.38207	16.51703	10.58626
**Drg2**	11.73747	7.80593	15.49188	4.48548	6.84252	5.16866	10.97315	31.38267	6.12864	26.56343	15.81105	2.31404	14.76976	6.90590	18.44395	5.06442
**Drosha**	2.60088	4.27682	3.86204	7.19088	0.06515	5.46796	5.37202	5.54165	5.83146	7.25141	9.44577	3.08283	6.43577	5.80347	7.65782	6.60084
**Dsc2**	0.00000	0.00856	0.03345	1.85171	0.00752	0.00802	0.00773	0.20509	0.12617	0.00212	0.01032	0.00854	0.73397	0.01382	0.00000	1.46700
**Dscam**	0.01360	0.00820	0.01192	0.30857	0.00927	0.00657	0.00753	0.01385	1.01154	0.01062	0.00758	0.00668	0.01292	0.01209	0.00379	0.00731
**Dscc1**	5.44696	0.43886	1.99828	2.93551	2.09377	0.70565	1.19299	2.30496	2.99498	12.24511	9.03048	11.74397	9.35604	5.49773	4.53759	6.17164
**Dscr3**	6.50666	1.99555	9.15701	0.13663	0.01428	5.42341	6.09328	3.42416	1.44091	4.30344	0.81845	5.39564	0.83153	2.16769	0.17393	1.55444
**Dse**	1.60044	0.00592	0.00345	0.78679	0.00508	0.00630	0.00617	0.00727	0.81039	0.01149	0.00723	0.02613	0.01779	0.00830	0.15535	0.00543
**Dsel**	0.01364	0.00779	0.01138	0.36650	0.00924	0.00653	0.00747	0.01308	0.90244	0.00781	0.00717	0.01659	0.01931	0.01928	0.03445	0.00727
**Dsg1b**	0.03180	0.00788	0.10649	0.37070	0.03343	0.01866	0.00712	0.01676	0.10991	0.06819	0.36488	0.03351	0.12212	0.33151	0.08677	0.01467
**Dsg2**	2.40950	3.32666	1.70287	0.00540	0.19879	5.63165	0.01194	0.00831	0.02524	0.00000	0.00475	0.02036	2.86666	0.17012	0.01773	2.82128
**Dsn1**	3.28027	9.14756	3.83750	11.89445	0.00821	0.47870	2.37758	6.58283	14.53472	10.13982	3.72055	0.74376	1.93174	9.86463	18.45838	0.00000
**Dsp**	0.42840	0.01992	4.49760	1.42037	0.04853	11.60056	1.00977	1.16305	0.00000	0.64195	1.15206	0.01563	4.63796	0.05030	0.00000	0.02165
**Dst**	2.09616	0.00324	0.00000	11.59794	0.01409	3.86856	4.68426	0.09889	2.18335	1.06252	0.97167	0.10404	0.89196	0.00000	3.70227	0.00803
**Dstn**	16.17043	16.20321	19.09147	8.21437	15.96411	16.62857	14.35256	12.58765	8.28284	14.78442	9.52808	15.78464	11.74270	14.22208	10.33597	16.02828
**Dstyk**	0.21151	0.00756	0.02661	3.21063	0.00731	3.12408	0.00681	0.00000	1.53563	0.95237	0.40106	0.01805	0.00645	0.01051	2.21987	0.00498
**Dtd1**	1.78837	1.30250	0.75960	0.64406	0.01403	3.51531	0.00422	2.07172	0.02376	1.81149	2.82222	0.18515	0.00000	0.66884	1.06215	1.72627
**Dtd2**	0.64732	0.12223	1.55973	0.20294	0.61508	0.59045	0.85687	0.83788	0.02595	0.00000	0.17794	0.03874	0.40749	0.00783	0.00000	3.16768
**Dtl**	4.45820	7.41921	5.98145	10.70995	2.44112	7.01040	9.61449	8.09304	18.42258	19.72061	15.27808	9.20297	4.53865	26.79511	18.72017	1.53935
**Dtnb**	3.32632	3.42783	2.43957	1.02734	0.00963	0.00808	1.92114	1.60776	2.99806	6.02568	4.18694	1.48776	1.65783	2.36321	1.77034	1.49470
**Dtnbp1**	1.96880	3.00887	5.19059	2.54845	0.11029	0.01136	4.59450	1.10609	1.44563	2.42449	3.88562	0.08452	0.47574	0.80599	2.81698	0.01554
**Dtwd1**	2.64598	0.48146	1.60419	0.00227	0.00000	0.00305	0.00474	0.73883	0.02317	0.81779	0.76732	0.00817	1.43881	0.01396	0.81457	0.64068
**Dtwd2**	0.43151	1.63030	1.01431	3.79016	0.00604	0.01076	0.47696	0.08849	3.26004	2.21430	0.30625	0.57866	0.07284	2.77102	0.75308	0.01198
**Dtx2**	1.41221	0.00247	0.17587	25.22986	4.71180	0.01908	1.07723	6.59347	13.51581	14.62224	2.72837	0.59672	4.92857	0.07795	0.00000	5.30493
**Dtx3**	1.54263	0.00628	0.00363	0.59060	0.00498	0.00679	0.00609	0.02614	0.01433	0.07007	0.02734	0.00693	0.01085	0.01016	0.00442	0.00561
**Dtx3l**	0.04974	0.00767	0.07946	0.37734	0.04227	0.00611	0.03737	0.01701	1.06534	0.01352	0.49427	0.05926	0.10900	0.19999	0.46009	0.02147
**Dtx4**	0.01319	0.00776	0.01047	0.91839	0.00927	0.00638	0.00752	0.01510	0.96583	0.38791	0.03702	0.04283	0.01141	0.00628	0.30645	0.00722
**Dtymk**	13.86918	2.78503	7.03280	2.45012	9.72894	8.88717	0.44652	15.62565	3.60998	17.16217	8.49478	1.42373	14.82093	3.77350	0.52929	11.18673
**Dubr**	0.85103	0.00793	0.00425	0.00476	0.01709	0.00694	0.00673	0.00816	0.50892	0.32140	0.00737	0.01597	0.01704	0.01202	0.00724	0.00643
**Duox1**	0.49547	0.00828	0.22290	0.01070	0.01540	0.00510	0.12170	0.00994	0.01238	0.00436	0.01234	0.00799	0.00604	0.01471	0.00613	2.64939
**Duoxa2**	0.06619	0.02772	0.00473	0.00416	0.00874	0.00737	0.85522	0.00943	0.24111	0.01063	0.01077	0.02625	0.01088	0.01280	0.00735	0.00727
**Dus1l**	9.49165	2.02951	8.23210	1.39959	4.25251	1.75841	7.95435	9.67743	3.89152	13.45341	9.49696	0.24262	7.32068	0.15686	2.41084	4.63880
**Dus2**	6.20979	2.93115	2.73879	1.70532	1.69992	5.03949	5.20204	4.56425	2.97125	4.54373	1.37353	3.15281	3.06231	0.00000	2.34957	1.67232
**Dus3l**	9.62869	0.18777	0.00000	3.97441	0.66299	5.02831	2.15461	10.88778	3.55885	12.48567	7.38433	0.53929	9.99234	0.01068	0.67118	3.46727
**Dus4l**	0.01158	0.00775	1.43731	0.34480	0.00743	0.00582	0.00741	0.00954	0.00517	0.02958	0.02425	0.00517	0.01508	0.58652	0.00000	0.00722

**Dusp1**	0.37177	1.68442	0.00000	3.67081	0.01511	0.07639	0.00607	0.30792	8.63645	0.19870	0.00000	0.00927	0.81597	0.00000	2.08305	0.00472
**Dusp10**	0.00000	0.00091	0.54783	1.89835	8.80258	0.01118	1.53056	1.28849	1.56585	4.59553	1.16449	2.47798	1.68920	1.61809	6.65559	0.93017
**Dusp11**	7.41378	1.32031	6.97277	2.21361	4.40798	1.25743	6.08312	12.51431	4.07708	5.11713	5.73086	5.49046	7.18408	2.64578	2.38048	8.47960
**Dusp12**	8.70782	0.02831	2.01527	3.28855	0.04581	0.02399	1.33626	8.40988	3.54049	18.61352	9.19956	1.72731	7.70877	10.34609	1.96389	7.52316
**Dusp14**	0.00000	2.48005	8.04441	19.20721	0.17458	15.53303	11.59362	18.13127	27.44581	7.32270	9.46211	1.26502	14.65764	3.41753	7.49300	1.72668
**Dusp16**	5.13087	1.92944	0.00000	8.12852	1.22209	0.86680	0.00000	2.43871	0.95804	2.14165	2.14054	0.19385	1.09759	0.90660	8.90916	0.06207
**Dusp18**	0.02422	0.00791	0.53495	2.05612	0.00833	0.26623	0.00743	0.05877	0.29063	1.63758	0.00659	0.00000	0.09339	0.07473	1.47590	0.00693
**Dusp19**	1.17942	0.19552	3.11362	0.00993	0.05221	0.01061	0.21989	0.30965	0.00904	1.51909	1.95536	0.19836	0.00000	0.98374	0.34205	0.59739
**Dusp2**	0.00872	0.00827	0.00301	1.01811	0.00930	0.00717	0.00773	0.28276	0.28203	0.80620	0.49110	0.11949	0.00426	0.00335	0.80910	0.00722
**Dusp22**	0.09571	0.02836	0.15350	1.78726	0.02860	0.04524	0.05742	0.01144	2.55571	1.38450	0.38316	0.11834	0.17484	0.17207	2.96664	0.03751
**Dusp23**	0.01297	0.00796	0.01227	0.10179	0.00889	0.00644	0.00730	0.01008	0.01808	1.47160	0.01097	0.00406	0.01042	0.01125	0.00548	0.00716
**Dusp26**	0.00000	0.00662	0.00169	0.21966	0.02020	2.37574	2.17687	2.03172	0.00695	6.35163	0.90186	0.25234	0.00000	0.02514	1.10275	0.00629
**Dusp28**	0.00974	0.00785	0.11456	0.09337	0.00910	0.00901	0.00747	0.01244	1.14879	0.42255	0.00816	0.01280	0.14133	0.00574	0.08709	0.25546
**Dusp3**	0.05623	0.00829	0.00000	0.10772	0.03232	4.10816	0.01814	1.72899	0.08978	1.46836	0.98539	0.08738	0.80413	0.17183	0.17413	0.01288
**Dusp4**	4.29342	2.11627	0.79722	0.34042	0.09067	0.01341	0.00000	0.77511	1.22549	0.00000	0.00000	0.18554	6.07841	0.00675	0.11804	12.28998
**Dusp5**	0.52560	0.00697	0.11000	0.17174	0.00896	0.00528	0.88733	0.15704	0.05318	0.02204	0.01072	0.00692	0.00580	0.00791	0.04503	0.33354
**Dusp6**	1.07251	0.02708	0.00000	1.63421	0.00556	0.05898	0.00631	0.00000	0.17098	0.00000	0.27641	0.01233	0.00728	0.82317	0.00000	0.54030
**Dusp7**	0.51262	0.00672	0.42229	5.89349	0.35694	0.00508	0.00588	0.00301	1.45323	0.68964	0.05678	0.01552	1.40280	0.01864	2.23994	0.30179
**Dusp8**	0.01330	0.00798	0.01173	0.00978	0.00934	0.00674	0.00746	0.02452	0.02350	0.02076	0.01264	0.01877	0.01218	0.01207	0.01055	0.00742
**Dusp9**	2.19866	0.05214	2.22306	0.97077	0.53341	0.00369	0.70156	0.13490	1.10211	0.05588	1.44687	0.00638	0.82613	0.00431	0.11660	0.13357
**Dut**	15.87532	5.26299	5.25640	18.14672	12.30521	2.50513	1.31549	0.70160	12.85372	12.85606	15.05803	0.46656	17.57077	1.23330	1.76286	17.57280
**Dvl1**	3.66581	0.12491	0.80474	0.94566	0.04185	0.22370	0.00000	5.93991	2.46086	3.39167	4.65901	0.06993	2.79803	0.00000	0.78774	2.20878
**Dvl2**	0.67193	0.41264	0.87034	1.76424	0.78056	0.11675	0.00510	0.74077	1.07944	2.14780	3.08288	0.02190	0.89491	0.25543	0.41863	3.01518
**Dvl3**	0.00274	0.00730	0.08566	0.00561	0.74299	0.17621	0.13116	0.94802	0.11970	0.52147	0.06565	0.01251	0.14470	0.21160	0.15607	0.40362
**Dxo**	6.34244	0.01025	0.00000	6.10626	0.09581	3.81229	0.01792	2.75994	9.55346	4.12847	3.74657	0.52197	4.09420	0.00000	0.94621	7.97146
**Dydc1**	0.01053	0.00790	0.00890	0.54579	0.00942	0.00660	0.00749	0.12991	0.00535	0.11261	0.05355	0.00658	0.12153	0.00986	0.33658	0.00702
**Dydc2**	0.00772	0.00768	0.00000	1.41587	0.00919	0.00815	0.00679	0.69281	0.00000	0.57962	1.09245	0.05763	0.48854	0.00147	0.67407	0.00658
**Dym**	6.29859	1.83697	4.62164	6.08942	3.54549	0.03398	3.21703	3.78658	3.64249	8.15406	2.69595	0.19698	4.01883	0.30985	2.97355	5.78055
**Dynap**	0.01024	0.00742	0.00000	0.00000	0.00916	0.00620	0.00694	0.00318	0.00709	0.20838	0.28786	1.81848	0.00982	2.31339	1.44997	0.00753
**Dync1h1**	9.44449	12.80693	9.84085	6.08608	9.87850	4.58656	7.94237	10.53222	5.85411	5.61299	2.23016	0.45368	10.50051	0.00000	2.38545	5.79939
**Dync1i1**	2.81138	0.00573	0.00574	0.23828	0.01275	0.00458	0.00504	0.00000	0.00521	1.49519	0.00981	0.00239	0.02256	0.01070	0.00529	0.00435
**Dync1i2**	4.25438	6.38897	5.96976	4.14176	8.90845	1.40258	5.16890	6.77637	2.75804	3.34393	2.68013	6.82019	2.28012	5.76864	5.08411	1.34449
**Dync1li1**	3.34492	3.84280	2.73100	0.22522	7.40522	2.06314	0.89456	5.59190	0.15586	6.67301	0.06962	0.28002	1.97949	3.00471	1.03162	1.64429
**Dync1li2**	2.27521	3.36959	1.42136	0.63748	6.25804	2.94601	5.55855	4.00915	0.34212	6.53805	3.80633	0.95714	0.00000	4.72879	6.23869	4.97153
**Dync2h1**	2.24038	3.47343	0.11076	0.40066	3.00414	0.59567	1.56717	1.43974	0.05296	0.09089	0.20423	0.03522	0.65015	0.01520	0.26839	0.48443
**Dync2li1**	0.31597	0.00822	0.00629	0.00769	0.01775	0.00696	0.00709	0.00990	0.01191	0.80628	0.01223	0.00535	0.01145	0.01208	0.00820	0.00753
**Dynll1**	29.20151	5.15229	20.67266	24.52400	22.36891	8.43297	12.34432	15.86712	38.02388	29.90517	23.73964	6.59645	15.24057	4.59049	2.32403	17.74399
**Dynll2**	5.17901	0.01027	0.57501	13.56801	2.94304	0.08098	1.81019	0.19645	4.63508	7.51210	4.85776	0.32191	1.16012	1.47649	3.40921	0.64207
**Dynlrb1**	9.31594	3.99683	9.49586	7.02339	9.51371	6.87633	3.61229	3.63963	9.20793	10.17702	13.02809	1.61654	5.91081	2.50472	2.42560	4.32694
**Dynlrb2**	0.14732	0.00784	0.00796	0.01022	0.00762	0.00688	0.00736	0.01418	0.01239	0.09450	0.01245	0.00754	0.01219	0.01144	0.01045	0.00714
**Dynlt1a**	6.68203	2.14663	1.39350	2.26722	4.96164	3.59122	1.76737	1.47486	3.47371	0.57543	3.71825	0.35593	1.77749	2.48465	1.74685	4.49170
**Dynlt1b**	6.96939	2.36917	1.40076	0.14130	5.28220	3.38945	1.65312	1.47925	0.42763	0.59185	4.29586	0.16136	1.72219	1.64460	1.25542	4.13530
**Dynlt1f**	0.00935	0.02915	0.01996	0.06360	0.00870	0.31495	0.36745	0.00983	0.01720	0.00285	0.10214	0.00735	0.10027	0.01103	0.00994	0.25352
**Dynlt3**	5.62171	2.12116	4.30441	7.42386	3.77714	6.01100	1.48192	1.21482	1.20313	1.77490	0.07655	6.63682	1.38667	0.99797	4.57994	3.19233
**Dyrk1a**	4.51954	4.75338	6.36087	15.70754	2.46065	0.83252	7.01430	6.37883	16.19839	18.00321	6.91437	12.79607	3.50275	4.00661	7.55876	4.15795
**Dyrk1b**	0.01267	0.00804	0.01990	0.10364	0.00903	0.00642	0.00725	0.01121	0.16024	0.29832	0.01066	0.00801	0.38926	0.01132	0.00843	0.00717
**Dyrk3**	7.16474	2.96058	6.35521	8.48109	0.18979	0.75193	6.83574	3.06578	7.30452	1.92674	2.46380	2.49703	3.55296	0.00000	7.10504	0.26894
**Dyrk4**	0.77392	0.00714	0.00269	0.00324	0.00637	0.00691	0.00672	0.01037	0.75126	0.00286	0.00608	0.02772	0.01191	0.00709	0.51574	0.00635
**Dysf**	1.22620	0.00425	0.69318	0.84985	2.88314	0.00625	0.00356	0.00000	1.49220	9.51966	2.93318	2.42925	3.45288	4.88316	5.85675	0.05426
**Dyx1c1**	0.00855	0.00800	0.00332	0.18648	0.01000	0.00681	0.00691	0.06131	0.00506	0.11646	0.25404	0.00000	0.08818	1.73939	0.09752	0.00732
**Dzank1**	0.00000	0.02921	0.00000	0.41070	0.06607	0.01529	0.01742	0.48168	1.28842	0.34090	1.13571	0.03063	0.03516	7.62188	1.14482	0.00685
**Dzip1**	0.01718	0.00806	0.01035	0.01408	0.00899	0.00683	0.00747	0.01411	0.01272	0.71909	0.01215	0.03311	0.01525	0.20292	0.00000	0.00775
**Dzip3**	1.14918	2.47968	0.00000	2.44335	0.01405	0.46724	0.00505	0.00391	0.31934	0.48482	0.00701	0.01051	0.48431	0.00242	1.41245	0.00468
**E2f1**	4.26268	0.38317	2.62624	13.83246	4.44076	0.02222	1.07245	3.26254	9.27554	13.43814	2.46894	0.67400	3.27710	0.31922	9.67179	4.34994
**E2f2**	0.07276	0.00615	1.82723	1.22503	2.75567	0.01443	0.00633	0.00000	3.70723	0.46437	0.59633	0.04667	0.57057	0.02384	1.17558	0.16651
**E2f3**	0.02185	0.00600	0.00044	0.00000	0.00875	2.10967	2.81513	0.01032	0.53331	0.00332	0.00626	0.00443	0.00313	0.00663	0.26242	0.06230
**E2f4**	4.98623	5.31960	5.40601	17.08484	4.81603	2.19962	3.59841	9.02447	13.06081	8.41216	6.74385	2.01017	8.98989	1.17143	7.49363	4.94801
**E2f5**	0.21774	1.93852	0.79376	21.11555	0.00615	0.00191	0.00467	1.05209	9.34912	1.57473	0.41005	4.76529	0.00674	1.94107	5.56120	0.00142
**E2f6**	0.78369	6.78118	2.67789	0.57502	5.05543	0.89944	3.86451	1.11010	0.00000	0.89832	0.31518	7.71053	1.46302	0.00242	2.29385	0.00000
**E2f7**	0.01222	0.00703	0.00301	1.14481	0.62921	0.00820	0.00645	0.74227	0.40498	0.29400	0.46807	0.02666	1.05800	0.00289	0.03947	0.00709
**E2f8**	0.00333	0.00725	1.09496	1.32516	0.00948	2.04979	0.00622	0.71935	0.44694	0.84427	0.92835	4.99828	1.72265	0.00244	0.39369	0.00495
**E4f1**	1.59406	0.02010	3.02279	7.76242	5.71557	5.16668	3.82131	5.94860	10.11835	4.76879	4.99046	0.37864	5.08019	0.00000	0.54714	2.29113

**Eaf1**	0.64183	1.40636	4.91976	8.79209	1.54895	1.06135	5.11745	5.46825	6.28157	3.41327	3.87468	6.98526	3.51298	10.06795	6.11796	2.53502
**Eaf2**	0.14303	0.01846	0.48593	3.38011	0.45310	0.43558	0.01684	0.44201	5.63065	1.82623	0.37896	0.59365	0.59404	0.94316	0.55678	0.03513
**Eapp**	10.94961	8.33611	9.97313	5.02933	4.41053	5.87697	5.64360	8.26280	6.90734	7.21705	12.07579	3.09046	4.92722	6.85583	4.76219	3.47036
**Ears2**	3.44832	6.88887	4.52176	3.64204	0.28084	0.01424	0.77132	3.36207	7.26653	3.23365	4.15474	0.45067	3.82780	0.74474	3.65166	3.99185
**Ebag9**	2.89614	4.03183	0.24612	0.30481	2.67371	1.62400	1.38838	0.65859	0.00671	1.12727	1.03222	0.00000	0.07468	0.03316	2.02165	2.11077
**Ebf3**	0.01338	0.00778	0.01121	0.69041	0.00936	0.00644	0.00736	0.01723	1.17942	0.00285	0.00002	0.00509	0.01282	0.02759	0.30808	0.00725
**Ebf4**	0.01313	0.00810	0.01213	0.01057	0.00912	0.00677	0.00759	0.01437	0.01226	0.03081	0.01259	0.00868	0.01249	0.01199	0.01153	0.07293
**Ebna1bp2**	20.52178	11.86518	16.36241	17.06951	14.02536	10.35123	13.76243	23.63263	28.25185	20.53975	17.06300	9.86070	15.69508	11.43374	11.72978	7.45844
**Ebp**	10.90007	5.78974	25.72837	3.31599	7.39235	6.16357	8.41645	10.37605	6.49687	5.23201	4.92197	2.06893	3.25986	2.73445	10.22012	0.07299
**Ebpl**	0.90833	0.00668	0.08916	8.25934	0.06262	0.00602	0.00565	0.12821	0.77139	1.04958	1.12235	0.13484	0.33944	0.18449	0.23294	2.06355
**Ecd**	6.60865	8.84692	4.38894	3.52906	5.08618	7.39730	4.43095	12.47527	4.58260	11.97048	9.65367	2.34964	3.08230	2.69034	9.09299	4.89682
**Ece1**	0.02182	0.00782	0.00740	1.03768	0.00843	0.00699	0.00703	0.00226	0.32214	0.85917	0.55327	0.03290	0.01498	0.02148	0.04614	0.00723
**Ece2**	1.04909	2.91438	1.76398	3.89689	0.02125	1.11726	4.83827	3.12741	8.08097	8.54041	3.63872	4.76011	1.37372	0.57465	4.07598	0.31282
**Ecel1**	0.00735	0.00797	0.01255	0.07864	0.01881	0.00660	0.00673	0.01050	0.01137	0.00518	0.01397	0.00725	1.46374	0.01274	0.00893	0.00663
**Ech1**	11.06737	1.99209	11.53504	0.45947	3.51171	6.15302	5.56959	4.51299	0.00000	3.90377	0.70433	0.00956	6.18822	0.00590	0.40124	7.18470
**Echdc1**	0.03518	0.00827	0.79303	0.01050	0.00693	0.00727	0.01916	0.03614	0.01228	0.00884	0.00997	0.00726	0.00927	0.25616	0.00000	0.00712
**Echdc2**	30.42771	4.35068	19.62947	5.73533	21.81564	12.22523	11.62422	28.08668	13.80028	26.92008	24.46780	6.01703	25.91479	2.92835	0.27409	17.98749
**Echdc3**	0.01316	0.00833	0.01313	1.37430	0.00985	0.00675	0.00769	0.01485	1.31031	0.03168	0.00000	0.00557	0.04337	0.02518	0.12924	0.01461
**Echs1**	16.05249	5.86370	12.82102	3.81318	9.11816	0.66661	6.73467	3.67111	1.48456	1.72517	1.20929	0.04531	10.30329	1.50341	1.23247	9.65169
**Eci1**	2.63454	0.74884	3.63834	6.55034	0.03507	1.94469	0.05989	1.82659	15.30862	8.85774	11.47958	4.03514	2.23009	2.34565	3.35281	5.20561
**Eci2**	8.48990	5.31195	2.20723	0.61390	7.90292	4.77966	6.88197	5.67200	3.70207	3.51236	7.55308	0.41830	1.43611	0.00000	1.34641	7.13802
**Eci3**	0.56155	0.10096	0.06911	0.00665	0.20811	0.09155	0.13319	0.12172	0.06737	0.04742	0.01101	0.00713	0.03122	0.00992	0.03174	0.19393
**Ecm1**	0.00640	0.00766	0.04156	0.83385	0.00814	0.03835	0.00718	1.39221	0.12280	2.78716	0.15395	0.11834	0.70179	0.00941	0.07882	0.43760
**Ecm2**	0.06974	0.01775	0.13767	0.47633	0.01028	0.04038	0.04681	0.63745	0.77583	0.28893	0.62090	0.84556	0.13326	0.35574	0.35735	0.04259
**Ecscr**	0.01349	0.00816	0.01167	0.00427	0.00929	0.00658	0.00756	0.01378	1.76037	0.00863	0.00000	0.00535	0.01318	0.01378	0.00891	0.00731
**Ecsit**	6.05551	4.97166	9.05256	4.92166	3.57436	3.03653	5.22783	7.11952	7.08917	12.12295	7.35653	2.10387	6.29360	1.15913	8.00127	1.96827
**Ect2**	0.50950	7.99698	1.44646	4.06803	6.90562	0.00437	3.41445	5.89742	15.25489	10.72548	6.20858	4.88769	0.79950	25.38127	14.47273	2.14426
**Eda**	2.02103	0.00382	0.07585	3.93704	0.00534	4.53627	0.06317	5.15662	12.31264	9.31472	0.94722	4.41362	4.62648	13.23259	6.22891	0.00682
**Eda2r**	0.54400	0.00797	0.48411	0.01056	0.01654	0.00863	0.00678	0.00841	0.01233	0.01118	0.01111	0.07425	0.00941	0.07389	0.00000	0.00690
**Edar**	0.01373	0.00821	0.01217	0.00524	0.00933	0.00661	0.00751	0.01348	0.17660	0.01073	0.01148	0.00815	0.01272	0.00857	0.08594	0.00739
**Edaradd**	1.26710	0.00619	0.00000	0.00145	0.01627	0.00512	1.44053	0.00000	0.03660	0.00000	0.34479	0.00000	0.02669	3.33393	0.48101	0.00587
**Edc3**	5.90465	0.00000	0.46360	1.23033	3.08046	4.59211	0.98685	2.15935	0.43231	3.85150	0.95738	0.28058	2.57031	1.08693	3.86393	0.01986
**Edc4**	10.02371	3.98984	3.23980	4.39520	4.47539	5.61554	2.89625	10.94504	2.88870	9.07868	5.70044	1.07850	2.63525	0.11013	2.87685	5.77432
**Edem1**	3.00932	0.61893	3.15506	4.44139	2.04481	0.76828	0.47216	1.51005	4.33896	1.90374	0.98903	0.02089	3.00391	0.02231	4.10453	4.81375
**Edem2**	5.44039	0.00000	0.41660	1.04039	3.78394	0.83592	6.12849	3.13994	0.30112	3.48131	0.81874	0.17359	7.32154	0.00696	0.00000	4.50033
**Edem3**	0.36614	2.25283	0.00000	0.83918	0.00699	1.75654	1.26739	1.71564	2.42012	0.00000	0.91534	5.72942	1.88842	7.34738	3.87215	0.00350
**Edf1**	12.60414	0.51881	4.79579	9.26380	7.44517	4.34431	2.42688	9.66389	5.29769	23.22136	15.15750	1.18013	20.13444	0.02920	0.00000	6.16579
**Edn2**	1.01038	0.00603	0.00223	0.01126	0.00576	0.00781	0.00634	0.93183	0.01136	0.00451	0.00725	0.00506	0.00540	0.01640	0.00000	0.00626
**Edn3**	0.01335	0.00776	0.01156	0.00297	0.00920	0.00645	0.00734	0.01074	0.40885	0.49535	0.00774	0.00484	0.01169	0.00647	0.62019	0.00728
**Ednra**	0.32783	0.00847	0.00434	0.23935	0.01639	0.00670	0.00684	0.00981	0.00528	0.00532	0.01177	0.00753	0.56752	0.01163	0.01158	2.25261
**Ednrb**	0.01687	0.00788	0.03022	0.39911	0.00899	0.00799	0.02794	0.01193	0.11721	0.03100	0.02665	0.00721	0.03255	0.07794	0.02698	0.00740
**Edrf1**	1.76176	3.85102	0.52007	2.32514	0.00401	0.00386	4.03487	0.91023	0.30079	1.09282	1.27225	2.06915	1.66045	2.04949	3.80588	0.33583
**Eea1**	3.65636	0.00000	1.30509	8.02829	0.00000	5.31605	0.20704	0.47171	3.10115	0.00000	0.66470	6.33138	1.53612	6.42352	6.97580	1.57206
**Eed**	16.31256	14.62607	15.44079	6.95726	12.65641	7.20952	11.15713	14.32302	5.82267	16.92989	8.26146	7.57310	12.71284	19.05807	12.09880	9.69664
**Eef1a1**	69.78303	43.64737	65.23476	20.36106	49.82072	32.30229	37.57081	67.94116	28.28754	62.50518	51.54959	32.15995	64.71997	58.40761	40.56137	40.06266
**Eef1akmt1**	11.79667	5.10159	9.40855	4.90914	0.31845	2.59886	4.72727	9.96479	7.05133	15.64947	13.78636	2.56030	7.28153	6.76825	9.53816	0.99604
**Eef1b2**	49.56292	27.29162	40.00142	13.70375	30.95040	17.71359	24.92137	44.77663	20.87978	46.01499	44.54728	20.99167	39.84460	13.01457	9.80809	24.34535
**Eef1d**	20.72687	17.65129	23.78617	10.37205	14.68614	7.96275	13.79385	15.27823	10.50457	15.97440	24.73174	11.61532	20.19897	5.13776	10.43813	15.80399
**Eef1e1**	17.20347	10.31405	18.14682	3.25692	13.52788	7.16889	7.00325	17.84262	4.71072	23.65616	14.71817	12.67334	16.70101	22.44259	12.07593	6.90375
**Eef1g**	49.87631	29.12437	43.58570	3.80897	31.53903	24.34191	26.61684	50.63878	8.53335	42.33679	35.31920	14.30629	46.33719	24.77649	23.18612	30.21435
**Eef2**	38.74268	23.87693	34.94108	14.98821	24.73652	17.69242	23.18076	51.71832	21.47609	40.49158	35.63027	9.80102	39.40588	3.24284	13.27953	24.19761
**Eef2k**	1.97239	0.33391	0.02861	0.88718	1.72397	0.00644	0.01500	0.75532	0.48773	0.08226	0.77465	0.01570	0.00000	0.04855	0.62442	3.14779
**Eef2kmt**	5.32680	4.79893	2.82173	0.07593	1.73998	0.03045	2.97392	3.90364	0.20715	1.11158	3.65183	0.01474	3.10391	0.21961	0.41033	0.26453
**Eefsec**	8.74701	0.14697	1.13478	2.58327	3.72502	0.00571	0.44908	1.69467	2.83163	2.74059	0.63027	0.12951	2.31252	0.00201	0.00000	1.97885
**Eepd1**	0.01368	0.00798	0.04801	1.02915	0.00842	0.00851	0.00717	0.22638	1.22666	0.16901	1.40846	0.00000	0.77038	0.00944	0.00000	0.04820
**Efcab10**	1.13880	0.01673	0.00175	0.00971	0.95359	0.00700	0.00642	0.00774	0.01179	0.05108	0.01334	0.00638	0.10393	0.01662	0.00935	1.41771
**Efcab11**	0.08334	0.00723	0.52480	0.30688	0.00741	0.00555	0.00707	0.00977	0.00642	0.03147	0.01117	0.00547	0.00949	0.00696	0.65056	0.00723
**Efcab12**	0.25305	0.00758	0.03766	2.17338	0.00747	0.00530	0.01696	0.00983	1.86954	0.00000	0.00000	0.00000	0.01194	0.20806	0.90343	0.00638
**Efcab14**	3.02406	0.02428	3.02258	5.43266	4.36248	0.04001	0.54334	0.01087	2.72435	3.34897	0.07600	0.02941	1.31016	0.84215	2.78662	3.51464
**Efcab2**	0.49465	1.55820	0.02744	0.08094	0.00693	0.00566	0.02602	0.00836	0.07310	0.02737	0.01087	0.02848	0.69016	0.00740	0.00000	0.04155
**Efcab3**	0.01016	0.00801	0.00000	0.72471	0.00926	0.00639	0.00655	0.00000	0.00000	0.29737	1.42901	0.00950	0.00000	2.20365	1.57665	0.00669
**Efcab5**	0.00631	0.00839	0.23546	1.18413	0.00895	0.00576	0.00732	0.07870	1.40514	1.06305	0.00000	0.01528	1.13256	0.00760	0.34227	0.00643

**Efcab6**	0.01369	0.00820	0.01182	1.67446	0.00939	0.00643	0.00740	0.01299	0.54550	0.03838	0.00948	0.02788	0.01292	0.00861	0.01208	0.00716
**Efcab7**	0.43836	0.00748	0.09279	0.49997	0.00703	0.00528	0.00702	0.01286	0.04748	0.04985	0.01253	0.00741	0.01086	0.01050	0.00347	0.00661
**Efcc1**	0.11951	0.00773	0.06210	2.26279	0.02652	0.02240	0.00605	0.00000	3.49106	0.00000	2.97282	0.00000	0.02178	0.75081	0.00000	0.01324
**Efemp1**	0.01945	0.00799	0.00628	0.00920	0.01046	1.97577	0.00721	0.01095	0.01270	0.05562	0.01012	0.00768	0.01125	0.01152	0.01020	0.00673
**Efemp2**	0.01134	0.00803	0.66959	1.32463	0.01712	0.00555	0.00746	0.01091	0.00000	0.71153	0.00993	0.09710	0.01516	0.01297	0.00000	0.00670
**Efhb**	0.31740	0.00728	0.00000	0.59028	0.00705	0.00547	0.00605	0.00000	1.23422	1.90722	2.46001	0.17308	0.00000	6.23121	2.78236	0.00625
**Efhc2**	0.12430	0.00732	0.36936	0.33190	0.00727	0.00551	0.00713	0.01191	0.00601	0.00658	0.01238	0.00645	0.01022	0.00560	0.33010	0.00690
**Efhd1**	1.78667	2.26705	0.97432	0.95733	0.07051	0.01023	0.02415	5.44415	1.14207	5.72557	2.31592	0.71828	1.39081	1.84223	1.50403	0.00324
**Efhd2**	1.35766	0.05814	3.89181	0.00000	7.48998	7.68803	3.68518	3.95705	0.01042	3.98290	2.00325	0.18408	9.27707	0.04982	0.00000	2.29037
**Efl1**	3.17530	0.87528	2.35212	0.06682	2.28857	1.89987	0.92012	1.07617	0.80964	0.27129	0.56326	0.10355	2.85123	0.40609	1.23351	1.88179
**Efna1**	0.18424	0.00634	3.85236	1.41155	0.00000	0.02256	0.00325	1.40556	1.17187	4.98466	5.05504	0.26288	2.77902	0.68393	1.94612	1.51685
**Efna2**	2.17428	0.00532	0.00000	0.05256	0.03440	1.86310	0.88021	2.47614	0.00744	2.02929	1.04752	0.13043	0.16067	0.01370	0.00280	0.04133
**Efna3**	0.36341	0.00700	0.00347	0.00889	0.05658	0.00720	0.16589	0.00765	0.01209	0.35289	0.01264	0.00569	0.55058	0.01111	0.01178	1.02746
**Efna4**	1.12665	0.00756	0.00000	0.10912	0.01410	0.00612	0.00567	0.00597	0.47114	0.00076	0.00945	0.01731	1.16080	0.00996	0.11121	4.02818
**Efna5**	0.01054	0.01809	0.02258	0.38812	0.02858	0.02348	0.02683	0.01108	0.03436	0.20991	0.10392	0.00000	0.01060	2.20289	0.00000	0.00728
**Efnb1**	0.00687	0.00862	0.00925	0.29340	0.01851	0.00698	0.00781	0.26310	0.00716	0.24519	0.19353	0.00803	0.83277	0.01123	0.00000	0.00690
**Efnb2**	1.50163	2.58965	0.25260	6.11004	1.53602	6.53994	0.00360	1.34079	5.87525	0.26749	1.45258	0.53543	0.39340	4.54731	4.56703	0.61280
**Efnb3**	3.15498	0.00401	0.00000	0.90031	0.01905	0.01355	0.00466	1.05844	0.13971	0.58803	0.00000	0.00152	0.00559	0.00771	0.12843	0.07274
**Efr3a**	4.51257	0.00000	0.65198	4.68881	0.08232	0.08019	2.98282	1.08446	0.45608	1.15696	0.03441	0.41156	1.19522	4.23361	3.98411	0.42363
**Efr3b**	0.05116	0.00804	0.10465	3.91614	0.01246	0.00578	0.05737	0.00000	2.62204	1.28940	0.76022	0.33181	0.06676	0.27802	0.73124	0.07219
**Efs**	0.01375	0.00813	0.01230	0.36292	0.00931	0.00662	0.00752	0.01393	0.00657	0.01541	0.01413	0.00822	0.01266	0.01118	0.00614	0.00735
**Eftud2**	15.27751	11.53767	11.11303	1.00928	14.57485	1.41995	11.51143	19.38463	0.35661	17.04526	8.24060	4.79694	13.31932	2.91942	9.27004	6.31283
**Egf**	0.31227	0.01841	0.00000	1.36891	0.00783	0.00821	0.00720	0.07068	0.01159	0.01758	0.11471	0.03410	0.18221	0.96657	0.69308	0.00702
**Egfl7**	2.72028	1.95270	1.97493	0.23220	0.04010	0.00433	3.25755	0.37102	0.00740	0.08045	1.09288	0.02946	2.84704	0.00548	0.08137	2.22399
**Egfl8**	0.01280	0.00742	0.00000	0.15253	0.01089	2.99764	0.00677	0.57115	0.11956	1.04829	0.32733	0.07900	0.00000	0.01126	0.00000	0.00612
**Egflam**	0.01261	0.00765	0.01212	0.14952	0.01865	0.00640	0.00728	0.01036	0.85994	0.76673	0.00583	0.00352	0.01156	0.00652	0.04973	0.00737
**Egfr**	0.01703	0.00781	0.01081	2.99849	0.00900	0.00624	0.00725	0.02289	1.41552	0.00167	0.01608	0.02424	0.01182	0.00928	0.19616	0.00670
**Egln1**	6.34449	11.57576	10.63597	6.87596	4.32232	12.13974	12.00017	18.37455	7.96268	10.83563	12.45085	4.87606	11.99324	0.66801	4.82286	3.25170
**Egln2**	9.23125	2.28479	3.06747	5.28893	5.44593	2.56377	0.43795	7.24686	5.95173	7.71552	2.02985	2.35443	6.64302	0.00000	0.15809	2.85283
**Egln3**	0.01651	0.01986	0.01012	0.09180	0.00959	0.00743	0.00739	0.00499	0.20032	0.00000	0.48252	0.02550	0.74593	0.02497	0.00000	0.00716
**Egr1**	5.05247	0.00225	0.00293	1.55563	0.00000	0.00356	0.00314	0.00000	0.00000	0.55023	0.01175	0.02156	0.00967	0.00789	0.01123	0.91639
**Egr3**	0.01306	0.00777	0.01187	0.00469	0.00888	0.00642	0.00722	0.00963	0.59368	1.23625	0.00881	0.00430	0.01039	0.02132	0.00502	0.00707
**Ehbp1**	0.01571	0.00786	0.00000	5.61022	0.00945	0.16371	0.00706	0.00000	6.92491	0.04738	0.71907	0.00000	0.20089	0.00000	0.90252	0.02254
**Ehbp1l1**	1.88983	0.06940	0.45761	0.64422	0.01450	0.06455	0.14123	0.09402	0.70542	0.79068	0.90699	0.05701	0.23811	0.46275	0.16847	0.06645
**Ehd1**	8.44508	6.28472	8.81445	1.17496	0.57623	5.48121	4.66663	15.31399	1.89696	10.54635	9.09363	0.27327	14.20461	0.00000	1.77300	11.19048
**Ehd2**	0.01357	0.00781	0.00394	0.12757	0.00909	0.00648	0.00710	0.00000	0.00862	0.02657	0.58942	0.06253	0.01209	0.00585	1.80134	0.00697
**Ehd4**	3.77793	8.37379	9.63398	4.97467	9.70842	3.30555	5.17589	5.09143	5.14667	1.13898	2.49010	4.15482	5.35013	2.34003	0.00000	3.05216
**Ehf**	0.01353	0.00768	0.01021	2.38263	0.00909	0.00652	0.00729	0.00689	0.34301	0.47574	0.14633	0.00497	0.01082	0.00688	0.43004	0.00695
**Ehmt1**	3.51092	0.43667	4.79010	4.37588	3.86914	2.98150	2.05630	2.76758	4.75619	9.52799	2.34022	2.32254	4.11886	0.65095	6.95345	3.10079
**Ehmt2**	3.83307	0.27603	0.00000	1.72595	4.46692	6.22714	0.28629	14.34448	1.98718	18.98841	6.17177	0.86380	10.62630	0.04584	0.16464	9.37488
**Ei24**	12.65220	6.05914	9.49914	21.80748	5.99540	0.11392	9.08272	14.61313	20.96674	25.28278	14.85829	7.73410	11.41420	22.83053	22.26230	0.47337
**Eid1**	0.01580	0.00761	0.09575	0.00714	0.00888	0.00581	0.00726	0.01038	0.01181	0.71509	0.01074	0.02446	0.01074	0.00608	0.16966	0.00729
**Eid2**	1.74398	0.26590	1.04184	12.55048	3.52648	2.43597	0.02477	0.05498	21.63492	1.58223	1.02400	0.00000	0.21572	0.25986	0.50654	0.85912
**Eid2b**	0.00000	0.00606	0.88062	1.31060	0.00849	0.00826	1.57356	0.36535	3.03001	0.00000	0.40190	2.83920	0.08352	0.00240	0.02819	0.00618
**Eid3**	0.01347	0.00787	0.01748	0.35495	0.00938	0.00624	0.00744	0.01225	0.02547	0.00677	0.01294	0.00706	0.01253	0.00576	0.42668	0.00729
**Eif1**	24.54950	7.59836	13.61329	37.55791	17.70441	7.94040	6.52911	17.81391	44.55763	39.73742	38.53285	8.46804	20.36753	17.77321	9.86203	11.32611
**Eif1a**	13.82694	17.05912	18.47677	9.65701	11.47493	10.54284	11.16429	18.80156	16.85141	25.84414	13.94800	15.07812	10.53513	29.17889	15.89177	8.89039
**Eif1ad**	14.62695	3.03241	11.32698	8.56970	9.09957	10.38663	5.36454	17.33078	19.55469	16.21668	12.50379	5.36432	14.54017	1.54977	4.99595	6.54190
**Eif1ax**	23.19782	3.69070	6.06279	8.39472	8.01014	6.95258	1.64358	14.65445	13.61661	18.85285	9.46521	1.45367	15.71806	5.48338	0.00000	5.28477
**Eif1b**	5.77757	0.01735	2.57249	8.38863	8.53736	0.14609	0.00000	1.28612	4.89553	8.62632	5.98436	2.03343	0.63026	2.77912	2.06020	5.14054
**Eif2a**	9.27829	9.32000	6.93034	6.12877	15.09660	4.48257	5.31116	11.46419	7.43606	16.56633	9.15875	8.81020	5.85407	23.00319	15.07627	2.57849
**Eif2ak1**	2.01491	0.01484	1.10695	3.39280	0.21068	1.09963	0.02401	1.22570	5.49771	3.46929	2.60291	5.50059	1.66013	0.68354	3.31177	0.51967
**Eif2ak2**	0.22620	0.00731	0.33909	0.06468	0.00706	0.01145	0.04911	0.05891	0.35616	0.05633	0.11602	0.07269	0.00935	0.20463	0.05350	0.00698
**Eif2ak3**	0.01454	0.02656	0.75376	0.82627	1.10208	0.00749	1.29525	0.64314	1.12675	0.07037	0.00000	0.88834	0.47953	0.75673	3.29428	0.01343
**Eif2ak4**	0.92102	2.32383	0.14925	6.13701	1.23920	4.34494	0.00371	0.00000	0.93632	0.12817	1.88208	3.69301	0.63171	0.00000	1.84569	2.24123
**Eif2b1**	18.17664	14.16980	13.25842	7.34668	5.42558	4.34798	12.25289	24.89500	9.82775	24.55394	9.95114	12.72005	13.52391	3.68895	18.09363	11.25108
**Eif2b2**	10.12457	0.45670	11.00459	5.97510	0.45634	4.36867	5.51220	12.27161	14.58429	13.24314	9.27163	4.05610	7.15016	0.00000	4.15379	9.93204
**Eif2b3**	6.08219	12.19652	11.39314	3.25920	10.61656	8.86264	8.92249	11.07402	8.69011	13.39218	17.55542	5.75016	10.18963	13.55104	13.63272	4.23637
**Eif2b4**	10.93180	7.23086	9.43511	14.06328	7.47139	4.85921	5.85705	14.53269	21.23071	18.23916	12.63950	7.84060	13.88299	0.00000	1.14090	8.34555
**Eif2b5**	12.25447	9.88647	14.25256	8.08048	8.98760	9.86940	6.72209	20.07701	6.09161	18.89739	13.28967	4.04282	18.45813	13.03819	16.68311	11.16175
**Eif2d**	16.02352	8.77954	13.39821	5.69860	10.75699	3.56885	13.26688	17.87622	1.74937	17.56060	7.97287	2.87021	13.83724	1.36445	10.58890	3.70707
**Eif2s1**	27.54814	19.10566	22.77688	11.31767	21.56123	13.25630	12.79381	22.24749	20.41568	28.00790	17.46522	11.69110	19.56242	28.65406	19.54991	11.69566

**Eif2s2**	46.68192	31.49928	38.57171	16.35664	34.38997	19.18241	24.55576	44.53640	30.71569	43.96765	39.31482	26.55256	35.45776	44.22434	29.98625	23.36122
**Eif2s3x**	19.14385	9.84722	16.40378	11.93706	15.28690	9.66585	10.58695	25.41552	10.11540	29.18551	19.03302	6.64166	17.66323	30.94417	29.33158	12.81585
**Eif2s3y**	0.00509	8.18490	0.00000	0.97519	0.00780	2.29461	1.70967	0.00000	2.00450	2.71252	5.13813	5.73444	1.40930	0.00000	0.00000	0.00919
**Eif3a**	19.80134	19.95734	16.82930	17.38766	13.67815	9.77948	12.79112	24.56321	28.44235	32.05918	23.42355	27.62029	16.73758	41.45475	34.86402	10.53568
**Eif3b**	22.88505	19.32347	21.09961	11.50669	14.53333	12.77647	15.48237	36.02174	19.83298	29.18064	18.39059	5.11454	22.53837	0.43649	7.96850	13.06757
**Eif3c**	25.41574	19.09624	23.66282	14.80617	18.26539	14.86759	20.04202	36.96488	29.05044	42.11566	29.40128	18.85484	27.88815	6.51775	13.89025	16.26887
**Eif3d**	23.98625	19.18822	23.86245	4.00423	18.07223	12.19081	16.36598	33.31907	6.62954	28.20345	24.22350	5.00863	22.42979	6.55014	20.55339	15.92413
**Eif3e**	27.28805	17.45547	19.97198	3.52203	22.04485	15.77863	14.01091	25.62616	9.65191	26.76203	21.33347	16.30201	21.07881	29.89960	22.83074	15.80110
**Eif3f**	16.60740	10.86606	10.55331	11.07901	14.47054	8.63250	8.90997	15.36067	23.70145	18.40280	25.59528	11.89743	11.05300	8.59917	3.69741	6.00718
**Eif3g**	32.72628	9.08771	19.80817	13.02862	15.43891	11.42313	16.53553	32.74664	16.13961	42.16239	19.52035	14.82928	33.45418	0.00000	0.00000	19.79536
**Eif3h**	33.07467	14.60200	27.62771	20.02241	19.97517	14.63389	16.65881	35.45166	32.17632	37.47156	30.24189	15.11862	33.16752	15.56048	11.42681	15.16587
**Eif3i**	30.22368	18.07279	25.92633	8.26964	18.80042	10.91678	16.76552	35.43436	8.70784	32.01858	18.34526	12.64167	27.41627	13.05108	17.38193	12.05183
**Eif3k**	17.60087	8.92936	17.86964	9.47675	5.64332	9.72555	10.42116	19.52360	8.04491	22.78090	21.63586	8.33817	27.83756	3.67454	7.21262	12.56677
**Eif3l**	24.35482	17.61992	19.82759	11.69103	19.56692	12.68931	15.40361	31.86987	11.10765	31.65587	24.47726	4.15454	23.64019	1.44890	14.76287	13.06421
**Eif3m**	24.96482	16.18361	26.97935	8.42239	18.99474	9.83577	12.20806	27.83995	13.53852	31.39223	21.41358	18.44860	24.00808	24.92641	19.98452	12.44821
**Eif4a1**	35.77397	28.56613	36.27921	16.73247	24.32095	18.13816	21.45622	43.17769	22.63623	42.30119	34.61161	11.99395	32.77261	23.39418	25.08586	20.90570
**Eif4a2**	13.97183	21.88639	31.09626	9.97246	7.17382	9.95707	17.98318	25.19510	10.33273	22.94375	22.64585	16.11194	23.40648	36.56475	17.11448	10.83556
**Eif4a3**	25.08237	14.94161	23.04259	17.92933	11.48126	11.21692	16.11019	37.08677	25.99772	35.11052	26.37073	13.71628	23.86790	6.84546	12.19693	10.92786
**Eif4b**	28.26226	16.82528	19.37781	13.14432	13.62239	11.66572	10.57685	26.34978	15.76686	27.24427	20.07540	10.51723	21.09055	24.68329	21.28212	15.19331
**Eif4e**	22.86821	16.23938	21.29121	27.46300	14.20950	11.05462	11.53171	31.58069	31.91825	45.12897	32.42149	30.68719	16.30425	46.31638	43.26046	6.40416
**Eif4e1b**	0.08172	1.27934	0.07820	3.17509	0.01636	0.00549	0.01686	0.00665	3.04671	0.23613	0.31609	0.05494	0.42716	0.00000	0.00000	1.15470
**Eif4e2**	18.78043	11.04969	11.95590	12.91624	9.18124	7.62833	7.62798	12.60492	10.72298	19.64585	16.22738	3.46272	13.44962	13.75893	14.79118	11.18687
**Eif4e3**	0.18613	0.00818	0.02125	10.35116	0.01451	0.00565	0.00670	0.00361	3.83892	1.48666	0.11992	0.05321	0.02269	0.00029	2.26578	0.00515
**Eif4ebp1**	22.06311	4.51224	14.13253	3.65866	15.71999	0.88833	7.68497	9.91894	0.30537	9.20106	15.66435	2.06248	26.60883	0.09305	0.00000	12.41020
**Eif4ebp2**	0.60686	0.03762	0.92294	0.71584	0.03172	0.00499	1.14781	0.19199	0.00000	6.01847	0.00485	0.03862	0.07851	0.01682	2.15106	0.20356
**Eif4ebp3**	1.73210	0.07074	0.78385	1.38034	0.00402	0.02103	0.00385	0.34101	2.57047	1.34255	1.53140	1.41335	2.31489	1.26556	0.65825	1.13768
**Eif4enif1**	6.50286	6.78383	5.88537	19.36305	1.69990	6.61913	3.13714	5.86721	16.84607	10.43819	3.10900	4.05776	2.34033	0.09493	13.42681	4.94897
**Eif4g1**	15.84960	0.20273	0.00000	4.50142	12.07012	9.22905	3.28210	22.86514	2.25672	23.33835	4.86465	0.89698	22.58964	2.50933	0.87556	11.29065
**Eif4g2**	21.29456	26.60150	27.09143	31.72455	23.17436	16.21036	20.41950	34.48577	37.06119	36.05911	28.15682	35.96657	25.94043	48.03146	36.34753	15.66978
**Eif4g3**	10.08709	7.43315	1.15109	4.69026	6.09227	7.28705	0.94244	11.43655	5.01087	8.50652	9.87449	4.26531	3.68528	5.10763	11.63802	2.15728
**Eif4h**	20.02557	12.08723	18.45381	13.00641	18.52957	16.30817	15.30095	36.20959	15.84569	45.50677	24.58546	11.90600	26.26414	8.47313	9.39296	14.58407
**Eif5**	15.19682	20.38734	27.00507	24.83876	16.49650	14.34660	16.89038	32.42591	33.18854	41.52279	36.28336	34.55694	19.83839	38.62902	31.39010	5.40640
**Eif5a**	46.88536	21.70137	40.56566	19.11828	30.59526	22.35643	22.61157	44.18563	16.18361	47.20896	31.37361	7.44778	47.46401	8.20090	21.35678	27.27967
**Eif5a2**	0.01323	0.00690	0.24612	1.33405	0.00904	0.00533	0.00670	0.00126	0.01179	0.00639	0.23289	1.27690	0.00965	2.61684	0.20915	0.00694
**Eif5b**	15.64280	14.36477	13.33717	13.21734	13.94325	3.79670	9.63570	15.95243	16.06669	24.47003	17.72511	22.76255	13.96495	35.55705	27.28031	7.08267
**Eif6**	21.09302	3.80007	13.37459	21.63877	13.00982	7.90749	6.33684	28.30643	24.61987	35.55646	17.87536	4.50459	19.74626	2.98662	4.24434	12.37639
**Elac1**	1.74941	4.68360	1.59215	0.58978	0.01442	0.00431	1.65610	0.53920	0.03999	0.85356	0.58167	0.04931	0.37276	0.07466	0.32200	0.01064
**Elac2**	4.38191	3.39771	5.69657	0.36559	1.63830	1.89589	5.94247	8.83044	0.37685	5.20385	0.27557	1.11509	9.42959	0.03653	2.33167	5.41919
**Elavl1**	10.23243	6.76424	14.89544	1.00554	3.38927	1.18593	3.94639	8.22794	2.50305	10.98246	5.29057	4.12633	6.52109	3.80009	4.83600	5.84416
**Elavl2**	0.24671	0.00805	0.00000	12.90499	0.01830	0.00471	0.00655	0.00608	2.20463	0.21116	0.00000	0.02774	0.01901	4.32581	5.46195	0.00516
**Elavl3**	0.34483	0.00640	0.54640	3.58423	0.00631	0.10207	0.00514	2.62577	1.08363	1.12320	2.98874	0.05361	0.66057	0.00083	1.44917	0.00534
**Eldr**	0.01283	0.00797	0.82176	0.00698	0.00790	0.00563	0.00752	0.00887	0.02268	0.00702	0.13595	0.01817	0.00758	0.07835	0.00000	0.00701
**Elf1**	0.48702	0.00578	1.02596	8.55856	0.04977	0.00542	1.86567	0.00000	14.20725	0.14692	0.79075	2.65056	0.25280	4.31687	3.05304	0.02638
**Elf2**	2.01903	2.46777	1.50596	1.06185	0.15142	0.02347	3.09995	3.64972	0.10169	2.54465	0.87556	0.07248	1.32503	1.52194	3.83822	0.02438
**Elf3**	8.64130	14.38786	15.52360	1.13699	19.01711	19.55228	18.66411	27.39984	0.57254	5.01188	5.81106	0.17300	29.72227	0.52353	2.75742	16.22996
**Elf4**	2.38714	0.00472	0.55122	0.40149	0.02306	0.03507	0.00515	0.87881	0.01688	2.27766	1.01833	0.65442	0.00000	0.05682	2.13509	0.02665
**Elf5**	0.84285	0.00705	0.00436	0.01062	0.00620	0.00709	0.00681	0.01334	0.01235	0.01501	0.01267	0.00751	0.01169	0.01231	0.01086	0.02793
**Elk1**	0.96273	1.30717	0.35229	2.49099	0.00714	0.04725	0.03648	2.24139	1.35554	1.96119	2.35856	0.12136	0.12618	4.18131	2.08245	0.12787
**Elk3**	0.44706	0.00757	0.00388	0.00533	0.00686	0.00772	0.00768	0.07620	0.01112	0.01936	0.51262	0.01719	0.01427	0.01219	0.00194	0.00698
**Elk4**	0.31841	0.00815	0.01267	1.45719	0.01544	0.00584	0.00696	0.00842	1.00220	0.24874	0.02037	0.00259	0.04544	0.12509	1.15072	0.00706
**Ell**	3.61103	0.36542	0.17161	5.50583	0.00000	6.09505	0.25460	7.25724	2.63895	1.92725	3.20878	0.22159	0.21452	0.85300	1.99189	1.79684
**Ell2**	5.74108	0.00718	0.17053	10.58348	8.85192	0.27157	6.81431	7.96650	19.43756	18.70124	10.91648	19.35587	0.00000	23.79378	19.19460	0.00000
**Ell3**	0.00000	1.12391	0.00000	0.66649	0.01605	5.64527	0.03030	5.10319	1.05543	10.12921	10.68309	0.45248	6.54894	2.73568	7.59579	0.00000
**Elmo2**	2.15742	3.20223	0.74755	1.19850	2.95218	0.00383	2.62937	0.45155	1.78586	2.48004	0.20573	0.15633	2.09657	0.01995	1.25241	1.20424
**Elmo3**	1.73043	0.00489	0.02269	0.00576	0.00550	0.23236	1.67644	0.08135	0.01177	0.13120	0.35438	0.01215	1.58462	0.00577	0.10405	0.01174
**Elmod1**	0.01383	0.00820	0.00872	0.00536	0.00909	0.00690	0.00760	0.00589	0.01129	0.00591	0.50249	0.00616	0.00739	0.01095	0.00298	0.00715
**Elmod2**	0.67618	1.44470	1.90037	10.88028	0.01127	0.01138	0.68095	0.00000	3.81817	2.66404	2.89608	2.00985	0.41204	7.29441	8.36744	1.19451
**Elmod3**	1.13809	0.00507	0.25103	0.66668	1.66942	0.00414	0.01654	2.60719	2.64226	0.27707	0.00000	0.00299	0.00339	0.12354	0.00000	0.22321
**Elmsan1**	0.00669	0.00732	0.41572	1.04494	0.00862	0.02830	0.00656	1.67961	0.80514	0.81953	1.36437	0.12682	0.63573	0.25969	0.13948	0.05000
**Eln**	0.01333	0.00828	0.02186	1.28167	0.00928	0.02001	0.00750	0.01394	0.00000	0.01498	0.01359	0.00847	0.01217	0.01055	0.00314	0.00718
**Elof1**	13.60122	5.12468	13.83972	12.48032	6.04979	8.28500	4.01859	14.80211	18.79525	24.34013	11.02538	8.49267	10.67581	0.98770	15.85950	5.68827
**Elovl1**	4.59310	2.58214	4.48629	1.72979	2.55373	7.69468	2.16551	1.46712	0.68547	0.00000	0.68411	0.17200	5.72839	0.00034	0.10478	4.39935

**Elovl2**	0.01376	0.00818	0.01214	0.06045	0.00938	0.00657	0.00747	0.01289	0.03539	0.00876	0.01204	0.00817	0.01270	0.00752	0.19438	0.00737
**Elovl3**	0.01362	0.00724	0.00731	0.40167	0.00928	0.00671	0.00705	0.00979	0.00547	0.28243	0.00752	0.76382	0.01251	0.04923	0.04043	0.00736
**Elovl5**	5.90522	4.11634	7.64989	0.11128	0.02429	1.71214	7.38798	5.99979	0.00331	1.18131	7.11216	0.00000	1.24304	7.77566	0.00000	1.84161
**Elovl6**	13.25463	12.74455	15.39927	7.46646	10.53238	14.33882	12.57105	19.03607	16.09876	15.60958	16.64536	7.92083	13.61736	34.23491	18.93537	9.10128
**Elovl7**	3.42150	6.28667	6.96011	1.44738	5.90103	0.13608	3.94097	0.54575	0.48829	0.00000	0.59954	0.04134	2.86650	2.37770	0.88638	1.51202
**Elp2**	10.88802	8.99823	10.67721	1.13576	1.45753	0.00000	7.35815	9.88938	4.39351	5.46183	9.31663	7.29782	6.61997	8.81672	7.88275	5.39030
**Elp3**	9.33581	12.02303	12.87955	0.00000	6.52641	0.21891	10.57644	12.62685	0.01674	4.52809	6.72227	0.22151	8.66848	2.22734	5.43907	2.64215
**Elp4**	0.19815	1.58567	3.89772	0.22369	0.01114	0.01207	1.08405	1.64559	0.10891	2.38002	3.50108	7.75469	2.23781	0.75141	0.00000	0.15248
**Elp5**	18.79399	10.08750	14.22193	13.89681	10.30660	1.26410	8.88268	9.88922	23.18363	17.27390	7.17662	4.45292	10.76564	9.36571	12.16927	9.54017
**Elp6**	4.48090	1.50899	2.94432	8.13225	0.04903	4.74294	2.69279	8.17588	8.77493	10.48435	3.95330	4.22127	6.00094	0.11595	0.00000	0.02302
**Emb**	0.54199	0.00841	0.01111	1.70631	0.03333	0.00790	0.00731	0.15617	1.08197	6.36537	0.00000	0.00000	0.00000	0.94575	0.35216	6.93388
**Emc1**	3.79529	3.54516	6.17294	4.36142	0.03432	0.01418	0.12984	5.52168	5.05168	6.13710	12.49488	1.96887	2.12017	14.23248	6.47643	3.83409
**Emc10**	9.75253	2.96002	4.26736	0.05168	5.61146	1.97319	1.74688	2.65536	0.01848	3.56311	8.51068	3.37984	13.05807	0.00000	0.00000	5.80904
**Emc2**	8.66916	8.08146	6.37332	1.50258	5.00353	8.22105	7.00881	2.00597	1.39802	5.36279	7.45858	6.92521	7.90888	9.62009	6.52581	7.27432
**Emc3**	5.17946	4.72808	12.31739	15.22533	3.85473	10.94124	7.88015	20.72003	20.60414	29.46971	19.41276	11.76030	15.06273	16.99696	22.26326	1.91519
**Emc4**	15.57844	6.30474	11.68086	6.74735	7.40288	9.43378	8.44948	20.51404	9.49637	20.48078	9.09384	5.57420	15.60423	4.49350	2.38960	9.11370
**Emc6**	2.70268	2.58310	5.69647	6.82336	8.47370	0.49690	1.75515	1.36782	2.40656	12.34008	7.59681	0.83715	7.53817	0.00000	3.25636	2.15653
**Emc7**	6.24905	6.73566	10.92804	7.11263	6.22834	6.79883	7.06399	9.11930	11.26975	7.69350	10.12325	10.92642	6.84910	4.52237	4.67765	3.39567
**Emc8**	8.19566	0.00565	3.87408	6.77448	2.71749	4.54925	1.53097	3.44705	13.00670	6.82529	6.45675	0.48863	10.87569	0.05461	1.50731	2.48586
**Emc9**	3.46865	0.00458	0.00000	0.01808	0.01560	3.59631	0.00420	0.66569	0.84306	0.68650	1.15489	0.00636	1.00094	0.00952	0.00000	0.36379
**Emd**	0.35262	3.34998	3.17594	1.25701	0.04516	0.00706	0.06593	0.01217	0.68088	1.03108	2.72171	0.08399	1.03901	0.50965	1.08796	0.30706
**Eme1**	7.17137	4.38143	3.47820	4.74279	1.89101	4.16768	5.18746	3.20179	2.96220	10.48724	0.25163	1.83870	3.63977	0.53012	8.52193	5.52447
**Eme2**	0.75878	0.00462	0.64009	0.06712	4.04018	0.01564	0.00548	0.95808	0.05950	0.00186	0.00652	0.00025	2.04447	0.00963	0.06194	1.15615
**Emg1**	11.64739	14.64635	12.20490	0.40534	9.70903	3.69407	10.30601	19.24749	1.52463	23.21788	4.22238	9.00139	13.85347	5.76275	16.22202	9.48164
**Emid1**	0.01367	0.00799	0.01165	0.37729	0.00919	0.00686	0.00756	0.01350	0.04664	0.15802	0.01200	0.03115	0.01299	0.00817	0.05041	0.00735
**Emilin1**	0.01802	0.00808	0.01958	0.01058	0.01826	0.00638	0.00765	0.01847	0.01228	0.02503	0.01272	0.01915	0.01239	0.01226	0.01067	0.00742
**Emilin2**	0.00946	0.00824	0.01151	4.25137	0.00893	0.00605	0.00711	0.01493	0.94665	1.35075	0.02663	0.01270	0.70926	0.00000	0.22969	0.00618
**Eml1**	0.11206	0.00713	0.18789	0.00581	0.00759	0.00563	0.26155	0.00907	0.09232	0.23821	0.01212	0.00615	0.01070	0.01195	0.00701	0.00746
**Eml2**	0.66507	0.02845	1.99276	0.09498	0.01319	0.02931	0.80663	0.91941	0.00757	0.00000	0.80287	0.00871	0.00000	0.00623	0.00000	0.00594
**Eml3**	1.04677	0.01697	0.00000	1.50188	0.00657	4.36371	0.00500	1.42641	6.25334	2.76466	0.64948	0.01733	1.88984	0.00000	1.78969	0.01818
**Eml4**	3.10866	0.02252	0.31161	1.91437	2.25752	4.50944	2.07922	1.60456	1.97930	0.85275	0.00000	0.56925	0.75656	2.81141	1.69114	1.84157
**Eml5**	0.24842	0.00726	0.03529	3.05703	0.00724	0.00546	0.00710	0.36974	0.39881	0.10950	0.29253	0.05557	0.00479	0.82535	0.95670	0.00633
**Eml6**	0.01484	0.00748	0.18808	0.05229	0.00943	0.01749	0.31076	0.01158	0.04998	0.03493	0.06076	0.00544	0.07686	0.00552	0.51541	0.00741
**Emp1**	0.01370	0.00774	0.01069	0.00905	0.00912	0.00674	0.00727	0.01070	0.01206	0.36660	0.01233	0.09032	0.01244	0.01145	0.00808	0.00780
**Emp2**	0.09451	0.00614	5.01526	1.10498	0.09370	3.78798	2.58198	1.74414	2.05001	0.69540	0.76645	0.09941	1.64229	0.51519	0.09842	4.15397
**Emp3**	0.01295	0.00797	0.01140	0.47100	0.00888	0.00665	0.00729	0.01035	0.05475	1.40116	0.00991	0.02605	0.01127	0.04306	0.04373	0.00744
**Emsy**	1.77413	0.31303	0.72048	0.59421	0.19819	1.91867	2.77431	1.09473	1.60628	4.08523	0.00000	4.50712	1.43862	3.20851	3.55930	0.00343
**Emx2**	0.01349	0.00812	0.01165	0.00464	0.00922	0.00660	0.00753	0.01301	1.71960	0.08160	0.00000	0.00488	0.01311	0.01246	0.00681	0.00743
**Emx2os**	0.03525	0.00777	0.05247	0.05580	0.00871	0.02663	0.00731	0.01044	0.10463	0.57224	0.03260	0.06271	0.03951	0.09466	0.02075	0.00729
**En2**	0.00838	0.00762	0.00583	0.45072	0.77776	0.00727	0.00748	0.00876	0.09293	0.00395	0.17798	0.00682	0.01607	0.00772	0.05644	0.00710
**Enah**	22.41586	17.85592	17.80800	6.12131	18.96433	13.03535	14.81634	20.20897	3.69346	21.42863	21.29973	20.27785	19.50439	27.09138	10.39312	8.04835
**Enc1**	1.10590	2.34972	2.51649	1.97989	0.06011	0.01060	3.29556	0.66996	0.20447	0.77529	0.65695	0.14899	0.00000	0.35545	1.19421	5.78233
**Endod1**	0.28847	0.00631	1.18406	1.68167	0.03379	1.08053	2.13559	0.00000	0.00000	0.77570	0.72158	0.13500	0.98985	0.00223	1.71979	0.00588
**Endog**	0.41152	0.05875	0.05591	0.22721	0.00918	0.00531	0.00627	0.25784	0.57776	2.52580	1.12316	0.09191	0.30968	0.00391	0.18206	0.69487
**Endov**	1.11823	0.04945	2.03693	0.68012	0.24154	0.07562	2.32636	2.36251	1.01895	0.10862	1.07021	0.22521	0.10809	0.13340	0.19087	0.08201
**Eng**	2.63666	0.00499	0.07083	0.00793	0.00532	0.00623	0.00507	0.00000	0.02625	5.22895	0.50159	0.07624	0.02031	0.00764	0.10521	0.00447
**Engase**	0.20084	0.54375	1.19148	0.00689	0.01450	0.00790	0.00666	0.00779	0.02302	0.70983	0.01063	0.00418	0.01510	0.01138	0.00000	2.43620
**Enho**	0.00902	0.00739	0.00654	0.83555	0.00944	0.01334	0.00703	1.29576	0.00000	0.44212	0.00487	0.03012	0.05865	0.00908	0.00000	0.00752
**Enkd1**	2.56810	0.08482	0.00000	1.15814	0.42697	3.67978	0.01538	0.31924	1.39294	1.39591	0.05823	2.82205	1.44242	0.00763	0.00000	0.01850
**Eno1**	0.14323	0.37197	0.33370	0.01009	0.07696	0.07777	0.15818	0.09006	0.01231	0.02435	0.01183	0.01698	0.34815	0.00905	0.01019	0.21229
**Eno1b**	8.70227	7.38973	10.80995	2.34428	7.95152	3.82013	4.57661	2.71286	0.19059	2.28439	1.08774	0.15872	8.49218	0.00000	0.00000	4.55686
**Eno2**	0.04515	0.00826	0.00398	0.05643	0.02328	0.00775	0.00700	0.00257	0.07310	0.00336	2.33825	0.00828	0.06152	0.00958	0.00315	0.01388
**Eno3**	0.00069	0.00781	0.81811	0.40023	0.00794	3.51452	0.01745	0.76866	0.00479	1.73472	1.28784	0.19673	2.01222	0.00955	0.09110	1.52338
**Eno4**	0.75654	0.00791	0.59267	0.00815	0.00600	0.00487	0.00797	0.30707	0.01169	0.74063	0.00775	0.00675	0.00331	0.01628	0.00000	0.00608
**Enoph1**	3.72169	1.71726	6.41046	3.57940	5.51715	3.85437	6.71303	18.56722	8.14309	12.95601	3.88180	2.36930	10.49008	0.64563	0.02691	8.19506
**Enox1**	0.00000	0.00515	0.00000	2.48670	0.00974	0.00472	0.00560	1.20032	0.00000	1.64466	1.69262	8.60032	0.00000	1.58803	1.79776	0.01362
**Enox2**	0.01374	0.00784	0.01231	0.00539	0.00929	0.00665	0.00747	0.01305	0.12027	0.04407	0.01190	0.00699	0.01305	0.00680	0.08667	0.00768
**Enpep**	0.00000	6.41287	3.66107	0.00000	0.07423	7.41976	1.53366	1.46379	0.07295	0.00000	0.00000	0.00000	3.62334	0.02380	0.39633	1.79400
**Enpp1**	0.00000	0.00776	0.47120	0.00948	0.01837	0.00952	0.00647	0.00851	0.01207	0.28327	0.01197	0.00564	1.34227	0.01280	0.00419	1.02509
**Enpp2**	0.01317	0.00761	0.01172	0.19492	0.00899	0.00646	0.00728	0.01019	0.68324	0.83104	0.00626	0.00298	0.01173	0.00436	0.40743	0.00763
**Enpp3**	0.00000	0.00469	0.00000	1.14187	0.00806	0.01009	0.00560	1.75255	1.53307	4.34903	1.13730	9.02410	0.00000	5.65167	5.91812	0.00612
**Enpp4**	0.29375	0.01727	0.16062	0.54448	0.00742	0.49943	0.00664	0.27999	0.00033	1.60050	0.31630	0.84213	0.49529	0.00482	1.27421	0.12696

**Enpp5**	0.01160	0.00804	0.00974	1.24420	0.01022	0.12705	0.00754	0.01407	0.35905	0.05022	0.00985	0.01537	0.48298	0.00369	0.47128	0.00711
**Ensa**	0.75649	0.04941	0.09268	2.49461	0.02401	0.01999	0.00693	0.62217	4.34258	1.16616	0.10608	0.45327	0.07890	2.49074	1.48367	0.01340
**Enthd2**	0.38115	0.41057	3.41538	1.78710	0.00859	0.00509	2.34113	4.62240	1.70028	3.65740	3.13844	0.12947	0.51346	0.00038	0.09274	5.27632
**Entpd1**	0.01192	0.00793	0.01608	0.22134	0.01928	0.01403	0.00723	0.01058	0.00534	1.16056	0.01113	0.00458	0.01437	0.01190	0.00782	1.26110
**Entpd2**	0.00000	0.00583	0.00000	0.83563	0.01085	1.56439	3.76080	3.27975	0.33489	1.46741	1.84847	0.13178	2.72794	0.00229	0.28950	0.01306
**Entpd3**	0.43053	0.00649	0.13514	0.00000	0.00629	1.61649	0.00639	6.60378	1.67321	8.86599	2.24688	0.18211	0.00000	6.45211	3.35263	0.00562
**Entpd4**	0.01332	0.00769	0.00902	1.16892	0.00884	0.00676	0.00715	0.00000	0.65460	1.10937	0.45190	0.00000	0.00844	0.00256	0.12773	0.00715
**Entpd5**	1.58323	0.00659	0.41245	4.94801	0.03455	0.70746	0.02643	0.39766	1.53544	2.34617	0.31102	1.26730	0.84835	0.02816	3.67354	0.01149
**Entpd6**	2.83559	3.08658	4.64124	3.49606	0.43052	1.52264	4.15125	0.00000	1.20468	0.26537	1.25621	0.28042	3.86945	0.10024	2.60379	3.41701
**Entpd7**	0.76604	0.00802	0.68346	0.60972	0.01379	0.00753	0.01797	1.12886	0.41462	0.97309	0.00000	0.04021	0.05718	0.69577	0.31523	0.28510
**Eny2**	5.06596	0.24095	2.86932	17.17980	8.23839	4.58905	1.27192	0.22282	21.63548	3.31640	9.37880	0.91743	2.60741	5.37198	2.70561	2.12657
**Eogt**	0.61304	0.00488	0.06922	0.44571	0.00616	0.00850	3.22347	2.89016	0.45200	1.10168	0.00000	0.01059	0.85023	4.56189	0.21592	0.02866
**Eomes**	8.79155	2.74133	3.16413	0.77274	6.83242	10.08196	0.78081	4.98572	0.00000	2.62364	1.06499	0.08647	1.01293	2.19076	0.31458	6.30457
**Ep300**	0.00019	0.00548	0.07175	17.74314	3.25816	0.00242	0.00369	0.00000	17.23658	0.26854	6.41800	0.19511	0.88311	1.26638	2.95865	0.00174
**Ep400**	5.63502	4.51979	8.70624	11.91341	9.21299	0.73277	7.07205	9.20706	10.50333	10.05816	5.21446	12.70369	4.84022	7.26676	6.07460	3.54300
**Epas1**	1.34309	2.16950	2.80597	0.40866	0.08266	12.63949	7.24555	18.99565	0.14405	10.39530	14.15342	2.83689	10.89886	0.70516	0.00000	0.40740
**Epb41**	3.13457	3.79687	4.33435	7.86138	3.69857	7.87928	3.61476	4.21431	7.18266	6.96896	0.00000	0.79997	1.91104	0.00000	6.20874	5.01107
**Epb41l1**	5.28923	2.69849	5.09749	0.29162	0.01968	2.96788	3.51659	0.37058	0.04983	0.00000	0.68371	0.03388	2.33995	0.00329	0.28056	0.30994
**Epb41l2**	5.67035	7.49085	6.74793	4.13539	8.07930	5.72552	3.46175	6.20713	3.17717	0.62071	3.33180	0.38574	3.70006	7.99875	3.30033	0.50769
**Epb41l3**	0.00000	0.00767	0.01314	0.51969	0.00820	0.00689	0.00742	0.01382	0.09661	0.24147	0.01454	0.05769	0.27873	0.03704	0.39229	1.86389
**Epb41l4a**	0.00240	1.72770	0.10923	0.76174	0.00807	0.00628	0.06247	0.01643	0.00000	0.19867	0.00947	0.01735	0.14470	0.55446	0.00000	0.01433
**Epb41l4aos**	0.76394	0.00757	0.21575	1.04646	0.02836	0.01180	0.22430	0.01386	1.82735	1.07909	4.33948	0.07197	0.87017	0.15385	0.05426	0.27158
**Epb41l4b**	10.88851	5.92267	4.61904	6.28578	6.09585	3.39099	10.50061	10.51855	3.25479	3.43362	3.62470	8.40698	5.73326	6.60732	5.46120	5.15400
**Epb41l5**	0.25851	3.91629	0.19381	5.62850	0.02324	3.01031	1.54545	5.15739	1.71760	0.00000	0.11934	0.93939	2.26821	0.83018	1.03783	0.25808
**Epb42**	0.04020	0.00788	0.08788	0.08922	0.03858	0.00695	0.01702	0.01512	0.07220	0.07983	0.46218	0.00000	0.05609	1.16287	0.05366	0.03576
**Epc1**	0.75147	0.21316	1.03720	6.25703	0.01661	2.71040	0.38927	0.91857	5.64079	1.90446	0.75814	0.36878	0.06847	1.36726	0.77021	2.21033
**Epc2**	0.92921	1.56656	0.65048	12.24789	5.19525	3.27526	1.94513	3.89065	3.77149	5.42371	5.96490	6.59372	1.74837	6.15888	15.20854	1.80065
**Epcam**	10.49859	8.80256	11.77248	6.19242	4.76827	18.32685	13.57079	7.37950	5.08700	2.29364	2.19026	0.18159	21.11244	0.86141	3.98022	4.33820
**Epg5**	2.68310	0.02520	7.70691	7.90854	0.03024	1.40827	0.79661	6.38540	3.19498	3.45055	3.08269	2.11546	3.62048	0.50143	3.82835	0.01956
**Epha1**	0.01327	0.00817	0.02666	0.00848	0.00915	2.32770	0.02780	0.01112	0.01244	0.00586	0.01010	0.00873	0.00347	0.00910	0.08637	0.02064
**Epha10**	0.61792	0.00613	0.21755	2.19261	0.00722	0.00806	1.36273	0.00000	1.00165	1.56435	1.01811	0.10412	0.89681	0.05970	1.13153	0.00568
**Epha2**	0.00000	0.00735	0.55293	0.01110	0.00474	0.00941	0.00709	1.03293	0.01119	0.04143	0.00545	0.03326	0.17456	0.01460	0.00000	5.48749
**Epha3**	0.01353	0.00811	0.01187	0.20351	0.00921	0.00676	0.00762	0.01434	1.03917	0.06461	0.00615	0.00681	0.01330	0.00891	0.02189	0.00733
**Epha4**	6.40013	6.27237	6.80830	1.05216	6.52962	0.00000	0.75416	3.10114	1.43887	0.28966	1.03778	2.16653	0.19283	5.43802	1.22875	5.75830
**Epha7**	0.01350	0.01834	0.01197	0.01794	0.00923	0.00668	0.00745	0.01287	0.01254	0.03551	0.01330	0.00750	0.01298	0.03144	0.01743	0.00764
**Ephb2**	0.00866	0.00523	0.00000	0.56788	4.46269	0.99226	0.02692	0.16039	1.52131	0.45289	0.49208	0.29457	0.77995	0.53301	0.56561	0.15000
**Ephb3**	4.39385	4.88998	8.05969	1.97559	2.76662	0.50935	5.88344	3.54239	2.52465	0.00000	1.12457	0.00000	6.11291	0.00000	0.00000	8.63297
**Ephb4**	3.10910	0.23142	0.66191	1.37272	0.02144	0.00309	0.01452	1.96270	0.99227	0.15100	0.12681	0.03181	0.00447	0.03328	1.88991	1.46098
**Ephb6**	4.62066	0.00000	1.69460	0.23833	0.00487	3.37385	2.83587	0.76376	0.00692	0.11591	0.00001	0.02663	0.24447	0.00531	0.36061	0.00000
**Ephx1**	0.56563	3.62232	2.91871	0.00000	0.01181	0.01421	5.89113	0.40825	0.01179	0.75123	0.02842	0.00939	0.08441	0.00517	0.02628	0.00465
**Ephx2**	7.58040	0.00000	0.00000	0.22443	3.54256	0.00000	0.15303	0.01920	1.00887	0.00222	0.00000	0.04212	0.00428	0.00683	0.55091	6.54012
**Ephx3**	0.06022	0.00791	0.00815	0.00464	0.00798	0.00687	0.01787	0.00857	0.66661	0.01036	0.24284	0.00594	0.02480	0.03165	0.00208	0.00716
**Ephx4**	0.02087	0.00816	0.48221	1.04832	0.00794	0.00563	0.05055	0.00862	0.00000	0.00383	0.26025	0.00688	0.00871	0.00587	0.02980	0.00688
**Epm2a**	0.01350	0.00807	0.01194	0.51620	0.00926	0.00677	0.00772	0.01756	0.01672	0.06494	0.01169	0.00835	0.01293	0.00545	0.22783	0.00733
**Epm2aip1**	0.00372	0.00852	1.28768	3.55021	0.02167	0.14562	0.00748	1.21019	6.62009	3.63433	0.50877	0.04522	0.00000	3.00564	3.25468	0.02037
**Epn1**	0.28326	0.04000	0.00000	6.02671	0.70989	1.81657	0.12638	0.15005	0.26028	0.44984	2.85884	0.11070	0.12107	0.04760	0.04284	0.14283
**Epn2**	2.34662	3.75342	1.70585	14.04613	0.01218	3.74799	1.64015	6.44673	8.37811	7.69445	8.78737	4.30672	1.02434	1.90746	11.32796	0.00348
**Epn3**	0.04149	0.00837	0.06475	0.01065	0.01375	0.00850	0.00734	0.01274	0.01235	0.01415	0.01261	0.00788	0.04349	0.01252	0.00967	0.00743
**Eprs**	19.54487	16.49795	15.95379	4.66405	13.72060	13.73636	12.79397	20.25204	6.51413	13.18811	9.81504	14.00260	14.32230	19.27184	17.15020	10.31709
**Eps15**	7.26770	2.46538	6.47191	7.44829	7.51424	5.69010	4.13956	6.69002	0.76697	3.95733	2.96832	0.49249	9.18132	11.85592	2.91155	1.30479
**Eps15l1**	3.44821	4.02381	2.89816	2.54685	5.31946	0.39409	0.24507	7.25034	3.52926	2.18285	3.53090	0.31792	0.67750	1.30992	3.85655	0.31961
**Eps8**	1.03931	1.13824	0.00000	7.24724	0.00606	0.00408	0.00569	0.00186	2.10758	0.00000	0.05572	0.02750	0.01144	3.18700	7.61450	0.00451
**Eps8l1**	0.01412	0.00806	0.03554	0.00499	0.01013	0.00832	0.00737	0.00318	0.01092	2.05815	0.51779	0.00000	0.28840	0.01487	0.00000	0.00721
**Eps8l2**	1.11388	0.00527	0.23959	0.44516	0.00456	3.91676	0.26975	5.31167	0.00365	0.69192	3.85479	0.00000	3.09759	0.00000	0.00000	1.17209
**Ept1**	2.98450	2.97349	2.37278	0.74989	2.32956	0.04768	3.05880	3.71100	0.16496	1.83959	2.84545	0.41283	2.50324	11.12593	4.87085	0.25684
**Eqtn**	0.01213	0.00780	0.01099	0.04332	0.00947	0.00664	0.00731	0.05750	0.62906	0.01050	0.00795	0.00664	0.01034	0.01529	0.01015	0.00737
**Eral1**	2.21602	2.17679	0.00000	0.87701	0.02024	3.45127	1.57113	2.68097	0.95938	0.96944	0.51660	0.11768	2.27989	1.83891	4.14744	0.02308
**Erap1**	0.01475	0.00702	0.00000	0.61022	0.00880	0.00625	0.00615	0.00000	0.77600	0.04683	1.62924	1.45354	0.00000	2.55220	0.23968	0.00682
**Eras**	0.75673	0.00688	0.00196	1.54090	0.00641	0.00757	0.00622	0.81273	0.35375	0.00301	0.00695	0.00487	0.56564	0.00529	0.21563	0.00591
**Erbb2**	0.00000	0.00660	1.01445	0.00000	0.02091	7.13510	1.12496	1.12968	0.01035	0.58684	0.52292	0.00000	2.72849	0.00379	0.09500	0.00341
**Erbb3**	0.01940	0.00856	0.13644	0.00706	0.00983	0.00918	0.00791	0.22250	0.01141	0.60109	0.00907	0.00577	0.09381	0.01051	0.26296	0.02897
**Erbin**	0.00000	4.40912	0.78323	3.93377	0.00799	3.02781	1.87537	2.80253	2.78532	2.09028	0.56579	10.31404	0.00000	1.15445	2.71850	0.00244

**Erc1**	1.50088	0.01736	2.12446	0.55889	0.20786	0.01425	0.00533	0.40257	1.79704	1.51003	1.39749	0.01820	1.10552	1.07272	0.00000	0.04185
**Erc2**	0.01316	0.00826	0.00967	1.47897	0.00922	0.00673	0.00769	0.02064	0.78864	0.27719	0.00491	0.16318	0.01206	0.01064	0.00000	0.00708
**Ercc1**	4.48995	0.00747	4.57879	13.76907	7.35974	2.89615	2.10580	4.41246	5.12549	4.25293	4.65863	1.76398	5.98144	0.03005	3.01312	1.22575
**Ercc2**	1.13364	0.46074	1.00003	1.69534	0.00942	0.32320	0.00468	4.04658	2.24390	3.07401	2.94488	0.14393	0.91627	0.00000	0.00000	2.51372
**Ercc3**	3.76213	8.07829	4.09158	3.66193	9.71658	3.13087	2.17834	8.01513	7.63550	8.05168	5.36246	1.76110	2.32517	0.30775	5.53280	0.14712
**Ercc4**	1.26154	0.01689	1.83393	3.40585	0.01080	2.00308	0.01453	1.80241	4.48332	1.10500	2.19599	0.03149	2.05825	0.19912	0.01887	1.79693
**Ercc5**	0.79440	1.44083	2.06620	0.62879	0.00262	0.00585	2.17723	0.35004	0.42308	0.00000	1.53578	0.14754	0.95308	0.00245	1.31857	0.53810
**Ercc6**	1.12329	0.00604	0.29117	5.72871	0.02742	0.03145	0.07526	1.32280	7.30332	0.18358	4.35876	0.09727	4.04445	5.31361	9.51428	0.07515
**Ercc6l**	2.90891	3.28425	0.83699	10.75110	0.01127	0.00000	2.87560	1.45144	11.49752	5.76235	6.29861	14.45795	0.71448	6.70669	14.02571	1.58914
**Ercc6l2**	0.93462	0.54016	0.02613	1.93839	0.00613	0.00717	3.59598	0.59380	0.86131	1.06173	0.79261	0.12148	1.01429	8.99068	3.84579	0.00505
**Ercc8**	5.23264	1.23871	3.34223	1.33325	0.00000	3.58464	0.00000	4.94996	0.00000	3.46202	2.09641	0.23490	3.18409	2.45351	2.46801	1.81877
**Erdr1**	1.20046	0.04667	1.01548	2.38059	0.10397	2.21713	0.36070	0.25419	4.51402	11.12799	9.45092	1.32675	3.48231	0.00000	0.00000	1.86469
**Erf**	1.43770	0.03940	0.57786	0.15751	0.86671	0.51033	0.09424	0.33355	0.91148	0.25907	2.88631	0.88749	0.07386	0.00550	0.16313	3.38982
**Erg**	0.01344	0.00793	0.01168	1.37697	0.00915	0.00664	0.00750	0.01237	1.10169	0.26297	0.01457	0.00554	0.01274	0.00258	0.17990	0.00714
**Ergic1**	1.63943	0.00382	1.87458	0.63072	1.31988	0.08583	1.82165	1.61178	1.21648	2.26177	0.56664	0.03173	2.57774	0.16011	0.20471	0.14643
**Ergic2**	6.39296	4.30356	4.72842	2.58285	2.70025	0.15669	5.39634	5.82951	0.70317	8.95856	0.96980	2.30032	2.15299	8.76757	9.89539	1.27374
**Ergic3**	14.50260	4.52220	15.33425	7.29981	2.32719	11.13128	4.80045	10.71695	6.39166	10.27690	8.69374	7.94530	18.69724	0.71675	6.41375	9.63194
**Erh**	24.13325	11.21110	24.72927	15.12687	13.87665	11.18092	8.32592	18.53548	19.38907	24.30768	22.01977	6.33703	19.98474	14.20225	12.48021	11.90794
**Eri1**	2.89170	4.66168	3.07268	7.12868	3.91388	0.00000	0.89904	4.48707	6.18851	9.12071	6.16480	13.29479	3.50774	6.66412	8.54285	2.28425
**Eri2**	1.43082	3.40143	1.33661	1.60303	4.00172	0.65329	0.05830	1.51244	1.06921	0.81691	0.88512	0.08724	0.12909	4.00341	6.06537	0.16618
**Eri3**	5.79585	4.64260	4.72081	0.14922	0.16611	4.00501	0.47907	3.79389	0.78527	3.47829	1.05582	0.02735	3.40733	0.00226	0.02859	0.05282
**Erich1**	2.35807	2.25047	5.74947	0.18594	1.14460	0.00461	3.16381	2.78842	0.71627	2.42965	1.11069	0.13294	0.53668	0.17379	1.65538	0.02421
**Erich5**	0.36686	0.15534	0.84875	1.26542	0.13788	0.09049	0.04630	0.00000	0.47783	0.08665	3.33708	0.12205	0.75146	0.37197	1.07447	0.11337
**Erlec1**	2.50897	0.00401	0.00000	0.99217	0.00394	0.00462	2.12205	0.00000	0.31832	1.42154	1.05172	0.09487	0.02121	1.24852	0.68428	0.00434
**Erlin1**	0.38385	1.06616	1.43990	3.19162	0.66380	0.00666	2.47997	5.05037	1.34511	4.04409	3.31770	7.34306	1.93961	11.66869	4.43584	2.79921
**Erlin2**	3.64123	2.55278	5.62005	0.00000	3.91883	3.73706	6.59691	3.92286	1.13322	3.50186	3.11285	1.07794	3.84632	10.14449	8.26892	0.48814
**Ermap**	0.02057	0.00791	0.00786	0.16537	0.00911	0.03849	0.01818	0.02175	0.06018	0.02933	0.01281	0.03898	0.01719	0.05509	0.10170	0.00743
**Ermard**	0.59667	0.47578	3.65105	0.35057	0.57755	0.01406	0.62243	1.55837	0.14457	0.26534	0.40423	1.27345	0.95279	0.00618	1.40826	1.18546
**Ermn**	0.02943	0.00787	0.03959	0.00574	0.00918	0.05755	0.04905	0.01889	0.04621	0.01194	0.23788	0.00652	0.03288	0.06287	0.03150	0.05759
**Ermp1**	1.18102	6.75916	11.52786	32.13809	0.00000	10.82692	8.32283	7.46195	2.58255	9.73622	7.33844	10.68560	11.04586	21.71610	18.94165	3.20675
**Ern1**	0.59811	0.00743	0.00000	2.64819	0.00722	0.00637	0.00774	0.21895	5.12165	0.22642	0.29630	0.03428	0.06024	0.01446	2.17225	0.15053
**Ero1l**	0.04247	0.19455	1.77036	3.55587	0.00972	0.00547	0.49799	0.36084	1.37447	2.55846	0.91709	0.11464	0.09694	4.05132	1.53361	1.55532
**Ero1lb**	0.00214	0.01909	0.16785	4.34325	0.01014	0.00518	0.00698	0.42814	1.34975	0.65083	0.56524	0.08389	0.00258	1.65927	3.13114	0.00620
**Erp29**	10.00464	7.39963	12.38871	4.15482	6.56934	7.74151	4.89188	10.00815	6.55304	8.59032	8.96988	0.47687	11.38264	0.00000	1.14157	10.93134
**Erp44**	5.10765	0.20510	3.54193	1.66002	6.44521	5.26119	6.89287	4.51648	0.53603	5.74289	4.60442	10.86005	4.95243	1.52465	2.37963	0.87278
**Errfi1**	0.08451	0.98771	0.15693	15.32765	0.04839	1.16915	0.00625	0.28361	12.63652	2.53011	0.86309	0.00000	0.44550	3.10504	5.14787	1.66886
**Esam**	2.01469	0.00590	0.00205	0.00319	0.73531	0.00698	0.00584	0.10212	0.48568	0.74194	1.82785	0.00000	0.08920	0.00291	0.26347	0.00522
**Esco1**	1.29088	2.27607	1.10302	24.17012	1.63962	0.32477	0.00000	1.76111	17.88291	1.39094	1.73271	12.95025	3.33155	19.17315	12.03281	0.00347
**Esco2**	3.45576	6.18240	6.48797	6.92768	1.46035	1.47887	8.95569	8.37863	21.71792	10.97593	9.59541	19.96956	3.08261	31.73943	17.28088	1.01669
**Esd**	10.13283	1.76634	3.37136	0.15766	0.64742	4.02194	4.58723	4.95129	1.49455	2.42222	2.87355	0.62856	4.13259	0.19429	2.70580	3.34114
**Esf1**	6.38275	5.01700	2.01234	6.04486	3.67916	3.61903	0.83078	4.69439	8.60455	3.63034	2.42157	12.47086	0.35951	19.19000	16.39078	0.50070
**Esp24**	0.01576	0.01753	0.00590	2.76609	0.00878	0.01825	0.01733	0.02391	8.00593	0.19126	0.00058	0.07432	0.01103	0.20580	0.58112	0.00619
**Espl1**	10.25146	0.01407	0.00000	18.74856	1.49790	3.83889	0.15222	13.14967	21.58030	27.44786	11.47618	0.67149	11.49930	0.19044	0.00000	2.11841
**Esr1**	0.01292	0.00804	0.01123	2.74890	0.00901	0.00621	0.00741	0.01316	0.49307	0.88158	0.00651	0.00342	0.01128	0.00035	0.82167	0.00694
**Esr2**	0.05656	0.00744	0.02067	1.00683	0.00822	0.00678	0.01749	0.01171	1.12152	0.01348	0.10910	0.06038	0.02445	0.15269	0.35693	0.06557
**Esrp1**	1.92104	0.00232	2.12608	6.55079	0.00000	1.31933	4.17928	1.86698	6.63240	6.99711	2.61194	1.96418	0.08667	7.96475	8.83293	0.19403
**Esrp2**	0.32103	0.87176	4.04711	0.29114	0.00883	6.17903	0.00368	7.48498	0.00857	7.95826	0.33327	1.35032	3.81148	2.61614	4.79529	1.84415
**Esrra**	5.83363	0.00000	2.29135	0.74819	0.04212	1.43274	1.42348	9.37503	3.03059	6.12513	3.30155	0.24233	2.24903	0.01965	0.36315	1.95468
**Esrrb**	19.19795	8.54005	13.67928	5.73643	6.32198	0.00000	6.17047	13.54081	10.77573	17.67601	19.31350	5.10845	4.09050	6.23331	5.41357	2.22626
**Esrrg**	0.01378	0.00763	0.01186	2.30927	0.00940	0.00632	0.00732	0.01185	0.00000	0.01914	0.01194	0.00659	0.01295	0.00599	0.42232	0.00709
**Esyt1**	0.13909	0.00605	2.67985	5.25906	0.87968	5.92778	0.38450	2.70822	7.84171	2.11933	1.21082	0.21944	1.20030	0.00432	1.08807	3.31652
**Esyt2**	2.09501	2.64328	2.79437	7.02639	1.10028	0.27518	5.56655	2.36499	6.53413	3.85064	4.22840	11.49679	1.49642	7.91878	4.47622	0.63849
**Esyt3**	1.80050	0.17029	1.09601	3.26485	1.31602	0.00639	0.13428	0.00977	0.47409	0.58482	0.15603	0.08761	0.01253	0.25440	2.17959	0.00414
**Etaa1**	1.64570	7.61918	2.55535	14.18598	3.03538	0.00710	2.98368	1.96161	17.45809	11.87328	7.62531	9.08740	2.36325	8.88479	9.84757	1.69901
**Etaa1os**	0.01597	0.00809	0.07080	0.52523	0.00913	0.00582	0.00743	0.01344	0.54927	0.01575	0.00948	0.00742	0.01179	0.01122	0.00433	0.00723
**Etd**	0.01375	0.00814	0.01230	0.40144	0.00931	0.00661	0.00752	0.01394	0.00587	0.01545	0.01399	0.00824	0.01266	0.01115	0.00578	0.00735
**Etf1**	20.05352	16.30691	21.37642	21.46591	19.14004	15.17057	15.42634	22.64421	25.78478	30.62665	14.23136	21.98052	17.19485	21.10368	26.66666	11.83751
**Etfa**	18.97341	8.33049	14.81997	5.92597	12.82781	11.52366	6.47561	13.72805	6.99986	12.69755	10.61089	7.79558	13.68226	4.56874	4.97487	7.74626
**Etfb**	25.02012	12.64355	22.53099	0.70519	9.55650	12.23899	8.35460	15.82816	0.38942	9.55298	9.08382	0.13204	14.81878	0.04177	0.34405	9.83277
**Etfdh**	1.08849	0.12472	0.26407	0.27021	3.23412	0.00453	2.78989	0.03099	0.00717	1.08674	0.05309	0.01406	0.88778	2.21499	0.83586	1.62509
**Ethe1**	1.68760	0.00298	7.03340	1.72766	1.20094	1.80810	1.73398	2.71026	0.64506	2.27396	7.36556	0.11738	5.83382	0.00000	0.00016	6.66831
**Etl4**	3.15189	0.14105	1.41344	0.38399	5.54587	2.65779	0.78592	2.60119	0.31596	2.16484	3.53772	0.02920	2.76442	5.19303	1.23457	0.32056

**Etnk1**	8.32607	0.06638	3.52321	2.80137	0.08195	5.28588	2.74459	1.37749	2.61717	17.67910	4.02445	9.63463	1.52184	10.50134	22.44218	0.37801
**Etnk2**	1.52397	0.00567	0.07151	0.00511	0.00714	0.00416	1.18806	0.00000	0.01145	0.00000	0.29859	0.00514	0.00837	0.01146	0.00385	0.01286
**Etohd2**	0.00595	3.40060	0.24318	0.00978	0.00746	0.54945	0.00685	0.06964	0.26502	0.30574	0.00300	0.49846	0.00342	0.00905	0.04843	0.00580
**Ets2**	1.06378	0.00648	0.02215	2.62583	0.00514	0.00693	0.00645	0.00698	0.00000	0.25213	0.00780	0.09417	0.01062	0.00131	1.35464	2.10440
**Etv1**	1.27686	0.00629	0.00000	0.63298	0.00737	0.01494	0.00637	0.00740	0.00000	0.00707	0.01133	0.03607	0.01153	0.01269	0.27060	0.00596
**Etv3**	0.32960	0.00632	0.00000	0.61211	0.00705	0.00602	0.04570	0.16677	0.22305	0.00000	0.29264	2.07687	0.00571	0.00584	1.63762	0.01346
**Etv4**	1.21387	0.00756	0.00231	0.00873	0.01583	0.00681	0.00652	0.00856	0.01220	0.00518	0.07121	0.00698	0.01931	0.01225	0.00958	1.46879
**Etv5**	20.59300	9.05285	3.10467	3.05642	10.79629	0.00000	7.13856	4.85685	5.58896	2.22362	0.66403	0.08504	0.00000	2.56065	1.41704	7.78680
**Etv6**	4.07372	1.33390	0.00000	0.51875	0.02994	0.21105	0.00280	1.56799	3.93072	2.76850	0.43422	2.31010	0.11532	8.77499	6.55211	0.06005
**EU599041**	0.93974	0.17859	0.56646	3.48379	0.05135	0.00447	2.17327	0.68104	0.47881	0.67237	1.66901	0.66908	0.45546	1.05560	2.19901	0.01929
**Eva1a**	3.40550	3.29654	3.64600	3.69205	9.26696	5.11928	6.66806	7.57989	2.72578	6.79737	6.77794	0.69013	3.26702	2.24016	0.53301	4.64658
**Eva1b**	0.01308	0.00799	0.02953	0.13437	0.00975	0.00721	0.00748	0.01272	0.00982	0.43154	0.01528	0.02614	0.15430	0.01082	0.00659	0.00789
**Evi5**	4.54340	7.71772	7.15410	15.72406	5.14060	6.14396	5.96063	6.47014	10.02815	10.71852	6.72157	8.72044	4.18769	23.63759	23.91803	2.45134
**Evi5l**	0.00446	2.01672	0.00725	0.51641	0.00846	0.00688	0.00612	0.01421	0.11031	0.00000	0.01216	0.04426	1.24582	0.01865	0.06214	0.00605
**Evl**	0.03911	0.00755	0.29156	0.41749	0.00785	0.00915	0.35572	0.01025	0.99325	0.47873	0.02179	0.04860	0.32929	0.01248	0.86351	0.12608
**Evpl**	0.01937	0.00737	0.43921	0.13669	0.00756	0.00896	0.00633	0.70519	0.00682	0.00116	0.00817	0.00517	1.23677	0.01090	0.02972	0.00642
**Evx1**	0.01324	0.00770	0.03107	0.69435	0.00893	0.00588	0.00729	0.00985	0.00000	1.45562	0.02180	0.00323	0.01033	0.00435	0.17091	0.00708
**Evx1os**	0.12854	0.00833	0.00780	0.09528	0.01253	0.00678	0.00741	0.01292	0.01043	0.01013	0.01330	0.00821	0.01672	0.01223	0.00886	0.41947
**Ewsr1**	15.18350	5.62322	9.22038	7.09521	8.23063	11.67635	9.56708	23.44245	8.72196	10.00088	14.42374	4.96092	13.39236	8.13133	2.95076	11.39390
**Exd1**	0.00384	0.00698	0.00000	0.61647	0.00893	0.00583	0.00485	1.65143	4.41555	2.27887	6.22876	0.20957	0.55156	0.08808	4.57514	0.00583
**Exd2**	5.35811	2.92055	0.16829	9.12262	1.31258	0.00000	3.95351	5.45510	7.27874	6.54124	3.73556	9.43777	0.74487	0.14217	4.32929	2.69387
**Exo1**	7.46244	6.48260	2.50569	5.15443	4.69294	6.45314	5.36807	4.74332	7.48469	15.52898	7.03901	10.18851	3.62796	1.41042	3.92981	0.84988
**Exo5**	4.25708	0.67284	1.42654	0.85900	0.01556	1.12585	0.00279	4.37653	0.43068	5.49961	1.88222	0.11291	0.80324	1.07778	1.77119	2.13459
**Exoc1**	3.61836	6.72510	3.12939	3.70402	0.49458	3.21398	2.11931	1.08832	3.07099	5.59774	2.53379	0.67984	2.77613	6.00711	3.98507	0.16356
**Exoc2**	2.45382	1.46582	2.31290	2.11101	5.42989	0.01103	4.09151	0.62705	0.63129	2.92003	0.84897	6.25802	1.61217	2.15312	2.83046	1.59933
**Exoc3**	2.44596	4.21269	5.60239	1.36414	7.06310	3.86835	2.75710	5.32091	3.77226	5.81830	9.82215	8.07235	3.59374	5.66317	6.41210	0.00000
**Exoc3l**	0.01357	0.00804	0.01185	0.00871	0.00909	0.00654	0.00742	0.01118	0.01221	0.46594	0.01122	0.02686	0.01103	0.01149	0.00879	0.00712
**Exoc3l4**	0.00849	0.00851	0.00003	0.00488	0.00949	0.01763	0.00799	0.51651	0.01031	0.33842	0.92032	0.00442	0.00000	0.01119	0.00186	0.00727
**Exoc4**	1.40689	0.24754	0.11271	0.00551	0.00548	0.00747	0.05859	0.68677	0.01011	0.19293	0.04884	0.00000	0.01662	1.79280	0.42022	0.33092
**Exoc5**	4.44171	3.85114	4.68720	4.02438	2.70829	5.84650	4.50787	5.35921	6.02513	6.57947	2.00177	12.77158	3.35978	13.74778	4.45136	0.80262
**Exoc6**	1.31383	0.00526	0.61516	0.14214	0.84847	1.54105	0.00448	1.89036	0.79118	2.53989	4.09892	0.19810	0.34092	0.23354	1.39557	0.23457
**Exoc6b**	0.44745	2.92771	0.12950	0.00000	0.01740	0.00884	0.00637	0.52811	0.01104	0.61817	0.02125	0.02539	0.00527	0.01333	0.47355	0.01356
**Exoc7**	3.85039	2.26345	1.45973	2.26346	0.21938	3.93015	0.00218	0.13059	0.59996	2.16774	2.41877	0.06873	0.19945	0.00000	0.57566	2.12522
**Exoc8**	2.03035	1.87268	4.24462	3.15810	0.02398	0.02258	2.26461	4.04241	11.96080	2.60336	1.80515	0.12259	1.22299	1.32929	3.82426	3.26978
**Exog**	1.27513	4.04764	0.68648	2.94413	0.03841	2.01449	0.01500	0.00570	0.82517	0.34876	0.60051	0.11562	0.03694	0.04786	2.05688	0.01011
**Exosc1**	12.91623	10.99906	15.13226	5.22571	0.48268	4.19493	2.40560	7.37757	4.03345	12.91655	6.89397	7.27792	8.77150	1.65072	9.03104	3.96134
**Exosc10**	11.06000	0.25675	8.58828	10.89903	1.78497	1.96101	4.03381	9.43257	10.30405	16.07495	10.42794	7.21512	0.15515	0.68142	7.97451	3.31906
**Exosc2**	11.72146	5.79508	5.56453	8.86030	7.59372	3.84532	5.07698	7.69891	15.23709	1.63958	12.24990	0.85609	4.40680	7.80394	6.51484	1.36373
**Exosc3**	2.35228	1.71755	1.20353	6.33536	8.39383	0.03510	0.35897	0.19290	11.47149	14.93787	7.96958	0.25452	3.77032	0.67237	2.44040	4.73540
**Exosc4**	9.22865	0.03696	0.00000	0.69424	7.91320	2.32272	0.08716	6.45789	2.90620	8.44087	0.36377	0.36127	13.31812	0.09346	0.33716	2.83919
**Exosc5**	8.51185	0.18117	0.56451	0.52331	4.98786	1.07636	6.04421	17.29642	0.59252	17.31151	0.83414	5.18031	11.81471	0.03808	0.00000	1.91349
**Exosc6**	0.75443	0.06174	0.20458	13.97255	0.42405	1.20492	0.07697	0.18051	21.21515	4.08240	6.42279	0.17042	0.27352	0.36018	0.32017	0.47584
**Exosc7**	14.78484	8.71902	16.23466	1.16608	6.55703	4.62600	5.49911	18.32903	2.35421	15.49706	9.71168	3.26651	10.73916	5.10528	7.17784	4.87900
**Exosc8**	19.45314	12.48062	13.59650	10.83432	10.02226	4.21181	8.96443	11.15491	6.54205	14.83170	12.53035	11.15125	16.65099	12.19379	6.73259	7.49911
**Exosc9**	5.83786	3.35372	11.30246	0.04006	9.06445	3.00186	8.99402	10.72783	1.64085	7.10121	4.91546	2.02412	5.63365	6.36904	6.78204	3.41083
**Exph5**	0.00568	0.00835	0.63129	0.00525	0.00915	0.00955	0.00727	1.25717	0.01008	0.30200	1.45753	0.00000	0.47811	0.00531	0.01466	0.01407
**Ext1**	2.11647	0.55715	0.28249	5.36484	0.00414	0.00310	1.09081	1.73381	0.88993	4.05910	0.49250	2.60756	0.00251	0.00000	4.51066	0.00361
**Ext2**	7.98966	0.27457	0.00000	0.45772	0.42561	3.20797	2.36325	4.66527	0.05597	0.48117	0.89128	0.01424	1.09292	0.01109	0.06376	0.00481
**Extl1**	0.08417	0.00774	2.29665	1.95474	0.02561	0.23961	0.02717	0.07704	1.79302	0.17601	0.00000	0.03826	0.00608	0.00067	0.15307	0.00602
**Extl2**	0.01793	0.00800	0.01095	0.01059	0.00898	0.01312	0.00750	0.01384	0.01234	0.01853	0.01307	0.01821	0.01270	0.01212	0.01052	0.00745
**Extl3**	4.00152	0.00386	0.94688	0.36886	0.02612	3.01363	0.02591	0.67537	0.77698	1.15512	0.12057	0.01083	1.29490	0.00424	0.63322	2.78688
**Eya1**	0.15873	0.00679	0.00333	1.46849	0.64927	0.00711	0.04778	0.00751	0.88170	0.00000	0.03946	0.02425	0.01021	0.00843	0.44197	0.00670
**Eya2**	0.01278	0.00825	0.01419	0.15427	0.01012	0.00710	0.00773	0.01643	0.00953	0.01117	0.01624	0.00795	0.16358	0.01171	0.00785	0.00763
**Eya3**	4.32979	4.98803	6.85359	1.82283	0.07853	0.10717	5.83441	7.72697	4.80010	1.95052	4.15184	6.16119	1.54222	10.06842	6.22718	0.00299
**Ezh1**	2.72977	1.67347	0.92572	9.70560	1.14955	0.41120	8.44219	1.18987	6.84496	8.86747	2.01147	0.34319	0.10908	2.77384	6.04499	3.70488
**Ezh2**	13.84431	18.96398	17.60416	15.69917	10.28348	7.01450	12.97703	18.19321	18.21996	21.44413	10.76382	19.66948	9.49353	24.91474	17.93561	4.94543
**Ezr**	18.52806	7.93000	5.65329	0.35012	12.59826	10.65716	10.48215	1.61768	0.18980	6.71583	0.00000	0.20388	8.23113	0.00000	0.00000	13.06797
**F10**	0.03611	0.01883	0.03342	0.38335	0.03513	0.00642	0.04874	0.02764	0.06007	0.02150	0.13365	0.00582	0.15037	0.35719	0.02739	0.00722
**F11r**	9.20203	7.80055	15.04752	3.64546	3.11241	13.20086	9.95680	19.88525	5.92177	15.56728	13.31893	2.56949	22.57490	27.35800	14.33907	9.26734
**F12**	0.01201	0.00839	0.01461	0.21146	0.01004	0.00708	0.00766	0.01369	0.00561	0.58240	0.01261	0.00632	0.25300	0.01056	0.00583	0.00740
**F13a1**	0.01376	0.00772	0.01132	0.47894	0.00939	0.00644	0.00736	0.01073	0.16115	0.00303	0.03225	0.00623	0.01232	0.01021	0.60692	0.00726
**F2**	0.00705	0.00731	0.17442	0.91389	0.00928	0.00879	0.00683	1.47039	2.08210	0.45084	0.73161	0.01534	0.00000	0.00436	0.13049	0.00705

**F2r**	1.38190	0.00532	0.00000	1.26723	0.00506	0.00745	1.22330	0.00671	1.44157	0.00920	0.00000	0.09580	0.00873	0.05306	0.05708	0.96407
**F2rl1**	1.48624	1.03502	0.02431	0.00970	0.45903	0.04088	0.02694	0.01277	0.01227	0.00148	0.01311	0.00644	0.38826	0.01390	0.00000	0.82986
**F3**	0.09370	0.00802	0.00578	0.01022	0.01461	0.00725	0.00729	0.03210	0.01144	0.01858	0.01040	0.10029	0.01170	0.01536	0.21669	0.00745
**F8**	0.01180	0.00828	0.11899	0.01020	0.00911	0.00887	0.00761	0.01894	0.01247	0.00801	0.01064	0.00665	0.01085	0.30746	0.05169	0.00738
**F8a**	0.70043	0.00747	0.42160	0.29948	0.01299	0.00493	0.00667	0.00318	0.38839	1.15483	0.17633	0.01334	0.02774	0.00999	0.00000	0.01355
**Fa2h**	0.01118	0.00833	0.00952	8.69436	0.00914	0.00540	0.00768	0.04928	6.68881	0.00000	0.03925	0.00590	0.00984	0.02879	0.08893	0.00534
**Faah**	1.06208	0.54599	2.11245	0.02593	0.01040	6.69476	0.00477	1.87938	0.81032	0.00000	0.00000	0.00000	1.71991	0.04171	0.01046	0.43078
**Faap100**	1.77828	0.00547	0.02816	0.07959	0.01371	0.00617	0.43521	0.41070	0.04772	2.55810	0.09935	0.01853	0.96153	0.01401	0.00000	1.65843
**Faap20**	1.70285	0.04309	0.64282	1.72559	4.07196	5.69117	0.85146	3.78089	0.84009	1.53815	3.04629	0.04991	3.92694	0.10453	0.00000	0.00847
**Faap24**	4.15762	0.90800	0.90743	6.25294	2.08126	0.00800	3.01918	3.14019	4.80389	10.60482	1.36604	0.23274	0.32918	0.00926	0.68330	4.78391
**Fabp3**	8.79590	7.22354	19.88338	0.06132	11.33711	18.23637	11.31470	4.92598	2.80739	1.88544	17.66010	1.49494	27.40737	0.00000	0.00000	14.76446
**Fabp4**	0.15585	0.00679	0.18359	2.95253	0.00713	0.00857	0.60142	0.00000	0.94331	2.80580	2.29483	0.37321	0.00000	4.79072	2.52552	0.00594
**Fabp5**	29.22517	14.91194	15.51012	3.39941	31.69057	2.82273	10.47511	4.25932	0.02216	0.58317	5.71782	0.02094	13.45074	0.00001	1.29349	14.41137
**Fabp6**	0.01191	0.00825	0.01174	1.09365	0.00912	0.00642	0.00719	0.01140	0.05313	0.00642	0.01266	0.00800	0.39096	0.03384	0.00161	0.00688
**Fabp9**	0.00465	1.01856	0.00739	0.00242	0.00810	0.00656	0.00686	0.00909	0.20537	0.00391	0.00846	0.00504	0.01144	0.58385	0.00000	0.01435
**Fadd**	0.96104	1.29387	0.93454	2.28070	0.00680	3.37170	0.06489	0.85240	2.20569	4.13275	0.57168	0.31362	2.45559	0.39071	1.38232	1.88859
**Fads1**	2.57337	2.36432	0.58914	0.37568	0.01969	0.00434	1.62299	0.00000	0.39313	3.28683	1.97241	0.04759	0.02291	0.00390	2.89274	0.00334
**Fads2**	0.01352	0.00827	0.01145	0.01046	0.00935	0.00670	0.00760	0.01565	0.01249	0.01272	0.01253	0.00766	0.01269	0.11374	0.00631	0.00744
**Fads3**	0.51599	0.00761	0.35931	0.00359	0.00677	0.00574	0.00667	0.00000	0.14773	0.01778	1.14729	0.38227	0.77133	0.09795	0.32455	0.00647
**Faf1**	8.76296	7.84562	12.10200	5.96296	8.14002	10.65787	6.52935	12.19442	13.71103	14.08590	9.68143	12.49454	9.40764	14.72148	12.42018	5.28914
**Faf2**	4.37279	4.89620	7.14544	6.92244	3.09922	1.34614	0.17996	3.42625	7.46799	4.39600	3.04045	7.26695	2.01101	6.30429	10.16661	1.47747
**Fah**	0.00674	0.00849	0.00616	0.00417	0.00901	0.00714	0.00827	0.64839	0.03391	2.26898	0.12001	0.00000	0.00526	0.23911	0.35314	0.00731
**Fahd1**	0.14949	0.01707	0.00000	0.36390	4.40880	2.98049	0.00650	0.00708	1.22261	0.00000	0.00000	0.04281	0.67454	0.01372	0.00167	0.01293
**Fahd2a**	0.26012	0.16063	0.03431	0.37872	0.00674	0.00571	0.10030	0.65402	0.05722	0.42184	0.77964	0.02043	1.24182	0.00451	0.18513	2.30204
**Faim**	0.92408	0.11075	0.58297	0.66406	0.11491	0.00464	0.07398	0.40576	1.33658	1.49797	1.34432	1.71396	1.13655	0.09991	0.38165	0.53611
**Faiml**	0.95114	1.03293	1.09686	0.14725	0.16031	0.00590	0.36030	0.93861	1.59407	0.52198	2.11065	0.12346	0.03358	0.88293	1.66235	0.04151
**Fam101a**	0.01298	0.00786	0.01147	1.08063	0.00944	0.00646	0.00729	0.03272	0.00000	0.00774	0.01333	0.00758	0.01136	0.00726	0.25867	0.00718
**Fam101b**	3.43234	0.93455	10.02936	0.25933	0.03259	7.05047	3.74624	13.19664	0.38896	14.36249	14.63236	12.83038	8.98238	3.89493	5.95895	6.37274
**Fam102a**	4.81658	4.36878	4.91584	4.32614	5.82155	3.68157	2.93821	9.11057	4.81803	8.56525	3.62120	0.42403	4.80627	4.36350	3.05192	7.09991
**Fam102b**	0.01360	0.00773	0.01059	1.40448	0.00944	0.00624	0.00727	0.01073	0.59397	0.00984	0.00643	0.02431	0.01314	0.00498	1.16413	0.00713
**Fam103a1**	6.30206	10.80433	11.85857	7.12954	2.39260	6.14037	6.40669	12.82975	16.37189	20.56132	11.77074	17.04543	4.37923	21.06841	16.32893	2.48142
**Fam104a**	2.33647	0.03419	2.62176	11.05192	4.10647	2.77474	1.37755	1.93621	11.81460	10.36096	7.42840	1.38139	1.75507	1.18546	0.65434	1.93095
**Fam105a**	0.01270	0.00752	0.00535	0.01088	0.00919	0.00682	0.00713	0.00424	2.33046	0.00000	0.31134	0.00000	0.00905	0.72772	0.00000	0.00713
**Fam107a**	0.01158	0.00809	0.46861	0.15947	0.00818	0.00564	0.00746	0.01537	0.50873	1.15721	0.00798	0.00450	0.00925	0.01161	0.00000	0.00719
**Fam107b**	0.48092	3.29077	0.00000	4.99650	0.07347	7.26824	0.03465	0.77135	2.55423	0.39100	0.41933	0.16110	4.69361	0.00000	1.25581	2.79570
**Fam109a**	1.41340	0.01516	0.13790	11.38575	2.57142	4.86741	1.40107	2.59115	18.20096	6.13704	1.58733	0.13388	2.95085	0.01810	0.33342	0.19325
**Fam110a**	0.01027	0.00800	0.01188	0.20064	0.00918	0.00648	0.00695	0.01054	0.25591	0.02960	0.01051	0.00692	0.77969	0.01135	0.00562	0.00713
**Fam110b**	0.84545	0.00769	0.00335	0.00642	0.01663	0.00682	0.00618	0.01318	0.09413	0.00438	0.01147	0.01702	0.89259	0.01243	0.01411	0.36566
**Fam110c**	0.01377	0.00823	0.01190	0.99144	0.00930	0.00658	0.00750	0.01293	0.00000	0.01330	0.05290	0.00820	0.01183	0.01063	0.00293	0.00722
**Fam111a**	0.01213	0.00794	0.73910	0.22655	0.00778	0.00971	0.00732	0.01129	0.00525	0.36896	0.01009	0.00762	0.00917	0.01131	0.00000	0.00707
**Fam114a1**	0.01306	0.00786	0.01158	0.00671	0.00890	0.00655	0.00729	0.01010	0.01166	1.35689	0.01058	0.03394	0.01073	0.01211	0.00650	0.00731
**Fam114a2**	7.49271	4.04451	3.84186	1.55722	2.70565	2.64972	6.88427	5.27311	0.64501	4.39073	3.80325	5.21546	3.02494	1.84286	3.95221	1.37488
**Fam117a**	0.00000	0.01966	3.43975	8.94018	0.01120	1.96700	0.00368	0.82711	4.91603	1.91870	0.79724	0.06291	6.43538	0.07765	1.20736	1.30145
**Fam117b**	2.77706	1.83646	3.63182	9.51408	5.57402	0.00754	2.70910	3.92356	14.75431	1.80398	2.90624	1.85794	2.13621	5.10488	5.79403	0.92523
**Fam118a**	1.40214	4.37512	4.60353	11.02418	4.09053	2.09279	1.27398	8.74511	17.16640	13.83302	9.57190	1.49185	4.02155	18.77778	12.39508	2.37014
**Fam118b**	7.36092	1.30269	5.27341	1.73549	5.53716	9.63204	0.47627	4.85003	7.85058	13.04434	4.27411	9.84656	2.87193	5.94441	9.26992	2.87152
**Fam120a**	5.59569	1.95181	6.51445	4.16530	8.86907	5.98581	8.47300	11.07236	4.15764	10.69754	8.82313	18.61720	6.61361	21.12798	12.11748	2.67484
**Fam120aos**	0.01338	0.00793	0.41762	0.53336	0.00840	0.00560	0.17606	0.00973	0.13271	0.60392	0.01077	0.01507	0.02318	0.05549	0.09866	0.00746
**Fam120b**	0.79681	0.00624	0.00000	0.49604	0.01805	0.00591	2.71547	0.00000	1.32498	0.79428	1.11670	0.03865	1.73857	0.18977	1.68265	0.00573
**Fam120c**	0.01783	0.00786	0.01081	0.60146	0.01023	0.00650	0.00738	0.01062	0.15056	0.65599	0.00829	0.01550	0.01119	0.01128	0.46686	0.00712
**Fam122a**	2.65547	0.00480	0.23921	2.83850	0.00409	0.00341	0.03424	0.38748	4.92621	1.57093	0.23398	0.03525	0.65033	0.15345	0.94010	0.01016
**Fam122b**	0.00446	0.00603	1.02954	0.70303	1.22528	0.37226	0.57728	0.00000	0.43772	1.10855	2.09951	4.15704	0.68582	3.88069	3.73263	0.01266
**Fam124a**	0.03153	0.01861	0.02736	0.06316	0.01030	0.00595	0.00722	0.01563	0.05964	0.71027	0.01192	0.02436	0.01758	0.06469	0.02044	0.00742
**Fam124b**	0.01345	0.00661	0.76702	0.18585	0.00801	0.00562	1.52131	0.00818	0.00821	0.34994	0.01287	0.00477	0.00874	0.01302	0.00000	0.00735
**Fam126a**	0.59424	0.00745	0.12296	1.50954	0.01648	0.00808	1.77139	0.41378	1.97388	0.00000	0.79119	0.59515	0.00638	0.02205	1.31147	0.00606
**Fam126b**	0.62341	0.00186	2.17880	10.36501	0.00258	0.00342	2.62869	1.03929	6.96064	1.73905	1.61865	9.86015	0.15018	11.59303	5.59413	1.02134
**Fam129a**	4.33995	8.17203	4.89903	0.00000	0.04679	10.53376	4.43724	4.64038	0.23371	0.95719	1.47378	1.69126	5.70938	1.72454	0.00000	0.45114
**Fam129b**	0.00000	0.00633	2.53860	0.17378	0.01429	0.01921	0.00467	3.67521	0.00548	0.34328	3.76869	0.06085	1.02820	0.00057	0.00000	4.68004
**Fam129c**	0.01332	0.00757	0.01102	0.74701	0.00929	0.00633	0.00723	0.01027	0.90707	0.58079	0.00146	0.03150	0.01190	0.00632	0.40062	0.00728
**Fam131a**	0.17794	0.00767	0.00515	0.99012	0.00753	0.00670	0.00714	0.00590	2.34010	0.00807	0.21588	0.00000	0.01012	0.03538	0.33244	0.00680
**Fam131c**	0.00855	0.00777	0.00454	0.00948	0.00945	0.00759	0.00733	0.63606	0.88043	0.53395	0.57223	0.00000	0.00322	0.01066	0.00066	0.00714
**Fam132a**	4.63656	0.04954	1.59924	4.43340	0.00000	5.22373	2.52918	5.39670	3.12741	1.52758	4.31761	1.55593	2.33187	0.19295	0.00000	3.25125

**Fam132b**	0.09588	0.00678	0.92515	0.28371	0.00528	0.30529	0.00543	2.66721	0.01127	0.00000	0.96645	0.00000	1.86045	0.40518	0.00000	2.15830
**Fam133b**	5.86353	7.91152	5.06059	4.51233	5.18457	0.00570	0.12354	5.27980	5.31983	1.49127	1.03440	0.80051	0.96503	0.09515	3.76917	1.23546
**Fam134a**	6.84801	2.97043	5.35236	6.92545	0.04528	4.46227	0.30085	11.04492	6.74809	12.97189	9.91491	1.63449	5.50530	2.06927	13.80464	1.82680
**Fam134b**	0.01471	0.00812	0.02051	0.01022	0.00930	0.00629	0.00748	0.01350	0.01219	0.01368	0.01328	0.00831	0.01249	0.00996	0.04065	0.00739
**Fam134c**	0.81547	2.74194	0.98769	2.54104	0.02870	0.15774	0.28123	3.99006	4.61632	3.22639	4.61596	0.11030	1.68876	4.80230	7.95725	1.13542
**Fam135a**	0.00000	0.24549	0.35475	1.94941	0.12081	0.01043	0.00615	0.10304	1.36439	1.16987	0.36523	6.90489	0.36766	1.75106	4.84854	0.00611
**Fam136a**	11.83453	8.44826	12.01766	0.00000	8.99567	9.08332	5.49978	8.01608	0.00000	2.22030	1.92747	0.14873	10.50827	3.36689	0.00000	6.06305
**Fam13b**	1.09077	4.08003	0.45044	1.13015	1.96959	0.00750	0.02426	1.94723	1.42468	1.81352	0.00000	0.09375	1.39180	5.91737	1.95147	0.01075
**Fam13c**	0.01379	0.00765	0.01086	0.05464	0.00940	0.00649	0.00734	0.01119	0.02105	0.00483	0.01129	0.03625	0.01289	0.01113	0.66800	0.00736
**Fam149b**	3.65658	0.16780	1.42493	3.15725	0.25665	0.02378	1.55093	1.32186	1.04573	0.00000	0.00000	0.12778	0.28767	0.77711	1.24346	0.04408
**Fam151a**	1.44470	0.00380	1.90797	0.00250	1.00255	0.49969	1.65071	4.00227	0.00765	1.38971	0.00000	0.00000	3.58019	0.14981	0.20370	1.99438
**Fam159a**	0.07473	0.00805	0.05288	0.00940	0.00782	0.00580	0.00732	0.02092	0.98630	0.20529	0.21916	0.00515	0.62227	0.01096	0.08212	0.00677
**Fam159b**	2.54151	0.00545	0.28792	0.20501	0.02718	0.00296	0.00461	0.00000	0.09854	0.72069	2.25186	0.20129	0.15717	0.45683	0.18441	0.45861
**Fam160a1**	0.00046	0.23861	1.26839	5.45167	3.60811	0.00875	0.00702	0.10560	0.95211	0.16330	0.29080	0.02210	1.04747	0.00000	1.08409	0.00521
**Fam160a2**	2.65581	2.37921	2.05820	1.18461	0.28977	0.00395	0.67467	0.25735	0.65973	1.54472	0.59132	0.08649	2.78966	0.01996	0.06131	3.24679
**Fam160b1**	0.28874	1.16342	1.60527	1.08757	0.03795	0.00668	0.33802	0.41589	0.31524	0.12552	0.30967	6.27851	0.61444	1.56304	0.27731	0.01243
**Fam160b2**	0.01689	2.97215	0.82595	2.14498	5.67705	3.97741	0.94659	9.25988	1.34715	4.84363	7.03727	0.12258	9.54681	0.31964	2.38121	2.63110
**Fam161a**	0.01901	2.85553	2.05994	0.39935	0.01562	0.24883	0.01589	0.00000	5.73565	1.79352	0.49332	0.22274	0.03325	0.00001	0.06612	0.01866
**Fam161b**	0.11347	0.01737	0.19390	1.53185	0.00735	0.01675	0.01627	0.52846	4.58932	4.05786	3.59154	2.57106	1.14611	0.50550	1.79357	0.00528
**Fam162a**	2.37818	0.00333	0.14105	0.22505	4.40477	0.15517	0.04012	0.02214	0.00201	0.55851	0.01308	0.04672	0.06234	0.18618	0.00000	1.77747
**Fam163a**	0.01366	0.00807	0.01211	0.20502	0.00928	0.00660	0.00753	0.01391	0.58364	0.01219	0.01021	0.00710	0.01281	0.01108	0.00976	0.00735
**Fam166a**	0.01115	0.00848	0.01039	0.05536	0.00949	0.00674	0.00816	0.10850	0.19130	0.01077	0.00997	0.00814	0.00857	0.00876	0.05862	0.00735
**Fam167a**	0.01260	0.00754	0.00760	2.46832	0.00941	0.00636	0.00711	0.00876	1.23070	0.01499	0.01606	0.00153	0.01268	1.04110	0.11014	0.00676
**Fam167b**	0.01252	0.00774	0.00972	0.29839	0.00922	0.00675	0.00714	0.14565	0.11217	0.26337	0.01297	0.00621	0.00813	0.01042	0.00363	0.00773
**Fam168a**	9.39740	1.24005	5.57577	4.79725	8.83922	0.00000	1.35889	3.15434	4.62238	4.40093	5.05761	3.92362	1.35097	9.63171	4.34296	4.70015
**Fam168b**	6.38183	1.72686	7.64026	9.81599	7.12448	4.48428	3.15259	2.76614	6.04701	6.75860	5.41305	10.44690	2.17240	0.44950	6.38391	1.95960
**Fam169a**	0.03842	0.01758	0.00000	1.37736	0.01888	3.55813	1.96656	0.11167	3.51071	1.13540	2.51580	0.74188	0.00000	3.38861	3.11403	0.00504
**Fam169b**	1.99846	0.22909	0.23179	8.33984	0.10696	0.18253	0.52166	1.12519	12.18795	7.22525	1.53828	0.62585	0.34859	2.65309	5.93888	1.10804
**Fam171a1**	0.01013	0.00748	0.01654	0.00474	0.00937	0.00625	1.60105	0.02362	0.18711	0.68971	0.00936	0.00463	0.00958	0.11357	0.00000	0.00718
**Fam171a2**	0.01388	0.00812	0.00865	0.00920	0.00908	0.00691	0.00755	0.00524	0.11378	0.00634	0.43662	0.02841	0.00809	0.00874	0.01093	0.00720
**Fam172a**	1.06815	0.93548	0.22092	5.53475	3.75577	0.00713	0.00566	1.88125	2.22467	0.17137	0.00038	0.00000	0.00380	1.62132	0.40221	0.00509
**Fam173a**	1.92161	0.01519	0.71748	0.17459	0.29749	2.53122	0.11624	4.34513	0.00590	2.40103	1.49532	0.01419	2.30254	0.00561	0.03035	2.20237
**Fam173b**	0.09296	0.97724	3.88346	0.18717	0.11160	2.25588	0.22207	3.67184	0.00753	0.72445	1.15344	0.25609	3.05556	0.01452	0.19554	0.09645
**Fam174a**	0.05838	0.01950	0.19638	0.33488	0.01429	0.00618	0.00732	0.02048	0.00436	0.00001	0.00745	0.06348	0.07581	2.12133	0.00676	0.00770
**Fam174b**	0.02154	0.00724	0.00618	0.47179	0.00851	0.00710	0.00704	0.85533	0.01363	0.02685	0.00684	0.00525	0.00567	0.01465	0.10179	0.00749
**Fam175a**	5.43913	2.96672	0.97347	1.91139	3.86214	0.00000	3.86995	2.58558	4.30034	5.42205	2.34229	0.16771	0.46750	1.98120	3.79265	0.00585
**Fam175b**	1.95513	8.59358	3.62404	5.84047	5.73695	1.81016	2.08638	4.40830	3.25047	6.97496	3.33530	4.68514	0.76196	9.51092	7.14691	1.99435
**Fam178a**	1.42094	2.14603	3.25523	8.06108	0.04923	0.81336	6.80664	0.05874	8.12366	4.46976	2.13793	1.69400	1.75640	5.25770	10.57514	2.86138
**Fam178b**	0.44297	0.00603	0.85508	3.28584	0.01386	0.00489	1.79979	0.00000	2.80350	0.75458	0.69881	0.14529	2.05056	0.00000	0.27937	0.00481
**Fam179b**	0.07062	2.61977	0.10788	8.41563	0.07402	0.00373	2.25371	1.70609	6.14828	2.11654	0.13759	8.78665	0.78416	8.29061	11.97908	0.13129
**Fam183b**	0.01298	0.00810	0.01388	0.00882	0.00990	0.00718	0.00765	0.01392	0.01204	0.41785	0.01418	0.00687	0.08761	0.01152	0.00994	0.00791
**Fam184a**	0.01086	0.00846	0.00769	2.94928	0.00945	0.00624	0.00806	0.13477	0.27811	0.11032	0.01239	0.00373	0.00830	0.00163	1.83769	0.00717
**Fam184b**	0.01782	0.00790	0.01205	0.01025	0.00897	0.00667	0.00749	0.01392	0.01247	0.02408	0.01318	0.00757	0.01287	0.00863	0.03968	0.00753
**Fam185a**	0.60444	2.49938	0.35103	1.97286	2.29816	0.39170	4.07096	0.00278	0.90364	0.00703	0.25980	0.01632	0.96278	0.00000	0.00000	0.00443
**Fam187b**	0.00776	0.00788	0.01188	0.27276	0.00766	0.00648	0.00733	0.01219	0.00482	0.11494	0.02469	0.00715	0.00840	0.01036	0.00672	1.34260
**Fam188a**	0.47613	1.33577	0.00000	8.02085	6.67177	4.85141	0.97548	0.85504	0.71204	3.59480	1.65673	0.07209	1.08246	6.91254	1.30627	0.86466
**Fam188b**	0.01375	0.00816	0.01232	0.11771	0.00927	0.00660	0.00755	0.01370	0.00993	0.03860	0.01301	0.00804	0.01222	0.01115	0.00860	0.00725
**Fam189a2**	8.63930	0.00000	0.00459	0.45883	2.06885	0.00974	0.00000	0.04555	0.00000	1.46806	0.53344	0.04965	0.02659	0.16537	0.09737	3.13441
**Fam189b**	0.73972	0.00686	0.87196	3.01290	0.01275	0.00447	0.00629	0.03374	2.30163	0.99724	0.49274	0.00713	0.22232	0.00000	0.07057	0.98980
**Fam192a**	8.85975	5.03721	7.80650	9.66907	11.20685	7.76618	6.06817	9.64214	10.14118	12.58336	2.52019	1.99257	7.28786	2.37620	6.68785	5.13804
**Fam193a**	2.05574	4.39228	0.71988	3.99238	0.00764	1.63854	3.00087	0.59370	1.71768	1.70821	0.01382	0.03439	1.14191	0.01564	2.36075	0.31702
**Fam193b**	3.10819	0.00269	0.51835	0.00507	2.52688	3.09728	1.41379	0.40618	0.02261	4.60080	0.12501	0.25130	2.33926	0.01160	0.01724	0.68984
**Fam195a**	2.36186	0.01214	1.31773	5.19511	1.24538	0.03618	0.48380	1.68275	2.09216	17.04657	15.50368	0.22414	12.33376	0.01278	0.19362	0.02503
**Fam195b**	1.36160	0.55846	1.71627	0.72344	0.00331	0.36156	0.56445	0.77981	0.86862	1.79824	0.47094	0.00005	0.94483	0.00190	0.26188	0.03358
**Fam196a**	0.01323	0.00752	0.01010	0.05499	0.00939	0.00661	0.26573	0.01095	0.00887	0.00779	0.01177	0.00674	0.01170	0.00609	0.28463	0.00732
**Fam198b**	0.04131	0.02903	1.49503	0.08636	0.14247	1.37021	0.03941	0.02762	0.11603	0.54129	0.27243	0.05337	0.79845	0.17324	0.00319	1.73404
**Fam199x**	0.00904	0.00735	0.00280	16.79644	0.00948	0.00444	0.66080	0.00415	2.48801	0.00000	0.12906	0.01176	0.45740	0.06960	4.23263	0.00395
**Fam19a4**	0.01365	0.00784	0.01177	0.00313	0.00937	0.00645	0.00741	0.01182	0.74249	0.00417	0.00676	0.00571	0.01276	0.00633	0.47099	0.00726
**Fam19a5**	0.00000	0.00648	0.00436	0.28600	3.47372	0.00824	0.01995	0.64249	0.03349	0.00438	0.00963	0.00437	0.03027	0.01412	0.00000	0.00741
**Fam204a**	4.03866	1.39188	0.78735	1.53064	2.06586	0.05968	0.02359	1.22700	3.03720	1.38588	1.49896	6.50572	0.10581	3.68097	1.62247	0.10665
**Fam206a**	7.28636	0.48857	4.83349	0.50129	0.07546	0.07976	0.85598	2.57657	0.08274	4.17323	1.45631	2.81491	1.40450	0.07804	2.41819	3.42878
**Fam207a**	3.29549	0.25521	0.79770	4.88594	3.00103	1.84128	0.90985	3.44153	9.69795	10.19534	4.13759	0.53667	3.52776	1.24979	2.15732	3.07328

**Fam208a**	1.43418	4.34118	2.61477	6.91393	3.49928	0.59867	1.80525	0.02451	2.52049	6.53769	3.58064	10.02845	2.18807	10.11789	6.13974	2.00263
**Fam208b**	2.54147	0.02351	1.28390	2.11168	1.32314	2.65503	1.18505	3.36573	7.99731	5.58822	0.66929	3.19139	2.99570	13.15688	9.95749	0.77116
**Fam209**	0.01339	0.00787	0.01073	1.42208	0.00927	0.00648	0.00744	0.01197	2.36001	0.00000	0.00000	0.01419	0.01363	0.09247	0.02844	0.00709
**Fam20b**	1.54211	2.32060	1.41364	2.53034	0.15012	1.61086	0.08099	2.49073	1.39523	1.20280	0.58194	0.20173	0.67807	4.01459	5.67200	1.13038
**Fam21**	8.09221	5.02330	3.67694	9.11926	6.60890	8.25796	7.91445	9.71691	13.39236	15.42389	14.55230	13.10193	2.52520	3.61476	14.73232	3.66909
**Fam210a**	7.67495	1.90003	0.64306	4.69910	2.17382	1.41261	0.00593	0.98772	9.60474	4.45058	3.30230	12.15503	3.40652	7.29089	8.21959	1.72441
**Fam210b**	1.04057	0.00761	0.00643	1.14117	0.02038	0.00643	0.00682	0.00892	0.76676	0.40432	0.00608	0.01352	0.11868	0.00639	0.43402	0.00640
**Fam212a**	0.00925	0.00735	0.00000	0.97948	0.00806	0.00666	0.00630	0.00000	7.65258	7.71955	2.00093	0.29456	0.00000	0.09200	1.15243	0.00621
**Fam212b**	0.00799	0.00727	0.08151	0.00897	0.00914	0.01861	0.98829	0.00000	0.01184	0.31810	0.41217	0.01622	0.40704	0.03255	0.00894	0.00708
**Fam213a**	0.00000	1.17937	7.71975	1.58566	1.07465	13.64644	4.75209	0.39406	0.07722	0.00680	0.00234	0.02397	3.20762	0.01380	0.18454	11.54813
**Fam213b**	0.01207	0.00806	0.38775	0.01079	0.02129	0.00934	0.00711	0.01170	0.01228	0.00543	0.01288	0.02763	0.68939	0.01467	0.00341	0.00704
**Fam214a**	0.00000	0.00486	0.00000	7.04249	0.00803	0.00315	0.20118	3.35744	11.53333	6.21513	5.49964	5.08854	0.00000	12.80572	8.82302	0.00375
**Fam214b**	1.60839	1.90754	2.12490	4.36527	1.07510	2.59263	4.96159	7.26120	11.36102	4.32395	9.29265	3.28446	0.12540	8.77026	6.35825	0.04976
**Fam216a**	5.02501	0.00000	1.17953	6.78554	2.65176	0.01135	0.00011	0.59725	9.86358	2.71074	1.75344	0.04931	2.34438	0.02546	0.92608	3.21663
**Fam217a**	0.06669	0.00730	0.91393	0.01020	0.00642	0.00557	0.40201	0.00939	0.01214	0.01563	0.02815	0.00701	0.00818	0.01521	0.00000	0.00694
**Fam219a**	0.01980	0.00744	1.30651	0.06241	0.00657	0.00639	0.11565	0.00000	0.01061	0.00000	0.44679	0.00340	0.00428	0.00500	0.54296	0.00654
**Fam219b**	5.15249	1.53282	1.33300	0.97564	0.02002	0.03045	0.14835	1.82550	0.33645	1.68657	1.81845	0.00000	1.95340	0.30322	0.47043	0.05140
**Fam220a**	2.27654	3.08855	1.95345	13.60500	0.02054	0.24052	2.85384	1.74037	4.21875	2.56515	1.37259	0.64676	2.12727	0.85661	2.73765	0.26337
**Fam221a**	2.22497	0.04757	0.95814	0.42589	0.00342	0.06045	1.58066	1.79847	0.13014	0.57660	0.64250	0.13748	1.86244	0.00350	0.51962	0.01829
**Fam222a**	0.38186	0.00857	0.00490	1.17968	0.01556	0.00707	0.00728	0.01106	4.56829	0.07560	0.00000	0.00000	0.01273	0.00788	0.00835	0.00670
**Fam222b**	2.23520	3.68820	2.72497	8.72440	0.01756	3.24971	4.55801	0.09797	5.54187	4.70343	1.97545	0.31169	1.72280	0.00000	5.65019	0.67995
**Fam227a**	0.12500	0.03958	0.13784	1.79419	0.01089	0.07188	0.03668	0.03890	4.07424	0.56098	0.50886	0.02487	0.17888	2.57426	0.34888	0.06796
**Fam228b**	0.00921	0.00772	0.00000	1.40714	0.00900	0.01426	0.00637	0.26487	0.56458	1.65395	1.48422	0.01213	0.55786	0.57368	0.82700	0.00661
**Fam234a**	10.44205	3.98725	14.47014	1.59922	8.20320	1.65218	8.11806	21.58951	4.67452	12.91600	7.80813	1.00603	16.64607	0.07180	6.37804	4.41745
**Fam234b**	0.01270	0.00761	0.01163	0.00438	0.00887	0.00642	0.00729	0.01045	0.01061	1.65805	0.00938	0.01100	0.01088	0.00513	0.58668	0.00751
**Fam25c**	4.60469	0.38367	0.03461	0.00807	5.46461	0.00731	0.03391	0.00000	0.01033	0.00451	0.01084	0.05017	0.07279	0.00984	0.01070	2.37416
**Fam26f**	0.01323	0.00803	0.00671	1.57934	0.00887	0.00680	0.00719	0.00000	3.67771	0.21573	0.68719	0.00000	0.00688	0.03386	0.02214	0.00691
**Fam32a**	15.90144	6.55237	16.62937	8.36536	8.74701	8.20552	9.47854	16.21159	12.47535	14.98019	7.72049	8.06138	13.15920	4.44896	12.58184	4.51505
**Fam35a**	2.32388	0.77599	1.90861	0.61976	0.59518	0.00459	0.00500	0.68541	1.34885	0.07016	0.00000	1.62176	0.00000	1.20866	0.68066	0.00329
**Fam3a**	0.01711	0.00693	2.25763	0.96064	0.01488	0.73998	0.74425	2.49845	1.53430	0.92813	0.86763	0.00071	3.28480	0.53518	2.08918	0.00453
**Fam3b**	0.36323	0.00678	1.81219	0.08378	0.00397	0.00742	0.31176	0.00000	0.01083	0.02524	0.60587	0.00303	0.00897	0.01326	0.02082	0.00590
**Fam3c**	6.35203	3.65788	4.29456	3.81656	10.00567	2.30510	0.17794	1.34165	1.19078	2.88469	2.90842	0.49926	3.82009	2.81166	1.25769	1.32873
**Fam43a**	0.01319	0.00781	0.01210	0.19059	0.00889	0.00642	0.00723	0.00986	0.12250	1.09821	0.01360	0.00554	0.01039	0.01790	0.00302	0.00705
**Fam43b**	0.29638	0.00746	0.00416	6.01013	0.00723	0.00603	0.00697	0.00970	10.04636	0.00000	0.00000	0.00000	0.01260	0.02534	0.20100	0.00521
**Fam45a**	0.26833	6.83799	6.63924	5.94305	0.21196	5.12083	3.67420	6.19096	3.71641	9.22291	10.13634	9.34025	8.47880	17.98444	16.34190	0.02999
**Fam46a**	1.85770	0.00534	0.00000	0.12004	0.00550	0.00571	0.00577	0.00358	0.12986	0.05963	0.00878	0.02081	0.01861	0.00646	1.40234	0.00576
**Fam46b**	0.00747	0.00755	0.00505	4.54069	0.00883	0.00576	0.00639	0.00000	8.67925	0.57359	0.43945	0.00000	1.06725	0.00000	0.73356	0.00560
**Fam46c**	5.64450	5.73743	0.00000	3.51570	5.53371	0.01775	1.69362	10.54423	0.13556	2.10963	8.75073	0.41202	1.37235	0.00000	1.13544	0.00000
**Fam47e**	0.14532	0.00756	0.00773	0.21282	0.00764	0.00684	0.00726	0.00920	0.57822	0.86385	0.01962	0.00501	0.01119	0.01251	0.00354	0.00708
**Fam49a**	0.00957	0.00681	0.22447	2.84325	0.00843	0.00620	0.00675	0.01024	0.53557	0.00782	0.00000	4.22304	0.01111	0.00028	0.72110	0.00651
**Fam49b**	5.20410	3.06144	4.27138	0.00000	2.53760	2.33882	7.94594	0.00000	0.69982	3.49130	2.04394	7.91778	0.52207	1.76042	1.19110	2.53075
**Fam50a**	11.95034	4.96026	11.79997	7.42591	3.26176	7.92334	2.43203	11.36071	4.98930	11.70976	8.17024	1.73797	12.02262	10.41414	6.58363	4.74317
**Fam53a**	1.78949	0.18439	0.06466	3.73733	3.27959	0.01366	1.90626	1.03902	6.47640	6.76188	0.55575	0.05299	2.07760	0.00000	0.00000	0.73446
**Fam53b**	1.56596	0.00624	0.20143	0.76756	0.02493	0.94598	0.00583	0.00418	0.31257	1.10719	0.00813	0.02239	0.01587	0.03386	0.00104	0.02033
**Fam53c**	2.28278	2.51148	0.00000	11.37255	0.03817	3.04131	5.14534	2.37039	4.24181	3.87831	2.99400	0.32059	0.36139	0.00285	2.51846	0.02279
**Fam57a**	2.33352	0.00396	0.13177	0.16453	1.77289	0.00641	0.00522	0.53595	0.00384	0.00295	0.00843	0.00354	0.08767	0.00813	0.19007	0.00463
**Fam57b**	0.06934	0.01650	0.24733	0.85267	0.01635	0.07484	0.63652	8.43158	0.63689	12.39465	1.39734	0.30341	5.25215	0.00000	0.00000	0.00452
**Fam58b**	2.11559	0.15572	5.34839	7.03271	3.10173	0.71733	0.50989	5.44647	15.89752	7.49511	0.84817	0.30588	2.52636	0.00000	0.76804	4.45724
**Fam60a**	8.48381	4.78080	4.97743	1.65134	4.63821	2.27357	4.71386	5.34721	1.76903	5.72971	4.13852	0.12158	0.73207	11.52494	8.20520	1.71870
**Fam63a**	7.63690	7.09790	10.27246	14.09944	8.58291	10.04893	3.72553	15.63291	13.16713	19.74768	12.70135	5.98357	9.42277	3.41241	10.08738	2.85006
**Fam63b**	0.00871	0.00748	0.20734	1.81065	0.00842	0.00837	0.04623	0.00901	0.11457	1.63207	0.00774	0.02034	0.46131	0.10724	0.89299	0.00678
**Fam65a**	1.36333	0.05352	1.42300	1.58300	1.45525	8.15702	4.19007	8.23098	1.36911	7.88949	6.26204	0.41881	7.17027	0.00000	1.18101	2.36518
**Fam65b**	0.06729	0.01810	0.24229	0.64526	0.01875	0.01293	0.04775	0.01008	0.28489	0.58228	0.59987	0.09695	0.08780	0.53646	0.12036	0.01410
**Fam65c**	0.01266	0.00811	0.01178	0.00651	0.00893	0.00665	0.00737	0.01673	0.01149	1.34535	0.01132	0.00511	0.01079	0.03295	0.02180	0.00734
**Fam69a**	1.20241	2.96454	0.00000	1.07118	0.02260	6.13973	4.73240	4.14996	2.37146	1.91339	0.61368	6.39861	2.84074	2.08552	4.09642	0.08023
**Fam69b**	0.05090	0.00752	0.41373	0.42936	0.00716	0.00901	0.00705	0.01001	0.51384	1.56729	0.00720	0.03075	0.00979	0.00532	0.09733	0.00711
**Fam72a**	0.75916	0.32030	0.26887	5.47500	1.10390	6.19863	0.01600	0.53529	15.76586	0.36145	0.81780	0.78088	0.50261	0.00000	0.39792	0.00201
**Fam73a**	0.02608	0.00631	1.18741	2.40337	2.48408	3.48060	0.00522	1.52546	2.42596	4.20985	2.88566	1.13625	0.87130	2.28198	4.26208	1.39256
**Fam73b**	0.19767	0.00895	1.78888	1.88154	0.01373	1.63659	0.00740	0.72239	0.53741	0.56169	0.88810	0.04374	0.00000	0.00642	0.15978	0.15105
**Fam76a**	5.05546	0.10030	2.96289	5.34698	0.02826	6.20164	4.41865	7.32766	5.02348	15.47614	8.98924	6.66987	4.40414	7.38018	15.26006	1.53018
**Fam76b**	0.04749	2.25609	1.82641	3.65404	0.00583	1.50177	0.69252	0.46526	6.62817	2.67994	1.00342	2.15363	1.80528	2.51386	5.24311	0.02473
**Fam78a**	0.11871	0.00802	0.04484	0.00959	0.00758	0.00576	0.00732	0.15830	0.01197	0.03755	0.01096	0.00792	0.00715	0.00703	0.12554	0.00697

**Fam83a**	0.31086	0.04064	0.36638	0.52044	0.05841	0.60083	0.10594	0.05394	0.69741	0.01611	0.66262	0.05774	0.29669	0.40805	0.03617	0.13097
**Fam83b**	0.00923	0.00851	0.00809	0.34139	0.00962	0.00689	0.00820	0.58056	0.25053	0.07108	0.00725	0.00803	0.00551	0.01786	0.00000	0.00710
**Fam83d**	1.19719	0.65249	0.99746	2.75578	0.27319	0.00376	0.01640	1.46727	7.63572	0.15730	0.75635	1.02059	0.25099	0.00000	1.31677	0.02018
**Fam83e**	0.00921	0.00753	0.31352	0.06583	0.00901	0.00915	0.00705	0.48724	0.02578	0.00460	0.04422	0.02485	0.00424	0.01300	0.00000	0.00730
**Fam83g**	0.99981	0.00720	0.05255	0.96516	0.01656	0.36241	0.00527	2.11382	0.07712	1.80969	2.24771	0.05367	0.59369	0.00000	0.12909	0.00558
**Fam83h**	1.09255	0.00526	0.00000	0.02777	0.03586	1.06561	2.39259	0.39786	0.01172	0.04634	1.33191	0.02303	0.82389	0.12553	0.00000	0.00577
**Fam84a**	0.01282	0.00794	0.01107	0.51419	0.00881	0.00643	0.00724	0.00933	0.38446	1.57545	0.02423	0.02331	0.01079	0.00474	0.40533	0.00736
**Fam84b**	0.00000	3.13261	0.83281	5.08688	0.01378	0.04696	0.02873	0.15117	1.59098	0.47829	0.03855	0.04595	1.69291	0.00000	3.24225	1.29915
**Fam89a**	0.00827	0.00902	0.01516	1.14533	0.00922	0.00716	0.00826	0.29791	2.20304	0.67109	0.00000	0.00261	0.03935	0.03425	0.00000	0.00678
**Fam89b**	1.70427	1.75481	0.00000	2.07212	0.12032	2.06596	0.00529	1.76121	4.94832	2.51414	0.74126	0.29098	0.00000	0.23179	0.27378	0.00400
**Fam8a1**	0.01314	0.00847	0.00526	1.37103	0.00987	0.01529	0.00744	0.00483	0.27729	0.01523	1.41148	0.00126	0.08201	0.00219	0.05550	0.00749
**Fam90a1a**	0.01358	0.00829	0.01179	1.11447	0.00927	0.00650	0.00750	0.01355	1.17869	0.00853	0.01784	0.00638	0.01264	0.01092	0.00260	0.00715
**Fam91a1**	4.68084	6.73643	5.06300	7.55979	6.95297	8.07855	6.36763	5.82327	7.19071	10.77427	8.20073	19.67966	2.83027	21.83782	10.68623	0.30406
**Fam92a**	0.96194	0.02889	1.00301	0.92622	0.20670	0.00806	1.04503	0.03337	0.44505	0.20255	0.02054	0.02104	1.09501	0.00885	0.00000	1.30150
**Fam92b**	0.01347	0.00779	0.01215	0.00811	0.00905	0.00661	0.00728	0.01049	0.01195	0.64346	0.01270	0.00578	0.01194	0.01149	0.00933	0.00778
**Fam96a**	28.14843	17.73567	27.45271	1.55749	10.28230	15.54554	15.31806	22.36568	2.21466	18.11556	14.99579	5.70737	23.40341	3.88257	8.06751	12.35802
**Fam96b**	10.42372	2.00600	6.07861	6.11306	4.82590	2.56469	4.33765	2.66651	2.34809	5.62976	6.69084	1.03613	8.05251	0.13019	0.00000	2.48153
**Fam98a**	8.80815	4.16373	3.07031	7.12922	5.07030	2.49990	2.98680	9.26442	4.92942	8.00704	9.50661	2.87743	6.76192	7.75316	6.21289	3.15252
**Fam98b**	4.01579	0.29487	1.64763	9.63457	0.16739	0.68709	1.09212	0.29461	11.59056	2.92077	10.80388	0.25834	3.40089	5.56287	6.03424	1.26962
**Fam98c**	0.05699	0.00727	0.38247	0.00559	1.26662	0.00573	0.00697	0.64171	0.01061	0.39836	0.32166	0.01184	0.66189	0.00720	0.02585	1.96441
**Fan1**	0.01355	0.00791	0.01232	0.08913	0.00946	0.00661	0.00751	0.01243	0.01098	0.06327	0.01199	0.01647	0.02563	0.00556	0.38976	0.01458
**Fanca**	0.57452	1.50909	0.88185	0.36000	0.00435	0.95383	2.07059	2.24014	0.16447	1.74089	0.00000	0.07473	1.68080	0.17335	0.92964	1.88481
**Fancb**	0.00000	3.36118	2.27966	1.03164	6.74810	0.00640	0.05671	1.16821	0.00000	1.95776	1.27428	0.75915	0.33608	2.24441	0.81127	1.44736
**Fancc**	3.61552	0.00321	1.05673	3.80879	0.30565	1.06427	1.48800	2.56570	0.36951	1.54079	0.59387	0.10987	0.00000	1.13581	4.62270	0.00208
**Fancd2**	4.03494	3.09639	2.87561	1.89983	0.01000	0.08461	0.96873	0.05199	0.70337	0.68217	1.67924	0.85651	4.59354	1.19477	6.00221	0.03500
**Fancd2os**	0.30643	0.00679	0.37421	3.69776	0.00655	0.00728	0.40076	0.12281	2.55205	0.80047	0.68406	0.04866	0.54623	1.19204	3.53163	0.00587
**Fance**	6.35151	0.06167	1.59680	1.90578	0.00000	6.16264	2.27761	1.89352	3.27729	3.51176	4.05815	0.11472	1.19464	0.04423	0.57923	3.00701
**Fancf**	0.02019	0.00811	0.00000	1.35930	0.00856	0.29881	0.00760	0.00303	3.67890	0.02220	0.66990	0.00000	0.00328	0.00377	0.00000	0.00666
**Fancg**	4.68482	0.00000	2.97549	1.92762	0.00901	4.86211	1.26231	1.71828	2.39374	0.54449	0.88091	0.06348	3.30133	0.01429	1.59887	0.01332
**Fanci**	2.63010	0.18655	2.68431	1.62626	4.08483	0.01916	2.18107	3.50516	1.09476	2.78451	1.09659	0.14638	1.56159	5.59504	2.83266	2.05646
**Fancl**	1.96124	4.27214	2.12618	0.00000	0.00994	0.01581	0.01382	4.33416	0.08023	3.86360	1.67372	0.92798	0.18029	0.67514	2.85339	3.41098
**Fancm**	6.98398	4.68283	0.31696	9.66752	0.02424	0.00000	2.15136	1.03735	13.44359	7.71749	7.12921	8.56725	0.76457	5.96719	8.58566	0.29910
**Fank1**	1.39319	2.52299	0.00000	4.81532	0.00499	0.26944	0.23887	0.00000	0.00000	0.26583	0.07087	0.13691	1.62045	0.00000	1.51445	0.91318
**Far1**	0.68242	2.42386	1.34405	4.47160	2.40300	2.85576	0.38641	0.98801	10.39774	0.00000	1.91109	0.14286	1.06217	3.61578	0.24509	0.39692
**Far2**	0.12121	0.00883	0.00379	0.01088	0.01536	0.00775	0.00809	0.27150	0.01153	0.00634	0.00979	0.00815	0.00659	0.02623	0.12238	0.00741
**Farp1**	0.04413	0.00818	1.88117	3.45995	0.01423	0.04377	0.00641	0.00657	2.71023	0.35278	0.18102	0.16328	1.51553	0.47985	2.30822	0.01227
**Farp2**	1.24696	0.00574	0.74403	0.24462	0.00409	0.00424	1.13601	1.51381	0.29646	0.98226	0.00000	0.07745	0.00449	0.75534	1.67106	0.00543
**Fars2**	0.45610	2.20015	3.39340	12.70944	1.98723	0.18958	0.00244	1.89533	0.00000	2.46946	1.03997	5.73106	3.93382	0.00000	1.24482	0.00829
**Farsa**	11.53106	5.10300	13.18465	2.55957	3.56571	0.43366	4.66347	12.63872	1.07091	11.13827	8.58084	1.87002	9.85730	4.23115	4.23299	2.09513
**Farsb**	20.33021	13.68696	17.11194	7.08924	16.77800	8.92927	13.65701	21.48948	6.81116	13.52227	14.50279	10.22837	19.41947	19.17117	11.60573	11.81955
**Fasn**	13.14685	6.66744	10.25928	3.08282	10.02227	2.10535	13.10758	22.69856	2.68220	21.85426	4.88851	2.45793	7.97263	1.50413	0.00000	10.77701
**Fastk**	6.76924	2.37041	5.37599	0.13367	2.44109	6.94531	5.88132	7.04560	0.40903	2.60628	1.22023	0.18221	8.70692	0.01336	0.00000	4.46875
**Fastkd1**	0.26152	0.10870	0.01151	1.45128	0.61784	0.00696	0.00633	0.72972	2.24692	0.50474	0.65757	0.17889	0.01970	10.73263	4.72031	0.00643
**Fastkd2**	5.97928	0.00000	6.52243	0.92323	3.11859	2.32414	0.67970	1.92932	0.14500	5.83983	2.76083	7.61967	4.90739	8.59473	3.77879	0.01618
**Fastkd3**	6.06736	2.47063	5.44824	12.00606	0.06220	1.14295	4.48618	2.18924	9.95491	5.65495	3.18200	12.43413	5.26107	0.00000	9.00153	1.43580
**Fastkd5**	1.69998	0.18708	2.15597	5.77644	0.01150	3.06768	4.15179	1.37199	5.93706	4.02864	0.61734	0.04345	1.05760	2.68032	4.52060	0.00028
**Fat1**	2.20755	1.79074	1.14013	2.37286	2.91651	0.25545	3.41167	1.87396	0.00000	0.04470	0.56695	0.20272	0.77504	0.72141	1.95622	0.04776
**Fau**	32.72855	4.67374	25.17728	18.30706	19.17026	9.64039	8.05121	18.43110	25.32398	34.23591	34.30803	4.58846	29.56085	0.17508	1.12184	18.16736
**Faxc**	0.00859	0.00785	0.00000	2.66319	0.00947	0.00622	0.00685	0.03365	1.95318	0.98272	0.58289	0.01804	0.00763	1.24371	1.91768	0.00665
**Fbf1**	0.06324	0.03702	1.35040	0.98464	0.00589	2.06881	0.01522	0.00000	0.70474	0.02258	0.76476	0.01784	2.48915	0.00433	0.57032	0.01180
**Fbl**	26.45791	14.09028	24.17513	19.17038	15.14633	9.03690	15.24944	27.17254	24.92524	27.66393	28.09752	5.00858	25.45051	7.49388	16.42940	14.11588
**Fblim1**	6.78176	10.70998	9.73944	3.98432	8.96947	2.45457	7.73238	14.44480	4.80342	10.62153	12.40953	2.41898	6.09064	1.92936	4.61723	4.99810
**Fbln1**	1.15869	2.29622	0.25287	0.05704	2.31708	2.88818	0.75545	0.84724	0.07492	0.56712	0.00000	0.01050	1.62331	0.06469	0.00860	5.55502
**Fbln2**	0.01385	0.00794	0.00969	0.24319	0.00935	0.00660	0.00730	0.01117	0.22616	0.00332	0.01037	0.14364	0.01306	0.00617	0.39626	0.00734
**Fbln5**	0.00459	0.00786	0.01097	0.01065	0.00667	0.00628	0.01752	0.01821	0.01242	0.00879	0.01208	0.02828	0.00522	0.01256	0.00992	3.41678
**Fbln7**	0.01001	0.00830	0.81432	0.00000	0.00741	0.00555	0.00748	0.01098	0.00857	2.92698	0.00615	0.02042	0.00803	0.06449	1.53805	0.00687
**Fbn1**	0.01366	0.00789	0.00969	0.00995	0.00919	0.00685	0.00749	0.01483	0.01213	0.15872	0.01169	0.17936	0.01275	0.01163	0.00762	0.00777
**Fbp1**	2.43238	0.00277	0.81491	0.31450	1.52560	0.00909	2.82525	0.14633	0.11792	0.00000	0.00950	0.01068	0.02994	0.06040	0.02603	3.06680
**Fbp2**	32.53176	20.72002	29.24078	0.26492	23.56631	15.81056	15.52719	24.04644	0.00000	11.66082	13.23383	0.96949	27.26825	7.43834	0.00000	18.93261
**Fbrs**	3.41081	0.14328	0.75274	6.76619	1.28264	0.23297	0.60660	4.28959	5.38378	1.92225	4.11649	0.07792	2.82748	0.42049	1.85753	4.71049
**Fbrsl1**	7.35149	3.49211	5.19838	13.28991	0.67911	2.20879	2.46969	9.91359	17.67880	18.86335	6.32549	1.62021	4.31080	1.23736	10.85226	2.53177
**Fbxl12**	1.65560	0.02606	0.14837	4.66702	0.01200	0.01074	0.00538	0.27853	7.74390	0.54777	0.53231	0.01591	0.39465	0.00629	0.66883	1.85661

**Fbxl12os**	0.00915	0.00776	0.01286	1.77049	0.00943	0.08531	0.05793	0.10128	2.25788	0.03906	0.00000	0.04270	0.03573	0.30366	0.02933	0.03605
**Fbxl13**	0.01059	0.00771	0.00457	1.34203	0.00916	0.00663	0.00702	0.00973	0.00000	1.63707	0.10425	0.05200	0.01037	1.94579	0.00000	0.00712
**Fbxl14**	0.16410	0.01731	0.90074	3.05541	0.03062	2.07349	0.92325	1.45981	0.40517	1.71432	0.96400	0.06955	1.35118	0.03833	2.09276	0.83361
**Fbxl15**	0.01233	0.00721	0.01882	0.00461	0.98898	0.00755	0.00734	0.01398	0.01089	1.91021	0.01232	0.00000	0.27854	0.01304	0.00647	0.15009
**Fbxl16**	0.32305	0.00696	0.00000	0.00253	0.00816	0.05435	0.01613	0.65491	0.57557	0.42746	1.59095	0.02329	0.52022	0.00586	0.85178	0.01400
**Fbxl17**	1.75758	0.03486	1.57642	5.63951	0.09489	1.41976	0.32014	1.49763	3.97665	0.29234	0.30185	0.07467	0.06671	0.60224	1.15983	0.00324
**Fbxl18**	0.42768	0.00611	3.77009	0.67484	0.02125	0.00591	0.00515	4.95580	0.09737	3.77743	1.48609	0.04944	1.35064	0.00043	0.00000	2.33536
**Fbxl19**	1.56517	0.65101	0.72759	2.38195	2.41060	0.00469	0.00439	1.29750	1.73862	2.01148	1.35647	0.00711	1.83842	0.00000	0.13845	0.83876
**Fbxl2**	0.01653	0.00755	0.23946	0.78449	0.00883	0.00556	0.00723	0.01098	0.58335	0.00238	0.00790	0.02529	0.01029	0.04250	0.14231	0.00708
**Fbxl20**	6.57267	5.52788	7.81179	1.55116	5.72604	0.00866	7.33379	19.36763	6.05824	16.14780	14.37473	2.86054	4.23109	10.88964	17.49733	0.00071
**Fbxl21**	0.01347	0.00800	0.01219	0.17838	0.00934	0.00660	0.00745	0.01829	0.01713	0.02351	0.01389	0.00787	0.01234	0.01119	0.00737	0.01475
**Fbxl22**	0.00739	0.00798	0.01184	0.07677	0.00760	0.00638	0.00744	0.01387	0.24900	0.00480	0.01058	0.00786	0.00754	0.01366	0.00661	1.43833
**Fbxl3**	0.33374	3.02979	1.67469	2.10510	1.22603	0.03732	3.73052	0.93617	4.19416	0.15135	3.47035	5.89805	0.77964	9.89147	11.34090	1.05181
**Fbxl4**	0.96417	0.00612	1.99343	1.30927	0.39705	0.00432	0.00631	0.00313	1.16193	0.56828	0.02168	0.00577	0.00726	0.44645	2.10654	0.00551
**Fbxl5**	3.90540	0.00084	1.27178	12.25956	0.17662	0.00528	1.53786	0.00000	3.67115	7.25703	4.68046	2.07782	1.40824	1.19198	9.18775	2.64938
**Fbxl6**	3.54991	1.50441	2.52170	0.21198	1.09783	3.01437	7.57503	6.78147	0.26690	6.79577	0.07190	0.00000	3.46522	0.10198	1.35320	0.08539
**Fbxl7**	0.01381	0.00806	0.01008	0.28131	0.00914	0.00667	0.00754	0.00805	0.15911	0.00237	0.28611	0.00650	0.00908	0.00620	0.05533	0.00718
**Fbxl8**	0.55616	0.00798	0.01223	0.58391	0.01710	0.01418	0.00658	0.00702	0.19095	0.85353	0.01129	0.00482	0.28294	0.01055	0.00345	0.00680
**Fbxo10**	0.01133	0.00774	0.01033	0.02444	0.01878	0.00662	0.00731	0.04942	0.99422	0.16557	0.00441	0.03397	0.01093	0.00776	0.19075	0.00773
**Fbxo11**	3.36466	6.31004	4.93764	20.24733	3.33880	0.95313	8.00659	3.66945	17.40668	7.34665	3.71525	0.46424	2.84141	21.50520	15.42330	0.17574
**Fbxo15**	32.38671	25.23479	42.12146	26.64958	23.67793	17.39557	30.30802	53.53515	37.83159	61.09934	52.34212	33.96273	40.61745	62.46051	46.41962	17.51902
**Fbxo16**	0.00000	1.09671	0.42539	4.55975	0.00787	0.00844	0.00569	0.00000	5.60793	0.54895	1.78394	0.00000	1.58922	0.39626	0.00000	0.00520
**Fbxo17**	1.97108	1.79542	1.15415	2.28634	1.89681	0.03506	0.04494	1.03031	3.21161	0.67995	1.93906	0.01399	0.55028	1.55945	1.35467	0.02482
**Fbxo18**	4.44096	3.38008	4.10179	7.44756	0.79407	4.18471	3.56157	0.14571	3.51241	3.22572	2.10018	0.22491	1.96883	0.04801	6.75236	0.18508
**Fbxo2**	4.43044	0.00282	0.16961	1.27915	0.15304	0.01227	0.00339	0.50079	0.37746	0.48934	0.01931	0.11714	0.44080	0.00891	0.00000	0.00201
**Fbxo21**	8.41770	4.85329	6.21398	0.53403	2.15940	1.53878	2.48448	4.22584	0.56755	0.48655	0.00000	2.92749	0.96495	0.17733	0.27213	3.62094
**Fbxo22**	10.20105	2.07185	4.96822	3.25580	5.25644	0.31811	7.22755	5.93826	1.90436	12.27202	4.43176	1.31594	7.30750	1.69313	7.13536	5.37028
**Fbxo25**	1.62635	0.00614	0.87491	1.08137	0.43812	0.00426	0.31533	0.27670	0.32860	1.19716	0.04273	0.02200	0.07045	0.01933	0.08162	0.01280
**Fbxo27**	1.92882	0.00515	0.52094	0.19687	0.00871	1.67873	0.94580	0.00186	0.18376	0.49217	0.01787	0.01352	0.03530	0.01061	0.16652	0.51109
**Fbxo28**	2.70613	5.39900	2.56396	5.44471	0.61923	0.00257	1.03202	1.17579	7.20667	1.91225	0.70456	2.50247	2.71290	2.59142	3.99406	1.36580
**Fbxo3**	6.73238	8.28095	4.38224	0.09532	8.91237	0.00000	2.68229	0.46031	0.19367	0.00000	0.00002	0.06301	2.72212	0.02011	0.03400	2.14694
**Fbxo30**	1.60453	6.94486	2.33223	2.28348	0.72899	1.54127	0.00344	1.78365	2.08106	2.40985	0.00000	0.16609	0.21214	7.41125	2.10378	0.33485
**Fbxo31**	5.94498	0.08133	2.14489	0.08314	4.89949	2.14847	0.64541	1.01538	1.19537	19.42211	6.16020	0.69785	6.06687	0.25768	0.00000	4.03727
**Fbxo32**	0.11236	0.00774	0.15087	3.57687	0.02975	0.00592	0.00676	0.00000	2.35575	0.00000	1.32975	0.06732	0.03808	0.47009	0.05103	0.02800
**Fbxo33**	3.29299	1.36341	1.44825	20.77480	0.19045	0.75698	4.72003	5.35155	27.00068	9.11763	10.58999	10.53878	2.03685	14.43143	18.60615	0.71017
**Fbxo34**	2.89084	0.00190	0.18269	12.15629	0.60695	0.01307	0.00190	11.92344	10.02124	13.09863	4.84540	1.71104	1.20166	3.81178	12.39803	0.00000
**Fbxo36**	0.01595	0.00758	0.70449	5.06969	0.00735	0.00508	0.00664	0.00000	7.28293	2.03564	0.90295	0.06939	0.00000	1.67211	1.78770	0.00547
**Fbxo38**	5.30566	7.60602	1.89302	4.08085	3.03652	3.29622	4.30454	4.50619	8.22186	7.41807	3.87647	10.07307	2.83363	5.76619	2.50546	3.96670
**Fbxo4**	0.01303	0.00825	0.01175	0.16418	0.00915	0.00650	0.00739	0.02025	0.44843	0.45802	0.00796	0.00593	0.01068	0.03211	0.00284	0.00710
**Fbxo41**	0.01276	0.00770	0.29373	0.71972	0.00866	0.63154	0.00710	0.00865	0.24454	0.13191	0.07744	0.01568	0.00631	0.01107	0.00000	0.01426
**Fbxo42**	2.45083	0.14773	3.63385	9.88093	11.48841	5.56698	1.09486	5.55124	7.21878	5.42301	2.63256	1.13011	3.88836	0.80475	2.54735	5.18052
**Fbxo43**	0.02637	0.00781	0.19756	6.05369	0.02807	0.00624	0.00684	0.01268	2.65642	0.52621	0.42409	0.01548	0.11190	2.66103	3.34067	0.02027
**Fbxo44**	1.80758	0.00669	0.19766	0.63152	0.00814	1.65435	0.00658	0.26874	1.12226	0.59426	0.00000	0.01427	0.00000	0.00507	0.14803	0.00501
**Fbxo45**	0.18138	0.00435	1.39600	11.42505	0.30646	2.85687	1.65146	2.12786	11.92709	6.66831	2.67316	5.13382	0.00000	4.41938	13.08348	3.57759
**Fbxo46**	0.31839	0.00805	0.01274	0.50422	0.03512	0.01574	0.00669	0.00000	2.01841	0.36411	0.37056	0.07402	0.00863	0.53245	0.16567	0.01460
**Fbxo47**	0.04374	0.00717	0.00150	2.53327	0.00826	0.00631	0.00701	0.32630	1.88695	0.13231	0.02868	0.01669	0.00638	0.02128	1.87905	0.00680
**Fbxo5**	3.15523	6.42392	7.89740	2.77716	7.81604	4.09165	5.61084	10.08240	12.09784	7.86608	8.90776	6.28825	7.00391	8.33861	8.96925	3.43389
**Fbxo6**	19.78399	10.93930	15.80641	5.19835	8.38596	5.76804	6.49543	15.86795	14.41743	15.53300	6.44621	2.66729	14.85247	1.32113	3.87385	6.87309
**Fbxo7**	0.00000	2.91658	0.00000	0.41874	2.83671	0.00699	3.85560	0.03809	0.01667	1.93848	0.00721	0.15676	0.65019	0.00411	1.07448	0.02825
**Fbxo8**	4.12689	0.00000	2.57040	7.36851	5.84592	0.00000	0.02265	2.84503	4.87810	8.01222	0.21371	8.06631	1.75008	3.05411	1.65474	3.88622
**Fbxo9**	0.19462	3.00003	2.67126	2.43202	1.96223	7.23559	0.41456	2.98456	5.50975	2.14575	3.61506	0.84476	4.10918	2.75042	5.89330	2.02010
**Fbxw11**	6.03909	0.09139	8.56814	11.63362	6.15285	4.72330	6.84497	7.27759	14.72075	7.31532	5.52451	0.63450	6.38356	0.84018	6.90782	4.17954
**Fbxw13**	0.01373	0.00789	0.00908	0.57786	0.00932	0.00654	0.00732	0.01096	0.86847	0.01883	0.00000	0.20336	0.01301	0.02865	0.41817	0.00714
**Fbxw14**	0.01058	0.00750	0.00018	12.04177	0.00900	0.00451	0.00661	0.15586	6.50872	1.01722	0.28220	0.02729	0.00593	0.00000	4.56466	0.00491
**Fbxw15**	0.00125	0.00748	0.00002	9.21779	0.01095	0.00485	0.00657	0.64835	2.59539	0.88512	0.23309	0.21543	0.00482	3.45195	5.83918	0.00560
**Fbxw16**	0.01133	0.00761	0.00433	8.10030	0.00920	0.00508	0.00694	0.02183	1.76602	0.81523	0.06448	0.10185	0.01036	0.04635	8.17595	0.00590
**Fbxw17**	0.01372	0.00771	0.01225	0.24507	0.00937	0.00648	0.00740	0.01221	0.00818	0.03570	0.01298	0.00678	0.01989	0.00529	0.25051	0.00740
**Fbxw18**	0.01346	0.00768	0.00793	4.38190	0.00925	0.00591	0.00712	0.00540	0.83792	0.00000	0.15898	0.02131	0.01066	0.00000	2.23368	0.00646
**Fbxw19**	0.00813	0.00763	0.00000	8.84096	0.00868	0.00459	0.00693	0.02490	6.04000	1.43796	1.33819	1.29278	0.00000	0.04181	5.34828	0.00504
**Fbxw2**	3.23352	0.29785	3.61216	4.40166	5.09028	2.84390	0.74331	5.26330	9.36453	5.71603	8.67255	2.19404	0.00000	3.48697	8.19705	0.64881
**Fbxw20**	0.00850	0.01898	0.00711	1.67756	0.01039	0.00627	0.00729	0.01460	4.77066	0.00000	0.00000	0.02753	0.22618	1.69452	1.45728	0.01452
**Fbxw21**	0.01145	0.00799	0.00468	3.30923	0.00945	0.00593	0.00709	0.01306	1.07594	0.24504	0.02318	0.15769	0.01137	1.39259	1.97623	0.00651

**Fbxw22**	0.01329	0.00792	0.00746	3.90196	0.00915	0.00612	0.00741	0.01032	1.87279	0.01283	0.22777	0.05408	0.00954	0.00062	0.86270	0.00652
**Fbxw24**	0.00002	0.00754	0.00088	18.78660	0.01926	0.00326	0.00603	0.00000	6.97822	1.27696	0.18836	1.01987	0.41850	3.79919	10.28679	0.00336
**Fbxw26**	0.01154	0.00741	0.00463	0.62895	0.00947	0.00680	0.00702	0.45896	1.00290	0.00000	0.20598	0.02055	0.00544	0.00921	0.81583	0.00720
**Fbxw27**	0.01057	0.00789	0.00363	4.13222	0.00932	0.00593	0.00722	0.32157	1.77629	0.25168	0.12472	0.00000	0.00580	0.00000	2.63203	0.00663
**Fbxw28**	0.00913	0.00748	0.00000	9.63979	0.00945	0.00491	0.00671	0.22705	4.22260	0.45251	0.30754	0.04042	0.00593	0.00000	5.36846	0.00545
**Fbxw4**	0.01748	0.00769	0.01762	6.32631	0.00889	0.00655	0.00704	0.00811	0.43646	0.30826	0.12065	0.00496	0.01081	0.01372	0.03207	0.00636
**Fbxw5**	0.14834	0.02858	0.02733	3.01781	1.17022	0.03986	1.72799	0.26228	0.99583	0.00000	0.63378	0.03866	1.66360	0.00034	0.03391	2.52307
**Fbxw7**	1.76620	0.07585	1.74985	0.93944	0.03434	0.00461	0.00635	0.01985	0.69506	0.26343	0.00437	0.02566	0.02705	0.00674	0.61093	0.24551
**Fbxw8**	5.30462	2.57054	5.20940	0.30053	5.43999	0.01580	2.21866	4.35111	0.39213	4.08392	1.68919	0.21372	2.82823	0.06363	0.21250	4.38844
**Fbxw9**	0.91478	0.10420	4.68064	6.27802	0.96734	4.31824	5.56450	7.70334	8.26631	6.12145	3.76029	1.67611	5.96028	0.24442	0.50570	0.10220
**Fcer1g**	0.01287	0.00784	0.01216	0.18342	0.00882	0.00651	0.00723	0.00981	0.00581	1.68793	0.01175	0.00402	0.01059	0.01475	0.00384	0.00728
**Fcer2a**	0.17372	0.00773	0.16265	0.09199	0.00954	0.00624	0.05870	0.04603	0.44773	0.09138	1.22131	0.08915	0.23121	0.37216	0.11025	0.05011
**Fcf1**	11.26535	8.98618	10.46095	6.68881	3.98194	2.14482	3.35264	5.38255	9.54455	21.02621	15.78093	3.12276	10.13290	16.00773	12.90401	3.61156
**Fcgbp**	0.01290	0.00813	0.01022	0.88504	0.00906	0.00669	0.00748	0.00707	0.09520	1.24187	0.22572	0.00313	0.01271	0.00569	0.55990	0.00728
**Fcgr1**	0.01337	0.00831	0.01103	0.22346	0.00921	0.00671	0.00760	0.01356	2.45989	0.05184	0.00000	0.02868	0.01361	0.01127	0.00332	0.00725
**Fcgr2b**	0.63919	0.27651	0.00000	0.00001	0.01447	0.00519	0.14489	1.78967	0.00548	5.66088	0.00000	0.72134	0.80577	6.58646	4.46011	0.74765
**Fcgr3**	1.53227	3.07864	0.00000	21.07981	0.13988	0.00000	1.52104	15.56529	23.12919	30.96545	12.22982	10.51533	0.67173	29.15465	21.25071	6.29878
**Fcgrt**	0.00042	0.00672	0.65880	1.65693	0.00907	0.00861	0.87216	1.63211	1.59601	2.53624	1.84898	0.20451	0.03541	0.00104	0.11679	0.00698
**Fcho1**	0.45301	0.00760	0.00549	0.28247	0.00693	0.00708	0.00713	0.01450	0.33037	0.01361	0.01139	0.00746	0.01222	0.01172	0.00504	0.00677
**Fcho2**	0.03195	1.06954	0.07958	4.23192	0.29361	3.64826	3.50136	0.65453	1.06277	3.17733	2.56365	8.52311	0.00000	5.04998	2.99909	1.00380
**Fchsd1**	0.37627	0.00729	0.00365	0.12129	0.00699	0.00751	0.00689	0.00000	0.44845	0.06126	0.92635	0.08283	0.02392	0.03401	0.00000	0.06913
**Fchsd2**	0.04399	0.00762	0.00000	11.80081	0.01535	0.87384	0.68266	0.13977	2.06147	0.00000	0.18332	0.03820	0.00000	2.05074	7.08353	0.07972
**Fcrl5**	0.08928	0.09960	0.01783	0.20409	0.00781	0.17588	0.16035	0.03014	0.19582	0.02448	0.37577	0.39061	0.10965	0.06490	0.06800	0.00709
**Fcrl6**	0.03141	0.00781	0.02333	0.01523	0.00822	0.06910	0.03957	0.02492	0.03659	0.02680	0.02493	0.00783	0.01632	0.01180	0.04022	0.07904
**Fdft1**	4.56301	9.73143	14.10743	19.14474	9.06872	5.40877	11.67622	26.82060	24.85839	25.47581	19.98618	12.59752	5.70548	12.67501	17.75891	2.61835
**Fdps**	20.74781	16.22580	32.15902	3.35839	19.98935	6.72648	16.53013	24.78334	5.40104	22.87446	17.35099	10.64873	11.53252	18.50068	8.65179	2.93253
**Fdx1**	0.03896	1.35372	0.73392	0.36216	2.25139	0.63974	0.04582	0.71106	1.00564	1.98909	6.62073	0.12970	1.40803	0.41598	0.06993	0.01221
**Fdx1l**	0.11194	0.00799	0.01438	0.00917	0.01061	0.00796	0.01753	0.09792	0.01200	0.33052	0.01341	0.00643	0.69598	0.01281	0.00828	0.48923
**Fdxacb1**	0.37380	2.67819	3.26340	0.28192	0.00305	0.00782	4.22134	0.35305	0.00319	0.60658	0.33343	0.01110	0.07826	1.71403	1.79210	0.01251
**Fdxr**	4.61234	3.86426	1.70168	0.83428	7.92589	0.00184	4.70996	1.56297	0.10792	1.58819	1.51834	0.09297	0.01202	0.00000	0.00000	0.00780
**Fech**	1.95476	2.56410	2.95110	2.28381	2.95065	0.01038	1.19733	4.09637	0.78855	6.47471	0.37350	0.50682	0.93772	0.17269	2.24812	0.03240
**Fem1a**	0.02128	1.52221	1.33673	6.10607	0.02198	0.00669	0.00585	0.00000	15.08789	0.23680	0.97634	0.00000	0.08433	0.10129	2.08493	0.01969
**Fem1b**	6.00816	6.14593	5.90448	14.45327	9.15956	6.32557	3.68006	4.65108	14.17459	7.60349	2.70141	5.15034	2.83234	12.98340	16.64848	5.18873
**Fem1c**	1.10974	3.25470	1.34405	16.20256	1.00796	0.00096	0.96609	2.13908	20.52806	4.46158	2.45691	3.84085	0.89782	6.17636	7.33584	2.53973
**Fen1**	5.19901	11.70098	12.02665	4.28268	5.31321	7.90446	4.19172	12.01615	4.98304	4.66742	4.29056	0.75788	7.09372	0.29178	7.41332	6.14237
**Fendrr**	0.01939	0.00780	0.00829	0.12052	0.01775	0.00684	0.00730	0.01188	0.03498	0.00549	0.35322	0.00595	0.00829	0.13101	0.00000	0.00716
**Fer**	2.82540	0.00551	1.34792	4.75709	0.00260	0.00823	1.29598	0.21656	0.00000	2.27360	0.02516	0.03651	0.90540	0.02727	0.04327	0.01007
**Fer1l4**	0.01169	0.00788	0.00723	0.50350	0.00947	0.03722	0.00723	0.10292	0.00768	0.01238	0.03034	0.00768	0.00811	0.01079	0.00299	0.00729
**Fer1l5**	0.01243	0.00786	0.00930	0.58024	0.00931	0.00672	0.00722	0.04460	0.04355	0.00650	0.22257	0.02710	0.00836	0.00824	0.00000	0.00718
**Fermt2**	2.96365	0.01042	2.64140	4.14853	0.40325	2.27154	2.49704	8.01308	5.52304	9.09638	8.83869	7.63381	2.94968	17.61379	4.60447	0.39961
**Fermt3**	0.00442	0.00747	1.46600	0.06022	0.00567	0.01121	0.21971	0.55020	0.37000	1.09898	0.41365	0.14210	0.99312	0.02720	0.04159	0.64542
**Fes**	0.00799	0.00724	0.00254	0.00527	0.00919	0.12436	0.00620	1.00898	0.12962	0.42907	0.02879	0.05658	1.53469	0.01393	0.00000	0.00668
**Fetub**	1.15768	0.00627	0.00000	0.10242	0.00559	1.49732	0.00572	0.00000	1.30916	0.00000	0.69037	0.05779	0.43091	0.00687	0.19312	0.00501
**Fez1**	0.01031	0.00776	0.05058	0.00000	0.00822	0.00548	0.00698	0.00000	0.23056	5.20457	0.85521	1.83368	0.00507	2.25453	3.24772	0.00695
**Fez2**	2.30922	3.22111	4.11433	0.28955	5.38181	4.01477	0.49797	5.54311	1.30874	0.57191	2.71804	0.10013	4.32152	0.51881	1.26613	7.50251
**Ffar2**	0.01216	0.00715	0.01857	0.04666	1.19908	0.00713	0.00732	0.00889	0.02658	0.01427	0.02501	0.04562	0.01894	0.19724	0.01843	0.00733
**Ffar3**	0.00894	0.00747	3.60539	0.01056	0.00354	0.00911	0.00674	0.00911	0.01244	0.00364	0.00997	0.00529	0.45575	0.01066	0.02336	0.00580
**Fgd1**	2.39667	0.00381	0.01184	0.35426	0.01192	0.00380	1.62145	0.14406	0.17539	0.66609	0.00903	0.01934	0.00723	0.02085	0.46205	0.06371
**Fgd2**	0.01364	0.00818	0.01112	0.33056	0.00918	0.00687	0.00749	0.01256	0.00681	0.24918	0.01262	0.05372	0.01275	0.03161	0.00449	0.00736
**Fgd3**	0.01295	0.00808	0.02322	0.00692	0.01020	0.00673	0.00754	0.01272	0.01133	0.00000	0.07278	0.00684	0.29980	0.00661	0.35038	0.00751
**Fgd4**	0.02741	0.00784	0.10449	0.05064	0.01205	0.00728	0.00715	2.60062	0.04576	0.00000	1.11261	0.00000	0.13462	1.75860	0.14315	0.02172
**Fgd6**	2.28956	1.44132	0.19778	6.59847	0.18866	0.00567	1.80236	0.00433	2.57362	1.11768	0.06795	0.00882	1.20158	0.00000	3.78612	0.44093
**Fgf1**	0.00506	0.00743	0.01126	4.75471	0.00845	0.00637	0.00671	0.95984	4.07694	3.59577	0.00000	0.08304	0.92143	0.00000	1.13976	0.00580
**Fgf10**	8.10863	2.52230	1.08850	0.20524	5.00441	0.00780	6.03443	0.00000	0.19085	0.04024	0.02263	0.03666	0.01297	0.05476	0.51165	0.73381
**Fgf11**	0.59099	0.00825	0.00529	0.01747	0.03664	0.02027	0.00714	0.01088	0.01259	0.19310	0.01193	0.00870	0.02618	0.02992	0.01036	0.00700
**Fgf13**	0.06675	0.01776	0.53647	0.01556	0.00810	0.06024	0.04642	0.02576	0.02467	0.48211	0.06274	0.01486	0.06768	0.32508	0.04365	0.02087
**Fgf17**	0.01366	0.00823	0.01209	0.37229	0.00932	0.00661	0.00753	0.01395	0.55662	0.01237	0.00904	0.00747	0.01284	0.01180	0.00395	0.00733
**Fgf18**	0.01327	0.00791	0.01037	0.25627	0.00932	0.00677	0.23929	0.01167	0.17288	0.02696	0.01157	0.00785	0.01212	0.01113	0.00605	0.00735
**Fgf3**	0.00567	0.00801	0.00232	0.17670	0.00938	0.00766	0.00625	1.03643	3.25939	0.47916	3.84566	0.04403	0.43795	0.14122	0.13399	0.00621
**Fgf4**	7.05660	0.00000	0.00000	6.19990	0.00000	0.00286	0.00000	1.78964	2.99549	1.94550	7.00558	2.22769	0.00000	8.59771	4.20683	0.07470
**Fgf5**	0.01375	0.00791	0.01163	0.03723	0.00937	0.00652	0.00741	0.01175	0.01129	0.00862	0.01185	0.00715	0.01284	0.00875	0.59624	0.00735
**Fgf7**	0.01364	0.00816	0.01171	1.29775	0.00929	0.00651	0.00747	0.01307	0.78685	0.00848	0.02352	0.00697	0.01257	0.02986	0.00068	0.00716

**Fgf9**	0.01359	0.00791	0.01157	1.29697	0.00931	0.00648	0.00741	0.01236	0.42976	0.00989	0.02577	0.00718	0.03520	0.04122	0.05627	0.00710
**Fgfbp1**	0.01564	0.00854	0.16709	0.00986	0.02942	0.00880	0.00756	0.18096	0.01208	0.00019	0.03694	0.00785	1.18514	0.01659	0.00398	0.00674
**Fgfbp3**	0.01280	0.00812	0.30878	0.57265	0.00875	0.00616	0.00753	0.01332	0.05093	0.01388	0.01211	0.00820	0.00986	0.02845	0.00000	0.00716
**Fgfr1**	10.76459	0.61870	2.58819	3.39855	5.25728	6.71288	4.63834	6.51383	0.54362	9.30123	4.35509	0.69379	2.56352	0.77619	0.24341	6.53182
**Fgfr1op**	1.04199	4.78799	1.34562	5.63979	4.15012	2.50294	0.01184	0.41679	4.67264	6.05782	4.83709	11.18146	1.28369	5.68573	7.13635	2.59474
**Fgfr1op2**	4.39429	2.19020	2.76929	4.33688	8.72356	0.58181	0.63208	4.30298	1.90245	4.79872	4.47623	9.69460	4.22829	1.05872	4.19660	4.65565
**Fgfr2**	2.67500	4.60184	1.18229	0.69330	5.97121	6.45933	0.62133	2.19524	0.15450	6.25481	6.27634	1.90416	0.23069	8.10240	0.13347	4.28433
**Fgfr3**	0.41863	0.17467	0.20112	0.24414	0.83700	0.01186	1.04744	1.56436	1.12822	1.56566	0.28978	0.25038	0.38826	0.84135	1.21373	2.19534
**Fgfr4**	0.68831	1.69081	0.30634	0.05896	0.01435	1.44462	1.98191	0.83623	2.47254	3.05123	0.70329	0.19300	0.55858	0.00759	1.32367	0.00515
**Fgfrl1**	0.31802	0.00725	0.00000	5.61110	0.00702	0.63801	0.00680	0.01550	1.73546	0.00000	0.00000	0.03185	0.00678	0.25581	0.72520	0.22132
**Fgl1**	0.77523	0.08070	0.00416	0.33180	0.00630	0.00706	0.00673	0.01159	0.00687	0.03930	0.01477	0.00775	0.01139	0.01325	0.00393	0.00626
**Fgl2**	0.01341	0.00790	0.01191	0.00437	0.00912	0.00661	0.00734	0.01080	0.65359	0.56990	0.00749	0.00521	0.01208	0.01216	0.00724	0.00758
**Fgr**	0.00364	0.00717	0.00000	0.46003	0.00883	0.00600	0.00655	0.74873	1.88371	5.65889	3.10080	2.17045	0.00000	0.15265	6.03700	0.00675
**Fh1**	7.91409	4.82697	8.62839	0.45725	7.40487	7.66109	5.04137	4.11239	1.36522	0.73070	3.21910	0.09746	6.17837	0.03501	0.65430	5.63716
**Fhad1**	0.63793	0.00725	0.00378	0.09275	0.00652	0.00703	0.00673	0.01538	0.59779	0.42432	0.00860	0.00451	0.01183	0.00854	0.30149	0.00699
**Fhl1**	0.01220	0.00791	0.01123	0.00546	0.00867	0.00663	0.00724	0.01001	0.01109	2.88779	0.00953	0.04172	0.01730	0.03551	0.00000	0.01485
**Fhl2**	0.80312	0.99338	4.95077	0.40997	4.44347	3.54104	3.74936	0.04074	0.00000	0.00000	0.00633	0.03517	2.24125	0.02348	0.23286	5.30158
**Fhl3**	0.06839	0.00790	0.00329	2.54219	0.00770	0.00734	0.00643	0.00000	2.68122	0.00000	2.14702	0.06578	0.05681	0.05559	0.09343	0.00607
**Fhl4**	0.07494	0.00790	0.00636	0.35164	0.01533	0.00682	0.24486	0.01473	0.41210	0.00568	0.01055	0.00599	0.01768	0.00540	0.30350	0.00731
**Fhod1**	2.82170	0.55739	1.50099	0.64859	1.33401	4.28716	0.00337	0.00000	0.00000	0.22671	1.07339	0.04604	1.26351	0.00869	0.00099	8.74608
**Fhod3**	1.33954	0.00507	1.48091	22.03935	0.00281	0.00727	3.02036	0.23787	4.78427	5.53697	0.10090	0.97819	0.00606	0.14969	3.53619	0.00000
**Fibcd1**	0.01376	0.00782	0.01236	0.40998	0.00933	0.00654	0.00748	0.01331	0.00629	0.01055	0.01375	0.00746	0.01263	0.00627	0.13062	0.00732
**Fibp**	10.05940	6.98904	0.00000	0.19286	1.53636	4.14774	4.77409	5.32865	0.00548	1.46649	2.43200	0.12513	6.43599	0.00000	0.56961	0.01859
**Ficd**	1.42912	0.01421	1.81687	9.36507	0.00367	0.00347	0.02148	6.50669	5.55041	12.71316	7.30429	4.23845	1.84698	7.04189	17.28710	0.01508
**Fig4**	3.29713	3.13762	4.78109	0.19069	0.00000	3.41022	0.55374	11.67548	0.58358	13.42126	7.34028	0.73333	2.34221	3.62182	1.01764	0.03480
**Fignl1**	4.05010	6.10356	3.23015	4.19880	1.38671	0.44845	1.91980	3.36327	2.92160	5.26737	1.42642	11.04715	1.90656	11.23342	9.18488	0.33284
**Filip1l**	0.00178	0.00670	0.13716	0.71439	0.01131	0.00948	2.04860	0.38780	0.00000	0.09377	0.00000	0.47450	0.00626	0.03111	1.03191	0.01462
**Fip1l1**	7.93522	5.12663	5.53184	4.67332	0.47053	2.36414	3.43428	8.80469	7.06325	7.05841	5.32918	4.29300	4.99714	0.12158	1.46392	4.38556
**Firre**	0.12987	0.00695	0.70769	0.00000	0.01505	2.94962	1.74953	1.32094	0.00936	0.00000	1.06948	0.00000	1.03102	0.71622	0.10286	0.00496
**Fis1**	12.58317	6.66609	9.44338	2.35620	9.51885	1.01381	7.21764	9.94308	1.69761	8.36139	4.56546	1.03208	7.57286	0.00585	0.51279	6.60786
**Fitm2**	0.00603	0.00857	0.16246	0.00483	0.00923	0.00831	0.00765	0.37731	0.62168	0.13253	0.05020	0.00575	0.68862	0.30044	0.00000	0.00701
**Fiz1**	8.15266	0.00000	0.84131	15.23852	4.05936	7.26502	2.13142	3.91179	21.82480	20.72884	8.88570	4.86958	8.23911	4.96032	11.35964	3.88202
**Fjx1**	1.93837	0.54635	0.00170	0.22342	0.43333	0.00662	0.00555	0.00256	0.00744	0.01207	0.01328	0.00591	0.01074	0.00797	0.00000	0.00513
**Fkbp10**	0.00454	0.00705	0.00000	0.02969	0.00800	0.00735	0.01622	1.62887	0.00964	8.60283	3.79961	0.16662	0.00000	0.03347	1.24171	0.00665
**Fkbp11**	1.25841	0.01203	7.44369	0.25020	0.00000	0.01985	3.69063	10.04373	0.02138	1.65328	7.48837	0.09728	7.10623	0.43728	0.61168	2.85181
**Fkbp14**	0.01357	0.00783	0.01136	1.98141	0.00909	0.00671	0.00744	0.01177	0.00000	0.41060	0.02717	0.01944	0.01228	0.01121	0.10409	0.00706
**Fkbp15**	1.48982	5.10311	5.88128	1.86599	4.76616	0.00502	2.59525	1.47600	1.92207	0.58552	1.25568	0.10583	3.80217	0.57389	0.99041	1.36448
**Fkbp1a**	10.34071	4.47761	4.21581	11.60384	4.90696	2.32036	5.46150	5.16667	10.19602	6.53698	11.49984	1.42756	4.69467	5.75326	3.45220	4.29804
**Fkbp1b**	1.93940	0.00423	0.03322	2.74139	0.00648	0.00297	0.00436	0.00000	3.91836	2.04786	2.26280	4.25942	0.35289	0.00000	0.21420	0.00427
**Fkbp2**	4.63321	0.99015	4.46915	1.75487	0.20082	3.23417	2.28831	1.33633	2.20492	4.28739	6.33920	0.11986	5.93085	0.07231	1.31042	1.53027
**Fkbp3**	20.25417	12.52986	13.00010	1.06845	9.59126	10.16670	8.96671	10.50699	1.91908	12.99910	9.63383	7.99602	12.24217	1.68157	2.32758	9.97399
**Fkbp4**	28.75872	13.55923	29.02292	11.94944	14.74054	13.10911	17.03696	20.12772	15.40473	23.57301	18.00070	7.45533	23.87930	3.39373	8.95716	14.18304
**Fkbp5**	13.31390	4.59025	5.93081	5.87628	2.18983	1.17095	1.65735	2.04373	4.68905	3.58595	0.54309	7.13126	1.52561	3.59755	8.73459	0.01620
**Fkbp6**	1.34724	10.12323	15.97149	1.60009	0.01808	4.20662	7.00180	11.33524	6.76503	12.13050	9.42861	4.02242	12.41277	3.18462	8.43297	3.75199
**Fkbp8**	13.79818	0.64568	6.49655	2.75274	4.77273	8.28392	4.97409	12.36164	1.65967	7.11386	6.95196	0.48871	13.72630	0.11766	0.00000	13.75733
**Fkbp9**	5.24731	1.59382	1.82902	0.00000	1.68825	2.94695	2.11527	2.25141	0.00517	2.22061	1.24307	0.04705	1.90089	2.80083	0.71374	3.03768
**Fkbpl**	2.31554	0.00564	0.45734	1.07214	0.00733	0.00316	1.38787	1.25164	2.97761	1.41240	0.44214	0.07250	1.28300	0.00366	0.04478	2.90378
**Fkrp**	0.29611	0.07466	0.58962	0.26576	3.26428	0.00524	0.00628	1.02238	0.00342	0.11722	0.02367	0.00068	0.86561	0.00818	0.09585	0.01327
**Fktn**	0.36500	0.15101	0.18981	0.67166	0.04327	0.04944	0.16324	0.38765	0.38264	0.21081	0.28632	0.15476	0.30928	1.14587	0.30692	0.02845
**Flad1**	5.09300	5.15317	3.70628	2.67143	0.39335	0.00000	4.10004	2.85754	1.68700	0.95933	2.42168	0.40372	4.51409	0.88026	1.48412	3.54738
**Flcn**	1.16132	4.40218	4.78820	11.19819	4.62715	4.26488	1.72749	5.98894	9.12934	1.22757	3.29039	0.22051	4.69151	0.21683	5.56653	0.58651
**Flii**	6.28576	4.17255	2.44791	0.28634	0.46769	9.87248	3.74895	10.35062	0.21192	2.82861	1.87041	0.68407	4.61590	0.00704	0.00000	0.15847
**Flna**	0.00000	0.00684	0.00000	0.58110	2.06869	0.17644	0.00394	1.35350	7.31050	3.55481	2.30962	0.94752	5.63485	1.83972	0.81188	4.28808
**Flnb**	8.07301	9.22697	9.20921	4.47335	2.73110	11.40459	9.39400	13.01404	5.18106	5.90392	4.08534	9.69552	8.54104	5.02593	3.33104	9.60686
**Flot1**	2.23957	0.02449	0.00000	3.23539	7.41819	2.53475	0.00384	2.22212	1.96891	1.65937	0.97956	0.01318	1.92816	0.00000	0.14246	0.01024
**Flot2**	1.99792	0.01817	3.25828	0.31814	0.02380	5.00828	0.01279	3.18851	1.66468	2.02816	0.64562	0.06194	6.98866	0.04992	1.58344	3.44298
**Flrt1**	0.01467	0.03999	2.22107	0.21715	0.00499	0.03707	0.00729	0.01482	0.00959	0.02764	0.03979	0.00638	0.00592	0.01528	0.00000	0.01360
**Flrt2**	0.68964	0.00709	0.00428	0.22716	0.00655	0.00723	0.00698	0.00991	0.35507	0.01136	0.00944	0.04844	0.01214	0.03450	0.00156	0.00661
**Flrt3**	0.24332	0.00871	0.00593	0.31781	0.01691	0.00684	0.00730	0.01206	0.36554	0.00501	0.02164	0.00771	0.00868	0.01244	0.00401	0.86455
**Flt1**	0.01377	0.00803	0.01205	0.01062	0.00932	0.00667	0.00752	0.01365	0.01235	0.01500	0.01302	0.01842	0.01272	0.01231	0.01038	0.00742
**Flt3**	0.01358	0.00783	0.01156	0.12011	0.00915	0.00683	0.00754	0.01265	0.24103	0.25881	0.00972	0.00764	0.01284	0.01316	0.18775	0.00738
**Flt3l**	0.00352	0.00654	0.08883	2.46343	0.00871	0.00520	0.33630	1.98131	3.05971	2.33260	5.19549	0.12766	1.11653	0.12714	0.00000	0.00533

**Flywch1**	0.11750	0.01849	0.02525	0.10430	0.00841	0.01232	0.00746	0.03778	0.74889	0.26450	0.00684	0.04214	0.01105	0.01122	0.00607	0.00721
**Flywch2**	0.01255	0.00786	0.00880	1.16145	0.00899	0.00659	0.00704	0.01629	1.02576	0.89972	0.40695	0.00003	0.00796	0.00432	0.04129	0.00719
**Fmc1**	2.19251	0.00496	0.39691	0.74923	0.02177	1.03435	0.00473	0.07817	0.00000	1.53600	0.78638	0.13250	0.80159	0.28744	0.00000	0.83603
**Fmn1**	0.82267	0.00705	0.00292	0.41179	0.00619	0.00727	0.23477	0.00870	0.10956	0.45392	0.00887	0.03431	0.01058	0.18282	0.00444	0.00609
**Fmn2**	0.10314	0.00774	0.00000	4.02473	0.00910	0.00586	0.00682	0.00711	2.00014	0.33016	0.00000	0.00000	0.01313	3.18739	1.62266	0.00686
**Fmnl1**	0.01846	0.00828	0.01171	0.01607	0.01052	0.00679	0.00758	0.01414	0.21417	0.02819	0.01148	0.00832	0.01302	0.01240	0.00936	0.00746
**Fmnl2**	1.77375	2.42844	0.00000	3.01972	0.00591	6.83839	0.00468	1.20998	0.18544	0.00000	0.00000	1.09164	0.23639	3.42402	0.56183	0.01650
**Fmnl3**	0.60088	0.00638	1.64668	18.15119	0.00396	0.01432	0.00529	0.60403	12.79494	0.08054	0.46276	0.16586	1.07152	0.00000	2.70372	0.00204
**Fmo1**	1.22622	1.77523	3.50100	2.88773	0.35275	1.59304	2.71915	4.43120	4.46622	9.27721	0.78129	5.21321	2.18266	11.50553	8.26648	0.87034
**Fmo5**	0.01746	0.00809	0.01540	0.01117	0.01020	0.01237	0.00736	0.01009	0.03539	0.65115	0.04645	0.00695	0.02343	0.07127	0.02258	0.00713
**Fmr1**	0.65431	5.42883	5.84029	6.04489	3.60888	3.79030	1.22929	3.74100	14.26424	0.30904	2.50970	9.88470	1.72759	20.17668	5.72249	2.31289
**Fmr1nb**	4.20322	1.93551	8.01492	11.34332	1.52256	4.52780	0.00000	8.80377	0.00000	10.13697	11.12177	0.92723	11.55681	8.66451	6.97595	13.27375
**Fn1**	19.39125	1.58446	2.37418	1.01610	4.06681	0.00000	0.00000	4.16740	1.31560	4.11899	0.20086	1.93349	2.58802	0.01563	0.41669	11.06675
**Fnbp1**	3.23801	0.01053	2.93195	1.13226	7.10260	0.11357	1.45689	2.95160	3.00337	0.11516	1.81191	0.14590	1.75186	2.03312	1.60482	0.44232
**Fnbp1l**	0.00000	2.61494	0.03432	10.11841	0.05721	0.11402	2.57437	0.54904	2.86443	0.00000	2.45523	1.55704	2.06460	3.38545	0.93182	0.48230
**Fnbp4**	7.79218	4.86201	6.01175	10.04581	4.19195	4.77853	2.57159	8.58326	11.13208	11.59393	9.33585	15.07749	6.96284	5.89181	17.19143	3.38427
**Fndc10**	0.05084	0.00820	0.00741	1.61821	0.00814	0.03508	0.00760	0.01643	0.00000	0.57636	0.01279	0.03185	0.16671	0.00799	0.04941	0.88384
**Fndc3a**	3.95146	2.98256	0.65313	4.22608	7.24285	1.56197	3.36087	0.00000	2.99319	0.00000	0.00000	0.00000	0.31748	1.76737	1.72530	2.81645
**Fndc3b**	0.32564	2.92479	0.10868	2.82869	4.19379	0.00501	1.07263	0.03663	3.96376	0.25464	0.00875	0.30652	0.03113	2.31613	2.12824	0.01243
**Fndc4**	0.00940	0.00749	0.61249	0.16726	0.01886	0.00598	0.00718	0.77191	0.31394	0.10991	0.26568	0.01534	0.07204	0.00840	0.00000	0.00719
**Fndc5**	0.01181	0.00767	0.57814	0.00761	0.00794	0.00931	0.00718	0.01035	0.01174	0.92977	0.01088	0.01452	0.00936	0.01387	0.00152	0.00739
**Fndc7**	0.01355	0.00798	0.01212	0.00890	0.00916	0.00675	0.00745	0.01201	0.01204	0.41432	0.01213	0.00746	0.01231	0.01169	0.01033	0.00748
**Fndc8**	0.01236	0.00787	0.00872	0.45579	0.00905	0.00645	0.00722	0.01419	0.06620	0.89326	0.00762	0.00274	0.01045	0.47788	0.47851	0.00702
**Fnip1**	4.27514	0.22196	0.12843	6.97971	1.45729	0.30074	0.45879	0.37639	6.09725	0.49659	1.35506	6.61170	0.37751	6.74194	3.01190	0.00758
**Fnip2**	0.59372	2.27791	0.37008	1.57383	2.43487	0.07142	0.82059	0.45729	1.60835	1.94624	2.17281	0.06350	0.08253	0.84296	4.44570	0.01953
**Fnta**	8.42133	5.69073	5.05470	7.55130	6.53820	2.37471	3.33966	3.93373	3.17568	9.45848	7.92403	5.21006	6.42047	11.01397	2.78164	0.46392
**Fntb**	8.20498	0.00000	3.04048	2.21775	3.77099	3.74104	0.00000	2.83100	4.01380	6.45898	1.74321	0.11580	2.87780	0.10854	0.69705	1.75753
**Focad**	5.64313	0.10925	2.52390	0.75952	2.43402	0.00000	0.00000	2.55839	0.00000	1.94446	0.45651	0.01787	2.38584	0.00301	0.26605	4.38622
**Folr1**	14.34889	12.62329	28.61415	12.58249	4.62514	10.93068	16.39186	27.39269	18.95451	35.53499	24.84791	12.82216	28.62525	2.11517	18.08742	9.37540
**Folr2**	0.00000	0.00687	0.89087	0.83880	0.87398	0.08478	0.02612	1.03886	0.94651	0.88617	0.07599	0.00749	1.80425	3.54938	0.16268	0.01345
**Fopnl**	4.82570	6.98755	6.52295	3.11999	6.12057	0.26163	3.07694	4.14356	3.78918	9.39160	6.03685	1.00880	3.56212	4.39657	6.45108	3.28452
**Fos**	0.00000	3.14466	0.51439	0.12573	0.03023	0.00577	0.21229	0.01009	0.00935	0.04243	0.01068	0.03234	0.00724	0.01072	0.16625	5.76960
**Fosb**	0.01350	0.00811	0.01207	0.67042	0.00927	0.00658	0.00751	0.01381	0.39073	0.01231	0.01007	0.00759	0.01261	0.01321	0.00629	0.02920
**Fosl2**	0.01731	0.00787	0.03541	0.08969	0.01033	0.00824	0.00736	0.01160	0.01094	0.24153	0.01175	0.03687	0.01231	0.00549	0.26073	0.00757
**Foxa1**	1.24942	0.00000	0.13807	0.55584	0.01138	0.00237	0.00000	5.82828	0.04903	17.98126	12.63345	11.66245	0.00000	6.75598	8.87764	0.00285
**Foxa3**	0.01361	0.00823	0.01221	0.36650	0.00932	0.00658	0.00749	0.01344	0.06860	0.01087	0.01191	0.00832	0.01257	0.00787	0.09087	0.02191
**Foxc1**	0.78119	0.06968	0.53737	1.32711	0.00535	0.03182	0.03919	0.43946	0.73849	2.22115	0.32910	0.45631	0.51213	0.01114	0.00000	0.01226
**Foxc2**	0.01296	0.00751	0.00547	0.01914	0.00895	0.00693	0.00701	0.00000	0.01203	0.99061	0.36537	0.12832	0.00845	0.36042	0.22897	0.00718
**Foxe1**	0.00917	0.00775	0.02790	0.00687	0.00925	0.00614	0.00706	0.19613	0.01141	1.28622	0.01046	0.00394	0.51108	0.01330	0.00515	0.00721
**Foxf1**	0.01353	0.00795	0.00379	0.05459	0.00897	0.00737	0.00706	0.00000	0.01063	0.19217	1.23188	0.04705	0.00000	0.00437	0.66160	0.00708
**Foxf2**	0.75130	0.01993	0.00497	0.01888	0.01952	0.00743	0.03877	0.01183	0.01119	0.01405	0.01365	0.00748	0.04415	0.01213	0.01035	0.01482
**Foxg1**	0.01679	0.00809	0.01407	0.09874	0.00894	0.00648	0.00763	0.02226	0.07537	0.01013	0.06975	0.01834	0.01845	0.13923	0.02501	0.00734
**Foxh1**	6.02274	0.01916	0.88639	0.07953	3.98426	0.41607	0.33379	1.03885	0.00408	0.12157	0.03853	0.07119	0.25682	0.00600	0.07222	2.84136
**Foxi3**	0.01374	0.00821	0.01202	0.83467	0.00931	0.00658	0.00749	0.01366	0.20265	0.01371	0.01234	0.01872	0.01274	0.01097	0.00301	0.00727
**Foxj1**	0.01336	0.00789	0.01223	0.00775	0.00906	0.00651	0.00732	0.01051	0.01198	0.79288	0.01165	0.00563	0.01111	0.01169	0.00829	0.00734
**Foxj2**	0.01008	0.00765	0.01253	0.47214	0.00800	3.44671	0.00700	0.00000	1.19605	0.00000	0.29589	0.00299	0.00000	0.00317	0.55229	1.14174
**Foxj3**	1.98344	0.40383	3.53536	23.37398	0.95520	0.28504	2.34261	2.56988	26.32975	3.75777	3.02210	3.85136	1.38633	4.16157	14.53096	1.19301
**Foxk1**	0.60902	0.00726	0.40946	2.77430	0.01483	0.22441	0.55125	1.82980	0.85103	0.40845	0.00000	0.03725	0.11610	0.23496	0.64363	0.00671
**Foxk2**	1.62037	0.00405	3.73340	8.62313	3.04798	0.01217	0.04784	0.00000	2.81043	2.80747	0.32195	0.20600	0.07702	4.13294	15.02612	2.52012
**Foxl1**	0.03866	0.00790	0.04778	0.06012	0.02755	0.00616	0.00731	0.01113	0.20998	0.00736	0.26770	0.04792	0.04389	0.10205	0.05550	0.00711
**Foxm1**	3.60684	0.03201	1.01876	17.08004	2.27597	7.75811	1.59364	4.14536	16.39202	4.99648	0.99988	0.38282	0.70076	0.60864	6.25739	1.51752
**Foxn1**	0.01385	0.00798	0.01050	0.01025	0.00914	0.00675	0.00746	0.00851	0.01221	0.03880	0.21335	0.00676	0.01017	0.01076	0.00691	0.00760
**Foxn2**	1.71229	1.82004	0.59600	10.49043	3.11548	1.34240	2.10176	1.78126	8.23920	2.76699	5.04120	4.77784	2.53382	14.93488	6.61508	1.04033
**Foxn3**	0.00780	0.00782	0.54130	0.72979	0.01054	0.00715	0.00714	0.42147	1.39776	1.25071	0.12790	0.25465	0.00337	1.27057	1.67112	0.01423
**Foxo1**	1.63239	0.03027	4.78088	16.94613	7.49782	2.52731	5.65275	2.47141	8.78379	5.43064	4.56897	0.55980	2.79943	3.31325	7.49730	0.01937
**Foxo3**	0.52453	3.27283	0.00841	0.10500	0.00681	0.00666	0.00506	1.96670	0.01761	4.03490	1.57698	0.04776	0.97200	0.00413	0.16482	0.01890
**Foxo4**	0.03393	0.01795	0.01805	0.22133	0.01133	0.00607	1.27419	0.00954	0.38332	0.27781	0.01030	0.00493	0.01060	0.02848	0.02117	0.00736
**Foxo6**	0.01810	0.00798	0.01173	0.01779	0.01030	0.00660	0.00729	0.01103	0.01106	0.39563	0.01264	0.00647	0.02561	0.01131	0.00978	0.00779
**Foxp1**	0.01334	0.00791	0.01126	0.01692	0.00898	0.00671	0.00736	0.01081	0.08923	0.84092	0.01093	0.04775	0.01143	0.01191	0.00714	0.00738
**Foxp4**	0.65619	0.00627	0.14151	0.67250	0.02354	0.00663	0.29088	3.11048	0.00000	1.85741	0.30822	0.04257	0.34779	0.00599	0.00000	0.28882
**Foxq1**	0.02425	0.00836	0.01079	0.01791	0.01143	0.00650	0.00744	0.01214	0.01296	0.11208	0.01194	0.01772	0.00870	0.01034	0.01531	0.78957
**Foxr1**	4.31387	1.16764	1.02126	14.52155	4.43222	0.00257	2.60185	3.70656	31.18933	6.79857	4.11092	1.24155	1.30990	0.93159	3.08800	1.27128

**Foxred1**	5.53759	0.80386	1.08984	0.84766	2.17591	3.68593	1.77113	2.11049	0.94845	0.00000	2.01355	0.16583	2.84593	0.15373	3.14895	0.64207
**Foxred2**	0.94612	0.00556	0.00000	0.29421	0.01480	3.19746	1.71872	0.14325	0.02514	0.00000	0.68750	0.00881	0.03645	1.56040	0.04328	0.08632
**Fpgs**	8.98651	2.02702	2.09903	4.18408	0.01974	0.00000	0.14062	5.15472	1.39410	12.78479	1.80239	0.40297	3.18382	0.00000	3.78202	3.65235
**Fpgt**	1.42743	0.00631	0.43690	3.11646	0.01052	0.00592	0.00569	0.00000	0.21802	0.19931	0.70286	0.02412	0.00876	0.02172	2.86256	0.00550
**Fra10ac1**	5.97959	4.33364	1.10645	1.17108	4.44410	3.79541	0.00000	1.11445	0.00012	0.20749	2.67157	0.10148	0.47303	2.20265	1.29768	2.01166
**Frat1**	0.19750	0.00680	2.08097	0.33213	0.00401	0.28222	0.22676	0.41727	2.41053	0.91389	0.00000	0.01574	0.00000	0.00430	0.55088	0.02082
**Frat2**	1.69052	0.00442	0.00000	1.74840	0.05118	1.68214	0.00447	1.36455	0.48626	2.60326	0.37594	4.67997	1.49181	0.00374	1.98587	0.00386
**Frem2**	0.00617	2.43073	0.00563	0.34716	0.00784	0.00625	0.00636	0.01289	1.16294	0.53998	0.00000	0.12940	0.01089	0.00352	0.75935	0.00655
**Frg1**	6.51738	4.29338	8.98305	7.37930	3.70116	0.09808	5.94754	12.40691	14.15959	8.06007	6.87552	0.96756	7.35501	1.35486	8.71703	5.52619
**Frk**	0.00000	0.03310	0.00000	0.11043	0.01149	3.22480	0.00716	0.40184	0.00843	1.68716	0.25550	0.03777	1.20573	3.30540	0.00000	0.61341
**Frmd3**	0.02309	0.01740	0.02170	0.17439	0.00863	0.03730	0.00728	0.01528	0.02525	0.00364	0.00979	0.00610	0.02834	0.01968	0.68816	0.02904
**Frmd4a**	0.03978	0.01974	0.02185	0.22948	0.01146	0.00610	0.45598	0.02709	0.91122	0.54541	0.00519	0.03540	0.01070	0.03088	0.08581	0.00770
**Frmd4b**	0.58247	0.12886	10.01329	0.21954	0.03035	0.00701	2.41343	4.18178	0.55675	5.30514	0.33751	4.35614	6.37520	0.24072	5.05356	1.85064
**Frmd6**	2.79112	2.51971	3.29792	1.06246	0.36871	2.75225	3.06882	5.16489	0.56043	0.82726	1.08172	0.22951	0.00000	0.33859	0.07418	0.03069
**Frmd8**	10.06697	2.53980	9.78509	8.35020	0.49940	10.33244	2.18086	15.74880	4.86694	4.65623	9.53936	5.40168	11.05000	1.10882	2.76922	4.80366
**Frmpd1**	0.00000	0.00698	2.35204	1.38821	0.26397	0.82305	0.00377	3.96648	1.65047	4.97789	0.48909	0.27284	5.62322	0.11506	0.25486	0.82406
**Frmpd3**	0.01365	0.00799	0.01185	0.00663	0.00930	0.00663	0.00754	0.01370	0.79108	0.01092	0.00737	0.00677	0.01294	0.01609	0.01197	0.00738
**Frrs1**	6.35105	6.26598	10.38239	7.70195	1.05064	6.56956	9.18520	10.96063	9.93352	8.00264	8.66622	0.97770	10.51994	17.02518	11.19123	2.16199
**Frrs1l**	0.01689	0.00768	0.69122	0.08107	0.00780	0.00709	0.00730	0.01190	0.02208	0.07914	0.01082	0.00695	0.00881	0.01725	0.01478	0.00712
**Frs2**	1.04404	3.02071	0.63451	4.87687	1.77487	1.28247	0.84771	0.93375	2.69601	0.45720	1.73393	8.97761	1.27167	0.00000	1.90837	0.00202
**Fry**	1.14634	0.00802	0.11335	0.90335	0.01081	0.00812	0.00627	0.86482	1.05850	0.20979	0.00000	0.01356	1.90809	0.57543	0.58090	0.00543
**Fryl**	2.46873	0.00427	0.44299	3.48105	0.00284	0.00342	0.02473	1.05370	2.85590	0.20750	0.50268	1.81622	0.00000	4.41739	3.53322	1.32202
**Fsbp**	0.00600	0.00758	0.95781	0.00619	0.00722	0.00733	0.12174	0.42236	0.01035	1.11015	0.00605	0.00101	1.70744	0.01362	0.37502	0.00641
**Fscn1**	5.42086	0.02154	0.00000	5.36821	2.84945	0.00000	0.19024	0.09031	14.51398	7.06094	7.91686	0.76874	0.09194	0.00000	0.00000	4.13318
**Fscn2**	0.01375	0.00805	0.01226	0.06152	0.00931	0.00665	0.00753	0.01390	0.01182	0.01536	0.01380	0.00812	0.01268	0.01197	0.00960	0.00740
**Fsd1**	0.01074	0.00837	0.01004	5.38573	0.00948	0.00618	0.00747	0.12851	1.24980	0.00652	0.00000	0.01763	0.00816	0.00954	0.00780	0.00639
**Fsd1l**	0.06582	0.00768	0.41645	0.43065	0.04646	0.14206	0.03636	0.00000	0.01679	0.48341	1.47491	0.07418	0.52950	1.55806	0.30822	0.01359
**Fsd2**	0.01997	0.00756	0.61794	0.68617	0.00793	0.00545	0.00696	0.01192	0.78601	0.00000	0.00000	0.00935	0.00886	1.57376	0.00000	0.00687
**Fsip1**	0.40825	0.00894	0.00088	0.00702	0.01715	0.00797	0.00781	0.22979	0.01067	0.13368	0.00795	0.13849	0.00686	0.00809	0.56757	0.00724
**Fstl1**	0.01354	0.00791	0.00883	0.25736	0.00908	0.00708	0.00745	0.01263	0.00490	0.48606	0.00871	0.28603	0.01241	0.01225	0.00298	0.00737
**Fstl3**	0.00000	0.00844	0.58257	0.00449	0.00875	0.86525	0.08191	0.22464	0.02150	2.20970	0.00747	0.00000	3.01354	0.01253	0.00878	0.59770
**Fth1**	30.19265	8.34431	12.82661	22.73191	26.76484	8.98063	8.21312	5.26437	37.99140	34.27989	28.07510	3.19621	28.79996	5.04288	0.00000	16.06732
**Fthl17.ps1**	2.44463	0.00585	1.83643	1.64329	0.00945	0.18446	0.00549	1.42960	8.14095	1.70406	0.00000	0.04538	0.00000	0.31858	0.37717	0.27337
**Fthl17a**	0.01332	0.00802	0.22218	0.00492	0.00862	0.29324	0.00737	0.00189	0.01132	0.04685	0.51673	0.00535	0.00432	0.01144	0.00129	0.00688
**Fthl17b**	1.82023	0.00632	1.76902	0.96500	0.00969	0.20289	0.00591	1.38404	8.11842	1.35451	0.00000	0.04573	0.00000	0.23476	0.33645	0.19684
**Fthl17d**	1.16764	0.00695	1.76923	0.52413	0.00921	0.16610	0.00648	1.44248	7.12878	1.37896	0.00000	0.04794	0.00000	0.28790	0.39352	0.16245
**Fthl17e**	1.44967	0.00669	1.63998	3.14933	0.00956	0.18040	0.00620	1.81169	8.29729	2.01918	0.00000	0.03139	0.00000	0.58333	0.63922	0.31621
**Fthl17f**	2.10289	0.00574	3.12839	1.88610	0.04309	1.74158	0.00540	2.89385	11.80584	2.64914	0.00000	0.05970	0.00000	0.33720	0.77556	1.05831
**Ftl1**	58.32151	32.92937	53.85800	13.85057	34.28309	29.43612	33.10425	49.88310	14.71463	51.61982	38.78860	18.45228	55.25262	0.24809	3.23031	30.41021
**Fto**	1.96572	0.01907	2.85790	0.65719	0.02153	0.00959	1.37627	1.24604	0.94146	1.22428	0.00000	0.02315	0.86090	0.52270	0.41353	4.16470
**Ftsj1**	9.34526	1.71546	12.93739	3.14864	7.05611	3.34265	7.54826	10.93128	3.17026	9.60053	5.86266	0.97557	2.84617	4.18010	7.91425	2.11815
**Ftsj3**	13.21861	13.61589	9.49723	8.49807	8.84499	7.20963	6.25734	20.40687	5.61197	11.56871	11.93135	4.43684	9.09339	21.00131	14.28366	8.85324
**Ftx**	0.43524	0.01693	0.04618	0.01377	0.00711	0.00594	0.02684	0.00485	0.46905	0.00000	0.10230	0.01187	0.04713	0.18220	1.46476	0.53957
**Fubp1**	8.79095	13.42417	8.27053	7.68278	8.90012	0.85989	5.08567	12.37914	6.65145	8.18881	0.00001	7.76883	2.73888	4.87821	5.20333	5.10053
**Fubp3**	5.40256	5.79669	1.58204	2.39489	0.01788	2.83094	1.67653	4.63123	4.86101	0.67367	2.43838	4.98890	1.83558	4.33367	2.49922	2.75374
**Fuca1**	6.26763	0.00000	4.29624	2.00121	4.92971	1.88909	2.03269	0.23565	0.39190	2.62698	0.54485	0.09436	3.06828	0.00380	1.24509	10.15787
**Fuca2**	2.00646	0.00388	4.25153	0.29782	1.47530	0.10151	0.07306	1.25518	0.56440	0.39125	0.94143	0.02341	5.10583	1.43443	0.00000	6.08460
**Fuk**	1.63381	0.05468	0.65539	0.31948	0.01174	0.15319	0.00345	3.92885	1.84581	2.19916	1.65294	0.18850	3.24657	0.04678	0.08947	0.10710
**Fundc1**	6.68215	5.41387	7.88713	12.74907	8.41627	4.95040	2.32514	0.60539	3.86942	3.79547	0.00000	5.36383	3.48722	4.13273	1.29990	2.30213
**Fundc2**	1.06338	0.00698	0.00000	0.02867	0.00570	0.44655	0.00621	0.09003	1.24985	2.78697	1.13265	0.00000	0.68451	0.00415	1.02685	0.02766
**Fuom**	3.33585	0.08606	0.40728	0.13047	2.60634	0.00828	0.49229	0.00000	0.22242	0.54872	0.01058	0.03187	1.45409	0.01085	0.00000	0.17481
**Furin**	1.41960	0.47388	2.46020	1.55087	0.01750	1.46522	0.08655	2.59908	0.58440	1.96197	2.28494	0.02876	2.32072	0.40222	0.45105	1.11803
**Fus**	2.18176	0.02412	2.45204	5.39553	1.87078	0.89776	0.18287	0.00000	16.20415	0.06347	20.61338	0.48349	1.27731	2.69509	1.00624	1.43361
**Fut10**	0.20445	0.00776	0.35670	2.76775	0.01336	0.00843	0.00687	0.01346	0.11335	0.00437	0.00850	0.01366	0.00934	0.01793	1.17622	0.00659
**Fut11**	0.02710	0.00810	0.00501	0.96141	0.01047	0.07390	0.00744	0.01103	2.08902	0.35113	0.00000	0.02664	0.01178	0.00488	0.93963	0.05842
**Fut2**	1.27418	0.28061	0.00656	0.45953	0.00556	0.00545	0.00600	0.06637	0.31299	0.54026	0.00904	0.00339	0.17182	0.00424	0.36629	0.00574
**Fut4**	1.26524	0.00724	0.00000	0.10762	0.01656	0.89593	0.00579	0.00493	0.00980	0.01989	0.01130	0.00638	0.71726	0.00693	0.21498	0.00565
**Fut8**	3.00005	5.84785	1.53894	1.35908	0.01721	1.42654	0.14393	0.65201	0.07441	3.25247	0.81662	1.04968	2.05483	2.96000	2.09421	1.79886
**Fut9**	0.01354	0.00803	0.01123	0.00710	0.00917	0.00682	0.00752	0.01249	0.01116	0.29520	0.01015	0.03134	0.01276	0.00588	0.42730	0.00741
**Fuz**	0.01113	0.00842	0.35098	0.01059	0.00912	0.00563	0.00795	0.38768	0.01191	0.03439	0.00993	0.00777	0.07252	0.01307	0.01103	0.00721
**Fv1**	0.01352	0.00803	0.01167	0.43346	0.00927	0.00647	0.00750	0.01320	1.70733	0.00581	0.00000	0.00518	0.01302	0.00980	0.08415	0.00719
**Fxn**	0.96946	0.02839	1.09168	0.11691	0.00457	0.00539	0.01642	0.00000	2.36842	1.88491	1.40769	0.07195	0.18803	0.03188	0.67161	0.28700

**Fxr1**	17.32667	15.78905	8.45241	12.43360	7.62881	1.32561	10.15535	9.82779	19.52417	5.36389	7.60726	5.06610	8.56282	6.50146	8.20762	4.23238
**Fxr2**	6.58811	5.74038	9.58237	10.24266	5.02918	4.78321	3.50335	10.04032	8.26881	5.38869	5.02138	0.58351	9.64755	1.27445	6.78280	3.67368
**Fxyd3**	0.01355	0.00791	0.01216	0.00873	0.00914	0.00667	0.00738	0.01137	0.01205	0.45445	0.01241	0.00678	0.01214	0.01153	0.00990	0.00758
**Fxyd4**	0.51632	0.00546	0.70591	0.00368	0.00564	0.21002	0.76373	0.93074	0.01861	4.31026	4.58705	0.00906	1.14638	0.00880	0.00000	0.01983
**Fxyd5**	0.01614	0.00763	0.25713	0.00796	0.00871	0.00572	0.00719	0.01006	0.01188	0.74911	0.01157	0.01567	0.01034	0.01267	0.00466	0.00729
**Fxyd6**	0.00000	0.00762	0.03281	0.10588	0.03185	9.26987	0.00506	0.00468	0.00710	0.75322	0.00000	0.00927	2.58192	0.90073	0.04670	4.33471
**Fyb**	0.01605	0.00779	0.16532	0.24156	0.00903	0.00576	0.00725	0.01136	0.00756	0.19776	0.01353	0.00701	0.01080	0.01074	0.00436	0.00732
**Fyco1**	1.43129	2.09554	3.22403	10.81539	0.84559	0.02189	1.40245	0.70674	2.25000	2.19614	1.86426	0.33204	0.02816	3.06039	3.24858	2.45381
**Fyn**	0.00897	0.00766	0.00662	4.04214	0.01059	0.00678	0.00733	0.75920	1.98943	0.00000	0.09287	0.00000	0.07710	0.01875	1.26190	0.00682
**Fyttd1**	3.03614	0.00000	2.67635	8.95948	5.51218	6.05576	1.12300	1.53514	6.02157	7.69472	2.08678	8.61673	3.34253	5.18034	5.80244	0.05627
**Fzd2**	0.01326	0.00813	0.01192	0.00989	0.00933	0.00654	0.00761	0.01715	0.59450	0.30704	0.00868	0.00618	0.01196	0.01278	0.00609	0.00743
**Fzd3**	0.67091	0.00628	0.50786	5.52689	0.01439	0.00409	0.00464	1.97891	5.56043	2.40719	4.00144	0.13258	1.03372	0.30274	2.24365	0.02659
**Fzd4**	0.01284	0.00787	0.00343	3.13196	0.00889	0.00628	0.00687	0.00000	3.00768	0.34707	0.94260	0.00000	0.00427	0.00000	1.18812	0.00621
**Fzd5**	1.54196	1.24466	0.33519	3.73414	1.06816	0.77472	0.00566	0.63756	0.49816	0.07914	1.16062	0.03506	0.15101	0.27114	0.07718	0.01851
**Fzd6**	0.24167	0.00798	0.00134	0.00943	0.01596	0.00831	0.00691	0.51094	0.01153	0.22059	0.02487	0.01590	0.00000	0.01260	0.00413	1.42881
**Fzd7**	0.84853	0.00666	0.01260	2.96096	0.01446	0.00468	0.00675	0.00810	4.10343	0.00000	0.00000	0.03091	0.01778	0.00967	1.20741	0.00546
**Fzd8**	0.02203	0.00773	0.00670	0.01630	0.00849	0.00693	0.00717	0.47213	0.01171	0.15797	0.00803	0.01617	0.00563	0.00778	0.18264	0.00706
**Fzd9**	0.01163	0.00778	0.00761	0.98492	0.00937	0.00674	0.00690	0.01308	5.70204	0.00000	0.46700	0.04140	0.83035	0.00218	0.00000	0.01390
**Fzr1**	7.70671	2.28320	5.48911	1.40037	5.48329	5.81185	2.81869	4.38925	2.08252	4.91655	2.01123	0.15345	7.15802	0.03010	0.00000	4.39787
**G2e3**	0.00532	0.00658	0.43375	3.24809	0.00889	1.29048	0.00666	0.53472	11.76468	1.46928	1.21126	5.37039	0.00000	0.00000	5.42277	0.00482
**G3bp1**	25.22897	11.13574	17.92519	14.14944	19.66481	10.20762	14.13278	29.69035	17.96885	23.24044	18.29709	4.38627	24.94682	11.12119	15.06273	13.42471
**G3bp2**	20.97919	15.44451	21.88771	20.01393	17.90044	19.10960	16.40513	29.81194	20.12859	33.69302	26.83494	17.00896	32.78623	35.34228	28.80268	13.23992
**G6pc**	0.01376	0.00782	0.01176	0.20618	0.00933	0.00664	0.00751	0.01292	0.24066	0.01001	0.02188	0.00734	0.01254	0.01903	0.05897	0.00736
**G6pc3**	0.01561	0.00556	2.73933	0.21706	3.54582	0.00686	0.01768	4.56433	1.20882	5.88275	1.37219	0.22681	0.00000	0.08923	0.95295	6.10738
**G6pd2**	0.00663	0.23673	0.15499	0.00953	0.23032	0.23142	0.05041	0.09771	0.01191	0.00507	0.01193	0.00744	0.04375	0.05989	0.10740	0.07253
**G6pdx**	0.14774	5.60922	3.52316	11.84388	9.31038	6.28020	4.42808	3.25382	2.34954	0.36286	1.39985	0.12716	3.01132	2.35304	3.26795	3.32736
**Gaa**	12.44352	5.33056	20.36670	4.61077	7.48181	7.30644	11.08297	27.38754	1.34017	16.84722	17.44035	5.21412	19.52416	0.01152	1.10016	6.59495
**Gab1**	6.12918	5.38573	5.24328	0.00000	4.67965	1.03082	3.69942	5.67350	1.55453	4.09089	3.42294	7.22918	3.23017	5.34359	4.12229	2.72445
**Gab2**	0.55093	3.22634	2.17356	1.64860	0.00290	0.00822	4.05933	0.45837	1.50591	0.00000	0.01292	0.00860	0.57044	0.02253	0.16203	0.00373
**Gab3**	0.02042	0.00798	0.01070	0.32889	0.00886	0.01372	0.00761	0.01313	0.08823	0.27225	0.01109	0.00831	0.01522	0.04690	0.04072	0.00739
**Gabarap**	7.31469	5.57962	3.12495	10.08660	2.81481	5.77949	1.90550	5.91222	9.39358	12.79361	7.23369	4.20705	7.15003	0.84492	5.58100	3.26152
**Gabarapl1**	8.51497	0.15215	7.23393	0.24601	10.96927	5.08489	2.08665	0.27485	0.00000	2.99906	0.37702	0.00000	2.65600	0.42921	0.00000	0.00000
**Gabarapl2**	45.70508	23.56372	40.73470	17.33871	32.21975	22.95576	23.24157	40.18810	25.09320	41.57961	44.24605	22.09509	43.54083	25.82968	20.41980	25.33494
**Gabbr1**	1.10644	0.00676	0.28250	1.04760	0.09435	0.01032	0.07650	0.01673	0.81322	0.11945	0.00000	0.02501	0.01689	0.69048	0.00000	0.01316
**Gabpa**	9.37939	10.79143	9.69277	18.46685	14.30121	4.94845	5.25145	14.70169	20.72292	11.51904	12.52732	12.10294	7.73819	18.31541	22.46354	3.54052
**Gabpb1**	1.83091	0.41749	2.84439	10.23905	3.14018	0.95552	5.01958	3.18643	10.43386	4.32631	5.41089	3.55422	0.43360	17.67074	6.05842	1.38006
**Gabpb2**	2.73108	0.10449	2.57864	4.04987	0.27079	1.00750	1.01008	2.23530	7.31135	0.55750	2.83534	8.03338	1.09464	11.11990	11.21700	0.06403
**Gabra3**	0.02164	0.00787	0.01618	0.96220	0.00876	0.00634	0.00746	0.01327	0.00000	0.01796	0.01270	0.00761	0.01283	0.05046	0.04937	0.00733
**Gabrb3**	0.29829	0.00784	0.00086	0.01911	0.01778	0.00779	0.00695	0.00821	0.01272	0.00719	0.00854	0.00387	0.01931	1.06734	0.00521	0.00733
**Gabrd**	0.00014	0.00726	1.76334	0.06513	0.00775	0.01305	0.01614	2.13216	0.75565	0.00000	1.25240	0.14793	1.45463	0.14331	0.01192	0.00577
**Gabre**	0.01663	0.00817	0.07340	0.11026	0.00878	0.00721	0.00752	0.01407	0.55755	0.01234	0.00924	0.00745	0.01163	0.01243	0.00750	0.00730
**Gabrp**	0.00976	0.00815	0.01132	0.89948	0.00916	0.00658	0.00696	0.01207	0.00464	0.03836	0.01142	0.03261	0.92347	0.00678	0.09111	0.00679
**Gad2**	0.10742	0.01835	0.15318	0.44920	0.07413	0.02940	0.03868	0.08188	0.37063	0.08461	0.44800	0.07376	0.18104	0.39787	0.19354	0.01407
**Gadd45a**	0.09358	0.00742	0.01774	0.00504	0.00769	0.00604	0.00718	0.20870	0.31525	1.72227	0.00707	0.01254	0.00912	0.02616	0.07016	0.00702
**Gadd45b**	0.00023	0.02981	0.21681	0.94010	0.03066	2.63000	0.00520	4.18755	6.17611	1.07579	0.38531	0.02146	3.86675	0.01942	0.00000	0.70663
**Gadd45g**	0.00000	0.00479	0.00000	0.00000	4.18292	1.46036	0.02702	2.79294	0.27589	2.76317	0.53594	0.00000	0.00000	0.01141	0.00000	0.01447
**Gadd45gip1**	2.98156	0.05404	0.96094	9.75128	0.91609	0.66324	0.32861	0.71265	13.08493	17.86281	20.26769	0.43745	6.45459	0.29080	0.33810	6.00648
**Gak**	4.21117	4.97375	6.31400	6.79308	7.72624	8.07522	5.09039	6.82479	9.90578	6.57509	6.84946	1.18307	9.61037	1.57116	3.15134	7.16503
**Gal**	0.01365	0.00827	0.01212	0.76540	0.00921	0.00681	0.00757	0.01396	0.00000	0.14740	0.01299	0.00914	0.01296	0.01026	0.00530	0.00729
**Gal3st1**	0.01263	0.00819	0.01115	0.21870	0.01902	0.00665	0.00767	0.01967	0.00546	0.05086	0.01163	0.06029	0.01232	0.00921	0.04455	0.00742
**Gal3st2**	0.02098	0.00800	1.37813	7.69676	0.00687	0.00890	0.01917	1.00608	2.06637	0.00168	0.01308	0.02275	0.35388	0.00052	0.53880	0.00547
**Gal3st3**	0.17988	0.03902	0.48573	0.54452	0.54095	0.04587	0.02788	0.03658	0.47780	0.30145	0.46830	0.07732	0.13712	0.46283	0.23602	0.08501
**Galc**	0.01264	0.00768	0.00607	0.83860	0.00879	0.00693	0.01742	0.00000	2.28020	1.00912	0.60572	0.00000	0.00726	0.24005	0.03994	0.00721
**Gale**	7.53649	1.77332	3.45994	2.02049	3.74038	2.48426	3.75523	2.42072	0.41608	1.54992	1.80178	0.11865	3.40475	0.00000	0.00000	0.01634
**Galk1**	10.92700	0.54630	4.88484	0.42991	12.22524	7.06323	5.55100	19.32460	0.00000	22.47617	19.23863	2.81980	26.43894	0.00000	0.00000	13.41054
**Galk2**	3.06742	1.07572	0.89997	0.30933	1.51766	5.11309	1.97083	2.11132	0.00187	2.53951	1.76662	0.10876	2.72107	0.02561	2.48766	3.28645
**Galm**	2.86490	0.17523	0.02136	0.43133	0.39506	0.00298	5.51936	0.28734	2.07507	3.87735	0.38189	0.00000	0.02303	0.47490	1.03189	0.00404
**Galns**	0.01214	0.00760	0.01004	2.39334	0.01869	3.46103	0.00701	0.03723	0.16403	0.00367	0.00755	0.02683	0.00000	0.00206	0.31394	0.00555
**Galnt1**	5.85938	4.56373	5.37007	3.70983	5.17761	4.29009	5.45994	2.04965	0.20968	4.36711	1.04957	7.34878	2.00029	3.92217	4.93538	4.54711
**Galnt10**	0.01324	0.00804	0.01211	0.27177	0.00896	0.00647	0.00737	0.01078	0.00738	0.71425	0.01178	0.01607	0.01062	0.00989	0.00816	0.02808
**Galnt11**	2.83357	0.00000	2.96569	3.32875	3.75692	1.42407	2.41128	6.34021	4.51534	2.88091	3.50648	0.70325	0.03924	4.47832	6.05720	2.60961
**Galnt12**	0.01371	0.00761	0.01097	1.13428	0.00925	0.00648	0.00731	0.01111	0.47130	0.08523	0.00762	0.03323	0.01321	0.00559	0.49623	0.00752

**Galnt16**	0.00000	0.00824	1.20878	0.28396	0.00738	0.00557	0.00712	0.62982	0.27716	2.13335	0.00000	0.03849	0.91363	1.43887	1.06985	0.00627
**Galnt2**	1.15406	2.15157	3.13329	0.52016	0.00002	3.39305	4.81740	5.46217	0.92152	4.94787	2.17973	0.11236	0.55163	0.37265	0.97544	3.09182
**Galnt3**	0.01026	0.00737	0.22107	0.41014	0.03026	0.00738	0.54858	0.01184	0.00348	0.02217	0.01119	0.04234	0.07847	0.00605	0.60798	0.00749
**Galnt4**	0.04802	0.00791	0.07383	1.12623	0.01260	0.08867	0.00710	0.00929	0.49968	0.00362	0.00693	0.00412	0.02350	0.00587	1.31364	0.01434
**Galnt6**	4.39704	0.00201	2.33473	0.57945	0.26251	4.30418	0.00000	2.13167	2.44028	4.00374	2.24248	0.01988	5.08842	0.02435	0.85995	0.84611
**Galnt7**	1.38590	0.00591	0.00288	0.00872	0.00529	0.00669	0.00638	0.00743	0.01198	0.01059	0.01190	0.00598	0.01154	0.00608	0.31253	0.00593
**Galnt9**	0.00644	0.00854	0.00693	0.00434	0.01855	0.00695	0.00824	0.40620	0.33877	1.65852	0.10672	0.02411	0.00534	0.31609	0.00000	0.00714
**Galntl5**	0.00838	0.00787	0.52305	1.47754	0.00898	0.00542	0.00708	0.15618	0.68562	0.00000	0.51638	0.00245	0.02259	0.26557	0.18723	0.00663
**Galntl6**	0.03078	0.01852	0.11369	0.18799	0.01051	0.01236	0.03799	0.01269	0.09773	0.04958	0.12312	0.02538	0.04005	0.21246	0.00735	0.01486
**Galt**	5.08248	0.86731	4.42969	1.06734	3.39516	3.57695	1.30769	1.35123	4.01885	3.55370	1.70479	1.69553	2.47919	0.03151	0.19041	2.15879
**Gamt**	0.80467	0.01650	0.80981	0.00486	0.00621	0.00801	0.00562	0.26111	0.25275	1.56667	2.43865	0.01625	0.18438	0.00362	0.23336	0.00594
**Gan**	1.30772	0.00712	0.25758	1.68630	0.01528	0.00396	0.00612	0.00000	2.43081	0.74959	0.52230	0.16093	0.06273	1.75276	0.00000	0.00544
**Ganab**	10.34328	0.01812	8.65299	2.50286	5.06955	7.31463	8.12878	12.09552	3.01953	6.22348	3.46890	0.60335	4.80932	5.39504	9.59146	4.23644
**Ganc**	0.00947	0.00727	0.97340	0.13665	0.00742	0.00734	0.00695	0.19502	0.39718	1.30580	0.00705	0.01239	0.00986	0.02851	0.02841	0.02837
**Gapdh**	40.96248	21.49199	28.30940	11.72569	28.67127	19.55495	17.02734	4.77568	15.77732	8.01435	2.16543	5.14605	31.19249	0.07348	4.30225	27.60821
**Gapdhs**	0.00783	0.00772	0.27724	0.00913	0.53068	0.00611	0.01863	0.34854	0.01161	0.25222	0.01058	0.00643	0.02572	0.01188	0.00817	0.41981
**Gapt**	0.00584	0.00584	0.00513	1.29220	0.00886	0.00495	0.00680	0.17796	0.68227	0.00872	0.00000	7.23518	0.00722	0.23103	0.69533	0.00641
**Gapvd1**	1.47434	2.72486	2.97602	5.54533	2.44936	3.34494	1.85749	7.19759	6.86432	7.02509	2.42284	0.69084	2.09740	7.55684	10.01910	1.61440
**Gar1**	4.50684	1.80017	4.35181	1.60142	7.42890	2.91128	3.49468	3.93235	4.24290	2.13258	6.42172	0.62418	3.93530	1.66654	4.50501	1.47069
**Garem**	0.00700	0.00803	0.09515	0.01131	0.01053	0.00605	0.00788	1.66072	2.93419	0.00000	0.26589	0.00737	0.29386	0.00331	0.35091	0.00724
**Gareml**	0.01103	0.00872	0.01794	0.00980	0.00946	0.00627	0.00821	0.19243	0.01179	0.10037	0.00945	0.09246	0.00685	0.01063	0.09283	0.00717
**Garnl3**	0.06176	0.00890	0.89118	1.45793	0.01579	0.00947	0.00775	0.04507	0.20072	1.06774	0.00076	0.34237	0.08844	0.00349	1.30527	0.00721
**Gars**	19.43104	14.44560	17.82430	4.86069	18.39616	8.93123	11.74680	16.29258	5.63364	13.71557	10.54701	4.09723	13.47779	14.69442	8.54337	9.87255
**Gart**	11.11282	12.15411	14.96371	5.93087	9.69819	4.22416	12.72264	19.68906	4.56912	13.95142	10.72175	1.31742	11.20306	0.44915	10.70533	10.51645
**Gas1**	0.01336	0.00784	0.01156	0.00672	0.00895	0.00669	0.00730	0.00861	0.01158	0.87490	0.07487	0.00548	0.01112	0.01164	0.00774	0.00760
**Gas2**	0.12326	0.00703	0.30901	0.29347	0.01549	0.01786	0.01692	0.00000	0.82226	2.26453	0.35209	0.01776	0.00989	0.01068	0.00000	0.01393
**Gas2l1**	5.29528	0.00118	0.18431	4.60332	0.00000	2.27865	0.00309	2.38937	6.92713	2.83693	0.00000	0.05886	0.00000	0.00974	0.26525	0.07680
**Gas2l3**	1.77989	1.05776	2.58839	10.26422	0.99491	0.55192	0.42608	0.00000	12.94771	2.23978	13.31570	1.06125	2.36462	4.98361	2.69077	0.42075
**Gas5**	13.54321	1.70742	15.27607	10.86344	13.21060	7.11134	9.49267	11.90780	22.22504	17.43886	30.52905	14.30255	11.42451	11.07703	10.39365	6.13315
**Gas6**	0.00984	0.00756	0.01353	0.07670	0.00914	0.01433	0.00698	0.08851	0.07696	0.90701	0.27054	0.01295	0.14620	0.01000	0.00000	0.00740
**Gas8**	0.57916	0.00773	1.33375	0.06458	0.01888	0.00501	0.00573	0.87042	0.52364	0.86476	1.76678	0.01432	1.17897	0.48252	0.57781	2.94440
**Gata1**	0.00000	0.00444	3.91009	12.28659	0.00950	4.52613	0.00232	13.94069	16.34015	16.16546	10.18901	0.58593	0.00000	1.02607	7.79336	0.00000
**Gata2**	0.01331	0.00799	0.01585	2.27700	0.21208	4.52640	0.00693	0.00906	0.52299	1.51288	0.00429	0.01411	0.01467	0.00157	0.18352	0.53688
**Gata3**	0.00000	4.40789	2.87154	0.00000	0.10077	6.80107	0.40428	2.18096	0.04808	1.44327	0.34463	0.00000	10.90905	1.08873	0.00000	1.86139
**Gata4**	4.90511	3.43219	0.00000	16.21447	8.44153	0.00000	0.00000	0.25767	12.22178	12.67000	3.46490	0.87378	0.00000	4.87330	19.08872	9.81466
**Gata6**	14.50544	17.49312	12.12391	0.20352	14.18921	8.56907	15.43088	22.77773	0.04134	28.83693	16.72868	9.23258	11.32362	7.93634	3.63183	13.88427
**Gatad1**	4.40240	4.67197	1.19471	0.36991	0.00713	0.78098	1.01247	0.78091	0.00339	0.53924	0.11392	0.02379	2.26518	0.73135	0.31027	2.14472
**Gatad2a**	11.09083	3.76503	6.50413	21.58237	8.32603	10.39536	7.06735	16.73889	19.28870	20.16802	8.77676	6.82123	14.72017	2.23060	5.60244	9.30251
**Gatad2b**	0.00704	0.00616	1.02284	0.64408	0.00954	0.00553	2.76901	0.86827	0.65999	2.23699	0.19084	0.10801	0.00000	3.89848	3.76993	0.00658
**Gatb**	9.37950	1.06136	2.84590	0.52198	1.20766	3.74558	2.62012	0.00000	0.01975	0.72594	0.02448	0.07469	1.94918	0.00436	1.96659	2.59053
**Gatc**	3.68658	0.83458	0.00000	0.27288	0.02868	2.38698	0.00289	1.17966	1.70910	2.70071	0.59277	1.35002	1.48693	0.67545	0.84071	0.13741
**Gatm**	0.01280	0.00778	0.00988	0.00473	0.00877	0.00676	0.00740	0.00604	0.01023	1.71665	0.19209	0.00252	0.00991	0.02067	0.38984	0.00735
**Gatsl2**	0.01350	0.00798	0.01220	0.46010	0.00907	0.00646	0.00740	0.01108	0.15636	0.52880	0.01041	0.00616	0.01094	0.01058	0.01794	0.00703
**Gatsl3**	4.87683	3.17425	14.22812	3.14918	8.29617	6.54725	12.58746	11.93277	6.22790	10.91321	7.22894	2.63229	10.66528	0.01024	3.37995	4.43390
**Gba**	11.41842	7.26616	18.97178	0.00000	0.95679	2.05384	8.69855	12.37506	2.19652	8.86438	10.84798	7.94209	12.31486	5.09539	6.48837	1.16966
**Gba2**	0.01739	0.00791	1.00632	0.92975	0.02093	4.56681	0.00681	1.07702	2.09730	0.07224	0.34508	0.81547	0.72785	0.00182	0.06878	0.01958
**Gbas**	1.12241	0.03602	0.00000	9.45906	1.87016	2.89330	0.00517	0.00000	4.56610	0.65027	1.15280	0.03404	0.63970	0.75592	1.71628	0.01668
**Gbe1**	0.00826	0.00752	2.60557	0.00492	0.02759	0.19121	0.00671	0.25005	0.01080	0.78894	0.35185	0.02934	0.14059	0.05206	0.00000	0.01437
**Gbf1**	2.71172	0.04042	1.47888	7.94096	0.02599	0.01519	0.01389	4.70116	3.40190	2.61213	0.50004	0.22207	1.58799	0.00000	0.55754	4.28042
**Gbp11**	0.01685	0.00818	0.01120	0.00849	0.01817	0.00667	0.00767	0.01663	0.01213	0.07854	0.07235	0.00794	0.01101	0.01161	0.00909	0.00725
**Gbp4**	0.58757	0.00750	0.00000	0.00702	0.00654	0.00812	0.00756	0.46299	0.01112	0.87231	0.01345	0.00012	0.01374	1.06144	0.00000	0.00666
**Gbp7**	0.01372	0.00760	0.00976	0.00331	0.00948	0.01266	0.00730	0.01070	0.18227	0.09004	0.00774	0.04316	0.01289	0.01036	0.97658	0.00747
**Gbp9**	0.00929	0.00846	0.00749	0.02106	0.00950	0.00689	0.00821	0.39807	0.01273	0.30645	0.06721	0.00620	0.00558	0.28304	0.03616	0.00716
**Gbx1**	0.01382	0.00822	0.00884	0.06223	0.00910	0.00686	0.00760	0.00619	0.00868	0.00624	0.48134	0.00646	0.00754	0.01026	0.00000	0.00715
**Gbx2**	0.03661	0.00831	0.00706	4.77495	0.01470	0.00656	0.00721	0.00887	4.40506	0.00000	0.00000	0.02377	0.13936	0.69141	0.00000	0.00653
**Gc**	0.01490	0.00710	0.01278	0.45039	0.00909	0.00637	0.06586	0.00000	0.00819	0.00455	4.07789	0.00581	0.00000	0.45110	0.00000	0.00655
**Gca**	3.10243	5.72075	2.94827	0.93703	0.11102	4.53454	4.21600	4.67219	2.25158	0.66860	0.15740	8.12648	3.58049	8.33856	6.32536	1.54798
**Gcat**	15.55010	2.74371	2.36363	0.26199	5.21726	0.23944	4.22283	4.70325	0.00019	2.04086	4.23004	0.04786	8.33222	0.00000	0.00000	2.18525
**Gcc1**	0.69394	0.00475	0.00000	0.65793	2.09672	0.00567	0.00587	0.84285	0.18178	0.23896	1.22966	3.21881	0.00582	0.00282	0.63645	0.35786
**Gcc2**	0.90273	5.01783	2.23743	3.08571	0.04648	0.55878	0.00239	1.22271	0.93897	4.32018	3.24116	5.38519	1.08994	0.00000	3.46899	0.08011
**Gcdh**	2.37092	4.33580	3.30523	1.68834	4.44917	4.14398	3.71798	7.61752	3.88937	6.25590	7.11395	2.37792	8.39877	1.28492	7.19368	0.02484
**Gcfc2**	0.40217	0.00783	0.01191	0.00736	0.00705	0.00598	0.69967	0.14615	0.01155	0.41244	0.03068	0.00000	0.00812	1.98454	0.00000	0.00683

Gch1	2.77666	0.07057	3.86443	8.12636	1.64238	4.81209	3.51494	4.99823	8.22973	11.23303	13.13348	2.33736	3.02770	4.53080	1.10879	0.95581
Gchfr	0.27553	0.00713	0.00000	0.07373	0.01500	1.07491	0.00707	0.02957	0.47714	0.00751	0.00939	0.00655	0.00979	0.00835	0.10181	1.81472
Gck	0.01383	0.00792	0.01147	0.40017	0.00933	0.00660	0.00743	0.01258	0.00574	0.00984	0.01323	0.03912	0.01276	0.01035	0.11450	0.00733
Gckr	0.01373	0.00814	0.01197	1.47531	0.00931	0.00651	0.00748	0.01338	0.24970	0.01229	0.02768	0.00781	0.01236	0.01218	0.00473	0.00714
Gclc	1.13174	0.14248	1.72608	5.13011	0.00475	0.00525	6.43018	3.79332	4.39315	1.88568	0.70505	9.32645	0.00000	0.32508	2.89761	1.70337
Gclm	1.09970	4.01089	2.27471	2.44292	7.28002	4.57172	3.17269	0.57950	3.87139	4.46097	0.61143	2.25859	3.60256	1.41477	2.70778	0.01428
Gcm1	0.01279	0.00753	0.00633	0.00000	0.00940	0.02990	0.00715	0.01018	0.00907	0.00000	0.00422	0.00188	0.11943	0.39416	2.01590	0.00719
Gcm2	0.01346	0.00819	0.01147	2.44757	0.00926	0.00627	0.00742	0.01301	2.19558	0.00512	0.00000	0.01570	0.01300	0.00639	0.05346	0.00681
Gcn1l1	8.20322	0.23074	0.91668	0.41444	0.16956	0.20626	3.85966	15.03052	1.10822	7.93665	0.56989	0.33544	9.56144	0.00000	0.00000	3.11800
Gcnt1	0.00000	0.09859	7.91364	1.19547	2.27575	6.86894	0.02284	4.50105	0.00001	0.66346	4.85705	0.67918	4.22057	5.57677	0.54308	3.98992
Gcnt2	0.31373	3.87592	0.00000	5.02114	0.01518	0.00525	0.01509	0.42321	3.65662	2.04021	0.03549	0.00434	0.00588	5.02387	2.72763	0.00420
Gcnt3	0.01329	0.00782	0.01182	0.00723	0.00897	0.00670	0.00732	0.01014	0.01175	0.88487	0.02794	0.00565	0.01161	0.02968	0.00523	0.00764
Gcnt4	0.01381	0.00758	0.00831	0.70472	0.00926	0.00677	0.00719	0.01034	0.60058	0.00438	0.00543	0.45127	0.01345	0.01041	0.00000	0.01483
Gcnt7	0.01373	0.00817	0.01196	0.10689	0.00933	0.00666	0.00749	0.01315	0.00938	0.01390	0.01323	0.00783	0.01280	0.05359	0.00682	0.00742
Gcsh	18.37219	4.74923	15.72874	20.67094	14.30275	9.31566	6.21174	34.83236	30.52631	53.55512	38.24339	26.20241	27.60095	28.49156	19.59671	12.34094
Gda	0.01708	0.00750	0.01549	7.58264	0.01721	0.00617	0.00666	0.00000	1.10781	2.93391	0.85120	0.00000	0.02046	0.00987	4.86318	0.00594
Gdap1	0.00000	0.00779	0.00101	12.72100	0.00919	0.00366	0.00640	0.00000	9.13161	3.34593	0.18775	0.00000	0.00932	11.22199	13.97716	0.00409
Gdap10	0.00000	0.00682	0.01296	0.00876	0.01040	0.00727	2.01908	0.00749	0.01176	0.00000	0.01351	0.00609	1.33379	0.01074	0.15418	0.02075
Gdap2	2.16025	10.62354	4.58876	14.25156	0.08468	12.23427	3.46829	19.85329	17.30398	21.80274	15.66561	15.50179	4.69654	22.85289	20.21738	0.10916
Gde1	14.00326	6.13143	8.10999	17.55566	2.40131	3.43066	6.10761	15.17178	14.89174	17.74517	12.94880	5.81917	7.24724	5.19560	5.80771	3.28398
Gdf11	0.01554	0.00811	0.10566	0.04042	0.00937	0.00638	0.01842	0.01307	0.01157	0.94906	0.01492	0.00589	0.11446	0.01789	0.06993	0.00737
Gdf15	0.05366	0.00811	0.00997	0.74044	0.00801	0.00669	0.00744	0.01503	0.08121	0.01308	0.01297	0.00823	0.01186	0.01103	0.00405	0.10732
Gdf3	15.19883	0.97025	6.85161	0.99463	6.52686	0.04947	4.89190	7.22856	0.70136	2.54644	1.74385	0.18883	6.41351	0.82598	0.54831	5.13938
Gdf5	0.01372	0.00806	0.01229	0.29909	0.00927	0.00656	0.00752	0.01294	0.00896	0.07924	0.01128	0.00756	0.01216	0.00562	0.17788	0.00718
Gdf9	0.77431	0.13792	0.49354	19.04854	0.85457	0.00242	0.04775	0.05536	12.06746	3.64316	2.30348	0.36487	0.39343	0.05570	6.16140	0.00210
Gdi1	1.43083	0.00659	0.00003	1.45202	0.00372	1.37628	0.01598	0.16783	1.81564	2.30801	0.01448	0.06736	1.92177	0.24021	3.83025	4.40953
Gdi2	20.48264	19.18741	16.77204	18.79670	19.41438	17.09641	14.19514	27.78715	21.17761	33.02070	20.17967	26.85192	24.31424	29.14062	20.49689	12.15482
Gdpd1	1.09329	0.00437	1.30202	1.65331	0.00346	0.25471	2.67957	0.00000	1.65821	1.91332	0.22570	0.15342	0.00467	0.00588	1.92173	0.00500
Gdpd2	0.01334	0.00813	0.01192	0.00672	0.00903	0.00660	0.00734	0.01071	0.01133	0.72862	0.01136	0.00598	0.01160	0.01113	0.21893	0.00752
Gdpd3	0.02052	0.00771	0.01127	0.00767	0.00886	0.00665	0.00753	0.01294	0.55853	0.00874	0.00874	0.00685	0.01289	0.01977	0.06335	0.00736
Gdpd5	2.47529	0.00552	0.00000	0.00834	0.02355	3.63991	0.00505	0.00780	0.01299	0.04925	0.00883	0.00528	0.00336	0.01042	0.01561	4.34965
Gdpgp1	1.76449	0.00554	0.31681	0.36252	0.03156	0.02990	0.03735	1.45581	1.20948	0.70015	0.83885	0.14079	0.08165	0.24732	1.61795	1.11977
Gemin2	4.50537	3.46081	5.50610	0.96246	4.14062	4.59532	2.56394	9.05473	4.46661	14.89248	11.81445	1.96266	9.29319	3.27112	8.73103	5.26307
Gemin4	8.91698	8.89267	6.64430	2.73487	0.00000	0.21547	6.82532	4.82130	2.92763	10.30452	3.74760	0.90207	3.95034	3.09676	10.93212	0.89368
Gemin5	10.68331	11.88416	14.98607	7.12722	10.31123	2.88324	7.25680	21.87873	13.14567	24.20672	15.83669	7.75802	6.30497	15.57752	22.63698	4.08647
Gemin6	5.55626	5.11195	4.91708	1.52698	3.92742	4.04981	0.69924	5.82383	1.52296	1.40905	0.00000	1.93787	1.48546	3.74583	2.51364	0.03154
Gemin7	3.92480	0.00153	3.79002	9.21783	0.36531	0.02069	1.92813	0.58071	4.99466	5.14208	3.58490	0.27696	2.13190	0.00000	0.55226	3.81822
Gemin8	2.33574	0.02397	4.08596	1.09426	4.96116	0.00944	0.07043	2.10441	1.86194	6.24044	1.68575	0.34397	0.78468	1.18742	3.67062	0.05337
Gen1	2.24117	0.72462	0.48751	0.62399	0.01341	1.08464	0.62006	0.06485	0.31666	0.64830	0.00000	0.14312	0.00287	3.25003	1.17552	0.00399
Get4	5.78760	0.57620	4.70856	2.10467	5.42008	7.71622	5.62723	9.09970	0.67377	12.67619	2.75726	0.57197	11.10572	0.00000	0.00000	3.33726
Gfer	3.23349	1.80456	3.58747	12.72328	5.55703	0.00154	2.74987	1.95785	10.31831	5.83247	8.43536	0.11218	2.12919	0.20356	2.27859	4.24798
Gfi1	0.01362	0.00802	0.01132	0.02651	0.00929	0.00655	0.00740	0.01200	1.17384	0.02208	0.00006	0.02618	0.01350	0.00578	0.27593	0.00758
Gfi1b	0.01877	0.00752	0.00959	0.01074	0.00880	0.00671	0.00722	0.10028	0.01206	0.04063	0.01308	0.01791	0.00837	0.01971	0.01169	0.00720
Gfm1	7.01215	4.91605	3.52818	1.34458	5.09971	0.15281	4.18846	4.61793	3.13159	0.00000	3.84093	3.34104	3.81094	4.05489	8.20827	1.86932
Gfm2	3.43002	2.25031	3.98248	1.55158	0.34486	0.97097	2.53775	3.24709	1.01307	3.15693	1.18250	0.30062	1.44586	2.48303	4.39613	1.40520
Gfod1	0.01319	0.00781	0.00922	0.97650	0.00932	0.00627	0.00726	0.00943	2.17209	0.00000	0.01077	0.00000	0.01280	0.25750	0.67444	0.00695
Gfod2	0.01127	0.00773	1.08929	1.16311	0.00865	0.00615	0.03708	0.88244	3.13718	1.62749	0.00000	0.06622	0.01089	0.03107	1.14743	0.00674
Gfpt1	4.40329	4.62619	1.54973	9.34887	2.57379	0.00000	2.37844	4.89105	11.94219	10.20299	9.96511	8.77004	3.37943	12.70263	13.42814	1.25296
Gfpt2	0.01019	0.00757	0.02859	0.01719	0.42804	0.00803	0.00738	0.01134	0.01248	0.06271	0.02996	0.04569	0.01341	0.01126	0.01051	0.00748
Gfra1	0.01246	0.00819	0.00624	1.20595	0.00953	0.00640	0.00724	0.00982	0.00000	0.07797	0.00000	0.01171	0.01261	1.15488	1.14664	0.00696
Gfra2	0.03147	0.02891	0.01267	0.01324	0.01897	0.01886	0.02798	0.01665	0.03948	0.03126	0.04215	0.02812	0.02711	0.07235	0.02854	0.00734
Gfra3	0.44327	0.00627	0.96850	2.14651	0.01483	0.00423	0.00562	1.47114	1.48530	4.94389	2.14040	4.42079	0.00000	0.03072	0.14209	0.01312
Gfra4	0.00641	1.93161	0.00666	1.30872	0.00811	0.00641	0.00651	0.00909	0.25501	0.47585	0.01076	0.01423	0.01236	0.00562	0.00000	0.00646
Gga1	6.12387	0.06795	2.73962	4.00253	4.09458	3.90698	2.73469	8.18410	3.61161	8.14086	2.97663	0.29623	6.60311	0.00000	0.00000	5.72961
Gga2	19.95757	10.23279	15.03377	1.56222	10.85524	6.92021	8.22069	16.96326	1.09665	11.62023	15.33605	1.16501	13.21149	5.73980	2.02001	8.58738
Gga3	10.87791	5.67539	9.12139	10.99694	5.67925	0.05267	5.28491	1.81210	10.58657	5.90501	2.24194	0.43366	5.80494	2.70638	6.87493	0.94546
Ggact	0.28536	0.00783	0.03554	0.39964	0.01691	0.00827	0.00687	0.01332	0.39968	0.48420	0.31520	0.02284	0.00873	0.07163	0.39126	0.00719
Ggct	0.21931	2.15023	3.48033	11.89407	3.68127	0.00384	1.57089	2.29943	3.88589	3.27098	0.00000	0.04454	0.00000	0.00000	1.48786	0.00984
Ggcx	1.67192	0.00593	0.40097	0.68652	0.07017	0.07907	0.01577	0.55743	0.06352	0.13106	0.72226	0.02528	1.45417	1.56166	0.04587	2.60135
Ggh	0.01639	0.00809	0.53131	0.00858	0.00934	0.00832	0.00734	0.01065	0.01193	0.56243	0.01054	0.00692	0.00929	0.01336	0.00338	0.00713
Ggnbp1	1.14143	0.00640	0.01842	0.12808	0.00596	0.00747	0.00595	0.00000	0.02840	1.30413	0.60745	0.04027	0.47254	0.01074	0.00000	0.00588
Ggnbp2	3.17234	3.92662	3.44847	6.18909	2.28405	2.63827	5.75081	0.89590	3.84181	4.84579	1.34515	1.39703	0.07876	9.35676	2.30501	3.60051

**Ggnbp2os**	0.01631	0.00785	0.21662	1.35868	0.00871	0.00569	0.00748	0.01171	0.00000	0.76878	0.01157	0.00599	0.01312	0.01540	0.04527	0.00717
**Ggps1**	3.26882	2.19821	3.23469	2.28377	1.06774	0.05604	1.51881	0.34244	11.97461	1.87377	2.72867	0.11965	0.17530	2.21578	1.80100	3.16940
**Ggt1**	10.05387	5.36822	13.73196	0.11699	2.76785	1.03372	5.23370	4.23777	0.00232	0.33036	1.53245	0.23165	2.55918	0.00000	0.60418	2.24651
**Ggt5**	0.01098	0.00780	0.00924	0.12691	0.00950	0.00680	0.00720	0.11786	0.23750	0.01304	0.11346	0.00681	0.00768	0.01099	0.00214	0.00740
**Ggt6**	2.41711	0.00459	0.06455	2.49783	0.01152	0.00567	0.06577	2.55756	6.54497	0.00000	2.96930	0.00000	0.00000	0.00000	0.53895	0.00351
**Ggt7**	0.01347	0.00807	0.01148	0.00782	0.00930	0.00676	0.00752	0.01798	0.01102	0.04510	0.01060	0.00814	0.01296	0.00706	0.49798	0.00742
**Ggta1**	0.00000	0.00799	0.00728	4.10475	0.01729	3.42893	0.00693	0.09001	0.75577	0.00000	1.30009	0.01895	0.29598	0.00000	1.22018	1.02951
**Ghdc**	0.83143	2.56854	3.16161	1.48363	0.37557	3.56155	2.07832	8.24956	1.64142	0.12054	1.44020	0.14048	2.90880	1.65677	0.49433	3.92468
**Ghitm**	21.35021	17.59802	23.71740	9.79572	18.42859	13.35260	16.43131	20.71959	17.69365	13.72643	15.27339	13.64325	22.73698	12.45861	10.89068	8.73739
**Ghrh**	0.01374	0.00813	0.01230	0.18447	0.00934	0.00665	0.00754	0.01398	0.00597	0.01538	0.01241	0.00820	0.01271	0.01157	0.00767	0.00739
**Ghrl**	0.01348	0.00827	0.01722	0.01030	0.00932	0.00637	0.00757	0.01353	0.14254	0.01209	0.02530	0.00825	0.01228	0.00983	0.04226	0.00738
**Gid4**	7.38169	3.85697	6.58539	6.10150	3.75731	0.02984	2.12295	3.13197	3.36325	6.47423	3.22304	9.48920	0.12827	7.42091	7.70011	0.26853
**Gid8**	8.80545	2.02970	4.92842	2.85592	2.53534	3.53795	3.53215	2.42257	4.80199	2.87379	1.24935	0.15250	2.34758	3.64652	1.66153	3.97357
**Gigyf1**	1.29646	0.01584	0.26006	0.16870	0.10012	0.00465	0.00600	0.64077	0.16603	0.09997	0.68022	0.02951	0.03104	0.00610	0.12489	0.13518
**Gigyf2**	3.42123	5.64945	2.82497	2.20169	1.05280	0.01806	2.83289	5.96234	2.69672	3.67756	3.94364	0.10657	2.30091	4.00487	5.50153	0.26734
**Gimap1**	0.06019	0.00833	0.25099	1.64405	0.00940	0.13302	0.05111	0.21867	2.01937	0.14626	0.24476	0.05711	0.26706	0.48171	0.32135	0.07835
**Gimap3**	0.01316	0.00785	0.00994	0.07359	0.00897	0.00645	0.00734	0.01015	1.54556	0.76179	0.00000	0.10939	0.01098	0.02852	0.23220	0.00703
**Gimap8**	0.01092	0.03959	0.03127	0.14347	0.00881	0.00709	0.01743	0.03399	0.11229	0.02207	0.01152	0.00781	0.04252	0.05657	0.08472	0.12932
**Gimap9**	1.69608	0.00604	0.45879	0.00389	0.00429	0.00379	0.00584	0.00983	0.18472	0.54588	0.00779	0.01435	0.00879	0.00572	0.50945	0.00505
**Gin1**	1.98910	4.63418	1.00004	0.00000	0.02996	0.03331	2.10058	4.00873	2.12737	3.70234	0.36382	1.43763	0.30138	6.92116	4.91377	0.39500
**Ginm1**	1.28219	1.34191	0.00000	0.05385	0.01328	0.46638	1.70593	0.60929	0.00901	1.45539	0.07164	0.06138	1.91203	0.00760	0.53077	2.17108
**Gins1**	15.00202	6.05389	9.38359	2.64949	10.78620	0.91488	9.89886	12.25167	7.67461	18.57394	9.56763	3.56290	7.48776	8.74291	2.93942	2.25103
**Gins2**	12.50045	2.89482	10.01141	5.91768	4.46627	3.11172	2.30481	4.54201	9.56664	9.83153	3.72515	0.21529	1.85052	0.08591	4.07272	5.58402
**Gins3**	2.03691	0.04876	1.56012	7.09810	0.00272	0.53917	1.29335	2.36680	7.41157	7.32325	3.12277	0.97270	4.49235	0.29227	10.82634	0.00736
**Gins4**	14.07667	1.49838	9.93109	5.48292	10.01963	1.57435	3.32538	5.57682	12.57819	14.27730	14.60390	9.22310	16.04033	5.36752	4.31804	7.38952
**Gip**	0.01306	0.00785	0.00604	0.23786	0.00955	0.00769	0.00746	0.00000	2.33145	0.36758	0.61832	0.11295	0.04433	0.03709	0.38522	0.00739
**Gipc1**	8.31814	0.01627	0.00000	0.00000	3.70102	8.66364	0.04586	5.71301	0.16923	1.58503	2.18013	0.06781	7.69304	0.00435	0.00228	0.76895
**Gipc2**	4.05919	0.01246	1.69464	1.51832	0.02913	0.02012	1.37954	0.00000	0.22887	0.00000	0.19563	0.01655	1.54884	0.03770	0.73382	0.02280
**Gipr**	0.00844	0.00774	0.05317	0.01084	0.00970	0.00690	0.00735	0.80248	0.01136	0.07620	0.00808	0.00690	0.00615	0.01755	0.00000	0.00733
**Git1**	7.63714	1.01902	0.22896	1.62087	5.54319	4.69276	0.24302	6.76703	0.93034	2.08355	0.47440	1.33063	6.03651	0.00000	0.00000	2.48238
**Git2**	4.23436	1.49412	3.39935	3.03755	1.90512	0.13985	3.69699	0.84240	2.62865	2.81386	4.21111	6.35472	1.54047	0.54883	1.84749	0.00533
**Gja1**	7.62973	6.41313	10.21861	0.74876	4.99794	4.70029	5.20537	11.53109	2.90939	0.00000	12.55813	6.08912	4.18637	33.60755	12.28981	0.44849
**Gja10**	0.01373	0.00807	0.01218	0.06027	0.00929	0.00664	0.00753	0.01390	0.19646	0.01438	0.01235	0.00788	0.01273	0.01245	0.00770	0.00740
**Gja3**	0.01371	0.00800	0.01186	0.43442	0.00943	0.00644	0.00738	0.01183	0.18632	0.01456	0.01068	0.00681	0.01292	0.00574	0.49072	0.00734
**Gja4**	0.01153	0.00816	0.00999	0.72123	0.01013	0.00723	0.00723	0.19653	0.20297	0.00000	0.08783	0.01669	0.55488	0.01056	0.34391	0.00724
**Gjb3**	17.99900	6.03223	14.95927	0.00000	10.59303	8.94982	15.64911	19.44452	0.01045	2.38037	9.61287	0.40181	11.11739	0.25633	0.00000	8.46321
**Gjb4**	7.33561	0.00803	0.01355	0.09112	1.19763	0.00586	1.82184	0.00000	0.00697	0.00000	0.01171	0.01969	0.00791	0.01338	0.01158	4.42341
**Gjb5**	17.69795	7.90644	9.74623	0.00823	8.65957	3.87854	12.27187	1.05525	0.00464	0.00000	0.00000	0.09066	1.22676	0.00856	0.00000	11.01038
**Gjc1**	0.00946	0.01683	0.00872	0.08647	0.00945	0.00646	2.31778	0.00927	1.31113	0.00636	0.00004	0.00387	0.01008	0.01501	0.00924	0.02155
**Gjc3**	0.09477	0.02858	0.26200	0.44996	0.01871	0.01474	0.11833	0.04770	0.34087	0.08178	0.47755	0.02175	0.20651	0.72439	0.15219	0.03621
**Gje1**	0.23302	0.04080	0.26398	0.15327	0.14126	0.03733	0.01679	0.00559	0.24106	0.09707	1.04153	0.09933	0.22454	0.37007	0.05410	0.05854
**Gk**	0.03111	0.92474	0.93271	2.68762	0.01002	4.16797	0.00581	0.00000	2.00576	2.32283	0.45593	0.09945	0.62083	2.51279	1.02755	0.02675
**Gk5**	0.18103	0.00826	0.21387	0.04717	0.01460	0.00677	0.01726	2.52586	0.01705	0.93948	0.00000	0.02610	0.14980	9.67049	0.22305	0.00691
**Gkap1**	4.49282	3.96418	3.11006	6.86208	4.39135	0.42218	0.83174	2.84433	4.27599	3.40141	0.23127	0.80691	3.13275	2.01337	1.63101	1.69255
**Gla**	4.89817	0.12955	18.87476	7.32417	6.02987	7.59705	9.15641	8.86245	14.47318	7.84297	5.44580	3.77942	13.02285	13.34121	13.01839	3.81440
**Glb1**	9.51369	3.69108	5.80014	2.49160	3.40745	8.22236	3.75579	15.85262	2.69928	13.70044	14.14675	2.23404	5.85880	6.84658	5.70378	4.73750
**Glb1l**	0.01779	0.00748	0.42953	0.29124	0.00858	0.00600	0.00706	0.01119	0.42678	0.04196	0.00969	0.00470	0.36494	0.00447	0.47776	0.00710
**Glcci1**	0.58733	0.95257	1.54537	14.34961	1.92755	0.61239	0.80679	1.41479	5.88259	3.15762	1.46675	0.89356	1.19809	2.59894	5.70085	0.53761
**Glce**	3.44994	0.00000	3.58682	6.24030	3.78914	0.29300	1.47654	2.28501	3.18832	1.48770	0.16492	0.13179	0.02297	0.03487	3.05992	0.86812
**Gldc**	19.43881	7.83282	17.96098	7.33146	9.31381	10.01666	9.42679	35.79494	16.09199	38.94771	25.34855	21.03517	23.29136	20.11985	27.68972	11.89222
**Gle1**	7.81714	1.38431	10.84470	17.84710	6.77569	1.50946	5.65031	15.23618	12.68108	19.10710	9.16249	4.71600	8.61977	12.16721	15.81339	5.72835
**Glg1**	4.90284	0.00000	6.08986	1.15107	1.93211	0.00186	1.46035	3.49649	0.28163	3.41574	0.00000	0.07931	1.97710	0.18156	2.11861	4.94989
**Gli1**	0.01625	0.00756	0.55128	0.51974	0.00824	0.00553	0.00716	0.01571	0.85224	0.05443	0.00635	0.00524	0.00921	0.00996	0.08097	0.00728
**Gli2**	0.01312	0.00802	0.01124	1.19056	0.00922	0.00622	0.00739	0.01073	0.71335	0.53639	0.00432	0.00267	0.01284	0.00263	1.24918	0.00693
**Gli3**	0.01313	0.00755	0.00893	0.00286	0.00923	0.00610	0.00731	0.00795	5.17027	0.00005	0.03227	0.06353	0.01249	0.00395	0.65738	0.00699
**Glipr1**	0.63297	0.00710	0.00000	0.12432	0.03505	2.27582	1.16286	0.00596	0.00693	0.00000	0.00875	0.03661	0.81216	0.01381	0.00655	2.12490
**Glipr2**	6.15696	1.54190	1.40404	0.64883	12.20557	5.71313	0.73180	1.85796	0.25967	1.81215	1.51459	0.05333	2.10544	0.03845	0.23652	6.23572
**Glis1**	0.01069	0.00823	0.01175	3.68046	0.00951	0.00661	0.00747	0.07959	0.77496	1.03591	0.00645	0.00414	0.01772	0.00432	0.02983	0.00675
**Glis2**	1.65986	0.00303	0.76330	7.76792	3.91411	0.00576	0.01470	0.98544	5.86077	4.11286	0.62060	0.66091	0.00000	5.20092	1.74315	5.62759
**Glis3**	0.01290	0.00792	0.00744	2.93643	0.00931	0.00590	0.00726	0.00963	2.06147	0.14611	0.00000	0.03780	0.01269	0.13604	2.81994	0.00664
**Glmn**	2.70965	1.75847	0.46365	0.25134	1.50936	0.00486	0.00386	2.50447	0.11660	1.50490	0.43400	5.26007	0.00326	0.43349	2.73970	0.18962
**Glmp**	8.43963	3.82905	13.87315	4.77922	0.95003	13.20068	6.62166	19.46362	6.81409	11.32506	8.17750	1.66204	14.46365	6.11166	8.35556	8.78274

**Glo1**	10.50411	10.72417	6.87985	0.11908	6.10290	3.93361	6.32726	0.90018	0.05340	2.10213	1.39589	0.16703	3.21452	2.16452	1.26848	8.68913
**Glod4**	8.34690	14.09119	14.00395	3.97925	17.43271	10.89540	9.88385	9.57197	6.15159	1.78743	7.29258	2.47799	9.56037	1.97677	3.82725	5.77033
**Glod5**	0.87043	0.00672	0.00000	3.55856	0.00608	0.00612	0.00678	0.00823	1.88605	0.40742	0.19704	0.00796	0.00933	1.19888	0.61867	0.00557
**Glrb**	0.01375	0.00807	0.01225	0.01058	0.00932	0.00666	0.00754	0.01389	0.01234	0.01523	0.01311	0.00815	0.01270	0.01220	0.01187	0.00741
**Glrx**	31.75589	9.95980	10.01822	1.05430	19.14032	6.89979	7.79641	0.00000	0.34467	0.00000	0.00717	0.07226	1.75469	0.11582	0.07550	13.14147
**Glrx2**	0.96054	0.01564	1.45219	8.73509	0.03752	0.16204	0.69354	0.17717	19.79811	3.87508	7.93027	0.41232	2.13449	4.09438	2.87034	0.86364
**Glrx3**	5.75980	2.35409	4.79648	3.91086	6.37150	3.47916	2.58982	5.43001	7.84035	6.82777	12.03752	7.07098	8.63737	3.79642	1.13048	4.13315
**Glrx5**	4.03297	0.20358	0.87943	5.30173	6.95532	4.17917	1.10339	2.64113	3.67062	3.55048	0.88061	0.56441	0.32983	0.03337	0.00000	0.00684
**Gls**	0.32927	0.08080	2.09391	7.73553	3.26044	0.32878	3.13305	4.85003	12.80482	7.00675	4.36637	13.62052	4.77439	7.80236	1.76598	4.00502
**Gls2**	4.67028	0.25235	4.18894	0.64561	0.00000	0.73328	1.46771	0.39811	1.23270	8.87472	6.35210	0.74982	0.96254	1.85152	3.28534	0.02101
**Glt1d1**	0.05925	0.00801	0.04428	0.12388	0.00907	0.00767	0.00742	0.00406	0.01750	0.00219	0.49350	0.01331	0.19657	0.52048	0.00000	0.00734
**Glt28d2**	0.05062	0.03993	0.10172	0.30369	0.03145	0.01275	0.00725	0.02910	0.06380	0.47022	0.21635	0.00651	0.02482	0.00962	0.02876	0.00724
**Glt8d1**	1.09787	3.29317	2.09937	5.54452	0.00270	3.08583	2.74146	0.87289	2.93098	1.55059	1.66389	0.53958	0.95250	2.00487	3.43615	0.01634
**Glt8d2**	0.01178	0.00676	0.00000	0.00358	0.00939	0.02146	1.59700	0.00000	0.13885	0.00000	0.39881	0.00441	0.00769	0.01113	0.00243	0.00709
**Gltp**	3.19310	2.37795	5.69444	3.37157	1.50645	10.00562	2.94213	2.15734	1.18436	1.07287	0.79027	0.08321	5.21900	0.02183	0.14906	0.26941
**Gltscr1**	0.18550	0.00715	0.00560	0.36237	0.00747	0.00815	1.23172	0.12155	0.05425	1.39084	0.04113	0.01128	0.01143	0.07911	0.97953	0.00714
**Gltscr1l**	3.17023	1.63801	0.81133	7.91388	0.00000	0.06267	0.00236	3.58398	8.70603	7.45878	3.11569	0.63385	0.00000	12.21549	10.44135	0.00760
**Gltscr2**	14.69528	0.12382	0.00000	7.62434	9.47767	2.27776	0.11570	11.54344	13.33607	30.60319	13.20678	0.76954	20.60023	0.00000	0.00000	11.69792
**Glud1**	23.27444	19.78983	14.30612	12.84527	18.97615	2.77426	16.13863	8.49710	10.21183	14.53682	7.37895	16.16517	9.82600	3.84999	13.77804	14.14409
**Glul**	10.83897	11.55083	16.15989	19.90070	6.47316	5.49941	12.47971	21.31414	30.20472	16.56533	21.18956	2.08002	18.96766	9.51416	13.95148	5.97395
**Glyctk**	0.63456	0.00623	0.32807	0.08465	0.01456	0.00498	0.00573	1.13521	0.07733	0.08514	0.06035	0.01271	1.38863	0.05030	0.33997	0.00563
**Glyr1**	7.12113	9.62145	8.28983	3.43208	11.53578	4.79855	7.87661	7.18995	8.18403	9.57337	8.64012	1.98762	4.79984	8.89590	11.20314	7.24142
**Gm10012**	5.31180	0.20715	4.41049	2.21272	3.23098	0.95879	0.67591	0.03043	8.56265	1.21725	17.58407	0.48385	2.21543	1.27951	0.72242	2.29906
**Gm10033**	0.22695	0.11579	0.45564	1.81978	0.08229	0.10215	1.23602	0.15319	2.24560	0.59511	0.87731	0.21926	0.17494	0.17938	0.48380	0.05244
**Gm10046**	0.03068	0.00777	0.01609	0.01035	0.00814	0.00731	0.00732	0.01087	0.01247	0.02903	0.01243	0.00676	0.01837	0.18457	0.00547	0.22170
**Gm10069**	3.91917	2.83368	3.10304	1.50591	1.52177	1.57190	1.00070	2.16124	0.29225	2.46055	1.38906	0.58316	2.55045	2.28545	2.66660	1.81038
**Gm10190**	0.16621	0.00774	0.07289	0.94484	0.04278	0.00635	0.02811	0.00000	2.08168	0.00000	0.80359	0.05230	0.04054	0.33482	0.02720	0.02871
**Gm10319**	0.01376	0.00812	0.01224	1.40691	0.00931	0.00653	0.00748	0.01384	0.00000	0.01481	0.01378	0.00826	0.01266	0.01192	0.00537	0.00718
**Gm10324**	2.57904	3.54272	3.06864	0.10390	1.94602	1.52740	2.28065	5.76011	0.23649	4.34974	5.51109	0.29215	2.46453	14.73753	9.39816	0.51500
**Gm10334**	0.01368	0.00832	0.01179	0.24159	0.00927	0.00687	0.00759	0.01388	0.06960	0.09796	0.01090	0.02163	0.01316	0.00773	0.07506	0.00738
**Gm10336**	0.25504	0.00736	0.26704	2.65842	0.01168	0.19179	0.48850	2.00319	0.33921	0.00000	0.63934	0.27476	1.18393	2.55441	0.78524	1.53730
**Gm10354**	0.01172	0.00805	0.07035	0.04896	0.00931	0.00600	0.00760	0.04203	0.00984	0.08440	0.01131	0.00840	0.01070	0.00777	0.16281	0.00745
**Gm10364**	0.14474	0.11615	0.01996	0.27349	0.12431	0.11337	0.04854	0.02421	1.12943	0.02332	0.00000	0.01489	0.07870	0.00491	0.31806	0.10204
**Gm10389**	0.04828	0.04858	0.08714	0.21600	0.00857	0.01515	0.03736	0.04327	0.70330	0.07191	0.17844	0.05136	0.06437	0.34711	0.09353	0.02956
**Gm10406**	0.04395	0.00787	0.03847	1.46822	0.00822	0.00587	0.00719	0.00968	0.12243	0.01563	0.06326	0.00669	0.02584	0.21421	0.00000	0.00707
**Gm10408**	0.01431	0.00802	0.02460	0.23620	0.00923	0.01981	0.01762	0.01184	0.03709	0.03009	0.02559	0.00730	0.01209	0.05450	0.02280	0.00748
**Gm10409**	0.09814	0.00790	0.01274	0.48136	0.00779	0.00652	0.00736	0.01391	0.01115	0.02701	0.02621	0.00812	0.01982	0.02800	0.00771	0.00722
**Gm10416**	1.14246	1.26821	0.76699	0.03135	0.27723	0.01216	0.01618	0.47017	0.00993	0.17210	0.23272	0.42277	0.79249	0.27703	0.02681	2.34625
**Gm10432**	0.03877	0.00789	0.00834	0.04057	0.00821	0.00713	0.03970	0.01546	0.02344	0.09449	0.01023	0.02000	0.02732	0.19528	0.02047	0.00732
**Gm10433**	0.00892	0.00776	0.01968	0.42228	0.00910	0.00816	0.00721	0.90029	0.57644	0.69445	0.00000	0.00396	0.01292	0.00832	0.03213	0.01482
**Gm10436**	0.00000	0.00753	0.00000	23.08931	0.00858	0.00349	0.00580	0.38361	12.16573	4.72848	0.66509	0.13785	0.00434	13.89717	17.80091	0.00219
**Gm10439**	0.01369	0.00780	0.01211	0.09203	0.00956	0.00655	0.00743	0.01173	0.03186	0.01914	0.02511	0.00632	0.01560	0.00619	0.55600	0.00748
**Gm10509**	0.53044	0.12413	0.51461	0.35089	0.00569	0.23278	0.00692	0.05190	1.87531	0.07094	0.12679	0.29990	0.01860	0.59699	1.05035	0.87037
**Gm10512**	0.01521	0.00783	0.06423	7.49929	0.00912	0.00495	0.00673	0.00096	1.17282	0.00000	0.25233	0.00000	0.00897	0.16222	3.36537	0.00570
**Gm10548**	0.10400	0.06203	0.26019	0.02987	0.03474	0.05033	0.11315	0.13279	0.15738	0.11668	0.22962	0.01771	0.04662	0.04348	0.01672	0.04279
**Gm10636**	0.13251	0.00809	0.08007	0.09267	0.09897	0.00625	0.06980	0.01777	0.10445	0.02671	0.30949	0.11152	0.03420	0.22818	0.06994	0.00739
**Gm10638**	0.01288	0.00818	0.01072	0.20298	0.00922	0.00615	0.00751	0.01214	5.97905	0.02119	0.00000	0.00000	0.01338	0.01045	0.00397	0.00706
**Gm10639**	0.18533	0.00741	0.08211	0.02897	0.00725	0.39659	0.09903	0.42519	0.00929	0.40401	0.35319	0.03380	0.67051	0.29402	0.17404	0.00653
**Gm10653**	12.49611	1.82009	7.46141	39.90341	9.51170	3.18747	2.04891	2.39834	38.77015	18.99856	19.00193	3.80707	8.37822	10.21077	15.75432	5.46635
**Gm10658**	0.01155	0.00828	0.83639	0.26690	0.00777	0.00911	0.00785	0.09947	0.45911	0.00000	0.01642	0.03941	0.00462	0.03221	0.61887	0.00686
**Gm10696**	0.02296	0.00868	0.67094	15.84472	0.01082	0.00298	0.00709	0.09976	11.88547	3.09931	0.48043	0.38130	0.03891	3.47003	7.55364	0.00349
**Gm10767**	1.02024	3.57163	2.99398	1.51632	0.47059	1.53087	1.39100	3.19422	5.05776	5.49101	2.03773	5.26288	1.05985	6.52850	5.03567	0.41315
**Gm10768**	0.14121	0.00811	0.00712	0.00395	0.01662	0.00679	0.00732	0.01108	0.34791	0.00469	0.00893	0.00639	0.01263	0.01311	0.36708	0.00739
**Gm10778**	0.15243	0.41717	2.08186	3.66405	0.74295	0.65974	1.20323	3.94025	4.75009	2.11975	5.82064	0.38782	2.46825	6.27142	3.32820	0.08342
**Gm10785**	0.00934	0.00851	0.00751	0.00540	0.00958	0.00706	0.03086	0.26169	0.03127	0.01107	0.48591	0.00671	0.02171	0.01505	0.03230	0.00720
**Gm10789**	0.03549	0.00796	0.10961	0.89404	0.01024	0.00741	0.00757	0.06852	1.73783	0.09339	0.03808	0.76947	0.02487	0.43131	0.26366	0.00721
**Gm10791**	0.02397	0.03846	0.09461	0.34556	0.02598	0.01451	0.01740	0.03296	0.16105	0.06298	0.16186	0.16146	0.11696	0.16372	0.09008	0.01437
**Gm10814**	0.01370	0.00761	0.01137	0.85514	0.00933	0.00642	0.00739	0.01175	0.75823	0.00186	0.00745	0.00575	0.01288	0.02216	0.33255	0.00716
**Gm10845**	1.22722	2.51962	7.96906	8.38153	0.84741	4.06719	3.93781	12.04996	5.09370	14.28411	2.04098	14.98243	2.90537	15.55234	13.29481	1.27187
**Gm10857**	0.84507	0.00710	0.48295	14.36288	0.00571	0.07919	0.02772	0.10053	2.39893	0.00000	0.05509	0.02549	0.07093	0.28374	4.23743	0.00360
**Gm10865**	0.05886	0.03885	0.43500	10.78869	0.00731	0.02229	0.14335	0.79356	8.57297	0.50190	0.28375	0.23855	0.46400	0.78791	1.22790	0.03381
**Gm10941**	0.02931	0.00777	0.02164	0.07034	0.00858	0.00706	0.02821	0.02645	0.19687	0.01431	0.05241	0.00685	0.01503	0.12160	0.05113	0.00734

Gm1110	0.80358	0.00740	0.00000	0.00352	0.01620	0.00670	0.00595	0.67068	0.35278	0.85428	1.28631	0.00978	0.00000	2.11474	0.00000	0.00674
Gm11127	0.75969	1.11325	4.06483	3.88935	0.53158	1.91965	1.70620	8.64468	7.74366	9.00334	6.85748	9.50822	1.35244	5.25714	4.19790	0.73562
Gm11166	0.01368	0.00813	0.01208	0.12904	0.00928	0.00662	0.00753	0.01389	0.48635	0.01292	0.01061	0.00748	0.01281	0.01247	0.00533	0.00737
Gm1123	0.01229	0.00792	0.00668	0.11728	0.00962	0.00668	0.00718	0.00927	0.00954	0.00614	0.00815	0.00213	0.01343	1.68241	0.00305	0.00742
Gm11238	0.01112	0.00813	0.01031	4.27702	0.00941	0.00617	0.00717	0.07833	3.13146	0.03174	0.00000	0.00213	0.00996	0.03147	0.00597	0.00652
Gm1140	0.20155	0.00845	0.01525	0.00975	0.20842	0.00622	0.00785	0.03442	0.01193	0.01065	0.01232	0.02112	0.01031	0.00925	0.08828	0.00720
Gm11413	0.00906	0.00815	0.22626	2.06478	0.00906	0.00533	0.00735	0.00897	1.15728	0.47377	0.00000	0.02743	0.00976	1.89963	1.39488	0.00656
Gm11423	0.00000	0.00648	0.00268	1.08720	0.46574	0.00746	1.74641	0.35900	1.91221	0.30300	0.00000	0.02442	0.00779	0.00620	0.28074	0.00690
Gm11487	0.01072	0.00773	0.00873	2.48450	0.00938	0.00602	0.00716	0.08585	4.09912	0.16630	0.00000	0.03594	0.00938	0.02503	0.70850	0.00678
Gm11517	4.39457	0.68650	7.55408	17.73581	1.34060	0.31760	5.88986	28.86866	30.11750	24.58784	38.37708	6.43287	7.70923	29.83406	25.48425	0.79908
Gm11532	0.01590	0.00799	0.05395	0.22623	0.00915	0.00588	0.00737	0.01264	0.00763	0.03755	0.01475	0.00849	0.01152	0.01126	0.04948	0.00719
Gm11544	0.00000	0.00720	0.21265	23.04041	0.00848	0.07178	0.19493	1.16235	22.42695	5.12022	1.34276	1.21484	1.70682	1.70721	10.02910	0.00000
Gm11545	5.09630	3.37677	17.92498	13.38501	0.44368	8.51737	11.84898	26.59109	14.43026	20.31376	17.88220	11.22349	15.72379	33.95933	19.24505	6.32885
Gm11549	0.10497	0.01864	0.08946	0.17194	0.00987	0.00582	0.01738	0.00003	0.23255	0.01610	0.77936	0.04438	0.07034	0.17699	0.00426	0.01431
Gm11665	0.01382	0.00789	0.01083	0.85016	0.00927	0.00649	0.00734	0.00737	0.07393	0.10983	0.18039	0.00585	0.01019	0.01072	0.15327	0.00720
Gm11696	1.36585	0.00611	0.05699	0.10300	0.01331	0.03774	0.00638	0.02726	0.20533	0.03712	0.42822	0.01412	0.03087	0.16080	0.03796	0.01995
Gm11744	0.01203	0.00822	0.01430	0.01055	0.01019	0.00713	0.00767	0.01614	0.01235	0.00859	0.01518	0.00783	0.31969	0.01272	0.01022	0.00756
Gm11944	0.01282	0.00756	0.00919	0.02547	0.00905	0.00664	0.00723	0.00894	0.01264	0.84917	0.04433	0.00409	0.01077	0.39511	0.15456	0.00721
Gm11961	0.01626	0.00763	0.17117	0.00281	0.00905	0.00572	0.00722	0.01002	0.52974	0.00525	0.01735	0.00496	0.01199	0.00885	0.83708	0.00731
Gm11992	0.01364	0.00822	0.01199	0.74586	0.00931	0.00650	0.00748	0.01343	0.80227	0.00813	0.00812	0.00706	0.01280	0.00750	0.07295	0.00721
Gm12018	0.00649	0.00764	0.01291	0.20599	0.06269	0.01268	0.07945	0.06933	0.15660	0.04746	0.02321	0.00625	0.06095	0.49346	0.00935	0.02158
Gm12060	0.80125	0.33553	0.40067	0.82798	0.45699	0.67713	0.21010	0.43216	1.33576	0.51801	0.36661	0.35030	0.56882	0.49916	0.62309	0.14973
Gm12070	32.50846	17.43914	21.25269	9.14701	22.96614	15.94727	13.32680	2.67507	13.51944	3.68784	2.19985	3.12464	23.14425	0.12121	2.98567	22.82521
Gm12169	0.00546	0.09412	0.47861	0.00913	0.00861	3.11194	0.13084	0.00902	0.01269	0.00000	0.00909	0.00761	1.44745	0.01824	0.00032	0.26816
Gm12171	0.01306	0.00803	0.02481	0.08572	0.00917	0.06039	0.00745	0.01318	0.00799	0.01443	0.01208	0.00804	0.01838	0.01196	0.00882	0.02191
Gm12185	0.18062	0.02776	0.30373	7.61388	0.00827	0.01031	0.27241	0.00941	5.19161	0.54931	0.52156	0.18439	0.16246	1.52069	2.63525	0.04824
Gm12238	0.01353	0.00789	0.01213	0.01057	0.00935	0.00664	0.00747	0.01899	0.01237	0.01409	0.01300	0.00778	0.01232	0.01098	0.03092	0.00741
Gm12250	0.01272	0.00760	0.23604	0.05850	0.00931	0.00564	0.00696	0.00722	0.00683	0.00000	0.01911	0.00411	0.01722	3.22969	1.01692	0.00722
Gm12253	0.04105	0.00805	0.01579	0.27736	0.01990	0.00637	0.02858	0.00423	0.38824	0.00978	0.39712	0.00447	0.01086	0.37442	0.02860	0.01445
Gm12338	3.73517	0.07440	1.61805	0.97570	1.19478	0.60650	0.38958	0.15051	2.52569	1.09796	8.18966	0.21608	1.99447	0.38049	0.87662	2.57588
Gm12504	21.23292	8.89322	19.76616	0.75713	13.66863	10.29075	10.66534	24.56782	1.83027	20.59881	10.25843	6.10413	22.74332	9.61023	7.34121	11.02203
Gm12505	0.08677	0.00746	0.19652	0.44830	0.17153	0.03017	0.28537	0.10226	0.23110	0.19313	0.00775	0.02519	0.06438	0.23331	0.25884	0.00713
Gm12530	0.01375	0.00805	0.01225	0.01061	0.00932	0.00666	0.00754	0.01390	0.01235	0.01531	0.01310	0.00811	0.01270	0.01234	0.01062	0.00741
Gm12596	0.01053	0.00792	0.01301	0.59879	0.00963	0.00734	0.00755	0.05094	0.54527	0.15753	0.00000	0.02399	0.02280	0.50276	1.34033	0.00743
Gm12657	15.65689	5.60200	15.07437	8.60983	9.47375	7.50404	8.01784	21.38758	12.96852	24.04604	12.02172	6.65857	15.51107	8.31027	3.01206	6.84616
Gm12789	0.01651	0.05003	0.16098	11.75887	0.00845	0.01127	0.16066	0.13482	7.55002	0.14596	0.05563	0.00000	0.13867	0.16898	0.68466	0.02693
Gm12794	0.01500	0.01915	0.02990	2.62877	0.00902	0.00613	0.04968	0.05117	2.62971	0.01922	0.00000	0.00307	0.02738	0.00556	0.05738	0.00676
Gm128	0.00991	0.00735	0.06195	3.06417	0.00976	0.00570	0.00689	1.35967	1.29470	0.12416	0.85032	0.00000	0.00416	0.00000	0.27642	0.00675
Gm12992	0.01140	0.00811	0.03750	2.10688	0.00937	0.00577	0.00739	0.04663	0.70503	0.08577	0.00766	0.02711	0.01080	0.09153	0.09830	0.00736
Gm13023	0.00000	0.00742	0.15984	18.64440	0.00925	0.00529	0.00586	0.12380	12.90572	0.72496	0.93659	0.56592	0.00000	8.08204	6.39991	0.00317
Gm13034	0.00253	0.22281	0.01665	1.00325	0.02015	0.01199	0.14183	0.10460	0.27256	0.31758	0.01326	0.00000	0.02471	1.91058	0.49883	0.01416
Gm13078	0.05651	0.24276	0.58330	7.14732	0.09753	0.12935	0.39192	1.53260	10.36395	0.18997	0.00000	0.00000	0.22574	0.87601	0.08390	0.01245
Gm13083	0.16345	0.41618	0.87223	2.97446	0.08279	0.23661	0.59341	2.01804	5.64323	0.36012	0.00000	0.61076	0.30542	1.47877	0.25564	0.03525
Gm13084	0.01422	0.00751	0.00000	7.80919	0.00882	0.00513	0.00637	0.00000	1.78396	0.24252	1.09975	0.00000	0.00004	4.38444	5.67057	0.00544
Gm13088	0.01304	0.00769	0.00648	1.04679	0.00944	0.00665	0.00718	0.00706	0.00000	0.04153	0.09967	0.00369	0.01145	0.75143	0.54394	0.00717
Gm13102	5.41859	13.84198	22.76193	6.09640	9.97307	5.56540	8.25749	26.43495	16.19560	22.72993	13.43230	18.47579	17.39487	30.29791	14.64443	1.44859
Gm13103	0.03586	0.00751	0.00097	2.21833	0.00838	0.00675	0.00714	0.00605	0.01712	0.05062	0.10393	0.00000	0.01135	1.58392	0.94550	0.00682
Gm13119	0.00263	0.38424	0.00516	0.72073	0.00837	0.00460	0.00630	0.03029	4.92616	0.00000	0.00000	5.58017	0.01079	0.49431	4.00882	0.00616
Gm13125	0.01315	0.00755	0.01201	1.16209	0.00896	0.00627	0.00720	0.00999	0.00000	1.02783	0.01230	0.00451	0.01045	0.00597	0.40815	0.00690
Gm13128	0.41078	1.49875	3.48556	0.68008	0.00513	0.02202	1.33346	0.88224	2.22476	0.00000	0.00000	0.00000	1.56592	3.96577	3.29308	0.51336
Gm13212	2.16041	5.79676	4.36607	5.92330	2.75570	1.63023	4.03595	7.56030	12.34690	4.20329	6.11148	23.19021	5.54815	28.95720	2.77878	2.53158
Gm13238	0.01284	0.00760	0.00997	0.16909	0.00981	0.00707	0.39144	0.01038	0.14601	0.00873	0.05962	0.00691	0.02420	0.01637	0.02685	0.00754
Gm13315	0.02497	0.05956	0.01625	0.08867	0.03726	0.00737	0.00735	0.01647	0.10696	0.01452	0.01222	0.02814	0.02599	0.01501	0.02322	0.02186
Gm13375	0.62266	0.00688	0.00944	0.01244	0.01454	0.00793	0.00686	0.25778	0.01229	0.57666	0.05168	0.00472	0.19841	0.00669	0.09228	0.07448
Gm13498	7.73595	5.95601	10.97833	0.00000	5.85800	2.41800	5.27757	12.02816	0.00921	7.15796	0.16004	3.50978	7.81050	7.46539	4.94114	3.24028
Gm13648	0.01193	0.00774	0.00297	1.32961	0.00987	0.00726	0.04885	0.00000	2.21193	0.83277	1.34491	0.01150	0.08355	0.00000	1.39250	0.00721
Gm13752	0.00886	0.00788	0.00692	1.85771	0.01010	0.00728	0.00731	0.51874	0.00000	0.30472	0.27131	0.01423	0.01048	0.00521	0.00000	0.00699
Gm13830	0.02027	0.01918	0.04236	0.06530	0.00994	0.01750	0.00741	0.02592	0.67770	0.06369	0.02122	0.05003	0.04461	0.02944	0.02162	0.00726
Gm13884	0.00895	0.00751	0.06656	0.00950	0.14396	0.00877	0.00729	0.01115	0.01216	0.19077	0.01302	0.00645	0.10888	0.00931	0.03714	0.00772
Gm13985	0.08476	0.03915	0.14212	0.30609	0.09129	0.03308	0.02753	0.04644	0.24867	0.13655	0.45985	0.09926	0.15768	0.21212	0.19836	0.05894
Gm13986	0.04065	0.00785	0.01484	0.00975	0.01053	0.00737	0.03750	0.01088	0.01187	0.02130	0.02444	0.11612	0.01843	0.03796	0.11116	0.01459
Gm14005	1.53575	0.14099	0.80234	4.90230	1.83512	0.08490	0.18776	0.00448	7.16623	0.00000	4.71818	0.29620	0.22294	2.48090	0.40088	0.14524

**Gm14023**	0.00764	1.56826	0.74618	0.00433	0.00677	0.00559	0.00653	0.00797	0.06006	0.00512	0.02392	0.00660	0.01511	0.02784	0.00000	0.01365
**Gm14057**	0.00067	0.00778	0.13995	1.68178	0.02045	0.00892	0.27395	0.85395	1.57704	0.82075	0.67762	0.10274	0.01164	1.37175	0.00000	0.00675
**Gm14085**	0.01364	0.00790	0.01222	0.32348	0.00914	0.00648	0.00732	0.01108	0.00503	0.35433	0.01439	0.00773	0.01119	0.01733	0.00379	0.00708
**Gm14124**	0.21832	0.04863	0.36223	0.17908	0.02371	0.01744	1.05637	0.43159	0.13244	0.06644	0.31236	0.05160	0.11354	1.75244	0.57442	0.01384
**Gm14137**	0.06259	0.00747	0.00372	0.68865	0.00789	0.00735	1.06827	0.06807	0.00000	0.00530	0.04143	0.00685	0.00854	0.00540	0.24371	0.00701
**Gm14139**	0.06233	0.01763	1.85173	0.02019	0.00602	0.00595	1.54641	0.29231	0.49127	0.00000	0.81977	0.04663	1.18544	1.78794	0.82907	0.01283
**Gm14164**	0.00562	0.00851	0.00627	0.64302	0.01080	0.00652	0.00807	0.20579	0.00000	0.00431	0.00340	0.01123	0.00736	2.61897	0.07971	0.00731
**Gm14204**	0.05192	0.01781	0.07731	0.96574	0.03540	0.01965	0.11017	0.05004	1.20474	0.09183	0.11718	0.05197	0.07389	0.81866	0.10898	0.01424
**Gm14288**	0.07955	0.09440	0.10026	0.06001	0.01025	0.11050	0.03866	0.02628	0.06252	0.01207	0.04112	0.00740	0.07558	0.04540	0.04783	0.05085
**Gm14295**	2.87968	2.15712	9.51084	0.72908	0.18822	3.15323	1.35606	0.55316	0.57083	1.14877	0.67044	0.03562	3.44934	4.30337	0.00000	1.36964
**Gm14305**	0.13250	0.03953	0.24109	0.03914	0.02568	0.00694	0.01736	0.02967	0.03715	0.04709	0.27729	0.03452	0.06044	0.48700	0.03410	0.14391
**Gm14322**	1.57915	2.43660	6.80010	2.69490	0.42071	0.46209	1.56796	0.89177	1.63248	1.51611	1.64758	0.46980	5.77299	2.56405	3.22158	2.08009
**Gm14325**	3.98540	4.87114	8.95820	1.50116	0.34856	1.17872	2.02662	2.21191	0.81295	4.26999	1.63560	0.59534	3.64852	9.49719	1.98536	1.92261
**Gm14326**	0.66159	0.71760	1.29566	0.60834	0.05763	0.29311	0.56447	0.15725	0.06549	0.85506	0.83220	0.33876	0.54340	2.06523	0.42062	0.48409
**Gm14327**	0.01268	0.01835	0.02949	0.00701	0.00910	0.00790	0.02794	0.01196	0.51073	0.01036	0.02088	0.00687	0.02596	0.01671	0.01828	0.00733
**Gm14391**	0.01379	0.00783	0.01135	0.18788	0.00923	0.00661	0.00740	0.00967	0.09670	0.10172	0.12004	0.00647	0.01107	0.00797	0.03558	0.00750
**Gm14393**	0.17015	0.23381	0.38926	0.29667	0.11461	0.05311	0.27044	0.17019	0.05813	0.08521	0.33637	0.01599	0.22075	0.33539	0.04290	0.47613
**Gm14403**	2.08121	3.56602	5.00866	2.07151	0.14209	0.13662	0.60113	0.14501	4.97732	1.92417	0.40609	3.49846	2.61617	6.60579	9.05762	2.01089
**Gm14405**	0.21718	0.21566	0.42497	0.05469	0.01151	0.33842	0.22925	0.05081	0.11112	0.11132	0.02636	0.01089	0.12979	1.80764	0.18721	0.03582
**Gm14440**	0.34131	2.04055	2.08371	0.44051	0.00489	1.92447	0.87785	0.13296	0.70701	0.19389	0.10295	0.13638	0.83153	3.65716	0.35934	0.56170
**Gm14461**	0.13092	0.17698	0.16748	0.38552	0.01795	0.06574	0.07022	0.10790	0.40655	0.29115	0.53910	0.33165	0.14053	0.20181	0.27097	0.07313
**Gm14634**	0.01360	0.00827	0.01198	0.30068	0.00922	0.00684	0.00760	0.01396	0.36304	0.13788	0.00980	0.00849	0.01307	0.01101	0.00578	0.00735
**Gm14635**	0.01375	0.00819	0.01231	0.56226	0.00934	0.00661	0.00752	0.01397	0.00058	0.01535	0.01319	0.00831	0.01268	0.01102	0.00498	0.00732
**Gm14827**	0.01803	0.00808	0.06308	0.11279	0.01038	0.00611	0.00700	0.02768	0.28813	0.02291	0.02165	0.01431	0.24054	0.59196	0.10031	0.00712
**Gm14851**	0.02031	0.00800	0.01021	0.96197	0.00872	0.00644	0.00745	0.01105	1.64384	0.00556	0.07648	0.01557	0.02409	0.00797	0.05150	0.00702
**Gm15056**	0.01369	0.00786	0.01139	0.00421	0.00931	0.00657	0.00740	0.01171	0.70403	0.02501	0.00684	0.01577	0.01333	0.00808	0.34922	0.00763
**Gm15107**	0.01390	0.00788	0.00991	0.00852	0.00921	0.00668	0.00745	0.00667	0.03378	0.00683	0.27024	0.00640	0.00941	0.01068	0.13446	0.00731
**Gm1527**	0.01323	0.00782	0.00913	0.80599	0.00938	0.00633	0.00727	0.00993	1.73793	0.00000	0.00000	0.01089	0.01316	0.26093	0.79447	0.00703
**Gm15319**	0.01371	0.00792	0.01234	0.35884	0.00946	0.00668	0.00755	0.01360	0.14908	0.01071	0.02595	0.00746	0.01530	0.01037	0.06514	0.00739
**Gm15401**	0.07252	0.07303	0.15298	0.07959	0.07513	0.01236	0.01741	0.01443	0.61813	0.06061	0.53079	0.04419	0.19766	0.10172	0.08298	0.05070
**Gm15417**	0.02674	0.00813	0.01791	0.08318	0.00974	0.00635	0.00757	0.02029	0.20667	0.02765	0.06744	0.00754	0.01963	0.05282	0.02662	0.00734
**Gm15421**	4.27010	0.36064	2.65379	1.24029	4.39692	0.25669	0.21235	0.26132	2.61832	0.85754	15.11639	0.50785	2.57162	2.13140	0.96270	2.01080
**Gm15446**	0.33806	0.61432	0.37820	0.25957	0.02475	0.22069	0.07787	0.34452	0.07682	0.25778	4.04067	0.79190	0.42463	5.48455	0.15178	1.17959
**Gm15455**	3.31594	0.90915	0.36738	11.73733	0.16377	1.35239	0.80894	1.43952	7.66324	1.60583	0.40030	0.19820	1.22158	0.54639	4.07841	0.71928
**Gm15545**	0.01207	0.00798	0.01113	0.63453	0.00942	0.00661	0.00729	0.06204	0.19991	0.01994	0.01214	0.00763	0.01014	0.01071	0.00325	0.00730
**Gm15645**	3.30320	1.56322	6.02774	7.70559	1.42803	2.54346	4.05533	5.94635	12.46445	10.12642	13.56894	8.34104	3.97746	4.70662	5.56634	1.46597
**Gm15694**	0.29214	0.00730	0.15606	1.93631	0.04426	0.03503	0.16985	0.64378	1.10196	0.05767	0.50868	0.08845	0.09286	0.03682	0.32589	0.07139
**Gm15698**	17.62846	11.41590	24.04569	17.60641	13.76557	11.36718	11.02310	16.47380	15.31873	9.61210	9.87783	6.65125	12.25022	12.53437	9.49543	8.06743
**Gm15706**	0.01374	0.00816	0.01228	0.30555	0.00932	0.00667	0.00755	0.01414	0.00544	0.01852	0.01297	0.00840	0.01279	0.01123	0.00668	0.00737
**Gm15708**	0.01337	0.00797	0.01202	0.07638	0.00922	0.00685	0.00755	0.01955	0.01140	0.17260	0.01254	0.00828	0.01253	0.00726	0.06693	0.00740
**Gm15760**	0.01123	0.02789	0.11881	1.60503	0.00895	0.45117	0.00699	0.01769	0.30591	1.26742	0.01394	0.01735	0.01458	1.80412	0.06636	0.00695
**Gm15787**	0.01314	0.00799	0.00894	0.00530	0.00946	0.00676	0.00729	0.01028	0.17222	0.00751	0.00867	0.00510	0.01329	0.64309	0.00000	0.00746
**Gm15800**	3.62492	5.31278	2.53832	5.45505	0.93862	0.00231	1.05012	3.67076	6.19053	6.51322	0.85823	0.30959	0.16085	0.40596	1.55134	1.68359
**Gm15910**	0.05549	0.00772	0.09687	0.04881	0.00788	0.00648	0.00744	0.04349	0.26419	0.02652	0.44688	0.00503	0.09179	0.11185	0.05626	0.01417
**Gm15915**	0.01120	0.00776	0.01034	0.01069	0.00956	0.00670	0.00724	0.11208	0.01224	0.01309	0.01266	0.00750	0.00847	0.01241	0.00888	0.00740
**Gm15941**	0.01363	0.00817	0.01167	2.57802	0.00926	0.00632	0.00742	0.01264	1.00052	0.00478	0.03973	0.00639	0.01205	0.00425	0.06626	0.00687
**Gm1600**	0.01622	0.00810	0.01716	0.05612	0.01805	0.00728	0.01838	0.01920	0.12160	0.08021	0.02313	0.03897	0.01127	0.03064	0.05357	0.00716
**Gm16023**	0.01345	0.00828	0.01185	0.32803	0.00925	0.00652	0.00762	0.01719	0.74257	0.04657	0.01021	0.00765	0.01196	0.01417	0.02270	0.00714
**Gm16062**	0.01346	0.01880	0.01200	0.00900	0.00925	0.00666	0.01792	0.01352	0.12484	0.01456	0.01218	0.00792	0.01267	0.01258	0.00927	0.00740
**Gm16070**	0.05961	0.00759	0.06394	8.59351	0.00903	0.06453	0.04662	0.09771	5.38331	3.48413	0.00000	0.16953	0.19648	6.84702	13.48244	0.00507
**Gm16157**	0.02672	0.00746	0.01051	0.27937	0.00849	0.00664	0.00729	0.00848	0.08863	0.56791	0.06803	0.00601	0.01199	0.05970	0.03675	0.00722
**Gm16223**	0.09499	0.28137	0.24670	0.03753	0.05770	0.22743	0.24239	0.19597	0.01768	0.40589	0.06774	0.23015	0.03521	0.78478	0.47771	0.01424
**Gm16287**	0.11361	0.01890	0.26209	0.04030	0.00921	0.01258	0.03879	0.01778	0.73334	0.06680	0.95378	0.04389	0.19991	0.29781	0.03737	0.00688
**Gm16381**	0.00991	0.00755	0.00421	25.75119	0.00901	0.00230	0.00622	0.00000	19.36304	0.00000	0.11015	0.00000	0.01316	0.64956	2.34730	0.00871
**Gm16386**	0.00963	0.06162	0.05809	0.69388	0.17814	0.02226	0.04851	0.03541	0.13129	0.03198	0.02263	0.01598	0.00984	0.52423	0.01406	0.00720
**Gm16513**	3.59773	3.06552	5.31308	8.41271	2.31089	0.20910	4.06896	4.11767	10.55515	9.94350	12.84178	6.98218	3.80043	20.83497	8.08123	0.23241
**Gm16523**	0.04162	0.02908	0.12115	0.18729	0.00957	0.06646	0.01710	0.00974	0.10964	0.08618	0.40971	0.05440	0.14727	2.12717	0.06744	0.03705
**Gm1653**	0.01347	0.00770	0.01189	0.86535	0.00907	0.00665	0.00733	0.01091	0.00000	0.62957	0.01141	0.00618	0.01207	0.01370	0.05059	0.00751
**Gm16551**	0.01352	0.00777	0.01007	2.26716	0.00934	0.00610	0.00724	0.01041	1.41864	0.00177	0.00000	0.03543	0.01300	0.00518	1.17944	0.00678
**Gm16702**	2.97168	0.43823	2.38600	18.71433	1.68772	0.33651	0.52783	0.00000	21.32065	0.89064	16.36592	1.85119	1.16961	8.94339	6.19440	0.61164
**Gm16740**	0.19514	0.00859	0.00000	0.06431	0.01745	0.37275	0.00739	0.01194	0.01139	0.03455	0.01273	0.00807	0.00861	0.01160	0.00908	0.00728
**Gm16796**	0.08004	0.00862	0.00922	0.01057	0.01575	0.00683	0.00750	0.01311	0.01239	0.02324	0.01269	0.00810	0.01238	0.01200	0.01077	0.00745

**Gm16845**	0.01167	0.01963	0.01266	1.51295	0.00996	0.00729	0.00765	0.01521	0.63099	0.11394	0.02653	0.04418	0.29715	0.04381	0.01014	0.00737
**Gm16853**	0.01325	0.00828	0.01140	3.42336	0.00922	0.00624	0.00743	0.01297	3.07150	0.09791	0.00000	0.00263	0.01333	0.00765	0.00520	0.00669
**Gm16907**	0.01370	0.00820	0.01216	0.40984	0.00929	0.00660	0.00752	0.01395	0.35105	0.01344	0.01110	0.00779	0.01277	0.01156	0.00421	0.00733
**Gm16938**	0.34929	0.01923	0.14504	8.06829	0.03922	0.12493	0.20701	0.60597	8.39016	1.43012	0.21016	0.42244	0.84692	1.46512	1.94573	0.07209
**Gm16973**	1.76361	1.89810	0.09137	6.02257	0.00454	0.00389	0.00600	0.13687	7.11878	0.79261	0.00000	0.03445	0.04021	0.00000	0.00000	0.01844
**Gm17019**	0.01098	0.00799	0.13687	0.06744	0.00927	0.00585	0.00755	0.06260	0.13622	0.06366	0.01023	0.01905	0.00933	0.00747	0.13504	0.00741
**Gm17026**	0.02892	0.00780	0.03229	0.07937	0.00866	0.00614	0.01806	0.01256	0.12873	0.01197	0.04882	0.00748	0.01381	0.01138	0.02120	0.00736
**Gm17066**	1.07016	0.69489	0.00000	3.59701	0.00670	1.49551	0.04540	0.48664	1.74270	0.00000	1.49211	5.78934	0.62364	5.43913	0.00000	0.00358
**Gm17644**	2.52132	0.33520	0.76750	0.00000	0.00966	2.08783	0.31638	1.06354	0.12058	7.54545	0.03408	0.09181	1.91348	4.69984	1.49757	2.25430
**Gm17751**	0.01352	0.00753	0.01066	3.56338	0.00924	0.00613	0.00715	0.00973	0.00000	0.19714	0.02674	0.00369	0.01264	0.00307	1.42304	0.00720
**Gm17762**	0.06727	0.00773	0.11629	0.00755	0.00777	0.00565	0.00711	0.02019	0.01194	0.00537	0.13139	0.00704	0.25303	0.01182	0.00628	0.06294
**Gm17801**	0.03921	0.02993	0.10232	0.05724	0.04846	0.01186	0.01844	0.00462	0.09563	0.02337	0.48400	0.03650	0.03939	0.15380	0.12738	0.02183
**Gm1821**	34.48891	23.04935	34.23132	15.95158	23.73089	10.88701	19.99742	22.05799	27.98440	20.56178	18.62087	10.16974	29.90762	4.51031	16.30327	18.82883
**Gm1943**	1.55131	2.89510	6.33049	10.59831	7.04879	5.45467	2.55760	6.65308	11.76463	11.76929	8.21172	4.06955	4.15280	5.89493	8.17651	1.54350
**Gm19434**	0.16474	0.03820	0.45116	3.02987	0.01702	0.01190	0.33291	0.05896	2.59524	0.44572	0.33516	0.15340	0.04529	0.56329	0.82565	0.05643
**Gm19510**	1.62506	0.00609	0.01103	0.06032	0.00394	0.01278	0.00630	0.00899	0.05290	0.00535	0.01259	0.00722	0.00651	0.01306	0.01284	2.40461
**Gm19557**	0.01375	0.00807	0.01225	0.01058	0.00932	0.00666	0.00754	0.01389	0.01234	0.01523	0.01311	0.00815	0.01270	0.01220	0.01187	0.00741
**Gm19583**	0.01589	0.00844	0.90592	0.34446	0.00739	0.00574	0.00751	0.01151	0.00588	0.06687	0.00798	0.00708	0.00875	0.22166	0.72678	0.00694
**Gm19589**	0.09727	0.09033	0.07564	0.98009	0.00779	0.01224	0.00727	0.06410	0.88403	0.11692	0.09778	0.02468	0.01884	0.02501	0.08100	0.06637
**Gm19619**	0.01758	0.00800	0.05165	0.02657	0.01116	0.00765	0.18254	0.01153	0.04105	0.01487	0.02456	0.00678	0.02642	0.14296	0.06073	0.00757
**Gm1965**	0.01021	0.00736	0.00000	22.38147	0.00866	0.00365	0.00592	0.00000	11.62217	0.80684	1.01768	0.01395	0.00873	0.27952	2.19428	0.00241
**Gm19705**	0.45410	0.00789	0.03866	0.57914	0.00905	0.00823	0.00725	0.03012	0.61993	0.01328	0.51862	0.00421	0.01228	0.00874	0.03416	0.00684
**Gm19710**	0.01100	0.00784	0.01089	0.04126	0.00982	0.00738	0.24868	0.01359	0.04948	0.14543	0.01121	0.00672	0.09580	0.26606	0.07895	0.12953
**Gm19757**	0.71461	1.72716	2.63034	3.68673	0.02528	1.44900	1.87768	2.97167	2.47140	6.87279	0.48555	0.41725	1.50816	11.68869	6.74451	0.52676
**Gm1976**	0.08047	0.01916	0.09118	0.37545	0.02057	0.00692	0.00721	0.02841	0.49126	0.08312	0.65582	0.01165	0.05258	0.43935	0.10984	0.01434
**Gm19784**	0.01380	0.00800	0.01166	0.62585	0.00934	0.00653	0.00739	0.01174	0.00000	0.01263	0.02884	0.01783	0.01256	0.00570	0.29745	0.00741
**Gm1995**	0.03653	0.00778	0.59976	19.33900	0.00703	0.00111	0.01785	0.46462	27.73276	1.76127	0.83216	0.06566	0.03233	0.00000	4.02845	0.02393
**Gm2011**	0.00957	0.06446	0.01685	0.05238	0.00896	0.00724	0.05206	0.05108	0.09135	0.00778	0.01124	0.00835	0.22343	0.01104	0.02226	0.02146
**Gm20110**	0.01736	0.00798	0.02924	0.00955	0.01060	0.00717	0.00736	0.02295	0.02360	0.05155	0.01370	0.00785	0.01126	0.01386	0.01348	0.00732
**Gm20125**	0.02638	0.04117	0.03256	0.01595	0.01077	0.00610	0.01796	0.01964	0.02392	0.09006	0.01191	0.01769	0.02099	0.11386	0.09632	0.00751
**Gm20139**	0.01379	0.00800	0.02920	0.01667	0.00929	0.00608	0.01789	0.01070	0.02467	0.03709	0.09403	0.00745	0.05552	0.03939	0.05266	0.01454
**Gm2016**	0.04402	0.05074	1.13735	8.07685	0.01648	0.00374	0.04839	0.02445	10.72129	0.32216	0.00000	0.49703	0.29558	1.74310	0.93477	0.02731
**Gm20172**	0.00364	0.00728	0.07805	1.05678	0.00899	0.00784	0.00666	0.42682	0.71573	1.90200	1.06798	1.08933	0.15042	0.87697	1.66118	0.02132
**Gm20187**	0.22869	0.00749	0.03214	0.21203	0.00845	0.03113	0.00720	0.06084	0.13277	0.00961	0.02520	0.00684	0.07771	0.05274	0.05887	0.04364
**Gm20199**	0.08888	0.00784	0.02479	0.00655	0.00834	0.00832	0.01806	0.01418	0.02197	0.55141	0.02836	0.00615	0.18566	0.05022	0.24684	0.00737
**Gm20219**	0.01371	0.00781	0.00647	0.14064	0.00896	1.14313	0.00726	0.01479	0.49580	0.99488	0.00613	0.00445	0.00542	0.01244	0.01675	0.00702
**Gm2022**	0.17537	0.02938	0.13930	11.86403	0.02595	0.02083	0.01751	0.03758	11.84379	0.14533	0.00000	0.00000	0.04697	0.45547	1.74302	0.01905
**Gm2027**	0.03568	0.04983	0.08284	4.85934	0.11954	0.09925	0.03776	0.14681	6.58032	0.26225	0.30294	1.47960	0.23015	0.86403	0.72623	0.02060
**Gm20300**	0.47983	2.72421	0.85958	0.05363	0.03860	2.83537	0.04571	0.40778	0.13652	0.53514	0.03367	0.17777	0.00000	1.17380	0.00000	1.63912
**Gm20324**	0.01362	0.00813	0.01282	0.01059	0.00967	0.00693	0.00767	0.01506	0.01231	0.01773	0.01371	0.01868	0.02318	0.01219	0.01056	0.00758
**Gm20337**	0.19337	0.02792	0.86097	3.35327	0.09076	0.02324	0.03640	0.10648	2.50889	0.45405	2.46414	0.09363	0.21899	0.61580	0.57246	0.09819
**Gm20356**	0.01751	0.00842	0.01348	3.54168	0.00906	0.01256	0.00817	0.34801	0.00000	0.00000	0.00950	0.00745	0.01748	0.10709	0.22703	0.00684
**Gm20362**	22.90131	7.88086	5.87447	0.54917	9.98188	6.03175	2.95395	5.68934	0.34929	0.39304	0.46003	6.98785	5.22895	1.97297	0.08221	17.70128
**Gm2042**	0.00000	0.00762	0.03392	17.27647	0.00915	0.00457	0.00635	0.18313	6.59393	3.12116	0.19675	0.15477	0.00599	7.04153	14.61657	0.00380
**Gm20594**	9.68874	11.12449	7.87143	15.03884	3.96011	3.09272	8.01756	18.56727	11.46087	19.69037	12.03269	12.91331	3.20458	38.98987	29.74824	2.26549
**Gm20597**	0.03297	0.04072	0.02693	0.18470	0.02059	0.00607	0.00739	0.01137	0.22302	0.00596	0.01032	0.00700	0.01246	0.15847	0.07841	0.00739
**Gm20605**	0.03433	0.00819	0.01116	0.00994	0.00839	0.24724	0.07277	0.04444	0.01232	0.11404	0.01198	0.00785	0.06100	0.01234	0.00966	0.00734
**Gm20611**	0.01290	0.00766	0.00822	0.00755	0.00930	0.00690	0.00715	0.23693	0.02607	0.00665	0.08686	0.00670	0.00639	0.01233	0.00549	0.00735
**Gm20750**	0.01275	0.00780	0.00742	0.81113	0.01823	0.00644	0.00717	0.00462	0.00000	0.39328	0.37100	0.03416	0.00794	0.00412	0.99591	0.00688
**Gm20752**	0.03696	0.00794	0.04933	0.34657	0.00819	0.38133	0.00732	0.01594	0.41617	0.09489	0.07041	0.04880	0.01512	0.01057	0.01484	0.08567
**Gm20759**	0.01373	0.00831	0.01211	1.14774	0.00932	0.00656	0.00747	0.01350	0.06636	0.01373	0.01279	0.00812	0.01275	0.03306	0.00159	0.00723
**Gm20767**	0.07014	0.04874	0.26756	40.23747	0.02803	0.08835	0.22601	1.57714	40.81483	9.01722	3.89969	1.97196	0.82831	9.25575	16.01234	0.03288
**Gm20939**	3.65985	5.18758	2.37758	1.28014	1.48500	1.81878	3.28242	5.80663	2.42843	6.20031	5.74126	2.56888	1.78047	21.36329	7.12944	0.88131
**Gm21119**	0.01374	0.00800	0.01229	0.26654	0.00933	0.00661	0.00752	0.01378	0.10078	0.01316	0.01241	0.00778	0.01269	0.00926	0.04006	0.00736
**Gm21190**	0.01528	0.00795	0.09629	0.10561	0.00890	0.00599	0.00751	0.03570	0.25229	0.08797	0.01017	0.00735	0.01065	0.00814	0.11391	0.00749
**Gm21283**	2.17247	0.00557	0.01150	0.01031	0.19481	0.00687	0.12217	0.00852	0.01230	0.01240	0.01441	0.00600	0.06286	0.01128	0.01579	0.03565
**Gm21284**	0.22544	0.00758	0.24869	0.09871	0.00912	0.06299	0.02732	0.00075	0.48770	0.12350	0.94117	0.06817	0.20139	0.47636	0.11155	0.00678
**Gm21319**	0.03522	0.02921	0.08437	6.91073	0.00881	0.01192	0.04791	0.03805	5.38534	0.03617	0.21703	0.00000	0.04952	1.15900	1.54528	0.03519
**Gm21637**	0.22586	0.00764	0.00968	0.53979	0.00750	0.00632	0.00744	0.01959	1.15309	0.14080	0.00000	0.03533	0.01217	0.00795	0.20447	0.00697
**Gm2381**	0.75958	1.65833	0.72289	1.33075	0.16032	0.01080	0.40481	0.37907	5.52971	2.45781	1.36931	0.41206	0.88320	4.05131	2.44130	0.03313
**Gm2518**	0.06635	0.00768	0.71289	0.01942	0.00683	0.00563	0.00730	0.01310	0.78674	0.03394	0.00663	0.00657	0.01536	0.01303	0.00400	0.01407
**Gm2560**	0.01344	0.00829	0.08210	0.24372	0.00928	1.98094	0.00792	0.11268	0.00533	0.00595	0.00831	0.01921	0.00000	0.00801	0.08497	0.00647

**Gm26705**	0.38771	0.00757	0.00981	0.17845	0.00720	0.00642	0.03821	0.03198	0.26295	0.03973	0.00894	0.05968	0.02254	0.17312	0.09381	0.00682
**Gm2694**	0.16646	0.00849	0.00617	5.01813	0.01131	0.00660	0.00736	0.01523	4.22389	0.71648	0.00000	0.03249	0.02125	0.00000	0.13540	0.00627
**Gm28042**	0.01373	0.00813	0.01219	0.01041	0.00927	0.00660	0.01799	0.01332	0.01243	0.05663	0.01225	0.00792	0.01204	0.01153	0.01071	0.00723
**Gm2837**	0.41446	0.00745	0.01468	1.99211	0.00712	0.02339	0.00726	0.01736	3.37402	0.20018	0.01709	0.08154	0.01142	0.02738	0.41488	0.00637
**Gm2897**	0.10619	0.00789	0.01775	0.39169	0.00784	0.00625	0.00732	0.01387	0.00516	0.01758	0.01335	0.00776	0.01259	0.04913	0.00590	0.00723
**Gm29443**	0.03713	0.01815	0.03749	0.38743	0.00889	0.00669	0.01734	0.01269	0.66999	0.02245	0.30027	0.02337	0.02527	0.27883	0.07273	0.01469
**Gm29683**	0.05751	0.01866	0.01920	0.04761	0.00784	0.00801	0.16485	0.03866	0.13638	0.01429	0.04353	0.00672	0.04879	0.36523	0.03405	0.00718
**Gm29811**	0.01288	0.00784	0.00994	5.60137	0.00899	0.00567	0.00705	0.00839	4.57095	0.51462	0.00000	0.00000	0.01250	0.00000	0.72422	0.00659
**Gm29968**	0.16421	0.15119	0.04827	0.21112	0.02396	0.02186	0.02719	0.14132	0.07586	0.12465	0.00699	0.00491	0.02046	0.58397	0.18833	0.02828
**Gm2a**	2.68469	4.26961	15.94398	4.72191	2.06555	16.55269	5.76842	14.54239	5.84442	13.87511	22.10944	3.37899	19.82008	14.57867	10.53411	5.92115
**Gm3002**	0.05378	0.00817	0.00977	0.24197	0.00810	0.00690	0.00747	0.01465	0.09401	0.02091	0.01293	0.00782	0.01491	0.05323	0.01008	0.10296
**Gm30505**	0.01352	0.00807	0.01217	0.25455	0.00935	0.00663	0.00746	0.01913	0.00639	0.01525	0.01332	0.00810	0.01232	0.01130	0.00688	0.00737
**Gm30551**	0.01367	0.01839	0.07380	0.42257	0.00932	0.00607	0.01769	0.02173	0.19762	0.06236	0.06178	0.00726	0.02310	0.01158	0.03826	0.00717
**Gm3086**	0.02039	0.00781	0.01275	0.01862	0.00941	0.00703	0.00772	0.01479	0.02476	0.01029	0.01324	0.00764	0.04397	0.01958	0.08027	0.00763
**Gm3139**	17.03241	11.39869	24.53123	21.11049	12.32468	7.56475	13.22957	25.66089	21.26907	32.89178	22.80720	16.35447	21.60369	37.86133	27.21709	2.58295
**Gm3143**	0.42866	0.00733	0.42245	6.46713	0.00652	0.00453	0.03802	0.04956	0.79395	0.07541	0.04624	0.04580	0.02520	0.01580	0.30605	0.02602
**Gm3219**	0.74158	0.74883	0.42573	13.44827	0.55161	0.01857	0.00584	0.16991	3.88202	0.99262	0.83799	0.03555	0.12504	0.00000	0.40869	0.03376
**Gm32511**	0.53431	0.00735	0.00000	0.00709	0.00674	0.02707	0.00615	0.00861	0.02243	0.54887	0.00957	0.00439	1.39562	0.16607	0.20564	0.00619
**Gm3259**	0.01317	0.00782	0.01071	3.56421	0.00937	0.00623	0.00733	0.01134	0.81045	0.18245	0.02487	0.00319	0.04336	0.02284	1.17500	0.00695
**Gm3264**	0.04614	0.00806	0.00986	0.01027	0.00811	0.00682	0.00738	0.01329	0.01231	0.04538	0.01309	0.00759	0.02035	0.05243	0.01215	0.00753
**Gm3286**	0.98422	1.06531	3.32707	9.50469	1.08215	0.71837	1.78197	5.10870	8.96429	8.54295	7.78006	4.51958	3.91209	14.58471	9.76655	0.18080
**Gm3336**	0.01345	0.00811	0.00621	0.00427	0.00952	0.00747	0.00748	0.00600	0.01107	0.06133	1.42519	0.00080	0.00282	0.00408	0.10368	0.00733
**Gm3383**	0.24920	0.00775	0.00569	0.98590	0.00732	0.00712	0.00714	0.01204	0.00000	0.01857	0.02592	0.01758	0.01968	0.11782	0.00000	0.01435
**Gm3414**	0.02108	0.01818	0.30010	0.08487	0.01105	0.00830	0.00707	0.00047	0.03858	0.00984	0.34721	0.04205	0.11889	0.05086	0.57468	0.01463
**Gm3435**	0.08350	0.41342	1.06171	0.21023	0.00624	0.00704	0.32141	0.16688	0.00778	0.13166	0.15616	0.00591	0.29370	0.00838	0.41761	0.79217
**Gm3500**	0.16499	0.00783	0.00980	0.50828	0.00755	0.00664	0.00727	0.01338	0.01036	0.01948	0.01283	0.00790	0.02686	0.06573	0.00389	0.00711
**Gm3558**	0.12290	0.00786	0.01560	0.62670	0.00775	0.00631	0.00724	0.01210	0.00418	0.03678	0.03987	0.01707	0.01235	0.08756	0.00000	0.00730
**Gm3604**	0.68962	7.81834	2.05395	2.52207	0.74129	1.43497	0.98077	0.44122	2.96918	0.72634	0.34426	6.36051	0.92987	4.59224	0.52508	0.07369
**Gm3636**	0.04308	0.00791	0.03233	1.00557	0.00828	0.00597	0.00729	0.01248	0.02725	0.01975	0.02665	0.00753	0.01223	0.08114	0.00000	0.00725
**Gm364**	0.00814	0.00748	2.07109	0.59059	0.00630	0.00767	0.00648	0.53008	0.00000	0.24977	1.08947	0.01168	0.00000	2.11697	0.69953	0.00674
**Gm3696**	0.08561	0.00802	0.00866	0.26289	0.00784	0.00690	0.00737	0.01399	0.05795	0.03194	0.01280	0.00747	0.01299	0.05249	0.00458	0.00742
**Gm3776**	0.50526	0.00699	0.00000	0.01940	0.00676	0.40591	0.07801	0.76137	0.01836	0.83900	1.16862	0.06961	0.85847	1.19590	0.00000	0.00595
**Gm38407**	0.01368	0.00825	0.01214	1.03712	0.00933	0.00655	0.00750	0.01391	0.43336	0.01248	0.00984	0.00767	0.01280	0.01135	0.00360	0.00722
**Gm38415**	0.02147	0.00741	0.02624	0.80280	0.00885	0.00596	0.03747	0.02560	1.12211	0.30402	0.00000	0.08269	0.01366	0.06582	1.42641	0.00755
**Gm38416**	0.02000	0.00753	0.01717	0.95733	0.00881	0.00710	0.00733	0.01611	0.00000	0.00720	0.01168	0.00714	0.01930	0.01338	0.25986	0.00718
**Gm38426**	0.33308	0.00766	0.60204	0.01088	0.01314	0.00526	0.00689	0.00842	0.01214	0.02190	0.00977	0.22813	0.00891	0.01337	0.00000	0.00685
**Gm3893**	0.00923	0.00739	0.01097	0.76980	0.00892	0.00637	0.00669	0.00806	0.06688	0.95417	0.01117	0.00463	0.86837	0.10239	0.10520	0.00659
**Gm40893**	0.12127	0.05347	0.06391	0.07638	0.78991	0.07777	0.01747	0.02801	0.17467	0.01654	0.16151	0.01523	0.11824	0.30938	0.03902	0.11622
**Gm4224**	0.01363	0.00815	0.01184	0.10181	0.00925	0.00664	0.00747	0.01285	0.20982	0.01557	0.01147	0.00764	0.01993	0.03516	0.01806	0.01482
**Gm4262**	0.94711	1.56454	7.00913	3.69007	0.51436	2.10652	2.63797	9.44309	9.04340	10.36186	6.38364	10.88127	1.39302	7.45071	4.44472	0.76469
**Gm4285**	0.08927	0.00763	0.10191	0.16031	0.01765	0.00615	0.01739	0.00788	0.36829	0.01478	0.57231	0.04810	0.06122	1.63948	0.07667	0.02169
**Gm4349**	0.81326	0.00631	1.09343	3.39785	0.00453	0.00594	0.00621	0.19166	3.12280	1.17476	0.03262	0.08533	0.00921	1.60089	0.91651	0.00518
**Gm4371**	0.72320	0.00738	0.00280	0.01939	0.01695	0.00777	0.00653	0.36865	0.01221	0.00893	0.01104	0.00631	0.00614	0.01544	0.01073	0.00686
**Gm4425**	0.21377	0.08125	0.60197	0.01883	0.02798	0.16283	0.12813	0.42460	0.25738	0.16320	0.70215	0.02305	0.43767	0.18488	0.28065	0.11255
**Gm4432**	0.01835	0.00773	0.00885	0.00827	0.01079	0.00667	0.00727	0.01000	0.01192	0.00269	0.02266	0.00520	0.01295	0.41326	0.32287	0.00751
**Gm4532**	0.01817	0.00836	0.01159	0.76182	0.01065	0.00645	0.00745	0.01324	1.47161	0.03977	0.00000	0.00539	0.01316	0.01039	0.00319	0.00731
**Gm4541**	0.01521	0.10499	0.00968	0.08627	0.01006	0.00674	0.02758	0.01077	0.02923	0.01036	0.01248	0.00668	0.01265	0.25042	0.00252	0.00743
**Gm4566**	0.01378	0.00786	0.01203	0.15284	0.00932	0.00664	0.00751	0.01350	0.00921	0.01387	0.01338	0.00793	0.01272	0.01694	0.02967	0.00739
**Gm4567**	0.01920	0.00855	0.02875	0.06572	0.00875	0.00613	0.00805	0.12275	0.15397	0.03642	0.05033	0.00733	0.10170	0.13155	0.09210	0.00740
**Gm4598**	0.01565	0.00759	0.13544	0.01065	0.00906	0.00575	0.00738	0.01206	0.01172	0.00869	0.01195	0.00738	0.01090	0.01964	0.14837	0.00729
**Gm4737**	17.15883	7.52888	14.10380	0.00000	12.34763	4.26249	10.53438	12.00802	0.00000	3.47339	1.91495	0.12638	11.05587	0.12120	0.78131	6.15565
**Gm4791**	0.01356	0.00808	0.01236	0.06459	0.00979	0.00692	0.00758	0.01290	0.04265	0.02204	0.03958	0.00705	0.02550	0.07916	0.03839	0.00766
**Gm4827**	0.01722	0.00836	0.01594	0.74521	0.00889	0.00642	0.00763	0.01436	1.72004	0.07801	0.01322	0.00619	0.01304	0.01067	0.00431	0.00717
**Gm4832**	0.09058	0.00865	0.12102	0.08821	0.09652	0.02353	0.03028	0.09616	0.96743	0.01722	0.87118	0.00298	0.05604	0.08644	0.12238	0.05148
**Gm4841**	0.05194	0.02863	0.04931	0.04146	0.03390	0.05618	0.00743	0.11618	0.08712	0.08271	0.12905	0.13069	0.05386	0.05304	0.05829	0.00719
**Gm4850**	0.01037	0.00760	0.06773	12.41640	0.00880	0.00404	0.00682	0.00004	5.80358	0.97634	0.19394	0.01452	0.00932	0.00891	4.78509	0.01191
**Gm4858**	0.01233	0.00792	0.00937	1.69477	0.00940	0.00626	0.00723	0.00924	0.82313	0.14145	0.02953	0.02153	0.01212	0.88211	0.45219	0.00702
**Gm4871**	0.01365	0.00829	0.01164	0.47830	0.00928	0.00676	0.00752	0.01332	0.20683	0.02870	0.01199	0.00810	0.01316	0.07543	0.00000	0.00734
**Gm4922**	0.16883	0.05220	0.10389	0.00880	0.01617	0.01265	0.00757	0.06928	0.01215	0.05594	0.04707	0.05010	0.04079	0.18779	0.04167	0.19568
**Gm4925**	0.03221	0.01840	0.21961	0.03074	0.03507	0.03088	0.02822	0.03693	0.60731	0.05600	0.68968	0.06275	0.08781	0.16656	0.08292	0.02864
**Gm4926**	0.00000	0.62659	2.03124	0.65440	1.78411	2.00495	0.16248	0.62187	0.09497	0.40960	0.36011	0.23327	0.72361	0.61845	0.30498	1.17655
**Gm4951**	0.04321	0.01794	0.16421	0.08251	0.01764	0.01306	0.01692	0.02260	0.14055	0.03788	0.64246	0.04009	0.18861	0.50230	0.04616	0.04355

**Gm4956**	0.01313	0.00803	0.01055	0.00356	0.00916	0.00637	0.00725	0.01152	0.67203	0.00154	0.00696	0.05113	0.13108	0.00758	0.27717	0.00711
**Gm4961**	1.10958	0.10750	0.00000	5.44137	0.42814	0.41421	0.01628	3.87109	1.22638	4.80116	1.78169	0.87961	1.14355	2.21785	4.41469	0.00441
**Gm4971**	0.01236	0.02908	0.01658	1.40912	0.00939	0.00624	0.00758	0.02221	0.85629	0.00437	0.00659	0.00605	0.01466	0.00879	0.25900	0.01437
**Gm4984**	0.38299	0.00740	0.01130	0.22917	0.00687	0.00731	0.00647	0.01213	0.16349	0.00000	0.06072	0.02618	1.22536	0.00622	0.17201	0.01299
**Gm5**	0.01064	0.00778	0.00492	9.69642	0.00871	0.00427	0.00672	0.00000	18.03860	0.61231	0.25515	0.00000	0.00959	0.24754	0.77619	0.00467
**Gm5039**	0.11052	0.39781	0.32318	11.60741	0.15661	0.11667	0.16201	0.40330	11.02799	0.40671	0.00000	0.00000	0.10931	1.64044	0.83105	0.20289
**Gm5065**	0.04693	0.00794	0.00571	0.56805	0.00805	0.00727	0.00743	0.04239	0.15092	0.28972	0.04804	0.26149	0.01049	0.00699	0.14812	0.00721
**Gm5069**	5.72260	2.64225	2.93836	0.29186	4.25927	2.87021	1.68389	0.14582	0.51062	0.13209	0.00703	0.12976	2.87531	0.00811	0.15142	5.55592
**Gm5071**	0.01259	0.00826	0.01074	0.00983	0.00939	0.00670	0.00798	0.04318	0.01233	0.00951	0.01173	0.06128	0.01092	0.00729	0.11951	0.00738
**Gm5072**	0.01074	0.00845	0.00957	0.00960	0.00952	0.00675	0.00819	0.14607	0.01218	0.01329	0.01052	0.04121	0.00768	0.00693	0.16182	0.00729
**Gm5088**	10.81589	0.64443	2.20219	11.16710	8.79722	5.42338	4.29405	20.25354	9.62789	29.62865	21.38486	3.16592	25.94093	3.33048	2.94979	4.31739
**Gm5113**	0.15934	0.05105	0.60790	0.74085	0.06992	0.05942	0.11118	0.15222	0.94163	0.00003	0.82132	0.02774	1.14704	0.94110	1.20575	0.05604
**Gm5114**	0.01375	0.00789	0.01239	0.01005	0.00933	0.00661	0.00752	0.01366	0.01247	0.01231	0.01298	0.00764	0.01266	0.00756	0.07766	0.00739
**Gm5127**	0.01226	0.00739	0.54131	0.90631	0.00823	0.00880	0.00703	0.00955	0.00000	0.00326	0.00837	0.41285	0.00926	0.00477	0.12152	0.00704
**Gm5129**	0.01595	0.00829	0.01227	0.39289	0.01021	0.00672	0.00755	0.01459	0.13714	0.02454	0.01375	0.00824	0.04435	0.01177	0.00516	0.19038
**Gm5134**	0.24620	0.02794	0.25164	3.04685	0.05942	0.95410	0.00616	1.56618	2.73442	1.04321	0.80275	6.41180	0.00000	3.18137	2.02055	0.00550
**Gm5141**	0.30506	0.76666	0.75118	1.76060	0.12270	0.15332	0.45692	0.47062	0.99802	0.22163	0.82841	1.05682	0.11294	2.14794	0.95136	0.10720
**Gm5148**	24.87101	2.86325	17.40314	8.65704	15.75061	6.39201	3.88464	7.12456	18.19470	24.06428	32.98443	2.68374	16.70704	4.49144	7.90348	10.62623
**Gm5176**	1.33786	0.72263	1.51784	0.65800	1.35790	0.41497	0.85279	1.23807	2.86801	1.69146	0.58761	0.15450	0.87957	2.62850	0.92565	0.62545
**Gm5177**	2.74776	1.27211	1.29802	4.00280	2.20761	1.34547	0.73424	0.05690	1.19857	0.07688	0.09223	0.03437	1.34808	0.00000	0.34260	2.90459
**Gm525**	0.01366	0.00783	0.01128	0.26662	0.00914	0.00664	0.00730	0.01102	0.04261	0.31280	0.01261	0.04549	0.01254	0.00565	0.19627	0.00772
**Gm527**	2.42715	0.00556	0.00016	6.44729	0.01130	0.00560	0.00565	0.11792	1.03941	1.31873	0.38136	0.00970	0.00570	0.05559	0.81924	0.00351
**Gm53**	0.65934	0.00702	0.00477	0.01061	0.00658	0.00712	0.00708	0.01107	0.01238	0.01911	0.01253	0.01804	0.01185	0.01220	0.01066	0.00663
**Gm5415**	0.02481	0.00750	0.00922	0.00911	0.00897	0.00712	0.00738	0.18381	0.02670	0.02769	0.01360	0.01701	0.01706	0.01443	0.03598	0.00742
**Gm5431**	0.05995	0.00841	0.06985	0.06465	0.00905	0.00758	0.00788	0.03094	0.04631	0.09558	0.01199	0.02975	0.01716	0.00923	0.06226	0.01523
**Gm5434**	0.22248	0.10985	0.63358	2.13061	0.30882	0.21735	0.02877	0.27882	0.00000	0.29801	0.58902	0.01453	0.44644	0.13768	0.00000	0.18819
**Gm5458**	0.01506	0.00798	0.03597	0.63066	0.00916	0.00599	0.00719	0.00997	0.19760	0.12710	0.01040	0.01573	0.01996	0.33467	0.00721	0.00758
**Gm5475**	0.01303	0.00803	0.01781	0.19471	0.00925	0.00745	0.00745	0.01185	0.31669	0.36705	0.00869	0.00592	0.01481	0.05573	0.22984	0.00757
**Gm5485**	0.01270	0.00877	0.01086	0.00511	0.00922	0.00668	0.00802	0.11746	0.01094	0.08126	0.35061	0.00698	0.00632	0.00704	0.12142	0.00752
**Gm5512**	0.51836	1.19686	0.45900	1.55188	0.07137	1.12115	1.23661	0.01148	0.32494	0.58492	0.46135	0.01192	0.30752	0.00337	0.40947	1.01144
**Gm5523**	7.61272	3.00540	4.05085	0.93688	6.29213	3.72760	2.13292	0.14582	0.82819	0.30501	0.00000	0.35155	3.67797	0.00401	0.16996	6.75061
**Gm5532**	0.01164	0.00785	0.00750	0.12245	0.00892	0.00649	0.00741	0.01276	0.00599	1.87749	0.00307	0.03128	0.01082	0.48226	1.26980	0.00734
**Gm5547**	0.09692	0.00782	0.00477	0.00480	0.00762	0.00747	0.00784	0.06556	0.01087	0.29916	0.69373	0.01750	0.00444	0.07155	0.00000	0.00686
**Gm5577**	0.02903	0.00815	0.03964	2.98933	0.00841	0.00738	0.01793	0.02285	0.38254	0.14223	0.04810	0.00765	0.02383	0.05291	0.01884	0.00687
**Gm5607**	0.15020	0.00750	0.34919	0.30802	0.15185	0.03861	0.13750	0.08262	0.62715	0.19688	0.54754	0.04864	0.21255	0.62825	0.15779	0.05071
**Gm561**	1.36698	0.02598	0.32707	3.69782	0.91489	0.24286	0.00474	0.05241	4.63462	0.49619	2.86602	0.17770	0.18791	1.20794	1.56828	0.07180
**Gm5617**	0.08792	0.00767	0.01496	0.15215	0.00819	0.00637	0.00690	0.13691	0.00949	0.36178	0.11032	0.00548	0.41941	0.00996	0.00337	0.02213
**Gm5622**	0.02371	0.28213	0.04665	1.09656	0.01355	0.23563	0.35541	0.43795	0.14602	0.06806	0.01906	0.26984	0.00000	0.13592	0.41659	1.53606
**Gm5640**	0.67989	0.00691	0.00314	0.46831	0.01062	0.00767	0.37052	0.18277	0.00544	0.05616	0.01324	0.00578	0.10114	0.00681	0.04170	0.01443
**Gm5643**	9.35542	6.48127	7.41474	1.44955	5.14794	3.86488	5.76365	5.74120	5.57768	8.62594	11.14838	0.77143	6.24027	22.75800	11.10803	4.74243
**Gm5662**	0.05796	0.19916	1.44466	11.88683	0.09995	0.01476	0.07876	0.12197	13.18712	0.42275	0.07749	0.62779	0.28161	2.30129	1.49127	0.01155
**Gm5712**	0.05405	0.01822	0.05938	0.00989	0.04977	0.02416	0.05817	0.05820	0.01235	0.01943	0.02591	0.00729	0.04102	0.01007	0.02004	0.02930
**Gm572**	0.01066	0.00773	0.00977	0.00694	0.00962	0.00673	0.00721	0.16146	0.06743	0.01186	0.01165	0.00726	0.00757	0.01285	0.00758	0.00740
**Gm5766**	0.40774	0.24735	0.17447	0.02640	0.20225	0.08033	0.13225	0.30678	0.17762	0.05827	0.56338	0.05588	0.18129	0.19828	0.11255	0.26681
**Gm5779**	1.64211	0.62562	1.28944	0.00179	0.63763	0.67845	1.27068	2.03583	0.02924	1.50818	0.00000	0.40515	1.70402	0.00605	0.02411	1.05463
**Gm5795**	0.01791	0.00809	0.01067	0.36510	0.00890	0.00672	0.00734	0.01108	0.10525	0.05708	0.03960	0.01675	0.01259	0.10795	0.00742	0.00763
**Gm5796**	0.08939	0.00789	0.00883	0.22412	0.00783	0.00687	0.00741	0.01479	0.02261	0.01819	0.01328	0.00797	0.01276	0.02901	0.00676	0.00728
**Gm5797**	0.01428	0.00801	0.01882	1.44010	0.00918	0.00612	0.00717	0.00987	0.74702	0.12281	0.00716	0.00489	0.02035	0.30856	0.00000	0.00747
**Gm5801**	0.76983	0.00593	0.12290	3.27266	1.21133	0.10146	0.49147	2.04065	3.58594	3.80610	1.51650	0.14894	0.70660	0.00000	0.00000	1.17053
**Gm5803**	1.51095	2.53480	2.17876	0.04914	1.10201	1.50205	2.54765	2.91867	0.31813	1.33015	0.32637	0.00627	1.28760	16.31594	3.81199	1.64406
**Gm5820**	0.01348	0.00778	0.01082	0.39928	0.00938	0.00654	0.00741	0.01167	0.66016	0.00555	0.00619	0.00563	0.02905	0.14112	0.21468	0.00723
**Gm5860**	2.10840	0.66286	3.13520	3.05091	0.49809	0.42566	1.72756	0.60247	4.03498	1.20754	1.55603	0.92977	0.56235	0.74804	0.45561	2.21160
**Gm5861**	0.02255	0.00787	0.05106	0.05800	0.00852	0.00718	0.00740	0.01028	0.17200	0.00733	0.15761	0.01701	0.01007	0.00869	0.02200	0.00723
**Gm5868**	0.01349	0.00784	0.01107	0.21568	0.00944	0.00664	0.00751	0.01398	0.00588	0.00616	0.01082	0.00678	0.01288	0.17411	0.14349	0.00741
**Gm5907**	0.01093	0.00722	1.04588	0.00563	0.00704	0.00921	0.00643	0.12276	0.01117	0.28550	0.37384	0.00312	0.76878	0.01192	0.00000	0.68376
**Gm5936**	0.65853	0.00677	0.00264	0.38453	0.00986	0.00762	0.43089	0.24267	0.01707	0.04888	0.01215	0.00564	0.13287	0.00716	0.03744	0.03642
**Gm5941**	0.01308	0.00800	0.01717	1.91990	0.00906	0.00683	0.00738	0.00990	2.60807	0.04187	0.10658	0.00282	0.01051	0.01037	0.00795	0.00669
**Gm595**	0.00925	0.00840	0.00455	0.00507	0.00957	0.00770	0.00797	0.29754	0.01070	0.00000	0.71053	0.06114	0.00292	0.01211	0.00000	0.00710
**Gm6083**	20.37422	4.71217	14.82164	6.49520	12.14688	5.66979	3.99058	6.33775	13.34707	6.51405	19.62416	1.95058	9.37399	4.76299	4.87178	9.53133
**Gm6086**	0.03923	0.00836	0.09666	0.12090	0.00916	0.00914	0.00799	0.10791	0.09904	0.01652	0.05482	0.02712	0.04596	0.09005	0.13751	0.03045
**Gm6093**	0.12756	0.00775	0.00676	0.38311	0.00765	0.00701	0.01815	0.01239	0.05140	0.02068	0.07400	0.01790	0.01122	0.10234	0.02623	0.00712
**Gm6194**	0.03302	0.00773	0.04639	0.24499	0.05150	0.00753	0.05864	0.12457	0.23988	0.19774	0.20641	0.00509	0.11000	0.07685	0.09652	0.01481

**Gm6225**	0.01407	0.00811	0.02500	0.86864	0.00935	0.00608	0.00760	0.01640	0.34826	0.01398	0.00997	0.00711	0.01239	0.06528	0.02559	0.00727
**Gm6297**	0.01253	0.00843	1.76546	0.02366	0.00647	0.00557	0.00778	0.04011	0.01178	0.00317	0.01066	0.02068	0.02267	0.00864	0.29498	0.00659
**Gm6313**	0.25982	0.03874	0.25499	6.90751	0.12858	0.18483	0.22739	0.08332	4.41582	0.59455	0.41333	0.16445	0.14022	2.17845	2.90771	0.21670
**Gm6367**	0.02564	0.01787	0.01163	8.12657	0.00862	0.01343	0.00711	0.01562	2.16264	0.15112	0.02265	0.07980	0.05179	0.09498	1.48017	0.00594
**Gm6402**	4.18398	0.24891	3.38300	3.11144	3.21483	0.83937	0.87797	0.13843	6.42555	1.85079	10.12269	0.30857	2.89165	3.15724	0.91034	2.15812
**Gm6455**	0.03174	0.00790	0.13114	0.03736	0.00807	0.00710	0.00740	0.00864	0.30828	0.00448	0.28828	0.00622	0.00855	0.00951	0.00814	0.00711
**Gm6460**	0.01352	0.00789	0.07775	0.01848	0.00910	0.00771	0.00743	0.01335	0.02190	0.01451	0.01240	0.00767	0.01150	0.01012	0.02356	0.00736
**Gm6498**	4.21980	1.69843	2.49709	1.05666	3.27218	2.55385	1.25883	0.23131	0.69595	0.15756	0.00005	0.16958	2.37414	0.00299	0.19052	4.73446
**Gm6524**	0.13365	0.28083	0.17240	0.15500	0.05437	0.12203	0.16086	0.13424	0.31686	0.24206	0.07632	0.19714	0.19125	0.37289	0.49060	0.10168
**Gm6525**	3.12719	0.16043	2.07699	1.08481	1.21137	0.29453	0.19233	0.08511	2.85765	0.76141	8.76644	0.07695	1.70997	1.85342	0.88650	0.96356
**Gm6548**	38.04270	24.86627	37.10702	3.60047	28.15698	17.11310	20.25091	36.13867	6.07803	27.23757	18.33284	13.80799	35.18348	30.52357	17.80960	22.88146
**Gm6566**	0.01049	0.00789	0.04573	0.34227	0.00920	0.01163	0.06866	0.08526	0.04013	0.49858	0.06398	0.17207	0.31568	0.00675	0.11836	0.01431
**Gm6568**	0.06296	0.36081	0.42189	9.79355	0.00957	0.51973	0.00603	0.62415	8.78443	2.03659	0.81190	2.27159	0.58100	1.59275	2.14804	0.01158
**Gm6578**	0.51000	0.08102	0.26885	0.91592	0.10591	0.11517	0.07860	0.21324	0.23042	0.67252	0.35891	0.07118	0.17110	0.06623	0.42513	0.03617
**Gm6602**	0.01258	0.03001	0.02061	0.01896	0.00926	0.01244	0.00769	0.01634	0.01115	0.01918	0.01191	0.00811	0.01233	0.07700	0.03153	0.00742
**Gm6607**	0.04920	0.00825	0.02028	0.04085	0.02337	0.00617	0.00747	0.00974	0.11008	0.02524	0.16508	0.00750	0.01647	0.01213	0.00609	0.01467
**Gm6623**	0.44045	1.57671	1.19298	1.41400	0.36915	0.68721	1.07034	2.38710	2.42588	3.62033	2.48948	3.68623	1.65075	1.09813	2.65250	0.36144
**Gm6639**	0.56750	0.00677	0.10092	2.24428	0.00731	0.01441	0.00657	0.23051	1.70089	0.26431	0.00000	1.30197	0.00611	1.62512	0.75619	0.00604
**Gm6654**	18.70583	1.85303	14.19236	11.06040	11.70098	4.06761	3.59983	4.44380	19.99132	11.21937	29.99706	2.14252	11.94152	0.97207	3.06451	7.08225
**Gm6710**	0.48247	0.46542	2.45865	0.14255	0.11803	0.33795	1.55577	0.52227	0.00554	0.98237	0.00000	0.06713	0.97297	2.73157	1.82211	0.60643
**Gm6712**	0.63546	5.20255	1.66101	0.92764	0.64631	3.14181	2.05751	3.67450	0.63146	6.66318	3.62870	16.71060	1.85963	17.39324	2.87720	1.68176
**Gm6756**	1.78717	2.04761	5.49388	0.12224	1.20057	1.68490	3.01251	9.50478	0.18324	4.46093	2.38110	0.74261	6.07989	0.03876	0.00000	1.46311
**Gm6787**	0.02135	0.02857	0.00934	0.45462	0.00871	0.01926	0.00755	0.01573	0.13068	0.01481	0.01138	0.00740	0.01231	0.02117	0.27616	0.00723
**Gm6792**	1.87617	0.01861	0.00064	1.34991	0.01216	0.01466	0.00594	0.00753	0.00000	0.01280	0.01235	0.01811	0.01757	0.00756	0.04419	0.00536
**Gm6815**	1.24863	0.44713	0.69898	0.58062	0.00590	0.67531	0.48608	0.86185	0.18418	1.26102	0.00000	0.11808	0.29169	6.16585	4.21455	0.23085
**Gm6880**	0.50792	0.00866	0.50401	0.50096	0.01192	0.00725	0.00703	0.15796	0.05760	2.33074	0.10987	0.02301	1.14780	0.00812	0.82588	0.02090
**Gm6890**	0.35019	0.00893	0.87720	0.77451	0.01271	0.00720	0.00712	0.03934	0.26597	1.43247	0.17593	0.00000	0.83990	0.02088	0.59383	0.00630
**Gm6902**	0.00000	0.00870	0.34491	9.92149	0.00892	0.00341	0.00761	0.70992	13.96386	0.00000	0.08745	0.00000	0.74950	0.65700	0.00000	0.00419
**Gm6904**	0.02351	0.12859	0.02529	0.01075	0.01883	0.00686	0.00782	0.04298	0.01223	0.01273	0.01250	0.00845	0.03753	0.01468	0.01357	0.00735
**Gm694**	0.12627	0.02957	0.08228	0.01388	0.00881	0.09213	0.00744	0.05954	0.04312	0.01691	0.01157	0.00822	0.11237	0.01166	0.01342	0.20014
**Gm6981**	4.42032	1.48272	2.18412	0.84614	2.96521	1.62848	0.76044	0.18532	1.04525	0.13784	0.05006	0.11936	1.77564	0.02245	0.11297	3.09704
**Gm7008**	0.01100	0.16179	0.01012	0.01053	0.00874	0.00673	0.00731	0.01141	0.01236	0.01242	0.01251	0.08603	0.01248	0.01093	0.00738	0.00735
**Gm7030**	0.08544	0.00761	0.02634	0.01016	0.04185	0.14095	0.01733	0.03635	0.01226	0.06016	0.01260	0.00751	0.12200	0.01123	0.01520	0.13715
**Gm7056**	0.00988	0.00762	0.00554	19.15024	0.00905	0.00389	0.00672	0.00291	10.41818	4.36418	0.08927	0.06973	0.03295	0.00000	4.32214	0.00380
**Gm7102**	0.75651	1.27411	1.75127	62.15898	0.43587	0.76637	0.94742	4.97058	58.38779	40.75876	11.49194	16.21901	2.01220	37.86173	46.14028	0.28444
**Gm7104**	0.00409	0.00793	0.41165	15.60988	0.00820	0.00202	0.00691	0.29214	22.33026	0.88636	0.66065	0.00000	0.00100	0.00000	2.74646	0.00316
**Gm715**	0.09113	0.00672	0.00407	0.71903	1.35801	0.00773	0.00745	0.01010	0.00000	0.01030	0.01271	0.00653	0.01115	0.01859	0.04657	0.00709
**Gm7244**	0.01373	0.00814	0.01219	2.58254	0.00932	0.00638	0.00742	0.01352	0.26543	0.01037	0.01153	0.00778	0.01268	0.00576	0.06700	0.00694
**Gm7325**	1.77064	0.00641	0.02769	0.25210	0.01176	0.25789	0.00576	0.04412	0.00791	0.00785	0.01292	0.00653	0.03095	0.01170	0.00604	0.47891
**Gm7361**	0.01585	0.00811	0.01290	0.01833	0.00905	0.05960	0.00744	0.04651	0.01120	0.04846	0.01156	0.00801	0.00953	0.00845	0.04625	0.00718
**Gm7534**	0.00815	0.00802	0.00497	0.00369	0.01030	0.00770	0.00738	1.19015	1.86923	0.12054	0.42589	0.00952	0.01864	0.00325	0.28003	0.00745
**Gm7609**	0.00960	0.00786	0.07068	1.24777	0.01174	0.01261	0.02732	0.38099	0.80269	1.16034	0.27145	0.02443	0.28904	1.95994	1.36826	0.01430
**Gm7616**	0.04325	0.00779	0.02699	0.32333	0.02961	0.00667	0.03842	0.00044	0.46810	0.00852	0.39800	0.01472	0.01912	0.15208	0.01710	0.00718
**Gm7694**	0.01356	0.00775	0.01172	1.02850	0.00933	0.00652	0.00740	0.01776	0.00000	0.01119	0.01163	0.00770	0.01870	0.01842	0.06920	0.00720
**Gm7788**	0.02269	0.00782	0.03533	0.23876	0.00878	0.01177	0.00730	0.02306	0.05057	0.03015	0.01307	0.00780	0.02373	0.01107	0.01264	0.01442
**Gm7827**	0.01349	0.00771	0.01026	1.16219	0.00941	0.00638	0.00739	0.01196	0.49033	0.00000	0.02266	0.00491	0.01251	0.13585	0.62018	0.00712
**Gm7849**	0.01370	0.00821	0.01212	0.16177	0.00927	0.00674	0.00758	0.01432	0.16836	0.02755	0.01234	0.00853	0.01299	0.01103	0.01353	0.00739
**Gm7904**	0.03921	0.00810	0.01055	0.00541	0.00898	0.00676	0.00749	0.01442	0.20699	0.02573	0.01144	0.00811	0.01260	0.01413	0.00984	0.00728
**Gm7977**	0.97747	0.64212	0.70642	0.00000	1.52430	2.00137	0.79516	4.00236	0.00596	2.94543	0.00000	2.87444	2.73587	0.90835	0.42555	0.62536
**Gm805**	0.07966	0.00791	0.40788	0.01074	0.00780	0.01662	0.00743	0.01691	0.01227	0.00763	0.01448	0.00734	0.32233	0.01446	0.00358	0.01473
**Gm8096**	1.38523	1.25429	3.73559	0.39041	0.91904	1.60939	2.10505	6.39407	0.19065	3.77620	3.49983	0.37936	4.44645	0.00016	0.00000	0.78480
**Gm813**	0.01363	0.00817	0.01182	0.25006	0.00932	0.00657	0.00751	0.01314	0.90866	0.00714	0.01852	0.00666	0.01257	0.00868	0.06756	0.00729
**Gm8234**	2.17722	0.69211	1.13240	18.65729	2.29484	0.24019	0.38678	3.43531	17.60799	4.18222	1.07392	1.35028	2.67603	6.62303	9.47885	1.51168
**Gm8267**	0.28217	0.44023	0.07162	1.31327	0.01794	0.01107	0.12966	0.90909	0.55085	0.17626	0.00000	1.13796	0.00694	0.26983	0.55746	0.02170
**Gm8300**	0.17810	0.33238	0.51793	15.88096	0.22111	0.07287	0.11094	0.24576	15.15140	0.84789	0.00000	0.00000	0.06510	1.85928	3.13037	0.12096
**Gm8363**	0.05060	0.00824	0.04463	0.03269	0.01905	0.03837	0.01799	0.10472	0.00992	0.05411	0.07575	0.03816	0.07337	0.02617	0.03907	0.03628
**Gm839**	0.01374	0.00826	0.01228	0.43406	0.00927	0.00658	0.00753	0.01378	0.05343	0.02601	0.01303	0.00829	0.01237	0.00982	0.01944	0.00723
**Gm8579**	0.35136	0.00819	0.08601	0.10174	0.03359	1.09416	0.00695	0.39225	0.01054	0.39154	0.00714	0.07677	0.00000	0.01153	0.00213	0.00650
**Gm8580**	13.46111	3.22694	9.19858	3.39665	6.57673	2.98227	4.59158	10.29015	5.43479	11.58862	14.18006	1.60780	10.23806	0.00000	0.31749	6.23878
**Gm8615**	5.23808	4.81542	3.13921	0.11086	3.22869	4.94605	4.20785	4.14863	1.35482	2.38605	4.80003	6.26628	2.15597	3.59198	7.89549	1.35808
**Gm8624**	11.22145	0.89148	8.62815	5.07782	7.32466	2.11882	1.51973	1.23301	11.78405	9.10217	21.49770	0.76372	6.76887	2.15419	2.25433	4.41167
**Gm867**	0.01656	0.00798	0.30599	0.00536	0.00867	0.00572	0.00733	0.01216	0.14630	0.01501	0.01079	0.01911	0.01004	0.00770	0.12300	0.00720

**Gm8709**	0.04043	0.01949	0.01931	0.22241	0.06919	0.03272	0.02753	0.02188	0.05589	0.02448	0.01213	0.00763	0.02438	0.01082	0.00788	0.02237
**Gm8801**	1.79001	0.07638	1.39989	6.86304	1.08873	2.79918	0.00399	1.70568	1.68492	4.33068	0.40539	2.29211	1.86953	0.17595	3.42689	0.78889
**Gm884**	0.01361	0.00779	0.01215	0.00885	0.00912	0.00652	0.00732	0.01089	0.01202	0.40783	0.01409	0.00725	0.01125	0.01805	0.00721	0.00721
**Gm8909**	0.16155	0.00708	0.07943	0.01238	0.33054	0.12990	0.44236	0.41571	0.00997	0.13859	0.78272	0.00238	0.40818	0.01135	0.00338	0.39363
**Gm8994**	5.53857	2.92813	5.09952	7.65476	2.09678	2.43342	3.82945	10.45608	10.35377	9.57145	10.11192	3.87015	4.75930	1.31093	2.89299	1.75027
**Gm9**	0.47030	0.00836	0.00001	4.38487	0.01374	0.00513	0.00654	0.06390	0.51040	0.00000	0.76030	0.00107	1.06706	4.29339	3.69294	0.00531
**Gm9079**	0.10077	0.06032	0.10727	0.13964	0.05363	0.10514	0.16330	0.10987	0.02898	0.16905	0.04125	0.01684	0.16769	0.14946	0.07284	0.07317
**Gm9125**	0.00678	0.00738	0.00000	14.43844	0.00887	0.00324	0.00648	0.00000	14.38035	1.09840	0.41790	1.08335	0.19034	0.68704	4.92977	0.00375
**Gm9159**	0.05122	0.01753	0.09644	0.10372	0.18286	0.04403	0.07805	0.06231	0.19973	0.12330	0.18067	0.06161	0.15280	0.72322	0.10403	0.02160
**Gm9199**	2.78871	0.49808	2.85518	2.52155	4.03881	1.67997	1.04851	5.80456	6.88127	18.17757	10.02593	9.40938	5.52565	7.26335	4.07254	2.28200
**Gm9268**	0.00823	0.00765	0.00704	0.01266	0.00936	0.00650	0.00673	0.00747	0.04994	0.00000	0.04577	0.00336	0.81112	1.10607	0.00135	0.00677
**Gm9376**	8.70883	2.73356	12.16091	4.09043	8.20467	4.06988	3.99904	5.80275	18.88685	8.49070	23.27932	4.52122	13.16191	9.05859	6.18951	2.63486
**Gm960**	0.02098	0.00822	0.02123	0.04833	0.00928	0.00698	0.01916	0.02370	0.00967	0.47122	0.01217	0.00822	0.05230	0.01151	0.08746	0.00765
**Gm973**	0.00294	3.09758	1.22263	0.02814	0.04200	0.08375	0.00654	0.00810	0.26451	0.00000	0.08577	0.01494	0.11920	0.08551	0.27716	0.00576
**Gm9731**	0.01146	0.00803	0.10182	0.34429	0.07631	0.01780	0.00755	0.04412	1.19063	0.08758	0.02107	0.00541	0.03645	0.02913	0.00956	0.06513
**Gm9758**	0.01141	0.00807	0.18652	0.07205	0.00912	0.00583	0.00765	0.04596	0.05155	0.04840	0.01093	0.01950	0.00956	0.00756	0.15533	0.00739
**Gm9776**	0.86813	0.02679	0.19991	3.01446	0.11638	0.04216	0.04553	0.00000	4.47802	0.22875	3.09374	0.09107	0.11285	1.30036	0.11663	0.08496
**Gm9833**	0.22239	0.91322	0.53382	0.16571	0.91763	0.72176	0.10223	0.03905	0.19174	0.21994	0.25364	0.02285	0.49698	0.40036	0.84908	0.24571
**Gm9895**	0.01365	0.00761	0.51414	0.62812	0.00832	0.00577	0.00707	0.30805	0.35252	0.18607	0.00460	0.02538	0.16802	0.01568	0.80339	0.00702
**Gm9926**	0.08370	0.00761	0.06880	0.08604	0.00824	0.00609	0.00725	0.01807	0.17487	0.05491	0.12853	0.02636	0.04525	0.26517	0.00874	0.00722
**Gm9958**	0.01234	0.00806	0.29254	0.27651	0.00875	0.00559	0.00757	0.01337	0.45663	0.00907	0.00642	0.28635	0.02316	0.01123	0.00000	0.00720
**Gm996**	0.01375	0.00805	0.01225	0.01061	0.00932	0.00666	0.00754	0.01390	0.01235	0.01531	0.01310	0.00811	0.01270	0.01234	0.01062	0.00741
**Gm9992**	0.42321	1.06849	0.00000	0.00719	0.00771	3.18454	0.00622	0.01002	0.01289	0.04070	0.00820	0.00745	0.55171	0.00611	0.09237	0.00500
**Gm9994**	0.01127	0.00848	0.06980	0.01055	0.00960	0.00889	0.00807	0.05219	0.01234	0.01421	0.01303	0.00840	0.01796	0.01215	0.01516	0.00754
**Gmcl1**	4.17922	4.27713	2.41539	4.98783	4.32811	3.62543	0.90922	1.03594	3.45739	1.74871	1.30512	0.01420	0.30815	4.13782	0.20184	0.06437
**Gmds**	1.87212	0.00531	0.54131	0.12010	0.62019	0.01599	0.00589	0.01025	0.63078	0.05285	0.02372	0.00433	0.01525	0.01548	0.00890	0.00517
**Gmeb1**	2.42325	0.19030	4.07457	13.18663	5.90871	0.41467	0.30775	0.31328	17.32468	2.41818	14.07576	1.25513	3.14177	7.78030	5.09752	0.50128
**Gmeb2**	1.66206	0.03423	0.45825	4.89673	1.35062	0.00629	2.03506	1.16859	3.59511	2.86349	0.87810	0.58922	0.69501	0.04896	3.72007	0.01790
**Gmfb**	4.84520	4.15286	2.31502	3.40123	0.31701	2.46815	1.61076	2.40369	1.70402	4.17659	3.92862	8.50169	2.23965	9.95481	2.33279	1.50877
**Gmfg**	1.03655	0.87219	1.30980	0.00000	1.72634	0.00545	0.00512	0.00000	0.00984	0.03902	2.59915	0.00000	0.00000	0.00783	0.01680	0.01224
**Gmip**	0.01505	0.00774	0.74884	0.55037	0.02535	0.00957	1.26555	0.00714	0.45283	0.00000	0.11451	0.07408	0.27549	0.02179	0.05925	0.01491
**Gml2**	0.86638	0.00711	0.00368	0.00428	0.41215	0.00733	0.00681	0.00438	0.01085	0.00480	0.94868	0.00306	0.00432	0.01171	0.00000	0.00608
**Gmnn**	21.99162	7.37899	22.31717	24.54752	8.78492	8.93210	9.38217	15.76108	32.56965	28.24652	20.62537	10.17184	15.51194	15.55919	16.78468	8.89105
**Gmppa**	5.24969	0.58988	7.12036	0.00000	2.29075	0.26616	0.00000	1.50326	0.01980	2.05545	2.11223	0.09573	3.27360	0.00200	0.49105	3.60332
**Gmppb**	4.69109	6.13728	7.93691	0.05283	4.87332	4.12723	0.18543	6.81700	0.20680	3.85642	2.85171	0.02582	6.17386	0.00007	1.43035	5.16733
**Gmpr**	0.01107	0.00708	0.21059	0.06590	0.00878	0.00576	1.59816	2.39223	0.00803	0.84037	0.24782	0.00000	0.00396	0.00835	0.00600	0.00726
**Gmpr2**	6.64149	5.90200	4.50954	0.63675	4.98308	0.06777	3.66636	0.00000	0.37434	0.83940	0.71231	0.02497	0.09496	0.00271	0.78257	1.10677
**Gmps**	12.95833	14.37559	8.36698	7.68497	13.94076	5.93103	9.73579	13.32006	14.11183	19.84939	10.46733	11.42721	11.33789	14.63245	13.30096	4.73984
**Gna11**	6.62020	0.00000	6.58690	3.29459	2.78232	6.54667	0.48390	6.33823	0.75596	10.40809	1.20645	0.33844	5.55646	0.00561	0.00000	2.20007
**Gna12**	0.11170	0.27555	0.01109	0.38485	0.04169	0.00652	0.53021	0.37676	0.35010	0.34606	0.00876	0.01371	0.15617	0.00519	0.06127	1.12523
**Gna13**	9.46364	7.50905	8.46889	2.50029	7.90727	3.71444	9.62144	4.29503	2.03120	6.11153	9.14920	0.39769	3.60036	6.19354	0.39408	4.45281
**Gna14**	0.01470	0.00789	0.01252	1.26261	0.00983	0.00715	0.00749	0.01354	0.13967	0.00000	0.05874	0.00678	0.47915	0.05443	0.28015	0.00730
**Gna15**	0.58896	0.05041	0.04277	0.33245	0.00762	0.00849	0.00666	0.00775	0.56956	0.00000	0.56521	0.00297	0.82516	0.00588	0.00000	0.00637
**Gnai1**	0.88633	0.01667	1.41912	2.63849	0.00382	0.00460	0.00654	0.00537	0.19736	0.14629	0.00400	0.05490	0.03574	0.05298	3.98032	0.78849
**Gnai2**	3.70046	0.10407	5.03948	0.39061	1.34324	3.56004	1.34748	2.34281	0.72297	6.23789	0.80117	0.37413	2.05020	0.01019	0.17729	3.07856
**Gnai3**	5.25772	11.07582	7.45788	7.15601	13.86732	5.74394	8.66038	6.65118	8.92343	9.92794	4.49509	18.70504	2.96492	11.98434	7.31899	1.64621
**Gnal**	1.21534	0.18322	1.16043	0.44907	1.11178	2.42496	1.45853	0.79896	0.77729	3.04254	2.01160	0.11487	0.37896	2.14603	0.79983	0.09203
**Gnaq**	2.80686	0.04610	1.43472	0.15134	1.53733	0.13839	2.60996	0.01009	1.06616	2.83111	0.00000	0.09350	0.01227	5.27470	2.17587	2.73280
**Gnas**	7.78156	7.86742	7.51184	6.00520	8.39454	2.58292	10.12426	10.37341	1.44924	16.48105	15.54961	5.73979	5.78588	4.11253	0.85086	7.02786
**Gnb1**	14.91139	9.09292	19.02551	10.11952	7.24062	8.11231	12.75729	13.33252	2.62035	10.60200	13.68726	3.44505	14.29254	9.09107	4.93901	9.97689
**Gnb1l**	1.35416	4.54120	5.30847	2.12928	0.00604	0.00418	0.00238	3.64562	0.59755	7.29410	0.97537	0.12800	2.98311	0.10019	0.53812	0.48520
**Gnb2**	5.18067	0.03231	3.31006	4.31098	1.19844	5.73524	0.38719	3.76119	0.77226	5.02058	1.28013	1.63366	7.24504	0.00000	0.00000	4.76637
**Gnb3**	0.00996	0.00803	0.01027	0.01019	0.01061	0.00735	0.00745	0.41016	0.01173	0.00502	0.01394	0.00708	0.12814	0.00998	0.04770	0.00769
**Gnb4**	0.01325	0.00748	0.00751	3.79154	0.00907	0.00605	0.00696	0.00000	1.76655	0.71158	0.33104	0.01687	0.00904	0.00086	0.90146	0.00663
**Gnb5**	0.01248	0.00788	0.42135	0.01082	0.00842	0.00920	0.00735	0.01268	0.01228	0.01485	0.01206	0.00769	0.00943	0.01402	0.00393	0.00721
**Gne**	2.61775	0.00508	0.00000	1.11799	0.02758	2.34781	0.00514	0.00392	0.42507	0.44025	0.00674	0.02177	0.02043	0.00361	0.13261	0.00380
**Gng10**	0.94797	2.76050	6.71934	0.00000	3.73699	0.00661	3.02455	0.01812	0.08363	1.00830	0.32510	0.01937	1.27030	0.33571	0.00000	0.17359
**Gng12**	2.73046	0.00446	0.00848	7.58547	0.00423	0.00378	0.00421	0.00000	2.36514	2.01560	0.64856	1.45652	0.88738	0.00000	0.77326	0.41459
**Gng13**	0.00848	0.03071	0.01144	0.27585	0.00826	0.00653	0.00787	0.03281	0.31004	0.00682	0.01096	0.00833	0.00866	0.01217	0.00843	0.57553
**Gng2**	0.00000	1.01502	1.03151	0.06184	0.01880	3.73342	0.00528	0.00000	0.07369	0.32440	0.20119	0.01229	1.84327	0.02167	0.00000	4.50286
**Gng3**	0.01359	0.00795	0.00967	6.03382	0.00905	0.00600	0.00716	0.00625	2.06322	0.08917	0.19499	0.00323	0.00957	0.04009	0.00000	0.00597
**Gng5**	10.47129	0.07470	2.30488	8.41200	8.89622	1.76802	0.47293	0.83691	5.54836	6.36855	7.68655	0.42335	5.01992	0.01711	0.37580	5.88369

**Gng7**	0.01322	0.00784	0.01141	0.00420	0.00908	0.00651	0.00726	0.01032	0.18481	0.86985	0.00927	0.02454	0.01163	0.00572	0.38280	0.00757
**Gngt1**	0.03122	0.05892	0.12850	0.05827	0.00823	0.03734	0.01725	0.00000	0.07254	0.16462	0.62990	0.04095	0.14993	0.22337	0.60000	0.02152
**Gnl1**	9.51614	5.51872	3.37379	6.52899	1.47402	6.28162	8.81191	12.95706	6.47719	13.75366	6.97191	0.73653	12.37832	0.21456	1.12512	7.28690
**Gnl2**	9.58679	13.73547	12.65918	3.67619	5.15313	0.31676	9.89280	18.55046	10.34507	13.58703	9.99614	8.60727	5.53508	12.36900	11.49312	4.95123
**Gnl3**	11.21732	18.17308	14.54776	18.64042	12.71801	9.03108	15.35708	22.36068	22.08984	28.18550	22.01337	20.51189	16.23734	31.57960	26.33059	14.37161
**Gnl3l**	6.93970	7.03883	6.27938	9.36395	3.66511	0.11483	4.00599	4.24997	8.87205	12.36596	12.01872	11.09157	6.68704	15.16006	12.67177	2.20567
**Gnmt**	0.01289	0.00813	0.03640	0.28298	0.00915	0.00802	0.00746	0.01356	0.72569	0.01177	0.00907	0.00698	0.01228	0.01203	0.00365	0.00732
**Gnpat**	7.41801	10.44768	5.89266	3.02947	9.32735	2.54500	3.11455	1.31569	2.30828	3.91386	4.22865	5.14775	3.72684	2.55451	3.71831	2.34314
**Gnpda1**	5.25404	5.26352	3.36745	0.29793	2.26118	5.88136	5.62658	4.95183	1.49886	3.16621	4.87581	5.46231	2.27079	4.19177	9.00067	1.66055
**Gnpda2**	1.78030	0.63019	0.00000	0.10549	0.00720	2.95201	0.95405	1.01466	0.79401	2.68902	0.58719	1.20961	0.00000	0.49996	0.91303	0.00405
**Gnpnat1**	9.10841	8.16591	12.02019	10.10887	1.50029	5.85005	12.12157	15.88359	14.77609	12.59746	17.31196	19.35734	11.05315	27.00708	14.33588	0.16879
**Gnptab**	4.03921	4.99214	7.26998	1.63178	4.79580	4.20813	5.04702	12.43450	8.71933	12.68033	7.48132	6.54476	5.75624	21.47492	11.13350	4.03873
**Gnptg**	0.23256	0.24053	6.20256	4.28088	2.62989	3.87059	3.37951	6.33839	6.99894	9.23435	4.67072	1.43812	7.07361	3.61153	6.66920	3.60260
**Gnrh1**	0.01312	0.00836	0.07031	5.89442	0.00889	0.00803	0.00726	0.00490	0.63687	0.01306	0.44257	0.01598	0.00740	0.00000	0.04289	0.00605
**Gns**	1.67883	5.61626	4.82657	1.17279	5.17626	0.90282	0.45320	3.09642	0.11657	1.14749	1.80487	2.35595	4.13560	0.00354	1.53864	0.45394
**Golga1**	1.23307	1.75157	1.32109	2.07168	0.00482	0.00605	0.85867	1.36131	6.06051	3.37821	1.36016	0.27287	0.46474	7.74679	3.18062	0.00385
**Golga2**	3.59577	0.12829	5.28788	7.56712	0.01842	0.00159	2.63902	6.39233	5.43831	2.16291	4.96418	0.24482	3.52899	2.74005	3.96438	3.89594
**Golga3**	2.55422	4.44829	2.30589	1.42571	2.78532	0.01015	2.13544	2.63352	2.30186	5.00297	0.98204	0.21090	0.37965	1.79519	2.88887	0.00000
**Golga4**	6.53348	2.41270	2.92804	3.28193	1.19153	7.38418	4.16234	2.65245	1.05826	3.72364	0.38068	0.20777	3.69491	4.57541	2.26494	2.12626
**Golga5**	3.76527	1.34016	4.91114	3.71019	4.62811	1.75592	0.97481	4.34120	3.52681	2.21532	3.10198	0.05245	0.00000	2.73984	6.13117	0.02944
**Golga7**	12.52783	4.52667	10.58724	1.62200	10.59348	7.39667	5.80249	13.49602	2.97189	14.53077	9.34258	6.39523	9.43730	0.78145	4.52186	4.31896
**Golga7b**	0.01215	0.00805	0.01377	0.25170	0.01011	0.00703	0.00765	0.01335	0.00772	0.19964	0.04043	0.02494	0.26914	0.00481	0.36929	0.00739
**Golgb1**	5.18581	2.19890	0.00000	3.05403	2.25898	2.26219	1.64885	2.04858	1.93669	1.61280	0.36958	0.15870	0.88867	1.26957	3.02461	1.04873
**Golm1**	7.85672	2.15352	3.54958	1.71040	6.78038	2.13734	1.92356	0.80312	0.50744	1.46021	0.00000	0.69300	0.91755	0.00438	0.61399	2.58274
**Golph3**	4.34255	3.46836	3.17935	12.07120	13.60542	7.36478	1.98482	3.25162	9.31202	7.31944	0.26724	10.55699	2.57528	10.05715	9.67009	3.19839
**Golph3l**	3.47059	0.01548	0.00000	0.58907	0.01256	1.42146	0.96300	0.18446	2.58693	1.07017	0.64407	0.18508	1.47590	0.00335	2.24987	2.00136
**Golt1a**	0.00414	0.00624	0.00000	0.00289	0.01059	0.00896	2.05731	0.42224	0.01004	0.38107	1.43318	0.00666	0.00000	0.01222	0.10319	0.00720
**Golt1b**	5.56493	3.10266	6.29844	4.63846	4.02134	0.16791	7.68970	4.70014	7.79975	7.12986	3.12006	6.14266	5.22716	12.15573	5.53310	0.99936
**Gon4l**	5.61131	3.11740	5.51000	3.95366	0.74698	4.86840	1.76954	6.25821	2.92592	4.02933	2.12121	0.24997	0.95916	0.61230	3.44749	3.56606
**Gopc**	0.47679	0.19024	0.00000	0.66308	0.50590	0.00712	1.10857	0.00000	0.00000	0.00000	0.69960	0.00923	0.10366	0.00501	1.45844	0.94586
**Gorab**	0.00000	2.85440	0.95393	0.15730	0.00618	0.00610	0.00626	1.41345	0.37476	0.47937	0.54162	0.08100	0.44216	0.00672	1.12362	0.24482
**Gorasp1**	3.64056	1.67002	1.05165	3.96230	6.30185	0.01334	3.92625	8.17883	4.50556	25.03275	9.16756	1.41188	5.34816	0.00000	6.90114	2.41584
**Gorasp2**	12.45710	7.22517	10.68906	7.53184	5.31685	4.84019	0.45267	8.50033	3.91899	9.04585	4.43207	2.64978	9.90814	0.16232	2.81560	8.55016
**Gosr1**	2.84112	5.55978	2.43005	1.09023	3.54380	0.00800	1.92328	4.45196	1.67823	5.63810	1.94792	0.19736	4.92600	0.00237	4.90120	0.00000
**Gosr2**	4.85347	3.63636	6.06188	4.67441	3.37675	1.12230	4.73836	7.05755	2.45214	10.60066	5.32937	0.48375	5.67491	7.66708	9.35472	1.37336
**Got1**	14.88726	16.69316	25.49907	12.04395	13.82781	15.03483	15.38985	20.73180	10.81757	8.50883	12.37810	3.34556	23.76490	1.94265	7.26552	11.28383
**Got1l1**	1.22287	4.06138	5.77022	0.00000	0.00000	0.58238	2.41287	2.65293	0.00796	0.08482	3.66013	0.01796	0.62121	1.33669	0.00000	0.00825
**Got2**	10.51113	2.50491	8.53353	4.80205	5.19843	2.95128	3.62318	10.89123	1.58081	11.25275	3.77212	1.38367	2.48483	1.40648	4.56583	3.84772
**Gp1ba**	0.32385	0.00731	0.15762	0.00434	0.00848	0.00539	0.00698	0.05437	0.60497	0.13776	0.00661	0.00514	0.02370	0.01254	0.22643	0.12051
**Gpa33**	0.99834	0.00642	0.21162	2.17548	0.16705	1.40562	0.00529	0.01077	0.41146	0.00155	0.01000	0.00439	1.59937	0.01025	0.00812	5.94513
**Gpaa1**	3.59062	0.08627	5.09428	2.95316	0.09516	2.10647	2.33467	3.99080	1.62884	2.60608	1.33017	0.05809	9.59797	0.00000	0.00000	6.72336
**Gpalpp1**	3.33770	6.95841	4.14757	3.50916	1.61341	0.09648	2.47056	6.35182	10.13438	4.56025	4.66181	1.91264	4.20382	7.81191	3.85508	1.74079
**Gpam**	0.01107	0.00792	0.04044	1.31329	0.00875	0.00571	0.07791	0.01007	0.25885	1.71028	0.01038	0.00304	0.01042	0.04158	0.01611	0.12339
**Gpank1**	7.83131	1.68534	4.52614	5.79313	6.86115	0.15959	6.18334	7.48668	11.21236	13.78848	6.16886	0.88121	8.07065	0.00000	1.86631	3.59578
**Gpat2**	0.41831	0.00755	0.00000	1.99350	0.01427	0.24076	0.54294	0.29802	1.65237	1.73709	2.56940	0.08620	3.44895	0.08178	1.08538	1.97207
**Gpat4**	7.96250	8.11502	0.55739	7.78349	2.17982	6.24546	2.58399	1.65232	3.17928	3.70301	0.80519	0.12056	5.97472	0.04542	1.07506	1.83222
**Gpatch1**	2.04462	0.33541	3.50880	0.15106	0.01344	0.00565	3.22609	1.67025	3.12452	4.42869	2.75136	0.18775	0.00000	0.21006	0.59359	0.01082
**Gpatch11**	1.41871	0.03815	6.62295	0.26655	0.01909	0.39395	1.60540	4.98349	0.97897	6.09549	3.62100	0.85858	1.07356	0.22593	2.53690	2.59343
**Gpatch2**	1.99083	4.71083	2.99894	2.07517	0.81310	0.00346	3.14225	1.21081	3.33265	0.12546	1.10980	0.06106	2.21887	0.23525	1.13431	0.03594
**Gpatch2l**	0.24805	1.34200	0.00000	3.03276	0.03258	0.49465	0.39396	1.29085	0.66115	0.42660	0.30711	0.02787	2.31213	0.83161	1.81485	0.41987
**Gpatch3**	0.79431	0.46640	0.00000	8.24550	0.00622	0.14417	0.00450	2.30541	15.95552	2.21891	1.06492	0.03861	2.23069	0.04030	0.10881	0.02482
**Gpatch4**	9.72883	1.67837	4.55378	2.71267	0.14601	4.85393	0.12675	10.44200	3.44184	3.81390	2.95667	0.25986	4.42552	2.07809	7.58883	5.27700
**Gpatch8**	2.68873	7.32477	2.52527	4.17095	0.02711	4.96405	3.04170	1.32975	4.50537	4.87015	1.78325	2.92464	0.00000	4.90924	5.17181	0.81630
**Gpbp1**	8.62141	7.54943	5.93668	33.55019	9.89435	3.66973	7.66040	13.93309	35.08698	20.93000	14.09140	18.68788	6.47064	30.87579	31.94881	5.10199
**Gpbp1l1**	1.53211	0.31195	0.24792	3.56824	3.85724	0.20869	2.11616	0.58811	9.44512	2.32051	2.89817	0.12296	0.71640	4.95059	7.76192	1.66673
**Gpc2**	0.01374	0.00808	0.01202	0.09285	0.00932	0.00661	0.00749	0.01316	0.00781	0.02701	0.01213	0.01824	0.01970	0.01139	0.00973	0.00737
**Gpc3**	0.01175	0.00711	0.00784	0.01010	0.00941	0.00686	1.13982	0.00966	0.01227	0.03069	0.01331	0.00659	0.01089	0.01149	0.01058	0.00754
**Gpc4**	1.51344	0.00577	0.00000	0.08823	0.78794	0.00463	0.95323	1.33939	2.90567	1.84085	1.86382	1.73028	0.16898	5.25148	0.79547	3.89215
**Gpc6**	0.03556	0.00740	0.01484	0.08995	0.00824	0.00742	0.07736	0.01544	0.12863	0.18472	0.01030	0.03413	0.04142	0.17981	0.08036	0.01516
**Gpcpd1**	4.40716	8.05527	8.92175	12.26707	6.83373	1.68494	12.85856	13.43574	17.84920	14.45483	17.98402	25.94085	2.83266	21.62958	21.66007	2.97815
**Gpd1**	0.01053	0.00779	0.01032	3.16673	0.00975	0.00641	0.00725	0.01071	1.96233	1.56660	0.00000	0.06939	0.33005	0.03138	2.13455	0.00729
**Gpd1l**	14.71683	12.10146	27.63109	21.16304	12.07225	16.27910	17.13303	49.79588	27.18047	44.82636	43.51721	25.38044	34.49545	42.62153	31.18325	4.67195

**Gpd2**	9.52259	11.22344	14.02869	3.46517	14.35449	6.87571	11.53423	15.87275	4.91955	23.89027	18.99239	15.99205	16.01332	29.75338	14.69999	9.07794
**Gpha2**	0.00832	0.04109	0.91961	0.55254	0.00994	0.00665	0.00559	0.17433	0.12577	0.05255	0.84459	0.02513	2.68504	0.00466	0.00000	0.01317
**Gphn**	0.62873	0.12323	0.00000	1.26860	3.86724	4.85806	1.25568	0.05479	0.62209	0.61644	0.68130	0.04956	0.01291	0.01933	1.00393	0.31729
**Gpi1**	23.00942	13.02844	13.44027	5.16739	10.26410	5.76118	7.48104	10.85566	4.59484	7.55909	9.48648	0.97795	17.67966	0.94201	3.97790	9.60999
**Gpkow**	0.58860	1.21820	2.70727	5.40866	1.22924	0.45298	0.49472	5.93377	11.25757	0.56218	0.12205	2.24521	1.84701	5.90981	12.86009	1.06599
**Gpld1**	0.01404	0.00786	0.00495	0.04971	0.00853	0.01291	0.00679	0.38303	0.11887	0.00987	0.00642	0.06940	1.41289	0.00805	0.99987	0.07963
**Gpm6a**	0.01858	0.00773	0.01085	1.57710	0.01066	0.00633	0.00729	0.01006	0.00000	0.00310	0.04585	0.00606	0.01206	0.00769	0.69460	0.00713
**Gpn1**	13.09341	9.48220	10.55151	6.13318	0.04970	4.07380	3.99003	6.52761	6.63156	12.39985	5.33892	1.43996	4.18995	5.01219	12.99811	3.32714
**Gpn2**	0.33883	1.31416	0.77847	1.23776	1.76222	0.40396	0.24861	1.84463	0.49633	0.06706	0.95211	0.07285	0.54184	0.20998	1.86547	0.14873
**Gpn3**	5.74278	2.71323	7.25888	12.33365	3.66528	0.57516	2.06439	14.15506	17.89070	10.47544	10.99015	10.01754	4.97010	0.55223	3.94969	0.09112
**Gpnmb**	0.01379	0.00801	0.01187	0.76022	0.00933	0.00661	0.00748	0.01339	0.01693	0.01423	0.01281	0.02848	0.01274	0.01166	0.01019	0.00729
**Gpr1**	0.01377	0.00778	0.01197	0.65518	0.00935	0.00645	0.00740	0.01180	0.04871	0.00578	0.02955	0.00690	0.01230	0.00516	0.35897	0.00724
**Gpr107**	5.75581	0.00000	6.67264	1.63006	0.00000	1.74833	2.98313	6.84572	1.77103	4.60072	0.90238	0.15841	3.45397	0.00000	3.73171	1.58696
**Gpr108**	5.95408	1.95441	6.67014	0.00000	6.36258	6.36334	3.89481	0.62993	0.00546	2.40137	0.71442	0.01521	6.41023	0.00570	0.21839	6.07752
**Gpr137**	0.19240	0.00789	3.77332	0.68306	0.00000	0.28636	0.00730	1.06621	0.25285	0.59934	0.41091	0.10776	0.40983	0.00594	0.00000	3.03463
**Gpr137b**	2.36051	2.83356	12.88404	4.42330	2.04457	9.29404	5.97591	18.52658	7.69588	20.27109	14.70407	13.32042	16.90993	14.74287	7.21200	2.81145
**Gpr137b.ps**	1.63828	2.03615	8.87138	5.88422	1.25618	8.92825	4.46151	15.07233	5.18163	15.31520	12.87914	9.37632	14.06428	8.30017	3.86707	0.71118
**Gpr143**	0.01393	0.00795	0.00921	1.26073	0.00909	0.00662	0.00744	0.00444	0.00000	0.00000	0.43222	0.00555	0.00785	0.00356	0.14834	0.00693
**Gpr146**	1.81585	0.00570	0.00000	1.13887	0.00443	0.01407	0.00586	0.00200	1.11137	0.00000	0.25961	0.00219	0.00896	0.00334	0.36677	0.01888
**Gpr155**	0.01500	0.00761	0.88277	0.09623	0.00768	0.00546	0.00716	0.00938	1.24738	0.00000	0.00000	0.23229	0.00867	0.01233	0.00000	0.00709
**Gpr156**	0.00956	0.00709	1.88191	0.28494	0.00661	0.00799	1.41713	0.00804	1.09961	0.21786	0.00000	0.00016	0.00944	0.00428	0.91623	0.00646
**Gpr157**	0.18886	0.07238	0.34483	1.19127	0.12224	0.02716	0.09896	0.29524	1.94774	0.87651	2.09577	0.64396	0.22340	1.40653	0.72613	0.08576
**Gpr160**	0.00000	2.20709	2.31929	0.21649	0.00402	0.01785	0.01550	0.64864	0.35037	3.60659	1.17447	0.17119	1.03128	0.73325	3.35260	0.01210
**Gpr161**	2.41641	2.22249	8.57389	9.66456	3.57008	1.69419	9.93237	17.58475	13.37363	9.74490	17.15351	10.68072	7.90487	16.60134	13.78874	2.19038
**Gpr171**	0.00021	0.00739	0.00843	0.01735	0.02300	2.90313	0.00687	1.36149	0.01050	0.00000	0.00000	0.01999	0.11531	3.39418	0.13880	0.00623
**Gpr173**	0.01102	0.01883	0.01516	0.02174	0.03658	0.01254	0.00739	0.02117	0.04084	0.04754	0.05442	0.00729	0.02469	0.04683	0.02870	0.00743
**Gpr174**	0.07340	0.00787	0.02731	0.03250	0.01780	0.03989	0.02800	0.02412	0.01135	0.05099	0.04098	0.00733	0.03004	0.05915	0.03953	0.02153
**Gpr176**	0.01380	0.00775	0.01176	0.06302	0.00934	0.00658	0.00744	0.01262	0.01146	0.00874	0.01300	0.03639	0.01272	0.00632	0.18168	0.00737
**Gpr180**	0.33146	0.00726	0.47664	2.38834	0.01593	0.00503	0.04838	3.84201	1.19529	1.13376	0.04925	0.00000	0.04247	0.01405	1.78434	0.00632
**Gpr19**	2.40930	1.59122	2.01076	1.87944	0.00362	0.95048	0.01460	0.59137	0.00000	0.24184	1.31302	0.00000	0.00000	0.20346	0.45211	0.12693
**Gpr20**	0.01551	0.00834	0.03973	0.01056	0.01167	0.01697	0.00755	0.01538	0.01235	0.00774	0.01501	0.00764	0.40470	0.01285	0.00973	0.00755
**Gpr3**	0.01088	0.00765	0.00586	3.10659	0.00913	0.00625	0.00693	0.05145	4.96972	0.23081	0.40230	0.05483	0.00678	0.02845	0.38352	0.00643
**Gpr35**	0.01540	0.00781	0.05999	1.14319	0.00931	0.00610	0.00735	0.01119	0.02755	0.01709	0.05276	0.00729	0.01311	0.07119	0.01166	0.01453
**Gpr4**	0.00000	0.00717	0.18706	0.04624	0.02839	0.04658	1.67548	1.28793	0.01048	0.60184	0.08027	0.02690	1.68967	0.02131	0.00000	2.82562
**Gpr50**	0.01161	0.00755	0.00219	6.82424	0.00877	0.00534	0.00692	0.00000	12.48282	0.32513	0.28137	0.72082	0.01112	0.00000	1.37552	0.01267
**Gpr68**	0.03893	0.00784	0.04739	1.41420	0.00897	0.00632	0.01786	0.01485	0.68081	0.00532	0.14717	0.00469	0.05531	0.03576	0.53786	0.00688
**Gpr75**	0.01369	0.00792	0.01145	0.54931	0.00939	0.00638	0.00734	0.01122	0.35634	0.00410	0.00962	0.00623	0.01277	0.00752	0.73314	0.00721
**Gpr85**	0.01215	0.00686	0.01634	0.00869	0.00933	0.00629	1.35515	0.00932	0.01179	0.25572	0.01272	0.00560	0.01089	0.01017	0.08191	0.00769
**Gpr89**	5.51910	3.87935	4.10424	0.41672	2.50682	4.03040	5.42578	3.39121	0.31344	0.65611	1.64973	0.04648	5.57323	2.18187	0.66767	1.26005
**Gprasp1**	1.97300	0.00629	0.91202	0.56871	0.00709	0.00394	0.00630	0.67510	0.95927	1.12364	0.35586	0.88333	0.00000	0.00898	1.49185	0.00474
**Gprasp2**	0.01775	0.00817	0.01178	0.35736	0.00895	0.00661	0.00751	0.01421	0.51469	0.01244	0.01069	0.00752	0.01279	0.01144	0.00724	0.00730
**Gprc5a**	4.16309	1.71503	15.04283	0.18029	2.71446	9.42106	8.98785	17.27628	0.01213	7.61485	5.06835	0.06901	13.32946	2.62594	1.62543	5.00660
**Gprc5b**	0.02016	0.00782	0.05885	0.01037	0.00888	0.00591	0.00744	0.01365	0.01249	0.01415	0.01306	0.00767	0.01176	0.00929	0.03336	0.00733
**Gprc5c**	0.00000	0.05785	4.10398	2.44468	0.12468	0.00601	1.54962	8.50380	1.31556	8.03276	6.21901	0.38550	3.39309	7.72070	8.97689	0.00994
**Gprin1**	0.01356	0.00798	0.01211	0.01059	0.00932	0.00666	0.00747	0.01832	0.01234	0.01873	0.01314	0.00792	0.01246	0.01208	0.01067	0.00748
**Gps1**	15.31262	11.23053	12.00101	16.37326	11.29485	11.87555	9.71445	10.52415	16.50015	15.15693	10.65035	10.30441	9.80123	0.16714	12.41390	10.44545
**Gps2**	7.58188	0.75018	3.86423	7.08430	3.73181	0.50069	1.14417	9.60144	4.66443	9.16141	4.52461	6.37299	9.99831	0.02821	1.10359	1.74397
**Gpsm1**	0.00000	0.00673	0.40234	0.00352	0.00751	0.00909	0.00587	6.05798	0.00699	3.88744	2.62804	0.06496	0.95899	0.43179	0.36003	0.02064
**Gpsm2**	2.53091	0.00000	0.28707	2.59823	3.44965	4.94598	0.92814	5.51557	1.45362	3.38694	1.58875	3.08076	3.39878	2.42010	1.43140	0.01441
**Gpsm3**	0.00829	0.00769	0.04747	0.01562	0.02068	0.00793	0.05923	0.53289	0.01196	0.31281	0.02591	0.00525	0.07583	0.01351	0.00091	0.00776
**Gpt**	0.00649	0.00788	1.41726	0.61685	0.00715	0.00529	0.03736	0.71058	1.24386	0.38773	0.00000	0.02782	0.54890	0.01485	0.02386	0.00617
**Gpt2**	0.16589	2.73102	0.91679	4.98191	6.66739	0.00703	1.27348	0.87786	6.69402	3.33112	0.92532	1.32439	0.13541	1.18241	10.69019	0.00441
**Gpx1**	32.76585	4.83781	17.39982	15.35907	19.58406	15.44386	7.76758	28.24476	4.84302	30.92400	25.88794	3.51474	29.61838	0.13963	0.98530	16.06435
**Gpx2**	6.06170	0.01758	0.64827	0.20888	0.00000	0.00265	8.56555	0.00000	0.00959	0.00000	1.33269	0.02260	0.08266	0.01489	0.05949	1.25601
**Gpx2.ps1**	1.36030	0.00484	0.13952	0.03065	0.00613	0.00764	2.74469	0.01880	0.28987	0.00000	0.23102	0.00344	0.03440	0.00996	0.04503	0.36337
**Gpx3**	1.72164	0.03154	0.07833	6.76060	0.21376	0.00440	0.01743	0.98833	0.19308	5.40890	0.18695	0.31775	0.55910	0.00000	0.62176	2.45865
**Gpx4**	28.07105	9.73527	19.27339	0.27005	16.67145	13.68015	13.52144	28.53013	1.28736	22.98575	7.35125	2.77320	29.70650	0.00000	0.00000	13.44559
**Gpx5**	0.03343	0.00840	0.39750	0.01077	0.01391	0.00941	0.00718	0.01208	0.01230	0.00596	0.01326	0.00734	0.57050	0.01479	0.00365	0.00719
**Gpx6**	0.01021	0.00725	0.00686	0.05024	0.00959	0.00674	0.00709	0.77627	0.02204	0.00000	0.00556	0.00506	0.00542	0.02428	0.46694	0.00725
**Gpx7**	1.30261	0.00470	1.77292	3.44619	0.01101	0.02757	1.59319	0.00000	11.60108	0.21570	1.60193	0.00000	1.99077	0.79568	0.00000	0.01731
**Gramd1a**	1.74541	0.02520	0.72599	0.00799	0.00333	0.00630	1.52734	0.00452	0.01189	0.47810	0.01072	0.00431	0.00914	0.01134	0.02150	0.01963
**Gramd1b**	0.12901	0.00738	0.00301	0.08757	0.00754	0.00751	0.00704	0.00000	0.88383	0.00000	0.45233	0.00186	0.00790	0.56744	0.00000	0.00685

**Gramd1c**	0.01166	0.00746	0.00324	0.08525	0.00957	0.00645	0.00703	0.00483	0.85969	0.00000	0.09988	0.00440	0.01133	3.23538	0.00000	0.00715
**Gramd2**	0.01202	0.00775	1.49757	1.19145	0.00682	0.00537	0.00736	0.01369	0.66037	0.00000	0.09635	0.01639	0.00612	0.01627	0.13870	0.00646
**Gramd3**	0.01236	0.00790	0.01221	0.00561	0.00864	0.00659	0.00732	0.01005	0.01117	2.71529	0.00965	0.00000	0.01056	0.01230	0.00730	0.00731
**Gramd4**	1.78661	2.66344	0.50757	0.93208	0.00440	0.00720	2.10716	0.00000	0.00000	0.06134	0.51088	0.01920	0.38629	0.12754	0.12266	0.00399
**Grap**	0.01439	0.00791	0.02019	0.45980	0.00925	0.00620	0.00744	0.01781	0.74942	0.01520	0.02039	0.00646	0.01205	0.00863	0.03978	0.00723
**Grasp**	0.40737	0.00694	0.30649	0.29415	0.00727	0.02644	0.00678	0.31311	0.35336	0.00755	0.00818	0.00562	0.00460	0.00898	0.02490	0.00674
**Grb10**	1.43296	0.00345	0.26489	2.08118	4.06163	0.00716	2.24555	0.00000	0.45540	1.11854	0.30542	0.04730	0.01877	3.23862	2.30801	0.00512
**Grb14**	0.01437	0.00775	0.00383	0.00906	0.00912	2.81950	0.00707	0.01031	0.01261	0.02756	0.00813	0.13458	0.17230	0.01443	0.00801	0.00616
**Grb2**	7.56841	2.73672	5.02088	8.45524	1.18791	6.68246	3.59026	15.18000	12.73375	17.03584	8.40351	2.29024	4.89242	7.36703	15.10569	2.68863
**Grb7**	1.61239	0.36026	0.00000	0.00000	0.00566	2.69949	0.00488	0.00000	0.01120	0.00000	1.18296	0.03973	1.21579	0.01000	0.00000	0.18472
**Grcc10**	4.58624	0.01248	0.36228	1.88308	0.23217	3.17524	0.55603	0.00000	0.18483	2.65811	0.27331	0.05786	1.19911	0.02775	0.08446	1.28218
**Greb1**	0.01208	0.00761	2.71201	0.43987	0.00494	0.00543	0.02841	0.01372	0.01962	0.49214	0.00870	0.01445	0.00563	0.03278	0.20167	0.00635
**Greb1l**	0.15002	0.00628	3.75576	0.33536	0.00280	0.00627	0.84354	4.75258	0.01941	4.22240	1.60884	0.67148	3.40781	15.38548	5.72329	0.01845
**Grhl1**	0.01536	0.00768	0.01031	1.93297	0.00905	4.88204	0.00684	0.00000	0.05191	0.14153	0.28301	0.01520	0.02100	0.00598	1.17020	0.01219
**Grhl2**	3.96991	1.14988	5.93148	10.00790	0.16484	4.61167	0.02107	3.00369	7.45203	2.01442	1.92759	0.28125	3.23630	4.95612	6.31849	2.59480
**Grhl3**	0.02148	0.00827	0.06456	0.00264	0.00880	0.00575	0.22500	0.11571	0.39678	0.10818	0.59157	0.01412	0.00466	0.06473	0.88055	0.00720
**Grhpr**	9.72631	0.02224	6.50371	0.07302	3.87106	5.04448	5.43298	7.02591	0.50230	5.15822	4.18880	0.98660	4.46480	0.00000	0.09966	3.53185
**Gria4**	0.01372	0.00763	0.01136	0.99737	0.00940	0.00638	0.00731	0.01104	0.00000	0.01056	0.01040	0.00565	0.01310	0.00775	0.92528	0.00735
**Grid1**	0.01349	0.01911	0.01167	0.12095	0.00927	0.00667	0.00749	0.01275	0.00711	0.01347	0.02754	0.01824	0.01242	0.03258	0.00674	0.00739
**Grid2**	0.01154	0.00784	0.00820	0.00905	0.00921	0.00667	0.00700	0.00878	0.01200	0.08959	0.00899	0.00442	0.28846	0.65768	0.17205	0.00751
**Grid2ip**	0.00846	0.00770	0.00813	0.00749	0.00930	0.00683	0.00720	0.71160	0.01129	0.93748	0.00632	0.00380	0.00530	0.01073	0.01032	0.00715
**Grik1**	0.01384	0.00795	0.01001	0.00572	0.00900	0.00668	0.00733	0.00309	0.01155	0.38734	0.28795	0.01530	0.00878	0.00924	0.00872	0.00715
**Grik5**	0.01356	0.00792	0.01223	0.27287	0.00928	0.00644	0.00732	0.01175	0.05639	0.28390	0.01328	0.00686	0.02518	0.01056	0.00509	0.00738
**Grin1**	0.00729	0.00808	0.01693	0.00576	0.00828	0.00621	0.00738	0.01754	0.01118	2.38388	0.01078	0.00002	0.51488	0.01777	0.00498	0.35316
**Grin2d**	0.00963	0.00781	0.00741	0.01696	0.00921	0.00744	0.00772	1.24560	0.11956	2.55940	0.07319	0.01916	0.01158	0.02900	0.10652	0.00752
**Grin3a**	0.01378	0.00792	0.01153	0.33735	0.00931	0.01289	0.00750	0.01377	0.00424	0.01281	0.01208	0.00777	0.01892	0.00862	0.05465	0.00731
**Grin3b**	0.00702	0.00787	0.06391	0.01017	0.00889	0.00831	0.01667	0.00965	0.01238	0.00905	0.02400	0.00685	1.48280	0.01251	0.00934	0.00647
**Grina**	0.51040	2.84801	0.00000	4.38263	0.03580	0.01619	0.02634	0.84051	1.06271	2.68183	0.18761	0.05570	0.80296	0.00000	0.70397	0.01362
**Grip1**	0.17937	0.00772	0.00398	0.34795	0.50677	0.00699	0.00713	0.01364	0.00411	0.07795	0.01030	0.00527	0.01086	0.00628	0.59179	0.00741
**Gripap1**	0.00000	0.00701	0.21031	1.54973	0.01173	0.81346	2.39458	2.40499	3.21287	0.16848	2.12125	0.17381	0.22480	0.00000	2.93378	0.00612
**Grk1**	0.12956	0.01814	0.04504	0.63259	0.03320	0.04229	0.06833	0.13834	0.52076	0.01751	0.15065	0.00406	0.01848	0.32837	0.40977	0.02127
**Grk4**	0.32070	0.62095	0.64112	2.25948	0.36734	0.04540	0.36884	0.18360	2.88866	1.20539	0.18649	1.02854	0.47321	3.87928	0.73449	0.15949
**Grk5**	2.20171	0.00389	1.44318	3.28619	0.38059	0.00617	1.71245	3.74201	6.53258	8.80976	1.39544	0.42645	0.05649	4.03011	7.75549	0.01086
**Grk6**	6.30455	5.52420	5.71082	0.32793	10.63646	5.20676	4.09513	7.43999	0.49269	4.69913	7.24822	5.48101	3.66109	0.55841	0.95702	3.44579
**Grm2**	0.01374	0.00788	0.01183	1.19508	0.00936	0.00645	0.00741	0.01252	0.33733	0.00652	0.01020	0.01727	0.01274	0.00598	0.22390	0.00714
**Grm4**	0.01347	0.00811	0.01118	0.07771	0.00916	0.00655	0.00747	0.01271	0.78142	0.00805	0.04265	0.01750	0.03725	0.01283	0.00374	0.01451
**Grm8**	0.01378	0.00781	0.01195	0.15255	0.00939	0.00654	0.00743	0.01231	0.00710	0.00734	0.01196	0.00734	0.01274	0.00974	0.30573	0.00735
**Grn**	8.70077	5.96232	12.62140	1.63666	5.78672	11.55198	16.90099	33.59950	2.64778	24.57674	29.81184	11.79529	26.92291	6.00703	4.62397	2.44347
**Grpel1**	24.05106	12.65919	19.06692	6.16436	9.03270	10.27577	10.10276	19.35129	7.98505	21.81081	13.63845	7.59107	21.86931	3.05773	5.47850	8.65303
**Grpel2**	2.98155	4.36800	6.76358	2.62860	0.17273	4.18289	4.47726	7.81756	1.72596	4.71681	3.92711	1.50963	0.81363	0.73538	1.79766	0.78901
**Grrp1**	0.01364	0.00818	0.01199	0.33095	0.00927	0.00659	0.00751	0.01376	0.78175	0.01520	0.00888	0.00692	0.01295	0.01166	0.00395	0.00739
**Grsf1**	14.79728	9.41043	7.72450	20.08601	8.77474	8.76477	1.96124	5.67072	18.25954	13.13380	7.24392	14.97797	8.25877	17.69764	21.23311	0.40489
**Grtp1**	8.28145	0.30916	3.78163	0.41150	7.35721	6.30457	3.21123	6.06865	0.00000	6.59832	3.76189	6.60547	5.84120	0.00000	0.00000	2.33889
**Grwd1**	15.25731	10.91156	15.36977	9.73380	13.73632	6.76439	9.98794	18.52446	12.16801	21.42976	8.43453	3.49768	14.73778	1.35030	8.61478	6.43235
**Grxcr2**	0.17767	0.00829	0.00561	0.00491	0.01718	0.00727	0.20841	0.01035	0.30461	0.01283	0.01041	0.00728	0.01191	0.01299	0.00829	0.00742
**Gsap**	0.00908	0.35812	0.00972	0.00497	0.00839	0.00670	0.00724	0.01088	0.15937	0.01511	0.01124	0.02725	0.01195	0.01078	0.00469	0.00719
**Gsc**	2.46449	0.00487	0.00333	0.00969	0.00332	0.00586	0.08744	0.00304	0.02381	0.00719	0.01216	0.00601	0.42303	0.01201	0.01571	0.01856
**Gsdma**	0.83863	0.00789	0.01152	0.52544	0.01993	0.00803	0.00667	0.42248	0.10836	0.93742	0.08819	0.01352	0.72515	0.00425	0.36278	0.03788
**Gsdma2**	0.01709	0.00888	0.01111	0.03181	0.01092	0.00684	0.00806	0.04551	1.10836	0.00737	0.00636	0.00689	0.02659	0.03988	0.00160	0.00751
**Gsdma3**	0.04369	0.00943	0.00666	0.01866	0.01579	0.00745	0.00807	0.58377	0.01078	0.00000	0.00794	0.00690	0.43422	0.22919	0.02436	0.00743
**Gsdmc**	0.30904	0.00784	0.14217	0.41278	0.00906	0.00621	0.08782	0.08984	0.08187	0.00015	0.13330	0.00656	0.17835	0.05282	0.01290	0.00678
**Gsdmc2**	1.90444	0.00545	1.45793	2.48974	0.00260	0.00387	0.41937	0.86694	1.08587	0.26991	0.25717	0.03527	1.97144	0.80866	0.65690	0.01032
**Gsdmc3**	0.30884	0.00769	0.40650	1.37224	0.00775	0.00581	0.13809	0.31666	0.36063	0.74021	0.07209	0.02959	0.51477	0.64815	1.23432	0.00634
**Gsdmc4**	0.46897	0.00760	0.42143	1.57711	0.00773	0.00559	0.14796	0.18961	0.31224	0.05093	0.41188	0.02421	0.48643	0.10264	0.14069	0.00596
**Gsdmd**	0.50326	0.64229	0.53510	0.46854	0.20876	0.00852	0.01637	0.00000	0.84803	0.00000	0.33988	0.00368	0.01427	0.00567	0.00000	0.04314
**Gse1**	0.71804	0.00682	0.80572	4.80962	0.01150	0.00639	0.00533	3.43497	2.08629	4.53930	1.62376	0.27850	1.06226	0.00000	0.57701	0.01921
**Gsg1**	0.01693	0.00796	0.01093	0.00451	0.00916	0.00679	0.00744	0.02778	0.44177	0.01435	0.00905	0.05770	0.01464	0.01325	0.00550	0.00740
**Gsg1l**	0.03341	0.01804	0.10925	0.00895	0.02591	0.02429	0.00724	0.02314	0.19646	0.02787	0.00830	0.30317	0.03118	0.13835	0.10344	0.00744
**Gsg2**	0.00000	0.39585	2.58538	15.70550	3.67206	0.06419	4.16147	3.96786	10.14299	2.85814	2.16296	3.88755	3.08245	0.00000	8.59374	0.01549
**Gsk3a**	1.11027	0.05750	0.08690	7.65556	3.76015	0.05928	0.00483	0.00000	11.47047	0.76342	3.70866	0.33969	0.15964	0.26237	0.00000	0.01070
**Gsk3b**	0.78639	1.02789	2.18786	9.37710	0.24279	0.08549	1.99005	3.68755	8.20705	6.60978	3.30011	3.51811	2.64762	9.15696	7.08468	0.08824
**Gskip**	1.59789	0.15802	0.00000	4.02870	0.01463	1.59352	0.00452	0.41516	3.89932	1.73959	1.90309	0.19461	1.15592	3.96602	1.89883	0.01079

**Gsn**	0.02694	3.34823	2.79528	0.00000	0.03918	5.79790	0.00000	0.01197	0.01065	4.62413	0.52284	0.05071	9.16080	0.00860	0.00000	0.02890
**Gspt1**	18.79631	16.78715	11.50834	7.13173	13.32252	9.70003	9.40987	15.13250	11.35096	19.98223	11.07634	19.38460	12.11954	28.81804	19.50039	6.24578
**Gspt2**	3.44353	2.41823	0.00000	2.25401	0.00001	0.00317	0.00373	0.27182	0.00000	2.26357	0.12030	0.13077	1.30845	0.01936	3.67418	0.00171
**Gsr**	6.49992	4.30022	2.51824	1.56124	4.60703	1.27529	2.69541	2.92146	0.80078	9.58019	6.27086	0.58436	2.81497	2.64177	4.56853	0.94808
**Gss**	12.32376	0.87122	8.77481	7.42926	13.02771	11.09366	7.58826	7.05787	3.26343	2.46580	0.62938	1.72338	9.21957	0.00674	0.06061	3.97673
**Gsta1**	0.59478	0.00680	0.00000	0.01860	0.00657	0.45664	0.10827	0.69928	0.00906	0.97848	1.41500	0.08648	1.25386	1.45510	0.00000	0.01276
**Gsta2**	0.62163	0.00673	0.00000	0.02604	0.01519	0.42845	0.41603	0.40703	0.00882	0.48831	1.10148	0.04456	0.91434	1.41266	0.22721	0.00595
**Gsta3**	2.22253	0.00559	0.00328	0.00999	0.00372	0.00660	0.00569	0.00773	0.01221	0.15229	0.01141	0.01787	0.06907	0.01154	0.01156	0.00490
**Gsta4**	10.29024	4.17826	6.46341	0.50731	6.35453	13.54514	3.22400	0.10937	0.36845	1.13511	2.10866	0.08769	10.94424	1.15551	2.36925	11.01591
**Gstcd**	0.03512	0.00817	0.29023	0.58109	0.00853	2.03384	0.00788	0.04683	1.68066	0.32139	0.17444	0.01521	0.03769	0.00409	0.19593	0.02149
**Gstk1**	0.00721	1.99996	0.00307	0.19198	0.00796	1.94759	0.00653	0.00980	0.00574	0.09400	0.00876	0.00808	0.00447	0.00554	0.00000	0.00585
**Gstm1**	7.55462	0.00000	0.48546	0.01759	4.45126	0.01980	2.49196	0.00000	0.23729	16.36803	0.00408	0.00000	0.01521	0.01488	0.00000	0.01362
**Gstm2**	2.75086	0.00486	0.00000	0.00000	0.00531	3.31735	0.00450	0.00299	0.18309	13.64261	0.00500	0.00000	0.60921	0.01401	0.00467	0.01040
**Gstm3**	1.38245	0.00471	0.05422	0.00362	0.44016	0.00438	2.52568	0.00429	0.01104	1.91252	0.01044	0.00000	0.01006	0.01303	0.00637	0.00579
**Gstm4**	3.71475	0.49839	2.12295	0.00342	0.00000	0.00723	0.00403	0.00000	0.78125	0.61247	0.00477	0.00000	0.00982	0.01152	0.00000	0.17822
**Gstm5**	0.01317	0.00764	0.01011	7.30781	0.00911	0.00578	0.00703	0.01022	0.00000	0.64118	0.00811	0.03132	0.01185	0.01176	1.54353	0.00636
**Gstm6**	2.65577	0.00464	0.19751	0.14150	0.82971	0.00936	0.15982	0.00252	0.00576	0.01752	0.01133	0.00557	0.00987	0.01215	0.00481	0.00439
**Gstm7**	1.60589	1.31127	0.77884	0.00000	2.99815	0.00385	0.00563	0.00000	0.00952	4.28674	0.00836	0.00000	0.00878	0.01108	0.00000	0.02000
**Gsto1**	15.86243	11.21661	19.05876	0.23920	23.34211	23.50333	16.97780	36.89981	1.75575	16.98852	11.71822	0.65946	40.29738	1.98509	0.00000	17.07542
**Gstp1**	26.37735	10.26765	20.95686	1.13851	16.93479	13.24004	7.78344	12.60348	0.00741	7.38724	11.86414	0.33676	19.43473	0.00000	0.83392	13.97593
**Gstp2**	22.50629	12.23862	23.92448	1.12989	16.88821	13.55358	8.94330	12.37483	0.00000	5.19268	11.12774	0.22118	17.35369	0.00000	0.27622	14.93033
**Gstt2**	0.01141	0.00803	0.06597	0.00581	0.00953	0.00642	0.01850	0.01288	0.01144	1.84895	0.01204	0.00274	0.08661	0.00703	0.09780	0.00783
**Gstz1**	5.67529	4.29059	5.78822	0.42416	0.04025	3.63462	0.91931	3.55570	0.16594	1.20023	1.52638	0.40059	6.50111	2.90076	0.25069	0.11009
**Gt.ROSA.26S**	0.21437	0.00781	0.78342	5.78349	0.00628	0.03857	0.00621	0.56582	2.12044	0.06419	0.70346	0.56071	2.41066	0.00000	1.56052	0.00460
**Gtdc1**	0.62912	2.46638	1.03786	14.49361	0.01138	0.03763	0.18999	0.96193	12.81086	2.76191	0.71052	0.70104	0.07016	1.97100	7.63146	0.05357
**Gtf2a1**	3.94567	0.21432	2.87869	15.56995	0.10702	2.94836	6.01874	4.30798	13.29298	0.00000	2.62544	5.40853	2.38424	13.02508	13.56620	0.15585
**Gtf2a1l**	0.01375	0.00812	0.01199	3.20223	0.00930	0.00636	0.00740	0.01329	0.22808	0.01176	0.02542	0.01777	0.01238	0.00802	0.00728	0.00685
**Gtf2a2**	12.76885	4.08893	3.77394	3.35290	5.68867	3.97403	4.31732	4.35107	0.66044	1.59085	2.21375	1.71078	6.22484	1.81736	0.59202	7.18729
**Gtf2b**	17.67007	8.83110	10.34055	6.53452	6.76632	2.24788	3.46831	5.33987	11.76493	4.07888	1.77456	1.37733	6.21273	2.56076	1.04757	8.99576
**Gtf2e1**	5.65817	8.91505	6.08905	5.75561	6.97077	0.64722	7.27939	5.80284	2.81694	3.91036	3.23547	1.29371	3.46253	1.96440	5.92389	3.34893
**Gtf2e2**	11.21792	7.79013	7.51844	3.50020	11.47587	3.45300	6.25381	7.59224	6.34910	5.80834	3.28441	8.09831	7.72456	6.91226	7.02227	7.57184
**Gtf2f1**	12.74452	9.44574	9.91555	12.22338	8.49822	8.08388	5.94468	21.88017	16.99848	20.68741	7.76343	5.48787	18.60047	0.00000	0.00000	12.15860
**Gtf2f2**	12.17457	6.94625	7.56859	10.59639	9.29414	2.54266	3.62041	16.90051	20.60663	13.60708	11.50708	8.09075	8.50099	4.11766	7.49499	4.94823
**Gtf2h1**	12.63561	8.08594	8.87822	10.88546	3.19832	2.98710	3.39382	4.43578	14.05258	12.44260	10.62489	1.97768	8.67427	18.56477	17.94427	4.83858
**Gtf2h2**	9.47509	8.33037	6.66990	8.03457	5.74505	9.16059	7.03897	13.49317	5.98526	15.08291	12.55962	19.04157	7.99822	10.33487	13.61538	6.68244
**Gtf2h3**	2.54048	2.07670	3.43665	0.88218	5.07707	0.01039	1.22858	4.67684	0.45051	4.13849	1.13794	0.17411	0.57909	3.30265	8.74684	2.00250
**Gtf2h4**	5.99584	0.00000	0.00000	0.01518	8.98878	3.57004	0.33295	6.52521	0.05009	16.15738	0.00000	0.12414	10.96849	0.00549	0.05883	4.18360
**Gtf2h5**	11.92652	8.17849	8.79905	9.95710	5.20628	4.48407	5.47923	8.00251	9.13185	6.64922	14.20784	3.31485	7.98639	6.87416	8.45908	1.05683
**Gtf2i**	5.76053	3.72670	2.00183	6.97734	0.01924	0.00000	0.61206	1.92000	4.63080	11.61060	3.15038	0.21362	3.54467	0.01758	4.06540	4.69544
**Gtf2ird1**	6.11587	6.25988	5.53875	3.05399	5.71658	3.49296	6.40283	3.45566	3.38588	2.67232	10.31061	3.65701	4.33971	1.32148	1.05731	2.86607
**Gtf2ird2**	0.01126	0.00751	0.00245	0.41260	0.00902	0.00741	0.00698	1.02041	1.54394	0.90973	0.61516	0.00545	0.00286	0.01789	0.51203	0.00714
**Gtf3a**	0.46937	0.11735	0.25559	0.00714	0.00674	0.00639	0.18708	0.11125	0.01167	0.96206	0.01084	0.00531	0.66891	0.01327	0.00369	0.01411
**Gtf3c1**	3.55049	1.02128	1.41757	14.04378	2.24463	5.61802	1.35807	6.26646	14.31924	15.27443	11.49631	1.28841	0.29518	0.00000	3.78552	2.03143
**Gtf3c2**	6.31207	7.85605	9.66785	16.66985	1.59730	0.82634	7.40545	10.18118	17.08733	13.20531	7.13087	1.15193	9.15872	17.76901	19.46958	5.93232
**Gtf3c3**	2.82680	3.67388	3.02010	6.60807	2.98685	0.00208	3.69404	3.12679	10.05999	2.92374	3.24331	1.05918	0.85708	12.79883	9.45903	0.02812
**Gtf3c4**	2.46056	3.85985	2.99150	12.66380	3.45692	2.85190	4.81665	6.35629	12.36619	6.79578	5.53662	8.97576	1.58109	11.41717	8.93242	0.01030
**Gtf3c5**	3.52872	2.23909	2.66291	14.94178	6.57074	0.00000	0.10227	1.77330	16.85360	8.10166	3.73326	2.29229	4.72065	0.56397	10.62373	1.24083
**Gtf3c6**	8.49022	3.31485	4.16317	9.22569	1.53570	0.52893	1.70879	2.35439	19.84924	5.59387	6.35588	0.93175	5.37730	4.67822	3.51600	1.59610
**Gtpbp1**	5.71140	4.83181	1.61601	1.26607	0.03809	2.19482	2.58856	2.53052	0.82454	3.01965	0.23554	0.08210	5.27248	0.01612	0.00000	0.16377
**Gtpbp10**	1.20949	1.71891	1.69357	2.10129	0.88417	0.16194	0.64540	2.34441	0.65822	1.03229	2.66224	4.56440	0.00000	0.84214	12.23124	0.11173
**Gtpbp2**	0.11107	0.00741	0.03127	0.78221	0.00793	0.00717	0.02755	0.10719	0.00000	0.05857	0.19206	0.28310	0.01604	0.15099	0.07304	0.06142
**Gtpbp3**	2.98346	0.00326	1.04100	1.02900	2.03072	0.00910	0.18018	1.43526	4.22435	1.74970	0.22446	0.07126	1.38793	0.26601	0.27205	0.34513
**Gtpbp4**	11.32072	18.46756	14.03327	14.90939	10.59029	6.60990	12.40337	15.55862	17.85837	18.05375	11.54000	8.27981	9.18334	25.88862	19.51335	7.58687
**Gtpbp6**	0.69696	0.00713	0.00000	0.00571	0.13578	0.50030	0.01699	0.99466	0.01045	1.63505	0.00320	0.04186	0.71875	0.01492	0.00000	0.18213
**Gtpbp8**	3.30400	4.99042	0.00000	0.65884	1.86264	4.01569	0.00000	2.31422	0.44313	0.13446	2.71740	6.63319	1.04359	3.39583	1.34080	0.08043
**Gtse1**	8.65521	7.29301	9.13782	5.38326	6.16215	1.15857	2.34111	2.60888	8.07555	2.55214	2.98248	0.52792	4.83612	0.00000	2.54880	1.40853
**Gtsf1**	1.62812	0.14810	1.32082	1.30290	0.00709	0.00513	0.00276	0.21993	1.87218	1.97182	4.32542	0.00000	2.51684	0.00000	1.43581	0.00310
**Gtsf1l**	14.90029	1.71386	3.89593	0.84908	5.65324	2.62758	0.00605	0.93423	0.14787	2.45616	0.38641	0.96861	0.61279	0.13829	0.68723	0.02530
**Guca1a**	16.81445	0.17659	0.10528	0.76695	6.10056	10.71268	6.29687	27.51000	3.26430	33.89529	1.78614	2.26454	24.36497	0.00000	0.41215	3.70131
**Guca1b**	0.00819	0.00784	0.01361	1.10831	0.00910	0.00668	0.00750	0.01342	1.68697	0.05926	0.00000	0.00326	0.26083	0.01133	0.55045	0.55805
**Gucd1**	2.12849	4.61176	3.98404	1.36555	0.96771	0.01128	0.00179	1.49616	1.84934	2.39746	2.19923	5.46835	0.82552	1.64171	4.17232	1.33307
**Gucy1a2**	0.05294	0.01828	0.06110	0.02686	0.00797	0.01307	0.00741	0.01807	0.02028	0.04139	0.01052	0.02718	0.01831	0.18231	0.07783	0.01446

**Gucy1b2**	0.00997	0.00756	0.02071	0.01090	0.00952	0.00780	0.00712	0.51435	0.01187	0.00769	0.01024	0.00647	0.00573	0.01303	0.00680	0.00739
**Gucy2c**	0.01377	0.00780	0.01194	0.80171	0.00935	0.00657	0.00748	0.01321	0.14847	0.01077	0.01138	0.00751	0.01277	0.01722	0.06707	0.00726
**Gucy2e**	0.00000	0.01817	0.00000	2.29463	0.00791	2.76989	0.00437	2.88030	2.43288	5.72933	4.16050	0.46852	2.29695	1.16001	3.91590	0.01122
**Guf1**	0.20483	0.00536	0.00000	0.90794	0.05841	0.00648	3.52811	0.00661	0.06217	1.29155	0.99166	0.09277	1.63254	0.39947	0.94444	0.01311
**Guk1**	10.94284	3.59928	8.13953	0.56480	6.11665	5.70549	1.88775	4.10697	0.33163	7.92401	9.38959	5.26193	10.63383	0.01427	0.53192	8.69741
**Gulo**	17.02431	5.71508	19.95769	2.32932	7.31648	7.52440	20.07667	33.69207	3.09322	29.89379	25.81134	11.98621	19.20430	6.65662	13.84579	4.21753
**Gusb**	1.17506	3.02961	0.00000	2.28810	0.07717	0.73030	0.00264	4.18616	3.93101	1.36948	1.87972	0.11716	3.74180	1.31565	3.69072	0.00218
**Gxylt1**	1.66626	0.01089	0.53158	9.41408	0.05330	1.63591	6.58066	2.60340	8.69338	3.95844	2.22535	6.42518	1.42681	7.51004	5.76191	1.32186
**Gyg**	0.41168	2.34455	0.51229	11.77740	2.44642	0.00554	0.02849	0.34340	8.51348	1.01189	1.01824	0.19661	1.88230	3.24352	9.15449	3.89127
**Gyltl1b**	0.01104	0.00769	0.12561	0.00592	0.01199	1.42726	0.00633	3.74437	0.00913	0.63135	0.05567	0.00000	1.81216	0.01390	0.00000	0.38903
**Gys1**	3.40756	1.05405	3.99124	2.02993	3.93171	0.28891	0.87871	4.43253	4.40332	1.92500	1.65570	0.42701	1.67192	0.64122	0.76603	3.74014
**Gzf1**	4.15161	3.51969	5.79325	6.15512	0.29060	0.65811	0.31411	0.48379	2.19512	3.19751	0.70141	0.00949	0.00000	0.55175	4.14396	0.53156
**Gzmm**	1.27242	0.00576	1.55472	1.03445	0.00282	0.00470	0.00617	0.21541	0.00010	0.91259	0.23949	0.37760	0.00696	0.08252	0.00000	0.00547
**H13**	7.43151	0.36929	6.79054	3.66987	5.20189	2.67538	1.69151	6.30593	4.67633	7.31457	5.96033	5.65153	6.09850	0.39011	0.43202	4.90627
**H19**	0.00447	0.00796	0.01101	0.01063	0.00582	0.00615	0.00727	0.01405	0.01241	0.00484	0.01252	0.00757	0.00463	0.01347	0.01019	6.02312
**H1f0**	1.45848	0.03034	0.55402	5.13519	0.03704	0.01183	0.15216	0.05346	0.80630	4.35406	0.69740	0.17463	0.71384	2.04343	1.67351	1.88876
**H1foo**	0.00768	0.06817	0.10300	8.85431	0.00924	3.74448	0.27700	0.90451	4.51933	2.73311	2.11182	0.03922	0.00000	0.62699	1.12706	0.00409
**H1fx**	0.01348	0.00798	0.01268	0.06394	0.00949	0.00665	0.00750	0.01358	0.01140	0.06767	0.01406	0.00752	0.01538	0.01130	0.00856	0.02983
**H2.Aa**	0.01223	0.00779	0.01224	0.02176	0.00860	0.00665	0.00731	0.01013	0.01114	3.02163	0.01005	0.00000	0.01092	0.01059	0.01372	0.00747
**H2.Ab1**	0.00879	0.00793	0.02160	0.00508	0.00767	0.00633	0.00716	0.01341	0.01080	4.23239	0.04962	0.00000	0.41518	0.01255	0.00597	0.99413
**H2.Bl**	0.80029	0.01778	0.42438	2.61200	0.21488	4.86046	0.08728	0.10881	0.88278	0.34729	0.39273	0.01140	2.30068	0.00349	0.00097	0.11535
**H2.D1**	0.46467	0.08238	0.86646	2.74198	0.51992	0.73901	0.16227	0.35969	2.39516	1.52842	1.64929	0.02605	0.70379	0.69146	0.50256	3.11181
**H2.DMa**	0.00134	0.94495	0.09863	1.71212	0.00780	0.00563	0.10552	0.65879	0.80061	1.01783	1.58493	0.01243	3.05037	0.03783	0.17607	0.00522
**H2.DMb1**	0.09924	0.00731	0.00809	0.23918	0.00789	0.00653	0.00730	0.00980	0.00812	0.32722	0.02295	0.00498	0.01183	0.00494	0.65864	0.00725
**H2.DMb2**	0.02053	0.00773	0.01193	0.00912	0.00891	0.00655	0.00746	0.01232	0.01230	0.09005	0.01223	0.00695	0.01249	0.00600	0.16867	0.00738
**H2.Eb1**	1.20094	0.01882	0.00881	0.89864	0.00540	0.00755	0.01822	0.50123	3.60815	5.03458	0.07716	0.01206	0.00574	0.00629	0.03029	0.01305
**H2.K1**	7.60147	0.04401	9.85157	10.06679	4.09080	11.35612	2.50842	6.31658	6.00114	5.79311	7.24991	0.12719	15.88256	0.04164	1.49056	4.87129
**H2.K2**	6.81665	0.00408	1.14637	0.21962	10.58757	0.01820	1.91968	0.26103	1.88087	0.84069	0.79976	0.03667	0.96615	0.12057	0.30150	0.04270
**H2.Ke6**	1.39304	0.00556	1.15716	0.51436	0.00301	0.00823	0.08480	1.05532	0.00351	0.06477	1.52466	0.03910	0.00000	0.00260	0.00000	0.02656
**H2.M5**	0.85342	0.00683	0.00401	0.01842	0.00613	0.00710	0.00662	0.02270	0.01241	0.01273	0.01248	0.01729	0.05731	0.01409	0.01508	0.01347
**H2.Oa**	1.26024	0.00568	2.04881	0.08240	0.01004	0.00791	0.00477	1.55122	0.00771	0.24160	0.34927	0.00000	1.97212	0.00815	0.00000	0.01189
**H2.Q1**	0.39678	0.00710	0.19399	0.51768	0.48299	0.34134	0.16044	0.28249	0.31783	0.30540	0.03484	0.01419	0.71753	0.01573	0.12978	0.15051
**H2.Q10**	0.17091	0.00744	0.12421	5.27012	0.31104	0.17825	0.22330	0.57064	10.27623	2.32877	0.21872	0.08988	0.87687	1.50453	1.26486	0.10846
**H2.Q2**	1.09397	0.00607	0.06891	0.00814	0.99473	0.09420	0.16970	0.11421	0.02360	0.10841	0.01100	0.00534	0.20943	0.00933	0.02636	0.09182
**H2.Q4**	0.31577	0.00728	0.09100	0.16234	0.11112	0.19641	0.13827	0.30359	0.21923	0.56215	0.00000	0.00786	1.10316	1.04487	2.08002	0.21892
**H2.Q5**	0.02110	0.00766	0.10864	2.41447	0.04225	0.04233	0.03946	2.84278	8.39645	1.98264	0.34172	0.03485	1.24589	0.09039	0.07618	0.01309
**H2.T.ps**	0.46414	0.00719	0.28298	0.48376	0.40663	0.26974	0.13977	0.39280	0.03016	0.14276	0.04809	0.00496	1.35505	0.04028	0.06918	0.27371
**H2.T10**	0.06865	0.17250	0.06030	2.04405	0.09845	1.07168	0.05652	0.13447	2.85440	0.00000	0.24228	0.01575	0.32122	0.66103	1.01227	0.02061
**H2.T23**	2.01654	0.16567	1.24988	7.43622	1.13737	0.43296	0.26839	0.00000	12.75852	0.22409	12.22807	1.01291	1.03941	4.95435	0.69886	0.49149
**H2.T24**	0.18499	0.01762	0.29640	2.38169	0.17936	0.03769	0.03739	0.00000	3.20046	0.00000	2.07747	0.10586	0.08640	1.27029	0.46646	0.06330
**H2.T3**	0.05248	0.03961	0.06777	0.09825	0.00864	0.01965	0.00734	0.00760	0.08325	0.05116	0.17288	0.02691	0.05797	0.05626	0.04751	0.00727
**H2afj**	0.21042	0.07562	2.88662	0.41537	0.63633	0.04132	0.11542	0.04399	0.69211	4.23982	0.49321	0.05327	0.34816	0.04932	0.25979	0.06398
**H2afv**	1.35387	0.06383	2.85890	8.49826	2.14674	2.55362	2.88152	2.40058	15.63922	14.85018	16.23864	0.62177	5.54181	1.59930	2.06599	1.09830
**H2afx**	5.21179	0.55124	1.91490	0.00000	2.48341	0.02555	0.91054	0.72370	7.70796	22.60505	6.97132	0.68530	11.41553	0.96183	3.17414	2.39845
**H2afy**	22.94358	8.58354	12.32594	0.00000	12.27400	12.37823	2.12277	8.67746	0.14334	1.66181	4.11255	0.03863	4.49385	2.09834	0.00000	1.10998
**H2afy2**	0.01605	0.00787	0.19780	0.02352	0.00846	0.00689	0.00750	0.01471	0.34907	0.41993	0.01098	0.00613	0.00971	0.00814	0.22640	0.00704
**H2afy3**	0.01296	0.00788	0.04281	0.07768	0.00923	0.00813	0.00741	0.01323	0.01157	0.02704	0.01361	0.00747	0.01209	0.00865	0.03643	0.00742
**H2afz**	55.69449	23.64024	38.01529	27.18616	33.56518	20.14050	23.18473	34.93476	42.57274	43.80811	26.62843	24.77626	37.99328	9.34287	1.57278	27.92578
**H3f3a**	32.24050	13.61988	30.45568	22.21944	20.17427	15.82698	17.73667	40.09516	31.56202	45.40683	29.85004	15.66410	31.91092	21.05936	9.64938	15.89499
**H3f3b**	30.38786	18.35493	25.98202	26.16459	21.46646	19.67266	22.96001	32.56240	40.78679	52.43823	33.76977	24.50596	35.23264	27.61345	22.66683	21.30798
**H60b**	0.02324	0.00666	0.41207	0.00703	0.06970	0.00535	0.00677	0.45839	0.01024	0.00770	0.10912	1.83107	0.00373	0.04686	0.00000	0.00716
**H6pd**	0.02069	0.00799	1.69432	0.00888	0.00652	0.00630	0.02950	0.01401	0.01175	0.49428	0.01082	0.05387	0.07767	0.01439	0.00000	0.00708
**Habp4**	0.29790	0.01791	0.10729	0.72860	0.00726	1.90516	0.03817	0.00795	0.41383	0.09406	0.02787	0.02808	0.45084	0.03222	0.21939	0.00609
**Hacd1**	0.63404	0.11315	0.12795	0.67585	0.00669	1.94935	0.03634	0.00677	0.00000	1.48643	0.00875	0.11605	0.00536	0.01592	0.00000	0.01257
**Hacd2**	3.64517	0.61708	3.66293	2.26767	5.07470	0.62554	3.35476	0.00000	6.02540	1.54970	2.35465	3.37157	1.10475	0.62833	1.05363	0.87118
**Hacd3**	2.89875	0.02216	5.99870	11.18273	2.51281	0.00000	2.24263	2.09960	19.04816	6.74261	13.03015	0.19506	2.67806	2.71652	10.42783	0.04519
**Hace1**	0.20895	2.18209	0.00000	0.00000	0.14047	2.09107	0.05594	0.24813	0.03623	0.00000	0.28972	1.32958	0.00000	0.00609	0.23745	0.00527
**Hacl1**	0.28681	0.00594	1.76290	0.78724	0.00432	0.00523	2.56859	0.10529	0.00000	0.00000	0.00760	0.00000	1.49791	0.03954	2.13520	0.00535
**Hadh**	12.61371	5.32666	13.35220	0.00000	14.87438	13.72235	6.13231	18.55950	0.00000	5.00532	8.15143	0.56946	16.48899	0.30636	0.65797	7.44769
**Hadha**	10.50563	11.52118	10.48799	2.09016	7.05846	9.96219	11.05370	6.56269	0.55738	3.48431	5.33444	0.27060	7.90456	3.14525	4.50740	7.44432
**Hadhb**	6.75467	10.03967	8.60766	7.15263	11.53840	5.79095	6.81926	12.08290	4.42929	9.73745	8.11651	3.86870	6.27378	7.32885	13.57149	5.38838
**Hagh**	3.58543	1.06116	4.67319	1.68731	4.72208	0.84727	1.07789	0.05950	0.86415	0.00780	0.00000	0.01544	2.34218	0.03065	0.21851	4.42443

**Haghl**	0.01367	0.00859	0.01157	0.01029	0.01062	0.00663	0.00738	0.02826	0.01236	0.07326	0.01223	0.00764	0.98488	0.01205	0.01056	0.00701
**Hal**	2.80781	0.00132	0.98686	0.00431	0.05025	0.01385	5.59206	0.01342	0.01157	0.96477	0.01048	0.00000	0.03947	0.01434	0.00000	0.00411
**Hamp**	0.01378	0.00795	0.01201	0.46975	0.00931	0.00661	0.00748	0.01345	0.00527	0.01390	0.01366	0.02778	0.01269	0.00866	0.02626	0.00733
**Hand1**	1.01254	0.00412	0.28132	0.81353	0.04669	0.00432	0.00000	4.78458	0.15524	24.66952	16.51394	1.63927	0.00000	1.31068	2.98972	0.00328
**Hap1**	0.19782	0.00834	0.28444	0.00518	0.01371	0.00838	0.09181	0.12665	0.01102	0.23909	0.58430	0.00431	0.00303	0.01321	0.00772	0.00687
**Hapln4**	0.00000	0.00738	0.00491	0.33683	0.00853	0.00677	0.00642	1.00763	0.03161	1.01320	0.17352	0.01495	1.75483	0.95519	0.37613	0.70244
**Harbi1**	1.63521	3.30865	4.61882	0.51570	2.76089	0.88343	2.31316	0.98525	0.07055	1.02659	0.65101	0.05790	1.70491	0.01665	3.09423	0.00853
**Hars**	13.30705	9.63023	10.94262	3.62858	13.52063	7.12316	9.64328	12.74380	10.16613	15.25583	8.39318	3.83357	10.18362	0.21814	8.36653	11.83184
**Hars2**	5.49356	5.40406	4.98600	3.10144	1.47817	1.14837	3.38191	2.62567	4.39808	5.86432	1.54712	4.28790	5.42197	6.40813	6.00011	2.32165
**Has3**	0.05497	0.00832	0.00000	2.45431	0.01463	0.00586	0.00709	0.00822	1.05838	0.00000	0.00000	0.01831	0.01335	1.83052	4.21052	0.00682
**Hat1**	25.16818	17.32705	24.85140	2.89467	21.59092	7.33397	10.70169	15.42069	4.56861	17.93412	14.03826	10.94876	14.34423	13.11622	14.01368	8.27574
**Haus1**	17.57268	7.02157	7.45836	0.68246	9.89369	3.13771	6.93547	4.89660	1.36266	3.43688	3.54423	0.71836	4.66015	3.44531	2.09856	2.91576
**Haus2**	3.99413	4.21302	6.21586	4.62533	5.20063	0.67821	2.17785	6.24947	6.16285	7.94122	9.17769	5.58769	5.98873	9.21135	12.90229	0.35745
**Haus3**	5.23797	10.22921	6.04381	10.15635	2.70894	1.04890	5.48745	4.99213	18.27491	10.31365	3.08512	13.99106	5.09343	11.27817	11.57542	1.03331
**Haus4**	5.90400	4.20336	5.80824	13.35642	0.04939	3.46544	3.48131	4.92063	10.32346	13.39763	12.29012	0.85974	9.82398	0.42830	2.64365	0.25533
**Haus5**	9.31804	9.79353	7.82724	1.79645	7.05304	7.69288	4.86862	11.41780	1.03543	7.44108	3.55061	2.43929	6.98150	0.05772	0.80669	1.67087
**Haus6**	1.79205	3.67439	0.80526	6.91927	3.71807	2.20544	3.92850	2.49212	9.25162	5.12392	1.79985	1.22714	2.46502	20.54865	16.37363	1.35399
**Haus7**	6.06215	6.69296	7.40064	1.82743	5.80832	0.01323	3.72209	0.27097	3.54290	2.28706	2.30805	0.02002	1.99128	1.26343	1.07370	2.72097
**Haus8**	3.67359	7.32065	7.62484	1.21531	7.46253	9.70332	9.96620	6.60999	1.62324	5.70661	2.64254	1.43718	9.69408	0.00000	0.37074	4.37577
**Havcr1**	2.79214	4.24703	10.14440	0.14976	5.92580	8.66956	8.57520	6.53628	1.88086	6.62775	7.06617	9.57760	7.27219	14.76534	6.38959	5.51007
**Havcr2**	0.02010	0.04012	0.01021	0.00840	0.02014	0.00662	0.00745	0.01950	0.01175	0.00691	0.01170	0.03610	0.01216	0.01322	0.37056	0.00740
**Hax1**	13.33290	14.48435	18.26707	18.80783	7.72867	8.73481	10.21654	27.50791	21.04372	28.58432	27.25076	8.47879	22.46336	24.58060	24.58255	8.87995
**Hbegf**	0.45967	0.00704	0.01117	2.06867	0.00684	0.00708	0.00679	0.00712	1.66613	0.63655	0.03262	0.03483	0.01658	0.06325	0.14060	0.00611
**Hbp1**	1.11566	9.57754	3.09966	14.90363	0.02773	1.26086	5.40851	9.36083	18.82358	16.91794	10.69505	9.80661	2.38072	14.52758	16.06916	1.46251
**Hbs1l**	9.09658	1.91912	7.26039	2.66674	2.83947	2.91853	8.28995	4.03101	0.49984	3.13754	4.49914	4.75445	9.60902	5.59587	3.07212	4.58140
**Hccs**	0.35788	2.77366	2.23660	8.87612	3.69043	0.79175	2.41824	3.94906	9.30472	6.14350	2.33360	8.89514	1.64538	13.42986	10.46536	1.27616
**Hcfc1**	11.93375	10.02126	6.07659	6.65813	9.00886	1.65896	7.61686	13.07168	12.12168	16.02284	9.75436	1.97192	7.05104	9.50024	19.11643	4.12486
**Hcfc1r1**	8.42001	0.60816	4.31303	0.99330	5.66863	1.92667	3.32171	6.27787	0.28951	7.44523	1.57750	0.10584	3.70318	0.00000	0.18830	1.84524
**Hcfc2**	0.94715	0.00769	0.00000	1.20029	0.01600	3.35923	0.00628	0.00618	0.00000	0.70923	0.02440	0.00547	0.00077	0.00539	0.11736	0.00492
**Hck**	0.47262	0.00503	0.00000	0.00855	4.14060	0.01407	0.00680	0.00263	0.01082	0.00635	0.00706	0.00887	0.02780	1.09318	0.00497	0.00665
**Hcn3**	0.00137	0.00630	0.00000	0.00186	0.00955	0.22187	0.59632	1.37998	0.23359	2.57660	0.40627	3.06512	0.24283	0.01532	0.25416	0.00703
**Hcrt**	0.01199	0.00676	0.01006	0.02011	0.00800	0.00657	0.00642	4.63229	0.01676	5.88352	0.98071	0.00001	1.07847	0.00807	0.00000	0.00678
**Hdac1**	22.95355	15.46995	18.06926	13.70053	14.49764	8.74096	14.61592	32.60723	17.57886	26.93883	21.45768	15.47413	15.92637	30.30307	26.00452	4.58550
**Hdac10**	1.69837	0.00599	0.00148	0.84031	0.01387	0.00661	0.00595	0.00231	1.19471	0.20564	0.08440	0.03145	0.01116	0.18370	0.38285	0.01341
**Hdac11**	0.01276	0.00799	0.01412	0.00736	0.00996	0.00705	0.00755	0.01275	0.03383	0.63433	0.01423	0.00567	0.10829	0.01165	0.00829	0.00796
**Hdac2**	10.80820	13.67962	10.32471	6.20746	9.61727	10.36674	7.94794	8.25258	2.65829	8.96013	5.72744	5.01947	7.13326	4.63515	3.85003	4.59737
**Hdac3**	3.86647	4.21836	1.74472	0.00000	0.00000	0.13144	1.28088	0.88244	0.01043	1.28987	0.04104	0.12841	2.42215	0.11711	0.37347	1.67879
**Hdac4**	1.11386	0.00631	2.19993	0.26055	0.01079	0.00601	0.00520	1.00178	1.90665	0.00000	0.00000	0.05518	1.26184	0.01336	0.00000	0.00464
**Hdac5**	0.74907	0.00579	0.35336	0.03990	0.00586	0.93892	2.10709	1.70250	0.59147	5.66357	0.87129	0.10301	3.24689	0.02961	0.00000	0.00445
**Hdac6**	4.56468	2.64201	0.98959	8.44328	0.12722	0.79906	3.04163	6.66851	8.91977	7.88928	4.40775	1.23201	5.92869	2.64202	10.80948	2.63140
**Hdac7**	0.02067	0.00804	0.05173	0.00817	0.00852	0.00596	0.00747	0.01209	0.01191	0.63494	0.01206	0.00673	0.01836	0.01187	0.00914	0.00757
**Hdac8**	0.00484	0.00725	0.00454	0.19112	0.00973	0.00645	0.42759	0.00186	0.68862	0.00000	0.18865	0.00745	1.02287	1.74320	0.21419	0.00657
**Hdac9**	0.01224	0.00834	0.01308	1.24606	0.01023	0.01376	0.00755	0.01415	0.04859	0.00760	0.01311	0.00719	0.27735	0.00636	0.59971	0.00741
**Hdc**	0.20645	0.00925	0.06363	1.42209	0.01677	0.00541	0.00778	1.11882	1.47383	2.38689	0.93871	0.03033	0.00000	0.50310	0.00000	1.68621
**Hddc2**	0.55838	0.00683	3.15287	0.23248	0.21794	0.04755	0.00655	0.98570	1.05961	2.98231	0.06351	1.74729	0.01255	0.00456	0.42210	0.05891
**Hddc3**	1.17633	0.00655	1.11120	0.32954	0.01422	3.25251	0.00396	5.08199	0.00856	1.62028	1.36101	0.00702	3.16872	0.02793	0.00000	0.45675
**Hdgf**	30.44539	18.86174	31.28059	14.88024	19.25780	17.03599	20.78900	41.98847	22.43287	43.62192	38.24719	13.50433	34.27636	33.77428	29.95831	13.94821
**Hdgfrp2**	11.30326	6.84255	7.28685	5.92106	6.42840	1.01096	7.71245	16.90325	8.68413	14.78253	5.57376	0.90119	9.02641	0.02849	2.66207	8.09888
**Hdgfrp3**	1.37446	0.07501	0.09883	0.01838	0.00504	0.00747	0.34090	0.00524	0.01263	0.06156	0.01254	0.04492	0.01758	0.01108	0.00772	0.00603
**Hdhd2**	4.07555	0.29725	4.19348	0.39037	0.00932	0.05978	0.00364	0.71631	0.19277	1.22492	1.30544	0.03952	0.72747	0.01120	0.35513	1.91906
**Hdhd3**	2.10846	0.00598	0.00459	0.00483	0.01525	0.00581	0.00587	0.00978	0.44550	0.01329	0.00884	0.00617	0.01124	0.01353	0.00785	0.00515
**Hdlbp**	21.13835	16.83209	15.97203	11.29650	16.18290	11.88647	15.24648	29.22033	9.80384	29.39877	16.92012	11.97919	20.63840	8.03912	8.69580	9.44340
**Hdx**	0.01453	0.00772	0.02007	0.12335	0.00917	0.00612	0.00739	0.01101	0.26513	0.20614	0.00853	0.00528	0.01153	0.00672	0.62130	0.00710
**Heatr1**	6.95396	8.97216	9.97115	4.55809	4.10904	0.13515	3.36863	15.68947	12.35123	25.19909	6.75772	11.07088	4.67526	7.41372	11.20796	0.63062
**Heatr3**	1.71204	1.25793	4.07200	5.08194	0.00854	0.00346	0.02312	7.01933	8.75678	7.27030	4.33013	1.45228	0.86972	16.64014	11.71211	2.90373
**Heatr5a**	1.98650	8.50111	2.29656	0.81368	2.79827	0.01264	4.70095	6.84913	2.38296	0.46235	0.50250	0.16720	2.08939	0.09982	2.81918	0.21987
**Heatr5b**	1.81604	2.15424	2.01549	1.84135	0.00392	0.02086	2.97559	1.50460	0.21596	3.35075	1.03920	0.35351	0.00000	0.01879	2.68036	0.85009
**Heatr6**	2.13252	0.45477	0.81842	0.56098	0.01103	2.74022	0.08405	0.99834	0.48486	1.99282	2.21458	0.57553	1.44665	0.50862	1.21057	0.74439
**Hebp1**	0.98796	0.00617	0.00000	0.00216	0.01398	2.02969	0.00630	0.00000	0.01101	0.00000	0.43737	0.01419	0.00000	0.01266	0.19287	0.01990
**Hebp2**	0.01308	0.00802	0.01790	0.00782	0.00920	0.00643	0.00745	0.01121	0.01195	0.75600	0.01215	0.00584	0.01440	0.01163	0.00882	0.00753
**Heca**	0.01720	0.00898	0.07021	1.20485	0.01250	0.53522	0.04323	0.63297	0.00000	0.08464	0.00484	0.00568	0.00000	0.00412	0.96223	0.00740
**Hectd1**	5.74479	10.40962	7.92188	9.80581	2.76799	11.12684	5.76313	4.84680	11.91527	14.24525	5.61028	9.52864	4.70706	10.13273	16.09237	2.23377

**Hectd2**	0.02187	0.00788	0.01140	0.22801	0.00872	0.00662	0.00747	0.01415	0.00801	0.01396	0.01344	0.00801	0.01905	0.01504	0.02449	0.00730
**Hectd3**	0.93144	0.25851	4.09654	1.68942	4.40517	1.17987	0.25091	2.81218	1.05061	0.32814	0.80347	0.01285	3.50750	0.28784	1.92334	0.01733
**Hecw2**	0.00151	0.00788	0.00550	1.20383	0.01156	0.00593	1.13656	0.19877	0.18163	0.66780	0.00000	0.00000	0.04454	4.47364	3.62042	0.00691
**Heg1**	1.64431	0.09550	0.00206	0.00832	0.17903	0.00673	0.00603	0.00495	0.02578	0.00561	0.01102	0.01559	0.01105	0.01989	0.27745	0.00562
**Helb**	0.34608	1.65476	0.90383	2.31546	0.02821	0.03990	0.87311	1.21072	1.57309	1.39742	0.80513	0.12077	0.04154	0.21139	1.45115	0.87790
**Hells**	2.82845	5.45601	1.13071	6.50916	3.14223	2.25730	3.69442	2.44945	4.72043	0.82004	2.34615	11.99428	1.02603	14.30516	9.64796	0.59607
**Helq**	0.25372	0.27936	0.43784	5.54222	0.00721	0.00472	0.10924	1.33051	3.66614	0.00000	1.18730	0.13209	0.64869	3.12779	6.89465	0.00492
**Helz**	1.77051	1.96943	0.00370	0.37077	0.06778	0.01607	0.71291	0.01596	0.00571	5.36810	0.06375	0.03719	1.87498	0.00413	1.46370	0.80165
**Hemk1**	1.87507	0.00375	1.43162	0.01676	0.01858	0.01614	3.33106	0.00000	0.04513	0.58971	1.31477	0.06963	3.93931	0.00452	0.11452	0.00998
**Henmt1**	0.01381	0.00768	0.01173	0.47330	0.00939	0.00653	0.00742	0.01226	0.00383	0.00740	0.01197	0.01705	0.01277	0.01071	0.27189	0.00730
**Hepacam**	0.00367	0.00806	0.00641	0.00767	0.00797	0.06654	0.00799	0.10243	0.01185	0.71050	0.00945	0.05750	0.00469	0.22028	0.00000	1.01007
**Herc1**	2.72057	0.22469	1.66683	1.19319	1.01331	3.35770	3.91034	3.95046	0.90095	1.91557	0.72979	2.21418	2.32314	0.61421	1.25753	0.12171
**Herc2**	1.72419	1.49975	0.57486	12.89183	1.03690	0.01219	1.13930	2.77235	4.68521	2.96445	0.21629	0.30123	3.53401	1.63880	7.76583	0.11043
**Herc3**	2.56564	0.00519	0.00000	0.04076	0.00791	0.16567	0.00519	0.00365	0.01871	0.00393	0.01101	0.00532	0.24953	0.00683	0.28788	0.00448
**Herc4**	6.49454	4.27980	6.23466	6.12513	7.79814	5.88714	8.20228	5.39474	13.82003	8.70720	6.55694	8.29818	4.18993	17.16773	7.47406	3.46644
**Herpud1**	0.00000	2.96587	1.01025	0.00489	0.00483	0.01869	0.01587	1.84185	0.01074	0.68146	0.00000	0.00000	0.00000	0.00907	0.00000	3.03446
**Herpud2**	0.70821	1.03493	0.35297	15.03009	0.29179	2.27836	0.30442	1.52890	3.23566	3.09851	0.41133	0.63409	0.77974	2.16632	7.86519	0.00953
**Hes1**	0.91120	0.53943	0.39775	0.87953	0.00522	0.00783	0.02152	0.07490	0.01514	4.51968	11.20284	0.40203	0.00000	0.04351	0.46097	0.01102
**Hes6**	1.98890	0.00579	0.08739	1.42209	0.15204	0.00674	0.00541	0.13426	0.00000	1.66187	0.01172	0.01124	0.00817	0.01108	0.00000	1.00680
**Hesx1**	7.07610	1.81128	0.95785	1.03537	0.00000	0.00000	2.51474	0.00000	4.49951	0.00000	0.09142	0.75855	0.32202	0.77661	0.14261	0.00000
**Hexa**	15.49576	7.75657	9.63923	0.33156	13.08986	13.25942	10.33572	10.56112	0.50970	5.04475	4.11072	0.13482	16.19805	0.00000	1.56105	6.45345
**Hexb**	9.71150	0.92599	9.37321	5.95294	6.44543	4.68700	8.70968	10.20794	3.72428	15.56036	12.74511	6.41738	13.08306	11.83462	9.41750	5.02613
**Hexdc**	1.83021	0.00504	0.77833	0.13616	0.14565	0.00495	0.42696	1.59644	0.49480	0.40679	0.34676	0.00000	2.17639	0.04037	0.06780	0.03194
**Hexim1**	4.50032	0.03960	0.00000	6.56785	4.20613	1.78830	0.15877	4.52937	13.41124	5.79442	2.97256	0.31251	2.67754	0.16966	0.00000	2.74681
**Hexim2**	1.10657	0.00693	0.00000	1.65538	0.01666	1.29539	0.00525	0.55713	1.35273	1.51623	1.10244	0.03135	1.06698	0.00000	0.26874	0.00489
**Hey1**	0.00974	0.00834	0.00819	0.59136	0.01893	0.00679	0.00809	0.12485	0.72315	0.00550	0.01800	0.32344	0.00819	0.01289	0.00000	0.00725
**Heyl**	1.78244	0.00567	0.00355	0.24182	0.01276	0.00659	0.00615	0.00792	0.00750	0.04261	0.01301	0.00661	0.01147	0.01316	0.01255	0.00553
**Hfm1**	0.04729	0.01849	0.20092	0.49205	0.01733	0.00693	0.01808	0.08110	1.32182	0.19395	0.09686	0.02631	0.01362	0.24035	0.07994	0.00689
**Hgh1**	5.29552	4.68975	5.07240	1.71075	0.19225	0.00138	0.00766	3.66623	0.51694	2.10300	0.29364	1.71567	5.39241	0.08432	3.28849	4.03097
**Hgs**	4.63523	0.22944	0.58960	7.17175	9.30231	3.44800	0.45378	7.73682	8.04032	7.88685	6.70528	0.92156	10.99616	0.00000	0.00000	4.77100
**Hgsnat**	0.25299	0.00601	1.81482	1.21834	1.53077	0.00675	0.00543	3.58172	1.10189	0.33981	0.37762	0.11714	2.27444	0.01927	1.52771	0.01237
**Hhex**	3.63260	0.02665	0.67529	10.12257	0.01253	0.05575	0.00215	2.14241	1.77670	0.47211	1.69253	0.07176	3.29964	0.00000	0.88059	0.00000
**Hhipl2**	0.02102	0.00781	0.01036	0.09194	0.01080	0.00657	0.00713	0.00574	0.01003	1.72086	0.12472	0.01302	0.00970	0.03647	0.00000	0.00729
**Hibadh**	4.07296	0.00918	2.45652	2.50472	4.70703	1.59846	3.68188	2.02827	0.14384	0.54597	0.00000	0.29119	1.87498	1.14642	0.36524	2.99399
**Hibch**	7.91990	10.33682	6.04652	1.62354	7.71690	7.43181	3.58762	1.57017	1.67342	6.43707	0.38623	0.88404	1.66386	5.14164	4.98906	0.53893
**Hic1**	0.02444	0.00791	0.00912	0.56935	0.01199	0.00676	0.01692	0.00000	0.58592	0.68066	1.22986	0.00997	0.35294	0.00531	0.00000	0.00702
**Hic2**	5.54554	0.02911	7.79927	16.39969	3.04915	10.49017	3.51385	5.86959	20.15290	7.93842	4.46244	0.77821	7.04699	6.25683	10.07207	4.64922
**Hif1a**	2.40777	3.45427	0.92221	2.24522	2.99570	0.50452	0.56302	0.72864	0.00000	0.67054	0.02280	2.51871	0.68073	0.00352	0.84315	0.13788
**Hif1an**	4.20722	4.78600	1.49215	3.02222	4.92844	3.67620	1.73811	2.88121	3.08490	6.86312	5.10869	0.16460	0.00000	4.25869	4.30289	1.36668
**Hif3a**	0.00774	0.00687	0.00000	1.15019	1.15639	0.07726	0.00687	0.00600	1.80900	1.08204	0.01990	0.05484	0.56518	0.00264	0.11105	0.01377
**Higd1a**	4.57645	0.32828	4.75375	15.50675	5.97888	0.79161	0.43129	0.87408	6.03001	2.88473	4.87554	0.41269	5.12128	1.01746	1.52769	0.85799
**Higd2a**	6.85259	1.31971	4.41870	5.01528	2.34620	0.04999	1.93606	1.68811	0.95089	4.86254	6.09839	0.34389	7.46993	0.03020	3.92223	5.46779
**Hilpda**	1.62712	4.24262	3.09821	4.23852	0.00234	0.00458	0.10213	4.66438	3.04198	1.39994	1.94023	0.06216	7.62709	0.02131	1.72799	0.00000
**Hinfp**	0.62770	0.01308	2.72226	6.33547	0.00962	0.00762	5.85509	5.48028	15.02862	4.75641	4.84367	5.08121	3.91549	0.04916	8.48461	0.00000
**Hint1**	15.04328	0.52666	7.46663	5.38189	11.53100	7.56467	1.36973	3.76059	12.45280	9.81157	13.13847	1.11238	7.32907	0.23429	0.00000	7.16545
**Hint2**	0.54367	0.00666	0.62415	0.12401	1.22913	1.40832	0.02730	0.00000	0.00556	0.02755	0.46644	0.01424	2.10021	0.00877	0.00000	2.17878
**Hint3**	1.69334	0.36709	1.40859	0.08249	0.47340	2.81779	1.16090	0.23061	1.63755	2.50117	8.73945	0.05699	0.97949	1.61723	0.00000	3.08967
**Hip1**	11.11934	7.23024	12.39148	1.08298	0.55475	10.02457	6.63909	10.72243	0.52621	10.34936	10.76571	3.87400	8.94437	9.61639	3.45075	6.54913
**Hip1r**	0.00508	0.00745	0.00270	2.28012	0.01967	0.88800	0.00532	1.21020	1.61781	1.43464	0.00000	0.05042	3.07505	0.00760	0.00000	0.78219
**Hipk1**	7.48046	3.21344	2.55975	19.60393	3.71098	5.38059	8.35792	8.14210	26.69770	14.71240	5.76530	12.47878	2.31677	16.63907	10.18774	2.26638
**Hipk2**	3.03309	0.02064	0.67819	0.69927	2.97554	0.00590	2.75767	0.00000	0.38366	1.13115	0.02256	0.03185	1.53444	0.01004	0.36609	0.01807
**Hipk3**	4.27902	2.81861	7.19675	11.33005	0.07769	4.28122	7.82665	11.27185	26.47542	13.11747	10.80737	19.38835	2.29886	25.36131	8.73264	2.48678
**Hipk4**	0.01250	0.00760	0.00933	0.00546	0.00896	0.00666	0.00710	0.02290	0.01120	0.83307	0.33072	0.00464	0.00747	0.02065	0.00000	0.00703
**Hira**	0.84168	2.25925	3.44880	1.33438	3.20405	0.02071	3.06593	5.41852	1.22851	9.48659	4.90462	1.55429	0.00000	0.10435	9.23132	1.63324
**Hirip3**	12.47889	6.63304	12.97389	16.95762	5.89227	1.08045	4.35256	10.79170	4.12313	5.34000	5.99862	1.46379	9.15546	0.00000	3.18364	0.25782
**Hist1h1a**	0.02542	0.00815	0.01129	0.01417	0.01756	0.00663	0.00753	0.01393	0.23642	0.07929	0.01000	0.00754	0.01194	0.01161	0.01013	0.00713
**Hist1h1b**	0.77744	0.00683	0.00000	0.00979	0.00617	1.22995	0.00666	0.00753	0.01280	0.00286	0.00896	0.00645	0.00528	0.22136	0.00574	0.33873
**Hist1h1c**	0.68312	0.00726	0.00000	0.00865	0.01850	0.05019	0.00660	0.01214	0.01208	0.46205	0.01136	0.00628	0.46828	0.01148	0.01036	0.00651
**Hist1h1d**	0.02072	0.00702	0.00370	0.00941	1.98495	0.00801	0.00744	0.01065	0.01140	0.01265	0.01228	0.00622	0.02304	0.01252	0.01114	0.00727
**Hist1h2ab**	0.02720	0.00764	0.01894	0.00886	0.02681	0.00631	0.00735	0.01102	0.01211	0.41046	0.01324	0.01658	0.03936	0.01323	0.00804	0.06551
**Hist1h2ac**	0.01311	0.00801	0.01758	0.00998	0.00934	0.00635	0.00749	0.01314	0.01231	0.14375	0.01312	0.00755	0.04493	0.01204	0.00975	0.02223
**Hist1h2ad**	0.03814	0.00760	0.00930	0.00836	0.03480	0.01321	0.00736	0.01516	0.01201	0.42694	0.03008	0.01673	0.04531	0.01337	0.00763	0.04378

**Hist1h2ae**	0.01703	0.00829	0.05361	0.00993	0.01062	0.00596	0.00760	0.01305	0.01228	0.15575	0.01328	0.00801	0.01332	0.01345	0.00858	0.03680
**Hist1h2af**	0.01643	0.00794	0.01177	0.00887	0.02067	0.00655	0.00731	0.01090	0.01211	0.41035	0.01288	0.00687	0.02486	0.01336	0.00852	0.02926
**Hist1h2ag**	0.02326	0.00791	0.01140	0.01019	0.02958	0.00663	0.00746	0.01247	0.01230	0.09456	0.01343	0.00778	0.01219	0.01338	0.00955	0.00736
**Hist1h2ah**	0.01786	0.00785	0.01187	0.01035	0.00897	0.00661	0.00743	0.01282	0.01221	0.10760	0.01312	0.00755	0.01237	0.01492	0.01464	0.00740
**Hist1h2ai**	0.03197	0.00769	0.01667	0.00852	0.01750	0.00643	0.00743	0.01571	0.01212	0.49520	0.01322	0.00659	0.04981	0.01248	0.01227	0.03662
**Hist1h2al**	0.18708	0.00790	0.00000	0.21755	0.02191	0.10801	0.01765	0.09579	0.36341	0.15962	0.07574	0.00651	0.11120	0.04592	0.04596	0.08041
**Hist1h2an**	0.01458	0.00787	0.02059	0.01002	0.00929	0.00628	0.00741	0.01242	0.01225	0.13194	0.01360	0.00767	0.02858	0.01358	0.00937	0.00737
**Hist1h2bb**	0.01846	0.00824	0.01174	0.00482	0.01059	0.00668	0.00754	0.01371	0.33896	0.01349	0.01022	0.00766	0.01281	0.01327	0.00800	0.00745
**Hist1h2bc**	1.76221	0.00584	0.01470	0.00205	1.30179	0.01033	0.05659	0.00000	3.70581	1.34352	0.00000	0.00000	0.17720	0.01151	0.00650	0.08058
**Hist1h2be**	0.14466	0.00820	0.01112	0.00872	0.12551	0.01303	0.00747	0.01187	0.12533	0.10371	0.01159	0.00790	0.01918	0.01186	0.01027	0.02253
**Hist1h4c**	0.02574	0.00818	0.02336	0.01062	0.01154	0.01218	0.00737	0.02198	0.01235	0.01396	0.01292	0.00793	0.02432	0.01233	0.01027	0.21830
**Hist1h4d**	0.05773	0.01939	0.00936	0.01058	0.01109	0.00697	0.01795	0.03339	0.01231	0.01763	0.01358	0.00808	0.03470	0.01333	0.00969	0.03697
**Hist1h4f**	0.01719	0.00808	0.01004	0.01065	0.01055	0.01308	0.00739	0.04840	0.01232	0.01352	0.01268	0.00791	0.02477	0.01242	0.01000	0.02936
**Hist1h4h**	0.04307	0.00862	0.01332	0.01056	0.01393	0.02485	0.00758	0.03145	0.01239	0.02637	0.01231	0.00823	0.01080	0.01206	0.01035	0.01473
**Hist1h4i**	0.60540	0.00752	0.24248	0.11222	0.01043	0.03918	0.05799	0.35023	0.90908	0.00000	0.25417	0.01389	0.46945	0.01018	0.00000	0.44241
**Hist2h2bb**	0.41257	0.00827	0.00264	0.01034	0.01791	0.02084	0.00723	0.01210	0.01250	0.01418	0.01285	0.00770	0.01199	0.00932	0.03472	0.00720
**Hist2h2be**	0.00632	2.40563	0.00597	0.20610	0.00790	0.00679	0.00659	0.01011	0.03950	0.23786	0.01090	0.00798	0.01191	0.00694	0.00000	0.00638
**Hist3h2a**	0.18829	0.00794	0.00000	0.00948	0.03080	0.50162	0.00702	0.48320	0.02285	0.06689	0.00907	0.00666	0.00648	0.01516	0.00268	0.01459
**Hivep1**	2.06285	1.21662	0.76811	2.52936	0.61491	0.00492	0.03531	0.53772	2.64280	2.51094	2.64912	0.07349	1.34877	0.00000	0.06307	2.03774
**Hivep2**	0.37962	0.00676	0.00514	0.01038	0.00712	0.00654	0.00696	0.00868	0.01159	0.06577	0.00432	1.42766	0.01196	0.01036	0.00451	0.00668
**Hivep3**	0.01248	0.01799	0.03655	0.01047	0.00940	0.00604	0.00724	0.10947	0.01231	0.01985	0.01243	0.00753	0.00850	0.00968	0.02366	0.00736
**Hjurp**	1.05102	0.06553	0.04539	0.74142	2.09332	0.02964	0.01658	0.25711	2.92608	0.05947	0.00000	0.12322	0.01202	0.02841	0.71925	0.00564
**Hk1**	0.64918	0.01488	0.01409	0.75019	0.01428	0.01278	0.00000	5.37906	0.84444	5.54518	11.12189	0.35025	2.38614	0.06565	0.07285	0.00354
**Hk1os**	0.01381	0.00783	0.01115	0.28533	0.00940	0.00660	0.00735	0.01193	0.00711	0.09126	0.01325	0.07438	0.01275	0.01158	0.00555	0.00743
**Hk2**	2.83061	1.12652	7.71380	0.23050	1.28763	0.06370	2.46202	1.63976	0.17277	0.00000	0.00000	0.02237	0.97637	0.03547	0.15161	2.84438
**Hkdc1**	2.51673	0.02703	11.05545	0.23031	0.30687	0.00422	10.59754	5.96571	0.82066	2.77223	4.06573	1.39658	1.80381	0.08789	0.51283	0.01234
**Hlcs**	0.54054	3.75125	3.41269	0.81608	2.20974	0.00709	1.21859	1.14523	0.54564	2.57782	2.42403	0.05091	0.51716	0.75482	1.89861	0.00308
**Hlf**	0.40654	0.00621	0.01165	0.24183	0.00696	0.00601	1.29372	0.03562	0.00565	0.68285	0.20374	0.01416	0.00813	0.01554	0.09639	0.00689
**Hltf**	1.77674	3.58513	1.60057	0.35795	0.16410	0.54774	3.99824	1.91838	0.16928	0.00000	1.10657	1.25002	0.08963	0.05663	2.34071	0.00279
**Hmbox1**	0.00000	2.46260	0.06848	2.12170	0.00750	0.00459	0.00589	1.60787	3.54403	4.25795	0.46446	3.53719	0.33494	0.75789	3.72324	0.00510
**Hmbs**	9.25850	3.70695	7.26097	11.21328	8.28038	6.89612	3.32556	4.52483	14.52157	14.15364	7.92282	4.09662	13.33668	1.62716	6.26493	5.44128
**Hmces**	5.57837	0.00534	0.77079	11.56234	10.46279	3.81821	2.69534	1.10077	7.50188	3.91740	0.73190	0.17878	0.11592	0.00000	1.08248	1.99548
**Hmg20a**	1.92529	1.62687	2.54474	1.87125	5.28206	4.30798	0.04455	3.36713	0.00000	4.67794	0.85522	0.08931	2.72917	2.89192	2.19664	1.54177
**Hmg20b**	1.39338	0.04770	0.01233	0.07250	2.34506	0.40068	0.03429	1.52603	0.02926	7.13631	0.19825	0.06294	5.87078	0.01063	0.00000	5.66255
**Hmga2**	17.27296	16.21270	16.37351	0.34546	12.49503	5.29449	13.51384	23.44830	0.15207	9.16585	13.00374	18.70371	12.65915	31.09772	2.72103	9.36100
**Hmga2.ps1**	0.01365	0.00757	0.00818	0.35426	0.04110	0.01278	0.00718	0.26144	0.07554	0.08373	0.00813	0.10855	0.00670	0.02077	0.56891	0.00735
**Hmgb1**	21.97254	13.53041	24.10313	6.27584	18.70268	9.13661	15.61065	22.99650	21.54049	28.14530	14.17101	15.15578	17.27441	37.66324	19.78082	12.45721
**Hmgb1.rs17**	2.28881	1.14932	2.34011	0.45527	2.26929	0.70713	1.86929	2.26744	2.90860	2.84107	1.15093	0.15611	1.23429	4.43889	1.90302	1.02938
**Hmgb2**	13.61732	14.03265	7.99578	1.45771	8.91883	7.17737	6.09782	20.47599	3.76512	5.45956	6.68214	6.87805	9.81218	17.92972	16.66474	6.82254
**Hmgb3**	10.71749	8.91611	12.39247	12.59989	9.76079	0.71092	1.84178	15.36137	21.33593	15.10571	6.71057	2.89454	7.09354	19.98363	21.53091	2.09410
**Hmgcl**	8.47888	5.24359	12.12015	3.23653	4.40699	6.56903	10.50896	5.65289	5.42842	6.42355	3.32849	0.23618	4.90019	0.08381	3.93416	6.96643
**Hmgcll1**	0.01372	0.00779	0.01119	5.47581	0.00927	0.00601	0.00724	0.01074	0.80019	0.00701	0.04660	0.01525	0.01177	0.00000	0.36513	0.00628
**Hmgcr**	2.76717	5.23129	6.80951	0.82903	0.84661	0.79602	6.08787	1.08089	1.60407	0.55081	1.20660	0.08600	0.00000	3.00184	0.87908	0.54559
**Hmgcs1**	6.31423	5.31935	8.45417	9.60240	0.46401	5.17995	0.69587	3.27200	9.31813	9.73084	2.97204	5.53650	3.07430	6.18526	8.89751	2.73276
**Hmgcs2**	0.38091	0.00744	0.00523	0.00807	0.00700	0.00740	0.00725	0.01407	0.01140	0.71160	0.01035	0.00690	0.01190	0.01418	0.11232	0.00692
**Hmgn1**	23.50380	8.65301	13.80074	13.79960	11.34589	8.43078	8.29418	4.52675	8.38504	33.32367	24.63822	9.79334	25.55026	12.19381	6.37830	15.73475
**Hmgn2**	18.35379	6.64394	16.94190	17.85249	14.41175	7.94062	11.32939	23.19439	25.66858	30.05963	23.68728	7.07158	16.02628	14.14007	8.99758	11.24495
**Hmgn3**	0.01353	0.00807	0.01222	1.92426	0.00925	0.00636	0.00725	0.01100	0.38688	0.41444	0.01048	0.00599	0.01212	0.00895	0.00288	0.00724
**Hmgn5**	11.06274	11.94085	12.26545	5.92743	18.50853	7.26718	7.07196	1.89076	21.01743	0.96395	0.00314	5.01922	2.36886	5.38159	7.03427	1.62992
**Hmgxb3**	3.45910	2.31091	4.63036	8.96901	2.91364	0.01440	5.44776	5.90291	11.00583	7.03924	2.39730	0.23072	5.69820	0.17323	2.24526	3.92701
**Hmgxb4**	3.33605	5.25978	5.26857	4.70390	6.78225	0.03828	3.37631	4.54872	5.37119	3.86149	4.47700	0.83926	1.08871	13.02800	9.04511	1.40292
**Hmha1**	3.06184	0.01353	0.96457	0.05085	0.01683	0.01411	0.00296	0.00000	0.01167	0.57244	2.86792	0.00000	0.96167	0.00788	0.06080	0.27247
**Hmmr**	2.80723	3.70768	3.46332	10.26362	4.08906	2.54174	0.92826	4.89701	14.64799	4.03729	9.89294	10.31370	1.46636	23.65482	18.92274	1.61548
**Hmox1**	0.00794	0.00734	0.00622	0.34091	0.00919	0.00691	0.00652	1.13587	0.09013	0.00000	0.09793	0.11392	0.89930	0.00953	0.00000	0.00670
**Hmox2**	9.40436	4.23657	3.24441	0.63852	5.67095	4.54982	6.30992	4.53388	0.74784	6.99134	0.12161	7.06282	2.59121	0.62459	5.91142	4.58203
**Hn1**	15.87414	10.58357	11.92039	15.35085	1.64976	9.68117	6.84741	18.53585	22.27785	21.28669	13.18737	3.18182	14.96005	0.15245	2.08263	6.76417
**Hn1l**	12.09459	6.13765	6.45870	6.23530	9.13839	5.51524	2.49340	2.98179	5.57758	2.99051	1.06617	1.54333	1.50482	3.13245	6.54793	4.22112
**Hnf1a**	0.19442	0.00756	0.02042	1.08531	0.01631	0.00602	0.03696	0.01380	0.28826	0.17418	0.34357	0.04791	1.75829	0.00541	0.02642	0.00610
**Hnf1b**	0.00634	0.97852	0.01194	0.64954	0.00843	0.00748	0.00683	0.98788	0.00000	0.01274	0.00598	0.00577	0.00537	0.00485	0.00000	0.01402
**Hnf4a**	6.68794	3.46321	3.70935	0.00682	10.50255	0.00000	0.74223	0.00000	0.00854	0.00000	0.01816	0.01103	0.01486	0.01222	0.00000	0.01180
**Hnf4aos**	0.01237	0.00799	0.01981	0.70486	0.00910	0.00599	0.00710	0.01215	0.29537	0.00368	0.01033	0.00747	0.48615	0.03140	0.00165	0.00682
**Hnmt**	0.01363	0.00766	0.01115	2.38864	0.00931	0.00626	0.00733	0.01131	1.38023	0.00279	0.01417	0.01445	0.01266	0.00715	0.35002	0.00685

**Hnrnpa0**	15.12692	12.15198	7.65664	8.22095	7.82737	8.21751	10.16372	2.71016	9.66955	13.91194	9.96986	6.63980	12.04205	25.94400	11.23129	9.75773
**Hnrnpa1**	0.53454	0.73816	1.48809	0.38001	0.86970	0.01361	0.61099	1.25088	0.22403	0.93176	2.10420	0.00000	0.74275	6.30947	1.20733	0.66433
**Hnrnpa2b1**	16.85378	14.14499	17.28732	10.33642	11.75883	11.98700	5.82919	24.64239	12.66962	10.54407	13.70760	13.99845	13.34777	24.51457	13.18039	12.53303
**Hnrnpa3**	18.07159	14.76658	19.64484	13.58500	13.76690	11.67811	9.16380	11.36887	10.02870	20.71751	19.03821	21.14965	13.91291	31.34764	16.14382	10.29074
**Hnrnpab**	31.76484	19.67667	25.15915	27.19944	23.29654	12.50219	17.52642	29.81239	34.50634	45.46617	36.55526	23.71565	24.62454	30.91638	28.22571	12.23153
**Hnrnpc**	34.22168	23.20034	32.41307	15.51894	23.35530	12.37867	19.51418	38.09457	23.38967	42.96462	30.51351	17.35849	31.04891	44.05131	35.38794	19.72748
**Hnrnpd**	23.67529	14.59755	12.30803	7.74864	17.36312	12.76045	13.00593	15.36542	11.98930	19.75660	17.28739	11.89524	16.57044	21.17137	14.67708	10.53464
**Hnrnpdl**	18.23278	12.36662	8.17077	18.24873	13.01659	6.02105	7.71951	11.29410	22.51671	26.44289	17.18669	16.96563	18.76050	17.86464	18.07363	15.01943
**Hnrnpf**	38.06716	23.74673	35.41632	22.11878	26.01931	16.57218	20.55126	40.13729	27.36288	41.08345	26.86345	21.53256	32.81596	15.64413	31.49733	16.13325
**Hnrnph1**	12.84109	12.97310	15.32748	6.06056	11.58056	9.78701	10.40880	21.83695	5.49195	30.48582	13.27927	17.04535	18.70663	29.81771	24.37454	9.75790
**Hnrnph2**	7.95127	11.02708	7.51113	4.53620	2.46623	3.12510	7.15758	10.15927	2.23332	7.65710	4.05794	0.74084	6.01984	26.29819	14.69806	2.00144
**Hnrnph3**	0.07737	0.00773	0.14966	0.12608	0.01626	0.00641	0.00704	0.09705	0.39491	0.18250	0.02121	0.00513	0.58267	0.10500	0.30425	0.85917
**Hnrnpk**	28.36681	24.74617	25.96798	17.58046	20.51426	13.52352	18.17608	32.75590	18.37923	28.67791	22.10088	7.51013	26.19020	24.59128	24.69597	13.22232
**Hnrnpl**	0.83545	1.08893	1.12974	2.03644	0.38130	0.04546	0.19993	0.00000	5.04845	0.00000	7.01991	0.09509	1.76845	11.20466	3.78621	2.08861
**Hnrnpll**	3.51800	9.88656	4.65670	4.86908	8.65378	3.71159	6.78163	3.30153	14.35204	12.34547	6.79687	18.75210	5.20807	16.81637	15.59175	1.86599
**Hnrnpm**	23.85765	4.22209	5.28571	12.89781	12.84075	10.19340	5.12717	23.97647	12.65088	26.98135	20.24650	4.52798	19.88436	1.09851	5.98901	14.61812
**Hnrnpr**	13.69808	11.33533	5.25021	11.21616	5.64499	7.56156	7.93681	10.42797	12.20431	15.83793	4.12586	14.77811	5.34119	4.31217	11.71168	4.70384
**Hnrnpu**	23.88685	23.70133	23.40038	16.03215	18.91093	7.41557	13.20277	25.86394	25.76246	28.37593	24.20604	8.22090	23.82907	33.73645	27.29163	12.44144
**Hnrnpul1**	1.30494	0.11104	0.91518	1.58350	0.11875	0.87746	0.90402	1.75024	1.20718	0.28777	0.00000	0.12786	2.79464	0.20511	0.94436	0.80379
**Hnrnpul2**	4.52713	3.36679	3.42660	14.01786	0.10632	3.16932	5.21502	2.75883	13.89575	5.35397	6.12730	6.09628	3.74381	15.25820	10.87762	1.18253
**Hoga1**	0.40460	0.00772	0.00324	0.08938	0.01738	0.00766	0.00666	0.22321	0.01064	0.00765	0.01205	0.00665	0.26341	0.01476	0.02644	0.00698
**Homer1**	0.98111	0.00652	0.00000	4.09509	0.03044	1.14201	0.00589	0.05885	0.05334	0.78466	0.67043	0.02125	0.00000	0.00000	3.34755	0.00537
**Homer2**	4.11663	2.57877	0.00000	4.63263	2.66492	3.94955	2.36085	1.90138	4.19878	1.97014	0.90131	0.06936	0.46182	1.53281	0.46213	2.01136
**Homer3**	0.01264	0.00813	0.18397	0.00566	0.00907	0.00600	0.01835	0.01179	0.01117	0.10995	0.93666	0.01743	0.03149	0.01146	0.00000	0.00722
**Homez**	0.35063	0.00759	0.42330	0.51344	0.01344	0.00538	0.03663	0.00769	0.70022	0.69154	0.00568	0.02120	0.00917	0.00403	0.45723	0.00673
**Hook1**	0.01182	0.00775	0.00694	0.78794	0.00998	0.00647	0.00745	0.00954	0.64789	0.41108	0.05695	0.00000	0.04393	0.29638	4.99861	0.00765
**Hook2**	9.12381	0.05860	1.51147	1.84680	4.04663	0.00000	4.05847	0.87409	1.02873	3.18312	4.46274	4.42050	8.31371	0.00000	0.00000	3.05180
**Hook3**	0.43191	0.00460	0.07400	0.61422	0.02175	0.00607	2.21299	0.00000	0.05160	0.46732	0.39474	4.68349	0.00979	2.03057	0.99856	0.00647
**Hopx**	0.01364	0.00798	0.01166	0.14046	0.00928	0.00659	0.00751	0.01336	0.98709	0.00955	0.00710	0.02674	0.01296	0.01044	0.01531	0.00733
**Hormad1**	0.00000	3.97535	5.64711	5.49246	0.00655	0.00246	5.44645	0.96490	7.85923	5.44090	1.69785	17.09658	0.43219	8.81335	6.89970	0.00000
**Hormad2**	1.15868	0.00626	0.72842	0.00845	0.00525	0.00567	0.00640	0.00653	0.01103	0.00000	0.00778	0.01306	0.27225	0.63244	0.41125	0.01344
**Hotairm1**	0.01370	0.00808	0.01262	0.16392	0.00943	0.00666	0.00754	0.01361	0.03826	0.05935	0.01391	0.00820	0.01486	0.01419	0.00650	0.00726
**Hoxa1**	0.00969	0.00766	0.00770	0.00444	0.00939	0.00693	0.00708	0.78247	0.26194	0.35922	0.00548	0.01399	0.00580	0.00665	0.08853	0.00779
**Hoxa10**	0.00896	0.00779	0.00759	0.32102	0.00968	0.00702	0.00740	1.15542	0.00529	0.01087	0.00560	0.00610	0.00544	0.01190	0.00000	0.00733
**Hoxa7**	0.01368	0.00798	0.01196	0.75429	0.00918	0.00665	0.00737	0.01177	0.00000	0.26210	0.01294	0.01729	0.01263	0.01080	0.00610	0.00760
**Hoxb13**	1.77231	0.00612	0.71667	0.14744	0.00468	0.00753	0.00603	0.00377	0.00939	0.00510	0.15062	0.00630	0.02683	0.00688	0.16568	0.01245
**Hoxb7**	1.21533	0.00719	0.00371	0.00643	0.03271	0.00717	0.00630	0.00573	0.01145	1.53904	0.01079	0.00343	0.01136	0.01192	0.00884	0.00654
**Hoxb9**	0.01360	0.00791	0.01215	0.02839	0.00913	0.00651	0.00746	0.01117	0.02526	0.25400	0.03535	0.00655	0.01102	0.00588	0.13668	0.01418
**Hoxc13**	0.06394	0.00794	0.00614	0.01044	0.01392	0.00718	0.47423	0.00999	0.01234	0.01351	0.01312	0.00742	0.01134	0.01212	0.01048	0.00746
**Hoxd11**	0.01341	0.00793	0.01671	0.02644	0.00939	0.01253	0.00756	0.01294	0.06882	0.03151	0.01083	0.01694	0.02144	0.02421	0.28065	0.00725
**Hoxd8**	0.01373	0.00785	0.01208	0.14927	0.00929	0.00649	0.00748	0.01222	0.00965	0.04669	0.01145	0.00672	0.01218	0.00578	0.40605	0.00719
**Hoxd9**	0.00985	0.00858	0.00886	0.01053	0.00959	0.00689	0.00823	0.30839	0.01220	0.02083	0.01031	0.00819	0.00636	0.01076	0.02469	0.00737
**Hp**	0.00688	0.00743	0.00788	1.34492	0.00898	0.00639	0.00642	0.67881	2.59249	0.58832	0.00000	0.00000	1.14268	0.01311	0.00000	0.00631
**Hp1bp3**	1.97829	0.10472	1.24932	1.79665	3.69355	3.27295	2.42234	1.60958	2.93612	4.88463	2.17303	3.71877	3.16588	3.61388	1.44412	3.37289
**Hpcal1**	0.11842	0.19621	1.34451	5.42767	0.01623	0.19337	0.16736	0.04265	2.97622	0.91986	0.05897	0.02815	0.65008	0.02005	0.04374	4.52334
**Hpd**	0.01331	0.00789	0.01171	0.71227	0.00906	0.00632	0.00738	0.01074	0.97905	0.52115	0.00397	0.00374	0.01107	0.02555	0.27500	0.00694
**Hpdl**	0.07260	2.52530	1.82634	2.79967	2.44205	7.41255	5.03422	11.89876	7.28045	12.94371	6.55976	0.70897	8.16072	0.03278	2.54309	1.27239
**Hpf1**	8.89665	5.64147	8.52925	19.15222	6.81186	1.22155	4.27829	8.07006	10.50689	10.32481	12.82295	7.13013	8.65892	6.92405	2.15916	7.71939
**Hpgds**	0.71112	0.00706	0.14806	0.26306	0.02856	0.00609	0.05655	0.01172	0.36642	0.67192	1.49837	0.03263	0.11784	0.74001	0.35806	0.04458
**Hprt**	22.09427	20.01838	35.86885	16.66580	17.33062	20.31810	18.24539	41.61418	20.06227	40.95460	32.64043	19.62429	32.13485	33.88437	21.25586	16.60624
**Hps1**	1.41560	0.00707	0.00325	0.31846	0.01449	0.00784	0.00595	0.28700	0.28416	0.60849	0.01176	0.00366	0.80183	0.01075	0.00052	0.00647
**Hps3**	1.72578	0.00488	1.43506	0.47396	0.02375	0.00502	2.10001	0.93843	0.17399	0.84848	1.31162	0.52172	1.34544	0.38689	1.61551	0.74032
**Hps4**	2.43169	0.22998	1.46039	0.61390	0.00000	1.90090	0.63711	3.61683	0.14219	2.53772	0.62216	0.30806	2.14705	0.00298	0.00000	5.25191
**Hps5**	3.94480	5.40308	1.87550	2.03129	0.15356	0.00150	4.21434	0.82231	5.99425	4.46377	2.54623	0.47168	0.00000	3.76884	3.38424	0.00000
**Hps6**	1.15828	0.10761	1.04966	3.00107	0.01090	0.00392	0.80445	1.06429	2.65827	3.93043	0.49409	0.08487	0.24982	0.30051	0.36487	0.01259
**Hpse**	0.00000	0.00499	0.00000	1.91303	1.55128	0.00747	3.48337	0.11623	0.79486	0.00000	0.47569	0.02390	1.12232	0.00802	0.44072	0.00606
**Hr**	0.01211	0.00767	0.05939	0.18530	0.00917	0.00845	0.30293	0.01079	0.00855	0.01403	0.01355	0.00741	0.01115	0.01066	0.01120	0.00733
**Hras**	6.54069	3.40711	1.64397	8.51806	5.74378	4.15596	4.75100	6.02849	12.83588	6.07902	9.03862	2.08142	5.70095	0.16371	2.14642	0.45563
**Hs1bp3**	5.41298	1.53350	4.55229	0.00000	0.90293	0.12125	2.37899	5.32519	0.00572	2.10627	1.82934	0.10793	7.01707	0.00000	1.87328	0.71054
**Hs2st1**	4.61948	3.12453	2.63543	7.06126	1.78919	0.90933	5.36473	4.18163	2.98238	3.26151	2.16870	1.49783	2.77654	4.05261	4.80593	4.16215
**Hs3st1**	4.78980	0.04509	0.00359	0.27596	0.01504	0.00392	0.00327	0.25606	0.00705	0.96091	0.01079	0.01044	0.00673	0.01029	0.00120	0.00275
**Hs3st3b1**	0.17617	0.42838	0.67340	0.43992	0.04814	2.23896	0.00630	0.08798	0.00554	0.00000	0.37924	0.00000	1.34413	1.13628	0.00000	1.98649

**Hs3st5**	0.01847	0.00833	0.01184	0.25215	0.01057	0.00665	0.00753	0.01380	0.23866	0.01407	0.01121	0.00793	0.01276	0.01177	0.00535	0.00741
**Hs3st6**	0.01276	0.00789	0.00847	0.61613	0.00923	0.00663	0.00705	0.01934	0.84470	0.35664	0.47692	0.00309	0.00741	0.00524	0.02946	0.00720
**Hs6st1**	3.14276	0.58646	4.23051	6.03756	0.01045	0.00283	1.48596	6.01785	7.40144	11.22408	6.20983	0.59677	2.34290	4.30675	2.77379	2.75676
**Hs6st2**	0.03200	0.00778	0.01568	2.54644	0.01776	0.00601	0.02737	0.01209	0.42117	0.00172	0.12350	0.02515	0.01239	0.08002	0.62714	0.01426
**Hsbp1**	11.38374	7.66584	10.91772	7.91776	6.49178	5.35050	6.16015	5.87884	0.00005	4.85443	11.50345	0.99455	8.19402	4.40567	2.25062	6.97125
**Hsbp1l1**	0.40497	0.33601	0.11986	0.27595	0.01650	0.04536	0.90863	0.20637	0.39448	1.49186	0.07720	0.05857	0.04216	0.01317	0.57596	0.01385
**Hscb**	5.03446	0.78825	3.24766	1.80619	5.40340	2.39762	1.39398	0.74041	2.06739	0.23763	1.25003	0.01788	0.63649	0.81908	0.04080	0.00624
**Hsd11b2**	0.19657	0.00735	0.03795	0.01057	0.00709	0.00830	0.00708	0.01518	0.01231	0.02420	0.01261	0.00742	0.26391	0.01199	0.01063	0.49032
**Hsd17b1**	0.07021	0.06522	0.00267	0.00595	0.00770	0.00801	0.00682	1.80008	0.02410	0.00000	0.24692	0.02069	0.22167	0.01221	0.00000	0.00704
**Hsd17b10**	5.11340	4.52768	9.42140	0.35912	2.28057	2.65855	4.58402	2.42200	1.09623	3.33662	1.34321	0.25338	2.62449	1.15761	0.60205	4.86532
**Hsd17b11**	0.33249	0.00716	0.60305	0.00470	0.00631	0.00542	0.00617	0.00000	0.35926	0.35094	1.41781	0.00722	0.98470	0.28113	0.00000	0.00647
**Hsd17b12**	7.09061	5.07484	11.41476	2.68332	6.43456	6.00980	3.08436	7.03988	6.41246	3.06114	3.58993	0.49841	6.46154	3.99253	1.87482	2.60346
**Hsd17b13**	0.02094	0.00770	0.01398	10.21572	0.00869	0.01693	0.00686	0.01324	1.50969	0.69241	0.04761	0.07996	0.02415	1.90077	1.20870	0.01221
**Hsd17b14**	0.00000	0.01295	3.61558	6.10466	0.01979	0.09849	1.14071	9.74647	13.49559	21.03357	15.36105	5.95542	3.35478	0.41196	1.14256	0.01424
**Hsd17b2**	0.03880	0.00761	0.00773	0.00452	0.00824	0.00703	0.00755	0.01747	0.97748	0.23887	0.00000	0.01669	0.01244	0.23888	0.11841	0.00724
**Hsd17b4**	9.42740	6.78527	6.51908	0.28785	4.01217	0.00001	7.73915	8.69549	0.82276	2.38943	6.48125	0.13945	4.51697	3.26873	0.52672	1.00495
**Hsd17b6**	1.10803	0.00648	0.00000	1.13231	0.01395	12.80281	0.00581	0.00000	1.60639	0.85658	0.66307	0.20991	0.00000	0.47055	0.86767	0.00000
**Hsd17b7**	2.64596	0.01574	2.06676	0.54558	0.00000	0.01475	0.00497	1.20981	0.85082	1.10419	0.48444	0.07195	0.63398	4.14568	0.00000	0.01874
**Hsd3b1**	0.01157	0.00778	0.01216	0.00463	0.00843	0.00657	0.00728	0.01006	0.11187	5.00515	0.00820	0.00000	0.01068	0.01269	0.00608	0.00754
**Hsd3b7**	0.30334	0.03924	0.07197	1.63842	0.00708	2.92296	0.00677	0.00000	0.02382	1.72249	0.86210	0.00000	0.07273	0.00509	0.00000	1.58057
**Hsdl1**	0.59809	5.19146	0.46994	1.89907	2.80591	2.75620	0.00464	2.08698	0.00000	0.00000	0.30920	0.06234	0.00000	0.95493	2.60491	0.45723
**Hsdl2**	3.06702	1.30054	1.59192	2.24696	0.06763	0.93472	2.04226	1.54743	3.28596	3.14212	1.96045	6.27125	1.49393	0.00000	0.00000	1.50336
**Hsf1**	4.50031	0.06283	1.16262	16.09980	0.18684	0.00000	2.00324	2.26096	13.69355	2.62427	2.60246	0.16002	2.26493	0.51899	2.40300	2.03810
**Hsf2**	3.32421	3.21826	0.44217	0.71411	0.03079	3.28273	0.47363	0.26348	0.02442	0.00000	3.80495	0.15259	0.23714	0.14403	0.00000	0.00783
**Hsf2bp**	5.75247	1.01743	3.43209	5.44348	4.19896	0.01049	4.44708	2.25662	1.89851	3.90681	0.00000	0.09371	1.39596	0.33670	0.60358	2.35367
**Hsf5**	0.01717	0.00800	0.03984	0.47763	0.01155	0.00855	0.00763	0.01424	0.00275	0.00687	0.02572	0.00726	0.07401	0.01893	0.13079	0.00765
**Hsh2d**	0.01528	0.00816	0.00434	0.00434	0.01028	0.00747	0.00700	0.03388	0.01051	1.19984	0.72607	0.18863	0.00554	0.15244	0.08491	0.00732
**Hsp90aa1**	53.12343	40.47319	57.51495	17.30512	42.73428	26.82772	29.13402	61.60745	31.13670	49.45409	50.46280	40.39495	52.03360	54.45097	31.79987	24.21778
**Hsp90ab1**	56.45178	37.24382	60.12696	17.77811	41.64058	24.87951	34.67133	67.94667	30.20247	59.72398	56.03662	27.97231	55.28660	52.94024	39.66574	29.58575
**Hsp90b1**	16.21606	12.92870	29.44046	8.95140	13.57285	18.67018	13.52846	7.08350	3.23752	5.92144	4.17614	12.04818	19.31055	3.07796	12.17332	12.97207
**Hspa12a**	0.19169	0.00712	0.00224	0.90132	0.00749	0.00731	0.00698	0.51605	0.17810	0.09444	0.03427	0.01382	0.00583	0.00338	0.60732	0.00702
**Hspa12b**	0.01372	0.00812	0.01219	0.07947	0.00932	0.00665	0.00754	0.01396	0.20479	0.01424	0.01109	0.00791	0.01277	0.01239	0.00732	0.00740
**Hspa13**	0.00241	0.00840	0.64076	0.34072	0.00728	0.00624	0.00823	0.33480	0.00409	0.01731	0.00908	0.01956	0.00000	0.01226	0.00000	0.74779
**Hspa14**	12.59827	9.91561	8.54076	7.86791	7.88585	6.83070	6.36196	11.52305	17.54911	9.37446	10.36668	3.63837	9.18550	6.47978	9.10295	6.35429
**Hspa1a**	0.11839	0.00801	0.07920	4.66758	0.00739	0.00481	0.00740	0.71588	9.61889	0.18523	0.24852	0.01827	0.00913	0.46463	0.19458	0.00555
**Hspa1b**	0.02716	0.04025	0.25396	1.81393	0.00769	0.00564	0.00635	0.20851	17.06998	0.00000	3.46043	0.00000	0.00000	0.03532	0.00000	0.00515
**Hspa1l**	0.01388	0.00815	0.01037	0.21497	0.00930	0.02540	0.00753	0.01390	0.25234	0.01390	0.01175	0.00791	0.01241	0.01188	0.00552	0.00735
**Hspa2**	2.10836	0.00445	0.01906	2.99505	0.00459	0.00637	1.81890	1.17014	0.08551	2.17416	0.95377	0.04208	0.55520	0.21225	1.30315	0.01142
**Hspa4**	18.85741	14.10759	12.42832	8.98625	14.13827	7.98797	9.73698	16.53514	12.12426	18.05372	6.97627	17.02328	8.77075	14.01618	15.06763	7.51590
**Hspa4l**	0.02315	0.00812	0.00456	3.67240	0.01113	2.68182	0.00707	0.00996	0.39329	0.56372	0.00498	0.04089	0.00336	0.00045	4.16541	0.00563
**Hspa5**	15.67733	12.37543	33.57085	19.05873	8.37425	12.77392	13.64232	15.87177	20.25418	7.45541	7.71685	4.05362	30.33644	17.63346	22.47952	13.65133
**Hspa8**	56.46366	33.02301	65.28548	16.36821	43.45648	27.73293	33.96195	71.96007	32.57189	67.82710	63.83474	34.31835	67.29732	33.68902	28.79418	32.96211
**Hspa9**	24.48572	18.47567	15.96361	17.06487	18.51408	13.08208	13.12449	21.20535	23.29314	19.81562	22.00688	16.48925	21.61524	29.05950	21.03982	12.02609
**Hspb1**	0.88341	0.00675	0.29224	22.00428	0.01781	0.02491	0.00303	0.00000	15.08547	5.01183	7.40432	0.57673	0.00000	0.00000	0.24353	0.39699
**Hspb11**	0.57128	0.31556	0.07003	0.23247	0.01512	0.55167	0.33706	1.02366	0.27315	0.45205	3.07987	0.09467	0.41636	0.70189	0.35604	0.09925
**Hspb2**	0.01239	0.00812	0.09611	0.01063	0.01043	0.00877	0.00681	0.01040	0.01240	0.00995	0.01244	0.00719	1.08112	0.01280	0.00893	0.00681
**Hspb6**	0.54010	1.96430	1.23426	0.39388	0.01537	0.00488	0.00620	0.01135	0.66207	0.00498	0.00781	0.01639	0.00735	0.00765	0.00000	0.00568
**Hspb7**	0.01901	0.00793	0.03311	0.02634	0.00872	0.00717	0.01784	0.03316	0.03056	0.04879	0.02323	0.00847	0.01757	0.01063	0.03243	0.00736
**Hspb8**	1.35760	5.75803	4.72949	0.00000	0.00551	0.00399	0.00380	0.94455	0.01166	1.12617	0.00000	0.03932	0.00000	0.00870	0.00000	0.02389
**Hspb9**	0.01270	0.00826	0.07256	0.00863	0.00902	0.00578	0.00758	0.01166	0.01221	0.49159	0.01119	0.00710	0.01041	0.01185	0.00825	0.00709
**Hspbap1**	1.89201	0.98531	0.82293	2.11835	2.10031	2.72186	2.18500	1.56795	2.70717	0.38808	1.46040	4.84253	0.59202	0.04359	1.23818	0.61112
**Hspbp1**	9.28170	0.08627	2.13257	1.79038	5.43069	4.13105	4.95538	19.56144	3.49594	15.09510	8.47464	0.98230	15.41124	0.00000	0.09475	6.06719
**Hspd1**	27.48433	18.87697	30.09299	7.06426	20.48956	19.89142	19.28290	27.51602	5.28338	20.45869	15.52383	6.41314	21.59637	15.89280	18.42721	17.10554
**Hspe1**	22.82783	2.50281	18.11948	9.89642	19.33180	6.62170	3.81820	4.71623	5.70977	10.27012	17.11633	1.55264	12.20264	1.38933	2.03822	8.66587
**Hspg2**	0.01585	0.00749	0.00197	0.41948	0.00776	0.99702	0.00709	0.00699	0.02418	0.06016	0.11533	0.23854	0.00481	0.00952	0.00000	1.13027
**Hsph1**	28.49946	21.44082	29.77312	11.90986	19.46378	9.44332	14.20921	13.25883	22.58925	8.72910	6.16756	8.23638	22.27970	17.49114	11.26501	14.78339
**Htatip2**	5.01268	3.25422	7.72831	0.33089	4.31486	1.28695	2.89812	2.39670	0.00000	0.00000	2.83188	0.08170	8.91340	0.32049	0.12712	4.75494
**Htatsf1**	3.65466	3.02042	3.09883	6.49892	2.95243	0.00154	2.74162	4.63728	3.75410	11.06586	6.32000	2.93201	2.36642	15.33657	13.79239	2.80836
**Htr5b**	0.00000	0.03996	0.00457	0.00330	0.00799	0.00698	1.34515	0.01661	0.00966	0.60986	0.24845	0.09464	0.07004	0.02611	1.15121	2.00858
**Htr7**	0.01375	0.00806	0.01226	0.07866	0.00931	0.00664	0.00753	0.01390	0.01133	0.01537	0.01385	0.00813	0.01267	0.01188	0.00929	0.00740
**Htra1**	0.56644	0.00615	3.46707	1.60183	0.17697	0.16711	2.06417	0.23208	2.42373	2.33775	0.51660	0.13935	1.51490	0.39730	1.49280	3.26757
**Htra2**	2.04871	2.71972	2.14910	0.00000	2.06708	5.23301	3.42621	4.69868	0.00533	1.90751	0.00000	0.23949	2.85676	0.41460	0.56191	4.03448

**Htra3**	0.01362	0.00784	0.01232	0.00919	0.00932	0.00653	0.00734	0.01140	0.01225	0.33062	0.01283	0.00665	0.01213	0.01159	0.00880	0.00750
**Htt**	2.51729	3.96182	0.92670	1.24564	1.37488	4.46808	0.00254	2.65732	1.46500	0.90431	0.74440	0.08790	1.89675	0.09890	0.11317	2.54514
**Hunk**	0.57282	0.00749	0.00512	0.29289	0.00666	0.00707	0.00703	0.01326	0.00698	0.10886	0.01277	0.00777	0.01154	0.01106	0.00675	0.01352
**Hus1**	1.34289	0.00569	0.81703	3.01215	0.11788	2.16705	1.04736	2.96429	1.26676	1.14237	1.16823	0.26132	1.69492	5.88372	3.57103	0.02571
**Hus1b**	0.13207	0.00852	0.00745	0.01062	0.01619	0.00673	0.00673	0.01059	0.01235	0.00823	0.01236	0.00775	1.12434	0.01287	0.01084	0.00677
**Huwe1**	14.13622	11.75275	11.43454	13.13934	7.41270	8.02297	8.01787	23.32590	14.52727	17.24349	15.07132	18.12854	14.69994	30.25027	11.48504	2.76959
**Hvcn1**	1.60422	0.00625	2.01655	6.00882	0.01826	0.34251	0.00521	1.38842	3.58115	2.79210	1.61563	0.07099	0.44934	0.00000	0.50898	0.64395
**Hyal1**	0.73408	0.00571	0.17585	0.09826	0.08056	0.00519	1.93385	0.08184	0.32549	0.04344	0.21036	0.01270	0.03257	0.22190	0.08284	0.02721
**Hyal2**	2.69137	0.00363	1.48314	0.52648	2.53456	0.13133	0.35007	0.80993	0.00000	0.31532	0.25549	0.00126	0.10585	0.00812	0.00000	1.10357
**Hyal3**	0.24864	0.00714	0.61634	0.59010	0.14642	0.04387	0.12976	0.06975	0.94252	0.31802	2.32552	0.11753	0.42514	0.66407	0.34068	0.08600
**Hydin**	0.14234	0.00641	0.00000	0.13557	0.00760	0.00817	1.89480	0.00925	0.00639	0.00333	0.01057	0.00537	0.01648	0.06835	0.35241	0.01425
**Hyi**	0.08438	0.00780	0.62479	0.00942	0.00693	0.74343	0.00732	0.01258	0.01235	0.19794	0.01096	0.00711	0.00783	0.01389	0.00183	0.00683
**Hykk**	1.51246	0.66161	1.18514	0.39481	0.02814	0.00943	0.02552	0.69570	1.07300	0.77808	0.44012	0.04917	0.07862	0.28203	0.07086	0.01851
**Hyls1**	0.76309	0.33094	0.00769	1.10203	0.01560	0.00741	0.00701	0.06932	2.16053	0.12854	0.00000	0.02081	0.01292	0.03767	0.61915	0.00621
**Hyou1**	1.76026	4.30434	3.30865	0.90581	0.00595	0.09318	0.70581	1.28334	1.80871	0.85120	1.06101	0.07815	4.07948	4.30506	1.95073	4.60854
**Hypk**	11.04384	1.59357	10.35191	17.21107	7.22665	2.84412	3.01148	2.03850	16.74200	7.98480	18.65887	1.28436	5.87401	4.64310	8.74204	9.78133
**Iah1**	2.77661	2.63295	2.71713	1.96848	0.02523	0.30658	1.14856	2.28166	1.19370	0.32994	1.00407	0.13928	1.58632	0.06370	1.05626	0.05484
**Iars**	13.87844	11.93073	10.83692	13.75292	18.69875	6.43021	7.15044	14.38793	15.99064	6.54387	6.94659	15.65195	6.61445	7.14529	8.86713	7.34177
**Iars2**	4.84007	4.40734	0.84147	0.58678	5.50590	3.71625	2.56099	4.33562	0.26883	0.00000	1.84324	0.04894	0.18075	0.01409	1.55018	0.09637
**Iba57**	0.00000	0.00714	0.00032	0.06521	0.01637	0.03166	0.01633	0.96543	0.04285	0.31865	1.30937	0.00606	0.00000	0.01087	0.28579	2.06163
**Ibtk**	6.76166	0.77414	1.80378	9.68845	2.79323	4.44800	0.73686	8.55970	4.35300	7.15760	4.66670	12.82032	2.96880	15.05033	13.93391	4.75383
**Ica1**	0.01674	0.00764	0.00568	5.20001	0.00900	0.00568	0.00699	0.01270	2.44230	0.18160	0.01706	0.07710	0.01272	0.23234	2.63850	0.00665
**Ica1l**	0.74208	0.00664	0.00000	1.32197	0.00560	0.00559	0.00630	0.45950	1.01539	2.06283	0.12663	0.01057	0.03517	0.45144	2.15813	2.11062
**Icam1**	0.00000	0.00505	0.81613	2.79577	0.00494	0.91209	1.31490	7.19684	13.64567	1.75963	3.91513	3.07742	0.00000	1.25817	3.39533	3.22128
**Icam2**	0.01191	0.00823	0.05347	0.06941	0.00997	0.00642	0.00733	0.01251	0.02856	0.53220	0.01415	0.01612	0.63620	0.00920	0.02774	0.00726
**Icam4**	1.13707	0.00560	3.33056	0.22131	0.00000	0.84455	0.04647	3.58768	0.58865	0.76684	0.75927	0.09580	3.01434	0.00333	0.00000	0.00329
**Ice1**	3.79471	7.33240	3.73253	20.71979	0.14890	2.08855	4.91071	5.67876	12.68337	6.07849	6.71930	11.37576	3.45717	3.13702	12.28039	2.44264
**Ice2**	3.42925	1.08095	1.63555	2.81000	3.95791	0.00185	0.00436	0.42842	0.96977	2.50554	1.05825	0.22882	0.11823	1.12816	1.03560	2.65854
**Ick**	2.11634	0.15995	1.29459	1.10878	0.00426	1.37152	0.00591	0.45529	0.00000	0.10744	0.00000	0.07415	0.53036	0.61259	0.30930	1.07094
**Icmt**	1.68835	0.42509	4.01224	4.15708	5.79357	5.47574	2.61011	6.07287	2.97478	2.72159	1.37071	0.52674	1.08396	0.05751	1.52158	2.01963
**Icosl**	0.00000	1.02285	0.16066	0.59397	0.44818	0.01818	0.00663	0.00766	0.24235	0.21397	0.04420	0.10867	0.13074	0.01149	0.23532	7.78226
**Ict1**	14.00888	0.72833	3.34160	2.88575	3.57997	4.95434	1.11026	9.71599	2.35322	12.99541	11.30037	0.74606	9.74360	0.00000	0.49021	5.84420
**Id1**	8.58850	0.00000	0.13282	1.98027	6.21078	0.00000	0.02950	5.55710	11.99331	4.69365	4.55589	0.04770	0.63949	0.23756	0.00000	0.00448
**Id2**	0.00000	1.28468	3.22914	1.32234	0.11075	0.06200	0.00600	0.03991	0.36377	0.00000	0.65283	0.00096	0.15290	0.00271	0.05356	0.01903
**Id3**	26.20049	1.76293	1.66990	0.01550	0.00000	0.02984	0.00000	23.41880	2.06982	14.48484	20.82396	0.36854	0.00000	0.01991	0.00458	1.29876
**Id4**	0.01377	0.00767	0.01166	0.20757	0.00936	0.00659	0.00742	0.01208	0.25327	0.02425	0.00955	0.00656	0.01314	0.01550	0.25190	0.00755
**Ide**	18.72121	13.95891	10.11882	2.78721	19.03138	8.84519	10.74385	9.47829	3.28352	4.97515	6.72444	7.77023	4.96858	7.65235	0.59719	3.11286
**Idh1**	18.79287	13.33546	8.46859	0.00000	13.19618	0.81138	7.14249	7.04905	0.37323	6.07379	7.22584	0.96377	8.97605	18.47092	2.87498	8.84485
**Idh2**	7.89024	5.78347	13.49668	2.87130	0.82166	0.91300	0.67264	10.08930	1.28520	10.31288	11.81833	0.47582	15.77587	0.84078	3.10602	4.48880
**Idh3a**	9.62596	18.37287	18.76192	5.24382	11.09870	17.93481	12.22337	11.34563	3.55535	5.63331	5.27257	6.05840	17.37907	1.22382	2.79955	9.41386
**Idh3b**	20.97440	11.73428	17.18725	4.86391	11.91765	11.05509	11.67121	19.55679	7.35571	17.82011	12.91502	2.08036	11.53381	4.19809	6.76882	11.96997
**Idh3g**	27.03527	11.00609	24.26184	8.47624	16.25111	12.62167	10.54415	25.80361	8.01911	24.37773	13.77879	13.62144	22.00694	14.73871	12.84347	17.94842
**Idi1**	2.05440	0.33080	0.86248	0.94094	0.01138	1.89841	1.81538	1.06008	0.27308	0.37978	1.51208	0.05713	0.16829	0.78865	2.32945	0.66957
**Idi2**	0.64769	0.08651	1.59495	0.05185	0.01175	0.01437	1.19170	3.49391	0.02509	1.10339	0.01727	0.02508	0.00000	2.69280	1.76640	0.00587
**Idnk**	0.20971	0.02634	0.50506	0.17449	3.47857	0.48112	1.06418	0.01716	0.40719	2.05645	0.37949	0.20076	0.06523	0.08924	1.44886	0.00661
**Ids**	3.67072	4.60482	3.09137	0.40496	0.01272	0.00257	0.01148	3.88739	3.26615	3.41774	5.71557	1.19603	0.00017	1.66515	4.11876	1.09993
**Idua**	1.12658	0.00344	0.20565	3.25461	0.00562	0.00648	0.00434	1.34411	0.94579	4.65837	2.64799	7.97147	0.03883	0.00000	2.19884	0.00460
**Ier2**	2.71400	0.02526	0.17039	0.29858	0.00959	0.02017	0.02359	0.02246	0.44871	3.97426	1.12389	0.10276	2.64855	0.00755	0.00000	4.42457
**Ier3**	3.29944	0.00262	0.63310	1.62384	2.65148	0.00581	0.00429	0.00000	0.14708	0.86768	1.11163	0.03367	0.01110	0.00136	0.05716	0.00315
**Ier3ip1**	10.31305	2.69667	4.68047	5.64151	3.31009	3.33904	2.83321	1.71688	10.00795	4.21133	5.59619	0.58158	2.43890	0.02056	0.00000	3.66917
**Ier5**	0.16971	0.00871	0.00381	4.68741	0.01718	0.01350	0.00730	0.01087	6.81083	0.01498	0.00000	0.00483	0.01244	0.00214	0.03141	0.00608
**Ier5l**	0.03340	0.00775	0.01696	0.07008	0.00824	0.00722	0.00699	0.00886	0.00862	0.34356	0.07487	0.00681	0.47515	0.01702	0.00446	0.00691
**Iffo1**	1.95598	0.01485	0.00000	0.82341	0.34441	0.15893	2.52575	0.00602	1.07316	0.01269	0.00523	0.02297	0.00916	0.00965	0.01033	0.00503
**Iffo2**	0.00000	0.00739	2.07599	20.78306	0.01320	0.34794	0.05468	0.00000	16.04096	1.91434	1.49513	0.09934	2.17597	4.98205	8.88961	3.30021
**Ifi203**	0.01463	0.00770	0.04974	0.68539	0.00863	0.00659	0.01785	0.30661	0.47795	1.13058	0.57266	0.37024	0.03605	0.46789	0.71322	0.02137
**Ifi27**	0.00585	0.00759	0.00000	0.04229	0.00852	0.00676	0.00708	0.34685	3.89863	3.78428	1.13750	0.44974	0.00000	0.04498	1.52621	0.00689
**Ifi30**	6.98640	0.06978	12.08299	2.21052	2.77096	9.08500	1.91157	6.73246	1.64379	1.31254	3.59718	0.05772	7.14280	0.64553	1.26505	2.82104
**Ifi35**	1.33324	1.69312	0.34372	6.42635	0.00440	0.00307	1.56473	4.74117	11.26028	11.00855	5.63029	0.50711	1.48850	1.13799	8.56695	0.04461
**Ifit1bl2**	0.02836	0.00800	0.11680	1.41977	0.00825	0.03049	0.02870	0.08006	0.95709	0.49725	0.00000	0.36620	0.02605	0.07822	0.01224	0.02223
**Ifitm1**	0.01209	0.00764	0.01126	0.56495	0.00860	0.00625	0.00721	0.00885	1.90949	2.96590	0.00000	0.00000	0.01048	0.00640	0.21361	0.00714
**Ifitm10**	0.01086	0.00765	0.00874	0.00828	0.00939	0.00669	0.00709	0.27365	0.04072	0.00700	0.01105	0.00702	0.12674	0.01372	0.00585	0.00723
**Ifitm2**	3.10174	0.27349	0.96151	0.38747	0.00913	0.11224	0.00401	0.07278	0.08088	6.13477	0.00345	0.11989	1.95538	0.15941	0.55788	0.59481

**Ifitm3**	0.03246	0.00791	0.03134	2.64777	0.01081	0.00620	0.00701	0.00891	0.09081	5.18407	0.00791	0.03282	0.01055	0.01899	0.14437	0.00708
**Ifitm6**	0.01361	0.00795	0.01170	4.53927	0.00918	0.00652	0.00739	0.01285	0.00000	0.25036	0.01137	0.00838	0.01283	0.03390	0.07208	0.00665
**Ifitm7**	0.01741	0.00802	0.01151	0.23362	0.00896	0.00658	0.01771	0.01897	0.20984	0.02078	0.02431	0.00750	0.01824	0.01030	0.01189	0.00727
**Ifnar1**	2.03245	0.61691	5.96137	2.28788	2.12176	0.58516	1.47340	2.57181	7.36271	3.60066	14.03179	1.54164	6.08682	3.72821	4.72626	1.03687
**Ifnar2**	0.14966	0.00843	0.00729	0.00617	0.01640	0.00697	0.00724	0.01017	0.06575	0.53753	0.01149	0.00659	0.01018	0.01166	0.00999	0.58827
**Ifngr1**	0.30278	0.00720	0.75776	0.00000	0.03782	0.00865	0.00676	0.00469	1.26966	2.14642	0.09261	0.03230	0.00711	0.03215	2.05484	1.59915
**Ifngr2**	0.18399	0.00747	0.32032	0.18254	0.01510	0.03063	0.02757	0.27137	0.07137	0.44984	0.03766	0.15541	0.00776	1.46234	0.14084	0.01422
**Ifnlr1**	0.01307	0.00756	0.01078	0.01361	0.00915	0.00642	0.00725	0.01036	0.62380	0.00000	0.00741	0.01606	0.14954	0.02482	0.33095	0.00714
**Ifrd1**	6.85333	10.69200	7.86275	14.29541	3.22719	2.23285	6.91302	9.12023	22.76055	12.00911	14.25072	18.98739	1.60796	9.20386	5.47579	4.29580
**Ifrd2**	2.03677	1.38152	0.53792	0.27126	0.24302	0.34120	0.57497	4.19941	1.08589	1.65557	0.32924	0.14829	1.28346	0.03883	0.14050	0.52397
**Ift122**	0.58329	0.00698	1.97582	0.17883	0.96095	0.00485	0.13424	0.50676	0.75859	0.96389	0.41180	0.15157	0.00000	0.00952	0.11819	1.19667
**Ift140**	1.29174	1.60998	1.89981	1.15782	0.00617	2.57900	4.62136	0.87550	0.32185	1.21895	0.00000	0.03228	0.09030	0.00331	0.43802	0.00367
**Ift172**	1.41519	0.01637	0.94410	1.10368	0.00370	0.00434	1.50256	1.73602	1.04203	0.93594	1.08610	0.01979	2.20326	0.03108	0.73855	0.01139
**Ift20**	13.79436	3.59891	7.86496	9.71278	1.41868	2.52151	6.61714	6.71411	18.55209	9.10567	4.28423	1.78156	4.09705	0.25293	1.10230	2.83262
**Ift22**	2.80214	0.00417	3.95138	0.01774	0.03879	0.24613	0.00415	1.65685	0.01102	0.62870	1.46222	0.00000	0.00000	0.03198	0.00987	0.00305
**Ift27**	0.32189	0.00696	0.00000	0.65383	0.06054	0.00928	1.29959	0.96045	0.00000	0.79087	0.04231	0.01948	0.49529	0.00879	0.12404	0.03135
**Ift43**	4.86002	1.04027	1.46080	9.27889	0.00000	0.00722	0.03147	0.99793	2.94088	3.35556	2.42370	0.08559	2.28294	1.53886	0.85668	0.02707
**Ift46**	11.09190	1.13777	11.02805	6.36664	6.35824	6.07709	4.22969	5.73849	4.14458	5.22950	2.88398	0.77643	7.27330	5.64001	5.14316	4.53394
**Ift52**	6.00100	8.76807	4.89947	3.67936	3.85631	2.66688	4.05658	0.36652	4.22972	0.66314	0.51876	0.07084	2.44666	2.44039	2.09987	1.80439
**Ift74**	0.71489	4.03704	2.04386	1.77072	0.00870	0.00442	0.00479	0.00405	1.54594	1.49779	0.00000	0.03921	0.60520	2.30794	1.89854	0.51350
**Ift80**	1.63017	0.00599	0.77997	0.29835	1.17951	0.00706	0.00568	0.06953	0.19907	0.54571	0.00741	0.04678	0.18540	1.39286	0.97330	0.00505
**Ift81**	0.18923	0.00610	0.00000	1.52668	2.89017	0.00620	0.00694	0.00549	0.05522	0.49038	0.00000	0.04527	0.00951	1.58118	1.49541	0.00646
**Ift88**	2.35220	3.57602	0.27724	2.76474	0.02046	1.72065	0.00388	0.77298	2.27175	0.97934	0.00000	0.08568	0.88809	0.05117	2.41744	0.42890
**Igbp1**	14.64181	10.18405	15.01376	0.00000	2.73018	6.00710	7.87800	13.72038	0.01080	8.40832	5.89547	9.96336	8.94308	9.99890	3.27091	13.47623
**Igdcc3**	0.01372	0.00764	0.01026	1.33992	0.00936	0.01255	0.00729	0.01144	0.72800	0.02262	0.00701	0.04285	0.01942	0.00317	0.48278	0.00714
**Igf1**	0.01332	0.00805	0.01211	0.12133	0.00907	0.00676	0.00744	0.01158	0.00697	0.76910	0.01103	0.00693	0.01184	0.01126	0.00738	0.00738
**Igf1r**	0.68641	0.00683	0.00655	0.85919	0.00661	0.00650	0.00677	0.01061	0.05642	0.35187	0.01286	0.06082	0.01720	0.00432	0.27673	0.26554
**Igf2**	0.71725	0.00802	0.00478	0.05299	0.02099	0.00771	0.00704	0.01142	0.01206	0.00858	0.01473	0.10618	0.14501	0.01184	0.00780	0.01505
**Igf2bp1**	17.89128	11.18443	7.89151	2.03288	0.09253	5.50609	3.07314	13.90469	5.15517	4.68415	5.31133	0.52276	14.35988	0.51895	1.68362	4.15426
**Igf2bp2**	15.41751	15.01582	14.32157	7.25323	11.39026	11.29549	9.28187	13.96146	2.36759	18.79526	9.29386	5.62914	8.84480	7.09185	10.94183	8.31500
**Igf2bp3**	14.64387	8.13663	9.96629	12.77269	3.34572	5.78614	7.07965	0.36535	9.59787	4.84525	2.02296	0.43125	8.21206	5.15710	11.86597	5.15749
**Igf2r**	2.92376	0.12670	3.91436	4.33215	0.17677	0.55074	0.92587	4.16070	10.82812	3.84747	13.60119	1.18491	1.91094	4.90785	10.64325	0.65402
**Igfbp2**	0.78216	0.00658	0.15342	1.34980	0.00591	0.00809	0.00627	0.00000	1.14659	1.02376	0.63462	0.34848	0.00706	0.00181	0.07107	0.00610
**Igfbp3**	0.01160	0.00808	0.00904	0.00287	0.00951	0.00685	0.00741	0.05133	0.00923	0.64836	0.14294	0.01614	0.01506	0.00610	1.09496	0.00754
**Igfbp4**	0.01210	0.00779	0.01220	0.00539	0.00856	0.00657	0.00725	0.00967	0.02128	3.42964	0.00953	0.00000	0.01067	0.01238	0.00675	0.00749
**Igfbp5**	0.01228	0.00771	0.01088	0.00547	0.00866	0.00666	0.00723	0.00979	0.01102	3.06450	0.00902	0.07063	0.01085	0.01312	0.00355	0.00747
**Igfbp6**	0.01624	0.00795	0.01107	0.31220	0.00875	0.00655	0.00723	0.02541	0.00438	1.40020	0.01043	0.03370	0.01014	0.01070	0.00247	0.00729
**Igfbp7**	0.01354	0.00778	0.01064	0.00837	0.00915	0.00672	0.00730	0.01083	0.01196	0.58014	0.01138	0.09531	0.01195	0.01193	0.00647	0.00749
**Igflr1**	0.42945	0.00586	1.09740	0.07498	0.00551	3.20443	1.85589	0.55385	0.01069	0.38929	0.72052	0.05635	0.17106	0.00721	0.00000	0.00587
**Ighmbp2**	2.85976	0.01330	0.57067	0.80754	0.12189	0.01336	0.76710	6.16427	1.90177	1.71865	1.51118	0.04474	3.35456	0.35650	0.03244	1.76418
**Igip**	1.23515	0.00621	0.00000	0.24694	0.00619	0.00628	0.00644	0.01075	0.00637	0.67671	0.00398	0.01787	0.15569	2.29088	0.65687	0.00613
**Igsf1**	0.01137	0.00762	0.00687	0.01053	0.00939	0.00652	0.00706	0.00936	0.01259	0.00014	0.00741	0.00302	0.19135	1.39032	0.00322	0.00718
**Igsf23**	0.01375	0.00793	0.01218	1.40552	0.00928	0.00641	0.00747	0.01242	0.04250	0.03836	0.02635	0.00704	0.01188	0.00313	0.23394	0.00698
**Igsf3**	0.03068	0.00658	1.78907	2.98812	0.01356	4.43058	0.75746	3.58843	2.04596	0.00000	1.07049	0.06279	1.22717	0.38017	0.71809	0.00359
**Igsf5**	0.01375	0.00805	0.01225	0.01061	0.00932	0.00666	0.00754	0.01390	0.01235	0.01531	0.01310	0.00811	0.01270	0.01234	0.01062	0.00741
**Igsf8**	0.01375	0.00815	0.01125	0.00646	0.00919	0.00684	0.00758	0.01175	0.04985	0.05547	0.10507	0.00821	0.01132	0.01133	0.00939	0.00734
**Igsf9**	0.01130	0.01769	1.17955	0.05905	0.02649	0.02760	0.00624	0.00384	0.01072	0.96427	0.34976	0.28788	1.58190	0.02070	0.14854	0.34826
**Igsf9b**	0.01627	0.00866	0.85243	0.39413	0.00868	0.00568	0.00732	0.07779	0.05917	0.00290	0.01217	0.00771	1.45458	0.01252	0.01907	0.00631
**Ihh**	0.01334	0.00760	0.01206	0.08200	0.00907	0.00653	0.00725	0.01016	0.01049	0.86588	0.01262	0.00507	0.01155	0.01441	0.02107	0.00759
**Ik**	10.16266	10.93507	9.42117	9.82412	2.15190	9.53651	6.10805	10.88057	19.78940	6.59605	9.15385	1.40872	8.30483	13.16134	7.98548	3.94185
**Ikbip**	1.10254	0.03966	0.94827	1.73708	0.06371	0.06355	0.16439	0.37585	2.27359	1.58788	0.89057	0.17431	1.95467	0.41674	2.10445	0.07738
**Ikbkap**	5.64401	12.05971	7.96143	2.47328	2.75018	0.00703	8.85524	8.13414	1.31392	8.38971	3.99454	4.05852	2.96151	2.96813	5.64474	4.38770
**Ikbkb**	0.00769	0.00805	0.00941	0.00553	0.00749	0.00682	0.00747	0.00695	0.01130	0.11322	0.30262	0.00738	0.00618	0.00715	0.05815	1.55101
**Ikbkg**	1.63334	0.33791	6.95406	6.78585	2.62210	0.15265	0.20423	4.04987	12.98134	5.08297	11.90078	5.13047	1.70793	14.26873	5.27320	0.58840
**Ikzf2**	0.01960	0.00765	0.00600	0.00711	0.01035	3.48111	0.00707	0.01046	0.01163	0.00766	0.00842	0.00694	0.00006	0.01798	0.37688	0.00622
**Ikzf4**	0.01315	0.00731	0.00736	0.00727	0.00925	0.00690	0.00709	0.63099	0.06629	0.00301	0.00821	0.00594	0.00563	0.02234	0.08885	0.00732
**Ikzf5**	0.87806	2.51628	0.44439	3.03095	3.81316	2.93582	0.00518	0.80883	0.85232	1.06663	0.13634	2.24107	0.00000	1.56523	0.88945	0.52260
**Il10rb**	1.60257	0.00591	0.62416	0.15442	0.00358	0.00785	0.01504	0.00383	0.05875	0.88498	0.00985	0.00315	1.33935	0.01145	0.00000	0.37290
**Il11**	0.14196	0.06088	0.46569	0.92848	0.25442	0.01796	0.09915	0.00746	2.08687	0.25790	1.67640	0.10853	0.48679	0.68867	0.54194	0.03546
**Il11ra1**	0.00572	0.00815	0.00822	0.41518	0.00773	2.39146	0.01789	0.01225	1.29435	0.42471	0.00000	0.00477	0.08598	0.01056	0.00431	2.34082
**Il12rb1**	0.01373	0.00794	0.01173	0.01074	0.00932	0.00660	0.00745	0.01290	0.01207	0.01633	0.01251	0.00772	0.01923	0.04182	0.05020	0.00741
**Il12rb2**	0.03757	0.00783	0.00251	0.01816	0.01182	0.00677	0.00691	0.00839	0.01206	0.01458	0.00753	0.01188	0.22101	1.88824	0.00779	0.00709

**Il13ra1**	0.01370	0.00762	0.01133	1.56956	0.00932	0.00633	0.00734	0.01184	0.60545	0.00254	0.00765	0.00603	0.01913	0.02079	0.32078	0.00702
**Il17b**	0.00972	0.00775	0.02101	0.00916	0.00948	0.00801	0.00724	0.34942	0.01171	0.21612	0.03414	0.00684	0.00793	0.01219	0.00558	0.00738
**Il17d**	0.01774	0.00800	0.01173	0.00922	0.00891	0.00685	0.00759	0.01414	0.01219	0.07420	0.02751	0.00842	0.01279	0.00696	0.09091	0.00739
**Il17ra**	1.00037	0.00564	1.95017	6.92736	0.04423	0.00531	0.57254	1.25229	12.43797	1.45625	0.25275	0.18786	0.00686	0.59364	1.71511	0.07487
**Il17rb**	1.86129	0.00576	0.52381	0.97476	0.00363	0.00349	0.00573	0.41956	0.47159	0.81516	0.06634	0.00007	0.24176	0.20429	0.11533	0.00482
**Il17rc**	0.02206	0.00797	0.01729	0.10249	0.00948	0.00707	0.00669	0.01154	0.01019	0.03107	0.01401	0.00731	1.20849	0.01334	0.00790	0.00674
**Il17rd**	2.94495	0.01354	1.14881	5.49608	0.01717	0.00167	0.04406	1.26285	5.95414	0.13169	0.77416	3.14804	0.00037	3.79497	3.04485	0.02286
**Il17re**	0.00876	0.00772	0.11543	0.00509	0.00887	0.00898	0.00672	0.01487	0.14051	0.04872	0.16912	0.00584	0.86348	0.01144	0.00532	0.00722
**Il18**	0.01124	0.00865	0.68443	0.00555	0.00839	0.00566	0.00800	0.24106	0.01106	0.51184	0.20509	0.00593	0.00254	0.00745	0.09127	0.00698
**Il18bp**	0.44011	0.79903	0.00000	0.00722	0.00768	0.00853	0.00670	0.01104	0.01185	0.63682	0.01132	0.01597	0.04195	0.08451	0.02708	0.01402
**Il1bos**	0.01115	0.00803	0.03502	0.02457	0.00942	0.00596	0.10104	0.04131	0.01198	0.03499	0.01095	0.00719	0.01602	0.21023	0.21572	0.00728
**Il1f9**	0.02152	0.04117	0.48637	7.22092	0.04240	2.11241	0.00710	0.02738	6.68450	0.03843	0.43275	0.38242	0.25106	0.58277	1.13133	0.21778
**Il1r1**	0.02179	0.00790	0.01091	0.47352	0.00858	0.00650	0.00722	0.01057	0.26841	0.67533	0.01121	0.02805	0.01109	0.00988	0.11005	0.00714
**Il1rap**	0.01543	0.00712	0.00450	0.41049	0.01807	0.00727	1.34981	0.00000	0.30048	0.00000	0.37605	0.01509	0.00799	0.00708	0.01725	0.00701
**Il1rapl2**	0.02165	0.00764	0.03892	0.03194	0.03548	0.03261	0.00729	0.01951	0.10566	0.04641	0.02261	0.01561	0.04133	0.45031	0.07951	0.00734
**Il1rl1**	0.17315	0.02997	0.18835	0.04320	0.23299	0.03508	0.00752	0.02120	0.14123	0.00696	0.60498	0.09421	0.39596	0.06132	0.03404	0.04945
**Il20rb**	0.01170	0.00803	0.00881	0.00394	0.00919	0.00664	0.00721	0.00939	0.23634	0.79373	0.00427	0.00000	0.01204	1.20832	0.12409	0.00765
**Il23a**	0.17261	0.00821	0.00479	0.01968	0.00738	0.00743	0.00772	0.09544	0.01106	0.02148	0.26095	0.03242	0.01090	0.01474	0.00995	0.00689
**Il2ra**	0.02787	0.00779	0.03012	0.00816	0.00841	0.00714	0.01742	0.02558	0.11251	0.02225	0.05408	0.01715	0.01725	0.15194	0.04080	0.00731
**Il2rg**	0.01008	0.01846	2.55776	0.42313	0.00464	0.00948	0.00654	0.00000	0.12576	1.85526	0.81838	0.71263	0.76101	1.83969	1.17400	0.00598
**Il31**	0.01374	0.00781	0.01185	0.00514	0.00931	0.00666	0.00750	0.01316	0.32609	0.01749	0.00990	0.00714	0.01300	0.01792	0.05160	0.00751
**Il33**	0.00957	0.00852	0.35035	0.00815	0.00960	0.00649	0.00790	0.02113	0.02661	0.22177	0.01276	0.02072	0.55978	0.00868	0.03932	0.00745
**Il34**	0.71136	0.00703	2.78239	0.69412	0.00151	0.00789	0.00540	0.00000	0.18748	0.00886	0.45888	0.08603	1.50432	0.02046	0.16257	0.01829
**Il3ra**	0.01171	0.00763	0.01333	0.01125	0.02159	0.00747	0.00703	0.89381	0.01143	0.00933	0.00704	0.00581	0.17534	0.01559	0.00000	0.01435
**Il4**	0.01362	0.00815	0.01207	0.10849	0.00918	0.00652	0.01789	0.01241	0.49461	0.15897	0.00929	0.00672	0.01169	0.01062	0.00854	0.00712
**Il4ra**	0.01133	0.00715	0.05499	0.00546	0.00937	0.00590	0.00666	0.78687	0.01035	0.34955	1.06195	0.00860	0.00000	0.00904	0.00105	0.00757
**Il5ra**	0.02558	0.04111	0.12176	0.03878	0.00927	0.00618	0.00748	0.02121	0.02225	0.03156	0.22592	0.01682	0.08792	0.02183	0.10097	0.01440
**Il6**	0.01374	0.00806	0.01221	0.00657	0.00932	0.00666	0.00754	0.01392	0.09824	0.01479	0.01210	0.00801	0.01273	0.01264	0.00978	0.00741
**Il6ra**	1.85991	0.00463	0.97838	1.27067	0.01082	0.00714	3.10758	0.15150	0.43681	0.71255	0.00000	0.05649	1.84950	0.11755	4.13315	0.01119
**Il6st**	5.44385	0.00000	0.00042	2.45447	1.24657	0.00195	0.00286	0.00492	2.01319	0.21818	0.00000	0.03876	0.01013	0.00136	1.33896	0.23291
**Il7**	0.01361	0.00758	0.01106	5.49999	0.00943	0.00586	0.00712	0.01036	0.00000	0.00499	0.01912	0.00430	0.01260	0.00000	1.52905	0.00639
**Ildr1**	3.56929	0.00023	0.00000	0.52686	4.24094	0.00981	0.12176	0.00000	0.41797	0.00000	0.00859	0.05007	3.31872	0.01109	0.17494	3.57573
**Ilf2**	13.90875	11.96592	12.34535	0.24286	7.43677	3.49792	5.35779	12.78979	1.52978	12.78000	3.03097	6.29147	5.70800	26.38885	18.27439	5.25481
**Ilf3**	8.20641	8.70891	11.14505	2.32002	1.36330	6.96156	6.87195	11.62962	2.43878	11.09378	4.05677	1.99294	7.22289	1.61780	13.98157	3.67804
**Ilk**	1.78597	0.56120	3.34111	1.53650	3.88628	8.35649	0.71200	3.18510	1.81209	4.10193	0.43783	0.02375	1.32599	2.30045	1.69770	0.03727
**Ilkap**	4.41538	7.04250	4.61280	0.99942	2.61841	0.00175	2.45813	0.64016	0.27542	2.48738	0.88074	0.16901	2.56111	0.00000	1.18255	2.43890
**Ilvbl**	0.00000	0.00786	0.02278	0.00769	0.00735	0.01527	0.00690	0.01038	0.01167	0.67356	0.01108	0.06505	1.05804	0.00957	0.10130	2.80733
**Immp1l**	2.13776	3.48100	1.86237	9.05140	0.00515	2.38916	0.01253	0.30828	1.21494	1.93391	2.08547	1.30809	1.70762	0.00000	1.70489	2.98629
**Immp2l**	0.00824	0.00819	0.01438	0.21991	0.00894	0.00704	0.00773	0.01682	0.00840	0.00740	0.01627	0.00798	0.15994	0.01188	0.00696	0.70830
**Immt**	14.22969	11.26948	7.22546	4.73265	14.61291	6.70392	5.38129	8.18444	4.31076	4.86621	5.04319	3.53445	8.21323	5.29528	12.28294	8.60356
**Imp3**	4.40155	0.75900	0.27533	22.50170	4.49211	3.29052	0.00000	1.99162	31.68638	15.21136	12.59082	0.51661	1.60397	1.72938	1.70818	0.23417
**Imp4**	19.10162	10.48356	17.94522	13.79245	7.77738	8.35952	9.74979	22.39734	20.71894	25.02550	12.63572	13.13459	19.00243	2.73364	11.13701	11.19080
**Impa1**	4.97346	10.38358	5.99627	0.00000	3.99889	0.00000	2.87933	0.00000	1.67703	3.86090	1.21134	1.21691	0.35614	0.49567	4.45939	1.46694
**Impa2**	4.39616	2.81292	5.19035	3.64850	3.22980	3.02269	3.14021	0.14344	5.94614	4.17944	3.59271	0.15738	8.14959	0.03695	1.02499	7.34241
**Impact**	8.57038	4.17503	3.34178	0.62550	3.46980	0.00000	8.39346	1.70707	0.03905	8.76592	2.30407	14.65128	7.57239	17.69941	11.59181	0.02680
**Impad1**	1.36421	0.19495	0.11188	0.00003	3.22209	0.61661	0.03558	0.18179	1.14900	0.23292	0.12358	1.18314	0.26005	0.55039	0.31077	0.01303
**Impdh1**	14.96840	1.95120	7.36576	9.77856	11.22029	6.94934	8.96629	18.15166	10.69071	16.68770	16.36270	1.74457	16.06998	0.18791	0.34881	6.45058
**Impdh2**	30.35209	23.24129	30.54070	20.26822	19.87651	15.79988	23.25704	57.85254	40.59877	61.34086	55.96853	29.02838	37.92826	34.90808	34.11602	22.59084
**Impg2**	0.00979	0.00793	0.00872	0.16571	0.17561	0.00677	0.00760	0.03025	0.19755	0.28578	0.00922	0.09922	0.00997	0.00926	0.03364	0.00705
**Ina**	1.37365	0.00631	0.00000	1.32560	0.00784	0.90304	0.00608	0.00489	0.00000	2.19587	0.02332	0.22040	0.30480	0.01695	1.90411	0.00527
**Inadl**	2.35651	3.23675	0.19885	1.59899	0.00450	3.83064	0.00317	0.25837	0.12602	0.29266	0.07361	0.03728	1.36348	0.02365	2.26968	2.18819
**Inafm1**	0.00747	0.00806	1.02788	0.46494	0.00979	0.00581	0.60444	0.19321	0.00544	0.00000	2.77244	0.04898	1.30173	0.00122	0.30049	0.00585
**Incenp**	12.09499	3.09840	7.34992	17.27053	3.12727	0.49835	0.69834	15.84231	23.22070	9.87154	7.61280	1.23964	7.46619	6.15735	7.31806	4.19820
**Inf2**	0.01793	0.03830	0.00000	0.05150	0.00946	5.58485	0.00581	0.42141	0.01144	1.25141	2.14458	0.17716	0.00000	0.00865	0.00736	4.23210
**Ing1**	10.52969	1.56527	10.46372	18.31027	0.35154	4.68825	2.98403	8.50837	21.02732	16.36527	7.50349	3.12648	6.44584	8.43601	11.94367	3.94251
**Ing2**	8.75847	3.14861	1.71819	5.58215	4.55444	2.93339	3.87033	4.05079	4.85392	5.03332	1.92097	0.48746	5.22106	2.01706	3.10426	5.20122
**Ing3**	0.11848	0.47921	1.06784	0.53855	0.61814	0.22764	0.00631	0.55484	0.07007	0.63639	0.62586	0.05658	0.50946	0.60171	2.06270	1.44503
**Ing4**	8.81652	3.69317	10.60955	4.72486	1.73389	0.02288	5.95392	4.39022	6.47946	13.57053	4.89610	1.23675	6.68822	1.03401	6.56928	4.58401
**Ing5**	4.11498	3.20472	6.23055	2.29436	3.16390	1.06323	2.12813	8.71640	1.03600	4.59425	3.38122	0.70553	6.14924	1.04408	4.99963	2.97581
**Inha**	0.01221	0.00799	0.01217	0.00494	0.00869	0.00655	0.00742	0.01165	0.08877	2.82334	0.00894	0.00000	0.01041	0.01268	0.00654	0.00725
**Inhba**	0.04787	0.04007	0.02561	0.02777	0.03741	0.01426	0.00731	0.01230	0.04463	0.02451	0.01064	0.26617	0.04791	0.05007	0.04165	0.02956
**Inhbb**	0.67681	0.00706	0.00903	0.00594	0.36488	0.00613	0.00674	0.00754	0.01150	0.93421	0.31920	0.03305	0.00876	0.00567	0.11515	0.01403

**Inip**	0.50981	0.02561	2.52265	4.23419	0.04559	2.60653	3.36988	3.84837	5.72492	4.23795	1.99573	0.40865	5.27342	0.91365	0.44158	1.83197
**Ino80**	5.24087	0.03657	3.38903	4.38259	2.74810	0.00000	6.29543	6.51309	6.50337	7.03614	3.42125	0.23523	2.63248	0.59863	3.33276	2.62512
**Ino80b**	0.01508	0.00812	0.02547	0.09810	0.00913	0.00615	0.00761	0.01416	0.01030	0.04917	0.20179	0.00740	0.00979	0.01047	0.00381	0.00736
**Ino80c**	12.06062	9.94640	6.80965	8.11586	0.40119	9.33714	9.19285	9.46194	6.71487	8.51178	9.43344	6.80303	9.03658	4.66201	13.48213	4.88198
**Ino80d**	4.43844	1.62078	1.17419	7.14338	2.70529	5.17572	3.14874	2.09104	3.69048	6.78426	6.67223	2.37029	2.86774	9.20629	10.64086	1.62280
**Ino80dos**	0.01508	0.00768	0.04974	0.03898	0.00992	0.00628	0.00750	0.01279	0.11303	0.10738	0.01098	0.01569	0.06031	0.00992	0.37513	0.00765
**Ino80e**	6.64585	0.62991	1.37900	0.57325	3.56614	1.33794	1.71441	6.43416	1.01021	5.33286	0.77181	0.18497	9.72226	0.00216	0.00000	5.13104
**Inpp1**	0.01836	0.00814	0.00811	7.28077	0.00953	0.02020	0.07408	0.02412	4.25336	0.00000	0.00000	0.03781	0.18639	0.27529	0.00000	0.00602
**Inpp4a**	0.02117	0.00797	0.99933	1.97864	0.01295	0.00878	0.00746	1.62555	0.58110	0.00110	0.01812	0.01012	0.01323	0.00621	1.42163	0.01439
**Inpp5a**	0.04140	0.01701	0.46261	1.09542	0.02450	0.00890	0.06635	0.59776	0.25469	0.51333	0.66230	1.57201	0.02409	0.00221	0.46487	0.30182
**Inpp5b**	3.32699	3.49306	0.00000	1.48654	3.47552	0.00348	0.00296	2.78427	1.49909	0.36036	0.91379	0.13416	1.00344	0.00000	2.15897	0.02356
**Inpp5d**	3.61427	5.96718	1.17050	0.37405	0.63494	1.50371	1.01330	2.49798	0.98313	0.00000	4.88621	0.12949	0.19697	0.46976	0.11051	0.37019
**Inpp5e**	0.92075	0.00553	0.00000	1.26281	0.01397	0.00575	1.21144	0.24276	0.08572	0.00000	0.21028	0.02577	0.16071	1.35489	0.63987	0.89543
**Inpp5f**	0.00000	1.06968	4.32575	2.58943	1.60275	1.34221	0.00582	1.00240	1.09855	1.05160	0.30616	1.23510	0.54361	3.92855	0.95019	2.87793
**Inpp5k**	3.56969	0.00000	2.24086	0.28900	4.60042	4.90166	2.27452	7.03513	1.78790	9.01476	4.25224	0.46882	5.13267	0.02273	0.45109	0.75422
**Inppl1**	0.28560	0.00797	0.00237	0.50995	0.12619	0.00816	0.00665	0.36264	0.08311	0.10457	0.77939	0.04299	0.01080	0.02447	0.13106	0.00701
**Insig1**	1.62960	3.69039	1.50609	4.17961	0.01099	6.54255	5.88637	3.01594	0.68935	2.92427	3.91911	0.06755	1.85944	5.50009	4.85442	0.73592
**Insig2**	2.34650	1.16478	0.87265	0.00000	0.01303	0.00294	1.35417	0.76916	0.07580	0.36836	0.39493	5.08988	1.21534	0.00463	0.95970	0.01003
**Insm1**	0.05528	0.00824	0.03122	0.41036	0.02717	0.04578	0.00744	0.00919	0.38638	0.00755	0.11321	0.00679	0.03034	0.04607	0.02370	0.02225
**Insr**	0.27731	0.00758	0.20680	0.07977	0.01317	0.00577	0.74522	0.50425	0.01623	0.00000	0.01078	0.05182	0.02739	1.92525	1.15849	1.54623
**Ints1**	9.74707	0.58461	3.81701	9.44573	0.30459	3.46699	2.61240	12.07021	7.65241	18.56287	4.22940	0.84617	13.35366	0.00000	0.75177	6.58602
**Ints10**	2.65768	4.84133	6.57095	1.85142	1.90153	1.01802	0.03501	1.81777	3.68989	5.54230	1.10190	0.54049	1.34108	0.81575	7.83940	0.26417
**Ints12**	9.23105	7.02254	3.01299	3.92105	1.50298	0.89599	3.41392	7.97489	8.98761	5.53534	7.14519	7.12984	4.30410	0.57684	6.15386	3.25034
**Ints2**	0.96732	1.38357	2.65542	3.70361	1.54373	0.80042	0.00272	1.11043	5.90683	5.42230	0.64615	8.25707	2.78092	12.55839	13.71511	0.03786
**Ints3**	14.23720	6.26203	14.63067	9.78064	11.35039	7.44309	5.02876	14.04932	9.92860	21.63495	3.12672	2.03355	8.36920	1.80224	15.46424	4.64202
**Ints4**	7.25884	10.64327	4.09765	5.31642	1.54614	4.76376	2.24084	9.20185	11.44514	13.23030	7.67403	5.86771	6.73561	21.20449	11.72994	0.02949
**Ints5**	8.47160	2.05015	2.89990	10.23653	3.91342	7.57219	4.04250	5.13916	9.16666	6.35688	2.33744	0.55095	2.53030	0.00000	0.60612	4.62869
**Ints6**	0.36911	4.14075	2.51230	3.53110	0.01733	2.99091	0.00388	0.00000	5.63315	8.39496	1.75772	3.03230	1.42043	1.69608	6.64957	0.01628
**Ints7**	9.65799	4.55137	5.71325	3.01142	0.61394	0.84760	5.86096	7.24717	2.60726	12.69747	5.59922	1.19810	2.87693	0.78823	5.52454	6.36311
**Ints8**	4.20359	1.33485	0.24304	1.00155	0.82137	0.77110	4.22115	2.75197	1.58628	1.99076	0.82992	0.42532	0.00000	15.47938	5.71147	1.88029
**Ints9**	3.99577	3.47401	2.72817	1.37898	3.42135	3.12288	5.48704	5.50823	2.43550	6.72580	1.70387	0.79731	1.11100	4.85213	4.84052	3.03939
**Intu**	0.29598	0.14882	0.32807	36.39591	0.03452	0.11422	0.42117	0.13402	32.45018	6.30898	2.69157	1.15109	0.63970	13.13038	20.04110	0.11058
**Invs**	2.32215	1.69927	2.01777	0.97503	1.88053	0.67335	1.45516	3.55406	0.91436	2.04711	1.23527	1.87478	1.68120	0.45126	1.98689	0.68336
**Ip6k1**	1.06075	0.09158	1.56460	2.90048	0.01690	0.01005	2.92133	2.95387	6.56485	1.94463	2.49323	0.03182	0.26911	1.47120	2.01071	0.00451
**Ip6k2**	0.88714	0.00757	0.97301	0.01039	0.01688	0.00537	0.00661	0.05416	0.01241	0.03944	0.01072	0.00678	1.06637	0.01069	0.01964	0.51717
**Ipcef1**	0.01319	0.01813	0.01696	0.27050	0.00932	0.00625	0.00746	0.01216	0.35031	0.00519	0.00932	0.00647	0.01258	0.00843	0.38267	0.00727
**Ipmk**	2.50695	0.02367	1.37483	14.07394	4.54867	0.00285	0.00336	3.07442	3.29854	1.52856	2.24519	1.02977	0.76623	3.38485	14.37991	3.52958
**Ipo11**	2.21711	8.42803	6.10608	3.35479	12.31610	0.61799	0.49652	10.92429	9.12371	12.83186	4.62156	2.85584	2.62533	14.63115	9.01987	0.01036
**Ipo13**	2.50305	0.02487	2.83312	2.32011	3.77320	4.62783	0.00260	0.31800	1.20455	3.50984	0.00000	2.44976	4.14410	0.00219	0.89977	4.10658
**Ipo4**	13.17577	3.02397	4.33925	12.25209	10.79163	7.50694	10.13506	28.87888	11.93182	28.27792	21.21962	5.08262	15.95431	0.43415	1.96822	5.30722
**Ipo5**	18.07591	14.49229	19.58868	16.47843	9.55087	9.59792	10.13214	36.32201	25.03919	42.06680	29.21778	25.16502	21.59900	39.94276	31.29528	13.05761
**Ipo7**	2.00990	4.89690	2.22617	0.92801	0.97493	0.15694	0.45559	3.89142	1.53291	0.65832	0.96574	5.83014	0.21811	10.30936	4.68535	0.01559
**Ipo8**	2.08795	6.15735	3.59160	15.78376	6.27637	5.38765	2.43717	3.43404	6.59991	3.65700	4.47274	0.06690	3.42652	7.00655	7.55096	3.65449
**Ipo9**	7.83854	6.24636	8.21783	7.52498	1.53381	2.60420	2.91103	4.78107	4.64262	4.32562	0.89895	0.17605	4.01519	6.63444	4.74818	4.70743
**Ipp**	1.36682	0.00597	0.58564	1.35475	0.00568	0.00773	0.00522	0.00000	0.36788	0.28476	0.88886	0.14304	0.79248	2.04370	3.25766	0.00515
**Ippk**	3.23565	3.33496	3.03095	5.65166	0.07662	0.00929	5.02425	0.35466	6.11277	7.81743	5.38553	0.10897	1.19138	5.69219	4.88772	0.01305
**Iqca**	0.01015	0.00603	0.19667	13.87824	0.00933	0.00427	2.75667	0.00000	1.27333	0.56971	0.32842	0.07882	0.02134	0.00000	2.98256	0.00472
**Iqcb1**	0.11635	0.05440	0.00543	0.22622	0.03152	0.01316	0.00731	0.01190	0.00798	0.00573	0.01322	0.00799	0.00732	0.01222	0.01397	1.58351
**Iqcc**	0.87513	0.00500	0.00000	0.56779	0.00633	4.01085	2.62616	0.00490	0.02140	0.91884	0.02931	0.06287	0.02302	0.01518	0.00000	0.00467
**Iqcd**	0.35198	0.00788	0.00365	0.01264	0.02106	0.00812	1.25235	0.01057	0.01052	0.01990	0.84188	0.00299	0.04314	0.01112	0.00000	0.02321
**Iqce**	1.61255	0.00463	0.57408	0.00322	0.01249	0.01546	3.01286	0.00000	0.01069	0.89975	0.22051	0.00000	1.18504	0.01126	0.15858	0.02078
**Iqcf1**	0.00909	0.00765	0.00879	2.32631	0.00911	0.00618	0.00704	0.18084	2.11854	1.33334	0.00000	0.06945	0.01424	0.00584	0.35266	0.00672
**Iqcf5**	0.01240	0.00838	0.00492	1.76860	0.00898	0.00742	0.00732	0.00758	0.12116	0.12400	1.71297	0.01127	0.15320	0.00081	0.00000	0.00645
**Iqcg**	2.98276	3.02621	5.88068	6.70450	0.00000	0.15974	0.26329	2.44940	8.36370	11.09223	3.79853	5.42248	0.78057	10.16116	8.91838	0.00468
**Iqgap1**	6.22121	4.26279	7.74393	3.21272	4.20922	6.98490	7.44737	8.75881	4.02570	3.20536	2.85365	0.95391	2.60243	7.87680	3.59498	8.13689
**Iqgap2**	0.88788	0.00784	0.00000	2.19774	0.01682	0.00575	0.44065	0.35153	0.74377	0.25497	0.00041	0.00000	0.28092	1.26720	1.02966	0.00603
**Iqgap3**	4.63146	1.34479	3.61056	0.76655	2.23803	3.46677	3.79817	16.37193	0.34162	5.73572	7.04659	1.66312	4.55782	0.00000	3.07502	6.06591
**Iqsec1**	1.42446	0.05095	0.13558	2.36835	0.21125	1.55944	0.00418	0.50270	0.75403	2.93739	1.84915	0.14244	3.41227	0.02117	0.03698	0.48567
**Iqsec2**	0.01219	0.00805	0.02809	0.35670	0.01009	0.00805	0.00766	0.01567	0.20950	0.00746	0.01345	0.02662	0.17928	0.00779	0.04863	0.00757
**Iqub**	0.00000	0.89238	1.90585	4.10716	0.00442	0.00370	0.00543	3.77787	10.74897	4.08042	1.76260	3.01464	0.53429	9.28945	3.50332	0.00394
**Irak1**	6.54497	1.74734	4.93898	18.08205	5.93535	0.66614	1.00350	0.00000	22.48907	0.99477	22.12789	2.47864	5.25770	11.48892	2.46049	1.24715
**Irak1bp1**	0.01541	0.00751	0.04912	1.07861	0.00930	0.00575	0.00723	0.00927	0.18582	0.03220	0.05888	0.00538	0.01140	0.00922	0.67888	0.00717

**Irak2**	1.28414	0.10510	2.83190	1.83172	0.21559	0.11557	0.39860	4.79911	3.67828	4.79672	6.23948	0.51155	4.28756	2.72832	3.18115	0.22771
**Irak3**	0.01105	0.00737	0.01424	0.51821	0.55812	0.01237	0.00728	0.69186	0.79030	1.02637	0.19780	0.12079	0.01385	0.04284	0.88737	0.00721
**Irak4**	0.29584	0.00827	0.00451	0.00734	0.01778	0.00725	0.23320	0.00857	0.01183	0.77082	0.01156	0.01519	0.01088	0.02770	0.04418	0.00724
**Ireb2**	5.66433	3.87562	3.20728	18.68543	8.55639	0.23296	2.72932	2.74244	9.45617	6.93011	5.24559	17.27314	0.73737	18.29532	16.36322	2.85882
**Irf1**	0.56336	0.00743	0.00000	0.75752	0.01368	0.00656	0.00659	0.96145	5.12853	0.03933	0.00000	0.03152	0.00569	0.00000	1.22554	0.00684
**Irf2bp1**	2.68682	0.09891	0.88286	0.00047	1.50458	0.03075	0.40991	1.52111	5.42289	1.90425	0.52782	0.01778	5.16484	0.00265	0.05993	0.60279
**Irf2bp2**	1.49939	0.00550	0.19803	0.14565	0.00476	0.00744	0.00599	0.00449	0.04708	0.75258	0.01025	0.00347	0.01047	0.01372	0.28534	0.00572
**Irf2bpl**	2.46641	0.03506	1.15208	5.48146	0.00453	6.17254	1.64196	8.99685	4.37971	10.30865	3.74571	3.02224	4.36917	5.79980	9.17774	0.24656
**Irf3**	5.93445	0.02273	0.00000	3.48985	0.43563	5.75264	1.42239	3.54461	4.80958	4.18987	0.43381	0.13306	6.11957	0.03186	0.00000	7.44301
**Irf5**	0.12838	0.00920	0.00248	0.00498	0.01621	0.01512	0.00793	0.12228	0.24275	0.54932	0.44609	0.00512	0.00859	0.01265	0.00111	0.00718
**Irf6**	0.72265	0.00697	0.42191	0.28946	0.00813	0.00905	0.00592	0.51360	0.00670	1.98462	1.95432	0.00000	0.07765	0.06311	0.00000	0.00641
**Irf7**	0.01112	0.00793	0.56026	0.70948	0.00807	0.00554	0.00739	0.00945	0.79286	0.94497	0.01436	0.04594	0.13477	0.00405	0.20463	0.00680
**Irf8**	0.01309	0.00812	0.01133	0.93789	0.00898	0.00654	0.00739	0.01062	0.00000	0.95901	0.02597	0.00555	0.01126	0.00444	0.64752	0.00713
**Irf9**	1.01081	0.00000	0.87225	2.76846	0.18370	0.00475	6.38476	2.78328	4.33042	3.39448	3.13623	7.31095	0.45387	1.13515	4.53291	1.52949
**Irgm1**	0.05345	0.00803	0.02175	0.46835	0.03796	0.01200	0.01767	0.00044	0.80831	0.00000	0.40240	0.00421	0.00813	0.12098	0.02555	0.01433
**Irgm2**	0.05057	0.01868	0.04828	0.07533	0.01903	0.02033	0.02799	0.03612	0.08720	0.03765	0.10558	0.00602	0.03579	0.28637	0.09942	0.00735
**Irgq**	0.31751	0.02891	5.24227	10.62336	0.01462	3.12211	0.00205	2.92278	19.32340	6.59764	3.17956	0.84669	5.21374	0.00000	3.68453	1.56881
**Irs3**	0.92035	0.00774	0.00420	0.57350	0.01712	0.00702	0.00660	0.02448	0.00425	0.01518	0.01375	0.00744	0.01099	0.01083	0.00492	0.00658
**Irx2**	0.92482	0.00668	0.00390	0.97130	0.37954	0.00710	0.00682	0.01002	0.01186	0.01454	0.01262	0.00746	0.01081	0.01089	0.00419	0.00619
**Isca1**	8.50093	8.05162	5.38478	4.52938	12.35762	9.81128	3.96654	9.72669	6.19604	10.59400	3.16220	7.19869	5.01693	0.29230	3.79140	3.42884
**Isca2**	3.84673	2.65374	1.87515	0.93718	5.25551	2.36822	0.00000	2.18090	2.30415	2.36272	2.89280	3.19616	1.82279	0.00000	2.15660	0.02000
**Iscu**	4.64188	2.23581	1.18329	6.16904	2.92739	2.54398	1.94072	0.73421	6.38670	4.81507	8.90658	6.38105	1.86357	0.41026	0.95142	1.40693
**Isg15**	0.01306	0.00777	0.01087	0.88634	0.00899	0.00631	0.01735	0.00887	3.58377	0.50349	0.01826	0.00000	0.01217	0.02218	0.00835	0.00742
**Isg20**	0.01146	0.00755	0.00960	0.99937	0.02625	1.18646	0.00713	0.00952	0.20196	4.39994	0.00756	0.00000	0.01253	0.00412	0.09651	0.00676
**Isg20l2**	1.83094	1.23347	1.21781	4.40707	0.72970	0.01276	0.20717	1.69886	0.40241	3.58080	1.02543	0.25651	2.35146	12.63387	10.45802	0.99383
**Isl2**	0.40937	0.00709	0.00350	2.89437	0.00731	0.00779	0.00671	0.23806	0.04917	3.83885	0.55170	0.00006	0.72650	0.00439	0.00000	0.00605
**Islr2**	0.00777	0.00801	0.01109	0.12901	0.00763	0.00644	0.00747	0.01189	0.00972	0.00500	0.10767	0.00777	0.00700	0.01111	0.00574	1.32847
**Ism2**	0.03409	0.00787	0.01030	0.34787	0.00842	0.00653	0.00727	0.01089	0.16534	0.32447	0.03976	0.01624	0.01164	0.00619	0.19895	0.01455
**Isoc1**	4.04649	4.67049	2.63523	0.25729	0.09208	0.97959	1.62757	0.60660	0.25030	1.51053	1.75850	3.19616	2.66687	3.06136	0.05531	0.02856
**Isoc2b**	0.11071	0.89736	0.86638	0.42345	0.02399	0.00579	0.72838	0.00000	0.58006	0.94575	2.09955	0.01394	1.04707	0.00263	0.10994	0.00567
**Israa**	0.01802	0.00744	0.00722	0.00461	0.00886	0.00670	0.13736	0.00246	0.01007	0.00000	0.28321	0.01436	0.00905	0.01351	0.60846	0.00713
**Ist1**	9.69976	10.18347	13.03622	14.96703	8.38788	7.75459	6.67896	15.10486	21.48199	17.76861	10.04505	8.38620	10.01816	15.50496	16.95474	0.04830
**Isy1**	11.09948	5.90703	6.40461	2.97657	6.36541	5.45133	3.19086	17.40972	2.42463	6.19966	5.81624	5.09836	10.82213	0.61217	2.85603	5.27936
**Isyna1**	36.34319	13.26649	32.47032	6.52954	24.28348	17.17990	22.33391	49.72426	4.52707	50.61146	38.16139	17.50849	53.00295	0.19095	0.00000	17.08185
**Itch**	2.74318	5.24627	6.34217	3.01298	1.53608	6.59231	7.88684	8.96056	1.78295	12.52823	3.99141	8.59527	2.11388	17.52864	14.11813	1.82681
**Itfg1**	2.63776	3.13141	3.54207	0.83656	2.18457	1.02381	0.37888	1.13400	0.20620	1.68359	1.02762	0.01473	1.17361	0.73839	1.29373	0.01523
**Itfg2**	1.68307	3.43560	3.88126	1.85937	4.66340	0.00302	5.17651	6.43500	3.80455	4.19165	0.25996	5.08754	2.56233	3.51906	1.96649	4.08024
**Itga1**	0.00934	0.00734	0.00597	0.01199	0.00956	0.00695	0.00681	0.50382	0.49774	0.02071	0.22534	0.03121	0.37513	0.05799	0.00000	0.00716
**Itga10**	0.01376	0.00780	0.01187	0.59884	0.00934	0.00658	0.00749	0.01322	0.25315	0.01050	0.01072	0.00737	0.01281	0.01911	0.05752	0.00730
**Itga11**	0.01396	0.00796	0.00488	0.00236	0.00837	0.02276	0.00750	0.02701	0.35986	3.04089	1.08367	0.25713	0.00705	0.00574	0.72262	0.00689
**Itga2b**	0.44911	0.00741	0.00475	0.09166	0.00680	0.00723	0.00674	0.02218	0.02013	0.23195	0.10564	0.00709	0.45552	0.01054	0.00688	0.00662
**Itga3**	6.98567	4.00154	5.01557	0.00000	0.04360	3.96188	8.85978	3.01336	0.02210	0.89840	2.94624	0.04988	3.19901	0.00657	0.00104	4.18783
**Itga4**	0.12792	0.01899	0.05870	0.48968	0.06311	0.00602	0.07108	0.03662	0.71557	0.74154	0.02616	0.03816	0.02716	0.53474	0.48160	0.01467
**Itga5**	4.84962	0.01044	3.87425	0.00007	0.51686	0.02952	5.29445	0.98450	0.14106	0.31451	1.14611	0.25988	5.89362	0.00880	0.00000	6.08923
**Itga6**	7.27787	3.55686	9.02764	5.89611	9.93319	2.08581	7.47585	1.75652	8.71319	3.13323	2.53817	3.05776	1.21064	4.11216	8.29670	2.12004
**Itga7**	0.00765	0.00793	0.74060	0.29965	0.00786	0.13261	0.00671	0.00403	0.00441	0.15304	0.21396	0.00657	1.03172	0.03512	0.00000	0.00657
**Itga9**	0.01367	0.00815	0.01141	4.00898	0.00922	0.00643	0.00739	0.01179	0.20964	0.08626	0.04882	0.00774	0.01249	0.00000	0.40030	0.00674
**Itgae**	0.11609	1.22847	1.35691	1.26784	0.00521	0.01171	1.25352	1.76017	0.47364	0.46637	2.38915	0.03049	1.67150	1.72652	2.25794	0.01168
**Itgal**	0.00636	1.76634	0.00594	6.87607	0.00788	0.00542	0.00634	0.00801	2.57541	1.12022	0.00000	0.00769	0.01018	0.00000	1.28571	0.00507
**Itgam**	0.01334	0.01858	0.01166	0.00467	0.00916	0.00686	0.00757	0.01300	0.34376	0.21806	0.00875	0.00776	0.01286	0.01365	0.05739	0.00738
**Itgav**	2.72068	0.00367	0.42658	5.90568	0.00214	2.32852	0.05562	1.19608	3.76057	5.10025	1.07825	2.41181	0.78230	11.01359	6.88874	0.00005
**Itgb1**	6.83284	1.66865	4.26908	3.57207	3.01104	0.04219	8.11391	7.32819	0.31963	2.53075	3.28188	0.78148	6.58420	5.74248	3.31264	3.92734
**Itgb1bp1**	6.42085	5.31734	11.10069	7.82502	7.05895	0.00000	5.48453	12.84091	9.90239	6.31602	9.48394	0.88242	9.23455	0.79454	0.00000	6.57869
**Itgb3**	0.17856	0.01836	0.00747	2.39569	0.00761	0.00635	0.00706	0.02251	0.11450	0.34659	0.02756	0.02547	0.01806	0.00131	0.75354	0.01466
**Itgb3bp**	0.59093	0.52182	0.01420	0.12003	0.00947	0.00578	0.00744	0.24816	0.00937	0.00825	0.01100	0.01886	0.00666	0.03588	0.00000	0.01393
**Itgb4**	0.00544	0.00705	0.16426	1.56230	0.00779	0.00900	0.65199	2.01551	1.01445	6.80303	0.58976	0.10229	0.00002	0.00346	0.00000	0.00678
**Itgb5**	6.43684	0.00001	5.41467	7.09148	0.01169	6.18883	6.38401	14.96187	5.14543	16.36811	6.30975	2.96636	7.64509	0.83179	10.26172	2.43129
**Itgb7**	1.94171	0.00447	1.78671	0.46210	0.00965	0.00310	1.06984	2.71660	4.96179	2.92449	2.27156	0.07066	0.36054	0.05150	0.81219	0.00366
**Itih5**	0.01320	0.00816	0.01217	0.00671	0.00895	0.00652	0.00738	0.01066	0.01161	1.01021	0.01064	0.00577	0.01065	0.00735	0.09066	0.00714
**Itln1**	0.01086	0.00783	0.00779	0.13542	0.00951	0.00687	0.00711	0.31013	0.00701	0.00000	0.08592	0.01710	0.00599	0.00733	0.11293	0.00735
**Itm2a**	0.01371	0.00756	0.00571	0.00624	0.00926	0.00672	0.00702	0.00781	0.01039	0.00000	0.07187	1.03288	0.01200	0.00790	0.41704	0.00735
**Itm2b**	3.42941	2.62481	2.07088	1.53102	2.97214	8.62480	0.16131	0.00000	3.27212	9.89659	4.99466	0.07531	2.69666	1.96922	0.00000	2.74075

**Itm2c**	1.11464	0.00731	0.17422	0.28311	0.01436	0.00483	0.00638	0.01355	0.14079	4.31242	0.00781	0.06396	0.96639	0.02572	0.45817	0.02097
**Itpa**	12.96160	10.48044	13.73493	5.46386	16.20409	9.47634	7.05336	17.80005	9.10949	17.56325	16.42249	1.65704	18.61809	6.07025	11.77846	6.14308
**Itpk1**	10.25397	0.02924	1.83750	6.56479	0.11653	0.00000	2.34613	0.08011	4.60885	1.90067	2.13772	0.68050	0.88676	0.00000	0.21029	4.27261
**Itpka**	0.43403	0.00696	0.00000	0.62650	0.00697	0.01594	0.00700	2.22664	0.00143	1.19699	0.71588	0.41695	0.00545	0.00350	0.00000	0.01381
**Itpkb**	0.01375	0.00812	0.01230	0.30812	0.00931	0.00662	0.00752	0.01394	0.00678	0.01549	0.01388	0.00821	0.01266	0.01128	0.00642	0.00736
**Itpkc**	1.58643	0.00293	0.33871	8.30667	1.60527	0.02728	2.02438	2.33397	14.46985	5.09081	6.12795	0.43150	0.58439	0.40774	8.11254	0.52875
**Itpr1**	0.01143	0.00773	0.00576	9.86184	0.00915	0.00477	0.00700	0.01233	4.68671	0.66811	0.05623	0.00000	0.01108	0.00544	6.46562	0.00532
**Itpr2**	0.01086	0.00776	0.00377	0.84889	0.00902	0.00711	0.01667	0.12751	2.01983	0.80535	0.67243	0.02632	0.00427	0.19103	0.35626	0.00716
**Itpr3**	4.23473	0.03417	2.09523	1.44410	0.00000	0.00000	1.45886	3.60437	2.79677	3.81325	2.10845	0.20692	2.51377	0.00000	0.00000	0.00742
**Itpripl1**	0.45770	0.00792	0.83801	0.00882	0.01168	0.00924	0.00713	0.01321	0.02323	0.17551	0.01017	0.01894	0.00877	0.00784	0.07294	0.00676
**Itpripl2**	0.01884	0.00783	0.25689	0.00693	0.02170	1.42870	0.00700	0.00924	0.01216	0.59703	0.00973	0.03319	0.00511	0.01220	0.00523	0.01483
**Itsn1**	6.02384	2.75667	1.93059	8.43871	4.63071	0.06748	5.82728	3.56839	9.02980	8.15581	8.48587	3.46433	0.13448	3.05688	8.14218	1.71736
**Itsn2**	1.66584	0.95175	1.85904	2.57050	0.00944	1.57705	0.02367	1.23394	0.00000	0.97558	0.81033	0.04434	1.64275	3.28804	0.69559	0.32515
**Ivd**	1.61732	3.57679	1.75270	6.23621	4.30090	2.84911	0.93558	2.94607	7.18682	0.77967	2.48792	0.11455	2.63835	0.00000	2.27691	5.99522
**Ivns1abp**	6.50063	12.71570	17.33124	12.61512	10.57590	5.22907	12.07525	16.64378	14.59473	21.60389	21.57782	16.25072	14.72609	30.84441	20.46238	4.74949
**Iws1**	6.16171	3.91458	2.15567	11.52747	8.69901	4.58748	3.23945	5.95160	14.70382	11.67155	8.68712	3.97960	4.69124	15.92915	14.96305	1.79674
**Izumo1**	0.04418	0.00803	0.00986	0.01056	0.00803	0.00666	0.00728	0.01276	0.01243	0.00866	0.01247	0.00787	0.21856	0.03410	0.00913	0.00708
**Izumo1r**	0.01369	0.00816	0.01057	1.60623	0.00930	0.00661	0.00750	0.01472	0.00000	0.01068	0.03815	0.08739	0.01208	0.02873	0.01353	0.00714
**Izumo4**	0.01921	0.00807	0.07271	0.57636	0.00857	0.02619	0.00746	0.01347	0.27885	0.02318	0.01095	0.00808	0.01158	0.01090	0.00502	0.00724
**Jade1**	4.73109	5.97500	10.41071	3.77778	5.05400	2.05196	1.50610	2.94572	9.49868	8.56806	1.36732	0.25434	1.14480	1.52766	7.59526	6.10347
**Jade2**	0.01070	0.00807	0.00569	3.68304	0.00984	0.00708	0.00721	0.00000	2.52612	0.24965	0.49732	0.10147	0.47651	0.00000	1.30995	0.01440
**Jade3**	0.37662	4.69714	2.29842	1.93608	2.02998	0.75213	0.00333	0.19892	2.27108	0.47381	0.06813	9.98517	1.29035	3.03795	0.00000	2.26781
**Jag1**	0.01394	0.00763	0.00923	0.86006	0.00929	0.00661	0.00728	0.00868	0.00000	0.00369	0.08985	0.09275	0.01141	0.03157	0.29822	0.00723
**Jag2**	0.52431	0.05289	0.03215	0.33694	0.00687	0.19037	0.02966	0.10921	0.09410	2.42364	0.00941	0.00000	1.89464	0.01410	0.00000	0.00579
**Jagn1**	10.43786	2.47387	8.56753	3.89866	7.13781	10.65048	2.02060	12.85721	2.88530	10.60715	4.29866	0.23613	10.25713	0.05817	3.59091	5.45909
**Jak1**	5.85029	2.13195	5.84161	5.56474	1.63733	4.84817	4.25277	4.22424	2.33190	3.43110	2.83420	7.41304	0.37214	8.15961	5.04871	1.45962
**Jak2**	0.83239	0.00564	1.66450	19.49471	1.31138	0.00101	0.00542	0.00000	17.17910	9.02192	0.83953	1.63601	0.04581	7.76877	26.31581	0.02245
**Jak3**	0.62903	0.01773	0.01140	0.00465	0.04684	0.00636	0.00684	0.04424	0.76618	1.39979	0.00618	0.00000	0.36784	0.01516	0.04398	0.01438
**Jakmip1**	0.61874	0.00643	1.37129	0.02917	0.00424	0.00472	0.01656	2.18038	0.10000	4.32301	1.16406	1.27228	0.00000	0.02991	0.62362	0.00564
**Jakmip3**	0.01359	0.00819	0.01164	0.17513	0.00924	0.00681	0.00762	0.01391	0.76062	0.05834	0.02136	0.00769	0.01296	0.01289	0.00833	0.00735
**Jam2**	7.90364	2.10228	1.55966	2.28221	11.29169	0.00223	0.02847	0.75476	0.81481	2.39452	2.65153	0.79296	1.61095	0.00000	2.63550	0.05585
**Jam3**	0.01512	0.00761	0.00468	4.84052	0.01003	0.00468	0.33246	0.00849	11.92722	0.33185	0.00000	0.07519	0.01751	0.00651	3.02963	0.00562
**Jarid2**	16.98446	12.73639	21.53959	6.72540	9.69858	8.28996	12.01782	25.67937	3.94646	15.04030	24.63800	8.05381	12.12135	17.95077	11.56590	9.36030
**Jazf1**	0.01068	0.00829	0.00677	9.24716	0.00938	0.00514	0.00775	0.09660	3.61328	0.00000	0.00000	0.01779	0.01314	0.07515	2.59714	0.00535
**Jchain**	0.01187	0.00786	0.01125	0.05746	0.00911	0.00643	0.00714	0.01189	0.02763	0.01876	0.01320	0.02904	0.40808	0.01539	0.03042	0.00705
**Jdp2**	0.00971	0.00774	0.08937	0.01063	0.00922	0.02578	0.00719	0.10549	0.01230	0.03190	0.01170	0.00721	0.00751	0.01203	0.00761	0.04340
**Jkamp**	8.34279	1.13622	4.45061	1.03730	2.39722	0.03069	1.41364	4.12070	1.64101	4.88955	2.15649	2.44165	5.19267	2.26552	1.12122	4.46069
**Jmjd1c**	3.91006	0.82246	0.36298	2.84739	0.00000	0.00785	0.99441	2.46179	4.80930	1.37649	1.96154	5.92238	0.17720	7.12037	4.64685	2.21776
**Jmjd4**	1.51748	0.00557	1.77945	4.00254	0.01289	2.61812	0.05818	2.69245	3.72392	0.96474	0.54041	0.03424	0.84189	0.13177	0.56106	1.99607
**Jmjd6**	7.74046	3.53918	8.55718	10.49401	5.58476	4.01587	4.82010	14.99823	21.64169	20.80504	8.12425	2.14736	4.16895	2.36951	10.52154	4.04091
**Jmjd7**	0.14841	0.05907	0.15952	0.11844	0.00784	0.06002	0.02887	0.04406	0.32012	0.22332	0.11715	0.00576	0.02382	0.08112	0.02017	0.14886
**Jmjd8**	0.08529	0.26933	0.36936	0.28064	0.01597	0.17820	0.62322	0.52819	0.22567	0.65974	0.67306	0.02036	0.80035	0.00443	0.19790	0.13460
**Jmy**	1.38569	0.00000	2.48731	16.06219	0.00000	0.37378	2.20679	6.80325	17.40849	9.69696	6.47958	6.97294	0.00000	22.14442	16.70696	0.04207
**Josd1**	7.30354	2.50308	4.37089	8.36962	4.19098	0.83818	6.42521	3.56706	5.08045	5.14946	0.32210	0.80551	0.39813	0.54348	3.33716	3.24812
**Josd2**	8.97608	0.31228	2.09533	0.20904	6.49935	4.79032	2.95528	7.04934	0.00001	0.26825	1.10711	0.06951	6.06031	0.00023	0.00000	4.16038
**Jph1**	0.94689	0.00718	0.00000	0.00552	0.01075	0.00631	0.00627	0.00000	0.01140	0.00000	0.22235	0.01837	0.01460	1.91202	0.01252	0.53840
**Jpx**	0.10380	0.01808	0.23677	0.24788	0.02704	0.02012	0.03783	0.00000	0.44415	0.05867	1.24361	0.08928	0.23710	0.38691	0.19391	0.41683
**Jrk**	0.00181	0.01897	2.86937	0.89661	0.00371	0.43430	0.00578	0.01270	0.00000	0.00000	0.02158	0.05018	2.30554	0.00622	0.35021	0.46512
**Jsrp1**	0.01102	0.00770	0.01014	0.02539	0.00951	0.00675	0.00719	0.12327	0.02451	0.06310	0.01321	0.00695	0.00854	0.01137	0.00885	0.00772
**Jtb**	7.68643	0.01337	1.13395	6.13991	5.32814	0.07207	2.59293	11.92009	9.48870	17.20174	14.91844	0.84584	11.64492	2.28441	0.26444	4.19035
**Jun**	0.00759	0.00778	0.04080	0.67064	0.01826	0.05259	0.00698	0.52140	0.00000	0.08164	0.90028	0.03509	0.55448	0.00542	0.00000	0.02811
**Junb**	0.90012	0.00678	0.00613	0.08074	0.00690	0.00635	0.02694	0.06494	0.08405	0.34206	0.35970	0.04810	0.74295	0.00969	0.00000	0.86600
**Jund**	0.20518	0.00678	0.00000	1.75090	0.92833	0.29356	0.00621	0.02461	2.34009	4.09877	2.02905	0.06505	0.07966	0.13827	0.33114	1.02589
**Jup**	0.25948	3.01266	0.61943	0.84954	4.46175	6.24543	3.66579	4.95161	0.33136	3.32159	3.86811	0.10497	2.75278	0.84045	0.10925	6.13304
**Kalrn**	0.29736	0.00500	0.99048	4.34459	4.21551	1.02306	0.00664	0.01318	1.34381	0.17955	0.14671	0.01181	0.38552	1.64964	0.65135	0.16693
**Kank1**	1.21194	0.00575	1.41097	1.35237	0.03416	2.67888	2.27981	0.14857	0.83435	2.69626	0.55649	0.01280	0.83887	0.01319	0.16371	0.00423
**Kank2**	1.27967	0.00601	2.01739	2.50118	0.01165	0.43238	0.00456	0.60952	8.93083	1.61404	5.46917	0.14432	0.43150	0.58278	1.01484	0.78664
**Kank3**	0.00000	0.00726	2.13968	1.12559	0.21923	0.00641	1.09261	0.09829	1.39944	0.61288	0.02356	0.01115	0.25443	0.00489	0.17324	0.00670
**Kank4**	0.00910	0.00762	0.00000	3.65868	0.00900	0.00628	0.01645	0.00000	3.70423	1.45655	2.76715	0.58068	0.00000	1.53420	0.83278	0.00612
**Kank4os**	0.00000	5.11433	0.19086	0.63937	0.00725	0.00838	1.36232	1.03774	1.04618	0.00000	0.25478	0.09558	0.00334	0.04385	1.01342	0.00488
**Kansl1**	3.05539	5.51193	1.76632	12.51825	4.00808	0.00000	4.87192	5.06996	12.88024	11.16358	4.20450	6.86047	1.98447	5.16808	6.05125	4.31585
**Kansl1l**	0.22282	0.00763	0.00000	1.12412	0.00872	0.57261	0.00707	0.01924	0.18097	0.00000	0.10224	0.00570	0.00593	0.00621	0.68434	0.00658

**Kansl2**	11.33537	8.97527	11.95999	15.03999	10.61949	0.05879	8.27336	12.30133	19.36174	17.38796	12.67807	8.33922	8.64905	11.80188	19.84861	9.47255
**Kansl3**	5.41795	0.85867	6.03832	8.52941	0.01186	0.05818	2.77618	6.97442	14.90052	10.13835	8.57720	4.65563	5.58382	9.35960	9.86131	3.36869
**Kantr**	0.73342	0.00616	0.00000	0.01477	0.00656	0.00625	0.67077	0.00482	0.40007	0.00000	0.02764	0.04390	0.01117	2.38703	0.08970	0.00661
**Kars**	21.24774	16.40869	21.50451	9.73279	15.20007	13.61695	10.09308	29.58119	13.96735	33.54849	19.51207	17.82112	18.76393	7.21295	20.02613	15.36363
**Kat2a**	14.86085	7.24182	6.70750	4.77900	8.65899	3.79185	6.46329	12.24321	5.37760	7.75437	4.79989	2.27719	5.43781	0.72590	7.28994	4.19822
**Kat2b**	5.13605	0.00691	0.00000	3.90465	2.82344	0.61981	0.00000	0.44087	4.25415	0.35979	2.86209	11.90849	0.00000	4.33403	3.55011	0.28376
**Kat5**	1.89504	2.54524	5.97974	2.46032	6.23134	0.01202	0.00940	8.49115	2.11785	7.82239	3.52726	1.50369	6.87734	2.32222	4.08282	4.17646
**Kat6a**	3.77391	1.35977	0.65937	15.13278	4.67337	0.00057	0.83264	0.00000	11.38810	5.50184	4.67015	0.80356	0.55427	2.87745	3.84065	1.59222
**Kat6b**	5.52363	2.88941	5.48998	6.44038	0.57074	2.59629	0.57108	8.54218	5.05384	7.91935	3.15238	7.29603	3.04551	9.71848	9.76865	4.40564
**Kat7**	9.99605	8.77916	9.84305	13.85833	7.19141	5.96722	7.60485	12.20546	13.68631	16.71520	9.61010	5.69560	11.51168	14.63021	14.06776	6.52819
**Kat8**	9.12531	0.44961	3.77968	4.38624	0.63823	0.00670	2.26840	9.56372	9.00008	11.39770	7.72976	2.06299	6.88558	0.95010	7.81231	1.09134
**Katna1**	9.33598	11.76576	10.87171	17.60831	4.40675	5.45029	9.86861	8.91577	30.08853	14.61539	8.89223	10.06289	7.53961	14.99473	17.39883	6.96961
**Katnal1**	0.02536	0.01708	0.29773	0.33376	0.00829	0.00540	0.00706	0.00910	1.60977	0.21443	0.00000	0.03612	0.01024	0.14105	0.99024	0.02164
**Katnal2**	0.05811	0.00763	0.00911	0.01767	0.00809	0.00672	0.00738	0.01323	0.01172	0.00715	0.01204	0.01741	0.01277	0.01482	0.26320	0.00726
**Katnb1**	3.76866	0.03558	2.20583	0.78631	0.47386	2.90523	0.00298	4.68847	3.76023	3.57737	0.31881	0.30331	1.87341	0.00000	0.06939	1.71381
**Katnbl1**	1.16576	3.14648	0.28101	9.47840	2.63727	3.92329	0.00272	1.65526	3.45609	4.48523	4.07359	1.16018	0.92193	2.65621	6.11921	0.02256
**Kazald1**	0.01172	0.00787	0.01163	0.26241	0.00861	0.00647	0.00737	0.01068	0.00764	0.11029	0.07287	0.00809	0.00966	0.01611	0.00336	0.28298
**Kazn**	2.69693	1.63139	3.12555	0.80192	0.17870	0.05954	1.50054	4.43906	1.37387	3.61904	2.28862	1.30605	0.03779	0.00000	1.07462	2.10956
**Kbtbd11**	0.01191	0.01882	0.01100	0.03353	0.00924	0.00651	0.00736	0.04723	0.99004	0.19697	0.00468	0.00523	0.00987	0.03122	0.03184	0.00708
**Kbtbd13**	0.01956	0.00867	0.00000	0.13003	0.01187	3.77858	0.00784	0.45896	1.67287	0.00000	0.17428	0.02526	0.18266	0.00380	0.40409	0.00574
**Kbtbd2**	1.10604	3.41470	0.55109	2.23894	0.00676	0.03986	0.38463	0.56527	4.97579	0.44218	2.56038	8.68804	0.14870	0.36650	5.23388	0.56331
**Kbtbd3**	0.58637	0.00720	0.07140	1.03527	0.00672	0.00488	0.00714	0.00665	2.69200	0.00507	0.31444	0.00000	0.00829	0.00593	0.00000	0.03435
**Kbtbd4**	6.10901	4.55532	0.80683	4.21078	0.00510	3.45053	0.09365	2.60825	6.51834	6.80386	3.13595	0.27494	1.82787	0.02311	2.85228	1.50048
**Kbtbd6**	0.02723	0.00739	0.00370	0.17643	0.00842	0.01980	0.00721	0.36233	2.50143	0.00000	0.00000	0.02100	0.00606	0.10117	0.35984	0.00703
**Kbtbd7**	0.06943	3.12899	0.72168	8.39606	0.02390	0.38158	0.00550	0.08591	10.62025	0.45700	0.63430	0.04179	0.50918	0.02011	3.69699	0.00325
**Kbtbd8**	0.00000	0.62882	0.00000	23.07362	0.88999	0.00841	0.93217	0.54775	9.56594	3.05139	3.00274	2.32878	0.00000	12.31035	8.45797	0.00156
**Kcmf1**	15.87034	13.29345	12.14805	21.58303	12.26228	8.27477	12.66494	15.31313	23.21920	18.07058	18.06304	19.06999	11.63498	4.23480	6.93932	7.27205
**Kcna2**	0.02014	0.00800	0.00947	0.03518	0.00891	0.01341	0.00759	0.01423	0.10144	0.03833	0.01115	0.00773	0.03940	0.20026	0.00825	0.00731
**Kcna7**	0.01259	0.00811	0.16561	0.40038	0.00887	0.00890	0.00736	0.01277	0.31272	0.01056	0.01068	0.00762	0.01062	0.00835	0.06147	0.00722
**Kcnab1**	0.01215	0.08277	0.01156	0.49318	0.00896	0.00662	0.00742	0.01253	0.00338	0.01459	0.01282	0.00812	0.01958	0.01103	0.00468	0.00727
**Kcnab2**	0.07445	0.00755	0.00611	0.20432	0.00773	0.00747	0.00729	0.00000	0.03603	0.31045	0.63738	0.05088	0.00731	0.00652	0.02933	0.01434
**Kcnb1**	0.01304	0.00691	0.00393	0.18829	2.47293	0.00909	0.00749	0.01273	0.00291	0.24770	0.03774	0.00552	0.60293	0.01060	0.00812	0.00738
**Kcnc3**	0.07442	0.00835	0.00903	0.06821	0.01595	0.00674	0.00729	0.02150	0.05714	0.09764	0.02981	0.00722	0.01164	0.01136	0.00727	0.00756
**Kcnd1**	0.01814	0.00810	0.00659	0.33004	0.00866	2.13881	0.00720	0.01039	0.01065	1.24177	0.00899	0.00503	0.00362	0.00987	0.01066	0.00627
**Kcnd3**	0.01363	0.00816	0.01173	0.10692	0.00930	0.00663	0.00753	0.01362	0.95875	0.01043	0.00601	0.01758	0.01300	0.01306	0.00415	0.00735
**Kcne1**	0.01235	0.00837	0.29462	0.40506	0.00875	0.00551	0.00768	0.01356	1.85712	0.00725	0.01516	0.00565	0.00992	0.01370	0.00000	0.00706
**Kcne3**	0.01229	0.00751	0.00915	0.00483	0.00853	0.00675	0.00701	0.00000	0.02374	3.56363	0.46607	0.00000	0.01379	0.01203	0.00000	0.00744
**Kcnf1**	0.00000	0.00616	1.96933	0.00000	0.01564	0.00916	0.00534	6.53294	0.00559	1.61287	3.00788	0.03396	0.72818	1.14627	2.79497	0.00570
**Kcng3**	0.01378	0.00769	0.01193	0.14560	0.00935	0.00654	0.00743	0.01225	0.00932	0.00906	0.01269	0.00701	0.01282	0.00881	0.34436	0.00739
**Kcng4**	0.01209	0.00787	0.41467	1.19173	0.00854	0.00894	0.00750	0.01903	0.07293	0.01177	0.01075	0.00690	0.00910	0.00506	0.24322	0.00699
**Kcnh1**	0.00662	0.00830	0.00255	5.02838	0.00982	0.00632	0.00785	0.24558	0.00000	0.64222	0.26489	0.01226	0.00507	1.04838	1.11825	0.00621
**Kcnh2**	0.01197	0.00863	0.01101	0.10040	0.00924	0.00676	0.00801	0.05396	0.04766	0.30614	0.01193	0.00746	0.01047	0.03391	0.00747	0.00776
**Kcnh3**	0.00841	0.00791	0.00862	0.06514	0.00943	0.00702	0.00719	0.67556	0.01727	0.56333	0.91594	0.02449	0.00000	0.00596	0.04444	0.00733
**Kcnh4**	0.01383	0.00815	0.00958	0.00576	0.00913	0.00679	0.00757	0.00749	0.01144	0.00699	0.34640	0.00696	0.00859	0.01114	0.00463	0.00721
**Kcnip2**	0.01197	0.00778	0.00773	1.28786	0.00906	0.00631	0.00722	0.01459	0.00000	1.13425	0.00360	0.00000	0.01029	0.58267	0.96461	0.00686
**Kcnip3**	0.10668	0.00773	0.01500	1.74057	0.00823	0.00728	0.00713	0.01048	0.00000	1.29050	0.02214	0.00406	0.01194	0.03692	0.04016	0.00693
**Kcnip4**	0.01338	0.00798	0.01240	0.49528	0.00940	0.00662	0.00744	0.01111	0.00368	0.50517	0.02527	0.00624	0.01683	0.00725	0.43509	0.00719
**Kcnj10**	0.01566	0.01775	0.01958	0.50411	0.00850	0.01936	0.00698	0.03721	0.02397	1.15679	0.01370	0.00461	0.41929	0.06191	0.05451	0.01368
**Kcnj13**	0.00954	0.00751	0.20815	0.37307	0.00910	0.00887	0.00689	0.00835	0.04950	0.07500	0.00737	0.00404	0.30218	0.84812	0.00903	0.00718
**Kcnj14**	0.01007	0.00711	0.94869	0.00671	0.00752	0.00786	0.00699	0.14835	0.04198	0.15409	0.00000	1.39200	0.00387	0.00634	0.28617	0.00691
**Kcnj15**	0.05244	0.03943	0.05359	0.01192	0.02658	0.07504	0.01800	0.08795	0.12033	0.03542	0.03645	0.01682	0.05996	0.08896	0.07448	0.02202
**Kcnj16**	0.01055	0.01870	0.02797	0.00823	0.01869	0.00730	0.01819	0.04493	0.03752	0.02914	0.02633	0.01808	0.02556	0.03701	0.04077	0.01454
**Kcnj2**	0.01780	0.01775	0.00600	0.19054	0.00882	0.00690	0.00711	0.00000	0.20811	0.00000	0.33769	0.01335	0.02319	0.37162	0.24477	0.00713
**Kcnk4**	0.01462	0.00751	0.02545	0.01085	0.00920	0.00605	0.00710	0.44931	0.01194	0.00832	0.01081	0.00657	0.00586	0.01269	0.00769	0.00734
**Kcnk5**	2.66772	0.19616	2.66017	4.12681	0.03852	0.52114	0.00954	9.35762	6.13040	18.28962	8.89949	2.45707	3.03265	1.82349	12.51442	0.35837
**Kcnk6**	1.16821	0.02402	1.96023	13.70729	0.04638	3.95130	0.00000	7.32670	16.42597	15.08454	19.59640	2.28914	6.54328	18.33159	11.80797	0.02368
**Kcnmb3**	0.01353	0.00820	0.01210	0.01062	0.00932	0.00668	0.00771	0.01864	0.01234	0.01531	0.01293	0.00836	0.01238	0.01264	0.01053	0.00741
**Kcnn1**	0.09796	0.02905	0.35780	0.16588	0.04167	0.04492	0.08643	0.00000	0.40188	0.74412	1.29520	0.02343	1.81284	0.55793	0.26008	0.08940
**Kcnn2**	0.32345	0.68375	0.00274	0.00343	0.00771	0.00822	0.00712	0.01513	0.35233	0.00770	0.01163	0.00686	0.08659	0.01305	0.00000	0.00700
**Kcnn4**	0.60406	0.00944	0.14572	0.59513	0.03625	3.12898	0.00775	0.20644	0.08346	0.05236	0.00647	0.01078	0.10500	0.01231	0.00000	0.01311
**Kcnq1**	0.01322	0.00756	0.01054	1.17834	0.00906	0.00658	0.00718	0.01007	0.01283	0.64701	0.01120	0.00506	0.01873	0.20097	0.00000	0.00758
**Kcnq1ot1**	0.88801	0.52648	4.94341	4.53770	0.55769	0.39756	1.02393	2.06985	8.33518	4.26347	1.92194	2.45390	1.99720	11.27291	1.74812	0.43805

**Kcnq5**	0.01353	0.00788	0.01084	0.00217	0.00938	0.00633	0.00734	0.01089	1.01584	0.00296	0.00000	0.01508	0.01296	0.00665	1.09685	0.00721
**Kcnrg**	0.01521	0.00822	0.01383	0.00968	0.02300	0.00713	0.00768	0.01428	0.01212	0.26754	0.01348	0.00694	0.22307	0.01387	0.02109	0.02270
**Kcns3**	4.39394	4.08036	6.93628	0.70374	5.26538	6.27923	0.43470	1.96891	0.74878	3.76557	3.05772	0.42200	0.30580	0.12730	3.96797	0.46629
**Kcnu1**	0.00569	0.00761	2.05034	0.58512	0.00701	0.00921	0.00688	0.35705	1.52073	0.00470	0.00000	0.00000	0.37112	0.00689	1.34000	0.00637
**Kcnv2**	0.01690	0.00775	0.01131	0.00493	0.02117	0.00639	0.00734	0.01153	0.05425	0.00557	0.01083	0.00615	0.02508	0.00604	0.64869	0.00731
**Kcp**	0.00577	0.00773	0.01196	0.28903	0.00909	0.00753	0.00604	0.25769	0.00452	0.00000	0.53233	0.01347	1.63728	0.00563	0.06666	0.00632
**Kctd1**	0.17331	0.00729	0.01700	0.27694	0.00781	0.00706	0.02678	0.00309	0.00796	0.66236	0.17208	0.05103	0.75277	0.01016	0.01289	0.00719
**Kctd10**	4.40709	1.40488	1.30737	0.12338	0.26666	1.66970	2.23672	6.56163	0.00533	6.72199	0.35430	0.21398	2.59965	0.04254	2.05619	2.78297
**Kctd12**	0.01359	0.00780	0.01057	2.03953	0.00915	0.01261	0.00735	0.01062	0.69762	0.29870	0.01666	0.00485	0.01144	0.01891	0.38445	0.00680
**Kctd12b**	0.00898	0.00871	0.00922	0.39894	0.01064	0.00757	0.00830	0.37045	0.00612	0.00000	0.14654	0.00736	0.19136	0.01066	0.00000	0.00757
**Kctd13**	1.84280	0.00451	0.00000	8.20669	1.05008	0.20078	0.21274	3.36339	6.72929	0.56700	2.80156	0.32123	0.00000	0.03261	1.51927	1.08071
**Kctd14**	0.02117	0.01812	0.12431	0.64066	0.02736	0.00696	0.01707	0.02245	1.09223	3.42125	0.21155	0.04414	0.01512	0.18471	0.03667	0.01480
**Kctd15**	0.74560	0.01530	0.00000	1.84685	0.01451	2.12237	2.28674	1.03062	1.26302	2.68949	0.56940	0.03163	0.46843	1.52689	0.63655	0.16748
**Kctd17**	0.01345	0.00805	0.01189	0.30883	0.00931	0.00659	0.00766	0.01763	0.34207	0.00927	0.01056	0.00751	0.01239	0.01095	0.09257	0.01461
**Kctd18**	0.00000	3.30013	1.01955	5.82260	1.22669	2.10158	0.01212	4.34152	5.82995	5.20436	0.00000	0.61624	7.00581	1.97718	1.37173	3.47080
**Kctd2**	0.53870	0.82709	1.62293	0.72879	0.37984	0.01424	0.00611	0.22403	0.44744	1.63250	0.00000	0.13796	0.24031	0.00372	0.52597	2.47036
**Kctd20**	2.81968	3.63926	6.09208	9.42441	5.14652	0.27678	4.40265	8.01523	8.11268	9.18497	2.22686	1.45922	3.32900	1.42126	8.67765	1.56330
**Kctd21**	0.24944	0.00789	0.00765	0.80999	0.00796	0.00735	0.00759	0.01413	0.76179	0.00387	0.01122	0.00669	0.11341	0.02669	0.06354	0.00720
**Kctd3**	2.25526	0.42771	3.00585	12.52958	3.78766	1.26822	3.83329	7.22832	8.87828	4.94006	3.71436	1.68479	0.55412	0.98956	9.31850	2.58611
**Kctd4**	0.01278	0.00764	0.00777	0.01067	0.00951	0.00675	0.00725	0.00983	0.01245	0.01117	0.00844	0.00409	0.01314	1.06483	0.00420	0.00738
**Kctd5**	8.19742	0.00000	3.35010	2.24846	4.58111	0.01105	4.78227	3.44805	3.54756	5.19729	6.55790	8.13671	7.46693	2.36888	2.32934	5.24844
**Kctd6**	2.41285	2.32300	0.59322	2.84192	0.00239	0.01477	0.02355	0.28072	5.78029	0.70977	1.46997	0.01915	0.42146	2.67543	1.36578	0.01762
**Kctd7**	0.01351	0.00768	0.00497	0.86218	0.00903	0.00651	0.00700	0.00000	1.16734	0.00000	0.65177	0.03919	0.02312	0.00346	0.99376	0.00677
**Kctd9**	1.24648	3.66978	3.00453	4.81664	2.50814	4.43051	5.43120	7.09855	6.25226	3.96728	4.24089	0.81470	1.97472	11.25498	1.38314	2.77791
**Kdelc1**	3.55387	2.78377	0.00000	0.00555	1.23738	0.00296	0.00333	0.00000	0.01150	0.00060	0.01257	0.01427	0.00847	0.00754	0.27710	0.86797
**Kdelc2**	0.46693	0.08853	0.46350	0.41467	2.40072	0.09500	0.11763	0.01763	2.07627	0.45206	2.41464	0.14901	0.43699	0.47365	0.35406	0.09240
**Kdelr1**	3.05816	0.04408	2.46279	1.67438	2.05577	5.38972	2.60277	2.46768	0.43293	3.01562	1.32751	0.01529	9.82151	0.00277	0.14372	7.63753
**Kdelr2**	3.81599	2.42557	6.79852	0.44982	2.13585	0.28098	0.09044	1.10870	0.00000	4.57427	3.18138	0.23370	4.62029	0.36966	0.69103	4.00579
**Kdelr3**	1.80279	3.20561	0.30695	0.28170	0.02109	0.00393	0.62119	0.00000	0.00410	3.40433	2.90301	0.04767	0.04417	0.55943	0.40179	3.04241
**Kdf1**	3.03016	0.00202	0.08183	0.78147	4.31158	0.03998	0.01322	0.04814	2.01469	5.39004	0.62441	0.00000	1.78379	0.02602	0.00000	6.28790
**Kdm1a**	7.29246	5.15747	1.47318	0.60748	3.50352	3.88873	2.80754	2.00740	0.25289	1.10233	0.47173	3.54850	1.63237	12.74827	14.57658	0.29596
**Kdm1b**	1.05321	0.01995	0.54319	8.39137	0.01480	0.13844	0.01673	0.17142	4.04627	0.19296	0.33535	0.11520	0.00580	2.66505	2.65887	0.00483
**Kdm2a**	1.67813	2.69186	2.19849	2.75231	0.00000	0.00628	0.00471	1.04408	2.20480	1.52956	1.15081	0.03282	0.00000	0.56333	3.84887	0.00298
**Kdm2b**	9.05182	5.22558	10.23752	15.52603	6.12699	6.17936	9.75955	17.82168	18.69623	24.76665	17.45454	17.73328	12.47264	17.60799	17.67784	0.53631
**Kdm3a**	3.60730	5.76216	3.88522	7.63063	0.20751	1.82204	6.35770	7.41004	11.03395	6.68109	3.45887	9.84881	0.72302	14.50487	8.47412	3.63577
**Kdm3b**	5.88635	5.26024	3.37753	1.59871	7.09097	5.27331	10.77744	5.89985	1.39493	12.07737	1.51739	0.83361	5.63105	6.80862	9.79247	0.45645
**Kdm4a**	0.29793	0.01849	0.04938	3.71460	0.01321	0.02472	0.00657	0.00000	6.19717	0.00000	1.37576	0.38344	0.05664	0.13070	0.22627	0.05040
**Kdm4b**	0.41978	0.95836	0.00000	1.50971	7.98484	2.68688	2.12011	2.08233	3.64599	1.75223	0.68041	0.01773	0.00000	0.03469	0.00000	3.15969
**Kdm4c**	0.14006	5.40385	0.07961	2.83795	0.01235	1.00210	0.00380	1.84006	6.00693	4.18020	1.58469	3.51167	0.52125	15.92559	13.71537	0.65029
**Kdm4d**	0.01190	0.00778	0.01078	0.94794	0.00932	0.00663	0.00719	0.06593	0.13362	0.14140	0.01261	0.01628	0.01016	0.00901	0.02641	0.00761
**Kdm5a**	1.41670	3.28035	5.47833	4.22712	0.08790	1.97141	0.09661	5.88546	5.00608	2.49219	1.10828	3.43332	1.75005	10.03948	13.32879	0.00705
**Kdm5b**	9.00977	12.83064	3.38952	19.74124	5.70873	3.62316	0.11151	13.46238	22.68060	13.12016	12.91153	10.85467	2.29400	27.38308	20.18742	1.85126
**Kdm5c**	12.98436	2.20509	4.30944	3.25429	0.89178	0.87800	4.20696	5.50286	2.90037	6.03068	2.03402	0.15217	6.71294	7.89665	5.03364	1.35860
**Kdm5d**	0.01455	0.00780	0.00000	0.00074	0.00914	2.47302	0.12949	0.00000	0.22510	0.00000	0.43282	0.00585	0.00000	0.01127	0.00258	0.00625
**Kdm6a**	1.86072	0.85561	2.30578	1.55052	3.90863	0.00424	1.64701	5.84532	0.23867	2.58139	0.00001	0.14326	0.67302	16.72195	11.73717	0.16786
**Kdm6b**	3.43809	0.06674	0.35302	1.05755	0.07772	1.45868	0.71236	1.13633	0.64667	1.40737	0.54181	0.03769	1.58740	0.02363	0.00000	2.62311
**Kdm7a**	0.51797	0.71489	0.12258	11.80143	0.06523	4.15367	0.32317	2.07973	10.22427	6.63190	2.64283	10.82914	1.01781	12.15074	18.98075	0.03649
**Kdm8**	0.18015	0.00724	0.00204	0.89974	0.00746	0.00726	0.02689	0.11343	0.55668	0.06262	0.49052	0.00000	0.00528	0.91010	0.09059	0.00688
**Kdsr**	1.12563	0.19675	1.89348	2.62379	0.00277	6.60857	3.45880	0.90170	1.20657	0.30759	2.97794	4.79979	2.09364	2.43497	3.42920	0.30202
**Keap1**	6.86154	0.04206	3.60620	2.07015	0.05753	4.15434	1.15305	4.39745	1.16596	3.40468	4.73013	0.13125	3.33282	0.11066	1.50960	2.56044
**Kel**	0.14549	0.00762	0.00801	0.01061	0.00766	0.00694	0.00751	0.01298	0.01235	0.01522	0.01301	0.00811	0.01250	0.01243	0.01072	0.01449
**Khdc1a**	2.95691	2.49334	5.97800	19.90923	3.13781	4.10640	3.90807	6.23440	19.18302	32.60888	24.20273	18.09002	5.44657	32.80042	22.84919	2.89997
**Khdc1b**	0.03323	0.11726	0.64622	20.50489	0.00707	0.01457	0.12648	1.12394	9.22988	6.56195	3.73361	0.85057	0.03507	11.29795	29.67687	0.00190
**Khdc1c**	0.84487	1.11971	0.63403	5.94818	0.00509	0.16714	0.71867	0.59009	10.69233	2.96623	11.86758	0.78663	0.41849	5.00253	5.73860	0.00169
**Khdc3**	18.42051	3.27512	7.50777	0.00000	21.22458	1.23398	2.99214	4.77013	19.56049	2.92262	26.10422	0.37264	12.12151	0.16653	0.00000	7.51415
**Khdrbs1**	7.44376	7.74770	6.35921	17.44002	3.57813	1.66092	6.77439	6.54315	19.86251	9.60593	13.49255	2.20639	6.78344	11.87766	13.24340	0.80598
**Khdrbs3**	0.01344	0.00776	0.01135	0.88581	0.00903	0.00647	0.00722	0.00974	0.00000	0.76293	0.02510	0.03778	0.01067	0.01932	0.00318	0.00702
**Khk**	0.01200	0.00788	0.01060	1.20817	0.00988	0.00631	0.00611	0.00521	0.17997	0.27574	0.13333	0.00453	2.10921	0.00378	0.10950	0.00613
**Khnyn**	2.20912	0.01375	1.14138	0.12888	0.01115	1.32298	0.86089	4.40200	0.00782	0.83469	3.72675	0.20548	7.13336	0.03718	1.40669	0.29892
**Khsrp**	2.82870	0.25418	2.09249	8.16462	0.81555	0.11585	0.06582	1.06435	9.50652	1.00490	11.21883	0.50685	1.79086	2.54566	2.29377	1.33711
**Kidins220**	0.00000	0.00387	8.07394	2.26067	0.02293	0.08791	2.48700	3.59586	3.02762	5.58292	6.37776	2.14873	3.66386	13.61746	7.65683	0.02971
**Kif11**	0.03870	6.54641	4.31588	7.17391	5.73431	0.61291	0.50983	6.54691	14.05813	3.79641	4.97117	6.56597	1.69902	16.11413	12.18420	0.67383

**Kif13a**	0.58069	4.75719	0.19100	0.77030	0.05573	0.00657	4.22095	0.00555	2.25427	1.45440	0.00000	0.00603	0.59859	0.00804	0.34086	0.00417
**Kif13b**	0.00089	0.00825	0.01217	0.29991	0.01246	0.20671	0.01821	2.73858	0.84926	0.31511	0.16199	0.02002	0.01582	3.47869	0.00000	0.00727
**Kif14**	0.21212	0.00859	1.23875	3.14442	0.03427	0.19108	0.14205	0.65228	1.44560	0.29478	0.42313	0.06126	0.08736	3.48189	1.39389	0.01489
**Kif15**	1.01596	2.76331	0.00000	0.12281	1.06722	1.55682	0.84235	0.65647	0.66244	0.51297	1.92712	0.07708	0.88358	4.15660	1.41853	0.41341
**Kif16b**	4.74046	5.58408	3.79711	0.56693	3.95899	1.99392	0.02937	5.53736	0.01523	10.13750	7.58732	2.13089	1.64051	8.09836	9.35752	0.00000
**Kif17**	0.01332	0.00782	0.00704	4.07641	0.00894	0.00604	0.00710	0.00000	2.69797	0.28936	0.43463	0.00000	0.00779	0.00000	0.82390	0.00620
**Kif18a**	0.17619	5.63082	1.55108	1.16287	2.71918	0.00497	1.65113	1.62131	15.25239	4.70471	2.56691	12.42807	0.01425	25.72735	11.71887	0.00884
**Kif18b**	1.46516	1.57326	2.86509	4.29769	0.00877	0.00379	0.13351	2.30839	11.31814	4.12163	2.45378	0.32735	0.69297	0.00000	3.19617	0.25016
**Kif1a**	0.02173	0.00779	0.01460	0.09461	0.00857	0.00631	0.00726	0.00000	0.01049	0.39373	0.43552	0.01470	0.00758	0.00868	0.00000	0.00697
**Kif1b**	4.31574	0.00000	4.51178	7.80606	4.81364	0.00637	4.49417	3.83661	15.08136	10.51034	6.64119	7.83783	2.21873	14.82338	14.16519	0.01732
**Kif1bp**	3.36934	1.66996	3.03263	4.63394	5.17104	0.04279	1.41209	6.17912	5.31548	6.56082	2.24887	9.02353	0.77677	2.34860	8.06877	0.02844
**Kif1c**	0.39753	1.29256	1.51901	0.84875	0.19245	2.28167	0.00579	0.27656	1.86697	0.99060	0.66681	0.16606	0.23266	0.01745	0.00000	0.25353
**Kif20a**	5.62002	8.16013	5.24061	0.21615	7.84191	6.48870	6.40098	19.93088	0.19580	15.94774	0.00000	4.77643	2.74249	0.06094	6.46153	1.90540
**Kif20b**	0.00000	1.80216	1.59169	5.28836	0.03158	0.00369	0.01500	0.17500	11.58646	1.46212	0.40545	6.77724	0.00000	14.06524	7.02402	0.00347
**Kif21a**	0.01170	0.00809	0.01081	0.21756	0.01006	0.00755	0.46511	0.01406	0.00768	0.24318	0.01432	0.02019	0.11473	0.00805	0.05613	0.00767
**Kif21b**	0.68251	0.00440	1.66556	0.74188	0.01590	0.00601	3.85335	0.00850	0.64067	1.60630	0.02118	0.00572	0.01038	0.00417	0.58407	0.47856
**Kif22**	7.77686	7.75982	9.93049	11.41464	12.19435	9.80225	7.75167	21.61069	23.20406	25.59704	11.48149	7.91578	6.00013	0.45483	16.93651	3.23238
**Kif23**	6.25780	11.97715	2.76732	12.51285	9.79906	6.30557	3.80421	20.49040	15.99567	18.14756	17.23088	13.18185	4.24344	23.70363	20.37161	4.63793
**Kif24**	0.01927	0.00697	0.63206	1.72719	0.03641	0.02380	1.65928	0.44194	1.01325	0.62916	0.44749	0.03358	0.23577	2.76780	2.38087	0.02902
**Kif26a**	0.75368	0.05804	0.00401	0.37988	0.00647	0.00699	0.04735	0.00576	0.12627	0.18918	0.09294	0.00613	0.01089	0.00678	0.06400	0.00652
**Kif27**	0.00984	0.00830	0.02497	0.88949	0.00946	0.00562	0.00814	0.49563	2.82207	0.00000	0.00000	0.05878	0.00560	0.00597	1.97510	0.00683
**Kif2a**	2.52014	1.95494	3.45397	13.26357	2.22351	4.80606	6.23527	5.62457	12.25254	7.16624	1.36057	6.09661	2.86154	15.57018	12.47710	1.89033
**Kif2c**	1.71817	3.85206	2.86571	12.44365	4.70611	5.46466	2.49614	8.23113	15.96830	7.24794	1.69132	0.47228	0.84149	0.53124	8.28593	0.07107
**Kif3a**	4.81484	0.06830	1.66527	2.59898	0.03117	1.98276	1.14868	2.74966	3.60370	0.67819	3.17287	0.58983	1.02493	1.93177	1.78427	4.13923
**Kif3b**	1.24119	0.00248	0.43738	1.88623	6.15933	0.00346	0.09127	1.11674	4.93736	0.00000	0.00000	1.60612	1.45074	0.00000	2.93493	1.00915
**Kif3c**	0.00175	0.00664	0.66020	2.39736	0.00693	0.00464	0.00641	6.46398	6.67829	5.90239	2.45438	0.31808	0.00000	0.37619	9.89577	0.00567
**Kif4**	5.27367	2.87187	3.39197	11.63655	4.13372	0.34692	2.91573	11.19208	16.64761	14.79649	5.55617	5.91098	0.23666	14.22191	19.49964	3.83928
**Kif5a**	0.01598	0.00787	0.01020	0.44517	0.00910	2.62615	0.00702	0.00393	0.07983	0.00000	0.22523	0.04571	0.00000	0.02724	0.00000	0.00613
**Kif5b**	8.28656	8.38997	8.33929	2.33382	4.75332	2.70519	4.23968	7.31530	5.69787	18.25829	6.19472	13.96961	8.68789	17.42830	19.52769	5.24385
**Kif5c**	0.01006	0.00765	0.00346	6.58343	0.00991	0.00526	0.00687	0.00633	2.34924	0.02456	0.03125	0.00000	0.01291	2.65212	4.50897	0.00607
**Kif7**	0.04859	0.00842	0.00915	0.56451	0.01404	0.00692	0.00750	0.00955	0.28551	0.06185	0.14325	0.00717	0.01109	0.00513	0.14506	0.00737
**Kif9**	0.17448	2.21721	0.00000	3.48345	0.11504	0.02294	2.74755	1.25233	5.10811	2.09130	0.28204	4.16414	1.97179	0.61777	2.57202	0.00470
**Kifap3**	0.01124	0.60145	1.93903	0.00193	0.01466	0.00744	0.39780	0.04108	0.00921	3.09554	1.71917	1.79694	0.00000	0.72443	0.00000	0.01297
**Kifc1**	1.57609	2.22863	2.08508	3.65351	3.86716	6.41401	1.95222	5.75108	9.06730	3.67712	5.04435	0.34875	3.38038	0.04770	1.74761	0.09213
**Kifc2**	4.62471	0.05176	0.54146	5.90257	2.65431	0.09845	0.52738	2.51122	4.26746	2.60001	0.88802	0.16449	0.09613	0.00000	0.46060	1.03315
**Kifc3**	4.91254	0.00582	1.02191	2.48436	0.38880	0.02705	3.77530	19.19290	4.37791	13.09241	9.02445	4.53921	5.78161	0.35501	1.04522	2.45178
**Kifc5b**	1.03856	0.70514	1.44255	1.86576	1.37725	3.63975	1.60640	3.23165	6.04091	1.98865	2.30350	0.12445	1.67468	0.08823	1.32151	0.02423
**Kin**	0.00000	4.62038	5.49923	2.04886	4.41293	0.25559	5.20947	7.11735	5.21193	5.91768	0.45015	0.67723	0.48820	5.26086	6.94060	0.01711
**Kirrel**	0.00000	0.00731	0.00748	0.01110	0.00580	0.00625	0.00689	0.58509	0.01167	0.00000	0.00930	0.02547	0.00000	0.01726	0.00028	6.05103
**Kirrel2**	0.01311	0.00768	0.01966	0.00833	0.00981	0.00735	0.00748	0.01251	0.01183	0.27795	0.01273	0.00592	0.02725	0.00732	0.24351	0.00769
**Kiss1r**	0.01327	0.00811	0.01196	0.00960	0.00910	0.00652	0.00744	0.01217	0.02378	0.05903	0.01170	0.00789	0.11132	0.01158	0.01065	0.00711
**Kit**	1.26019	0.00522	0.00000	4.14896	0.01604	0.00390	2.73390	0.00134	2.79115	0.00000	0.04969	0.03662	0.00973	0.44721	8.16112	0.00498
**Kitl**	0.01375	0.00814	0.01231	1.25509	0.00931	0.00650	0.00747	0.01368	0.04064	0.01260	0.01374	0.00815	0.01263	0.00689	0.05348	0.00719
**Kiz**	0.97932	0.00246	0.97487	1.67274	5.23274	0.03133	1.20601	1.14231	3.95186	5.50955	1.84849	1.32914	1.44319	1.65095	3.28268	0.15554
**Kl**	0.02897	0.00774	0.05554	0.20293	0.07844	0.01405	0.01803	0.03686	1.12082	0.11484	0.24254	0.09188	0.08156	0.47190	0.41952	0.20162
**Klb**	0.57479	6.87926	4.61056	4.37361	0.03470	0.51241	3.48653	2.41257	7.42388	3.40599	3.06131	3.38943	0.11185	0.00000	3.65303	0.00750
**Klc1**	3.88493	0.00314	0.00983	1.57083	0.00000	0.01070	0.43527	0.84864	1.50066	1.09363	0.25696	0.06548	1.91636	0.00727	0.04290	2.27895
**Klc2**	0.01324	0.00796	0.01177	0.07079	0.00923	0.00654	0.00745	0.01244	0.43610	0.02446	0.02716	0.01616	0.01214	0.02140	0.06145	0.05862
**Klc3**	0.01206	0.00791	0.01216	0.00805	0.00889	0.00669	0.00740	0.01128	0.01197	0.67498	0.01193	0.02608	0.01360	0.01170	0.00832	0.18946
**Klc4**	4.21801	0.36375	1.24795	2.95186	0.04813	3.39019	0.01001	5.82998	0.23491	8.28902	3.74021	0.84932	3.71803	0.01547	0.47590	0.01280
**Klf1**	0.01375	0.00795	0.01237	0.12039	0.00931	0.00662	0.00752	0.01387	0.01046	0.01424	0.01387	0.00782	0.01265	0.00906	0.03253	0.00739
**Klf10**	1.67164	0.01550	1.10144	1.10945	0.08061	0.28745	2.62151	7.18706	0.09650	2.22945	5.63299	0.16280	2.24674	3.13042	1.26239	0.02515
**Klf11**	2.19525	0.00435	0.21823	7.44529	0.01078	0.04044	0.00324	4.06890	13.90020	9.57604	4.88797	0.59254	0.44030	0.00000	6.34701	1.51444
**Klf13**	5.98990	2.29419	4.47748	0.55955	2.15069	5.31276	1.34030	4.19923	4.00318	5.81312	5.28946	0.62845	2.31613	5.37684	3.82885	1.81673
**Klf15**	0.52614	0.00773	0.23775	0.00506	0.03583	4.33588	0.00643	0.00729	0.26432	0.00279	0.00697	0.00689	0.00000	0.01562	0.00408	0.00527
**Klf16**	2.61665	0.01572	2.61604	7.50956	0.03223	0.02727	0.00299	2.07623	11.23955	3.06063	3.92689	0.22369	0.92374	3.04628	6.75188	2.10314
**Klf17**	0.00000	0.00258	0.00000	25.41376	0.00735	0.00000	0.00000	3.90117	21.66711	31.73864	9.33571	11.35540	0.00000	24.89650	40.82393	0.00000
**Klf2**	5.50860	0.00000	0.32914	2.08005	0.00000	0.00000	0.95509	5.01547	13.42005	17.81908	14.50790	4.79971	1.10455	0.07703	1.37404	1.50004
**Klf3**	2.56479	2.78394	0.17392	1.70969	0.05294	0.00580	0.00387	0.75083	3.69122	0.93566	1.17319	0.03047	1.23146	0.00000	0.00000	2.92291
**Klf4**	18.21371	1.74529	3.95950	4.22969	7.75119	2.00739	3.06696	15.39786	7.93424	13.06190	8.73108	3.36040	7.47582	10.14990	6.32545	2.16763
**Klf5**	10.59863	0.00000	0.00000	0.09194	4.56910	6.34933	0.93177	10.12040	0.78334	29.45608	0.00000	0.42131	12.53285	0.23928	0.13197	11.04562
**Klf6**	5.57983	0.27947	1.12827	0.00014	3.26538	0.01038	0.09191	0.47951	0.00982	1.27950	0.00622	0.06870	0.01106	0.03340	0.00000	5.50046

**Klf7**	0.01184	0.00810	0.01328	0.00664	0.00996	0.00710	0.12454	0.01300	0.01168	0.86662	0.01214	0.01491	0.17314	0.00629	0.17242	0.00744
**Klf8**	1.93308	1.41214	3.01535	1.52004	4.47187	0.00394	0.00414	2.53082	0.53827	4.25133	1.74613	0.58204	0.32673	9.93213	0.06419	0.15330
**Klf9**	5.47203	4.47618	3.47524	6.21126	0.80032	4.98023	5.19290	5.81338	5.20145	4.87257	3.58403	0.15890	2.90295	5.27116	6.69819	3.72379
**Klhdc1**	0.01005	0.00745	0.00597	0.19500	0.00951	0.00634	0.00715	0.26258	0.26014	0.05195	0.00000	0.27260	0.00660	0.00595	1.88166	0.01459
**Klhdc10**	4.34349	1.94312	3.60399	6.10741	1.22019	0.00753	2.76835	3.54684	3.39958	7.00806	5.02246	6.30228	1.95441	3.69639	3.42243	1.10085
**Klhdc2**	4.02835	0.53288	0.87068	25.33538	10.68870	0.61351	0.01045	2.69213	11.36025	4.26614	3.86165	1.97226	2.04153	0.00000	8.02933	2.16368
**Klhdc3**	8.71099	3.28754	3.55375	2.74114	5.61103	6.27346	3.81513	2.75842	2.01993	3.63434	0.03192	0.30910	6.77258	0.00000	0.00000	9.03078
**Klhdc4**	8.64707	6.96233	13.20515	7.55486	10.35358	7.73266	4.49889	19.59310	8.72170	5.90365	7.78250	0.72631	12.73908	0.25355	5.20914	9.12804
**Klhdc9**	2.14225	0.52065	1.08880	0.07124	1.20425	1.55046	1.98259	0.52181	0.23091	2.95903	0.45531	0.13075	0.40998	4.30618	0.12932	1.10161
**Klhl10**	0.00000	0.09945	0.00000	0.90802	0.00799	0.00577	0.63850	2.04833	0.01516	7.11178	6.13419	0.42919	1.40869	2.66256	8.46657	0.00538
**Klhl11**	0.17995	0.75143	0.00000	18.78203	2.16752	0.00277	0.00485	3.16617	26.00001	1.84230	3.61958	0.21281	0.02546	0.85982	4.15736	0.00885
**Klhl12**	5.59179	4.37522	3.03745	7.65901	4.13273	0.01367	7.77215	4.21080	12.08246	11.78995	4.64387	3.08883	3.56989	11.72686	12.08863	1.58220
**Klhl13**	0.04321	0.00802	0.00903	0.68318	0.00891	0.00643	0.00729	0.01167	0.59077	0.00321	0.00756	0.01688	0.01280	0.00609	0.82053	0.00709
**Klhl15**	2.01246	4.21504	0.57929	6.65879	0.32918	2.43039	0.49868	1.68810	1.01885	3.13592	1.54361	1.87244	0.12188	0.62600	3.09795	1.50478
**Klhl17**	1.40250	0.00601	0.52507	0.22108	0.00678	0.00427	0.00618	1.27280	0.45875	0.77389	0.01282	0.00000	0.00313	0.03015	0.15571	0.11308
**Klhl18**	0.99761	0.92892	0.26350	4.13129	0.00567	0.00499	0.00581	1.79030	4.88818	2.04366	0.56960	0.09126	0.57329	0.50743	0.36521	0.00467
**Klhl2**	1.12299	0.00509	1.29882	2.27460	0.36528	0.00446	1.34342	4.96885	0.48007	1.58765	2.35732	0.06170	1.65892	1.84146	4.52362	0.21539
**Klhl20**	3.86504	1.02139	0.95728	0.92729	0.58553	2.22696	0.49401	0.41503	0.24827	1.02858	0.12728	0.02267	1.05711	0.84387	1.28798	1.85039
**Klhl21**	6.27902	1.24843	5.49106	9.02035	4.88941	3.51791	1.71151	8.55344	10.18575	8.25834	4.92088	2.03773	1.06700	7.47205	13.91974	0.03667
**Klhl22**	1.12735	0.01558	6.50479	4.56673	0.03466	0.00458	3.55601	1.45252	1.35538	3.41236	1.34933	0.05908	9.49318	0.00000	1.99801	0.04296
**Klhl23**	0.01230	0.00769	0.89034	0.58511	0.00769	0.96651	0.03719	0.00976	0.34765	0.00548	0.01782	0.01614	0.00530	0.01074	0.00000	0.00652
**Klhl24**	0.42021	0.00318	0.71744	0.88712	2.79261	0.00703	0.00542	0.02341	3.74252	1.40286	1.04392	7.75493	0.23998	0.55307	3.63355	2.00589
**Klhl25**	0.00000	0.05709	4.11169	1.07268	0.00230	3.61398	0.22958	0.00439	0.76995	2.17250	0.00000	0.03515	1.32148	0.00798	0.00000	0.01097
**Klhl26**	0.48792	0.01975	2.09625	0.45636	0.01417	0.00646	0.17285	0.01217	0.00361	0.32546	0.00933	0.03033	0.02203	0.00967	0.00000	0.08905
**Klhl28**	0.06891	0.00800	0.18287	0.22820	0.00812	0.00623	0.00779	0.37197	1.30493	0.26555	0.00000	0.00315	0.08934	0.00664	1.18123	0.00739
**Klhl3**	0.01344	0.00824	0.01357	0.00946	0.00972	0.00689	0.00769	0.01382	0.01233	0.25694	0.01284	0.00725	0.02934	0.01133	0.00996	0.00738
**Klhl30**	0.00850	0.00756	0.28950	0.44915	0.00920	0.00901	0.00706	0.53662	0.00308	0.00685	0.00830	0.03443	0.00443	0.01104	0.00000	0.00725
**Klhl32**	0.01375	0.00805	0.01225	0.01061	0.00932	0.00666	0.00754	0.01390	0.01235	0.01531	0.01310	0.00811	0.01270	0.01234	0.01062	0.00741
**Klhl35**	0.01351	0.00797	0.01228	0.14188	0.00922	0.00655	0.00734	0.01120	0.00926	0.50136	0.01316	0.00673	0.01189	0.01011	0.01614	0.00742
**Klhl36**	0.82199	0.00747	0.04053	0.27594	0.00767	0.00507	0.00675	0.00963	0.04287	0.27914	0.10045	0.00630	0.33926	0.01113	0.00558	0.02863
**Klhl41**	1.23394	0.20407	3.50824	0.20727	0.67174	0.25995	0.40964	0.00000	2.65355	0.81399	9.58582	0.72777	3.65271	1.53605	0.85489	0.73573
**Klhl42**	0.15226	1.79748	1.26370	1.00702	1.87215	0.00519	1.07067	0.00000	1.21310	0.70419	0.57063	0.02953	0.58181	0.01239	0.31822	0.00543
**Klhl5**	0.00830	0.00742	0.00676	0.00610	0.00903	0.00698	0.00716	0.97258	0.01033	1.60738	0.08806	0.02911	0.00477	0.01415	0.05665	0.00708
**Klhl6**	0.01375	0.00791	0.01194	0.29820	0.00933	0.00670	0.00754	0.01372	0.10410	0.01820	0.01156	0.00803	0.01293	0.01668	0.04024	0.00736
**Klhl7**	0.89072	1.15799	0.80665	4.96454	0.27075	1.20755	3.53918	0.29604	5.74658	1.02888	5.64427	3.71638	0.31758	3.25798	2.06677	0.14306
**Klhl8**	0.95578	0.00702	0.67985	19.02848	0.01274	0.00201	0.00546	0.00596	8.46697	1.73887	0.59616	0.29306	0.00449	0.00610	9.42758	0.00224
**Klhl9**	0.85076	0.05722	1.13318	0.61782	0.00519	2.18338	0.03564	0.00000	0.51721	0.63596	1.65102	0.04111	0.74994	0.41911	2.63965	0.41236
**Klk1b24**	0.01038	0.00853	0.00910	0.00766	0.00966	0.00685	0.00814	0.17117	0.02629	0.06378	0.07118	0.00786	0.00720	0.01320	0.00587	0.00743
**Klrb1a**	0.01291	0.00778	0.02396	0.01006	0.00914	0.00764	0.01744	0.01672	0.01231	0.11290	0.01300	0.00710	0.01897	0.02881	0.00798	0.00774
**Klrg2**	1.19354	1.91184	0.06185	9.84977	2.98420	0.00271	5.35357	1.82187	7.39879	7.44685	3.86183	0.47026	0.12023	0.52007	7.35987	0.20952
**Klri2**	0.01331	0.00782	0.01784	0.10286	0.00928	0.00728	0.04868	0.01279	0.15806	0.01972	0.01118	0.00768	0.01240	0.01512	0.03674	0.00734
**Kmt2a**	1.92372	0.01601	0.00000	0.04091	0.01367	0.82462	0.00467	0.00257	0.58110	0.20432	0.00752	0.02280	1.96212	0.00754	0.05084	1.87329
**Kmt2b**	6.92413	0.12870	5.21416	12.08647	0.00000	3.98217	3.73259	5.78309	2.54420	3.50453	1.99001	5.23087	4.92081	0.00000	0.70314	3.26297
**Kmt2c**	1.10718	2.84672	5.59971	10.75836	0.27545	4.02162	3.67176	8.50327	12.12664	12.50792	9.60645	18.16377	1.30712	21.32999	18.52869	2.79833
**Kmt2d**	3.83538	0.18987	1.92260	5.35428	0.24066	1.03742	0.00203	0.59298	5.75770	0.65916	0.63916	0.02932	3.04472	1.51929	0.22563	2.59145
**Kmt2e**	0.82871	2.88833	0.00985	0.00000	0.01303	0.00717	0.00567	0.59336	0.17594	0.78817	0.10999	0.02283	0.33499	4.15744	0.23120	0.00539
**Kmt5a**	11.11852	6.41097	9.94519	9.87380	10.43570	4.54955	9.69828	19.32396	11.60434	17.40635	12.04732	2.01729	8.76128	4.42392	6.89418	3.34679
**Kncn**	0.01331	0.00839	0.01175	0.00933	0.00933	0.00653	0.00770	0.02124	0.01188	0.01880	0.01242	0.00846	0.02476	0.00753	0.17365	0.00737
**Kndc1**	1.76337	0.00643	0.02395	0.78171	0.00701	0.00411	0.00619	0.00835	0.57660	0.04547	0.00956	0.00664	0.01721	0.01325	0.00262	0.01252
**Kng1**	0.00000	5.20091	5.04023	0.00000	0.00704	0.01970	9.92907	7.33188	0.64646	1.30193	3.46525	6.61247	4.86991	0.00000	1.10235	0.00675
**Kng2**	0.00000	0.61256	1.13388	0.05039	0.00594	0.00703	2.25342	1.80077	0.35425	0.22774	0.95331	0.92392	1.45209	0.06922	0.00023	0.00552
**Knop1**	14.44511	7.06361	14.71457	8.81896	9.26179	7.71278	10.43124	11.42815	15.86987	19.85881	12.32435	8.06958	10.47230	21.88315	16.71190	3.20964
**Knstrn**	7.83950	0.00669	1.90530	15.84865	5.39540	5.08317	0.00000	3.95726	4.45478	5.89833	2.31766	0.13636	1.81945	2.59119	1.62885	4.03823
**Kntc1**	2.35828	0.11632	4.83816	5.76377	5.14978	2.45936	4.89076	1.72209	8.69267	3.02354	2.60577	4.14194	0.97097	4.75021	6.18475	1.65101
**Kpna1**	3.70969	10.36553	9.43168	13.27572	7.76123	1.16650	0.55060	8.25018	12.98857	16.60760	8.83149	6.74227	6.44164	32.54362	20.76141	3.13398
**Kpna2**	24.54105	22.27845	24.28693	20.83171	14.82530	10.16826	13.11871	38.48489	27.18217	38.19802	25.16210	25.33314	16.31312	43.89174	39.22468	6.72913
**Kpna3**	5.73935	5.27543	4.70639	9.57117	7.86772	1.32710	2.95397	2.94060	3.43825	2.13101	1.29579	8.05325	6.89506	7.10590	13.43633	2.06293
**Kpna4**	1.49330	4.10128	0.44901	6.53278	4.12999	2.26532	1.83240	1.66921	10.61661	4.69767	4.75673	5.80815	1.96990	10.42392	2.66696	0.63281
**Kpna6**	9.88190	5.97057	6.97945	7.55865	7.72143	7.66413	7.62882	11.85445	13.02911	7.81360	3.20617	0.67990	5.67093	5.69251	8.60673	7.63684
**Kpna7**	0.01169	0.11247	0.01104	2.65565	0.00887	0.00640	0.00732	0.01167	0.00000	0.02842	0.02349	0.02695	0.01185	0.00197	0.13237	0.00675
**Kpnb1**	25.75640	18.66526	20.54634	17.44485	21.02657	11.73475	17.56101	25.53772	18.78628	23.99662	16.09363	8.30476	22.08069	28.10607	22.93652	13.81247
**Kptn**	3.55387	0.09338	0.10832	0.12290	0.07205	0.00326	3.20291	6.32865	0.02628	4.45444	2.03213	0.05529	2.29918	0.13964	0.00000	3.29815

**Kras**	3.35892	2.39909	0.49008	2.72960	3.33902	2.37570	2.20964	0.63344	1.51665	3.48585	2.40167	12.61840	0.59970	3.76780	4.40742	0.09976
**Krba1**	0.07312	0.00878	0.91870	0.57399	0.01366	0.00744	0.00610	0.41256	0.00000	0.00000	1.21503	0.00879	2.58431	0.00381	0.83433	0.04915
**Krcc1**	1.94822	0.11753	0.70553	3.29022	3.69967	7.71956	1.70200	1.29082	0.00000	2.83696	0.00000	1.05872	0.69367	5.47779	1.47283	0.00823
**Kremen1**	0.29485	0.00830	0.52323	2.53958	0.00899	0.00922	0.00687	0.00000	0.60945	1.40591	0.62064	0.08385	0.31037	0.10748	0.18016	3.22462
**Kremen2**	0.11205	0.08861	0.33224	0.11146	0.01962	5.25603	0.16018	0.75683	0.36337	4.50018	2.01745	0.16376	5.79671	1.08155	0.17645	0.07517
**Kri1**	3.02387	3.88094	2.59662	0.00000	2.33336	2.48849	2.56991	1.23145	0.14359	2.49005	0.02875	0.05203	2.04357	2.22762	3.56780	3.15789
**Krit1**	0.45137	3.67560	1.28874	0.90688	0.00429	0.15955	3.03253	1.50775	0.74136	3.15160	1.34293	15.18303	1.77801	7.94721	3.31293	1.74222
**Krr1**	7.08061	4.78988	3.96662	8.12345	3.88915	3.03025	5.19826	10.22142	12.28310	9.38052	6.98606	13.12421	3.05439	24.10702	13.82308	3.41732
**Krt10**	0.12631	0.00794	0.30181	0.74174	0.03324	0.00786	0.00640	0.02387	0.43509	0.48545	0.54939	0.02078	0.78407	0.00410	0.02723	0.00684
**Krt12**	0.01377	0.00810	0.01219	1.35856	0.00936	0.00650	0.00745	0.01331	0.00000	0.01096	0.01254	0.00820	0.01268	0.00771	0.10073	0.00717
**Krt18**	0.52551	0.11232	6.39765	0.00000	0.85786	30.35846	3.59175	6.88247	0.59928	11.07762	6.30096	0.00000	30.57578	0.08994	0.00000	21.03458
**Krt19**	0.01087	0.00741	0.16621	0.00494	0.00794	0.00887	0.00695	0.00000	0.06104	6.04983	0.41508	0.00000	0.00761	0.01208	0.00000	0.00730
**Krt222**	0.00101	0.01980	0.04190	0.12557	0.04534	0.00847	0.00811	0.53863	0.30113	0.76529	0.07088	0.01067	0.00595	1.26083	0.52701	0.00768
**Krt25**	0.00982	0.00806	0.06487	0.00809	0.00927	0.00860	0.00754	0.10687	0.01180	0.33914	0.05617	0.00734	0.02448	0.01472	0.00683	0.00725
**Krt27**	0.01163	0.00794	1.12553	0.00858	0.00795	0.00953	0.00746	0.01164	0.01225	0.45380	0.01091	0.08133	0.04442	0.00920	0.01906	0.00707
**Krt28**	0.01114	0.00721	0.00000	0.79734	0.00844	0.00825	0.00644	2.75455	5.56771	7.12621	1.90399	0.25195	0.64610	0.21312	0.03599	0.00616
**Krt7**	0.03441	0.00771	0.00268	0.00479	0.01021	0.00806	0.00616	0.65383	0.19008	0.16159	0.71525	0.01232	1.37046	0.00670	0.01460	0.00676
**Krt8**	5.02824	8.20894	11.62984	1.41454	8.19874	28.17821	5.58374	5.46887	0.74121	0.63948	0.04655	0.02402	27.54653	0.00000	0.00000	23.78959
**Krt84**	0.01360	0.00787	0.01167	1.23490	0.00925	0.00640	0.00746	0.01246	1.23373	0.00360	0.03858	0.00528	0.01206	0.00441	0.10124	0.00706
**Krtcap2**	5.37073	0.38789	6.13815	0.53850	5.36819	3.16130	2.03884	2.80457	0.62756	5.99856	9.04470	0.39330	8.98722	0.06121	0.00000	0.70627
**Krtcap3**	1.03891	0.01639	0.07677	2.41300	0.00561	0.00785	0.00509	0.01779	0.00000	1.18767	1.37446	0.01446	1.10636	0.00000	0.29685	0.00538
**Krtdap**	0.01001	0.00774	0.04096	0.01058	0.00921	0.20459	0.00702	0.07012	0.01234	0.00694	0.01192	0.00742	0.29629	0.01299	0.00879	0.00704
**Ksr1**	0.59409	0.00617	0.00172	0.21092	0.00709	0.00846	1.52267	0.00839	0.00545	0.00892	0.01348	0.01640	0.02848	0.00977	0.06100	0.00688
**Kti12**	2.75221	1.76638	0.46998	11.13117	3.79339	2.24116	1.68222	7.16214	16.98836	14.74433	5.76688	2.06931	3.17305	2.32413	5.68310	1.25852
**Ktn1**	1.15408	1.85821	4.76916	8.67093	6.22846	4.80942	7.39359	3.69966	2.17678	1.06067	2.20387	0.74538	2.59116	8.27475	12.69621	4.32784
**Kxd1**	16.26531	8.74301	12.64825	1.91159	1.42878	9.18778	9.16897	15.90158	2.53896	14.51262	13.15011	6.66894	13.89996	2.47538	5.16393	7.16977
**Kyat1**	3.70975	0.00301	0.52670	1.72961	0.53098	0.00256	0.01341	1.52058	0.80096	5.37229	1.15219	1.13191	0.51895	0.01857	1.84118	1.06327
**Kyat3**	11.55664	7.31677	7.88077	4.99189	13.85245	0.00000	6.94201	0.88413	11.02157	1.91261	0.53053	0.05349	7.11716	0.03573	4.26829	2.18579
**L1cam**	0.02013	0.00828	0.13798	3.09397	0.00941	0.05936	0.00719	0.08082	2.39985	0.75920	0.76226	0.04781	0.89833	0.00000	1.64685	0.00622
**L1td1**	20.68785	14.49730	15.43223	13.75060	19.91244	9.89403	13.68757	2.14139	19.90382	0.00128	1.57314	0.21998	4.04686	5.76219	0.51330	12.95121
**L2hgdh**	5.34648	2.42986	1.60327	11.93557	0.62641	1.79100	1.65097	5.87472	8.30735	13.17954	12.99973	18.85973	7.53156	22.37797	18.34733	0.51513
**L3hypdh**	1.04889	0.00694	0.00379	0.07198	0.01144	0.00684	0.00656	0.00829	0.01130	0.05543	0.01284	0.01539	0.01207	0.00570	0.21047	0.00677
**L3mbtl2**	5.53298	4.62986	6.10062	13.05524	5.25841	0.87102	5.71898	10.24340	17.36848	10.53637	5.50231	0.70951	5.15169	0.18021	1.65364	10.63476
**L3mbtl3**	0.00000	0.14450	0.90514	0.29645	0.00675	0.00565	1.01347	0.15078	0.00408	0.16984	0.10832	0.05729	0.11323	0.00587	1.21945	0.74646
**l7Rn6**	19.43614	5.37831	12.82715	5.62647	15.39658	8.54999	4.72544	12.23352	3.67716	15.23252	8.81577	3.57814	13.98080	2.06962	0.00000	4.04714
**Lacc1**	0.01033	0.00739	0.00750	0.08431	0.00974	0.00673	1.00468	0.02904	0.00807	0.00684	0.01003	0.01399	0.01014	0.52494	0.00002	0.00740
**Lace1**	0.00536	2.26218	0.57018	2.43685	0.00754	0.00709	0.00645	0.00000	0.00000	0.21760	0.75061	0.01403	0.17385	0.00000	0.16579	0.00606
**Lactb**	3.06428	0.00213	3.53039	2.85191	0.00000	6.47252	2.08174	1.30249	4.55050	3.61640	2.69509	0.76561	3.06564	5.29357	3.62461	0.02660
**Lactb2**	7.02911	2.24108	3.06353	2.53290	9.55584	3.98875	5.01013	5.54359	3.76920	4.15243	12.45273	6.13842	1.51510	3.58750	2.76979	0.34560
**Lad1**	0.54254	0.00677	0.00000	0.00000	0.00664	2.90326	0.00530	3.18866	0.00846	0.53983	3.39422	0.00419	1.38183	0.00865	0.00000	0.00447
**Lag3**	0.00710	0.00698	0.00539	0.01477	1.31489	0.01373	0.00729	0.01351	0.03513	0.25470	0.02734	0.01493	0.01705	0.06315	0.01229	0.00746
**Lage3**	10.50733	2.82676	4.58813	8.22459	1.48892	0.01846	4.47148	5.43682	1.17245	5.98857	3.80452	0.37915	3.91288	3.19759	3.55627	5.14182
**Lama1**	7.28020	2.56380	0.55706	0.00674	0.02171	0.01014	0.00000	0.01664	0.01285	0.00113	0.01085	0.00000	0.00460	0.02409	0.18760	23.16235
**Lama2**	0.01274	0.00770	0.04263	1.18266	0.00942	0.00578	0.00738	0.01086	0.00000	0.00463	0.00953	0.00445	0.01210	0.00821	1.67441	0.00711
**Lama3**	2.26882	2.04440	0.09086	2.74394	2.84722	0.01552	0.92525	0.00000	0.16069	0.00000	0.23672	0.00925	0.90634	0.00000	0.00000	0.00322
**Lama4**	0.01623	0.00766	0.96638	0.93811	0.00736	0.00552	0.00729	0.01024	0.00000	0.42052	0.00827	0.03435	0.00841	0.00413	1.11322	0.00686
**Lama5**	5.50232	1.67893	1.83085	0.48815	6.44929	8.91466	2.19857	7.29322	0.00000	1.15469	4.49478	0.10765	5.06486	0.00000	0.00000	11.74659
**Lamb1**	14.35090	2.15577	10.49768	3.03721	4.21937	0.89284	14.56411	2.35915	0.07189	1.61703	1.85374	0.65706	5.02721	4.84813	3.18666	18.12575
**Lamb2**	0.00000	0.00727	1.56804	0.65669	0.00604	0.36612	0.00429	0.82062	0.98454	1.09035	0.84959	0.05090	5.11210	0.00308	0.00000	0.01186
**Lamc1**	5.05592	0.01561	1.84425	2.74298	4.15109	9.23071	6.12284	1.29236	0.46580	0.00000	0.00000	4.72076	3.11496	0.00000	0.29016	8.39794
**Lamc2**	0.74994	0.00759	0.00000	0.53508	0.00687	0.00660	0.03782	0.00000	0.00000	0.12844	0.59453	0.04886	0.45930	0.47580	2.01761	0.01344
**Lamc3**	0.01307	0.00774	0.00931	1.41811	0.00899	0.00639	0.00717	0.00919	0.75969	1.05067	0.03951	0.10840	0.01122	0.01896	0.46741	0.00735
**Lamp1**	28.19441	16.33826	28.54420	8.11297	12.20633	15.30827	19.20490	27.85608	0.93880	24.85612	25.51809	9.91344	28.41137	2.29464	13.14903	9.59593
**Lamp2**	4.32860	4.22875	8.33862	16.80795	1.20729	2.48440	8.07296	9.39103	20.03905	19.94973	6.34629	16.66820	8.59857	30.81769	17.31355	0.00783
**Lamp3**	1.51989	1.35668	1.69361	0.92129	2.27551	0.63693	1.00489	1.16650	1.21798	0.74805	0.44090	0.97794	0.66065	0.75603	0.69879	0.76969
**Lamtor1**	16.26145	4.49562	19.84285	9.29718	12.87992	9.78273	2.22499	16.35883	6.73306	17.16541	10.72564	0.83275	14.58216	2.99455	2.80611	5.74512
**Lamtor2**	4.00008	0.27396	1.52389	2.92018	1.17258	0.33033	0.76688	0.66425	0.62303	1.28937	0.88769	0.07805	2.28832	0.20088	0.87552	0.02947
**Lamtor3**	1.72032	6.33346	9.52815	3.65891	7.72086	6.69428	0.66534	6.15268	3.53515	6.80645	3.37185	6.23494	5.92382	0.64187	5.74188	4.13870
**Lamtor4**	3.13240	0.84038	1.80387	0.50707	2.97398	2.65113	1.28193	2.56970	0.68353	2.27113	2.56842	0.06018	4.32284	0.09059	0.43554	2.70887
**Lamtor5**	17.16808	10.57014	6.47436	2.60352	12.54023	3.18478	5.24709	2.42720	0.00000	6.98909	3.41460	1.32937	3.68452	1.80294	0.74945	7.76914
**Lancl1**	5.68699	1.97839	4.44388	1.12716	6.56728	0.62923	7.72896	1.93178	1.47625	2.85816	1.85509	0.09362	3.17030	1.50937	0.96499	3.62549
**Lancl2**	3.07416	1.69285	0.00000	0.70952	0.00364	2.47259	0.00406	0.00785	0.00000	1.25299	0.11753	0.00000	0.01532	0.00297	0.36096	1.60425

**Lap3**	24.41827	15.40590	16.59601	5.56695	20.25417	14.22556	15.15048	16.02182	6.58010	8.51565	6.03659	3.58990	15.32630	0.78012	8.68750	15.11745
**Laptm4a**	0.76420	3.29463	4.95269	8.02883	6.61589	4.90293	4.64301	9.68715	5.81215	2.28869	13.06205	0.51087	5.74617	1.93393	2.10307	4.53736
**Laptm4b**	5.06160	3.66036	2.99034	22.10353	0.01060	3.37644	2.61095	0.27534	14.81584	7.32128	0.49604	0.35823	7.96815	3.93999	7.55442	1.53617
**Laptm5**	0.01724	0.00828	0.01143	0.42241	0.01004	0.00667	0.00734	0.01083	0.37667	1.07889	0.00976	0.00523	0.01829	0.03202	0.00274	0.00740
**Large**	0.82043	0.00630	1.04248	0.54278	0.00468	0.08827	0.00634	0.91712	0.00014	0.00199	0.00552	0.02526	0.00000	0.00656	0.46953	0.00586
**Larp1**	18.01568	8.36699	9.79652	14.69918	10.84213	7.42619	11.01420	19.14982	17.83724	23.47680	21.93771	3.42767	14.11687	8.82292	16.84848	9.23349
**Larp1b**	0.00000	2.33581	1.04872	29.50454	2.61435	3.97207	0.57398	9.30122	31.64384	35.08275	16.34631	20.10551	8.76972	19.25768	32.59999	0.03937
**Larp4**	4.04907	6.70085	4.04331	18.37414	1.72167	3.07442	2.50002	19.56735	22.66685	17.16120	34.48672	27.72624	6.57027	48.67540	26.46372	1.66519
**Larp4b**	11.78702	7.22391	9.60228	11.14842	7.56961	9.65978	5.89724	10.71973	12.99789	12.25912	7.37560	5.89445	6.82781	17.16991	15.03570	6.03837
**Larp7**	16.45040	13.47188	12.09730	8.48875	12.59848	6.77646	10.19080	14.08145	9.49713	15.74835	8.69161	2.97818	6.06283	12.52590	16.17397	6.35733
**Lars**	7.92128	9.15065	5.44405	0.14173	16.83751	3.52537	9.02440	8.64151	0.00000	10.08979	1.88164	1.22894	6.63840	6.86177	5.03460	1.78917
**Lars2**	3.47572	2.20545	0.23412	1.86660	4.64614	3.49535	0.00409	0.10819	0.82727	0.39819	0.45805	0.10208	0.39766	1.37619	1.07622	1.07697
**Las1l**	11.74562	4.41342	10.29902	7.94287	3.54410	6.36486	6.07678	3.55703	19.49242	8.97464	8.07175	1.26630	7.72260	6.76369	8.79801	3.80092
**Lasp1**	11.21760	9.04719	10.40690	0.04563	15.03291	7.83993	7.44867	17.85141	0.74411	11.40531	16.28352	0.91182	12.83324	1.11645	0.00000	8.47373
**Lat**	0.01418	0.07147	0.48224	0.82654	0.00821	0.00704	1.13023	0.00000	0.69862	2.76674	1.54373	0.20927	0.05494	0.01052	0.68207	0.02199
**Lat2**	0.00000	0.00676	1.35487	0.92814	0.00681	0.01227	0.00462	5.15764	1.82265	0.74874	3.62699	0.00000	2.04655	0.19766	0.76078	0.01267
**Lats1**	0.00000	0.00313	0.51061	2.39156	0.81255	0.14201	1.42474	1.22798	3.58056	3.10264	1.33027	10.60658	0.00000	7.60122	6.67801	2.71149
**Lats2**	0.00000	1.35844	1.20312	1.06199	0.00643	2.30121	0.00182	3.92855	3.59274	3.72686	6.78419	10.03138	1.52213	1.11379	1.86707	0.01032
**Lbh**	0.12099	0.00489	0.01498	0.00000	5.27829	0.00852	0.00707	0.00000	3.01933	0.74480	0.07792	0.03643	0.01649	0.01071	0.00713	0.00706
**Lbhd1**	0.00952	0.00866	0.00507	0.00000	0.00952	0.00628	0.00813	0.41071	0.00692	0.51591	0.00038	0.10382	0.00618	0.01616	3.53961	0.00739
**Lbp**	0.01212	0.00812	0.01654	0.96255	0.00871	0.01228	0.00732	0.00982	0.84701	2.54045	0.01554	0.00000	0.01093	0.00901	0.00723	0.00717
**Lbr**	5.83232	2.08141	2.89025	11.41643	3.05859	8.32314	5.08262	12.54060	11.91316	12.19702	13.54954	14.59082	3.50749	21.10878	14.41954	0.06881
**Lbx2**	0.01289	0.00819	0.01036	1.05360	0.00891	0.00632	0.00738	0.00822	3.48613	0.82793	0.08368	0.00000	0.01069	0.00842	0.00119	0.00693
**Lca5**	0.01309	0.00776	0.01176	0.19032	0.00915	0.00671	0.00738	0.02090	0.00586	0.41953	0.02202	0.00628	0.01173	0.00538	0.20955	0.00757
**Lca5l**	0.01371	0.00820	0.01195	0.76050	0.00930	0.00657	0.00749	0.01362	0.39800	0.01264	0.01155	0.01844	0.01278	0.01114	0.00285	0.00727
**Lcat**	0.00941	0.00756	0.00592	0.00682	0.00959	0.00701	0.00702	1.63935	0.00998	0.00000	0.12258	0.03407	0.00460	0.00609	0.15100	0.00721
**Lck**	0.67506	0.00307	1.17393	1.55828	3.43687	0.00549	3.09062	3.21683	5.43799	2.90216	0.40885	0.12157	0.00000	0.87136	2.70066	0.01253
**Lclat1**	0.00000	0.42308	4.99658	14.87039	0.01676	0.10376	3.88569	5.23267	11.59134	14.08024	11.66477	12.93954	3.75530	13.62944	16.75963	1.45913
**Lcmt1**	6.98106	3.05830	7.26554	1.49478	7.30295	4.89389	1.86394	2.44205	0.00000	2.35584	1.43594	0.18945	4.22390	0.15630	0.49355	0.03619
**Lcmt2**	2.21799	1.98028	1.08594	9.20409	0.79359	0.07551	1.17288	1.22738	11.94502	4.13859	9.87232	0.82443	0.46517	4.22356	2.58104	0.33985
**Lcn10**	0.02390	0.01924	0.00641	0.00885	0.01199	3.26775	0.00731	0.01144	0.01283	0.00516	0.01055	0.00801	0.02384	0.01304	0.00993	0.00637
**Lcn2**	0.01043	0.00829	0.00987	0.08926	0.00846	0.00669	0.00771	0.02019	0.00759	4.60321	0.00565	0.00000	0.00991	0.36228	0.00000	0.00726
**Lcn9**	0.00762	0.00846	0.74280	0.52986	0.00854	0.00928	0.00810	0.45107	0.49346	0.00668	0.00601	0.00677	0.00250	0.01241	0.00000	0.00702
**Lcor**	0.35736	0.00688	0.00142	1.06765	0.65612	0.00825	0.00714	0.00853	0.00000	0.45864	0.00891	0.00305	0.28730	0.77336	0.17257	0.00697
**Lcorl**	0.01109	0.00727	0.56484	0.03144	0.12189	0.00550	0.00704	0.50820	0.01540	0.43836	0.00000	1.18176	0.20996	0.00703	0.69967	0.00734
**Lcp1**	5.74896	5.61515	1.23529	0.50300	1.33824	14.49863	5.47007	0.09867	0.96616	0.68981	0.09459	0.02551	0.08457	0.00248	0.00000	8.19690
**Lct**	0.00510	0.00820	0.44640	0.68174	0.00805	0.80314	0.00670	0.01722	1.50212	0.49941	0.06284	0.03345	1.75016	0.00314	0.41584	0.00573
**Lctl**	0.00000	1.95053	2.15766	1.26362	0.00606	0.01486	0.03510	4.97799	0.76063	1.65713	1.54113	4.40604	0.32742	2.02205	2.38171	0.00489
**Ldah**	5.98599	6.23125	9.92082	1.02556	6.72528	0.64884	2.24234	10.75553	1.63030	11.09916	3.99247	2.07236	5.23429	14.33530	4.97261	1.87574
**Ldb1**	1.35950	1.97005	0.91688	1.03738	2.31656	1.43564	1.94183	4.03284	1.65408	0.84175	5.03684	0.63239	0.36571	0.20167	0.58373	1.13812
**Ldha**	16.89623	13.81224	10.60901	6.94016	12.82024	4.08920	0.66207	3.13324	5.28874	6.96748	1.32795	8.78435	4.99453	0.00000	1.43530	6.20953
**Ldhb**	0.00000	0.00665	0.86370	22.53793	0.01684	0.00233	0.00240	0.77634	13.34882	11.28856	5.01758	1.98615	3.43953	7.52104	17.57153	0.00000
**Ldhc**	0.02312	0.00802	0.00763	3.68359	0.00930	0.00622	0.00745	0.01178	3.96260	0.25287	0.00000	0.07528	0.19956	0.01562	2.77028	0.00657
**Ldhd**	0.19687	0.00771	0.00645	0.15077	0.00742	0.00711	0.00714	0.01140	0.46598	0.52437	0.00863	0.03518	0.01220	0.01094	0.00588	0.00741
**Ldlr**	0.80509	0.00317	3.51263	0.21382	0.01027	2.06248	4.33096	2.70080	1.89524	0.52764	4.55498	0.06025	0.00000	1.50914	0.34909	0.04064
**Ldlrad2**	0.22788	0.07252	0.63372	0.14743	0.19998	0.05521	0.12036	0.01199	0.12848	0.18936	1.42674	0.10745	0.71377	0.46137	0.15893	0.12928
**Ldlrad3**	0.01346	0.00782	0.01178	0.18241	0.00907	0.00664	0.01765	0.01203	0.03680	0.47929	0.02692	0.00613	0.01190	0.01260	0.01401	0.00776
**Ldlrad4**	0.01379	0.00786	0.01176	0.67044	0.00939	0.00650	0.00740	0.01216	0.00000	0.00659	0.01300	0.00736	0.01279	0.02391	0.27132	0.00728
**Ldlrap1**	3.87032	0.00000	3.80493	3.11703	3.15422	3.85017	4.94102	6.67028	4.02661	5.58476	4.34262	4.41793	4.30278	0.02310	2.36577	0.01862
**Ldoc1**	0.02061	0.00777	0.01431	0.00562	0.01982	5.85958	0.00660	0.00813	0.01314	0.06735	0.00721	0.00657	0.58917	0.00522	0.21660	0.00482
**Ldoc1l**	0.14030	0.00748	0.01925	1.58581	0.00787	0.00846	0.02750	1.68800	0.36825	0.21502	0.37264	0.07221	0.22799	0.00141	1.86743	0.00693
**Lect1**	0.00864	0.00838	0.00728	0.24784	0.01023	0.00757	0.00802	0.21532	0.25494	0.51866	0.46163	0.01385	0.22900	0.00747	0.00000	0.01520
**Lef1**	0.01376	0.00795	0.01172	0.28907	0.00940	0.00657	0.00737	0.01186	0.00593	0.11779	0.02618	0.03639	0.01236	0.01043	0.00421	0.00742
**Lekr1**	0.01332	0.00762	1.51063	0.00996	0.00646	0.00658	0.00711	0.00806	0.57990	0.69586	0.02111	0.00380	0.00670	0.01368	0.00000	0.00648
**Lemd1**	0.01337	0.00760	0.00964	0.00855	0.00952	0.00661	0.00729	0.01066	0.01188	0.00324	0.00943	0.00531	0.01330	0.38171	0.35711	0.00748
**Lemd2**	0.42532	0.33936	0.39965	0.00000	2.32709	1.70236	1.32470	2.54191	0.72854	2.19690	0.46838	0.00000	2.37060	0.00716	0.00478	5.10643
**Lemd3**	0.00907	0.05552	0.07692	7.67815	0.00891	0.00576	0.00503	0.92900	6.35783	0.00000	1.20131	8.78256	0.00000	8.52874	1.49162	0.00471
**Lenep**	0.18344	1.19135	0.30487	0.46733	0.01490	0.00822	1.40590	0.03581	0.23047	0.31826	0.73260	0.02234	0.09732	0.00375	0.06482	0.00623
**Leng1**	1.38124	0.00346	1.48197	7.74386	2.55096	2.60453	0.24013	6.22154	24.08429	4.36856	2.36671	0.32198	0.00000	0.07513	0.56145	4.06394
**Leng8**	1.34825	0.00572	0.73895	5.86481	0.10478	1.00741	0.01436	1.55196	4.70021	3.52017	1.23484	0.15861	2.18385	0.37915	0.00000	0.23297
**Leng9**	0.01326	0.00828	0.01671	0.26161	0.00934	0.00631	0.00752	0.01288	0.44021	0.01754	0.04583	0.00724	0.02796	0.03362	0.00114	0.01469
**Leo1**	10.59517	8.33213	6.10644	10.59480	10.03968	3.36123	7.85591	15.40992	20.57503	17.57088	11.97444	11.42091	10.10654	3.86324	3.58653	5.57590

**Lepr**	0.45362	0.18521	0.15447	5.39135	0.22773	0.11018	0.11742	0.27062	10.48576	0.00000	4.27878	0.32201	0.20729	2.46140	1.50475	0.09672
**Leprot**	6.01475	0.01888	3.87859	0.00000	0.10370	4.20639	7.10200	0.67608	0.53738	3.14148	0.50976	0.05982	4.24241	0.95617	1.54474	6.10194
**Leprotl1**	1.95826	0.09159	2.67663	0.16758	0.34406	0.00457	0.18936	1.21261	1.74660	2.10354	1.56580	0.11388	1.05059	1.34092	1.28588	1.85845
**Letm1**	9.00370	0.22500	7.10071	5.54587	7.15938	5.19084	8.68358	10.76625	4.40938	3.59086	4.29100	0.74533	10.03018	1.11629	4.37723	0.09443
**Letm2**	0.00577	0.00755	0.00934	2.21219	0.00893	0.00580	0.00653	0.34522	5.18233	0.84088	0.00000	0.03277	1.19993	0.00000	0.48723	0.00605
**Letmd1**	0.64156	2.84303	0.59301	0.16223	0.01606	0.00535	3.16342	0.00000	0.02591	6.21255	2.13060	0.18008	2.03983	0.00734	1.49731	0.00404
**Lexm**	0.01064	0.00775	0.01355	0.28468	0.00937	0.00737	0.00715	0.56100	0.00679	0.11215	0.00807	0.00573	0.00558	0.00577	0.35021	0.00722
**Lgals1**	0.00000	0.37839	0.49137	3.89261	0.65973	3.39607	0.00500	0.00000	0.61809	2.92031	2.18316	0.10509	1.40742	0.00000	0.67531	3.76455
**Lgals12**	0.00943	0.00753	0.01066	1.02115	0.00980	2.44724	0.00685	0.60083	0.00000	0.00001	0.17124	0.00546	0.00000	0.00760	0.00000	0.00639
**Lgals2**	0.00790	0.00763	0.42082	0.97068	0.00889	0.00526	0.00725	0.44339	2.02170	0.00000	0.00000	0.01376	0.10919	0.02464	0.09177	0.02094
**Lgals3**	2.88275	0.12694	1.76479	1.29613	0.02154	6.13431	0.01389	0.04287	3.52575	4.96148	2.65882	0.15860	0.75955	0.06700	0.41139	0.03003
**Lgals3bp**	0.01327	0.00792	0.01216	0.00739	0.00896	0.00657	0.00734	0.01048	0.01184	0.95010	0.01137	0.00543	0.01106	0.01169	0.00868	0.00738
**Lgals4**	0.00901	0.00768	0.24011	0.00942	0.00821	2.37807	0.00689	0.01414	0.01266	0.00266	0.00977	0.00754	0.31186	0.01491	0.00571	0.27679
**Lgals7**	0.00689	0.06897	0.01148	0.00973	0.00914	0.00646	0.33847	0.09943	0.01219	0.16634	0.01246	0.00666	0.43463	0.01335	0.00647	0.00709
**Lgals8**	6.45831	8.12229	6.95419	0.54432	5.62754	8.17690	6.82700	11.21823	0.92672	8.59690	19.97254	6.41095	5.28588	12.76350	4.17526	1.82026
**Lgals9**	5.69046	11.84790	10.37342	0.34011	0.03237	0.47224	3.45552	0.00000	0.21868	2.48451	2.29370	0.18037	4.05472	1.52079	0.97543	0.02801
**Lgalsl**	3.14392	0.05315	0.00000	4.93038	4.35895	1.94659	0.04294	4.46621	3.92004	3.31969	3.67289	4.95357	1.27753	8.26210	1.10227	0.44832
**Lgi2**	0.01366	0.00785	0.01169	0.47238	0.00938	0.00642	0.00738	0.01175	0.51982	0.00183	0.00855	0.00601	0.01286	0.02435	0.46402	0.00725
**Lgi3**	0.03421	0.00801	0.30346	0.12182	0.00978	0.00582	0.00739	0.00966	0.00990	0.05718	0.14987	0.00780	0.00938	0.01210	0.00866	0.00721
**Lgmn**	4.92564	3.87288	8.07261	3.80953	1.57753	4.09640	8.54569	4.35281	7.52098	20.65441	4.98412	6.46790	11.13859	0.07004	1.50245	4.56891
**Lgr4**	0.43898	2.44975	0.47800	1.06909	2.96183	0.96796	0.00567	0.49870	0.44832	1.10784	0.01292	0.61815	0.00000	5.48918	0.00000	0.01287
**Lgr5**	0.06129	0.01904	0.06884	0.01947	0.02058	0.01263	0.03713	0.00223	0.64067	0.03640	0.41679	0.02974	0.11089	1.33079	0.32131	0.02899
**Lhb**	0.01323	0.00803	0.01133	0.14557	0.00930	0.00687	0.00751	0.02515	0.13272	0.06367	0.01108	0.03125	0.01246	0.01251	0.01134	0.00739
**Lhfp**	0.01404	0.00697	0.00000	1.44801	0.00889	3.09417	0.00653	0.00000	0.00000	0.00000	0.35701	1.46138	0.00000	0.00460	0.00000	0.00565
**Lhfpl2**	5.53765	7.34617	2.91105	2.67993	5.88475	0.01651	7.14677	0.00000	1.93188	0.88956	0.00000	0.03783	1.24899	0.00000	0.67774	2.79692
**Lhfpl5**	0.01713	0.00781	1.21390	0.00432	0.00860	0.01171	0.00719	1.76730	0.02287	0.15766	0.88442	0.00056	0.00000	0.00557	0.13647	0.00683
**Lhpp**	0.01335	0.00819	0.01219	2.99683	0.00915	0.00665	0.00743	0.01243	0.00000	0.61767	0.01148	0.00785	0.01405	0.00193	0.16330	0.00694
**Lhx2**	0.00948	0.00847	0.08570	0.66141	0.00919	0.00795	0.00800	0.12927	0.01938	0.06611	0.00881	0.06582	0.26789	0.01515	0.06141	0.00695
**Lhx8**	0.01307	0.00818	0.01597	2.20423	0.00926	0.00602	0.00750	0.01337	2.70951	0.00258	0.00000	0.00350	0.01303	0.02783	0.00271	0.00686
**Lias**	9.13181	5.26426	9.33702	6.17755	0.01491	10.74140	6.66599	10.60095	5.73458	16.60044	13.47656	9.48552	5.45951	23.38441	17.62006	4.80446
**Lif**	0.01427	0.00768	0.00598	0.01477	0.03499	0.00694	0.00709	1.69225	0.01047	0.30092	0.00000	0.06665	0.00914	0.02778	0.26574	0.00735
**Lifr**	1.83443	0.55678	0.45583	1.26870	4.24572	0.33869	0.12837	0.26450	0.79214	0.11215	0.68775	0.08569	0.21597	0.97137	0.22334	0.25043
**Lig1**	13.43036	1.74054	7.38168	0.81615	8.29104	0.01044	8.72636	12.37278	0.00000	3.51124	7.38270	0.86727	8.64830	0.12047	1.90741	11.36991
**Lig3**	7.89742	7.65169	6.44066	1.69704	4.84316	1.99921	7.43362	13.74126	1.75154	12.43855	3.09090	2.06851	4.84185	10.86208	13.24568	3.97249
**Lig4**	0.01783	0.00800	0.00520	1.12188	0.00937	0.01284	0.00735	0.07223	1.24707	0.00000	0.00000	0.35920	0.02538	2.77487	3.18815	0.00727
**Lilra6**	0.34485	0.00728	0.02591	0.30912	0.00733	0.00580	0.00708	0.00911	0.00677	0.01435	0.01183	0.00625	0.01237	0.38173	0.00000	0.00697
**Lim2**	0.01366	0.00813	0.01213	0.05596	0.00915	0.00654	0.00749	0.01246	0.01169	0.09321	0.01233	0.00783	0.03153	0.01116	0.00955	0.00715
**Lima1**	3.98876	0.00000	6.21669	1.77210	4.60690	2.99032	2.44773	4.75366	4.39269	2.13150	2.52354	0.17748	1.35001	0.00000	4.31243	0.00559
**Limch1**	0.01315	0.00795	0.00333	3.57057	0.00979	0.08923	0.00755	0.00750	6.17312	0.00000	0.18080	0.06487	0.09501	0.00000	0.13637	0.00650
**Limd1**	2.86157	0.01402	2.85126	8.33921	3.22046	2.48968	0.51240	0.98274	3.28465	1.93556	0.76701	0.13545	0.32984	0.00000	5.09376	0.00000
**Limd2**	1.56367	0.01652	1.71045	5.33444	0.01911	0.13997	0.14584	3.10717	10.40149	3.00799	3.58607	0.05394	0.90587	2.88295	1.66901	1.17941
**Lime1**	1.38971	0.00586	2.92740	3.86953	0.00844	1.35140	0.02861	1.78155	5.03448	0.86145	1.40623	0.45860	0.56001	0.02368	0.67561	1.61712
**Limk1**	0.05828	0.04180	0.06236	2.48068	0.01351	2.18480	0.00698	0.00000	1.94544	0.02228	0.48870	0.04409	0.01786	0.00415	0.07617	0.00612
**Limk2**	7.99737	0.17647	6.53372	6.28681	2.16164	10.90914	4.21131	11.09513	7.74808	5.09011	8.00496	0.42827	6.88184	1.40947	2.69124	7.29839
**Lims1**	1.75042	2.17820	2.49090	6.74495	1.07473	2.72338	3.53021	0.35707	3.34343	3.44749	2.16814	6.21668	4.19669	8.44091	4.56150	1.11726
**Lims2**	1.53111	0.00383	3.27777	0.16019	0.00000	3.46393	1.97782	2.86767	0.75202	0.94863	1.66539	0.05169	1.00448	0.00731	0.00000	0.00989
**Lin28a**	0.00000	0.00727	0.25367	1.41296	0.00682	0.33300	0.02583	1.97224	0.31008	1.78091	2.29418	0.08790	2.23256	2.47773	2.06787	1.59753
**Lin28b**	1.64622	5.24448	2.64778	1.48130	7.30694	0.00592	4.81577	0.52333	0.67422	0.00000	1.04382	0.01552	0.00000	6.19983	1.05842	0.00196
**Lin37**	8.23326	0.54253	3.53472	2.45732	5.82748	0.59248	1.85671	6.65717	0.00000	4.60515	3.57238	0.09346	4.89699	0.06016	0.00000	5.68388
**Lin52**	0.26788	1.26120	0.96324	1.39422	0.03412	1.07312	0.04674	0.00000	5.71945	0.11027	1.17089	6.29260	0.00000	5.57814	6.99155	0.33626
**Lin54**	0.10468	2.18468	2.26735	8.00012	3.61469	0.00554	0.58008	2.11864	10.05029	1.26727	1.41156	1.06091	0.00000	0.18838	9.51672	0.00331
**Lin7b**	0.01372	0.00817	0.00969	1.51084	0.00910	0.00661	0.00750	0.00852	0.66873	0.00299	0.28785	0.00583	0.00919	0.00621	0.00000	0.00703
**Lin7c**	1.35345	6.22590	7.35976	9.43232	7.76208	5.00797	6.42098	8.24043	5.24945	14.26250	8.22301	14.20018	3.83131	27.87840	14.83463	3.36113
**Lin9**	0.07944	0.02953	0.00000	1.45790	0.02939	0.31934	0.04631	1.37967	1.07931	0.00000	0.28966	0.03932	0.00000	7.24923	3.38766	0.00658
**Lincred1**	0.01379	0.00802	0.01189	0.01049	0.00932	0.00667	0.00750	0.01342	0.01236	0.01426	0.01297	0.02861	0.01274	0.01098	0.01889	0.00741
**Lingo1**	0.55767	0.00822	0.00515	0.14788	0.01718	0.00710	0.00708	0.01170	0.00917	0.01539	0.01349	0.00789	0.01197	0.01157	0.00970	0.01454
**Lingo2**	0.01346	0.00797	0.00971	1.23899	0.00942	0.00666	0.00728	0.01062	0.00000	0.01009	0.01103	0.00624	0.01314	0.39410	0.00000	0.00725
**Lins1**	0.00667	0.00748	1.61272	0.65464	1.93674	0.00559	0.00726	0.37490	0.00000	0.07081	0.00716	0.00396	1.02187	0.09027	0.09630	0.02208
**Lipe**	0.27858	0.03883	0.00590	0.03198	0.00719	0.00695	0.00708	0.00795	0.09992	1.00439	0.09222	0.00446	0.00991	0.01394	0.02375	0.00674
**Lipg**	0.01796	0.00822	0.01036	0.01048	0.01054	0.00674	0.00750	0.01356	0.01258	0.01085	0.01184	0.00709	0.01259	0.22486	0.00468	0.03703
**Liph**	3.13757	0.06098	1.23913	0.44671	0.00000	1.59961	0.04974	0.34781	0.25947	0.33446	0.11785	0.03568	0.00000	0.07831	0.00332	0.02590
**Lipo2**	0.01275	0.00801	0.01216	0.08401	0.01002	0.00719	0.16447	0.01227	0.14043	0.07023	0.05246	0.00679	0.09936	0.00716	0.09400	0.00803

**Lipo3**	0.48612	0.01593	0.19696	2.22123	0.12129	0.00454	2.31225	0.00000	4.99739	0.72451	0.61585	0.02190	0.02089	0.00000	0.28556	0.00621
**Lipt1**	1.54376	0.68929	2.27635	1.09610	0.00179	1.85595	0.00342	1.33255	0.00000	2.44903	1.12700	0.07740	3.60999	1.10259	2.45691	0.01598
**Lipt2**	0.76512	0.00782	0.00497	0.00481	0.01702	0.00685	0.05784	0.00940	0.41783	0.01165	0.00914	0.03810	0.01834	0.01308	0.00815	0.00679
**Litaf**	5.49180	4.80279	14.09004	8.31242	8.49628	12.18366	7.11668	10.75182	3.13889	19.23715	10.58005	4.15417	9.52114	10.67288	14.20354	7.24917
**Lix1**	0.24200	0.05881	0.63500	0.42535	0.08652	0.05551	0.23217	0.04925	0.36012	0.17289	1.04476	0.13780	0.08248	0.61478	0.33474	0.16614
**Lix1l**	0.01377	0.00809	0.01183	0.20981	0.00929	0.00672	0.00753	0.01373	0.00810	0.02093	0.01341	0.02986	0.01289	0.01129	0.00707	0.00739
**Llgl1**	4.96918	1.22690	6.24539	1.13021	6.22181	4.28767	3.64266	12.52092	3.00174	2.33140	3.55193	0.50049	1.34561	0.11136	4.83691	1.35403
**Llgl2**	0.83371	0.00445	0.00000	1.27411	2.01408	6.92638	0.86776	5.67162	3.33558	8.83696	0.31683	0.08106	3.79529	0.00253	0.00000	8.02233
**Llph**	22.58106	11.11157	18.17945	14.47539	14.84713	6.25220	7.87234	19.82263	20.68465	26.41947	21.91059	9.68497	23.68320	20.03742	13.49046	5.19146
**Lman1**	4.72034	0.60042	3.98709	0.13488	4.21627	3.10741	1.34319	2.29379	0.47788	0.76150	1.57063	1.81374	2.34499	2.75187	0.85557	1.57155
**Lman1l**	0.01273	0.02980	0.04365	0.02782	0.00882	0.00575	0.00740	0.00060	0.72423	0.52691	0.35458	0.01436	0.00801	0.00634	0.12689	0.00695
**Lman2**	13.84097	8.09978	17.79003	7.49391	7.72188	7.45045	8.94462	16.30774	14.96165	12.04201	10.81697	3.03292	16.39250	3.96140	6.83337	9.67067
**Lman2l**	3.13746	3.36583	0.39949	2.98945	0.01263	0.00130	0.00219	1.94995	0.08732	0.26385	0.11681	5.21621	1.06086	1.36784	1.02469	0.00000
**Lmbr1**	1.14649	0.00720	1.03933	0.85201	0.00934	0.01064	0.00595	0.00980	1.61154	3.55441	0.09191	0.01172	1.07680	0.43670	1.69353	0.87090
**Lmbr1l**	0.05276	0.00823	0.05762	3.23956	0.02824	0.00669	0.04776	0.00000	3.85676	0.64831	1.78354	0.05231	0.17575	0.10430	0.46567	0.30197
**Lmbrd1**	2.06061	0.01966	4.57513	0.96390	0.08156	0.03317	1.68912	3.27152	0.32482	4.42179	5.41319	14.08531	3.88230	10.24630	10.26968	0.53245
**Lmbrd2**	0.00129	0.00819	0.13746	1.31423	0.00734	0.00559	0.00793	0.19523	0.11882	0.00000	0.01055	0.03233	0.00336	0.00993	0.16274	1.80797
**Lmf1**	0.14075	1.76527	0.58006	0.00000	0.22680	0.00559	1.44418	0.00000	0.01091	0.00000	0.74016	0.00167	0.52816	0.00987	0.00000	0.06885
**Lmf2**	6.30020	1.68023	0.80643	6.25812	0.00000	0.52758	0.00000	3.80467	1.34575	2.83489	1.32150	0.11885	3.23446	0.00000	0.00000	2.44887
**Lmln**	0.01376	0.00787	0.01116	0.33035	0.00942	0.01266	0.00736	0.01144	0.06233	0.00557	0.01085	0.00652	0.01265	0.00653	0.71477	0.00728
**Lmna**	15.24860	0.10090	1.09471	3.88526	3.40274	9.44924	0.13432	8.45679	12.98581	7.85660	4.06664	0.43329	10.18150	0.00000	0.00000	12.87875
**Lmnb1**	3.63285	3.70200	3.24831	2.70738	2.67408	2.87000	0.04325	2.03204	5.45639	0.00000	2.95922	0.15202	1.58080	3.89274	4.03765	3.64884
**Lmnb2**	2.95826	1.65440	3.65572	0.43092	5.93355	0.91677	1.63881	3.61642	0.06890	1.80773	2.79029	0.28440	3.32209	0.26016	2.75996	6.94779
**Lmntd2**	0.42132	0.00709	0.00312	0.31679	0.01852	0.00813	1.89431	0.00687	0.00664	0.00000	0.09680	0.00565	0.19030	0.01056	0.00306	0.00731
**Lmo1**	0.01178	0.00788	0.01140	1.06405	0.00987	0.00720	0.07011	0.00387	0.41411	0.90124	0.27446	0.01196	0.25752	0.00617	0.00000	0.00780
**Lmo2**	1.72071	2.92068	0.78204	0.00000	8.64857	0.00365	0.26828	0.56994	0.00718	0.85343	0.19793	0.00373	0.00000	0.00997	0.00000	0.04890
**Lmo4**	0.44637	0.00574	0.00000	1.18277	1.83040	0.98837	0.10644	0.00396	0.67045	1.07510	0.01620	0.00000	0.00594	0.52062	0.00000	0.00623
**Lmo7**	0.56520	0.00261	3.04838	5.24929	0.18059	0.00727	2.75096	12.60252	3.45005	8.63103	6.23774	0.56422	2.37668	5.76756	3.51703	0.00957
**Lmod1**	0.01322	0.00792	0.00796	1.22059	0.00878	0.00679	0.00706	0.00000	0.41867	1.32338	0.53090	0.04330	0.00723	0.00557	0.00462	0.00689
**Lmod3**	0.01009	0.00776	0.00352	0.00001	0.00953	0.00694	0.00722	0.80541	0.02305	0.73978	0.20884	0.02963	0.00592	0.02114	1.29814	0.00755
**Lmtk2**	1.61023	0.01519	0.00000	4.09353	0.01312	4.84980	1.46054	2.00802	1.68964	2.83863	2.08026	1.25883	1.28550	0.52240	1.89501	0.00205
**Lmx1a**	0.01275	0.00747	0.01033	4.99956	0.00903	0.00576	0.00706	0.01410	3.55683	0.56119	0.00001	0.00901	0.01091	0.04299	0.33574	0.00606
**Lmx1b**	0.01179	0.00807	0.01447	0.06369	0.01028	0.00704	0.00750	0.01360	0.00874	0.33511	0.01420	0.00640	0.33172	0.01119	0.00731	0.00774
**Lncenc1**	1.28056	3.26036	8.17702	0.00000	0.09084	0.00519	1.89578	0.49162	0.42504	0.00177	0.00000	0.00861	0.00000	0.01266	0.00000	0.00911
**Lnpep**	0.01501	0.00811	0.04031	3.87439	0.00891	0.00787	0.00715	0.00298	0.36919	0.01304	0.37680	0.00000	0.00834	0.86000	2.88105	0.00649
**Lnpk1**	0.57507	0.04271	1.50029	2.67721	0.02298	0.01189	0.01551	2.41033	2.36242	0.00000	1.54666	2.84441	1.12858	5.44646	0.57115	0.12729
**Lnx1**	0.02913	1.52472	0.77471	0.08371	0.01004	0.45726	0.38575	0.00733	0.12003	0.00000	0.01721	0.05312	0.37636	1.06626	0.13693	0.15770
**Lnx2**	0.52688	0.05758	1.00210	2.45836	0.12355	3.57093	0.00575	1.38144	2.88019	0.04106	0.57535	0.15296	1.06761	0.00000	0.10048	0.00406
**LOC10050470**	0.01378	0.00789	0.01206	0.02729	0.00933	0.00666	0.00753	0.01359	0.01118	0.01419	0.01264	0.00797	0.01274	0.01656	0.02563	0.00741
**LOC10050502**	0.01619	0.01888	0.08098	0.54773	0.02123	0.00617	0.01766	0.01612	0.68849	0.01154	0.17822	0.04586	0.01278	0.21757	0.00640	0.00726
**LOC10086161**	0.07763	0.00803	0.00888	0.31811	0.00787	0.00689	0.00744	0.01449	0.00956	0.01669	0.03853	0.01893	0.01215	0.01078	0.00701	0.01441
**LOC10105607**	0.62326	2.14289	0.51738	0.31369	1.06910	0.22349	0.69184	0.76838	0.12976	0.53930	0.24672	2.24768	0.40271	5.50255	0.77382	0.29266
**LOC10263481**	1.30536	0.07713	1.14442	3.06404	0.41897	0.12765	0.07298	0.00000	5.28177	0.17048	8.40633	0.10352	0.42704	2.51502	0.16544	0.46466
**LOC10524239**	0.01345	0.00814	0.26459	2.08269	0.01067	0.00560	0.00751	0.16225	0.84112	0.31598	0.00426	0.00494	0.00708	0.00270	0.36555	0.00719
**LOC10524257**	0.23791	0.13591	0.36876	0.22692	0.14967	0.07858	0.09701	0.05418	0.44789	0.25492	1.47726	0.17203	0.43557	0.70867	0.29539	0.07838
**LOC10524586**	0.09152	0.17244	0.00375	0.00257	0.01495	0.00696	0.00727	0.54554	0.79256	0.22787	0.58974	0.31519	0.00344	0.18799	0.46790	0.00757
**LOC10524605**	0.01856	0.00801	0.01167	0.01103	0.01059	0.00668	0.00751	0.01333	0.01209	0.01376	0.01265	0.00794	0.01273	0.01740	0.03251	0.00746
**LOC10524650**	3.93553	0.27897	0.33798	0.03790	4.00253	0.01882	0.00501	0.21896	0.04852	0.36222	0.05589	0.01235	0.00972	0.03007	0.00000	1.10911
**LOC10602923**	0.00877	0.00819	0.01149	9.32401	0.00914	0.00409	0.00751	0.04006	19.48173	0.01217	0.26298	0.00000	0.04876	0.02554	0.26128	0.00458
**LOC106740**	0.56081	0.01993	0.09088	0.38559	0.01002	0.00548	0.00773	0.37095	0.24454	0.88919	0.00829	0.00487	0.01213	0.01077	0.54378	0.00680
**LOC215458**	0.26713	0.00772	0.02928	0.01421	0.00815	0.00569	0.00723	0.09630	0.03744	0.36000	0.02001	0.03840	0.07853	0.18640	0.54506	0.00696
**LOC666331**	0.01373	0.00791	0.01236	0.00576	0.00933	0.00661	0.00752	0.01370	0.13078	0.01167	0.01171	0.00746	0.01269	0.00760	0.07498	0.00739
**Lockd**	0.86960	0.00759	0.00415	0.00495	0.01781	0.00709	0.00678	0.00983	0.51048	0.05432	0.00828	0.00620	0.01204	0.01632	0.02460	0.00676
**Lonp1**	11.24581	4.12825	10.83755	8.28661	10.93357	7.19571	8.34036	14.30725	9.42181	18.85757	10.62671	6.94524	9.24548	0.19230	9.55048	9.45450
**Lonp2**	4.65699	0.37313	4.71929	9.20173	1.60801	0.00331	6.12616	10.89626	8.07181	8.47270	7.63376	8.30590	3.98381	5.24462	6.06918	5.63611
**Lonrf1**	1.91951	0.92048	1.14334	4.54061	0.00687	1.22642	2.47995	0.13328	0.00000	0.73095	0.01642	0.01784	0.00000	0.00000	0.80389	0.01898
**Lonrf2**	0.00921	0.00742	0.01128	0.02737	0.00966	0.02171	0.00715	0.50288	0.01151	0.00838	0.00945	0.00654	0.00565	0.01898	0.05388	0.00738
**Lonrf3**	0.86300	0.00560	0.00000	10.56488	0.00570	0.00318	1.09370	5.39754	11.92260	4.80296	3.50488	0.07247	0.00000	2.34273	3.15502	0.00318
**Loxhd1**	0.01290	0.00800	0.01382	0.12586	0.00990	0.00706	0.00764	0.01359	0.00950	0.56412	0.01386	0.00612	0.07386	0.00597	0.26284	0.00771
**Loxl1**	0.01025	0.00799	0.01086	0.63619	0.00948	0.00728	0.00727	0.00946	0.01778	3.66153	0.26804	0.09854	0.31312	0.00848	0.01295	0.00751
**Loxl2**	1.20273	0.08000	1.02577	5.96154	0.37584	0.12180	0.16474	0.00000	10.66053	0.00000	9.75425	1.35307	0.50332	4.12647	0.40289	0.15300
**Lpar1**	3.11005	3.23238	2.18527	0.00000	0.00000	0.72052	0.00000	2.07364	0.56733	2.50581	3.69375	2.46180	1.44546	4.99226	2.56377	0.22517

**Lpar2**	0.21417	0.00753	0.19250	0.01927	0.00710	0.00549	0.00739	0.43153	0.01173	0.00000	0.00946	0.00714	0.57823	0.02837	0.02895	0.50010
**Lpar5**	0.02200	0.00813	0.10574	0.00847	0.01263	0.20120	0.00756	0.46941	0.01162	0.33694	0.01091	0.00614	0.35464	0.00743	0.09811	0.00753
**Lpar6**	0.00758	0.00751	0.01054	1.64143	0.00770	0.00592	0.00722	0.01047	2.89298	0.00272	0.00000	0.01143	0.00795	0.00421	0.53306	1.29829
**Lpcat1**	2.51410	1.76483	0.00000	0.00000	0.00323	2.90435	0.03617	1.06705	0.01053	0.64381	0.00000	0.02105	1.15201	0.00678	0.58435	1.05333
**Lpcat2**	0.01052	0.00881	0.00672	0.84841	0.01003	0.00816	0.00784	0.04567	0.56153	0.26791	1.05770	0.03132	0.26184	0.00375	0.09049	0.00735
**Lpcat3**	16.88330	6.51817	15.43220	0.54704	6.39619	6.90283	11.05521	15.39013	1.14546	9.19998	11.58445	2.90272	16.19145	4.02426	4.40015	6.00759
**Lpcat4**	0.95453	0.00743	0.73235	0.00480	0.17187	6.21671	0.00528	0.27506	0.12028	1.87291	0.03041	0.01200	1.56806	0.05134	0.01477	0.00346
**Lpgat1**	2.60505	0.00000	3.38865	6.29666	0.22548	9.15266	6.69633	3.62616	1.21108	9.14818	5.43520	4.42584	6.64975	9.93538	10.93296	3.02555
**Lpin1**	1.79463	0.00306	0.21103	0.43278	0.01200	2.23441	5.46926	0.48756	0.00304	3.46728	0.70440	0.09616	0.00000	0.02079	0.00000	0.91925
**Lpin2**	0.69990	0.00742	0.00000	2.20057	0.01653	0.01148	0.00610	0.00986	2.21033	1.68611	0.00000	0.04693	0.83934	0.17780	2.93815	0.00626
**Lpin3**	1.65000	0.00252	1.89433	0.99056	0.00508	3.21502	4.93483	3.65452	0.77578	0.00000	1.06039	0.07961	0.00000	0.00517	0.62131	0.00311
**Lpl**	0.07459	0.00809	0.04043	1.90784	0.00798	0.01409	0.00733	0.03712	0.99654	0.06677	0.09054	0.02694	0.05212	0.12241	0.37384	0.03593
**Lpo**	0.01335	0.00806	0.01228	0.00735	0.00907	0.00648	0.00734	0.01062	0.01189	0.79145	0.01137	0.00591	0.01089	0.00827	0.04948	0.00721
**Lpp**	5.16969	0.03162	3.02075	0.86929	2.87663	1.75265	0.15551	0.94904	0.80199	2.26924	0.70091	0.18918	2.64103	0.38407	0.36547	3.52967
**Lppos**	0.97361	0.00725	0.14144	0.91304	0.01694	0.00483	0.00649	0.18203	1.34178	0.43543	0.05106	0.08753	0.00663	0.00259	0.23728	0.00633
**Lrba**	1.69729	1.71213	1.99723	2.17603	0.31320	5.07125	1.64113	4.01905	2.15610	4.13826	2.77613	1.18501	6.73287	0.62475	1.17960	0.42021
**Lrch1**	0.82565	0.00670	0.64371	0.99867	0.00517	0.00463	0.00685	0.00899	0.73291	0.19895	0.01277	0.04405	0.00868	0.00535	0.57288	0.09259
**Lrch3**	0.44459	1.04485	0.00000	0.12353	0.01690	0.00806	0.00652	0.01313	0.00938	0.02348	0.01254	0.01728	0.01756	0.01063	0.04538	0.00673
**Lrcol1**	0.01276	0.00817	0.01166	0.00577	0.00888	0.00645	0.00747	0.01384	0.01095	1.35402	0.01237	0.00544	0.01010	0.02967	0.20245	0.00708
**Lrfn1**	0.22378	0.03993	0.00000	0.04726	0.08093	0.04478	0.00717	0.01452	0.01211	0.27441	0.06580	0.00653	0.01749	0.01058	0.00788	0.00752
**Lrfn4**	0.03680	0.00829	0.00870	0.10313	0.01460	0.06732	0.00727	0.01611	0.01076	0.16559	0.25241	0.00588	0.22212	0.00998	0.00000	0.00761
**Lrguk**	0.01628	0.00792	0.12022	0.08328	0.00897	0.00596	0.00747	0.01322	0.00961	0.12328	0.01220	0.00840	0.01156	0.00989	0.01908	0.00731
**Lrif1**	0.00292	0.00341	1.58526	1.90108	0.00760	0.00475	0.00421	0.33640	2.71824	0.00000	1.58882	13.22588	0.55825	9.05774	3.51625	0.00485
**Lrig1**	0.26044	0.00683	0.98343	0.95149	0.01650	0.62891	0.33468	0.95638	0.18435	0.13782	0.03284	0.02821	0.00000	0.00403	1.34379	0.01378
**Lrig2**	0.04351	0.07706	0.44451	2.10275	0.00787	0.00876	0.00722	0.00000	7.38228	0.04902	0.48134	0.07043	0.41514	3.43569	1.83311	0.00617
**Lrig3**	0.01039	0.00648	0.00659	0.00219	0.00955	0.00674	2.05077	0.01001	0.51935	0.00928	0.00536	0.20010	0.01052	0.00532	0.32995	0.00722
**Lrmp**	0.16491	0.00793	0.00782	0.01023	0.00953	0.00688	0.00736	0.01391	0.01256	0.03923	0.01284	0.00742	0.01252	0.00878	0.03335	0.00727
**Lrp1**	0.00834	0.00584	0.00696	4.22457	3.14064	1.96103	0.00688	0.00349	0.50842	0.46515	0.11735	0.25988	0.30303	0.00000	0.37526	0.00555
**Lrp10**	2.77351	0.03432	1.98297	0.45265	0.06592	0.05632	2.36264	2.62645	1.21515	3.56089	0.74649	0.09985	4.14894	0.11682	0.04874	6.95347
**Lrp11**	1.61965	2.76176	0.99774	0.22099	4.95157	0.08257	1.93533	2.76787	0.01613	1.13406	1.12256	7.47900	1.29607	0.90622	0.70462	0.90685
**Lrp12**	0.22397	0.00783	0.30643	0.55912	0.00669	0.40189	0.00712	0.00955	0.04242	0.50584	0.00003	0.01823	0.00696	2.09708	1.23820	0.35662
**Lrp2**	0.00000	5.59585	9.21626	0.44649	0.04388	15.54434	10.41246	2.46805	0.30068	0.00224	0.00000	0.01890	19.90247	0.00527	0.62053	4.54809
**Lrp3**	0.01203	0.00771	0.34910	0.00864	0.00840	0.00918	0.00716	0.01479	0.01201	0.51422	0.01174	0.00582	0.00967	0.01246	0.00482	0.00747
**Lrp4**	0.01215	0.01871	0.08626	0.13708	0.00893	0.00842	0.01731	0.00761	0.32749	0.04384	0.28843	0.03714	0.03615	0.15225	0.19238	0.00715
**Lrp5**	0.01335	0.00872	0.01205	0.57052	0.00857	0.23349	0.00793	0.05802	1.13206	0.63378	0.00000	0.02850	0.00642	0.01106	0.00152	0.15411
**Lrp6**	0.99584	0.00628	1.56594	9.32549	0.01103	0.66177	0.17841	0.85553	2.23056	2.94777	2.40823	1.02440	0.87136	8.95062	6.57354	1.63265
**Lrp8**	3.35263	0.01380	0.51301	0.52881	3.81891	2.99596	0.00440	0.65415	0.17473	0.00000	0.40684	0.04138	0.88585	0.00568	0.87492	0.00881
**Lrpap1**	13.13297	10.93424	34.96778	4.69095	10.76116	10.62368	21.04426	40.94514	6.12764	24.17563	27.82008	13.73731	42.67399	12.05368	4.43220	14.94268
**Lrpprc**	9.06137	12.76069	13.72600	0.46750	19.34580	6.56382	7.27156	11.65893	1.02782	10.00276	7.80029	8.61262	9.62824	7.38594	7.98038	4.19628
**Lrr1**	6.41725	6.61640	10.32590	16.80245	7.55423	7.62623	3.44819	19.09360	13.11042	39.02812	21.70382	21.91976	16.92145	15.61938	14.06492	0.26341
**Lrrc1**	1.30981	0.00605	0.00361	1.03183	0.00540	0.00692	0.00641	0.01762	0.00000	0.01585	0.01174	0.00702	0.01116	0.01667	0.02978	0.00581
**Lrrc10b**	0.02251	0.00859	0.01811	0.00502	0.01168	0.00731	0.01838	0.01675	0.32597	0.10467	0.00945	0.00739	0.01400	0.01225	0.00811	0.00732
**Lrrc14**	3.96342	1.44955	3.10624	2.31047	0.15502	0.00208	2.89116	2.85516	2.45108	0.26653	0.37023	0.04864	2.87051	0.00000	1.68776	1.27709
**Lrrc14b**	0.51287	0.07873	0.26667	0.39860	0.04121	0.01373	0.06093	0.05151	0.24861	0.35522	0.79251	0.02080	0.20233	0.95474	0.14511	0.03554
**Lrrc15**	1.23535	0.00697	1.32485	3.54124	0.01341	0.00394	0.00596	0.00282	0.00000	1.23131	0.13912	0.16902	0.01394	2.23401	4.66235	0.01307
**Lrrc16a**	0.69280	3.87589	0.76865	7.37649	3.87337	0.03207	2.70408	0.62585	1.28232	0.25463	0.90728	0.03489	0.33473	0.00000	0.00000	0.10835
**Lrrc17**	0.03482	0.06808	0.04739	2.48167	0.00867	0.00597	0.00670	0.00000	1.16712	1.09877	0.94870	0.11055	0.00524	0.16379	2.73264	0.05087
**Lrrc2**	2.43587	3.37743	1.33225	3.34867	2.53994	0.95870	4.03674	0.00000	6.18177	0.08740	3.70041	0.20516	0.44977	2.08298	1.25929	0.12902
**Lrrc20**	1.90184	0.00516	1.25145	0.54490	0.05132	0.01244	0.02539	0.04944	0.22458	0.40534	0.26698	0.00086	0.67284	0.18968	0.27543	0.02746
**Lrrc23**	0.01002	0.00644	0.00254	0.00417	1.94873	0.00850	0.00691	0.65181	0.00986	1.39243	0.00767	0.01023	0.51287	0.01726	0.00000	0.00704
**Lrrc25**	0.00890	0.00749	0.00852	0.09511	0.00938	0.00673	0.00711	0.38770	0.32398	0.83590	0.00823	0.03566	0.00574	0.01883	0.00000	0.00709
**Lrrc27**	0.05546	0.00702	0.00465	0.23888	0.00848	0.00731	1.19777	0.01012	0.00692	0.01649	0.01330	0.00672	0.01067	0.01214	0.00879	0.00720
**Lrrc28**	0.07271	0.68345	2.08158	3.92503	1.17425	0.55931	0.74829	2.77531	1.74708	1.20581	0.41457	0.06504	0.00000	1.14480	0.28288	0.00521
**Lrrc3**	0.05458	0.02925	0.23450	0.17822	0.03290	0.02632	0.03766	0.01293	0.29601	0.13093	0.48602	0.03242	0.13652	0.26400	0.17550	0.01447
**Lrrc31**	0.08834	0.01842	0.09272	0.05467	0.02055	0.03210	0.06826	0.00255	0.17165	0.03952	0.61174	0.07134	0.16221	0.26863	0.10863	0.02889
**Lrrc32**	0.01375	0.00796	0.01208	0.53333	0.00932	0.00660	0.00751	0.01368	0.14875	0.01377	0.01246	0.00781	0.01274	0.01487	0.01306	0.00732
**Lrrc34**	0.75883	0.00768	0.00451	0.09285	0.01177	0.00709	0.01715	0.01602	0.00786	0.02286	0.01176	0.00740	0.01140	0.01061	0.01425	0.00664
**Lrrc39**	0.50674	0.69306	0.63989	0.70094	0.55204	0.30037	0.16747	0.27975	1.07368	0.38088	0.55965	1.01325	1.05755	0.80565	0.72246	0.28811
**Lrrc4**	0.01612	0.00748	0.32541	1.77710	0.00871	0.00538	0.00706	0.01048	1.22466	0.00000	0.13324	0.02132	0.00979	0.00174	0.95951	0.00668
**Lrrc40**	5.09882	5.54215	5.88899	10.17530	5.21531	0.01878	5.96438	8.21745	2.75146	5.15864	5.56707	1.32214	6.05869	1.01801	10.88303	3.12016
**Lrrc41**	1.43532	1.66183	2.96464	2.83121	0.03038	0.40757	3.35357	2.33949	0.93493	3.89986	0.59940	2.61439	1.41052	1.91270	0.45852	2.09254
**Lrrc42**	4.05899	2.09645	5.92594	11.28643	0.26803	3.72042	1.80435	22.38334	23.08863	20.83035	15.29884	3.63761	6.09575	8.34013	11.13847	0.17940

**Lrrc43**	0.01361	0.00795	0.01214	0.23698	0.00910	0.00660	0.00731	0.01109	0.10685	0.36382	0.01318	0.00653	0.01239	0.00774	0.05137	0.00776
**Lrrc45**	0.00000	1.38754	3.34713	1.10183	3.01503	2.21286	2.43899	2.09932	0.47922	0.70989	1.27089	0.00620	2.58314	0.01634	0.00000	2.00973
**Lrrc46**	0.01909	0.00779	0.24018	0.59786	0.00914	0.00581	0.00731	0.03407	0.93140	0.00000	0.00678	0.00386	0.02320	0.02589	0.78510	0.00718
**Lrrc47**	1.39723	0.38056	1.73809	0.20043	0.02431	0.00463	0.51249	2.02070	0.35725	0.49647	1.32921	0.05740	0.69501	0.04237	2.45052	0.67525
**Lrrc48**	0.01267	0.00807	0.01367	0.40212	0.01015	0.00713	0.00763	0.01431	0.00828	0.18943	0.02932	0.01745	0.17326	0.01179	0.03144	0.00770
**Lrrc49**	0.01245	0.00792	0.09308	0.64511	0.00901	0.00570	0.00739	0.01032	0.00000	0.86573	0.02882	0.00532	0.01083	0.00759	0.02327	0.00741
**Lrrc51**	0.25017	0.00763	1.19431	0.07174	0.00535	3.00509	0.00700	0.11451	0.24513	0.10866	0.00000	0.50649	0.00013	0.83181	1.39228	0.00553
**Lrrc56**	0.04800	0.00788	0.00000	1.24980	0.01085	3.11937	0.01725	0.01260	3.43101	0.35705	0.02033	0.00000	0.06160	0.00151	0.09264	0.00588
**Lrrc57**	2.00333	1.86401	1.95574	2.31720	1.75852	3.30761	1.21553	5.29016	3.43663	2.16879	1.36589	0.18427	0.13662	4.49308	7.94657	0.00124
**Lrrc58**	7.11636	4.96761	11.02240	4.42092	3.87177	2.94089	3.84636	2.54912	3.66085	12.41174	11.02795	14.55988	7.35445	20.91902	17.03577	5.90007
**Lrrc59**	28.04224	16.48544	22.28567	6.17859	19.74906	14.71820	13.66339	24.34190	11.00547	13.72989	9.54837	6.26545	21.71308	10.19159	10.33481	15.39372
**Lrrc61**	0.06731	0.04973	0.18060	0.16460	0.02690	0.13007	0.06773	0.04004	0.02798	0.43407	0.13582	0.02340	0.77307	0.06232	0.13133	0.03625
**Lrrc73**	0.01116	0.00832	0.01317	0.60534	0.01016	0.00686	0.00748	0.01408	2.07697	0.01737	0.01726	0.02443	0.44662	0.00996	0.00669	0.00739
**Lrrc74a**	0.01375	0.00794	0.01194	0.00658	0.00929	0.00662	0.00749	0.01305	0.07958	0.01200	0.01186	0.00792	0.01975	0.01442	0.05594	0.00738
**Lrrc74b**	0.01941	0.04113	0.03459	0.27411	0.00973	0.00824	0.03753	0.01795	0.18391	0.58034	0.09956	0.00548	0.01928	0.04472	0.18187	0.00736
**Lrrc75a**	0.01551	0.00769	0.04098	0.15213	0.01031	4.17992	0.00657	0.00706	0.00636	0.00000	0.03254	0.00704	0.65671	0.02144	0.04146	0.00543
**Lrrc75b**	2.01388	0.00413	1.27103	0.00267	0.15833	0.01513	0.03423	0.33890	0.01627	1.73692	0.74754	3.28954	0.00000	0.02810	0.05209	2.51388
**Lrrc8a**	1.00868	0.00515	0.16363	3.28919	0.01374	2.29520	0.65789	2.34063	6.10829	1.22324	1.28635	1.78159	0.00000	0.82062	1.77187	0.00391
**Lrrc8b**	0.06727	0.00769	0.28895	0.23324	0.00732	0.00790	0.00745	0.01293	0.00585	0.03304	0.00692	0.07320	0.01045	0.00762	1.80069	0.00709
**Lrrc8d**	1.19068	0.20186	1.92356	3.47146	2.00222	2.02992	0.36310	0.74505	0.45827	0.37897	1.26814	0.26603	0.45732	1.25103	1.03888	0.00360
**Lrrc8e**	0.07975	0.00761	0.02969	4.52862	0.00876	0.02247	0.00641	0.18625	2.76113	1.50628	1.31539	0.20555	0.05749	1.11978	1.73082	0.04098
**Lrrcc1**	1.25855	0.00553	1.41066	1.29868	0.00368	0.00476	0.04716	1.30091	0.55422	0.43114	0.71497	0.00000	0.00848	0.00345	1.43822	0.01294
**Lrrfip1**	0.46583	3.93598	3.97897	7.26403	0.02661	8.04421	0.03506	0.03992	0.04360	0.00000	0.44224	0.08083	3.14981	2.62086	7.27852	2.04548
**Lrrfip2**	4.73649	5.04026	5.94853	2.53838	7.54825	7.38438	4.12875	7.07243	2.11139	6.68842	4.54180	1.24762	1.51323	5.00932	6.58289	0.02358
**Lrriq3**	0.01379	0.00774	0.01188	1.00735	0.00941	0.00647	0.00740	0.01227	0.01272	0.00719	0.01147	0.00725	0.01277	0.01130	0.29527	0.00721
**Lrrn4**	0.00206	0.00818	0.93619	0.50091	0.01682	0.00933	0.01876	0.29356	0.39943	2.26256	1.30744	0.00546	0.00000	0.09803	0.00000	0.66127
**Lrrn4cl**	0.11465	0.02910	0.32318	0.59525	0.06078	0.01825	0.06805	0.02089	0.34449	0.11893	0.88580	0.08040	0.26112	0.47706	0.21070	0.05003
**Lrrtm2**	0.03801	0.00795	0.01079	0.01054	0.00830	0.00674	0.00749	0.01491	0.01241	0.01505	0.01304	0.00799	0.01268	0.01126	0.01579	0.00732
**Lrsam1**	0.58590	0.09062	0.12875	1.55706	0.40099	0.00504	3.25041	1.94019	0.00000	0.00041	1.65151	1.28623	2.57447	0.00000	0.00000	0.01251
**Lrwd1**	10.19742	0.66147	3.10556	4.25975	2.00885	0.00239	0.40333	9.15336	5.88318	4.85308	5.70001	0.40990	8.82861	0.00719	0.27261	4.38466
**Lsg1**	11.14725	8.11346	10.65312	2.75542	8.79596	5.93498	7.93449	13.42377	5.12973	11.19824	4.11890	6.91919	8.24048	0.60825	0.37105	4.76342
**Lsm1**	7.88064	0.14798	2.39962	5.24849	4.50354	3.73901	1.32241	2.77569	6.64839	8.30441	2.64652	0.94622	4.74959	0.75660	0.56612	4.90363
**Lsm10**	4.04313	0.00000	1.68802	5.90434	4.81685	5.02214	0.52255	0.78957	3.58140	3.52382	1.21226	0.03725	0.00000	0.00000	2.90615	2.27988
**Lsm11**	0.99814	0.01662	0.05376	2.07751	0.01422	2.37952	3.02818	0.09478	0.44005	0.77407	2.26823	0.05778	0.10698	1.49330	0.36546	1.85503
**Lsm12**	1.02812	0.00000	5.35440	3.70883	5.21532	3.15362	3.88627	8.02562	7.37301	14.12452	15.04740	1.75687	9.95059	0.00000	3.80567	5.53762
**Lsm14a**	10.49669	7.97514	3.20456	22.40358	10.42913	4.60667	1.24905	5.67926	12.42597	4.72932	4.88789	12.86016	8.34576	7.27210	10.64737	6.24517
**Lsm14b**	1.07510	0.01952	0.00000	10.03258	0.01241	0.58729	0.03911	0.16555	7.05435	1.61972	1.54587	0.10665	0.14839	0.33326	1.26949	0.35400
**Lsm2**	3.51049	0.72977	5.58543	7.34906	3.21227	0.00738	0.48640	1.37916	9.44871	15.77962	6.61061	0.43406	3.98034	3.38408	3.11327	0.48541
**Lsm3**	11.31615	5.40929	6.98528	0.21148	10.98453	5.23826	2.69255	3.31177	0.14370	5.20371	5.34236	0.43809	8.65817	7.58194	2.11578	2.97750
**Lsm4**	3.50082	0.38682	0.83780	1.05349	1.41151	0.02695	0.08229	0.03697	2.96886	13.80042	6.90197	0.09809	19.15460	0.00000	0.00000	10.94333
**Lsm5**	6.78175	0.81666	4.20528	0.00000	6.28350	1.59869	0.96007	1.01651	1.13165	6.02999	12.37053	1.27465	5.30125	9.42378	4.54155	3.54735
**Lsm6**	8.83921	0.57091	7.57983	5.05709	3.96233	2.21517	1.71127	1.68522	5.09333	2.58996	5.07767	0.45702	6.21742	2.08902	1.11697	3.57608
**Lsm7**	6.00296	0.49525	5.31327	5.55629	5.26769	1.18685	0.24653	0.42742	2.51518	4.82229	10.02069	0.46164	2.47576	0.05463	0.31664	0.92075
**Lsm8**	8.08647	3.96808	5.80558	8.01611	8.05437	1.72718	3.06140	6.15044	1.88958	5.44622	9.85563	3.74041	9.56292	4.06596	6.14075	7.31370
**Lsp1**	0.01192	0.00769	0.01064	5.74977	0.00858	0.00587	0.00700	0.00885	1.96915	3.39669	0.00000	0.03461	0.01079	0.00000	0.36238	0.00640
**Lsr**	8.37062	4.37849	7.35294	1.14642	1.89721	4.22186	3.02078	3.57050	0.29027	0.00000	1.32528	0.09474	6.47726	0.00000	1.09239	7.97114
**Lss**	1.45163	6.25048	4.02636	4.66750	0.29311	0.41849	2.05308	7.28000	1.39105	5.62278	4.09185	0.09241	1.75653	4.73394	6.02855	0.30602
**Lst1**	0.01049	0.00727	0.00001	2.66968	0.00906	0.00715	0.00510	0.00000	4.12820	0.30023	5.07179	0.07662	0.10822	0.63625	0.70397	0.00590
**Lta**	0.01303	0.00769	0.01111	0.81461	0.00889	0.00633	0.00731	0.00999	0.91097	1.17928	0.00001	0.02235	0.01047	0.01089	0.23486	0.00690
**Lta4h**	9.26263	4.98897	5.00186	0.18915	5.49971	5.55252	3.70748	3.11140	0.00105	0.10426	0.00000	0.06426	6.36606	0.08285	0.72782	6.41948
**Ltb**	0.01530	0.00748	0.03897	0.04177	0.00878	0.00595	0.00695	0.00000	0.15479	1.78251	0.78560	0.00000	0.01152	0.00895	0.00000	0.00719
**Ltbp1**	0.01380	0.00790	0.01164	0.01031	0.00925	0.00671	0.00742	0.01243	0.01227	0.07154	0.01317	0.03645	0.01305	0.01143	0.01038	0.00774
**Ltbp2**	0.00000	0.00562	0.64904	0.41725	3.56036	0.00878	0.00652	0.00449	0.00000	0.93086	0.02147	0.16155	0.86690	0.00986	0.00598	0.00661
**Ltbp3**	0.01227	0.00782	0.00778	0.85033	0.00862	0.00678	0.00692	0.00000	2.92878	1.70960	1.25591	0.05082	0.00911	0.00266	0.03013	0.01413
**Ltbp4**	2.62144	0.00534	0.00000	4.26926	0.01904	1.38803	0.01373	1.49250	1.46623	4.31295	0.57694	0.22023	2.53961	0.66282	0.07145	2.43683
**Ltf**	0.01039	0.00767	0.01233	0.00456	0.00808	0.00648	0.00721	0.00880	0.01013	12.57147	0.00686	0.00000	0.01022	0.01361	0.00433	0.00735
**Ltk**	0.01186	0.00693	0.00343	0.57091	0.00930	0.00715	1.05331	0.00000	0.06749	0.00000	0.47722	0.01224	0.00723	0.00469	0.47068	0.00694
**Ltn1**	4.65674	4.65977	4.79241	4.07487	3.81948	0.03157	0.77609	7.76938	5.32943	0.83733	4.63251	15.81282	0.73690	5.70023	9.56439	0.01762
**Ltv1**	12.85364	10.68319	15.21429	15.32265	11.03526	8.92110	9.39456	22.15230	20.41046	26.05977	21.21644	15.41479	16.29610	21.47067	19.75970	9.57515
**Luc7l**	9.47368	6.03939	11.48892	8.05962	4.00094	1.67334	8.95290	17.27069	7.89978	18.39975	11.61877	5.38310	11.15388	17.42615	9.18419	2.79632
**Luc7l2**	7.19890	1.52811	2.22668	12.08956	6.71641	3.25116	1.18238	1.74808	17.46869	5.66880	13.25341	4.77536	3.10035	11.81462	10.41225	1.43946
**Luc7l3**	9.01977	9.82666	9.82587	7.81870	8.46206	5.25601	5.22192	12.56639	11.80936	11.85077	4.91473	14.22293	5.68824	16.02767	12.32659	4.14917

**Lurap1**	0.16680	0.00755	0.00576	0.00468	0.00744	0.00704	0.00715	0.00122	0.30542	0.13517	0.49730	0.01386	0.00717	0.02821	0.00000	0.29749
**Lurap1l**	0.00778	0.00764	0.11903	0.26344	0.02064	0.02652	0.00603	0.57778	0.41176	0.00066	0.00589	0.00485	1.92664	0.01651	0.00000	0.32693
**Luzp1**	2.25058	0.87541	6.41713	10.23874	7.15712	2.14085	2.04107	6.62015	9.49484	8.00679	1.55235	2.71245	2.51655	1.52559	7.04015	0.02649
**Luzp2**	0.00751	0.00707	0.00661	0.52064	0.00942	0.00642	0.00694	0.73848	0.00484	0.09919	0.01307	1.70689	0.00529	0.00927	0.00000	0.00694
**Luzp4**	0.01372	0.00799	0.01251	0.08335	0.00949	0.00667	0.00754	0.01359	0.02136	0.00985	0.01380	0.00782	0.01577	0.00853	0.14342	0.00744
**Lxn**	1.44037	0.00469	1.21187	0.06463	0.74395	0.00796	0.94275	0.00178	0.06628	0.45536	0.01830	0.01240	0.00677	0.02015	0.29049	1.65692
**Ly6a**	9.27321	2.07287	12.41221	4.36362	2.59895	0.53312	2.83797	0.27170	1.69443	3.23685	0.04019	0.16169	7.06396	0.01522	0.00000	4.51299
**Ly6c1**	2.11276	0.00582	0.35738	0.00400	0.02044	0.00386	0.15230	0.03857	0.73394	0.37371	0.26150	0.03299	1.67693	0.09041	0.00000	0.18772
**Ly6c2**	0.31223	0.00795	0.00682	0.02697	0.00706	0.00671	0.00740	0.03083	0.01158	0.02904	0.01167	0.00822	0.22229	0.01223	0.01030	0.10360
**Ly6e**	4.85327	2.83708	7.36363	1.47618	5.75546	0.00000	4.61201	4.14188	0.51867	3.75452	0.00000	0.20608	1.76448	0.00000	0.03306	0.72394
**Ly6f**	0.20665	0.00690	1.13319	0.01726	1.64992	0.34731	0.00222	0.52117	0.01070	0.00000	0.00782	0.00000	9.90710	0.01948	0.00000	2.06863
**Ly6g**	0.04668	0.00830	0.02909	0.07146	0.01665	0.00800	0.01862	0.04258	0.00990	0.00846	0.02865	0.00839	0.18151	0.01280	0.00916	0.11399
**Ly6g5b**	1.80972	0.00571	0.60565	0.34479	0.00368	0.00767	0.00569	0.00539	2.13275	0.79183	0.00000	0.45970	0.30777	0.03615	0.00000	0.01223
**Ly6g5c**	0.01209	0.00836	0.01002	0.34227	0.00923	0.00688	0.00812	0.05100	0.04099	0.31678	0.00895	0.04603	0.01083	0.00636	0.48221	0.00733
**Ly6g6c**	0.27045	0.00714	1.25735	0.01101	0.00500	0.00764	0.00613	0.08158	0.01219	0.00055	0.01115	0.00597	1.30254	0.01797	0.01056	0.01276
**Ly6g6e**	2.89105	0.32720	0.33405	0.14409	1.67638	0.00295	0.00505	0.61809	0.24623	0.43178	0.31101	0.10213	0.28638	0.01026	0.05186	0.24471
**Ly6i**	0.27413	0.00831	0.16101	0.69569	0.00715	0.00657	0.07424	0.29560	0.22388	0.03686	0.00927	0.00850	0.06245	0.00952	0.11710	0.01361
**Ly75**	0.17812	0.00827	0.00641	0.27014	0.01754	0.00674	0.00724	0.01062	0.00743	0.00610	0.01257	0.01711	0.01252	0.00684	0.52163	0.00735
**Ly96**	0.01132	0.89991	0.06694	0.11450	0.00804	0.05658	1.13315	0.00000	0.35554	0.09979	3.35637	0.19017	0.02966	3.11827	0.00000	0.00615
**Lyar**	18.58910	6.49810	12.34033	20.40349	11.01479	10.03032	9.53108	22.00323	24.06699	31.40369	21.25899	10.85804	18.86561	3.11412	6.70713	9.64039
**Lyn**	1.23067	0.00652	0.56321	0.48442	0.00421	0.00816	0.00682	0.55505	0.05583	1.04381	0.86103	0.01026	0.01633	0.00489	0.03581	0.57741
**Lypd1**	0.33615	0.03888	0.23824	2.44004	0.22269	0.03011	0.07742	0.00000	4.03531	0.02557	2.75381	0.15292	0.05730	1.04588	0.07095	0.06987
**Lypd3**	0.00946	0.00828	0.95380	1.96538	0.00707	0.06573	0.00741	0.01348	0.00000	0.34870	0.01125	0.02910	0.02087	0.00885	0.00424	0.35492
**Lypd5**	0.01367	0.00781	0.01147	0.01045	0.00935	0.00668	0.00744	0.01222	0.01261	0.01187	0.01223	0.00726	0.01294	0.13505	0.01480	0.00745
**Lypd6**	0.01358	0.00753	0.01100	0.95895	0.00955	0.00627	0.00728	0.01068	0.01215	0.05460	0.00846	0.00426	0.01309	0.00866	1.38872	0.00732
**Lypd8**	0.01478	0.00854	0.01063	0.01066	0.00911	0.00681	0.00809	0.07132	0.01226	0.01717	0.01211	0.00901	0.00982	0.01344	0.00985	0.00737
**Lypla1**	5.08615	5.64836	2.92139	4.03111	2.70428	1.57898	7.00950	11.68265	3.19965	13.93474	6.75231	18.31726	3.28814	12.22637	10.90380	1.86692
**Lypla2**	5.09292	0.01241	0.00000	0.18785	0.05625	0.72460	0.00000	4.80525	0.05367	6.27722	1.92446	0.12775	6.06934	0.01011	0.01228	4.91260
**Lyplal1**	0.05005	0.00769	0.00932	1.58626	0.00808	0.00676	0.00739	0.01284	0.00000	0.16539	0.02088	0.00717	0.01281	0.01015	0.37563	0.00714
**Lyrm1**	1.35495	2.09989	4.26143	0.47433	2.70173	0.01998	2.38070	1.05893	2.01481	1.26456	0.46867	0.04419	3.59191	1.06617	0.07847	0.00890
**Lyrm2**	0.38453	0.00190	3.01757	1.65050	4.00409	4.72133	2.75260	0.07682	0.03637	0.28755	9.37283	0.02908	0.84971	1.25495	3.68786	0.19967
**Lyrm4**	1.25725	1.63656	2.65429	2.94992	3.01722	0.10048	2.43943	1.27696	0.06349	0.75891	1.97860	0.43326	3.05750	1.01796	0.00000	0.04485
**Lyrm5**	3.08406	0.46868	3.83881	0.88543	3.43218	3.18506	7.06044	1.78438	0.92494	0.20963	2.23088	3.38462	2.24483	0.24555	2.34095	2.38313
**Lyrm7**	0.61042	0.00786	0.94985	0.00672	0.03403	0.13005	0.00633	0.46817	0.20595	0.81898	1.08107	0.00000	0.00389	1.09676	0.04078	0.00640
**Lyrm9**	1.19114	0.07951	1.47248	1.01789	0.02533	0.26108	0.08608	0.05185	0.27420	0.12349	0.73166	0.00978	1.15692	0.66744	0.00000	0.00450
**Lysmd1**	0.00000	0.55120	0.07924	7.28711	0.00813	4.14531	0.00577	0.00549	12.10917	0.47805	0.00000	0.27824	1.38782	0.71936	0.52054	1.48261
**Lysmd2**	0.01200	0.02764	0.32700	0.46515	0.07002	0.00947	1.36078	0.00000	0.00466	0.47715	0.88732	0.15345	0.01007	0.01429	0.00000	0.00707
**Lysmd3**	0.01601	0.00801	0.55773	0.53977	0.01041	3.96706	0.00749	0.86735	0.06939	0.07355	0.26140	0.01686	0.00000	0.01199	0.44766	0.01329
**Lysmd4**	1.24908	0.00465	0.01816	3.26293	0.00550	0.00569	2.55048	0.70509	0.63943	0.82727	0.10663	0.27540	2.30362	0.00719	0.47852	0.35855
**Lyst**	0.01061	0.00754	0.01613	0.36908	0.00962	0.00624	0.00724	0.17678	0.00607	0.04010	0.01014	0.00596	0.00747	0.37209	0.05126	0.02208
**Lyz1**	0.01311	0.00796	0.01179	0.84163	0.00907	0.00642	0.00724	0.01023	0.62860	1.05490	0.00772	0.01452	0.01142	0.00533	0.09457	0.00727
**Lyz2**	0.01296	0.00803	0.01162	0.05924	0.00888	0.00671	0.00737	0.01082	0.14491	1.33940	0.01003	0.01667	0.01150	0.00643	0.18849	0.00745
**Lzic**	2.49456	2.87492	1.73263	4.53578	1.67658	0.00208	1.50399	2.83679	6.96635	0.00000	1.51213	0.24802	0.49501	0.20397	1.94677	0.46278
**Lztfl1**	0.96826	1.43254	2.23958	1.58728	0.00376	0.00387	0.18900	0.90997	7.63750	1.56336	0.94719	11.97119	0.00000	6.73317	7.29979	0.01790
**Lztr1**	1.84450	1.50644	1.82683	3.84976	4.76764	0.02365	1.60150	7.76627	4.13803	3.76022	7.56104	0.04858	5.22607	0.04823	2.91977	2.47761
**Lzts2**	0.20826	0.00840	0.00000	0.10220	0.01677	0.55096	0.03955	1.54453	0.70010	7.52840	0.93859	0.00000	0.01035	0.00380	0.02895	0.01459
**Lzts3**	0.05621	0.02904	0.01869	0.25020	0.00812	0.02675	0.02759	0.01055	0.58613	0.03184	0.04880	0.00527	0.02117	0.14505	0.14557	0.01450
**M6pr**	11.63514	8.47065	12.35403	6.33532	11.06229	4.68768	10.88321	16.02788	9.24984	10.27898	9.14447	13.43528	9.44205	16.48919	13.02579	2.51130
**Maats1**	1.70705	0.00603	0.00349	0.01055	0.00466	0.00667	0.00608	0.01010	0.01239	0.01436	0.01225	0.03703	0.01139	0.01110	0.01677	0.00550
**Mab21l3**	0.00979	0.00780	0.04555	0.01046	0.00955	0.01666	0.00725	0.14715	0.01222	0.06335	0.01175	0.00702	0.00906	0.01164	0.00816	0.00747
**Macf1**	10.37543	3.66875	6.77463	4.60011	4.13386	3.86318	3.39933	13.61127	10.11535	14.13704	8.98729	0.95702	5.13520	11.91251	4.45848	2.71407
**Macrod1**	0.01323	0.00810	0.01756	0.00789	0.00909	0.00751	0.00752	0.01213	0.03389	0.06759	0.05849	0.00857	0.04726	0.01149	0.01080	0.00736
**Macrod2**	2.10208	2.07124	0.48861	2.56954	0.99098	0.00303	0.09567	0.01001	1.19825	0.00389	0.00000	0.03397	0.08898	0.00009	0.21685	0.00359
**Mad1l1**	7.18521	1.22721	4.51947	3.54960	10.53037	3.91905	2.32296	8.55762	1.61604	1.90654	7.56702	0.60331	2.86400	0.00190	0.10489	2.98694
**Mad2l1**	10.26076	11.34290	8.78504	12.38838	12.68324	12.65020	4.46657	14.03239	10.65331	13.57899	9.85455	10.14448	4.86637	16.67967	11.78075	4.76656
**Mad2l1bp**	4.17804	6.24559	10.19763	21.24817	3.10031	5.49610	6.73564	20.54944	30.43064	31.16987	12.09300	15.56040	15.73507	14.50003	30.25491	7.58894
**Mad2l2**	12.41523	7.31025	6.96355	6.54899	6.48450	9.05664	8.72662	7.82544	11.46386	27.85759	10.05284	4.91779	5.77499	0.00000	1.26140	1.37494
**Madd**	0.10600	0.00700	0.23792	1.90518	0.01545	0.22871	0.01719	1.87286	0.26615	1.82362	1.09985	0.11567	0.08070	0.04144	0.24796	1.16816
**Maea**	12.95055	6.89936	6.52875	6.44172	6.54725	7.45338	9.58385	14.21327	6.30490	9.78739	5.52203	4.14161	12.34254	0.00000	0.00000	8.60930
**Mael**	0.00768	0.00627	0.06979	3.83366	0.00979	0.00597	2.43292	0.00000	0.84133	2.36418	0.97523	0.17043	0.17377	0.00000	1.37936	0.00686
**Maf1**	10.39327	2.88467	3.00609	3.11441	11.93819	3.59990	3.53714	2.35124	2.91019	6.68624	0.78951	0.28178	7.13582	0.38454	3.23622	1.38803
**Mafb**	0.01139	0.00774	0.00966	0.13024	0.00942	0.01332	0.00733	0.06895	0.01283	0.60283	0.01019	0.00599	0.01076	0.07564	0.06408	0.00745

**Maff**	0.74234	0.00682	0.01176	0.31521	0.01538	0.02880	0.00586	0.00007	0.44641	0.31010	1.40455	0.00312	0.31357	0.53932	1.36962	0.00642
**Mafg**	0.82705	0.23389	1.82019	7.61630	0.00345	0.00371	2.69825	3.16754	7.05462	1.64459	3.13173	0.25980	0.74009	0.29196	4.25182	2.10202
**Mafk**	0.40745	0.33137	0.12736	2.86801	0.00858	0.01587	0.79215	0.58282	7.17377	1.41076	0.93244	0.08259	0.88861	0.01513	0.30398	0.00518
**Magea1**	0.32195	0.00728	0.06001	0.43270	0.00716	0.07583	0.00692	0.01220	0.00521	0.06523	0.01200	0.00631	0.15147	0.00793	0.34342	0.00686
**Magea10**	0.01367	0.00769	0.01138	1.09790	0.00940	0.00629	0.00730	0.01098	0.06813	0.00511	0.01096	0.00551	0.01279	0.00590	1.06790	0.00715
**Magea2**	0.36563	0.00715	0.02282	0.17306	0.00717	0.11990	0.00679	0.01104	0.00800	0.07972	0.01015	0.04290	0.26930	0.00845	0.78547	0.00680
**Magea3**	0.19476	0.00766	0.00667	0.01675	0.00761	0.72696	0.00713	0.01873	0.01200	0.00675	0.01168	0.00722	0.14485	0.01954	0.23767	0.00681
**Magea4**	0.03781	0.00785	0.01033	0.01067	0.00822	0.00662	0.00741	0.01443	0.01211	0.01207	0.01250	0.00800	0.03763	0.01324	0.05249	0.00723
**Magea5**	1.41461	0.00590	0.04397	0.00000	0.00525	0.04182	0.01515	0.01109	0.00758	0.00000	0.00718	0.03790	1.56486	0.01283	3.36979	0.01208
**Magea6**	0.17451	0.00760	0.03152	0.00815	0.00740	0.00791	0.00708	0.01242	0.01115	0.00424	0.01147	0.01754	0.08314	0.00876	0.45791	0.00696
**Magea8**	3.78776	0.00341	0.31298	0.52414	0.00000	0.00527	0.00415	0.00600	0.00372	0.00000	0.00930	0.06712	0.29969	0.00991	2.64769	0.00323
**Mageb16**	8.26007	6.41751	8.39602	0.00000	8.45977	3.38184	5.11738	3.30911	1.64179	4.77456	0.45964	0.18920	3.08960	8.74185	0.00000	7.42736
**Mageb16.ps1**	1.10108	0.66930	0.96163	0.06346	0.91688	0.41778	0.52423	0.32857	0.08825	0.46372	0.01132	0.09536	0.30627	1.21921	0.05756	0.92070
**Mageb3**	0.01458	0.00813	0.05134	0.00444	0.00896	0.00579	0.00776	0.02134	0.65396	0.86800	0.10411	0.01352	0.00915	0.01041	0.45128	0.00705
**Mageb4**	0.01244	0.00751	0.01239	1.40193	0.00891	0.00683	0.00715	0.00950	1.51863	0.71644	0.00000	0.55548	0.01089	0.00191	1.01320	0.00674
**Maged1**	1.93104	1.03190	0.00000	2.43483	6.63419	3.76814	0.00577	1.13903	1.08839	4.04998	0.00000	0.10763	0.00000	0.32590	2.32992	12.61584
**Maged2**	0.13369	0.00795	0.00000	0.00618	0.03350	0.00865	0.00706	1.24118	0.01008	1.94205	0.00000	0.09950	0.00000	0.01385	0.00000	2.27383
**Magee2**	0.00959	0.00885	0.00935	0.00702	0.01049	0.00717	0.00835	0.32777	0.01051	0.00453	0.01081	0.00771	0.06748	0.00786	0.64426	0.00761
**Magef1**	0.01372	0.00825	0.01110	0.02406	0.00926	0.01347	0.01811	0.01390	0.02611	0.07231	0.01214	0.00875	0.01299	0.03049	0.01942	0.00742
**Mageh1**	0.01335	0.00805	0.01279	0.29448	0.00949	0.00704	0.01820	0.01344	0.00526	0.44330	0.01265	0.00778	0.02254	0.01248	0.01724	0.00748
**Magi1**	2.77743	0.00575	0.00000	1.69330	0.01873	0.01354	0.00567	0.35178	1.05188	0.30143	0.00000	0.06852	0.64812	0.00722	0.55153	0.54336
**Magi2**	0.21280	0.00682	0.00968	1.12997	0.00734	0.04719	0.00698	0.78636	0.73063	0.11437	0.18288	2.30272	0.01315	0.00694	0.00000	0.02116
**Magi3**	1.18468	0.00564	0.00000	0.59873	0.00542	0.00762	0.01608	0.00506	0.91185	0.00000	0.00000	0.85151	0.00994	0.04675	0.11584	0.47867
**Magoh**	14.37306	9.53347	11.48673	20.75281	13.68111	4.91207	6.33285	9.90670	33.81367	21.02706	24.20900	4.29480	17.62737	25.25955	18.88411	5.16267
**Magohb**	2.61687	0.01331	0.36942	1.54696	2.45276	0.02017	1.26545	0.83445	0.49974	4.51465	3.39575	0.19845	1.57611	3.36621	2.99145	1.12658
**Magt1**	4.02047	4.38952	9.62380	7.26306	7.46535	0.18050	7.20531	2.68282	5.14644	6.32401	7.11080	9.12562	5.19964	12.85335	7.61763	2.35141
**Majin**	0.76778	0.00770	0.50951	0.00000	0.04134	0.00484	0.00428	1.35687	0.00741	2.45136	3.71608	1.09468	3.18910	0.44340	2.12793	0.01174
**Mak**	0.01379	0.00765	0.01176	0.60597	0.00938	0.00646	0.00738	0.01194	0.00363	0.00665	0.01270	0.01655	0.01274	0.00719	0.40569	0.00726
**Mak16**	13.52442	6.04538	10.28856	15.82540	4.25935	1.62333	5.26586	9.04795	13.19016	8.54731	7.34286	1.26468	5.03652	10.99942	17.49397	4.81482
**Mal**	0.03046	0.00763	0.00825	0.00941	0.00838	0.00696	0.00722	0.01114	0.01205	0.28308	0.01152	0.19026	0.01269	0.01139	0.00683	0.00770
**Malat1**	2.15620	4.54094	3.35323	4.89101	0.13763	0.28337	0.14017	2.55886	4.29935	0.14361	2.45915	3.53865	3.26478	10.21994	9.69434	2.32960
**Malsu1**	2.51338	0.12395	0.67954	1.15100	4.56097	0.00362	0.02084	0.00000	1.94369	3.79778	11.71268	0.05426	0.24343	0.52996	0.00000	0.51078
**Malt1**	0.25337	2.57645	1.00047	0.91858	0.05461	0.19479	0.15527	1.19356	3.21323	1.46568	2.26348	0.15220	0.48937	1.20618	1.51870	0.01912
**Mamdc2**	0.02286	0.00752	2.68366	0.02544	0.00570	0.00553	0.00709	0.01057	0.01214	0.01100	0.02757	0.00621	0.01266	0.01423	0.00000	0.00638
**Mamdc4**	0.01074	0.01795	1.03159	0.30489	0.00783	0.00566	0.00709	0.13408	0.30679	0.00624	0.00829	0.00619	0.00806	0.07713	0.00000	0.00697
**Maml1**	1.87453	2.66113	0.47318	4.05617	0.05275	0.00288	0.01396	1.21544	9.84581	1.42872	0.00000	3.79511	0.99214	0.00000	0.04792	0.16145
**Maml2**	0.01656	0.00676	0.00293	0.00350	0.00892	0.04911	1.79096	0.01008	0.13270	0.49376	0.00973	0.00481	0.00966	0.00623	0.12358	0.00702
**Man1a2**	2.26111	1.44821	3.76681	4.56592	3.40872	0.44631	3.27637	5.51725	3.47993	2.96109	3.03153	1.29452	1.84589	11.83012	4.92341	0.70095
**Man1b1**	3.29280	0.91681	1.53547	7.86870	0.00794	2.23893	2.76628	3.48714	8.23192	3.96370	5.54378	0.99273	2.65923	2.15704	1.19233	1.65290
**Man1c1**	0.30370	0.00745	0.01011	1.67152	0.00722	0.00615	0.00756	0.01883	0.34275	0.00822	0.05808	0.00693	0.01692	0.00397	0.23866	0.01371
**Man2a1**	3.73891	1.51225	2.40328	2.56262	6.76010	0.00177	6.10662	0.01111	0.00000	0.00000	0.01704	0.00180	1.49350	0.02044	0.36620	2.29291
**Man2a2**	1.17855	0.08617	0.00000	13.77424	0.00573	0.10184	1.75351	0.61077	11.93978	0.14415	0.16397	0.30482	0.01796	0.16895	0.08373	0.02467
**Man2b1**	6.25208	2.48341	5.82211	3.19265	2.47830	7.54388	6.56994	6.28899	2.09050	1.94353	9.37885	0.51916	5.33074	0.58799	1.39385	0.03421
**Man2b2**	0.44387	0.00694	0.00171	0.00478	0.00686	0.00847	0.08595	1.24354	0.01004	0.41225	0.56049	0.00125	0.14824	0.00488	0.05498	0.00655
**Man2c1**	4.95360	0.01024	3.15515	0.10632	0.09634	0.53827	2.43558	1.43903	3.11989	1.35403	0.67159	0.17205	5.00014	0.00380	0.11391	0.01331
**Man2c1os**	0.01421	0.00797	0.13299	2.15368	0.00881	0.00653	0.00714	0.01020	0.82404	0.44571	0.00777	0.00509	0.01068	0.00950	0.00092	0.00697
**Manbal**	4.67487	2.79696	9.58419	0.43347	0.00000	2.40584	6.19543	8.66619	2.00796	8.83564	0.96764	0.45130	2.03904	0.36024	0.86039	2.10720
**Manea**	0.59298	0.00776	0.01714	0.23508	0.02754	0.00736	0.03015	0.12855	0.11992	0.06240	0.83723	0.04492	0.30819	1.06636	0.03548	0.00630
**Maneal**	0.99054	0.00701	0.49503	0.85682	0.00513	0.00449	0.00706	0.01695	0.77834	0.46768	0.44058	0.06387	0.00622	3.89070	3.20142	0.00609
**Manf**	7.12411	0.70896	7.82124	9.00159	0.55375	8.96281	2.57159	7.64113	12.08849	15.07079	10.17630	1.08302	18.29620	1.59744	2.40391	9.10460
**Mansc1**	0.01374	0.00797	0.01208	0.23159	0.00927	0.00672	0.00756	0.01379	0.01471	0.02395	0.02620	0.00811	0.01264	0.00879	0.05189	0.00737
**Mansc4**	0.55379	0.00708	0.00470	0.01064	0.00664	0.00694	0.00665	0.00889	0.01240	0.00612	0.01240	0.00740	0.77836	0.01325	0.01050	0.00623
**Maoa**	2.31446	0.00530	0.00000	0.29740	0.02597	0.08235	0.03538	0.02629	0.67865	0.00000	0.04786	0.00196	0.75769	0.56530	0.13634	1.36792
**Map10**	0.13368	0.00727	0.00529	1.57719	0.00770	0.00647	0.00733	0.00897	5.60435	0.00281	0.00000	0.00674	0.01278	0.00580	0.39221	0.01356
**Map1a**	0.49100	0.00713	0.72999	0.00535	0.00564	0.00511	0.00680	0.00000	0.01113	0.86668	0.40928	0.02265	0.00654	0.01208	0.00000	0.00665
**Map1b**	0.08305	0.00682	0.00195	1.38161	1.63237	0.00790	0.00738	0.00948	0.31747	4.07386	0.00694	0.01162	0.05240	0.00323	0.92890	0.00724
**Map1lc3a**	0.79877	1.91912	4.34824	4.54213	4.70355	3.81479	2.87240	8.88381	10.57932	24.58643	22.50402	1.53094	8.48476	0.06251	5.05459	0.29073
**Map1lc3b**	46.44801	29.47417	49.21889	35.32413	33.29238	19.75516	29.43826	52.02937	45.12898	62.73587	54.46611	27.33657	46.77590	27.42221	38.06402	20.51227
**Map1s**	2.97661	0.00407	0.23911	1.82368	0.76787	0.03187	0.00357	0.79295	0.67744	8.10180	1.52175	0.09319	1.66110	0.00457	0.00000	2.70454
**Map2**	0.01261	0.00790	0.00903	0.76639	0.00894	0.00649	0.00724	0.00860	0.00000	1.24996	0.04506	0.00303	0.01009	0.35991	0.26158	0.00694
**Map2k1**	9.23219	3.82227	3.06680	7.69765	11.80419	2.39485	1.34078	6.89809	8.19724	10.73698	3.25404	1.66430	1.71753	9.20817	11.30896	2.99262
**Map2k2**	13.36569	0.90926	2.84054	3.54729	1.59754	9.95765	3.18843	26.06566	4.65796	22.55324	8.82807	4.29540	15.32946	0.02929	0.33601	8.96320

**Map2k3**	8.52915	5.86369	4.64687	2.21206	4.48860	3.73711	8.47595	6.71085	1.32882	5.59618	3.88612	0.53442	5.95781	0.00000	2.83330	2.10038
**Map2k3os**	0.45903	0.00714	0.05922	0.36610	0.00710	0.00640	0.00695	0.27904	0.00390	0.01366	0.01054	0.00710	0.00617	0.01079	0.00681	0.00674
**Map2k4**	3.19644	3.50159	6.75059	5.14695	8.39922	4.73763	4.34010	3.79663	3.98280	4.37273	1.81226	12.97298	2.11054	0.16368	2.19161	2.19507
**Map2k5**	0.68809	2.70952	0.00000	0.09781	0.00801	0.35849	0.00569	0.00759	0.06346	0.01290	0.01020	0.01643	0.00626	0.00641	0.00000	1.54697
**Map2k6**	6.83135	2.85058	3.09181	0.33204	3.11427	4.51897	2.61091	1.40021	0.00000	0.98402	0.00000	0.18903	1.92989	0.00680	1.36827	0.03219
**Map2k7**	5.72235	0.00000	1.27866	2.76015	0.06200	4.41966	2.57234	4.40962	2.83869	6.83657	3.66900	0.35277	2.30679	0.98000	3.63300	2.75445
**Map3k1**	0.05828	1.95535	0.05539	0.27917	0.00773	0.00834	0.01605	0.00966	0.00671	0.44751	0.00541	0.00000	0.57331	1.63107	0.20540	0.21357
**Map3k10**	0.01678	0.00799	0.01597	0.34046	0.00878	0.00626	0.00716	0.06198	0.48949	0.12599	0.35624	0.03606	0.13356	0.00763	0.01364	0.02149
**Map3k11**	2.63468	0.03553	0.20453	2.77145	0.02919	0.00316	0.00431	0.38723	0.06484	2.19228	1.27402	0.39568	0.66082	0.00000	0.20892	0.05401
**Map3k12**	0.71201	0.92763	0.43148	0.48089	0.00650	0.00543	1.32846	0.01329	0.12882	0.06378	0.01483	0.03754	0.17372	0.02827	0.17157	0.11651
**Map3k13**	0.04632	0.00728	0.00592	0.03388	0.00810	0.00717	0.77287	0.01070	0.01001	0.01265	0.01256	0.00715	0.01097	0.01201	0.00973	0.00724
**Map3k14**	0.01321	0.00778	0.01658	0.59110	0.00933	0.00609	0.00739	0.01123	1.15754	0.00549	0.00000	0.00394	0.01293	0.00454	0.93837	0.00718
**Map3k15**	1.47499	0.00574	0.00179	0.00965	0.03363	0.00741	0.00635	0.09221	0.01156	0.02407	0.00980	0.04950	0.06757	0.40477	0.24619	0.00622
**Map3k2**	1.06688	0.00468	0.72614	0.00000	0.00436	0.67952	1.63329	0.66006	1.53039	0.72501	0.68007	1.72654	1.09655	0.62255	3.39268	0.57706
**Map3k3**	4.12020	0.04621	0.58064	0.47742	0.78492	0.09347	1.84018	1.86365	4.08795	5.19559	0.00000	0.05610	1.07738	3.38782	5.65710	0.67312
**Map3k4**	1.88032	2.26422	3.65073	0.98847	0.85433	0.53578	0.18003	3.00959	0.08071	0.35887	0.00000	0.10472	4.33034	1.60130	0.43729	2.85066
**Map3k5**	1.28183	4.86591	0.00000	2.01598	0.29065	3.02344	0.49690	1.49662	1.02278	1.67139	1.37319	0.05462	2.57378	1.91666	4.21690	0.03063
**Map3k6**	1.85185	0.39812	0.00000	4.16048	5.99426	0.00456	0.00620	0.35388	2.28091	0.00000	0.00000	0.01923	0.00942	0.10309	0.01840	5.19505
**Map3k7**	2.38170	1.24323	0.22256	3.38056	8.37420	2.36971	0.34292	3.43668	3.45625	6.43768	4.63383	7.84210	2.12347	5.31680	7.90064	3.38890
**Map3k9**	0.00428	0.01893	1.35080	1.01353	0.00603	0.00898	0.04127	0.94364	0.00000	1.51714	1.83367	0.03683	0.00000	0.51438	0.65935	0.94713
**Map4**	10.36605	5.41804	13.66813	11.16182	12.80301	11.25653	6.97595	12.17579	10.07413	7.70640	12.34603	0.70416	16.74442	7.45833	2.40887	5.46744
**Map4k1**	0.69649	0.02667	0.83195	1.51536	0.00751	0.00488	0.55698	0.72606	1.91373	2.28536	4.10574	0.01298	1.95674	0.00000	0.03879	0.00473
**Map4k2**	3.40252	0.00400	0.25062	0.81133	0.00646	0.01425	0.23991	0.94034	0.17255	0.14982	1.02191	0.00482	2.21133	0.00523	0.19306	0.73603
**Map4k3**	1.44856	3.76889	2.04791	1.02482	0.60878	0.52993	2.08453	1.23954	1.37194	0.59846	3.36762	1.10784	0.00000	7.86758	2.63854	0.14941
**Map4k4**	10.36924	1.34834	3.61431	0.72045	8.45626	5.49033	5.06667	3.08364	0.52680	1.19296	2.89563	2.70506	2.89863	0.08416	0.30136	8.75048
**Map4k5**	2.51533	5.11853	0.35891	17.18061	3.45226	2.52610	3.41201	4.09043	10.79096	13.52265	6.22950	7.15960	1.94820	9.22407	18.32318	2.75625
**Map6**	0.42161	0.00773	0.00486	1.24234	0.00867	0.00702	0.01725	0.01184	1.30710	0.04472	0.00000	0.01523	0.01290	0.00493	0.13093	0.00700
**Map6d1**	0.01220	0.00766	0.00091	0.10653	0.00897	0.00657	0.00682	0.00000	0.15610	1.19065	1.16078	0.00361	0.00000	0.00416	2.78161	0.00724
**Map7**	9.10225	7.06831	8.81271	2.45580	5.27066	7.29257	10.36724	18.68007	3.65444	16.62567	17.73573	18.58826	10.08044	12.12981	10.36732	4.85667
**Map7d1**	0.67666	0.00746	0.79227	1.30408	0.02236	2.96064	0.15275	0.00725	0.00000	0.01720	0.00842	0.06434	0.19619	0.00966	0.00000	0.00523
**Map7d2**	0.97997	0.00379	2.39237	0.71057	0.01160	0.00573	0.00284	1.78135	1.65064	4.90032	2.76182	7.28181	2.56960	12.89433	12.52019	0.00288
**Map9**	0.01621	0.00725	1.68782	0.30769	0.00632	0.00542	0.00660	0.00000	0.00384	0.00000	0.74116	0.41254	0.00000	0.00651	1.51578	0.00627
**Mapk1**	13.63608	9.58694	10.96660	5.89375	8.95745	2.00337	5.09062	7.12427	10.12539	8.35803	6.65675	6.39709	5.16262	7.96968	11.39651	5.49964
**Mapk10**	0.01304	0.00796	0.01019	0.00606	0.01848	0.00667	0.00750	0.00868	0.01175	0.00474	0.23274	0.01724	0.01566	0.00717	0.05171	0.00724
**Mapk11**	0.16688	0.00866	0.00651	3.29539	0.01710	0.00681	0.00717	0.01073	1.34314	0.09409	0.00000	0.00489	0.01315	0.08087	0.00958	0.00712
**Mapk12**	1.52434	0.00577	0.00308	0.38635	0.00502	0.00701	0.00631	0.00686	0.14759	0.19257	0.01076	0.00641	0.01185	0.01434	0.11875	0.00571
**Mapk13**	0.01737	0.00556	0.15162	2.24092	2.50768	0.00788	0.01477	2.57056	0.46285	2.15035	3.98358	0.06233	2.46547	0.12832	0.48940	0.01949
**Mapk14**	5.24302	2.94887	8.46166	16.23156	8.36944	0.01893	6.64656	8.59043	10.26985	7.21575	1.34568	1.40313	2.85124	8.20197	10.76334	4.36055
**Mapk1ip1**	1.88976	1.04948	2.01230	1.68571	0.00375	2.47700	3.89063	1.30732	0.51019	0.00000	1.06411	5.92826	0.03448	1.00763	0.16003	3.07968
**Mapk1ip1l**	15.17719	6.43385	8.55683	10.76870	5.73374	4.87058	6.75839	11.44168	13.15270	14.35112	11.46286	2.08654	8.85673	18.36092	9.29342	3.91306
**Mapk3**	5.39487	0.00000	1.18351	5.00787	0.50741	0.58896	0.28171	6.51165	0.10767	3.94257	0.64578	0.56689	5.35575	0.00043	0.34141	1.30050
**Mapk6**	3.91587	8.33028	8.22879	16.76508	0.61144	6.92901	6.93191	13.22674	16.93900	22.57836	14.93309	20.58860	8.26636	11.20325	13.45515	3.93623
**Mapk7**	5.48889	2.38284	0.38311	1.01034	0.07543	0.90355	0.60306	0.38821	3.18139	3.10125	1.18404	0.00853	1.84655	0.00000	0.60125	0.46445
**Mapk8**	2.14143	0.02345	0.54648	11.65711	1.86366	2.99195	0.01373	0.53465	5.82269	0.71816	0.92864	7.09268	0.63512	3.46843	3.99973	3.40547
**Mapk8ip1**	0.00966	0.00734	0.00000	0.25217	0.00964	0.38087	0.47509	0.06538	0.39290	0.83703	0.41292	0.02966	0.00743	0.00705	0.01263	0.00746
**Mapk8ip3**	3.56534	0.00173	2.53202	1.60039	0.13928	1.03426	3.05699	1.81230	5.61627	1.03221	0.00000	0.01250	0.36526	0.03192	0.00005	0.08315
**Mapk9**	1.21235	2.42984	1.50522	2.25215	0.01382	2.58557	2.38841	0.64448	4.72885	0.42492	1.77527	0.03812	1.43820	0.00000	4.28616	1.12104
**Mapkap1**	1.30452	0.00000	2.19041	11.14028	4.57480	0.89383	4.10582	5.14248	4.94663	4.14331	1.40081	0.19778	1.25604	0.09601	1.47697	0.00167
**Mapkapk2**	1.88284	0.05339	0.85054	4.16389	0.00326	3.57922	3.06206	1.03769	4.41584	2.51389	0.25031	0.65350	2.82174	0.00000	0.82172	0.07368
**Mapkapk3**	0.00679	0.00822	1.24887	0.19584	0.02246	0.00923	0.00595	2.33965	0.19865	2.16598	1.50109	0.08008	2.14215	0.10428	1.97150	0.00593
**Mapkapk5**	6.01925	2.95137	4.95980	14.04229	7.59116	0.02100	2.52239	4.83943	11.65619	8.93181	6.75350	1.99626	4.34183	1.12684	8.14932	3.92222
**Mapkbp1**	0.00696	0.00797	1.10205	5.66534	0.00721	0.01598	0.00603	0.00000	5.43116	1.03962	1.38577	0.03878	1.16745	0.06962	5.07640	0.00491
**Mapre1**	20.03581	15.73641	20.86305	15.48056	14.63607	13.10521	18.88241	26.21799	14.10827	25.90605	16.71360	19.37367	18.28625	23.92639	18.79122	7.68932
**Mapre2**	1.21142	0.00784	0.81809	2.54100	0.25378	0.16449	0.00655	0.69869	0.83809	0.44482	0.36903	0.13769	1.05517	0.27885	0.30365	0.25115
**Mapre3**	0.08310	0.00789	0.23008	0.20001	0.00739	0.00907	0.03749	0.01796	0.00711	0.00481	0.01253	0.02728	0.54484	0.03421	0.00106	0.12028
**Mapt**	4.48085	1.82772	2.76539	0.03136	1.52194	0.01550	2.02911	5.03305	0.00584	5.10585	4.70851	0.72366	1.95957	1.95099	1.42248	1.38279
**X2.Mar**	9.22504	0.02844	0.00000	9.42473	0.04068	2.23604	0.00000	1.84582	7.89367	6.77205	8.13257	2.15840	0.12216	2.99722	3.04520	0.00000
**X2.Mar.1**	2.42067	0.02718	1.73597	3.77476	0.00000	0.00546	0.00576	0.38711	0.75878	4.82430	0.36695	0.05169	0.02375	0.05512	1.38347	0.03984
**X3.Mar**	0.00868	0.00772	0.00766	0.46765	0.51386	0.00696	0.00749	0.01300	0.41745	0.01229	0.01021	0.00724	0.01085	0.01160	0.00414	0.00718
**X5.Mar**	7.79810	4.39815	5.17521	22.42224	5.66666	2.60288	5.77580	7.89141	13.40466	11.02698	15.03387	9.79140	5.40804	11.26835	12.93016	0.21289
**X6.Mar**	4.70492	4.28075	5.74849	6.65903	3.71001	7.33791	2.55620	6.29383	5.51228	5.29675	9.76271	9.81619	5.33749	12.12362	7.15771	1.90435
**X7.Mar**	0.52837	0.70095	2.24631	0.94352	0.06813	0.41230	0.25033	1.03225	0.61944	0.00000	0.00000	3.68748	0.65151	6.01020	3.50321	0.26731

**X8.Mar**	0.89850	0.00725	0.53989	3.06661	0.01084	0.00436	0.00593	0.00000	0.01219	0.08973	0.26616	0.08093	0.62063	0.01126	0.50239	0.00531
**X9.Mar**	0.06990	0.00828	0.00396	0.57035	0.00779	0.00744	0.00755	0.08839	1.45758	0.59158	0.93771	0.02229	0.00129	0.00276	0.23745	0.00692
**Marcks**	8.42458	17.12675	11.78899	0.40477	14.67232	7.24924	7.28921	0.29554	0.41334	1.33357	7.57342	5.06658	4.83723	0.08313	0.00000	6.51492
**Marcksl1**	17.88358	6.19925	14.76564	2.29828	3.82311	14.13621	12.48384	21.74997	3.36118	16.86281	16.43216	1.74881	21.75672	8.05350	8.65004	12.99411
**Marf1**	2.58684	0.08897	2.99642	7.08225	2.37614	1.10146	1.10449	3.85391	4.03894	1.21189	3.96093	1.17573	0.69864	4.25015	5.09945	0.00835
**Mark1**	0.01150	0.00591	0.00000	1.70707	0.01276	1.02423	2.96033	3.51170	0.76312	5.50641	8.66228	0.34890	0.00000	0.04607	1.45249	0.09568
**Mark2**	6.84341	0.50171	2.01308	4.12185	2.17931	4.66103	0.80971	4.56268	4.28821	15.72665	1.65399	0.28076	3.37192	0.01570	3.56246	4.58827
**Mark3**	8.31704	7.47884	7.36862	7.62026	10.40216	7.64302	6.53290	15.88972	13.52963	16.30728	7.02382	6.26682	5.44883	0.92112	9.10861	1.64370
**Mark4**	0.04092	0.00836	0.01824	0.17606	0.01556	0.00667	0.00766	0.01448	0.02923	0.06325	0.01493	0.00695	0.04813	0.00589	0.12418	0.08549
**Mars**	18.43649	13.70197	14.51811	3.38741	11.76818	6.76699	11.09547	18.75214	3.28689	12.40790	10.76950	7.07430	14.87340	0.59278	5.99887	9.40144
**Mars2**	0.23563	0.01646	0.00000	3.45031	0.01488	0.02923	1.12002	0.88883	6.26247	3.20424	0.42976	0.20231	0.22975	4.17226	7.66307	1.79305
**Marveld1**	0.01599	0.00777	0.01064	0.33829	0.00980	0.00642	0.00730	0.01011	0.00645	2.13472	0.00859	0.01058	0.01050	0.05269	0.75331	0.02153
**Marveld2**	0.22601	0.00716	0.00000	3.49384	0.00713	0.13610	0.00655	1.61503	4.50737	2.64693	0.85109	0.05889	0.16544	5.38901	0.60569	0.20239
**Marveld3**	0.01009	0.00754	0.04286	0.00654	0.00936	0.00842	0.00701	0.00770	0.02498	0.54738	0.10389	0.01412	0.55606	0.01780	0.08831	0.00748
**Masp2**	0.02438	0.00748	0.25504	0.54504	0.00830	0.55480	0.00713	0.01005	1.27287	0.00000	0.00000	0.01420	0.00683	0.01972	0.33752	0.00671
**Mast1**	1.32737	0.00516	0.00313	4.99780	0.00505	0.00591	0.00314	1.92284	3.87021	7.94692	7.18311	0.08250	0.08075	0.03711	0.00000	0.03934
**Mast2**	4.07914	3.62115	0.13549	6.40396	0.04185	0.06022	6.94592	4.63751	5.76383	4.86423	2.10337	0.33747	3.83638	0.00000	3.10776	2.70873
**Mast3**	0.15731	0.00604	0.35648	1.83368	1.34257	4.26062	0.00000	2.37342	0.68404	7.58226	8.98925	0.39223	5.80069	0.10753	2.83483	0.00002
**Mast4**	0.93322	0.00523	0.44821	1.76605	0.00550	0.00485	2.25097	0.69776	0.30995	0.05700	0.65377	0.02304	0.01678	0.00456	0.80983	0.00575
**Mastl**	0.11577	0.00421	1.91479	5.46167	4.97202	0.87859	0.02470	5.08543	0.52101	3.87583	3.09480	0.10534	2.54421	11.30720	6.79509	0.03959
**Mat1a**	0.01375	0.00772	0.01200	0.48288	0.00941	0.00645	0.00739	0.01194	0.00357	0.00663	0.01168	0.00669	0.01273	0.00610	0.48892	0.00728
**Mat2a**	12.87707	17.78948	18.60297	14.52660	7.09612	15.28231	16.15284	36.86554	23.65105	40.57692	20.47703	12.59777	21.58494	39.83224	37.46540	11.37329
**Mat2b**	2.26321	4.09525	5.49619	24.81428	1.70969	1.07554	2.14550	5.59808	20.82270	18.17783	9.61148	9.79672	2.14432	12.37285	18.51536	2.66121
**Matk**	0.01374	0.00808	0.01225	0.08387	0.00931	0.00665	0.00753	0.01394	0.06476	0.01509	0.01285	0.00808	0.01271	0.01203	0.00847	0.00740
**Matn2**	0.01332	0.00787	0.00976	0.00423	0.00919	0.00673	0.00724	0.00948	0.49740	0.30721	0.02306	0.01370	0.01247	0.28953	0.00000	0.00778
**Matr3**	6.98509	10.72923	8.53650	4.29272	5.07821	3.96760	5.87948	15.03874	8.58062	9.91078	8.76559	15.69752	5.19512	28.47227	12.44286	3.23403
**Mau2**	3.75358	0.11428	4.00791	1.44056	0.07695	0.14698	3.66358	6.28809	0.89825	4.81641	2.91945	0.22248	1.77043	5.56886	6.37836	0.11679
**Mavs**	3.28713	2.45113	6.23756	1.79719	0.00000	7.77420	0.05266	1.09863	6.75583	4.80178	1.90429	0.35670	0.00000	0.00000	3.18513	1.21662
**Max**	9.75353	3.41215	3.20606	3.33836	3.56129	0.18810	6.24659	7.66662	3.98540	3.71829	3.33518	0.53025	3.21756	0.88726	3.39855	2.02081
**Maz**	3.06779	0.24728	0.06585	0.26413	0.01007	0.00323	0.04971	0.03705	1.23266	0.31152	0.00000	0.00402	0.26484	0.00987	0.04136	2.30536
**Mb21d1**	0.12988	0.00757	1.29666	1.46620	0.02924	0.05662	0.07863	0.02658	0.93856	0.65004	0.83944	0.04467	0.17267	0.50699	0.75231	0.02935
**Mb21d2**	0.01649	0.00788	0.00841	1.73752	0.00850	0.00639	0.00797	0.07942	0.00007	2.13574	0.03455	0.33779	0.00792	0.01390	0.50789	0.16596
**Mbd1**	1.10247	0.00574	0.85943	4.42345	0.69186	0.00585	2.10376	0.21095	0.08482	0.10751	1.32532	0.10554	1.66314	0.00434	1.67205	0.73109
**Mbd2**	0.49645	1.62253	0.29922	2.50903	0.00647	0.00484	1.08687	0.00000	1.53247	0.21757	0.57317	0.09689	1.43480	0.00000	0.00000	0.12408
**Mbd3**	14.84628	5.27235	11.08828	9.17326	13.90001	6.90091	4.17635	13.83124	6.77046	19.55132	9.83930	1.06055	14.31327	0.00000	0.33587	6.08422
**Mbd3l2**	0.00000	0.00729	0.00000	14.70152	0.00849	0.00314	0.00537	0.00000	10.22167	2.89616	2.05952	1.45120	0.73408	13.03835	10.80260	0.00308
**Mbd4**	1.40908	0.02743	0.50639	1.18292	0.21418	0.65407	0.07657	0.47133	0.94024	0.39957	1.14177	0.01295	0.21814	0.66466	1.78750	0.48095
**Mbd5**	0.76196	0.00785	0.00150	2.17778	0.01039	0.00689	0.00768	0.22076	0.83401	0.22872	0.00006	0.00236	0.05591	0.00179	1.47206	0.00689
**Mbd6**	3.26906	0.00285	1.75612	2.76822	2.43493	0.40139	0.02383	1.15891	0.83365	1.76866	2.55655	0.03102	2.59458	0.00000	0.07134	2.14462
**Mbip**	5.43054	4.09521	1.88830	2.56413	3.59111	2.52066	1.33301	1.64367	1.95177	8.64495	0.92000	1.29643	0.45840	0.86447	3.65615	2.55920
**Mbl2**	0.00000	0.00651	0.00000	2.09748	0.01024	0.00599	2.03431	1.36211	0.33038	1.95809	0.51032	0.08237	0.01024	2.52096	2.80971	0.00670
**Mblac1**	1.75300	0.00633	0.00000	0.00655	0.00506	0.20525	0.00649	0.05153	0.01158	1.28544	0.01099	0.04586	0.04310	0.01303	0.00571	0.00577
**Mblac2**	1.11438	0.00638	0.00000	0.00274	0.01655	0.95126	1.10144	0.00562	0.01033	0.96698	0.00748	0.01173	0.02603	0.02890	0.77921	0.00589
**Mbnl1**	0.01250	0.00796	0.00791	0.14395	0.00915	0.01238	0.00732	0.01051	0.00472	0.98539	0.00561	0.06628	0.01156	0.00569	3.52503	0.01468
**Mbnl2**	0.00450	0.43275	1.44370	0.00978	0.00561	0.66965	0.00668	0.00747	0.01252	0.00310	0.00916	0.01571	0.39255	0.06073	0.00000	0.00611
**Mbnl3**	0.00000	0.00845	1.14964	0.18739	0.05382	2.51142	0.00645	0.37366	0.03350	2.61900	1.26061	0.12395	1.32724	0.80102	1.36780	4.65660
**Mboat1**	1.28222	0.03061	0.85142	0.00137	0.08678	0.35865	0.66365	0.99140	0.00857	0.97637	1.59073	0.02628	0.34124	2.69228	0.23842	0.01210
**Mboat2**	3.76005	6.96716	3.47109	0.96795	3.88199	1.24791	5.47149	3.57787	3.05820	5.81144	7.69243	1.14562	6.83613	7.59083	5.19862	0.02301
**Mboat4**	0.03200	0.00820	2.03428	0.21262	0.05878	0.00600	0.03828	0.00858	0.01735	1.71376	0.02745	0.03420	0.01064	0.01037	0.00000	0.01373
**Mboat7**	5.63992	0.11773	0.81092	3.43867	0.88051	3.01116	0.02933	4.81635	3.17205	8.90693	2.83711	0.41406	4.67580	0.09072	0.71884	3.62115
**Mbp**	0.16653	0.01753	0.08328	0.14759	0.02286	4.18964	0.00581	0.00969	0.59577	0.00000	0.08301	0.02333	1.66930	0.14080	0.04153	2.98217
**Mbtd1**	10.87289	3.47837	10.32369	22.91087	0.11029	0.27487	7.81055	7.44573	15.96486	11.33342	4.30287	9.75072	2.79604	21.24008	21.65449	0.60179
**Mbtps1**	7.68449	2.47406	5.01398	7.86562	4.98921	5.52355	3.91232	7.11918	8.75624	13.69923	6.36710	5.30913	2.56846	1.37686	2.64001	3.28763
**Mbtps2**	4.61301	0.15871	1.06593	2.79568	0.02676	0.00646	1.12952	1.70711	3.04257	2.30878	0.32097	8.60518	3.15833	2.71020	1.58098	1.26625
**Mc1r**	0.02317	0.01908	0.11148	0.36768	0.01097	0.00726	0.03736	0.04904	0.19979	0.03995	0.33602	0.04085	0.12634	1.30405	0.10865	0.01452
**Mcam**	0.00962	0.00786	0.00814	0.00991	0.00965	0.00691	0.00743	0.39855	0.01181	0.00678	0.03847	0.02727	0.00593	0.01414	0.00343	0.00740
**Mcat**	7.62873	2.49288	3.60594	1.60526	6.91968	0.02055	0.27609	2.01516	0.82158	0.55312	0.32565	0.02273	1.97141	0.00223	0.00000	0.41169
**Mcc**	0.35642	0.05974	0.60186	4.27951	0.08113	0.12100	0.17747	0.02368	1.41338	0.36446	1.75372	0.24846	0.65091	3.65052	0.59880	1.08413
**Mccc1**	3.79237	3.59400	0.58213	0.00000	3.99963	3.14151	2.92360	5.39566	1.92285	0.51541	1.81099	8.00580	1.59613	0.00000	0.76914	5.30942
**Mccc2**	4.84248	2.14513	6.04210	0.00000	0.21111	6.37989	3.59125	4.20078	0.70593	3.22899	0.63656	0.05540	3.85082	1.53924	1.19468	8.19284
**Mcee**	3.82385	1.53095	2.58712	2.68637	4.15052	1.08148	2.84642	1.30266	0.79475	1.81138	0.97583	0.68789	2.24129	0.05516	0.69173	0.93052
**Mcf2**	2.54505	0.00359	0.00000	2.07486	1.97181	0.00375	0.00505	0.00000	1.09033	1.71166	0.00000	0.08184	0.00986	0.02590	2.33479	0.00391

**Mcfd2**	7.06695	6.77659	8.85053	14.38744	2.10650	6.16090	6.40585	4.97518	6.46810	8.17202	5.50847	0.72131	6.57595	5.49455	8.19256	7.33391
**Mcl1**	12.49806	11.82700	18.22562	17.48066	8.40438	4.41723	9.31900	19.59627	23.12871	31.98765	25.61234	15.96488	16.12527	27.15468	27.12355	9.15543
**Mcm10**	13.13333	15.17631	9.86910	7.60841	11.28896	5.57365	3.91724	17.21433	11.76923	18.82415	12.77317	7.77453	12.37941	11.52413	16.43952	4.53363
**Mcm2**	21.37665	12.96063	16.59888	14.80257	12.88850	8.68814	13.65282	22.99169	15.47008	16.62050	14.93504	3.59354	13.64571	4.51288	14.98071	7.37582
**Mcm3**	17.26389	14.49312	11.74393	10.37237	6.71195	4.55960	12.08189	20.37285	10.02420	16.08175	8.41401	8.51014	8.54958	3.84746	14.97749	9.41618
**Mcm3ap**	3.23580	6.48625	4.43375	6.20813	9.44363	5.56017	4.18995	3.05589	7.59426	6.90602	2.33789	0.42885	0.57390	3.93301	1.64180	0.02481
**Mcm4**	11.12590	12.58709	9.49812	8.61846	6.66325	1.98076	9.31230	14.90789	12.17688	7.74036	7.34814	8.29261	5.74379	13.13537	18.50880	6.16102
**Mcm5**	17.85355	14.44858	16.87934	4.01248	14.82700	9.26262	11.31171	21.58874	6.76901	20.11317	10.23667	7.57176	10.57317	3.56117	10.28303	13.27660
**Mcm6**	17.00298	12.74304	17.22485	15.75122	11.96258	3.48517	10.29851	19.38615	26.05312	28.76009	17.88220	13.55688	10.90186	23.25649	25.94703	9.78082
**Mcm7**	18.92906	14.22099	19.49873	10.39455	10.48569	3.89233	16.08608	25.54494	9.38793	19.91328	21.07259	3.96517	19.07664	4.16160	9.69545	16.36857
**Mcm8**	4.98287	7.48709	7.08489	3.24289	7.90612	0.06339	3.99656	3.20546	1.06915	4.51425	2.73710	8.49695	0.48483	3.14108	4.17269	4.02975
**Mcm9**	0.48836	0.65424	1.34273	0.78364	0.01153	0.76746	0.00671	0.02050	0.65015	0.31604	0.00000	0.06713	0.19820	0.32814	0.12279	0.00598
**Mcmbp**	8.34841	5.50151	5.17537	13.01524	9.55210	10.06252	0.18789	9.71552	19.89329	12.85305	9.46448	7.13403	6.73472	6.97432	8.93383	2.54828
**Mcmdc2**	0.22644	0.01800	0.11903	0.44046	0.00740	0.03052	0.24566	0.06798	0.62404	0.26087	0.05759	0.10656	0.04800	0.70600	0.16974	0.04330
**Mcoln1**	0.00000	2.12652	1.35066	0.82165	0.01468	0.12658	0.00604	4.79115	0.00000	0.00000	1.10371	0.02792	0.01165	0.00106	0.97440	0.02060
**Mcph1**	2.44651	1.65597	3.27706	5.02695	3.01630	0.01091	0.09578	4.83158	3.37670	4.23948	2.79039	0.14860	1.90406	1.26296	11.99717	0.01537
**Mcrs1**	14.22075	12.56669	14.49878	13.91374	8.11209	5.67059	8.08706	28.12703	7.71093	25.25371	13.45569	2.06791	19.46390	0.02215	5.80826	10.23067
**Mctp1**	0.01365	0.00811	0.01202	0.66050	0.00929	0.00656	0.00752	0.01387	0.69281	0.01134	0.00844	0.00704	0.01286	0.01224	0.00666	0.00727
**Mctp2**	0.01270	0.00770	0.00672	2.15586	0.00954	0.00658	0.00711	0.00857	0.00000	0.00588	0.03202	0.01253	0.01275	1.27021	0.00000	0.00706
**Mcts1**	6.54183	4.18785	5.80273	3.42545	3.00557	0.10626	4.37514	4.24089	3.36805	5.69960	6.73023	0.00000	7.62369	8.30854	3.82187	7.20422
**Mcts2**	2.06850	0.44376	0.94174	4.85049	0.01749	2.66381	1.91411	3.84019	2.85395	6.94201	1.42981	0.57620	2.27984	1.31894	2.45927	0.00877
**Mcu**	0.08130	0.03917	0.19707	2.72679	1.45787	0.00875	0.01754	0.01070	0.32968	0.02144	0.06792	0.07285	0.00960	0.00759	0.47966	0.00657
**Mcur1**	4.82312	2.43465	0.46756	3.94229	0.90141	2.79011	2.68199	0.55693	1.90897	3.44611	6.38517	0.14402	2.64625	2.49378	0.78806	0.00000
**Mdc1**	7.13862	2.52840	5.77465	9.08002	1.03820	2.81879	1.82779	1.61470	13.56127	3.37069	2.33556	0.26282	4.36817	4.42483	9.67588	3.57970
**Mdfic**	0.01087	0.00783	0.01062	0.01068	0.00846	0.00661	0.00738	0.01218	0.01232	0.00654	0.01207	0.09908	0.01019	0.01234	0.00972	0.44553
**Mdga1**	0.01354	0.00802	0.01703	0.00698	0.00915	0.00731	0.00749	0.00977	0.01168	0.01137	0.17421	0.00742	0.01022	0.01245	0.01726	0.00737
**Mdh1**	13.73692	9.63461	25.24977	2.74039	8.48123	1.05992	6.86656	4.48023	2.42191	7.43506	0.45239	0.70688	8.49658	2.64258	2.17094	9.14282
**Mdh2**	36.24715	8.64001	21.64987	4.95123	21.69101	17.21373	12.96109	35.48682	8.94186	30.97258	19.03592	7.30956	31.83267	3.78485	9.66643	15.44672
**Mdk**	8.89933	0.00000	0.00000	0.38688	2.48507	0.91147	0.09969	1.14917	0.00000	0.11184	0.49615	0.06510	0.00000	0.00444	0.12417	3.08581
**Mdm1**	1.67631	0.01357	0.00333	0.87045	2.02032	0.00562	1.42595	0.00000	0.11971	0.65721	0.34701	0.04981	0.20590	0.00461	0.53086	0.01254
**Mdm2**	4.42745	1.63937	5.11497	19.62788	6.10992	0.52724	5.05108	6.69422	15.23785	10.04530	3.38052	9.57487	2.25461	7.34240	16.55128	2.52472
**Mdm4**	7.93436	4.00415	6.28488	28.33421	6.33268	2.22189	8.16790	3.67042	10.87807	11.00136	8.66011	5.16193	5.70455	22.14668	29.62864	2.78558
**Mdn1**	9.93421	15.89494	8.57662	11.01530	0.03205	4.27033	2.53957	10.73030	11.95786	23.66048	11.28876	15.93233	7.41740	26.43892	19.08898	3.83734
**Mdp1**	0.80508	4.46118	6.89517	2.29898	4.64847	0.05253	1.49874	1.57451	7.95322	10.45127	7.99499	0.33538	2.10542	1.67981	3.79813	1.60388
**Mdrl**	0.86271	3.54015	0.00000	1.13810	0.00632	0.00486	0.96081	0.02284	1.52865	1.47325	1.04019	1.86600	0.69073	0.12310	0.41549	0.00405
**Me1**	19.17904	17.98605	9.51308	6.46482	17.36872	6.44538	8.22153	1.19678	1.51871	10.19252	8.79786	4.59989	6.09830	3.32757	8.29050	2.68378
**Me2**	0.80062	3.25053	1.70582	6.45052	0.03313	0.00407	0.29782	2.07087	2.92738	3.61884	0.41270	0.43494	0.48011	0.00000	3.36171	0.17940
**Me3**	0.02232	0.01954	0.01062	0.01912	0.01120	0.00682	0.01783	0.01177	0.01167	0.44640	0.01065	0.00762	0.01240	0.00672	0.45654	0.00747
**Mea1**	11.27585	6.10171	8.16284	3.42437	4.83405	8.58997	3.62303	5.70849	1.46902	7.02870	2.56589	0.78953	5.73315	0.18635	2.97950	8.10375
**Meaf6**	10.34322	3.27814	9.55740	6.74780	0.24767	5.95746	2.61566	8.91819	7.39399	7.46910	10.50306	6.74207	6.61210	1.58767	2.78798	2.17834
**Mecom**	0.01340	0.00764	0.01163	0.03455	0.00916	0.00666	0.00739	0.01109	0.00930	0.62613	0.01014	0.00601	0.01197	0.01370	0.26685	0.00743
**Mecp2**	0.09035	0.00820	0.74497	0.29350	0.00679	0.00748	0.13043	0.51096	0.19514	0.37951	0.18416	0.02306	0.00220	0.76560	0.07640	0.01421
**Mecr**	5.46269	0.13348	0.00000	1.36951	1.27349	5.62277	2.27256	5.59630	1.93077	2.93681	1.59562	0.13691	2.41774	0.00000	0.61845	4.93980
**Med1**	6.07222	4.87805	6.35800	8.45596	3.23553	0.71355	2.27865	4.82110	10.44932	5.68814	5.79378	1.29649	1.94874	8.52748	9.30091	1.08288
**Med10**	15.71582	8.77622	12.50257	11.95251	7.99936	8.68189	6.52009	12.17617	17.73875	17.66411	8.66020	9.03712	14.39111	2.13919	6.95069	2.27261
**Med11**	9.76067	0.61660	4.30775	2.78450	6.01292	0.00796	2.96951	6.69788	7.79024	6.94773	3.29370	0.78535	2.19976	0.00000	0.80811	1.44292
**Med12**	3.17803	0.01486	0.00000	3.64993	1.56383	1.62124	0.11137	0.65947	5.12318	4.75903	1.48805	0.00000	2.76488	0.00000	0.00000	3.48171
**Med12l**	0.78391	0.00581	0.00000	0.10080	1.94716	4.05016	0.00605	1.19068	0.06383	0.18820	0.67612	0.00587	1.19716	1.67428	0.21679	0.00422
**Med13**	0.65515	3.32240	0.96807	13.98363	2.28604	0.00255	2.39168	1.06007	16.95935	0.64584	5.27112	3.40162	0.00000	11.81753	8.51742	1.59348
**Med13l**	2.59396	2.90259	3.01210	4.54482	0.01486	0.00200	0.81424	3.02018	2.72396	2.16598	1.68581	0.14438	0.58247	1.45901	3.07403	0.09162
**Med14**	0.51678	1.73244	2.48208	10.64953	0.14829	0.06993	0.03302	6.34184	12.41884	12.78888	2.97489	13.56431	0.47883	27.65881	21.13296	0.05153
**Med15**	8.72207	0.87978	2.61760	8.80225	4.43046	1.45636	1.27405	8.56658	2.97852	2.36494	1.94502	0.18442	2.82801	1.11373	2.56889	3.26556
**Med16**	5.64550	0.01081	0.73206	2.92441	2.98874	0.01141	0.06323	0.06480	4.08733	7.41602	3.14172	0.00000	3.00012	0.15187	0.35649	4.30987
**Med17**	8.51399	10.53099	8.87816	2.05567	6.54125	0.52412	3.26159	10.62403	6.14146	13.92943	7.86917	5.77809	7.31798	8.26839	12.54518	3.59522
**Med18**	0.29349	0.01887	0.03553	0.01522	0.01364	0.00542	0.02665	0.01487	0.94252	0.71397	0.05270	0.03301	1.91076	0.23714	0.51117	0.02037
**Med19**	8.69006	3.65130	3.62305	1.73108	3.91402	2.42150	1.04924	6.87210	4.71815	9.97118	4.65164	3.17748	3.29386	1.80147	4.15892	0.82657
**Med20**	6.32472	5.18360	6.53805	6.53536	3.61256	4.15712	6.75755	7.78014	6.77664	12.42553	7.85722	3.29093	8.03228	0.18865	5.61610	4.46680
**Med21**	10.22144	4.68896	7.22560	9.29027	10.26170	6.91230	2.91885	4.76393	14.26384	14.72652	11.26546	2.74845	7.32949	14.72374	9.45825	0.67475
**Med22**	5.14633	0.80936	6.39760	5.59695	0.09298	3.48646	3.83936	6.56571	3.16815	15.30215	2.77914	0.96850	6.12317	0.34711	5.09634	0.22974
**Med23**	5.32364	5.28648	2.27394	7.92285	0.00000	1.98003	5.67640	2.33586	8.18861	11.41709	3.38852	7.16105	1.90868	9.76707	12.41399	2.15510
**Med24**	0.99216	2.36488	4.52526	6.07069	5.80514	0.16329	4.41871	9.31894	6.24897	9.56855	7.34979	8.18276	5.30950	0.20528	0.31067	6.46931
**Med25**	5.08788	0.02122	0.00000	0.11734	3.88795	0.89714	0.03864	0.61699	0.21071	11.03583	0.31444	0.00000	13.44902	0.00906	0.00000	2.01913

**Med26**	3.00921	2.48263	3.69344	11.94325	1.83759	3.14553	1.20773	1.39278	11.56917	9.98946	1.84851	0.52957	1.63873	3.55673	3.60779	0.07677
**Med27**	10.76066	8.41510	11.29022	16.44874	7.44330	0.00000	6.92072	12.73220	9.24715	26.10518	12.86306	3.22277	12.59001	2.43446	11.43647	5.65497
**Med28**	4.67566	1.70074	5.95222	12.94330	3.88719	1.31289	0.22369	4.38970	9.03582	20.67488	19.69636	1.10462	13.18344	2.02237	3.87138	5.90326
**Med29**	5.57062	0.05901	1.56650	20.50589	0.84728	0.38790	3.54275	7.03144	30.71079	18.10901	13.67751	1.55371	7.96484	1.21643	2.49690	1.39776
**Med30**	1.13311	0.29924	0.68788	2.17596	3.01444	0.25631	0.04708	0.00000	0.00000	0.16098	0.73311	0.48081	0.05410	0.00542	0.05144	0.59219
**Med31**	2.25099	1.10855	1.69234	7.47936	4.34092	0.74431	0.06202	1.35785	16.58496	6.85097	5.32531	0.54119	1.26659	3.87119	7.45865	0.97043
**Med4**	9.92721	11.95378	13.31387	8.87132	10.10018	1.81156	5.77337	10.28064	15.34003	16.67637	6.09859	10.90731	8.65301	7.70810	15.77506	1.24267
**Med6**	2.87946	5.34890	2.36911	5.80596	2.71364	1.81827	0.07831	7.60703	10.73423	5.61519	7.26568	9.65898	3.37538	13.85748	13.72835	1.19028
**Med7**	4.57867	6.22674	7.14034	9.88334	0.37810	1.98600	2.30845	1.72521	16.55734	9.36202	5.42087	3.02143	2.85077	6.30171	3.26579	0.55292
**Med8**	13.95023	13.07063	11.93183	4.03699	6.03421	0.63975	8.15848	8.79542	4.20072	10.89684	4.04582	7.07115	9.90266	5.46665	4.89657	2.85297
**Med9**	5.73575	1.76713	0.61554	5.89601	1.63573	0.00698	0.08049	0.63038	9.74261	6.00578	3.63110	0.83015	0.40733	0.17606	0.45617	0.75893
**Medag**	0.02148	0.01856	0.07502	0.11829	0.00867	0.00640	0.00763	0.01740	0.08812	0.02052	0.11227	0.00716	0.01013	0.06485	0.02133	0.00730
**Mef2a**	1.77026	0.00512	0.85575	4.88516	0.00303	0.00673	0.00578	0.03210	1.90898	0.33985	0.00000	2.77212	0.20033	0.13296	0.00003	0.00388
**Mef2d**	0.86965	0.54230	1.73242	0.68692	0.00247	4.45418	2.15275	4.84910	1.55818	5.83575	7.60679	0.04687	5.94574	4.65454	0.27985	1.43620
**Meg3**	3.26007	0.00444	0.01562	0.57664	0.01587	0.01334	0.01541	0.00637	0.00512	0.01225	0.10739	0.01528	0.02510	0.01272	0.05465	0.02570
**Megf11**	0.01358	0.00733	1.46413	0.53771	0.01370	0.21011	0.04672	0.93576	0.69724	0.32868	1.25001	0.00000	1.18976	0.00757	0.82786	0.92216
**Megf6**	0.00728	0.00770	0.01004	0.00422	0.00884	0.00833	0.00701	0.59666	0.11029	2.64896	1.37326	0.00000	0.00000	0.08681	0.00000	0.00692
**Megf8**	0.60899	0.00708	0.91538	0.49503	0.06385	0.00642	0.00515	0.28062	1.02807	1.26794	0.38204	0.00689	2.56113	0.13692	0.01129	0.01228
**Megf9**	0.01359	0.00761	0.01189	1.99156	0.00942	0.00625	0.00728	0.01133	0.55517	0.15627	0.00750	0.00491	0.01270	0.00246	0.42860	0.00712
**Mei1**	0.04541	0.00712	1.04805	3.50393	0.01388	0.00889	1.90774	0.51334	1.47486	3.56522	3.13381	0.13194	0.00000	0.06987	2.56405	0.00579
**Mei4**	0.03117	0.00794	0.06197	0.10769	0.00920	0.00717	0.00750	0.01272	0.12391	0.06439	0.10955	0.03608	0.05412	0.16028	0.02790	0.03623
**Meikin**	0.00461	0.00775	0.00000	1.03738	0.01033	0.00717	0.00658	0.37336	0.76129	0.61391	0.63427	0.00401	0.84502	0.86625	1.26497	0.00699
**Meioc**	0.01103	0.00755	0.00718	0.48259	0.01948	0.00660	0.00723	0.00923	1.38946	0.00000	0.00000	0.00000	0.01694	1.60153	0.05218	0.00723
**Meis1**	0.42055	0.00415	1.71974	0.27966	4.26223	0.00877	1.74712	0.02203	0.00181	0.54510	0.01000	0.01032	0.00409	0.01271	0.00000	2.74279
**Meis2**	0.00901	0.00778	0.00414	4.73479	0.00946	0.00550	0.00694	0.00766	1.27675	1.11504	0.00000	0.02302	0.01124	2.90689	4.01706	0.00633
**Meis3**	0.01362	0.00823	0.01208	0.43230	0.00917	0.00684	0.00757	0.01371	0.07671	0.18733	0.01229	0.00888	0.01289	0.01060	0.00625	0.00734
**Melk**	6.41256	9.94112	5.23177	4.64268	8.13610	5.42633	6.71166	10.13017	6.66973	9.93313	9.51681	14.34468	7.74689	13.11382	10.51820	5.04810
**Memo1**	5.54483	4.04784	1.28182	15.92047	5.56708	5.90943	2.01119	10.12863	19.38238	18.27028	14.17161	7.15274	4.08683	7.64335	10.29487	1.28466
**Men1**	8.13619	0.69321	3.90793	3.13287	1.98945	4.58371	5.30683	6.61214	0.00000	6.07389	1.77977	0.22211	4.48702	0.00000	2.00873	0.09992
**Mep1b**	6.36242	0.02097	0.00000	2.19745	0.00000	0.00456	0.00000	4.12432	0.78063	7.70138	3.30073	1.13099	0.58873	8.90220	5.12593	0.00000
**Mepce**	5.93305	3.52251	8.74911	13.42442	5.97403	1.52109	6.53413	6.42345	15.58428	10.68805	2.70634	3.32506	3.45268	0.11901	9.34306	0.02703
**Mertk**	0.07172	0.00626	0.44288	2.80832	0.00702	0.00700	0.00615	0.70465	3.91808	1.18653	0.31544	5.96793	0.67598	3.65606	3.32093	0.00551
**Mesdc1**	2.68025	0.06451	1.95446	1.13397	0.05129	0.00299	0.78251	1.24738	6.62491	1.48831	0.48228	0.21676	0.45776	0.16133	0.83283	3.55444
**Mesdc2**	15.95651	10.96173	23.74380	5.71693	14.08582	9.69018	14.01587	17.47918	5.91675	9.43631	8.79849	13.32187	16.17707	5.88946	10.02231	16.16544
**Mesp2**	0.04836	0.00795	0.57991	10.94651	0.01373	0.00415	0.00678	3.12118	6.89514	1.67887	0.64692	0.02083	0.00000	0.00000	0.19387	0.00530
**Mest**	0.52206	0.04643	0.00000	0.24268	0.01740	1.14680	0.00741	0.38681	0.00568	0.34191	0.00436	0.04621	0.70759	0.00786	1.15752	0.17003
**Metap1**	9.28740	5.59421	9.18259	3.95712	9.10744	6.20886	3.34860	14.43176	8.02650	10.40839	8.25827	6.71352	3.81327	9.79939	11.66860	3.16045
**Metap1d**	4.42662	5.63560	1.04634	0.17971	4.18104	1.59439	0.95268	1.60997	0.00138	3.30547	0.77805	0.18003	1.66456	1.39329	2.28351	0.03499
**Metap2**	21.32978	18.42017	26.02806	21.67600	19.15269	9.71341	16.00296	32.81056	24.45817	40.97060	34.81501	18.39117	24.97227	34.44104	31.64027	8.40780
**Metrn**	0.01193	0.00801	0.04682	0.09529	0.00907	0.00781	0.00732	0.01220	0.01042	0.01008	0.01313	0.00796	0.21367	0.01227	0.00806	0.00718
**Mettl1**	1.37408	0.02432	1.75904	0.11158	0.92691	0.65050	1.64415	1.85910	0.20868	3.23376	0.29200	3.21579	1.59100	0.41524	0.42362	0.46775
**Mettl10**	1.11246	0.78402	0.25781	0.37161	0.00772	0.00470	0.09937	0.33814	0.00320	0.44566	0.69215	0.06690	1.55320	0.97345	0.09122	2.97605
**Mettl13**	5.20201	3.43198	6.65063	3.31176	4.23561	0.95962	2.66905	8.49358	3.87534	4.35365	1.25746	0.45255	2.51187	1.74718	6.99048	2.07302
**Mettl14**	0.44631	0.01851	2.64799	0.18487	0.02214	0.00644	0.00466	3.41550	0.64351	3.67504	4.24268	0.32793	0.96255	2.14250	4.55002	0.35958
**Mettl15**	0.83191	0.00867	0.00083	0.01026	0.02172	0.00783	0.08793	0.25438	0.86821	0.87480	0.11975	0.09826	0.24594	0.02940	0.84519	0.02315
**Mettl16**	7.43996	9.55167	8.52094	3.41326	1.10690	0.00271	9.43742	6.17523	5.78103	9.42414	4.68451	3.86309	6.68944	0.64896	5.98175	3.48784
**Mettl17**	2.41509	0.02200	5.67833	2.71683	0.65691	4.31079	5.17480	6.33617	0.65938	4.22382	3.31591	0.13479	4.44112	0.04312	0.41073	1.95657
**Mettl18**	0.62270	0.01693	2.15908	0.25374	0.01149	0.01531	3.12017	0.06964	0.68074	1.22807	1.27076	0.07513	2.78445	0.00380	2.50693	0.02560
**Mettl2**	8.63960	2.13930	3.10030	3.32394	4.45307	3.15487	3.40939	6.86679	10.24208	9.21119	5.85103	5.31875	4.67096	3.06502	7.46516	0.74530
**Mettl20**	0.12167	0.00810	0.65118	0.61276	0.00590	0.00723	0.00786	0.46718	0.08108	0.21737	0.02021	0.01706	0.00000	0.09307	1.45387	1.41129
**Mettl21a**	2.24854	2.04418	0.84784	0.38395	0.01300	0.00694	0.00393	1.15343	0.18983	1.21925	0.31134	0.00000	2.30033	0.00377	1.40358	0.28613
**Mettl21c**	0.01359	0.00801	0.01213	0.32796	0.00912	0.00660	0.00732	0.01106	0.16691	0.39299	0.01129	0.00644	0.01237	0.00803	0.04169	0.00775
**Mettl22**	4.94944	0.17248	2.17799	0.56322	0.00000	1.85819	3.08839	2.35771	0.72012	0.39380	0.39929	0.16738	2.43511	0.02240	0.08614	0.86760
**Mettl23**	1.51140	0.10423	0.65162	0.47260	2.84905	0.00456	1.81990	0.45281	2.01335	2.28190	1.00475	0.05692	1.29211	0.24518	0.57269	1.89764
**Mettl25**	0.42464	0.11256	1.18427	0.10994	0.43882	0.00827	0.00603	0.00000	0.43350	0.59767	1.49601	0.02541	0.69964	0.01996	0.53137	0.51151
**Mettl26**	6.02487	0.04927	1.93133	0.63221	1.82347	0.28539	1.08710	2.87692	0.96746	0.76795	4.75666	0.01174	5.75447	0.00000	0.00000	3.62330
**Mettl3**	3.69841	3.64054	3.59720	2.07753	0.95953	0.66511	3.78676	2.02432	1.96164	2.84194	0.91152	0.26818	1.33372	2.86788	4.69838	2.73229
**Mettl4**	1.32965	4.85892	0.72427	5.58037	0.00406	0.00544	0.00364	1.75454	1.83608	1.78459	1.17491	0.55087	0.39720	1.64825	4.64752	0.00204
**Mettl5**	5.91573	3.91210	3.23721	0.51528	4.28627	0.86381	0.56978	0.85120	0.57764	3.37845	1.66352	0.19995	2.98611	3.32013	0.49507	4.38089
**Mettl6**	3.07867	2.72685	4.89984	4.23630	0.09258	0.01122	2.33108	2.47805	2.17109	2.61852	1.66528	0.57050	0.33324	0.00000	3.44164	3.60781
**Mettl7a1**	0.24776	0.00764	0.00370	0.78662	0.00693	0.04933	0.07161	0.01268	0.14425	0.15670	0.09199	0.00670	0.06361	0.01100	0.01137	1.51348
**Mettl7a2**	0.05081	0.00778	0.00267	1.26005	0.00815	0.11988	0.00764	0.00752	0.79032	0.02486	0.25083	0.00533	0.01241	0.01378	0.04042	0.23623

**Mettl7a3**	0.01437	0.00799	0.01125	0.01057	0.00926	0.01334	0.08025	0.01282	0.01237	0.00655	0.01377	0.00769	0.51037	0.01296	0.01023	0.03623
**Mettl8**	4.41661	0.00348	0.83369	1.63612	0.00000	0.00172	0.24648	0.57920	0.01156	1.77261	0.00708	0.03363	0.72974	0.00435	1.01321	0.00980
**Mettl9**	6.60499	3.09480	4.31426	23.46282	5.61068	0.30141	4.08822	1.09127	11.60931	7.40873	4.17341	0.21675	0.80627	3.74203	4.85151	0.55080
**Mex3a**	0.09821	0.00786	0.09078	0.15597	0.01028	1.41108	0.00713	0.00000	1.16274	0.00000	0.43649	0.01164	0.01656	0.00507	0.44102	0.00647
**Mex3b**	0.01009	0.01757	0.52137	0.03842	2.69962	0.00573	0.00708	0.01897	0.82177	0.00000	1.54109	0.13113	0.00000	0.00725	0.65808	0.01417
**Mex3c**	0.49335	3.39177	0.99675	10.86102	5.43095	0.09234	0.68768	4.59615	11.17839	1.75464	4.78318	5.13804	0.76219	25.66683	7.85220	0.02196
**Mex3d**	0.29575	0.53942	0.00000	0.87897	0.00989	0.00750	0.01607	0.18041	0.04645	1.06387	1.34733	0.02874	0.33370	0.00712	0.69446	0.00635
**Mfap1a**	2.40062	6.78977	5.10165	4.88143	1.49789	0.33320	4.75643	2.77542	9.27576	8.39881	2.52046	0.98737	3.54492	8.81005	10.13989	1.86802
**Mfap1b**	0.72370	1.75254	1.24779	0.00000	0.48664	0.03974	1.53171	0.85088	0.17245	1.92125	0.00000	0.10713	0.86389	2.01194	3.32332	0.48431
**Mfap2**	0.01301	0.00821	0.00988	2.70449	0.00893	0.00641	0.00735	0.00893	3.82286	0.50452	0.05943	0.03724	0.01864	0.00462	0.02391	0.00667
**Mfap3**	4.12525	1.54218	3.49030	1.44689	1.20529	0.00000	4.09088	0.00000	0.59299	1.03776	0.92619	3.77013	0.10813	3.79000	2.47221	1.51660
**Mfap3l**	0.01387	0.00782	0.00824	0.76120	0.00932	0.00662	0.00719	0.01006	0.51465	0.00000	0.01972	0.33512	0.01286	0.00470	0.31603	0.00721
**Mfap4**	0.01461	2.73481	0.08238	1.47442	0.00752	0.00558	0.00642	2.55130	0.63947	0.34937	0.00000	0.01263	0.02411	0.00172	0.69352	0.00572
**Mfap5**	0.37872	0.00646	0.30668	4.82631	0.02764	0.00570	0.33157	1.86050	1.65980	2.10044	3.58802	0.68711	1.39088	1.01078	2.15195	0.00477
**Mff**	6.31794	2.21117	4.17793	10.66244	9.33087	8.27812	2.50032	2.73178	2.99410	3.90580	0.07711	0.33757	2.59959	1.81354	2.43604	0.11154
**Mfge8**	10.22776	2.15405	7.78022	0.52362	7.12400	3.20757	7.52424	11.85050	0.02526	10.67225	7.08547	0.25927	15.10138	0.13800	0.05369	12.74567
**Mfhas1**	0.01120	0.06157	0.20662	0.17566	0.00855	0.00567	0.00739	0.01054	0.19621	0.76634	0.01021	0.01553	0.01053	0.01045	0.00236	0.00751
**Mfi2**	0.00789	0.00726	0.88578	0.01157	0.00830	0.00914	0.00696	2.06962	0.01042	0.00000	0.00000	0.00337	0.00747	0.01912	0.00000	0.00700
**Mfn1**	17.24894	13.77738	17.56211	8.84917	11.44353	10.18929	9.59207	22.41371	9.36041	16.65738	11.69678	10.32604	8.45984	6.79465	10.69384	3.85470
**Mfn2**	2.10735	4.65411	4.26538	9.26705	3.58292	2.27601	5.59759	1.85867	1.40805	10.70569	6.75087	0.42735	4.44159	0.21645	4.87171	1.77761
**Mfng**	0.28006	0.00680	0.00267	0.01042	0.84948	0.00825	0.00689	0.47290	0.01133	0.00899	0.01002	0.00579	0.00567	0.01409	0.00389	0.00696
**Mfsd1**	19.06529	7.18646	21.29429	7.84700	16.49742	9.68610	14.18217	13.81430	1.47048	13.66687	16.23000	12.55272	17.72167	14.59564	13.62269	13.19706
**Mfsd10**	5.90813	0.00000	1.38289	0.63278	1.66192	0.35403	2.09947	10.22759	0.73556	3.30801	0.73365	0.99500	8.00750	0.00000	0.00000	4.82764
**Mfsd11**	2.79682	0.12446	6.99765	8.36198	1.22379	5.58343	4.30303	0.39925	10.39916	0.21311	10.59495	0.98450	3.09878	3.81471	3.31657	2.28901
**Mfsd12**	0.01275	0.00796	0.04206	0.06592	0.00917	0.00812	0.00738	0.01251	0.57941	0.04188	0.00857	0.00663	0.03300	0.01151	0.00766	0.00737
**Mfsd13a**	4.48030	0.00357	0.05923	2.36730	0.15818	0.45562	0.05916	0.34451	0.18979	1.03964	0.00767	0.01000	2.89858	0.00864	0.00419	1.36476
**Mfsd14a**	3.41918	3.09834	4.20202	15.31878	2.44820	1.55904	7.84486	13.00793	14.83611	6.07904	7.45653	11.67428	4.22308	25.03588	23.43365	0.01373
**Mfsd14b**	1.10915	0.00000	3.33984	0.86161	1.10284	0.89946	3.33585	6.20030	2.60387	2.70577	4.96439	12.20549	1.53588	28.66554	13.56076	1.19485
**Mfsd2a**	0.01272	0.00796	0.00608	6.52593	0.00891	0.00546	0.00711	0.00000	8.92207	0.01382	0.21198	0.03285	0.01042	0.00000	0.99970	0.00559
**Mfsd2b**	0.43029	0.00741	0.02914	1.34226	0.01520	0.00616	0.00574	0.02177	2.14604	0.00000	2.94570	0.00000	1.70729	0.32381	0.05905	0.01199
**Mfsd3**	0.14950	0.00691	1.02902	0.00933	0.00607	0.00930	0.54729	0.96575	0.02652	0.00278	0.00718	0.00409	0.62723	0.01525	0.00211	0.00668
**Mfsd4a**	0.08840	0.02939	0.14942	0.09862	0.05211	0.00605	0.01764	0.01338	0.27565	0.02456	0.32815	0.01499	0.05021	0.16304	0.07052	0.03646
**Mfsd4b1**	1.75932	0.00632	0.07642	0.00418	0.00506	0.00748	0.00614	0.00770	0.42849	0.00249	0.00868	0.00459	0.07968	0.38546	0.02692	0.00578
**Mfsd4b2**	0.07033	0.00801	0.00951	0.04100	0.00798	0.00680	0.00747	0.01448	0.00954	0.01286	0.01247	0.00829	0.01265	0.00897	0.05857	0.00726
**Mfsd4b3**	0.07980	0.00810	0.01005	0.01035	0.00822	0.00710	0.00761	0.01665	0.01224	0.01407	0.01413	0.00824	0.02658	0.01098	0.02410	0.00744
**Mfsd4b5**	0.05434	0.00795	0.03689	0.09795	0.07786	0.00775	0.01807	0.02801	0.15189	0.06692	0.01130	0.00738	0.04382	0.04011	0.16963	0.00743
**Mfsd5**	5.80431	0.07278	2.53732	10.11388	0.03862	1.61162	2.18845	19.50164	19.21866	22.49513	12.50900	2.71693	6.33910	0.08814	7.81143	0.14516
**Mfsd6**	0.01180	0.00747	0.01291	1.38919	0.00912	0.00698	0.00710	0.00793	1.79734	0.50627	0.02578	0.00000	0.01209	0.65356	0.79837	0.00745
**Mfsd7a**	0.00751	0.00776	1.41171	0.00427	0.00787	0.00562	0.00651	0.47585	0.21274	0.03653	2.24689	0.00000	0.00000	0.17663	0.14022	0.00638
**Mfsd7b**	1.71586	0.50528	0.17141	4.14213	0.00538	0.05944	0.00580	0.32637	5.49320	0.83954	0.96167	0.01144	0.43324	0.00378	1.79973	0.86054
**Mfsd7c**	5.65240	6.50114	12.00394	2.43485	0.14950	3.63370	9.82543	24.86569	4.25719	16.41592	18.51620	2.77960	19.39539	15.17261	16.93711	0.36410
**Mfsd8**	0.85101	0.00409	0.00000	0.74474	2.17633	2.57786	0.06578	0.81099	0.00000	1.94426	1.30029	3.33239	0.20387	0.09320	1.56871	0.00503
**Mfsd9**	0.01341	0.00751	0.01057	2.14413	0.00925	0.00612	0.00714	0.00995	0.00000	0.00323	0.00982	0.01512	0.07615	0.00875	1.17087	0.00686
**Mga**	2.57480	4.16749	2.26344	13.77754	2.06624	0.03449	0.38204	3.70574	13.74870	3.35490	5.41289	0.84979	1.85035	21.57330	15.57193	0.11597
**Mgarp**	22.51104	8.99422	13.96071	0.15036	12.02446	0.00000	9.24652	4.35020	0.69178	4.12688	4.86444	0.22646	1.61713	0.32347	0.00000	7.05695
**Mgat1**	0.10736	0.00737	0.89697	0.88693	0.00765	0.33182	0.00699	0.27598	0.00000	0.72154	0.56885	0.06000	0.03512	0.00305	0.44961	0.00701
**Mgat2**	1.46923	3.29134	4.60526	14.80849	3.77498	4.80595	2.91085	7.87464	22.28284	9.62106	4.67393	5.14876	3.81646	8.60417	13.31957	3.05393
**Mgat3**	0.48454	0.00681	0.00000	0.85416	0.00657	1.68095	0.00522	1.67508	1.36934	0.00000	2.40414	0.03370	0.79057	2.64862	3.17299	0.42954
**Mgat4a**	0.00000	0.00686	1.51844	3.01642	0.01581	2.10116	0.00585	3.64370	1.18800	2.69915	2.03956	0.05895	0.57945	1.50855	2.80997	0.72650
**Mgat4b**	0.07046	0.00770	0.11131	0.45724	0.02064	0.04055	0.03833	0.01208	1.03711	0.46177	0.00272	0.02212	0.08400	0.00384	0.24709	0.21350
**Mgat4e**	0.01381	0.00807	0.01102	0.26007	0.00932	0.00660	0.00741	0.00879	0.00505	0.07843	0.17926	0.00685	0.01032	0.00964	0.00000	0.00736
**Mgat5**	0.01575	0.00795	0.04547	0.09456	0.00923	0.01189	0.00744	0.01341	0.00820	0.01532	0.01269	0.00796	0.01192	0.01188	0.00827	0.00735
**Mgea5**	8.92168	1.96875	0.50219	10.91502	3.17734	3.94271	2.88143	6.60052	10.02074	21.34790	8.40982	13.60751	0.67569	16.06494	18.01365	2.21233
**Mgl2**	0.24527	0.19904	0.54092	1.19572	0.01637	0.05295	0.17682	4.84813	1.11892	1.36496	0.21048	0.21237	4.28953	0.62388	0.91471	0.32707
**Mgll**	1.05430	0.94443	2.43060	0.00835	4.22582	0.01663	2.23319	0.00140	0.00999	0.00000	0.01081	0.01934	0.09932	0.01358	0.00000	0.42505
**Mgme1**	1.50952	3.35618	7.69187	2.80864	0.00515	1.68781	0.10630	8.71320	1.23289	7.27539	2.71653	1.06920	2.26839	2.63283	12.13884	1.30504
**Mgmt**	0.07080	0.00423	1.00081	0.00691	5.57323	0.00541	0.00686	0.36851	0.02230	0.00480	0.00922	0.01952	0.00000	0.01585	0.00000	0.02108
**Mgp**	0.01357	0.00801	0.01219	0.00893	0.00916	0.00665	0.00744	0.01177	0.01213	0.40494	0.01192	0.00720	0.01184	0.01156	0.00991	0.00734
**Mgrn1**	3.64157	0.00000	1.64253	6.10400	0.00000	1.46178	5.43475	7.52073	2.46014	10.70706	8.94526	0.52028	0.78269	0.03213	3.56643	1.55777
**Mgst1**	0.01295	0.00790	0.01206	0.00657	0.00885	0.00672	0.00735	0.01063	0.01148	1.46672	0.01093	0.00469	0.01137	0.01202	0.00869	0.00750
**Mgst2**	0.59079	0.79992	0.00000	0.00950	0.02271	1.43165	0.00565	0.00708	0.01269	0.00000	0.01087	0.02618	1.29299	0.01368	0.00000	0.44496
**Mgst3**	0.11795	0.33938	0.39171	0.21324	0.02242	4.13668	0.00632	0.01724	0.40004	0.09354	0.02927	0.01312	0.55608	0.00483	0.59194	5.56515

**Mia**	0.01349	0.00779	0.00910	0.00635	0.00915	0.00665	0.00723	0.00608	0.01186	0.30068	0.16362	0.04297	0.02853	0.17052	0.00000	0.00720
**Mia2**	0.01361	0.00814	0.01108	0.00367	0.00938	0.00663	0.00744	0.01195	0.32777	0.00571	0.00815	0.00708	0.01333	0.07921	0.48590	0.00741
**Mia3**	5.72263	2.64620	7.42414	2.15738	1.36355	3.95180	7.86228	7.89372	3.35532	3.25949	4.71134	1.58614	2.01408	0.83801	2.60210	1.78337
**Mib1**	0.09704	0.00662	0.08805	0.08701	0.00829	0.94221	0.16660	1.91340	2.83317	1.49549	0.68792	1.84256	0.00000	0.56741	7.27071	0.09597
**Mib2**	2.14843	0.01493	0.00000	0.01155	1.12620	0.01122	0.03460	0.26756	0.09707	0.77195	0.00937	0.00000	3.28923	0.00821	0.03446	7.65762
**Mical1**	3.14331	2.96148	0.66499	0.09859	2.27843	0.04370	1.29824	1.96752	0.00660	0.00000	0.66089	0.00139	3.83573	0.00438	0.00000	5.42769
**Mical2**	0.28104	0.00718	0.00519	0.15420	0.00731	0.00715	0.00715	0.00946	0.00645	0.30186	0.01106	0.05484	0.01229	0.00886	0.18729	0.00736
**Mical3**	2.61283	3.09814	5.34184	2.29400	0.01950	0.00354	0.00138	8.14875	1.03907	5.11914	4.24185	0.11154	0.96149	2.80427	8.00374	0.00696
**Micall1**	0.82605	0.01629	0.00000	8.72076	0.00771	0.00615	0.00552	1.59824	9.92346	3.58638	2.46798	0.15386	0.00000	0.00000	0.95544	0.00428
**Micall2**	0.04167	0.00776	0.71754	0.73328	0.00713	0.00553	0.01724	0.01014	0.00000	0.00958	0.08098	0.04800	0.02192	0.00728	0.04614	0.01411
**Micu1**	2.84352	0.08464	5.59237	4.32161	0.17821	0.01173	0.07153	0.57354	4.83753	0.60804	1.70034	0.18402	1.73108	0.99391	0.00001	2.31368
**Micu2**	3.94595	0.00218	0.00000	2.53280	1.86387	2.70465	0.00380	0.91793	1.98705	0.83635	0.68000	0.00000	0.00000	0.09217	1.44989	0.00947
**Mid1**	0.33507	0.00867	0.00465	0.38205	0.01954	1.27942	0.00733	0.01190	0.18195	0.17386	0.01008	0.00657	0.23508	0.00528	0.25636	0.11339
**Mid1ip1**	0.00000	0.00819	0.43161	2.14155	0.01292	0.23406	0.00659	0.45232	7.66261	3.67150	0.19561	0.00000	2.14363	0.00509	0.00000	6.35256
**Midn**	0.17708	0.68835	0.82575	4.45658	0.31480	0.31513	0.29747	0.00000	1.88968	0.13864	1.79137	0.00000	0.36912	0.68629	0.01216	0.20925
**Mief1**	3.41477	1.78679	3.02634	2.78960	2.21853	4.30464	0.14110	2.72396	4.83271	6.97978	2.64843	5.09665	4.04705	5.95241	9.81367	2.33025
**Mief2**	0.69267	0.02893	1.06523	0.51914	0.04447	0.00530	0.17870	2.14150	1.50080	0.67481	1.20221	0.00796	0.50308	0.45020	1.62021	0.99861
**Mien1**	15.73031	6.52972	9.08280	1.33148	6.49198	8.74599	7.53562	8.87998	0.57771	8.45728	7.18746	0.41012	19.10762	0.02321	0.25192	7.59059
**Mier1**	2.11585	2.12820	0.31199	1.04537	0.00394	3.06543	0.07636	0.15446	1.12600	1.23304	0.93093	6.84374	0.20155	6.64681	1.11161	0.32052
**Mier2**	3.75910	0.00395	0.11815	0.60095	0.02855	0.09534	0.19601	0.42542	0.56142	1.33831	0.10714	0.04867	3.20696	0.00846	0.00000	3.65530
**Mier3**	4.50609	6.01900	11.34619	13.73155	1.25689	1.18849	12.34509	17.81131	19.98520	33.56780	24.99625	30.44471	7.86096	44.35948	36.42490	1.74257
**Mif**	12.52828	0.17802	4.87356	4.53913	11.71003	2.37821	2.59116	5.27633	3.98804	11.49969	9.60715	0.67111	9.60836	0.00000	0.00000	4.44625
**Mif4gd**	7.41332	0.45301	0.67772	5.32616	0.04176	1.17745	3.66130	7.48291	5.09723	4.32530	6.23142	0.35625	2.19126	0.00000	0.37477	4.31776
**Miip**	6.90174	0.52985	7.71365	0.30329	11.20458	5.65346	3.37810	17.81686	3.05624	2.04921	4.13764	0.10850	10.99948	0.00693	0.25450	5.16329
**Milr1**	1.07604	0.46247	0.29915	0.30863	0.00679	0.03439	0.00622	0.00000	0.43171	0.67954	1.00277	0.02543	0.01553	3.65715	0.00000	0.00544
**Mina**	0.45854	0.00556	0.47417	0.20161	1.03933	1.66017	1.05732	3.99113	0.79790	3.84577	0.00000	0.17249	2.61261	3.37172	5.84618	0.01933
**Mink1**	0.17326	0.01800	0.21015	0.73941	0.01716	0.08205	0.06796	0.80480	0.71148	2.70905	3.88663	0.23386	0.28629	0.00000	0.00000	0.00633
**Minos1**	9.52063	1.27392	12.69965	13.92114	7.26011	1.73553	3.13247	0.69958	14.27723	7.03186	16.30322	1.14892	7.44553	1.81780	3.54805	3.62866
**Minpp1**	3.72569	0.14932	3.75080	0.65354	4.38688	0.08253	0.00189	0.96615	0.14916	3.91798	0.03467	0.17797	4.16195	0.00591	9.58835	2.03206
**Mios**	4.70505	4.53151	0.00000	8.49256	2.08052	0.46567	0.01009	2.87612	2.70247	5.39750	2.99924	2.75787	0.35633	4.18955	13.40768	0.14651
**Miox**	0.00090	0.00625	0.00000	6.15969	3.22652	0.00648	0.00775	0.75143	6.19699	0.45724	0.00000	0.09887	0.09006	0.25176	2.39038	0.00623
**Mip**	0.01843	0.00850	0.01015	0.01012	0.01057	0.00671	0.00740	0.01111	0.01231	0.03730	0.01137	0.00724	0.01244	0.24867	0.02527	0.00730
**Mipep**	1.55467	0.00268	1.71261	1.04719	5.79957	3.38843	0.01403	1.74184	1.28534	0.44972	1.90576	0.18102	1.97833	1.06093	3.20692	1.16034
**Mipol1**	0.05370	0.00814	0.01050	0.02102	0.00853	0.00721	0.00768	0.01522	0.35715	0.00800	0.11430	0.00698	0.03802	0.01222	0.00523	0.00748
**Mir1291**	0.01118	0.00850	0.07266	0.00965	0.02802	0.00585	0.00790	0.02645	0.01199	0.03714	0.01164	0.00807	0.06138	0.06338	0.14621	0.00734
**Mir17hg**	0.00000	1.29362	0.13994	0.21728	3.33300	0.00561	0.01632	0.80090	0.17686	1.21558	0.90497	0.00000	0.61919	0.00875	0.38446	0.92403
**Mir1949**	0.02853	0.00810	0.00869	0.01047	0.01242	0.00684	0.00732	0.01054	0.01258	0.01022	0.01114	0.00639	0.01310	0.38155	0.00460	0.00755
**Mir1982**	0.04632	0.06017	0.02239	0.00890	0.00802	0.00769	0.01735	0.01650	0.01225	0.11619	0.04897	0.01655	0.10010	0.01076	0.00817	0.02878
**Mir1a.1hg**	0.03119	0.01869	0.01486	0.10732	0.01740	0.01263	0.01825	0.01724	0.13279	0.03197	0.01167	0.00771	0.01217	0.03251	0.04516	0.00734
**Mir22hg**	0.00867	0.00771	0.01547	0.33897	0.00906	0.00683	0.00663	0.00844	0.20421	0.00000	0.00791	0.01416	1.08816	0.05068	1.00777	0.01365
**Mir25**	0.00937	0.00800	0.11779	0.01045	0.01010	0.00596	0.00753	0.17788	0.01219	0.07522	0.01279	0.00722	0.01458	0.01253	0.00535	0.00765
**Mir3064**	0.07089	0.00787	0.00359	0.00863	0.10891	0.36457	0.14450	0.03937	0.01168	0.07358	0.00844	0.05089	0.10159	0.36957	0.20425	0.11158
**Mir3068**	0.01637	0.00787	0.11185	0.01032	0.00907	0.00579	0.00737	0.01264	0.01205	0.01212	0.01286	0.00792	0.01110	0.01088	0.06811	0.00730
**Mir3091**	0.20896	0.00754	0.09677	0.02610	0.00773	0.00630	0.01830	0.01021	0.02394	0.18450	0.09608	0.00726	0.05437	0.01624	0.00447	0.08863
**Mir3471.1**	0.09417	0.03805	0.06133	0.43988	0.00776	0.01234	0.01732	0.01551	0.41182	0.10463	0.00840	0.00588	0.02435	0.24107	0.07480	0.00701
**Mir3471.2**	0.01376	0.00785	0.01203	0.19197	0.00932	0.00661	0.00750	0.01327	0.19548	0.01390	0.01157	0.00739	0.01284	0.01231	0.07422	0.00742
**Mir3473f**	0.01205	0.00763	0.03305	0.15696	0.03663	0.02027	0.01749	0.00512	0.16820	0.02387	0.21341	0.00568	0.01811	0.17381	0.04471	0.00728
**Mir425**	0.01465	0.00807	0.02040	0.01170	0.00927	0.00627	0.00749	0.01361	0.43715	0.01262	0.01052	0.00749	0.01261	0.01207	0.01473	0.00737
**Mir6236**	0.04023	0.11269	0.02702	0.01440	0.10557	0.09196	0.01718	0.03045	0.03980	0.02983	0.01298	0.00725	0.00976	0.01088	0.00870	0.00736
**Mir6385**	0.01598	0.00763	0.03358	0.00701	0.01776	0.00818	0.00701	0.05666	0.01163	0.14487	0.15128	0.00544	0.11035	0.16595	0.00757	0.00754
**Mir6516**	0.01314	0.00806	0.01145	0.00444	0.09060	0.08135	0.00745	0.03577	0.43924	0.01100	0.00902	0.01797	0.00992	0.01254	0.01259	0.00734
**Mir677**	0.01375	0.00818	0.01224	0.01030	0.00932	0.00664	0.00753	0.01378	0.01223	0.01415	0.01322	0.00839	0.01268	0.01053	0.03136	0.00741
**Mir680.2**	0.05471	0.00781	0.01514	0.13471	0.00820	0.00636	0.00737	0.01601	0.06020	0.03198	0.03699	0.03714	0.01178	0.03947	0.08332	0.00726
**Mir682**	0.05381	0.00809	0.02279	0.06815	0.04110	0.01968	0.01807	0.00520	0.30794	0.01605	0.38728	0.00629	0.03354	0.01056	0.03338	0.03607
**Mir684.1**	0.23964	0.24449	0.71155	0.60250	0.24752	0.15418	0.40634	0.53177	0.75924	1.06870	3.87210	0.97984	0.45455	1.36995	0.20925	0.29410
**Mir684.2**	0.11198	0.01894	0.17285	0.15738	0.06428	0.04373	0.03855	0.11111	0.09669	0.30455	0.47696	0.22338	0.07680	0.28413	0.06762	0.01444
**Mir6910**	0.01119	0.00802	0.01025	0.01015	0.00940	0.00661	0.00742	0.10501	0.01203	0.06167	0.01333	0.00845	0.02316	0.01527	0.02282	0.00717
**Mir692.1**	1.71281	0.63815	1.17258	0.16642	0.87103	1.04436	0.74576	0.68039	0.09348	0.53181	0.67791	0.10309	1.60421	0.00653	0.00000	0.54052
**Mir6935**	0.04314	0.00778	0.10822	0.01027	0.00794	0.08396	0.00735	0.03657	0.01221	0.01259	0.01267	0.00815	0.01617	0.01047	0.03149	0.00723
**Mir6951**	0.01318	0.00778	0.08466	0.01758	0.00931	0.00579	0.27428	0.03617	0.01240	0.01263	0.01343	0.00780	0.00987	0.01051	0.02809	0.00732
**Mir697**	0.01270	0.00807	0.11336	0.00968	0.00905	0.00878	0.00735	0.01254	0.01201	0.01537	0.01260	0.00794	0.01111	0.00781	0.11775	0.00735
**Mir7013**	0.01284	0.00793	0.04824	0.10124	0.00907	0.00816	0.00731	0.01151	0.33312	0.16506	0.01199	0.00753	0.01111	0.01758	0.01182	0.00711

**Mir7014**	0.01315	0.00774	0.00990	0.01071	0.00925	0.00673	0.00722	0.12772	0.01223	0.01281	0.01227	0.00745	0.00812	0.01245	0.00863	0.00738
**Mir7024**	0.01263	0.00822	0.01718	0.01005	0.01853	0.00626	0.00755	0.01332	0.01237	0.10296	0.01192	0.00805	0.02387	0.01075	0.01693	0.00713
**Mir703**	15.72767	9.22024	15.04841	0.98959	12.59559	5.11577	6.57794	8.06564	4.09362	8.88567	17.00820	4.86606	11.43050	10.31249	4.91944	7.81702
**Mir7036**	0.12609	0.00753	0.11211	0.01059	0.00736	0.00882	0.34632	0.04936	0.01228	0.00889	0.01263	0.00740	0.08528	0.01327	0.00781	0.12795
**Mir7036b**	0.01369	0.00817	0.01216	0.02030	0.00924	0.00673	0.00759	0.01379	0.10926	0.08349	0.01127	0.00826	0.01254	0.01171	0.01009	0.00731
**Mir7051**	0.01070	0.06181	0.05331	0.03549	0.00916	0.00662	0.00769	0.03724	0.00984	0.02001	0.01219	0.00804	0.02188	0.01616	0.02463	0.00753
**Mir7067**	0.02805	0.00771	0.04848	0.11346	0.00818	0.00764	0.09613	0.10189	0.10563	0.09255	0.01266	0.01765	0.00780	0.01199	0.01632	0.12106
**Mir7069**	0.01335	0.00767	0.03637	0.01060	0.00933	0.00592	0.00719	0.07576	0.01235	0.01282	0.01273	0.00739	0.03198	0.01125	0.01400	0.00731
**Mir7079**	0.05036	0.17587	0.20349	0.19489	0.52655	0.00680	0.00714	0.23519	0.64494	0.20005	0.29365	0.09885	0.21349	0.00548	0.15286	0.26770
**Mir763**	0.01233	0.00764	0.01469	0.01051	0.00947	0.00735	0.00724	0.01000	0.01261	0.00803	0.00915	0.00456	0.01308	0.88457	0.00299	0.00745
**Mir8091**	0.01161	0.00844	0.01250	0.02548	0.01025	0.00713	0.00824	0.06485	0.07271	0.00569	0.01365	0.00801	0.08058	0.01214	0.22494	0.00766
**Mir8112**	0.06962	0.00838	0.00152	0.00972	0.14420	0.13334	0.00760	0.01499	0.01217	0.15084	0.01328	0.01974	0.01760	0.01678	0.00847	0.00716
**Mira**	0.01368	0.00790	0.01136	1.90846	0.00922	0.00669	0.00749	0.01281	0.18736	0.12298	0.02126	0.03002	0.01289	0.00373	0.21302	0.00708
**Mirc35hg**	0.30861	0.02782	0.54650	0.07301	0.15127	0.05924	0.08614	0.00000	2.17801	0.06951	2.26275	0.17528	0.68964	1.52615	0.48425	0.12058
**Mirt1**	0.09812	0.00744	0.21879	0.28679	0.06483	0.03121	0.07827	0.04534	0.88963	0.09270	0.34403	0.09133	0.11232	0.39077	0.11827	0.02158
**Mis12**	1.09538	4.15810	4.41550	7.65829	0.00000	0.72412	2.59826	3.00860	3.89058	2.73521	0.15952	2.07642	0.00000	5.62312	9.16361	1.09978
**Mis18a**	9.78952	0.57911	2.99225	13.01840	2.00711	0.00000	0.96574	7.98827	15.19026	18.84549	6.16126	3.55622	8.54880	2.51242	6.58965	3.16911
**Mis18bp1**	2.12612	2.62413	2.36907	7.63736	0.00341	5.12407	0.05135	1.27839	9.22697	3.45689	2.67588	14.26361	2.08028	5.55264	5.86978	0.02007
**Misp**	0.19219	0.00708	0.17803	0.43860	0.00727	0.00877	0.00696	0.57682	0.09616	0.00699	0.00894	0.00598	0.00488	0.01137	0.00000	0.00700
**Mitd1**	1.77417	4.91200	4.23337	0.00000	2.74101	2.15980	2.63959	0.92627	0.66217	2.72283	0.78331	4.57137	2.15958	4.05714	1.64014	2.66842
**Mitf**	1.78430	0.00708	0.20499	0.14263	0.01959	0.00436	0.00651	0.01009	0.03422	0.02073	0.01247	0.00600	0.12931	0.00558	0.29987	0.00586
**Mixl1**	0.26714	0.00628	0.00000	0.35172	0.00711	0.00586	0.00615	1.16971	0.00363	1.27435	1.33548	1.67692	0.00000	1.41093	1.66651	0.00663
**Mki67**	6.84866	5.25063	4.74744	19.57566	11.96662	0.57446	0.02547	4.76790	20.96991	6.91084	3.55327	5.12368	4.87171	12.31630	16.22458	0.04023
**Mkks**	5.60597	0.49679	4.51554	8.79511	7.70351	4.97268	3.74406	3.03591	3.60522	5.75881	6.88528	9.83889	3.90008	6.44367	9.71565	3.03268
**Mkl1**	1.19999	0.94379	0.29285	1.87527	0.01198	0.00606	2.35478	3.76703	1.08929	0.28624	0.40404	0.05389	1.44512	0.00012	0.05859	0.01213
**Mkl2**	0.06407	0.00740	0.30228	2.50752	0.04587	0.03063	0.23902	0.51056	0.13482	0.38170	0.89033	0.14842	0.10186	0.18421	0.63406	0.19251
**Mkln1**	3.65844	5.61645	1.99402	3.90408	1.06681	3.50552	0.55522	4.25623	1.44195	0.57873	4.44152	6.68132	1.00774	3.04684	2.28542	1.91650
**Mknk1**	1.44880	0.00552	0.95036	4.25207	0.00290	2.30873	0.00513	2.84570	8.36287	4.63609	1.26086	0.57325	0.00000	0.11807	4.42202	1.13247
**Mknk2**	4.24926	0.32855	0.48171	7.67016	0.64362	0.33687	0.98400	5.42185	11.06021	10.60117	8.65174	0.49562	0.84506	0.65362	2.32021	2.03199
**Mkrn1**	21.90979	13.33158	21.22295	16.10314	20.97321	8.62630	21.13364	21.90482	27.55099	33.11440	35.87794	17.45094	22.20912	12.73970	10.30246	5.35947
**Mkrn2**	5.10612	2.38814	7.46550	15.18471	0.01309	0.96717	1.13941	10.18035	15.57859	11.46942	9.28277	0.84778	4.53135	6.73110	17.13359	2.80896
**Mkrn2os**	0.01587	0.00732	0.00000	3.02900	0.01822	0.00695	0.01622	0.00000	3.93823	5.49374	5.25833	0.38690	0.00000	0.69637	3.36681	0.04268
**Mks1**	1.77804	0.02787	1.97721	0.00433	0.01842	0.00607	0.63767	0.06590	0.01088	0.00000	0.31047	0.04700	2.12488	0.02427	0.23454	0.01114
**Mlec**	8.28847	0.36158	9.28943	2.02263	11.10252	9.24467	7.16009	6.12054	1.20399	3.53901	0.98531	2.50042	3.63468	1.68260	3.29266	7.26074
**Mlf1**	0.01295	0.00801	0.02082	0.75826	0.00919	0.00595	0.00760	0.01246	2.12640	0.09304	0.00000	0.00372	0.01197	0.01415	0.06683	0.01403
**Mlf2**	24.38399	6.11258	32.07277	17.01498	19.57434	11.27966	15.19482	37.17365	14.06107	40.08944	33.41572	4.49999	30.37183	1.61337	3.97201	15.65774
**Mlh1**	1.44774	3.25831	1.10045	0.01020	0.20045	0.01629	4.48909	3.34615	1.22903	1.08541	0.89763	1.46882	3.89391	0.00909	0.08871	0.00189
**Mlh3**	1.50103	0.00549	1.62650	0.23783	0.08463	0.01268	0.04538	0.01116	0.35646	0.43247	0.15285	0.04785	0.10516	3.80069	0.89199	0.01936
**Mlkl**	0.01124	0.00709	0.00744	0.21313	0.00951	0.00680	1.49258	0.00961	0.07204	0.00933	0.01158	0.05433	0.01052	0.03249	0.00476	0.00732
**Mllt1**	0.78693	0.00542	0.32520	3.06701	0.10095	0.00453	2.54994	0.35526	4.80027	1.73074	2.68045	0.14388	1.73260	0.00000	0.42297	0.01208
**Mllt10**	2.07032	2.83639	4.70243	7.74561	0.01289	0.01207	1.05864	5.87211	2.63487	3.43313	2.93053	4.74100	2.28437	9.06139	11.22132	0.98051
**Mllt11**	1.38797	0.00602	0.45804	6.26788	0.00560	0.00318	0.04614	0.00000	4.66311	0.52726	0.46526	0.00000	0.00775	0.14238	1.29675	0.01147
**Mllt3**	0.87437	0.00653	0.81056	8.29064	0.67309	0.00378	0.11591	0.01947	3.11196	0.70850	0.54317	0.04162	0.01656	1.41960	2.51470	0.71824
**Mllt4**	6.05562	0.05187	2.51095	9.71311	1.77554	3.66094	2.66207	2.54383	2.51028	8.52467	2.08509	8.59705	3.59969	9.28766	4.60900	0.63382
**Mllt6**	5.38165	5.86556	3.78125	2.48239	0.01460	5.92753	6.07672	6.93529	1.46235	5.08709	6.34027	0.69615	6.66113	2.15804	2.40255	8.41888
**Mlph**	0.02936	0.01813	0.02840	0.21127	0.00834	0.00722	0.01760	0.02138	0.03005	0.03258	0.01255	0.01743	0.01879	0.00909	0.02840	0.00732
**Mlst8**	4.01955	6.39977	7.84881	0.98492	3.84769	0.40580	4.04253	7.34509	2.10248	3.56522	4.09880	0.32447	2.58903	5.68996	4.42806	0.03244
**Mlx**	5.57973	3.80636	0.23938	0.35253	8.31305	3.09483	0.29376	4.90565	1.20270	0.71291	1.29054	0.53177	1.65631	2.73713	4.10328	2.30923
**Mlxip**	2.84058	0.00344	0.22427	3.09974	2.48758	2.34984	0.00465	1.71829	1.46226	2.82978	0.00000	0.39180	0.48873	2.22964	5.51684	0.27943
**Mlxipl**	1.59139	0.00512	0.90471	7.39692	0.01082	2.04823	0.66960	0.29316	7.53158	1.37602	6.34936	0.00000	0.38412	0.00000	0.47934	0.00183
**Mlycd**	0.28932	1.22079	0.48732	0.19709	2.04687	0.01707	0.23326	0.80752	0.00312	0.00571	0.00715	0.00408	0.00538	0.00944	0.00000	0.00639
**Mmaa**	3.05967	4.00046	3.53770	0.23792	2.52530	0.70843	1.59377	2.58949	2.97980	1.18670	1.30133	0.27644	1.66366	0.82404	1.00843	0.03592
**Mmab**	1.35635	0.89389	1.68560	0.45269	0.00834	0.00414	0.00498	0.31680	0.02041	0.92673	0.00681	0.00000	1.37473	0.00392	0.36985	3.17615
**Mmachc**	4.97889	4.41475	6.53204	0.03499	3.73255	3.44084	4.08158	1.70002	0.82530	2.82534	1.97431	0.13594	0.11342	4.62797	4.32480	0.05413
**Mmadhc**	15.58909	8.76991	9.44666	3.52235	11.41933	8.67956	3.60092	8.19066	0.52858	4.22968	3.49632	9.70039	6.18386	1.40941	3.40202	2.25205
**Mmd**	0.01351	0.00782	0.01069	0.21133	0.00922	0.00674	0.00752	0.01209	0.58990	0.19317	0.00492	0.01771	0.01320	0.01069	0.70392	0.00735
**Mmd2**	1.08095	0.00618	0.00315	0.00476	0.00586	0.00738	0.00649	0.00000	0.24933	0.19605	0.50971	0.01284	0.01126	0.01197	0.16351	0.00652
**Mme**	0.25728	1.63065	0.82750	0.00857	0.00732	0.00557	2.29437	0.92331	0.01168	0.06425	0.00711	0.00270	0.00000	0.02492	0.00000	1.56412
**Mmel1**	0.01394	0.00785	0.00703	0.00900	0.00917	3.05771	0.00706	0.00951	0.01281	0.00406	0.00947	0.00806	0.13663	0.01350	0.01013	0.00608
**Mmgt1**	1.25416	0.11334	2.81820	8.68817	1.62546	1.65015	2.97623	1.80874	5.73586	5.40467	4.72024	5.76928	6.03077	10.07638	9.63858	0.82196
**Mmgt2**	0.00329	0.00730	1.47028	0.92915	0.01544	0.01772	0.01578	0.58061	0.08392	1.84073	1.63638	0.08035	1.26321	0.00144	0.71779	0.01270
**Mmp11**	1.78660	0.00631	0.00354	0.13546	0.00723	0.00659	0.00607	0.00951	0.00689	0.01466	0.01171	0.01680	0.01132	0.00982	0.02309	0.00554

**Mmp12**	2.28470	3.66486	0.05837	0.82839	0.00323	0.00351	0.00409	0.00000	0.00000	0.00076	0.01561	0.00542	0.01290	0.00519	0.00000	5.14883
**Mmp14**	0.02359	0.00784	0.03421	0.01774	0.01915	0.01363	0.00722	0.01878	0.10183	0.77243	0.04812	0.07071	0.01072	0.10890	0.02604	0.00768
**Mmp15**	2.42021	0.00160	0.93142	0.47264	6.37739	0.70437	0.00537	0.16171	0.01553	0.05172	0.00909	0.00003	0.00000	0.00572	0.11813	0.07611
**Mmp16**	0.04600	0.00771	0.03631	0.29217	0.00811	0.01252	0.00726	0.01456	0.19756	0.02938	0.15616	0.00648	0.00973	0.00726	0.07658	0.00710
**Mmp17**	0.01805	0.00792	0.00688	0.14759	0.01036	0.00698	0.00731	0.00000	0.46339	0.03342	0.72147	0.01371	0.05992	0.01361	0.14223	0.00724
**Mmp19**	0.01465	0.00772	0.11208	1.38218	0.00861	0.00826	0.00708	0.00726	5.32922	0.00000	0.00000	0.01176	0.01204	0.53364	1.47555	0.00709
**Mmp1b**	2.47473	0.00554	0.49258	0.00767	0.01011	0.00297	0.00520	0.00728	0.01117	0.00500	0.01141	0.00520	0.01548	0.00709	0.50166	0.00464
**Mmp2**	0.01367	0.00812	0.01122	1.96758	0.00940	0.00638	0.00744	0.01425	0.00000	0.00614	0.01171	0.04003	0.01262	0.00335	0.46282	0.00703
**Mmp21**	0.14551	0.00775	0.00873	0.01100	0.00815	0.00731	0.00732	0.01643	0.01196	0.00365	0.01392	0.00738	0.58347	0.02155	0.06076	0.00710
**Mmp23**	1.50237	0.00572	0.00000	0.07817	0.00452	2.15923	0.00587	0.45888	0.01080	1.96294	0.04881	0.01073	0.00000	0.01196	0.00000	1.08370
**Mmp24**	0.09241	0.00752	0.00727	0.01356	0.00780	0.00678	0.00707	0.01027	0.17793	0.85598	0.01002	0.06941	0.11673	0.00617	0.11133	0.00718
**Mmp25**	0.03313	0.00750	0.13734	0.03042	0.00789	0.00887	0.02777	0.00625	0.28340	1.23590	0.43967	0.02887	0.06062	0.94665	0.45662	0.00714
**Mmp9**	0.01206	0.00771	0.01693	0.22887	0.09484	0.00722	0.00726	0.04460	0.00768	0.01758	0.01365	0.00763	0.02248	0.01103	0.00802	0.00732
**Mmrn2**	0.00603	0.00740	0.00534	0.19442	0.00716	0.00628	0.00701	0.00473	1.60315	0.95456	0.36465	0.09700	0.00423	0.00284	0.81267	2.51215
**Mms19**	7.96636	6.62298	8.69804	3.15894	1.60414	4.78581	2.89998	7.01032	7.83213	8.97688	5.33682	1.83601	1.57959	2.12139	3.69510	6.69285
**Mms22l**	3.81893	2.82258	2.94834	6.55852	5.40669	0.22076	0.70279	5.41152	2.93830	9.67222	4.02959	9.63297	2.05789	7.95317	4.58609	1.37505
**Mn1**	0.01267	0.00765	0.00670	1.85633	0.00934	0.00628	0.00726	0.00869	2.72818	0.00000	0.02410	0.03899	0.01331	0.55566	1.27150	0.00688
**Mnat1**	10.13975	8.59073	7.46997	3.39586	7.26295	3.59681	2.75137	8.02440	5.42174	3.16650	5.66488	1.29569	3.67376	6.06908	4.21385	7.22518
**Mnd1**	0.95536	0.03569	1.93747	0.55292	0.02678	3.48510	2.83975	0.00000	1.29724	0.72168	2.31158	0.04939	0.80456	0.00211	0.87069	6.86188
**Mnd1.ps**	0.12704	0.00696	0.56236	0.36285	0.00623	1.06945	0.82797	0.00553	0.50177	0.19254	0.81281	0.03309	0.14103	0.00424	0.24151	2.12366
**Mns1**	0.00071	0.00590	0.35137	0.15406	0.01027	0.00546	2.36944	1.26104	0.34034	1.09660	0.03857	0.84989	0.00396	0.59360	1.70574	0.00707
**Mnt**	1.64073	0.01614	0.25262	1.46337	0.01270	4.90313	0.03595	4.88534	2.22958	3.49903	1.23851	0.69095	1.30192	0.32679	3.20672	0.02432
**Mob1a**	0.03906	0.00728	0.01308	5.16768	0.04522	0.15771	0.01735	0.15585	1.95368	0.66690	0.90252	0.92469	0.06884	0.77671	0.39592	0.00638
**Mob1b**	0.74441	0.00655	0.27275	7.60734	0.00568	0.01540	0.00522	0.14347	1.88262	1.24564	2.61513	0.24861	0.50090	7.21568	1.49141	0.71162
**Mob2**	3.64749	3.21766	6.03781	2.74195	1.70104	0.69732	2.29425	4.45021	2.32116	9.20786	2.16126	0.37031	3.52675	0.00000	0.53593	1.20893
**Mob3a**	3.80488	0.00343	0.00000	0.36216	0.01214	3.09240	0.00311	0.00000	2.00077	0.00000	0.16210	0.04658	1.36901	0.00342	0.23821	2.94479
**Mob3b**	0.20056	0.01848	0.60343	0.27354	0.02596	0.03272	0.07792	0.00000	0.95782	0.35281	2.92204	0.05040	0.60922	0.40505	0.12218	2.04901
**Mob3c**	0.81645	0.01679	2.83443	0.30383	0.00167	0.05677	0.17949	0.06103	0.35125	0.75977	0.12774	0.02985	0.12918	0.26246	0.22115	0.00515
**Mob4**	6.72048	5.13392	6.25095	9.87900	4.34233	5.04759	4.54309	6.92499	12.99357	13.96992	8.25345	12.36316	8.91343	7.66406	8.19402	1.49420
**Mocos**	2.82368	0.00457	0.00000	0.00403	1.75304	2.86467	0.00483	0.85183	0.31726	0.48382	2.77111	0.02616	0.00000	0.00470	0.09489	0.00265
**Mocs1**	4.44674	0.00302	0.03896	0.04918	0.00963	0.00401	0.03437	0.00000	0.00956	0.01924	0.02205	0.19842	0.97145	0.01166	0.00712	2.20524
**Mocs2**	0.00000	0.00780	0.16846	1.07701	0.01080	0.00601	0.03795	1.06092	0.12686	0.29390	0.03904	0.00000	0.06643	1.84427	0.00000	1.78715
**Mocs3**	0.47539	0.89897	0.03492	4.37223	2.03902	2.34283	0.00491	0.00000	9.55394	2.46012	1.44409	0.04631	2.21035	0.02249	1.06117	0.17739
**Mog**	0.01616	0.00739	0.36415	0.14705	0.00854	0.00544	0.00718	0.00995	1.51256	0.00324	0.00000	0.00344	0.00948	0.00796	0.57929	0.00692
**Mogat1**	0.01366	0.00807	0.01227	0.47517	0.00938	0.00648	0.00738	0.01235	0.31060	0.14264	0.01196	0.00698	0.01256	0.00894	0.02218	0.00738
**Mogat2**	1.68219	0.00420	0.75260	0.00374	0.00226	0.00374	2.47052	0.00000	0.01106	0.00000	0.38268	0.03252	0.02494	0.01254	0.00000	4.02363
**Mogs**	2.97055	0.01163	4.12287	2.41089	0.01842	0.06007	2.91651	6.11969	6.49726	4.03455	3.53875	0.15917	7.24195	0.18004	1.92900	5.05329
**Mok**	0.80623	0.00681	1.17465	0.00506	0.00637	0.47623	0.00608	1.63464	0.03789	1.49023	0.03941	0.07844	1.01610	0.01305	0.00000	0.11101
**Mon1a**	3.17980	0.27120	0.81180	9.72213	0.02483	4.15332	0.09900	2.20443	12.78898	25.58490	12.24069	2.32442	13.34131	0.00000	1.08117	0.01423
**Mon1b**	0.12474	2.15514	7.11818	2.94455	3.29398	5.89969	0.05759	1.66757	2.68854	2.67913	0.41723	0.10936	0.50176	0.99847	1.03923	0.00794
**Mon2**	6.78635	2.16470	2.92797	10.55718	3.35059	4.45130	4.42841	6.36338	4.38867	6.71309	4.42049	2.88206	3.51909	5.34262	7.05463	2.86083
**Morc1**	4.63097	0.40336	0.00000	0.98415	1.27294	0.37529	0.01379	1.56125	0.58791	0.81235	0.59102	0.02624	1.01836	2.72807	0.00000	3.12506
**Morc2a**	2.48538	1.25551	4.23197	6.96963	0.80134	6.17135	2.32065	7.50513	5.01721	7.93413	3.59032	8.68864	3.10612	11.71878	15.69545	2.45316
**Morc3**	7.54591	7.47442	3.55534	2.26487	2.36319	4.60861	1.79831	11.92207	4.62043	8.67056	5.00715	8.42188	2.19856	10.65072	14.29108	0.45346
**Morc4**	1.12708	0.00661	0.00403	0.40801	0.00572	0.00694	0.00670	0.01020	0.25133	0.01426	0.01190	0.00719	0.01161	0.01159	0.00480	0.00609
**Morf4l1**	22.75051	17.26788	17.45941	5.28166	15.23208	10.79057	12.53109	12.52453	4.85103	8.36398	9.42554	1.63398	10.73956	20.58893	15.06690	9.97518
**Morf4l1.ps1**	14.38892	11.30858	10.06841	2.21434	9.13605	6.72102	7.79368	7.15089	2.00700	4.05631	4.84621	0.66142	5.48601	12.99860	8.76110	5.69390
**Morf4l1b**	13.71732	11.04173	9.15499	2.08282	8.72046	6.27099	7.61866	5.95818	2.33160	3.69439	4.95773	0.54900	5.19295	12.61978	8.81634	5.15221
**Morf4l2**	24.66010	16.87151	29.05960	9.71531	19.24418	8.74757	10.54520	13.68486	8.32115	9.24772	5.42181	1.02160	18.05601	9.19069	10.01099	16.95299
**Morn1**	0.00873	0.00787	0.01447	0.49327	0.00995	0.00789	0.00734	0.84956	0.27711	0.43131	0.21510	0.00334	0.01206	0.00615	0.01991	0.00727
**Morn2**	2.51310	0.33000	0.52619	0.02248	0.01073	0.00944	0.29035	0.61320	0.81901	0.32356	0.69821	0.01553	0.00578	0.33392	0.00000	1.14474
**Morn4**	1.12961	0.00683	1.22830	1.85768	0.00422	0.00776	0.00604	0.00000	0.59384	0.31751	0.43852	0.01200	0.71437	0.02154	0.54296	0.00514
**Morn5**	0.01382	0.00819	0.00624	0.00536	0.00903	0.00720	0.00752	0.00094	0.02450	0.01984	1.04619	0.02563	0.00880	0.01115	0.00000	0.00699
**Mos**	0.01356	0.00791	0.01162	0.14762	0.00928	0.00649	0.00729	0.00959	0.41029	0.41983	0.05318	0.01544	0.01144	0.01093	0.00250	0.00740
**Mospd1**	2.60442	2.86035	2.01404	15.73090	3.38364	2.54731	1.28192	8.40207	11.31060	12.13030	9.91735	7.05788	1.99815	35.47696	23.59053	0.00000
**Mospd2**	1.39967	0.07464	0.39917	1.23918	0.08823	2.07806	2.96362	1.54541	0.00000	0.00000	0.41486	0.00000	0.66441	0.00247	0.73404	0.45850
**Mospd3**	4.52799	1.13428	1.63919	2.59684	3.00586	0.04875	0.68381	1.66946	1.56571	8.50263	2.02550	1.32319	6.14225	0.00000	0.09128	5.02723
**Mospd4**	0.01238	0.00822	0.01286	0.71916	0.00910	0.00657	0.00735	0.01035	0.08109	1.31273	0.01109	0.00452	0.12653	0.00989	0.00358	0.00703
**Mov10**	8.29353	1.60410	1.30122	0.00000	3.80983	0.13389	0.00983	4.82812	0.13186	10.40664	1.89034	0.22653	0.00000	0.01994	2.77616	2.96536
**Mov10l1**	0.07504	2.28613	1.79559	0.68918	0.00369	0.00518	0.00610	0.00000	2.05188	1.19461	0.54590	0.01483	0.00000	0.00181	0.70112	2.31215
**Mpc1**	11.76379	7.73220	13.48115	14.12033	11.35642	6.05039	8.16981	13.20453	23.43521	24.74633	22.51609	17.33447	15.08077	7.43832	6.24850	2.16093
**Mpc2**	5.00101	0.11432	1.30181	15.47559	0.04418	0.65933	0.12696	0.64442	26.96434	29.48520	23.58917	1.37428	18.15402	3.05176	0.00000	2.28950

**Mpdu1**	9.57034	4.26908	16.03733	2.64661	4.34757	10.02082	11.16512	19.33206	4.45924	10.79889	6.05416	0.57493	9.50476	0.05056	5.99368	6.59187
**Mpdz**	0.60155	0.00567	0.00000	9.50952	0.22788	0.00425	1.50838	0.00364	2.84090	0.83603	0.17062	0.00000	0.00569	2.06563	5.42094	2.06235
**Mpeg1**	0.01624	0.00797	0.01040	0.00848	0.01046	0.00680	0.00729	0.07047	0.01191	0.53752	0.01197	0.00629	0.00970	0.01160	0.00920	0.00775
**Mpg**	4.94289	0.00953	1.67211	0.58588	6.00217	0.48121	0.16783	2.19900	0.08761	0.17195	0.42909	0.01881	2.36773	0.00460	0.02325	3.89445
**Mphosph10**	7.07722	7.16850	7.21568	12.74094	9.35657	2.51112	2.38584	9.41085	15.41434	9.76933	7.09053	14.42520	3.42401	16.23773	13.89884	3.51081
**Mphosph6**	8.33746	2.89355	4.96238	15.77402	10.22081	2.60898	6.42574	9.66012	7.11566	8.52354	6.02313	1.93295	4.03567	16.56402	12.74431	7.07519
**Mphosph8**	3.28045	7.05223	3.37130	0.14378	2.50890	6.14607	1.91338	3.65026	0.50633	1.82592	0.47924	0.56908	4.55887	1.36810	6.37568	3.23376
**Mphosph9**	2.24820	2.02862	0.00000	7.36511	3.09538	2.45365	2.59410	1.35973	3.55523	2.92006	0.63564	8.28296	0.07306	0.32236	9.07065	0.10987
**Mpi**	4.69526	0.00000	2.82038	0.72507	3.91509	0.00093	0.00000	0.00000	0.80879	2.76931	1.42278	0.06163	3.92376	3.08694	1.96645	3.89392
**Mpl**	0.00815	0.00829	0.00908	1.23379	0.00869	0.00635	0.00786	0.12182	0.00000	3.20801	0.00822	0.00000	0.36413	0.00854	0.77077	0.00691
**Mplkip**	2.22083	1.20043	0.60406	5.51521	0.10739	0.01873	1.39727	0.07458	8.98706	3.54432	2.92288	0.33782	1.54948	1.42811	1.65496	1.00955
**Mpnd**	4.27165	0.01203	0.05165	0.21183	2.61021	0.01523	0.01272	0.80215	0.00517	0.00923	0.00630	0.01803	2.04238	0.00533	0.48859	0.00901
**Mpp1**	13.81952	6.91662	16.66091	7.59045	9.91028	6.39072	6.50741	3.66123	7.46912	14.76784	1.60127	7.93263	5.39332	11.10280	15.07879	8.72761
**Mpp3**	0.02449	0.00741	0.00425	0.08377	0.00840	0.00702	0.01675	0.10588	0.00975	0.40911	0.57160	0.01320	0.00785	0.08558	0.52462	0.00692
**Mpp4**	0.02670	0.00777	0.23773	0.17076	0.00794	0.00552	0.00614	0.12763	0.30253	0.55780	1.82007	0.10828	1.24577	0.56814	0.00000	0.00641
**Mpp5**	2.10924	0.00311	0.64600	4.43599	0.00336	1.05848	0.01484	0.00657	3.81140	0.53470	0.00000	6.46775	0.08149	0.02139	4.39291	0.32228
**Mpp6**	4.49701	5.99970	0.53303	1.94022	0.06633	2.60078	1.44647	1.37195	3.74695	6.98882	2.49225	7.97132	0.95094	4.31169	2.56502	3.17525
**Mpp7**	4.62328	1.52066	0.18188	0.15272	0.07086	0.60119	0.18038	1.15257	0.25801	0.01764	0.68008	0.19204	2.61746	1.33219	0.18413	1.36340
**Mppe1**	0.31660	0.07611	1.30482	0.01606	2.56679	0.58434	1.32823	1.65444	0.00584	1.37050	0.03179	0.12398	0.17712	0.04693	0.66613	0.17318
**Mpped1**	0.01373	0.00817	0.01176	0.75684	0.00943	0.00642	0.00735	0.01166	0.00000	0.00539	0.01204	0.00717	0.01281	0.01606	0.54477	0.00725
**Mpped2**	2.14880	3.69420	1.81386	0.18892	0.98972	0.13446	0.36918	0.35872	0.00459	0.00124	0.00814	0.00356	0.00000	0.00999	0.00000	1.30465
**Mprip**	10.07595	5.81981	9.03217	14.39680	4.73124	8.08925	11.07602	17.82465	8.45799	13.27799	7.19866	5.88801	11.35670	2.70591	8.58381	7.25162
**Mpst**	0.01773	0.00786	0.01197	0.02606	0.00902	0.00650	0.00733	0.01193	0.01056	0.29403	0.01240	0.00681	0.01873	0.01130	0.00968	0.00742
**Mpv17**	3.74796	0.77717	2.62906	4.34070	0.30527	2.91368	0.12143	2.02885	7.41349	0.00000	5.38338	5.57418	0.64090	1.85669	2.67959	0.34760
**Mpv17l**	2.68379	0.12872	2.57150	11.37481	1.30874	0.29964	0.31317	0.00000	16.97210	0.17134	14.03228	1.64670	1.06690	6.46777	1.47141	0.56289
**Mpv17l2**	1.26655	0.00623	3.31067	1.20020	0.21821	0.04106	0.22478	0.00000	0.98129	3.80279	1.94084	0.05115	1.48996	0.26071	0.06367	2.27496
**Mpzl1**	0.98238	3.50037	0.98470	2.85897	0.01305	0.00497	3.72017	0.00297	0.10213	2.10582	0.14290	0.01796	0.15878	0.00000	0.82668	0.03609
**Mpzl2**	3.23878	0.90759	5.41749	0.19084	3.25931	5.22315	4.87183	5.22457	1.82505	1.55923	2.12667	7.33367	4.44626	4.34185	0.00000	1.87791
**Mpzl3**	0.01808	0.00817	0.01829	0.09273	0.01058	0.00725	0.00751	0.01362	0.00789	0.01859	0.01212	0.00784	0.01240	0.00971	0.02352	0.00740
**Mrap**	0.38837	0.00718	0.00000	0.40584	0.01718	0.00840	1.40298	0.00811	0.03372	0.80285	0.00992	0.02391	0.01088	0.00485	0.42262	0.00729
**Mras**	8.48342	4.52296	5.12816	1.19816	3.45193	5.56982	5.76640	2.51215	0.64149	0.65204	0.37585	0.49644	7.99498	0.00175	0.10712	6.29549
**Mrc1**	0.01365	0.00811	0.00840	0.50760	0.01005	0.15676	0.00766	0.01351	0.55416	0.00848	0.00706	0.01709	0.05646	0.00752	0.90646	0.00746
**Mre11a**	6.50042	2.22053	2.52988	2.01486	1.39846	0.09472	7.03709	3.02092	1.56745	3.51573	4.33153	2.30965	1.20107	2.92767	1.36899	2.83480
**Mreg**	6.57378	3.80657	10.20701	0.88768	3.68806	4.46435	6.51714	8.18449	0.63877	6.70641	8.44919	0.49281	7.80380	20.26213	6.91668	4.20604
**Mrfap1**	22.86793	8.41185	18.78246	6.65902	7.72576	8.80965	11.37350	11.08941	11.51228	21.03283	20.58616	2.91557	17.90025	12.97556	9.20384	11.44258
**Mrgbp**	0.21101	1.97767	0.78129	0.00901	0.01091	0.00658	0.00627	0.21834	0.01221	0.03208	0.01097	0.00672	0.46501	0.05345	0.01376	0.00621
**Mrgpra6**	0.86754	0.73915	2.80396	1.16482	0.51568	0.33629	2.34071	2.71240	3.01360	2.42231	0.99414	0.64640	1.10283	16.06625	2.36476	0.29077
**Mrgprb1**	0.03674	0.00796	0.05693	0.07202	0.01833	0.00693	0.01807	0.01046	0.14066	0.03773	0.18146	0.04633	0.06761	0.24224	0.02961	0.00717
**Mrgpre**	0.01111	0.00800	0.03289	0.02454	0.05912	0.01510	0.01814	0.04056	0.02439	0.04589	0.01257	0.00749	0.02840	0.08582	0.03480	0.03017
**Mri1**	3.38176	0.63200	0.93050	0.85994	2.44917	0.97646	1.13357	1.02204	0.98433	2.42625	3.11387	0.48962	0.42663	0.00812	0.00000	1.96877
**Mrln**	0.01370	0.00811	0.01209	0.30772	0.00930	0.00664	0.00751	0.01370	0.00578	0.01496	0.01323	0.01865	0.01264	0.01127	0.00613	0.01468
**Mrm1**	0.00000	0.01551	4.28510	2.83078	0.39185	0.03631	2.58476	1.00454	3.02770	0.65088	0.00000	0.11392	0.91199	0.00000	1.59977	2.26651
**Mrm2**	4.43737	0.01010	4.88804	0.00000	2.35830	3.96419	1.52027	5.19689	0.22689	4.75837	1.82376	0.31391	4.70028	0.00002	0.62971	1.63182
**Mro**	0.02669	0.00803	0.03797	0.51086	0.00849	0.00768	0.00768	0.01556	0.50537	0.20373	0.03478	0.00776	0.01391	0.02953	0.00432	0.01462
**Mroh1**	2.45821	3.44001	2.84288	0.25010	1.59037	5.08732	2.99692	0.00000	0.51529	0.89412	1.23142	0.01106	1.03809	0.31733	0.52097	0.00769
**Mroh5**	0.01331	0.00803	0.01217	0.17316	0.00901	0.00652	0.00733	0.01046	0.18370	0.83967	0.00993	0.00537	0.01105	0.01086	0.00541	0.00732
**Mroh8**	0.00898	0.00749	0.00000	0.31998	0.00932	0.00610	0.00629	0.00000	1.56397	0.30487	1.69977	0.18023	0.00000	3.36425	1.37471	0.00700
**Mrpl1**	7.36577	8.15155	1.75437	0.03735	2.04374	4.15733	4.17036	2.71385	0.21208	1.47923	2.11386	8.25303	4.85039	6.58646	3.14644	4.01686
**Mrpl10**	8.05588	1.60212	8.13149	4.07058	2.84759	1.90342	3.56929	8.80665	2.86946	5.68675	3.63924	0.24943	5.88338	1.32093	3.58350	4.26460
**Mrpl11**	15.92629	4.15972	10.34195	2.81705	10.81958	8.17422	8.89472	7.69524	3.09268	5.21679	8.61169	2.93539	12.68752	0.95019	1.12856	4.23796
**Mrpl12**	20.38600	3.03222	10.12564	8.81565	9.85239	10.01107	5.49698	14.15686	14.84438	27.18480	13.27736	2.34143	17.88861	0.67170	1.01327	14.03745
**Mrpl13**	10.42522	9.99687	11.08881	1.97311	9.83253	5.39539	7.65985	8.55121	2.32760	8.63565	8.55604	3.18206	6.16627	3.59698	2.17692	6.07057
**Mrpl14**	7.58324	0.16412	2.48824	0.19746	7.71102	2.12708	2.36881	5.54513	0.20636	9.14928	5.08878	0.09558	3.28657	0.00000	0.16348	3.56607
**Mrpl15**	23.76298	4.98265	11.68071	2.47535	12.31235	8.14082	4.77471	19.74789	5.99344	24.58284	22.23528	11.30880	13.20133	5.81570	2.50744	12.21557
**Mrpl16**	9.86878	7.09130	6.56108	0.79841	9.62776	3.23259	1.38740	9.80593	2.00461	9.11017	6.73325	2.04998	4.07203	1.29757	0.94840	4.41995
**Mrpl17**	21.92933	1.46707	9.31869	0.63623	18.02450	8.15174	7.14900	22.20321	0.64156	13.87459	14.32384	1.39329	24.47617	3.90418	0.00000	15.35293
**Mrpl18**	18.44902	11.98190	20.25813	7.38125	14.47948	8.20968	9.16726	19.14758	7.51538	22.39091	11.83405	9.67916	18.14421	5.07663	7.45748	10.53120
**Mrpl19**	12.36150	4.87552	7.79422	1.14083	10.85217	5.66273	4.63381	8.83082	0.84722	10.50791	4.49678	1.17155	11.64203	7.52377	1.97711	1.13417
**Mrpl2**	10.35267	0.95919	7.68348	1.21963	6.20116	6.16192	5.01729	18.86898	2.03682	6.38560	6.33690	0.18840	14.99631	0.01085	0.00000	9.42860
**Mrpl20**	15.29765	5.30622	13.39461	9.61944	7.54972	5.94719	2.35353	13.16500	13.17774	23.79465	15.78819	2.49629	16.73444	2.09292	3.77762	7.85350
**Mrpl21**	10.33875	4.23612	5.75144	0.29884	11.44671	3.26446	1.16272	2.86123	0.71934	9.60636	6.82566	3.57798	9.00144	0.68942	5.24305	4.33855
**Mrpl22**	12.25545	7.01434	13.04792	0.74225	6.86656	5.99818	7.60736	19.29551	0.23273	23.92150	18.31530	6.51020	19.17632	12.96212	14.09845	3.82494

**Mrpl23**	8.61600	0.67646	4.34198	2.28370	4.46485	2.21310	1.54851	1.12154	1.10023	5.20921	14.65979	0.33214	5.81801	2.59018	1.16381	2.68558
**Mrpl24**	11.24510	3.19795	14.51157	0.05200	5.59407	1.41224	3.47755	10.13018	0.09752	5.54379	2.13392	0.17308	6.72439	5.91698	4.38578	6.12260
**Mrpl27**	10.38579	5.00455	2.05135	3.12511	0.08619	3.09096	3.36490	6.94357	5.50099	5.51051	9.45729	0.80633	5.58642	0.98600	1.08374	2.93865
**Mrpl28**	18.33186	3.88170	11.19306	3.17682	7.44222	8.89658	9.92601	17.34913	0.89183	10.69160	5.01204	1.26580	16.51887	0.00061	0.00000	12.15575
**Mrpl3**	6.94464	5.96636	4.12397	3.51886	4.83882	5.13849	2.62718	2.93100	0.97798	9.37698	5.25468	7.10023	5.47838	3.25634	3.46666	4.24779
**Mrpl30**	14.94303	3.70764	12.62698	1.75369	5.93817	4.84618	6.97378	10.02509	0.36266	12.43909	12.35661	2.20891	10.04450	3.81552	3.62172	3.30870
**Mrpl32**	11.23687	2.40921	4.67765	3.97714	4.30579	2.90875	3.63531	5.49166	5.41060	9.73556	8.33600	1.68577	4.69862	1.30693	3.16641	1.44175
**Mrpl33**	6.94690	0.36550	4.94248	5.44828	2.38169	1.27291	0.45973	0.50387	3.09226	1.89809	15.36949	0.43913	1.65810	4.55179	1.50551	0.64470
**Mrpl34**	10.29127	0.00000	0.00000	9.52875	3.15254	2.87358	0.15656	3.94079	11.43286	10.65171	2.10125	0.14435	5.64695	0.01929	0.00000	4.41903
**Mrpl35**	14.34040	0.54203	8.51227	1.82664	7.44427	5.23020	4.42218	10.60944	2.98301	14.00762	12.20122	2.34724	5.70751	7.36251	4.87947	4.84836
**Mrpl36**	16.98384	1.25822	5.70201	0.00000	9.18655	6.77323	2.62262	7.84800	0.07118	6.66387	4.68381	1.54974	11.14979	0.78947	0.00000	5.00797
**Mrpl37**	7.88511	4.44517	3.80700	5.06403	6.32119	4.59181	2.33450	8.94896	0.83104	9.48960	6.11517	6.58488	10.14785	0.11308	1.41583	3.78333
**Mrpl38**	8.28772	5.02571	3.17583	3.53644	2.86485	3.84251	0.44555	3.42538	2.72679	5.67034	4.26774	0.51521	6.77247	0.11579	1.15228	6.56216
**Mrpl39**	11.88113	4.17508	2.58491	0.80547	8.20108	5.56936	5.25034	10.03010	2.01224	6.26832	10.90136	6.01183	7.17462	2.93198	0.74325	4.18684
**Mrpl4**	19.48572	1.69859	7.03025	7.55961	13.88862	11.95802	6.39991	21.27727	12.04364	21.64120	7.54096	3.31233	19.00093	0.92007	1.80066	11.24991
**Mrpl40**	19.21387	10.38813	15.54986	14.55394	9.03137	7.58507	7.75829	14.86421	24.79763	26.86156	21.16248	9.00607	13.81309	7.42877	5.45686	6.43143
**Mrpl41**	8.65072	4.66081	9.25014	7.69185	8.07613	0.58723	5.15159	5.26737	10.47057	8.44400	6.51409	1.05693	3.64469	1.05280	5.19121	6.30406
**Mrpl42**	14.41278	6.28460	11.63926	4.66637	6.02468	7.46102	3.60829	2.56969	4.67655	10.06124	13.13578	1.88293	8.85591	6.24765	4.36553	5.58930
**Mrpl43**	13.89859	0.93653	1.10478	4.61121	12.11794	6.54767	1.31334	11.95132	12.10958	16.47150	6.81827	1.97631	6.34593	1.20162	0.61579	5.00780
**Mrpl44**	14.52694	2.26338	10.46711	3.36942	16.27839	6.61223	8.06228	20.20857	1.20201	18.86967	9.27607	1.46866	12.90120	7.93916	5.39061	6.55092
**Mrpl45**	13.32960	12.79452	13.24195	6.43859	12.67774	6.78097	9.89916	12.68784	5.64094	14.22565	7.04082	1.58030	8.73436	1.51883	8.28397	6.98203
**Mrpl46**	13.93745	10.52927	10.55255	2.42346	10.20623	4.66585	7.30661	14.68339	3.71754	19.24333	8.44922	1.21654	12.14718	1.02682	0.97670	8.84117
**Mrpl47**	10.84159	8.24718	9.34420	0.93084	9.28267	8.39028	5.86000	7.54337	2.06272	8.24482	10.09889	3.33765	9.08976	5.83547	6.24361	6.06005
**Mrpl48**	11.80093	4.24613	7.03166	11.65262	8.90549	3.33304	3.36456	8.70659	11.21471	7.97592	9.44727	7.04281	8.17795	5.34305	5.87350	3.02844
**Mrpl49**	9.94736	4.81040	13.73627	4.85338	7.21138	3.36634	6.41969	10.71471	8.84616	13.81992	9.48627	4.12868	12.98900	0.57361	4.85958	5.48710
**Mrpl50**	8.43166	5.35651	5.26926	0.81287	6.84899	2.50956	0.00000	2.49262	1.55959	3.90599	2.49730	2.58057	6.41949	2.57198	5.50480	1.00974
**Mrpl51**	18.10760	6.94644	9.73265	10.31198	9.52411	6.83221	1.08487	11.31717	19.70456	14.54674	15.23136	1.06048	10.38088	1.26637	6.97341	6.26406
**Mrpl52**	3.45117	0.02371	0.20322	0.43673	3.57530	0.27778	0.11255	0.32633	0.65309	1.19774	2.78051	0.09734	3.42956	0.19098	0.00000	3.21401
**Mrpl53**	12.64057	3.11560	3.91508	1.75174	9.49882	3.27771	3.55285	2.62101	4.25684	10.97867	3.42571	0.50868	8.42740	0.03229	0.00000	5.69045
**Mrpl54**	5.09473	0.02999	1.95230	3.78432	5.89429	0.14395	0.26918	0.00000	6.91734	4.96659	8.30391	0.16144	6.84299	0.22638	0.00000	0.59866
**Mrpl55**	8.23557	1.70970	9.34202	1.47192	6.32238	3.55557	3.21591	9.53044	4.10784	5.44991	6.57460	3.59708	1.71693	0.03650	2.11218	1.38687
**Mrpl57**	12.99718	0.11300	0.57191	3.76798	5.90301	1.37866	0.50599	1.65586	6.16675	11.73642	7.14137	0.56126	8.75133	0.00000	0.35969	1.74940
**Mrpl9**	4.17519	1.85590	2.16720	5.40849	4.45160	1.98504	4.20132	7.22926	5.54011	2.09113	6.07436	1.88960	2.59058	2.52483	4.13188	1.19963
**Mrps10**	13.48626	0.27104	0.00000	0.06799	0.79166	5.78402	1.51890	8.67709	0.66785	14.87552	2.81780	1.84449	11.60795	0.00000	0.00000	3.11648
**Mrps11**	11.09782	7.11011	8.44042	2.92756	7.05994	1.16469	6.41622	7.89577	1.54560	7.29982	2.10311	0.76597	8.69045	0.00000	0.00000	4.47681
**Mrps12**	14.42610	0.11705	1.40748	2.65207	9.30276	6.48304	3.42503	7.80278	5.32343	8.19062	4.47422	0.38467	9.60708	0.00000	0.00000	8.09791
**Mrps14**	14.08037	5.04707	7.94819	5.75083	4.14348	4.36022	5.15096	4.74055	7.59350	8.41541	9.62585	1.02993	8.06697	2.75903	3.33259	1.23623
**Mrps15**	2.31765	1.61830	1.54005	3.79510	4.16650	0.06649	0.73617	0.00000	4.52360	8.37406	11.44114	1.20248	10.88752	0.46131	0.00000	5.71983
**Mrps16**	7.64161	1.11492	6.36273	3.26512	4.36902	2.16646	3.18468	1.12314	2.46254	3.02140	10.26241	0.19320	8.19738	0.47973	0.30633	3.04829
**Mrps17**	11.82689	5.57947	9.10163	3.47164	1.52663	6.01093	4.33046	4.10846	4.81110	8.01554	6.73695	1.00790	4.32723	0.50345	5.38846	2.44671
**Mrps18a**	10.07745	2.82804	7.14987	0.36338	6.60160	6.17883	5.01291	10.25948	1.30616	13.57168	11.51374	4.89052	11.24923	0.40766	0.54386	5.66429
**Mrps18b**	20.26546	9.74093	27.61645	7.93230	13.37475	5.12153	13.73467	28.99402	9.83085	27.66474	23.58326	13.87423	26.26132	16.16626	15.57593	13.69952
**Mrps18c**	7.22893	0.60192	2.82797	0.96870	4.94609	2.32803	1.06629	0.00000	5.38341	5.60846	17.11568	0.41526	3.47162	3.59032	2.13233	1.59641
**Mrps2**	14.03332	3.54446	8.55193	4.64488	9.02530	5.51292	8.70289	6.68527	5.34403	12.75700	8.23414	0.35041	12.98815	3.69095	9.96426	5.67891
**Mrps21**	6.56598	0.24602	4.19766	2.93133	5.60225	2.79381	0.47369	0.83727	2.73767	3.46613	10.77904	0.58315	1.83029	1.77983	1.19057	1.10257
**Mrps22**	6.50928	3.30088	5.09100	2.39286	1.53320	0.28859	1.72264	1.94690	7.98058	15.90512	11.53714	2.79516	8.59679	4.26485	1.30150	8.22157
**Mrps23**	15.93782	9.35266	16.31046	5.67675	9.48621	9.84464	6.54446	12.63749	3.82104	15.66352	6.46206	2.07970	20.01806	0.12011	0.57674	10.60674
**Mrps24**	5.09794	0.46173	2.36445	8.12486	5.10005	0.73607	0.38516	0.12382	9.97298	13.42838	19.16583	0.29403	3.41619	0.39904	0.08115	0.51388
**Mrps25**	7.85714	2.10144	6.68406	2.35269	2.48803	0.38943	3.74168	3.47179	3.59614	3.04461	4.29390	0.27775	3.60495	1.38738	2.30090	2.72920
**Mrps26**	7.51076	0.00000	0.00000	12.52044	4.95437	3.16760	0.44113	4.33526	10.77490	15.02898	10.50265	0.93725	6.29866	0.79849	0.22221	3.37982
**Mrps27**	6.58306	8.88613	7.26217	0.70701	4.25201	4.58037	0.94778	8.28293	0.92927	5.29913	6.30859	0.29562	7.68967	0.05058	3.63509	0.04776
**Mrps28**	5.53404	0.26238	0.00000	3.87734	2.57505	0.75702	1.33986	3.34425	4.65379	0.68758	4.86240	0.32316	1.42349	0.06294	0.00000	0.65177
**Mrps30**	10.10415	3.27749	2.78587	8.31642	1.01317	0.00000	2.11724	1.79559	11.90534	11.43913	14.78054	2.13537	0.95563	1.62262	2.47553	2.78312
**Mrps31**	9.42475	4.80688	5.78813	0.00000	4.83719	1.62320	3.58675	6.86916	0.00013	4.15070	3.73219	3.39973	5.46289	6.54448	8.43344	4.68958
**Mrps33**	8.06181	2.37858	6.01817	3.06087	10.44729	0.84618	1.68450	4.63786	2.45373	5.47958	6.02023	0.74567	4.13384	5.66918	2.95874	2.82169
**Mrps34**	1.33177	0.28890	0.74345	0.98115	4.96855	0.78717	0.27070	0.76348	3.45247	3.57568	6.14242	0.21057	3.52477	0.00000	0.34652	2.69718
**Mrps35**	10.69622	1.97684	3.97865	1.17563	1.91356	0.10640	0.00402	3.19189	0.39672	6.07271	3.48180	4.47265	3.98948	0.17999	0.73303	3.67783
**Mrps36**	8.80718	2.69646	4.23474	4.43929	5.81396	3.22964	2.16680	1.87652	2.95332	3.72837	5.61210	0.39172	5.25427	0.49083	0.82608	6.14446
**Mrps5**	12.01843	6.24429	7.99154	0.87069	1.10037	0.65402	3.36196	10.35906	0.00000	3.19987	3.52533	0.31240	2.62537	0.01622	1.82449	6.68730
**Mrps6**	5.69596	0.02010	1.98479	9.29536	1.84692	1.59651	2.59618	4.70871	17.33657	10.06007	7.77755	0.31920	9.65522	2.60183	0.62537	2.47978
**Mrps7**	16.60912	6.60966	10.19758	17.30484	10.12873	7.14056	6.43258	14.35783	9.36665	25.20713	12.92084	2.91548	16.21375	1.64435	7.72009	8.20701
**Mrps9**	9.81312	5.47881	8.59634	1.49808	7.68387	8.61490	3.23579	9.95273	1.52664	10.71459	5.29421	2.87717	9.30386	0.00000	2.65716	8.71145

**Mrrf**	6.06954	1.01537	12.22168	1.09642	0.94973	0.84948	2.76485	5.38205	2.16253	5.85825	3.64491	5.82585	5.21812	0.48023	3.99163	0.00691
**Mrs2**	0.00000	0.12562	2.52984	1.10960	0.51600	3.46604	0.00411	1.43473	0.37943	2.91481	4.88333	0.20264	2.49438	0.00000	1.12569	0.01013
**Mrto4**	22.04693	9.07480	22.30651	14.25045	15.67714	4.12826	6.06056	28.08280	15.64840	25.78588	24.49052	3.88926	23.38793	6.79592	11.22966	9.89307
**Ms4a1**	0.01377	0.00761	0.01152	0.08112	0.00941	0.00650	0.00738	0.01143	0.00742	0.00740	0.01041	0.00638	0.01296	0.01341	0.60828	0.00743
**Ms4a6c**	0.01284	0.00848	0.03496	0.08331	0.00963	0.00629	0.03010	0.02183	0.11105	0.24610	0.05218	0.01789	0.02068	0.17772	0.04569	0.00733
**Msantd1**	0.01445	0.00778	0.02023	0.00514	0.00931	0.00611	0.00727	0.01071	0.71225	0.40849	0.00748	0.01469	0.01197	0.01248	0.00541	0.00742
**Msantd2**	4.76220	6.74868	2.56363	5.99853	0.00520	0.67366	5.82279	5.64974	3.29150	9.06884	4.10209	2.00936	0.45331	16.32292	15.15079	0.00190
**Msantd3**	3.76955	0.00235	0.00344	0.46759	2.29419	0.00473	0.01424	0.07190	0.36754	0.01346	0.00870	0.00203	0.00790	0.00991	0.06571	0.01753
**Msantd4**	3.02525	0.00242	0.85022	2.51717	0.00544	0.55976	0.01313	1.70108	0.00000	0.00000	1.95119	7.73484	0.94233	0.56731	3.06102	0.32368
**Msc**	4.01562	1.55131	0.76734	0.00000	0.00000	2.86054	2.08844	0.66615	0.02023	0.33215	0.65218	0.00725	0.06959	0.01008	0.00000	1.46930
**Msh2**	12.71971	7.17736	8.78575	1.65157	16.87528	2.36026	6.60623	11.35409	4.42710	6.72553	6.88724	0.93389	6.25950	7.29305	4.20980	10.90893
**Msh3**	0.96552	0.72984	1.23649	3.66768	4.10784	3.37236	2.58396	0.77153	2.47178	3.77391	1.13672	6.48023	0.24297	2.44770	7.40448	0.00268
**Msh4**	1.58360	7.21114	1.00179	1.39579	0.00142	3.26357	2.38618	7.33098	0.55370	1.13145	1.63816	0.06746	0.74243	3.33447	1.68973	4.08655
**Msh5**	0.00139	0.36219	0.09722	2.05241	0.00808	0.00558	0.81871	0.03869	1.03978	1.85085	0.00000	0.05373	0.01056	0.00382	0.00000	0.15629
**Msh6**	17.18532	16.27996	11.24407	2.74283	13.65445	7.28659	6.06703	12.07326	8.15832	11.46158	7.49490	5.76287	7.07257	25.50046	18.24370	5.91542
**Msi2**	0.01343	0.01878	0.00993	0.35092	0.00897	0.00693	0.00744	0.00650	0.29980	0.35584	0.20826	0.01900	0.01045	0.01118	0.00469	0.00726
**Msl1**	2.92410	0.00868	5.68320	4.36220	0.20813	1.92499	3.47066	2.51626	2.49384	8.79881	2.49251	9.56264	5.42385	6.23637	6.54745	2.02027
**Msl2**	2.03385	1.36136	0.51961	9.77997	2.66731	2.55598	4.09753	1.61180	10.02200	1.49094	0.97936	0.11664	1.65020	2.66895	10.21340	0.01447
**Msl3**	1.91212	0.05906	6.66351	10.21215	0.00707	0.13558	1.67492	7.39268	2.19157	6.26172	3.11515	2.74586	2.90036	0.18335	5.76766	0.79538
**Msln**	0.01364	0.00797	0.01275	0.01839	0.00946	0.00683	0.00749	0.01279	0.01111	0.17532	0.01361	0.00706	0.01874	0.01124	0.01052	0.00793
**Mslnl**	0.00753	0.00752	0.06267	2.27712	0.00854	0.00823	0.00656	0.00770	0.77512	2.02122	0.00425	0.02621	0.94016	0.00069	0.69879	0.00672
**Msmo1**	0.00000	3.93986	6.03140	0.32147	0.01298	0.10727	3.25348	11.65616	0.31635	3.10966	8.79511	10.74985	3.98630	18.22597	3.99782	0.02820
**Msn**	1.96145	0.00645	0.00000	0.12802	0.00375	10.66230	0.00431	0.38910	0.95967	1.20165	0.40403	0.15851	2.51546	1.00749	0.31935	1.70523
**Msra**	1.44723	0.00727	0.00000	0.42287	0.01447	0.31113	0.00607	0.00651	0.72016	0.00141	0.00734	0.00583	0.03707	0.00871	0.18218	4.16975
**Msrb1**	0.28344	0.31722	0.37028	6.61561	0.01543	0.02573	0.04900	0.19762	18.62135	0.09035	2.91711	0.06854	0.02064	0.06334	0.06942	0.03983
**Msrb2**	2.15395	0.00242	0.88958	0.00000	3.47813	0.04683	0.01515	0.00000	0.04131	0.00000	0.00000	3.23887	0.03318	0.00610	0.38005	1.84128
**Msrb3**	0.38402	0.00841	0.00745	0.50352	0.01840	0.00683	0.00700	0.00000	0.82161	0.13372	0.42343	0.06239	0.20719	0.00520	0.61508	0.00706
**Mss51**	0.01587	0.00791	0.12447	0.21467	0.00895	0.00574	0.00733	0.01224	0.00801	0.01305	0.01346	0.00792	0.08244	0.01178	0.00495	0.00720
**Mst1r**	0.05409	0.00800	0.00888	0.00643	0.00799	0.00686	0.00747	0.01088	0.13471	0.00812	0.19844	0.00711	0.00996	0.01154	0.00594	0.00717
**Msto1**	3.89814	0.01038	1.97558	1.01876	2.23950	2.84221	3.85545	0.39689	1.56613	3.49349	0.62885	0.08739	4.58221	0.02387	1.94332	4.53754
**Msx2**	2.44183	0.00352	0.38769	0.02130	4.26801	0.01608	0.02901	0.12070	0.02130	0.00953	0.03694	0.00261	0.00649	0.07116	0.00103	0.09106
**Mt1**	4.86703	0.74453	5.52482	8.58908	0.19299	10.99499	3.98084	1.54221	20.40807	28.84238	35.71204	4.47520	15.26843	0.30150	0.00000	6.88796
**Mt2**	3.03402	0.62749	6.85350	20.04316	1.32518	9.92383	4.24140	0.00000	37.76954	19.31845	52.89436	5.76360	13.92113	1.02781	9.13605	6.28284
**Mt4**	0.08519	0.00809	0.00612	3.18410	0.00766	0.00690	0.00729	0.00466	0.00000	0.00400	0.96085	0.00397	0.00431	0.00027	0.03965	0.00630
**Mta1**	2.10202	0.09036	0.00000	0.46606	0.04160	0.49857	0.00548	0.60487	0.04146	0.67052	0.00488	0.32657	0.16703	0.00888	0.00555	1.25844
**Mta2**	0.14787	4.07870	2.50195	15.62665	0.61697	0.03173	2.64073	3.62422	18.20934	7.85149	6.86506	0.75833	7.87869	4.41413	11.28528	5.00892
**Mta3**	15.37943	9.49058	18.96157	4.33228	14.39536	15.84330	11.17980	37.98635	2.88910	38.49686	20.37813	13.99263	24.94258	0.23631	0.00000	10.24967
**Mtap**	8.94628	2.74893	0.00000	0.50186	12.65359	2.10427	0.07646	0.78854	0.00000	0.57219	0.12859	1.30438	3.25711	0.00307	0.58506	4.39288
**Mtap7d3**	0.01335	0.00814	0.01086	0.80901	0.00914	0.00664	0.00745	0.01189	0.00000	0.42443	0.00949	0.01927	0.01254	0.00449	0.97321	0.00726
**Mtbp**	0.99009	3.80326	0.22772	0.49248	0.04797	1.98538	1.25580	0.62857	0.02233	0.11241	1.30676	0.95974	0.00000	0.13815	3.30512	0.62030
**Mtch1**	9.71067	0.27189	7.13283	1.30647	2.24598	4.25009	3.80066	3.11801	0.96773	4.41236	5.02296	0.36231	0.00000	0.08278	0.61743	3.23321
**Mtch2**	27.78808	17.43280	28.43960	6.61377	17.18957	13.48268	14.21167	24.65559	8.48549	23.43653	22.69680	3.84610	26.57359	6.14383	6.31402	13.59441
**Mtcl1**	4.19510	0.22320	0.95486	0.66784	3.78616	4.25765	4.31288	0.01795	0.21959	0.19701	0.01515	0.00899	0.07548	1.05193	0.93686	2.92351
**Mtcp1**	0.01570	0.00790	0.02257	0.01804	0.00905	0.00690	0.00736	0.03978	0.01252	0.01463	0.01321	0.00785	0.01092	0.01221	0.00974	0.00738
**Mtdh**	6.24582	9.56900	5.96069	9.81280	2.36450	2.96899	8.26094	5.40369	7.52540	4.46377	1.56056	2.54361	1.49472	7.56206	10.51437	2.53492
**Mterf1a**	0.06372	0.01824	0.00483	0.00977	0.00851	0.00711	0.00720	0.01076	0.01202	0.00460	0.00918	0.00507	0.01331	0.64809	0.15651	0.00735
**Mterf1b**	0.04753	0.01832	0.00678	0.00931	0.00894	0.00708	0.00731	0.01147	0.02352	0.00570	0.01025	0.03407	0.01658	0.45345	0.09513	0.00745
**Mterf2**	1.63421	0.03773	0.00000	0.00000	0.00493	3.40150	0.03631	0.00000	0.53694	0.06236	0.73346	0.00093	1.09037	0.00434	0.41247	0.72730
**Mterf3**	1.23149	5.30595	0.49736	1.24519	2.81763	1.68465	1.48024	0.99077	0.17117	0.06589	0.18245	0.18681	1.22830	3.16653	2.16853	0.73781
**Mterf4**	7.28188	3.67116	1.71980	1.64372	0.15568	0.00000	0.00000	2.21266	0.73338	6.71574	3.34062	0.59409	3.62129	5.38152	3.23587	4.95518
**Mtf1**	3.70759	3.22881	0.00000	9.91124	0.07320	4.72923	2.50962	6.67712	10.70886	9.58506	6.83807	1.68450	2.74016	4.84880	10.96161	0.02521
**Mtf2**	9.83285	13.23418	19.96184	11.88031	8.44002	8.99137	6.98899	22.37290	21.12488	20.72781	22.52836	26.31273	10.27105	40.64113	20.02072	4.07642
**Mtfmt**	2.33084	0.01575	0.32985	1.22970	1.50268	1.02236	0.48152	1.44997	1.33431	1.15474	1.13984	0.05831	0.41682	0.43451	0.70703	0.92794
**Mtfp1**	4.18874	0.13520	0.00000	0.19544	0.01184	4.41504	2.24338	0.00667	0.00818	0.00000	0.00797	0.05938	3.36795	0.01244	0.01029	0.87437
**Mtfr1**	4.38619	8.80377	13.18624	5.45336	14.45574	5.46407	7.19009	6.18415	4.67378	3.06360	2.61331	7.19618	7.99624	2.98574	4.20727	3.54201
**Mtfr1l**	12.78702	6.19255	9.99322	8.47331	5.98388	0.26783	8.56903	4.12669	1.84147	6.78153	0.88769	0.94401	5.74408	0.00001	2.80302	1.52144
**Mtfr2**	0.01350	0.00808	0.01272	0.26061	0.00989	0.00697	0.00765	0.01386	0.03867	0.00777	0.02538	0.00732	0.03456	0.06354	0.08404	0.00760
**Mtg1**	12.41069	2.43172	8.99537	2.03746	3.64672	4.18205	6.48456	5.93596	0.24098	5.06923	3.98902	0.20268	8.97761	0.00000	1.15291	2.26508
**Mtg2**	6.18504	0.00000	0.01957	0.02916	0.11395	0.00202	0.27682	3.78085	0.04471	1.48784	0.09324	0.02915	4.07888	0.00742	0.02423	1.58129
**Mthfd1**	20.97461	15.29921	17.22485	2.80900	14.06892	9.21606	14.10821	22.30037	4.48194	20.05489	15.44660	8.07253	16.98488	2.23732	2.89940	10.66845
**Mthfd1l**	5.75160	0.25402	2.15894	0.08684	0.20390	0.01373	0.47568	0.10749	0.11478	2.08076	0.02308	0.03053	1.80762	0.68749	1.54976	0.00799
**Mthfd2**	1.20783	2.90318	1.98973	11.03164	2.45059	0.01251	2.59208	10.39293	1.56399	3.11077	0.40716	4.26305	6.12912	0.25733	2.46089	6.54812

**Mthfr**	3.97707	2.77476	0.58783	8.57843	4.51316	3.83887	0.05885	10.31507	10.37472	14.97159	5.84015	4.69901	3.03753	1.27766	8.75097	2.03540
**Mthfs**	1.43287	1.01478	0.47304	1.96700	3.02109	0.21765	0.16731	1.06946	0.69575	0.15911	1.24607	0.22757	0.51590	0.12363	0.00000	0.09344
**Mthfsd**	0.26617	0.28454	5.42364	0.00709	0.00000	0.63581	3.71862	5.16812	0.00720	2.49818	3.52941	0.19780	5.01596	0.02962	0.00000	0.02351
**Mthfsl**	1.05151	0.85759	0.48219	1.78427	3.20773	0.32928	0.20768	1.18724	0.52129	0.28591	1.18047	0.16210	0.25651	0.32998	0.00000	0.11502
**Mtif2**	2.58160	1.40328	3.59733	1.85168	4.09422	2.86043	0.76632	0.00000	2.98436	2.80235	3.82249	0.37878	2.88670	6.74921	2.62045	0.14577
**Mtif3**	0.51473	0.11221	0.21013	3.06893	0.00644	2.29550	0.01463	4.07300	2.85917	4.85424	1.66609	0.09033	4.34354	0.00000	0.00000	1.05190
**Mtl5**	0.01786	0.00790	0.01185	0.01025	0.00894	0.00669	0.00744	0.01319	0.01216	0.05815	0.01317	0.00748	0.01303	0.01028	0.04626	0.00770
**Mtm1**	0.18697	2.99444	0.44374	0.03724	0.61229	0.00894	0.79675	0.54931	0.00685	0.95724	1.81925	0.02318	0.00000	2.13701	0.97356	0.08023
**Mtmr1**	0.75732	0.00198	0.41290	1.26145	1.81326	0.00582	0.00476	0.01397	1.39224	1.75573	0.08580	13.23972	1.09070	3.15947	0.38194	0.00451
**Mtmr10**	0.00000	2.03347	0.37566	1.58131	4.26723	5.15851	1.84905	0.86976	0.52765	3.30938	3.26504	0.20307	1.22307	0.08148	3.48291	2.01958
**Mtmr11**	0.03977	0.00816	1.17242	0.81792	0.01173	0.00607	0.00744	0.01285	0.09512	0.01859	0.03340	0.00667	0.13405	0.03472	0.00000	0.00711
**Mtmr12**	4.78430	0.16998	3.84816	2.18352	0.00000	3.70508	2.88534	2.31586	3.64891	3.08983	1.78142	9.32864	0.99412	2.83025	6.22174	2.08816
**Mtmr14**	1.16116	3.29461	3.47139	16.94773	0.06817	1.82255	0.03269	6.94719	8.62108	7.60660	5.47012	0.58885	1.58729	0.02267	0.65647	0.33629
**Mtmr2**	2.79299	0.04932	0.10043	6.62909	2.17533	0.52243	0.03718	0.09221	1.42094	0.60064	2.56518	0.04614	0.93114	0.20265	5.02944	1.08944
**Mtmr3**	2.80437	1.00287	7.23991	9.33475	2.24037	0.00924	1.87399	5.95261	6.93148	7.27930	3.07811	1.88495	4.79500	5.21483	6.17280	0.28993
**Mtmr4**	3.08591	6.36574	6.97447	4.19580	4.50368	0.16990	6.37637	3.47898	2.87871	1.57470	1.39370	11.48048	5.01259	0.40637	5.39812	4.74328
**Mtmr6**	0.82241	1.94055	0.00000	2.07542	0.03475	2.46835	0.00545	0.01204	1.86570	3.24508	0.00000	0.14300	1.02372	8.93618	7.57771	1.40773
**Mtmr7**	5.74812	0.01103	3.50199	0.25525	3.14789	0.00000	0.00000	0.79423	0.01875	0.00000	1.05805	0.10008	2.31574	0.03500	2.48716	5.37622
**Mtmr9**	1.56572	0.03531	2.48360	1.74266	0.00000	0.84583	0.00533	2.21293	1.21998	0.09888	0.57868	0.07780	0.18951	0.00686	0.51953	0.01200
**Mto1**	4.05181	0.23533	0.77528	0.80296	1.43117	0.00000	0.11246	0.15735	1.49026	2.09812	1.93589	8.58091	2.24348	0.92960	1.32973	0.05882
**Mtor**	2.27282	4.35628	5.20707	6.09123	0.02546	3.35577	0.00000	4.52316	6.48270	4.60473	3.49223	4.78366	3.09047	4.97731	6.09252	2.66892
**Mtpap**	5.48977	2.96457	5.49898	3.63971	7.37198	0.01618	7.98003	5.61576	0.00000	9.73606	2.74755	0.53524	2.11964	2.91946	3.72152	1.58969
**Mtpn**	13.06612	9.02278	8.98511	11.84073	13.78505	6.46337	7.81685	7.47091	11.54580	5.30734	6.26097	11.11078	7.40032	14.52456	6.66586	3.34369
**Mtr**	2.67522	5.46805	1.61847	0.05223	0.01069	3.47718	0.83699	3.44880	2.85293	3.75934	0.37735	0.08240	0.57958	0.05000	1.23279	1.39593
**Mtrf1**	0.01526	0.49370	0.40511	1.36931	0.31264	0.02148	0.01814	0.02305	3.61640	0.02327	0.00000	0.00000	0.42609	0.00916	0.18238	0.00664
**Mtrf1l**	1.69832	0.66238	1.48397	1.01707	1.90039	0.00439	3.75984	0.61677	1.59561	0.90737	2.29119	0.09240	0.48945	0.04610	1.74599	0.01153
**Mtrr**	3.15612	6.32324	4.57832	3.23121	4.47831	3.00133	5.50039	7.76922	3.35965	5.44070	7.25918	4.89113	3.53014	4.83464	6.95895	3.96854
**Mtss1**	0.00000	0.00687	0.00000	2.31210	0.00696	0.45221	0.00610	4.30909	0.87284	1.69616	1.95822	0.15084	0.68274	0.20383	0.88030	1.86594
**Mtss1l**	0.01655	0.00810	0.00806	0.57219	0.02427	0.00654	0.00709	0.15365	0.08612	0.00407	0.01214	0.00737	0.00000	0.01287	0.00266	1.89887
**Mttp**	0.00371	0.00878	0.00000	1.19384	0.01223	0.00625	0.00779	0.61950	0.56606	1.88647	0.66343	0.04113	0.00290	3.31242	1.59636	0.00716
**Mturn**	0.00451	0.00762	0.31209	0.01085	0.00873	0.00916	0.00611	0.95774	0.01122	0.00000	0.00780	0.02511	2.04400	0.01541	0.00861	0.01350
**Mtus1**	0.00152	3.13461	4.17032	24.64892	0.00754	7.23159	3.12029	10.22884	16.54471	18.53823	13.39464	10.35484	4.36973	18.95347	24.87497	0.34883
**Mtus2**	2.22815	0.01676	0.00000	3.60085	0.00467	0.00394	0.00514	0.00000	8.08429	0.32993	1.60116	0.19751	0.20708	0.03186	1.83251	0.00349
**Mtx1**	18.94206	0.92883	2.29371	0.12219	7.51973	5.76049	6.37916	11.37735	0.01587	10.69398	1.23753	0.52417	14.12631	0.01213	0.00776	5.68312
**Mtx2**	3.19638	2.57691	4.81519	2.90835	8.94927	2.14951	4.68716	0.00000	1.64761	4.45332	3.31945	9.91781	1.12069	3.67029	0.00000	0.22718
**Mtx3**	0.00000	2.26247	0.54721	0.52363	0.00750	0.00500	0.00574	0.98614	0.77212	2.24872	0.15772	6.00171	0.01877	1.25734	3.50435	0.00594
**Muc1**	0.90127	0.02471	2.42351	0.31584	0.01018	0.00549	2.92948	1.77578	0.00413	0.68004	0.00000	0.00746	0.00000	0.00883	0.00000	1.34323
**Muc13**	1.06882	0.00626	0.00321	1.32937	1.12306	0.00700	0.00648	0.01675	1.83789	0.00568	0.00000	0.01430	0.02210	0.00965	0.02108	0.00584
**Muc4**	0.01783	0.00795	0.01205	2.20097	0.00903	0.00642	0.00741	0.01371	0.00000	0.01084	0.01258	0.00775	0.01264	0.00424	0.11141	0.00699
**Mul1**	6.55285	3.18187	5.18519	5.74091	1.09050	0.56672	2.47846	7.38742	3.29363	7.70144	1.60905	0.10435	6.05370	0.02697	0.62931	1.44127
**Mum1**	6.09315	5.00684	11.87570	2.02863	2.26063	0.38569	0.73219	14.49062	3.03973	7.75572	5.23093	0.32563	16.39956	0.86934	0.05458	9.49296
**Mum1l1**	0.01333	0.00785	0.01148	1.86294	0.00933	0.00617	0.00721	0.01044	0.37851	0.45536	0.00781	0.00408	0.01200	0.00187	0.86490	0.00713
**Murc**	0.01033	0.00646	0.00766	0.00983	0.00964	0.00670	2.36804	0.00944	0.01225	0.01028	0.01334	0.00603	0.01014	0.01249	0.00935	0.00729
**Mus81**	0.50629	0.00451	1.16966	1.78456	4.45739	0.64743	0.30147	2.00605	1.27657	1.71034	0.15936	0.08642	0.35929	0.00613	0.66757	1.93520
**Musk**	0.01375	0.00794	0.01245	1.58294	0.00935	0.00647	0.00746	0.01366	0.00000	0.01193	0.01272	0.00766	0.01263	0.00479	0.08076	0.00713
**Mustn1**	0.03226	0.00812	0.01050	0.00818	0.01256	0.00690	0.00743	0.01110	0.01148	0.69731	0.01087	0.00679	0.01205	0.01421	0.08898	0.00757
**Mut**	2.60400	3.16809	0.72658	0.14202	0.00000	0.09444	1.19518	1.08745	0.00486	0.25004	1.01173	0.03269	0.02626	2.92465	0.57193	2.64030
**Mutyh**	2.44122	0.27126	3.89087	0.45732	9.98397	1.38145	0.54843	7.14091	1.36892	1.42427	7.01342	0.12078	2.18852	0.28544	0.00000	2.79492
**Mvb12a**	2.86630	0.01467	1.09172	3.82018	0.00000	0.03550	0.00000	3.16523	2.63837	6.61020	2.64882	0.04115	4.62678	0.00931	0.00000	4.58881
**Mvb12b**	0.00000	0.00804	0.00000	6.76672	0.00811	0.00736	0.00763	0.74942	4.21851	0.94114	1.26679	0.00000	0.00000	0.02369	1.03807	1.32114
**Mvd**	2.19722	0.00000	4.82061	0.38447	0.05770	0.08694	6.85768	5.89303	0.00774	3.81675	1.16127	0.08993	2.97124	0.00151	0.00006	0.30431
**Mvk**	6.70899	0.00000	0.32059	3.76582	0.00497	6.26145	3.84109	2.46703	2.01155	0.00000	0.00000	0.03064	2.86570	0.01059	0.00000	0.00959
**Mvp**	5.91857	3.74221	11.25726	4.12731	0.00000	3.09070	1.90469	4.27750	5.59469	5.66889	4.94639	0.27208	1.82490	0.00000	0.00000	3.31838
**Mxd1**	0.01258	0.00728	0.00000	3.52230	0.00927	2.14431	0.00679	1.53192	0.93090	0.16063	0.32656	0.06624	0.00000	0.00014	0.82643	0.00601
**Mxd3**	3.63301	0.16486	2.44085	0.16283	0.00000	4.84170	0.00189	4.53708	0.16560	2.96974	1.30626	0.13160	2.32905	0.03121	0.05206	1.92762
**Mxd4**	0.01332	0.00832	0.12023	0.67033	0.00897	0.00563	0.00767	0.00899	1.15181	0.00000	0.30083	0.00513	0.00849	0.00684	0.00341	0.00702
**Mxi1**	2.58170	0.64984	1.67129	1.58771	0.00343	0.00422	0.00416	1.15316	1.35108	3.37117	0.04410	3.20187	1.19874	0.00186	0.64231	0.05479
**Mxra7**	0.01214	0.00774	0.13553	0.12004	0.00875	0.00675	0.00672	0.00000	0.41429	0.31511	0.86495	0.01298	0.45927	0.12511	0.22213	0.01393
**Mxra8**	0.00879	0.01719	0.00864	1.77399	0.00892	0.00625	0.44816	0.00769	1.42460	0.77116	0.00000	0.00963	1.23941	0.00118	0.40474	0.00651
**Myadm**	3.04127	0.08979	1.61579	1.57901	1.47152	1.42555	1.59851	3.33074	4.08426	1.40874	5.22853	1.35423	0.30682	9.09112	5.28810	0.13360
**Myadml2**	0.01357	0.00821	0.01207	3.10656	0.00937	0.00623	0.00736	0.01321	1.12450	0.04930	0.00684	0.00584	0.01295	0.00841	0.00312	0.00689
**Mybbp1a**	19.36321	12.72545	12.68023	17.31758	18.57823	5.30399	12.06176	31.99685	25.27929	31.39799	30.05568	11.54885	16.32395	15.09300	14.81140	9.76591

**Mybl1**	0.01294	0.00751	0.00709	6.37231	0.00916	0.00575	0.04681	0.00588	2.55486	0.00000	0.08064	0.00885	0.04383	0.26403	1.00884	0.00592
**Mybl2**	23.10452	9.52798	15.44625	12.88270	13.08368	9.28969	12.00874	20.29320	12.07496	14.74670	10.46093	1.25629	15.59612	1.61913	6.96629	13.60899
**Mybpc1**	0.93777	0.82899	0.00000	0.36827	2.55041	0.00669	0.08684	0.07184	0.20364	0.00000	0.00801	0.04220	0.01720	0.47483	0.10205	1.03418
**Mybpc2**	0.00000	0.02410	4.57572	0.03508	4.21374	0.25923	0.97181	11.39085	0.43512	3.53327	4.96584	0.25897	4.91960	0.27682	1.48800	0.00241
**Myc**	9.30214	2.88126	0.00000	1.41751	3.34840	2.28013	0.61374	9.02062	0.29328	2.96681	1.45584	0.68906	0.06357	1.74126	3.13436	3.62809
**Mycbp**	5.52172	5.39690	10.96771	4.83742	10.01668	8.27562	7.59379	7.66534	4.87975	9.72591	8.08134	2.88284	5.58694	5.70773	4.86372	6.76515
**Mycbp2**	5.34976	7.34792	5.02255	7.18454	2.08723	0.00000	5.18855	3.48879	3.71052	5.21140	0.46051	0.37072	5.71401	6.26882	11.29760	1.10781
**Mycbpap**	0.00348	0.00722	0.07524	0.00178	0.09860	1.98731	0.44238	0.15131	0.01134	0.00075	0.45203	0.01314	0.45427	0.04273	0.00000	0.01417
**Mycl**	0.95513	0.25265	2.53633	0.00320	0.00208	0.00489	1.19048	0.00465	0.02414	0.23092	0.38649	0.05493	0.02380	0.42419	0.00000	0.01342
**Mycn**	3.53795	0.00344	0.00000	0.00970	1.23714	0.11620	0.00501	0.00954	0.01176	0.01014	0.01362	0.00449	0.05713	0.01276	0.01102	0.15176
**Mycs**	0.01366	0.00795	0.01188	0.40574	0.00928	0.00674	0.00758	0.01403	0.45772	0.03235	0.00867	0.00758	0.01321	0.01023	0.07973	0.00733
**Myd88**	1.89914	0.09295	0.88509	3.06559	0.05165	0.00660	0.01631	0.02572	0.04466	2.55810	0.06632	0.15904	1.05025	0.00054	0.49050	0.01957
**Mydgf**	14.26521	1.50413	4.51618	0.56271	6.12038	0.11395	1.70238	5.09341	0.33039	5.39939	2.65209	0.08705	7.65898	0.41142	0.40070	7.84012
**Myef2**	2.49231	5.03389	3.45534	1.62616	4.09625	2.25238	1.74419	1.04713	2.37388	1.57166	7.80556	4.75437	2.11695	4.03294	4.72521	1.49319
**Myeov2**	2.56061	0.07693	1.41069	16.73243	0.18016	0.66899	0.70706	0.00000	7.52860	0.12657	8.28580	0.08142	0.48434	1.07556	1.93039	0.41649
**Myg1**	3.44610	2.63799	6.28677	6.60163	2.43337	1.98982	1.85686	7.57427	6.29290	8.55155	3.44516	0.92807	2.47243	0.00000	2.02442	3.43838
**Myh10**	4.20076	6.94367	5.83486	2.32675	2.62025	13.90360	7.80639	16.52732	1.94142	8.45406	7.27064	2.69080	10.91144	6.97761	2.22716	1.31320
**Myh11**	0.01043	0.00760	0.00853	2.77773	0.00979	0.00650	0.00729	0.03251	1.37960	1.23336	0.01367	0.02495	0.02977	0.62674	0.92707	0.00720
**Myh13**	0.53187	0.01627	0.00000	0.01023	0.00586	0.00726	1.59709	0.18396	0.01205	0.00000	0.01239	0.00585	0.00000	0.01594	0.00581	2.49776
**Myh14**	0.98631	0.01990	0.00000	0.33465	0.01024	0.01635	0.00686	0.16499	0.00662	0.95960	0.79480	0.01388	0.39540	0.00622	0.00000	0.00611
**Myh15**	0.01334	0.00795	0.01200	0.01062	0.00935	0.00665	0.00744	0.02350	0.01234	0.01503	0.01308	0.00795	0.01204	0.01224	0.01045	0.00741
**Myh3**	0.01336	0.00792	0.00922	0.28925	0.00903	0.00676	0.05873	0.00731	0.27507	0.00456	0.17687	0.06781	0.01001	0.00949	0.00000	0.05770
**Myh6**	0.01083	0.00862	0.01025	0.04897	0.00984	0.00720	0.00829	0.23460	0.01050	0.50245	0.01324	0.01920	0.02193	0.01695	0.00698	0.00761
**Myh7**	0.00855	0.00870	0.01042	0.25272	0.01017	0.00734	0.00830	0.27851	0.91368	0.85261	0.04981	0.02804	0.14851	0.01408	0.00784	0.00767
**Myh7b**	1.69805	0.19340	0.64920	0.00606	0.00862	0.93270	0.00507	0.00000	0.01197	0.00000	0.14247	0.00518	1.60174	0.01370	0.00000	0.00418
**Myh9**	0.46611	0.24869	0.91193	0.89259	0.00692	4.20361	0.05706	1.35439	1.63067	0.00000	0.37327	0.44178	1.13217	0.19232	0.05259	0.07764
**Myl12a**	20.41898	13.60890	21.06066	4.84719	11.64834	16.73761	16.07254	18.44997	2.86240	18.08896	13.53693	8.75881	20.21890	3.43543	7.07585	12.96060
**Myl12b**	10.89856	10.76627	15.26511	2.25859	6.63790	13.73899	10.95920	13.92346	0.99719	9.38059	7.22374	2.51625	20.04641	2.32425	4.44385	10.47968
**Myl2**	0.01372	0.00785	0.01244	0.31460	0.00935	0.00651	0.00746	0.01280	0.00622	0.07672	0.01237	0.00704	0.01235	0.00551	0.14071	0.00729
**Myl4**	0.01254	0.00781	1.65068	0.07488	0.00636	0.00865	0.00730	0.00590	0.01122	0.00921	0.42745	0.00498	0.00403	0.00954	0.00000	0.00678
**Myl6**	23.76172	10.52550	20.02605	9.80836	13.84168	14.02390	6.84504	17.31536	5.90356	23.28497	21.48608	2.24818	30.43045	9.67022	7.12536	18.66266
**Myl6b**	0.12128	0.02821	0.08122	0.31315	0.02885	0.07912	0.01722	0.05819	0.06302	0.10599	0.08947	0.00620	0.20923	0.17083	0.01681	0.09471
**Myl9**	0.02989	0.00886	0.73747	0.00556	0.01037	0.00884	0.09675	0.15586	0.01098	2.50618	0.00678	0.09378	0.00413	0.01387	0.00478	0.00716
**Mylip**	0.02491	0.01703	0.54408	1.24632	0.00717	0.00902	1.26232	0.01748	1.91392	0.57064	0.01286	0.00000	1.18385	0.00457	0.55109	0.00635
**Mylk**	1.85201	0.00558	0.00350	0.36044	0.00434	0.00651	0.00572	0.00458	0.41745	1.34168	0.00967	0.00168	0.01139	0.00762	0.02773	0.00557
**Mylk2**	0.01368	0.00785	0.01101	0.67391	0.00894	0.00655	0.00715	0.00000	0.61200	0.22440	0.96077	0.00153	0.00510	0.00391	0.00000	0.00741
**Mylk3**	0.01366	0.00783	0.01212	0.04020	0.00923	0.00660	0.00745	0.01271	0.62615	0.07216	0.00986	0.00594	0.01310	0.00695	0.05843	0.00771
**Mylk4**	0.01914	0.00758	0.03499	0.11308	0.00919	0.01205	0.00719	0.01503	0.10998	0.03179	0.03972	0.22829	0.09075	0.21509	0.02917	0.00737
**Mylpf**	6.18642	4.02674	12.43201	0.73133	2.90788	2.06985	3.55154	8.03237	1.47484	6.71460	20.05518	1.42857	11.32452	2.11290	1.84470	3.80379
**Mynn**	1.04936	4.20089	1.72639	4.38811	2.60939	1.10398	2.31994	1.49048	3.68237	4.10150	2.98708	0.80951	0.98508	3.43908	8.51949	0.46271
**Myo10**	0.19986	0.00694	0.77744	5.77879	0.01374	0.00515	1.07284	0.01442	2.00989	5.82254	0.09701	0.06161	0.15285	0.00000	4.72395	2.28712
**Myo15**	0.01006	0.00845	0.00910	0.99411	0.00954	0.00671	0.00812	0.17327	0.14169	0.02534	0.00873	0.01882	0.02860	0.15619	0.02372	0.00707
**Myo16**	0.01358	0.00827	0.01201	1.05967	0.00911	0.00641	0.00742	0.01114	0.46899	0.32984	0.02411	0.00652	0.01109	0.00978	0.00330	0.00693
**Myo18a**	0.67862	0.00601	0.06983	2.04216	0.01286	0.00776	0.31159	3.88551	2.33740	0.92955	1.61945	0.02271	0.87177	0.00000	0.07843	3.26466
**Myo19**	2.04093	1.72650	2.92467	3.70240	0.40234	0.12090	1.81100	2.57563	3.21112	0.41334	0.67442	0.02396	0.57009	0.00003	0.76404	0.01658
**Myo1a**	0.02514	0.00821	0.05817	0.82561	0.02224	0.01152	0.00735	0.01107	0.88349	0.00603	0.06793	0.02778	0.01740	0.01097	0.00000	0.00718
**Myo1b**	7.20577	10.27830	7.52230	7.31365	2.30618	0.13087	9.89179	15.01845	10.92405	19.04874	11.49753	10.62758	8.80733	16.07089	13.63946	5.16513
**Myo1c**	2.80893	0.39696	6.08615	9.34506	0.05965	7.80822	0.65920	11.77914	7.85950	6.95063	5.67872	5.02392	9.27383	1.41743	2.75882	1.42634
**Myo1d**	2.19889	0.00547	0.68101	2.32804	0.02595	0.01627	0.00577	2.52261	2.05315	1.15894	0.00000	0.02240	0.19061	0.00000	0.42515	0.01232
**Myo1e**	0.11357	0.00732	0.91125	0.93030	0.01316	0.02601	0.02682	0.00606	0.04585	0.05812	0.28963	0.07205	0.08322	0.27580	0.03870	4.57371
**Myo1g**	0.01076	0.00768	0.99201	0.50945	0.00773	0.00570	0.00694	0.46317	0.88982	0.43364	0.49515	0.01397	0.14899	0.00662	0.00000	0.00673
**Myo1h**	0.01314	0.00760	0.00758	0.09465	0.02189	0.00686	0.00710	0.55531	0.01989	0.00578	0.01049	0.00628	0.00568	0.00786	0.08946	0.00735
**Myo3b**	0.01367	0.00770	0.00754	1.16685	0.00914	0.00614	0.00712	0.00041	0.74203	0.00000	0.31804	0.00120	0.00876	0.00356	1.74868	0.00685
**Myo5a**	0.24339	0.01757	1.54449	5.63985	0.04600	0.00732	0.01576	0.93325	0.62518	0.69897	2.56281	0.09980	1.18124	1.09149	4.87535	0.00459
**Myo5b**	0.55112	0.35982	2.81776	6.18135	0.01195	1.15862	0.00595	1.56540	1.71617	0.33093	0.20805	0.01116	0.50147	0.62455	6.46446	0.30703
**Myo5c**	0.28366	0.00709	0.21637	0.01681	0.42354	0.00532	0.02684	0.01486	0.03489	0.00000	0.01041	0.10733	0.00710	0.03349	0.07956	1.26560
**Myo6**	2.57316	0.00421	0.16518	2.16728	0.01140	3.97584	0.24868	1.21045	3.59407	3.15997	2.55116	0.01294	0.00000	0.00000	0.83033	0.00219
**Myo7a**	0.02539	0.27520	2.69262	0.57719	0.00372	2.08011	0.00693	0.00924	0.00000	1.08390	0.00765	0.00353	0.00701	0.00767	0.00000	0.98091
**Myo7b**	0.00816	0.01796	0.02433	0.00815	0.00962	0.00683	2.48455	0.01090	0.01166	0.04969	0.01151	0.00739	0.05847	0.00680	0.19386	0.00724
**Myo9a**	2.86344	1.43577	0.96125	3.69791	0.00330	0.56577	0.46778	4.94324	2.55846	9.63439	6.09834	8.71419	1.92038	26.34059	8.62533	3.65727
**Myo9b**	0.98279	0.02454	0.00725	5.09433	0.00402	4.11822	2.49626	1.39130	2.48942	3.33982	0.00000	0.08664	2.18587	0.00000	0.84644	5.23342
**Myod1**	2.86533	0.00434	0.00310	0.00912	1.57382	0.00581	0.00537	0.00498	0.01143	0.01106	0.01207	0.00518	0.01012	0.00982	0.05382	0.00436

**Myof**	0.07681	0.00841	0.00546	0.24875	0.01324	3.32533	0.00709	0.01545	0.00510	0.65012	0.00720	0.04016	0.00000	0.01070	0.00502	0.16582
**Myom1**	0.01354	0.00756	0.00992	0.00651	0.00953	0.00649	0.00730	0.01077	0.01070	0.00224	0.00875	0.02336	0.01331	0.17078	0.85606	0.00747
**Myom2**	0.01009	0.00745	0.17536	2.17493	0.00913	0.00867	0.00694	0.00831	0.05964	0.00000	0.01452	0.01959	0.10590	1.23644	0.65595	0.00679
**Myom3**	0.01182	0.00810	0.01162	0.49058	0.00910	0.00645	0.00719	0.01141	0.26746	0.00572	0.01175	0.01854	0.41354	0.01209	0.00344	0.00695
**Myoz1**	0.00904	0.00759	0.00872	0.01117	0.01031	0.00720	0.00731	0.84581	0.01143	0.00482	0.00946	0.00588	0.01288	0.01620	0.00000	0.00764
**Myoz3**	0.06418	0.02759	0.07258	0.72708	0.02454	0.03125	0.01702	0.00000	1.10403	0.04664	0.65341	0.00999	0.03203	0.33542	0.32408	0.01409
**Mypn**	2.16896	0.01464	4.11595	0.72670	0.00000	3.37439	0.07402	3.80988	0.61717	2.90261	0.64417	0.56385	2.09089	1.64371	3.75096	0.04525
**Mypop**	0.01170	0.00813	0.20846	2.95743	0.01798	0.01724	0.00720	0.00000	0.66803	0.10150	1.08278	0.00196	0.13757	0.00215	0.34928	0.00647
**Myrf**	0.01103	0.00774	0.01097	0.07750	0.01862	0.00683	0.00740	0.05853	0.00808	0.40644	0.01051	0.00683	0.01030	0.00605	0.15841	0.00748
**Myrfl**	0.22517	0.00716	0.25353	0.00552	0.00705	0.00532	0.93478	0.01004	0.63583	0.05056	0.00455	0.06141	0.01649	0.57116	0.00000	0.00690
**Myrip**	0.01359	0.00778	0.01064	1.11019	0.00924	0.00664	0.00747	0.01211	0.40905	0.13183	0.00708	0.02882	0.01327	0.00686	0.73925	0.00720
**Mysm1**	0.54175	0.23483	0.90327	4.58676	0.81169	0.18425	2.38369	8.42589	4.26482	9.85505	7.32066	8.90101	0.92983	19.26860	17.46491	1.64967
**Myt1**	0.01377	0.00805	0.01216	0.96926	0.00935	0.00653	0.00746	0.01329	0.01924	0.01093	0.01255	0.00809	0.01269	0.00871	0.09937	0.00723
**Myt1l**	0.00769	2.08159	0.09187	0.09245	0.00784	0.00574	0.00609	0.51802	0.38508	0.00000	0.89056	0.00083	0.00000	0.05457	0.05033	0.00612
**Myzap**	0.01772	0.00778	0.01135	0.00440	0.00886	0.00651	0.00743	0.01132	0.32428	0.35676	0.00775	0.00580	0.01131	0.01355	0.23135	0.00709
**Mzb1**	0.01368	0.00815	0.01083	0.85101	0.00898	0.00654	0.00733	0.00558	0.00000	0.51342	0.19729	0.00622	0.00940	0.00802	0.00000	0.00696
**Mzf1**	0.11871	0.00715	0.00549	0.00787	0.01369	0.00708	1.27357	0.00165	0.17054	0.18610	0.23792	0.00433	0.00954	0.00743	0.02549	0.00769
**Mzt1**	5.76397	2.67771	5.79154	7.24032	3.82759	1.12719	1.67460	2.54052	0.92218	6.10139	2.48011	0.95583	2.60888	6.79959	3.19756	1.41609
**Mzt2**	4.61123	0.24356	0.35515	0.21976	1.08235	3.99712	1.04359	5.58034	0.40093	3.54642	3.28235	0.20556	0.50995	0.00193	0.04611	0.01493
**N4bp1**	6.62095	4.88050	0.69346	15.16560	10.68943	5.84026	6.97427	7.40814	3.29974	8.85035	3.52996	2.95284	4.83111	2.42257	6.54726	2.39429
**N4bp2**	1.27794	0.00581	0.00000	5.50506	0.00570	0.00396	0.04564	0.00737	3.04161	0.80006	0.00000	0.11108	0.01125	0.05378	5.55040	0.00492
**N4bp2l1**	0.18192	0.00729	0.00000	0.00998	0.00827	0.00781	0.00713	0.00842	0.01239	0.08986	0.00738	0.02169	0.01223	1.45620	0.01114	0.00699
**N4bp2l2**	3.39962	8.51215	7.43342	14.41145	2.25853	0.02124	4.46892	8.14006	17.07145	15.07428	9.77748	8.82099	6.77721	19.82670	22.88883	5.04968
**N4bp3**	0.17094	0.08212	0.04901	0.42742	0.03105	0.00800	0.00691	2.64444	0.00391	0.64530	0.39380	0.00000	0.00267	0.00423	0.01983	0.47326
**N6amt1**	3.19278	0.02590	0.51893	0.98117	0.01667	2.11143	1.33565	1.68102	2.47034	2.45513	4.12005	1.57920	0.38855	2.52297	0.62281	0.03098
**Naa10**	20.50080	6.10036	14.03636	5.07080	8.21664	2.82083	8.13009	13.87964	7.59459	11.89248	5.49977	1.68588	8.12842	0.21279	1.44058	11.04958
**Naa11**	3.90353	4.42073	6.31487	2.47684	7.17492	2.07464	2.88448	1.52009	0.07327	2.43066	1.13614	4.94557	0.06157	0.15333	0.80546	6.98509
**Naa15**	9.14831	11.64579	9.42021	9.33424	9.23488	9.62666	7.74863	11.11213	8.67482	9.61211	8.89426	10.04987	3.98950	28.27211	14.64242	7.15333
**Naa16**	5.10011	9.87391	2.85820	4.59475	0.76445	1.40252	2.59370	4.92652	10.44656	7.55240	4.18026	1.65000	4.23711	6.35948	6.83610	4.27657
**Naa20**	3.44255	4.58448	0.00000	6.99877	3.85099	1.65916	0.43243	2.42582	5.16028	0.46582	0.67457	3.54960	0.64919	6.49459	0.00000	0.19020
**Naa25**	3.65130	9.08609	6.78287	4.16448	0.01528	4.94647	5.83928	5.94936	3.55136	7.29477	1.46979	13.73472	3.41104	10.27914	5.28163	0.02129
**Naa30**	1.13368	4.99311	2.36609	21.19399	2.47728	2.48020	0.03182	2.59049	4.91786	1.48283	2.64528	4.20911	2.74309	10.51373	6.06315	0.07628
**Naa35**	10.53050	10.67518	5.84169	8.71790	5.99424	5.83847	6.75454	16.18300	9.73571	15.01581	10.31260	7.43268	6.42095	13.34293	11.79874	2.25578
**Naa38**	5.03145	0.83051	2.34903	4.02921	6.01911	1.14341	0.72019	1.23986	1.69489	2.66529	4.27506	0.28295	4.96458	0.00000	0.00000	3.19628
**Naa40**	7.00805	5.33103	6.56175	4.43299	7.96336	0.08260	8.07313	2.04550	2.69909	4.05097	0.97347	5.95249	7.30533	0.08883	3.66547	5.89627
**Naa50**	13.86543	12.98175	13.44687	17.82402	6.29641	7.24395	10.17330	10.26067	17.97133	17.36636	15.68557	15.55539	11.47720	27.58216	8.67308	3.25588
**Naa60**	3.18483	4.28756	1.08119	5.18503	5.85559	7.78751	6.56202	6.86440	1.34315	3.81695	2.49468	1.46749	4.08896	0.00000	0.86622	0.00001
**Naaa**	2.09725	1.47921	0.56225	0.13965	0.05918	6.42886	3.45683	3.41334	0.91584	3.55913	4.79216	0.06449	3.38249	2.36811	1.12119	3.88054
**Naalad2**	1.66603	3.09787	7.49353	7.37359	0.03170	2.61916	0.04365	22.88409	5.45302	15.06355	19.59249	19.49099	3.82786	27.92294	19.42159	0.49288
**Naaladl1**	0.01521	0.00870	0.00799	0.00938	0.01044	0.00704	0.00796	0.25163	0.01182	0.08961	0.00949	0.00888	0.42755	0.00684	0.14994	0.00719
**Nab1**	1.47922	3.56249	0.47032	7.88747	0.08182	0.00337	1.97508	0.00000	0.66869	0.42667	0.08514	0.00799	0.15212	0.01521	2.08559	0.00259
**Nab2**	0.37778	0.00740	0.00000	1.21778	0.01523	0.03334	0.00709	0.02035	0.06986	0.38689	0.02837	0.00586	0.01785	0.00422	0.09762	0.00706
**Nabp1**	0.33528	0.37780	2.95967	3.29165	0.26138	3.18894	1.68004	7.21570	17.70515	5.78560	3.69170	12.12572	5.38782	19.83994	17.17194	0.00588
**Nabp2**	2.84635	0.11738	0.00000	0.93676	4.98120	3.73853	0.15759	3.36764	1.11880	4.16758	1.22227	0.08648	0.73393	0.01654	0.05405	1.64568
**Naca**	46.17900	27.48332	40.48891	17.79562	30.35640	20.09737	22.17904	37.86927	27.03181	43.36190	40.82920	19.54800	45.12242	23.58332	21.14898	25.93257
**Nacad**	0.00963	0.00747	0.00690	0.00610	0.00977	0.00702	0.00702	0.25007	0.01127	0.19721	0.26512	0.04119	0.00552	0.01158	0.00119	0.00742
**Nacc1**	2.69695	2.97079	2.26175	5.14938	0.54686	2.04794	4.85298	2.75317	6.62017	3.79351	2.05833	0.09554	5.12029	0.03660	0.05873	1.57247
**Nacc2**	3.34228	0.01172	5.07706	7.77401	0.01660	0.08943	2.43633	7.33432	4.93571	11.07777	6.04622	1.15078	3.69813	5.02577	5.28440	0.84548
**Nadk**	10.26828	10.41173	12.78741	8.32983	8.16186	7.70842	6.78268	9.74095	4.66783	15.20130	11.11568	14.31327	11.08022	10.21837	10.35112	6.31583
**Nadk2**	2.33995	2.33329	0.94747	4.26651	0.22164	0.87277	0.00414	0.17581	1.01665	1.84409	0.59237	0.02097	0.58028	4.38429	2.31977	0.00936
**Nadsyn1**	0.84693	0.03746	0.08322	0.01050	0.00626	0.01075	0.00634	0.00866	0.01193	0.09236	0.01215	0.04618	0.99972	0.01557	0.05347	0.00636
**Nae1**	4.53707	7.36767	2.61292	3.09226	1.91646	0.01631	5.09053	5.01868	3.17317	5.55636	5.87862	2.54301	3.63468	3.58783	9.23847	4.76169
**Naf1**	0.93423	0.61843	0.13580	3.74789	2.46668	0.00266	0.22778	0.99507	5.28436	2.81589	5.96516	9.09465	0.22279	2.44098	5.10080	0.37916
**Naga**	1.94950	0.24479	0.65037	0.00000	0.01974	4.18948	1.87129	2.77099	0.09394	0.33841	0.00000	0.00681	2.18244	0.01247	0.00000	0.03061
**Nagk**	5.97157	5.87301	7.56742	0.00000	5.06888	2.76784	0.00123	4.04093	0.58746	2.91397	2.89569	0.38698	9.63928	0.03359	0.07090	7.83464
**Naglu**	0.06127	0.00792	0.01873	0.00710	0.00869	0.00787	0.00735	0.01229	0.01180	0.43003	0.10424	0.00584	0.41762	0.01132	0.00659	0.00754
**Nagpa**	2.53447	1.04429	2.44693	2.44647	0.46859	6.06026	2.15187	6.29459	0.16268	1.80094	3.89536	0.29074	11.19317	0.00000	1.35642	3.27257
**Nags**	0.34886	0.00749	0.00255	0.00295	0.00700	0.00700	0.00625	0.00758	1.55836	0.34047	0.04531	0.07047	1.17697	0.00633	0.48183	0.00666
**Naif1**	0.01042	0.03068	0.22427	0.00694	0.00935	0.00954	0.00803	0.03735	0.06690	0.06081	0.01112	0.00758	0.12402	0.01590	0.08432	0.00733
**Naip2**	0.05037	0.01842	0.03127	0.18857	0.00918	0.03837	0.01770	0.01410	0.30088	0.04575	0.16708	0.01554	0.06215	0.28161	0.05023	0.00725
**Naip5**	0.18820	0.02771	0.28097	1.83422	0.10471	0.06062	0.03687	0.06926	2.90998	0.53989	1.40510	0.13661	0.09977	1.00811	0.25822	0.05154
**Nampt**	5.38802	2.45517	1.64162	8.45834	2.50601	3.52563	1.02896	2.67378	15.57221	3.91830	4.44683	4.07706	2.66054	4.84686	10.36780	3.52219

**Nanog**	19.07460	9.93228	21.02989	12.57850	1.03580	5.54358	14.54913	7.93302	19.62326	3.05676	18.29695	1.55574	8.81598	11.19488	2.44013	4.15383
**Nanos1**	0.01156	0.00866	1.13645	7.54286	0.00817	0.01199	0.00726	0.39233	6.84290	0.28631	0.19054	0.48788	1.23995	0.00000	4.08290	0.00461
**Nanos2**	0.00944	0.00841	0.00796	3.25156	0.00872	0.00624	0.00802	0.09058	0.47313	2.71382	0.15813	0.00000	0.01089	0.00000	0.98599	0.00646
**Nanos3**	0.01285	0.00772	0.01086	1.49349	0.00909	0.00624	0.00732	0.01166	0.61334	1.06080	0.00600	0.02181	0.01136	0.00213	0.86940	0.00713
**Nanp**	1.53565	0.00186	0.00629	2.06799	7.48434	0.00675	0.00520	0.03050	0.00000	0.00126	0.01146	0.00835	1.44236	0.00508	0.72260	0.01154
**Nans**	2.33062	0.06127	0.33371	3.49167	2.89660	0.01022	1.30939	0.00000	5.29198	4.35182	0.32090	0.17643	0.37077	0.00359	0.00542	5.68758
**Nap1l1**	30.63744	20.01769	26.07023	20.77023	23.87153	14.92538	14.25026	25.73667	28.53359	42.62229	34.31363	26.34394	23.87926	36.50610	29.48656	13.28037
**Nap1l2**	0.01255	0.00818	0.01394	0.13056	0.01017	0.00715	0.00770	0.01593	0.00687	0.01197	0.01400	0.01793	0.21417	0.01328	0.01122	0.00768
**Nap1l3**	0.00644	0.01654	0.00452	0.04047	2.15164	0.00736	0.00741	0.00915	0.00753	0.00309	0.01094	0.00551	0.00975	0.01611	0.20999	0.00713
**Nap1l4**	4.90288	2.30724	7.57934	16.57751	7.10997	5.94536	4.91040	10.68558	15.45973	12.71862	11.26329	0.39559	8.92483	1.15901	7.76049	5.93576
**Napa**	12.38263	4.72819	14.67412	2.95668	12.90872	10.68203	9.81664	16.77656	4.06485	10.93647	5.44307	0.59536	10.97274	0.01086	3.37878	6.56661
**Napb**	0.01745	0.00794	0.01051	0.11085	0.00859	0.00742	0.14657	0.01047	0.00633	0.37184	0.00107	0.01296	0.31524	1.30046	0.53003	0.03570
**Napepld**	0.99818	0.00413	1.80419	1.60537	3.99985	2.37719	0.00442	0.92247	1.55337	4.21373	2.65546	0.06894	1.12293	0.00000	0.99677	1.30137
**Napg**	6.00876	1.99263	6.82766	3.70773	4.40420	3.97923	2.37646	3.77723	3.77158	2.77745	4.82891	11.94417	2.13709	2.15840	8.96606	4.83990
**Naprt**	2.96660	0.00404	0.00000	0.00274	0.01916	0.98446	0.00471	0.56255	0.65356	0.02605	0.00000	0.00328	0.04250	0.04196	0.00000	0.00372
**Napsa**	0.00000	0.00683	1.54529	0.00468	0.01749	0.00571	2.53633	0.00656	0.01124	0.52357	0.10111	0.05491	0.99702	0.00759	0.07517	0.01353
**Narf**	0.10890	0.00750	0.67665	1.75457	0.01635	0.00552	1.43381	2.00008	1.94537	0.11279	0.10528	0.00000	0.04374	0.83742	0.28970	1.97047
**Narfl**	4.21880	7.60238	8.66920	4.22722	6.42181	7.41431	3.79746	9.99298	8.32803	13.83780	2.61222	3.55165	11.47476	0.00000	2.17593	5.04986
**Nars**	22.89023	19.33025	18.80494	3.49633	15.79206	8.31535	15.53380	22.67425	5.29002	14.29474	8.05903	8.37350	16.73887	16.91110	13.66399	11.71709
**Nars2**	0.27580	0.00845	0.00877	0.00000	0.03512	0.00806	0.00664	0.50356	0.20240	0.08721	0.17586	0.27228	0.86643	1.06908	2.30078	0.01442
**Nasp**	26.70205	22.37761	20.33619	24.13854	12.84075	14.19040	18.71256	29.30812	40.61431	40.12860	31.59164	23.25909	20.93769	35.08779	32.22305	13.67710
**Nat1**	0.01254	0.00826	0.57368	0.22655	0.00835	0.00565	0.00763	0.01376	0.00542	0.01466	0.01131	0.00847	0.00891	0.01232	0.00000	0.00711
**Nat10**	10.12356	6.55163	8.57804	4.23614	0.77593	3.87619	8.33612	15.26134	7.01835	10.84601	6.32907	4.64081	5.84268	0.78984	3.81819	2.65599
**Nat14**	0.00861	0.00833	0.03819	0.56949	0.02738	0.00854	0.00795	0.22852	0.32385	0.07946	0.42732	0.04903	0.00710	0.00897	0.00000	0.00742
**Nat6**	2.79891	0.00387	2.25388	0.20820	0.00000	3.12254	0.20635	1.91599	0.19483	1.64613	0.00000	0.00488	1.52571	0.00925	0.00000	0.01644
**Nat8**	0.01351	0.00782	0.01563	0.04181	0.00931	0.13583	0.00737	0.01234	0.06161	0.05071	0.01261	0.00734	0.01077	0.04530	0.00489	0.00739
**Nat8f1**	0.01578	0.00753	0.21287	0.13533	0.00999	3.13862	0.00687	0.00833	1.84419	0.54018	0.00000	0.00000	0.00312	0.27361	0.08209	0.00615
**Nat8f2**	0.26603	0.00738	0.03589	6.57403	0.00714	0.00462	0.00708	1.46852	4.34930	0.53812	0.10387	0.06369	0.00672	0.55076	0.98959	0.01276
**Nat8f3**	0.01378	0.00805	0.01203	0.01616	0.00933	0.00655	0.00744	0.01250	0.01211	0.01287	0.01338	0.00811	0.01253	0.00890	0.24049	0.00732
**Nat8f4**	0.02069	0.00788	0.93300	0.21582	0.00707	0.00546	0.00724	0.07308	0.48111	0.01996	0.00757	0.03806	0.31514	0.00932	0.05421	0.00674
**Nat8f7**	0.01378	0.00796	0.01207	0.01062	0.00933	0.00664	0.00751	0.01342	0.01204	0.01251	0.01277	0.00806	0.01272	0.01355	0.06110	0.00740
**Nat8l**	0.01256	0.00786	0.10072	0.74632	0.00904	0.00877	0.00748	0.01559	0.00000	0.01199	0.01263	0.05767	0.01108	0.01271	0.03791	0.00724
**Nat9**	6.74669	0.04168	6.02687	0.00268	2.08542	0.02074	0.00833	2.50753	0.00781	0.18297	0.21254	0.00013	6.65894	0.02633	0.00783	3.15639
**Natd1**	4.78558	2.89647	9.68298	9.94956	0.00887	5.58239	6.03122	5.62754	13.05420	9.18529	5.80140	0.65631	2.26995	5.19104	7.62822	4.38105
**Nav1**	1.33595	2.89042	0.88687	1.23330	0.29645	1.68819	0.12605	0.70069	8.22394	2.22654	2.19165	0.35429	3.34994	16.00279	0.00000	0.12100
**Nav2**	0.00000	0.00795	0.03618	2.94989	0.00859	2.95714	0.02602	1.19642	1.24549	1.07374	0.12872	0.03919	3.69696	0.00830	0.38774	0.00394
**Nav3**	0.01129	0.00747	0.47701	0.12401	0.00859	0.00572	0.00727	0.15444	0.38463	1.06402	0.00494	0.00000	0.00685	0.01040	2.49599	0.00698
**Nbas**	0.00000	0.00519	1.71010	0.78680	3.74029	0.00814	0.00671	2.03467	0.05266	0.67036	0.89717	0.29230	0.00000	0.03849	0.00000	2.12453
**Nbea**	0.01137	0.00801	0.00535	0.00893	0.00946	0.00685	0.00730	0.00988	0.01152	0.33966	0.00461	0.00000	0.01305	2.72772	0.00435	0.00737
**Nbeal1**	1.02293	0.00626	0.00000	0.89390	0.00586	0.01764	0.00609	0.00000	0.86857	0.00000	0.14046	0.66944	0.01031	2.18776	0.95560	0.00573
**Nbeal2**	1.07337	0.00586	0.82415	0.49971	0.18903	0.21429	0.16404	2.86429	0.00375	4.17804	0.56469	0.00000	0.75478	0.03520	0.00000	1.21621
**Nbl1**	0.00599	0.06885	0.08671	0.35042	0.01703	0.00871	0.00644	0.00613	0.00613	1.99797	0.06068	0.02071	1.21209	0.01067	0.00000	0.00686
**Nbn**	3.19131	0.00424	1.83534	1.96971	0.00970	0.00504	0.00232	0.00000	4.17770	7.15380	5.19823	0.48396	1.24422	5.29084	5.16718	1.74388
**Nbr1**	6.07114	0.11592	7.02688	13.95287	0.51000	7.00838	5.62853	6.71426	11.76330	7.33138	4.86698	7.24868	7.81973	5.02596	12.68307	2.60448
**Ncald**	0.01333	0.00781	0.01317	0.21747	0.01002	0.00688	0.00762	0.01370	0.39779	0.01163	0.00958	0.00573	0.05394	0.00741	0.50334	0.00768
**Ncam1**	0.78439	0.00704	0.00815	0.00636	0.00636	0.00632	0.00704	0.01340	0.03614	1.01213	0.00996	0.10134	0.02833	0.06885	0.02056	0.00676
**Ncan**	0.02380	0.00818	0.04979	0.02933	0.02640	0.01167	0.00758	0.01121	0.10198	0.01881	0.19548	0.02883	0.05016	0.01013	0.26249	0.02164
**Ncapd2**	17.00474	11.00631	11.81240	6.23634	9.92021	5.48334	9.03055	11.06925	8.88831	10.19378	2.04101	4.83950	8.88478	5.19587	12.64610	6.66409
**Ncapd3**	0.84992	1.92821	1.46788	2.49351	0.10129	0.00455	2.56063	2.29762	3.11563	0.29053	2.50006	7.70128	0.48061	0.76030	3.15718	0.64410
**Ncapg**	2.62685	5.88216	4.33343	1.25417	0.28494	0.54572	3.00128	2.71375	0.94571	1.05272	0.33865	0.36289	1.93693	0.00000	5.55048	1.43747
**Ncapg2**	1.07964	1.95962	2.97791	10.84491	0.04067	3.55660	3.39183	3.65974	9.47939	0.74729	3.26783	10.87686	0.78607	2.55469	10.84074	0.39928
**Ncaph**	9.20179	1.66013	2.64460	1.24619	0.73526	0.00649	3.54606	9.28169	2.05965	2.57153	2.19993	0.20108	4.87259	0.13057	5.13325	1.33587
**Ncaph2**	14.23428	2.33634	14.57177	4.01763	5.80775	10.16263	8.70396	18.54285	4.40261	12.33078	9.54014	0.83415	20.88467	0.49621	7.85415	11.39508
**Ncbp1**	5.05763	7.94682	7.86304	10.80654	0.38584	7.48070	11.99055	15.64429	15.77524	22.25445	10.38809	5.08508	13.15713	21.51219	23.20190	4.64808
**Ncbp2**	13.09157	13.04774	13.95160	15.79177	1.97049	5.90236	7.59064	12.04263	19.80874	24.35233	16.41775	11.78984	12.20661	15.27202	20.90560	3.62062
**Ncbp3**	4.60274	4.90681	4.58752	10.19082	5.69410	4.77483	4.72117	3.48420	14.07600	7.33898	9.37094	4.56311	1.54071	13.95236	12.28429	1.94426
**Ncdn**	5.84358	0.04361	0.46488	1.95501	0.68556	0.15401	0.00977	0.80857	1.70590	6.14760	0.80394	0.06363	4.81440	0.04523	0.17237	0.11429
**Nceh1**	0.00651	0.00823	1.78859	5.48861	0.00687	0.00717	0.00695	0.01871	3.03844	0.91262	0.00000	0.03117	0.40235	0.32442	3.15528	0.00546
**Ncf1**	0.03282	0.00777	0.02783	0.08820	0.00805	0.03364	0.00706	0.00000	0.20473	0.22517	0.84810	0.02350	0.02603	0.07112	1.24281	0.00705
**Ncf2**	0.66730	0.01951	0.24591	0.05106	0.01502	0.00507	0.00657	0.00263	0.01887	1.80487	0.50378	0.02121	0.01051	0.02905	0.01643	1.77081
**Nck1**	2.48416	5.37647	0.16305	0.16677	0.02145	0.01378	1.85670	1.34084	2.43876	5.55562	1.13588	10.00170	2.97576	0.79081	1.28480	0.01517
**Nck2**	0.01420	0.04949	0.00908	0.11465	0.01057	0.00665	0.00715	0.08616	0.00744	0.00144	0.16386	0.11896	0.00775	0.32654	0.00129	0.00735

**Nckap1**	7.27133	6.13966	8.19587	19.64701	5.42906	8.44978	3.69904	7.02269	9.34955	12.96509	11.14288	18.86992	7.83032	20.27523	17.13835	0.82550
**Nckap1l**	0.00734	0.00788	0.01180	0.36680	0.00760	0.00636	0.00740	0.01207	0.36617	0.93363	0.00702	0.00443	0.00777	0.00472	0.31763	1.40087
**Nckap5**	0.01368	0.00789	0.01016	0.50222	0.00900	0.00686	0.00742	0.00517	0.00452	0.40667	0.25446	0.00648	0.01006	0.00476	0.17568	0.00724
**Nckap5l**	0.80406	0.00659	0.00342	0.09820	0.00628	0.00737	0.00656	0.15853	0.58729	0.01520	0.00745	0.00580	0.04936	0.01261	0.00304	0.00644
**Nckipsd**	3.32142	0.00346	1.16443	0.41282	0.00504	0.01531	1.49988	0.27703	0.00405	0.00000	0.42831	0.03768	3.47446	0.02258	0.01951	2.00275
**Ncl**	34.87694	28.35505	32.97174	17.87219	25.84932	14.50073	20.40271	39.87150	26.27602	30.07491	34.63108	23.19390	31.79202	31.53150	34.05280	19.59727
**Ncln**	5.62937	0.03669	0.06566	0.01304	5.09725	0.61468	3.04376	8.45408	0.53743	3.99516	0.58323	0.32723	10.52280	0.01567	0.04307	5.74573
**Ncmap**	0.67081	0.01791	0.00202	4.46819	0.01455	0.00662	0.08289	0.01473	5.69537	0.95739	0.00000	0.03680	0.01708	0.05848	0.41590	0.00531
**Ncoa1**	2.19079	0.00466	1.38605	6.18233	0.78400	4.79839	0.02583	0.79693	2.67293	1.48638	0.74532	0.03425	0.26137	0.00000	1.17282	2.04075
**Ncoa2**	0.00000	0.00354	0.40858	13.21808	4.07180	4.17925	3.23090	2.38230	2.83128	0.60787	0.51138	0.12716	2.38651	0.00000	1.34947	0.23012
**Ncoa3**	11.31546	10.10467	9.62117	16.41097	9.96565	8.06673	7.47426	21.25571	17.92156	22.30630	18.82627	15.78083	8.26544	13.17076	18.49726	5.86064
**Ncoa4**	6.88130	8.93477	12.45160	7.68607	10.06316	7.02376	4.13444	9.71912	4.06411	9.10697	10.48443	16.65068	9.26705	14.03442	6.18631	4.86677
**Ncoa5**	0.81260	2.85747	2.99790	1.53950	4.81282	0.01240	0.00426	0.86279	0.33042	2.15962	0.69003	0.04163	2.94602	0.32469	1.69032	0.03960
**Ncoa6**	2.28663	5.07579	3.16806	19.27545	5.06919	3.67727	4.91156	1.61286	7.70696	2.78318	4.41958	1.77582	2.43671	11.41124	16.61969	2.12407
**Ncoa7**	0.38145	0.00696	0.00000	0.00903	0.00714	0.40553	0.00717	0.00977	0.01125	0.01206	0.01032	0.00688	0.00843	0.01805	0.39450	0.00677
**Ncor1**	9.28016	2.52140	5.42376	4.95298	4.50326	5.87817	0.07964	4.18712	4.64438	5.67184	2.36036	0.53142	2.96874	7.36723	10.25365	7.01787
**Ncor2**	2.08687	0.00391	0.60194	0.07004	1.62758	0.01388	1.25241	0.64577	0.15772	0.22119	0.16043	0.11492	0.37303	0.01136	0.00816	2.67194
**Ncs1**	0.00854	0.00771	0.37376	0.00525	0.00747	0.00567	0.00735	0.00984	0.22487	1.07549	0.00878	0.06404	0.02124	0.01408	0.01988	0.66006
**Ncstn**	0.19647	1.63544	1.71107	0.13861	0.00453	0.00749	0.56551	4.23051	0.29347	1.16856	0.93131	0.00000	0.84468	6.96272	3.58591	2.41830
**Ndc1**	2.31659	3.02533	9.88620	0.85816	7.06314	1.54748	6.61683	7.38857	0.76604	6.27270	8.55141	3.46385	4.68598	5.61840	8.83641	0.34046
**Ndc80**	3.82525	5.50418	5.27388	2.68701	6.13138	6.37911	6.58307	10.93607	13.64598	15.62894	8.61225	3.58235	3.60463	17.78134	11.06352	2.65881
**Nde1**	2.98447	0.01119	5.69767	5.85883	3.67863	2.09993	2.57820	0.00000	3.00487	1.98269	1.89202	0.09412	0.00000	0.00000	1.94596	1.95087
**Ndel1**	6.32745	0.52812	2.31070	8.37928	7.04289	1.90808	1.53903	0.78755	7.54620	11.38955	5.20787	4.95201	5.26935	0.00555	1.96590	5.00530
**Ndfip1**	2.44302	3.53480	1.08305	1.47261	7.36078	3.39122	0.32358	0.75677	0.79555	0.45811	2.78523	1.09263	1.63548	1.09082	0.65593	0.87475
**Ndfip2**	0.92260	5.03114	0.13528	6.30018	0.54903	0.43703	3.71513	0.92896	1.12536	0.54290	3.04045	0.18431	1.18838	2.73912	0.85134	0.00229
**Ndnl2**	2.68435	5.05961	2.34319	3.46652	6.45214	0.03064	0.05529	4.12853	4.84463	5.98022	2.58855	0.99706	0.89270	2.91981	2.95659	0.06446
**Ndor1**	3.59171	0.01084	5.14396	0.73401	0.00558	0.78581	4.03036	9.50488	5.73210	10.26988	4.23143	1.25163	4.95430	0.26570	4.88082	3.24102
**Ndrg1**	0.03035	0.00396	3.30466	0.00000	0.06041	8.03575	4.72807	0.00000	0.05131	0.00000	0.27786	1.85328	0.02623	0.00693	0.00000	4.11514
**Ndrg2**	0.00421	0.00711	5.32637	0.00668	0.00000	0.00715	0.00666	0.75275	0.01065	1.47912	0.00271	0.00000	0.00000	0.01313	0.00000	0.00568
**Ndrg3**	6.81001	5.73983	11.38116	1.26675	10.55161	8.21003	6.84623	12.64346	0.66452	5.75261	8.72669	4.74109	5.89353	10.20321	2.01331	8.39260
**Ndrg4**	0.01367	0.00830	0.01094	1.19099	0.00920	0.00651	0.00748	0.01094	0.88625	0.00574	0.11756	0.00637	0.01077	0.00667	0.04320	0.00705
**Ndst1**	2.45007	0.00508	1.21764	1.78943	0.00000	0.00274	0.00556	1.29052	1.15362	2.99803	0.26430	0.42027	0.17083	1.72710	2.66315	0.02525
**Ndst2**	0.01019	0.00782	0.76482	0.18907	0.00753	0.00945	0.00712	0.00000	0.00777	0.00000	0.53627	0.00334	0.00931	0.00524	0.78906	0.85683
**Ndufa1**	1.39852	0.07719	1.65987	2.26639	4.92623	0.41655	0.24731	0.04954	1.64500	0.58064	1.99729	0.11061	0.35401	0.28710	0.81043	1.42852
**Ndufa10**	14.16378	2.71480	12.90717	0.67852	3.47315	7.70224	6.07528	4.27264	1.00083	9.07051	3.84712	2.69516	12.15836	0.23585	2.69469	8.44406
**Ndufa11**	7.38403	2.07917	3.33332	6.96884	9.55022	3.09544	2.61720	4.69431	1.84552	8.53582	7.86452	0.89604	9.58937	0.16090	2.93987	4.15321
**Ndufa12**	11.46405	0.32638	6.01756	2.21327	7.78186	3.00072	1.19568	1.95595	0.39532	8.38621	12.28177	0.41769	8.65798	0.23111	0.00000	6.09530
**Ndufa13**	9.84333	0.54253	5.40273	3.05181	7.87865	1.69441	0.65019	1.25216	2.69198	4.98633	7.23090	0.38226	2.99963	0.13089	0.23199	5.61917
**Ndufa2**	3.14538	0.08672	2.05239	12.03155	6.67917	1.74164	1.01696	0.09384	18.84652	2.65758	5.05848	0.31062	1.93776	0.00000	0.02187	3.34245
**Ndufa3**	3.32107	0.44478	2.47602	0.11543	1.14390	0.55834	0.12183	0.57387	0.80116	1.90141	11.01030	0.18422	1.81343	1.45563	0.38580	3.06969
**Ndufa4**	11.14697	1.81517	10.80181	2.08312	13.70962	2.58924	1.99045	1.29955	0.67598	2.41948	7.04352	0.80418	6.63299	3.97377	5.06243	3.83066
**Ndufa5**	5.54250	0.41586	5.70556	0.98526	10.16849	1.42648	0.45845	0.00000	0.84734	1.25496	12.55663	0.43813	1.87758	0.89793	0.73714	2.03301
**Ndufa6**	8.08954	1.08945	1.37767	4.66126	1.49240	4.34973	0.20872	0.95850	4.83198	4.79819	3.14239	0.01914	2.94971	0.05787	0.00000	3.45894
**Ndufa7**	8.36865	0.97699	5.80513	4.69865	3.93007	2.27897	1.05421	0.68459	9.43495	4.02828	11.37346	0.35244	3.28975	0.11215	1.37382	5.53979
**Ndufa8**	9.95568	8.03747	9.21457	0.13313	10.75712	3.14282	4.96917	7.16383	0.00000	7.99890	7.75709	0.41277	11.53597	0.21299	0.18652	7.26367
**Ndufa9**	17.16394	10.81027	10.27852	4.41855	8.78751	6.27193	11.38243	17.92029	7.37614	10.96036	16.20470	5.92525	17.51476	10.09811	4.02578	6.92160
**Ndufab1**	3.21044	1.19577	1.80241	9.59312	5.65315	0.49525	0.14585	0.00000	8.50796	4.38811	5.20937	0.18736	1.57524	0.40007	0.07118	2.64252
**Ndufaf1**	3.81440	1.69705	6.73072	3.77442	1.85436	0.00733	1.53193	2.79578	3.19774	7.86544	3.96099	3.18012	2.46433	4.62797	4.04519	1.37257
**Ndufaf2**	4.63516	2.76702	2.08541	6.22446	6.81533	1.92659	2.23180	1.73560	3.69874	4.62449	2.67647	0.47046	3.04079	0.53867	1.57104	3.34290
**Ndufaf3**	3.71161	0.85261	2.68738	0.43378	1.38569	0.08291	0.44232	0.00000	0.00010	0.33361	0.77321	0.00000	2.29503	0.00898	0.00000	0.37202
**Ndufaf4**	8.53988	6.00811	4.25270	7.47337	4.35596	6.98145	3.14707	8.23787	3.02324	4.18895	4.42359	1.32550	2.70803	5.26108	6.09570	2.91472
[truncated: 950,416 more chars]
